# Comparison of Gene Expression and Genome-Wide DNA Methylation Profiling between Phenotypically Normal Cloned Pigs and Conventionally Bred Controls

**DOI:** 10.1371/journal.pone.0025901

**Published:** 2011-10-11

**Authors:** Fei Gao, Yonglun Luo, Shengting Li, Jian Li, Lin Lin, Anders Lade Nielsen, Charlotte Brandt Sørensen, Gábor Vajta, Jun Wang, Xiuqing Zhang, Yutao Du, Huanming Yang, Lars Bolund

**Affiliations:** 1 Department of Biomedicine, Aarhus University, Aarhus C, Denmark; 2 Department of Animal Science, Aarhus University, Tjele, Denmark; 3 Department of Biology, University of Copenhagen, Copenhagen, Denmark; 4 BGI/Huada, Shenzhen, Guangdong, People's Republic of China; University of Insubria, Italy

## Abstract

Animal breeding via Somatic Cell Nuclear Transfer (SCNT) has enormous potential in agriculture and biomedicine. However, concerns about whether SCNT animals are as healthy or epigenetically normal as conventionally bred ones are raised as the efficiency of cloning by SCNT is much lower than natural breeding or In-vitro fertilization (IVF). Thus, we have conducted a genome-wide gene expression and DNA methylation profiling between phenotypically normal cloned pigs and control pigs in two tissues (muscle and liver), using Affymetrix Porcine expression array as well as modified methylation-specific digital karyotyping (MMSDK) and Solexa sequencing technology. Typical tissue-specific differences with respect to both gene expression and DNA methylation were observed in muscle and liver from cloned as well as control pigs. Gene expression profiles were highly similar between cloned pigs and controls, though a small set of genes showed altered expression. Cloned pigs presented a more different pattern of DNA methylation in unique sequences in both tissues. Especially a small set of genomic sites had different DNA methylation status with a trend towards slightly increased methylation levels in cloned pigs. Molecular network analysis of the genes that contained such differential methylation loci revealed a significant network related to tissue development. In conclusion, our study showed that phenotypically normal cloned pigs were highly similar with normal breeding pigs in their gene expression, but moderate alteration in DNA methylation aspects still exists, especially in certain unique genomic regions.

## Introduction

DNA methylation occurs at most CpG dinucleotides in the mammalian genome [1]. Reprogramming of DNA methylation is essential for early embryonic development, as genome-wide reprogramming of DNA methylation ensures removal of zygotic methylation marks in the nucleus and reestablishment of a different set of marks important for generating toti- and pluripotency [2]. DNA methylation is one of the most studied epigenetic regulatory mechanisms which plays a key role in gene expression regulation. It is essential for establishing genomic imprints for tissue-specific differentiation in the early stage embryo. Often regulatory DNA methylation is occurring in promoter-associated CpG islands (CGIs), which are relatively unmethylated stretches of DNA with high CpG density [3].

Somatic Cell Nuclear Transfer (SCNT) is a promising tool for animal breeding. It has been successfully used to generate cloned animals for both agricultural applications and development of animal models for human diseases [4], [5]. SCNT has been successfully applied in many mammalian animals including pigs, and many efforts have been endeavored to simplify the procedure and to increase the efficiency. This is exemplified in an approach named “handmade cloning (HMC)” [6], [7] that is completely free of micromanipulations as well as in an approach involving pretreatment of the oocytes with high hydrostatic pressure (HHP) to improve developmental competence [8]. To produce viable offspring by SCNT, a drastic spatial and temporal remodeling of gene expression, invovling DNA methylation, is required to mimic the embryonic development *in vivo* [2].

The reprogramming by DNA methylation during normal development takes place in a much longer time frame than in SCNT. In SCNT incomplete or aberrant reprogramming may happen and this is postulated to attribute to the low efficiency of cloning and the improper development of cloned animals sometimes observed [9], [10], [11]. In cloned pigs, postnatal mortality can arise from unknown reasons, but macroscopical phenotypic anomalies are seldom observed. This is possibly due to strong selection mechanisms. Often more *in vivo* fetuses develop to birth with average litter than in litters of cloned pigs, despite the fact that 80–100 blastocysts are transferred in the cloning procedure. The early cloned embryos with aberrant reprogramming might be selected out and thereby fail to establish pregnancy. Thus, it's of great interest to see whether cloned animals with normal phenotype possess abnormal DNA methylation.

To date, only a limited number of studies have addressed gene expression patterns and DNA methylation status for cloned pigs produced by SCNT, and no studies have addressed genome-wide gene expression and DNA methylation patterns in parallel. We have developed a high-throughput method, Modified Methylation Specific Digital Karyotyping (MMSDK), to detect genome-wide DNA methylation patterns by combining methylation-sensitive enzyme enrichment of unmethylated DNA tags and massively parallel tag-sequencing technology [12]. In the present study, we used Affymetrix Pig arrays to perform gene expression profiling in cloned pigs with normal phenotypes. In parallel, we applied the MMSDK method to examine tissue-specific and genome-wide DNA methylation patterns. Our primary aim was to investigate whether the gene expression and DNA methylation were normal in muscle and liver of phenotypically normal cloned pigs compared to conventionally bred control pigs.

## Materials and Methods

### Ethics Statement

All procedures involving animals described in present study were reviewed and approved by the Danish Experimental Animal Inspectorate (“Rådet for Dyreforsøg”), Danish Ministry of Justice. Procedures for transfer to recipient pigs of cloned embryos, and to have such piglets born and raised were conducted under approval ID no. 2004/561-925. All other procedures were also reviewed and approved by the Danish Experimental Animal Inspectorate, Danish Ministry of Justice; however, no specific approval ID is issued for other aspects of experimental animal use, such as animal care and killing of the animals.

### Pig samples

Cloned pigs and *in vivo* bred control pigs were obtained from the Faculty of Agricultural Sciences, Aarhus University. Liver and muscle tissue samples were collected from one litter of two male cloned pigs and one litter of two male control pigs both at six weeks after birth. The cloned pigs were from the same fetal cell line which originated from the ear cell of an offspring of a sow which is a hybrid of Landrace and Yorkshire and a boar of Duroc and were age, breed, and sex matched to control pigs from their conventionally bred counterparts. They were produced by HMC with pretreatment of HHP of the oocyte, as described previously, and were confirmed to be genetically identical using microsatellite DNA analysis. All pigs were placed in adjacent identical pens and given continuous access to a standard commercial feed ration and water [13].

### Genomic DNA and total RNA isolation

Genomic DNA and total RNA were isolated from frozen tissue samples using DNeasy® Blood & Tissue Kit (QIAGEN) and TRIzol reagent (Invitrogen), respectively, following the manufacturer's protocols. RNA integrity was assessed by 1% agarose gel electrophoresis and by Agilent Bioanalyzer QC.

### Gene expression profiling and data analysis

High quality total RNA from each sample was used for microarray experiments. Microarrays were hybridized and scanned in Molecular Diagnostic Laboratory, Clinical Biochemiccal Department, Aarhus University Hospital/Skejby Sygehus. Briefly, cRNA probes were synthesized from 5 µg total RNA, labeled by biotin and hybridized to Affymetrix GeneChip® Porcine Genome Array (Santa Clara, California), which contains a total of 24,123 probe sets, of which 23935 probe sets interrogate 23,256 transcripts in pig, which represents 20,201 genes, and 11,265 genes among them were annotated.

After scanning, the raw expression data was generated by GeneChip Operating Software (GCOS) and assessed with affyQCReport to ascertain individual array quality, homogeneity between arrays, variance mean dependency, RNA degradation and data distribution. The raw expression data was processed with RMA (Robust Multi-Array) normalization, and absent/present (A/P) calls [14], and the hierarchical clustering “*pvclust*” was applied to group samples based on similarity of gene expression data [15]. 18432 out of the total 24123 probe sets remained for statistical comparison to identify the differentially expressed genes (DEG) in muscle or liver between cloned and control pigs. The comparison was performed using the linear modelling of the limma package [16], [17] from publicly available R software. The following criteria were used to determine the significantly and differentially expressed genes: fold change (FC) of probe intensity ≥2 or ≤0.5 and *P* value<0.05. Finally, DEGs were analyzed by Ingenuity Pathway Analysis (IPA, Ingenuity Systems, www.ingenuity.com, Redwood City, CA) for their functional involvement in biological processes, pathways and molecular networks.

### Quantitative real time PCR (Q-PCR)

Synthesis of cDNA was performed with the iScript cDNA Synthesis Kit (BIO-RAD, Cat #: 170-8890) from one µg of total RNA of the samples used for microarray. One µl (10 times diluted) cDNA product was used as template for quantitative real-time PCR analysis. Quantitative RT–PCR was carried out using LightCycler 480 SYBR Green I Master (Cat. 04887352001) on LightCycler 480 (Roche). Beta-Actin was chosen as reference gene for Q-PCR. Comparison of relative gene expression level among the samples was calculated as fold changes of the sample, in which the gene expression level is the lowest. Primers are listed in Table S7.

### Generation of MMSDK tag libraries

Eight MMSDK tag libraries, one library for each tissue and individual pig, were constructed followed the protocol described previously [12]. Briefly, for each sample, 4 µg of genomic DNA were digested with methylation-sensitive mapping enzyme *Mlu*I (New England Biolabs), ligated to a biotinylated linker, and fragmented by *Nla*III (New England BioLabs) cleavage following the protocol. As *Mlu*I only cuts unmethylated regions, the DNA fragments ligated with biotinylated linkers were captured by streptavidin-conjugated beads to separate unmethylated and methylated fragments. Controls were set up to monitor unspecific binding of fragments without biotinylated linker and were called bead-controls. Next the isolated DNA fragments were ligated with linkers (N) containing a *Mme*I recognition site, and then digested with the Type IIS restriction enzyme (tagging enzyme) *Mme*I (New England Biolabs) to generate short sequence tags (17-bp). Finally, the tags were ligated with P7 linker and amplified by PCR using Phusion polymerase (Finnzymes) and primers N and P7. PCR products were purified using a QIAquick MiniElute kit (Qiagen) and stored as library for each sample. Before Solexa sequencing, conventional clone sequencing was carried out to verify the library quality as described previously [12]. Massively parallel Sequencing-By-Synthesis (SBS) was performed for the sequencing of tags using Illumina Cluster Generation (Illumina) and 1G Genome Analyzer (Illumina) according to the manufacturer's instructions. All reagents for the sequencing process were purchased from Illumina Inc. As the method detects unmethylated CpG sites, the status of DNA methylation is derived indirectly.

### Data analysis for MMSDK tag libraries

The final build pig genome (Sscrofa10) was downloaded from Ensembl database (ftp://ftp.sanger.ac.uk/pub/S_scrofa/assemblies/PreEnsembl_Sscrofa) and annotated by blasting to the human genome in the RefSeq database (http://www.ncbi.nlm.nih.gov/refseq).

We extracted 17-bp tags from all reads of 1G Genome Analyzer for each sample with the Solexa pipeline, and applied MAQ (Mapping and Assembly with Qualities) algorithm [18], [19] to map tags back to a virtual *Mlu*I library in order to avoid ambiguous mapping to the whole genome. To simulate the *Mlu*I enzyme digestion of the pig genome we located the predicted *Mlu*I sites, identified the nearest *Nla*III sites in both directions and took the derived DNA fragments as the reference for mapping. All the virtual tags that were not unique in the genome were removed so as to ensure unambiguous mapping. We defined two types of mapping quality score according to the description in the MAQ manual: the low-confidence type with a mapping quality score more than zero (MQ0), and the high-confidence type with a mapping quality score more than twenty (MQ20). As described in the MAQ manual, if a read can be mapped to several equally best positions, MAQ will randomly choose one position and give the alignment a zero mapping quality. In principle, a tag with MQ20 should have 1% error rate. SNPs and sequencing errors are considered in calculating this score. We used MQ0 as a low-confidence criterion for addressing whether a tag can be mapped back to the genome, and MQ20 as a criterion for collecting high-confidence tags used for data analysis.

For the analysis of tag information, we divided the tags into two categories considering whether they are located in repeat sequences according to RepeatMasker [20]. The tags located in repeat sequences were categorized and analyzed according to normalized tag counts of MQ0 by a Wilcoxon rank-sum test [21]. Tags located in unique sequences were first filtered through two steps: in the first step, we kept the tags having a mean among all libraries equal to or greater than 5; in the second step, we kept the tags having a standard deviation equal to or greater than 5 among all libraries of tags having a mean (among all libraries) of less than 5 in the first step. After filtering, the remained tags were normalized for their MQ20 tag numbers by dividing the tag count of each *Mlu*I site in a library with the total tag number in the library and clustered by hierarchical clustering using the “pvclust” package [15] from publicly available software R (www.r-project.org, Vienna, Austria): This groups samples by measuring the similarity between two DNA methylation patterns represented by tags' positions and counts based on Pearson's correlation coefficient values. Concerning the uncertainty of clustering, pvclust calculates p-values for hierarchical clustering via multiscale bootstrap resampling. *P* value of a cluster is a value between 0 and 1, and indicates how strong the cluster is supported by data. Pvclust provides two types of P values (%) on the edge of the cluster: AU (Approximately Unbiased) *P* value and BP (Bootstrap Probability) value. AU *P* values are computed by multiscale bootstrap re-sampling, and BP values are computed by normal bootstrap re-sampling. Clusters with AU larger than 95% are strongly supported by data.

We used a Poisson-based Significance Analysis algorithm (SA) [22] for the filtered tags to perform pair-wise comparisons for the tissue libraries between cloned and control pigs. *P* values were calculated from raw tag counts and adjusted by False Discovery Rate (FDR) [23] to correct for multiple comparisons. Promoters associated with CGIs in pig genomic sequences were predicted by using CpGProD (CpG Island Promoter Detection) software [24], in which tags were identified. We identified the genes neighboring the identified tag sites and analyzed their potential functional involvements in biological processes, pathways and molecular networks by Ingenuity Pathway Analysis (IPA).

### Bisulfite sequencing PCR (BSP)

Specific validation regions were selected and bisulfite sequencing PCR primers were designed by online MethPrimer software (www.urogene.org/methprimer/index.html). These primers were designed to recognize regions without CpG sites to avoid amplification bias of methylated versus unmethylated sequences. BSP validation experiments were conducted as follows: 500 ng of genomic DNA was converted using ZYMO EZ DNA Methylation-Gold Kit™ according to the manufacturer's instructions. After purification the converted DNA, PCR amplification was carried out in a final reaction volume of 50 µl consisting of 3 µl purified conversion fractions, 4 µl 2.5 mM dNTP, 5 µl 10×buffer, 1 µl BSP primers, 0.5 µl JumpStart™ Taq DNA Polymerase and 36.5 µl water and the following thermal cycling program was 94°C 1 min, 30cycles of 94°C 10 s, 58°C 30 s, 72°C 30 then prolong with 5 min at 72°C and products could be hold at 12°C. Primers are listed in Table S7. Following amplification, the PCR products were gel selected and purified using QIAquick gel extraction kit (Qiagen) and the purified PCR products were subcloned. The colonies from each region were sequenced on a 3730 genetic Analyzer (Applied Biosystems) to analyze the methylated cytosine level.

## Results

### Global gene expression profiling analysis

To examine the global gene expression profiles between cloned and control pigs, a transcriptome study for eight tissue samples was conducted using Affymetrix GeneChip® Porcine Genome Array. The data quality for each array, including homogeneity, variance, and RNA degradation was ascertained using affyQCReport (data not showed). After RMA normalization (Robust Multi-Array) and absent/present (A/P) calls [14], 18432 out of total 24123 probe sets remained for further analysis.

**Figure 1:**
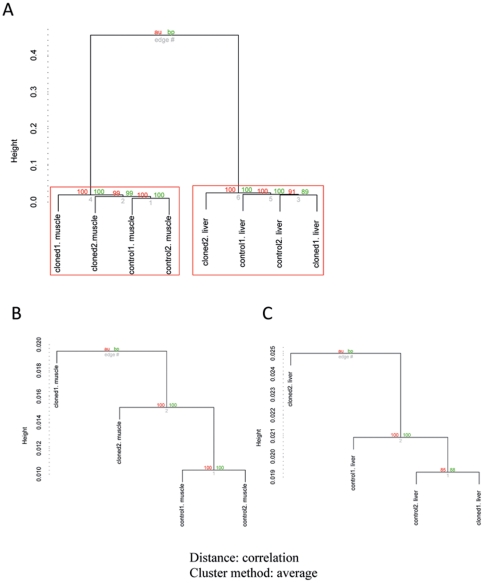
Hierarchical clustering of gene expression data from cloned and control pigs. The hierarchical clustering of gene expression data are shown for all 8 pig samples (A), only among the muscles (B), and only among the livers (C). *P* value of a cluster is a value between 0 and 1, indicating how strong the cluster is supported by data. *Pvclust* provides two types of *P* values (%) on the edge of the cluster: AU (Approximately Unbiased) *P* value and BP (Bootstrap Probability) *P* value. AU *P* values, shown in red, are computed by multiscale bootstrap re-sampling, and are better approximations to unbiased *P* values than the BP *P* value, shown in green, computed by normal bootstrap re-sampling. Clusters with AU *P* value larger than 95% are highlighted by rectangles and are strongly supported by data.

We performed hierarchical clustering on the gene expression profiles of eight samples using the *pvclust* method. As shown in Figure 1A, these eight samples were significantly grouped into two clusters. One cluster was comprised of muscle samples while the other was comprised of liver samples, showing typical tissue-specific patterns of gene expression. However, hierarchical clustering shows that there was no principal difference between cloned and control pigs neither in muscle (Figure 1B) nor in liver (Figure 1C), supported by relatively small value of height, a measurement of correlation distance. The small difference among the samples within each tissue was resulted from individual variations rather than the cloning effects. Thus, cloned pigs presented highly similar patterns of gene expression relative to controls.

**Figure 2:**
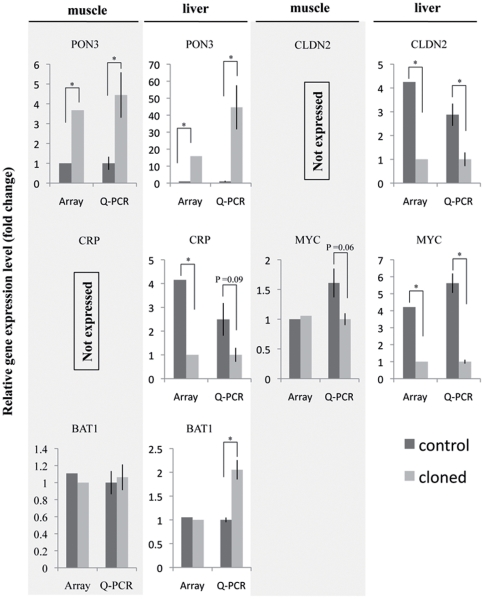
Q-PCR validation of differentially expressed gene found by microarray. Quantitative real time PCR was used to validate the gene expression of PON3, CLDN2, CRP, MYC, and BAT1 in muscle and liver by microarray. Relative gene expression level for Q-PCR was first normalized to the reference gene (Beta-Actin), and then calculated as fold changes to the sample, in which the gene expression level is the lowest. Fold changes of gene expression from microarray were plotted together with the Q-PCR results. Results were shown as Mean ± SE (duplicate). “*” represents p value<0.05 by ttest.

A few transcripts seemed differentially expressing (p value<0.05, fold change (FC)≥2) in cloned pigs versus controls. A total of 116 transcripts, which accounted for 0.63% of total transcripts, seemed differentially expressed in muscle samples. Of the 116 transcripts, 67 transcripts were expressed at higher level in muscle samples of cloned pigs, whereas 49 transcripts were more abundantly expressed in control samples (Table S1). In liver samples, 131 transcripts, which accounted for 0.71% of total transcripts, seemed differentially expressed. Of the 131 transcripts, 75 transcripts were expressed at higher level in samples from cloned pigs, whereas 56 transcripts were expressed at higher level in control samples (Table S2). Comparing to the total number of examined transcripts, the number of differentially expressed genes were remarkably low. However, the expressions of some genes were extremely altered (FC≥10) in muscle or liver of clone pigs, such as collagen type IV alpha 2 (COL4A2) (muscle, FC = +15.58), Serine/threonine-protein kinase tousled-like 2 (muscle, FC = +12.43), paraoxonase 3 (PON3) (liver, +15.81), and HSUP1 protein (liver, FC = −12.05). The top 10 most differentially expressed transcripts in cloned vs. control muscle or liver samples are shown in Table 1. To validate the differentially expressed genes found by microarrays, we selected four genes from Table 1 (PON3, CLDN2 (claudin 2), CRP (C-reactive protein, pentraxinrelated), and MYC (v-myc myelocytomatosis viral oncogene homolog (avian))) and one gene (BAT1 (HLA-B associated transcript), non-differentially expressed) and validate gene expression level by quantitative real time PCR (Q-PCR). There is good agreement between the microarray and Q-PCR data for PON3, CLDN2, CRP, and MYC expression in muscle or liver (Figure 2). However, Q-PCR showed that BAT1 expression was significantly higher expressed in liver of cloned pigs while microarray did not detect the difference.

**Table 1:** The 10 most differentially expressed genes in muscle and liver of cloned pigs compared to controls.

Top-10 significantly and differentially expressed genes in muscle and liver of cloned pigs in compared to normal ones at p<0.05, FC≥2					
Gene	Symbol	Affy Probe ID	FC^a^	P value	GO-biological process
**Muscle**					
collagen, type IV, alpha 2	COL4A2	Ssc.9939.1.A1_at	15.58	4.25E-07	extracellular matrix organization and biogenesis;regulation of transcription\, DNA-dependent
Serine/threonine-protein kinase tousled-like 2 (EC 2.7.1.37)	TLK2	Ssc.30422.1.A1_at	12.43	5.16E-09	cell cycle;chromatin modification;intracellular signaling cascade;protein amino acid phosphorylation;regulation of chromatin assembly/disassembly;response to DNA damage stimulus
enabled homolog (Drosophila)	ENAH	Ssc.12686.1.A1_at	9.53	3.09E-06	cellular component organization and biogenesis
Nanos homolog 1 (NOS-1)	NANOS1	Ssc.29246.1.A1_at	8.12	7.49E-06	biological_process unknown
tumor necrosis factor receptor superfamily, member 12A	TNFRSF12A	Ssc.1864.1.A1_a_at	6.81	1.81E-05	angiogenesis;apoptosis;cell motility
ankyrin repeat domain 1 (cardiac muscle)	ANKRD1	Ssc.7678.1.A1_at	6.80	2.76E-04	defense response;signal transduction
enabled homolog (Drosophila)	ENAH	Ssc.24086.1.A1_at	6.75	1.48E-05	cellular component organization and biogenesis
enabled homolog (Drosophila)	ENAH	Ssc.3771.1.A1_at	6.40	1.76E-05	cellular component organization and biogenesis
peroxisomal trans-2-enoyl-CoA reductase	PECR	Ssc.30628.1.S1_at	−7.86	8.28E-08	apoptosis;enterobactin biosynthesis
cytochrome P450, family 3, subfamily A, polypeptide 4	CYP3A4	Ssc.204.1.S1_at	−7.99	4.26E-06	electron transport;lipid metabolism;oncogenesis;xenobiotic metabolism
**Liver**					
paraoxonase 3	PON3	Ssc.21810.1.S1_at	15.81	1.14E-06	response to external stimulus
Protocadherin 15 precursor	PCDH15	Ssc.30063.1.A1_at	9.78	1.21E-07	system process; cell adhesion
cytochrome P450, family 3, subfamily A, polypeptide 4	CYP3A4	Ssc.929.1.S1_at	8.28	4.00E-06	electron transport;lipid metabolism;oncogenesis;xenobiotic metabolism
collagen, type IV, alpha 2	COL4A2	Ssc.9939.1.A1_at	4.96	3.55E-05	extracellular matrix organization and biogenesis;regulation of transcription\, DNA-dependent
C-reactive protein, pentraxin-related	CRP	Ssc.16157.1.S1_at	−4.15	4.64E-03	acute-phase response;inflammatory response
v-myc myelocytomatosis viral oncogene homolog (avian)	MYC	SscAffx.8.1.S1_s_at	−4.21	2.91E-05	cell cycle arrest;iron ion homeostasis;pathogenesis;regulation of transcription from Pol II promoter
claudin 2	CLDN2	Ssc.19842.1.S1_at	−4.25	3.71E-05	protein complex assembly
prominin 1	PROM1	Ssc.4065.1.A1_at	−4.71	3.01E-06	vision
Vacuolar ATP synthase subunit G 2	ATP6V1G2	Ssc.12005.1.A1_at	−4.96	2.89E-05	cellular iron ion homeostasis
Unknown	Q8N5E3	Ssc.1256.1.A1_at	−12.05	2.41E-08	Unknown

Gene symbol, Affymetrix Probe ID (Porcine), fold change (FC), statistical *P* value, and gene ontology of biological processes are shown. Any feature with a P value<0.05 and FC not less than 2 was considered to be significantly and differentially expressed.

**Figure 3:**
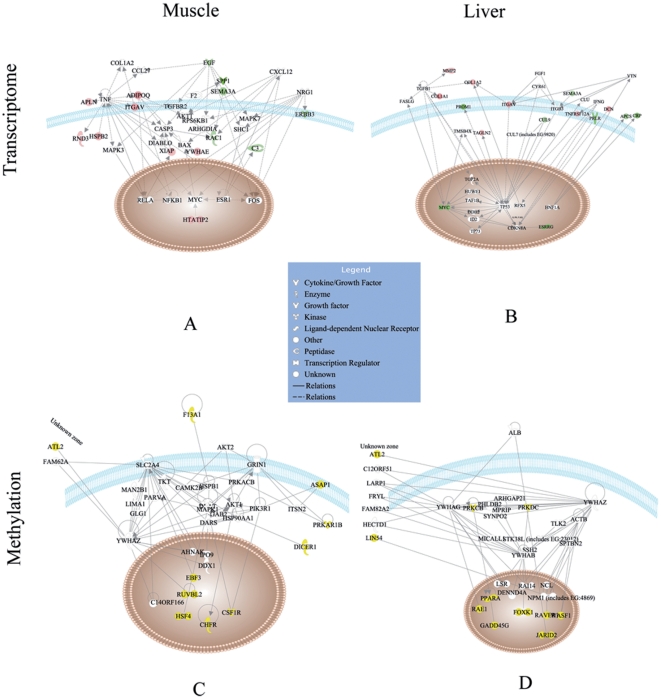
Representative molecular networks in IPA analysis of differential DNA methylation and gene expression. The representative molecular networks of IPA analysis based on genomic loci for differential DNA methylation and gene expression in muscle and liver from cloned and control pigs. Figure 5 A and B shows the molecular network of “cellular growth and proliferation and cancer” that are representative of the differentially expressed genes in muscle and liver, respectively. Figure 5 C and D shows the molecular networks of “muscular and hepatic development” that are representative of differentially methylated loci in muscle and liver, respectively. Molecules represented by yellow color have different DNA methylation. Molecules filled in red or green color are more highly or sparsely expressed in cloned pigs respectively. Molecules in white color are from the IPA network database.

We then used IPA software to investigate whether there was any specific molecular network over-represented for the differentially expressed genes in muscle or liver of the cloned pigs. 22 out of 116 genes in muscle and 26 out of 131 genes in liver were eligible for network analysis. As shown in Table S3, six and nine networks were significantly over-represented for the differentially expressed genes in muscle and liver, respectively (network score ≥2, the network's score = −log (right-tailed Fisher's Exact Test *P* value)). Among these networks, one molecular network with a function related to cellular growth and proliferation, cell death, and cancer was over-represented both in muscle (14 genes involved, network score = 19) (Figure 3A) and liver (15 genes involved, network score = 21) (Figure 3B). However, no significant molecular networks (network score>10) related to tissue development was highlighted. Most of the other highlighted networks only obtained a network score of 2. These results were in good agreement with the normal phenotypes of these cloned pigs.

### Sequencing and mapping results of methylation-tag libraries

We constructed a virtual *Mlu*I/*Nla*III tag library for methylation-tag mapping in the pig genome. *In silico* analyses showed that there are 23759 recognition sites for the *Mlu*I restriction enzyme in pig genome, and we obtained 47132 virtual fragments by simulating the digestion of *Mlu*I and *Nla*III on pig genome under the hypothesis that all cytosines were unmethylated. 193 fragments between adjacent *Mlu*I recognition sites lacked *Nla*III recognition site inside. The length distribution of the virtual *Mlu*I/*Nla*III fragments is presented in Figure S1. The majority of fragments are shorter than 1000 bp. By defining CGIs according to three criteria (GC content>50%, ratio of the observed CpGs to the expected CpGs>0.6, length>500 bp), we predicted that 7028 *Mlu*I recognition sites (29.6% of all *Mlu*I recognition sites) are located in promoter-associated CGIs in the pig genome using CpGProD (CpG Island Promoter Detection) software [24]. According to Repeatmasker [25] (Smit 1996), 4699 *Mlu*I recognition sites (19.8% of all *Mlu*I recognition sites) are located within repeat sequences.

Results of conventional clone sequencing confirmed that correct tag inserts were found in the vast majority of sample clones, while none of the bead-controls presented correct tag inserts (Data not shown). After Solexa sequencing, we applied the MAQ (Mapping and Assembly with Qualities) algorithm [18], [19] for mapping the tags to genome, and we obtained 7728424 tags with MQ0, and 1689970 tags with MQ20. On average 77.8% of the tags obtained from the samples were mapped to *Mlu*I recognition sites in the pig genome. Among the 23759 *Mlu*I recognition sites, there were 358 “empty” sites to which no tags could be mapped back, giving 98.5% mappable sites. Among the 4699 sites located in repeat sequences and the 7028 sites located in CGIs, there were 64 and 63 “empty” sites, respectively giving 98.6% and 99.1% mappable sites. All information of the libraries for the samples is available in Table S4.

### Overview of DNA methylation pattern

**Figure 4:**
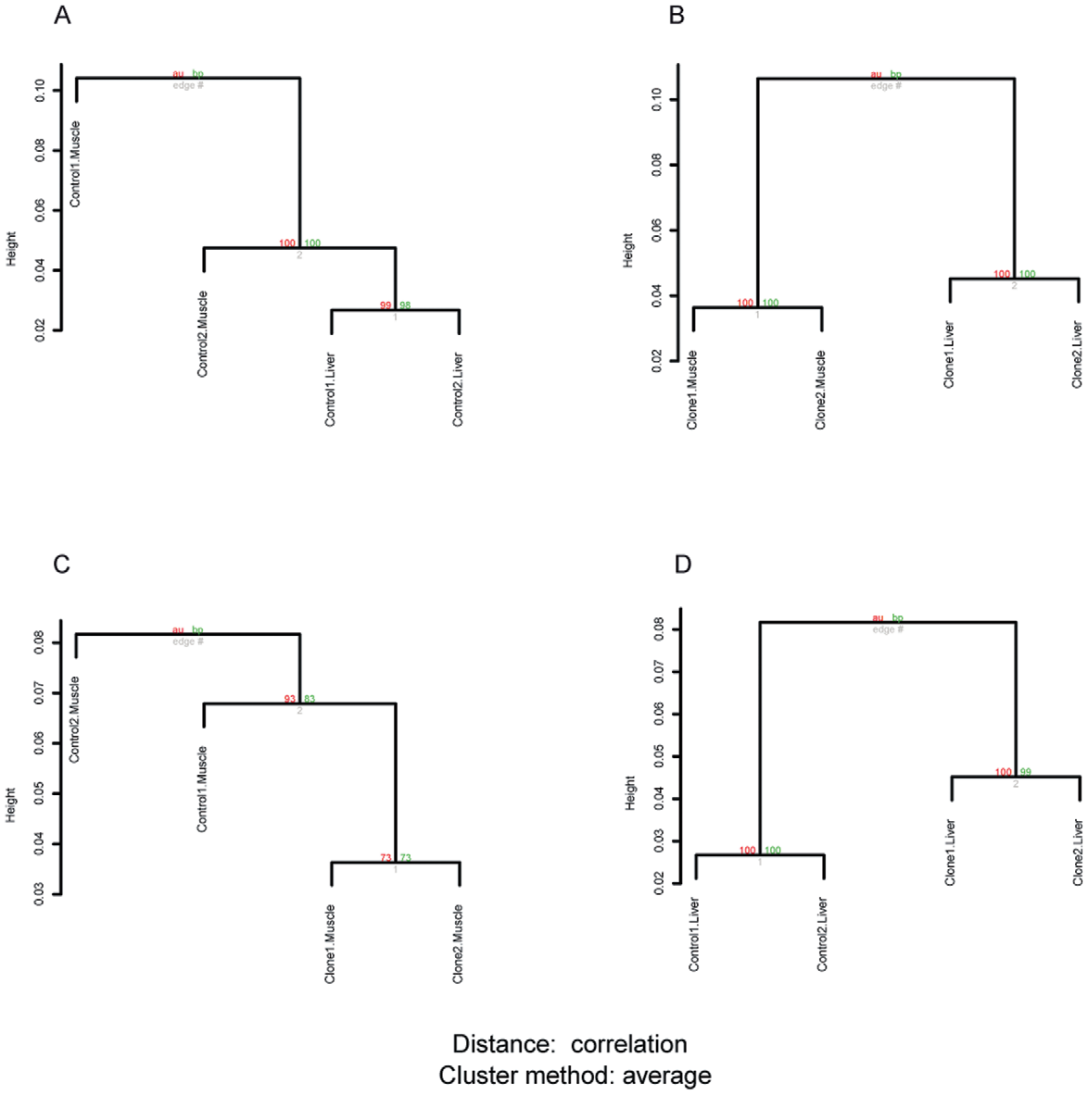
Hierarchical clustering of DNA methylation data from cloned and control pigs. The hierarchical clustering of DNA methylation data are shown for all 8 pig samples. In A, the clustering result is shown for four controls samples, while in B, it is shown for four cloned samples. In C and D, the clustering results for four muscle samples and four liver samples are shown, respectively. The meaning of AU *P* value and BP *P* value is the same as in Figure 1.

We examined the genome-wide DNA methylation patterns of muscle and liver tissues based on categorized methylation tags. Figure 4 provides an overview of the hierarchical clustering of DNA methylation patterns for 14094 tags that are located in non-repeat sequences. From a pretest of the clustering analysis, it was clear the genome-wide DNA methylation patterns exhibited bigger differences than the gene expression pattern. Thus, we clustered the samples according to pig types (cloned pigs or controls) as well as to tissue types (muscle or liver), in order to see the cluster structure more clearly. As shown in the tree presentations, there were differences in methylation patterns both with respect to different tissue types and with respect to cloned pigs versus controls. The two types of tissue were split into two clusters in cloned pigs, supported by high-value of AU/BP *P* values (Figure 4B). In fact the cloned pigs showed clearer tissue difference in the DNA methylation pattern than the controls did (Figure 4A). For the clustering with respect to pig types, the liver tissue of cloned pigs were clearly separated from controls (Figure 4D), but in muscle tissue, the difference between cloned pigs and controls was more moderate, as suggested by low AU/BP *P* values (Figure 4C).

For the analysis of the repeat families, we isolated the tags in different categories of repeat sequences and analyzed them according to MQ0 tag counts. The results of the tags collected in muscle and liver genomes from cloned and control pigs are summarized in Table 2. Notably, the majority of the repeat categories in both muscle and liver did not show significant differences between cloned and control pigs. The only exception was the short interspersed nuclear element (SINE/tRNA-Glu) in muscle with a FDR adjusted *P* value of 0.049 (FDR *P* value<0.05).

**Table 2:** Summary of DNA methylation tag information in repeat sequences.

RepeatClass	Tag Sites Counts	Normalized Tag Numbers of Muscle				Normalized Tag Numbers of Liver			
		Control mean	Clone mean	P-value	FDR-Pvalue	Control mean	Clone mean	P-value	FDR-Pvalue
SINE/tRNA-Glu	1588	519	568	0.0017	0.0494	349	324	0.0017	0.0509
LINE/L1	1155	457	441	0.0073	0.1064	287	247	0.0035	0.0517
LTR/ERVL-MaLR	261	466	454	0.2271	0.9563	303	245	0.0908	0.6649
DNA/hAT-Charlie	230	557	674	0.3304	1.0000	367	360	0.3594	1.0000
SINE/MIR	224	440	472	0.2543	0.9563	291	261	0.3422	1.0000
DNA/Maverick	183	271	279	0.2542	0.9563	186	162	0.3623	1.0000
LTR/ERV1	152	348	338	0.2612	0.9563	221	188	0.2710	1.0000
LINE/L2	146	416	438	0.4044	1.0000	264	231	0.2302	1.0000
LTR/ERVL	140	319	309	0.2576	0.9563	227	193	0.4301	1.0000
Other	617	376	395	0.0188	0.1831	242	212	0.0179	0.1743

Summary of the statistical results for the normalized tag numbers collected for different repeat categories in muscle and liver genomes of cloned and control pigs. Both raw P values and FDR adjusted P values are presented in the table.

### Pair-wise comparison of DNA methylation status in individual genomic loci

**Figure 5:**
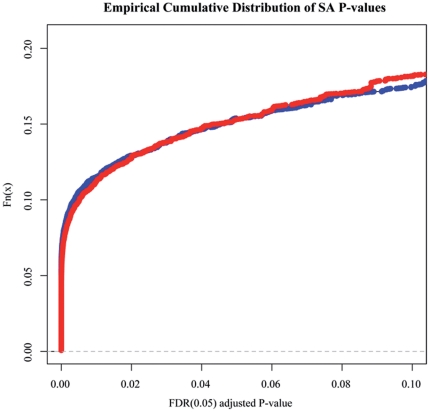
Empirical cumulative curves of SA *P* values for DNA methylation. The empirical cumulative curves of SA *P* values for DNA methylation of individual genomic loci in cloned and control pigs for muscle and liver tissue, respectively. Blue curve represents the result from muscle samples and red curve represents the result from liver samples.

**Figure 6:**
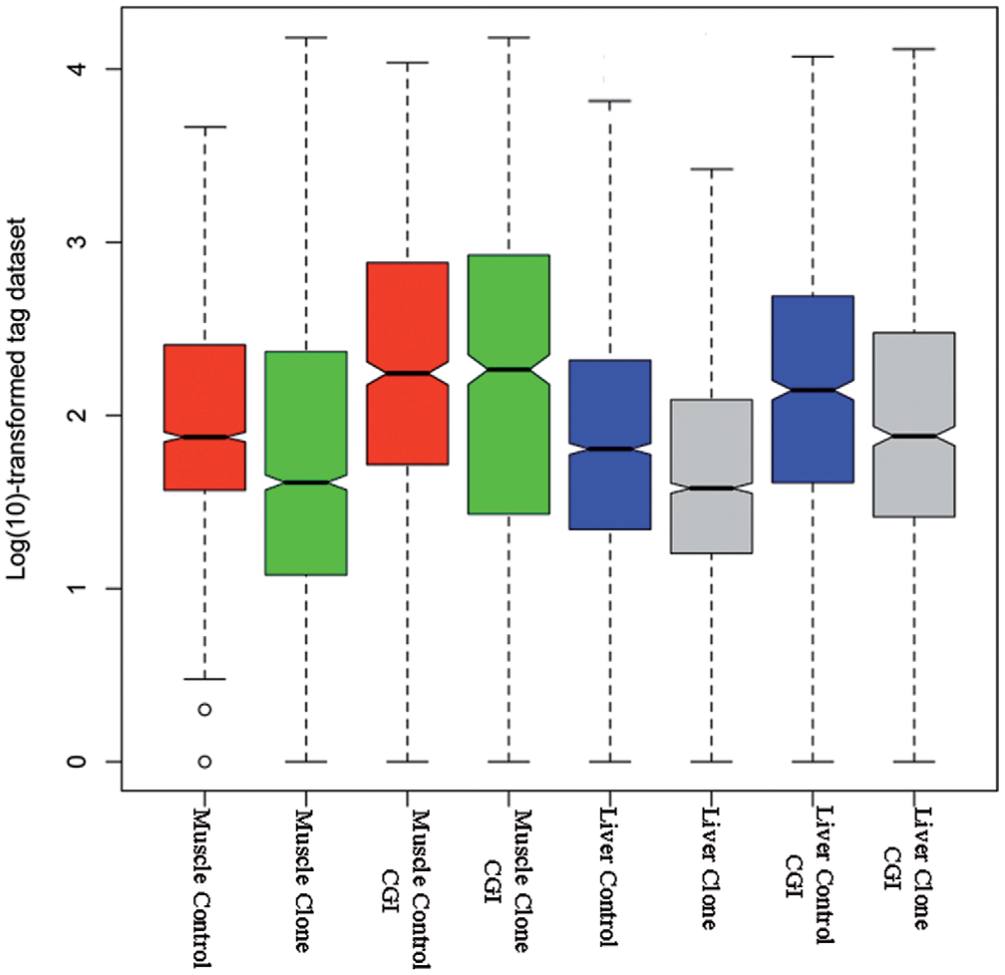
Boxplot of the distribution of all tag counts for significantly different methylation loci. Boxplot of the distribution of all tag counts for loci with significantly different methylation in muscle and liver samples of cloned and control pigs. First quartile (*x*_.25_), median (*x*_.50_), and third quartile (*x*_.75_) of the dataset, are represented by the boxes. All data are log(10)-transformed. Red and green boxes represent significantly different tag loci of clone and controls in muscle while blue and grey boxes represent significantly different tag loci in liver, respectively. All significantly different tag loci as well as those located in promoters associated with CGIs are presented. Lower log(10)-transformed data suggest higher methylation levels as all tags are referred to unmethylated *Mlu*I sites.

Though the hierarchical clustering results showed overall similarity in DNA methylation patterns of non-repeat sequences with respect to cloned pigs versus controls, cloned pigs and controls were further separated in subclusters. This result suggests that a portion of the non-repeat sequences still has different methylation status. Thus, we performed significance analysis for pair-wise comparisons by a Poisson-based algorithm to identify the individual genomic loci that have significantly different DNA methylation status in cloned and control pigs. Figure 5 presents the empirical cumulative curves based on the FDR adjusted *P* values. The result indicates a similar level of differently methylated cytosines for liver and muscle in cloned and control pigs. We identified 2167 and 2157 significantly different tag sites out of 14094 ones in muscle and liver, respectively (FDR *P* value<0.05), with a percentage of 15.38% and 15.30%. 747 and 881 tag sites out of the 2167 and 2157 ones were located in promoters associated with CGIs. Figure 6 shows the general distribution of the log-transformed data for both the total number of significantly different tag sites and the tags in the promoters associated with CGIs. Heavier average methylation was observed in cloned pigs compared to controls. We tried to identify the genes neighboring the significantly different sites. However, only 118 and 122 tag sites could be annotated to neighboring genes due to limited annotation information. Some genes had more than one tag site, which resulted in a total annotation of 113 and 120 genes in muscle and liver, respectively. All information related with Poisson-based Significance Analysis (SA) of non-repeat sequences are listed in Table S5. Ingenuity Pathway Analysis (IPA) showed that 74 out of 113 genes in muscle and 89 out of 120 genes in liver were eligible for molecular network analysis, and IPA enriched three main networks in each tissue (network score>2, the network's score = −log (right-tailed Fisher's Exact Test *P* value)) (Table S6). Notably, one main network (network score = 14) in muscle including 10 eligible genes (ASAP1, ATL2, CHFR, CSF1R, DICER1, EBF3, F13A1, HSF4, PRKAR1B, RUVBL2) was “skeletal and muscular function and development”, while one main network (network score = 16) in liver including 11 eligible genes (ATL2, FOXK1, GADD45G, JARID2, LIN54, PPARA, PRKCB, PRKDC, RAE1, RAVER1, WASF1) was “hepatic system function and development” (Figure 3C and 3D). Randomly seven genes either from muscle or liver were chosen for validation of the differentially methylated sites by bisulfite sequencing PCR, less discrepancy of DNA methylation between clones and controls were observed based on Fisher's Exact Test (Table S8), probably due to less sequencing depth.

Besides, the “gene expression and cellular growth and proliferation” network was significantly enriched with differentially methylated genes in muscle and liver, although genes involved were mostly different. This suggested that DNA methylation status in the promoter regions of certain cellular growth-related genes was altered in cloned pigs, indicating a potential risk of abnormal gene expression.

We tried to correlate the DNA methylation changes with the gene expression profile changes by performing a cross-comparison between the genes neighboring the significantly different methylation sites and the differentially expressed genes, especially for the genes in the molecular networks highlighted by differentially expressed genes and genes containing differentially methylated genomic loci. We were not able to identify genes that differed significantly both in DNA-methylation and gene expression status between cloned and control pigs.

## Discussion

In order to identify the mechanisms related to the inefficiency of SCNT technology, we compared global gene expression profiles between cloned animals and conventionally bred controls. A limited number of studies have already addressed this problem and demonstrated that SCNT embryos were able to undergo significant nuclear remodeling and their gene expression patterns were similar to those of artificial insemination (AI) embryos with only a small set of differentially expressed genes [26], [27], [28], [29], [30], [31], [32], [33]. However, these studies were mostly restricted to cloned embryos. Few studies have been conducted on full-term cloned pigs that have survived the full pressure from SCNT and embryonic development. In the present study, we compared the global gene expression profiles in muscle and liver tissues between 6-week-old cloned pigs without any detected phenotype abnormalities and their age-matched conventionally bred controls. It was revealed by clustering analysis that a clear tissue-specific gene expression pattern was established both in the cloned pigs and the controls, and the global gene expression profiles in cloned pigs were remarkably similar to those of control pigs, both in muscle and liver. The analysis of differentially expressed genes (DEGs) in cloned and control pigs was consistent with the clustering analysis. Only a small portion of genes were differentially expressed in cloned pigs. However, these DEGs may also result from other factors, such as breed specific genetic background, coincident of individual transcriptome variations (Figure 1B and 1C). The functional analysis on these DEGs didn't show significant enrichment in molecular networks related to tissue development (network score>10). However, one molecular network identified by IPA analysis, “cellular growth and proliferation, cell death and cancer”, was over-represented among the differentially expressed genes in both muscle and liver (Figure 3A and 3B).

If cloned animals with abnormal phenotypes can be subjected to conventional breeding, all their offspring tend to show normal phenotypes, showing that the defects coming from the cloning procedure are due to inefficient or aberrant nuclear remodeling after SCNT rather than to genetic mutations [34]. Many studies have examined cloned mouse and bovine embryos and identified epigenetic alterations in X chromosome inactivation and imprinting. Differences in DNA methylation both in general and of specific genes and repeat sequences have been reported [35]. One previous study observed similar demethylation activity in cloned and control pig embryos with respect to two repetitive sequences: a centromeric satellite and the PRE1 short interspersed element (SINE) [36]. Another study reported that DNA methylation patterns of many CpG islands differed between cloned pig embryos and normal control embryos [37]. Moreover, a study examined the DNA methylation pattern of the IFG2-H19 differentially methylated regions (DMRs) and found that the CTCF3 and DMR2 loci of the IGF2 gene showed abnormal methylation in fetus of cloned pigs [38].

In the present study, we have identified a similar methylation status of repetitive sequences in both muscle and liver tissues of cloned and control pigs (Table 2). However, repetitive sequences constitute a large proportion of the genome that was not fully covered in our analysis due to technological limitations [12]. Therefore it is difficult to draw absolute general conclusions about the DNA methylation pattern from our data. We have mainly focused on the analysis of non-repeat sequences in our analysis. We corroborated previous observations that a higher level of DNA methylation was found in non-repeat sequences in cloned embryos, as some differentially methylated loci in non-repeat sequences were detected with an averagely higher level of methylation in cloned pigs (Figure 6). Differences between regions of repetitive and unique sequences could indicate that reprogramming of DNA methylation take place independently in different genomic regions and by different mechanisms [39]. Furthermore, the differential methylation loci suggest that even cloned pigs with normal phenotype have incomplete or aberrant reprogramming of DNA methylation in some genomic regions of the non-repeat sequences.

DNA methylation plays an important role in tissue differentiation, and numerous tissue-specific differentially methylated regions (T-DMR) have been reported throughout the mammalian genome [40]. Similarly, tissue-specific DNA methylation patterns were revealed in our hierarchical clustering analysis for the two tissues, muscle and liver. The clustering results also show a more clear tissue difference in cloned pigs than in controls. Interestingly, the molecular networks for muscular or hepatic tissue development were highlighted in the IPA function analysis of genes containing differential DNA methylation loci in their promoter regions. It is reasonable to infer that normally the methylation of these genes, involved in tissue development, should contribute most to the tissue-specific difference in the clustering structure. In the case of cloned pigs, the tissue difference might in fact be enlarged during reprogramming and somatic selection. Based on the differential DNA methylation of some genes in the molecular networks related to muscular or hepatic system development, we expected that similar molecular networks would also be highlighted in genes with differential expression data. However, in the expression data, no significant molecular networks were over-represented with relation to muscular or hepatic system development.

We further compared genes neighboring the significantly different methylation sites with the set of differentially expressed genes. No overlapping genes could be detected (with both DNA methylation change and gene expression alterations). One explanation could be that only one-third of the genes in mammalian genome are regulated by DNA methylation [41]. Moreover, the different information contents of the two applied technologies (methylation and expression profiling) severely hamper such comparative analysis. Only one molecular network “cellular growth and proliferation, cell death and cancer” was over-represented in DEGs found by methylation and expression profiling.

The global gene expression profiles for all samples presented much less differences when compared with the DNA methylation patterns. Also, the observation that some genomic loci existed with a higher degree of DNA methylation should be considered together in relation to the normal phenotype of these cloned pigs. This implies that the developing cloned pigs can tolerate more alterations of DNA methylation in those genes than expected. One possible explanation could be that the alteration of DNA methylation in certain regions will not change the expression level of the genes, as many methylation tags are located in the regions, for which the regulation mechanism by DNA methylation is not yet clarified. Another possible explanation might be that most of the influenced genes are functionally irrelevant, while the important genes are correctly reprogrammed and expressed in phenotypically normal cloned pigs.

It remains unclear whether the higher methylation state is biologically important and whether it increases the risk of developmental or pathological disorders. However, it is reasonable to infer that DNA methylation reprogramming in SCNT to some extent is a stochastic process. If the DNA methylation reprogramming is not even fulfilled completely in phenotypically normal cloned pigs, there is clearly a risk in cloned pigs not selected for a phenotypically normal appearance. In aborted and/or abnormal pigs, aberration in DNA methylation profiles and gene expression are expected to be more severe. Further work is required to study the epigenetic reprogramming and tissue development in cloned pigs with abnormal phenotypes as well as in neonatally dead or aborted cloned pigs.

## Supporting Information

Figure S1 **Tag distribution for DNA methylation sequencing.** Figure A presents the length distribution of DNA fragments digested with *MluI* in a digital enzyme cutting simulation of the pig genome. Table B presents the obtained sequence reads from MMSDK and the mapping results for cloned piglets and control samples, respectively.
(PDF)

Table S1 **List of significantly (** ***P*** **<0.05) and differentially (FC≥2) expressed genes in muscle of cloned pigs.**
(PDF)

Table S2 **List of significantly (** ***P*** **<0.05) and differentially (FC≥2) expressed genes in liver of cloned pigs.**
(PDF)

Table S3 **IPA molecular network analysis of significantly and differentially expressed genes in muscle or liver of cloned pigs.** Network score≥2 was considered as over-represented.
(PDF)

Table S4 **Summary of all raw data of MMSDK, including DNA fragment ID, counts of mapped tags by low-confidence MQ0 and high-confidence MQ20 standards for all samples.** Promoters associated with CGIs, repeat sequence information and annotation information are also given.
(XLSX)

Table S5 **Summary table of SA analysis of DNA methylation in unique sequences, including DNA fragment ID, counts of mapped tags by high-confidence MQ20 standards, CGIs location, annotation information and SA** ***P*** **values.**
(PDF)

Table S6 **Summary table of molecular network analysis by Ingenuity Pathways Analysis of genes neighboring significantly-differently methylated tags in muscle and liver.**
(PDF)

Table S7 **Primer sequences using for Q-PCR.**
(XLSX)

Table S8 **Bisulfite sequencing PCR validation of differential methylation.** In the MMSDK result, the numbers in the column of control 1 indicates the tag counts of control sample 1. In the BSP result, two samples of control pigs were pooled and the numbers in the column of control-sup indicates the clone counts that support the methylated C of control sample 1, while the Control-nonsup means for the reads that doesn't support.
(XLSX)
