# Supplementary material for: Comparison of Gene Expression and Genome-Wide DNA Methylation Profiling between Phenotypically Normal Cloned Pigs and Conventionally Bred Controls
Source: PLoS One. 2011 Oct 11;6(10):e25901. doi: 10.1371/journal.pone.0025901 (PMC3191147; doi:10.1371/journal.pone.0025901)
Supplement: Figure S1 — Tag distribution for DNA methylation sequencing. Figure A presents the length distribution of DNA fragments digested with MluI in a digital enzyme cutting simulation of the pig genome. Table B presents the obtained sequence reads from MMSDK and the mapping results for cloned piglets and control samples, respectively. (PDF) [file pone.0025901.s001.pdf]

Supplemental file 4

A

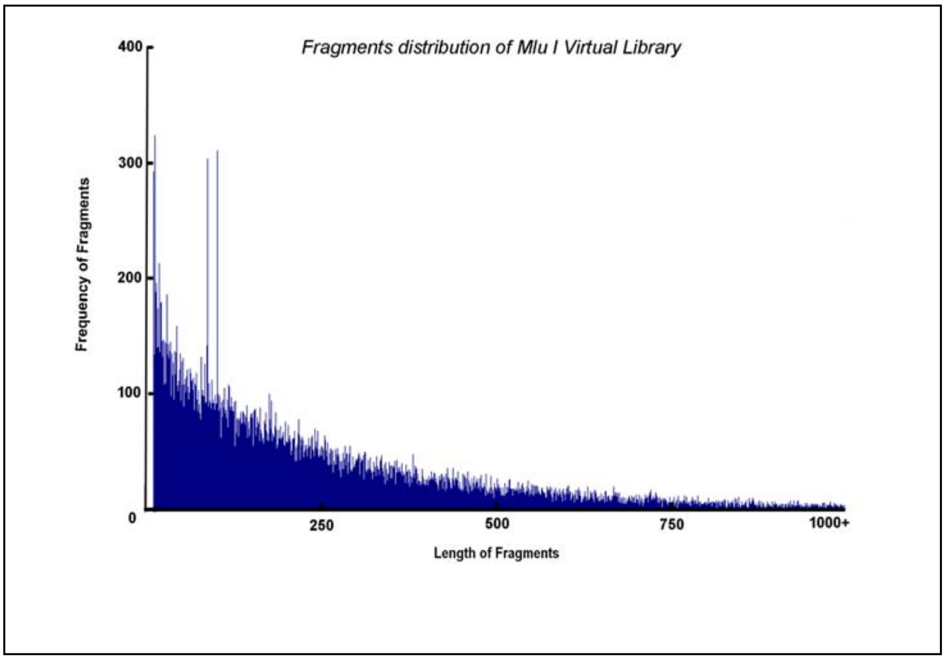

B

sample	Total	Mapped(MQ0)	%	Mapped(MQ20)	%
Muscle-Control1	11506568	8891119	77.27%	2220779	19.30%
Liver-Control1	10103313	7746433	76.67%	1546643	15.31%
Muscle-Control2	10584962	8365627	79.03%	1618015	15.29%
Liver-Control2	7829938	6110439	78.04%	1202819	15.36%
Muscle-Clone1	11874159	8979204	75.62%	2222131	18.71%
Liver-Clone1	10702004	8396078	78.45%	1723660	16.11%
Muscle-Clone2	11416946	9000938	78.84%	2198265	19.25%
Liver-Clone2	5581501	4337557	77.71%	787449	14.11%
AVERAQGE	9949924	7728424	77.80%	1689970	16.70%
