# Supplementary material for: Comparison of Gene Expression and Genome-Wide DNA Methylation Profiling between Phenotypically Normal Cloned Pigs and Conventionally Bred Controls
Source: PLoS One. 2011 Oct 11;6(10):e25901. doi: 10.1371/journal.pone.0025901 (PMC3191147; doi:10.1371/journal.pone.0025901)
Supplement: Table S1 — List of significantly ( P <0.05) and differentially (FC≥2) expressed genes in muscle of cloned pigs. (PDF) [file pone.0025901.s002.pdf]

Supplementary File 1

Differentially expressed genes between cloned and normal pigs in muscle at $p < 0.05$, FC ⩾ 2

Gene	Symbol	Affy Probe ID	FC	p value	GO-biological process
**High expressed**					
collagen, type IV, alpha 2	COL4A2	Ssc.9939.1.A1_at	15.582	4.25E-07	extracellular matrix organization and biogenesis;regulation of transcription\, DNA-dependent
Serine/threonine-protein kinase tousled-like 2 (EC 2.7.1.37) (Tousled-like kinase 2) (PKU alpha).	TLK2	Ssc.30422.1.A1_at	12.429	5.16E-09	cell cycle;chromatin modification;intracellular signaling cascade;protein amino acid phosphorylation;regulation of chromatin assembly/disassembly;response to DNA damage stimulus
enabled homolog (Drosophila)	ENAH	Ssc.12686.1.A1_at	9.528	3.09E-06	cellular component organization and biogenesis
Nanos homolog 1 (NOS-1)	NANOS1	Ssc.29246.1.A1_at	8.116	7.49E-06	biological_process unknown
tumor necrosis factor receptor superfamily, member 12A	TNFRSF12A	Ssc.1864.1.A1_a_at	6.807	1.81E-05	angiogenesis;apoptosis;cell motility
ankyrin repeat domain 1 (cardiac muscle)	ANKRD1	Ssc.7678.1.A1_at	6.797	2.76E-04	defense response;signal transduction
enabled homolog (Drosophila)	ENAH	Ssc.24086.1.A1_at	6.750	1.48E-05	cellular component organization and biogenesis
enabled homolog (Drosophila)	ENAH	Ssc.3771.1.A1_at	6.403	1.76E-05	cellular component organization and biogenesis
protein tyrosine phosphatase, receptor type, K	PTPRK	Ssc.13820.1.A1_at	4.712	4.10E-03	protein amino acid dephosphorylation;transmembrane receptor protein tyrosine phosphatase signaling pathway
ankyrin repeat domain 1 (cardiac muscle)	ANKRD1	Ssc.10429.1.S1_at	4.428	5.63E-05	defense response;signal transduction
Protocadherin 15 precursor	PCDH15	Ssc.30063.1.A1_at	4.278	5.36E-06	hearing;homophilic cell adhesion;vision

tumor necrosis factor receptor superfamily, member 12A	TNFRSF12A	Ssc.1864.1.A1_at	3.687	1.43E-05	angiogenesis;apoptosis;cell motility
paraoxonase 3	PON3	Ssc.21810.1.S1_at	3.679	4.14E-04	response to external stimulus
neuronal calcium binding 2	NECAB2	Ssc.5292.1.S1_at	3.652	1.15E-04	biological_process unknown
chloride intracellular channel 5	CLIC5	Ssc.21139.1.S1_at	3.478	1.09E-02	chloride transport;pregnancy
heat shock 70kDa protein 6 (HSP70B')	HSPA6	Ssc.5145.2.S1_at	3.473	8.87E-03	response to biotic stimulus
adipose most abundant gene transcript 1	APM1	Ssc.18549.1.S1_at	3.439	3.57E-03	energy pathways
chloride intracellular channel 5	CLIC5	Ssc.21139.2.S1_at	3.353	6.14E-03	chloride transport;pregnancy
trichorhinophalangeal syndrome I	TRPS1	Ssc.30243.1.A1_at	3.175	2.70E-05	regulation of transcription\, DNA-dependent
membrane-spanning 4-domains, subfamily A, member 7	MS4A7	Ssc.1203.1.S1_at	3.022	7.57E-03	cell communication
KIAA0826 protein	KIAA0826	Ssc.14247.2.A1_at	2.999	3.34E-04	RAS protein signal transduction
uronyl-2-sulfotransferase	UST	Ssc.12009.1.A1_at	2.887	1.37E-02	protein amino acid sulfation
heat shock 70kDa protein 6 (HSP70B')	HSPA6	Ssc.5145.1.S1_a_at	2.858	1.72E-02	response to stress
integrin, alpha V (vitronectin receptor, alpha polypeptide, antigen CD51)	ITGAV	Ssc.6737.2.A1_at	2.857	5.58E-05	cell-matrix adhesion;integrin-mediated signaling pathway
cardiomyopathy associated 1	CMYA1	Ssc.19093.1.S1_at	2.856	2.15E-03	biological_process unknown

chloride intracellular channel 4	CLIC4	Ssc.14592.2.A1_at	2.771	1.28E-03	chloride transport
proteasome (prosome, macropain) subunit, beta type, 5	PSMB5	Ssc.21655.1.A1_at	2.666	2.17E-03	ubiquitin-dependent protein catabolism
LIM and cysteine-rich domains 1	LMCD1	Ssc.15397.2.S1_at	2.659	1.69E-05	negative regulation of transcription from RNA polymerase II promoter
syntaxin binding protein 5 (tomosyn)	STXBP5	Ssc.27354.1.S1_at	2.649	6.32E-04	vesicle-mediated transport
ribosomal protein L38	RPL38	Ssc.28256.1.A1_at	2.644	2.10E-05	protein biosynthesis
Novel protein (Fragment)	DNAH14	Ssc.6036.2.S1_at	2.547	8.98E-05	biological_process unknown
cellular retinoic acid binding protein 2	CRABP2	Ssc.24250.1.S1_at	2.541	5.50E-03	epidermal differentiation;regulation of transcription\, DNA-dependent;signal transduction;transport
Novel protein (Fragment)	DNAH14	Ssc.17766.1.A1_at	2.502	4.20E-03	biological_process unknown
heat shock 27kDa protein 2	HSPB2	Ssc.19588.2.A1_at	2.500	5.47E-03	response to stress
myosin light chain 1 slow a	MLC1SA	Ssc.26508.1.S1_at	2.434	3.92E-02	muscle development
retinoid binding protein 7	CRBPIV	Ssc.27385.1.S1_at	2.407	5.84E-04	transport
farnesyltransferase, CAAX box, beta	FNTB	Ssc.5679.1.S1_at	2.393	1.41E-03	protein amino acid farnesylation;proteolysis and peptidolysis
KIAA1411	KIAA1411	Ssc.17821.1.A1_at	2.378	2.37E-03	lipid metabolism;proteolysis and peptidolysis
CDC10 cell division cycle 10 homolog (S. cerevisiae)	CDC10	Ssc.1837.2.A1_at	2.319	3.44E-02	cell cycle;cytokinesis
ankyrin repeat domain 2 (stretch responsive muscle)	ANKRD2	Ssc.18494.2.A1_at	2.294	7.05E-03	muscle development

BENE protein	BENE	Ssc.12145.1.A1_at	2.293	1.91E-03	homeostatic process
death-associated protein kinase 3	DAPK3	Ssc.4309.1.A1_at	2.271	4.82E-04	induction of apoptosis;protein amino acid phosphorylation;protein kinase cascade
Baculoviral IAP repeat-containing protein 4	BIRC4	Ssc.8950.1.A1_at	2.270	2.40E-04	anti-apoptosis;cytoskeleton organization and biogenesis
HIV-1 Tat interactive protein 2, 30kDa	HTATIP2	Ssc.4271.1.S1_at	2.260	1.74E-03	anti-apoptosis;induction of apoptosis;regulation of transcription from Pol II promoter
major histocompatibility complex, class II, DR beta 3	HLA-DRB3	Ssc.210.6.S1_x_at	2.242	3.41E-03	antigen presentation\, exogenous antigen;antigen processing\, exogenous antigen via MHC class II;pathogenesis;perception of pest/pathogen/parasite;signal transduction
LIM and cysteine-rich domains 1	LMCD1	Ssc.15397.1.S1_at	2.234	7.52E-04	negative regulation of transcription from RNA polymerase II promoter; nucleobase, nucleoside, nucleotide and nucleic acid metabolic process
myosin binding protein C, slow type	MYBPC1	Ssc.25877.1.S1_at	2.232	2.72E-04	cell adhesion;muscle development;striated muscle contraction
tyrosine 3-monooxygenase/tryptophan 5-monooxygenase activation protein, epsilon polypeptide	YWHAE	Ssc.21462.1.S1_at	2.216	6.88E-04	intracellular signaling cascade
Novel protein (Fragment)	DNAH14	Ssc.6036.1.S1_at	2.213	1.80E-03	biological_process unknown
heat shock 70kDa protein 6 (HSP70B')	HSPA6	Ssc.5145.1.S1_at	2.203	4.68E-02	response to stress
FLJ46154 protein	FLJ46154	Ssc.30368.1.A1_at	2.199	1.66E-04	intracellular signaling cascade
ras homolog gene family, member E	ARHE	Ssc.9453.1.A1_at	2.195	6.83E-04	actin cytoskeleton organization and biogenesis;cell adhesion;small GTPase mediated signal transduction
Heat-shock protein beta-1 (HspB1)	HSPB1	Ssc.11197.1.S1_at	2.190	6.73E-03	regulation of translational initiation

fatty acid binding protein 4, adipocyte	FABP4	Ssc.1089.1.S1_at	2.142	1.29E-02	transport
neuritin 1	NRN1	Ssc.1664.2.S1_at	2.134	3.23E-03	biological_process unknown
hook homolog 2 (Drosophila)	HOOK2	Ssc.4342.1.A1_at	2.125	6.30E-03	endocytosis
ankyrin repeat domain 2 (stretch responsive muscle)	ANKRD2	Ssc.18494.1.S1_at	2.123	6.91E-03	muscle development
cysteine and glycine-rich protein 3 (cardiac LIM protein)	CSRP3	Ssc.19310.1.S1_at	2.087	1.22E-02	myogenesis
methionyl aminopeptidase 1	METAP1	Ssc.26446.1.S1_at	2.069	7.19E-04	proteolysis and peptidolysis
methionyl aminopeptidase 1	METAP1	Ssc.26446.2.S1_a_at	2.068	3.49E-03	proteolysis and peptidolysis
secretory leukocyte protease inhibitor (antileukoproteinase)	SLPI	Ssc.6080.1.S1_at	2.061	1.03E-03	copulation
Calcipressin 1	DSCR1	Ssc.9607.1.A1_at	2.060	1.89E-02	system process; signal transduction; cell communication
nebulin	NEB	Ssc.18417.1.A1_at	2.056	1.07E-03	regulation of actin filament length;somatic muscle development
X/potassium-transporting ATPase beta-m chain	ATP1B4	Ssc.15887.1.S1_at	2.050	6.50E-03	potassium ion transport;proton transport;sodium ion transport
T-cell lymphoma invasion and metastasis 2	TIAM2	Ssc.13284.1.A1_at	2.042	2.52E-03	intracellular signaling cascade
S-methyl-5-thioadenosine phosphorylase	MTAP	Ssc.27049.1.A1_at	2.029	4.22E-04	nucleobase\, nucleoside\, nucleotide and nucleic acid metabolism
apelin	APLN	Ssc.3436.1.A1_at	2.005	2.19E-03	immune response;lactation;signal transduction

Low expressed

zinc finger, DHHC domain containing 14	ZDHHC14	Ssc.19641.1.S1_at	-2.010	4.39E-02	regulation of transcription\, DNA-dependent
secreted frizzled-related protein 2	SFRP2	Ssc.3232.1.S1_at	-2.010	1.30E-02	development
Tripartite motif protein 7	TRIM7	Ssc.6571.1.S1_at	-2.011	4.69E-03	Unknown
solute carrier family 4	SLC4A8	Ssc.8245.1.A1_at	-2.016	2.49E-03	anion transport
ankyrin repeat domain 9	ANKRD9	Ssc.2847.1.S1_at	-2.019	9.00E-03	Unknown
family with sequence similarity 14, member A	FAM14A	Ssc.12504.1.A1_at	-2.034	1.28E-02	Unknown
ras-related C3 botulinum toxin substrate 1 (rho family, small GTP binding protein Rac1)	RAC1	Ssc.7247.2.A1_at	-2.057	2.78E-04	cell adhesion;cell motility;cell proliferation;inflammatory response;morphogenesis;small GTPase mediated signal transduction
hypothetical protein FLJ14054	FLJ14054	Ssc.7890.1.S1_at	-2.061	4.34E-03	Unknown
chromosome 5 open reading frame 13	C5orf13	Ssc.20917.1.S1_at	-2.096	9.19E-03	cell communication; signal transduction
glycoprotein (transmembrane) nmb	GPNMB	Ssc.18572.1.S1_at	-2.117	2.11E-02	negative regulation of cell proliferation
potassium channel modulatory factor 1	KCMF1	Ssc.4486.1.S1_at	-2.119	9.21E-04	germ-cell migration
Opioid binding protein/cell adhesion molecule precursor (OBCAM)	OPCML	Ssc.10007.1.A1_at	-2.128	8.82E-04	neuronal cell recognition

ATPase, Na+/K+ transporting, alpha 2 (+) polypeptide	ATP1A2	Ssc.16126.1.S1_at	-2.139	2.80E-03	ATP hydrolysis coupled proton transport;hydrogen ion homeostasis;metabolism;potassium ion transport;sodium ion transport;sperm motility
complement component 1, q subcomponent, beta polypeptide	C1QB	Ssc.11004.1.S1_at	-2.142	2.62E-04	complement activation\, classical pathway
Serine/threonine-protein kinase DCAMKL1	DCAMKL1	Ssc.29226.1.S1_at	-2.143	2.10E-02	central nervous system development;intracellular signaling cascade;protein amino acid phosphorylation
tyrosine 3-monooxygenase/tryptophan 5-monooxygenase activation protein, theta polypeptide	YWHAQ	Ssc.10023.1.A1_at	-2.162	3.52E-04	exocytosis;regulation of cell cycle;small GTPase mediated signal transduction
profilin 1	PFN1	Ssc.835.1.S1_at	-2.198	2.88E-03	actin cytoskeleton organization and biogenesis
complement component 3	C3	Ssc.61.1.S1_at	-2.215	1.18E-02	G-protein coupled receptor protein signaling pathway;complement activation\, alternative pathway;complement activation\, classical pathway;inflammatory response
solute carrier family 16 (monocarboxylic acid transporters), member 3	SLC16A3	Ssc.22067.1.A1_at	-2.219	1.85E-03	monocarboxylic acid transport
major histocompatibility complex, class I, A	HLA-A	Ssc.13780.9.S1_a_at	-2.245	1.36E-03	antigen presentation\, endogenous antigen;antigen processing\, endogenous antigen via MHC class I
Maltase-glucoamylase, intestinal	MGAM	Ssc.18021.1.A1_at	-2.253	1.23E-02	polysaccharide catabolic process
glutathione S-transferase M3 (brain)	GSTM3	Ssc.12273.1.A1_at	-2.302	2.36E-04	establishment of blood/nerve barrier;glutathione conjugation reaction;metabolism
epidermal growth factor (beta-urogastrone)	EGF	Ssc.87.1.S1_at	-2.357	7.41E-03	DNA replication;EGF receptor signaling pathway;activation of MAPK;chromosome organization and biogenesis (sensu Eukarya);positive regulation of cell proliferation

Lysozyme C precursor	LYZ	Ssc.670.1.S1_at	-2.425	1.72E-02	response to stress
insulin-like growth factor 2 (somatomedin A)	IGF2	Ssc.9365.1.S1_at	-2.446	1.77E-03	growth pattern;imprinting;insulin receptor signaling pathway;regulation of cell cycle;skeletal development
troponin T2, cardiac	TNNT2	Ssc.19141.1.S1_at	-2.449	3.67E-02	muscle development;regulation of heart rate;regulation of muscle contraction
PREDICTED: similar to carcinoembryonic antigen-related cell adhesion molecule 1		Ssc.6653.1.S1_at	-2.476	4.48E-03	Unknown
Glutamate carboxypeptidase II	FOLH1	Ssc.14488.1.S1_at	-2.493	7.79E-03	proteolysis and peptidolysis
Serine/threonine-protein kinase DCAMKL1	DCAMKL1	Ssc.24075.1.A1_at	-2.499	1.04E-02	central nervous system development;intracellular signaling cascade;protein amino acid phosphorylation
SRY (sex determining region Y)-box 8	SOX8	Ssc.23983.1.A1_at	-2.520	1.19E-04	central nervous system development;regulation of transcription from Pol II promoter
ectonucleotide pyrophosphatase/phosphodiesterase 4 (putative function)	ENPP4	Ssc.3980.1.A1_at	-2.543	4.19E-05	nucleotide metabolism
interferon, alpha-inducible protein (clone IFI-6-16)	G1P3	Ssc.20101.1.S1_at	-2.552	2.05E-03	immune response
v-erb-b2 erythroblastic leukemia viral oncogene homolog 3 (avian)	ERBB3	Ssc.4203.1.S1_at	-2.565	5.10E-03	protein amino acid phosphorylation;transmembrane receptor protein tyrosine kinase signaling pathway
interferon induced transmembrane protein 3 (1-8U)	IFITM3	Ssc.11098.1.S1_at	-2.566	5.33E-04	immune response

acyl-CoA:lysocardiolipin acyltransferase 1 isoform 1	IF	Ssc.17353.1.S1_at	-2.597	4.74E-05	adaptive immune response
sema domain, immunoglobulin domain (Ig), short basic domain, secreted, (semaphorin) 3A	SEMA3A	Ssc.29388.1.A1_at	-2.656	1.56E-04	neurogenesis
hypothetical protein FLJ14054	FLJ14054	Ssc.15291.1.A1_at	-2.664	1.48E-03	Unknown
interferon, alpha-inducible protein (clone IFI-15K)	G1P2	Ssc.11557.1.A1_at	-2.718	2.01E-03	cell-cell signaling;immune response
ATP/GTP binding protein 1	AGTPBP1	Ssc.4825.1.A1_at	-2.733	1.46E-04	proteolysis and peptidolysis
type II transmembrane protein DCAL1	CLECSF6	Ssc.12825.1.A1_at	-2.762	2.19E-04	antimicrobial humoral response (sensu Vertebrata);cell adhesion;cell surface receptor linked signal transduction
myosin, heavy polypeptide 8, skeletal muscle, perinatal	MYH8	Ssc.27020.1.S1_at	-3.088	9.54E-03	muscle development;striated muscle contraction
chromosome 1 open reading frame 22	C1orf22	Ssc.22734.1.A1_at	-3.187	1.67E-05	N-linked glycosylation;carbohydrate metabolism;proteolysis and peptidolysis
viperin	cig5	Ssc.286.1.S1_s_at	-3.388	4.22E-05	immune effector process
Ig alpha-1 chain C region	IGHM	Ssc.11056.2.S1_a_at	-4.643	1.89E-02	immune response
Vacuolar ATP synthase subunit G 2	ATP6V1G2	Ssc.12005.1.A1_at	-4.907	3.04E-05	cellular iron ion homeostasis
leucine rich repeat containing 37, member A3	FLJ34306	Ssc.24800.2.A1_at	-6.136	2.54E-07	Unknown

secreted phosphoprotein 1 (osteopontin, bone sialoprotein I, early T-lymphocyte activation 1)	SPP1	Ssc.101.1.S1_at	-6.179	3.32E-02	T-helper 1 type immune response;anti-apoptosis;cell-cell signaling;cell-matrix adhesion;immune cell chemotaxis;negative regulation of bone mineralization;positive regulation of T-cell proliferation;regulation of myeloid blood cell differentiation
peroxisomal trans-2-enoyl-CoA reductase	PECR	Ssc.30628.1.S1_at	-7.863	8.28E-08	apoptosis;enterobactin biosynthesis
cytochrome P450, family 3, subfamily A, polypeptide 4	CYP3A4	Ssc.204.1.S1_at	-7.991	4.26E-06	electron transport;lipid metabolism;oncogenesis;xenobiotic metabolism
