# Supplementary material for: Comparison of Gene Expression and Genome-Wide DNA Methylation Profiling between Phenotypically Normal Cloned Pigs and Conventionally Bred Controls
Source: PLoS One. 2011 Oct 11;6(10):e25901. doi: 10.1371/journal.pone.0025901 (PMC3191147; doi:10.1371/journal.pone.0025901)
Supplement: Table S2 — List of significantly ( P <0.05) and differentially (FC≥2) expressed genes in liver of cloned pigs. (PDF) [file pone.0025901.s003.pdf]

Supplementary File 2

Differentially expressed genes between cloned and normal pigs in liver at $p < 0.05$, FC ⩾ 2

Gene	Symbol	Affy Probe ID	FC	p value	GO-process
**High expressed**					
paraoxonase 3	PON3	Ssc.21810.1.S1_at	15.809	1.14E-06	response to external stimulus
Protocadherin 15 precursor	PCDH15	Ssc.30063.1.A1_at	9.777	1.21E-07	system process; cell adhesion
cytochrome P450, family 3, subfamily A, polypeptide 4	CYP3A4	Ssc.929.1.S1_at	8.282	4.00E-06	electron transport;lipid metabolism;oncogenesis;xenobiotic metabolism
collagen, type IV, alpha 2	COL4A2	Ssc.9939.1.A1_at	4.964	3.55E-05	extracellular matrix organization and biogenesis;regulation of transcription\, DNA-dependent
collagen, type I, alpha 1	COL1A1	Ssc.1091.1.S1_at	3.827	3.98E-04	epidermal differentiation;skeletal development
transmembrane protein with EGF-like and two follistatin-like domains 1	TMEFF1	Ssc.24298.1.S1_at	3.721	1.51E-04	multicellular organismal development
asporin (LRR class 1)	ASPN	Ssc.1716.1.S1_at	3.579	3.33E-04	Unknown
adenylate cyclase activating polypeptide 1 (pituitary)	ADCYAP1	Ssc.27598.1.S1_at	3.414	4.28E-03	adenylate cyclase activation;cell-cell signaling;pregnancy
synovial sarcoma, X breakpoint 2 interacting protein	SSX2IP	Ssc.28283.1.A1_at	3.334	8.72E-03	regulation of transcription\, DNA-dependent
ectonucleotide pyrophosphatase/phosphodiesterase 3	ENPP3	Ssc.19431.1.S1_at	3.274	3.75E-02	nucleotide metabolism;phosphate metabolism
fibromodulin	FMOD	Ssc.11858.1.S1_at	3.138	1.73E-04	TGFbeta receptor complex assembly
CDC10 cell division cycle 10 homolog (S. cerevisiae)	CDC10	Ssc.1837.2.A1_at	3.072	8.95E-03	cell cycle;cytokinesis
doublecortin domain containing 2	DCDC2	Ssc.30216.1.A1_at	3.070	2.05E-05	cellular defense response;intracellular signaling cascade
integrin, alpha V (vitronectin receptor, alpha polypeptide, antigen CD51)	ITGAV	Ssc.6737.2.A1_at	3.042	3.56E-05	cell-matrix adhesion;integrin-mediated signaling pathway
Apolipoprotein A-IV precursor (Apo-AIV)	APOA4	Ssc.14503.1.S1_at	3.009	4.36E-02	response to reactive oxygen species; lipid metabolic process

potassium voltage-gated channel, KQT-like subfamily, member 1	KCNQ1	Ssc.6056.1.S1_at	2.956	9.72E-04	hearing;muscle contraction;potassium ion transport;regulation of heart rate
hypothetical gene BC008967	BC008967	Ssc.26539.1.S1_at	2.931	1.47E-02	invasive growth
osteoglycin (osteoinductive factor, mimecan)	OGN	Ssc.26180.1.S1_at	2.912	1.24E-02	biological_process unknown
UDP glycosyltransferase 2 family, polypeptide B17	UGT2B17	Ssc.10441.1.S1_at	2.851	1.48E-02	steroid metabolism;xenobiotic metabolism
lysyl oxidase	LOX	Ssc.8224.1.S1_at	2.822	2.48E-02	protein modification
glutamate receptor, ionotropic, kainate 2	GRIK2	Ssc.19883.1.S1_at	2.808	1.53E-05	glutamate signaling pathway;potassium ion transport;synaptic transmission
collagen, type XII, alpha 1	COL12A1	Ssc.1049.1.S1_at	2.776	5.28E-03	cell adhesion;skeletal development
glycine N-methyltransferase	GNMT	Ssc.16163.1.S1_at	2.744	1.12E-03	protein modification
decorin	DCN	Ssc.10245.2.A1_a_at	2.743	9.89E-03	organogenesis
NEL-like 2 (chicken)	NELL2	Ssc.11561.1.A1_at	2.719	4.54E-03	cell adhesion
secreted frizzled-related protein 1	SFRP1	Ssc.2283.1.S1_at	2.623	6.46E-04	anti-apoptosis;morphogenesis;signal transduction
galactosylceramidase (Krabbe disease)	GALC	Ssc.7531.1.A1_at	2.542	3.88E-05	carbohydrate metabolism;galactosylceramide catabolism
coagulation factor XIII, A1 polypeptide	F13A1	Ssc.26345.1.S1_at	2.488	8.72E-05	blood coagulation;peptide cross-linking
anthrax toxin receptor 1	ANTXR1	Ssc.20172.1.A1_at	2.470	3.59E-04	cell-matrix adhesion
dorsal root ganglia homeobox	NP_001073989	Ssc.16652.1.S1_at	2.447	5.56E-03	Unknown
ribosomal protein L38	RPL38	Ssc.28256.1.A1_at	2.447	4.04E-05	protein biosynthesis
collagen, type I, alpha 2	COL1A2	Ssc.21011.1.S1_at	2.435	1.76E-03	cell adhesion;skeletal development
angiopoietin-like 2	ANGPTL2	Ssc.29929.1.S1_at	2.434	4.03E-03	development
Alpha-2-HS-glycoprotein precursor (Fetuin-A)	AHSG	Ssc.22211.2.S1_at	2.418	4.71E-03	cell morphogenesis; skeletal development;acute inflammatory response
Rhesus blood group, B glycoprotein	RHBG	Ssc.17.1.S1_at	2.415	1.37E-03	transport
protease, serine, 35	PRSS35	Ssc.26815.1.A1_at	2.411	2.69E-02	Unknown
similar to delta 5 fatty acid desaturase	FADS6	Ssc.21691.3.S1_at	2.406	1.08E-04	Unknown
myosin IB	MYO1B	Ssc.13601.1.A1_at	2.400	1.48E-03	Unknown

galactosylceramidase (Krabbe disease)	GALC	Ssc.7531.2.S1_at	2.397	1.55E-04	carbohydrate metabolism;galactosylceramide catabolism
collagen, type VI, alpha 1	COL6A1	Ssc.5895.1.A2_at	2.392	8.65E-03	cell adhesion;regulation of transcription\, DNA-dependent
Baculoviral IAP repeat-containing protein 4	BIRC4	Ssc.8950.1.A1_at	2.390	1.54E-04	apoptosis; anti-apoptosis; cell death
Ig alpha-1 chain C region	IGHM	Ssc.11070.1.S1_at	2.376	4.10E-02	MAPKKK cascade; activation of MAPK activity
C1q and tumor necrosis factor related protein 3	C1QTNF3	Ssc.4231.1.A1_at	2.367	3.61E-02	transport
lysyl oxidase-like 1	LOXL1	Ssc.10137.1.A1_at	2.348	6.85E-03	protein modification;regulation of transcription\, DNA-dependent
AE binding protein 1	AEBP1	Ssc.16584.1.A1_at	2.342	2.74E-03	cell adhesion;muscle development;proteolysis and peptidolysis;regulation of transcription\, DNA-dependent;skeletal development
chromosome 10 open reading frame 45	C10orf45	Ssc.14418.1.A1_at	2.318	3.98E-04	biological_process unknown
odd Oz/Ten-m homolog 3	ODZ3	Ssc.27124.1.A1_at	2.308	3.10E-02	neurogenesis
ryanodine receptor 2 (cardiac)	RYR2	Ssc.25615.1.A1_at	2.293	3.01E-02	calcium ion transport;muscle contraction;regulation of heart rate;signal transduction
exportin 6	XPO6	Ssc.23544.1.S1_at	2.288	1.26E-02	protein-nucleus import\, docking
solute carrier family 27 (fatty acid transporter), member 6	SLC27A6	Ssc.24721.1.A1_at	2.283	5.89E-03	very-long-chain fatty acid metabolism
elaC homolog 2 (E. coli)	ELAC2	Ssc.21167.2.A1_at	2.261	4.96E-03	biological_process unknown
chromosome 14 open reading frame 136	C14orf136	Ssc.26910.1.S1_at	2.253	3.45E-04	Unknown
LAP1 protein (Densin-180)	NP_065845	Ssc.23015.1.S1_at	2.248	9.29E-03	Unknown
ets variant gene 6 (TEL oncogene)	ETV6	Ssc.22398.1.A1_at	2.247	1.49E-03	cell growth and/or maintenance;regulation of transcription\, DNA-dependent
major histocompatibility complex, class II, DR beta 3	HLA-DRB3	Ssc.210.6.S1_x_at	2.240	3.44E-03	antigen presentation\, exogenous antigen;antigen processing\, exogenous antigen via MHC class II;pathogenesis;perception of pest/pathogen/parasite;signal transduction
lumican	LUM	Ssc.1128.1.S1_at	2.222	3.16E-04	cartilage condensation;vision
DAZ interacting protein 1	DZIP1	Ssc.24505.1.A1_at	2.206	1.52E-02	cytoskeleton organization and biogenesis

matrix metalloproteinase 2 (gelatinase A, 72kDa gelatinase, 72kDa type IV collagenase)	MMP2	Ssc.5713.1.S1_at	2.169	4.66E-04	collagen catabolism
cryptochrome 1 (photolyase-like)	CRY1	Ssc.2033.1.S1_at	2.163	6.81E-04	DNA repair;circadian rhythm;vision
Laminin alpha-2 chain precursor	LAMA2	Ssc.29333.1.A1_at	2.116	2.31E-04	cell motility; multicellular organismal development
steroid sensitive gene 1	URB	Ssc.3825.1.S1_at	2.106	1.80E-02	Unknown
tenascin XB	TNXB	Ssc.19638.1.S1_at	2.099	5.44E-03	cell-matrix adhesion
formin binding protein 1	FNBP1	Ssc.8956.2.A1_at	2.096	2.39E-02	regulation of transcription\, DNA-dependent;signal transduction
Follistatin-related protein 1 precursor	FSTL1	Ssc.23242.1.A1_at	2.083	6.32E-03	Unknown
plakophilin 2	PKP2	Ssc.14866.1.S1_at	2.083	8.69E-04	cell-cell adhesion
Alpha-2-HS-glycoprotein precursor (Fetuin-A)	AHSG	Ssc.22211.1.A1_at	2.078	3.63E-02	cell morphogenesis; skeletal development
dapper homolog 1, antagonist of beta-catenin (xenopus)	DACT1	Ssc.19575.1.S1_at	2.076	5.74E-03	cell communication
growth arrest-specific 1	GAS1	Ssc.21626.2.S1_at	2.068	1.18E-02	cell cycle arrest;negative regulation of S phase of mitotic cell cycle;negative regulation of cell proliferation
transgelin 2	TAGLN2	Ssc.17300.1.S1_at	2.067	2.55E-04	muscle development
NADH dehydrogenase (ubiquinone) Fe-S protein 4, 18kDa (NADH-coenzyme Q reductase)	NDUFS4	Ssc.24221.2.A1_at	2.066	3.26E-02	mitochondrial electron transport\, NADH to ubiquinone
NG,NG-dimethylarginine dimethylaminohydrolase 1	DDAH1	Ssc.16839.1.S1_at	2.065	4.73E-03	urea cycle intermediate metabolic process
collagen, type III, alpha 1 (Ehlers-Danlos syndrome type IV, autosomal dominant)	COL3A1	Ssc.11302.1.S2_at	2.053	3.28E-03	circulation;histogenesis and organogenesis;organogenesis
cytochrome P450, family 2, subfamily J, polypeptide 2	CYP2J2	Ssc.5327.2.A1_at	2.023	1.51E-02	electron transport;icosanoid metabolism;regulation of heart rate
Laminin beta-1 chain precursor (Laminin B1 chain)	LAMB1	Ssc.15823.1.S1_at	2.010	5.01E-03	cell morphogenesis; cell motility

tumor necrosis factor receptor superfamily, member 12A	TNFRSF12A	Ssc.1864.1.A1_a_at	2.009	1.49E-02	angiogenesis;apoptosis;cell motility

Low expressed

sema domain, immunoglobulin domain (Ig), short basic domain, secreted, (semaphorin) 3A	SEMA3A	Ssc.29388.1.A1_at	-2.014	1.57E-03	neurogenesis
poly(A) binding protein, cytoplasmic, pseudogene 3	PABPCP3	Ssc.23408.1.A1_s_at	-2.015	8.11E-04	Unknown
cell adhesion molecule with homology to L1CAM (close homolog of L1)	CHL1	Ssc.8308.1.A1_at	-2.016	3.22E-02	cell adhesion;signal transduction
dachshund homolog 2 (Drosophila)	DACH2	Ssc.7799.1.A1_at	-2.033	1.12E-02	cell growth and/or maintenance
calmodulin-like 4	CALML4	Ssc.4434.1.S1_at	-2.037	2.68E-04	Unknown
ATP-binding cassette, sub-family A (ABC1), member 6	ABCA6	Ssc.31166.1.S1_at	-2.038	1.42E-03	transport
interferon-induced protein with tetratricopeptide repeats 1	IFIT1	Ssc.30752.2.A1_at	-2.039	2.43E-03	biological_process unknown;immune response
Alpha-2-macroglobulin precursor	A2M	Ssc.9440.1.A1_at	-2.048	2.08E-03	protein complex assembly
Tara-like protein	HRIHFB2122	Ssc.11763.1.A1_at	-2.051	3.04E-03	actin modification
ets variant gene 1	ETV1	Ssc.10686.1.S1_at	-2.070	9.30E-04	cell growth and/or maintenance;regulation of transcription\, DNA-dependent;transcription from Pol II promoter
ets variant gene 1	ETV1	Ssc.7186.1.A1_at	-2.080	2.17E-04	cell growth and/or maintenance;regulation of transcription\, DNA-dependent;transcription from Pol II promoter
5-hydroxytryptamine 2A receptor (5-HT-2A)	HTR2A	Ssc.13380.1.A1_at	-2.092	1.84E-03	regulation of neurotransmitter levels
chromosome 1 open reading frame 22	C1orf22	Ssc.22734.1.A1_at	-2.118	4.43E-04	N-linked glycosylation;carbohydrate metabolism;proteolysis and peptidolysis
Olfactory receptor 5D14	O5DE	Ssc.13469.1.A1_at	-2.129	2.35E-03	Unknown

p53-associated parkin-like cytoplasmic protein	PARC	Ssc.19484.1.A1_at	-2.132	2.25E-04	cell cycle
forkhead box A2	FOXA2	Ssc.17964.2.A1_at	-2.136	3.26E-03	regulation of transcription\, DNA-dependent
TGFB inducible early growth response	TIEG	Ssc.17289.1.A1_at	-2.139	2.82E-03	TGFbeta receptor signaling pathway;cell-cell signaling;negative regulation of cell proliferation;negative regulation of transcription from Pol II promoter;skeletal development
interferon, alpha-inducible protein (clone IFI-15K)	G1P2	Ssc.11557.1.A1_at	-2.154	9.28E-03	cell-cell signaling;immune response
profilin 1	PFN1	Ssc.835.1.S1_at	-2.182	3.04E-03	actin cytoskeleton organization and biogenesis
interferon induced transmembrane protein 3 (1-8U)	IFITM3	Ssc.11098.1.S1_at	-2.194	1.78E-03	immune response
coactivator-associated arginine methyltransferase 1	CARM1	Ssc.4282.1.S1_at	-2.211	4.18E-03	nucleobase, nucleoside, nucleotide and nucleic acid metabolic process
glutathione S-transferase A1	GSTA1	Ssc.2825.1.S1_at	-2.216	2.55E-04	biological_process unknown;metabolism;response to stress
villin 1	VIL1	Ssc.2884.1.S1_at	-2.246	4.87E-03	cytoskeleton organization and biogenesis;protein complex assembly
PREDICTED: dynein, cytoplasmic, heavy polypeptide 2	Q6ZUM6	Ssc.19304.1.A1_at	-2.253	1.42E-02	Unknown
formin binding protein 1	FNBP1	Ssc.7958.1.A1_at	-2.272	2.78E-04	regulation of transcription\, DNA-dependent;signal transduction
Traf2 and NCK interacting kinase	KIAA0551	Ssc.19465.1.A1_at	-2.282	2.38E-04	protein amino acid phosphorylation;protein kinase cascade;regulation of translation;response to stress
KIAA1205 protein	KIAA1205	Ssc.7685.1.A1_at	-2.283	2.57E-04	Unknown
regucalcin gene promotor region related protein	NP_149118	Ssc.2554.1.S1_at	-2.287	3.68E-04	Unknown
acyl-Coenzyme A dehydrogenase, very long chain	ACADVL	Ssc.19654.1.S1_at	-2.313	3.48E-05	electron transport;energy derivation by oxidation of organic compounds;fatty acid beta-oxidation
alpha-methylacyl-CoA racemase	AMACR	Ssc.18280.1.A1_at	-2.335	5.08E-03	metabolism
crumbs homolog 1 (Drosophila)	CRB1	Ssc.19471.1.A1_at	-2.346	4.67E-02	cell-cell signaling;establishment and/or maintenance of cell polarity;vision

leucine rich repeat containing 37, member A3	FLJ34306	Ssc.24800.2.A1_at	-2.347	1.17E-04	Unknown
nebulin	NEB	Ssc.20198.1.S1_at	-2.351	3.96E-03	regulation of actin filament length;somatic muscle development
dedicator of cytokinesis 1	DOCK1	Ssc.7408.1.A1_at	-2.390	1.33E-04	apoptosis;integrin-mediated signaling pathway;phagocytosis\, engulfment;small GTPase mediated signal transduction
alanine-glyoxylate aminotransferase 2-like 1	AGXT2L1	Ssc.8295.1.A1_at	-2.441	2.00E-02	organic acid metabolic process
MSTP111	Q7Z2T0	Ssc.4729.1.S2_at	-2.456	2.45E-02	Unknown
histamine receptor H1	HRH1	Ssc.9262.1.A1_at	-2.559	1.63E-04	G-protein signaling\, coupled to IP3 second messenger (phospholipase C activating);inflammatory response
kinesin family member 14	KIF14	Ssc.22601.1.S1_at	-2.576	9.25E-03	transport; organelle organization and biogenesis
cathelicidin antimicrobial peptide	CAMP	Ssc.420.1.S1_a_at	-2.596	2.10E-02	defense response;xenobiotic metabolism
ectonucleotide pyrophosphatase/phosphodiesterase 4 (putative function)	ENPP4	Ssc.3980.1.A1_at	-2.621	3.27E-05	nucleotide metabolism
coagulation factor III (thromboplastin, tissue factor)	F3	Ssc.19907.1.S1_at	-2.738	1.85E-02	blood coagulation;immune response
tyrosine 3-monooxygenase/tryptophan 5-monooxygenase activation protein, theta polypeptide	YWHAQ	Ssc.10023.1.A1_at	-2.746	4.72E-05	exocytosis;regulation of cell cycle;small GTPase mediated signal transduction
afamin	AFM	Ssc.22142.1.A1_at	-2.834	4.45E-03	transport
usherin isoform A	USH2A	Ssc.7180.1.A1_at	-2.864	2.18E-03	system process; multicellular organismal development
amyloid P component, serum	APCS	Ssc.14517.1.S1_at	-2.879	4.58E-03	DNA packaging;heterophilic cell adhesion;pathogenesis;protein complex assembly\, multichaperone pathway;protein folding
oxoglutarate dehydrogenase-like	OGDHL	Ssc.10785.1.A1_at	-3.480	4.49E-02	Unknown

glutathione S-transferase M3 (brain)	GSTM3	Ssc.12273.1.A1_at	-3.511	1.00E-05	establishment of blood/nerve barrier;glutathione conjugation reaction;metabolism
putative L-type neutral amino acid transporter	KIAA0436	Ssc.2441.1.S1_at	-3.922	1.33E-03	proteolysis and peptidolysis
prolactin receptor	PRLR	Ssc.24638.1.S1_at	-4.089	9.55E-06	T-cell activation;anti-apoptosis;embryo implantation;lactation;steroid biosynthesis;transmembrane receptor protein tyrosine kinase activation (dimerization);tyrosine phosphorylation of JAK2 protein
estrogen-related receptor gamma	ESRRG	Ssc.28547.1.S1_at	-4.130	3.06E-02	regulation of transcription\, DNA-dependent
C-reactive protein, pentraxin-related	CRP	Ssc.16157.1.S1_at	-4.152	4.64E-03	acute-phase response;inflammatory response
v-myc myelocytomatosis viral oncogene homolog (avian)	MYC	SscAffx.8.1.S1_s_at	-4.207	2.91E-05	cell cycle arrest;iron ion homeostasis;pathogenesis;regulation of transcription from Pol II promoter
claudin 2	CLDN2	Ssc.19842.1.S1_at	-4.254	3.71E-05	protein complex assembly
prominin 1	PROM1	Ssc.4065.1.A1_at	-4.715	3.01E-06	vision
Vacuolar ATP synthase subunit G 2	ATP6V1G2	Ssc.12005.1.A1_at	-4.959	2.89E-05	cellular iron ion homeostasis
Unknown	Q8N5E3	Ssc.1256.1.A1_at	-12.046	2.41E-08	Unknown
