# Supplementary material for: Comparison of Gene Expression and Genome-Wide DNA Methylation Profiling between Phenotypically Normal Cloned Pigs and Conventionally Bred Controls
Source: PLoS One. 2011 Oct 11;6(10):e25901. doi: 10.1371/journal.pone.0025901 (PMC3191147; doi:10.1371/journal.pone.0025901)
Supplement: Table S3 — IPA molecular network analysis of significantly and differentially expressed genes in muscle or liver of cloned pigs. Network score≥2 was considered as over-represented. (PDF) [file pone.0025901.s004.pdf]

# Supplemental File 3

| Tissue | Molecules in Network [a] | Score | Focus Molecules | Top Functions |
|---|---|---|---|---|
| **muscle** | **ADIPOQ**, AKT1, **APLN**, ARHGDIA, BAX, **C3**, CASP3, CCL27, COL1A2, CXCL12, DIABLO, **EGF**, **ERBB3**, ESR1, F2, FOS, **HSPB2**, **HTATIP2**, **ITGAV**, MAPK3, MAPK7, MYC, NFKB1, NRG1, **RAC1**, RELA, **RND3**, RPS6KB1, **SEMA3A**, SHC1, **SPP1**, TGFBR2, TNF, **XIAP**, **YWHAE** | 19 | 14 | Cell Death, Cancer, Cellular Growth and Proliferation |
| **muscle** | **SEPT7**, SEPT9 | 2 | 1 | Cell Morphology, Cellular Assembly and Organization, Cellular Compromise |
| **muscle** | **TNFRSF12A**, TNFSF12 | 2 | 1 | Cellular Development, Cellular Growth and Proliferation, Hepatic System Development and Function |
| **muscle** | **ENAH**, TES, ZYX | 1 | 1 | Nervous System Development and Function, Organ Development, Cell Morphology |
| **muscle** | **PFN1**, SMN1, XPO6 | 1 | 1 | Organismal Survival, Cellular Compromise, Embryonic Development |
| **muscle** | **ENPP4**, MECP2 | 2 | 1 | Behavior, Genetic Disorder, Nervous System Development and Function |
| **muscle** | **DAPK3**, MYL2 | 2 | 1 | Cellular Assembly and Organization, Skeletal and Muscular System Development and Function, Cardiac Hypertrophy |
| **muscle** | KDM1, **SFRP2** | 2 | 1 | Skeletal and Muscular Disorders, Cancer, Cell Death |
| **muscle** | **CYP3A4**, NR1I2, NR1I3 | 1 | 1 | Gene Expression, Molecular Transport, Small Molecule Biochemistry |
| **liver** | **APCS**, CDKN1A, CLU, **COL1A1**, **COL1A2**, **CRP**, **CUL9**, CUL7 (includes EG:9820), CYR61, **DCN**, DDB2, **ESRRG**, FASLG, FGF1, HNF1A, HUWE1, ID2, IFNG, **ITGAV**, ITGB3, **MMP2**, **MYC**, **PRLR**, **PROM1**, RFX5, **SEMA3A**, TAF1B, **TAGLN2**, TGFB1, TMSB4X, **TNFRSF12A**, TOP2A, TP53, TP73, VTN | 21 | 15 | Cancer, Cellular Movement, Cellular Growth and Proliferation |
| **liver** | **PFN1**, **XPO6** | 4 | 2 | Cardiovascular Disease, Cellular Assembly and Organization, Cellular Function and Maintenance |
| **liver** | **ENPP4**, MECP2 | 2 | 1 | Behavior, Genetic Disorder, Nervous System Development and Function |
| **liver** | CIT, **KIF14** | 2 | 1 | Cell Cycle, Cellular Movement, Cancer |
| **liver** | ELN, **LOXL1** | 2 | 1 | Organ Morphology, Reproductive System Disease, Respiratory System Development and Function |
| **liver** | **MYO1B**, ZNF217 | 2 | 1 | Cardiovascular Disease, Cellular Development, Cellular Assembly and Organization |
| **liver** | **GSTA1**, IL1B | 2 | 1 | Cancer, Carbohydrate Metabolism, Cardiovascular Disease |
| **liver** | **SEPT7**, SEPT9 | 2 | 1 | Cell Morphology, Cellular Assembly and Organization, Cellular Compromise |
| **liver** | **COL6A1**, COL6A3 | 2 | 1 | Genetic Disorder, Skeletal and Muscular Disorders, Connective Tissue Disorders |
| **liver** | **CYP3A4**, NR1I2, NR1I3 | 1 | 1 | Gene Expression, Molecular Transport, Small Molecule Biochemistry |

[a] molecules in bold red or bold green were high or low expressed in cloned pigs
