# Supplementary material for: Comparison of Gene Expression and Genome-Wide DNA Methylation Profiling between Phenotypically Normal Cloned Pigs and Conventionally Bred Controls
Source: PLoS One. 2011 Oct 11;6(10):e25901. doi: 10.1371/journal.pone.0025901 (PMC3191147; doi:10.1371/journal.pone.0025901)
Supplement: Table S6 — Summary table of molecular network analysis by Ingenuity Pathways Analysis of genes neighboring significantly-differently methylated tags in muscle and liver. (PDF) [file pone.0025901.s007.pdf]

Analysis	Molecules in Network	Score	Focus Molecules	Top Functions
Muscle	AHNAK, AKT1, AKT2, **ASAP1, ATL2**, C14ORF166, CAMK2B, **CHFR, CSF1R**, DARS, DDX1, **DICER1**, DLAT, **EBF3, F13A1**, FAM62A, GAB2, GLG1, GRIN1, **HSF4**, HSP90AA1, HSPB1, IPO9, ITSN2, LIMA1, MAN2B1, MAPK1, PARVA, PIK3R1, PRKACB, **PRKAR1B, RUVBL2**, SLC2A4, TKT, YWHAZ	14	10	Carbohydrate Metabolism, Cell Morphology, Skeletal and Muscular System Development and Function
Muscle	**ABLIM1**, ACTB, **ADARB1**, ANXA2, C22ORF9, **CAB39, CALD1, DNAJA2, DYSF**, FAM82A2, FRMD6, HECTD1, LARP1, MFAP1, MICALL1, MOBKL3, MYC, MYCBP2, **NSMAF**, OSBPL3, PABPC1, **PAN3, PDCL, RADIL**, RAI14, RALGPS2, SSH1, SSH2, STK38L (includes EG:23012), TAOK1, TCP1, **TESK1**, YWHAB, YWHAG, ZAK	16	11	Cellular Assembly and Organization, Post-Translational Modification, Protein Folding
Muscle	ASL, **CCND1**, CEBPA, DBH, E2F4, EDF1, ESRRG, **EXOC2**, FDFT1, FOS, **GTF3A, H2AFX**, HEXIM1, HNRNPK, **KHDRBS3**, LDHB, LITAF, MKL1, **MKL2, NAP1L4**, NCOA3, NPDC1, NR1H3, **PPARGC1B**, PPP6C, **RASA3**, RBM7, RPS5, **SCHIP1**, SP2, TBK1, TIA1, TNF, TRIB2, TSC22D3	14	10	Gene Expression, Cancer, Cellular Growth and Proliferation
Muscle	ATXN1, **FAT4**	2	1	Genetic Disorder, Neurological Disease, Skeletal and Muscular Disorders
Muscle	**PCDH12**, PRKCE	2	1	Cancer, Cell Morphology, Dermatological Diseases and Conditions
Muscle	FAM118B, **RASGRP2**	2	1	Cancer, Cell Death, Cellular Development
Muscle	**FAM125B**, TSG101, VPS28	2	1	Cancer, Cell Cycle, Cellular Assembly and Organization
Liver	ACTB, ALB, ARHGAP21, **ATL2**, C12ORF51, DENND4A, FAM82A2, **FOXK1**, FRYL, **GADD45G**, HECTD1, **JARID2**, LARP1, **LIN54**, LSR, MICALL1, MPRIP, NCL, NPM1 (includes EG:4869), PHLDB2, **PPARA, PRKCB, PRKDC, RAE1**, RAI14, **RAVER1**, SPTBN2, SSH2, STK38L (includes EG:23012), SYNPO2, TLK2, **WASF1**, YWHAB, YWHAG, YWHAZ	16	11	Protein Degradation, Digestive System Development and Function, Hepatic System Development and Function
Liver	BCL6, C14ORF156, CFL2, CNBP, CYP2J2, **DBH, DHX40, DNAJA2, EBNA1BP2**, ERLIN1, GSTO1, HNF4A, **HSF4**, JUN, KDM5C, **MAFB**, MIRLET7B (includes EG:406884), NAT13, NDUFA7, NUDT5, PECI, PREP, PRKAB1, **PRKAG2**, REXO2, SEC31A, SLC39A9, TARSL2, THRA, **TNFRSF19**, TRAF6, UBP1, UFM1, WBSCR22, ZNF644	16	11	Gene Expression, Cell-mediated Immune Response, Cellular Growth and Proliferation
Liver	APEX1, **CALD1**, CCND3, CCT2, CCT3, CCT4, CCT5, CCT7, CCT8, CCT6A, DDX3X, **DTNBP1 (includes EG:84062)**, **EXOC2**, EXOC3, EXOC4, EXOC6, EXOC7, GAPDH (includes EG:2597), GCN1L1, ID2, KPNA2, MED12, **MXI1**, MYC, NONO, **PDCL, PFDN5**, PPP2CB, PPP6C, RALA, RPL11, RPS18, TBK1, TCP1, TRIM28	7	6	Cancer, Cell Death, Neurological Disease
Liver	**AK3**, GOT1	2	1	Amino Acid Metabolism, Small Molecule Biochemistry, Cancer
Liver	**PCDH12**, PRKCE	2	1	Cancer, Cell Morphology, Dermatological Diseases and Conditions
Liver	**INPP5A**, RAC1	2	1	Cellular Assembly and Organization, Cellular Function and Maintenance, Cell Morphology
Liver	**DYSF**, SMARCA4	2	1	Skeletal and Muscular System Development and Function, Genetic Disorder, Skeletal and Muscular Disorders
Liver	CNOT6, PABPC1, **PAN3**	2	1	RNA Post-Transcriptional Modification, Gene Expression, RNA Damage and Repair
Liver	**DMXL2**, MADD, RAB3GAP1, WDR7	2	1	Endocrine System Disorders, Genetic Disorder, Metabolic Disease
Liver	GDF5, MIR152 (includes EG:406943), **NOG**, SOX2	2	1	Developmental Disorder, Cellular Development, Nervous System Development and Function

Annotation Input genes were marked with blue color.Network Score=-log (right-tailed Fisher's Exact Test P value)
