# Supplementary material for: Comparison of Gene Expression and Genome-Wide DNA Methylation Profiling between Phenotypically Normal Cloned Pigs and Conventionally Bred Controls
Source: PLoS One. 2011 Oct 11;6(10):e25901. doi: 10.1371/journal.pone.0025901 (PMC3191147; doi:10.1371/journal.pone.0025901)
Supplement: Table S5 — Summary table of SA analysis of DNA methylation in unique sequences, including DNA fragment ID, counts of mapped tags by high-confidence MQ20 standards, CGIs location, annotation information and SA P values. (PDF) [file pone.0025901.s006.pdf]

Summery for SA analysis of DNA methylation

Chr	Start	Fragment_ID	Control1-Muscle(MQ20)	Control2-Muscle(MQ20)	Clone1-Muscle(MQ20)	Clone2-Muscle(MQ20)	M_controlmean	M_clonemean	M_pvalue	M_FDR_pvalue	Control1-Liver(MQ20)	Control2-Liver(MQ20)	Clone1-Liver(MQ20)	Clone2-Liver(MQ20)	L_controlmean	L_clonemean	L_pvalue	L_FDR_pvalue	mean	sd	mRNA_Start	mRNA_End	mRNA_ID	Gene_symbol	CpGProD_Start	CpGProD_End	CpGProD_score	CpGProD_ID
1	25238	1_25238	125	33	109	76	79	93	0.999719	1	11	14	17	0	13	9	0.79589064	1	48.125	48.4751409					23565	26187	0.34566	ID=promoter_00008;
1	38856	1_38856	12	21	13	12	17	13	0.50439	1	0	16	45	2	8	24	0.00970297	0.3824351	15.125	13.902081								
1	43125	1_43125	4	7	2	1	6	2	0.258649	1	13	15	6	21	14	14	0.98010536	1	8.625	7.02927552								
1	59782	1_59782	10	14	6	15	12	11	0.839207	1	0	13	9	2	7	6	0.99011599	1	8.625	5.55331304								
1	64242	1_64242	56	25	32	33	41	33	0.269309	1	322	119	258	66	221	162	0.07510353	1	113.88	113.954173					63994	64964	0.20813	ID=promoter_00018;
1	73425	1_73425	6	4	21	3	5	12	0.389128	1	21	0	13	2	11	8	0.87190251	1	8.75	8.48107136					72923	74289	0.255	ID=promoter_00021;
1	93368	1_93368	116	61	2	17	89	10	2.18E-17	#######	60	71	1	0	66	1	2.17E-13	9.81E-11	41	42.53402					92816	93914	0.38256	ID=promoter_00029;
1	167349	1_167349	1	9	12	20	5	16	0.124289	1	1	10	1	0	6	1	0.20114407	1	6.75	7.20614817								
1	212582	1_212582	87	15	83	83	51	83	0.181015	1	49	18	27	1	34	14	0.0317829	0.9755061	45.375	34.9609476					212065	212797	0.1489	ID=promoter_00057;
1	254048	1_254048	196	77	67	47	137	57	5.48E-11	#######	10	0	14	0	5	7	0.77775394	1	51.375	65.7091807					253044	256724	0.62203	ID=promoter_00068;
1	261583	1_261583	102	77	4	3	90	4	4.83E-21	#######	71	80	6	7	76	7	4.33E-12	1.67E-09	43.75	42.3783975					261032	262130	0.36019	ID=promoter_00070;
1	267942	1_267942	33	3	22	13	18	18	0.89158	1	20	26	23	4	23	14	0.45771946	1	18	10.5559733					267516	271838	0.649	ID=promoter_00073;
1	296555	1_296555	225	79	262	122	152	192	0.792191	1	11	20	29	16	16	23	0.39752759	1	95.5	99.3191105					294516	298017	0.5303	ID=promoter_00079;
1	297920	1_297920	2	26	2	20	14	11	0.605836	1	0	32	18	1	16	10	0.61601721	1	12.625	12.8612097					294516	298017	0.5303	ID=promoter_00079;
1	308577	1_308577	14	20	29	45	17	37	0.100517	1	47	58	24	22	53	23	0.00785432	0.3240285	32.375	15.6290709								
1	319744	1_319744	46	15	32	30	31	31	0.820663	1	9	14	10	7	12	9	0.89054267	1	20.375	13.989154					318134	319962	0.32068	ID=promoter_00087;
1	321645	1_321645	0	7	8	7	4	8	0.677894	1	0	17	5	3	9	4	0.4571786	1	5.875	5.46253473								
1	322196	1_322196	162	57	108	128	110	118	0.796721	1	8	34	9	3	21	6	0.02669383	0.8538132	63.625	61.4885297					321730	322555	0.15534	ID=promoter_00088;
1	391285	1_391285	25	5	20	15	15	18	0.997876	1	17	10	2	12	14	7	0.39930956	1	13.25	7.62982868					390670	391932	0.67383	ID=promoter_00107;
1	440470	1_440470	2128	725	1169	986	1427	1078	5.83E-27	#######	1085	400	395	251	743	323	3.97E-30	4.20E-27	892.38	606.906897					439339	440681	0.69534	ID=promoter_00118;
1	515150	1_515150	25	6	14	5	16	10	0.283594	1	1	3	1	1	2	1	0.87709017	1	7	8.46842876					513120	515548	0.38597	ID=promoter_00143;
1	520865	1_520865	0	23	33	1	12	17	0.88186	1	33	0	0	8	17	4	0.02867352	0.9010784	12.25	14.9833241					520620	523540	0.43954	ID=promoter_00146;
1	564773	1_564773	72	71	227	177	72	202	4.62E-10	#######	64	54	93	51	59	72	0.29145896	1	101.13	64.9558916					564722	566492	0.26536	ID=promoter_00157;
1	608605	1_608605	10	51	11	7	31	9	3.43E-04	0.02597	47	45	42	19	46	31	0.38685872	1	29	18.9057814								
1	628819	1_628819	126	88	101	95	107	98	0.207374	1	56	56	41	0	56	21	0.00123801	0.0745848	70.375	39.9604715					628167	629429	0.67383	ID=promoter_00176;
1	639728	1_639728	1	1	10	1	1	6	0.241978	1	0	8	31	12	4	22	9.67E-04	0.0606639	8	10.4197614	630393	643115	ID=ENSP000003418 05-D2;PHF10;;	PHF10	638712	640410	0.38518	ID=promoter_00179;
1	697737	1_697737	7	48	13	15	28	14	0.027228	0.89953	36	47	45	22	42	34	0.84122738	1	29.125	16.796577								
1	722541	1_722541	26	4	3	12	15	8	0.181584	1	0	0	19	1	0	10	0.00454609	0.2107253	8.125	9.8479512					721941	723976	0.46481	ID=promoter_00202;
1	806980	1_806980	5	53	10	20	29	15	0.030102	0.97706	17	17	23	30	17	27	0.21252244	1	21.875	14.7011904					806610	807825	0.53397	ID=promoter_00221;

1	814986	1_814986	182	191	225	165	187	195	0.516216	1	146	193	179	40	170	110	0.01272811	0.477247	165.13	55.4602496					812213	816654	0.8152	ID=promoter_00223;
1	821612	1_821612	1	19	1	10	10	6	0.41703	1	6	17	6	0	12	3	0.09316162	1	7.5	7.30948503					820817	822071	0.40033	ID=promoter_00224;
1	858088	1_858088	8	13	3	6	11	5	0.189421	1	13	7	11	14	10	13	0.72167512	1	9.375	3.96187185								
1	879688	1_879688	23	7	17	11	15	14	0.827043	1	36	9	2	2	23	2	3.18E-04	0.0243067	13.375	11.5997229					878998	881819	0.54022	ID=promoter_00245;
1	903433	1_903433	12	30	3	13	21	8	0.017144	0.62492	1	2	15	8	2	12	0.01895406	0.6519669	10.5	9.51690226					901963	909543	0.94729	ID=promoter_00251;
1	907550	1_907550	9	19	5	27	14	16	0.99748	1	1	0	23	15	1	19	1.49E-04	0.0128238	12.375	10.1831443					901963	909543	0.94729	ID=promoter_00251;
1	911580	1_911580	7	26	6	5	17	6	0.027279	0.89953	8	1	10	17	5	14	0.08295177	1	10	7.92824967					911080	913462	0.3881	ID=promoter_00253;
1	911596	1_911596	65	6	33	66	36	50	0.741166	1	14	21	8	27	18	18	0.97449422	1	30	23.7125645					911080	913462	0.3881	ID=promoter_00253;
1	927678	1_927678	33	69	76	84	51	80	0.26579	1	24	48	60	13	36	37	0.90740746	1	50.875	25.7040158					926013	929630	0.60264	ID=promoter_00259;
1	940149	1_940149	16	10	13	15	13	14	0.975695	1	24	11	4	1	18	3	0.00813808	0.3342859	11.75	7.16638981	931433	949664	ID=ENSP000003759 48;THBS2 ;;	THBS2	934245	941404	0.93799	ID=promoter_00261;
1	942545	1_942545	9	27	47	30	18	39	0.091386	1	2	19	18	14	11	16	0.51039395	1	20.75	13.9360786	931433	949664	ID=ENSP000003759 48;THBS2 ;;	THBS2	942369	949071	0.9368	ID=promoter_00262;
1	956730	1_956730	2	13	10	16	8	13	0.766846	1	65	25	35	14	45	25	0.11611057	1	22.5	19.8206242					956057	957169	0.21663	ID=promoter_00265;
1	968444	1_968444	12	59	22	5	36	14	0.001122	0.06988	8	24	20	2	16	11	0.74569574	1	19	18.0870909					967944	970135	0.33789	ID=promoter_00268;
1	969766	1_969766	40	51	41	42	46	42	0.500364	1	50	42	39	31	46	35	0.67768685	1	42	6.32455532					967944	970135	0.33789	ID=promoter_00268;
1	988001	1_988001	16	34	16	16	25	16	0.157912	1	7	18	39	18	13	29	0.02500319	0.8111854	20.5	10.5559733					985595	988692	0.47173	ID=promoter_00272;
1	1E+06	1_1005623	156	128	245	164	142	205	0.162796	1	107	82	72	61	95	67	0.22811303	1	126.88	60.875605					1003227	1010103	0.94098	ID=promoter_00277;
1	1E+06	1_1040270	2	12	0	10	7	5	0.693374	1	0	23	0	2	12	1	0.01422424	0.5202299	6.125	8.28833087					1039621	1041167	0.30171	ID=promoter_00284;
1	1E+06	1_1050302	4	15	12	6	10	9	0.84607	1	2	8	7	6	5	7	0.77775394	1	7.5	4.20883425					1049176	1050524	0.42026	ID=promoter_00286;
1	1E+06	1_108882	1	0	1	0	1	1	0.993645	1	19	9	19	1	14	10	0.81923661	1	6.25	8.39642781								
1	1E+06	1_109248	65	71	25	58	68	42	0.003983	0.19559	44	111	39	50	78	45	0.0379945	1	57.875	26.0133894								
1	1E+06	1_1102966	0	12	13	7	6	10	0.79153	1	13	1	30	3	7	17	0.08351885	1	9.875	9.7165176					1100849	1104342	0.59188	ID=promoter_00302;
1	1E+06	1_1103124	1	13	7	26	7	17	0.253572	1	0	13	11	7	7	9	0.80856893	1	9.75	8.22452778					1100849	1104342	0.59188	ID=promoter_00302;
1	1E+06	1_1125656	10	4	24	24	7	24	0.032448	1	32	38	49	33	35	41	0.59499216	1	26.75	14.6458185					1125380	1126321	0.23986	ID=promoter_00311;
1	1E+06	1_1130285	36	42	22	21	39	22	0.019446	0.69119	26	35	38	21	31	30	0.98602473	1	30.125	8.54295867					1128514	1130693	0.44575	ID=promoter_00312;
1	1E+06	1_114187	456	96	168	144	276	156	8.77E-13	#######	12	42	10	2	27	6	0.00268813	0.1381379	116.25	151.191222								
1	1E+06	1_1148252	12	13	14	19	13	17	0.957342	1	22	12	5	24	17	15	0.99031847	1	15.125	6.1976378					1146685	1149550	0.42281	ID=promoter_00318;
1	1E+06	1_116347	15	18	9	17	17	13	0.50439	1	50	2	24	4	26	14	0.2525037	1	17.375	15.1179883								
1	1E+06	1_116912	8	9	6	5	9	6	0.554309	1	1	0	12	2	1	7	0.08320466	1	5.375	4.20671232								
1	1E+06	1_1169938	157	32	52	63	95	58	3.51E-04	0.02654	32	16	18	5	24	12	0.20726737	1	46.875	48.3984578					1169823	1170357	0.12883	ID=promoter_00323;

1	1E+06	1_117298	14	27	7	17	21	12	0.1273	1	26	8	22	0	17	11	0.64636287	1	15.125	9.68706796								
1	1E+06	1_1326229	38	39	56	44	39	50	0.916945	1	11	8	18	11	10	15	0.49251655	1	28.125	18.2790864					1322791	1326939	0.69298	ID=promoter_00369;
1	1E+06	1_1349896	14	2	14	13	8	14	0.661714	1	3	0	4	0	2	2	0.99713337	1	6.25	6.29625739	1E+06	1432208	ID=ENSP00000375952;SMOC2;;	SMOC2	1346179	1351307	0.75867	ID=promoter_00375;
1	1E+06	1_1370846	2	11	2	5	7	4	0.503048	1	0	10	3	10	5	7	0.77775394	1	5.375	4.3404246	1E+06	1432208	ID=ENSP00000375952;SMOC2;;	SMOC2	1369081	1371277	0.42262	ID=promoter_00380;
1	1E+06	1_1403499	9	7	23	4	8	14	0.661714	1	5	0	0	0	3	0	0.24893445	1	6	7.6718409	1E+06	1432208	ID=ENSP00000375952;SMOC2;;	SMOC2				
1	1E+06	1_145527	4	10	17	7	7	12	0.724589	1	27	0	0	2	14	1	0.00574567	0.2548307	8.375	9.45572389								
1	1E+06	1_146464	13	31	13	29	22	21	0.795425	1	0	26	2	3	13	3	0.06317581	1	14.625	12.6371052								
1	1E+06	1_1498662	7	4	12	16	6	14	0.355886	1	4	7	2	1	6	2	0.42506085	1	6.625	5.12521777	1E+06	1572484	ID=ENSP00000375952-D2;SMOC2;;	SMOC2	1494945	1500072	0.75895	ID=promoter_00429;
1	2E+06	1_1501848	10	9	6	4	10	5	0.276106	1	10	4	7	7	7	7	0.99000271	1	7.125	2.4164614	1E+06	1572484	ID=ENSP00000375952-D2;SMOC2;;	SMOC2	1501245	1508389	0.92512	ID=promoter_00430;
1	2E+06	1_1537163	32	11	12	10	22	11	0.058934	1	14	15	7	2	15	5	0.11779525	1	12.875	8.75764972	1E+06	1572484	ID=ENSP00000375952-D2;SMOC2;;	SMOC2	1534734	1537865	0.5101	ID=promoter_00441;
1	2E+06	1_1542376	0	5	5	11	3	8	0.459247	1	4	3	5	14	4	10	0.21760186	1	5.875	4.48608961	1E+06	1572484	ID=ENSP00000375952-D2;SMOC2;;	SMOC2	1541924	1543801	0.42816	ID=promoter_00443;
1	2E+06	1_1543900	1	7	8	9	4	9	0.54411	1	23	3	9	20	13	15	0.84581615	1	10	7.69043933	1E+06	1572484	ID=ENSP00000375952-D2;SMOC2;;	SMOC2				
1	2E+06	1_1553098	9	9	16	4	9	10	0.993074	1	7	0	5	7	4	6	0.75330063	1	7.125	4.64258241	1E+06	1572484	ID=ENSP00000375952-D2;SMOC2;;	SMOC2				
1	2E+06	1_1562080	34	17	20	13	26	17	0.164334	1	27	4	13	3	16	8	0.35023049	1	16.375	10.6360237	1E+06	1572484	ID=ENSP00000375952-D2;SMOC2;;	SMOC2	1559642	1563796	0.61044	ID=promoter_00449;
1	2E+06	1_1575602	10	25	5	11	18	8	0.059652	1	2	4	13	12	3	13	0.02959638	0.9232309	10.25	7.20614817					1575507	1576078	0.15102	ID=promoter_00454;
1	2E+06	1_1638090	244	75	153	170	160	162	0.417898	1	24	31	24	31	28	28	0.96060652	1	94	84.3936355					1636187	1638986	0.46279	ID=promoter_00472;
1	2E+06	1_1639618	22	33	22	20	28	21	0.295201	1	1	19	15	0	10	8	0.95278883	1	16.5	11.1226924					1639387	1639909	0.13908	ID=promoter_00473;
1	2E+06	1_1660392	47	39	80	45	43	63	0.530095	1	10	12	16	3	11	10	0.99899888	1	31.5	25.9835113					1657015	1661102	0.68159	ID=promoter_00480;
1	2E+06	1_1673415	7	12	3	3	10	3	0.082185	1	7	10	4	2	9	3	0.27712561	1	6	3.62530787					1672343	1674019	0.28845	ID=promoter_00484;
1	2E+06	1_1689747	52	19	42	29	36	36	0.794914	1	35	24	22	0	30	11	0.02428668	0.7953491	27.875	15.7610505					1687316	1689771	0.5545	ID=promoter_00491;
1	2E+06	1_169597	7	0	8	4	4	6	0.929647	1	90	7	11	1	49	6	2.40E-07	4.64E-05	16	30.1235551								
1	2E+06	1_1704499	0	5	8	1	3	5	0.88968	1	26	2	10	1	14	6	0.26891969	1	6.625	8.61788008					1704018	1706282	0.30362	ID=promoter_00498;
1	2E+06	1_172211	6	28	14	17	17	16	0.818283	1	0	0	4	1	0	3	0.19828566	1	8.75	10.0676285								

1	2E+06	1_1754299	139	13	92	57	76	75	0.568479	1	23	48	21	23	36	22	0.30031675	1	52	43.7100838					1753871	1754740	0.21487	ID=promoter_00516;
1	2E+06	1_1762923	26	4	24	32	15	28	0.341844	1	0	0	0	0	0	0	1	1	10.75	13.977022	2E+06	1766329	ID=ENSP0000355760;DACT2;NM_214462;	DACT2	1760356	1763950	0.58013	ID=promoter_00518;
1	2E+06	1_1777811	24	5	10	23	15	17	0.995306	1	6	6	0	5	6	3	0.67473421	1	9.875	8.83883476					1777457	1779237	0.24393	ID=promoter_00521;
1	2E+06	1_182962	0	13	4	1	7	3	0.314307	1	0	27	7	1	14	4	0.08931834	1	6.625	9.36463713								
1	2E+06	1_1840973	727	484	697	761	606	729	0.89974	1	361	394	396	78	378	237	1.26E-05	0.0015403	487.25	232.031864					1840259	1843623	0.78558	ID=promoter_00543;
1	2E+06	1_186083	83	81	245	340	82	293	7.52E-20	#######	93	68	144	140	81	142	1.98E-05	0.0022985	149.25	95.9758154								
1	2E+06	1_1868262	50	56	57	73	53	65	0.971414	1	28	27	50	38	28	44	0.08728134	1	47.375	15.6564683					1866839	1868611	0.23425	ID=promoter_00555;
1	2E+06	1_1892146	170	44	101	87	107	94	0.121828	1	78	65	30	17	72	24	3.48E-05	0.0037136	74	48.2493523					1891588	1892578	0.28095	ID=promoter_00561;
1	2E+06	1_1894713	337	162	224	280	250	252	0.235963	1	170	50	128	75	110	102	0.99999839	1	178.25	98.280575					1893636	1896430	0.43246	ID=promoter_00562;
1	2E+06	1_1950041	349	75	219	165	212	192	0.033773	1	12	26	35	44	19	40	0.01024281	0.401014	115.63	119.307451					1948998	1950389	0.27355	ID=promoter_00583;
1	2E+06	1_1979351	12	6	10	5	9	8	0.848833	1	0	2	1	6	1	4	0.36109265	1	5.25	4.23421438					1977640	1980687	0.60178	ID=promoter_00594;
1	2E+06	1_200990	199	72	172	145	136	159	0.999607	1	116	112	105	53	114	79	0.13674027	1	121.75	48.7610793								
1	2E+06	1_2048020	34	43	44	23	39	34	0.446846	1	15	14	44	10	15	27	0.11059826	1	28.375	14.5694151					2046654	2056016	0.98462	ID=promoter_00620;
1	2E+06	1_2059922	28	29	25	35	29	30	0.890024	1	39	43	47	26	41	37	0.9929878	1	34	8.29802042					2059818	2060417	0.14645	ID=promoter_00622;
1	2E+06	1_2089311	22	110	28	25	66	27	8.72E-06	0.00107	43	54	43	52	49	48	0.96380713	1	47.125	28.1447153					2086535	2094538	0.96186	ID=promoter_00633;
1	2E+06	1_2096538	80	40	62	29	60	46	0.091653	1	102	70	36	92	86	64	0.41098218	1	63.875	27.0155379					2094747	2097297	0.38913	ID=promoter_00634;
1	2E+06	1_2100243	0	26	32	11	13	22	0.573969	1	7	0	13	8	4	11	0.14780855	1	12.125	11.5069855					2099280	2101900	0.44711	ID=promoter_00636;
1	2E+06	1_2111336	396	315	436	429	356	433	0.879323	1	256	127	275	120	192	198	0.57487744	1	294.25	124.901962					2110789	2112135	0.2951	ID=promoter_00638;
1	2E+06	1_2115020	194	87	106	78	141	92	5.26E-05	0.00506	0	17	0	1	9	1	0.05455957	1	60.375	69.3375542					2114544	2117574	0.45799	ID=promoter_00639;
1	2E+06	1_2135136	34	37	28	11	36	20	0.023274	0.79554	15	13	15	37	14	26	0.10164664	1	23.75	11.3735282					2131655	2136106	0.77918	ID=promoter_00644;
1	2E+06	1_2137613	40	0	16	16	20	16	0.517469	1	1	3	16	4	2	10	0.05067035	1	12	13.3202317					2136456	2141614	0.82109	ID=promoter_00645;
1	2E+06	1_2140184	9	14	16	9	12	13	0.980472	1	0	4	14	1	2	8	0.1303879	1	8.375	6.16296543					2136456	2141614	0.82109	ID=promoter_00645;
1	2E+06	1_2141186	65	107	80	42	86	61	0.009946	0.40658	122	36	87	38	79	63	0.67166335	1	72.125	32.5463955					2136456	2141614	0.82109	ID=promoter_00645;
1	2E+06	1_214967	3	14	14	11	9	13	0.89076	1	9	0	7	0	5	4	0.97610903	1	7.25	5.75077635								
1	2E+06	1_2153866	60	5	24	7	33	16	0.011269	0.44898	29	3	21	57	16	39	0.00325528	0.1634313	25.75	22.3143132					2153141	2154792	0.27622	ID=promoter_00649;
1	2E+06	1_2162965	20	3	39	3	12	21	0.538277	1	20	34	16	11	27	14	0.20179887	1	18.25	13.1339255					2162846	2163354	0.19238	ID=promoter_00650;
1	2E+06	1_2166128	38	13	40	29	26	35	0.867873	1	31	12	13	2	22	8	0.06315916	1	22.25	13.9974487					2164057	2166667	0.41547	ID=promoter_00651;

1	2E+06	1_2169257	38	47	29	23	43	26	0.024991	0.84297	24	43	38	52	34	45	0.28919113	1	36.75	10.633505					2166916	2169781	0.48599	ID=promoter_00652;
1	2E+06	1_2182379	12	68	31	77	40	54	0.796576	1	69	76	36	13	73	25	4.44E-05	0.0045939	47.75	27.8247064					2180841	2182528	0.22215	ID=promoter_00656;
1	2E+06	1_2188849	20	8	10	14	14	12	0.725787	1	6	5	7	9	6	8	0.79537884	1	9.875	4.94072002					2187651	2189362	0.31244	ID=promoter_00657;
1	2E+06	1_2222065	8	12	5	7	10	6	0.41703	1	32	2	8	13	17	11	0.64636287	1	10.875	9.23405962					2221008	2222797	0.35499	ID=promoter_00667;
1	2E+06	1_224251	8	22	24	10	15	17	0.995306	1	33	19	14	1	26	8	0.01635039	0.5814583	16.375	10.1831443								
1	2E+06	1_225364	69	53	38	62	61	50	0.168052	1	36	28	32	14	32	23	0.64682985	1	41.5	18.4390889								
1	2E+06	1_2292603	1411	1028	1785	2064	1220	1925	4.32E-15	#######	479	412	531	491	446	511	0.0046925	0.2164812	1025.1	655.657018					2289665	2292917	0.69091	ID=promoter_00688;
1	2E+06	1_2305873	1	12	1	1	7	1	0.062633	1	25	1	4	0	13	2	0.02644099	0.8480056	5.625	8.74948978					2304602	2306226	0.27861	ID=promoter_00691;
1	2E+06	1_232260	79	28	67	39	54	53	0.653183	1	1	8	30	19	5	25	5.78E-04	0.0398304	33.875	27.2052385								
1	2E+06	1_234703	13	27	1	26	20	14	0.325699	1	19	25	15	16	22	16	0.76021399	1	17.75	8.63133825								
1	2E+06	1_2357653	56	63	66	69	60	68	0.981356	1	42	40	29	35	41	32	0.76585638	1	50	15.3622915					2357449	2359133	0.32725	ID=promoter_00708;
1	2E+06	1_2370127	9	18	7	7	14	7	0.165008	1	24	6	11	0	15	6	0.20085144	1	10.25	7.51664819					2368798	2370538	0.2784	ID=promoter_00711;
1	2E+06	1_239551	9	14	6	9	12	8	0.45534	1	1	4	20	2	3	11	0.0740992	1	8.125	6.40172968								
1	2E+06	1_242227	72	80	44	57	76	51	0.007569	0.32601	85	100	52	58	93	55	0.0286992	0.9011995	68.5	18.9887185								
1	2E+06	1_2422950	125	66	47	112	96	80	0.076186	1	33	113	76	44	73	60	0.78755089	1	77	35.6009631					2422299	2422973	0.14048	ID=promoter_00731;
1	2E+06	1_244033	12	25	19	41	19	30	0.595918	1	49	18	27	60	34	44	0.3405954	1	31.375	16.8602449								
1	3E+06	1_252686	30	30	15	5	30	10	0.001158	0.07164	54	0	4	0	27	2	5.20E-05	0.0052176	17.25	19.2780408								
1	3E+06	1_256084	212	93	167	151	153	159	0.564371	1	40	29	8	21	35	15	0.03750218	1	90.125	77.6888253								
1	3E+06	1_2564605	15	19	25	28	17	27	0.617018	1	0	12	35	5	6	20	0.0132456	0.49354	17.375	11.7951261					2564549	2565100	0.12186	ID=promoter_00762;
1	3E+06	1_257293	54	38	6	13	46	10	4.10E-07	#######	37	49	11	38	43	25	0.17340376	1	30.75	18.2658933								
1	3E+06	1_258997	0	5	21	32	3	27	3.96E-04	0.02918	0	2	26	4	1	15	0.00126073	0.075413	11.25	12.947697								
1	3E+06	1_269290	7	73	12	10	40	11	2.07E-05	0.00227	2	1	1	2	2	2	0.99713337	1	13.5	24.4189622								
1	3E+06	1_2695705	376	147	150	135	262	143	4.56E-13	#######	106	101	56	15	104	36	7.83E-07	0.0001342	135.75	107.626006					2695492	2697317	0.29151	ID=promoter_00790;
1	3E+06	1_2700174	2	4	2	4	3	3	0.981055	1	23	9	13	2	16	8	0.35023049	1	7.375	7.44383734					2698645	2702412	0.63372	ID=promoter_00791;
1	3E+06	1_2732366	8	23	14	24	16	19	0.999341	1	35	15	46	18	25	32	0.47866995	1	22.875	12.3570396	3E+06	2794532	ID=ENSP00000386050;RPS6KA2;;	RPS6KA2				
1	3E+06	1_2760863	60	108	83	83	84	83	0.540384	1	90	117	123	86	104	105	0.82670647	1	93.75	20.8515159	3E+06	2794532	ID=ENSP00000386050;RPS6KA2;;	RPS6KA2	2760853	2764075	0.50757	ID=promoter_00807;
1	3E+06	1_2765298	19	37	28	35	28	32	0.994967	1	13	16	33	34	15	34	0.01174317	0.4468879	26.875	9.49341877	3E+06	2794532	ID=ENSP00000386050;RPS6KA2;;	RPS6KA2				
1	3E+06	1_2791491	43	33	15	13	38	14	4.70E-04	0.03357	0	0	3	1	0	2	0.34003807	1	13.5	16.405574	3E+06	2794532	ID=ENSP00000386050;RPS6KA2;;	RPS6KA2				
1	3E+06	1_281261	12	28	8	7	20	8	0.026297	0.87605	23	6	5	0	15	3	0.02833385	0.8918846	11.125	9.56836305								
1	3E+06	1_2816343	13	0	4	14	7	9	0.983538	1	0	0	19	17	0	18	6.08E-05	0.0059624	8.375	8.1929848					2815742	2816945	0.32685	ID=promoter_00821;
1	3E+06	1_284315	25	17	18	20	21	19	0.713172	1	1	0	4	1	1	3	0.56022579	1	10.75	10.2225242								
1	3E+06	1_2891924	26	16	8	2	21	5	0.001802	0.10311	23	19	8	0	21	4	0.00573189	0.2548307	12.75	9.66215001					2891345	2892448	0.26074	ID=promoter_00842;
1	3E+06	1_2902939	14	0	24	25	7	25	0.023401	0.7984	0	16	9	3	8	6	0.92562801	1	11.375	10.0560927					2901839	2903397	0.28987	ID=promoter_00846;

1	3E+06	1_2907476	16	42	28	22	29	25	0.526429	1	86	57	65	60	72	63	0.94544753	1	47	24.1601798					2905823	2909059	0.50338	ID=promoter_00848;
1	3E+06	1_291223	17	7	24	16	12	20	0.626521	1	35	27	26	37	31	32	0.91294871	1	23.625	10.0276404								
1	3E+06	1_294293	7	9	4	0	8	2	0.097885	1	12	15	6	6	14	6	0.26891969	1	7.375	4.65794253								
1	3E+06	1_2966218	135	99	153	130	117	142	0.96333	1	93	78	63	55	86	59	0.2023408	1	100.75	35.5718589					2965231	2966533	0.33437	ID=promoter_00868;
1	3E+06	1_3000206	37	13	33	32	25	33	0.905451	1	54	26	71	79	40	75	0.00117882	0.0720531	43.125	22.8250332					2999997	3001734	0.2225	ID=promoter_00877;
1	3E+06	1_3031100	14	18	4	19	16	12	0.497978	1	2	11	17	3	7	10	0.67636194	1	11	7.09124208					3030736	3034786	0.64695	ID=promoter_00884;
1	3E+06	1_3033525	19	10	10	26	15	18	0.997876	1	13	3	24	9	8	17	0.13786384	1	14.25	7.99553447					3030736	3034786	0.64695	ID=promoter_00884;
1	3E+06	1_3043781	496	87	290	200	292	245	5.28E-04	0.03699	157	29	42	41	93	42	4.05E-04	0.0297464	167.75	161.02329					3041997	3045253	0.44594	ID=promoter_00888;
1	3E+06	1_305120	8	12	3	5	10	4	0.162164	1	13	2	12	6	8	9	0.92351511	1	7.625	4.30738568								
1	3E+06	1_3055855	30	46	9	25	38	17	0.002761	0.14629	28	13	1	68	21	35	0.09793111	1	27.5	21.5472902					3054747	3056691	0.31111	ID=promoter_00891;
1	3E+06	1_308438	12	25	14	9	19	12	0.233425	1	21	15	3	10	18	7	0.13207736	1	13.625	6.92691438								
1	3E+06	1_3138478	18	5	12	17	12	15	0.986595	1	2	29	7	2	16	5	0.08348313	1	11.5	9.42640668					3138015	3139916	0.29053	ID=promoter_00905;
1	3E+06	1_3150533	11	24	3	9	18	6	0.017303	0.62786	37	23	16	3	30	10	0.01387572	0.5103594	15.75	11.7442995	3E+06	3163847	ID=ENSP0000355812;FGFR1OP;NM_007045;	FGFR1OP	3147872	3151874	0.63465	ID=promoter_00909;
1	3E+06	1_316916	391	136	303	266	264	285	0.623648	1	193	54	115	42	124	79	0.03224386	0.9875313	187.5	124.123211								
1	3E+06	1_318096	80	128	90	98	104	94	0.185198	1	97	20	53	23	59	38	0.21227106	1	73.625	38.3030678								
1	3E+06	1_319241	30	13	26	11	22	19	0.617881	1	23	40	43	58	32	51	0.05193594	1	30.5	15.8835045								
1	3E+06	1_3227390	5	18	30	40	12	35	0.018854	0.672	21	8	54	1	15	28	0.0827714	1	22.125	18.3415025					3227230	3227983	0.18757	ID=promoter_00927;
1	3E+06	1_3238761	98	118	80	99	108	90	0.055221	1	61	35	78	3	48	41	0.9285271	1	71.5	37.5499667					3237228	3238965	0.2225	ID=promoter_00928;
1	3E+06	1_3250470	6	4	6	11	5	9	0.74134	1	21	26	14	1	24	8	0.03264408	0.9940318	11.125	8.72496418					3249487	3250637	0.24681	ID=promoter_00932;
1	3E+06	1_325706	10	8	4	14	9	9	0.944235	1	20	11	16	1	16	9	0.48000706	1	10.5	6.23354978								
1	3E+06	1_3259121	98	96	89	61	97	75	0.024077	0.81932	114	98	60	78	106	69	0.07137274	1	86.75	19.0544333					3258621	3259684	0.20507	ID=promoter_00935;
1	3E+06	1_328651	189	296	106	164	243	135	9.47E-12	#######	227	296	295	184	262	240	0.99109449	1	219.63	71.32809								
1	3E+06	1_3288139	93	77	70	77	85	74	0.170179	1	40	17	67	33	29	50	0.02604249	0.8420339	59.25	26.15749					3287861	3289224	0.24445	ID=promoter_00944;
1	3E+06	1_3292976	11	28	11	19	20	15	0.418324	1	7	5	33	4	6	19	0.02032977	0.6899793	14.75	10.8594922					3291134	3294251	0.49229	ID=promoter_00945;
1	3E+06	1_335127	6	6	2	2	6	2	0.258649	1	0	24	5	0	12	3	0.09316162	1	5.625	7.81824789								
1	3E+06	1_340243	172	70	179	84	121	132	0.847003	1	0	15	11	1	8	6	0.92562801	1	66.5	74.2101264								
1	3E+06	1_344762	85	107	89	44	96	67	0.004345	0.20959	20	45	30	1	33	16	0.09595749	1	52.625	37.2287811								
1	3E+06	1_3465226	5	5	13	26	5	20	0.033145	1	7	0	12	26	4	19	0.00415462	0.1961166	11.75	9.70640437					3464727	3465242	0.11428	ID=promoter_00967;
1	4E+06	1_356355	5	30	27	8	18	18	0.89158	1	0	0	7	0	0	4	0.11562589	1	9.625	12.1059312								
1	4E+06	1_3567363	10	1	5	9	6	7	0.999953	1	13	5	6	0	9	3	0.27712561	1	6.125	4.42194204					3566836	3568783	0.38309	ID=promoter_00974;
1	4E+06	1_356908	12	1	60	27	7	44	2.24E-05	0.00242	13	46	18	23	30	21	0.61223556	1	25	19.316906								
1	4E+06	1_357532	13	16	11	6	15	9	0.269309	1	0	0	15	10	0	13	9.01E-04	0.0577931	8.875	6.28916301								
1	4E+06	1_357853	44	10	6	1	27	4	2.68E-05	0.00281	15	5	3	1	10	2	0.09294023	1	10.625	14.2922307								
1	4E+06	1_363476	26	26	22	4	26	13	0.035221	1	0	14	12	1	7	7	0.99000271	1	13.125	10.7893797								
1	4E+06	1_366581	24	28	5	0	26	3	1.49E-05	0.00168	19	28	11	1	24	6	0.00867909	0.3505674	14.5	11.7716366								
1	4E+06	1_3736213	38	37	30	35	38	33	0.447527	1	87	22	17	5	55	11	2.11E-06	0.0003257	33.875	24.27484					3735331	3739562	0.84497	ID=promoter_00985;
1	4E+06	1_387846	4	8	20	12	6	16	0.210908	1	12	10	10	6	11	8	0.87190251	1	10.25	4.83292281								

1	4E+06	1_3906932	19	34	0	1	27	1	6.04E-07	#######	31	14	21	11	23	16	0.67590535	1	16.375	12.4892811					3906055	3908154	0.38286	ID=promoter_01007;
1	4E+06	1_390993	14	7	6	12	11	9	0.722819	1	168	103	159	36	136	98	0.16249222	1	63.125	69.6489924								
1	4E+06	1_395648	15	13	19	13	14	16	0.99748	1	10	4	2	0	7	1	0.13113995	1	9.5	6.78232998								
1	4E+06	1_4003472	1	13	10	8	7	9	0.983538	1	1	19	1	4	10	3	0.19568363	1	7.125	6.6211242	4E+06	4174593	ID=ENSP0000375972;PDE10A;;	PDE10A				
1	4E+06	1_4113132	13	5	3	5	9	4	0.244237	1	17	0	6	21	9	14	0.47220951	1	8.75	7.38241153	4E+06	4174593	ID=ENSP0000375972;PDE10A;;	PDE10A				
1	4E+06	1_4150613	7	9	36	31	8	34	0.002133	0.1181	0	8	9	0	4	5	0.90483023	1	12.5	13.5330284	4E+06	4174593	ID=ENSP0000375972;PDE10A;;	PDE10A				
1	4E+06	1_4164730	9	49	21	16	29	19	0.13502	1	43	21	20	6	32	13	0.03583578	1	23.125	15.2450226	4E+06	4174593	ID=ENSP0000375972;PDE10A;;	PDE10A	4164670	4166141	0.21655	ID=promoter_01039;
1	4E+06	1_4171908	13	19	5	1	16	3	0.003782	0.18716	11	2	9	1	7	5	0.9051169	1	7.625	6.52330985	4E+06	4174593	ID=ENSP0000375972;PDE10A;;	PDE10A	4171766	4172341	0.13687	ID=promoter_01041;
1	4E+06	1_4174677	51	74	25	27	63	26	1.69E-05	0.00188	39	29	48	12	34	30	0.98073068	1	38.125	19.3201412					4174165	4175151	0.19437	ID=promoter_01042;
1	4E+06	1_4177704	20	15	23	22	18	23	0.963662	1	1	6	5	2	4	4	0.99427496	1	11.75	9.25434261					4176981	4178224	0.25491	ID=promoter_01043;
1	4E+06	1_4178137	0	11	10	0	6	5	0.851367	1	2	0	33	0	1	17	4.35E-04	0.0314643	7	11.4766844					4176981	4178224	0.25491	ID=promoter_01043;
1	4E+06	1_4183160	0	6	1	8	3	5	0.88968	1	1	1	5	21	1	13	0.0036419	0.1775497	5.375	6.94750726					4183049	4184048	0.22439	ID=promoter_01045;
1	4E+06	1_429800	510	230	308	345	370	327	9.11E-04	0.05851	287	186	185	49	237	117	1.05E-07	2.17E-05	262.5	135.87284								
1	4E+06	1_431916	12	0	9	11	6	10	0.79153	1	0	2	30	3	1	17	4.35E-04	0.0314643	8.375	9.98480989								
1	5E+06	1_467970	1	0	18	28	1	23	2.11E-04	0.01699	0	0	0	0	0	0	1	1	5.875	10.9078936								
1	5E+06	1_4993856	61	97	93	53	79	73	0.338129	1	74	47	37	64	61	51	0.86215916	1	65.75	21.2249853					4993411	4994213	0.14193	ID=promoter_01064;
1	5E+06	1_5011458	10	5	28	6	8	17	0.371907	1	6	32	5	8	19	7	0.09662636	1	12.5	10.9805022					5011091	5011675	0.13162	ID=promoter_01065;
1	5E+06	1_502221	6	26	12	5	16	9	0.196587	1	1	5	4	0	3	2	0.9363008	1	7.375	8.34843869								
1	5E+06	1_507878	31	96	35	58	64	47	0.048525	1	21	50	39	25	36	32	0.98514677	1	44.375	24.2011659								
1	5E+06	1_513284	2	0	2	25	1	14	0.009319	0.38477	8	0	35	1	4	18	0.00668749	0.2868305	9.125	13.3997601								
1	5E+06	1_517949	1	23	2	6	12	4	0.066899	1	4	17	10	21	11	16	0.51039395	1	10.5	8.73416935								
1	5E+06	1_5182993	48	24	36	51	36	44	0.983377	1	3	8	11	18	6	15	0.10150651	1	24.875	18.3025174					5182771	5183355	0.13162	ID=promoter_01070;
1	5E+06	1_542739	14	57	33	24	36	29	0.317511	1	84	24	44	18	54	31	0.0997364	1	37.25	23.6084125								
1	5E+06	1_543369	12	9	0	9	11	5	0.189421	1	10	3	3	1	7	2	0.29886173	1	5.875	4.6117087								
1	5E+06	1_545937	24	8	3	18	16	11	0.385778	1	13	9	19	0	11	10	0.99899888	1	11.75	8.27647268								
1	5E+06	1_549470	139	67	89	68	103	79	0.016654	0.61076	14	34	22	20	24	21	0.98147478	1	56.625	42.9948087								
1	6E+06	1_556003	0	1	7	12	1	10	0.048828	1	1	0	20	0	1	10	0.01766414	0.6154554	5.125	7.41499446								
1	6E+06	1_5777156	96	21	70	26	59	48	0.166754	1	17	32	6	37	25	22	0.9842524	1	38.125	30.0544149					5776616	5777790	0.30508	ID=promoter_01086;
1	6E+06	1_590455	18	23	9	6	21	8	0.017144	0.62492	12	12	22	3	12	13	0.92692342	1	13.125	7.29848321								
1	6E+06	1_616114	66	44	86	44	55	65	0.999156	1	0	7	24	4	4	14	0.04184353	1	34.375	31.1949515								
1	6E+06	1_625306	48	69	41	17	59	29	3.98E-04	0.02931	84	70	87	68	77	78	0.85865745	1	60.5	23.6099253								
1	6E+06	1_6296466	270	65	221	189	168	205	0.930423	1	132	92	72	18	112	45	6.86E-06	0.000912	132.38	86.9300827					6295374	6297565	0.56052	ID=promoter_01106;
1	6E+06	1_637662	7	24	8	8	16	8	0.127562	1	0	15	17	37	8	27	0.00271213	0.139047	14.5	11.6741473								
1	6E+06	1_639324	16	17	10	10	17	10	0.210142	1	3	0	13	90	2	52	1.24E-11	4.56E-09	19.875	28.9405178								
1	6E+06	1_639328	22	14	6	16	18	11	0.22238	1	0	0	4	0	0	2	0.34003807	1	7.75	8.51469318								
1	6E+06	1_644959	0	20	5	1	10	3	0.082185	1	1	7	7	1	4	4	0.99427496	1	5.25	6.60627407								
1	7E+06	1_671387	3	19	5	22	11	14	0.979671	1	0	14	36	3	7	20	0.02608553	0.8424716	12.75	12.4183045								
1	7E+06	1_6757540	5	15	7	1	10	4	0.162164	1	9	3	3	1	6	2	0.42506085	1	5.5	4.75093976					6757040	6757602	0.12182	ID=promoter_01114;
1	7E+06	1_683556	2	30	8	4	16	6	0.042495	1	32	78	1	7	55	4	1.72E-09	4.80E-07	20.25	26.3533679								
1	7E+06	1_688197	308	312	11	13	310	12	8.30E-72	#######	159	142	7	4	151	6	1.65E-27	1.61E-24	119.5	132.969384								

1	7E+06	1_708325	8	2	3	0	5	2	0.402696	1	21	11	0	0	16	0	6.01E-04	0.0409216	5.625	7.4246212								
1	7E+06	1_735520	12	18	30	10	15	20	0.931941	1	45	30	22	1	38	12	0.00296809	0.1507727	21	13.8873015								
1	7E+06	1_735879	25	47	19	27	36	23	0.069202	1	67	35	60	19	51	40	0.73048207	1	37.375	18.6082739								
1	7E+06	1_746867	371	92	133	166	232	150	5.91E-08	#######	44	33	34	11	39	23	0.22193731	1	110.5	118.303484								
1	7E+06	1_7494463	6	34	32	18	20	25	0.977759	1	13	0	3	20	7	12	0.42229866	1	15.75	12.7251158					7493869	7494786	0.16749	ID=promoter_01140;
1	8E+06	1_7510136	31	22	27	42	27	35	0.926583	1	18	14	22	11	16	17	0.92610249	1	23.375	9.94179489					7509140	7510313	0.28007	ID=promoter_01142;
1	8E+06	1_7577685	41	71	27	50	56	39	0.040954	1	108	80	35	46	94	41	2.02E-04	0.0165855	57.25	27.0858424					7577571	7578158	0.18624	ID=promoter_01148;
1	8E+06	1_7602859	8	19	0	14	14	7	0.165008	1	0	10	5	4	5	5	0.99284883	1	7.5	6.67618368	8E+06	7671945	ID=ENSP00000314036;AGPAT4;NM_020133;	AGPAT4				
1	8E+06	1_7614738	132	130	34	41	131	38	2.22E-15	#######	86	105	27	14	96	21	5.54E-10	1.69E-07	71.125	47.8642052	8E+06	7671945	ID=ENSP00000314036;AGPAT4;NM_020133;	AGPAT4	7614275	7615360	0.19764	ID=promoter_01154;
1	8E+06	1_7638647	38	32	32	20	35	26	0.205248	1	65	22	29	36	44	33	0.65373342	1	34.25	13.9052919	8E+06	7671945	ID=ENSP00000314036;AGPAT4;NM_020133;	AGPAT4	7638305	7639049	0.12727	ID=promoter_01157;
1	8E+06	1_7648543	0	2	0	9	1	5	0.353258	1	39	9	81	0	24	41	0.05407158	1	17.5	28.7799533	8E+06	7671945	ID=ENSP00000314036;AGPAT4;NM_020133;	AGPAT4				
1	8E+06	1_7654691	2	7	12	11	5	12	0.389128	1	2	1	9	0	2	5	0.46647814	1	5.5	4.81070235	8E+06	7671945	ID=ENSP00000314036;AGPAT4;NM_020133;	AGPAT4				
1	8E+06	1_7662270	1	7	10	18	4	14	0.128496	1	22	18	16	0	20	8	0.11762508	1	11.5	8.28078671	8E+06	7671945	ID=ENSP00000314036;AGPAT4;NM_020133;	AGPAT4				
1	8E+06	1_7728629	118	122	129	122	120	126	0.685761	1	190	96	99	232	143	166	0.1421332	1	138.5	47.5334469					7727719	7729198	0.3985	ID=promoter_01181;
1	8E+06	1_781013	13	5	11	12	9	12	0.95859	1	28	6	1	9	17	5	0.05852787	1	10.625	8.08768906								
1	8E+06	1_783560	3	8	7	5	6	6	0.962469	1	11	8	1	1	10	1	0.03495212	1	5.5	3.62530787								
1	8E+06	1_784175	36	109	42	45	73	44	0.001813	0.1035	143	149	138	106	146	122	0.69866377	1	96	48.0594869								
1	8E+06	1_793063	430	224	657	500	327	579	1.57E-08	#######	59	66	87	162	63	125	2.98E-06	0.0004391	273.13	227.281096								
1	8E+06	1_797445	43	39	47	48	41	48	0.99996	1	5	8	15	3	7	9	0.80856893	1	26	19.9928559								
1	8E+06	1_8000190	248	111	253	126	180	190	0.59782	1	8	4	48	34	6	41	5.51E-07	9.74E-05	104	100.514391	8E+06	8048023	ID=ENSP00000375986-D2;MAP3K4;NM_005922;	MAP3K4				
1	8E+06	1_8016711	48	17	20	47	33	34	0.868497	1	3	10	19	1	7	10	0.67636194	1	20.625	18.0074389	8E+06	8048023	ID=ENSP00000375986-D2;MAP3K4;NM_005922;	MAP3K4	8015540	8016898	0.32844	ID=promoter_01222;
1	8E+06	1_8018858	7	10	1	20	9	11	0.995818	1	0	19	2	3	10	3	0.19568363	1	7.75	7.95972002	8E+06	8048023	ID=ENSP00000375986-D2;MAP3K4;NM_005922;	MAP3K4				
1	8E+06	1_8037009	0	5	3	5	3	4	0.986002	1	17	2	16	7	10	12	0.83305499	1	6.875	6.31183695	8E+06	8048023	ID=ENSP00000375986-D2;MAP3K4;NM_005922;	MAP3K4	8035804	8037750	0.19969	ID=promoter_01227;
1	8E+06	1_808768	5	8	10	10	7	10	0.922856	1	56	51	53	50	54	52	0.98103932	1	30.375	23.7663478								
1	8E+06	1_812461	272	199	137	122	236	130	1.25E-11	#######	35	17	30	3	26	17	0.53107524	1	101.88	97.5652712								

1	8E+06	1_825605	7	8	2	23	8	13	0.766846	1	0	31	4	0	16	2	0.00719486	0.3031284	9.375	11.4634512								
1	8E+06	1_832401	9	0	13	20	5	17	0.090491	1	8	1	1	0	5	1	0.30486099	1	6.5	7.34846923								
1	8E+06	1_8360651	497	2544	367	295	1521	331	########	#######	116	338	83	68	227	76	1.07E-14	5.34E-12	538.5	824.622338					8360240	8360971	0.13126	ID=promoter_01258;
1	8E+06	1_840210	12	13	7	0	13	4	0.041893	1	11	7	4	1	9	3	0.27712561	1	6.875	4.94072002								
1	8E+06	1_8432647	27	8	23	18	18	21	0.99986	1	8	2	4	5	5	5	0.99284883	1	11.875	9.46327488					8432276	8432861	0.11258	ID=promoter_01266;
1	9E+06	1_8528408	6	14	1	7	10	4	0.162164	1	0	0	3	22	0	13	9.01E-04	0.0577931	6.625	7.78161753	8E+06	8581400	ID=ENSP0000386019;IGF2R;;	IGF2R	8527649	8529406	0.39462	ID=promoter_01290;
1	9E+06	1_8541488	25	41	30	30	33	30	0.598685	1	77	49	66	62	63	64	0.87546314	1	47.5	19.2205247	8E+06	8581400	ID=ENSP0000386019;IGF2R;;	IGF2R	8538583	8544298	0.81889	ID=promoter_01293;
1	9E+06	1_8558630	13	27	11	28	20	20	0.880283	1	26	7	18	21	17	20	0.769441	1	18.875	7.95410945	8E+06	8581400	ID=ENSP0000386019;IGF2R;;	IGF2R	8557457	8559973	0.45458	ID=promoter_01298;
1	9E+06	1_8566577	4	5	3	4	5	4	0.848147	1	5	0	17	5	3	11	0.0740992	1	5.375	4.98390266	8E+06	8581400	ID=ENSP0000386019;IGF2R;;	IGF2R	8565147	8567051	0.27077	ID=promoter_01301;
1	9E+06	1_861109	3	6	2	4	5	3	0.645778	1	3	11	16	10	7	13	0.3188207	1	6.875	4.96955014								
1	9E+06	1_8616031	58	25	15	26	42	21	0.004493	0.21484	68	24	13	5	46	9	1.51E-05	0.0018118	29.25	22.1665772					8615633	8616203	0.12449	ID=promoter_01309;
1	9E+06	1_8775252	447	104	227	252	276	240	0.003051	0.15794	215	68	82	254	142	168	0.10024675	1	206.13	124.123714					8774768	8776763	0.78535	ID=promoter_01324;
1	9E+06	1_896173	36	51	43	48	44	46	0.861515	1	1	11	15	3	6	9	0.65381141	1	26	20.695065								
1	9E+06	1_915317	25	29	11	20	27	16	0.087673	1	15	13	18	3	14	11	0.91900834	1	16.75	8.1897148								
1	9E+06	1_9166948	3	29	3	1	16	2	0.001284	0.07728	48	25	9	1	37	5	1.63E-05	0.0019379	14.875	17.3241161					9166596	9167451	0.15124	ID=promoter_01348;
1	9E+06	1_9218854	7887	5891	13487	14798	6889	####	0	0	6143	5585	13395	6819	5864	10107	0	0	9250.6	3928.82352					9218443	9220588	0.60299	ID=promoter_01357;
1	9E+06	1_923433	27	21	23	60	24	42	0.289742	1	3	27	74	91	15	83	3.88E-12	1.51E-09	40.75	30.4947302								
1	9E+06	1_925655	4	0	9	3	2	6	0.490921	1	0	20	9	18	10	14	0.60490119	1	7.875	7.69856202								
1	9E+06	1_9363514	0	2	0	2	1	1	0.993645	1	39	0	0	0	20	0	9.42E-05	0.0087339	5.375	13.6165603	9E+06	9379511	ID=ENSP0000252655;RSPH3;NM_031924;	RSPH3				
1	9E+06	1_941457	4	29	7	8	17	8	0.087954	1	10	15	19	4	13	12	0.99994263	1	12	8.61891607								
1	9E+06	1_948014	12	5	4	14	9	9	0.944235	1	25	1	32	2	13	17	0.64388579	1	11.875	11.3821602								
1	9E+06	1_9484956	100	9	77	68	55	73	0.786701	1	30	32	31	3	31	17	0.21261815	1	43.75	34.2584713					9484864	9486269	0.41766	ID=promoter_01382;
1	1E+07	1_959160	15	8	22	38	12	30	0.076246	1	11	18	26	3	15	15	0.97869956	1	17.625	11.0961705								
1	1E+07	1_9608817	6	18	13	2	12	8	0.45534	1	124	13	32	36	69	34	0.00907631	0.3628056	30.5	39.5618864					9608059	9609318	0.19668	ID=promoter_01406;
1	1E+07	1_9674804	3	28	5	2	16	4	0.009581	0.39335	11	9	3	27	10	15	0.49251655	1	11	10.650285	1E+07	9725779	ID=ENSP0000297239;SYTL3;;	SYTL3				
1	1E+07	1_9729119	66	38	48	32	52	40	0.129757	1	15	23	13	10	19	12	0.57792516	1	30.625	19.4491094					9728914	9729749	0.23541	ID=promoter_01422;
1	1E+07	1_976540	19	55	4	21	37	13	3.79E-04	0.02808	46	23	32	6	35	19	0.16432451	1	25.75	17.9025138								
1	1E+07	1_976998	9	3	13	11	6	12	0.558139	1	20	22	19	3	21	11	0.29862283	1	12.5	7.4065608								
1	1E+07	1_978145	110	73	167	126	92	147	0.065539	1	36	14	9	3	25	6	0.00589984	0.2600594	67.25	61.6389255								
1	1E+07	1_986747	90	75	90	72	83	81	0.498938	1	27	30	26	34	29	30	0.9150801	1	55.5	28.8543139								
1	1E+07	1_989376	12	32	18	7	22	13	0.135741	1	20	9	24	2	15	13	0.98432481	1	15.5	9.85610761								
1	1E+07	1_996243	1	6	2	0	4	1	0.312866	1	27	8	13	16	18	15	0.95456211	1	9.125	9.20306626								
1	1E+07	1_999511	91	86	123	77	89	100	0.957409	1	23	46	52	30	35	41	0.59499216	1	66	34.0923117								
1	1E+07	1_101499	8	13	1	0	11	1	0.006522	0.28949	0	5	0	23	3	12	0.04708211	1	6.25	8.24187912								
1	1E+07	1_101609	8	0	7	6	4	7	0.813458	1	3	9	4	3	6	4	0.87665919	1	5	3.02371578								
1	1E+07	1_102353	11	52	20	13	32	17	0.02552	0.85718	82	42	47	22	62	35	0.06026057	1	36.125	24.3042471								
1	1E+07	1_104414	10	17	8	3	14	6	0.098789	1	6	3	1	3	5	2	0.58563225	1	6.375	5.23552426								

1	1E+07	1_10509363	6	21	9	21	14	15	0.970741	1	21	16	20	1	19	11	0.45547225	1	14.375	7.96308447	1E+07	1.1E+07	ID=ENSP0000352821;ZDHHC14;NM_024630;	ZDHHC14				
1	1E+07	1_105744	1	36	20	18	19	19	0.885913	1	14	14	16	58	14	37	0.00223765	0.1207017	22.125	17.3899602								
1	1E+07	1_106443	36	46	12	31	41	22	0.009991	0.40815	35	18	34	51	27	43	0.08307182	1	32.875	12.9663025								
1	1E+07	1_10716500	8	13	2	1	11	2	0.020291	0.71411	14	0	0	2	7	1	0.13113995	1	5	5.83095189					1.1E+07	1.1E+07	0.35608	ID=promoter_01491;
1	1E+07	1_10749060	67	20	34	73	44	54	0.975459	1	0	0	0	0	0	0	1	1	24.25	30.8672272					1.1E+07	1.1E+07	0.13601	ID=promoter_01500;
1	1E+07	1_107705	72	19	45	51	46	48	0.85141	1	6	38	35	1	22	18	0.92557456	1	33.375	23.8023858								
1	1E+07	1_111154	0	0	14	19	0	17	7.13E-04	0.0478	8	0	0	26	4	13	0.06462238	1	8.375	10.2530483								
1	1E+07	1_111481	23	24	14	23	24	19	0.435991	1	41	18	37	24	30	31	0.91402185	1	25.5	9.08688223								
1	1E+07	1_112763	6	47	5	36	27	21	0.36385	1	22	19	22	19	21	21	0.97030696	1	22	14.0610912								
1	1E+07	1_115781	10	75	1	9	43	5	1.09E-08	#######	6	1	12	28	4	20	0.00256748	0.1345788	17.75	24.6446169								
1	1E+07	1_116407	2	18	0	2	10	1	0.011548	0.45484	45	16	0	5	31	3	2.73E-05	0.0030031	11	15.4457576								
1	1E+07	1_118777	0	10	6	2	5	4	0.848147	1	31	4	0	0	18	0	2.38E-04	0.0190698	6.625	10.4599577								
1	1E+07	1_120286	31	0	4	11	16	8	0.127562	1	19	22	15	0	21	8	0.08664698	1	12.75	11.132321								
1	1E+07	1_120713	715	237	608	651	476	630	0.138075	1	302	190	199	83	246	141	2.59E-05	0.0028712	373.13	245.20396								
1	1E+07	1_12137600	9	1	7	0	5	4	0.848147	1	30	0	0	5	15	3	0.02833385	0.8918846	6.5	10.1277554					1.2E+07	1.2E+07	0.18759	ID=promoter_01541;
1	1E+07	1_12145148	27	46	12	19	37	16	0.002386	0.12916	37	25	52	36	31	44	0.1873109	1	31.75	13.5198267					1.2E+07	1.2E+07	0.15324	ID=promoter_01542;
1	1E+07	1_121660	111	115	92	128	113	110	0.378519	1	163	138	209	4	151	107	0.10286958	1	120	59.1656271								
1	1E+07	1_12212776	4	12	4	4	8	4	0.357158	1	1	12	6	8	7	7	0.99000271	1	6.375	3.99776723					1.2E+07	1.2E+07	0.29864	ID=promoter_01546;
1	1E+07	1_122663	272	124	218	271	198	245	0.855089	1	114	115	96	23	115	60	0.00123632	0.0745461	154.13	89.6460301								
1	1E+07	1_12363751	0	13	4	17	7	11	0.831726	1	9	0	4	4	5	4	0.97610903	1	6.375	6.11642986	1E+07	1.2E+07	ID=ENSP0000356142;TIAM2;;	TIAM2				
1	1E+07	1_12375445	27	47	24	14	37	19	0.010763	0.4351	74	39	34	44	57	39	0.34082797	1	37.875	18.2164565	1E+07	1.2E+07	ID=ENSP0000356142;TIAM2;;	TIAM2				
1	1E+07	1_12591236	33	65	15	25	49	20	1.71E-04	0.01419	68	16	33	29	42	31	0.627947	1	35.5	20.3259159	1E+07	1.3E+07	ID=ENSP0000159060;NOX3;NM_015718;	NOX3				
1	1E+07	1_12596240	5	23	3	5	14	4	0.02589	0.86655	29	41	23	0	35	12	0.00825069	0.3370693	16.125	14.9230167	1E+07	1.3E+07	ID=ENSP0000159060;NOX3;NM_015718;	NOX3				
1	1E+07	1_130432	66	14	21	25	40	23	0.020841	0.72739	21	8	16	0	15	8	0.44137572	1	21.375	19.7262515								
1	1E+07	1_130561	25	28	24	14	27	19	0.225928	1	31	49	20	40	40	30	0.67948902	1	28.875	11.1667555								
1	1E+07	1_13098128	14	1	16	27	8	22	0.104172	1	8	4	32	5	6	19	0.02032977	0.6899793	13.375	11.2114419	1E+07	1.3E+07	ID=ENSP0000356182;CNKSR3;NM_173515;	CNKSR3				
1	1E+07	1_131749	337	165	477	299	251	388	0.0029	0.15122	33	13	70	42	23	56	2.72E-04	0.0212224	179.5	172.330248								
1	1E+07	1_133833	0	16	9	15	8	12	0.864244	1	17	21	5	18	19	12	0.57792516	1	12.625	7.22965125								
1	1E+07	1_141778	9	32	28	21	21	25	0.998746	1	36	18	1	6	27	4	4.55E-04	0.0327286	18.875	12.7440462								
1	1E+07	1_142046	14	7	3	0	11	2	0.020291	0.71411	0	17	14	1	9	8	0.99626583	1	7	7.05083582								
1	1E+07	1_142390	19	100	15	20	60	18	3.08E-07	#######	26	18	54	18	22	36	0.10388249	1	33.75	29.5526165								
1	1E+07	1_144328	75	66	106	67	71	87	0.963348	1	18	12	6	0	15	3	0.02833385	0.8918846	43.75	39.4561239								
1	1E+07	1_147366	19	30	7	16	25	12	0.031505	1	87	8	14	0	48	7	1.03E-06	0.0001704	22.625	27.5159045								
1	1E+07	1_148565	38	88	54	42	63	48	0.076128	1	103	129	119	73	116	96	0.71274599	1	80.75	34.7305958								
1	1E+07	1_148628	1	2	1	1	2	1	0.773064	1	20	10	8	11	15	10	0.7195765	1	6.75	6.84000835								
1	1E+07	1_149622	8	0	3	9	4	6	0.929647	1	0	0	20	0	0	10	0.00454609	0.2107253	5	7.11135912								
1	2E+07	1_155765	185	157	86	130	171	108	2.06E-06	0.00029	54	34	53	100	44	77	0.00289594	0.1476228	99.875	53.7864494								
1	2E+07	1_15839815	7	11	6	9	9	8	0.848833	1	12	12	7	8	12	8	0.76853133	1	9	2.39045722	2E+07	1.6E+07	ID=ENSP0000356259;C6orf97;;	C6orf97				
1	2E+07	1_158850	17	17	4	6	17	5	0.013234	0.50759	23	19	24	1	21	13	0.51498325	1	13.875	8.91928088								
1	2E+07	1_159265	9	18	0	0	14	0	2.72E-04	0.02125	38	33	7	7	36	7	1.63E-04	0.0138451	14	14.4815153								

1	2E+07	1_16010314	10	13	6	6	12	6	0.213448	1	42	12	8	5	27	7	0.00575936	0.2548307	12.75	12.174329					1.6E+07	1.6E+07	0.3979	ID=promoter_01616;
1	2E+07	1_16035304	9	52	11	14	31	13	0.005055	0.23696	12	22	22	71	17	47	2.68E-04	0.0209332	26.625	22.6270224					1.6E+07	1.6E+07	0.23649	ID=promoter_01618;
1	2E+07	1_160738	2	29	16	23	16	20	0.982167	1	53	26	48	8	40	28	0.51986598	1	25.625	17.8480691								
1	2E+07	1_161713	199	89	141	185	144	163	0.952731	1	221	225	108	110	223	109	1.82E-07	3.61E-05	159.75	54.3763341								
1	2E+07	1_16254192	2	26	8	13	14	11	0.605836	1	5	0	8	23	3	16	0.00701108	0.2971393	10.625	9.47081985					1.6E+07	1.6E+07	0.28454	ID=promoter_01624;
1	2E+07	1_162623	16	24	1	6	20	4	0.0012	0.07347	21	1	35	6	11	21	0.13989775	1	13.75	12.3027291								
1	2E+07	1_163302	16	29	14	2	23	8	0.007066	0.30636	53	11	49	38	32	44	0.23264674	1	26.5	18.7235528								
1	2E+07	1_163768	174	47	125	156	111	141	0.820878	1	44	28	76	41	36	59	0.02403133	0.7884333	86.375	57.2436834								
1	2E+07	1_168191	12	14	14	16	13	15	0.999061	1	2	8	35	9	5	22	0.00242132	0.1280458	13.75	9.66215001								
1	2E+07	1_168528	130	98	224	115	114	170	0.138625	1	59	79	104	47	69	76	0.58352567	1	107	54.850446								
1	2E+07	1_169187	8	7	9	4	8	7	0.850897	1	6	24	7	11	15	9	0.58227053	1	9.5	6.21059003								
1	2E+07	1_169350	29	25	4	35	27	20	0.290795	1	11	19	18	36	15	27	0.11059826	1	22.125	11.2939617								
1	2E+07	1_170169	5	28	14	14	17	14	0.615566	1	21	12	40	7	17	24	0.40932813	1	17.625	11.648881								
1	2E+07	1_170730	88	29	56	64	59	60	0.738249	1	59	31	34	27	45	31	0.44260393	1	48.5	21.7977718								
1	2E+07	1_174039	104	712	181	162	408	172	1.00E-30	#######	702	467	683	368	585	526	0.8787024	1	422.38	257.006358								
1	2E+07	1_175607	22	34	10	23	28	17	0.092775	1	11	49	19	15	30	17	0.26150314	1	22.875	13.0431975								
1	2E+07	1_176286	1	10	8	4	6	6	0.962469	1	8	7	23	4	8	14	0.34554715	1	8.125	6.66413642								
1	2E+07	1_188786	16	20	21	37	18	29	0.568842	1	35	8	19	2	22	11	0.23631249	1	19.75	11.9492976								
1	2E+07	1_189859	129	67	17	19	98	18	9.99E-16	#######	34	29	9	3	32	6	3.53E-04	0.0265555	38.375	41.5518179								
1	2E+07	1_190594	133	86	114	51	110	83	0.009292	0.38389	45	38	27	23	42	25	0.20942954	1	64.625	41.4002674								
1	2E+07	1_191107	7	29	3	5	18	4	0.003432	0.17404	9	17	10	1	13	6	0.35360803	1	10.125	9.06228448								
1	2E+07	1_191264	37	56	54	40	47	47	0.741076	1	51	57	109	90	54	100	1.61E-04	0.0137078	61.75	24.9213047								
1	2E+07	1_191724	18	12	8	10	15	9	0.269309	1	29	12	24	24	21	24	0.78178162	1	17.125	7.77243297								
1	2E+07	1_194951	139	65	120	76	102	98	0.368535	1	0	11	16	5	6	11	0.39136385	1	54	54.5055698								
1	2E+07	1_196593	4	1	13	2	3	8	0.459247	1	1	4	16	0	3	8	0.26303803	1	5.125	6.01040764								
1	2E+07	1_199161	0	17	8	9	9	9	0.944235	1	0	1	6	0	1	3	0.56022579	1	5.125	6.10474055								
1	2E+07	1_204543	20	12	34	7	16	21	0.944185	1	85	30	10	0	58	5	1.44E-09	4.08E-07	24.75	26.9218446								
1	2E+07	1_206207	30	25	19	13	28	16	0.064038	1	14	19	34	1	17	18	0.92557631	1	19.375	10.4599577								
1	2E+07	1_20660754	2739	1156	2890	1998	1948	2444	0.085748	1	1106	1136	1638	387	1121	1013	0.84054526	1	1631.3	865.307006					2.1E+07	2.1E+07	0.45311	ID=promoter_01696;
1	2E+07	1_20712705	3	55	46	42	29	44	0.558719	1	57	21	13	1	39	7	4.70E-05	0.0048349	29.75	22.9704779	2E+07	2.1E+07	ID=ENSP00000356489;EPM2A;NM_005670;	EPM2A				
1	2E+07	1_213731	0	21	8	10	11	9	0.722819	1	27	20	15	3	24	9	0.0567576	1	13	9.38083152								
1	2E+07	1_215041	67	83	36	50	75	43	6.71E-04	0.04528	97	46	70	36	72	53	0.44245022	1	60.625	22.3347103								
1	2E+07	1_215584	13	13	4	9	13	7	0.234546	1	16	0	0	0	8	0	0.02452223	0.7977568	6.875	6.68553876								
1	2E+07	1_216721	30	30	19	26	30	23	0.302742	1	3	26	34	12	15	23	0.30916076	1	22.5	10.5017005								
1	2E+07	1_222731	0	15	15	8	8	12	0.864244	1	31	14	1	0	23	1	9.32E-05	0.0086863	10.5	10.6234243								
1	2E+07	1_22345490	11	0	3	0	6	2	0.258649	1	29	1	14	0	15	7	0.31064188	1	7.25	10.3337174	2E+07	2.2E+07	ID=ENSP00000325546-D3;PLAG1;NM_002655;	PLAG1	2.2E+07	2.2E+07	0.12529	ID=promoter_01710;
1	2E+07	1_225618	18	44	43	17	31	30	0.753666	1	9	61	16	22	35	19	0.16432451	1	28.75	18.2345825								
1	2E+07	1_234283	156	73	97	160	115	129	0.941198	1	12	24	17	3	18	10	0.42261869	1	67.75	64.333395								
1	2E+07	1_234535	43	0	28	23	22	26	0.999662	1	9	0	0	0	5	0	0.09850879	1	12.875	16.5480901								
1	2E+07	1_236904	3	87	24	25	45	25	0.00909	0.37919	53	15	54	24	34	39	0.66137313	1	35.625	27.1289908								
1	3E+07	1_255018	1	1	0	2	1	1	0.993645	1	18	8	20	14	13	17	0.64388579	1	8	8.26351707								
1	3E+07	1_271346	36	25	25	14	31	20	0.106722	1	31	26	42	2	29	22	0.77701989	1	25.125	12.5178444								
1	3E+07	1_273693	2	8	10	9	5	10	0.615109	1	3	22	28	20	13	24	0.12479476	1	12.75	9.45289071								
1	3E+07	1_275766	32	35	41	52	34	47	0.765861	1	67	28	64	35	48	50	0.84654018	1	44.25	14.9451378								
1	3E+07	1_27764166	4	28	10	19	16	15	0.822707	1	18	22	23	19	20	21	0.92353071	1	17.875	7.58640702	3E+07	2.8E+07	ID=ENSP00000265603;PERP;NM_022121;	PERP				
1	3E+07	1_28255035	3	6	7	146	5	77	3.76E-12	#######	0	1	60	22	1	41	1.11E-09	3.23E-07	30.625	50.6978092	3E+07	2.8E+07	ID=ENSP00000356708;OLIG3;NM_175747;	OLIG3	2.8E+07	2.8E+07	0.63034	ID=promoter_01753;
1	3E+07	1_284271	1354	618	1142	1063	986	1103	0.557232	1	722	627	569	148	675	359	7.59E-17	4.30E-14	780.38	384.999049								
1	3E+07	1_288211	19	56	26	15	38	21	0.018051	0.65084	16	15	41	15	16	28	0.11940283	1	25.375	15.277785								
1	3E+07	1_29497803	46	0	40	28	23	34	0.691592	1	1	0	0	0	1	0	0.62906424	1	14.375	20.1702575	3E+07	3E+07	ID=ENSP00000356761;PDE7B;;	PDE7B				

1	3E+07	1_303217	1	0	3	26	1	15	0.006136	0.2768	10	2	0	10	6	5	0.98461866	1	6.5	8.88015444								
1	3E+07	1_30517974	127	475	171	128	301	150	1.19E-17	#######	452	264	327	180	358	254	0.00476953	0.2198216	265.5	139.788616					3.1E+07	3.1E+07	0.29538	ID=promoter_01773;
1	3E+07	1_30597970	30	54	14	16	42	15	1.54E-04	0.01291	21	42	21	36	32	29	0.99609288	1	29.25	13.9667974	3E+07	3.1E+07	ID=ENSP0000356811;HBS1L;NM_006620;	HBS1L				
1	3E+07	1_310862	0	4	6	14	2	10	0.124792	1	8	10	10	38	9	24	0.01819934	0.631944	11.25	11.609725								
1	3E+07	1_312584	14	25	18	17	20	18	0.715835	1	51	19	25	15	35	20	0.21889545	1	23	12.0356613								
1	3E+07	1_31358092	1399	496	1106	724	948	915	1.40E-04	0.01195	441	433	339	160	437	250	6.25E-09	1.61E-06	637.25	418.230883					3.1E+07	3.1E+07	0.46887	ID=promoter_01779;
1	3E+07	1_314374	0	12	21	0	6	11	0.674578	1	21	0	3	0	11	2	0.06154634	1	7.125	9.47835881								
1	3E+07	1_314774	16	17	11	10	17	11	0.29617	1	23	19	13	3	21	8	0.08664698	1	14	6.164414								
1	3E+07	1_317398	4	11	1	1	8	1	0.03583	1	46	7	16	3	27	10	0.0371409	1	11.125	15.018441								
1	3E+07	1_320171	359	157	331	329	258	330	0.580776	1	221	146	157	87	184	122	0.01562259	0.558637	223.38	103.18906								
1	3E+07	1_322538	0	0	8	12	0	10	0.014084	0.53368	0	13	7	24	7	16	0.11989665	1	8	8.36660027								
1	3E+07	1_328822	81	69	97	132	75	115	0.194175	1	62	67	47	61	65	54	0.83736983	1	77	26.6886814								
1	3E+07	1_33231083	15	26	21	29	21	25	0.998746	1	68	59	40	1	64	21	8.94E-05	0.0083924	32.375	22.3730546	3E+07	3.3E+07	ID=ENSP0000354238;ENPP1;NM_006208;	ENPP1				
1	3E+07	1_335601	6	10	20	21	8	21	0.137587	1	30	14	36	36	22	36	0.10388249	1	21.625	11.4883481								
1	3E+07	1_335687	26	26	17	15	26	16	0.118485	1	35	18	26	28	27	27	0.96198634	1	23.875	6.70687281								
1	3E+07	1_342093	13	35	4	4	24	4	1.39E-04	0.01187	1	21	21	8	11	15	0.61974467	1	13.375	11.5997229								
1	4E+07	1_36633977	109	48	35	53	79	44	2.73E-04	0.02129	40	9	65	53	25	59	2.64E-04	0.0206659	51.5	28.575714	4E+07	3.7E+07	ID=ENSP0000357196;PTPRK;;	PTPRK				
1	4E+07	1_373452	160	153	183	203	157	193	0.909091	1	7	44	55	2	26	29	0.79045324	1	100.88	82.2147667								
1	4E+07	1_37658416	402	234	390	545	318	468	0.007487	0.32286	344	173	186	145	259	166	9.41E-04	0.0599013	302.38	140.369856	4E+07	3.8E+07	ID=ENSP0000357300;RSPO3;;	RSPO3	3.8E+07	3.8E+07	0.70448	ID=promoter_01808;
1	4E+07	1_387918	0	8	8	0	4	4	0.97482	1	40	19	0	13	30	7	0.00181176	0.1006964	11	13.5751874								
1	4E+07	1_38814261	3922	2619	3450	3615	3271	3533	0.00309	0.15972	2500	2520	2526	887	2510	1707	4.53E-22	3.44E-19	2754.9	946.053825					3.9E+07	3.9E+07	0.82455	ID=promoter_01816;
1	4E+07	1_389061	9	0	7	6	5	7	0.955252	1	27	0	0	5	14	3	0.04246461	1	6.75	8.90826262								
1	4E+07	1_391922	16	27	12	13	22	13	0.135741	1	66	26	44	18	46	31	0.38685872	1	27.75	18.6298532								
1	4E+07	1_39340038	5	30	0	27	18	14	0.509661	1	0	8	7	0	4	4	0.99427496	1	9.625	12.1059312	4E+07	3.9E+07	ID=ENSP0000376275;TPD52L1;;	TPD52L1				
1	4E+07	1_413421	29	30	9	7	30	8	2.50E-04	0.01962	23	5	14	21	14	18	0.65378371	1	17.25	9.86697232								
1	4E+07	1_422100	6	33	24	12	20	18	0.715835	1	68	63	13	27	66	20	2.40E-05	0.0026856	30.75	23.2117581								
1	4E+07	1_44524806	5107	5614	6757	11240	5361	8999	1.84E-95	#######	7090	5659	13947	3786	6375	8867	4.14E-135	2.36E-131	7400	3436.32029					4.5E+07	4.5E+07	0.59856	ID=promoter_01843;
1	4E+07	1_44760211	1534	824	1900	1612	1179	1756	1.66E-09	#######	915	721	788	204	818	496	5.41E-13	2.33E-10	1062.3	564.838978					4.5E+07	4.5E+07	0.58402	ID=promoter_01844;
1	4E+07	1_449761	0	7	4	7	4	6	0.929647	1	15	0	13	15	8	14	0.34554715	1	7.625	6.18610193								
1	5E+07	1_45394439	128	59	88	114	94	101	0.829152	1	46	40	26	0	43	13	9.59E-04	0.0604009	62.625	44.1132876					4.5E+07	4.5E+07	0.1334	ID=promoter_01849;
1	5E+07	1_461653	3	47	8	8	25	8	0.002813	0.14803	8	6	58	33	7	46	1.26E-07	2.58E-05	21.375	21.5269764								
1	5E+07	1_500090	30	35	47	37	33	42	0.940245	1	57	17	47	67	37	57	0.05200343	1	42.125	15.779168								
1	5E+07	1_509294	62	41	128	85	52	107	0.003432	0.17404	81	84	103	38	83	71	0.88358709	1	77.75	30.3397429								
1	5E+07	1_518785	7	7	0	2	7	1	0.062633	1	10	7	4	4	9	4	0.4571786	1	5.125	3.22656385								
1	5E+07	1_530020	34	2	16	39	18	28	0.644351	1	1	11	8	0	6	4	0.87665919	1	13.875	15.0374532								
1	5E+07	1_53071001	26	29	21	3	28	12	0.009759	0.39939	25	19	15	14	22	15	0.65424549	1	19	8.3323809	5E+07	5.3E+07	ID=ENSP0000354913;FAM135A;;	FAM135A				
1	5E+07	1_53193730	251	136	242	280	194	261	0.350614	1	77	69	108	42	73	75	0.82235972	1	150.63	93.4083776					5.3E+07	5.3E+07	0.62258	ID=promoter_01885;
1	5E+07	1_532954	10	38	0	10	24	5	3.79E-04	0.02808	40	25	11	34	33	23	0.5739607	1	21	15.2033831								
1	5E+07	1_53454198	102	64	107	106	83	107	0.811638	1	31	29	60	11	30	36	0.57812402	1	63.75	38.1641193					5.3E+07	5.3E+07	0.72204	ID=promoter_01887;

1	5E+07	1_54542839	38	21	46	29	30	38	0.95166	1	47	52	42	27	50	35	0.44143244	1	37.75	11.0032463	5E+07	5.5E+07	ID=ENSP0000264839;RIMS1;NM_014989;	RIMS1				
1	5E+07	1_54883568	8771	5430	9850	7110	7101	8480	0.53655	1	5820	3975	7829	2893	4898	5361	4.31E-16	2.31E-13	6459.8	2375.43065					5.5E+07	5.5E+07	0.80883	ID=promoter_01894;
1	6E+07	1_55307378	17	26	10	11	22	11	0.058934	1	1	0	0	0	1	0	0.62906424	1	8.125	9.70180396	6E+07	5.5E+07	ID=ENSP0000345055;KCNQ5;NM_001160133;	KCNQ5				
1	6E+07	1_557563	10	7	26	6	9	16	0.603365	1	0	0	0	0	0	0	1	1	6.125	8.95125689								
1	6E+07	1_56159477	43	10	36	40	27	38	0.7689	1	36	13	27	32	25	30	0.63340892	1	29.625	12.1999707					5.6E+07	5.6E+07	0.13076	ID=promoter_01909;
1	6E+07	1_56187681	2	14	1	14	8	8	0.950274	1	25	14	14	2	20	8	0.11762508	1	10.75	8.39642781	6E+07	5.6E+07	ID=ENSP0000358677;TBX18;NM_001080508;	TBX18				
1	6E+07	1_56208787	229	102	185	100	166	143	0.025002	0.84314	73	94	96	53	84	75	0.9724143	1	116.5	59.3801314	6E+07	5.6E+07	ID=ENSP0000358677;TBX18;NM_001080508;	TBX18	5.6E+07	5.6E+07	0.68414	ID=promoter_01910;
1	6E+07	1_56852581	9	7	5	3	8	4	0.357158	1	0	5	16	4	3	10	0.11508955	1	6.125	4.79396943	6E+07	5.7E+07	ID=ENSP0000257770;NT5E;NM_002526;	NT5E				
1	6E+07	1_56866335	10	7	10	8	9	9	0.944235	1	15	18	6	1	17	4	0.02867352	0.9010784	9.375	5.28981501	6E+07	5.7E+07	ID=ENSP0000257770;NT5E;NM_002526;	NT5E				
1	6E+07	1_578332	196	600	195	281	398	238	5.72E-16	#######	529	456	676	338	493	507	0.26011924	1	408.88	183.991023								
1	6E+07	1_586725	1	34	16	17	18	17	0.813792	1	12	30	32	15	21	24	0.78178162	1	19.625	11.4260042								
1	6E+07	1_596196	7	7	8	3	7	6	0.851931	1	0	25	34	1	13	18	0.54030568	1	10.625	12.2467197								
1	6E+07	1_597660	3	35	24	9	19	17	0.718296	1	31	9	27	13	20	20	0.97170071	1	18.875	11.8374406								
1	6E+07	1_606272	120	47	144	205	84	175	6.61E-05	0.0062	23	11	8	6	17	7	0.1782776	1	70.5	75.9304193								
1	6E+07	1_608902	0	20	15	4	10	10	0.938234	1	28	0	0	2	14	1	0.00574567	0.2548307	8.625	10.91444								
1	6E+07	1_60941319	2	8	4	9	5	7	0.955252	1	27	39	35	0	33	18	0.18694358	1	15.5	15.6661601	6E+07	6.1E+07	ID=ENSP0000237177;CASP8AP2;;	CASP8AP2				
1	6E+07	1_611864	10	23	11	4	17	8	0.087954	1	0	17	33	23	9	28	0.00358036	0.1770746	15.125	10.9731816								
1	6E+07	1_620892	70	56	67	79	63	73	0.997486	1	28	44	103	2	36	53	0.09687545	1	56.125	31.3935275								
1	6E+07	1_637151	42	102	30	31	72	31	7.32E-06	0.00091	120	84	53	83	102	68	0.10668992	1	68.125	33.9219166								
1	6E+07	1_638414	8	0	1	2	4	2	0.597627	1	0	0	6	32	0	19	3.54E-05	0.003758	6.125	10.8816687								
1	7E+07	1_658092	14	4	6	11	9	9	0.944235	1	23	16	19	43	20	31	0.1936646	1	17	12.2591074								
1	7E+07	1_683724	3	73	28	27	38	28	0.169685	1	6	46	13	20	26	17	0.53107524	1	27	23.1146212								
1	7E+07	1_687660	12	50	7	5	31	6	2.44E-05	0.00261	30	22	26	21	26	24	0.99988526	1	21.625	14.5301411								
1	7E+07	1_689117	43	80	52	72	62	62	0.673487	1	100	51	42	23	76	33	9.67E-04	0.0606639	57.875	24.5963848								
1	7E+07	1_691677	56	66	37	42	61	40	0.014978	0.56219	81	71	98	47	76	73	0.97683313	1	62.25	20.837809								
1	7E+07	1_69267177	11	13	16	5	12	11	0.839207	1	18	29	14	28	24	21	0.98147478	1	16.75	8.20713975	7E+07	6.9E+07	ID=ENSP0000358247;FBXL4;NM_012160;	FBXL4				
1	7E+07	1_706042	10	27	12	2	19	7	0.021688	0.74727	14	16	16	7	15	12	0.93002578	1	13	7.3872477								
1	7E+07	1_70629959	41	37	24	40	39	32	0.321643	1	44	9	29	24	27	27	0.96198634	1	31	11.7837661					7.1E+07	7.1E+07	0.27914	ID=promoter_02005;
1	7E+07	1_70630268	15	1	7	7	8	7	0.850897	1	32	13	7	16	23	12	0.26207533	1	12.25	9.42261717					7.1E+07	7.1E+07	0.27914	ID=promoter_02005;
1	7E+07	1_714338	567	527	599	634	547	617	0.801137	1	1502	1098	1812	371	1300	1092	0.05573428	1	888.75	524.207633								
1	7E+07	1_71829635	1	7	9	9	4	9	0.54411	1	0	24	12	19	12	16	0.63262749	1	10.125	8.21909797	7E+07	7.2E+07	ID=ENSP0000362185-D2;GRIK3;NM_000831;	GRIK3				
1	7E+07	1_721791	12	25	5	13	19	9	0.06806	1	13	16	20	23	15	22	0.38478854	1	15.875	6.55607679								
1	7E+07	1_731562	17	12	15	13	15	14	0.827043	1	28	10	40	54	19	47	8.61E-04	0.0561096	23.625	15.8649434								

1	7E+07	1_734945	59	132	2012	2570	96	2291	0	0	143	110	3466	571	127	2019	0	0	1132.9	1351.10795								
1	7E+07	1_746336	245	235	273	215	240	244	0.289639	1	169	153	73	114	161	94	0.00156887	0.0893884	184.63	69.2901508								
1	8E+07	1_757165	1527	1206	1458	1411	1367	1435	0.013357	0.51173	221	236	298	70	229	184	0.35155452	1	803.38	647.831534								
1	8E+07	1_757350	666	160	486	400	413	443	0.425385	1	157	156	200	45	157	123	0.37563307	1	283.75	211.203862								
1	8E+07	1_759724	33	28	24	20	31	22	0.19035	1	80	12	75	5	46	40	0.95710435	1	34.625	27.9076922								
1	8E+07	1_76197396	16	6	19	10	11	15	0.930409	1	2	23	13	6	13	10	0.90601506	1	11.875	7.1999504	8E+07	7.6E+07	ID=ENSP00000358059;RTN4IP1;NM_032730;	RTN4IP1				
1	8E+07	1_766824	51	65	51	59	58	55	0.526975	1	20	45	21	13	33	17	0.1362937	1	40.625	19.7841026								
1	8E+07	1_767400	0	20	0	4	10	2	0.03464	1	37	15	13	0	26	7	0.00838865	0.3419228	11.125	13.0322402								
1	8E+07	1_77096368	0	10	8	29	5	19	0.046679	1	11	16	3	2	14	3	0.04246461	1	9.875	9.3722615					7.7E+07	7.7E+07	0.29509	ID=promoter_02051;
1	8E+07	1_77195709	113	79	95	69	96	82	0.106148	1	65	82	92	29	74	61	0.79386907	1	78	25.00857	8E+07	7.7E+07	ID=ENSP00000318900;SOBP;NM_018013;	SOBP				
1	8E+07	1_77288031	8	14	0	6	11	3	0.050503	1	141	15	60	35	78	48	0.07969257	1	34.875	47.0514536	8E+07	7.7E+07	ID=ENSP00000318900;SOBP;NM_018013;	SOBP	7.7E+07	7.7E+07	0.56333	ID=promoter_02053;
1	8E+07	1_78171050	51	38	44	45	45	45	0.750586	1	0	13	16	2	7	9	0.80856893	1	26.125	20.615095	8E+07	7.8E+07	ID=ENSP00000357973;LACE1;NM_145315;	LACE1				
1	8E+07	1_781965	4	19	6	12	12	9	0.592799	1	0	5	5	20	3	13	0.02959638	0.9232309	8.875	7.33752586								
1	8E+07	1_782043	4	29	20	18	17	19	0.989587	1	29	12	10	3	21	7	0.05007015	1	15.625	10.155048								
1	8E+07	1_782119	54	104	25	38	79	32	7.28E-07	0.00011	105	63	36	19	84	28	6.29E-06	0.0008449	55.5	33.4023096								
1	8E+07	1_78220721	2059	1376	2605	1636	1718	2121	0.2881	1	1014	1057	1037	472	1036	755	3.03E-06	0.000446	1407	676.311847					7.8E+07	7.8E+07	0.92456	ID=promoter_02066;
1	8E+07	1_782412	9	25	26	8	17	17	0.897282	1	32	1	22	20	17	21	0.67722313	1	17.875	10.6829303								
1	8E+07	1_783260	0	34	7	2	17	5	0.013234	0.50759	23	36	24	0	30	12	0.0403413	1	15.75	15.2385789								
1	8E+07	1_783477	0	24	5	0	12	3	0.030625	0.98374	23	1	5	2	12	4	0.18067673	1	7.5	10.0711753								
1	8E+07	1_786228	30	2	46	40	16	43	0.014369	0.54261	0	10	11	1	5	6	0.91271846	1	17.5	18.4855156								
1	8E+07	1_800305	7	37	7	28	22	18	0.522468	1	26	16	20	0	21	10	0.21021716	1	17.625	12.5235492								
1	8E+07	1_800471	60	27	16	31	44	24	0.008407	0.35544	17	17	17	23	17	20	0.769441	1	26	14.8034745								
1	8E+07	1_803373	55	26	46	76	41	61	0.49837	1	29	43	74	24	36	49	0.21237046	1	46.625	20.5074028								
1	8E+07	1_80531017	14	19	17	8	17	13	0.50439	1	17	11	10	6	14	8	0.54462139	1	12.75	4.71320334					8.1E+07	8.1E+07	0.67147	ID=promoter_02094;
1	8E+07	1_80615779	83	53	59	30	68	45	0.010481	0.4265	18	15	10	7	17	9	0.38755874	1	34.375	27.5781357	8E+07	8.1E+07	ID=ENSP00000357962;;					
1	8E+07	1_807179	12	30	8	5	21	7	0.0088	0.36783	13	12	5	0	13	3	0.06317581	1	10.625	9.00694177								
1	8E+07	1_810949	11	2	3	5	7	4	0.503048	1	1	4	9	19	3	14	0.01844097	0.6375434	6.75	6.01783065								
1	8E+07	1_811860	18	10	10	8	14	9	0.360318	1	25	2	17	6	14	12	0.98032967	1	12	7.46420027								
1	8E+07	1_812389	0	0	0	7	0	4	0.181755	1	33	0	16	1	17	9	0.38755874	1	7.125	11.8856156								
1	8E+07	1_813554	98	54	73	88	76	81	0.835185	1	68	17	24	4	43	14	0.00178346	0.0995187	53.25	34.6688415								
1	8E+07	1_816325	140	35	25	24	88	25	9.47E-11	#######	34	33	37	0	34	19	0.20290377	1	41	41.7338506								
1	8E+07	1_825774	32	72	4	0	52	2	1.17E-12	#######	20	30	5	0	25	3	3.93E-04	0.0289246	20.375	24.5760482								
1	8E+07	1_832487	3	4	6	0	4	3	0.840045	1	12	26	14	0	19	7	0.09662636	1	8.125	8.85498246								
1	8E+07	1_83525411	8331	7579	8947	10282	7955	9615	0.141011	1	6354	5582	14000	2582	5968	8291	1.02E-125	4.70E-122	7957.1	3386.67964					8.4E+07	8.4E+07	0.80256	ID=promoter_02113;
1	8E+07	1_83980485	46	59	49	58	53	54	0.766673	1	92	70	72	43	81	58	0.32195054	1	61.125	16.3395882					8.4E+07	8.4E+07	0.53529	ID=promoter_02116;
1	8E+07	1_842948	1	12	0	1	7	1	0.062633	1	25	15	3	2	20	3	0.00346999	0.1723921	7.375	9.05439909								
1	8E+07	1_845052	22	55	17	26	39	22	0.019446	0.69119	41	22	56	22	32	39	0.51586212	1	32.625	15.7836941								
1	9E+07	1_86628182	324	227	253	249	276	251	0.013853	0.52965	68	116	61	70	92	66	0.28084425	1	171	103.708382					8.7E+07	8.7E+07	0.39086	ID=promoter_02131;
1	9E+07	1_868031	21	42	10	25	32	18	0.038646	1	36	22	39	82	29	61	9.35E-04	0.0596362	34.625	21.9215321								
1	9E+07	1_873795	9	23	13	18	16	16	0.903021	1	16	6	2	2	11	2	0.06154634	1	11.125	7.67998326								
1	9E+07	1_885351	13	25	3	0	19	2	2.36E-04	0.01868	20	31	13	12	26	13	0.18179217	1	14.625	10.4599577								
1	9E+07	1_899148	3	7	7	20	5	14	0.226542	1	38	21	43	17	30	30	0.95785282	1	19.5	14.5798295								
1	9E+07	1_905298	43	11	22	0	27	11	0.00829	0.35136	2	13	4	14	8	9	0.92351511	1	13.625	13.9072181								
1	1E+08	1_953221	3	23	2	0	13	1	0.002067	0.11504	1	1	1	27	1	14	0.00214405	0.1161796	7.25	11.0421271								
1	1E+08	1_955811	4	30	13	6	17	10	0.210142	1	13	30	38	15	22	27	0.61865829	1	18.625	12.4434435								

1	1E+08	1_95859727	570	253	433	370	412	402	0.031502	1	341	172	190	67	257	129	5.72E-08	1.24E-05	299.5	161.311588					9.6E+07	9.6E+07	0.64957	ID=promoter_02170;
1	1E+08	1_989133	41	24	49	19	33	34	0.868497	1	66	47	24	12	57	18	1.62E-04	0.0137848	35.25	18.3750607								
1	1E+08	1_995941	7	14	1	4	11	3	0.050503	1	0	1	11	2	1	7	0.08320466	1	5	5.18238776								
1	1E+08	1_99744928	336	155	202	151	246	177	3.58E-06	0.00048	145	104	64	63	125	64	4.62E-04	0.0331819	152.5	88.1411206					1E+08	1E+08	0.18559	ID=promoter_02197;
1	1E+08	1_998953	18	0	10	2	9	6	0.554309	1	15	0	4	0	8	2	0.20550608	1	6.125	7.25923058								
1	1E+08	1_100154072	44	89	34	13	67	24	8.67E-07	0.00013	81	35	31	8	58	20	3.76E-04	0.027889	41.875	29.1911802								
1	1E+08	1_100155385	35	49	44	25	42	35	0.324205	1	20	35	39	1	28	20	0.67114862	1	31	15.3343685								
1	1E+08	1_100504833	657	182	477	389	420	433	0.16905	1	145	187	123	44	166	84	2.65E-05	0.0029309	275.5	210.265139					1E+08	1E+08	0.68996	ID=promoter_02206;
1	1E+08	1_100520344	8	11	8	10	10	9	0.84607	1	1	1	0	1	1	1	0.99856566	1	5	4.65985898								
1	1E+08	1_100997060	0	5	2	8	3	5	0.88968	1	20	2	7	1	11	4	0.25131486	1	5.625	6.47936284								
1	1E+08	1_101262981	19	1	6	14	10	10	0.938234	1	1	9	1	0	5	1	0.30486099	1	6.375	7.08998287								
1	1E+08	1_101416019	12	3	30	26	8	28	0.016511	0.60652	1	10	23	0	6	12	0.2888538	1	13.125	11.8374406	1E+08	1E+08	ID=ENSP00000372459;KIAA0427;NM_001142397;	KIAA0427				
1	1E+08	1_101493284	18	14	10	12	16	11	0.385778	1	7	10	3	1	9	2	0.1390433	1	9.375	5.60452623	1E+08	1E+08	ID=ENSP00000372459;KIAA0427;NM_001142397;	KIAA0427				
1	1E+08	1_101666928	152	38	121	70	95	96	0.587781	1	24	61	34	15	43	25	0.17340376	1	64.375	48.7176633								
1	1E+08	1_101801770	2	2	7	15	2	11	0.085797	1	30	9	52	1	20	27	0.43992527	1	14.75	17.8385618								
1	1E+08	1_101834516	143	76	59	49	110	54	4.43E-07	#######	1	30	39	1	16	20	0.67029873	1	49.75	45.8717155								
1	1E+08	1_102082043	242	100	170	214	171	192	0.917273	1	83	27	66	27	55	47	0.91936513	1	116.13	82.8793228								
1	1E+08	1_102260834	19	92	42	52	56	47	0.235754	1	33	55	47	77	44	62	0.10136647	1	52.125	23.3264871								
1	1E+08	1_102321330	9	14	21	6	12	14	0.999907	1	31	11	21	8	21	15	0.7415049	1	15.125	8.54295867								
1	1E+08	1_103048341	31	11	68	101	21	85	4.32E-07	#######	0	15	5	6	8	6	0.92562801	1	29.625	36.1976223								
1	1E+08	1_103535376	15	4	8	19	10	14	0.912507	1	4	0	12	1	2	7	0.20455692	1	7.875	6.87516234								
1	1E+08	1_103878051	19	0	7	11	10	9	0.84607	1	27	18	15	4	23	10	0.12305918	1	12.625	8.86304527					1E+08	1E+08	0.34508	ID=promoter_02248;
1	1E+08	1_104333485	42	75	50	53	59	52	0.320882	1	65	69	106	37	67	72	0.68428358	1	62.125	22.0806153								
1	1E+08	1_104889209	8	9	14	15	9	15	0.704209	1	14	0	29	12	7	21	0.01731975	0.610307	12.625	8.21040281								
1	1E+08	1_106150850	200	123	172	138	162	155	0.192238	1	264	290	523	243	277	383	2.22E-06	0.0003405	244.13	127.253894								
1	1E+08	1_106488545	43	116	56	91	80	74	0.3365	1	88	90	153	38	89	96	0.5881409	1	84.375	38.6668463								
1	1E+08	1_107304428	14	67	14	14	41	14	1.22E-04	0.01067	43	25	34	39	34	37	0.79599102	1	31.25	18.5760983								
1	1E+08	1_107376259	19	22	17	10	21	14	0.252399	1	4	47	53	17	26	35	0.3499771	1	23.625	17.2869687								
1	1E+08	1_108303306	6	5	7	2	6	5	0.851367	1	41	9	6	13	25	10	0.06888504	1	11.125	12.4835606								
1	1E+08	1_108438146	8	14	99	82	11	91	8.51E-12	#######	0	4	14	0	2	7	0.20455692	1	27.625	39.4459395					1.1E+08	1.1E+08	0.10007	ID=promoter_02268;
1	1E+08	1_108627482	8	3	0	2	6	1	0.108618	1	22	0	4	1	11	3	0.13591373	1	5	7.34846923								
1	1E+08	1_108660407	0	2	7	5	1	6	0.241978	1	11	7	9	2	9	6	0.8208167	1	5.375	3.81491434								
1	1E+08	1_108789308	0	7	5	7	4	6	0.929647	1	0	24	0	0	12	0	0.00384008	0.1844256	5.375	8.17552969								

1	1E+08	1_109114426	4	1	5	3	3	4	0.986002	1	8	14	14	3	11	9	0.96206785	1	6.5	5.04267503								
1	1E+08	1_109204035	281	115	188	186	198	187	0.103668	1	26	69	49	28	48	39	0.82910971	1	117.75	92.4488275	1E+08	1.1E+08	ID=ENSP000002175 15;TXNL1;NM_004786;	TXNL1	1.1E+08	1.1E+08	0.3897	ID=promoter_02274;
1	1E+08	1_109373679	9	14	26	16	12	21	0.538277	1	0	0	11	3	0	7	0.02292696	0.7568963	9.875	8.93528319								
1	1E+08	1_110843616	253	179	294	225	216	260	0.961263	1	167	128	124	51	148	88	0.00395557	0.1882624	177.63	78.3361575					1.1E+08	1.1E+08	0.47785	ID=promoter_02302;
1	1E+08	1_110907375	1634	623	1182	985	1129	1084	1.48E-05	0.00168	603	539	559	213	571	386	9.35E-06	0.001193	792.25	449.783043					1.1E+08	1.1E+08	0.52423	ID=promoter_02303;
1	1E+08	1_111034867	10	7	3	2	9	3	0.131542	1	19	0	8	7	10	8	0.95278883	1	7	5.90399381								
1	1E+08	1_111275827	1	17	3	9	9	6	0.554309	1	13	1	5	15	7	10	0.67636194	1	8	6.41426981								
1	1E+08	1_112255079	423	224	450	374	324	412	0.553384	1	223	162	271	97	193	184	0.96374602	1	278	126.509175								
1	1E+08	1_112689962	10	14	2	7	12	5	0.126784	1	0	2	1	4	1	3	0.56022579	1	5	4.9280538								
1	1E+08	1_113052688	14	7	4	17	11	11	0.932271	1	15	7	21	30	11	26	0.02624811	0.8458091	14.375	8.55131569								
1	1E+08	1_113249326	1910	2065	2877	3665	1988	3271	8.92E-32	#######	2423	1573	1673	961	1998	1317	5.73E-21	4.05E-18	2143.4	839.80745					1.1E+08	1.1E+08	0.67295	ID=promoter_02330;
1	1E+08	1_113463292	75	57	103	112	66	108	0.101094	1	12	6	27	2	9	15	0.36943449	1	49.25	43.9732061								
1	1E+08	1_114804586	136	51	105	92	94	99	0.755847	1	0	0	22	1	0	12	0.00154584	0.0883233	50.875	53.9376293								
1	1E+08	1_115181107	202	83	144	109	143	127	0.072845	1	27	4	18	0	16	9	0.48000706	1	73.375	73.9767097	1E+08	1.2E+08	ID=ENSP000002615 20;NARG2;NM_024611;	NARG2				
1	1E+08	1_115222366	1011	659	1109	764	835	937	0.648684	1	459	379	346	301	419	324	0.04911804	1	628.5	310.419072					1.2E+08	1.2E+08	0.48235	ID=promoter_02340;
1	1E+08	1_115299155	24	38	38	24	31	31	0.820663	1	8	44	85	69	26	77	4.33E-07	7.88E-05	41.25	25.1097591								
1	1E+08	1_115559795	29	79	26	22	54	24	2.12E-04	0.01705	0	28	49	48	14	49	1.50E-05	0.0018043	35.125	23.4851777								
1	1E+08	1_116182128	123	218	105	104	171	105	7.46E-07	0.00012	241	144	101	130	193	116	9.98E-04	0.062444	145.75	54.0733629					1.2E+08	1.2E+08	0.35171	ID=promoter_02350;
1	1E+08	1_116675454	0	21	1	1	11	1	0.006522	0.28949	10	0	10	0	5	5	0.99284883	1	5.375	7.670677	1E+08	1.2E+08	ID=ENSP000002882 35;MYO1E;NM_004998;	MYO1E				
1	1E+08	1_116943415	150	179	112	88	165	100	7.72E-07	0.00012	143	74	86	87	109	87	0.5809817	1	114.88	38.0767552					1.2E+08	1.2E+08	0.43232	ID=promoter_02356;
1	1E+08	1_117403607	150	15	178	105	83	142	0.022911	0.7858	9	21	28	23	15	26	0.14599233	1	66.125	67.9168346								
1	1E+08	1_117509927	0	2	5	1	1	3	0.700657	1	0	10	22	7	5	15	0.05518429	1	5.875	7.43423548								
1	1E+08	1_118229668	14	1	4	6	8	5	0.532535	1	19	0	3	1	10	2	0.09294023	1	6	6.88684045								
1	1E+08	1_118783832	56	96	30	41	76	36	2.15E-05	0.00234	120	67	86	92	94	89	0.98987841	1	73.5	30.3879675					1.2E+08	1.2E+08	0.46123	ID=promoter_02367;
1	1E+08	1_119401118	228	55	218	175	142	197	0.311866	1	59	57	77	26	58	52	0.98478119	1	111.88	81.3852172					1.2E+08	1.2E+08	0.73812	ID=promoter_02368;
1	1E+08	1_121558854	57	20	59	45	39	52	0.83255	1	56	27	7	0	42	4	6.10E-07	0.0001066	33.875	23.5701839								
1	1E+08	1_123653124	443	259	292	251	351	272	1.59E-06	0.00023	44	42	88	0	43	44	0.89932795	1	177.38	156.453496					1.2E+08	1.2E+08	0.2142	ID=promoter_02395;
1	1E+08	1_124576589	18	27	39	22	23	31	0.880117	1	24	18	12	10	21	11	0.29862283	1	21.25	9.17683419								
1	1E+08	1_124623844	19	5	21	32	12	27	0.161087	1	23	12	44	22	18	33	0.0599435	1	22.25	11.8532696					1.2E+08	1.2E+08	0.64898	ID=promoter_02408;

1	1E+08	1_125110728	7	27	23	14	17	19	0.989587	1	2	26	15	0	14	8	0.54462139	1	14.25	10.57963								
1	1E+08	1_125153571	88	23	95	43	56	69	0.963227	1	36	16	6	25	26	16	0.43033428	1	41.5	32.9111358					1.3E+08	1.3E+08	0.46414	ID=promoter_02414;
1	1E+08	1_125181430	16	61	25	17	39	21	0.012903	0.49818	26	6	54	16	16	35	0.01355156	0.5007583	27.625	19.5297392	1E+08	1.3E+08	ID=ENSP0000251076;DMXL2;NM_015263;	DMXL2				
1	1E+08	1_125236126	137	72	77	76	105	77	0.006802	0.29841	132	77	19	22	105	21	1.47E-11	5.38E-09	76.5	43.1575188	1E+08	1.3E+08	ID=ENSP0000251076;DMXL2;NM_015263;	DMXL2	1.3E+08	1.3E+08	0.46199	ID=promoter_02415;
1	1E+08	1_125269384	7	16	9	2	12	6	0.213448	1	2	11	16	2	7	9	0.80856893	1	8.125	5.9386747	1E+08	1.3E+08	ID=ENSP0000251076;DMXL2;NM_015263;	DMXL2				
1	1E+08	1_125512703	112	15	94	108	64	101	0.176018	1	12	7	1	0	10	1	0.03495212	1	43.625	51.043224								
1	1E+08	1_125607601	1	17	2	11	9	7	0.713906	1	1	4	2	20	3	11	0.0740992	1	7.25	7.70435869								
1	1E+08	1_125637369	2	13	4	6	8	5	0.532535	1	0	10	4	15	5	10	0.35527535	1	6.75	5.36523199								
1	1E+08	1_126330453	318	130	335	352	224	344	0.007045	0.30636	83	91	88	21	87	55	0.08212693	1	177.25	134.253226					1.3E+08	1.3E+08	0.29081	ID=promoter_02425;
1	1E+08	1_126474601	8577	8510	9486	11038	8544	####	0.270177	1	8959	7222	13928	4742	8091	9335	5.94E-46	1.06E-42	9057.8	2682.23525					1.3E+08	1.3E+08	0.30334	ID=promoter_02427;
1	1E+08	1_126620935	210	70	123	172	140	148	0.677671	1	27	53	38	25	40	32	0.82411274	1	89.75	70.5888498	1E+08	1.3E+08	ID=ENSP0000267842;SLC27A2;NM_003645;	SLC27A2	1.3E+08	1.3E+08	0.37056	ID=promoter_02429;
1	1E+08	1_126994392	32	33	41	25	33	33	0.810265	1	71	15	77	17	43	47	0.73693019	1	38.875	23.3387324								
1	1E+08	1_127995212	20	72	28	7	46	18	1.82E-04	0.01496	69	46	55	51	58	53	0.99713413	1	43.5	23.2440469	1E+08	1.3E+08	ID=ENSP0000370314;SECISBP2L;;	SECISBP2L				
1	1E+08	1_128480426	42	36	43	30	39	37	0.651187	1	17	23	31	0	20	16	0.90780655	1	27.75	14.2803561								
1	1E+08	1_128501704	3631	2676	3739	3146	3154	3443	0.014248	0.53861	2475	1983	2149	1056	2229	1603	7.50E-14	3.53E-11	2606.9	898.208286					1.3E+08	1.3E+08	0.54791	ID=promoter_02440;
1	1E+08	1_129019698	21	35	37	69	28	53	0.117855	1	29	0	0	0	15	0	9.56E-04	0.060345	23.875	24.133187								
1	1E+08	1_129345704	326	282	348	373	304	361	0.987544	1	271	105	260	174	188	217	0.08804427	1	267.38	89.8092224					1.3E+08	1.3E+08	0.47697	ID=promoter_02443;
1	1E+08	1_129435596	0	20	9	1	10	5	0.276106	1	1	12	0	29	7	15	0.16935974	1	9	10.8232554								
1	1E+08	1_129506803	16	39	12	7	28	10	0.00287	0.14985	1	12	15	14	7	15	0.16935974	1	14.5	11.045361								
1	1E+08	1_130851252	155	261	116	150	208	133	2.06E-07	#######	205	192	262	115	199	189	0.97202063	1	182	58.3927588								
1	1E+08	1_133240617	0	19	1	3	10	2	0.03464	1	0	11	10	1	6	6	0.99142475	1	5.625	6.96803928								
1	1E+08	1_133444338	0	0	3	6	0	5	0.118674	1	0	15	16	1	8	9	0.92351511	1	5.125	6.7281392								
1	1E+08	1_133827088	25	17	68	51	21	60	0.001384	0.0827	8	46	13	4	27	9	0.02128286	0.7187388	29	23.2256016	1E+08	1.3E+08	ID=ENSP0000267812;MFAP1;NM_005926;	MFAP1				
1	1E+08	1_133993072	17	2	10	10	10	10	0.938234	1	13	0	18	71	7	45	2.09E-07	4.08E-05	17.625	22.4940468								
1	1E+08	1_134449718	39	32	37	75	36	56	0.415189	1	49	36	35	2	43	19	0.02267705	0.755811	38.125	20.1312657								
1	1E+08	1_135703511	24	24	7	6	24	7	0.00213	0.11798	69	44	48	33	57	41	0.46221979	1	31.875	21.3771407								

1	1E+08	1_135774682	753	358	665	603	556	634	0.888549	1	228	165	185	37	197	111	1.25E-04	0.0110719	374.25	266.003625	1E+08	1.4E+08	ID=ENSP0000220325;EHD4;NM_139265;	EHD4				
1	1E+08	1_135803379	16	15	12	1	16	7	0.076817	1	19	15	27	0	17	14	0.9475482	1	13.125	8.96720214								
1	1E+08	1_136662790	0	18	14	0	9	7	0.713906	1	1	0	7	0	1	4	0.36109265	1	5	7.2702918								
1	1E+08	1_136806780	814	336	564	556	575	560	0.007303	0.31581	192	227	274	112	210	193	0.99623341	1	384.38	238.082781					1.4E+08	1.4E+08	0.35896	ID=promoter_02530;
1	1E+08	1_136820822	232	165	242	189	199	216	0.732257	1	104	116	106	66	110	86	0.49547429	1	152.5	64.5290632					1.4E+08	1.4E+08	0.72424	ID=promoter_02531;
1	1E+08	1_136927595	46	164	13	31	105	22	1.21E-15	#######	89	70	82	39	80	61	0.51539745	1	66.75	47.1524882								
1	1E+08	1_137141277	18	14	11	0	16	6	0.042495	1	1	4	11	3	3	7	0.3829009	1	7.75	6.62786326								
1	1E+08	1_137217999	2566	1221	2734	2276	1894	2505	4.16E-04	0.03032	1457	970	992	542	1214	767	6.44E-16	3.44E-13	1594.8	821.660818					1.4E+08	1.4E+08	0.3563	ID=promoter_02544;
1	1E+08	1_137470161	11	24	3	1	18	2	4.15E-04	0.03028	0	9	6	6	5	6	0.91271846	1	7.5	7.65319728								
1	1E+08	1_137654017	15	24	15	28	20	22	0.978625	1	170	88	60	20	129	40	1.66E-09	4.67E-07	52.5	53.989417					1.4E+08	1.4E+08	0.27763	ID=promoter_02561;
1	1E+08	1_138788499	11	12	2	1	12	2	0.011798	0.46127	5	5	22	32	5	27	2.18E-04	0.0177562	11.25	10.7935166								
1	1E+08	1_139744009	758	297	606	514	528	560	0.251915	1	511	213	279	176	362	228	2.54E-05	0.0028245	419.25	208.171461					1.4E+08	1.4E+08	0.54759	ID=promoter_02573;
1	1E+08	1_139808483	7	1	8	19	4	14	0.128496	1	38	7	4	0	23	2	3.18E-04	0.0243067	10.5	12.5470543								
1	1E+08	1_143943380	2273	1061	2106	1680	1667	1893	0.628161	1	1031	852	859	597	942	728	0.00109598	0.0677139	1307.4	627.831401					1.4E+08	1.4E+08	0.44359	ID=promoter_02598;
1	1E+08	1_144293331	8	33	14	12	21	13	0.183347	1	0	10	18	2	5	10	0.35527535	1	12.125	10.3155569								
1	1E+08	1_144323484	43	20	70	35	32	53	0.301348	1	31	10	46	15	21	31	0.25173633	1	33.75	19.5064092								
1	1E+08	1_144649625	0	20	0	0	10	0	0.002838	0.14869	16	13	37	1	15	19	0.66253156	1	10.875	13.3570474								
1	1E+08	1_144773023	510	170	353	260	340	307	0.003877	0.19153	175	106	111	57	141	84	0.00534938	0.2427073	217.75	150.863941					1.4E+08	1.4E+08	0.19599	ID=promoter_02603;
1	1E+08	1_145053352	7	26	5	10	17	8	0.087954	1	0	43	28	39	22	34	0.16818144	1	19.75	16.4033969	1E+08	1.5E+08	ID=ENSP0000303830-D4;INSR;NM_000208;	INSR				
1	1E+08	1_145293025	3	7	29	32	5	31	5.64E-04	0.03913	0	1	3	0	1	2	0.81304206	1	9.375	13.2550529								
1	1E+08	1_145562864	0	4	1	0	2	1	0.773064	1	9	17	18	0	13	9	0.79589064	1	6.125	7.66135945								
1	1E+08	1_145871685	70	121	79	69	96	74	0.023831	0.81152	186	91	152	77	139	115	0.66507565	1	105.63	43.4246063								
1	1E+08	1_145970125	14	34	10	7	24	9	0.00876	0.36763	0	26	3	2	13	3	0.06317581	1	12	12.1772622								
1	1E+08	1_146930407	0	12	9	5	6	7	0.999953	1	19	11	1	1	15	1	0.00364468	0.1775497	7.25	6.69221296								
1	1E+08	1_147036673	28	85	21	17	57	19	2.63E-06	0.00037	18	34	17	2	26	10	0.05079892	1	27.75	24.9327667								
1	1E+08	1_147444040	2	2	0	2	2	1	0.773064	1	36	0	14	0	18	7	0.13207736	1	7	12.6038543								
1	1E+08	1_147466119	17	50	18	4	34	11	3.70E-04	0.02765	93	39	60	1	66	31	0.00630672	0.2732901	35.25	31.5311637								
1	1E+08	1_147929976	2	39	1	4	21	3	2.46E-04	0.01935	1	21	28	1	11	15	0.61974467	1	12.125	15.0848789								
1	1E+08	1_148365714	1	28	2918	3071	15	2995	0	0	2	18	1917	717	10	1317	1.35E-302	1.75E-298	1084	1348.8306	1E+08	1.5E+08	ID=ENSP0000382953;;;					
1	1E+08	1_148537500	13	46	5	9	30	7	1.06E-04	0.00938	2	18	14	26	10	20	0.12622058	1	16.625	14.0401211								
1	1E+08	1_149178568	4	32	6	2	18	4	0.003432	0.17404	21	27	31	3	24	17	0.69596948	1	15.75	13.2853303								

1	2E+08	1_150331677	101	85	69	73	93	71	0.023051	0.78947	88	87	93	58	88	76	0.90267448	1	81.75	14.0280332				
1	2E+08	1_150727720	55	57	15	31	56	23	5.37E-05	0.00515	57	44	76	18	51	47	0.99957097	1	44.125	21.2766639				
1	2E+08	1_150959413	3	15	31	35	9	33	0.006022	0.27245	0	3	7	2	2	5	0.46647814	1	12	13.7840488	1.5E+08	1.5E+08	0.83725	ID=promoter_02654;
1	2E+08	1_151832977	25	8	39	2	17	21	0.98748	1	23	26	16	0	25	8	0.02318551	0.7636665	17.375	13.4157424	1.5E+08	1.5E+08	0.43824	ID=promoter_02669;
1	2E+08	1_151869933	167	93	143	137	130	140	0.781878	1	38	45	50	39	42	45	0.79614219	1	89	53.2889696				
1	2E+08	1_152189893	15	19	4	15	17	10	0.210142	1	6	3	4	41	5	23	0.00150862	0.0878849	13.375	12.7496499				
1	2E+08	1_153197746	6	8	1	7	7	4	0.503048	1	24	13	24	7	19	16	0.96066018	1	11.25	8.51469318				
1	2E+08	1_153247535	14	18	9	15	16	12	0.497978	1	0	12	22	5	6	14	0.1466131	1	11.875	7.07990113				
1	2E+08	1_153275535	95	74	61	25	85	43	2.24E-05	0.00242	30	45	26	11	38	19	0.08276384	1	45.875	28.6178641	1.5E+08	1.5E+08	0.54027	ID=promoter_02679;
1	2E+08	1_153410550	139	132	210	105	136	158	0.996648	1	79	14	24	16	47	20	0.01141841	0.4373265	89.875	70.2229867				
1	2E+08	1_153533937	2	0	3	7	1	5	0.353258	1	12	0	4	14	6	9	0.65381141	1	5.25	5.31171213	1.5E+08	1.5E+08	0.37894	ID=promoter_02700;
1	2E+08	1_153536221	15	3	12	6	9	9	0.944235	1	8	0	0	3	4	2	0.76928717	1	5.875	5.4886246				
1	2E+08	1_153704184	153	65	169	134	109	152	0.385821	1	124	50	74	1	87	38	3.90E-04	0.0287779	96.25	57.8069447	1.5E+08	1.5E+08	0.3798	ID=promoter_02703;
1	2E+08	1_153723426	2	42	8	18	22	13	0.135741	1	24	0	2	3	12	3	0.09316162	1	12.375	14.735162				
1	2E+08	1_153794215	12	22	10	15	17	13	0.50439	1	83	13	19	1	48	10	1.56E-05	0.0018671	21.875	25.480735				
1	2E+08	1_153802127	6	21	8	35	14	22	0.691767	1	1	23	15	4	12	10	0.96947392	1	14.125	11.5935635				
1	2E+08	1_153850667	33	81	51	27	57	39	0.031869	1	103	15	47	7	59	27	0.0081436	0.3343203	45.5	32.7544981				
1	2E+08	1_153970010	6	29	3	10	18	7	0.033499	1	18	30	24	2	24	13	0.28736897	1	15.25	11.5232684				
1	2E+08	1_154146723	57	42	53	53	50	53	0.876335	1	74	35	45	36	55	41	0.5709762	1	49.375	12.8167023				
1	2E+08	1_154200285	21	50	49	28	36	39	0.942553	1	17	9	13	32	13	23	0.16598565	1	27.375	15.5557752	1.5E+08	1.5E+08	0.17383	ID=promoter_02722;
1	2E+08	1_154269131	41	31	77	99	36	88	7.45E-04	0.04918	63	58	76	60	61	68	0.57800607	1	63.125	21.3905019				
1	2E+08	1_154271213	8	14	8	2	11	5	0.189421	1	16	0	14	22	8	18	0.09791732	1	10.5	7.3872477				
1	2E+08	1_154447347	4	16	1	3	10	2	0.03464	1	13	0	18	11	7	15	0.16935974	1	8.25	7.0862039				
1	2E+08	1_155119567	448	158	241	235	303	238	1.92E-05	0.00212	74	116	90	37	95	64	0.14013563	1	174.88	132.408931				
1	2E+08	1_155126593	15	8	8	0	12	4	0.066899	1	46	23	27	24	35	26	0.69250145	1	18.875	14.4067197	1.6E+08	1.6E+08	0.10389	ID=promoter_02743;
1	2E+08	1_155139604	0	25	5	14	13	10	0.600182	1	0	9	14	10	5	12	0.17891578	1	9.625	8.29694436	1.6E+08	1.6E+08	0.22973	ID=promoter_02748;
1	2E+08	1_155151202	6	23	1	3	15	2	0.002248	0.12273	0	3	1	3	2	2	0.99713337	1	5	7.50238057	1.6E+08	1.6E+08	0.59827	ID=promoter_02752;
1	2E+08	1_155154781	21	44	21	47	33	34	0.868497	1	6	28	25	3	17	14	0.9475482	1	24.375	15.7111198				
1	2E+08	1_155160151	238	129	222	217	184	220	0.981764	1	0	7	12	44	4	28	4.79E-05	0.0049133	108.63	105.147431	1.6E+08	1.6E+08	0.49913	ID=promoter_02756;
1	2E+08	1_155174464	25	53	6	31	39	19	0.00521	0.24279	15	8	28	7	12	18	0.42746936	1	21.625	16.088483	1.6E+08	1.6E+08	0.33981	ID=promoter_02761;
1	2E+08	1_155181229	318	232	222	334	275	278	0.216377	1	105	124	158	71	115	115	0.84783628	1	195.5	97.4180975				
1	2E+08	1_155198168	198	73	139	129	136	134	0.357592	1	62	97	125	36	80	81	0.85507086	1	107.38	51.3167684	1.6E+08	1.6E+08	0.19568	ID=promoter_02768;

1	2E+08	1_155202027	0	0	3	3	0	3	0.278365	1	40	9	23	0	25	12	0.16190573	1	9.75	14.4790489				
1	2E+08	1_155204037	15	12	4	21	14	13	0.831264	1	0	16	2	2	8	2	0.20550608	1	9	7.94624799	1.6E+08	1.6E+08	0.35635	ID=promoter_02772;
1	2E+08	1_155204590	4	48	24	4	26	14	0.055012	1	29	22	31	48	26	40	0.12680108	1	26.25	16.8586561	1.6E+08	1.6E+08	0.35635	ID=promoter_02772;
1	2E+08	1_155214042	56	52	46	69	54	58	0.897169	1	81	55	32	26	68	29	0.0015925	0.0904103	52.125	17.9955352				
1	2E+08	1_155216732	1870	1927	3043	4232	1899	3638	2.99E-67	#######	350	235	963	339	293	651	1.94E-36	2.59E-33	1619.9	1444.59371				
1	2E+08	1_155259131	6	0	4	0	3	2	0.821455	1	21	0	7	12	11	10	0.99899888	1	6.25	7.30459738	1.6E+08	1.6E+08	0.16072	ID=promoter_02793;
1	2E+08	1_155383955	4	7	8	15	6	12	0.558139	1	2	9	6	2	6	4	0.87665919	1	6.625	4.27409138	1.6E+08	1.6E+08	0.23907	ID=promoter_02802;
1	2E+08	1_155492733	1	7	0	3	4	2	0.597627	1	8	12	6	8	10	7	0.84912739	1	5.625	4.03334317				
1	2E+08	1_155932597	9	1	4	9	5	7	0.955252	1	1	2	18	2	2	10	0.05067035	1	5.75	5.94618725				
1	2E+08	1_156007466	241	67	13	2	154	8	8.76E-35	#######	226	38	13	5	132	9	4.45E-22	3.40E-19	75.625	99.788258				
1	2E+08	1_156052145	141	320	153	223	231	188	9.11E-04	0.05851	423	295	395	203	359	299	0.39183384	1	269.13	106.355788				
1	2E+08	1_156140620	38	55	20	37	47	29	0.021726	0.74838	1	11	17	2	6	10	0.51486293	1	22.625	19.115719				
1	2E+08	1_156163602	5	29	12	15	17	14	0.615566	1	3	17	31	31	10	31	0.00200647	0.1098505	17.875	11.3318451				
1	2E+08	1_156197187	10	23	13	8	17	11	0.29617	1	36	20	10	1	28	6	0.00180324	0.1003399	15.125	10.8816687				
1	2E+08	1_156316278	35	29	19	23	32	21	0.11093	1	34	31	44	2	33	23	0.5739607	1	27.125	12.6878739	1.6E+08	1.6E+08	0.55124	ID=promoter_02817;
1	2E+08	1_156611833	31	3	20	27	17	24	0.842518	1	0	1	13	0	1	7	0.08320466	1	11.875	12.766446				
1	2E+08	1_156776046	5	30	4	1	18	3	0.001281	0.07728	14	17	19	27	16	23	0.39752759	1	14.625	10.7296319				
1	2E+08	1_157732402	62	18	29	94	40	62	0.386509	1	97	39	28	0	68	14	1.35E-07	2.75E-05	45.875	35.3004957				
1	2E+08	1_157733860	8	4	7	11	6	9	0.896349	1	17	0	6	0	9	3	0.27712561	1	6.625	5.65527567				
1	2E+08	1_157925097	24	30	7	10	27	9	0.002276	0.124	21	19	9	42	20	26	0.52081498	1	20.25	11.9013805	1.6E+08	1.6E+08	0.26409	ID=promoter_02833;
1	2E+08	1_157925164	359	227	281	403	293	342	0.998117	1	114	161	123	164	138	144	0.61030914	1	229	109.017692	1.6E+08	1.6E+08	0.26409	ID=promoter_02833;
1	2E+08	1_158163928	82	82	82	91	82	87	0.808426	1	81	17	40	23	49	32	0.30115968	1	62.25	30.3114783	1.6E+08	1.6E+08	0.65693	ID=promoter_02835;
1	2E+08	1_158169750	395	198	319	256	297	288	0.070268	1	267	179	252	98	223	175	0.2555291	1	245.5	90.080916				
1	2E+08	1_158330326	1	20	9	1	11	5	0.189421	1	21	1	2	26	11	14	0.73202476	1	10.125	10.5889093				
1	2E+08	1_159186401	0	17	2	4	9	3	0.131542	1	16	0	1	0	8	1	0.0848225	1	5	7.23088613				
1	2E+08	1_159528913	141	115	68	75	128	72	2.23E-06	0.00031	54	51	34	30	53	32	0.15525887	1	71	38.9138242				
1	2E+08	1_159612117	6	31	6	6	19	6	0.010861	0.43721	31	9	3	0	20	2	0.00122165	0.0738454	11.5	12.3172353				
1	2E+08	1_160455652	3	21	2	4	12	3	0.030625	0.98374	0	13	17	0	7	9	0.80856893	1	7.5	8.26351707				
1	2E+08	1_160481461	265	192	190	284	229	237	0.400994	1	48	132	149	46	90	98	0.54406322	1	163.25	88.3867314	1.6E+08	1.6E+08	0.68234	ID=promoter_02846;
1	2E+08	1_161675989	18	33	17	10	26	14	0.055012	1	59	59	50	84	59	67	0.52305481	1	41.25	25.932605				
1	2E+08	1_162158627	12	44	25	13	28	19	0.175883	1	26	17	12	20	22	16	0.76021399	1	21.125	10.776131				
1	2E+08	1_162526814	2200	1107	1578	1368	1654	1473	1.22E-13	#######	772	612	631	232	692	432	7.12E-10	2.14E-07	1062.5	634.509935				
1	2E+08	1_165604334	8	10	7	8	9	8	0.848833	1	5	5	0	5	5	3	0.8350419	1	6	3.02371578				

1	2E+08	1_165956335	101	67	99	222	84	161	0.001125	0.07	43	23	71	44	33	58	0.01165358	0.4440705	83.75	62.126024					1.7E+08	1.7E+08	0.6688	ID=promoter_02864;
1	2E+08	1_166694877	6	36	5	10	21	8	0.017144	0.62492	0	67	27	13	34	20	0.26570036	1	20.5	22.3031068	2E+08	1.7E+08	ID=ENSP0000262717;CDH20;NM_031891;	CDH20				
1	2E+08	1_166908202	0	10	20	12	5	16	0.124289	1	4	13	7	10	9	9	0.98716474	1	9.5	6.04743157								
1	2E+08	1_167012042	13	7	1	22	10	12	0.998584	1	0	2	0	3	1	2	0.81304206	1	6	7.81938982								
1	2E+08	1_167198624	14	19	43	16	17	30	0.39938	1	0	1	2	19	1	11	0.01045793	0.4067594	14.25	14.1598023								
1	2E+08	1_167557373	11	40	21	8	26	15	0.082345	1	40	17	36	24	29	30	0.9150801	1	24.625	12.7384401								
1	2E+08	1_167857037	11060	9234	12820	12760	10147	####	4.32E-07	#######	9890	7035	16638	5669	8463	11154	2.01E-131	9.90E-128	10638	3497.86428					1.7E+08	1.7E+08	0.09425	ID=promoter_02876;
1	2E+08	1_168198054	23	0	4	7	12	6	0.213448	1	12	11	3	1	12	2	0.04045894	1	7.625	7.59581652								
1	2E+08	1_169085936	20	42	28	42	31	35	0.988098	1	89	33	44	9	61	27	0.00474005	0.2185334	38.375	23.7783665								
1	2E+08	1_169209843	64	102	86	58	83	72	0.170668	1	126	56	39	33	91	36	4.86E-05	0.0049779	70.5	31.8926772	2E+08	1.7E+08	ID=ENSP0000381305;;					
1	2E+08	1_169227516	400	321	574	448	361	511	0.023561	0.80345	224	209	240	103	217	172	0.30739029	1	314.88	152.182632	2E+08	1.7E+08	ID=ENSP0000381305;;					
1	2E+08	1_169282772	1	5	0	5	3	3	0.981055	1	1	2	26	3	2	15	0.00415768	0.1961166	5.375	8.53459347								
1	2E+08	1_169283633	34	17	12	10	26	11	0.012533	0.48773	81	49	17	113	65	65	0.91092096	1	41.625	37.5078563								
1	2E+08	1_169321936	74	82	52	94	78	73	0.380917	1	0	71	39	0	36	20	0.17860656	1	51.5	36.0238017								
1	2E+08	1_169459747	184	73	301	303	129	302	1.76E-10	#######	0	31	43	34	16	39	0.00325528	0.1634313	121.13	124.321516	2E+08	1.7E+08	ID=ENSP0000350569;;NM_001144968;					
1	2E+08	1_169508974	0	16	4	1	8	3	0.206144	1	1	7	15	17	4	16	0.01700101	0.6008545	7.625	7.28868987	2E+08	1.7E+08	ID=ENSP0000350569;;NM_001144968;					
1	2E+08	1_169844817	142	35	72	132	89	102	0.987302	1	68	41	22	20	55	21	0.0016923	0.0952056	66.5	47.5214237								
1	2E+08	1_169968549	10846	9594	13152	13349	10220	####	2.57E-13	#######	8258	6587	17555	4228	7423	10892	1.95E-206	1.86E-202	10446	4234.49067					1.7E+08	1.7E+08	0.40623	ID=promoter_02898;
1	2E+08	1_170146437	126	77	92	87	102	90	0.14218	1	30	31	30	9	31	20	0.44595779	1	60.25	40.7772696					1.7E+08	1.7E+08	0.62932	ID=promoter_02901;
1	2E+08	1_170418776	14	59	18	18	37	18	0.006738	0.29841	14	39	53	38	27	46	0.03881692	1	31.625	18.1023874	2E+08	1.7E+08	ID=ENSP0000332773;IGDCC3;NM_004884;	IGDCC3				
1	2E+08	1_170427013	27	10	51	8	19	30	0.595918	1	0	0	0	0	0	0	1	1	12	18.2912938	2E+08	1.7E+08	ID=ENSP0000332773;IGDCC3;NM_004884;	IGDCC3				
1	2E+08	1_170459628	107	45	125	84	76	105	0.553183	1	85	79	74	87	82	81	0.92114189	1	85.75	23.4261758								
1	2E+08	1_170538091	81	86	52	62	84	57	0.005441	0.2519	74	16	49	37	45	43	0.98996144	1	57.125	23.6065215					1.7E+08	1.7E+08	0.54951	ID=promoter_02910;
1	2E+08	1_170727228	16	1	21	7	9	14	0.802715	1	36	1	16	0	19	8	0.15782218	1	12.25	12.5328141								
1	2E+08	1_170800825	51	8	42	36	30	39	0.914165	1	26	21	22	3	24	13	0.28736897	1	26.125	16.3657962								
1	2E+08	1_170944065	1	3	6	10	2	8	0.255957	1	36	13	4	0	25	2	1.29E-04	0.0113348	9.125	11.7161366					1.7E+08	1.7E+08	0.45613	ID=promoter_02916;
1	2E+08	1_171248738	46	96	59	48	71	54	0.053742	1	46	79	90	61	63	76	0.30076688	1	65.625	20.1489986								

1	2E+08	1_171664933	5	8	11	6	7	9	0.983538	1	7	5	10	7	6	9	0.65381141	1	7.375	2.19983766								
1	2E+08	1_171769932	6	10	25	11	8	18	0.296056	1	22	12	27	6	17	17	0.97589399	1	14.875	8.49264388								
1	2E+08	1_171795974	111	51	124	88	81	106	0.759299	1	12	5	24	1	9	13	0.58766318	1	52	49.5061324								
1	2E+08	1_171944137	4	11	5	1	8	3	0.206144	1	17	2	0	0	10	0	0.00970398	0.3824351	5	6.04743157								
1	2E+08	1_172079549	16	20	0	6	18	3	0.001281	0.07728	21	23	8	45	22	27	0.61865829	1	17.375	13.8144593								
1	2E+08	1_172178277	2	0	1	7	1	4	0.505922	1	19	10	7	0	15	4	0.06170633	1	5.75	6.54107898								
1	2E+08	1_172817776	10	15	2	1	13	2	0.006819	0.29841	0	4	8	2	2	5	0.46647814	1	5.25	5.25764749	2E+08	1.7E+08	ID=ENSP00000342101;MAP2K5;;	MAP2K5				
1	2E+08	1_172976682	147	65	117	230	106	174	0.023389	0.79837	58	57	60	64	58	62	0.73746359	1	99.75	62.256956	2E+08	1.7E+08	ID=ENSP00000369374;LBXCOR1;;	LBXCOR1	1.7E+08	1.7E+08	0.91028	ID=promoter_02940;
1	2E+08	1_173532795	5	11	2	5	8	4	0.357158	1	12	11	6	1	12	4	0.18067673	1	6.625	4.24053568	2E+08	1.7E+08	ID=ENSP00000327290;ITGA11;NM_001004439;	ITGA11				
1	2E+08	1_173848260	5	9	15	12	7	14	0.506445	1	5	15	23	30	10	27	0.01023344	0.4008669	14.25	8.69729022								
1	2E+08	1_174079212	11	0	2	0	6	1	0.108618	1	29	10	50	0	20	25	0.60650656	1	12.75	17.9900766								
1	2E+08	1_174193921	811	372	1423	1047	592	1235	2.43E-30	#######	421	359	404	138	390	271	0.00124345	0.0748183	621.88	432.896701					1.7E+08	1.7E+08	0.6932	ID=promoter_02958;
1	2E+08	1_174887581	3	32	23	3	18	13	0.404491	1	12	32	23	25	22	24	0.85866092	1	19.125	11.7526593								
1	2E+08	1_174897082	17	1	9	20	9	15	0.704209	1	31	0	10	0	16	5	0.08348313	1	11	11.1098412								
1	2E+08	1_175065222	13	12	34	23	13	29	0.146708	1	15	16	30	0	16	15	0.99951315	1	17.875	10.8422125								
1	2E+08	1_175120593	51	30	45	43	41	44	0.919489	1	138	93	68	42	116	55	1.66E-04	0.0140498	63.75	35.7441304								
1	2E+08	1_175257979	2	15	13	5	9	9	0.944235	1	13	25	21	2	19	12	0.57792516	1	12	8.53563957								
1	2E+08	1_175608979	14	19	18	21	17	20	0.999959	1	12	16	17	13	14	15	0.92682316	1	16.25	3.10529502								
1	2E+08	1_175850962	460	181	266	187	321	227	2.80E-08	#######	4	51	41	43	28	42	0.13765066	1	154.13	154.085725					1.8E+08	1.8E+08	0.10215	ID=promoter_02979;
1	2E+08	1_175871097	4	11	1	3	8	2	0.097885	1	8	9	23	5	9	14	0.47220951	1	8	6.9075528								
1	2E+08	1_175921140	13	33	14	23	23	19	0.524147	1	64	45	62	6	55	34	0.1739869	1	32.5	22.4690263								
1	2E+08	1_175934905	76	70	80	64	73	72	0.579377	1	12	21	2	6	17	4	0.02867352	0.9010784	41.375	34.0207395								
1	2E+08	1_176820591	6	70	38	101	38	70	0.077925	1	47	41	56	11	44	34	0.72702788	1	46.25	30.7420141								
1	2E+08	1_177020914	6	4	5	10	5	8	0.86114	1	37	22	4	17	30	11	0.02428668	0.7953491	13.125	11.6917248								
1	2E+08	1_177800746	7	26	17	16	17	17	0.897282	1	6	17	18	14	12	16	0.63262749	1	15.125	6.37937525								
1	2E+08	1_179444616	55	143	10	21	99	16	7.28E-17	#######	82	105	48	21	94	35	1.16E-05	0.0014428	60.625	46.4540864								
1	2E+08	1_180422044	45	391	106	76	218	91	5.59E-17	#######	336	306	416	224	321	320	0.65514136	1	237.5	146.595654								
1	2E+08	1_180579195	66	35	49	51	51	50	0.665611	1	3	18	19	44	11	32	0.00257396	0.1347203	35.625	20.9075345					1.8E+08	1.8E+08	0.60289	ID=promoter_03005;
1	2E+08	1_182448684	130	95	38	89	113	64	1.22E-05	0.00143	103	21	19	0	62	10	4.64E-08	1.02E-05	61.875	47.9178985								
1	2E+08	1_183464550	19	64	6	7	42	7	1.78E-07	#######	21	38	32	23	30	28	0.99966898	1	26.25	18.7749834								
1	2E+08	1_183501106	324	171	258	256	248	257	0.379222	1	314	153	174	11	234	93	9.97E-12	3.68E-09	207.63	102.715888					1.8E+08	1.8E+08	0.48442	ID=promoter_03011;
1	2E+08	1_183763007	20	33	16	10	27	13	0.024366	0.82541	8	12	8	5	10	7	0.84912739	1	14	9.05538514								

1	2E+08	1_186237246	391	190	268	215	291	242	3.74E-04	0.02789	195	152	270	40	174	155	0.93689671	1	215.13	101.720117					1.9E+08	1.9E+08	0.7544	ID=promoter_03019;
1	2E+08	1_187872751	124	53	100	72	89	86	0.438653	1	21	32	39	0	27	20	0.74799297	1	55.125	41.481278					1.9E+08	1.9E+08	0.34609	ID=promoter_03020;
1	2E+08	1_187893948	496	154	449	465	325	457	0.043667	1	134	124	194	64	129	129	0.83096889	1	260	178.013643					1.9E+08	1.9E+08	0.6653	ID=promoter_03022;
1	2E+08	1_187905006	555	288	462	440	422	451	0.388778	1	225	223	177	130	224	154	0.01643854	0.5841568	312.5	153.690784	2E+08	1.9E+08	ID=ENSP0000298292;C14orf104;NM_018139;	C14orf104				
1	2E+08	1_187924506	5	25	12	11	15	12	0.610117	1	2	41	43	20	22	32	0.26050199	1	19.875	15.5327994	2E+08	1.9E+08	ID=ENSP0000216367;POLE2;NM_002692;	POLE2				
1	2E+08	1_187961535	668	331	649	525	500	587	0.999893	1	412	301	271	69	357	170	3.06E-12	1.21E-09	403.25	203.636055					1.9E+08	1.9E+08	0.58634	ID=promoter_03025;
1	2E+08	1_188234950	8	27	17	25	18	21	0.99986	1	33	16	22	41	25	32	0.47866995	1	23.625	10.363914								
1	2E+08	1_188863975	3	103	16	29	53	23	1.83E-04	0.01503	6	11	50	27	9	39	2.59E-05	0.0028712	30.625	32.945572								
1	2E+08	1_189941425	65	33	88	60	49	74	0.388131	1	30	31	32	40	31	36	0.65410506	1	47.375	21.3403541					1.9E+08	1.9E+08	0.4978	ID=promoter_03050;
1	2E+08	1_190065807	39	697	716	884	368	800	3.12E-22	#######	69	263	686	203	166	445	3.25E-33	3.77E-30	444.63	335.015112								
1	2E+08	1_190490656	37	132	44	39	85	42	1.40E-05	0.0016	142	117	156	98	130	127	0.91554142	1	95.625	49.1206605								
1	2E+08	1_190963957	0	2	3	5	1	4	0.505922	1	26	1	2	6	14	4	0.08931834	1	5.625	8.46737436								
1	2E+08	1_191898026	8	43	4	8	26	6	3.38E-04	0.02566	27	32	37	44	30	41	0.26677765	1	25.375	16.4745475								
1	2E+08	1_192501929	137	58	153	131	98	142	0.272625	1	59	55	70	44	57	57	0.92144132	1	88.375	44.0192734					1.9E+08	1.9E+08	0.67654	ID=promoter_03077;
1	2E+08	1_193073948	17	53	6	3	35	5	9.64E-07	0.00015	5	34	16	4	20	10	0.26942782	1	17.25	17.7904469								
1	2E+08	1_193174927	36	132	41	45	84	43	3.20E-05	0.00329	49	94	159	37	72	98	0.04510121	1	74.125	48.339388								
1	2E+08	1_193745357	8	20	8	1	14	5	0.053576	1	1	16	3	1	9	2	0.1390433	1	7.25	7.32412842								
1	2E+08	1_193886616	4	18	2	6	11	4	0.10516	1	21	5	6	12	13	9	0.79589064	1	9.25	6.98467711								
1	2E+08	1_194689716	86	70	29	4	78	17	1.54E-11	#######	83	51	81	24	67	53	0.68854646	1	53.5	31.3551089								
1	2E+08	1_194859000	4	11	3	15	8	9	0.996316	1	14	0	10	4	7	7	0.99000271	1	7.625	5.5789784								
1	2E+08	1_194972545	8	3	2	7	6	5	0.851367	1	5	17	0	20	11	10	0.99899888	1	7.75	7.16638981								
1	2E+08	1_194994228	76	45	54	82	61	68	0.959207	1	0	7	4	1	4	3	0.96209564	1	33.625	34.7683662								
1	2E+08	1_196856968	3	4	7	3	4	5	0.995512	1	20	20	7	6	20	7	0.06990458	1	8.75	7.12640963								
1	2E+08	1_197214771	187	83	142	107	135	125	0.16148	1	78	121	23	83	100	53	0.00385961	0.1846363	103	48.905156					2E+08	2E+08	0.47364	ID=promoter_03109;
1	2E+08	1_197306035	1	64	23	38	33	31	0.673228	1	27	38	39	28	33	34	0.91076368	1	32.25	17.8385618	2E+08	2E+08	ID=ENSP0000378557;DAAM1;NM_014992;	DAAM1				
1	2E+08	1_197398337	227	89	197	179	158	188	0.991234	1	53	30	60	6	42	33	0.7761727	1	105.13	83.8662839					2E+08	2E+08	0.46826	ID=promoter_03110;
1	2E+08	1_197483808	310	912	387	418	611	403	1.09E-18	#######	828	886	1002	620	857	811	0.91550187	1	670.38	271.530023								
1	2E+08	1_198119442	29	10	18	23	20	21	0.939065	1	9	17	5	3	13	4	0.12786863	1	14.25	9.0514403								
1	2E+08	1_199170423	439	273	394	429	356	412	0.982917	1	451	267	295	122	359	209	4.77E-07	8.55E-05	333.75	114.650712								

1	2E+08	1_199256723	420	203	301	234	312	268	8.65E-04	0.0564	153	119	92	55	136	74	9.44E-04	0.0600655	197.13	120.213426					2E+08	2E+08	0.49428	ID=promoter_03137;
1	2E+08	1_199352114	10	41	12	15	26	14	0.055012	1	2	1	49	51	2	50	3.62E-11	1.28E-08	22.625	20.9143662								
1	2E+08	1_199482381	104	21	101	108	63	105	0.085884	1	0	0	16	39	0	28	2.76E-07	5.26E-05	48.625	47.785047	2E+08	2E+08	ID=ENSP0000306124-D2;PRKCE;NM_005400;	PRKCE				
1	2E+08	1_199668562	21	23	37	32	22	35	0.531372	1	27	19	20	2	23	11	0.18449623	1	22.625	10.4326069								
1	2E+08	1_199901764	360	434	294	308	397	301	6.44E-08	#######	739	537	487	234	638	361	3.53E-13	1.56E-10	424.13	163.27666								
1	2E+08	1_199904150	56	42	16	19	49	18	4.95E-05	0.00479	38	26	38	16	32	27	0.9371194	1	31.375	14.4512728								
1	2E+08	1_200321756	77	99	84	66	88	75	0.124601	1	45	94	31	36	70	34	0.00708719	0.2999196	66.5	26.408873					2E+08	2E+08	0.30152	ID=promoter_03148;
1	2E+08	1_200550735	34	13	12	0	24	6	9.38E-04	0.05976	0	16	5	0	8	3	0.3843038	1	10	11.6496475								
1	2E+08	1_201639350	3	28	11	3	16	7	0.076817	1	2	5	18	5	4	12	0.09850366	1	9.375	9.24179172								
1	2E+08	1_202349825	3	17	5	5	10	5	0.276106	1	21	10	25	1	16	13	0.9394435	1	10.875	9.01486867								
1	2E+08	1_202767700	21	9	3	8	15	6	0.065303	1	0	0	6	0	0	3	0.19828566	1	5.875	7.12014245	2E+08	2E+08	ID=ENSP0000343925;ESR2;NM_001437;	ESR2				
1	2E+08	1_203553589	242	182	167	174	212	171	0.001214	0.07432	87	172	120	77	130	99	0.33611656	1	152.63	54.7460044								
1	2E+08	1_203774694	4	30	3	20	17	12	0.395844	1	26	30	14	20	28	17	0.38227241	1	18.375	10.6628527								
1	2E+08	1_205423227	13	24	14	36	19	25	0.931133	1	15	24	47	46	20	47	0.00147388	0.0863896	27.375	13.9789382	2E+08	2.1E+08	ID=ENSP0000369423;;					
1	2E+08	1_207512149	12	12	9	9	12	9	0.592799	1	1	7	18	1	4	10	0.21760186	1	8.625	5.73055719								
1	2E+08	1_207850056	28	88	37	39	58	38	0.01823	0.65682	62	34	53	8	48	31	0.28825568	1	43.625	24.1006076								
1	2E+08	1_209922635	22	42	39	6	32	23	0.194505	1	38	11	23	14	25	19	0.80752973	1	24.375	13.8454479								
1	2E+08	1_210648624	68	39	39	34	54	37	0.03878	1	24	87	46	28	56	37	0.27526121	1	45.625	21.4272023								
1	2E+08	1_211524818	54	30	18	54	42	36	0.38232	1	18	47	13	1	33	7	5.50E-04	0.0381958	29.375	20.1985678								
1	2E+08	1_212998223	124	88	76	84	106	80	0.011061	0.44213	216	200	202	76	208	139	0.01107478	0.4273755	133.25	62.2707223								
1	2E+08	1_213005574	132	62	79	84	97	82	0.090298	1	56	18	34	16	37	25	0.47119089	1	60.125	38.7609432								
1	2E+08	1_213109166	10	0	8	1	5	5	0.968625	1	0	16	4	21	8	13	0.4490005	1	7.5	7.81938982								
1	2E+08	1_213889193	4	9	5	7	7	6	0.851931	1	1	0	21	16	1	19	1.49E-04	0.0128238	7.875	7.29848321					2.1E+08	2.1E+08	0.37106	ID=promoter_03227;
1	2E+08	1_213901727	20	12	13	10	16	12	0.497978	1	0	11	21	6	6	14	0.1466131	1	11.625	6.86476511								
1	2E+08	1_214995011	5	19	1	0	12	1	0.003675	0.18279	22	25	15	13	24	14	0.38067267	1	12.5	9.56182887								
1	2E+08	1_216935213	63	106	85	111	85	98	0.993282	1	11	61	82	34	36	58	0.03081596	0.9525854	69.125	34.3238255								
1	2E+08	1_217192509	29	132	77	69	81	73	0.261624	1	74	60	58	42	67	50	0.50851753	1	67.625	30.6451464								
1	2E+08	1_217251648	2	38	10	11	20	11	0.118302	1	52	26	18	0	39	9	2.52E-04	0.020023	19.625	18.094494								
1	2E+08	1_217357927	2	2	8	10	2	9	0.179815	1	16	16	10	3	16	7	0.23724011	1	8.375	5.75543222								
1	2E+08	1_217597104	35	40	20	21	38	21	0.018051	0.65084	5	22	16	1	14	9	0.68990751	1	20	13.2664992								
1	2E+08	1_217809201	13	27	7	12	20	10	0.076235	1	2	23	26	13	13	20	0.35609139	1	15.375	9.08589959								
1	2E+08	1_218146704	240	158	294	180	199	237	0.987533	1	89	31	29	2	60	16	1.45E-05	0.0017545	127.88	107.112007								
1	2E+08	1_218848762	14	8	6	0	11	3	0.050503	1	0	0	23	0	0	12	0.00154584	0.0883233	6.375	8.45048604								

1	2E+08	1_220688336	34	58	28	43	46	36	0.18711	1	69	22	26	76	46	51	0.67825371	1	44.5	20.729551								
1	2E+08	1_221943314	8	56	25	40	32	33	0.87385	1	0	76	21	27	38	24	0.33399749	1	31.625	24.9853529								
1	2E+08	1_222238515	15	19	15	25	17	20	0.999959	1	21	13	15	1	17	8	0.27279187	1	15.5	7.07106781								
1	2E+08	1_222903281	28	39	20	6	34	13	0.001425	0.08487	25	28	33	4	27	19	0.6528836	1	22.875	12.3570396								
1	2E+08	1_223201536	2	13	2	5	8	4	0.357158	1	2	2	33	1	2	17	0.00148567	0.0868666	7.5	11.0194633	2E+08	2.2E+08	ID=ENSP00000347106;PTPRS;;	PTPRS				
1	2E+08	1_223470332	7	1250	2332	1646	629	1989	########	#######	40	749	972	386	395	679	4.55E-22	3.44E-19	922.75	806.644682								
1	2E+08	1_224493862	26	100	34	32	63	33	5.95E-04	0.04096	107	113	109	63	110	86	0.49547429	1	73	38.3443048								
1	2E+08	1_225368030	142	50	110	108	96	109	0.973359	1	4	53	36	5	29	21	0.68832436	1	63.5	51.1524333					2.3E+08	2.3E+08	0.30419	ID=promoter_03271;
1	2E+08	1_226015760	36	15	37	37	26	37	0.751146	1	34	7	15	2	21	9	0.13945494	1	22.875	14.6720093					2.3E+08	2.3E+08	0.59167	ID=promoter_03275;
1	2E+08	1_227059722	122	53	103	89	88	96	0.886243	1	31	31	23	13	31	18	0.28104051	1	58.125	41.0833213					2.3E+08	2.3E+08	0.43847	ID=promoter_03284;
1	2E+08	1_227340401	252	109	158	147	181	153	0.011235	0.4486	88	90	62	7	89	35	5.45E-05	0.0054455	114.13	73.1914076	2E+08	2.3E+08	ID=ENSP00000371230;AK3;NM_016282;	AK3	2.3E+08	2.3E+08	0.42615	ID=promoter_03287;
1	2E+08	1_227609142	16	36	15	11	26	13	0.035221	1	42	17	37	54	30	46	0.09558038	1	28.5	15.7570846								
1	2E+08	1_227747946	27	16	5	5	22	5	0.001077	0.06758	21	60	30	41	41	36	0.97213063	1	25.625	18.5313441								
1	2E+08	1_227800352	24	46	13	15	35	14	0.001717	0.09971	0	30	17	27	15	22	0.38478854	1	21.5	13.6591152								
1	2E+08	1_229150484	9	3	1	1	6	1	0.108618	1	0	8	7	13	4	10	0.21760186	1	5.25	4.68279526								
1	2E+08	1_229331129	17	21	1	9	19	5	0.004955	0.23359	23	4	32	3	14	18	0.65378371	1	13.75	11.1963004								
1	2E+08	1_229695000	45	47	18	37	46	28	0.020633	0.72153	36	26	30	0	31	15	0.10937197	1	29.875	15.3942243								
1	2E+08	1_230729809	9	68	11	26	39	19	0.00521	0.24279	1	20	17	6	11	12	0.92663959	1	19.75	21.0696125								
1	2E+08	1_230931133	2	0	5	21	1	13	0.014136	0.5348	11	16	7	0	14	4	0.08931834	1	7.75	7.70435869								
1	2E+08	1_231084598	2	15	8	3	9	6	0.554309	1	12	5	15	9	9	12	0.70935077	1	8.625	5.09726818								
1	2E+08	1_231138107	4	4	4	0	4	2	0.597627	1	33	0	2	1	17	2	0.0046333	0.2138882	6	11.045361								
1	2E+08	1_231182567	10	2	5	12	6	9	0.896349	1	0	0	3	9	0	6	0.0393172	1	5.125	4.67325216					2.3E+08	2.3E+08	0.49863	ID=promoter_03308;
1	2E+08	1_231249934	16	103	28	41	60	35	0.00364	0.18261	36	15	89	5	26	47	0.02078637	0.7038032	41.625	35.7288899								
1	2E+08	1_231477807	57	25	15	23	41	19	0.002448	0.13209	16	6	44	10	11	27	0.01818526	0.631944	24.5	17.5417868					2.3E+08	2.3E+08	0.48807	ID=promoter_03310;
1	2E+08	1_231504647	16	36	9	26	26	18	0.220252	1	41	49	33	21	45	27	0.1974124	1	28.875	13.3677383								
1	2E+08	1_232176749	1	6	8	6	4	7	0.813458	1	16	1	11	6	9	9	0.98716474	1	6.875	4.96955014								
1	2E+08	1_232897722	58	62	73	83	60	78	0.835698	1	47	39	145	47	43	96	4.89E-06	0.0006772	69.25	33.8600059	2E+08	2.3E+08	ID=ENSP00000366453;TJP2;NM_004817;	TJP2				
1	2E+08	1_233077966	39	12	50	25	26	38	0.687307	1	0	5	7	0	3	4	0.89184014	1	17.25	18.8357563	2E+08	2.3E+08	ID=ENSP00000265381;APBA1;NM_001163;	APBA1				
1	2E+08	1_233968263	5	39	0	0	22	0	2.49E-06	0.00035	27	9	8	1	18	5	0.0406475	1	11.125	14.2972275								
1	2E+08	1_234800528	1	34	10	14	18	12	0.307258	1	2	0	10	42	1	26	3.52E-06	0.0005061	14.125	15.6974748								

1	2E+08	1_234858432	0	23	3	8	12	6	0.213448	1	1	0	11	0	1	6	0.13767373	1	5.75	8.10202796								
1	2E+08	1_235528048	20	68	64	74	44	69	0.319562	1	67	76	61	33	72	47	0.17077858	1	57.875	20.2656465					2.4E+08	2.4E+08	0.25233	ID=promoter_03340;
1	2E+08	1_236108561	13	72	31	24	43	28	0.048681	1	76	46	49	56	61	53	0.94212905	1	45.875	22.3059089					2.4E+08	2.4E+08	0.67367	ID=promoter_03345;
1	2E+08	1_236758603	7	12	5	0	10	3	0.082185	1	10	25	22	0	18	11	0.54801163	1	10.125	9.3110917								
1	2E+08	1_237885345	224	190	222	195	207	209	0.310132	1	300	300	386	111	300	249	0.43488452	1	241	84.7298227								
1	2E+08	1_239073454	214	102	152	152	158	152	0.21713	1	36	15	27	33	26	30	0.71540497	1	91.375	74.5959354								
1	2E+08	1_239307507	16	78	24	41	47	33	0.073589	1	45	54	102	84	50	93	2.73E-04	0.0212771	55.5	30.1330383					2.4E+08	2.4E+08	0.53437	ID=promoter_03356;
1	2E+08	1_239381743	115	43	47	121	79	84	0.821783	1	16	21	14	1	19	8	0.15782218	1	47.25	46.2253795								
1	2E+08	1_239700143	43	222	88	49	133	69	1.16E-07	#######	143	191	162	69	167	116	0.0566128	1	120.88	67.9567877								
1	2E+08	1_240295405	239	88	158	284	164	221	0.403369	1	85	75	132	24	80	78	0.95099755	1	135.63	87.9787921					2.4E+08	2.4E+08	0.73916	ID=promoter_03362;
1	2E+08	1_240666765	80	28	102	48	54	75	0.638077	1	13	23	56	0	18	28	0.22298182	1	43.75	34.6688415					2.4E+08	2.4E+08	0.75528	ID=promoter_03367;
1	2E+08	1_241158957	32	18	17	20	25	19	0.35609	1	6	0	3	0	3	2	0.9363008	1	12	11.5263673								
1	2E+08	1_241267138	33	17	43	25	25	34	0.853863	1	28	13	29	3	21	16	0.83919704	1	23.875	12.5064269								
1	2E+08	1_241627552	14	103	9	16	59	13	7.55E-09	#######	25	8	13	1	17	7	0.1782776	1	23.625	32.8108758								
1	2E+08	1_241723890	20	53	22	23	37	23	0.052004	1	48	61	50	35	55	43	0.70389935	1	39	16.053482								
1	2E+08	1_241888683	114	60	117	153	87	135	0.124295	1	34	43	36	1	39	19	0.06479145	1	69.75	52.2022167					2.4E+08	2.4E+08	0.37469	ID=promoter_03373;
1	2E+08	1_242259306	14	8	12	12	11	12	0.985018	1	37	7	22	1	22	12	0.32694425	1	14.125	11.0510181								
1	2E+08	1_242313591	1	28	14	5	15	10	0.374041	1	0	10	19	2	5	11	0.25523295	1	9.875	9.93460762								
1	2E+08	1_242548327	22	7	13	4	15	9	0.269309	1	0	22	7	25	11	16	0.51039395	1	12.5	9.45666809								
1	2E+08	1_243098633	1	19	14	8	10	11	0.989255	1	25	6	3	34	16	19	0.76520201	1	13.75	11.5604004								
1	2E+08	1_243421081	0	25	1	6	13	4	0.041893	1	1	3	7	3	2	5	0.46647814	1	5.75	8.15475322								
1	2E+08	1_244532000	7	28	3	10	18	7	0.033499	1	25	27	39	4	26	22	0.95151398	1	17.875	13.5059511								
1	2E+08	1_244546761	4	22	4	0	13	2	0.006819	0.29841	55	19	21	15	37	18	0.07385545	1	17.5	17.3945804								
1	2E+08	1_244772068	17	2	4	1	10	3	0.082185	1	6	0	17	10	3	14	0.01844097	0.6375434	7.125	6.85435211								
1	2E+08	1_245394096	3	15	1	7	9	4	0.244237	1	0	0	9	14	0	12	0.00154584	0.0883233	6.125	6.10474055								
1	2E+08	1_245586857	5	13	0	4	9	2	0.058587	1	36	0	6	2	18	4	0.01933304	0.6616555	8.25	11.9612469								
1	2E+08	1_246606842	226	109	163	130	168	147	0.033683	1	91	75	62	33	83	48	0.03176695	0.9755061	111.13	61.4989837					2.5E+08	2.5E+08	0.49043	ID=promoter_03393;
1	2E+08	1_247114239	5	18	7	10	12	9	0.592799	1	0	5	20	22	3	21	5.76E-04	0.0398271	10.875	8.1141411								
1	2E+08	1_247433958	75	27	97	131	51	114	5.09E-04	0.0358	43	16	33	46	30	40	0.31832457	1	58.5	39.3809236	2E+08	2.5E+08	ID=ENSP00000338127;TESK1;NM_006285;	TESK1	2.5E+08	2.5E+08	0.52656	ID=promoter_03408;
1	2E+08	1_247508987	6	22	5	21	14	13	0.831264	1	36	13	8	16	25	12	0.16190573	1	15.875	10.3845696								
1	2E+08	1_247518204	8	32	27	15	20	21	0.939065	1	40	50	47	39	45	43	0.98996144	1	32.25	14.8876747								
1	2E+08	1_247579898	50	137	58	50	94	54	1.10E-04	0.00971	109	64	73	49	87	61	0.24474733	1	73.75	32.354068								
1	2E+08	1_247936101	26	67	29	45	47	37	0.188658	1	21	51	54	34	36	44	0.46706033	1	40.875	15.9412986								

1	2E+08	1_248111133	4	6	2	7	5	5	0.968625	1	12	8	21	31	10	26	0.01509923	0.5480081	11.375	9.85520167					2.5E+08	2.5E+08	0.28243	ID=promoter_03429;
1	2E+08	1_248375168	17	51	33	35	34	34	0.805115	1	38	38	19	3	38	11	0.0015633	0.0891068	29.25	15.2010338	2E+08	2.5E+08	ID=ENSP0000259605;RNF38;NM_022781;	RNF38				
1	2E+08	1_248547847	20	9	24	15	15	20	0.931941	1	3	9	1	0	6	1	0.20114407	1	10.125	8.88718982								
1	2E+08	1_249015248	1	1	4	10	1	7	0.163779	1	7	1	1	15	4	8	0.43697044	1	5	5.26443594	2E+08	2.5E+08	ID=ENSP0000350844;PAX5;NM_016734;	PAX5				
1	2E+08	1_249138105	195	132	221	218	164	220	0.428356	1	45	45	68	76	45	72	0.01458897	0.5322062	125	76.777973	2E+08	2.5E+08	ID=ENSP0000350844;PAX5;NM_016734;	PAX5				
1	2E+08	1_249144454	143	29	165	133	86	149	0.014977	0.56219	63	75	51	25	69	38	0.03342088	1	85.5	54.1716847					2.5E+08	2.5E+08	0.63615	ID=promoter_03447;
1	2E+08	1_249698168	8	40	4	7	24	6	9.38E-04	0.05976	44	46	30	13	45	22	0.04373955	1	24	17.896528								
1	3E+08	1_250006474	14	29	17	8	22	13	0.135741	1	2	6	14	1	4	8	0.43697044	1	11.375	9.1953016								
1	3E+08	1_250039822	1	19	1	12	10	7	0.570701	1	2	13	4	1	8	3	0.3843038	1	6.625	7.02927552								
1	3E+08	1_250068289	355	123	278	227	239	253	0.523388	1	165	109	120	67	137	94	0.07859753	1	180.5	98.0670325					2.5E+08	2.5E+08	0.48294	ID=promoter_03459;
1	3E+08	1_250690270	41	27	23	24	34	24	0.156692	1	16	28	12	23	22	18	0.92557456	1	24.25	8.6478734								
1	3E+08	1_250761085	15	45	24	26	30	25	0.447286	1	9	15	48	16	12	32	0.00478712	0.2203477	24.75	14.4790489								
1	3E+08	1_250881730	805	738	1854	1695	772	1775	1.12E-55	#######	744	483	2044	649	614	1347	5.48E-73	1.35E-69	1126.5	625.294673					2.5E+08	2.5E+08	0.56147	ID=promoter_03471;
1	3E+08	1_250961869	295	124	169	141	210	155	5.89E-05	0.00561	83	66	62	46	75	54	0.36489186	1	123.25	81.5243171	3E+08	2.5E+08	ID=ENSP0000364289;NCBP1;NM_002486;	NCBP1	2.5E+08	2.5E+08	0.60008	ID=promoter_03473;
1	3E+08	1_251216496	9	44	20	62	27	41	0.579427	1	1	27	25	26	14	26	0.10164664	1	26.75	19.1516504								
1	3E+08	1_251247214	2128	1519	1999	1704	1824	1852	6.89E-05	0.00643	1369	1173	1216	563	1271	890	9.83E-10	2.88E-07	1458.9	500.649132					2.5E+08	2.5E+08	0.2855	ID=promoter_03478;
1	3E+08	1_251316572	72	22	16	12	47	14	7.35E-06	0.00091	44	7	14	8	26	11	0.081859	1	24.375	22.5511324					2.5E+08	2.5E+08	0.38239	ID=promoter_03479;
1	3E+08	1_251338819	0	6	2	4	3	3	0.981055	1	67	70	96	50	69	73	0.73345856	1	36.875	38.3273028					2.5E+08	2.5E+08	0.38183	ID=promoter_03480;
1	3E+08	1_251469490	13	24	15	18	19	17	0.718296	1	2	12	17	27	7	22	0.01139713	0.4370832	16	7.6718409	3E+08	2.5E+08	ID=ENSP0000364239;TRIM14;NM_033220;	TRIM14	2.5E+08	2.5E+08	0.4468	ID=promoter_03483;
1	3E+08	1_251506660	157	81	143	165	119	154	0.722807	1	23	37	64	35	30	50	0.03617913	1	88.125	58.523347	3E+08	2.5E+08	ID=ENSP0000364218;CORO2A;;	CORO2A				
1	3E+08	1_251589635	329	306	297	267	318	282	0.002786	0.14699	141	49	122	47	95	85	0.97216946	1	194.75	117.916132	3E+08	2.5E+08	ID=ENSP0000364207;TBC1D2;NM_018421;	TBC1D2				
1	3E+08	1_251596701	1117	362	826	770	740	798	0.257292	1	381	340	404	137	361	271	0.0314678	0.9666659	542.13	326.334376					2.5E+08	2.5E+08	0.38098	ID=promoter_03486;
1	3E+08	1_251722385	6	48	42	31	27	37	0.827591	1	26	70	82	81	48	82	0.00292374	0.1489331	48.25	27.5201225	3E+08	2.5E+08	ID=ENSP0000259455;GABBR2;NM_005458;	GABBR2				

1	3E+08	1_251885512	3	8	5	1	6	3	0.462004	1	0	12	7	4	6	6	0.99142475	1	5	3.92792202	3E+08	2.5E+08	ID=ENSP00000259455;GABBR2;NM_005458;	GABBR2				
1	3E+08	1_252029235	2	27	32	53	15	43	0.008706	0.36691	36	12	31	28	24	30	0.54878546	1	27.625	15.3337862								
1	3E+08	1_252185756	6	18	0	8	12	4	0.066899	1	0	2	6	0	1	3	0.56022579	1	5	6.14119579	3E+08	2.5E+08	ID=ENSP00000364150;GALNT12;NM_024642;	GALNT12				
1	3E+08	1_253253839	15	30	14	9	23	12	0.065071	1	0	29	6	0	15	3	0.02833385	0.8918846	12.875	11.6672619	3E+08	2.5E+08	ID=ENSP00000333122;NR4A3;NM_173200;	NR4A3				
1	3E+08	1_253306713	2302	953	2157	1599	1628	1878	0.885882	1	1029	730	689	396	880	543	5.62E-13	2.40E-10	1231.9	707.108082					2.5E+08	2.5E+08	0.34459	ID=promoter_03504;
1	3E+08	1_253458291	11	12	19	16	12	18	0.803442	1	2	5	29	3	4	16	0.01700101	0.6008545	12.125	9.14076427								
1	3E+08	1_254396643	34	55	36	13	45	25	0.00909	0.37919	29	53	30	17	41	24	0.19624561	1	33.375	14.9946419								
1	3E+08	1_254802739	460	187	297	227	324	262	3.97E-05	0.00398	97	115	133	47	106	90	0.8285738	1	195.38	132.809141					2.5E+08	2.5E+08	0.41039	ID=promoter_03515;
1	3E+08	1_256434123	108	73	86	48	91	67	0.014115	0.53473	20	24	26	69	22	48	0.00281867	0.1441467	56.75	32.3849612								
1	3E+08	1_258261875	3105	878	2804	2031	1992	2418	0.526417	1	1192	800	911	481	996	696	7.00E-08	1.51E-05	1525.3	993.574463					2.6E+08	2.6E+08	0.3884	ID=promoter_03528;
1	3E+08	1_259377444	297	174	254	263	236	259	0.760807	1	292	200	409	270	246	340	9.79E-06	0.0012447	269.88	70.6650803								
1	3E+08	1_260487344	6	20	9	14	13	12	0.835334	1	56	21	28	33	39	31	0.81486795	1	23.375	15.999442								
1	3E+08	1_260503597	692	322	754	438	507	596	0.999335	1	184	189	245	72	187	159	0.72519401	1	362	247.658175					2.6E+08	2.6E+08	0.32639	ID=promoter_03539;
1	3E+08	1_260506715	362	179	278	238	271	258	0.055664	1	156	128	145	77	142	111	0.40325255	1	195.38	92.0526829								
1	3E+08	1_261138671	6	17	12	5	12	9	0.592799	1	0	45	27	27	23	27	0.70626281	1	17.375	14.9373692								
1	3E+08	1_261415558	110	99	57	89	105	73	0.002417	0.1305	29	19	15	13	24	14	0.38067267	1	53.875	40.4384453								
1	3E+08	1_261445648	26	0	30	4	13	17	0.957342	1	24	5	1	0	15	1	0.00364468	0.1775497	11.25	12.9917556								
1	3E+08	1_261599493	7	5	9	9	6	9	0.896349	1	20	10	3	3	15	3	0.02833385	0.8918846	8.25	5.47070118								
1	3E+08	1_262276792	0	15	16	15	8	16	0.45954	1	0	0	13	0	0	7	0.02292696	0.7568963	7.375	7.92712342								
1	3E+08	1_262522786	37	67	39	49	52	44	0.277399	1	18	15	3	3	17	3	0.01239331	0.4669004	28.875	22.9187229								
1	3E+08	1_262760030	4	24	33	19	14	26	0.376149	1	0	19	23	1	10	12	0.83305499	1	15.375	12.1999707								
1	3E+08	1_263039185	3	69	11	39	36	25	0.12572	1	45	15	58	7	30	33	0.79414776	1	30.875	25.2101878								
1	3E+08	1_263085927	7	24	24	11	16	18	0.992646	1	31	19	35	11	25	23	0.99965163	1	20.25	10.0676285								
1	3E+08	1_263221370	8	67	12	25	38	19	0.007518	0.3239	54	80	102	25	67	64	0.98543033	1	46.625	34.3841222								
1	3E+08	1_263244039	30	27	44	46	29	45	0.499058	1	60	36	44	43	48	44	0.99854654	1	41.25	10.3475325					2.6E+08	2.6E+08	0.31129	ID=promoter_03572;
1	3E+08	1_263361942	22	32	8	12	27	10	0.004472	0.21437	29	19	42	5	24	24	0.96613769	1	21.125	12.7440462								
1	3E+08	1_263772342	8	30	9	27	19	18	0.809248	1	10	2	22	5	6	14	0.1466131	1	14.125	10.6293059								
1	3E+08	1_263779627	3	14	5	4	9	5	0.390586	1	25	5	11	2	15	7	0.31064188	1	8.625	7.79995421								
1	3E+08	1_264190228	36	30	40	29	33	35	0.917442	1	9	7	14	0	8	7	0.9937864	1	20.625	14.9278024								
1	3E+08	1_264273383	971	403	679	554	687	617	8.68E-06	0.00106	446	312	281	223	379	252	2.17E-04	0.0177051	483.63	246.985215					2.6E+08	2.6E+08	0.55518	ID=promoter_03581;
1	3E+08	1_264812711	53	83	137	80	68	109	0.127589	1	47	42	18	4	45	11	1.13E-04	0.0101411	58	41.8637245								

1	3E+08	1_264969863	11	5	1	22	8	12	0.864244	1	5	7	16	8	6	12	0.2888538	1	9.375	6.78101341								
1	3E+08	1_264987593	32	64	40	34	48	37	0.154373	1	66	28	41	6	47	24	0.05449507	1	38.875	19.4307378								
1	3E+08	1_265166850	9	43	8	6	26	7	7.99E-04	0.0525	34	4	24	14	19	19	0.97309647	1	17.75	14.4098181								
1	3E+08	1_265254960	14	3	24	20	9	22	0.165185	1	2	1	26	1	2	14	0.00692302	0.294895	11.375	10.8751026					2.7E+08	2.7E+08	0.11758	ID=promoter_03588;
1	3E+08	1_265337464	17	29	9	21	23	15	0.200003	1	31	25	12	20	28	16	0.29661245	1	20.5	7.74596669					2.7E+08	2.7E+08	0.62975	ID=promoter_03591;
1	3E+08	1_265337653	0	75	345	328	38	337	6.08E-43	#######	0	97	164	25	49	95	1.01E-04	0.0092979	129.25	139.13996					2.7E+08	2.7E+08	0.62975	ID=promoter_03591;
1	3E+08	1_265867606	17	100	15	26	59	21	4.20E-06	0.00055	110	28	59	7	69	33	0.00628004	0.272465	45.25	40.0419423	3E+08	2.7E+08	ID=ENSP00000363373;ROD1;NM_005156;	ROD1				
1	3E+08	1_265940690	84	78	101	70	81	86	0.812872	1	43	62	16	15	53	16	1.88E-04	0.0155974	58.625	31.4412718					2.7E+08	2.7E+08	0.69936	ID=promoter_03597;
1	3E+08	1_266414727	20	72	29	17	46	23	0.002685	0.14289	78	39	45	3	59	24	0.00219251	0.1186258	37.875	26.3950076	3E+08	2.7E+08	ID=ENSP00000363349;SNX30;NM_001012994;	SNX30				
1	3E+08	1_266859798	385	206	254	131	296	193	9.97E-10	#######	32	31	48	1	32	25	0.81386949	1	136	135.572859	3E+08	2.7E+08	ID=ENSP00000259406;RGS3;NM_144488;	RGS3				
1	3E+08	1_266893308	24	6	22	3	15	13	0.725173	1	36	0	5	0	18	3	0.00813808	0.3342859	12	13.4907376	3E+08	2.7E+08	ID=ENSP00000259406;RGS3;NM_144488;	RGS3				
1	3E+08	1_266904951	117	50	61	67	84	64	0.032264	1	21	16	15	1	19	8	0.15782218	1	43.5	38.1425896	3E+08	2.7E+08	ID=ENSP00000259406;RGS3;NM_144488;	RGS3				
1	3E+08	1_266973211	12	7	13	19	10	16	0.741563	1	3	23	44	6	13	25	0.09257925	1	15.875	13.1848127	3E+08	2.7E+08	ID=ENSP00000259406;RGS3;NM_144488;	RGS3				
1	3E+08	1_267055614	16	11	16	5	14	11	0.605836	1	0	0	1	0	0	1	0.58312783	1	6.125	7.16015762								
1	3E+08	1_267178991	161	41	91	57	101	74	0.008093	0.34587	0	0	9	0	0	5	0.06742467	1	44.875	57.4492012								
1	3E+08	1_267198180	139	68	95	93	104	94	0.185198	1	32	33	53	74	33	64	0.00201799	0.1104388	73.375	35.7488761								
1	3E+08	1_267375733	69	8	17	30	39	24	0.040785	1	0	0	4	1	0	3	0.19828566	1	16.125	23.7573297								
1	3E+08	1_267582256	9	11	5	1	10	3	0.082185	1	9	13	22	0	11	11	0.98433491	1	8.75	7.06601524					2.7E+08	2.7E+08	0.19948	ID=promoter_03625;
1	3E+08	1_267634509	1	22	0	6	12	3	0.030625	0.98374	0	6	12	0	3	6	0.53745121	1	5.875	7.79079127								
1	3E+08	1_267696048	6	41	11	7	24	9	0.00876	0.36763	20	0	6	10	10	8	0.95278883	1	12.625	12.8055513	3E+08	2.7E+08	ID=ENSP00000363241;ZNF618;;	ZNF618				
1	3E+08	1_267735468	13	13	14	47	13	31	0.09038	1	57	33	23	20	45	22	0.04373955	1	27.5	16.7332005	3E+08	2.7E+08	ID=ENSP00000265132;AMBP;NM_001633;	AMBP				
1	3E+08	1_267785385	23	21	28	32	22	30	0.865661	1	11	16	14	36	14	25	0.13553211	1	22.625	8.87914893								
1	3E+08	1_267821536	31	18	60	45	25	53	0.046724	1	38	34	23	3	36	13	0.01036973	0.4053038	31.5	17.3616984								
1	3E+08	1_267829779	117	49	151	147	83	149	0.007442	0.3212	40	62	61	1	51	31	0.17497609	1	78.5	53.9417675								

1	3E+08	1_267834064	4	6	7	6	5	7	0.955252	1	11	0	7	24	6	16	0.069189	1	8.125	7.12014245								
1	3E+08	1_267959997	53	23	42	2	38	22	0.026787	0.89052	128	30	54	15	79	35	9.91E-04	0.0620332	43.375	38.6926996								
1	3E+08	1_267968355	57	25	28	75	41	52	0.932362	1	171	128	111	50	150	81	3.82E-04	0.0282168	80.625	51.7216106								
1	3E+08	1_268023560	5	18	2	3	12	3	0.030625	0.98374	18	0	10	25	9	18	0.15524483	1	10.125	9.18753037								
1	3E+08	1_268201186	19	32	19	16	26	18	0.220252	1	38	16	1	32	27	17	0.45364599	1	21.625	11.8675729					2.7E+08	2.7E+08	0.21322	ID=promoter_03643;
1	3E+08	1_268746734	4	0	13	1	2	7	0.358464	1	39	13	2	5	26	4	6.99E-04	0.0468789	9.625	12.8944894								
1	3E+08	1_269116194	1373	14	57	32	694	45	########	#######	1392	32	62	14	712	38	1.41E-121	6.29E-118	372	623.956729								
1	3E+08	1_269430378	10	9	5	9	10	7	0.570701	1	25	14	7	7	20	7	0.06990458	1	10.75	6.34147347								
1	3E+08	1_269749643	689	179	492	393	434	443	0.119881	1	177	152	163	111	165	137	0.63442253	1	294.5	207.968404					2.7E+08	2.7E+08	0.49442	ID=promoter_03657;
1	3E+08	1_269875442	232	74	354	280	153	317	3.54E-08	#######	150	146	177	41	148	109	0.18839564	1	181.75	103.907583								
1	3E+08	1_270072195	46	10	17	13	28	15	0.042594	1	1	7	8	19	4	14	0.04184353	1	15.125	13.7262782								
1	3E+08	1_270135972	8	18	1	3	13	2	0.006819	0.29841	18	21	6	1	20	4	0.00863789	0.3495868	9.5	8.26351707								
1	3E+08	1_270334589	537	290	636	338	414	487	0.999077	1	266	196	230	79	231	155	0.00753492	0.3151311	321.5	182.296462					2.7E+08	2.7E+08	0.36752	ID=promoter_03662;
1	3E+08	1_270524146	9	18	80	30	14	55	1.02E-04	0.00906	0	3	16	1	2	9	0.08177705	1	19.625	26.430164								
1	3E+08	1_270617101	223	128	138	166	176	152	0.021244	0.74019	156	106	44	9	131	27	5.98E-14	2.84E-11	121.25	68.3368756								
1	3E+08	1_270691185	3	8	11	4	6	8	0.9722	1	0	6	8	19	3	14	0.01844097	0.6375434	7.375	5.80486249								
1	3E+08	1_271157583	157	114	109	99	136	104	0.004189	0.20359	0	38	104	5	19	55	3.76E-05	0.0039592	78.25	56.8550789								
1	3E+08	1_271351920	31	18	20	25	25	23	0.701069	1	27	21	9	9	24	9	0.0567576	1	20	7.94624799								
1	3E+08	1_272271927	26	114	18	29	70	24	2.24E-07	#######	52	88	83	27	70	55	0.6555371	1	54.625	35.9202987								
1	3E+08	1_273017489	18	0	21	11	9	16	0.603365	1	36	3	6	1	20	4	0.00863789	0.3495868	12	12.3981565								
1	3E+08	1_273453316	13	70	24	34	42	29	0.084977	1	40	35	41	30	38	36	0.99566183	1	35.875	16.5221713								
1	3E+08	1_273553589	17	15	33	29	16	31	0.257385	1	46	8	34	0	27	17	0.45364599	1	22.75	15.3040611								
1	3E+08	1_273906226	32	21	7	8	27	8	0.001088	0.0681	2	17	24	30	10	27	0.01023344	0.4008669	17.625	11.0961705								
1	3E+08	1_273939579	13	37	14	34	25	24	0.781475	1	0	32	32	30	16	31	0.04981266	1	24	13.2557265								
1	3E+08	1_274005770	0	7	14	8	4	11	0.322758	1	29	44	13	0	37	7	1.08E-04	0.0098158	14.375	15.1274349	3E+08	2.7E+08	ID=ENSP00003438 18;CDK5RAP2;NM_018249;	CDK5RAP2				
1	3E+08	1_274330224	506	195	423	408	351	416	0.989809	1	232	169	105	45	201	75	3.02E-11	1.08E-08	260.38	165.843246	3E+08	2.7E+08	ID=ENSP0000363040;MEGF9;;	MEGF9	2.7E+08	2.7E+08	0.54753	ID=promoter_03686;
1	3E+08	1_274664165	125	25	50	47	75	49	0.005401	0.25031	0	0	0	7	0	4	0.11562589	1	31.75	43.0008306								
1	3E+08	1_275188509	18	22	10	5	20	8	0.026297	0.87605	13	21	42	12	17	27	0.21252244	1	17.875	11.3066036								
1	3E+08	1_275206897	47	77	58	22	62	40	0.01139	0.45198	36	30	59	51	33	55	0.02595995	0.8395553	47.5	17.7844234								
1	3E+08	1_275225329	6	19	6	22	13	14	0.975695	1	14	12	8	17	13	13	0.98151319	1	13	6.07100839								
1	3E+08	1_275234306	4	4	4	0	4	2	0.597627	1	0	9	16	19	5	18	0.01516798	0.5480081	7	7.11135912								
1	3E+08	1_275263540	82	28	78	80	55	79	0.512893	1	32	0	10	2	16	6	0.14768941	1	39	35.7611122								
1	3E+08	1_275678550	1221	557	1073	852	889	963	0.231282	1	454	250	413	167	352	290	0.33015757	1	623.38	384.82387								
1	3E+08	1_275710933	66	42	101	41	54	71	0.818632	1	0	17	42	1	9	22	0.0389747	1	38.75	33.8979561								

1	3E+08	1_275719651	71	93	86	57	82	72	0.19816	1	110	33	63	53	72	58	0.72801134	1	70.75	24.6620008								
1	3E+08	1_275743877	9	23	1	11	16	6	0.042495	1	4	46	23	18	25	21	0.94599092	1	16.875	14.3371396								
1	3E+08	1_275929740	6	12	0	0	9	0	0.005101	0.2381	2	37	0	3	20	2	0.00122165	0.0738454	7.5	12.6038543								
1	3E+08	1_277396480	125	563	172	236	344	204	3.59E-14	#######	451	274	583	296	363	440	7.52E-04	0.0497787	337.5	174.276463								
1	3E+08	1_278247087	1	24	0	0	13	0	4.88E-04	0.03443	0	2	3	31	1	17	4.35E-04	0.0314643	7.625	12.4549187	3E+08	2.8E+08	ID=ENSP000002594 67;PDCL;NM_00538 8;	PDCL	2.8E+08	2.8E+08	0.17497	ID=promoter_03739;
1	3E+08	1_278271146	8	11	0	1	10	1	0.011548	0.45484	0	18	7	2	9	5	0.6500913	1	5.875	6.40172968								
1	3E+08	1_278551204	84	15	50	88	50	69	0.681201	1	116	39	19	45	78	32	3.47E-04	0.0262008	57	35.6490833	3E+08	2.8E+08	ID=ENSP000003627 47;RABGAP1;;	RABGAP1				
1	3E+08	1_278653005	4	23	7	4	14	6	0.098789	1	55	5	3	2	30	3	4.27E-05	0.004447	12.875	18.3103211	3E+08	2.8E+08	ID=ENSP000003627 41;STRBP;NM_0183 87;	STRBP				
1	3E+08	1_278831424	14	31	6	10	23	8	0.007066	0.30636	73	48	15	29	61	22	4.28E-04	0.0312589	28.25	22.7014789	3E+08	2.8E+08	ID=ENSP000003627 34;CRB2;NM_17368 9;	CRB2	2.8E+08	2.8E+08	0.30649	ID=promoter_03752;
1	3E+08	1_278838030	0	26	3	0	13	2	0.006819	0.29841	9	0	25	12	5	19	0.0096847	0.3824351	9.375	10.9013433	3E+08	2.8E+08	ID=ENSP000003627 34;CRB2;NM_17368 9;	CRB2	2.8E+08	2.8E+08	0.62582	ID=promoter_03753;
1	3E+08	1_278838077	264	99	241	229	182	235	0.622809	1	72	79	36	31	76	34	0.00147918	0.0866643	131.38	96.8252292	3E+08	2.8E+08	ID=ENSP000003627 34;CRB2;NM_17368 9;	CRB2	2.8E+08	2.8E+08	0.62582	ID=promoter_03753;
1	3E+08	1_278989914	3	48	24	33	26	29	0.982786	1	4	26	15	16	15	16	0.92652531	1	21.125	15.0279502								
1	3E+08	1_279019521	31	20	37	21	26	29	0.982786	1	0	4	0	0	2	0	0.39572182	1	14.125	15.0754057								
1	3E+08	1_279129854	1	6	11	1	4	6	0.929647	1	16	26	4	12	21	8	0.08664698	1	9.625	8.53459347								
1	3E+08	1_279206239	14	43	0	0	29	0	4.11E-08	#######	1	15	12	0	8	6	0.92562801	1	10.625	14.6963309								
1	3E+08	1_279538553	992	379	1287	1125	686	1206	1.73E-16	#######	431	311	302	136	371	219	6.36E-07	0.0001109	620.38	441.257922					2.8E+08	2.8E+08	0.59009	ID=promoter_03782;
1	3E+08	1_279657180	337	173	310	230	255	270	0.50272	1	87	57	49	47	72	48	0.2060485	1	161.25	119.471156					2.8E+08	2.8E+08	0.1648	ID=promoter_03784;
1	3E+08	1_279885409	2	5	8	6	4	7	0.813458	1	3	11	14	16	7	15	0.16935974	1	8.125	5.11126208								
1	3E+08	1_279901837	0	7	1	4	4	3	0.840045	1	9	3	5	11	6	8	0.79537884	1	5	3.81725406								
1	3E+08	1_279981721	193	222	54	76	208	65	1.90E-22	#######	185	94	70	22	140	46	1.44E-09	4.08E-07	114.5	74.4388531								
1	3E+08	1_280119781	204	84	166	150	144	158	0.845467	1	120	77	118	2	99	60	0.03253722	0.9928954	115.13	62.05398					2.8E+08	2.8E+08	0.22608	ID=promoter_03809;
1	3E+08	1_280265947	32	52	12	28	42	20	0.002773	0.14678	41	6	26	25	24	26	0.85918702	1	27.75	14.7042268								
1	3E+08	1_280397804	28	21	7	22	25	15	0.11231	1	54	58	24	30	56	27	0.01771021	0.6168629	30.5	17.2046505								
1	3E+08	1_280527442	3	14	2	12	9	7	0.713906	1	15	24	20	1	20	11	0.37181688	1	11.375	8.61788008	3E+08	2.8E+08	ID=ENSP000003626 56;GOLGA1;NM_002 077;	GOLGA1				
1	3E+08	1_280798623	10	26	5	12	18	9	0.098614	1	18	8	6	11	13	9	0.79589064	1	12	6.94879229								
1	3E+08	1_280869655	93	57	82	120	75	101	0.662912	1	19	24	53	14	22	34	0.16818144	1	57.75	38.3284676								
1	3E+08	1_281175356	102	106	85	37	104	61	6.71E-05	0.00628	40	53	86	113	47	100	8.44E-06	0.0010945	77.75	30.3491586								

1	3E+08	1_281365318	15	6	7	4	11	6	0.3028	1	20	2	17	2	11	10	0.99899888	1	9.125	7.14017807	3E+08	2.8E+08	ID=ENSP00000384751;FAM125B;NM_033446;	FAM125B				
1	3E+08	1_281365557	18	63	28	46	41	37	0.510548	1	7	10	18	1	9	10	0.92505409	1	23.875	21.0674936	3E+08	2.8E+08	ID=ENSP00000384751;FAM125B;NM_033446;	FAM125B				
1	3E+08	1_281394283	10	28	0	5	19	3	7.41E-04	0.04898	18	12	27	1	15	14	0.99983448	1	12.625	10.9013433	3E+08	2.8E+08	ID=ENSP00000384751;FAM125B;NM_033446;	FAM125B	2.8E+08	2.8E+08	0.30709	ID=promoter_03842;
1	3E+08	1_281500569	101	85	71	83	93	77	0.075676	1	79	32	51	48	56	50	0.98152393	1	68.75	23.0325856								
1	3E+08	1_281513387	0	5	0	0	3	0	0.172143	1	26	0	2	12	13	7	0.50249747	1	5.625	9.22632104					2.8E+08	2.8E+08	0.22788	ID=promoter_03864;
1	3E+08	1_281628024	0	0	9	0	0	5	0.118674	1	79	0	0	0	40	0	8.87E-09	2.21E-06	11	27.6560507								
1	3E+08	1_281628363	10	3	9	12	7	11	0.831726	1	58	0	17	15	29	16	0.24173971	1	15.5	18.0870909								
1	3E+08	1_281690257	9	61	20	35	35	28	0.315717	1	39	38	45	15	39	30	0.74371451	1	32.75	17.1610023					2.8E+08	2.8E+08	0.15744	ID=promoter_03882;
1	3E+08	1_281849801	13	18	5	3	16	4	0.009581	0.39335	16	29	35	5	23	20	0.9783171	1	15.5	11.6619038								
1	3E+08	1_281867402	36	14	31	36	25	34	0.853863	1	16	8	15	48	12	32	0.00478712	0.2203477	25.5	14.1219181					2.8E+08	2.8E+08	0.12677	ID=promoter_03887;
1	3E+08	1_281962133	1	6	24	10	4	17	0.046186	1	43	45	25	66	44	46	0.84986922	1	27.5	22.4181049	3E+08	2.8E+08	ID=ENSP00000259351;RALGPS1;NM_014636;	RALGPS1				
1	3E+08	1_282022321	3	7	0	3	5	2	0.402696	1	28	9	9	0	19	5	0.02799778	0.8875672	7.375	9.08589959	3E+08	2.8E+08	ID=ENSP00000259351;RALGPS1;NM_014636;	RALGPS1	2.8E+08	2.8E+08	0.12988	ID=promoter_03910;
1	3E+08	1_282022364	1	19	3	25	10	14	0.912507	1	0	12	28	3	6	16	0.069189	1	11.375	11.32554	3E+08	2.8E+08	ID=ENSP00000259351;RALGPS1;NM_014636;	RALGPS1	2.8E+08	2.8E+08	0.12988	ID=promoter_03910;
1	3E+08	1_282025387	122	41	156	133	82	145	0.011387	0.45198	0	30	28	11	15	20	0.56420259	1	65.125	61.487368	3E+08	2.8E+08	ID=ENSP00000259351;RALGPS1;NM_014636;	RALGPS1				
1	3E+08	1_282091607	18	15	2	7	17	5	0.013234	0.50759	11	0	9	63	6	36	6.81E-06	0.0009075	15.625	20.0708566	3E+08	2.8E+08	ID=ENSP00000259351;RALGPS1;NM_014636;	RALGPS1				
1	3E+08	1_282114039	13	1	6	24	7	15	0.409369	1	29	7	6	3	18	5	0.0406475	1	11.125	10.1901563								
1	3E+08	1_282169637	28	53	46	73	41	60	0.549601	1	49	21	38	72	35	55	0.04744077	1	47.5	18.7388062	3E+08	2.8E+08	ID=ENSP00000362485;GARNL3;NM_032293;	GARNL3				
1	3E+08	1_282266416	28	1	14	11	15	13	0.725173	1	0	2	1	1	1	1	0.99856566	1	7.25	9.91031209	3E+08	2.8E+08	ID=ENSP00000362485;GARNL3;NM_032293;	GARNL3				
1	3E+08	1_282268957	8	20	14	12	14	13	0.831264	1	22	14	21	32	18	27	0.27948435	1	17.875	7.49166203	3E+08	2.8E+08	ID=ENSP00000362485;GARNL3;NM_032293;	GARNL3	2.8E+08	2.8E+08	0.13619	ID=promoter_03931;

1	3E+08	1_282320404	8	24	6	6	16	6	0.042495	1	17	4	12	16	11	14	0.73202476	1	11.625	6.92691438	3E+08	2.8E+08	ID=ENSP0000362419;LRSAM1;;	LRSAM1				
1	3E+08	1_282345830	30	106	27	36	68	32	5.98E-05	0.00568	2	101	50	20	52	35	0.33918381	1	46.5	37.7359245	3E+08	2.8E+08	ID=ENSP0000362419;LRSAM1;;	LRSAM1				
1	3E+08	1_282363334	9	0	4	19	5	12	0.389128	1	0	0	5	5	0	5	0.06742467	1	5.25	6.40869944	3E+08	2.8E+08	ID=ENSP0000362411;FAM129B;NM_001035534;	FAM129B	2.8E+08	2.8E+08	0.29413	ID=promoter_03941;
1	3E+08	1_282367327	126	38	81	84	82	83	0.638308	1	13	16	20	16	15	18	0.76034467	1	49.25	42.4491292	3E+08	2.8E+08	ID=ENSP0000362411;FAM129B;NM_001035534;	FAM129B				
1	3E+08	1_282532839	14	31	4	7	23	6	0.001551	0.09145	0	62	48	38	31	43	0.22708292	1	25.5	22.7156334	3E+08	2.8E+08	ID=ENSP0000362399;STXBP1;NM_003165;	STXBP1				
1	3E+08	1_282537936	11	9	19	35	10	27	0.068117	1	64	2	12	2	33	7	5.50E-04	0.0381958	19.25	20.9472126	3E+08	2.8E+08	ID=ENSP0000362399;STXBP1;NM_003165;	STXBP1				
1	3E+08	1_282566649	750	296	987	575	523	781	1.03E-04	0.00914	195	189	308	86	192	197	0.60596869	1	423.25	315.728523					2.8E+08	2.8E+08	0.46567	ID=promoter_03966;
1	3E+08	1_282650296	8	21	8	7	15	8	0.181584	1	26	2	54	24	14	39	0.00101734	0.0634872	18.75	16.8077363								
1	3E+08	1_282978555	154	138	59	106	146	83	5.06E-07	#######	72	31	33	2	52	18	8.85E-04	0.0572814	74.375	54.065932	3E+08	2.8E+08	ID=ENSP0000300434;FAM102A;NM_001035254;	FAM102A				
1	3E+08	1_283096253	448	141	236	169	295	203	2.49E-08	#######	151	95	81	27	123	54	1.70E-05	0.0020144	168.5	129.184254					2.8E+08	2.8E+08	0.201	ID=promoter_04012;
1	3E+08	1_283194995	440	122	465	456	281	461	4.92E-05	0.00477	513	212	381	216	363	299	0.31767021	1	350.63	145.93143	3E+08	2.8E+08	ID=ENSP0000362157;SLC25A25;;	SLC25A25	2.8E+08	2.8E+08	0.19907	ID=promoter_04016;
1	3E+08	1_283220810	974	672	1100	1444	823	1272	4.90E-09	#######	779	456	1135	318	618	727	7.33E-05	0.0070383	859.75	375.340131					2.8E+08	2.8E+08	0.5109	ID=promoter_04021;
1	3E+08	1_283260103	22	42	13	16	32	15	0.009996	0.40823	44	36	45	44	40	45	0.67176911	1	32.75	13.5514891	3E+08	2.8E+08	ID=ENSP0000377232;CIZ1;NM_012127;	CIZ1				
1	3E+08	1_283609986	39	147	43	18	93	31	7.89E-10	#######	70	69	73	12	70	43	0.10149677	1	58.875	42.6059603								
1	3E+08	1_283620075	11	17	19	9	14	14	0.914609	1	21	23	5	0	22	3	0.001462	0.085933	13.125	8.1842619								
1	3E+08	1_283646420	5	30	19	25	18	22	0.991654	1	2	43	28	37	23	33	0.26888704	1	23.625	14.4017608					2.8E+08	2.8E+08	0.18912	ID=promoter_04055;
1	3E+08	1_283672030	4	5	7	14	5	11	0.495311	1	22	18	9	6	20	8	0.11762508	1	10.625	6.63190341					2.8E+08	2.8E+08	0.28252	ID=promoter_04060;
1	3E+08	1_283672154	40	15	36	46	28	41	0.662931	1	84	34	11	30	59	21	4.74E-04	0.0339584	37	22.4181049								
1	3E+08	1_283774473	484	189	387	471	337	429	0.531236	1	178	177	168	26	178	97	1.15E-04	0.0102689	260	165.917363					2.8E+08	2.8E+08	0.45298	ID=promoter_04073;
1	3E+08	1_283955971	0	0	6	11	0	9	0.02157	0.74463	53	12	48	32	33	40	0.5200223	1	20.25	21.2384422	3E+08	2.8E+08	ID=ENSP0000361658;NUP188;NM_015354;	NUP188	2.8E+08	2.8E+08	0.107	ID=promoter_04087;
1	3E+08	1_284038230	7	6	11	0	7	6	0.851931	1	15	0	20	7	8	14	0.34554715	1	8.25	6.9230464					2.8E+08	2.8E+08	0.16318	ID=promoter_04100;

1	3E+08	1_284128683	36	51	22	10	44	16	1.22E-04	0.01067	66	39	39	15	53	27	0.03678266	1	34.75	18.6834533					2.8E+08	2.8E+08	0.79816	ID=promoter_04109;
1	3E+08	1_284157120	18	124	16	22	71	19	3.17E-09	#######	68	90	99	23	79	61	0.56167202	1	57.5	43.1674481								
1	3E+08	1_284166577	17	38	38	15	28	27	0.767529	1	67	54	86	29	61	58	0.99051172	1	43	24.646066								
1	3E+08	1_284223075	5	3	0	0	4	0	0.09576	1	29	6	0	0	18	0	2.38E-04	0.0190698	5.375	9.85520167								
1	3E+08	1_284328402	1	6	5	2	4	4	0.97482	1	17	15	5	21	16	13	0.9394435	1	9	7.54036755								
1	3E+08	1_284347085	6	26	11	27	16	19	0.999341	1	0	0	5	0	0	3	0.19828566	1	9.375	11.2368971								
1	3E+08	1_284366222	510	188	495	424	349	460	0.258936	1	272	92	213	133	182	173	0.97257761	1	290.88	164.266715					2.8E+08	2.8E+08	0.24444	ID=promoter_04123;
1	3E+08	1_284432291	20	22	16	31	21	24	0.996223	1	12	23	32	28	18	30	0.13648184	1	23	7.07106781								
1	3E+08	1_284439866	17	25	27	17	21	22	0.933629	1	57	27	14	7	42	11	3.56E-04	0.0266828	23.875	15.0943462								
1	3E+08	1_284535321	109	71	56	62	90	59	0.002022	0.11403	28	31	22	32	30	27	0.99379101	1	51.375	29.3741261					2.8E+08	2.8E+08	0.20828	ID=promoter_04141;
1	3E+08	1_284539676	206	65	0	0	136	0	2.29E-35	#######	164	95	0	1	130	1	2.88E-26	2.71E-23	66.375	82.2347467					2.8E+08	2.8E+08	0.45968	ID=promoter_04142;
1	3E+08	1_284552732	6	3	8	9	5	9	0.74134	1	15	4	7	46	10	27	0.01023344	0.4008669	12.25	14.1193889								
1	3E+08	1_284570984	274	78	120	165	176	143	0.004617	0.21996	120	123	56	163	122	110	0.97791586	1	137.38	66.594589					2.8E+08	2.8E+08	0.18582	ID=promoter_04148;
1	3E+08	1_284578557	9	31	22	4	20	13	0.243396	1	20	19	1	19	20	10	0.26942782	1	15.625	10.0844647								
1	3E+08	1_284682394	99	14	3	0	57	2	6.32E-14	#######	39	14	6	2	27	4	4.55E-04	0.0327286	22.125	33.4810714	3E+08	2.8E+08	ID=ENSP00000259339-D3;TOR1B;NM_014506;	TOR1B	2.8E+08	2.8E+08	0.40886	ID=promoter_04155;
1	3E+08	1_284707080	11	19	9	3	15	6	0.065303	1	5	2	13	0	4	7	0.58783259	1	7.75	6.43095194					2.8E+08	2.8E+08	0.15766	ID=promoter_04162;
1	3E+08	1_284772328	6	16	10	10	11	10	0.842816	1	9	15	5	0	12	3	0.09316162	1	8.875	5.24914959					2.8E+08	2.8E+08	0.31436	ID=promoter_04174;
1	3E+08	1_284809694	18	10	51	39	14	45	0.002737	0.14543	40	46	23	20	43	22	0.0708179	1	30.875	14.951708	3E+08	2.8E+08	ID=ENSP00000342385;PTGES;NM_004878;	PTGES				
1	3E+08	1_284842542	0	6	6	3	3	5	0.88968	1	12	3	7	9	8	8	0.98858271	1	5.75	3.77018378	3E+08	2.8E+08	ID=ENSP00000361493;FNBP1;;	FNBP1				
1	3E+08	1_284873268	12	15	7	8	14	8	0.253058	1	6	13	15	1	10	8	0.95278883	1	9.625	4.95515604	3E+08	2.8E+08	ID=ENSP00000361493;FNBP1;;	FNBP1	2.8E+08	2.8E+08	0.28953	ID=promoter_04183;
1	3E+08	1_284933030	319	103	7	4	211	6	1.76E-50	#######	93	89	7	15	91	11	4.37E-13	1.89E-10	79.625	105.936554					2.8E+08	2.8E+08	0.45968	ID=promoter_04193;
1	3E+08	1_284937385	123	61	46	63	92	55	2.98E-04	0.02305	42	19	28	22	31	25	0.87406593	1	50.5	33.6494534					2.8E+08	2.8E+08	0.20828	ID=promoter_04194;
1	3E+08	1_284961916	337	151	257	228	244	243	0.195227	1	128	77	98	2	103	50	6.80E-04	0.0457983	159.75	108.340403	3E+08	2.8E+08	ID=ENSP00000259339;TOR1B;NM_014506;	TOR1B	2.8E+08	2.8E+08	0.39028	ID=promoter_04202;
1	3E+08	1_285134561	114	15	101	63	65	82	0.908335	1	18	17	30	24	18	27	0.27948435	1	47.75	40.0704736								
1	3E+08	1_285202541	35	10	14	27	23	21	0.707364	1	4	0	2	0	2	1	0.87709017	1	11.5	13.1583759								
1	3E+08	1_285222256	249	112	200	238	181	219	0.953351	1	129	63	81	44	96	63	0.1010226	1	139.5	79.8337558					2.9E+08	2.9E+08	0.34407	ID=promoter_04214;

1	3E+08	1_285249796	743	438	773	643	591	708	0.930786	1	529	279	274	192	404	233	4.31E-08	9.56E-06	483.88	224.05767					2.9E+08	2.9E+08	0.21661	ID=promoter_04219;
1	3E+08	1_285283962	177	92	51	88	135	70	8.94E-08	#######	54	69	50	21	62	36	0.07898377	1	75.25	46.9886004								
1	3E+08	1_285349947	198	92	87	190	145	139	0.234635	1	91	76	51	44	84	48	0.02609565	0.8426079	103.63	58.5855601								
1	3E+08	1_285356538	118	42	118	106	80	112	0.480715	1	53	42	46	15	48	31	0.28825568	1	67.5	40.1995025					2.9E+08	2.9E+08	0.58949	ID=promoter_04231;
1	3E+08	1_285443815	21	44	30	21	33	26	0.311396	1	32	34	28	22	33	25	0.74763976	1	29	7.9102104								
1	3E+08	1_285536456	10	25	16	19	18	18	0.89158	1	35	6	6	6	21	6	0.02669383	0.8538132	15.375	10.5821615								
1	3E+08	1_285547966	16	44	28	18	30	23	0.302742	1	31	15	22	1	23	12	0.26207533	1	21.875	12.7776311								
1	3E+08	1_285572774	303	47	155	168	175	162	0.093673	1	48	60	42	0	54	21	0.00230401	0.1234682	102.88	99.4763971								
1	3E+08	1_285706885	160	54	74	59	107	67	2.28E-04	0.01817	72	4	37	1	38	19	0.08276384	1	57.625	49.9683829								
1	3E+08	1_285778873	111	31	130	96	71	113	0.129208	1	29	43	28	6	36	17	0.06533483	1	59.25	46.0178537					2.9E+08	2.9E+08	0.38483	ID=promoter_04269;
1	3E+08	1_285833302	1194	680	1128	1286	937	1207	0.098611	1	612	742	830	345	677	588	0.51227336	1	852.13	324.766482					2.9E+08	2.9E+08	0.38483	ID=promoter_04271;
1	3E+08	1_285940251	12	41	8	24	27	16	0.087673	1	27	22	2	9	25	6	0.00589984	0.2600594	18.125	12.7328316								
1	3E+08	1_286000658	10	10	19	10	10	15	0.833288	1	13	3	1	2	8	2	0.20550608	1	8.5	6.164414					2.9E+08	2.9E+08	0.40815	ID=promoter_04295;
1	3E+08	1_286001497	0	13	3	1	7	2	0.160905	1	0	11	22	1	6	12	0.2888538	1	6.375	8.10533334					2.9E+08	2.9E+08	0.40815	ID=promoter_04295;
1	3E+08	1_286097426	662	341	492	469	502	481	0.006464	0.28906	189	116	127	271	153	199	0.00669335	0.2869955	333.38	195.003983	3E+08	2.9E+08	ID=ENSP00000361413;FIBCD1;NM_032843;	FIBCD1	2.9E+08	2.9E+08	0.29433	ID=promoter_04311;
1	3E+08	1_286203090	16	105	24	45	61	35	0.002653	0.14147	75	33	60	34	54	47	0.95121577	1	49	29.6262432					2.9E+08	2.9E+08	0.35109	ID=promoter_04319;
1	3E+08	1_286261272	16	32	31	15	24	23	0.786129	1	29	22	15	1	26	8	0.01635039	0.5814583	20.125	10.5348063					2.9E+08	2.9E+08	0.13562	ID=promoter_04324;
1	3E+08	1_286423822	2	14	2	3	8	3	0.206144	1	42	15	9	4	29	7	0.00267552	0.137714	11.375	13.4370224					2.9E+08	2.9E+08	0.10824	ID=promoter_04337;
1	3E+08	1_286447790	13	6	5	6	10	6	0.41703	1	5	5	3	1	5	2	0.58563225	1	5.5	3.46410162					2.9E+08	2.9E+08	0.17443	ID=promoter_04341;
1	3E+08	1_286451100	45	79	18	45	62	32	5.42E-04	0.03781	22	41	31	20	32	26	0.8825541	1	37.625	20.0566163					2.9E+08	2.9E+08	0.43921	ID=promoter_04343;
1	3E+08	1_286484193	25	0	20	7	13	14	0.975695	1	1	0	0	0	1	0	0.62906424	1	6.625	10.1691059								
1	3E+08	1_286510109	5	10	17	15	8	16	0.45954	1	296	255	388	280	276	334	0.00452515	0.2107253	158.25	161.191767								
1	3E+08	1_286532523	17	16	6	11	17	9	0.140533	1	7	9	17	6	8	12	0.56746184	1	11.125	4.8825491					2.9E+08	2.9E+08	0.17443	ID=promoter_04355;
1	3E+08	1_286534960	0	23	2	10	12	6	0.213448	1	11	4	7	0	8	4	0.59180275	1	7.125	7.67998326					2.9E+08	2.9E+08	0.17344	ID=promoter_04356;
1	3E+08	1_286633132	281	105	213	195	193	204	0.58403	1	1	22	0	2	12	1	0.01422424	0.5202299	102.38	113.404381								
1	3E+08	1_286734255	29	23	13	25	26	19	0.285911	1	71	37	96	54	54	75	0.07423494	1	43.5	28.2033433								
1	3E+08	1_286790093	0	0	10	0	0	5	0.118674	1	0	12	25	1	6	13	0.20797135	1	6	9.11827051					2.9E+08	2.9E+08	0.19651	ID=promoter_04372;
1	3E+08	1_286793479	6	16	29	27	11	28	0.082798	1	20	4	27	18	12	23	0.11381058	1	18.375	9.48589178					2.9E+08	2.9E+08	0.2704	ID=promoter_04374;
1	3E+08	1_286811027	27	9	34	8	18	21	0.99986	1	0	16	38	22	8	30	7.18E-04	0.0480178	19.25	13.3603892					2.9E+08	2.9E+08	0.15695	ID=promoter_04379;

1	3E+08	1_286819706	15	15	9	4	15	7	0.113635	1	14	14	6	7	14	7	0.39930956	1	10.5	4.50396651								
1	3E+08	1_286904591	149	52	74	57	101	66	8.88E-04	0.05748	117	76	46	16	97	31	4.43E-07	8.03E-05	73.375	42.0813413								
1	3E+08	1_286935411	9	15	57	31	12	44	0.001096	0.06846	16	3	8	5	10	7	0.84912739	1	18	18.0396389					2.9E+08	2.9E+08	0.22255	ID=promoter_04407;
1	3E+08	1_286994009	643	458	657	875	551	766	0.009679	0.39691	410	231	318	285	321	302	0.98323695	1	484.63	222.134411								
1	3E+08	1_286998140	329	148	313	394	239	354	0.020227	0.7141	2	34	82	5	18	44	0.00151903	0.0882005	163.38	159.341446					2.9E+08	2.9E+08	0.14435	ID=promoter_04424;
1	3E+08	1_287077034	33	53	46	32	43	39	0.506623	1	7	5	2	1	6	2	0.42506085	1	22.375	21.0844391								
1	3E+08	1_287089924	10	32	14	17	21	16	0.42382	1	4	9	13	2	7	8	0.92121674	1	12.625	9.28804916					2.9E+08	2.9E+08	0.42622	ID=promoter_04443;
1	3E+08	1_287091181	22	35	22	36	29	29	0.831194	1	16	4	8	3	10	6	0.69729498	1	18.25	12.9256113					2.9E+08	2.9E+08	0.42622	ID=promoter_04443;
1	3E+08	1_287183556	23	35	21	289	29	155	1.88E-15	#######	17	45	80	70	31	75	1.87E-05	0.0021814	72.5	90.4591463	3E+08	2.9E+08	ID=ENSP0000292035;MED27;NM_004269;	MED27	2.9E+08	2.9E+08	0.15857	ID=promoter_04458;
1	3E+08	1_287292924	26	110	39	43	68	41	0.002807	0.14801	116	139	145	75	128	110	0.84365141	1	86.625	47.1197183	3E+08	2.9E+08	ID=ENSP0000292035;MED27;NM_004269;	MED27	2.9E+08	2.9E+08	0.3215	ID=promoter_04482;
1	3E+08	1_287398828	68	26	39	35	47	37	0.188658	1	99	98	142	76	99	109	0.46341	1	72.875	39.4694728								
1	3E+08	1_287436838	31	0	7	3	16	5	0.021336	0.74103	30	30	6	0	30	3	4.27E-05	0.004447	13.375	14.2622128								
1	3E+08	1_287524373	61	10	44	26	36	35	0.730868	1	0	0	0	1	0	1	0.58312783	1	17.75	23.6446913					2.9E+08	2.9E+08	0.21588	ID=promoter_04510;
1	3E+08	1_287594819	41	8	10	22	25	16	0.157912	1	0	40	8	0	20	4	0.00863789	0.3495868	16.125	16.5308154								
1	3E+08	1_287612373	28	0	11	0	14	6	0.098789	1	0	0	11	0	0	6	0.0393172	1	6.25	10.0959681								
1	3E+08	1_287634131	44	18	24	16	31	20	0.106722	1	27	21	31	20	24	26	0.85918702	1	25.125	9.04650682	3E+08	2.9E+08	ID=ENSP0000353888;NTNG2;;	NTNG2				
1	3E+08	1_287709447	4	28	9	6	16	8	0.127562	1	22	29	1	1	26	1	2.34E-05	0.002637	12.5	11.9163753								
1	3E+08	1_287715992	29	34	32	32	32	32	0.815447	1	12	27	25	38	20	32	0.15276046	1	28.625	7.85470741					2.9E+08	2.9E+08	0.32692	ID=promoter_04535;
1	3E+08	1_287811592	12	446	810	610	229	710	3.88E-39	#######	56	359	352	91	208	222	0.34382321	1	342	281.767381								
1	3E+08	1_287812477	4	18	10	14	11	12	0.985018	1	27	0	12	13	14	13	0.99999086	1	12.25	8.25919401					2.9E+08	2.9E+08	0.10835	ID=promoter_04542;
1	3E+08	1_287821835	2	5	0	7	4	4	0.97482	1	1	4	0	30	3	15	0.01140593	0.4370832	6.125	9.96332561	3E+08	2.9E+08	ID=ENSP0000384576;;					
1	3E+08	1_287944858	20	23	11	12	22	12	0.091688	1	7	13	2	0	10	1	0.03495212	1	11	8								
1	3E+08	1_288073509	13	46	13	15	30	14	0.012913	0.49818	67	17	28	48	42	38	0.99411291	1	30.875	20.4201406					2.9E+08	2.9E+08	0.87064	ID=promoter_04593;
1	3E+08	1_288134637	12	63	66	57	38	62	0.273978	1	38	43	44	25	41	35	0.93777492	1	43.5	18.6470986								
1	3E+08	1_288166038	34	27	16	6	31	11	0.001462	0.08667	18	32	16	13	25	15	0.40601431	1	20.25	9.77971662								
1	3E+08	1_288167882	33	42	24	20	38	22	0.026787	0.89052	48	24	55	16	36	36	0.94963899	1	32.75	14.1900367								
1	3E+08	1_288170572	446	315	547	536	381	542	0.015341	0.57367	238	212	262	20	225	141	0.00119319	0.0728378	322	179.605122								
1	3E+08	1_288173792	14	35	2	8	25	5	2.23E-04	0.01779	31	16	13	6	24	10	0.09254106	1	15.625	11.6978325					2.9E+08	2.9E+08	0.19188	ID=promoter_04616;
1	3E+08	1_288192251	30	109	30	46	70	38	4.63E-04	0.0332	132	124	129	80	128	105	0.64918773	1	85	44.4618617								

1	3E+08	1_288225932	16	37	7	28	27	18	0.170316	1	43	37	32	20	40	26	0.36657272	1	27.5	12.2474487								
1	3E+08	1_288226261	5	10	9	16	8	13	0.766846	1	19	2	10	3	11	7	0.73613127	1	9.25	5.99404466								
1	3E+08	1_288232595	11	5	27	5	8	16	0.45954	1	8	0	1	26	4	14	0.04184353	1	10.375	10.5551274								
1	3E+08	1_288233388	17	29	11	2	23	7	0.003444	0.1741	0	12	5	13	6	9	0.65381141	1	11.125	9.2803556					2.9E+08	2.9E+08	0.31158	ID=promoter_04631;
1	3E+08	1_288248575	45	128	40	50	87	45	2.73E-05	0.00286	126	119	153	79	123	116	0.99124837	1	92.5	44.313816								
1	3E+08	1_288270157	90	21	23	30	56	27	4.59E-04	0.03297	61	19	20	22	40	21	0.10160257	1	35.75	25.9436202	3E+08	2.9E+08	ID=ENSP000003842 83;;					
1	3E+08	1_288298176	0	0	1	0	0	1	0.652937	1	0	9	11	25	5	18	0.01516798	0.5480081	5.75	8.97217922								
1	3E+08	1_288356769	13	21	10	5	17	8	0.087954	1	7	30	15	5	19	10	0.34022508	1	13.25	8.69729022								
1	3E+08	1_288368750	9	32	7	32	21	20	0.800056	1	1	21	6	20	11	13	0.83819512	1	16	12					2.9E+08	2.9E+08	0.14529	ID=promoter_04665;
1	3E+08	1_288511588	634	203	410	267	419	339	2.10E-06	0.0003	118	87	127	12	103	70	0.1320029	1	232.25	203.118368								
1	3E+08	1_288530323	0	5	1	0	3	1	0.508575	1	0	0	22	12	0	17	1.04E-04	0.0094884	5	8.05339325	3E+08	2.9E+08	ID=ENSP000003767 76;DBH;NM_000787 ;	DBH	2.9E+08	2.9E+08	0.16465	ID=promoter_04691;
1	3E+08	1_288540881	47	72	38	45	60	42	0.03455	1	25	31	12	32	28	22	0.84457634	1	37.75	17.7904469					2.9E+08	2.9E+08	0.21646	ID=promoter_04693;
1	3E+08	1_288568846	11	26	28	6	19	17	0.718296	1	62	2	1	4	32	3	1.74E-05	0.0020499	17.5	20.8120844	3E+08	2.9E+08	ID=ENSP000003609 16;VAV2;NM_00113 4398;	VAV2				
1	3E+08	1_288579406	6	53	2	11	30	7	1.06E-04	0.00938	16	2	16	2	9	9	0.98716474	1	13.5	17.0377732	3E+08	2.9E+08	ID=ENSP000003609 16;VAV2;NM_00113 4398;	VAV2				
1	3E+08	1_288661516	109	77	66	97	93	82	0.167479	1	97	14	22	54	56	38	0.3292632	1	67	35.1892841	3E+08	2.9E+08	ID=ENSP000003514 46;WDR5;NM_05282 1;	WDR5	2.9E+08	2.9E+08	0.33187	ID=promoter_04729;
1	3E+08	1_288753852	34	0	317	321	17	319	5.28E-50	#######	27	14	344	97	21	221	4.87E-40	7.10E-37	144.25	154.407392					2.9E+08	2.9E+08	0.24074	ID=promoter_04750;
1	3E+08	1_288773547	1380	737	1407	1032	1059	1220	0.912254	1	676	446	601	142	561	372	3.13E-06	0.0004579	802.63	443.338612					2.9E+08	2.9E+08	0.22718	ID=promoter_04756;
1	3E+08	1_288774789	59	27	84	57	43	71	0.205751	1	40	55	20	2	48	11	3.51E-05	0.0037401	43	26.0548871								
1	3E+08	1_288838418	42	49	42	29	46	36	0.18711	1	34	63	39	2	49	21	0.01008507	0.3959241	37.5	17.6068169	3E+08	2.9E+08	ID=ENSP000003487 49;RXRA;NM_00295 7;	RXRA				
1	3E+08	1_288852793	1	10	0	2	6	1	0.108618	1	11	1	5	16	6	11	0.39136385	1	5.75	5.89794153	3E+08	2.9E+08	ID=ENSP000003487 49;RXRA;NM_00295 7;	RXRA				
1	3E+08	1_288858703	2	19	12	31	11	22	0.343316	1	31	12	22	20	22	21	0.99541892	1	18.625	9.88415037	3E+08	2.9E+08	ID=ENSP000003487 49;RXRA;NM_00295 7;	RXRA	2.9E+08	2.9E+08	0.26352	ID=promoter_04777;
1	3E+08	1_288864830	25	17	33	29	21	31	0.717186	1	1	4	7	14	3	11	0.0740992	1	16.25	11.9133778								
1	3E+08	1_288871214	4	36	0	3	20	2	1.33E-04	0.01141	0	1	5	1	1	3	0.56022579	1	6.25	12.162589					2.9E+08	2.9E+08	0.27647	ID=promoter_04781;
1	3E+08	1_288920363	6	4	15	5	5	10	0.615109	1	16	4	6	0	10	3	0.19568363	1	7	5.58057857								

1	3E+08	1_288999877	530	300	329	413	415	371	6.96E-04	0.04677	342	247	433	95	295	264	0.91697691	1	336.13	131.125281								
1	3E+08	1_289001119	6	10	7	4	8	6	0.705675	1	1	11	1	14	6	8	0.79537884	1	6.75	4.71320334					2.9E+08	2.9E+08	0.22615	ID=promoter_04814;
1	3E+08	1_289002670	340	140	293	297	240	295	0.865005	1	85	95	127	47	90	87	0.96199102	1	178	113.624696								
1	3E+08	1_289020815	191	123	469	481	157	475	1.66E-25	#######	93	85	443	105	89	274	2.18E-24	1.85E-21	248.75	181.68556					2.9E+08	2.9E+08	0.24074	ID=promoter_04820;
1	3E+08	1_289031028	23	14	29	39	19	34	0.331673	1	12	37	63	6	25	35	0.2845844	1	27.875	18.4734365					2.9E+08	2.9E+08	0.19987	ID=promoter_04821;
1	3E+08	1_289047296	0	2	39	38	1	39	2.39E-07	#######	16	1	26	11	9	19	0.11219606	1	16.625	16.088483					2.9E+08	2.9E+08	0.49136	ID=promoter_04824;
1	3E+08	1_289056782	3	43	4	9	23	7	0.003444	0.1741	3	14	5	5	9	5	0.6500913	1	10.75	13.5514891					2.9E+08	2.9E+08	0.32696	ID=promoter_04828;
1	3E+08	1_289096606	119	231	128	67	175	98	1.58E-08	#######	185	201	64	45	193	55	3.13E-15	1.61E-12	130	69.6337357	3E+08	2.9E+08	ID=ENSP0000360900;BRD3;;	BRD3	2.9E+08	2.9E+08	0.70785	ID=promoter_04842;
1	3E+08	1_289106477	30	18	34	26	24	30	0.97337	1	10	17	6	8	14	7	0.39930956	1	18.625	10.4872371					2.9E+08	2.9E+08	0.51531	ID=promoter_04844;
1	3E+08	1_289139623	30	27	44	45	29	45	0.499058	1	0	6	10	26	3	18	0.00260948	0.1361302	23.5	16.835337								
1	3E+08	1_289140063	36	11	34	25	24	30	0.97337	1	19	19	15	13	19	14	0.80779431	1	21.5	9.38083152					2.9E+08	2.9E+08	0.37433	ID=promoter_04848;
1	3E+08	1_289157070	167	186	167	185	177	176	0.299148	1	98	67	94	100	83	97	0.29963265	1	133	47.833043					2.9E+08	2.9E+08	0.35583	ID=promoter_04852;
1	3E+08	1_289192246	20	29	1	9	25	5	2.23E-04	0.01779	15	22	14	2	19	8	0.15782218	1	14	9.73946317								
1	3E+08	1_289206729	135	27	81	101	81	91	0.960946	1	49	40	28	36	45	32	0.51614664	1	62.125	39.4984177					2.9E+08	2.9E+08	0.34913	ID=promoter_04871;
1	3E+08	1_289247695	12	22	11	28	17	20	0.999959	1	7	15	14	22	11	18	0.32230807	1	16.375	6.98851098					2.9E+08	2.9E+08	0.47255	ID=promoter_04888;
1	3E+08	1_289252259	216	126	225	222	171	224	0.553126	1	57	43	53	22	50	38	0.65210426	1	120.5	88.3742044								
1	3E+08	1_289351995	21	20	9	9	21	9	0.03105	0.99404	0	4	0	0	2	0	0.39572182	1	7.875	8.64270956	3E+08	2.9E+08	ID=ENSP0000347458;COL5A1;;	COL5A1				
1	3E+08	1_289456063	5	24	11	0	15	6	0.065303	1	22	1	8	1	12	5	0.30420563	1	9	9.44154951					2.9E+08	2.9E+08	0.20425	ID=promoter_04949;
1	3E+08	1_289475968	18	8	4	0	13	2	0.006819	0.29841	0	10	12	16	5	14	0.08295177	1	8.5	6.82432624					2.9E+08	2.9E+08	0.16866	ID=promoter_04956;
1	3E+08	1_289476013	17	47	15	19	32	17	0.02552	0.85718	41	20	35	29	31	32	0.91294871	1	27.875	12.0408531					2.9E+08	2.9E+08	0.16866	ID=promoter_04956;
1	3E+08	1_289495583	29	3	8	13	16	11	0.385778	1	23	8	4	5	16	5	0.08348313	1	11.625	9.53096457								
1	3E+08	1_289522938	22	0	27	14	11	21	0.417506	1	0	0	0	0	0	0	1	1	7.875	11.4197511								
1	3E+08	1_289593184	28	2	6	20	15	13	0.725173	1	0	0	15	5	0	10	0.00454609	0.2107253	9.5	10.3647755								
1	3E+08	1_289609149	8	0	5	9	4	7	0.813458	1	0	15	5	17	8	11	0.69450319	1	7.375	6.254998								
1	3E+08	1_289610127	9	26	11	5	18	8	0.059652	1	19	0	15	35	10	25	0.022088	0.7420679	15	11.4267856					2.9E+08	2.9E+08	0.70291	ID=promoter_04993;
1	3E+08	1_289617564	173	47	117	101	110	109	0.458165	1	63	54	73	71	59	72	0.29145896	1	87.375	41.7610122					2.9E+08	2.9E+08	0.27624	ID=promoter_04995;
1	3E+08	1_289618181	28	21	33	46	25	40	0.473554	1	2	13	14	0	8	7	0.9937864	1	19.625	15.6655901					2.9E+08	2.9E+08	0.27624	ID=promoter_04995;
1	3E+08	1_289618368	58	41	42	116	50	79	0.254423	1	42	7	0	1	25	1	3.71E-05	0.0039152	38.375	38.3552474					2.9E+08	2.9E+08	0.27624	ID=promoter_04995;

1	3E+08	1_289656443	98	50	84	70	74	77	0.762583	1	89	88	71	39	89	55	0.05878087	1	73.625	20.4306318	2.9E+08	2.9E+08	0.56313	ID=promoter_05003;
1	3E+08	1_289658349	56	21	65	47	39	56	0.623837	1	40	26	38	40	33	39	0.58889164	1	41.625	14.4907606	2.9E+08	2.9E+08	0.77041	ID=promoter_05004;
1	3E+08	1_289671014	1	29	3	5	15	4	0.015825	0.58672	3	3	1	3	3	2	0.9363008	1	6	9.38083152	2.9E+08	2.9E+08	0.4741	ID=promoter_05007;
1	3E+08	1_289691486	25	4	25	15	15	20	0.931941	1	25	33	21	20	29	21	0.68832436	1	21	8.60232527	2.9E+08	2.9E+08	0.64105	ID=promoter_05013;
1	3E+08	1_289697044	9	6	16	9	8	13	0.766846	1	43	8	12	2	26	7	0.00838865	0.3419228	13.125	12.7440462	2.9E+08	2.9E+08	0.9795	ID=promoter_05014;
1	3E+08	1_289699135	10	86	36	39	48	38	0.190088	1	2	26	24	52	14	38	0.00151301	0.0879967	34.375	26.2403043	2.9E+08	2.9E+08	0.9795	ID=promoter_05014;
1	3E+08	1_289713130	100	38	59	70	69	65	0.445904	1	16	33	0	0	25	0	9.28E-06	0.0011901	39.5	35.2298575				
1	3E+08	1_289742837	117	119	178	146	118	162	0.428569	1	169	137	269	239	153	254	5.75E-08	1.25E-05	171.75	55.648771	2.9E+08	2.9E+08	0.40935	ID=promoter_05024;
1	3E+08	1_289762370	14	25	94	62	20	78	2.40E-06	0.00034	31	29	43	21	30	32	0.85844914	1	39.875	26.3841809				
1	3E+08	1_289782651	3	9	33	15	6	24	0.016769	0.61408	1	13	5	8	7	7	0.99000271	1	10.875	10.1198179	2.9E+08	2.9E+08	0.18228	ID=promoter_05032;
1	3E+08	1_289798705	5	5	8	2	5	5	0.968625	1	17	7	20	2	12	11	0.99963644	1	8.25	6.71352579				
1	3E+08	1_289813073	163	141	202	116	152	159	0.600511	1	0	12	29	0	6	15	0.10150651	1	82.875	81.7215438				
1	3E+08	1_289824426	15	18	27	8	17	18	0.955187	1	40	16	31	13	28	22	0.84457634	1	21	10.6904497	2.9E+08	2.9E+08	0.47939	ID=promoter_05045;
1	3E+08	1_289861510	8	8	12	16	8	14	0.661714	1	21	5	12	37	13	25	0.09257925	1	14.875	10.2600125	2.9E+08	2.9E+08	0.16409	ID=promoter_05054;
1	3E+08	1_289861958	140	32	136	91	86	114	0.69266	1	78	75	54	14	77	34	0.00112478	0.0691941	77.5	44.9952378				
1	3E+08	1_289894557	73	54	87	82	64	85	0.754159	1	87	22	16	51	55	34	0.1739869	1	59	28.2842712	2.9E+08	2.9E+08	0.38768	ID=promoter_05065;
1	3E+08	1_289914350	17	6	2	4	12	3	0.030625	0.98374	20	0	7	43	10	25	0.022088	0.7420679	12.375	14.2321317				
1	3E+08	1_289924781	11	21	8	4	16	6	0.042495	1	15	16	23	5	16	14	0.9876114	1	12.875	7.07990113				
1	3E+08	1_290020484	37	8	28	25	23	27	0.999996	1	1	43	19	1	22	10	0.16182597	1	20.25	15.9171965				
1	3E+08	1_290122311	560	497	570	544	529	557	0.205489	1	466	236	242	164	351	203	4.64E-07	8.37E-05	409.88	167.236733				
1	3E+08	1_290609520	5	51	148	154	28	151	3.64E-15	#######	23	23	56	92	23	74	1.99E-07	3.92E-05	69	57.0914054	2.9E+08	2.9E+08	0.27187	ID=promoter_05087;
1	3E+08	1_290824980	6	38	1	10	22	6	0.002551	0.13706	25	0	4	1	13	3	0.06317581	1	10.625	13.731481				
1	3E+08	1_290852448	731	219	433	351	475	392	1.50E-06	0.00022	169	160	218	43	165	131	0.41524678	1	290.5	214.28419	2.9E+08	2.9E+08	0.5094	ID=promoter_05094;
1	3E+08	1_290929782	3	21	14	34	12	24	0.311519	1	27	13	36	8	20	22	0.85756598	1	19.5	12.0593769				
1	3E+08	1_291036234	389	68	224	218	229	221	0.116848	1	142	66	73	45	104	59	0.00974064	0.3837738	153.13	118.285898	2.9E+08	2.9E+08	0.43275	ID=promoter_05099;
1	3E+08	1_291249786	4	15	1	4	10	3	0.082185	1	34	3	30	15	19	23	0.68897774	1	13.25	12.7811245				
1	3E+08	1_292102621	23	38	45	59	31	52	0.284785	1	68	45	57	81	57	69	0.32705107	1	52	18.1501673				
1	3E+08	1_292245835	13	21	1	8	17	5	0.013234	0.50759	9	12	2	7	11	5	0.40227598	1	9.125	6.40172968				
1	3E+08	1_292266062	29	14	11	13	22	12	0.091688	1	46	18	45	7	32	26	0.8825541	1	22.875	15.3756533				
1	3E+08	1_292382336	37	6	52	16	22	34	0.599769	1	68	22	20	13	45	17	0.00491937	0.2246966	29.25	21.3725458				
1	3E+08	1_292470971	16	16	29	54	16	42	0.01893	0.67421	45	23	37	21	34	29	0.94710596	1	30.125	14.0044636				

1	3E+08	1_293451925	42	47	35	56	45	46	0.806124	1	3	58	23	23	31	23	0.7197219	1	35.875	18.7802138								
1	3E+08	1_294095739	7	46	19	7	27	13	0.024366	0.82541	37	0	25	4	19	15	0.89728723	1	18.125	16.7198556								
1	3E+08	1_294108192	594	93	385	344	344	365	0.410245	1	101	92	102	34	97	68	0.20782266	1	218.13	199.15639					2.9E+08	2.9E+08	0.31922	ID=promoter_05132;
1	3E+08	1_294117787	42	48	79	62	45	71	0.295373	1	10	36	11	21	23	16	0.67590535	1	38.625	24.4244462	3E+08	2.9E+08	ID=ENSP0000360761;AGPAT2;NM_006412;	AGPAT2	2.9E+08	2.9E+08	0.35119	ID=promoter_05134;
1	3E+08	1_294127043	13	23	20	19	18	20	0.986196	1	68	21	51	0	45	26	0.15312477	1	26.875	21.8660696	3E+08	2.9E+08	ID=ENSP0000338013;TMEM141;NM_001039374;	TMEM141				
1	3E+08	1_294133497	1018	1106	3910	5172	1062	4541	0	0	942	684	7010	3349	813	5180	0	0	2898.9	2353.7373					2.9E+08	2.9E+08	0.9612	ID=promoter_05138;
1	3E+08	1_294174491	101	106	124	133	104	129	0.913955	1	101	80	194	9	91	102	0.41916818	1	106	52.0054942								
1	3E+08	1_294191533	75	39	67	75	57	71	0.944477	1	23	78	19	2	51	11	1.07E-05	0.0013461	47.25	30.1839598								
1	3E+08	1_294227882	11	53	11	7	32	9	2.11E-04	0.01699	2	11	24	2	7	13	0.3188207	1	15.125	16.796577								
1	3E+08	1_294239965	48	22	34	33	35	34	0.735392	1	42	62	43	22	52	33	0.23056449	1	38.25	13.4668906					2.9E+08	2.9E+08	0.23732	ID=promoter_05163;
1	3E+08	1_294248709	7	7	4	3	7	4	0.503048	1	24	10	21	7	17	14	0.9475482	1	10.375	7.81824789					2.9E+08	2.9E+08	0.1893	ID=promoter_05166;
1	3E+08	1_294261245	65	41	36	99	53	68	0.887025	1	0	4	11	2	2	7	0.20455692	1	32.25	35.503521					2.9E+08	2.9E+08	0.19733	ID=promoter_05169;
1	3E+08	1_294307822	108	27	73	65	68	69	0.697468	1	15	1	29	19	8	24	0.00970297	0.3824351	42.125	36.2114327					2.9E+08	2.9E+08	0.19986	ID=promoter_05178;
1	3E+08	1_294324177	247	149	215	192	198	204	0.429151	1	127	75	127	55	101	91	0.98073661	1	148.38	66.6396225	3E+08	2.9E+08	ID=ENSP0000350102;C9orf167;NM_017723;	C9orf167	2.9E+08	2.9E+08	0.73148	ID=promoter_05179;
1	3E+08	1_294365606	5	7	2	11	6	7	0.999953	1	35	22	16	12	29	14	0.12465658	1	13.75	10.6603404								
1	3E+08	1_294382206	16	10	15	1	13	8	0.344217	1	49	30	41	7	40	24	0.23641017	1	21.125	17.0833043								
1	3E+08	1_295272701	25	105	68	63	65	66	0.710817	1	56	51	42	33	54	38	0.426257	1	55.375	24.790191								
2	14936	2_14936	83	101	122	185	92	154	0.025205	0.84837	51	21	77	7	36	42	0.59776306	1	80.875	57.1875049								
2	55558	2_55558	2	10	20	14	6	17	0.158911	1	8	12	15	21	10	18	0.2304087	1	12.75	6.25071424					55543	65307	0.99008	ID=promoter_26423;
2	70701	2_70701	34	18	12	8	26	10	0.006911	0.3015	0	12	6	2	6	4	0.87665919	1	11.5	10.7835854								
2	77942	2_77942	34	16	22	10	25	16	0.157912	1	59	26	58	9	43	34	0.78602279	1	29.25	19.8332333								
2	85438	2_85438	11	28	13	23	20	18	0.715835	1	20	2	27	12	11	20	0.1879092	1	17	9.00793301					83756	87393	0.77198	ID=promoter_26430;
2	86175	2_86175	1485	601	1356	1267	1043	1312	0.241425	1	374	329	344	179	352	262	0.0264102	0.8480056	741.88	535.312576					83756	87393	0.77198	ID=promoter_26430;
2	117405	2_117405	65	47	97	77	56	87	0.258135	1	9	9	24	13	9	19	0.11219606	1	42.625	34.108807	116727	127086	ID=ENSP0000386899;TSPAN4;;	TSPAN4	115939	118749	0.50624	ID=promoter_26439;
2	133943	2_133943	1630	448	1990	1303	1039	1647	2.27E-13	#######	892	501	489	324	697	407	7.34E-13	3.10E-10	947.13	624.489377					133602	135760	0.65706	ID=promoter_26443;
2	177895	2_177895	664	132	580	434	398	507	0.467742	1	48	39	41	77	44	59	0.17614908	1	251.88	263.759326								
2	251926	2_251926	16	9	30	27	13	29	0.146708	1	0	14	4	3	7	4	0.73798468	1	12.875	11.0897314					251012	254271	0.65699	ID=promoter_26477;
2	261211	2_261211	7	13	7	5	10	6	0.41703	1	1	1	10	2	1	6	0.13767373	1	5.75	4.36708468					259959	262175	0.46556	ID=promoter_26479;

2	292018	2_292018	2769	1387	3701	2599	2078	3150	1.04E-18	#######	1475	882	877	536	1179	707	3.14E-19	2.09E-16	1778.3	1118.41224					289210	292603	0.53182	ID=promoter_26485;
2	314879	2_314879	78	73	175	209	76	192	4.92E-08	#######	52	33	92	38	43	65	0.04261438	1	93.75	64.4842173	295157	317949	ID=ENSP00000264555;PHRF1;;	PHRF1	314804	318130	0.50675	ID=promoter_26492;
2	317074	2_317074	8	17	27	21	13	24	0.413792	1	16	7	18	4	12	11	0.99963644	1	14.75	7.81482108	295157	317949	ID=ENSP00000264555;PHRF1;;	PHRF1	314804	318130	0.50675	ID=promoter_26492;
2	370603	2_370603	8	8	2	9	8	6	0.705675	1	0	3	32	2	2	17	0.00148567	0.0868666	8	10.2678416								
2	415683	2_415683	10	16	76	38	13	57	2.45E-05	0.00261	30	34	53	33	32	43	0.2783992	1	36.25	20.7622597	401322	417982	ID=ENSP00000382533;NAP1L4;;	NAP1L4	412445	416391	0.63348	ID=promoter_26511;
2	430276	2_430276	0	21	2	1	11	2	0.020291	0.71411	2	4	2	9	3	6	0.53745121	1	5.125	6.97828264								
2	481061	2_481061	26	5	0	0	16	0	8.41E-05	0.00761	5	0	2	3	3	3	0.99570314	1	5.125	8.69215574								
2	554638	2_554638	13	11	16	15	12	16	0.945172	1	3	9	22	4	6	13	0.20797135	1	11.625	6.32314343					554138	555570	0.34627	ID=promoter_26541;
2	555098	2_555098	62	21	59	61	42	60	0.617738	1	50	1	4	22	26	13	0.18179217	1	35	25.8788386					554138	555570	0.34627	ID=promoter_26541;
2	575424	2_575424	0	18	6	10	9	8	0.848833	1	8	7	16	10	8	13	0.4490005	1	9.375	5.68048036					571238	576647	0.81034	ID=promoter_26547;
2	613235	2_613235	6	7	2	3	7	3	0.314307	1	7	13	25	4	10	15	0.49251655	1	8.375	7.52021086	588717	618322	ID=ENSP00000263650;OSBPL5;NM_020896;	OSBPL5				
2	626451	2_626451	3	17	5	12	10	9	0.84607	1	1	4	10	0	3	5	0.7175395	1	6.5	5.92814112								
2	627544	2_627544	12	6	41	18	9	30	0.016049	0.59429	0	0	0	0	0	0	1	1	9.625	14.3620283					627165	628347	0.29212	ID=promoter_26558;
2	628723	2_628723	3	4	0	1	4	1	0.312866	1	15	10	8	6	13	7	0.50249747	1	5.875	4.99821397								
2	659616	2_659616	207	361	188	152	284	170	1.41E-11	#######	406	201	388	146	304	267	0.81261759	1	256.13	109.488992								
2	661763	2_661763	4	10	1	12	7	7	0.956352	1	0	1	22	4	1	13	0.0036419	0.1775497	6.75	7.53562965								
2	721766	2_721766	0	16	0	6	8	3	0.206144	1	32	36	2	3	34	3	7.09E-06	0.0009373	11.875	14.6232447								
2	763240	2_763240	1	1	9	24	1	17	0.002653	0.14147	1	3	1	0	2	1	0.87709017	1	5	8.19407451					762410	763735	0.20725	ID=promoter_26586;
2	789884	2_789884	5	6	4	8	6	6	0.962469	1	4	22	10	23	13	17	0.64388579	1	10.25	7.83308004								
2	794452	2_794452	16	22	7	19	19	13	0.317047	1	22	6	15	34	14	25	0.13553211	1	17.625	8.991067								
2	805135	2_805135	2	31	8	5	17	7	0.051092	1	19	22	4	19	21	12	0.40192224	1	13.75	10.4437268					804641	805664	0.17561	ID=promoter_26598;
2	816563	2_816563	10	26	29	18	18	24	0.918896	1	20	28	31	28	24	30	0.54878546	1	23.75	7.14642768					816065	816847	0.1436	ID=promoter_26600;
2	823917	2_823917	290	62	138	177	176	158	0.050053	1	119	38	23	45	79	34	6.44E-04	0.043538	111.5	90.2520281					823571	824412	0.1485	ID=promoter_26603;
2	833390	2_833390	0	5	10	22	3	16	0.03035	0.98285	0	50	3	22	25	13	0.22997911	1	14	17.0461558					832777	833603	0.17792	ID=promoter_26608;
2	946257	2_946257	15	1	6	10	8	8	0.950274	1	47	10	27	2	29	15	0.17706132	1	14.75	15.4156692								
2	979740	2_979740	15	38	7	11	27	9	0.002276	0.124	1	0	7	0	1	4	0.36109265	1	9.875	12.5861318								
2	983350	2_983350	36	22	11	1	29	6	7.05E-05	0.00656	2	4	14	1	3	8	0.26303803	1	11.375	12.4434435								
2	991893	2_991893	900	451	738	771	676	755	0.651637	1	357	264	182	89	311	136	6.98E-13	2.96E-10	469	300.235146								
2	1E+06	2_1039720	3	29	12	3	16	8	0.127562	1	4	25	19	9	15	14	0.99983448	1	13	10.2399777					1039204	1040545	0.2202	ID=promoter_26639;
2	1E+06	2_1050463	31	7	10	6	19	8	0.039867	1	39	19	29	0	29	15	0.17706132	1	17.625	14.0604562					1050411	1051340	0.15848	ID=promoter_26644;
2	1E+06	2_1072595	25	12	15	14	19	15	0.513973	1	34	8	0	3	21	2	7.81E-04	0.0515074	13.875	11.205069					1072093	1072899	0.15755	ID=promoter_26651;
2	1E+06	2_1118671	1	54	19	6	28	13	0.016679	0.61094	10	18	28	47	14	38	0.00151301	0.0879967	22.875	19.0670808					1117404	1119343	0.2677	ID=promoter_26660;
2	1E+06	2_113224	21	70	51	32	46	42	0.500364	1	6	9	26	14	8	20	0.04735057	1	28.625	22.0385215								
2	1E+06	2_1146315	15	39	13	17	27	15	0.059587	1	17	3	34	5	10	20	0.12622058	1	17.875	12.6878739					1145804	1146812	0.23071	ID=promoter_26670;

2	1E+06	2_1147391	39	17	28	33	28	31	0.975765	1	0	23	7	6	12	7	0.61698677	1	19.125	13.994259	1147015	1148054	0.16102	ID=promoter_26671;
2	1E+06	2_119707	158	134	178	159	146	169	0.993165	1	205	239	258	12	222	135	5.14E-04	0.0362959	167.88	75.8597155				
2	1E+06	2_1212636	46	0	18	10	23	14	0.143642	1	60	2	14	0	31	7	0.00122211	0.0738454	18.75	22.4483534	1212107	1212916	0.21018	ID=promoter_26683;
2	1E+06	2_121374	49	49	28	42	49	35	0.07705	1	3	25	26	11	14	19	0.55289753	1	29.125	16.8814141				
2	1E+06	2_1216780	119	85	116	93	102	105	0.643105	1	61	45	63	44	53	54	0.88746565	1	78.25	29.6587737	1216328	1216829	0.18134	ID=promoter_26685;
2	1E+06	2_1219551	19	29	8	22	24	15	0.151024	1	27	42	15	19	35	17	0.08418543	1	22.625	10.2391057	1217743	1219653	0.36116	ID=promoter_26686;
2	1E+06	2_1226178	124	98	209	201	111	205	5.27E-04	0.03696	39	15	21	4	27	13	0.14207	1	88.875	82.8724278	1225382	1226513	0.25695	ID=promoter_26688;
2	1E+06	2_124599	28	10	42	37	19	40	0.103616	1	12	11	15	15	12	15	0.74080056	1	21.25	12.6462869				
2	1E+06	2_125433	74	37	60	122	56	91	0.155939	1	14	12	31	37	13	34	0.00397338	0.1887956	48.375	36.4061906				
2	1E+06	2_1257152	19	11	7	2	15	5	0.034023	1	1	10	0	0	6	0	0.06196836	1	6.25	6.79810898	1256927	1257502	0.14435	ID=promoter_26695;
2	1E+06	2_127113	51	90	39	50	71	45	0.004586	0.21862	22	15	43	9	19	26	0.43045343	1	39.875	25.7484396				
2	1E+06	2_129655	23	0	4	3	12	4	0.066899	1	0	0	9	1	0	5	0.06742467	1	5	7.8921299				
2	1E+06	2_130602	13	28	8	25	21	17	0.520268	1	21	10	26	3	16	15	0.99951315	1	16.75	9.43776608				
2	1E+06	2_1340375	53	30	62	59	42	61	0.565516	1	14	40	6	4	27	5	0.00115665	0.0708495	33.5	23.6159752	1337028	1342393	0.81394	ID=promoter_26713;
2	1E+06	2_136089	67	31	14	35	49	25	0.0023	0.12489	79	18	35	17	49	26	0.06630134	1	37	23.8985953				
2	1E+06	2_136989	43	37	17	12	40	15	3.72E-04	0.02775	9	14	42	7	12	25	0.0604166	1	22.625	15.3337862				
2	1E+06	2_1371576	24	159	28	42	92	35	1.78E-08	#######	37	20	8	2	29	5	5.02E-04	0.0355894	40	49.9170741	1371261	1371925	0.15817	ID=promoter_26719;
2	1E+06	2_1393840	315	363	681	1037	339	859	1.28E-33	#######	4297	2853	8749	2919	3575	5834	8.51E-159	5.79E-155	2651.8	2858.20322	1393524	1394636	0.25968	ID=promoter_26722;
2	1E+06	2_1408330	176	83	71	39	130	55	3.09E-10	#######	88	47	30	23	68	27	6.27E-04	0.0425498	69.625	49.3209243	1407960	1409482	0.2645	ID=promoter_26727;
2	1E+06	2_1412830	9	19	17	1	14	9	0.360318	1	40	1	3	3	21	3	0.0022553	0.1213334	11.625	13.4688795	1410436	1418861	0.97504	ID=promoter_26728;
2	1E+06	2_1416609	22	1	10	0	12	5	0.126784	1	13	1	8	17	7	13	0.3188207	1	9	8.10643483	1410436	1418861	0.97504	ID=promoter_26728;
2	1E+06	2_1420365	4	1	7	15	3	11	0.181935	1	9	34	34	20	22	27	0.61865829	1	15.5	12.8840987	1419182	1422674	0.58767	ID=promoter_26729;
2	1E+06	2_1424025	9	31	3	8	20	6	0.006754	0.29841	1	0	13	19	1	16	7.41E-04	0.0492332	10.5	10.4471459	1423462	1425015	0.42967	ID=promoter_26730;
2	1E+06	2_1430501	40	40	11	30	40	21	0.009148	0.37984	8	28	32	70	18	51	1.01E-04	0.0092979	32.375	19.3016469	1430001	1434393	0.73525	ID=promoter_26732;
2	1E+06	2_1432199	104	161	96	57	133	77	3.15E-06	0.00043	86	114	59	37	100	48	6.87E-04	0.0462479	89.25	39.1325586	1430001	1434393	0.73525	ID=promoter_26732;
2	1E+06	2_1452824	10	30	19	2	20	11	0.118302	1	42	14	6	1	28	4	2.96E-04	0.0229072	15.5	14.3427433	1451508	1453998	0.5821	ID=promoter_26740;
2	1E+06	2_1460738	4	20	9	14	12	12	0.926346	1	5	4	21	5	5	13	0.12287893	1	10.25	7.16638981	1458643	1463100	0.7301	ID=promoter_26743;
2	1E+06	2_1473142	126	30	0	4	78	2	2.93E-19	#######	0	36	21	7	18	14	0.88543974	1	28	41.9285106	1471906	1474288	0.43072	ID=promoter_26745;
2	1E+06	2_1475044	10	1	11	4	6	8	0.9722	1	6	6	11	3	6	7	0.91781435	1	6.5	3.81725406	1474556	1476607	0.32765	ID=promoter_26746;
2	1E+06	2_149023	1211	570	1126	1009	891	1068	0.892177	1	552	375	380	354	464	367	0.07515768	1	697.13	359.475188				
2	2E+06	2_150561	169	71	353	301	120	327	4.30E-15	#######	30	19	75	43	25	59	2.64E-04	0.0206659	132.63	129.19082				
2	2E+06	2_154349	47	33	35	12	40	24	0.030246	0.98061	1	5	2	50	3	26	4.42E-05	0.0045798	23.125	20.4201406				
2	2E+06	2_1547329	153	90	101	81	122	91	0.004373	0.21078	10	21	61	46	16	54	7.36E-06	0.000965	70.375	46.3339971	1546202	1547429	0.19301	ID=promoter_26752;
2	2E+06	2_156058	38	40	32	25	39	29	0.17243	1	100	139	123	54	120	89	0.27886666	1	68.875	44.894917				
2	2E+06	2_158183	117	23	47	58	70	53	0.053072	1	2	9	73	24	6	49	9.21E-09	2.29E-06	44.125	38.1816616				

2	2E+06	2_159478	3	10	3	1	7	2	0.160905	1	29	1	7	1	15	4	0.06170633	1	6.875	9.50845488								
2	2E+06	2_1595221	275	88	222	209	182	216	0.993209	1	14	20	41	1	17	21	0.67722313	1	108.75	109.544185					1595041	1596499	0.24087	ID=promoter_26761;
2	2E+06	2_1632691	2287	1680	2041	2336	1984	2189	0.14237	1	417	396	697	410	407	554	2.46E-08	5.72E-06	1283	885.699723					1632014	1632733	0.16868	ID=promoter_26764;
2	2E+06	2_1657774	55	11	54	50	33	52	0.412259	1	55	16	15	2	36	9	8.09E-04	0.0530112	32.25	23.1501311					1657451	1658271	0.17404	ID=promoter_26771;
2	2E+06	2_1732468	59	42	111	124	51	118	1.96E-04	0.01596	34	43	13	38	39	26	0.42501095	1	58	39.0018315					1732201	1733355	0.19747	ID=promoter_26782;
2	2E+06	2_1766514	59	17	39	21	38	30	0.264168	1	25	24	21	0	25	11	0.10848128	1	25.75	17.1942681					1765210	1767768	0.40501	ID=promoter_26789;
2	2E+06	2_1767393	3	4	14	6	4	10	0.424038	1	19	18	13	4	19	9	0.23949138	1	10.125	6.6211242					1765210	1767768	0.40501	ID=promoter_26789;
2	2E+06	2_178902	120	76	116	50	98	83	0.090354	1	71	88	36	18	80	27	1.35E-05	0.0016433	71.875	36.2705607								
2	2E+06	2_1813846	443	301	374	269	372	322	3.26E-04	0.02485	74	66	57	33	70	45	0.15629834	1	202.13	163.191769	2E+06	1818000	ID=ENSP0000227507-D2;CCND1;NM_053056;	CCND1	1813707	1814310	0.16378	ID=promoter_26795;
2	2E+06	2_182772	0	12	16	11	6	14	0.355886	1	8	11	22	18	10	20	0.12622058	1	12.25	6.69221296								
2	2E+06	2_1848490	6	68	8	7	37	8	6.99E-06	0.00087	2	22	68	30	12	49	3.10E-06	0.000454	26.375	27.3074427					1846846	1848792	0.32207	ID=promoter_26802;
2	2E+06	2_1876077	866	493	536	618	680	577	6.80E-08	#######	463	257	436	168	360	302	0.44018565	1	479.63	214.023321					1875800	1876585	0.11776	ID=promoter_26809;
2	2E+06	2_1946175	1	29	12	5	15	9	0.269309	1	10	17	25	1	14	13	0.99999086	1	12.5	10.5288719					1945704	1946487	0.24692	ID=promoter_26817;
2	2E+06	2_200656	424	225	325	310	325	318	0.070736	1	75	98	142	19	87	81	0.99961309	1	202.25	141.743985								
2	2E+06	2_204215	0	13	12	8	7	10	0.922856	1	8	11	7	8	10	8	0.95278883	1	8.375	4.03334317								
2	2E+06	2_206538	16	17	3	9	17	6	0.027279	0.89953	7	8	10	35	8	23	0.01462253	0.532614	13.125	9.96332561								
2	2E+06	2_206754	1	0	14	5	1	10	0.048828	1	11	0	38	1	6	20	0.0132456	0.49354	8.75	12.9587257								
2	2E+06	2_2080876	198	92	205	213	145	209	0.161349	1	145	105	115	18	125	67	0.00121647	0.0738454	136.38	67.481082					2080019	2081184	0.28887	ID=promoter_26839;
2	2E+06	2_220519	10	28	7	8	19	8	0.039867	1	22	14	18	4	18	11	0.54801163	1	13.875	8.25378701								
2	2E+06	2_2238016	32	8	24	16	20	20	0.880283	1	16	4	7	1	10	4	0.34296513	1	13.5	10.5830052					2237320	2238297	0.1604	ID=promoter_26859;
2	2E+06	2_2284741	87	67	68	93	77	81	0.791187	1	116	62	86	48	89	67	0.43545179	1	78.375	21.3470473					2284507	2285236	0.16977	ID=promoter_26867;
2	2E+06	2_230098	39	98	28	46	69	37	4.26E-04	0.03093	21	61	50	50	41	50	0.42646522	1	49.125	23.5459278								
2	2E+06	2_2308964	30	59	26	28	45	27	0.019532	0.69375	19	26	19	10	23	15	0.56706676	1	27.125	14.4067197					2308817	2310952	0.33064	ID=promoter_26872;
2	2E+06	2_2331903	1388	1130	1733	1739	1259	1736	7.01E-05	0.00654	800	538	971	275	669	623	0.99671454	1	1071.8	533.128167					2331569	2332754	0.29384	ID=promoter_26875;
2	2E+06	2_235876	3	4	15	13	4	14	0.128496	1	2	6	2	0	4	1	0.45332778	1	5.625	5.47559521								
2	2E+06	2_2444685	8	1	9	11	5	10	0.615109	1	48	32	55	1	40	28	0.51986598	1	20.625	21.4405324					2443984	2445494	0.39191	ID=promoter_26894;
2	2E+06	2_2480349	43	29	23	24	36	24	0.094407	1	46	13	15	1	30	8	0.00380767	0.1842991	24.25	15.1256641					2476538	2480731	0.81527	ID=promoter_26899;
2	3E+06	2_250693	72	80	67	65	76	66	0.20028	1	167	157	83	21	162	52	2.81E-11	1.00E-08	89	48.9868787								
2	3E+06	2_252425	0	12	5	5	6	5	0.851367	1	0	14	2	37	7	20	0.02608553	0.8424716	9.375	12.3049061								
2	3E+06	2_253416	53	24	105	86	39	96	3.44E-04	0.02603	19	23	17	1	21	9	0.13945494	1	41	36.9130252								
2	3E+06	2_2555924	0	11	0	0	6	0	0.029633	0.9649	1	10	18	0	6	9	0.65381141	1	5	7.0305456	3E+06	2582941	ID=ENSP0000255087;MTL5;NM_004923;	MTL5				
2	3E+06	2_2582972	2	18	11	4	10	8	0.719353	1	0	16	8	27	8	18	0.09791732	1	10.75	9.17683419					2582358	2583469	0.19619	ID=promoter_26916;

2	3E+06	2_2593887	9	1	1	7	5	4	0.848147	1	1	1	7	15	1	11	0.01045793	0.4067594	5.25	5.1754917					2592958	2594513	0.24197	ID=promoter_26917;
2	3E+06	2_260318	0	15	9	7	8	8	0.950274	1	0	0	11	2	0	7	0.02292696	0.7568963	5.5	5.83095189								
2	3E+06	2_261787	1	10	5	2	6	4	0.674789	1	5	9	12	0	7	6	0.99011599	1	5.5	4.44007722								
2	3E+06	2_263803	3	3	8	10	3	9	0.343967	1	1	9	9	7	5	8	0.62517663	1	6.25	3.41216312								
2	3E+06	2_2641374	7	19	21	8	13	15	0.999061	1	2	5	13	23	4	18	0.00668749	0.2868305	12.25	7.94175224					2640756	2641867	0.18324	ID=promoter_26924;
2	3E+06	2_2714908	24	28	22	5	26	14	0.055012	1	29	23	19	48	26	34	0.41458891	1	24.75	11.9731843	3E+06	2768604	ID=ENSP00000377390;SAPS3;;	SAPS3				
2	3E+06	2_2716727	14	36	17	35	25	26	0.911793	1	33	10	37	31	22	34	0.16818144	1	26.625	11.0445526	3E+06	2768604	ID=ENSP00000377390;SAPS3;;	SAPS3	2715500	2716833	0.29838	ID=promoter_26939;
2	3E+06	2_2739873	4	29	13	6	17	10	0.210142	1	13	19	7	14	16	11	0.74569574	1	13.125	8.09651601	3E+06	2768604	ID=ENSP00000377390;SAPS3;;	SAPS3	2739612	2741637	0.27832	ID=promoter_26946;
2	3E+06	2_2990047	39	24	52	36	32	44	0.790995	1	17	31	30	4	24	17	0.69596948	1	29.125	14.5252244	3E+06	3062096	ID=ENSP00000261349-D2;LRP6;NM_002336;	LRP6				
2	3E+06	2_309563	1	62	3	1	32	2	1.33E-07	#######	0	17	25	14	9	20	0.07989336	1	15.375	20.9553096								
2	3E+06	2_309605	4	23	2	0	14	1	0.001161	0.07164	1	22	0	0	12	0	0.00384008	0.1844256	6.5	9.97138764								
2	3E+06	2_310858	19	29	11	16	24	14	0.105787	1	39	40	22	1	40	12	0.00146628	0.0860502	22.125	13.4635752								
2	3E+06	2_3158642	58	60	49	53	59	51	0.276863	1	24	50	72	25	37	49	0.25792312	1	48.875	16.685644	3E+06	3185031	ID=ENSP00000265689;CHKA;NM_001277;	CHKA	3157309	3159117	0.26905	ID=promoter_27017;
2	3E+06	2_3170803	59	96	70	44	78	57	0.023372	0.79837	33	34	35	2	34	19	0.20290377	1	46.625	28.3243333	3E+06	3185031	ID=ENSP00000265689;CHKA;NM_001277;	CHKA	3170401	3171274	0.17425	ID=promoter_27021;
2	3E+06	2_338165	16	24	16	11	20	14	0.325699	1	22	38	30	26	30	28	0.99966898	1	22.875	8.67570499								
2	4E+06	2_3556074	14	14	29	27	14	28	0.256487	1	32	15	38	36	24	37	0.14603802	1	25.625	9.98480989					3555774	3556531	0.36591	ID=promoter_27069;
2	4E+06	2_377748	1	23	6	0	12	3	0.030625	0.98374	9	13	3	1	11	2	0.06154634	1	7	7.87400787								
2	4E+06	2_403259	20	92	15	27	56	21	1.58E-05	0.00177	21	53	43	52	37	48	0.30396646	1	40.375	25.5786602								
2	4E+06	2_4045478	0	7	8	8	4	8	0.677894	1	12	0	0	5	6	3	0.67473421	1	5	4.5669621					4045091	4045590	0.20738	ID=promoter_27107;
2	4E+06	2_4053968	395	125	361	358	260	360	0.124653	1	151	167	127	60	159	94	0.0022905	0.1230876	218	131.128073	4E+06	4101890	ID=ENSP00000386894-D2;RBM14;;	RBM14	4052751	4054730	0.6267	ID=promoter_27108;
2	4E+06	2_422651	9	7	4	1	8	3	0.206144	1	27	11	21	20	19	21	0.85675254	1	12.5	9.16515139								
2	4E+06	2_4234860	714	340	870	566	527	718	0.032914	1	290	338	235	140	314	188	1.10E-05	0.0013789	436.63	253.740552					4233298	4235258	0.54618	ID=promoter_27132;
2	4E+06	2_432720	114	15	83	49	65	66	0.710817	1	25	20	16	0	23	8	0.04560275	1	40.25	39.3364607								
2	4E+06	2_4423157	329	107	291	280	218	286	0.460105	1	55	129	95	39	92	67	0.32158127	1	165.63	115.591569	4E+06	4423357	ID=ENSP00000310832-D2;CTSF;NM_003793;	CTSF	4422657	4423852	0.45908	ID=promoter_27150;
2	4E+06	2_4475518	165	80	196	172	123	184	0.111764	1	77	45	72	39	61	56	0.99860817	1	105.75	61.8979575					4474506	4475888	0.37872	ID=promoter_27156;
2	4E+06	2_4498478	6	49	0	1	28	1	3.37E-07	#######	14	26	11	7	20	9	0.18397305	1	14.25	16.2810144	4E+06	4499103	ID=ENSP00000259708-D2;KLC4;NM_201523;	KLC4				
2	5E+06	2_4532635	56	25	55	40	41	48	0.99996	1	26	0	27	1	13	14	0.92696813	1	28.75	21.3123036					4531839	4533194	0.24902	ID=promoter_27168;

2	5E+06	2_4533819	41	99	75	93	70	84	0.990128	1	52	26	44	31	39	38	0.97643104	1	57.625	27.9486136					4533401	4535040	0.24975	ID=promoter_27169;
2	5E+06	2_460497	5	91	7	17	48	12	8.80E-07	0.00013	32	65	15	10	49	13	1.07E-04	0.0097311	30.25	31.3903989								
2	5E+06	2_464005	8	33	7	0	21	4	7.04E-04	0.04721	0	15	13	0	8	7	0.9937864	1	9.5	11.1739493								
2	5E+06	2_4661790	18	52	37	18	35	28	0.315717	1	26	40	47	11	33	29	0.97818147	1	31.125	15.0089259	5E+06	4666738	ID=ENSP0000318861;SF3B2;NM_006842;	SF3B2	4659881	4662401	0.43697	ID=promoter_27189;
2	5E+06	2_469447	15	11	15	6	13	11	0.725732	1	18	2	8	1	10	5	0.51906437	1	9.5	6.30192714								
2	5E+06	2_4732329	14	19	10	2	17	6	0.027279	0.89953	4	31	6	43	18	25	0.42027749	1	16.125	14.357054					4731660	4733967	0.40811	ID=promoter_27198;
2	5E+06	2_4744676	4	14	8	4	9	6	0.554309	1	34	14	33	16	24	25	0.92007618	1	15.875	11.7890688	5E+06	4750290	ID=ENSP0000310448;SART1;NM_005146;	SART1	4742876	4745910	0.54762	ID=promoter_27201;
2	5E+06	2_475202	15	10	4	8	13	6	0.146758	1	0	0	6	19	0	13	9.01E-04	0.0577931	7.75	6.77706215								
2	5E+06	2_479530	40	53	31	36	47	34	0.095427	1	22	33	18	15	28	17	0.38227241	1	31	12.5356634								
2	5E+06	2_4823123	51	10	52	66	31	59	0.088267	1	55	38	18	6	47	12	1.10E-04	0.0099406	37	22.7972429					4822631	4823136	0.17053	ID=promoter_27210;
2	5E+06	2_4824833	7	0	17	28	4	23	0.005032	0.23611	19	7	13	3	13	8	0.65599869	1	11.75	9.30053762	5E+06	4825838	ID=ENSP0000311300;CTSW;NM_001335;	CTSW				
2	5E+06	2_4846621	92	19	43	106	56	75	0.754031	1	1	11	28	25	6	27	5.47E-04	0.0380615	40.625	38.2433468	5E+06	4849989	ID=ENSP0000308591-D2;GAL3ST3;NM_033036;	GAL3ST3	4845536	4847297	0.77305	ID=promoter_27213;
2	5E+06	2_486467	0	0	831	693	0	762	########	#######	9	7	61	37	8	49	7.83E-08	1.66E-05	204.75	346.54406								
2	5E+06	2_4890409	1455	937	1126	1161	1196	1144	5.00E-06	0.00064	880	628	898	438	754	668	0.6946929	1	940.38	316.990733					4890281	4892542	0.38641	ID=promoter_27220;
2	5E+06	2_5024491	11	1	3	14	6	9	0.896349	1	1	2	4	5	2	5	0.46647814	1	5.125	4.82367672					5024100	5025326	0.24749	ID=promoter_27236;
2	5E+06	2_5137949	13	134	295	262	74	279	4.23E-20	#######	18	21	31	43	20	37	0.03978904	1	102.13	115.84033	5E+06	5148390	ID=ENSP0000312671;EHBP1L1;NM_001099409;	EHBP1L1				
2	5E+06	2_5149566	254	79	176	111	167	144	0.024981	0.84297	14	49	49	27	32	38	0.58553412	1	94.875	82.6099916					5148093	5150233	0.59046	ID=promoter_27254;
2	5E+06	2_5152254	633	232	516	538	433	527	0.850293	1	389	270	160	158	330	159	4.57E-11	1.59E-08	362	183.865634					5151044	5152554	0.37031	ID=promoter_27255;
2	5E+06	2_5196234	87	102	131	102	95	117	0.939537	1	54	81	112	42	68	77	0.48432602	1	88.875	29.5994088					5194843	5196555	0.33021	ID=promoter_27260;
2	5E+06	2_520833	3	0	41	56	2	49	1.26E-08	#######	0	4	4	0	2	2	0.99713337	1	13.5	22.0389266								
2	5E+06	2_520987	231	188	197	192	210	195	0.062529	1	227	69	105	56	148	81	5.88E-04	0.040383	158.13	70.5092446								
2	5E+06	2_5414001	2	49	5	28	26	17	0.164334	1	39	47	29	4	43	17	0.00910684	0.3639239	25.375	19.4564532	5E+06	5422972	ID=ENSP0000279247;CAPN1;NM_005186;	CAPN1				
2	6E+06	2_551776	392	158	236	254	275	245	0.007215	0.31236	101	63	79	9	82	44	0.01242431	0.467945	161.5	125.740095								
2	6E+06	2_5710684	118	42	63	100	80	82	0.691672	1	44	5	0	0	25	0	9.28E-06	0.0011901	46.5	45.2295731	6E+06	5726022	ID=ENSP0000263277;EHD2;NM_014601;	EHD2	5710440	5711181	0.16513	ID=promoter_27306;
2	6E+06	2_5832290	38	34	107	70	36	89	5.75E-04	0.03976	11	19	78	45	15	62	9.20E-08	1.93E-05	50.25	32.3805497	6E+06	5837629	ID=ENSP0000316385;RASGRP2;;	RASGRP2	5831853	5832635	0.1536	ID=promoter_27323;
2	6E+06	2_587614	83	119	210	130	101	170	0.015344	0.57367	59	57	57	37	58	47	0.79031295	1	94	56.9586316								
2	6E+06	2_590657	203	63	158	149	133	154	0.994059	1	62	58	66	46	60	56	0.99933807	1	100.63	59.7851809								
2	6E+06	2_590786	60	55	38	71	58	55	0.526975	1	102	41	33	51	72	42	0.05516392	1	56.375	22.2321357								

2	6E+06	2_5959407	29	30	10	20	30	15	0.021049	0.7339	1	0	17	0	1	9	0.02974549	0.9232309	13.375	12.5349511	6E+06	5963155	ID=ENSP00000367102-D2;SLC22A6;NM_004790;	SLC22A6				
2	6E+06	2_603713	14	22	2	8	18	5	0.008132	0.34618	17	5	6	2	11	4	0.25131486	1	9.5	7.36788398								
2	6E+06	2_607158	10	20	18	22	15	20	0.931941	1	1	26	9	5	14	7	0.39930956	1	13.875	8.87110075								
2	6E+06	2_630623	132	65	85	66	99	76	0.019816	0.70156	100	205	201	42	153	122	0.46216816	1	112	62.1840586								
2	6E+06	2_634538	84	129	115	109	107	112	0.701131	1	58	75	43	61	67	52	0.6291173	1	84.25	30.6535945								
2	6E+06	2_636177	23	31	60	20	27	40	0.643072	1	57	38	33	30	48	32	0.34885608	1	36.5	14.7066554								
2	6E+06	2_645623	31	57	43	93	44	68	0.359723	1	21	32	24	10	27	17	0.45364599	1	38.875	26.0737279								
2	7E+06	2_679861	5	5	7	1	5	4	0.848147	1	0	7	8	22	4	15	0.026797	0.8546242	6.875	6.74933859								
2	7E+06	2_688656	153	156	213	293	155	253	0.005015	0.23539	92	40	54	29	66	42	0.1591225	1	128.75	92.7142307								
2	7E+06	2_708551	20	24	12	2	22	7	0.005528	0.25484	0	2	9	9	1	9	0.02974549	0.9232309	9.75	8.69729022								
2	7E+06	2_740377	75	8	56	65	42	61	0.565516	1	0	23	0	0	12	0	0.00384008	0.1844256	28.375	31.9326859								
2	7E+06	2_740919	182	56	136	103	119	120	0.504652	1	0	23	18	27	12	23	0.11381058	1	68.125	65.2674443								
2	7E+06	2_746178	73	40	75	46	57	61	0.883395	1	2	0	12	15	1	14	0.00214405	0.1161796	32.875	30.2297748								
2	8E+06	2_7520110	2	10	14	6	6	10	0.79153	1	0	17	10	1	9	6	0.8208167	1	7.5	6.27921742	8E+06	7523933	ID=ENSP00000367258;EEF1G;;	EEF1G				
2	8E+06	2_774433	9	1	5	0	5	3	0.645778	1	16	4	8	2	10	5	0.51906437	1	5.625	5.26273965								
2	8E+06	2_7842275	105	126	110	106	116	108	0.223433	1	55	107	180	74	81	127	9.24E-04	0.05912	107.88	36.821723	8E+06	7860546	ID=ENSP00000257261;FADS2;;	FADS2	7842205	7842772	0.14403	ID=promoter_27441;
2	8E+06	2_7842600	1	41	0	4	21	2	7.54E-05	0.00697	0	9	0	0	5	0	0.09850879	1	6.875	14.1465544	8E+06	7860546	ID=ENSP00000257261;FADS2;;	FADS2	7842205	7842772	0.14403	ID=promoter_27441;
2	8E+06	2_791487	1	10	6	10	6	8	0.9722	1	11	1	12	6	6	9	0.65381141	1	7.125	4.35685011								
2	8E+06	2_791558	8	48	5	12	28	9	0.001431	0.08503	1	120	13	1	61	7	3.49E-09	9.35E-07	26	40.8831086								
2	8E+06	2_802249	13	57	32	35	35	34	0.735392	1	10	9	23	8	10	16	0.39084582	1	23.375	17.1958259								
2	8E+06	2_8047397	378	122	234	224	250	229	0.025417	0.8545	116	106	56	54	111	55	5.78E-04	0.0398304	161.25	110.473979					8047120	8048311	0.42965	ID=promoter_27452;
2	8E+06	2_814046	124	65	120	59	95	90	0.347064	1	1	5	0	0	3	0	0.24893445	1	46.75	53.4836424								
2	8E+06	2_821779	45	8	69	59	27	64	0.007474	0.32245	17	7	13	13	12	13	0.92692342	1	28.875	24.8908331								
2	8E+06	2_825701	73	15	118	134	44	126	9.41E-07	0.00014	0	8	15	1	4	8	0.43697044	1	45.5	54.9831143								
2	8E+06	2_828195	2	6	6	11	4	9	0.54411	1	1	3	3	33	2	18	8.85E-04	0.0572814	8.125	10.5348063								
2	8E+06	2_838212	8	17	12	11	13	12	0.835334	1	0	30	10	20	15	15	0.97869956	1	13.5	8.94427191								
2	8E+06	2_846257	35	1	9	9	18	9	0.098614	1	40	22	22	13	31	18	0.28104051	1	18.875	13.4953696								
2	9E+06	2_860293	1	19	17	13	10	15	0.833288	1	33	20	13	20	27	17	0.45364599	1	17	8.99205999								
2	9E+06	2_900196	36	76	89	87	56	88	0.229003	1	25	34	24	16	30	20	0.51779033	1	48.375	30.3641589								
2	9E+06	2_925743	17	10	2	3	14	3	0.010924	0.43763	1	5	21	3	3	12	0.04708211	1	7.75	7.53562965								
2	9E+06	2_9269710	430	172	346	313	301	330	0.698229	1	302	272	157	115	287	136	4.29E-10	1.33E-07	263.38	107.104672	9E+06	9274621	ID=ENSP00000227524;PRPF19;NM_014502;	PRPF19				
2	9E+06	2_930359	35	56	34	60	46	47	0.801096	1	47	33	52	9	40	31	0.75504669	1	40.75	16.5421539								
2	9E+06	2_934992	26	32	10	8	29	9	8.94E-04	0.05763	39	1	8	1	20	5	0.01914528	0.6572777	15.625	14.6281285								
2	9E+06	2_9405153	1941	682	1699	1176	1312	1438	0.206263	1	946	588	603	295	767	449	5.93E-14	2.82E-11	991.25	577.292139					9404305	9405456	0.30303	ID=promoter_27502;
2	1E+07	2_958227	7	2	10	22	5	16	0.124289	1	0	2	19	14	1	17	4.35E-04	0.0314643	9.5	8.24621125								
2	1E+07	2_959365	1	16	14	22	9	18	0.416978	1	25	5	8	19	15	14	0.99983448	1	13.75	8.44731572								
2	1E+07	2_959791	9	13	6	5	11	6	0.3028	1	89	34	11	0	62	6	7.45E-10	2.23E-07	20.875	29.3327584								
2	1E+07	2_976842	54	61	53	43	58	48	0.199164	1	14	40	55	32	27	44	0.06498379	1	44	15.3622915								
2	1E+07	2_9905629	22	69	27	7	46	17	9.70E-05	0.00867	81	16	67	22	49	45	0.99896345	1	38.875	28.5928937	1E+07	9925518	ID=ENSP00000263847;OSBP;NM_002556;	OSBP				
2	1E+07	2_9909115	25	46	23	28	36	26	0.163613	1	66	73	68	9	70	39	0.03624353	1	42.25	24.388229	1E+07	9925518	ID=ENSP00000263847;OSBP;NM_002556;	OSBP				
2	1E+07	2_126030	0	10	3	4	5	4	0.848147	1	1	23	9	6	12	8	0.76853133	1	7	7.36788398								
2	1E+07	2_126429	158	85	80	40	122	60	9.45E-08	#######	19	19	29	14	19	22	0.7764064	1	55.5	49.7220848								
2	1E+07	2_130661	15	15	16	5	15	11	0.4902	1	0	3	2	1	2	2	0.99713337	1	7.125	6.95778084								
2	1E+07	2_13279786	54	22	41	307	38	174	2.35E-15	#######	41	44	114	56	43	85	1.87E-04	0.0155325	84.875	93.6931733					1.3E+07	1.3E+07	0.47205	ID=promoter_27575;

2	1E+07	2_13328364	11	11	3	1	11	2	0.020291	0.71411	8	3	3	0	6	2	0.42506085	1	5	4.37525509	1E+07	1.3E+07	ID=ENSP0000378758;MADD;;	MADD				
2	1E+07	2_13420164	25	23	23	26	24	25	0.917255	1	67	48	41	11	58	26	0.00707604	0.2995366	33	17.896528	1E+07	1.3E+07	ID=ENSP0000327309;ARFGAP2;NM_032389;	ARFGAP2				
2	1E+07	2_136729	11	19	10	13	15	12	0.610117	1	33	32	16	1	33	9	0.00251067	0.1318429	16.875	10.9731816								
2	1E+07	2_139315	0	3	3	4	2	4	0.823343	1	0	7	23	1	4	12	0.09850366	1	5.125	7.58640702								
2	1E+07	2_143729	2	37	7	17	20	12	0.173914	1	54	6	30	10	30	20	0.51779033	1	20.375	18.2908057								
2	1E+07	2_144757	93	27	81	69	60	75	0.934749	1	45	4	49	11	25	30	0.63340892	1	47.375	32.3107125								
2	1E+07	2_14780407	115	64	137	113	90	125	0.472921	1	39	51	44	29	45	37	0.86385836	1	74	41.3175853	1E+07	1.5E+07	ID=ENSP0000257821;PHF21A;;	PHF21A				
2	1E+07	2_14781227	53	106	36	26	80	31	2.60E-07	#######	78	34	48	30	56	39	0.38794685	1	51.375	27.635058	1E+07	1.5E+07	ID=ENSP0000257821;PHF21A;;	PHF21A				
2	1E+07	2_149177	69	25	101	59	47	80	0.125401	1	14	11	22	0	13	11	0.97545578	1	37.625	34.9364218								
2	1E+07	2_149213	36	145	21	20	91	21	7.06E-13	#######	95	123	78	44	109	61	0.00616815	0.2683255	70.25	47.5927666								
2	2E+07	2_150008	0	63	8	16	32	12	0.001806	0.10314	0	17	24	40	9	32	6.35E-04	0.0429702	21	21.4675835								
2	2E+07	2_151056	55	43	17	39	49	28	0.008152	0.34652	202	27	84	42	115	63	0.00314276	0.1583386	63.625	59.3438828								
2	2E+07	2_151083	0	0	0	11	0	6	0.077487	1	26	0	8	3	13	6	0.35360803	1	6	9.11827051								
2	2E+07	2_151745	10	44	9	4	27	7	4.86E-04	0.03443	35	6	7	33	21	20	0.99628266	1	18.5	16.0089261								
2	2E+07	2_151772	179	26	70	135	103	103	0.518567	1	462	226	180	167	344	174	3.20E-10	1.00E-07	180.63	130.738493								
2	2E+07	2_152370	0	35	7	0	18	4	0.003432	0.17404	13	13	8	0	13	4	0.12786863	1	9.5	11.6741473								
2	2E+07	2_152744	219	106	226	227	163	227	0.246224	1	22	89	104	99	56	102	1.91E-04	0.0158188	136.5	77.243585								
2	2E+07	2_153757	8	31	6	6	20	6	0.006754	0.29841	19	24	11	2	22	7	0.03554381	1	13.375	10.1971635								
2	2E+07	2_154172	1300	731	1324	1025	1016	1175	0.947055	1	573	478	493	292	526	393	0.00522175	0.2374447	777	393.383855								
2	2E+07	2_156007	132	81	104	138	107	121	0.962749	1	2	17	21	22	10	22	0.06514386	1	64.625	55.61587								
2	2E+07	2_158431	3021	1440	3202	2297	2231	2750	0.219368	1	657	341	667	401	499	534	0.06979971	1	1503.3	1183.49298								
2	2E+07	2_158933	19	11	38	53	15	46	0.003558	0.17915	17	0	28	0	9	14	0.47220951	1	20.75	18.3750607								
2	2E+07	2_159624	0	28	3	3	14	3	0.010924	0.43763	11	7	0	9	9	5	0.6500913	1	7.625	9.16417715								
2	2E+07	2_160631	22	78	42	51	50	47	0.550897	1	79	45	106	40	62	73	0.38185007	1	57.875	27.3570962								
2	2E+07	2_162454	219	82	186	164	151	175	0.994162	1	44	13	31	4	29	18	0.40423266	1	92.875	84.7288743								
2	2E+07	2_164591	51	104	55	40	78	48	0.001663	0.09698	0	0	0	0	0	0	1	1	31.25	38.2277012								
2	2E+07	2_168252	20	9	17	10	15	14	0.827043	1	1	15	9	1	8	5	0.78486764	1	10.25	6.94365075								
2	2E+07	2_16838613	158	70	204	59	114	132	0.994906	1	126	65	37	106	96	72	0.39558322	1	103.13	56.8065326	2E+07	1.7E+07	ID=ENSP0000368109;ACCSL;NM_001031854;	ACCSL				
2	2E+07	2_17130520	565	212	444	771	389	608	5.32E-05	0.00511	339	209	265	194	274	230	0.539608	1	374.88	206.314211					1.7E+07	1.7E+07	0.32008	ID=promoter_27688;
2	2E+07	2_17226006	5	2	4	5	4	5	0.995512	1	24	6	11	1	15	6	0.20085144	1	7.25	7.40173725	2E+07	1.7E+07	ID=ENSP0000039989;TTC17;NM_018259;	TTC17				
2	2E+07	2_17318223	88	29	78	41	59	60	0.738249	1	8	45	15	30	27	23	0.95651383	1	41.75	28.3334734	2E+07	1.7E+07	ID=ENSP0000039989;TTC17;NM_018259;	TTC17	1.7E+07	1.7E+07	0.33344	ID=promoter_27690;
2	2E+07	2_17343485	15	23	14	8	19	11	0.163668	1	35	46	33	12	41	23	0.14981223	1	23.25	13.4350288	2E+07	1.7E+07	ID=ENSP0000368129;API5L1;NM_006595;	API5L1				
2	2E+07	2_175407	147	191	195	167	169	181	0.696605	1	140	103	192	21	122	107	0.91610949	1	144.5	59.131814								
2	2E+07	2_181147	33	82	26	15	58	21	6.55E-06	0.00082	30	22	35	21	26	28	0.85926991	1	33	20.880613								
2	2E+07	2_191626	9	16	4	8	13	6	0.146758	1	23	21	20	0	22	10	0.16182597	1	12.625	8.55131569								
2	2E+07	2_192468	0	2	16	4	1	10	0.048828	1	4	0	21	24	2	23	6.46E-05	0.0062789	8.875	9.8479512								
2	2E+07	2_199296	1	91	19	30	46	25	0.006513	0.28949	62	39	26	60	51	43	0.90026258	1	41	28.6256628								
2	2E+07	2_20222323	7	22	5	9	15	7	0.113635	1	52	2	18	0	27	9	0.02128286	0.7187388	14.375	16.9784527					2E+07	2E+07	0.36774	ID=promoter_27694;
2	2E+07	2_20222698	491	219	347	330	355	339	0.025234	0.84916	364	226	246	148	295	197	0.00164214	0.0926759	296.38	107.993303					2E+07	2E+07	0.36774	ID=promoter_27694;
2	2E+07	2_205239	9	79	23	39	44	31	0.089434	1	109	138	30	26	124	28	2.23E-12	8.94E-10	56.625	46.6321088								
2	2E+07	2_205310	52	90	20	29	71	25	2.82E-07	#######	42	28	21	35	35	28	0.8442835	1	39.625	22.9840783								
2	2E+07	2_210248	12	55	6	0	34	3	1.56E-07	#######	36	8	42	18	22	30	0.38408346	1	22.125	19.7732684								

2	2E+07	2_226818	10	29	8	6	20	7	0.013885	0.52965	27	2	48	15	15	32	0.02335039	0.7680341	18.125	15.4682855								
2	2E+07	2_230085	19	78	19	65	49	42	0.325711	1	46	61	27	27	54	27	0.02898642	0.9098185	42.75	23.0077627								
2	2E+07	2_230819	1	0	0	2	1	1	0.993645	1	10	1	20	55	6	38	2.50E-06	0.0003753	11.125	19.0520902								
2	2E+07	2_23235542	8	23	5	4	16	5	0.021336	0.74103	2	14	6	10	8	8	0.98858271	1	9	6.78232998	2E+07	2.3E+07	ID=ENSP0000368144;;NM_024841;					
2	2E+07	2_23270751	8	6	4	19	7	12	0.724589	1	3	8	7	0	6	4	0.87665919	1	6.875	5.61725657	2E+07	2.3E+07	ID=ENSP0000368144;;NM_024841;					
2	2E+07	2_234268	3	14	2	1	9	2	0.058587	1	12	3	12	0	8	6	0.92562801	1	5.875	5.74300817								
2	2E+07	2_238021	1	11	27	40	6	34	4.32E-04	0.03125	52	62	44	13	57	29	0.02830192	0.8918846	31.25	21.684425								
2	2E+07	2_238115	20	8	15	6	14	11	0.605836	1	25	17	11	7	21	9	0.13945494	1	13.625	6.80204801								
2	2E+07	2_238901	3	43	18	14	23	16	0.267873	1	2	11	26	19	7	23	0.00743961	0.3120589	17	13.2449451								
2	2E+07	2_239246	43	49	45	25	46	35	0.15092	1	1	82	18	5	42	12	7.13E-04	0.0477506	33.5	26.7154316								
2	2E+07	2_241011	21	1	3	4	11	4	0.10516	1	36	4	7	0	20	4	0.00863789	0.3495868	9.5	12.5698051								
2	2E+07	2_241276	22	0	0	10	11	5	0.189421	1	0	26	0	1	13	1	0.00904697	0.3617342	7.375	10.8619585								
2	2E+07	2_24149743	20	11	12	2	16	7	0.076817	1	52	8	23	2	30	13	0.06382438	1	16.25	16.2897865	2E+07	2.4E+07	ID=ENSP0000278385;CD44;NM_000610;	CD44				
2	2E+07	2_24169302	13	0	14	21	7	18	0.195146	1	0	0	5	0	0	3	0.19828566	1	6.625	8.27970841	2E+07	2.4E+07	ID=ENSP0000278385;CD44;NM_000610;	CD44				
2	2E+07	2_242809	161	426	560	302	294	431	0.012728	0.49332	98	189	215	72	144	144	0.81326888	1	252.88	167.915317								
2	2E+07	2_24891301	7	2	0	0	5	0	0.05327	1	1	5	12	13	3	13	0.02959638	0.9232309	5	5.23722937	2E+07	2.5E+07	ID=ENSP0000311010;ELF5;NM_198381;	ELF5				
2	2E+07	2_24894878	2	35	14	3	19	9	0.06806	1	2	1	2	1	2	2	0.99713337	1	7.5	11.9163753	2E+07	2.5E+07	ID=ENSP0000311010;ELF5;NM_198381;	ELF5				
2	3E+07	2_25047623	29	57	35	25	43	30	0.087253	1	51	76	34	3	64	19	2.57E-05	0.0028557	38.75	22.2630379					2.5E+07	2.5E+07	0.13692	ID=promoter_27725;
2	3E+07	2_25138010	0	2	2	0	1	1	0.993645	1	0	8	0	35	4	18	0.00668749	0.2868305	5.875	12.0763937	3E+07	2.5E+07	ID=ENSP0000298992;ABTB2;NM_145804;	ABTB2				
2	3E+07	2_252851	233	99	164	95	166	130	0.002405	0.13005	139	98	104	19	119	62	9.52E-04	0.060345	118.88	62.1781714								
2	3E+07	2_261230	34	13	10	12	24	11	0.027804	0.91326	0	15	11	1	8	6	0.92562801	1	12	10.4471459								
2	3E+07	2_262898	23	11	36	38	17	37	0.100517	1	0	5	5	4	3	5	0.7175395	1	15.25	15.0973034								
2	3E+07	2_265997	13	16	6	1	15	4	0.015825	0.58672	19	0	11	1	10	6	0.69729498	1	8.375	7.40535521								
2	3E+07	2_266378	36	36	90	69	36	80	0.005283	0.24537	0	10	10	0	5	5	0.99284883	1	31.375	33.3335417								
2	3E+07	2_267045	5	2	7	1	4	4	0.97482	1	9	4	5	20	7	13	0.3188207	1	6.625	5.97464882								
2	3E+07	2_267519	6033	2599	3728	4033	4316	3881	2.98E-32	#######	4315	2390	3395	1537	3353	2466	2.45E-17	1.45E-14	3503.8	1379.67747								
2	3E+07	2_267985	1020	907	1488	1496	964	1492	1.45E-10	#######	788	609	1391	314	699	853	4.55E-07	8.23E-05	1001.6	433.340822								
2	3E+07	2_26946032	324	98	276	230	211	253	0.972526	1	119	68	95	24	94	60	0.07576647	1	154.25	108.032733	3E+07	2.7E+07	ID=ENSP0000368424;PAX6;;	PAX6	2.7E+07	2.7E+07	0.52482	ID=promoter_27776;
2	3E+07	2_282670	31	48	38	39	40	39	0.712977	1	27	68	29	24	48	27	0.11059688	1	38	14.3825291								
2	3E+07	2_284076	9	0	13	8	5	11	0.495311	1	22	3	15	0	13	8	0.65599869	1	8.75	7.74135462								
2	3E+07	2_290774	0	16	19	0	8	10	0.991043	1	35	1	16	1	18	9	0.30718372	1	11	12.7166932								
2	3E+07	2_29366761	160	57	244	122	109	183	0.011798	0.46127	76	59	47	0	68	24	1.32E-04	0.0115204	95.625	77.0731332					2.9E+07	2.9E+07	0.32573	ID=promoter_27785;
2	3E+07	2_304629	64	38	33	66	51	50	0.665611	1	18	25	23	23	22	23	0.92188352	1	36.25	18.8205814								
2	3E+07	2_306088	50	67	71	50	59	61	0.787332	1	66	40	43	1	53	22	0.00501977	0.2287702	48.5	22.4181049								
2	3E+07	2_30881013	3893	2192	3655	2778	3043	3217	2.14E-04	0.0172	1947	1643	1901	780	1795	1341	1.60E-08	3.82E-06	2348.6	1043.3987					3.1E+07	3.1E+07	0.251	ID=promoter_27794;
2	3E+07	2_31018954	257	140	376	218	199	297	0.030981	0.99404	186	92	91	68	139	80	0.00279709	0.1430946	178.5	103.934045					3.1E+07	3.1E+07	0.26379	ID=promoter_27796;

2	3E+07	2_31025041	90	69	80	106	80	93	0.998278	1	25	68	15	13	47	14	4.39E-04	0.0317376	58.25	35.8478929	3E+07	3.1E+07	ID=ENSP00003302 40;CCDC34;NM_030771;	CCDC34				
2	3E+07	2_331823	36	46	52	41	41	47	0.99425	1	7	28	52	39	18	46	7.16E-04	0.0479065	37.625	14.7835575								
2	3E+07	2_34519137	419	203	290	285	311	288	0.015053	0.56455	59	167	172	133	113	153	0.00892161	0.3589328	216	111.79701	3E+07	3.5E+07	ID=ENSP0000263160;SLC17A6;NM_020346;	SLC17A6	3.5E+07	3.5E+07	0.53855	ID=promoter_27804;
2	3E+07	2_34642495	500	240	821	595	370	708	1.28E-13	#######	311	215	312	364	263	338	3.49E-04	0.0263379	419.75	206.650671					3.5E+07	3.5E+07	0.31363	ID=promoter_27805;
2	4E+07	2_351429	26	43	7	16	35	12	4.73E-04	0.03365	13	17	27	20	15	24	0.24434768	1	21.125	10.9991883								
2	4E+07	2_358131	36	40	6	5	38	6	5.50E-07	#######	70	29	33	1	50	17	9.73E-04	0.0610135	27.5	23.10844								
2	4E+07	2_359113	12	19	5	0	16	3	0.003782	0.18716	4	11	34	0	8	17	0.13786384	1	10.625	11.4634512								
2	4E+07	2_36197510	23	6	19	46	15	33	0.121516	1	38	11	18	0	25	9	0.04125862	1	20.125	15.5419938	4E+07	3.6E+07	ID=ENSP0000298923;SLC6A5;NM_004211;	SLC6A5				
2	4E+07	2_36624337	143	98	216	149	121	183	0.093904	1	118	58	124	84	88	104	0.24364698	1	123.75	47.9843725					3.7E+07	3.7E+07	0.16405	ID=promoter_27815;
2	4E+07	2_366376	0	9	4	1	5	3	0.645778	1	32	28	3	13	30	8	0.00380767	0.1842991	11.25	12.3722039								
2	4E+07	2_368087	22	10	29	21	16	25	0.667718	1	1	0	1	0	1	1	0.99856566	1	10.5	11.8683252								
2	4E+07	2_369136	0	4	0	0	2	0	0.309451	1	84	0	42	13	42	28	0.39793809	1	17.875	30.3523946								
2	4E+07	2_37013033	832	725	1026	951	779	989	0.255136	1	122	112	119	143	117	131	0.33137545	1	503.75	415.219649					3.7E+07	3.7E+07	0.13764	ID=promoter_27830;
2	4E+07	2_372024	10	7	17	24	9	21	0.212308	1	29	24	33	40	27	37	0.29896222	1	23	11.2630876								
2	4E+07	2_374181	13	9	5	5	11	5	0.189421	1	0	2	3	4	1	4	0.36109265	1	5.125	4.12093956								
2	4E+07	2_376096	45	41	38	17	43	28	0.048681	1	92	48	28	0	70	14	5.99E-08	1.30E-05	38.625	26.8750415								
2	4E+07	2_37705491	30	8	10	13	19	12	0.233425	1	20	6	13	1	13	7	0.50249747	1	12.625	8.97516414	4E+07	3.8E+07	ID=ENSP0000322422;IGSF22;NM_173588;	IGSF22				
2	4E+07	2_37722130	1086	535	758	805	811	782	4.68E-04	0.03346	744	528	255	143	636	199	1.59E-43	2.67E-40	606.75	307.255013					3.8E+07	3.8E+07	0.31347	ID=promoter_27841;
2	4E+07	2_37789986	556	152	301	373	354	337	0.022938	0.78656	114	153	88	27	134	58	4.46E-06	0.0006213	220.5	176.427241					3.8E+07	3.8E+07	0.25633	ID=promoter_27843;
2	4E+07	2_379713	30	17	24	21	24	23	0.786129	1	10	18	14	43	14	29	0.03997116	1	22.125	10.4120466								
2	4E+07	2_38123587	6	36	8	15	21	12	0.1273	1	84	49	13	21	67	17	2.11E-06	0.0003257	29	26.619005	4E+07	3.8E+07	ID=ENSP0000265963;GTF2H1;NM_005316;	GTF2H1				
2	4E+07	2_38652586	69	48	55	56	59	56	0.523991	1	82	47	91	13	65	52	0.73026127	1	57.625	24.0353163					3.9E+07	3.9E+07	0.14232	ID=promoter_27854;
2	4E+07	2_38828130	33	6	20	6	20	13	0.243396	1	0	7	4	1	4	3	0.96209564	1	9.625	11.2496032	4E+07	3.9E+07	ID=ENSP0000382329;OTOG;;	OTOG				
2	4E+07	2_388794	341	238	210	181	290	196	1.21E-08	#######	169	145	76	36	157	56	1.52E-09	4.30E-07	174.5	94.7492933								
2	4E+07	2_389851	36	69	39	54	53	47	0.37511	1	37	50	70	29	44	50	0.61464676	1	48	15.4550038								
2	4E+07	2_39428025	44	36	17	17	40	17	0.001238	0.07553	40	10	50	4	25	27	0.8592775	1	27.25	17.2606075					3.9E+07	3.9E+07	0.64346	ID=promoter_27869;
2	4E+07	2_39512250	17	9	34	203	13	119	8.00E-16	#######	8	10	69	25	9	47	5.62E-07	9.91E-05	46.875	66.2321404					4E+07	4E+07	0.40264	ID=promoter_27870;
2	4E+07	2_396462	32	22	19	33	27	26	0.772172	1	33	28	12	19	31	16	0.15534889	1	24.75	7.88760329								
2	4E+07	2_396564	2	9	31	11	6	21	0.046061	1	0	4	8	0	2	4	0.6610374	1	8.125	10.1339246								
2	4E+07	2_396582	20	28	11	3	24	7	0.00213	0.11798	22	7	11	0	15	6	0.20085144	1	12.75	9.7650982								
2	4E+07	2_40277061	103	79	92	118	91	105	0.993446	1	95	89	103	45	92	74	0.66100252	1	90.5	21.6992429					4E+07	4E+07	0.18147	ID=promoter_27873;
2	4E+07	2_403219	31	0	47	25	16	36	0.087649	1	51	17	42	26	34	34	0.95236908	1	29.875	16.839049								
2	4E+07	2_41943396	6	51	1	2	29	2	7.56E-07	0.00012	2	0	9	2	1	6	0.13767373	1	9.125	17.1750442					4.2E+07	4.2E+07	0.11533	ID=promoter_27889;

2	4E+07	2_42337213	48	73	27	41	61	34	0.001788	0.10267	50	52	53	26	51	40	0.73048207	1	46.25	15.2104288					4.2E+07	4.2E+07	0.39617	ID=promoter_27891;
2	4E+07	2_424236	35	34	38	23	35	31	0.520594	1	6	11	15	25	9	20	0.07989336	1	23.375	11.8916237								
2	4E+07	2_424598	1	33	5	9	17	7	0.051092	1	27	1	17	3	14	10	0.81923661	1	12	12.3750902								
2	4E+07	2_431125	14	1	21	15	8	18	0.296056	1	399	289	289	171	344	230	5.95E-04	0.0406682	149.88	158.856123								
2	4E+07	2_433250	0	33	0	11	17	6	0.027279	0.89953	0	0	0	0	0	0	1	1	5.5	11.7594946								
2	4E+07	2_436288	10	10	15	4	10	10	0.938234	1	25	39	3	0	32	2	5.33E-06	0.0007304	13.25	13.0466197								
2	4E+07	2_436315	6	30	7	0	18	4	0.003432	0.17404	1	27	16	25	14	21	0.37101303	1	14	12.1184629								
2	4E+07	2_447560	13	21	20	0	17	10	0.210142	1	32	37	14	36	35	25	0.60746796	1	21.625	12.8389753								
2	4E+07	2_447778	1	1	16	19	1	18	0.001742	0.10079	37	31	25	91	34	58	0.01639336	0.5828415	27.625	28.6602637								
2	5E+07	2_45165023	14	65	13	8	40	11	2.07E-05	0.00227	27	22	29	20	25	25	0.96475192	1	24.75	17.7582979	5E+07	4.5E+07	ID=ENSP0000340281;EIF4G2;NM_001418;	EIF4G2				
2	5E+07	2_452487	20	175	47	26	98	37	4.65E-09	#######	94	63	86	30	79	58	0.40094173	1	67.625	51.3056318								
2	5E+07	2_45389695	105	23	74	56	64	65	0.71532	1	10	15	26	49	13	38	8.08E-04	0.0530112	44.75	32.8361734					4.5E+07	4.5E+07	0.37013	ID=promoter_27914;
2	5E+07	2_458619	1302	599	1341	1968	951	1655	2.10E-21	#######	204	219	182	117	212	150	0.0397089	1	741.5	703.371676								
2	5E+07	2_460781	20	15	4	1	18	3	0.001281	0.07728	0	1	40	0	1	20	8.76E-05	0.0082396	10.125	14.2572037								
2	5E+07	2_462301	39	65	56	14	52	35	0.036495	1	126	48	14	5	87	10	8.83E-13	3.72E-10	45.875	38.9924443								
2	5E+07	2_46233547	1106	576	1090	1212	841	1151	0.003046	0.15775	693	510	639	216	602	428	1.42E-04	0.0123104	755.25	347.267188					4.6E+07	4.6E+07	0.70167	ID=promoter_27928;
2	5E+07	2_462447	12	1	7	1	7	4	0.503048	1	0	0	6	29	0	18	6.08E-05	0.0059624	7	9.85610761								
2	5E+07	2_463683	131	145	240	165	138	203	0.120638	1	96	204	273	104	150	189	0.01890169	0.6517357	169.75	63.9949775								
2	5E+07	2_46411494	205	54	206	169	130	188	0.18436	1	46	61	19	38	54	29	0.05601166	1	99.75	79.2640253					4.6E+07	4.6E+07	0.26699	ID=promoter_27946;
2	5E+07	2_464178	36	19	63	29	28	46	0.368904	1	17	8	23	0	13	12	0.99994263	1	24.375	19.2571879								
2	5E+07	2_46531659	1442	496	1119	924	969	1022	0.060118	1	450	547	377	159	499	268	2.73E-12	1.09E-09	689.25	430.931135					4.7E+07	4.7E+07	0.34676	ID=promoter_27960;
2	5E+07	2_46733882	494	208	433	355	351	394	0.835774	1	192	128	162	115	160	139	0.85477096	1	260.88	145.937793					4.7E+07	4.7E+07	0.34326	ID=promoter_27976;
2	5E+07	2_46829332	155	102	155	142	129	149	0.991844	1	87	20	76	1	54	39	0.49191155	1	92.25	58.8988237	5E+07	4.7E+07	ID=ENSP0000250974;FAM108A4;NM_031213;	FAM108A4	4.7E+07	4.7E+07	0.71513	ID=promoter_27985;
2	5E+07	2_46923643	2005	1447	2226	1823	1726	2025	0.999974	1	901	1095	1094	384	998	739	2.11E-05	0.0024277	1371.9	620.026368					4.7E+07	4.7E+07	0.36388	ID=promoter_27994;
2	5E+07	2_469815	5	19	0	2	12	1	0.003675	0.18279	15	5	0	17	10	9	0.99792604	1	7.875	7.86379588								
2	5E+07	2_470028	0	6	5	8	3	7	0.596562	1	4	7	7	27	6	17	0.046525	1	8	8.07111251								
2	5E+07	2_47026361	0	23	1	0	12	1	0.003675	0.18279	11	15	0	1	13	1	0.00904697	0.3617342	6.375	8.87914893					4.7E+07	4.7E+07	0.51111	ID=promoter_28001;
2	5E+07	2_47045768	440	104	374	319	272	347	0.583783	1	175	173	96	77	174	87	1.11E-05	0.0013866	219.75	139.094778	5E+07	4.7E+07	ID=ENSP0000318075;PLEKHJ1;NM_018049;	PLEKHJ1	4.7E+07	4.7E+07	0.66333	ID=promoter_28002;
2	5E+07	2_47083196	23	0	13	16	12	15	0.986595	1	13	5	0	3	9	2	0.1390433	1	9.125	8.37406711					4.7E+07	4.7E+07	0.34584	ID=promoter_28004;
2	5E+07	2_470923	265	68	109	107	167	108	6.32E-06	0.0008	52	72	50	22	62	36	0.07898377	1	93.125	75.283156								
2	5E+07	2_471442	28	21	40	37	25	39	0.536005	1	46	32	40	23	39	32	0.87710631	1	33.375	8.87914893								
2	5E+07	2_471921	239	505	183	165	372	174	4.62E-24	#######	218	118	68	35	168	52	3.47E-12	1.36E-09	191.38	144.993534								
2	5E+07	2_47213517	32	22	30	48	27	39	0.706776	1	24	15	16	8	20	12	0.48622029	1	24.375	12.4204612					4.7E+07	4.7E+07	0.66828	ID=promoter_28009;
2	5E+07	2_47233458	87	91	103	192	89	148	0.032705	1	68	49	42	23	59	33	0.06332576	1	81.875	52.1164561	5E+07	4.7E+07	ID=ENSP0000369328-D3;LINGO2;;	LINGO2	4.7E+07	4.7E+07	0.62372	ID=promoter_28011;
2	5E+07	2_472372	1	13	10	0	7	5	0.693374	1	0	1	24	34	1	29	7.03E-07	0.0001217	10.375	12.7720398								
2	5E+07	2_47282506	1160	538	1533	960	849	1247	2.68E-06	0.00037	583	551	494	142	567	318	3.89E-12	1.51E-09	745.13	442.837099					4.7E+07	4.7E+07	0.20893	ID=promoter_28014;
2	5E+07	2_47411911	192	65	162	157	129	160	0.894618	1	65	29	69	0	47	35	0.61680793	1	92.375	69.2241442					4.7E+07	4.7E+07	0.359	ID=promoter_28020;

2	5E+07	2_48250876	50	26	43	30	38	37	0.721881	1	83	31	44	29	57	37	0.23694992	1	42	18.6700677	5E+07	4.8E+07	ID=ENSP00000262968;TJP3;NM_014428;	TJP3				
2	5E+07	2_485216	71	90	64	44	81	54	0.00494	0.23359	42	31	95	63	37	79	9.23E-05	0.0086362	62.5	22.8097723								
2	5E+07	2_48694925	3	4	16	16	4	16	0.065514	1	0	0	10	4	0	7	0.02292696	0.7568963	6.625	6.56696277	5E+07	4.9E+07	ID=ENSP00000301284;;NM_001001520;					
2	5E+07	2_487237	51	32	70	104	42	87	0.009045	0.37762	65	28	26	22	47	24	0.05449507	1	49.75	28.5594618								
2	5E+07	2_48782998	4941	2969	4762	5399	3955	5081	9.95E-05	0.00884	3413	2498	2292	1798	2956	2045	2.26E-23	1.84E-20	3509	1358.79569					4.9E+07	4.9E+07	0.61152	ID=promoter_28100;
2	5E+07	2_489089	55	9	52	44	32	48	0.557885	1	0	0	24	0	0	12	0.00154584	0.0883233	23	24.1720027								
2	5E+07	2_489186	17	23	34	34	20	34	0.415985	1	15	2	16	0	9	8	0.99626583	1	17.625	12.7047516								
2	5E+07	2_490128	108	58	147	90	83	119	0.372076	1	77	38	54	34	58	44	0.60375992	1	75.75	38.3172844								
2	5E+07	2_491719	153	15	57	36	84	47	1.77E-04	0.01461	1	0	2	1	1	2	0.81304206	1	33.125	52.6536866								
2	5E+07	2_49382408	500	225	373	334	363	354	0.046728	1	91	56	84	43	74	64	0.92058609	1	213.25	172.306828					4.9E+07	4.9E+07	0.38095	ID=promoter_28134;
2	5E+07	2_49411934	251	111	231	190	181	211	0.998151	1	139	75	66	18	107	42	7.20E-06	0.0009501	135.13	83.0996605					4.9E+07	4.9E+07	0.52682	ID=promoter_28137;
2	5E+07	2_49464122	28	18	7	7	23	7	0.003444	0.1741	0	8	14	5	4	10	0.21760186	1	10.875	8.82265752	5E+07	4.9E+07	ID=ENSP00000371037;TMEM146;NM_152784;	TMEM146				
2	5E+07	2_49503282	185	79	201	137	132	169	0.752497	1	46	79	55	21	63	38	0.10826755	1	100.38	66.3947879	5E+07	5E+07	ID=ENSP00000302648;NRTN;NM_004558;	NRTN	5E+07	5E+07	0.51114	ID=promoter_28143;
2	5E+07	2_498159	32	50	28	32	41	30	0.140157	1	20	11	55	19	16	37	0.00673411	0.288397	30.875	15.1792857								
2	5E+07	2_501458	57	7	45	22	32	34	0.922482	1	14	61	29	0	38	15	0.01554903	0.5562856	29.375	22.871926								
2	5E+07	2_504832	476	321	501	398	399	450	0.849733	1	109	52	44	15	81	30	5.12E-05	0.0051875	239.5	205.942433								
2	5E+07	2_505717	49	12	29	69	31	49	0.426941	1	5	35	1	0	20	1	3.70E-04	0.0275298	25	25.0656281								
2	5E+07	2_50760493	466	156	385	337	311	361	0.990936	1	117	137	97	32	127	65	4.05E-04	0.0297464	215.88	157.37166					5.1E+07	5.1E+07	0.57988	ID=promoter_28186;
2	5E+07	2_508350	54	23	62	24	39	43	0.961581	1	35	17	15	0	26	8	0.01635039	0.5814583	28.75	20.6726182								
2	5E+07	2_510186	10	0	9	26	5	18	0.065263	1	0	30	18	30	15	24	0.24434768	1	15.375	12.4778375								
2	5E+07	2_510188	47	40	37	40	44	39	0.441907	1	13	16	33	10	15	22	0.38478854	1	29.5	14.2928554								
2	5E+07	2_510473	11	18	5	5	15	5	0.034023	1	0	1	0	1	1	1	0.99856566	1	5.125	6.40172968								
2	5E+07	2_51047715	84	39	81	85	62	83	0.733091	1	36	52	21	27	44	24	0.10689254	1	53.125	26.6052492					5.1E+07	5.1E+07	0.13533	ID=promoter_28198;
2	5E+07	2_51094670	12	26	9	20	19	15	0.513973	1	0	9	6	0	5	3	0.8350419	1	10.25	9.08295106					5.1E+07	5.1E+07	0.213	ID=promoter_28200;
2	5E+07	2_51267258	3382	2917	3956	4151	3150	4054	4.84E-04	0.03438	3146	2267	5599	1371	2707	3485	1.01E-36	1.37E-33	3348.6	1275.38688					5.1E+07	5.1E+07	0.21475	ID=promoter_28212;
2	5E+07	2_51279925	2	3	8	18	3	13	0.09118	1	24	16	2	4	20	3	0.00346999	0.1723921	9.625	8.55131569	5E+07	5.1E+07	ID=ENSP00000381070;MAP2K7;;	MAP2K7	5.1E+07	5.1E+07	0.10462	ID=promoter_28215;
2	5E+07	2_519449	48	52	22	23	50	23	5.99E-04	0.04108	15	55	48	25	35	37	0.8561429	1	36	16.1775859								
2	5E+07	2_52220051	245	174	226	150	210	188	0.026177	0.87531	156	117	90	50	137	70	2.10E-04	0.0172126	151	65.5678493					5.2E+07	5.2E+07	0.33011	ID=promoter_28249;
2	5E+07	2_52392074	44	1	25	28	23	27	0.999996	1	43	16	60	1	30	31	0.91402185	1	27.25	21.0831686	5E+07	5.2E+07	ID=ENSP00000348757;CCDC151;NM_145045;	CCDC151				
2	5E+07	2_525250	3	71	41	23	37	32	0.448082	1	43	8	18	5	26	12	0.12503864	1	26.5	23.6159752								
2	5E+07	2_527050	0	0	0	3	0	2	0.426327	1	15	0	3	21	8	12	0.56746184	1	5.25	8.13721609								
2	5E+07	2_52757261	12	24	6	5	18	6	0.017303	0.62786	13	21	11	1	17	6	0.10713464	1	11.625	7.85470741	5E+07	5.3E+07	ID=ENSP00000325690;CARM1;NM_199141;	CARM1				

2	5E+07	2_52775997	553	218	465	616	386	541	0.027863	0.91478	155	254	168	90	205	129	0.00243599	0.1287739	314.88	200.25301	5E+07	5.3E+07	ID=ENSP00000270502;C19orf52;NM_138358;	C19orf52	5.3E+07	5.3E+07	0.5592	ID=promoter_28273;
2	5E+07	2_52949718	7935	3037	8405	6526	5486	7466	6.33E-16	#######	5067	2838	2500	2166	3953	2333	8.44E-67	1.83E-63	4809.3	2537.13842					5.3E+07	5.3E+07	0.5627	ID=promoter_28287;
2	5E+07	2_53167770	259	205	295	356	232	326	0.108903	1	228	92	36	27	160	32	3.10E-17	1.83E-14	187.25	122.380846	5E+07	5.3E+07	ID=ENSP00000293677;RAVER1;NM_133452;	RAVER1	5.3E+07	5.3E+07	0.38988	ID=promoter_28309;
2	5E+07	2_53213872	450	237	506	481	344	494	0.015505	0.57849	262	143	204	84	203	144	0.04806546	1	295.88	161.906002					5.3E+07	5.3E+07	0.4801	ID=promoter_28314;
2	5E+07	2_53287013	603	317	677	546	460	612	0.124093	1	429	334	156	119	382	138	9.08E-22	6.72E-19	397.63	203.467047					5.3E+07	5.3E+07	0.43287	ID=promoter_28316;
2	5E+07	2_534263	46	16	38	18	31	28	0.60342	1	32	9	23	25	21	24	0.78178162	1	25.875	12.2058241								
2	5E+07	2_534424	910	284	658	692	597	675	0.807574	1	254	164	151	93	209	122	2.27E-04	0.0184157	400.75	306.743611								
2	5E+07	2_540151	363	189	221	264	276	243	0.004731	0.22457	128	28	105	22	78	64	0.76931549	1	165	117.6581								
2	5E+07	2_548892	56	158	32	81	107	57	5.04E-06	0.00065	181	128	133	42	155	88	0.00102396	0.0638282	101.38	56.1476052								
2	6E+07	2_557697	7	25	3	5	16	4	0.009581	0.39335	5	0	27	10	3	19	0.0015822	0.0898971	10.25	10.15241								
2	6E+07	2_559898	51	33	69	99	42	84	0.016873	0.61727	68	6	6	14	37	10	0.00114024	0.0700243	43.25	34.1833293								
2	6E+07	2_56285421	34	17	53	61	26	57	0.027119	0.89904	13	16	17	10	15	14	0.99983448	1	27.625	19.5808762	6E+07	5.6E+07	ID=ENSP00000350352;TRMT1;NM_017722;	TRMT1	5.6E+07	5.6E+07	0.29694	ID=promoter_28409;
2	6E+07	2_563813	43	44	20	26	44	23	0.005474	0.25327	1	13	18	27	7	23	0.00743961	0.3120589	24	14.5209209								
2	6E+07	2_56629178	13	13	5	10	13	8	0.344217	1	13	18	15	0	16	8	0.35023049	1	10.875	5.79254447	6E+07	5.7E+07	ID=ENSP00000353362;CACNA1A;NM_001127222;	CACNA1A				
2	6E+07	2_568295	71	86	75	55	79	65	0.103793	1	68	11	33	43	40	38	0.9941866	1	55.25	24.9270364								
2	6E+07	2_56963449	37	42	40	43	40	42	0.881842	1	75	41	87	2	58	45	0.66857123	1	45.875	25.7040158					5.7E+07	5.7E+07	0.12355	ID=promoter_28439;
2	6E+07	2_57092697	43	12	28	30	28	29	0.895448	1	14	0	1	14	7	8	0.92121674	1	17.75	14.8780759					5.7E+07	5.7E+07	0.54017	ID=promoter_28450;
2	6E+07	2_580691	3	16	6	7	10	7	0.570701	1	2	2	14	0	2	7	0.20455692	1	6.25	5.87367006								
2	6E+07	2_60015097	76	12	66	53	44	60	0.749369	1	0	5	1	0	3	1	0.65196691	1	26.625	32.601216	6E+07	6E+07	ID=ENSP00000329213;SIVA1;NM_006427;	SIVA1	6E+07	6E+07	0.27183	ID=promoter_28473;
2	6E+07	2_60346306	279	117	349	298	198	324	0.001033	0.06525	152	99	108	22	126	65	5.11E-04	0.0361198	178	115.706525					6E+07	6E+07	0.29775	ID=promoter_28484;
2	6E+07	2_603500	133	49	108	79	91	94	0.688034	1	61	40	41	34	51	38	0.59345148	1	68.125	35.9063763								
2	6E+07	2_60744763	96	27	114	85	62	100	0.141172	1	67	24	36	1	46	19	0.00967	0.3824351	56.25	40.027669					6.1E+07	6.1E+07	0.29473	ID=promoter_28494;
2	6E+07	2_60856935	26	8	12	23	17	18	0.955187	1	21	41	7	1	31	4	8.02E-05	0.0076341	17.375	12.9276835					6.1E+07	6.1E+07	0.41543	ID=promoter_28495;
2	6E+07	2_60964680	4	22	6	3	13	5	0.083134	1	36	6	33	3	21	18	0.97064008	1	14.125	14.0350327	6E+07	6.1E+07	ID=ENSP00000248070;EPS15L1;NM_021235;	EPS15L1				
2	6E+07	2_61458235	20	17	15	4	19	10	0.108738	1	6	1	3	2	4	3	0.96209564	1	8.5	7.57816412	6E+07	6.2E+07	ID=ENSP00000291440;CPAMD8;NM_015692;	CPAMD8				
2	6E+07	2_61671618	295	116	310	267	206	289	0.144787	1	113	74	104	0	94	52	0.01043924	0.4067594	159.88	114.922878					6.2E+07	6.2E+07	0.74934	ID=promoter_28522;
2	6E+07	2_617732	27	19	45	25	23	35	0.623149	1	5	34	31	33	20	32	0.15276046	1	27.375	11.8072314								
2	6E+07	2_61869733	60	30	44	63	45	54	0.993642	1	25	34	35	25	30	30	0.95785282	1	39.5	14.8996644					6.2E+07	6.2E+07	0.48217	ID=promoter_28535;

2	6E+07	2_61952731	30	31	20	41	31	31	0.820663	1	0	8	8	23	4	16	0.01700101	0.6008545	20.125	13.9328748					6.2E+07	6.2E+07	0.33944	ID=promoter_28540;
2	6E+07	2_62179218	889	647	972	871	768	922	0.893099	1	470	371	670	211	421	441	0.20003433	1	637.63	270.394388					6.2E+07	6.2E+07	0.23458	ID=promoter_28551;
2	6E+07	2_62195302	1214	443	1206	1261	829	1234	7.21E-07	0.00011	492	477	1021	350	485	686	6.65E-12	2.51E-09	808	401.109889					6.2E+07	6.2E+07	0.17713	ID=promoter_28554;
2	6E+07	2_62332410	355	55	275	212	205	244	0.988144	1	110	123	138	84	117	111	0.98490295	1	169	103.136525					6.2E+07	6.2E+07	0.2812	ID=promoter_28562;
2	6E+07	2_62362394	19	13	39	32	16	36	0.087649	1	4	16	3	14	10	9	0.99792604	1	17.5	12.5470543					6.2E+07	6.2E+07	0.50553	ID=promoter_28566;
2	6E+07	2_62425769	2434	1431	3198	2346	1933	2772	9.51E-11	#######	997	766	931	375	882	653	7.29E-05	0.0070188	1559.8	987.683256					6.2E+07	6.2E+07	0.21806	ID=promoter_28572;
2	6E+07	2_62556602	6617	6722	8016	9975	6670	8996	5.72E-17	#######	7092	6370	12778	3607	6731	8193	1.35E-60	2.79E-57	7647.1	2727.10691					6.3E+07	6.3E+07	0.20318	ID=promoter_28580;
2	6E+07	2_62966531	8	1	1	6	5	4	0.848147	1	12	12	6	5	12	6	0.45526626	1	6.375	4.24053568	6E+07	6.3E+07	ID=ENSP0000222271;COMP;NM_000095;	COMP				
2	6E+07	2_632083	0	0	7	8	0	8	0.033035	1	15	12	89	12	14	51	6.17E-06	0.0008311	17.875	29.254731								
2	6E+07	2_63736922	295	169	477	221	232	349	0.01309	0.5046	191	112	149	13	152	81	2.46E-04	0.0196336	203.38	137.560311					6.4E+07	6.4E+07	0.28305	ID=promoter_28620;
2	7E+07	2_653543	206	123	134	210	165	172	0.556137	1	59	100	128	35	80	82	0.81509105	1	124.38	62.0574331								
2	7E+07	2_655479	34	36	37	42	35	40	0.993713	1	20	23	11	46	22	29	0.4569972	1	31.125	11.9575141								
2	7E+07	2_655667	95	69	90	50	82	70	0.146246	1	4	82	16	73	43	45	0.85065963	1	59.875	33.8713427								
2	7E+07	2_662706	30	122	34	28	76	31	1.41E-06	0.00021	125	69	79	94	97	87	0.97526254	1	72.625	39.6266054								
2	7E+07	2_66525101	114	53	134	121	84	128	0.174383	1	75	58	62	25	67	44	0.20312041	1	80.25	38.432687	7E+07	6.7E+07	ID=ENSP0000355672;Clorf69;NM_001010867;	Clorf69	6.7E+07	6.7E+07	0.37854	ID=promoter_28646;
2	7E+07	2_67011546	511	147	369	380	329	375	0.930564	1	198	219	207	67	209	137	0.00660036	0.2842033	262.25	145.206011	7E+07	6.7E+07	ID=ENSP0000338711-D4;TRIM6;NM_001003819;	TRIM6	6.7E+07	6.7E+07	0.43153	ID=promoter_28661;
2	7E+07	2_678496	4	0	7	38	2	23	6.93E-04	0.04661	17	54	43	59	36	51	0.14571533	1	27.75	23.5538896								
2	7E+07	2_680116	1	22	0	5	12	3	0.030625	0.98374	0	31	6	13	16	10	0.61601721	1	9.75	11.4361582								
2	7E+07	2_683727	116	49	95	53	83	74	0.22741	1	92	101	42	50	97	46	6.92E-04	0.0465626	74.75	29.0799391								
2	7E+07	2_685227	101	26	79	60	64	70	0.921511	1	16	14	16	3	15	10	0.7195765	1	39.375	35.9202987								
2	7E+07	2_686115	0	25	9	12	13	11	0.725732	1	2	3	18	0	3	9	0.17582851	1	8.625	9.1953016								
2	7E+07	2_68705959	240	103	211	193	172	202	0.999928	1	124	91	119	48	108	84	0.48189682	1	141.13	66.3011258					6.9E+07	6.9E+07	0.42027	ID=promoter_28697;
2	7E+07	2_68892271	755	372	664	648	564	656	0.98931	1	303	218	256	117	261	187	0.02634951	0.8480056	416.63	238.793358					6.9E+07	6.9E+07	0.44669	ID=promoter_28702;
2	7E+07	2_692015	7	10	7	10	9	9	0.944235	1	7	10	13	1	9	7	0.94097808	1	8.125	3.56320482								
2	7E+07	2_692023	41	29	43	29	35	36	0.857856	1	8	9	1	0	9	1	0.05455957	1	20	17.5580669								
2	7E+07	2_69824709	6	2	12	29	4	21	0.010729	0.43446	31	20	20	20	26	20	0.8208633	1	17.5	10.2539191	7E+07	7E+07	ID=ENSP0000348031;NSD1;NM_022455;	NSD1				
2	7E+07	2_69944426	13	16	0	3	15	2	0.002248	0.12273	0	6	1	11	3	6	0.53745121	1	6.25	6.31890587	7E+07	7E+07	ID=ENSP0000261948;ZNF346;;	ZNF346				
2	7E+07	2_701437	23	7	36	12	15	24	0.638589	1	0	10	9	3	5	6	0.91271846	1	12.5	11.6985958								
2	7E+07	2_701439	183	117	200	188	150	194	0.667622	1	47	35	19	24	41	22	0.11146441	1	101.63	79.5270619								
2	7E+07	2_701677	42	32	17	37	37	27	0.166749	1	0	9	33	21	5	27	2.18E-04	0.0177562	23.875	14.6232447								
2	7E+07	2_705942	5	19	7	6	12	7	0.325249	1	25	22	34	6	24	20	0.93987967	1	15.5	11.0194633								
2	7E+07	2_707861	6	35	9	10	21	10	0.052655	1	32	27	35	31	30	33	0.79414776	1	23.125	12.5520346								
2	7E+07	2_714356	36	28	23	13	32	18	0.038646	1	20	11	12	43	16	28	0.11940283	1	23.25	11.7564572								
2	7E+07	2_715901	2	62	23	8	32	16	0.016272	0.6002	2	33	50	24	18	37	0.01748595	0.6144624	25.5	22.0648395								
2	7E+07	2_721037	58	143	69	82	101	76	0.01299	0.50102	30	23	46	33	27	40	0.16464486	1	60.5	39.0054941								
2	7E+07	2_721425	2	1	7	0	2	4	0.823343	1	28	56	37	0	42	19	0.02977583	0.9232309	16.375	21.373799								
2	7E+07	2_721465	8	61	14	6	35	10	1.08E-04	0.00955	0	21	49	4	11	27	0.01818526	0.631944	20.375	22.5321199								

2	7E+07	2_722280	22	1	15	14	12	15	0.986595	1	0	0	7	0	0	4	0.11562589	1	7.375	8.61788008								
2	7E+07	2_725988	31	28	13	38	30	26	0.525876	1	0	20	22	0	10	11	0.92604858	1	19	13.9078601								
2	7E+07	2_72625344	295	125	331	207	210	269	0.632707	1	67	55	43	7	61	25	0.00194492	0.1071378	141.25	122.392518					7.3E+07	7.3E+07	0.12967	ID=promoter_28773;
2	7E+07	2_728504	15	14	16	21	15	19	0.975531	1	56	34	34	52	45	43	0.98996144	1	30.25	16.6883193								
2	7E+07	2_72858689	986	508	741	678	747	710	3.09E-04	0.02375	427	265	402	171	346	287	0.37872246	1	522.25	267.385089					7.3E+07	7.3E+07	0.62189	ID=promoter_28778;
2	7E+07	2_728888	10	6	4	2	8	3	0.206144	1	1	5	8	29	3	19	0.0015822	0.0898971	8.125	8.93528319								
2	7E+07	2_73275304	1	6	6	7	4	7	0.813458	1	2	8	7	13	5	10	0.35527535	1	6.25	3.69362385	7E+07	7.3E+07	ID=ENSP00000369862;COL4A3BP;NM_001130105;	COL4A3BP				
2	7E+07	2_74033736	0	2	19	16	1	18	0.001742	0.10079	0	0	15	0	0	8	0.01336935	0.4945372	6.5	8.51888658	7E+07	7.4E+07	ID=ENSP00000358142-D3;SV2A;NM_014849;	SV2A				
2	7E+07	2_741920	9	38	29	15	24	22	0.704268	1	43	20	68	43	32	56	0.01425704	0.5211625	33.125	19.0295822								
2	7E+07	2_743884	11	31	7	7	21	7	0.0088	0.36783	2	35	11	27	19	19	0.97309647	1	16.375	12.6144758								
2	7E+07	2_745916	1	25	6	12	13	9	0.46931	1	1	12	7	2	7	5	0.9051169	1	8.25	8.10202796								
2	7E+07	2_749142	50	72	58	44	61	51	0.200424	1	26	25	4	6	26	5	0.00174818	0.0978457	35.625	24.4478308								
2	8E+07	2_75187453	15	36	1	18	26	10	0.006911	0.3015	15	1	29	0	8	15	0.25959112	1	14.375	13.4157424	8E+07	7.5E+07	ID=ENSP00000264917;PDE8B;NM_003719;	PDE8B				
2	8E+07	2_75196571	82	37	30	23	60	27	9.91E-05	0.00881	1	5	18	3	3	11	0.0740992	1	24.875	26.5407369	8E+07	7.5E+07	ID=ENSP00000264917;PDE8B;NM_003719;	PDE8B				
2	8E+07	2_756788	81	144	53	91	113	72	2.15E-04	0.01726	111	66	113	61	89	87	0.94000913	1	90	31.0621497								
2	8E+07	2_756845	0	12	4	10	6	7	0.999953	1	0	18	7	2	9	5	0.6500913	1	6.625	6.39056224								
2	8E+07	2_758539	0	17	2	1	9	2	0.058587	1	1	0	19	0	1	10	0.01766414	0.6154554	5	8.07111251								
2	8E+07	2_764063	19	33	10	6	26	8	0.001755	0.10104	4	24	26	20	14	23	0.23132274	1	17.75	10.2364894								
2	8E+07	2_765267	180	88	189	96	134	143	0.733999	1	39	38	29	43	39	36	0.99982789	1	87.75	64.5152252								
2	8E+07	2_766178	458	253	328	331	356	330	0.00852	0.35992	16	3	12	0	10	6	0.69729498	1	175.13	187.455471								
2	8E+07	2_77043727	7	11	7	10	9	9	0.944235	1	2	13	17	5	8	11	0.69450319	1	9	4.75093976	8E+07	7.7E+07	ID=ENSP00000255189;DMGDH;NM_013391;	DMGDH				
2	8E+07	2_771567	2	0	24	31	1	28	2.54E-05	0.00269	17	0	4	40	9	22	0.0389747	1	14.75	15.6090633								
2	8E+07	2_772703	14	18	9	0	16	5	0.021336	0.74103	0	1	13	2	1	8	0.04988758	1	7.125	7.25923058								
2	8E+07	2_777513	53	47	38	52	50	45	0.433543	1	13	12	3	0	13	2	0.02644099	0.8480056	27.25	22.5119016								
2	8E+07	2_78568786	281	87	304	296	184	300	0.001949	0.11028	154	122	130	94	138	112	0.57832943	1	183.5	93.7473809					7.9E+07	7.9E+07	0.42405	ID=promoter_28848;
2	8E+07	2_790808	1	51	10	17	26	14	0.055012	1	13	21	13	5	17	9	0.38755874	1	16.375	15.3524079								
2	8E+07	2_795140	17	55	41	17	36	29	0.317511	1	60	28	71	19	44	45	0.89815416	1	38.5	21.420951								
2	8E+07	2_801584	12	31	13	4	22	9	0.020582	0.72012	2	0	16	0	1	8	0.04988758	1	9.75	10.6200619								
2	8E+07	2_812581	8	17	34	15	13	25	0.34413	1	90	175	174	75	133	125	0.99395421	1	73.5	68.8082221								
2	8E+07	2_816896	39	70	21	35	55	28	0.001063	0.06694	83	53	69	58	68	64	0.99623345	1	53.5	20.7433294								
2	8E+07	2_824276	7	22	7	1	15	4	0.015825	0.58672	8	9	16	36	9	26	0.0081957	0.3355909	13.25	11.1835338								
2	8E+07	2_82810933	385	101	265	262	243	264	0.689516	1	133	81	106	50	107	78	0.26995054	1	172.88	117.22681	8E+07	8.3E+07	ID=ENSP00000369344;GPR150;NM_199243;	GPR150	8.3E+07	8.3E+07	0.62475	ID=promoter_28866;
2	8E+07	2_844095	11	39	7	3	25	5	2.23E-04	0.01779	11	13	24	7	12	16	0.63262749	1	14.375	11.7222316								
2	8E+07	2_847370	27	28	14	6	28	10	0.00287	0.14985	59	16	0	0	38	0	2.24E-08	5.23E-06	18.75	19.5283676								
2	9E+07	2_854591	45	40	22	37	43	30	0.087253	1	7	42	79	23	25	51	0.00411117	0.1947595	36.875	21.323612								
2	9E+07	2_85610803	59	20	52	26	40	39	0.712977	1	15	30	39	49	23	44	0.01592227	0.5683568	36.25	16.0156174					8.6E+07	8.6E+07	0.34042	ID=promoter_28878;
2	9E+07	2_85623986	2546	1746	3204	2918	2146	3061	3.05E-11	#######	1222	1089	1692	468	1156	1080	0.98337271	1	1860.6	954.437896					8.6E+07	8.6E+07	0.81757	ID=promoter_28880;
2	9E+07	2_85797547	179	81	196	151	130	174	0.522495	1	62	71	39	1	67	20	1.68E-05	0.001994	97.5	69.8733548					8.6E+07	8.6E+07	0.56798	ID=promoter_28883;
2	9E+07	2_877237	99	32	108	61	66	85	0.850088	1	24	0	0	0	12	0	0.00384008	0.1844256	40.5	44.1814441								

2	9E+07	2_87750354	48	44	71	62	46	67	0.526313	1	90	63	31	37	77	34	0.00112478	0.0691941	55.75	19.4917565	9E+07	8.8E+07	ID=ENSP00000265138;ARRDC3;NM_020801;	ARRDC3				
2	9E+07	2_882044	42	31	43	76	37	60	0.297878	1	107	54	27	6	81	17	8.60E-09	2.15E-06	48.25	31.2946823								
2	9E+07	2_889746	14	2	7	8	8	8	0.950274	1	2	13	22	1	8	12	0.56746184	1	8.625	7.3277847								
2	9E+07	2_897770	33	38	16	8	36	12	2.99E-04	0.02308	43	8	15	0	26	8	0.01635039	0.5814583	20.125	15.8153407								
2	9E+07	2_906064	4	28	3	0	16	2	0.001284	0.07728	45	38	11	4	42	8	3.29E-05	0.0035427	16.625	17.7437112								
2	9E+07	2_94156644	1473	578	1169	982	1026	1076	0.0371	1	323	533	425	291	428	358	0.35640769	1	721.75	434.418741					9.4E+07	9.4E+07	0.39499	ID=promoter_28926;
2	9E+07	2_941966	42	91	53	31	67	42	0.005325	0.24715	62	34	18	2	48	10	1.56E-05	0.0018671	41.625	27.4691385								
2	9E+07	2_94240456	798	1083	1005	993	941	999	0.089728	1	1513	1310	1623	664	1412	1144	0.00320245	0.1610055	1123.6	335.047092	9E+07	9.4E+07	ID=ENSP00000284049;CHD1;NM_001270;	CHD1				
2	9E+07	2_944013	47	212	89	74	130	82	4.59E-05	0.00451	210	169	221	137	190	179	0.98810159	1	144.88	68.5678757								
2	1E+08	2_954400	3	1	10	0	2	5	0.651182	1	14	0	12	7	7	10	0.67636194	1	5.875	5.6426311								
2	1E+08	2_96076077	1	0	0	8	1	4	0.505922	1	0	2	0	35	1	18	2.55E-04	0.0200383	5.75	12.127301					9.6E+07	9.6E+07	0.25939	ID=promoter_28935;
2	1E+08	2_97246938	16	101	19	40	59	30	6.35E-04	0.04342	103	82	69	102	93	86	0.99986063	1	66.5	36.9130252	1E+08	9.7E+07	ID=ENSP00000369135;SLCO6A1;NM_173488;	SLCO6A1				
2	1E+08	2_975332	4	12	19	30	8	25	0.042844	1	36	6	40	20	21	30	0.30941224	1	20.875	13.4529604								
2	1E+08	2_100790622	7	14	6	3	11	5	0.189421	1	18	10	33	15	14	24	0.17833616	1	13.25	9.43776608								
2	1E+08	2_101279976	15	7	15	0	11	8	0.5832	1	0	0	17	0	0	9	0.00779604	0.3222758	6.75	7.77817459								
2	1E+08	2_101757345	17	17	9	24	17	17	0.897282	1	19	16	7	1	18	4	0.01933304	0.6616555	13.75	7.45941399								
2	1E+08	2_101911083	15	54	14	10	35	12	4.73E-04	0.03365	0	26	21	11	13	16	0.74830213	1	18.875	16.1460964								
2	1E+08	2_102241940	8	20	17	0	14	9	0.360318	1	34	12	52	53	23	53	8.08E-04	0.0530112	24.5	19.8710126								
2	1E+08	2_103284638	66	23	63	41	45	52	0.99731	1	0	1	1	0	1	1	0.99856566	1	24.375	28.7448753	1E+08	1E+08	ID=ENSP00000261483;MAN2A1;NM_002372;	MAN2A1				
2	1E+08	2_103767996	5	40	43	31	23	37	0.48988	1	69	8	34	0	39	17	0.02919595	0.915188	28.75	23.3100224								
2	1E+08	2_104435701	815	264	507	438	540	473	1.99E-05	0.00219	217	312	297	103	265	200	0.08902131	1	369.13	219.101697					1E+08	1E+08	0.23575	ID=promoter_28954;
2	1E+08	2_104511486	31	14	8	27	23	18	0.432563	1	87	18	38	4	53	21	0.00312373	0.1574356	28.375	26.3543843								
2	1E+08	2_105055495	2	24	4	1	13	3	0.018372	0.65917	26	7	21	9	17	15	0.99031847	1	11.75	10.2782711								
2	1E+08	2_105191760	4	3	16	3	4	10	0.424038	1	22	13	4	22	18	13	0.78939737	1	10.875	8.42509008								
2	1E+08	2_105686310	10	13	4	0	12	2	0.011798	0.46127	31	60	36	3	46	20	0.01514356	0.5480081	19.625	20.9416707								
2	1E+08	2_105764168	5	32	17	23	19	20	0.944476	1	91	38	58	1	65	30	0.00552854	0.2489376	33.125	29.7102075								
2	1E+08	2_105908907	29	126	55	44	78	50	0.003238	0.16629	69	43	77	51	56	64	0.51867409	1	61.75	30.0321257								
2	1E+08	2_105961252	144	25	183	122	85	153	0.00617	0.27827	96	58	60	25	77	43	0.02676542	0.8546242	89.125	57.3122712								
2	1E+08	2_106165849	0	5	3	4	3	4	0.986002	1	15	6	7	2	11	5	0.40227598	1	5.25	4.52769257								
2	1E+08	2_106338944	237	68	139	171	153	155	0.436847	1	74	60	66	12	67	39	0.066012	1	103.38	73.1338059					1.1E+08	1.1E+08	0.42803	ID=promoter_28969;
2	1E+08	2_106809149	31	109	13	26	70	20	1.16E-08	#######	89	67	129	6	78	68	0.93369573	1	58.75	46.5395377								
2	1E+08	2_107448934	9	3	0	0	6	0	0.029633	0.9649	0	16	22	0	8	11	0.69450319	1	6.25	8.59817256								
2	1E+08	2_107530811	244	167	136	258	206	197	0.121635	1	1	14	37	48	8	43	1.53E-06	0.000243	113.13	102.801386								
2	1E+08	2_107998674	778	452	496	447	615	472	2.57E-11	#######	227	192	376	254	210	315	3.47E-07	6.50E-05	402.75	189.750776					1.1E+08	1.1E+08	0.64758	ID=promoter_28975;

2	1E+08	2_108102127	6	0	0	1	3	1	0.508575	1	6	24	1	12	15	7	0.31064188	1	6.25	8.29371535								
2	1E+08	2_108133138	0	47	5	0	24	3	4.60E-05	0.00451	0	0	0	0	0	0	1	1	6.5	16.4577381								
2	1E+08	2_109131608	32	86	35	55	59	45	0.090603	1	32	79	76	14	56	45	0.77467809	1	51.125	26.717771								
2	1E+08	2_109146449	4	14	13	18	9	16	0.603365	1	29	40	12	27	35	20	0.21889545	1	19.625	11.5750656								
2	1E+08	2_109242844	1	56	26	36	29	31	0.937432	1	39	45	29	9	42	19	0.02977583	0.9232309	30.125	18.1929146								
2	1E+08	2_109347059	17	23	7	23	20	15	0.418324	1	122	124	29	13	123	21	8.38E-15	4.23E-12	44.75	48.764009								
2	1E+08	2_111455942	355	130	342	291	243	317	0.459269	1	150	143	153	38	147	96	0.02731432	0.8689879	200.25	114.37501					1.1E+08	1.1E+08	0.50044	ID=promoter_28996;
2	1E+08	2_111704113	71	58	85	76	65	81	0.93604	1	27	89	58	17	58	38	0.24734975	1	60.125	26.1557833								
2	1E+08	2_112146873	137	44	99	79	91	89	0.474645	1	24	10	37	10	17	24	0.40932813	1	55	45.8132857								
2	1E+08	2_113970319	82	26	77	28	54	53	0.653183	1	0	0	31	0	0	16	1.79E-04	0.0149638	30.5	33.0194748								
2	1E+08	2_114415504	24	107	62	105	66	84	0.884061	1	984	607	594	510	796	552	9.84E-07	0.0001639	374.13	349.852801								
2	1E+08	2_114462209	0	17	6	1	9	4	0.244237	1	0	22	2	0	11	1	0.02232329	0.7455867	6	8.66849798								
2	1E+08	2_114669976	73	95	118	58	84	88	0.759798	1	41	18	95	87	30	91	1.86E-08	4.40E-06	73.125	32.6515696	1E+08	1.1E+08	ID=ENSP0000384725;PRDM6;NM_001136239;	PRDM6				
2	1E+08	2_114988954	4	20	9	17	12	13	0.980472	1	1	1	0	0	1	0	0.62906424	1	6.5	8.01783726								
2	1E+08	2_115007643	1763	1744	2200	2999	1754	2600	3.02E-13	#######	1815	1527	4159	737	1671	2448	1.12E-46	2.02E-43	2118	1039.41728					1.2E+08	1.2E+08	0.41253	ID=promoter_29016;
2	1E+08	2_115533874	96	81	72	107	89	90	0.610593	1	228	117	170	42	173	106	0.0032916	0.1649067	114.13	59.2221906								
2	1E+08	2_115614802	8	22	1	0	15	1	6.51E-04	0.04418	31	12	6	40	22	23	0.92188352	1	15	14.5896245								
2	1E+08	2_116432655	5	2	6	12	4	9	0.54411	1	1	0	8	10	1	9	0.02974549	0.9232309	5.5	4.34248119								
2	1E+08	2_117184759	0	22	7	5	11	6	0.3028	1	43	1	9	1	22	5	0.00878788	0.3541514	11	14.7454593								
2	1E+08	2_117195862	13	38	0	1	26	1	1.08E-06	0.00016	37	9	30	4	23	17	0.77734386	1	16.5	16.0445807					1.2E+08	1.2E+08	0.26405	ID=promoter_29024;
2	1E+08	2_117325798	4	32	18	1	18	10	0.152558	1	65	23	22	3	44	13	6.70E-04	0.0451887	21	20.9897934								
2	1E+08	2_117394777	178	65	122	153	122	138	0.958246	1	60	105	51	58	83	55	0.15226692	1	99	48.3794525					1.2E+08	1.2E+08	0.77166	ID=promoter_29028;
2	1E+08	2_117417183	46	24	22	23	35	23	0.091136	1	0	11	36	24	6	30	1.30E-04	0.0113876	23.25	14.007651	1E+08	1.2E+08	ID=ENSP0000387123;ALDH7A1;;	ALDH7A1				
2	1E+08	2_117611972	79	59	144	75	69	110	0.133731	1	146	46	65	30	96	48	0.00184554	0.1024142	80.5	42.7584578	1E+08	1.2E+08	ID=ENSP0000309141;MARCH3;NM_178450;	3-Mar				
2	1E+08	2_117707384	4	44	10	12	24	11	0.027804	0.91326	21	0	3	0	11	2	0.06154634	1	11.75	14.8396188								
2	1E+08	2_117962056	0	8	4	8	4	6	0.929647	1	11	12	13	7	12	10	0.96947392	1	7.875	4.3239367								
2	1E+08	2_118145031	101	105	205	215	103	210	1.97E-05	0.00218	44	30	59	31	37	45	0.47096942	1	98.75	74.3942202								
2	1E+08	2_118219395	72	104	154	40	88	97	0.914714	1	37	27	50	71	32	61	0.00336532	0.1683049	69.375	42.1762882								
2	1E+08	2_118609319	3	43	5	14	23	10	0.024151	0.81985	19	5	13	3	12	8	0.76853133	1	13.125	13.4423372								
2	1E+08	2_118661121	29	33	34	25	31	30	0.753666	1	138	18	36	6	78	21	5.90E-07	0.0001036	39.875	40.8671629								
2	1E+08	2_118735618	13	35	47	16	24	32	0.89335	1	19	1	57	38	10	48	8.75E-07	0.0001484	28.25	19.0093962	1E+08	1.2E+08	ID=ENSP0000262461;SLC12A2;NM_001046;	SLC12A2				

2	1E+08	2_118984433	7	29	18	22	18	20	0.986196	1	0	2	33	24	1	29	7.03E-07	0.0001217	16.875	12.4721117	1E+08	1.2E+08	ID=ENSP00000262464;FBN2;NM_001999;	FBN2				
2	1E+08	2_119309563	755	3514	671	802	2135	737	########	#######	1467	1193	3169	1917	1330	2543	1.61E-104	5.89E-101	1686	1106.06678								
2	1E+08	2_119430771	1	16	1	0	9	1	0.020382	0.71452	22	0	11	0	11	6	0.57179039	1	6.375	8.76580206								
2	1E+08	2_119506633	367	139	301	226	253	264	0.412821	1	104	243	103	70	174	87	1.11E-05	0.0013866	194.13	106.626769					1.2E+08	1.2E+08	0.34664	ID=promoter_29047;
2	1E+08	2_119539286	237	466	193	210	352	202	1.38E-15	#######	348	305	349	241	327	295	0.9441201	1	293.63	91.7760278								
2	1E+08	2_119571526	6	18	1	10	12	6	0.213448	1	1	6	7	21	4	14	0.04184353	1	8.75	7.32412842								
2	1E+08	2_120020057	19	3	23	28	11	26	0.138574	1	83	19	24	0	51	12	2.36E-05	0.002647	24.875	25.5199502								
2	1E+08	2_120377394	711	158	542	677	435	610	0.017248	0.62786	255	249	189	103	252	146	3.04E-05	0.0032958	360.5	243.853586					1.2E+08	1.2E+08	0.7492	ID=promoter_29052;
2	1E+08	2_120790097	7	27	5	14	17	10	0.210142	1	65	0	25	17	33	21	0.39890162	1	20	20.4869296								
2	1E+08	2_121281220	335	138	1816	1736	237	1776	########	#######	153	126	707	464	140	586	7.06E-68	1.63E-64	684.38	702.068157					1.2E+08	1.2E+08	0.62212	ID=promoter_29053;
2	1E+08	2_121519669	13	20	9	11	17	10	0.210142	1	0	22	9	0	11	5	0.40227598	1	10.5	8.05339325								
2	1E+08	2_122206340	49	136	54	42	93	48	1.26E-05	0.00147	188	86	181	101	137	141	0.68386904	1	104.63	58.1965573								
2	1E+08	2_122236906	14	17	10	5	16	8	0.127562	1	1	3	28	4	2	16	0.00248877	0.1311269	10.25	9.09866552								
2	1E+08	2_122691435	2	1	2	9	2	6	0.490921	1	24	1	1	0	13	1	0.00904697	0.3617342	5	8.17662173	1E+08	1.2E+08	ID=ENSP00000245407-D3;SLC22A5;NM_003060;	SLC22A5				
2	1E+08	2_123141213	85	119	29	49	102	39	2.90E-09	#######	153	76	129	130	115	130	0.29987848	1	96.25	43.4930208	1E+08	1.2E+08	ID=ENSP00000265343;AFF4;NM_014423;	AFF4				
2	1E+08	2_123216904	1044	508	1095	599	776	847	0.35012	1	489	468	334	270	479	302	8.87E-07	0.0001502	600.88	307.064645					1.2E+08	1.2E+08	0.38146	ID=promoter_29090;
2	1E+08	2_123339288	55	47	37	30	51	34	0.035312	1	52	12	8	0	32	4	5.18E-05	0.0052176	30.125	21.2296121								
2	1E+08	2_123600802	19	44	31	23	32	27	0.44852	1	16	9	17	8	13	13	0.98151319	1	20.875	11.8976288								
2	1E+08	2_123644918	84	113	64	96	99	80	0.044719	1	117	156	152	153	137	153	0.2845753	1	116.88	34.6345719								
2	1E+08	2_123739977	44	0	20	33	22	27	0.987654	1	0	4	7	0	2	4	0.6610374	1	13.5	17.0377732								
2	1E+08	2_124088956	39	38	26	30	39	28	0.134871	1	33	36	46	20	35	33	0.99757329	1	33.5	8.14160391	1E+08	1.2E+08	ID=ENSP00000231512;C5orf15;NM_020199;	C5orf15	1.2E+08	1.2E+08	0.19791	ID=promoter_29111;
2	1E+08	2_124132987	3	7	6	32	5	19	0.046679	1	9	5	20	18	7	19	0.03889743	1	12.5	9.98570407					1.2E+08	1.2E+08	0.59128	ID=promoter_29116;
2	1E+08	2_124133082	691	221	484	404	456	444	0.020026	0.7081	94	168	101	69	131	85	0.03629124	1	279	224.591057					1.2E+08	1.2E+08	0.59128	ID=promoter_29116;
2	1E+08	2_124158638	39	2	38	1	21	20	0.800056	1	0	7	31	4	4	18	0.00668749	0.2868305	15.25	17.4662941								
2	1E+08	2_124167307	1952	1534	2541	2178	1743	2360	3.22E-05	0.00331	1511	1239	1322	577	1375	950	2.39E-11	8.55E-09	1606.8	610.563615					1.2E+08	1.2E+08	0.23421	ID=promoter_29117;
2	1E+08	2_124202510	25	9	7	14	17	11	0.29617	1	0	0	0	0	0	0	1	1	6.875	9.04650682					1.2E+08	1.2E+08	0.13529	ID=promoter_29120;
2	1E+08	2_124235024	209	131	227	187	170	207	0.936813	1	160	126	122	115	143	119	0.6845972	1	159.63	43.2333783								

2	1E+08	2_124339554	25	14	13	8	20	11	0.118302	1	32	8	12	0	20	6	0.03829208	1	14	10.1277554					1.2E+08	1.2E+08	0.13529	ID=promoter_29128;
2	1E+08	2_124676223	0	16	0	0	8	0	0.00917	0.37984	1	0	4	19	1	12	0.00617711	0.2683255	5	7.87400787								
2	1E+08	2_124732021	13	3	5	32	8	19	0.232241	1	9	0	0	1	5	1	0.30486099	1	7.875	10.776131								
2	1E+08	2_124750087	245	75	120	80	160	100	3.36E-06	0.00045	65	90	77	28	78	53	0.21543412	1	97.5	64.8492758								
2	1E+08	2_124756962	49	21	38	17	35	28	0.315717	1	14	15	38	5	15	22	0.38478854	1	24.625	15.1839906								
2	1E+08	2_124812033	13	15	18	22	14	20	0.851664	1	62	30	11	19	46	15	0.00116293	0.0711421	23.75	16.5421539								
2	1E+08	2_124839840	91	57	130	129	74	130	0.020244	0.71411	28	36	47	5	32	26	0.8825541	1	65.375	46.5892002								
2	1E+08	2_124905208	15	11	22	19	13	21	0.660505	1	0	8	10	2	4	6	0.75330063	1	10.875	7.67998326								
2	1E+08	2_124912345	333	115	356	223	224	290	0.539991	1	47	9	48	1	28	25	0.9907189	1	141.5	143.922598								
2	1E+08	2_124941178	16	47	17	19	32	18	0.038646	1	54	26	20	1	40	11	7.51E-04	0.0497587	25	17.3534682	1E+08	1.2E+08	ID=ENSP00000362352-D2;H2AFY2;NM_018649;	H2AFY2				
2	1E+08	2_125037149	26	92	24	46	59	35	0.004964	0.23382	5	49	27	22	27	25	0.99999055	1	36.375	26.4193517								
2	1E+08	2_125075776	190	61	262	181	126	222	0.001176	0.07233	81	59	103	67	70	85	0.24492782	1	125.5	75.9360633					1.3E+08	1.3E+08	0.15271	ID=promoter_29172;
2	1E+08	2_125108754	1	33	21	35	17	28	0.540451	1	63	17	31	35	40	33	0.88417976	1	29.5	17.8805561								
2	1E+08	2_125198328	7	63	17	18	35	18	0.013919	0.53027	71	20	59	10	46	35	0.67768685	1	33.125	26.3895948					1.3E+08	1.3E+08	0.40026	ID=promoter_29182;
2	1E+08	2_125207371	3	13	17	12	8	15	0.557319	1	10	16	8	0	13	4	0.12786863	1	9.875	5.98659216								
2	1E+08	2_125334634	6	0	3	6	3	5	0.88968	1	10	0	23	1	5	12	0.17891578	1	6.125	7.66135945					1.3E+08	1.3E+08	0.13621	ID=promoter_29191;
2	1E+08	2_125354091	43	9	19	57	26	38	0.687307	1	1	30	16	1	16	9	0.48000706	1	22	20.0926426								
2	1E+08	2_125370392	17	37	23	29	27	26	0.772172	1	17	5	23	26	11	25	0.03752473	1	22.125	9.49341877					1.3E+08	1.3E+08	0.16246	ID=promoter_29192;
2	1E+08	2_125376689	71	37	76	80	54	78	0.499532	1	46	16	53	28	31	41	0.3242471	1	50.875	23.4364643								
2	1E+08	2_125616343	27	37	27	22	32	25	0.308825	1	16	4	6	19	10	13	0.72167512	1	19.75	11.0808715								
2	1E+08	2_125643439	36	30	24	20	33	22	0.114927	1	19	2	4	2	11	3	0.13591373	1	17.125	13.1522676								
2	1E+08	2_125728852	6	0	18	9	3	14	0.063635	1	22	0	1	0	11	1	0.02232329	0.7455867	7	8.73416935	1E+08	1.3E+08	ID=ENSP00000265193;TRPC7;NM_020389;	TRPC7				
2	1E+08	2_125741541	19	36	21	10	28	16	0.064038	1	35	53	40	41	44	41	0.99972002	1	31.875	14.0655354	1E+08	1.3E+08	ID=ENSP00000265193;TRPC7;NM_020389;	TRPC7				
2	1E+08	2_125814117	22	52	35	31	37	33	0.517593	1	47	26	19	4	37	12	0.00419427	0.1971915	29.5	15.4642445	1E+08	1.3E+08	ID=ENSP00000265193;TRPC7;NM_020389;	TRPC7				
2	1E+08	2_126154870	7	24	6	16	16	11	0.385778	1	9	18	15	65	14	40	6.80E-04	0.0457983	20	19.1684264								
2	1E+08	2_126350639	0	7	14	4	4	9	0.54411	1	0	15	27	2	8	15	0.25959112	1	8.625	9.44060379								
2	1E+08	2_126396847	51	26	38	22	39	30	0.216196	1	2	4	30	27	3	29	9.29E-06	0.0011901	25	16.2744322								
2	1E+08	2_126435572	19	17	21	12	18	17	0.813792	1	2	9	0	8	6	4	0.87665919	1	11	7.70899289								
2	1E+08	2_126525384	45	0	54	51	23	53	0.022415	0.77045	0	7	5	16	4	11	0.14780855	1	22.25	23.6386487								

2	1E+08	2_126964912	32	30	17	15	31	16	0.023279	0.79554	89	49	64	22	69	43	0.12041403	1	39.75	25.9105605								
2	1E+08	2_127080988	1	40	18	14	21	16	0.42382	1	0	32	16	4	16	10	0.61601721	1	15.625	14.4611549								
2	1E+08	2_127226584	32	71	48	53	52	51	0.661444	1	20	44	31	45	32	38	0.58553412	1	43	15.6204994								
2	1E+08	2_127589250	3922	3603	4623	6324	3763	5474	2.97E-23	#######	3865	3027	6265	1805	3446	4035	7.69E-23	6.13E-20	4179.3	1540.35977					1.3E+08	1.3E+08	0.4441	ID=promoter_29255;
2	1E+08	2_127629192	12	29	8	10	21	9	0.03105	0.99404	1	33	28	0	17	14	0.9475482	1	15.125	13.0541455								
2	1E+08	2_127667594	276	189	212	205	233	209	0.018552	0.66505	202	150	201	96	176	149	0.71696213	1	191.38	51.8126777								
2	1E+08	2_127675321	201	123	161	156	162	159	0.278679	1	18	106	139	1	62	70	0.52686309	1	113.13	69.9906882								
2	1E+08	2_127762981	1	25	8	16	13	12	0.835334	1	1	1	1	18	1	10	0.01766414	0.6154554	8.875	9.58328157								
2	1E+08	2_127890975	17	18	16	35	18	26	0.793453	1	24	18	11	48	21	30	0.30941224	1	23.375	12.2350492					1.3E+08	1.3E+08	0.4536	ID=promoter_29266;
2	1E+08	2_127895853	41	94	86	35	68	61	0.312526	1	22	87	32	4	55	18	3.22E-04	0.024573	50.125	34.0815723					1.3E+08	1.3E+08	0.3493	ID=promoter_29267;
2	1E+08	2_127911816	8	19	11	10	14	11	0.605836	1	21	8	11	12	15	12	0.93002578	1	12.5	4.86973159								
2	1E+08	2_127915963	5	22	2	0	14	1	0.001161	0.07164	0	18	13	1	9	7	0.94097808	1	7.625	8.79833588								
2	1E+08	2_127954739	42	8	46	57	25	52	0.058037	1	32	20	18	0	26	9	0.02974521	0.9232309	27.875	19.7298722								
2	1E+08	2_128150429	1	29	12	0	15	6	0.065303	1	30	11	43	4	21	24	0.78178162	1	16.25	15.8542468								
2	1E+08	2_128180662	13	20	17	30	17	24	0.842518	1	4	16	9	64	10	37	1.47E-04	0.0127053	21.625	18.7688001								
2	1E+08	2_128259285	773	285	254	303	529	279	1.91E-27	#######	230	180	147	81	205	114	5.67E-05	0.0056417	281.63	211.811195					1.3E+08	1.3E+08	0.71891	ID=promoter_29284;
2	1E+08	2_128349576	15	108	22	31	62	27	4.48E-05	0.00443	80	40	39	34	60	37	0.14539173	1	46.125	31.6021586	1E+08	1.3E+08	ID=ENSP00000302917;;;					
2	1E+08	2_128587496	3	41	7	2	22	5	0.001077	0.06758	45	17	13	38	31	26	0.931505	1	20.75	17.8305516					1.3E+08	1.3E+08	0.71108	ID=promoter_29308;
2	1E+08	2_128649641	917	758	110	127	838	119	########	#######	1169	740	95	83	955	89	5.28E-143	3.28E-139	499.88	443.163445					1.3E+08	1.3E+08	0.73296	ID=promoter_29318;
2	1E+08	2_128651072	324	129	404	414	227	409	1.17E-06	0.00017	99	58	174	43	79	109	0.02549222	0.8255505	205.63	152.720976					1.3E+08	1.3E+08	0.73296	ID=promoter_29318;
2	1E+08	2_128751263	29	74	8	15	52	12	1.14E-07	#######	27	50	44	30	39	37	0.99494211	1	34.625	20.9757513								
2	1E+08	2_128865078	65	120	67	68	93	68	0.011457	0.45416	63	48	52	31	56	42	0.58217751	1	64.25	25.7778975								
2	1E+08	2_128884952	164	12	94	77	88	86	0.483625	1	44	1	18	51	23	35	0.17556452	1	57.625	53.5882117					1.3E+08	1.3E+08	0.16838	ID=promoter_29345;
2	1E+08	2_128893812	20	43	8	43	32	26	0.376129	1	26	11	32	11	19	22	0.7764064	1	24.25	14.13961								
2	1E+08	2_128906760	67	49	50	172	58	111	0.009495	0.39165	26	21	83	0	24	42	0.04125931	1	58.5	52.9177259					1.3E+08	1.3E+08	0.48495	ID=promoter_29347;
2	1E+08	2_128987027	186	93	216	153	140	185	0.567385	1	82	65	88	40	74	64	0.92058609	1	115.38	62.2344358								
2	1E+08	2_129010841	462	265	551	438	364	495	0.100494	1	85	106	73	57	96	65	0.14529553	1	254.63	202.654483					1.3E+08	1.3E+08	0.38354	ID=promoter_29358;
2	1E+08	2_129320080	129	131	122	160	130	141	0.812656	1	113	33	64	53	73	59	0.73533013	1	100.63	44.7945708	1E+08	1.3E+08	ID=ENSP00000337513;SRA1;NM_001035235;	SRA1				
2	1E+08	2_129375218	1	15	10	3	8	7	0.850897	1	1	12	2	2	7	2	0.29886173	1	5.75	5.65053727	1E+08	1.3E+08	ID=ENSP00000261812;;;		1.3E+08	1.3E+08	0.23911	ID=promoter_29375;
2	1E+08	2_129854647	761	756	714	1013	759	864	0.834199	1	880	541	1119	353	711	736	0.11051519	1	767.13	245.713793					1.3E+08	1.3E+08	0.45205	ID=promoter_29422;

2	1E+08	2_129900462	54	39	59	78	47	69	0.493091	1	50	61	64	18	56	41	0.51562687	1	52.875	18.0351839	1E+08	1.3E+08	ID=ENSP0000377862-D7;FAT4;NM_024582;	FAT4	1.3E+08	1.3E+08	0.6056	ID=promoter_29432;
2	1E+08	2_129909896	2	69	6	4	36	5	5.53E-07	#######	17	41	22	36	29	29	0.95922868	1	24.625	23.0399498	1E+08	1.3E+08	ID=ENSP0000377862-D7;FAT4;NM_024582;	FAT4	1.3E+08	1.3E+08	0.75706	ID=promoter_29436;
2	1E+08	2_129910407	160	71	101	122	116	112	0.338736	1	186	110	80	2	148	41	3.39E-12	1.34E-09	104	56.4396011	1E+08	1.3E+08	ID=ENSP0000377862-D7;FAT4;NM_024582;	FAT4	1.3E+08	1.3E+08	0.75706	ID=promoter_29436;
2	1E+08	2_129938363	45	30	29	37	38	33	0.447527	1	91	48	22	26	70	24	6.81E-05	0.0065833	41	22.1423963	1E+08	1.3E+08	ID=ENSP0000377862-D7;FAT4;NM_024582;	FAT4	1.3E+08	1.3E+08	0.70517	ID=promoter_29446;
2	1E+08	2_129949012	43	50	7	59	47	33	0.073589	1	27	32	31	72	30	52	0.02131376	0.7194421	40.125	20.3430402	1E+08	1.3E+08	ID=ENSP0000377862-D7;FAT4;NM_024582;	FAT4	1.3E+08	1.3E+08	0.72471	ID=promoter_29450;
2	1E+08	2_129949558	87	14	48	64	51	56	0.943505	1	31	25	38	7	28	23	0.91166341	1	39.25	26.5101059	1E+08	1.3E+08	ID=ENSP0000377862-D7;FAT4;NM_024582;	FAT4	1.3E+08	1.3E+08	0.72471	ID=promoter_29450;
2	1E+08	2_129959412	59	11	48	58	35	53	0.500901	1	22	26	31	13	24	22	0.99927301	1	33.5	19.2056539	1E+08	1.3E+08	ID=ENSP0000377862-D7;FAT4;NM_024582;	FAT4	1.3E+08	1.3E+08	0.72418	ID=promoter_29454;
2	1E+08	2_129968300	11	15	6	5	13	6	0.146758	1	63	47	16	2	55	9	3.53E-07	6.60E-05	20.625	22.1742289					1.3E+08	1.3E+08	0.80061	ID=promoter_29457;
2	1E+08	2_129968642	911	939	1848	2477	925	2163	2.14E-70	#######	664	957	1620	641	811	1131	4.56E-18	2.86E-15	1257.1	655.895775					1.3E+08	1.3E+08	0.80061	ID=promoter_29457;
2	1E+08	2_130056249	85	23	50	87	54	69	0.8949	1	5	10	15	0	8	8	0.98858271	1	34.375	35.2944249								
2	1E+08	2_130063355	6	32	14	8	19	11	0.163668	1	14	2	14	0	8	7	0.9937864	1	11.25	10.0249688					1.3E+08	1.3E+08	0.15685	ID=promoter_29470;
2	1E+08	2_130079516	69	30	51	33	50	42	0.276891	1	1	4	7	1	3	4	0.89184014	1	24.5	25.6793413					1.3E+08	1.3E+08	0.17248	ID=promoter_29475;
2	1E+08	2_130122493	49	75	56	47	62	52	0.200726	1	22	34	82	4	28	43	0.11009196	1	46.125	25.9969092					1.3E+08	1.3E+08	0.14489	ID=promoter_29481;
2	1E+08	2_130133425	18	47	22	14	33	18	0.027763	0.91318	13	20	46	28	17	37	0.01104205	0.4262276	26	13.4907376								
2	1E+08	2_130169770	10	0	2	10	5	6	0.999292	1	0	23	9	0	12	5	0.30420563	1	6.75	8.01338167								
2	1E+08	2_130202241	18	23	10	13	21	12	0.1273	1	0	0	19	1	0	10	0.00454609	0.2107253	10.5	9.2736185								
2	1E+08	2_130219989	51	13	24	27	32	26	0.376129	1	0	0	4	0	0	2	0.34003807	1	14.875	18.2086126	1E+08	1.3E+08	ID=ENSP0000239440-D2;ARAP3;NM_022481;	ARAP3				
2	1E+08	2_130236078	22	36	47	37	29	42	0.681988	1	12	16	19	12	14	16	0.84864027	1	25.125	13.1631249	1E+08	1.3E+08	ID=ENSP0000239440-D2;ARAP3;NM_022481;	ARAP3				

2	1E+08	2_130248760	36	9	33	38	23	36	0.555384	1	60	53	40	19	57	30	0.03938211	1	36	16.5270341								
2	1E+08	2_130265614	0	6	28	33	3	31	7.72E-05	0.0071	0	1	20	0	1	10	0.01766414	0.6154554	11	13.8460928					1.3E+08	1.3E+08	0.13535	ID=promoter_29506;
2	1E+08	2_130296458	16	11	18	9	14	14	0.914609	1	7	20	18	5	14	12	0.98032967	1	13	5.70713839								
2	1E+08	2_130442367	18	0	0	1	9	1	0.020382	0.71452	31	13	2	61	22	32	0.26050199	1	15.75	21.3658606	1E+08	1.3E+08	ID=ENSP0000378028;RNF14;;	RNF14	1.3E+08	1.3E+08	0.12173	ID=promoter_29529;
2	1E+08	2_130464021	186	99	203	239	143	221	0.036049	1	8	23	2	1	16	2	0.00719486	0.3031284	95.125	100.665553	1E+08	1.3E+08	ID=ENSP0000231484;PCDH12;NM_016580;	PCDH12	1.3E+08	1.3E+08	0.44693	ID=promoter_29532;
2	1E+08	2_130474671	886	349	534	440	618	487	3.30E-10	#######	843	479	327	250	661	289	1.42E-26	1.34E-23	513.5	234.707721	1E+08	1.3E+08	ID=ENSP0000231484;PCDH12;NM_016580;	PCDH12	1.3E+08	1.3E+08	0.34598	ID=promoter_29533;
2	1E+08	2_130550574	37	0	21	31	19	26	0.877519	1	33	17	3	0	25	2	1.29E-04	0.0113348	17.75	15.2947236								
2	1E+08	2_130567461	161	56	159	79	109	119	0.863726	1	52	103	55	62	78	59	0.49788993	1	90.875	45.8177679								
2	1E+08	2_130570095	77	88	96	77	83	87	0.764236	1	2	13	47	9	8	28	0.00175088	0.0978457	51.125	38.4909544								
2	1E+08	2_130633659	72	40	12	35	56	24	9.52E-05	0.00854	25	82	46	19	54	33	0.16457707	1	41.375	24.7382728								
2	1E+08	2_130699606	80	37	68	71	59	70	0.99791	1	0	18	4	0	9	2	0.1390433	1	34.75	34.0451381								
2	1E+08	2_130732282	11	67	19	24	39	22	0.019446	0.69119	6	21	63	0	14	32	0.01437521	0.5246762	26.375	25.1506177								
2	1E+08	2_130865902	19	4	7	13	12	10	0.724827	1	1	12	16	1	7	9	0.80856893	1	9.125	6.87516234	1E+08	1.3E+08	ID=ENSP0000377879-D2;FGF2;;	FGF2				
2	1E+08	2_131072766	22	4	41	25	13	33	0.053925	1	20	24	29	1	22	15	0.65424549	1	20.75	12.980755								
2	1E+08	2_131082940	2	18	6	8	10	7	0.570701	1	50	26	33	9	38	21	0.15716135	1	19	16.4142795								
2	1E+08	2_131101255	12	33	11	8	23	10	0.024151	0.81985	12	34	43	9	23	26	0.7859394	1	20.25	13.977022								
2	1E+08	2_131157755	102	40	98	88	71	93	0.782247	1	1	5	4	0	3	2	0.9363008	1	42.25	46.4658092								
2	1E+08	2_131176869	10	19	0	1	15	1	6.51E-04	0.04418	26	3	17	1	15	9	0.58227053	1	9.625	9.97049218								
2	1E+08	2_131273864	38	13	6	49	26	28	0.95198	1	12	26	5	25	19	15	0.89728723	1	21.75	15.7457477								
2	1E+08	2_131334538	4	26	28	0	15	14	0.827043	1	2	4	13	18	3	16	0.00701108	0.2971393	11.875	11.0897314								
2	1E+08	2_131385266	5	35	10	8	20	9	0.046279	1	0	0	16	2	0	9	0.00779604	0.3222758	9.5	11.6619038								
2	1E+08	2_131467845	442	90	6	13	266	10	6.64E-62	#######	58	30	40	2	44	21	0.03878541	1	85.125	147.147581								
2	1E+08	2_131526432	57	18	34	555	38	295	1.36E-35	#######	0	0	10	1	0	6	0.0393172	1	84.375	191.199923								
2	1E+08	2_131717130	5	43	14	21	24	18	0.351411	1	57	22	20	1	40	11	7.51E-04	0.0497587	22.875	18.7421412								
2	1E+08	2_131727158	66	47	45	15	57	30	0.001286	0.07733	60	8	55	2	34	29	0.94710596	1	37.25	25.0926853								
2	1E+08	2_131846937	3	40	21	15	22	18	0.522468	1	6	26	67	14	16	41	0.00153524	0.0883233	24	20.9216224								
2	1E+08	2_132150698	68	82	8	0	75	4	2.99E-17	#######	101	56	31	19	79	25	5.75E-06	0.0007856	45.625	36.6798407								
2	1E+08	2_132254755	0	16	2	6	8	4	0.357158	1	0	11	6	1	6	4	0.87665919	1	5.25	5.77556429								
2	1E+08	2_132278032	20	40	720	701	30	711	########	#######	42	45	222	135	44	179	8.62E-21	6.03E-18	240.63	297.584532								
2	1E+08	2_132415564	331	129	343	333	230	338	0.030416	0.98374	31	20	20	14	26	17	0.53107524	1	152.63	155.995364					1.3E+08	1.3E+08	0.15247	ID=promoter_29642;
2	1E+08	2_133241385	292	150	294	171	221	233	0.524325	1	120	77	82	62	99	72	0.29121341	1	156	92.3115842								
2	1E+08	2_133331244	15	76	3	23	46	13	5.45E-06	0.00069	35	24	88	0	30	44	0.14806222	1	33	32.4521626								

2	1E+08	2_133403402	148	61	102	94	105	98	0.264665	1	18	0	2	36	9	19	0.11219606	1	57.625	53.3504251					1.3E+08	1.3E+08	0.10594	ID=promoter_29646;
2	1E+08	2_133450907	14	10	9	24	12	17	0.88186	1	1	41	30	23	21	27	0.52862919	1	19	12.9614814								
2	1E+08	2_133458822	210	48	198	133	129	166	0.732143	1	106	24	35	27	65	31	0.00815344	0.3345322	97.625	76.3618033								
2	1E+08	2_133640597	19	4	10	7	12	9	0.592799	1	2	3	12	1	3	7	0.3829009	1	7.25	6.13537751					1.3E+08	1.3E+08	0.12487	ID=promoter_29662;
2	1E+08	2_133733518	268	40	143	215	154	179	0.996584	1	80	65	40	16	73	28	2.24E-04	0.0182034	108.38	91.5094648					1.3E+08	1.3E+08	0.32111	ID=promoter_29664;
2	1E+08	2_133823785	129	101	126	153	115	140	0.957284	1	137	114	92	64	126	78	0.01865343	0.6441088	114.5	28.2084081					1.3E+08	1.3E+08	0.82061	ID=promoter_29667;
2	1E+08	2_133916749	41	14	27	29	28	28	0.83651	1	0	5	13	1	3	7	0.3829009	1	16.25	14.7817455								
2	1E+08	2_134094651	9	14	7	8	12	8	0.45534	1	0	6	0	2	3	1	0.65196691	1	5.75	4.86239212								
2	1E+08	2_134115416	0	60	20	13	30	17	0.049763	1	25	19	27	4	22	16	0.76021399	1	21	18.4080728					1.3E+08	1.3E+08	0.14197	ID=promoter_29681;
2	1E+08	2_134199143	5	41	10	3	23	7	0.003444	0.1741	1	0	18	0	1	9	0.02974549	0.9232309	9.75	14.0382132								
2	1E+08	2_134213231	54	54	65	112	54	89	0.14046	1	183	132	138	136	158	137	0.84868705	1	109.25	47.1615764								
2	1E+08	2_134275518	94	18	60	60	56	60	0.888	1	11	46	17	0	29	9	0.01067645	0.4139111	38.25	32.0301198								
2	1E+08	2_134357099	1	6	0	2	4	1	0.312866	1	29	2	0	45	16	23	0.39752759	1	10.625	16.9363303								
2	1E+08	2_134365706	86	83	57	13	85	35	3.48E-07	#######	47	69	80	48	58	64	0.62952401	1	60.375	24.5411462								
2	1E+08	2_134402926	141	85	220	74	113	147	0.709902	1	79	44	52	1	62	27	0.00359287	0.1775262	87	66.8815584					1.3E+08	1.3E+08	0.09993	ID=promoter_29686;
2	1E+08	2_134561680	64	34	90	71	49	81	0.16288	1	23	40	15	9	32	12	0.02181216	0.7347023	43.25	28.9420603	1E+08	1.3E+08	ID=ENSP00000386711-D2;JAKMIP1;NM_001099433;	JAKMIP1				
2	1E+08	2_134581065	15	1	0	14	8	7	0.850897	1	18	25	24	1	22	13	0.43054584	1	12.25	10.3337174	1E+08	1.3E+08	ID=ENSP00000386711-D2;JAKMIP1;NM_001099433;	JAKMIP1				
2	1E+08	2_134655860	64	26	38	27	45	33	0.117793	1	16	3	8	0	10	4	0.34296513	1	22.75	21.1575721								
2	1E+08	2_134785822	0	1	13	29	1	21	4.92E-04	0.03466	1	15	23	27	8	25	0.00638831	0.2759882	13.625	12.0111555								
2	1E+08	2_134803078	1	11	10	8	6	9	0.896349	1	39	61	19	3	50	11	1.59E-05	0.001895	19	20.7364414								
2	1E+08	2_134810185	4	12	16	10	8	13	0.766846	1	14	6	37	19	10	28	0.006881	0.2937185	14.75	10.2643628								
2	1E+08	2_135205850	16	61	27	33	39	30	0.216196	1	106	56	99	46	81	73	0.9848103	1	55.5	32.6058715								
2	1E+08	2_135240483	26	21	28	30	24	29	0.994218	1	76	6	14	35	41	25	0.25084268	1	29.5	20.9489175	1E+08	1.4E+08	ID=ENSP00000342023;FBXO38;NM_205836;	FBXO38	1.4E+08	1.4E+08	0.34755	ID=promoter_29715;
2	1E+08	2_135263040	4	51	10	7	28	9	0.001431	0.08503	30	2	54	15	16	35	0.01355156	0.5007583	21.625	20.9416707	1E+08	1.4E+08	ID=ENSP00000342023;FBXO38;NM_205836;	FBXO38	1.4E+08	1.4E+08	0.3341	ID=promoter_29724;
2	1E+08	2_135267765	16	19	5	31	18	18	0.89158	1	46	13	18	46	30	32	0.85844914	1	24.25	15.2292013	1E+08	1.4E+08	ID=ENSP00000342023;FBXO38;NM_205836;	FBXO38	1.4E+08	1.4E+08	0.79871	ID=promoter_29725;
2	1E+08	2_135287548	8	31	8	4	20	6	0.006754	0.29841	7	15	2	3	11	3	0.13591373	1	9.75	9.49812011					1.4E+08	1.4E+08	0.54223	ID=promoter_29732;

2	1E+08	2_135297945	183	41	169	121	112	145	0.734841	1	45	11	19	29	28	24	0.96104627	1	77.25	69.6721916								
2	1E+08	2_135342759	46	11	21	41	29	31	0.937432	1	26	21	8	1	24	5	0.00395202	0.1881563	21.875	15.6427756	1E+08	1.4E+08	ID=ENSP00000353915;HTR4;NM_001040173;	HTR4				
2	1E+08	2_135347750	76	177	83	109	127	96	0.004689	0.22281	174	135	147	151	155	149	0.95123787	1	131.5	38.633812	1E+08	1.4E+08	ID=ENSP00000353915;HTR4;NM_001040173;	HTR4				
2	1E+08	2_135359757	17	17	5	19	17	12	0.395844	1	25	1	6	18	13	12	0.99994263	1	13.5	8.38365757	1E+08	1.4E+08	ID=ENSP00000353915;HTR4;NM_001040173;	HTR4				
2	1E+08	2_135726090	39	29	21	13	34	17	0.012579	0.48874	9	8	25	31	9	28	0.00358036	0.1770746	21.875	11.1795413					1.4E+08	1.4E+08	0.3372	ID=promoter_29773;
2	1E+08	2_135734222	60	47	112	101	54	107	0.006428	0.2884	56	119	81	45	88	63	0.29140519	1	77.625	29.8373567								
2	1E+08	2_135741125	0	2	4	15	1	10	0.048828	1	16	0	12	1	8	7	0.9937864	1	6.25	6.90238054								
2	1E+08	2_135938412	9	57	25	22	33	24	0.19836	1	106	56	27	43	81	35	5.68E-04	0.0393693	43.125	30.4979508	1E+08	1.4E+08	ID=ENSP00000296721;AFAP1L1;NM_152406;	AFAP1L1	1.4E+08	1.4E+08	0.3394	ID=promoter_29814;
2	1E+08	2_135968021	0	9	0	0	5	0	0.05327	1	0	0	3	38	0	21	1.21E-05	0.0014894	6.25	13.2098448	1E+08	1.4E+08	ID=ENSP00000329558;GRPEL2;NM_152407;	GRPEL2	1.4E+08	1.4E+08	0.22232	ID=promoter_29822;
2	1E+08	2_135992707	33	31	80	35	32	58	0.13755	1	14	43	15	12	29	14	0.12465658	1	32.875	22.1774499					1.4E+08	1.4E+08	0.35516	ID=promoter_29826;
2	1E+08	2_135993146	72	25	16	20	49	18	4.95E-05	0.00479	1	11	2	12	6	7	0.91781435	1	19.875	22.5985935					1.4E+08	1.4E+08	0.35516	ID=promoter_29826;
2	1E+08	2_135998519	495	306	669	738	401	704	7.15E-10	#######	315	406	863	438	361	651	6.89E-24	5.75E-21	528.75	205.262028					1.4E+08	1.4E+08	0.28152	ID=promoter_29830;
2	1E+08	2_136007756	51	12	16	56	32	36	0.985347	1	10	40	27	2	25	15	0.40601431	1	26.75	20.1618452								
2	1E+08	2_136014650	113	126	47	126	120	87	0.002605	0.1394	63	48	94	63	56	79	0.05334532	1	85	33.8230691					1.4E+08	1.4E+08	0.5435	ID=promoter_29834;
2	1E+08	2_136021393	84	74	108	293	79	201	1.74E-08	#######	53	55	148	49	54	99	2.15E-04	0.0176022	108	81.8290902					1.4E+08	1.4E+08	0.30913	ID=promoter_29836;
2	1E+08	2_136031028	15	453	568	802	234	685	1.12E-34	#######	1	219	142	107	110	125	0.29618563	1	288.38	289.224548					1.4E+08	1.4E+08	0.2372	ID=promoter_29840;
2	1E+08	2_136035155	194	81	104	73	138	89	4.67E-05	0.00457	36	46	72	26	41	49	0.48482588	1	79	53.0309882					1.4E+08	1.4E+08	0.47353	ID=promoter_29842;
2	1E+08	2_136043252	28	5	3	1	17	2	7.31E-04	0.04855	1	25	5	4	13	5	0.22538616	1	9	10.9414024								
2	1E+08	2_136045314	5	3	35	33	4	34	6.54E-05	0.00616	8	6	28	23	7	26	0.00198609	0.1088597	17.625	13.5006614					1.4E+08	1.4E+08	0.24549	ID=promoter_29844;
2	1E+08	2_136132667	0	36	7	0	18	4	0.003432	0.17404	0	12	5	2	6	4	0.87665919	1	7.75	12.174329								
2	1E+08	2_136133820	998	574	645	735	786	690	1.79E-07	#######	413	324	456	157	369	307	0.37463755	1	537.75	260.89393								
2	1E+08	2_136185479	152	94	125	128	123	127	0.601249	1	0	7	0	0	4	0	0.15659576	1	63.25	67.6054732	1E+08	1.4E+08	ID=ENSP00000328083;;NM_001001669;		1.4E+08	1.4E+08	0.22197	ID=promoter_29859;
2	1E+08	2_136219028	24	17	18	16	21	17	0.520268	1	23	18	25	22	21	24	0.78178162	1	20.375	3.50255009								
2	1E+08	2_136223437	94	34	89	48	64	69	0.888648	1	19	17	4	0	18	2	0.00297675	0.1507727	38.125	36.3610368					1.4E+08	1.4E+08	0.32067	ID=promoter_29866;

2	1E+08	2_136255120	3	41	22	14	22	18	0.522468	1	33	12	52	15	23	34	0.21851877	1	24	16.5788161								
2	1E+08	2_136260915	79	64	112	50	72	81	0.967332	1	22	67	54	29	45	42	0.99950351	1	59.625	28.4601852								
2	1E+08	2_136277240	242	103	198	175	173	187	0.740712	1	43	25	21	53	34	37	0.79599102	1	107.5	86.4175908					1.4E+08	1.4E+08	0.33027	ID=promoter_29877;
2	1E+08	2_136284599	16	0	11	19	8	15	0.557319	1	16	10	15	18	13	17	0.64388579	1	13.125	6.15136454					1.4E+08	1.4E+08	0.21073	ID=promoter_29880;
2	1E+08	2_136297629	98	49	80	83	74	82	0.939018	1	24	7	10	14	16	12	0.85678722	1	45.625	36.9591956					1.4E+08	1.4E+08	0.33311	ID=promoter_29884;
2	1E+08	2_136299473	12	22	17	7	17	12	0.395844	1	47	20	46	24	34	35	0.90965436	1	24.375	14.7060483					1.4E+08	1.4E+08	0.44601	ID=promoter_29885;
2	1E+08	2_136301753	15	21	7	5	18	6	0.017303	0.62786	6	6	12	0	6	6	0.99142475	1	9	6.63324958								
2	1E+08	2_136310482	20	48	33	13	34	23	0.11872	1	24	11	39	22	18	31	0.10487282	1	26.25	12.8257553					1.4E+08	1.4E+08	0.22642	ID=promoter_29889;
2	1E+08	2_136325516	275	95	90	139	185	115	3.80E-07	#######	53	7	9	17	30	13	0.06382438	1	85.625	89.9570334								
2	1E+08	2_136332050	66	5	36	2	36	19	0.015282	0.57166	40	11	30	16	26	23	0.98669459	1	25.75	21.5588894					1.4E+08	1.4E+08	0.43494	ID=promoter_29897;
2	1E+08	2_136338406	157	14	22	23	86	23	5.01E-11	#######	81	49	41	89	65	65	0.91092096	1	59.5	47.9583152	1E+08	1.4E+08	ID=ENSP0000377855;PPARGC1B;;	PPARGC1B	1.4E+08	1.4E+08	0.77497	ID=promoter_29900;
2	1E+08	2_136341555	376	108	320	297	242	309	0.613145	1	133	98	80	87	116	84	0.22447736	1	187.38	121.910901	1E+08	1.4E+08	ID=ENSP0000377855;PPARGC1B;;	PPARGC1B	1.4E+08	1.4E+08	0.77497	ID=promoter_29900;
2	1E+08	2_136345970	4	3	20	39	4	30	3.25E-04	0.02482	2	15	32	8	9	20	0.07989336	1	15.375	13.9993622	1E+08	1.4E+08	ID=ENSP0000377855;PPARGC1B;;	PPARGC1B	1.4E+08	1.4E+08	0.75481	ID=promoter_29901;
2	1E+08	2_136368199	12	92	17	18	52	18	1.29E-05	0.00149	52	28	36	34	40	35	0.96931568	1	36.125	25.9694188	1E+08	1.4E+08	ID=ENSP0000360502-D3;PDE6C;NM_006204;	PDE6C	1.4E+08	1.4E+08	0.77976	ID=promoter_29907;
2	1E+08	2_136372608	1	5	5	5	3	5	0.88968	1	0	10	12	2	5	7	0.77775394	1	5	4.20883425	1E+08	1.4E+08	ID=ENSP0000360502-D3;PDE6C;NM_006204;	PDE6C	1.4E+08	1.4E+08	0.26659	ID=promoter_29909;
2	1E+08	2_136372660	6	37	34	13	22	24	0.97026	1	37	38	0	1	38	1	9.20E-08	1.93E-05	20.75	17.318446	1E+08	1.4E+08	ID=ENSP0000360502-D3;PDE6C;NM_006204;	PDE6C	1.4E+08	1.4E+08	0.26659	ID=promoter_29909;
2	1E+08	2_136385121	11	18	4	0	15	2	0.002248	0.12273	0	13	10	3	7	7	0.99000271	1	7.375	6.58868077	1E+08	1.4E+08	ID=ENSP0000360502-D3;PDE6C;NM_006204;	PDE6C	1.4E+08	1.4E+08	0.43721	ID=promoter_29913;
2	1E+08	2_136407454	742	362	574	619	552	597	0.388668	1	354	148	161	84	251	123	2.92E-08	6.71E-06	380.5	243.838354	1E+08	1.4E+08	ID=ENSP0000360502-D3;PDE6C;NM_006204;	PDE6C	1.4E+08	1.4E+08	0.30208	ID=promoter_29920;
2	1E+08	2_136420006	18	65	26	36	42	31	0.142577	1	6	24	30	32	15	31	0.03250921	0.9922526	29.625	17.0540108	1E+08	1.4E+08	ID=ENSP0000360502-D3;PDE6C;NM_006204;	PDE6C				
2	1E+08	2_136446757	563	213	446	379	388	413	0.389258	1	218	153	214	59	186	137	0.1229343	1	280.63	166.948185					1.4E+08	1.4E+08	0.59881	ID=promoter_29931;

2	1E+08	2_136450024	150	62	155	144	106	150	0.333881	1	71	58	68	4	65	36	0.04318173	1	89	54.48722								
2	1E+08	2_136460516	1590	760	1147	1149	1175	1148	5.97E-05	0.00568	803	321	410	277	562	344	7.95E-09	2.00E-06	807.13	466.381646					1.4E+08	1.4E+08	0.82179	ID=promoter_29935;
2	1E+08	2_136462176	18	7	6	7	13	7	0.234546	1	34	16	45	20	25	33	0.40760787	1	19.125	13.984047					1.4E+08	1.4E+08	0.82179	ID=promoter_29935;
2	1E+08	2_136475687	0	59	28	16	30	22	0.240296	1	10	25	3	1	18	2	0.00297675	0.1507727	17.75	19.7683007					1.4E+08	1.4E+08	0.1824	ID=promoter_29939;
2	1E+08	2_136476358	21	48	19	10	35	15	0.003067	0.15869	20	61	25	4	41	15	0.00610396	0.2666968	26	19.1236577					1.4E+08	1.4E+08	0.1824	ID=promoter_29939;
2	1E+08	2_136487870	11	22	12	2	17	7	0.051092	1	0	16	6	0	8	3	0.3843038	1	8.625	8.01672359					1.4E+08	1.4E+08	0.36624	ID=promoter_29944;
2	1E+08	2_136492546	13	47	17	17	30	17	0.049763	1	35	30	56	11	33	34	0.91076368	1	28.25	16.7054311					1.4E+08	1.4E+08	0.62485	ID=promoter_29946;
2	1E+08	2_136519745	24	2	44	19	13	32	0.070074	1	12	12	9	7	12	8	0.76853133	1	16.125	13.1739733					1.4E+08	1.4E+08	0.89207	ID=promoter_29952;
2	1E+08	2_136530150	633	228	605	385	431	495	0.950117	1	47	44	57	66	46	62	0.15396776	1	258.13	252.316376	1E+08	1.4E+08	ID=ENSP0000377844;CSF1R;NM_005211;	CSF1R	1.4E+08	1.4E+08	0.53294	ID=promoter_29955;
2	1E+08	2_136547788	8	5	3	9	7	6	0.851931	1	0	19	2	4	10	3	0.19568363	1	6.25	5.94618725	1E+08	1.4E+08	ID=ENSP0000377844;CSF1R;NM_005211;	CSF1R	1.4E+08	1.4E+08	0.23529	ID=promoter_29959;
2	1E+08	2_136548083	236	91	106	122	164	114	8.14E-05	0.0074	147	95	70	74	121	72	0.01099969	0.424822	117.63	54.0130606	1E+08	1.4E+08	ID=ENSP0000377844;CSF1R;NM_005211;	CSF1R	1.4E+08	1.4E+08	0.23529	ID=promoter_29959;
2	1E+08	2_136566080	54	33	33	73	44	53	0.991545	1	21	15	15	30	18	23	0.59196476	1	34.25	20.1050811					1.4E+08	1.4E+08	0.77154	ID=promoter_29961;
2	1E+08	2_136570557	1	15	20	19	8	20	0.179808	1	33	33	26	10	33	18	0.18694358	1	19.625	11.0832886					1.4E+08	1.4E+08	0.26285	ID=promoter_29962;
2	1E+08	2_136580155	11	23	5	2	17	4	0.005755	0.26328	21	7	21	0	14	11	0.91900834	1	11.25	9.23889295								
2	1E+08	2_136580190	148	96	158	131	122	145	0.994255	1	0	6	20	5	3	13	0.02959638	0.9232309	70.5	69.6357872								
2	1E+08	2_136597230	2	13	8	5	8	7	0.850897	1	38	24	17	36	31	27	0.97229028	1	17.875	13.6845847					1.4E+08	1.4E+08	0.23824	ID=promoter_29970;
2	1E+08	2_136607098	14	2	6	3	8	5	0.532535	1	0	7	12	1	4	7	0.58783259	1	5.625	5.15301576					1.4E+08	1.4E+08	0.48068	ID=promoter_29972;
2	1E+08	2_136638400	24	29	39	36	27	38	0.7689	1	53	33	65	3	43	34	0.78602279	1	35.25	18.6145105					1.4E+08	1.4E+08	0.66743	ID=promoter_29984;
2	1E+08	2_136641114	23	4	41	132	14	87	7.40E-10	#######	21	29	32	17	25	25	0.96475192	1	37.375	39.7669551					1.4E+08	1.4E+08	0.49725	ID=promoter_29985;
2	1E+08	2_136654536	102	16	37	81	59	59	0.686493	1	22	12	7	11	17	9	0.38755874	1	36	35.8966771								
2	1E+08	2_136659872	83	48	82	83	66	83	0.914627	1	152	177	107	24	165	66	2.15E-08	5.05E-06	94.5	50.4579032					1.4E+08	1.4E+08	0.3867	ID=promoter_29989;
2	1E+08	2_136707121	3	12	1	28	8	15	0.557319	1	10	15	31	2	13	17	0.64388579	1	12.75	11.5108644					1.4E+08	1.4E+08	0.24043	ID=promoter_30002;
2	1E+08	2_136708601	263	1762	2762	4976	1013	3869	########	#######	339	1651	6156	1669	995	3913	0	0	2447.3	2110.29421					1.4E+08	1.4E+08	0.19059	ID=promoter_30003;
2	1E+08	2_136710802	83	29	61	25	56	43	0.109057	1	19	5	7	11	12	9	0.89054267	1	30	27.8567766					1.4E+08	1.4E+08	0.25098	ID=promoter_30004;
2	1E+08	2_136756909	535	207	586	331	371	459	0.746957	1	21	25	57	40	23	49	0.00321925	0.1617361	225.25	233.619196								

2	1E+08	2_136796208	70	21	50	49	46	50	0.932872	1	0	15	17	44	8	31	4.57E-04	0.0328393	33.25	23.4444633								
2	1E+08	2_136836227	0	33	4	5	17	5	0.013234	0.50759	0	13	11	9	7	10	0.67636194	1	9.375	10.676242					1.4E+08	1.4E+08	0.30315	ID=promoter_30041;
2	1E+08	2_136851751	19	59	29	27	39	28	0.134871	1	31	22	9	2	27	6	0.00268813	0.1381379	24.75	17.0775542					1.4E+08	1.4E+08	0.30367	ID=promoter_30045;
2	1E+08	2_136866128	44	73	48	53	59	51	0.276863	1	42	98	38	78	70	58	0.8185593	1	59.25	21.3457925					1.4E+08	1.4E+08	0.41952	ID=promoter_30050;
2	1E+08	2_136873667	474	135	397	349	305	373	0.863986	1	131	128	191	83	130	137	0.57757992	1	236	148.159181					1.4E+08	1.4E+08	0.48486	ID=promoter_30053;
2	1E+08	2_138284532	204	123	108	132	164	120	3.86E-04	0.02851	202	123	111	39	163	75	1.99E-06	0.0003099	130.25	53.3017019								
2	1E+08	2_138451167	78	31	63	43	55	53	0.592668	1	62	16	41	4	39	23	0.22193731	1	42.25	25.0128538								
2	1E+08	2_138452393	0	8	0	0	4	0	0.09576	1	0	23	13	0	12	7	0.61698677	1	5.5	8.61891607	1E+08	1.4E+08	ID=ENSP00000377010;MGAT1;NM_001114619;	MGAT1				
2	1E+08	2_139846494	0	25	6	1	13	4	0.041893	1	0	2	7	1	1	4	0.36109265	1	5.25	8.41342465	1E+08	1.4E+08	ID=ENSP00000372191;SIRT3;NM_012239;	SIRT3	1.4E+08	1.4E+08	0.15556	ID=promoter_30068;
2	1E+08	2_139873896	653	478	655	905	566	780	0.01399	0.53231	68	11	133	7	40	70	0.00492648	0.2249494	363.75	351.915879								
2	1E+08	2_140126481	18	8	24	36	13	30	0.115644	1	5	3	9	5	4	7	0.58783259	1	13.5	11.6004926								
2	1E+08	2_140132075	23	9	34	52	16	43	0.014369	0.54261	4	5	13	11	5	12	0.17891578	1	18.875	16.6942034								
3	36170	3_36170	666	225	441	342	446	392	1.59E-04	0.01329	84	48	95	73	66	84	0.15387023	1	246.75	221.119069					35871	36656	0.19471	ID=promoter_30108;
3	45539	3_45539	1	40	2	12	21	7	0.0088	0.36783	0	44	3	15	22	9	0.10444527	1	14.625	17.7598061	42905	114645	ID=ENSP00000385749;PRKAR1B;;	PRKAR1B	41712	49158	0.9438	ID=promoter_30110;
3	50955	3_50955	4793	2933	537	338	3863	438	0	0	2539	2312	267	162	2426	215	0	0	1735.1	1681.07444	42905	114645	ID=ENSP00000385749;PRKAR1B;;	PRKAR1B	49607	51153	0.3067	ID=promoter_30111;
3	56110	3_56110	23	0	19	10	12	15	0.986595	1	0	0	3	0	0	2	0.34003807	1	6.875	9.41787814	42905	114645	ID=ENSP00000385749;PRKAR1B;;	PRKAR1B	54842	58290	0.60878	ID=promoter_30113;
3	97742	3_97742	193	94	181	159	144	170	0.998375	1	124	93	119	52	109	86	0.53456821	1	126.88	48.16471	42905	114645	ID=ENSP00000385749;PRKAR1B;;	PRKAR1B				
3	99745	3_99745	7	33	6	16	20	11	0.118302	1	55	0	58	2	28	30	0.85900118	1	22.125	23.6246693	42905	114645	ID=ENSP00000385749;PRKAR1B;;	PRKAR1B				
3	104358	3_104358	1	51	17	2	26	10	0.006911	0.3015	74	34	82	4	54	43	0.75792271	1	33.125	32.7083978	42905	114645	ID=ENSP00000385749;PRKAR1B;;	PRKAR1B	103474	104842	0.30478	ID=promoter_30130;
3	106101	3_106101	21	11	46	49	16	48	0.003374	0.17222	16	10	0	1	13	1	0.00904697	0.3617342	19.25	18.790195	42905	114645	ID=ENSP00000385749;PRKAR1B;;	PRKAR1B	105111	106718	0.35808	ID=promoter_30131;
3	118382	3_118382	14	24	15	11	19	13	0.317047	1	0	28	6	8	14	7	0.39930956	1	13.25	9.23889295								
3	118759	3_118759	10	10	4	11	10	8	0.719353	1	35	1	0	0	18	0	2.38E-04	0.0190698	8.875	11.5441698								
3	128223	3_128223	15	49	14	21	32	18	0.038646	1	36	19	42	7	28	25	0.9907189	1	25.375	15.0327024	121339	182548	ID=ENSP00000297440;HEATR2;NM_017802;	HEATR2				
3	130111	3_130111	21	1	31	18	11	25	0.176768	1	0	21	19	7	11	13	0.83819512	1	14.75	10.9381377	121339	182548	ID=ENSP00000297440;HEATR2;NM_017802;	HEATR2	128809	136904	0.95985	ID=promoter_30137;

3	135736	3_135736	16	32	30	47	24	39	0.450945	1	66	23	13	27	45	20	0.01997236	0.6799503	31.75	17.35141	121339	182548	ID=ENSP000002974 40;HEATR 2;NM_017 802;	HEATR2	128809	136904	0.95985	ID=promoter_ 30137;
3	141282	3_141282	17	14	19	25	16	22	0.889379	1	80	2	39	22	41	31	0.69213115	1	27.25	23.7231051	121339	182548	ID=ENSP000002974 40;HEATR 2;NM_017 802;	HEATR2	141010	142682	0.34093	ID=promoter_ 30139;
3	180668	3_180668	4	5	9	24	5	17	0.090491	1	0	14	5	0	7	3	0.51857467	1	7.625	8.0522845	121339	182548	ID=ENSP000002974 40;HEATR 2;NM_017 802;	HEATR2				
3	182323	3_182323	16	16	6	22	16	14	0.724026	1	29	22	15	16	26	16	0.43033428	1	17.75	6.73477118	121339	182548	ID=ENSP000002974 40;HEATR 2;NM_017 802;	HEATR2	182021	183850	0.30564	ID=promoter_ 30151;
3	207704	3_207704	213	43	191	145	128	168	0.631622	1	4	56	52	0	30	26	0.96889533	1	88	83.3478083					206649	208135	0.31783	ID=promoter_ 30156;
3	207798	3_207798	1860	766	1839	1301	1313	1570	0.876089	1	583	571	689	635	577	662	8.83E-04	0.0572814	1030.5	556.688166					206649	208135	0.31783	ID=promoter_ 30156;
3	211540	3_211540	14	24	13	6	19	10	0.108738	1	15	11	43	28	13	36	0.0018128	0.1007146	19.25	11.9013805					210682	214546	0.70238	ID=promoter_ 30157;
3	214337	3_214337	1	18	2	16	10	9	0.84607	1	42	37	25	3	40	14	0.00488665	0.2236314	18	15.8564993					210682	214546	0.70238	ID=promoter_ 30157;
3	216768	3_216768	97	48	49	101	73	75	0.72239	1	169	111	128	12	140	70	1.03E-04	0.0094698	89.375	50.4577263					214926	218211	0.65471	ID=promoter_ 30158;
3	312955	3_312955	169	115	196	7	142	102	6.88E-04	0.04634	111	74	0	5	93	3	1.27E-17	7.82E-15	84.625	76.3281216					310748	314430	0.75255	ID=promoter_ 30182;
3	372514	3_372514	47	113	74	109	80	92	0.991678	1	81	158	94	78	120	86	0.1885037	1	94.25	33.1996127								
3	436932	3_436932	105	83	92	92	94	92	0.465821	1	27	68	46	39	48	43	0.98678626	1	69	28.645618					436523	437517	0.19979	ID=promoter_ 30213;
3	437324	3_437324	393	324	529	471	359	500	0.045503	1	438	398	466	125	418	296	0.00179112	0.099899	393	124.524237					436523	437517	0.19979	ID=promoter_ 30213;
3	463472	3_463472	58	8	37	22	33	30	0.598685	1	1	0	2	2	1	2	0.81304206	1	16.25	21.3055995					460715	464610	0.71406	ID=promoter_ 30222;
3	478339	3_478339	8	6	0	11	7	6	0.851931	1	0	0	1	26	0	14	5.26E-04	0.0367435	6.5	8.94427191					472852	479194	0.92869	ID=promoter_ 30225;
3	486021	3_486021	21	29	45	26	25	36	0.732372	1	49	33	2	8	41	5	2.83E-06	0.0004196	26.625	16.3439591					485377	486666	0.27369	ID=promoter_ 30227;
3	491196	3_491196	36	43	31	66	40	49	0.979456	1	22	87	147	29	55	88	0.00570204	0.2548307	57.625	42.0881303					487218	491808	0.83306	ID=promoter_ 30228;
3	502841	3_502841	22	7	30	31	15	31	0.189122	1	0	12	0	1	6	1	0.20114407	1	12.875	13.1631249					500815	504971	0.78177	ID=promoter_ 30232;
3	524016	3_524016	17	9	5	8	13	7	0.234546	1	0	14	22	22	7	22	0.01139713	0.4370832	12.125	7.98994904	519667	524414	ID=ENSP000003860 31;ZFAND 2A;;	ZFAND2A	523726	524568	0.11	ID=promoter_ 30239;
3	550895	3_550895	66	22	49	38	44	44	0.755386	1	10	42	15	11	26	13	0.18179217	1	31.625	20.3325039								
3	552972	3_552972	62	12	53	21	37	37	0.789862	1	86	23	20	32	55	26	0.01564739	0.5592438	38.625	25.7789366					552518	554139	0.37648	ID=promoter_ 30247;
3	572502	3_572502	11	17	18	15	14	17	0.995406	1	43	12	9	14	28	12	0.0723178	1	17.375	10.7827574					571861	574299	0.62829	ID=promoter_ 30252;
3	577771	3_577771	540	288	611	378	414	495	0.959435	1	177	166	226	103	172	165	0.95192369	1	311.13	183.975493					576675	579952	0.60568	ID=promoter_ 30254;
3	579571	3_579571	2991	1548	3784	2592	2270	3188	4.34E-10	#######	1934	1007	1753	611	1471	1182	0.00121808	0.0738454	2027.5	1048.41309					576675	579952	0.60568	ID=promoter_ 30254;

3	598126	3_598126	51	116	82	63	84	73	0.170434	1	67	44	113	20	56	67	0.36865297	1	69.5	33.1705549	597078	598141	0.2335	ID=promoter_30262;
3	616211	3_616211	3	1	4	8	2	6	0.490921	1	0	21	9	0	11	5	0.40227598	1	5.75	7.04576874				
3	623160	3_623160	36	29	28	6	33	17	0.018001	0.65052	14	21	8	2	18	5	0.0406475	1	18	12.3635409				
3	624271	3_624271	14	0	8	4	7	6	0.851931	1	1	8	0	11	5	6	0.91271846	1	5.75	5.31171213	623807	624990	0.21178	ID=promoter_30270;
3	629063	3_629063	0	1	0	1	1	1	0.993645	1	20	0	25	1	10	13	0.72167512	1	6	10.2817453				
3	652641	3_652641	0	7	1	8	4	5	0.995512	1	14	7	12	0	11	6	0.57179039	1	6.125	5.38350656				
3	682725	3_682725	438	311	519	318	375	419	0.791708	1	517	301	333	222	409	278	3.24E-04	0.0246729	369.88	108.662567	682418	684978	0.37849	ID=promoter_30284;
3	694413	3_694413	474	27	289	201	251	245	0.122668	1	37	29	35	57	33	46	0.19775469	1	143.63	165.177772	693789	694751	0.19579	ID=promoter_30286;
3	711961	3_711961	62	43	25	22	53	24	3.15E-04	0.02414	1	19	7	67	10	37	1.47E-04	0.0127053	30.75	24.3119606	709905	712272	0.44297	ID=promoter_30291;
3	785439	3_785439	28	44	29	28	36	29	0.317511	1	34	32	12	75	33	44	0.28389463	1	35.25	18.336147	783517	787111	0.66675	ID=promoter_30312;
3	786963	3_786963	3	15	13	3	9	8	0.848833	1	19	10	5	24	15	15	0.97869956	1	11.5	7.70899289	783517	787111	0.66675	ID=promoter_30312;
3	862275	3_862275	203	183	180	137	193	159	0.004095	0.20009	12	16	4	33	14	19	0.55289753	1	96	87.5410108	862139	862765	0.14112	ID=promoter_30333;
3	864374	3_864374	3	2	7	11	3	9	0.343967	1	1	13	5	6	7	6	0.99011599	1	6	4.24264069	863854	864653	0.19216	ID=promoter_30334;
3	930090	3_930090	44	87	55	44	66	50	0.063172	1	19	84	94	35	52	65	0.27250363	1	57.75	27.421316				
3	955905	3_955905	123	57	86	104	90	95	0.773188	1	29	21	23	24	25	24	0.99255599	1	58.375	40.9108351	954627	956257	0.33403	ID=promoter_30357;
3	966113	3_966113	5	47	13	41	26	27	0.906335	1	27	25	29	1	26	15	0.33618131	1	23.5	16.3095064	957633	966261	0.97144	ID=promoter_30358;
3	998968	3_998968	30	37	7	37	34	22	0.087717	1	32	13	10	54	23	32	0.32689554	1	27.5	16.2392822	997967	999690	0.41706	ID=promoter_30366;
3	1E+06	3_100487	39	25	41	59	32	50	0.445847	1	21	4	28	5	13	17	0.64388579	1	27.75	18.5684063				
3	1E+06	3_1017192	101	36	86	139	69	113	0.089967	1	0	30	30	2	15	16	0.92652531	1	53	50.0656712	1016778	1017871	0.22322	ID=promoter_30372;
3	1E+06	3_1043445	46	57	49	59	52	54	0.821444	1	143	34	61	57	89	59	0.13349143	1	63.25	33.418344	1040869	1045049	0.68068	ID=promoter_30378;
3	1E+06	3_105573	235	101	242	198	168	220	0.560827	1	130	17	73	33	74	53	0.35569714	1	128.63	88.192869				
3	1E+06	3_1067782	90	49	67	64	70	66	0.443462	1	5	1	18	53	3	36	2.36E-07	4.58E-05	43.375	32.0577047	1065297	1068015	0.54794	ID=promoter_30386;
3	1E+06	3_106860	5	14	8	17	10	13	0.970528	1	1	4	4	19	3	12	0.04708211	1	9	6.76123404				
3	1E+06	3_1082631	1	28	5	12	15	9	0.269309	1	13	47	28	4	30	16	0.19481252	1	17.25	15.8181632	1080830	1082697	0.48059	ID=promoter_30391;
3	1E+06	3_1091878	32	47	32	14	40	23	0.020841	0.72739	19	34	58	47	27	53	0.0051219	0.2332014	35.375	14.7932176	1090736	1092134	0.25743	ID=promoter_30394;
3	1E+06	3_1096042	17	26	33	18	22	26	0.999662	1	25	5	22	13	15	18	0.76034467	1	19.875	8.62616452	1093973	1096586	0.50427	ID=promoter_30396;
3	1E+06	3_1096207	41	15	30	31	28	31	0.975765	1	68	14	52	4	41	28	0.4570143	1	31.875	21.3102088	1093973	1096586	0.50427	ID=promoter_30396;
3	1E+06	3_1110958	98	31	98	118	65	108	0.083315	1	73	69	29	17	71	23	2.70E-05	0.0029792	66.625	37.4201531	1110428	1111303	0.30752	ID=promoter_30401;
3	1E+06	3_111303	3	20	12	7	12	10	0.724827	1	13	9	10	34	11	22	0.10262264	1	13.5	9.63624112				
3	1E+06	3_1125656	8	1	9	2	5	6	0.999292	1	23	10	16	4	17	10	0.5154613	1	9.125	7.41499446	1124573	1126063	0.22163	ID=promoter_30407;
3	1E+06	3_1125718	177	55	173	285	116	229	2.32E-05	0.00251	96	60	72	42	78	57	0.39204356	1	120	84.5762547	1124573	1126063	0.22163	ID=promoter_30407;

3	1E+06	3_1128836	66	61	135	115	64	125	0.003614	0.18156	42	45	56	5	44	31	0.50195989	1	65.625	41.4451014					1128391	1129916	0.38014	ID=promoter_30409;
3	1E+06	3_1129021	1	21	10	14	11	12	0.985018	1	0	8	17	29	4	23	5.91E-04	0.0404335	12.5	9.87059124					1128391	1129916	0.38014	ID=promoter_30409;
3	1E+06	3_1142470	8	0	29	32	4	31	2.18E-04	0.01746	0	27	13	6	14	10	0.81923661	1	14.375	13.1468356					1142092	1144345	0.39793	ID=promoter_30414;
3	1E+06	3_115130	2	60	5	13	31	9	3.43E-04	0.02597	1	1	0	36	1	18	2.55E-04	0.0200383	14.75	21.8942262								
3	1E+06	3_1161291	24	43	14	14	34	14	0.00261	0.13949	34	19	10	26	27	18	0.55301708	1	23	11.1994898					1156509	1161592	0.80183	ID=promoter_30419;
3	1E+06	3_1205614	0	14	6	11	7	9	0.983538	1	10	7	7	2	9	5	0.6500913	1	7.125	4.6117087					1203098	1207696	0.74072	ID=promoter_30434;
3	1E+06	3_1214905	29	81	13	27	55	20	1.29E-05	0.00149	60	46	70	11	53	41	0.68416401	1	42.125	26.2974062					1211422	1215443	0.6587	ID=promoter_30437;
3	1E+06	3_1221300	2	14	5	2	8	4	0.357158	1	1	22	15	30	12	23	0.11381058	1	11.375	10.7163093					1220622	1221323	0.21128	ID=promoter_30441;
3	1E+06	3_1232966	12	75	23	36	44	30	0.067875	1	90	30	25	9	60	17	2.90E-05	0.0031754	37.5	29.3987366					1232960	1233646	0.1723	ID=promoter_30444;
3	1E+06	3_1236990	104	47	73	71	76	72	0.428872	1	0	0	5	1	0	3	0.19828566	1	37.625	41.5690046					1236841	1237966	0.25785	ID=promoter_30446;
3	1E+06	3_1260282	13	63	24	31	38	28	0.169685	1	67	41	17	14	54	16	1.31E-04	0.0114541	33.75	21.439283					1259115	1260702	0.58157	ID=promoter_30453;
3	1E+06	3_1281213	60	37	24	11	49	18	4.95E-05	0.00479	53	14	57	47	34	52	0.07203607	1	37.875	19.4674637					1280133	1281529	0.22741	ID=promoter_30459;
3	1E+06	3_1287386	16	26	10	8	21	9	0.03105	0.99404	21	22	3	41	22	22	0.96891521	1	18.375	12.0111555	1E+06	1339518	ID=ENSP0000385935;GNA12;;	GNA12	1284389	1292468	0.96255	ID=promoter_30460;
3	1E+06	3_1318833	385	156	271	301	271	286	0.459626	1	40	25	49	75	33	62	0.00369661	0.1799564	162.75	138.849096	1E+06	1339518	ID=ENSP0000385935;GNA12;;	GNA12	1314603	1319501	0.81585	ID=promoter_30467;
3	1E+06	3_1321155	182	135	206	204	159	205	0.669773	1	67	142	129	124	105	127	0.12935428	1	148.63	46.8369131	1E+06	1339518	ID=ENSP0000385935;GNA12;;	GNA12	1320129	1321916	0.33116	ID=promoter_30468;
3	1E+06	3_1345459	262	264	417	598	263	508	2.77E-10	#######	521	440	542	149	481	346	0.00148883	0.0870157	399.13	158.75987					1343989	1347198	0.52537	ID=promoter_30473;
3	1E+06	3_136436	20	66	22	40	43	31	0.113462	1	49	18	56	38	34	47	0.20276349	1	38.625	17.7678482								
3	1E+06	3_137497	780	1494	760	733	1137	747	1.52E-34	#######	912	892	1211	562	902	887	0.45922627	1	918	298.401934								
3	1E+06	3_141384	22	41	23	52	32	38	0.998681	1	33	11	45	3	22	24	0.85866092	1	28.75	17.027289								
3	1E+06	3_1431737	0	20	3	0	10	2	0.03464	1	20	0	7	0	10	4	0.34296513	1	6.25	8.82771609					1429727	1433115	0.51585	ID=promoter_30484;
3	1E+06	3_144220	7	0	0	0	4	0	0.09576	1	62	51	56	2	57	29	0.02830192	0.8918846	22.25	28.4692817								
3	1E+06	3_145601	421	144	420	311	283	366	0.466874	1	178	189	154	80	184	117	0.00583819	0.2580785	237.13	130.270529								
3	1E+06	3_1464188	22	6	33	15	14	24	0.52503	1	0	0	0	0	0	0	1	1	9.5	12.6038543					1463965	1464533	0.20351	ID=promoter_30492;
3	1E+06	3_1467910	47	81	72	52	64	62	0.56111	1	22	39	27	42	31	35	0.72585335	1	47.75	20.4083316					1467376	1468623	0.37584	ID=promoter_30493;
3	2E+06	3_1543257	2168	1054	2754	2262	1611	2508	6.51E-18	#######	1442	961	1075	667	1202	871	2.05E-07	4.02E-05	1547.9	751.033847					1540867	1543996	0.6955	ID=promoter_30502;
3	2E+06	3_1574275	8	0	7	9	4	8	0.677894	1	4	5	1	7	5	4	0.97610903	1	5.125	3.27053949					1572694	1574688	0.25504	ID=promoter_30510;
3	2E+06	3_162095	314	100	296	163	207	230	0.852267	1	78	30	66	46	54	56	0.84115418	1	136.63	111.42318								
3	2E+06	3_166258	387	161	281	186	274	234	0.001648	0.09615	77	30	33	1	54	17	2.48E-04	0.0197712	144.5	136.342007								
3	2E+06	3_176687	120	103	61	91	112	76	9.57E-04	0.06092	10	43	15	10	27	13	0.14207	1	56.625	44.1327138								
3	2E+06	3_184563	0	0	6	9	0	8	0.033035	1	0	1	7	22	1	15	0.00126073	0.075413	5.625	7.53918336								
3	2E+06	3_1858007	2	12	11	1	7	6	0.851931	1	30	0	6	3	15	5	0.11779525	1	8.125	9.90580638					1855238	1866225	0.99228	ID=promoter_30526;

3	2E+06	3_1869758	52	30	44	51	41	48	0.99996	1	116	62	60	6	89	33	1.97E-05	0.0022887	52.625	31.4458151					1867809	1870323	0.49514	ID=promoter_30527;
3	2E+06	3_1870773	44	30	55	27	37	41	0.969029	1	25	34	59	51	30	55	0.00918129	0.3665913	40.625	13.3623939					1870551	1871469	0.19807	ID=promoter_30528;
3	2E+06	3_1880255	2	3	13	6	3	10	0.252243	1	3	6	27	2	5	15	0.05518429	1	7.75	8.58154165					1879728	1880630	0.24492	ID=promoter_30530;
3	2E+06	3_1888810	1566	639	1614	1352	1103	1483	0.002732	0.14524	493	351	560	160	422	360	0.51072551	1	841.88	576.81377					1888471	1889542	0.28282	ID=promoter_30532;
3	2E+06	3_197047	168	50	112	116	109	114	0.692961	1	62	82	70	5	72	38	0.017388	0.6125608	83.125	49.191862								
3	2E+06	3_2030217	37	11	28	0	24	14	0.105787	1	0	10	0	1	5	1	0.30486099	1	10.875	14.2872321					2029728	2031161	0.3542	ID=promoter_30559;
3	2E+06	3_2038087	8	24	5	2	16	4	0.009581	0.39335	36	10	17	62	23	40	0.05045435	1	20.5	20.1281608					2037411	2038704	0.19659	ID=promoter_30560;
3	2E+06	3_204087	0	29	13	1	15	7	0.113635	1	1	25	17	7	13	12	0.99994263	1	11.625	11.3002844								
3	2E+06	3_227315	50	60	41	13	55	27	6.65E-04	0.04502	0	12	1	0	6	1	0.20114407	1	22.125	24.433218								
3	2E+06	3_244212	106	55	102	128	81	115	0.419312	1	53	18	48	50	36	49	0.21237046	1	70	37.3898382								
3	2E+06	3_2442640	0	1	29	2	1	16	0.004036	0.19749	11	0	21	6	6	14	0.1466131	1	8.75	10.8988859					2442159	2443124	0.15499	ID=promoter_30593;
3	2E+06	3_246710	10	24	8	12	17	10	0.210142	1	8	53	42	9	31	26	0.931505	1	20.75	17.5560327								
3	2E+06	3_2474734	8	15	1	10	12	6	0.213448	1	7	1	2	3	4	3	0.96209564	1	5.875	5.02671435					2474316	2475952	0.26532	ID=promoter_30595;
3	3E+06	3_250626	11	4	5	4	8	5	0.532535	1	1	8	24	2	5	13	0.12287893	1	7.375	7.44383734								
3	3E+06	3_2513275	52	120	85	95	86	90	0.750969	1	102	72	43	41	87	42	0.00193707	0.1067879	76.25	29.178025					2512792	2513322	0.12789	ID=promoter_30604;
3	3E+06	3_255582	98	91	95	61	95	78	0.063686	1	147	80	94	135	114	115	0.81509545	1	100.13	28.0328251								
3	3E+06	3_258238	38	22	52	45	30	49	0.357262	1	80	0	16	0	40	8	7.46E-05	0.0071391	31.625	27.5470377								
3	3E+06	3_2592805	19	31	20	40	25	30	0.996463	1	12	7	29	1	10	15	0.49251655	1	19.875	13.076014					2592062	2594339	0.3513	ID=promoter_30623;
3	3E+06	3_2653432	51	29	14	31	40	23	0.020841	0.72739	51	19	44	69	35	57	0.02917818	0.915188	38.5	18.516402	3E+06	2734849	ID=ENSP00000374181-D2;SDK1;;	SDK1	2652212	2654215	0.34625	ID=promoter_30644;
3	3E+06	3_2676775	23	6	4	10	15	7	0.113635	1	0	14	0	13	7	7	0.99000271	1	8.75	7.86947085	3E+06	2734849	ID=ENSP00000374181-D2;SDK1;;	SDK1	2674588	2677700	0.57605	ID=promoter_30650;
3	3E+06	3_2719422	3	80	17	24	42	21	0.004493	0.21484	68	118	72	39	93	56	0.03671058	1	52.625	38.4928101	3E+06	2734849	ID=ENSP00000374181-D2;SDK1;;	SDK1				
3	3E+06	3_2722810	0	2	14	7	1	11	0.032372	1	40	9	28	16	25	22	0.9842524	1	14.5	13.56466	3E+06	2734849	ID=ENSP00000374181-D2;SDK1;;	SDK1	2721084	2722854	0.31708	ID=promoter_30663;
3	3E+06	3_2732321	15	52	15	7	34	11	3.70E-04	0.02765	38	4	17	4	21	11	0.29862283	1	19	17.2378321	3E+06	2734849	ID=ENSP00000374181-D2;SDK1;;	SDK1	2730287	2735945	0.84988	ID=promoter_30667;
3	3E+06	3_2733894	462	206	422	385	334	404	0.917189	1	39	53	28	9	46	19	0.00967	0.3824351	200.5	194.855991	3E+06	2734849	ID=ENSP00000374181-D2;SDK1;;	SDK1	2730287	2735945	0.84988	ID=promoter_30667;
3	3E+06	3_2735789	27	25	19	14	26	17	0.164334	1	49	1	25	15	25	20	0.88611817	1	21.875	13.8092878					2730287	2735945	0.84988	ID=promoter_30667;
3	3E+06	3_275543	7	0	12	10	4	11	0.322758	1	0	17	10	0	9	5	0.6500913	1	7	6.43650304								
3	3E+06	3_2792517	4	0	4	5	2	5	0.651182	1	28	0	1	0	14	1	0.00574567	0.2548307	5.25	9.42261717					2791629	2793151	0.23905	ID=promoter_30684;

3	3E+06	3_2811106	7	13	3	3	10	3	0.082185	1	26	19	8	0	23	4	0.00249416	0.131314	9.875	8.91928088					2810535	2811275	0.22755	ID=promoter_30686;
3	3E+06	3_2896394	48	16	34	17	32	26	0.376129	1	96	34	36	27	65	32	0.01179676	0.4484493	38.5	25.4782821					2895463	2898576	0.5762	ID=promoter_30703;
3	3E+06	3_291122	39	0	7	0	20	4	0.0012	0.07347	0	1	0	29	1	15	0.00126073	0.075413	9.5	15.5379719								
3	3E+06	3_2920533	381	159	256	239	270	248	0.020575	0.72012	549	357	231	276	453	254	7.51E-10	2.24E-07	306	120.94745					2919745	2921779	0.349	ID=promoter_30710;
3	3E+06	3_2924094	11	5	9	10	8	10	0.991043	1	16	14	1	0	15	1	0.00364468	0.1775497	8.25	5.8002463					2923735	2924569	0.19523	ID=promoter_30711;
3	3E+06	3_2995808	4	1	10	16	3	13	0.09118	1	6	4	25	0	5	13	0.12287893	1	8.25	8.4978989					2994920	2996959	0.33813	ID=promoter_30731;
3	3E+06	3_304030	7	15	5	2	11	4	0.10516	1	7	9	0	10	8	5	0.78486764	1	6.875	4.70372193								
3	3E+06	3_305756	13	19	23	13	16	18	0.992646	1	84	18	33	4	51	19	0.00210847	0.1150505	25.875	24.9481605								
3	3E+06	3_3078116	24	73	34	35	49	35	0.07705	1	70	76	100	100	73	100	0.03847355	1	64	29.7465484					3077264	3078766	0.27685	ID=promoter_30746;
3	3E+06	3_3092673	40	3	28	12	22	20	0.710339	1	0	0	0	0	0	0	1	1	10.375	15.4729026					3092430	3093413	0.19518	ID=promoter_30750;
3	3E+06	3_317219	156	43	89	137	100	113	0.963749	1	23	30	14	18	27	16	0.35958363	1	63.75	56.4566838								
3	3E+06	3_3207591	72	42	49	84	57	67	0.999931	1	27	98	33	8	63	21	1.26E-04	0.0111328	51.625	30.6358217	3E+06	3257691	ID=ENSP0000328720;FOXK1;NM_001037165;	FOXK1	3206225	3209658	0.48875	ID=promoter_30774;
3	3E+06	3_3215036	8	48	7	13	28	10	0.00287	0.14985	1	12	1	0	7	1	0.13113995	1	11.25	15.6729977	3E+06	3257691	ID=ENSP0000328720;FOXK1;NM_001037165;	FOXK1				
3	3E+06	3_3224998	0	104	0	9	52	5	6.56E-11	#######	1	15	9	1	8	5	0.78486764	1	17.375	35.4358072	3E+06	3257691	ID=ENSP0000328720;FOXK1;NM_001037165;	FOXK1				
3	3E+06	3_3229541	16	0	32	31	8	32	0.004292	0.20739	0	0	14	1	0	8	0.01336935	0.4945372	11.75	13.8021737	3E+06	3257691	ID=ENSP0000328720;FOXK1;NM_001037165;	FOXK1	3228108	3231462	0.58697	ID=promoter_30780;
3	3E+06	3_3236338	1	24	13	1	13	7	0.234546	1	66	56	39	32	61	36	0.09555509	1	29	24.0713226	3E+06	3257691	ID=ENSP0000328720;FOXK1;NM_001037165;	FOXK1	3235839	3236871	0.19412	ID=promoter_30782;
3	3E+06	3_3257917	86	25	40	25	56	33	0.005995	0.27181	27	30	52	35	29	44	0.11481867	1	40	20.7157635					3254830	3262440	0.94441	ID=promoter_30788;
3	3E+06	3_326386	36	148	50	32	92	41	5.86E-07	#######	124	98	136	29	111	83	0.33248101	1	81.625	50.3415123								
3	3E+06	3_3277105	5	36	15	26	21	21	0.874689	1	45	45	35	35	45	35	0.73773014	1	30.25	14.1497097	3E+06	3319811	ID=ENSP0000297562;FOXK1;NM_014855;	FOXK1	3274634	3277683	0.5145	ID=promoter_30791;
3	3E+06	3_3326439	180	89	322	241	135	282	1.63E-07	#######	16	21	11	21	19	16	0.96066018	1	112.63	120.635149	3E+06	3392995	ID=ENSP0000382492;RADIL;NM_018059;	RADIL	3325392	3326687	0.33456	ID=promoter_30799;
3	3E+06	3_3336377	19	31	12	5	25	9	0.005635	0.25889	45	18	41	3	32	22	0.55606214	1	21.75	15.7910463	3E+06	3392995	ID=ENSP0000382492;RADIL;NM_018059;	RADIL	3335045	3336768	0.42035	ID=promoter_30802;
3	3E+06	3_3344025	2	1	19	10	2	15	0.01804	0.65084	1	12	15	4	7	10	0.67636194	1	8	6.96932052	3E+06	3392995	ID=ENSP0000382492;RADIL;NM_018059;	RADIL	3342320	3344362	0.36023	ID=promoter_30804;

3	3E+06	3_3352863	17	112	37	24	65	31	1.13E-04	0.00996	15	66	13	43	41	28	0.4570143	1	40.875	33.82914	3E+06	3392995	ID=ENSP0000382492;RADIL;NM_018059;	RADIL	3352485	3353558	0.18011	ID=promoter_30807;
3	3E+06	3_3358878	16	13	7	6	15	7	0.113635	1	0	1	14	0	1	7	0.08320466	1	7.125	6.55607679	3E+06	3392995	ID=ENSP0000382492;RADIL;NM_018059;	RADIL	3358563	3359441	0.25946	ID=promoter_30808;
3	3E+06	3_344788	48	57	66	56	53	61	0.994924	1	57	106	128	44	82	86	0.72621256	1	70.25	30.1650223								
3	3E+06	3_346907	125	60	108	180	93	144	0.111619	1	46	108	56	26	77	41	0.01471027	0.5356731	88.625	50.7738332								
3	4E+06	3_3539585	64	17	36	20	41	28	0.082602	1	61	44	27	21	53	24	0.01197842	0.4547496	36.25	18.4990347					3539393	3539954	0.15212	ID=promoter_30841;
3	4E+06	3_3567865	301	102	41	37	202	39	8.02E-31	#######	183	92	41	6	138	24	2.41E-16	1.32E-13	100.38	97.9240777					3567322	3568811	0.59957	ID=promoter_30843;
3	4E+06	3_3579293	0	1	19	17	1	18	0.001742	0.10079	112	53	48	33	83	41	0.00362558	0.1775497	35.375	36.6174724					3578793	3580233	0.35814	ID=promoter_30847;
3	4E+06	3_3585004	26	0	14	2	13	8	0.344217	1	4	7	2	1	6	2	0.42506085	1	7	8.89622713					3584587	3585783	0.23904	ID=promoter_30849;
3	4E+06	3_362449	64	5	94	52	35	73	0.017806	0.64429	41	17	31	17	29	24	0.91882689	1	40.125	29.2791369								
3	4E+06	3_377418	28	62	7	41	45	24	0.005987	0.27181	496	340	328	106	418	217	1.89E-11	6.88E-09	176	184.772447								
3	4E+06	3_3792105	969	438	300	275	704	288	1.08E-54	#######	765	346	207	181	556	194	5.39E-33	6.16E-30	435.13	283.425696					3785592	3794526	0.98402	ID=promoter_30886;
3	4E+06	3_4101828	67	49	57	44	58	51	0.321621	1	28	31	8	0	30	4	1.24E-04	0.0109901	35.5	23.3054255	4E+06	4102761	ID=ENSP0000223029;AIMP2;NM_006303;	AIMP2	4100669	4103131	0.47041	ID=promoter_30925;
3	4E+06	3_414744	14	23	41	17	19	29	0.670114	1	32	11	8	35	22	22	0.96891521	1	22.625	12.1530419								
3	4E+06	3_4200026	19	33	39	44	26	42	0.436267	1	73	35	37	40	54	39	0.49191155	1	40	15.2596574					4199594	4203479	0.59084	ID=promoter_30936;
3	4E+06	3_4217884	984	429	897	941	707	919	0.121191	1	171	126	92	3	149	48	2.19E-10	7.03E-08	455.38	420.381778					4216935	4221158	0.70527	ID=promoter_30941;
3	4E+06	3_4231291	25	30	32	45	28	39	0.785678	1	0	10	3	1	5	2	0.58563225	1	18.25	16.9852878					4230646	4232533	0.32397	ID=promoter_30942;
3	4E+06	3_431896	30	80	29	40	55	35	0.016158	0.59693	58	64	62	37	61	50	0.81184792	1	50	18.5703296								
3	4E+06	3_439230	32	105	43	29	69	36	2.76E-04	0.0215	70	63	50	37	67	44	0.20312041	1	53.625	25.2752702								
3	4E+06	3_4423854	2	7	21	17	5	19	0.046679	1	4	30	23	17	17	20	0.769441	1	15.125	9.90580638					4423119	4423923	0.15124	ID=promoter_30960;
3	5E+06	3_453589	197	101	175	149	149	162	0.799697	1	122	67	68	32	95	50	0.0045586	0.2111223	113.88	57.4666052								
3	5E+06	3_4600865	13	33	13	19	23	16	0.267873	1	13	13	25	0	13	13	0.98151319	1	16.125	9.78975412	5E+06	4612922	ID=ENSP0000297293;LMTK2;NM_014916;	LMTK2				
3	5E+06	3_4646248	4	17	15	32	11	24	0.223145	1	2	10	17	25	6	21	0.00855938	0.348087	15.25	10.0818082	5E+06	4646491	ID=ENSP0000369121;TECPR1;;	TECPR1	4645851	4646803	0.22694	ID=promoter_31003;
3	5E+06	3_4658024	11	2	5	27	7	16	0.324814	1	19	11	13	58	15	36	0.00572725	0.2548307	18.25	17.8465683					4657597	4659036	0.20702	ID=promoter_31005;
3	5E+06	3_4672436	19	24	8	5	22	7	0.005528	0.25484	28	8	22	2	18	12	0.67374005	1	14.5	9.85610761					4671446	4673157	0.48318	ID=promoter_31010;
3	5E+06	3_4694087	126	131	205	146	129	176	0.426599	1	75	38	73	13	57	43	0.59310339	1	100.88	62.7226036					4693380	4694508	0.28817	ID=promoter_31017;
3	5E+06	3_4703565	40	45	43	41	43	42	0.699784	1	38	27	37	1	33	19	0.24811784	1	34	14.3924583					4703435	4704458	0.35033	ID=promoter_31018;
3	5E+06	3_470982	56	134	181	177	95	179	8.23E-04	0.0539	123	53	47	75	88	61	0.21586731	1	105.75	55.4069361								
3	5E+06	3_473103	1	21	0	3	11	2	0.020291	0.71411	1	1	52	1	1	27	2.06E-06	0.0003194	10	18.3536684								
3	5E+06	3_475793	107	72	27	25	90	26	7.88E-11	#######	16	20	12	31	18	22	0.68341813	1	38.75	33.2426146								
3	5E+06	3_476060	719	498	274	277	609	276	6.44E-41	#######	255	101	153	153	178	153	0.79042781	1	303.75	207.261567								

3	5E+06	3_4786217	232	140	210	274	186	242	0.568016	1	126	174	69	94	150	82	5.17E-04	0.0364897	164.88	70.5882173					4785694	4786712	0.26372	ID=promoter_31025;
3	5E+06	3_487593	6	36	23	11	21	17	0.520268	1	56	34	28	11	45	20	0.01997236	0.6799503	25.625	16.5868924								
3	5E+06	3_493987	4	46	47	3	25	25	0.852664	1	26	11	61	45	19	53	8.44E-05	0.0079966	30.375	22.3985809								
3	5E+06	3_505979	27	36	40	47	32	44	0.790995	1	40	14	22	26	27	24	0.98883926	1	31.5	11.0324198								
3	5E+06	3_506457	103	66	69	126	85	98	0.993282	1	25	41	3	6	33	5	9.18E-05	0.0085951	54.875	44.5018057								
3	5E+06	3_5165257	0	12	0	6	6	3	0.462004	1	1	0	24	1	1	13	0.0036419	0.1775497	5.5	8.5857024	5E+06	5235978	ID=ENSP0000352925;TRRAP;;	TRRAP	5164370	5166027	0.31642	ID=promoter_31057;
3	5E+06	3_5171039	0	8	0	0	4	0	0.09576	1	0	14	31	2	7	17	0.08351885	1	6.875	10.9991883	5E+06	5235978	ID=ENSP0000352925;TRRAP;;	TRRAP	5170447	5171683	0.2892	ID=promoter_31059;
3	5E+06	3_5192043	12	28	2	11	20	7	0.013885	0.52965	5	11	4	0	8	2	0.20550608	1	9.125	8.85498246	5E+06	5235978	ID=ENSP0000352925;TRRAP;;	TRRAP				
3	5E+06	3_5218326	170	96	28	26	133	27	5.55E-20	#######	80	48	47	14	64	31	0.01049516	0.407985	63.625	51.1299116	5E+06	5235978	ID=ENSP0000352925;TRRAP;;	TRRAP	5217954	5218621	0.12221	ID=promoter_31074;
3	5E+06	3_5221092	17	13	20	0	15	10	0.374041	1	0	18	0	0	9	0	0.01542606	0.5535504	8.5	9.2890104	5E+06	5235978	ID=ENSP0000352925;TRRAP;;	TRRAP	5220232	5221686	0.1664	ID=promoter_31076;
3	5E+06	3_5226445	0	26	11	0	13	6	0.146758	1	0	0	3	10	0	7	0.02292696	0.7568963	6.25	9.20791585	5E+06	5235978	ID=ENSP0000352925;TRRAP;;	TRRAP	5225949	5227769	0.32078	ID=promoter_31078;
3	5E+06	3_527245	0	83	13	21	42	17	5.42E-04	0.03781	13	5	55	2	9	29	0.00234316	0.1251016	24	29.553825								
3	5E+06	3_538898	775	521	678	827	648	753	0.984889	1	48	110	199	104	79	152	5.12E-07	9.13E-05	407.75	327.418365								
3	5E+06	3_5393346	23	4	5	3	14	4	0.02589	0.86655	22	0	5	1	11	3	0.13591373	1	7.875	9.20306626					5391885	5401611	0.98645	ID=promoter_31091;
3	5E+06	3_5403716	273	106	218	279	190	249	0.515082	1	35	65	27	23	50	25	0.03767958	1	128.25	111.004183					5402752	5405376	0.51396	ID=promoter_31092;
3	5E+06	3_541033	84	124	23	3	104	13	1.60E-19	#######	89	33	35	82	61	59	0.97337494	1	59.125	41.3398372								
3	5E+06	3_5420132	59	44	66	74	52	70	0.753203	1	36	38	52	25	37	39	0.85493945	1	49.25	16.5335157					5419691	5421704	0.28936	ID=promoter_31094;
3	6E+06	3_558578	40	0	24	21	20	23	0.997913	1	0	16	2	2	8	2	0.20550608	1	13.125	14.6525229								
3	6E+06	3_559167	45	42	57	48	44	53	0.991545	1	39	16	7	2	28	5	7.63E-04	0.0504367	32	20.6328448								
3	6E+06	3_565361	175	107	123	153	141	138	0.317928	1	134	9	50	31	72	41	0.0422523	1	97.75	60.5563492								
3	6E+06	3_568093	4	0	4	6	2	5	0.651182	1	6	11	14	7	9	11	0.82669107	1	6.5	4.34248119								
3	6E+06	3_5687230	7	17	8	1	12	5	0.126784	1	16	18	20	50	17	35	0.0215018	0.7254463	17.125	14.8173402					5687117	5689397	0.55391	ID=promoter_31121;
3	6E+06	3_5688075	0	1	8	15	1	12	0.021411	0.74203	0	38	0	26	19	13	0.6985193	1	11	14.3527001					5687117	5689397	0.55391	ID=promoter_31121;
3	6E+06	3_5734538	27	28	26	52	28	39	0.785678	1	1	38	47	32	20	40	0.01593163	0.5684069	31.375	15.5649561					5733149	5735003	0.35885	ID=promoter_31127;
3	6E+06	3_5776503	9	9	41	16	9	29	0.02202	0.75814	5	16	32	20	11	26	0.02624811	0.8458091	18.5	12.3404098					5775350	5776954	0.32387	ID=promoter_31134;
3	6E+06	3_5810038	1181	585	1002	574	883	788	1.59E-07	#######	646	510	299	377	578	338	9.82E-11	3.29E-08	646.75	300.743721					5809266	5810344	0.27246	ID=promoter_31139;
3	6E+06	3_632507	52	59	23	28	56	26	2.78E-04	0.02161	2	1	4	4	2	4	0.6610374	1	21.625	23.2805099								
3	7E+06	3_6827923	671	328	526	366	500	446	1.37E-04	0.01173	152	199	142	122	176	132	0.18264288	1	313.25	200.430073					6826527	6829275	0.5907	ID=promoter_31188;
3	7E+06	3_6856133	258	107	243	229	183	236	0.628945	1	188	124	84	11	156	48	1.91E-11	6.94E-09	155.5	87.7968434					6853013	6856644	0.61497	ID=promoter_31192;
3	7E+06	3_6875567	550	29	218	217	290	218	3.65E-06	0.00049	262	100	87	21	181	54	1.24E-13	5.75E-11	185.5	172.926904	7E+06	6876967	ID=ENSP0000259034-D2;ACTL6A;NM_004301;	ACTL6A				

3	7E+06	3_6876748	157	67	112	112	112	112	0.489649	1	15	21	12	6	18	9	0.30718372	1	62.75	58.0289337	7E+06	6876967	ID=ENSP0000259034-D2;ACTL6A;NM_004301;	ACTL6A				
3	7E+06	3_6880694	1	73	15	13	37	14	7.29E-04	0.04855	2	19	15	21	11	18	0.32230807	1	19.875	22.6617204					6880539	6881486	0.2113	ID=promoter_31194;
3	7E+06	3_6904331	6	10	23	2	8	13	0.766846	1	47	11	21	23	29	22	0.77701989	1	17.875	14.2070154	7E+06	6904388	ID=ENSP00000304353;POP7;NM_005837;	POP7	6902841	6904391	0.35456	ID=promoter_31197;
3	7E+06	3_6941540	15	25	24	21	20	23	0.997913	1	30	26	14	45	28	30	0.85900118	1	25	9.73946317	7E+06	6950499	ID=ENSP00000381043;ZAN;;	ZAN				
3	7E+06	3_698605	13	7	23	15	10	19	0.459788	1	13	16	14	23	15	19	0.66253156	1	15.5	5.34522484								
3	7E+06	3_735376	65	100	50	65	83	58	0.009256	0.38264	0	21	15	15	11	15	0.61974467	1	41.375	34.112995								
3	8E+06	3_760253	9	31	15	23	20	19	0.804666	1	10	6	23	22	8	23	0.01462253	0.532614	17.375	8.70036945								
3	8E+06	3_7659452	0	15	4	12	8	8	0.950274	1	2	13	1	30	8	16	0.19094317	1	9.625	10.1268737					7658771	7659923	0.23541	ID=promoter_31240;
3	8E+06	3_766395	20	43	17	25	32	21	0.11093	1	56	19	30	4	38	17	0.03846005	1	26.75	16.263456								
3	8E+06	3_767869	23	33	33	34	28	34	0.990834	1	16	2	4	1	9	3	0.27712561	1	18.25	14.5184612								
3	8E+06	3_7727856	4	0	1	2	2	2	0.98733	1	38	32	22	1	35	12	0.00825069	0.3370693	12.5	15.6934927					7727175	7728179	0.23885	ID=promoter_31253;
3	8E+06	3_7808103	478	183	375	374	331	375	0.899455	1	117	108	127	48	113	88	0.4707746	1	226.25	158.901362					7807143	7808136	0.18786	ID=promoter_31277;
3	8E+06	3_7846613	20	18	11	18	19	15	0.513973	1	52	39	38	50	46	44	0.9890416	1	30.75	15.9171965					7844907	7847856	0.37196	ID=promoter_31290;
3	8E+06	3_7846784	17	19	7	13	18	10	0.152558	1	129	25	93	16	77	55	0.33400382	1	39.875	45.2277964					7844907	7847856	0.37196	ID=promoter_31290;
3	8E+06	3_7902161	17	65	7	0	41	4	1.01E-08	#######	53	26	21	0	40	11	7.51E-04	0.0497587	23.625	23.9758063					7901580	7902978	0.19643	ID=promoter_31306;
3	8E+06	3_791497	26	43	25	13	35	19	0.02151	0.74463	0	2	2	1	1	2	0.81304206	1	14	15.8564993								
3	8E+06	3_799968	55	14	73	54	35	64	0.103601	1	1	6	18	3	4	11	0.14780855	1	28	28.1932109								
3	8E+06	3_802445	32	20	18	15	26	17	0.164334	1	0	9	1	0	5	1	0.30486099	1	11.875	11.5069855								
3	8E+06	3_8033007	4	8	3	0	6	2	0.258649	1	23	27	6	13	25	10	0.06888504	1	10.5	9.78336781	8E+06	8132319	ID=ENSP00000353401;CUX1;;	CUX1				
3	8E+06	3_8069336	2	18	6	25	10	16	0.741563	1	1	0	14	2	1	8	0.04988758	1	8.5	9.35032467	8E+06	8132319	ID=ENSP00000353401;CUX1;;	CUX1				
3	8E+06	3_8086628	20	22	14	5	21	10	0.052655	1	56	13	21	2	35	12	0.00825069	0.3370693	19.125	16.5998064	8E+06	8132319	ID=ENSP00000353401;CUX1;;	CUX1				
3	8E+06	3_8086971	23	0	0	16	12	8	0.45534	1	12	0	14	0	6	7	0.91781435	1	8.125	9.23405962	8E+06	8132319	ID=ENSP00000353401;CUX1;;	CUX1	8086753	8089398	0.37024	ID=promoter_31343;
3	8E+06	3_8134230	26	0	19	8	13	14	0.975695	1	0	2	3	5	1	4	0.36109265	1	7.875	9.58328157					8133742	8134423	0.16629	ID=promoter_31358;
3	8E+06	3_816971	5	22	8	0	14	4	0.02589	0.86655	22	10	7	1	16	4	0.04223275	1	9.375	8.48422907								
3	8E+06	3_8206432	5	17	8	17	11	13	0.999831	1	0	10	2	1	5	2	0.58563225	1	7.5	6.78232998					8205944	8206583	0.1314	ID=promoter_31365;
3	9E+06	3_8758937	406	253	318	391	330	355	0.527178	1	255	178	128	44	217	86	5.58E-11	1.92E-08	246.63	126.088334					8757821	8759331	0.37025	ID=promoter_31395;
3	9E+06	3_876339	1220	590	1227	1253	905	1240	0.001781	0.1024	701	312	519	54	507	287	1.32E-10	4.32E-08	734.5	456.248366								
3	9E+06	3_894775	3	28	6	13	16	10	0.283594	1	0	5	15	0	3	8	0.26303803	1	8.75	9.52815377								
3	9E+06	3_899432	1	62	22	16	32	19	0.056624	1	11	39	30	39	25	35	0.2845844	1	27.5	19.2650683								
3	9E+06	3_9055154	115	32	93	81	74	87	0.999893	1	1	29	15	23	15	19	0.66253156	1	48.625	41.6445417					9054918	9055776	0.21851	ID=promoter_31411;

3	9E+06	3_9080774	0	27	0	17	14	9	0.360318	1	32	23	17	11	28	14	0.15944813	1	15.875	11.7404977	9E+06	9093263	ID=ENSP00000257665;POM121C;;	POM121C				
3	9E+06	3_912202	29	18	5	14	24	10	0.016074	0.59429	13	23	15	4	18	10	0.42261869	1	15.125	8.40811683								
3	9E+06	3_9174830	1211	315	804	630	763	717	1.09E-04	0.00963	303	246	397	126	275	262	0.9511309	1	504	359.114785					9173799	9175800	0.51291	ID=promoter_31422;
3	9E+06	3_9365505	144	76	138	141	110	140	0.814336	1	102	89	57	10	96	34	3.55E-06	0.0005089	94.625	47.0256389					9364885	9365835	0.33164	ID=promoter_31435;
3	9E+06	3_940235	52	30	27	46	41	37	0.510548	1	0	11	14	8	6	11	0.39136385	1	23.5	18.5626353								
3	1E+07	3_965248	28	245	70	72	137	71	6.91E-08	#######	111	132	100	41	122	71	0.00706261	0.2990571	99.875	68.1582979								
3	1E+07	3_967640	28	16	1	13	22	7	0.005528	0.25484	16	6	0	1	11	1	0.02232329	0.7455867	10.125	9.87692115								
3	1E+07	3_970333	4	7	3	2	6	3	0.462004	1	29	0	23	6	15	15	0.97869956	1	9.25	10.6871084								
3	1E+07	3_973627	0	2	1	5	1	3	0.700657	1	20	2	31	6	11	19	0.24832023	1	8.375	11.1475494								
3	1E+07	3_991988	17	72	23	14	45	19	5.00E-04	0.0352	5	24	21	67	15	44	2.54E-04	0.0200383	30.375	24.9223795								
3	1E+07	3_992559	59	156	66	45	108	56	2.30E-06	0.00032	184	65	158	107	125	133	0.54189675	1	105	54.1057166								
3	1E+07	3_995890	6	9	0	1	8	1	0.03583	1	1	8	20	11	5	16	0.03625394	1	7	6.67618368								
3	1E+07	3_996488	7	40	27	12	24	20	0.525373	1	40	23	15	10	32	13	0.03583578	1	21.75	13.0466197								
3	1E+07	3_101890	210	275	279	111	243	195	3.75E-04	0.02793	143	134	116	32	139	74	4.90E-04	0.0349469	162.5	85.7921075								
3	1E+07	3_103131	2	15	20	32	9	26	0.054763	1	74	26	24	0	50	12	3.48E-05	0.0037136	24.125	23.0678347								
3	1E+07	3_103398	30	149	33	41	90	37	1.40E-07	#######	68	113	103	75	91	89	0.93755155	1	76.5	42.5608808								
3	1E+07	3_104798	170	81	82	99	126	91	0.001772	0.10191	50	1	51	22	26	37	0.24075912	1	69.5	52.0027472								
3	1E+07	3_105409	15	30	0	0	23	0	1.39E-06	0.0002	0	1	7	20	1	14	0.00214405	0.1161796	9.125	11.3947043								
3	1E+07	3_108826	93	42	42	20	68	31	3.58E-05	0.00364	10	0	11	0	5	6	0.91271846	1	27.25	31.2992469								
3	1E+07	3_11068557	0	2	8	27	1	18	0.001742	0.10079	12	15	0	1	14	1	0.00574567	0.2548307	8.125	9.55342123					1.1E+07	1.1E+07	0.08861	ID=promoter_31480;
3	1E+07	3_111403	6	30	33	10	18	22	0.991654	1	0	18	6	6	9	6	0.8208167	1	13.625	12.1647911								
3	1E+07	3_112339	5	74	1	2	40	2	1.28E-09	#######	0	24	53	0	12	27	0.03064003	0.9477626	19.875	28.6128717								
3	1E+07	3_112902	308	200	444	417	254	431	1.64E-05	0.00184	126	140	163	69	133	116	0.89107303	1	233.38	139.889689								
3	1E+07	3_114604	2	11	4	9	7	7	0.956352	1	1	2	18	6	2	12	0.01895406	0.6519669	6.625	5.80486249								
3	1E+07	3_116402	1	26	0	13	14	7	0.165008	1	28	0	10	36	14	23	0.23132274	1	14.25	14.1497097								
3	1E+07	3_118130	0	13	8	16	7	12	0.724589	1	13	22	13	3	18	8	0.20901227	1	11	7.09124208								
3	1E+07	3_118223	0	17	0	5	9	3	0.131542	1	45	0	6	0	23	3	9.46E-04	0.0600857	9.125	15.6245								
3	1E+07	3_123117	1	3	7	1	2	4	0.823343	1	1	32	9	7	17	8	0.27279187	1	7.625	10.3501208								
3	1E+07	3_124212	34	79	24	48	57	36	0.012719	0.4931	5	58	32	3	32	18	0.23040678	1	35.375	25.8784936								
3	1E+07	3_124902	74	10	17	24	42	21	0.004493	0.21484	35	1	26	32	18	29	0.17554477	1	27.375	21.9475837								
3	1E+07	3_124905	8	0	3	3	4	3	0.840045	1	27	0	0	25	14	13	0.99999086	1	8.25	11.2852622								
3	1E+07	3_12498953	718	300	495	592	509	544	0.320274	1	100	105	71	3	103	37	1.89E-06	0.0002956	298	271.676278					1.2E+07	1.2E+07	0.17979	ID=promoter_31492;
3	1E+07	3_12541570	87	19	53	96	53	75	0.577824	1	0	32	18	1	16	10	0.61601721	1	38.25	37.0318396					1.3E+07	1.3E+07	0.26894	ID=promoter_31496;
3	1E+07	3_125589	19	57	8	26	38	17	0.002761	0.14629	10	13	14	0	12	7	0.61698677	1	18.375	17.3776334								
3	1E+07	3_12769837	26	13	65	40	20	53	0.006122	0.27646	40	41	8	43	41	26	0.31320499	1	34.5	18.2912938					1.3E+07	1.3E+07	0.26173	ID=promoter_31525;
3	1E+07	3_12770719	60	16	10	8	38	9	1.04E-05	0.00124	31	30	16	1	31	9	0.00523026	0.2377558	21.5	18.6700677					1.3E+07	1.3E+07	0.26173	ID=promoter_31525;
3	1E+07	3_127758	54	32	21	26	43	24	0.011717	0.46024	63	31	23	13	47	18	0.00440209	0.2061474	32.875	17.0665713								
3	1E+07	3_127952	14	17	9	5	16	7	0.076817	1	0	0	24	2	0	13	9.01E-04	0.0577931	8.875	8.79021371								
3	1E+07	3_128729	4	59	14	11	32	13	0.003339	0.17064	1	16	9	8	9	9	0.98716474	1	15.25	18.3439363								
3	1E+07	3_129098	10	11	18	21	11	20	0.500229	1	0	4	5	0	2	3	0.86792893	1	8.625	7.85470741								
3	1E+07	3_129652	15	48	30	3	32	17	0.02552	0.85718	22	29	33	68	26	51	0.00629899	0.2730383	31	19.9570968								
3	1E+07	3_129789	6	0	8	0	3	4	0.986002	1	17	0	11	2	9	7	0.94097808	1	5.5	6.23354978								
3	1E+07	3_133309	3	20	0	4	12	2	0.011798	0.46127	0	6	24	1	3	13	0.02959638	0.9232309	7.25	9.39224604								
3	1E+07	3_138957	301	138	218	209	220	214	0.148696	1	51	56	72	1	54	37	0.36386527	1	130.75	103.515009								
3	1E+07	3_14006467	9	24	3	32	17	18	0.955187	1	13	4	3	1	9	2	0.1390433	1	11.125	11.3066036					1.4E+07	1.4E+07	0.15411	ID=promoter_31601;
3	1E+07	3_142884	5	75	19	17	40	18	0.002142	0.11834	15	19	62	183	17	123	5.73E-20	3.90E-17	49.375	59.4208657								
3	1E+07	3_145625	210	200	125	145	205	135	8.81E-07	0.00013	86	91	43	125	89	84	0.9930738	1	128.13	56.7864106								
3	1E+07	3_14584757	2403	990	3051	2067	1697	2559	8.75E-15	#######	842	865	919	260	854	590	2.52E-07	4.84E-05	1424.6	961.286183					1.5E+07	1.5E+07	0.51728	ID=promoter_31616;
3	2E+07	3_150243	232	87	110	116	160	113	1.65E-04	0.01374	123	69	112	39	96	76	0.58882154	1	111	56.4876725								
3	2E+07	3_151526	26	38	37	40	32	39	0.987189	1	2	17	25	6	10	16	0.39084582	1	23.875	14.5546998								
3	2E+07	3_15188527	283	121	236	160	202	198	0.198761	1	102	88	66	58	95	62	0.09684313	1	139.25	81.739569	2E+07	1.5E+07	ID=ENSP00000302728;GUSB;NM_000181;	GUSB				

3	2E+07	3_15447000	12	26	16	15	19	16	0.617999	1	2	7	57	9	5	33	1.10E-05	0.0013789	18	17.2874852	2E+07	1.5E+07	ID=ENSP00000378854;PSPH;NM_004577;	PSPH				
3	2E+07	3_15471559	258	115	262	256	187	259	0.222425	1	42	42	77	1	42	39	0.99997259	1	131.63	110.081707					1.5E+07	1.5E+07	0.60548	ID=promoter_31651;
3	2E+07	3_156278	23	53	7	22	38	15	8.82E-04	0.05714	42	2	36	28	22	32	0.26050199	1	26.625	17.1041975								
3	2E+07	3_156573	164	45	189	176	105	183	0.004992	0.2346	127	80	47	38	104	43	2.84E-05	0.0031193	108.25	63.308203								
3	2E+07	3_157179	95	125	157	159	110	158	0.26306	1	147	136	129	47	142	88	0.01149355	0.4382061	124.38	37.3819571								
3	2E+07	3_15874226	2	24	2	2	13	2	0.006819	0.29841	34	27	12	1	31	7	0.00122211	0.0738454	13	13.4483138	2E+07	1.6E+07	ID=ENSP00000219797;MYST1;NM_032188;	MYST1				
3	2E+07	3_15919325	1	1	0	9	1	5	0.353258	1	22	1	19	13	12	16	0.63262749	1	8.25	8.89221168					1.6E+07	1.6E+07	0.47212	ID=promoter_31672;
3	2E+07	3_15920569	6	2	9	3	4	6	0.929647	1	1	9	22	0	5	11	0.25523295	1	6.5	7.15142343					1.6E+07	1.6E+07	0.47212	ID=promoter_31672;
3	2E+07	3_159371	78	100	74	50	89	62	0.006261	0.28201	191	60	124	93	126	109	0.86944235	1	96.25	44.8067278								
3	2E+07	3_15947259	16	18	38	14	17	26	0.694872	1	25	14	15	1	20	8	0.11762508	1	17.625	10.5686531					1.6E+07	1.6E+07	0.1564	ID=promoter_31675;
3	2E+07	3_16063525	28	0	31	13	14	22	0.691767	1	0	0	1	0	0	1	0.58312783	1	9.125	13.3570474	2E+07	1.6E+07	ID=ENSP00000369674;BCL7C;;	BCL7C				
3	2E+07	3_16235965	223	102	248	178	163	213	0.583212	1	117	46	88	26	82	57	0.24577229	1	128.5	80.5764943					1.6E+07	1.6E+07	0.43867	ID=promoter_31718;
3	2E+07	3_16344789	1710	525	1067	1097	1118	1082	3.74E-05	0.00379	529	512	461	255	521	358	6.87E-05	0.0066368	769.5	481.680392					1.6E+07	1.6E+07	0.47435	ID=promoter_31727;
3	2E+07	3_16376413	11	9	20	20	10	20	0.378359	1	0	9	1	0	5	1	0.30486099	1	8.75	8.20713975	2E+07	1.6E+07	ID=ENSP00000348489;FBRS;NM_001105079;	FBRS				
3	2E+07	3_16412718	24	25	18	11	25	15	0.11231	1	9	24	17	4	17	11	0.64636287	1	16.5	7.83763813					1.6E+07	1.6E+07	0.47595	ID=promoter_31736;
3	2E+07	3_16516320	4	27	0	8	16	4	0.009581	0.39335	0	17	0	0	9	0	0.01542606	0.5535504	7	10.0427657	2E+07	1.7E+07	ID=ENSP00000321989;ITGAL;;	ITGAL				
3	2E+07	3_16544581	837	326	788	801	582	795	0.020103	0.71049	310	304	406	123	307	265	0.69605855	1	486.88	278.129691					1.7E+07	1.7E+07	0.23731	ID=promoter_31745;
3	2E+07	3_16596934	7	37	0	6	22	3	1.41E-04	0.01198	27	42	36	3	35	20	0.21889545	1	19.75	17.4499284					1.7E+07	1.7E+07	0.78032	ID=promoter_31752;
3	2E+07	3_16597270	28	16	36	25	22	31	0.804879	1	20	13	5	7	17	6	0.10713464	1	18.75	10.633505					1.7E+07	1.7E+07	0.78032	ID=promoter_31752;
3	2E+07	3_166601	71	47	67	22	59	45	0.090603	1	55	8	11	3	32	7	8.21E-04	0.0536252	35.5	27.6715429								
3	2E+07	3_168258	156	44	183	94	100	139	0.431614	1	108	58	28	23	83	26	2.65E-06	0.0003962	86.75	59.4420486								
3	2E+07	3_170699	24	18	23	19	21	21	0.874689	1	26	13	5	1	20	3	0.00346999	0.1723921	16.125	9.10945349								
3	2E+07	3_172745	14	6	20	33	10	27	0.068117	1	36	3	78	15	20	47	0.00147388	0.0863896	25.625	24.1479813								
3	2E+07	3_173252	0	0	43	31	0	37	1.41E-07	#######	0	0	0	12	0	6	0.0393172	1	10.75	17.027289								
3	2E+07	3_174357	31	0	31	7	16	19	0.999341	1	0	1	1	7	1	4	0.36109265	1	9.75	13.4243914								
3	2E+07	3_175082	14	23	4	10	19	7	0.021688	0.74727	0	10	1	17	5	9	0.48000906	1	9.875	8.02562859								
3	2E+07	3_17532079	56	43	50	54	50	52	0.831366	1	7	17	22	7	12	15	0.74080056	1	32	20.976177	2E+07	1.8E+07	ID=ENSP00000348510;GTF3C1;NM_001520;	GTF3C1	1.8E+07	1.8E+07	0.16341	ID=promoter_31813;
3	2E+07	3_17539677	1	0	40	24	1	32	4.66E-06	0.0006	0	1	8	0	1	4	0.36109265	1	9.25	14.9164339	2E+07	1.8E+07	ID=ENSP00000348510;GTF3C1;NM_001520;	GTF3C1				
3	2E+07	3_176676	4	63	26	19	34	23	0.11872	1	31	91	58	52	61	55	0.98889628	1	43	28.1323403								
3	2E+07	3_176870	29	111	31	27	70	29	5.32E-06	0.00068	65	37	54	43	51	49	0.98415652	1	49.625	28.0557481								
3	2E+07	3_177024	2	2	6	3	2	5	0.651182	1	2	1	10	35	2	23	6.46E-05	0.0062789	7.625	11.4509825								

3	2E+07	3_178022	44	49	25	22	47	24	0.002974	0.15459	48	26	20	25	37	23	0.31728647	1	32.375	12.3396863								
3	2E+07	3_179217	169	140	24	39	155	32	6.06E-23	#######	131	90	23	15	111	19	2.07E-13	9.38E-11	78.875	61.531496								
3	2E+07	3_17943433	37	70	4	14	54	9	2.10E-09	#######	54	16	20	3	35	12	0.00825069	0.3370693	27.25	24.2649071					1.8E+07	1.8E+07	0.17342	ID=promoter_31851;
3	2E+07	3_180307	17	13	12	7	15	10	0.374041	1	17	23	0	0	20	0	9.42E-05	0.0087339	11.125	8.27107697								
3	2E+07	3_181682	2	21	10	6	12	8	0.45534	1	2	36	28	24	19	26	0.43045343	1	16.125	12.8778603								
3	2E+07	3_183758	24	18	11	10	21	11	0.084122	1	27	27	11	17	27	14	0.20179887	1	18.125	7.18008158								
3	2E+07	3_186539	13	15	17	13	14	15	0.970741	1	2	8	28	3	5	16	0.03625394	1	12.375	8.34843869								
3	2E+07	3_187978	0	13	13	35	7	24	0.032448	1	23	12	60	0	18	30	0.13648184	1	19.5	19.9785599								
3	2E+07	3_189350	1	37	19	0	19	10	0.108738	1	1	0	7	1	1	4	0.36109265	1	8.25	13.2960789								
3	2E+07	3_189364	1	0	26	31	1	29	1.67E-05	0.00187	0	5	4	9	3	7	0.3829009	1	9.5	12.1772622								
3	2E+07	3_189389	71	44	75	69	58	72	0.949665	1	34	11	18	15	23	17	0.77734386	1	42.125	26.707075								
3	2E+07	3_191930	60	0	26	32	30	29	0.758275	1	0	15	29	22	8	26	0.00417605	0.1967234	23	19.3390796								
3	2E+07	3_191948	28	21	24	15	25	20	0.43889	1	0	0	33	3	0	18	6.08E-05	0.0059624	15.5	13.1039798								
3	2E+07	3_198184	960	378	375	807	669	591	3.07E-06	0.00042	360	396	276	220	378	248	1.19E-04	0.0105864	471.5	264.265018								
3	2E+07	3_20149678	263	119	251	184	191	218	0.962762	1	93	169	91	81	131	86	0.04405893	1	156.38	72.3225661					2E+07	2E+07	0.16879	ID=promoter_31883;
3	2E+07	3_206109	3	0	7	6	2	7	0.358464	1	2	14	9	0	8	5	0.78486764	1	5.125	4.85320218								
3	2E+07	3_206405	7	26	5	10	17	8	0.087954	1	45	0	8	24	23	16	0.67590535	1	15.625	14.9373692								
3	2E+07	3_209741	582	429	803	1059	506	931	2.12E-15	#######	1126	643	1253	485	885	869	0.48418608	1	797.5	313.406172								
3	2E+07	3_21057519	1	25	2	1	13	2	0.006819	0.29841	11	4	38	1	8	20	0.04735057	1	10.375	13.8969421					2.1E+07	2.1E+07	0.34509	ID=promoter_31903;
3	2E+07	3_21058042	459	154	305	293	307	299	0.072385	1	205	115	152	63	160	108	0.03789803	1	218.25	127.911521					2.1E+07	2.1E+07	0.34509	ID=promoter_31903;
3	2E+07	3_21079126	0	1	4	7	1	6	0.241978	1	0	11	8	27	6	18	0.03091566	0.954015	7.25	8.94027804	2E+07	2.1E+07	ID=ENSP0000300061;SCNN1G;NM_001039;	SCNN1G				
3	2E+07	3_211901	11	10	11	6	11	9	0.722819	1	0	11	13	0	6	7	0.91781435	1	7.75	5.1754917								
3	2E+07	3_21201558	13	22	4	12	18	8	0.059652	1	3	5	0	0	4	0	0.15659576	1	7.375	7.670677					2.1E+07	2.1E+07	0.28503	ID=promoter_31906;
3	2E+07	3_212135	174	62	63	71	118	67	7.89E-06	0.00097	77	39	2	23	58	13	3.29E-06	0.0004789	63.875	51.3710244								
3	2E+07	3_212763	68	76	26	40	72	33	2.15E-05	0.00234	25	42	49	27	34	38	0.73008239	1	44.125	19.3423111								
3	2E+07	3_216057	42	27	62	72	35	67	0.059993	1	0	18	9	24	9	17	0.21129426	1	31.75	25.1779382								
3	2E+07	3_216857	3	21	18	12	12	15	0.986595	1	12	2	11	27	7	19	0.03889743	1	13.25	8.54818276								
3	2E+07	3_220704	0	39	17	33	20	25	0.977759	1	69	47	38	1	58	20	3.76E-04	0.027889	30.5	23.5189893								
3	2E+07	3_22209060	0	1	4	1	1	3	0.700657	1	20	0	6	12	10	9	0.99792604	1	5.5	7.13141941	2E+07	2.2E+07	ID=ENSP0000375440-D7697;;					
3	2E+07	3_22415400	645	350	522	450	498	486	0.015427	0.57632	405	270	80	86	338	83	3.02E-30	3.22E-27	351	199.50725					2.2E+07	2.2E+07	0.25572	ID=promoter_31930;
3	2E+07	3_226637	4	26	8	6	15	7	0.113635	1	2	1	8	4	2	6	0.31385293	1	7.375	7.94512429								
3	2E+07	3_23112938	36	69	0	0	53	0	3.17E-14	#######	37	8	1	49	23	25	0.85898626	1	25	26.4359063	2E+07	2.3E+07	ID=ENSP0000370765;GP2;NM_001007240;	GP2				
3	2E+07	3_232663	36	73	59	7	55	33	0.008146	0.34652	79	40	56	14	60	35	0.08940859	1	45.5	26.0932394								
3	2E+07	3_233003	18	16	5	23	17	14	0.615566	1	1	15	1	22	8	12	0.56746184	1	12.625	9.02278861								
3	2E+07	3_23384093	12	32	7	8	22	8	0.011059	0.44213	15	8	4	1	12	3	0.09316162	1	10.875	9.56836305	2E+07	2.3E+07	ID=ENSP0000370779;GPR139;NM_001002911;	GPR139				
3	2E+07	3_234153	19	6	20	3	13	12	0.835334	1	65	8	11	17	37	14	0.01279999	0.4795643	18.625	19.7479655								
3	2E+07	3_23606414	6	60	10	9	33	10	2.83E-04	0.02195	2	2	35	27	2	31	9.34E-07	0.0001565	18.875	20.4550902					2.4E+07	2.4E+07	0.25182	ID=promoter_31951;
3	2E+07	3_236396	1	10	1	10	6	6	0.962469	1	0	15	18	0	8	9	0.92351511	1	6.875	7.29848321								
3	2E+07	3_236836	0	18	2	14	9	8	0.848833	1	0	0	10	7	0	9	0.00779604	0.3222758	6.375	7.04956939								
3	2E+07	3_23754719	729	275	457	394	502	426	5.14E-06	0.00066	191	124	276	40	158	158	0.79708919	1	310.75	216.55072					2.4E+07	2.4E+07	0.38675	ID=promoter_31953;
3	2E+07	3_237684	104	35	83	76	70	80	0.987466	1	44	0	38	1	22	20	0.99799876	1	47.625	37.6864413								
3	2E+07	3_24228588	17	17	4	0	17	2	7.31E-04	0.04855	45	1	6	0	23	3	9.46E-04	0.0600857	11.25	15.3227189					2.4E+07	2.4E+07	0.15357	ID=promoter_31964;

3	2E+07	3_24403609	56	34	8	14	45	11	1.69E-06	0.00024	3	26	8	21	15	15	0.97869956	1	21.25	17.4417398	2E+07	2.5E+07	ID=ENSP0000261381;XYLT1;NM_022166;	XYLT1	2.4E+07	2.4E+07	0.15357	ID=promoter_31971;
3	2E+07	3_245284	3	17	0	5	10	3	0.082185	1	0	11	24	1	6	13	0.20797135	1	7.625	8.91126895								
3	2E+07	3_245609	290	177	375	489	234	432	1.26E-07	#######	58	85	219	85	72	152	2.51E-08	5.83E-06	222.25	153.896765								
3	2E+07	3_245714	8	15	2	0	12	1	0.003675	0.18279	0	7	13	5	4	9	0.31291743	1	6.25	5.65053727								
3	2E+07	3_24578857	98	61	83	63	80	73	0.298318	1	25	21	12	15	23	14	0.45771946	1	47.25	33.2898526					2.5E+07	2.5E+07	0.13314	ID=promoter_31981;
3	2E+07	3_247669	13	62	9	11	38	10	2.46E-05	0.00262	17	13	44	23	15	34	0.01174317	0.4468879	24	19.0112749								
3	3E+07	3_254239	1955	2074	3318	3107	2015	3213	2.79E-26	#######	1777	1130	3059	985	1454	2022	2.40E-31	2.66E-28	2175.6	900.924595								
3	3E+07	3_256358	49	60	35	33	55	34	0.011572	0.45508	44	40	63	34	42	49	0.54863551	1	44.75	11.6833214								
3	3E+07	3_256936	7	8	5	6	8	6	0.705675	1	8	21	12	4	15	8	0.44137572	1	8.875	5.46253473								
3	3E+07	3_257019	31	44	37	21	38	29	0.213776	1	27	35	25	3	31	14	0.07401504	1	27.875	12.414709								
3	3E+07	3_257876	20	47	18	7	34	13	0.001425	0.08487	41	3	36	15	22	26	0.70257106	1	23.375	16.1328193								
3	3E+07	3_26001466	925	266	631	480	596	556	5.13E-04	0.03605	359	304	258	182	332	220	5.38E-04	0.0374718	425.63	246.887445					2.6E+07	2.6E+07	0.26192	ID=promoter_32006;
3	3E+07	3_26014174	69	63	85	82	66	84	0.884061	1	91	32	51	37	62	44	0.39687748	1	63.75	22.1730209					2.6E+07	2.6E+07	0.10476	ID=promoter_32007;
3	3E+07	3_26093264	1	18	25	20	10	23	0.193823	1	1	17	23	36	9	30	0.00152443	0.0882297	17.625	11.8314049	3E+07	2.6E+07	ID=ENSP0000345456;;NM_001134477;		2.6E+07	2.6E+07	0.13697	ID=promoter_32017;
3	3E+07	3_26150060	2	24	3	6	13	5	0.083134	1	25	23	32	2	24	17	0.69596948	1	14.625	12.512137	3E+07	2.6E+07	ID=ENSP0000345456;;NM_001134477;					
3	3E+07	3_26156627	19	14	5	25	17	15	0.722447	1	11	22	15	1	17	8	0.27279187	1	14	8.19407451	3E+07	2.6E+07	ID=ENSP0000345456;;NM_001134477;					
3	3E+07	3_26229326	41	19	32	6	30	19	0.102296	1	0	5	3	2	3	3	0.99570314	1	13.5	15.501152	3E+07	2.6E+07	ID=ENSP0000345456;;NM_001134477;					
3	3E+07	3_26246998	109	74	99	125	92	112	0.965678	1	36	62	20	1	49	11	2.36E-05	0.002647	65.75	44.3033375	3E+07	2.6E+07	ID=ENSP0000345456;;NM_001134477;					
3	3E+07	3_26302403	29	26	19	32	28	26	0.690999	1	62	17	19	2	40	11	7.51E-04	0.0497587	25.75	17.3019404					2.6E+07	2.6E+07	0.20958	ID=promoter_32035;
3	3E+07	3_263341	104	6	76	25	55	51	0.479858	1	89	22	45	4	56	25	0.00803918	0.3312719	46.375	38.7185356								
3	3E+07	3_263557	50	34	25	23	42	24	0.016205	0.59851	50	72	50	39	61	45	0.50725599	1	42.875	16.0751583								
3	3E+07	3_263781	9	4	11	34	7	23	0.044696	1	1	6	23	3	4	13	0.06462238	1	11.375	11.4260042								
3	3E+07	3_26406109	1	17	7	5	9	6	0.554309	1	17	11	24	2	14	13	0.99999086	1	10.5	8.21149021					2.6E+07	2.6E+07	0.31307	ID=promoter_32039;
3	3E+07	3_266530	32	6	29	41	19	35	0.279151	1	0	24	0	0	12	0	0.00384008	0.1844256	16.5	16.8183573								
3	3E+07	3_274601	55	24	27	54	40	41	0.831668	1	0	0	16	11	0	14	5.26E-04	0.0367435	23.375	21.5535049								
3	3E+07	3_275451	5	30	9	9	18	9	0.098614	1	4	6	20	2	5	11	0.25523295	1	10.625	9.56089506								
3	3E+07	3_275708	5	16	10	11	11	11	0.932271	1	4	23	7	0	14	4	0.08931834	1	9.5	7.30948503								
3	3E+07	3_275821	1681	901	2114	1839	1291	1977	7.43E-13	#######	589	522	602	504	556	553	0.50346725	1	1094	670.779504								
3	3E+07	3_277623	5	18	1	4	12	3	0.030625	0.98374	0	30	19	43	15	31	0.03250921	0.9922526	15	15.4734796								
3	3E+07	3_27777074	17	19	16	10	18	13	0.404491	1	53	2	11	1	28	6	0.00180324	0.1003399	16.125	16.3045787					2.8E+07	2.8E+07	0.12087	ID=promoter_32102;
3	3E+07	3_278398	79	68	48	30	74	39	1.81E-04	0.01492	98	36	93	64	67	79	0.3486794	1	64.5	25.1623301								
3	3E+07	3_279758	68	34	20	58	51	39	0.128321	1	26	13	24	10	20	17	0.96598195	1	31.625	20.9211957								
3	3E+07	3_28015234	4	41	20	12	23	16	0.267873	1	43	39	47	14	41	31	0.69213115	1	27.5	16.758793	3E+07	2.8E+07	ID=ENSP0000219627-D2;;;		2.8E+07	2.8E+07	0.55082	ID=promoter_32111;
3	3E+07	3_280846	32	0	9	12	16	11	0.385778	1	8	24	0	1	16	1	0.00230968	0.1234682	10.75	11.7442995								
3	3E+07	3_28113968	3	62	14	8	33	11	5.89E-04	0.04065	336	110	19	5	223	12	1.60E-38	2.24E-35	69.625	113.838153	3E+07	2.8E+07	ID=ENSP0000349439;TXNDC11;;	TXNDC11	2.8E+07	2.8E+07	0.22483	ID=promoter_32120;
3	3E+07	3_281409	33	0	32	3	17	18	0.955187	1	23	21	23	25	22	24	0.85866092	1	20	12.224098								

3	3E+07	3_282941	129	57	144	125	93	135	0.284893	1	72	29	106	35	51	71	0.08311546	1	87.125	44.7387256								
3	3E+07	3_284040	36	41	0	16	39	8	2.44E-06	0.00034	15	51	76	47	33	62	0.00369661	0.1799564	35.25	24.2118625								
3	3E+07	3_284582	25	8	12	22	17	17	0.897282	1	33	5	14	0	19	7	0.09662636	1	14.875	11.0639376								
3	3E+07	3_285135	12	12	17	8	12	13	0.980472	1	28	19	11	4	24	8	0.03264408	0.9940318	13.875	7.39570339								
3	3E+07	3_285259	22	33	12	7	28	10	0.00287	0.14985	22	31	52	61	27	57	0.00141232	0.0835702	30	18.6547581								
3	3E+07	3_285330	5	20	8	0	13	4	0.041893	1	4	10	7	0	7	4	0.73798468	1	6.75	6.43095194								
3	3E+07	3_285752	12	31	3	0	22	2	4.25E-05	0.00421	3	3	23	3	3	13	0.02959638	0.9232309	9.75	11.3986215								
3	3E+07	3_287386	99	64	51	69	82	60	0.019644	0.69635	47	61	23	0	54	12	7.23E-06	0.0009506	51.75	29.9845198								
3	3E+07	3_287811	21	18	21	31	20	26	0.941925	1	42	20	34	1	31	18	0.28104051	1	23.5	12.3635409								
3	3E+07	3_290166	6	66	20	22	36	21	0.034407	1	43	11	63	4	27	34	0.49096177	1	29.375	24.8649926								
3	3E+07	3_294742	7	20	19	37	14	28	0.256487	1	57	28	21	24	43	23	0.09790572	1	26.625	14.9182295								
3	3E+07	3_29483377	1	24	5	13	13	9	0.46931	1	1	28	7	24	15	16	0.92652531	1	12.875	11.0510181					2.9E+07	2.9E+07	0.21739	ID=promoter_32163;
3	3E+07	3_296778	7	12	12	0	10	6	0.41703	1	0	0	0	18	0	9	0.00779604	0.3222758	6.125	7.18008158								
3	3E+07	3_296906	23	63	24	24	43	24	0.011717	0.46024	43	14	31	1	29	16	0.24173971	1	27.875	18.673415								
3	3E+07	3_296907	9	0	5	15	5	10	0.615109	1	0	4	17	28	2	23	6.46E-05	0.0062789	9.75	9.70640437								
3	3E+07	3_297324	14	62	2	33	38	18	0.004632	0.22041	43	17	36	22	30	29	0.98716634	1	28.625	18.8826262								
3	3E+07	3_29816672	21	34	40	29	28	35	0.969001	1	33	27	5	71	30	38	0.43894807	1	32.5	18.7920956	3E+07	3E+07	ID=ENSP00000371310;USP7;;	USP7	3E+07	3E+07	0.1727	ID=promoter_32173;
3	3E+07	3_298299	4	13	9	0	9	5	0.390586	1	29	27	11	1	28	6	0.00180324	0.1003399	11.75	11.0421271								
3	3E+07	3_299808	156	91	140	94	124	117	0.239004	1	127	78	51	5	103	28	7.19E-09	1.83E-06	92.75	49.3840634								
3	3E+07	3_299809	9	6	7	14	8	11	0.943069	1	2	0	8	0	1	4	0.36109265	1	5.75	4.86239212								
3	3E+07	3_30115221	71	8	33	41	40	37	0.580086	1	54	20	30	23	37	27	0.63815857	1	35	20.0713015	3E+07	3E+07	ID=ENSP00000371345;C16orf68;NM_024109;	C16orf68	3E+07	3E+07	0.27951	ID=promoter_32197;
3	3E+07	3_304631	26	12	16	38	19	27	0.813903	1	13	45	0	0	29	0	1.45E-06	0.0002324	18.75	16.4815915								
3	3E+07	3_304669	65	26	84	63	46	74	0.239982	1	100	129	106	35	115	71	0.02529704	0.8199738	76	35.4964787								
3	3E+07	3_304940	27	1	22	21	14	22	0.691767	1	75	68	27	44	72	36	0.00890416	0.3584328	35.625	25.1165142								
3	3E+07	3_306425	40	21	16	30	31	23	0.244309	1	118	42	27	34	80	31	1.17E-04	0.010441	41	32.3419233								
3	3E+07	3_307277	15	12	8	18	14	13	0.831264	1	49	27	28	4	38	16	0.02490784	0.8088272	20.125	14.3769409								
3	3E+07	3_308372	11	9	0	17	10	9	0.84607	1	0	20	3	1	10	2	0.09294023	1	7.625	7.89099849								
3	3E+07	3_30854160	11	25	28	21	18	25	0.86091	1	35	32	25	41	34	33	0.98250989	1	27.25	9.1456469	3E+07	3.1E+07	ID=ENSP00000309117;;NM_145891;					
3	3E+07	3_31029290	0	1	0	19	1	10	0.048828	1	1	10	16	3	6	10	0.51486293	1	6.25	7.70435869	3E+07	3.1E+07	ID=ENSP00000309117;;NM_145891;					
3	3E+07	3_31049827	68	50	26	24	59	25	4.94E-05	0.00479	92	50	34	53	71	44	0.10720797	1	49.625	22.6900702					3.1E+07	3.1E+07	0.24538	ID=promoter_32215;
3	3E+07	3_312795	11	43	14	16	27	15	0.059587	1	78	11	14	24	45	19	0.01291957	0.4837906	26.375	23.402915								
3	3E+07	3_313402	1791	951	1508	1183	1371	1346	2.08E-05	0.00228	298	149	214	43	224	129	7.84E-05	0.0074877	767.13	679.962066								
3	3E+07	3_313820	43	9	32	53	26	43	0.380683	1	5	44	22	47	25	35	0.2845844	1	31.875	18.0826476								
3	3E+07	3_315435	41	34	34	37	38	36	0.654883	1	3	88	79	20	46	50	0.7378574	1	42	28.3951706								
3	3E+07	3_317016	118	82	110	110	100	110	0.897595	1	23	24	57	32	24	45	0.01749321	0.6144624	69.5	40.6799354								
3	3E+07	3_317944	74	104	51	25	89	38	3.75E-07	#######	67	40	43	53	54	48	0.97780309	1	57.125	24.3746795								
3	3E+07	3_31847616	9	5	24	14	7	19	0.148295	1	16	11	12	16	14	14	0.98010536	1	13.375	5.65527567					3.2E+07	3.2E+07	0.12592	ID=promoter_32227;
3	3E+07	3_319675	46	68	8	12	57	10	1.17E-09	#######	40	10	47	1	25	24	0.99255599	1	29	24.2899156								
3	3E+07	3_320666	15	43	12	27	29	20	0.181062	1	0	0	6	1	0	4	0.11562589	1	13	15.2690162								
3	3E+07	3_32092725	1841	548	989	768	1195	879	4.11E-25	#######	427	302	154	74	365	114	2.41E-25	2.14E-22	637.88	573.856487					3.2E+07	3.2E+07	0.13695	ID=promoter_32231;
3	3E+07	3_321524	3	14	22	20	9	21	0.212308	1	0	0	19	0	0	10	0.00454609	0.2107253	9.75	9.92471662								
3	3E+07	3_322204	122	89	124	84	106	104	0.432046	1	100	30	53	61	65	57	0.95416238	1	82.875	33.211175								
3	3E+07	3_324411	187	58	173	212	123	193	0.040647	1	75	75	85	45	75	65	0.92404296	1	113.75	65.6761535								
3	3E+07	3_325476	251	89	269	149	170	209	0.901578	1	37	26	61	6	32	34	0.85766582	1	111	101.92294								
3	3E+07	3_325930	28	14	24	15	21	20	0.800056	1	22	19	8	1	21	5	0.01300804	0.4860836	16.375	8.83074985								
3	3E+07	3_332832	14	26	16	22	20	19	0.804666	1	0	30	12	50	15	31	0.03250921	0.9922526	21.25	14.8492424								
3	3E+07	3_333689	136	72	84	83	104	84	0.037933	1	36	39	59	33	38	46	0.47469033	1	67.75	34.4165658								
3	3E+07	3_334608	1	2	7	6	2	7	0.358464	1	27	29	7	0	28	4	2.96E-04	0.0229072	9.875	11.5193936								
3	3E+07	3_33471269	232	166	207	197	199	202	0.350185	1	103	72	142	92	88	117	0.0368544	1	151.38	58.7292517					3.3E+07	3.3E+07	0.20229	ID=promoter_32281;
3	3E+07	3_33471454	220	61	229	109	141	169	0.982205	1	66	96	56	0	81	28	1.72E-05	0.0020364	104.63	80.6933127					3.3E+07	3.3E+07	0.20229	ID=promoter_32281;
3	3E+07	3_334934	6	4	13	2	5	8	0.86114	1	17	0	8	0	9	4	0.4571786	1	6.25	6.15861766								
3	3E+07	3_335443	28	12	2	3	20	3	4.27E-04	0.03093	1	8	1	40	5	21	0.00386645	0.1846363	11.875	14.5350561								

3	3E+07	3_335652	11	31	14	15	21	15	0.333352	1	214	20	76	20	117	48	6.46E-06	0.0008661	50.125	69.4620914								
3	3E+07	3_337563	2	16	13	13	9	13	0.89076	1	0	37	4	0	19	2	0.00190863	0.1054641	10.625	12.4204612								
3	3E+07	3_337581	43	23	70	49	33	60	0.125172	1	0	0	11	7	0	9	0.00779604	0.3222758	25.375	25.9060804								
3	3E+07	3_337723	1	2	9	3	2	6	0.490921	1	108	78	36	4	93	20	8.16E-10	2.42E-07	30.125	41.2221768								
3	3E+07	3_33832696	80	20	56	49	50	53	0.876335	1	21	18	12	10	20	11	0.37181688	1	33.25	25.3588531					3.4E+07	3.4E+07	0.59342	ID=promoter_32317;
3	3E+07	3_338549	51	41	55	57	46	56	0.982689	1	33	30	6	0	32	3	1.74E-05	0.0020499	34.125	21.596544								
3	3E+07	3_339095	16	9	22	19	13	21	0.660505	1	0	0	1	0	0	1	0.58312783	1	8.375	9.42545944								
3	3E+07	3_33941921	17	8	8	3	13	6	0.146758	1	0	0	2	10	0	6	0.0393172	1	6	5.87974732					3.4E+07	3.4E+07	0.15319	ID=promoter_32326;
3	3E+07	3_340112	37	0	8	29	19	19	0.885913	1	0	48	43	25	24	34	0.27690884	1	23.75	19.0469345								
3	3E+07	3_340156	10	13	18	20	12	19	0.716431	1	0	0	9	0	0	5	0.06742467	1	8.75	8.11964109								
3	3E+07	3_341154	6	10	5	1	8	3	0.206144	1	35	46	24	3	41	14	0.0035084	0.1738771	16.25	16.7992347								
3	3E+07	3_34131292	6	18	9	7	12	8	0.45534	1	0	0	8	1	0	5	0.06742467	1	6.125	6.03412912					3.4E+07	3.4E+07	0.14549	ID=promoter_32343;
3	3E+07	3_34163217	132	19	154	82	76	118	0.160486	1	4	3	5	1	4	3	0.96209564	1	50	63.556499					3.4E+07	3.4E+07	0.15319	ID=promoter_32350;
3	3E+07	3_34235780	290	163	286	230	227	258	0.941789	1	306	127	161	22	217	92	9.24E-10	2.72E-07	198.13	98.2030513					3.4E+07	3.4E+07	0.31791	ID=promoter_32357;
3	3E+07	3_342800	349	147	332	282	248	307	0.819326	1	237	156	146	52	197	99	2.92E-06	0.0004312	212.63	104.203013								
3	3E+07	3_342880	46	265	17	45	156	31	1.36E-23	#######	122	95	188	71	109	130	0.15082601	1	106.13	83.5368824								
3	3E+07	3_34313960	11	47	17	4	29	11	0.003535	0.17827	25	16	9	25	21	17	0.91718448	1	19.25	13.4030913					3.4E+07	3.4E+07	0.1884	ID=promoter_32366;
3	3E+07	3_34317720	0	6	6	1	3	4	0.986002	1	5	12	23	2	9	13	0.58766318	1	6.875	7.52970309					3.4E+07	3.4E+07	0.11064	ID=promoter_32367;
3	3E+07	3_34321986	282	198	353	279	240	316	0.401693	1	73	58	56	30	66	43	0.19469786	1	166.13	127.125852					3.4E+07	3.4E+07	0.17587	ID=promoter_32368;
3	3E+07	3_343764	54	204	88	111	129	100	0.00744	0.3212	128	204	318	156	166	237	9.67E-05	0.0089482	157.88	83.3759772								
3	3E+07	3_34512054	46	51	22	22	49	22	5.23E-04	0.0367	36	25	45	64	31	55	0.01322973	0.49354	38.875	15.2918045	3E+07	3.5E+07	ID=ENSP00000262367;CREBBP;NM_004380;	CREBBP				
3	3E+07	3_34522640	0	27	12	1	14	7	0.165008	1	2	8	9	1	5	5	0.99284883	1	7.5	9.05538514	3E+07	3.5E+07	ID=ENSP00000262367;CREBBP;NM_004380;	CREBBP				
3	3E+07	3_346726	0	7	10	12	4	11	0.322758	1	14	14	9	36	14	23	0.23132274	1	12.75	10.430039								
3	3E+07	3_346913	12	13	25	9	13	17	0.957342	1	89	40	18	15	65	17	4.52E-06	0.0006278	27.625	26.6829935								
3	3E+07	3_34694289	88	78	126	153	83	140	0.030922	0.99238	83	42	25	33	63	29	0.00629125	0.2727856	78.5	45.0111097					3.5E+07	3.5E+07	0.24981	ID=promoter_32403;
3	3E+07	3_34698073	17	10	34	57	14	46	0.001998	0.11283	121	28	67	24	75	46	0.08479286	1	44.75	36.3779761					3.5E+07	3.5E+07	0.31405	ID=promoter_32405;
3	3E+07	3_34716855	141	140	146	118	141	132	0.175437	1	27	56	20	32	42	26	0.26520775	1	85	56.3281204					3.5E+07	3.5E+07	0.29298	ID=promoter_32408;
3	3E+07	3_347841	8	44	3	9	26	6	3.38E-04	0.02566	21	0	22	0	11	11	0.98433491	1	13.375	15.0422025								
3	3E+07	3_34803578	18	12	13	4	15	9	0.269309	1	0	0	5	1	0	3	0.19828566	1	6.625	6.84392327					3.5E+07	3.5E+07	0.1237	ID=promoter_32422;
3	4E+07	3_350301	770	354	456	302	562	379	3.58E-16	#######	587	246	140	122	417	131	1.20E-28	1.22E-25	372.13	223.321957								
3	4E+07	3_350725	2	26	1	5	14	3	0.010924	0.43763	1	16	17	6	9	12	0.70935077	1	9.25	9.28516482								
3	4E+07	3_35193140	23	20	11	17	22	14	0.192025	1	13	36	23	7	25	15	0.40601431	1	18.75	9.01981945	4E+07	3.5E+07	ID=ENSP00000385495-D2;C16orf35;;	C16orf35	3.5E+07	3.5E+07	0.16271	ID=promoter_32463;
3	4E+07	3_35289911	47	18	50	27	33	39	0.999494	1	13	5	11	2	9	7	0.94097808	1	21.625	18.2829937	4E+07	3.5E+07	ID=ENSP00000293872;LUC7L;NM_201412;	LUC7L	3.5E+07	3.5E+07	0.34482	ID=promoter_32483;

3	4E+07	3_35318345	11	25	17	9	18	13	0.404491	1	28	11	16	20	20	18	0.99585638	1	17.125	6.87516234	4E+07	3.5E+07	ID=ENSP0000382814;ARHGDIG;NM_032039;	ARHGDIG				
3	4E+07	3_35377212	5	15	8	4	10	6	0.41703	1	21	1	8	0	11	4	0.25131486	1	7.75	7.12640963	4E+07	3.5E+07	ID=ENSP0000262320;AXIN1;NM_003502;	AXIN1				
3	4E+07	3_35392122	14	31	19	12	23	16	0.267873	1	57	19	43	14	38	29	0.73182863	1	26.125	16.2870457	4E+07	3.5E+07	ID=ENSP0000262320;AXIN1;NM_003502;	AXIN1	3.5E+07	3.5E+07	0.28491	ID=promoter_32512;
3	4E+07	3_35408017	7	17	7	3	12	5	0.126784	1	1	10	19	7	6	13	0.20797135	1	8.875	6.28916301	4E+07	3.5E+07	ID=ENSP0000262320;AXIN1;NM_003502;	AXIN1	3.5E+07	3.5E+07	0.34092	ID=promoter_32516;
3	4E+07	3_35423923	607	267	460	341	437	401	0.001776	0.10214	167	164	202	140	166	171	0.62589496	1	293.5	166.334431					3.5E+07	3.5E+07	0.64749	ID=promoter_32522;
3	4E+07	3_35430250	1	18	20	16	10	18	0.549586	1	25	15	22	55	20	39	0.02178763	0.7342224	21.5	15.3157249					3.5E+07	3.5E+07	0.38361	ID=promoter_32523;
3	4E+07	3_35439715	12	18	19	4	15	12	0.610117	1	13	8	5	22	11	14	0.73202476	1	12.625	6.67484617	4E+07	3.5E+07	ID=ENSP0000250930;TMEM8;NM_021259;	TMEM8				
3	4E+07	3_35447664	192	94	222	150	143	186	0.648969	1	156	122	135	81	139	108	0.38670738	1	144	47.2107736					3.5E+07	3.5E+07	0.62301	ID=promoter_32526;
3	4E+07	3_354986	10	5	27	30	8	29	0.011876	0.46354	0	0	5	1	0	3	0.19828566	1	9.75	12.0682582								
3	4E+07	3_35509708	7	17	5	7	12	6	0.213448	1	0	0	14	0	0	7	0.02292696	0.7568963	6.25	6.49725217					3.6E+07	3.6E+07	0.37265	ID=promoter_32537;
3	4E+07	3_35521728	96	35	62	38	66	50	0.063172	1	48	17	20	3	33	12	0.01587283	0.5667339	39.875	29.3035225					3.6E+07	3.6E+07	0.17011	ID=promoter_32538;
3	4E+07	3_355223	0	14	3	8	7	6	0.851931	1	0	5	19	0	3	10	0.11508955	1	6.125	7.12014245								
3	4E+07	3_355836	141	34	87	119	88	103	0.999892	1	23	16	19	22	20	21	0.92353071	1	57.625	50.4718804								
3	4E+07	3_35585141	44	77	71	61	61	66	0.90177	1	65	37	61	88	51	75	0.03727818	1	63	16.6218445					3.6E+07	3.6E+07	0.44794	ID=promoter_32556;
3	4E+07	3_35642401	8598	6081	10852	13134	7340	####	########	#######	8510	6736	11486	4199	7623	7843	7.52E-10	2.24E-07	8699.5	3001.79032					3.6E+07	3.6E+07	0.29193	ID=promoter_32572;
3	4E+07	3_356961	63	24	73	65	44	69	0.319562	1	32	20	58	33	26	46	0.02755124	0.8757446	46	20.8669253								
3	4E+07	3_35712929	229	74	228	160	152	194	0.737785	1	70	26	62	15	48	39	0.82910971	1	108	86.0514796					3.6E+07	3.6E+07	0.43994	ID=promoter_32588;
3	4E+07	3_35722822	323	94	329	372	209	351	1.98E-04	0.0161	151	158	218	62	155	140	0.97538945	1	213.38	116.307398					3.6E+07	3.6E+07	0.43867	ID=promoter_32590;
3	4E+07	3_35740281	57	25	72	50	41	61	0.49837	1	52	30	18	8	41	13	0.00193653	0.1067879	39	21.9805108					3.6E+07	3.6E+07	0.62657	ID=promoter_32596;
3	4E+07	3_35745169	76	10	55	51	43	53	0.971253	1	28	62	60	16	45	38	0.91413411	1	44.75	23.8012605	4E+07	3.6E+07	ID=ENSP0000248142;WDR24;;	WDR24	3.6E+07	3.6E+07	0.35361	ID=promoter_32597;
3	4E+07	3_35760841	24	20	16	26	22	21	0.795425	1	1	10	6	6	6	6	0.99142475	1	13.625	9.22632104					3.6E+07	3.6E+07	0.42439	ID=promoter_32603;
3	4E+07	3_35772497	6	32	21	18	19	20	0.944476	1	15	27	26	4	21	15	0.7415049	1	18.625	9.97049218					3.6E+07	3.6E+07	0.2369	ID=promoter_32607;
3	4E+07	3_358189	13	37	12	22	25	17	0.214068	1	49	11	0	0	30	0	9.14E-07	0.0001543	18	17.3040045								
3	4E+07	3_358430	10	59	13	35	35	24	0.122315	1	48	12	36	10	30	23	0.79011982	1	27.875	19.2831347								
3	4E+07	3_358606	14	43	5	7	29	6	7.05E-05	0.00656	11	21	27	19	16	23	0.39752759	1	18.375	12.3628187								
3	4E+07	3_358635	97	18	82	90	58	86	0.384512	1	7	5	7	1	6	4	0.87665919	1	38.375	42.928304								
3	4E+07	3_35947169	51	13	96	72	32	84	3.58E-04	0.02695	12	55	19	3	34	11	0.0064318	0.2773637	40.125	33.4426481	4E+07	3.6E+07	ID=ENSP0000250894;MAPK8IP3;;	MAPK8IP3				

3	4E+07	3_35966205	121	68	101	43	95	72	0.018899	0.67328	4	5	24	22	5	23	0.00150862	0.0878849	48.5	44.0746121	4E+07	3.6E+07	ID=ENSP00000250894;MAPK8IP3;;	MAPK8IP3				
3	4E+07	3_35968867	698	351	758	646	525	702	0.07567	1	24	18	18	2	21	10	0.21021716	1	314.38	340.768014	4E+07	3.6E+07	ID=ENSP00000250894;MAPK8IP3;;	MAPK8IP3	3.6E+07	3.6E+07	0.38207	ID=promoter_32652;
3	4E+07	3_36017547	2	11	9	20	7	15	0.409369	1	17	9	12	6	13	9	0.79589064	1	10.75	5.75077635	4E+07	3.6E+07	ID=ENSP00000380562;IFT140;NM_014714;	IFT140				
3	4E+07	3_36019587	2	9	22	39	6	31	0.00134	0.08025	55	15	18	4	35	11	0.00454855	0.2107253	20.5	18.197331	4E+07	3.6E+07	ID=ENSP00000380562;IFT140;NM_014714;	IFT140				
3	4E+07	3_36104180	8	14	6	10	11	8	0.5832	1	19	4	0	14	12	7	0.61698677	1	9.375	6.16296543	4E+07	3.6E+07	ID=ENSP00000372193;CLCN7;;	CLCN7				
3	4E+07	3_36144468	19	14	15	15	17	15	0.722447	1	0	10	19	25	5	22	0.00242132	0.1280458	14.625	7.38603895					3.6E+07	3.6E+07	0.32746	ID=promoter_32704;
3	4E+07	3_36161810	79	65	84	65	72	75	0.771739	1	20	0	29	1	10	15	0.49251655	1	42.875	34.3986607					3.6E+07	3.6E+07	0.26548	ID=promoter_32710;
3	4E+07	3_361874	13	0	13	5	7	9	0.983538	1	0	60	26	38	30	32	0.85844914	1	19.375	20.9757513								
3	4E+07	3_36196766	2	4	2	4	3	3	0.981055	1	28	0	10	34	14	22	0.29543416	1	10.5	13.082158					3.6E+07	3.6E+07	0.51515	ID=promoter_32718;
3	4E+07	3_362039	594	185	393	420	390	407	0.2563	1	108	107	110	125	108	118	0.46631763	1	255.25	188.045397								
3	4E+07	3_362950	27	25	7	6	26	7	7.99E-04	0.0525	0	0	3	1	0	2	0.34003807	1	8.625	11.0445526								
3	4E+07	3_36381091	23	3	10	5	13	8	0.344217	1	0	17	28	63	9	46	9.14E-07	0.0001543	18.625	20.4585819	4E+07	3.6E+07	ID=ENSP00000248125;ZNF598;;	ZNF598	3.6E+07	3.6E+07	0.35138	ID=promoter_32754;
3	4E+07	3_364077	403	164	289	338	284	314	0.770617	1	165	147	192	46	156	119	0.27603473	1	218	116.307474								
3	4E+07	3_36443990	37	0	36	3	19	20	0.944476	1	0	0	0	1	0	1	0.58312783	1	9.625	16.6213073	4E+07	3.6E+07	ID=ENSP00000219476;TSC2;NM_000548;	TSC2	3.6E+07	3.6E+07	0.4871	ID=promoter_32773;
3	4E+07	3_36445494	37	29	29	27	33	28	0.448796	1	14	1	19	17	8	18	0.09791732	1	21.625	11.2496032	4E+07	3.6E+07	ID=ENSP00000219476;TSC2;NM_000548;	TSC2	3.6E+07	3.6E+07	0.4871	ID=promoter_32773;
3	4E+07	3_36475915	217	49	255	169	133	212	0.020767	0.72593	33	45	64	5	39	35	0.99032895	1	104.63	94.6722731					3.6E+07	3.6E+07	0.7862	ID=promoter_32782;
3	4E+07	3_364884	42	23	71	42	33	57	0.204975	1	0	0	11	1	0	6	0.0393172	1	23.75	25.932605								
3	4E+07	3_364943	80	42	46	41	61	44	0.045895	1	56	14	21	19	35	20	0.21889545	1	39.875	21.957345								
3	4E+07	3_365029	0	22	7	0	11	4	0.10516	1	3	17	11	6	10	9	0.99792604	1	8.25	7.95972002								
3	4E+07	3_36533721	135	187	134	103	161	119	5.95E-04	0.04096	25	11	59	14	18	37	0.01748595	0.6144624	83.5	65.9046931					3.7E+07	3.7E+07	0.25458	ID=promoter_32793;
3	4E+07	3_366134	15	14	8	0	15	4	0.015825	0.58672	1	0	2	7	1	5	0.22510075	1	5.875	6.12809688								
3	4E+07	3_36738906	0	11	2	1	6	2	0.258649	1	44	16	4	23	30	14	0.09649838	1	12.625	15.0611847	4E+07	3.7E+07	ID=ENSP00000371818;ABCA3;;	ABCA3				
3	4E+07	3_36781958	3348	2235	4958	5294	2792	5126	7.64E-81	#######	2842	1734	2578	1117	2288	1848	5.98E-05	0.0059171	3013.3	1471.27823					3.7E+07	3.7E+07	0.75381	ID=promoter_32832;
3	4E+07	3_368053	143	90	126	96	117	111	0.276132	1	37	29	43	12	33	28	0.94230752	1	72	48.3617322								
3	4E+07	3_36820917	6	27	9	6	17	8	0.087954	1	57	17	9	3	37	6	4.34E-05	0.0045067	16.75	17.9900766					3.7E+07	3.7E+07	0.19684	ID=promoter_32845;
3	4E+07	3_369143	2	21	12	5	12	9	0.592799	1	0	8	11	0	4	6	0.75330063	1	7.375	7.20986427								
3	4E+07	3_36960689	257	79	227	205	168	216	0.671412	1	149	121	120	20	135	70	3.35E-04	0.0254157	147.25	79.3054673					3.7E+07	3.7E+07	0.48719	ID=promoter_32864;

3	4E+07	3_37011471	28	2	1	10	15	6	0.065303	1	0	0	0	0	0	0	1	1	5.125	9.8479512	4E+07	3.7E+07	ID=ENSP00000373876;PDPK1;;	PDPK1				
3	4E+07	3_370392	88	86	104	64	87	84	0.444013	1	55	8	6	11	32	9	0.00363228	0.1775497	52.75	39.7087612								
3	4E+07	3_370680	12	6	10	50	9	30	0.016049	0.59429	0	5	0	0	3	0	0.24893445	1	10.375	16.664226								
3	4E+07	3_371424	17	8	7	4	13	6	0.146758	1	0	4	10	1	2	6	0.31385293	1	6.375	5.47559521								
3	4E+07	3_371608	101	26	85	77	64	81	0.901756	1	42	19	41	12	31	27	0.97229028	1	50.375	33.1228773								
3	4E+07	3_372514	174	104	144	129	139	137	0.350875	1	28	62	136	90	45	113	2.93E-08	6.73E-06	108.38	47.4068636								
3	4E+07	3_37373602	291	167	305	196	229	251	0.759351	1	108	108	58	63	108	61	0.00756488	0.3162913	162	96.1546374					3.7E+07	3.7E+07	0.37293	ID=promoter_32911;
3	4E+07	3_37578262	136	15	105	112	76	109	0.403167	1	19	21	18	65	20	42	0.00833392	0.3402748	61.375	50.0083922					3.8E+07	3.8E+07	0.61636	ID=promoter_32913;
3	4E+07	3_37579180	3575	1735	4162	3261	2655	3712	4.06E-11	#######	2116	1610	2320	700	1863	1510	5.34E-04	0.0372477	2434.9	1150.17023	4E+07	3.8E+07	ID=ENSP00000349351;BICD2;NM_001003800;	BICD2	3.8E+07	3.8E+07	0.61636	ID=promoter_32913;
3	4E+07	3_378091	221	80	102	82	151	92	3.02E-06	0.00041	14	27	28	5	21	17	0.91718448	1	69.875	70.7539752								
3	4E+07	3_378863	0	28	15	1	14	8	0.253058	1	0	2	7	4	1	6	0.13767373	1	7.125	9.80433578								
3	4E+07	3_379251	21	22	11	16	22	14	0.192025	1	0	14	15	2	7	9	0.80856893	1	12.625	8.03452372								
3	4E+07	3_38018710	0	77	1	7	39	4	3.14E-08	#######	2	0	26	1	1	14	0.00214405	0.1161796	14.25	26.8208341	4E+07	3.8E+07	ID=ENSP00000277165;FAM120A;NM_014612;	FAM120A				
3	4E+07	3_391402	10	16	8	17	13	13	0.920459	1	1	41	13	13	21	13	0.51498325	1	14.875	11.7039371								
3	4E+07	3_394892	2	5	5	3	4	4	0.97482	1	10	0	36	8	5	22	0.00242132	0.1280458	8.625	11.5131912								
3	4E+07	3_39714525	17	126	32	40	72	36	9.46E-05	0.00849	115	52	55	219	84	137	1.97E-04	0.0162685	82	67.5193094	4E+07	4E+07	ID=ENSP00000283206;TMEM87B;NM_032824;	TMEM87B				
3	4E+07	3_40047760	0	27	9	15	14	12	0.725787	1	11	13	8	1	12	5	0.30420563	1	10.5	8.51888658					4E+07	4E+07	0.32582	ID=promoter_32952;
3	4E+07	3_40273857	208	57	213	188	133	201	0.075463	1	46	42	76	1	44	39	0.97939611	1	103.88	84.9898103					4E+07	4E+07	0.13772	ID=promoter_32953;
3	4E+07	3_404876	17	13	2	6	15	4	0.015825	0.58672	21	5	18	13	13	16	0.74830213	1	11.875	6.85435211								
3	4E+07	3_40774257	30	41	33	13	36	23	0.069202	1	47	32	25	70	40	48	0.4816091	1	36.375	16.9531919	4E+07	4.1E+07	ID=ENSP00000374461;ACOXL;NM_001105516;	ACOXL				
3	4E+07	3_40786285	0	24	0	1	12	1	0.003675	0.18279	0	17	0	0	9	0	0.01542606	0.5535504	5.25	9.60282696	4E+07	4.1E+07	ID=ENSP00000374461;ACOXL;NM_001105516;	ACOXL				
3	4E+07	3_409568	16	6	11	0	11	6	0.3028	1	44	4	21	0	24	11	0.1422748	1	12.75	14.6653138								
3	4E+07	3_409716	623	273	361	375	448	368	2.36E-06	0.00033	85	73	98	106	79	102	0.0849804	1	249.25	196.393737								
3	4E+07	3_41293357	767	434	1068	973	601	1021	3.97E-12	#######	247	380	411	145	314	278	0.85543043	1	553.13	340.54051					4.1E+07	4.1E+07	0.16851	ID=promoter_32968;
3	4E+07	3_420571	8	27	20	17	18	19	0.949854	1	30	20	34	39	25	37	0.18968026	1	24.375	10.0418766								
3	4E+07	3_420601	11	26	1	25	19	13	0.317047	1	12	26	10	3	19	7	0.09662636	1	14.25	10.1945364								
3	4E+07	3_42290994	37	74	35	38	56	37	0.023015	0.78841	48	59	73	44	54	59	0.68287454	1	51	15.8384703					4.2E+07	4.2E+07	0.22395	ID=promoter_32996;
3	4E+07	3_426040	70	24	37	12	47	25	0.004634	0.22041	55	58	19	26	57	23	0.00246953	0.1303537	37.625	21.0097766								
3	4E+07	3_42639431	2899	2920	3822	4311	2910	4067	4.58E-12	#######	2173	1747	3034	780	1960	1907	0.32316272	1	2710.8	1129.17008					4.3E+07	4.3E+07	0.1802	ID=promoter_33000;
3	4E+07	3_42734522	6285	4123	7863	8244	5204	8054	8.06E-54	#######	3836	3069	7389	2351	3453	4870	4.10E-79	1.19E-75	5395	2320.73381					4.3E+07	4.3E+07	0.65469	ID=promoter_33002;
3	4E+07	3_42792695	883	373	899	726	628	813	0.177995	1	516	327	365	129	422	247	5.23E-08	1.14E-05	527.25	281.121606					4.3E+07	4.3E+07	0.75986	ID=promoter_33004;
3	4E+07	3_42901824	0	18	2	7	9	5	0.390586	1	2	20	12	3	11	8	0.87190251	1	8	7.76438757					4.3E+07	4.3E+07	0.23104	ID=promoter_33007;

3	4E+07	3_42984701	78	32	79	36	55	58	0.852149	1	0	0	0	0	0	0	1	1	28.125	34.4691891	4E+07	4.3E+07	ID=ENSP0000376996;GCC2;;	GCC2	4.3E+07	4.3E+07	0.14151	ID=promoter_33010;
3	4E+07	3_435802	13	4	2	13	9	8	0.848833	1	2	0	6	1	1	4	0.36109265	1	5.125	5.19443383								
3	4E+07	3_436926	95	60	125	89	78	107	0.574494	1	0	24	26	32	12	29	0.01494463	0.5435394	56.375	43.1308226								
3	4E+07	3_437243	10	28	58	101	19	80	5.91E-07	#######	119	9	10	0	64	5	9.53E-11	3.20E-08	41.875	45.9236556								
3	4E+07	3_437545	9	24	8	3	17	6	0.027279	0.89953	69	32	54	2	51	28	0.07905636	1	25.125	25.0281984								
3	4E+07	3_43855995	0	17	2	2	9	2	0.058587	1	16	0	1	2	8	2	0.20550608	1	5	7.15142343	4E+07	4.4E+07	ID=ENSP0000355273;ST6GAL2;;	ST6GAL2	4.4E+07	4.4E+07	0.41346	ID=promoter_33018;
3	4E+07	3_43863119	7	34	36	20	21	28	0.906033	1	1	35	40	4	18	22	0.68341813	1	22.125	16.1549417	4E+07	4.4E+07	ID=ENSP0000355273;ST6GAL2;;	ST6GAL2				
3	4E+07	3_439044	34	37	21	39	36	30	0.380805	1	53	68	69	20	61	45	0.50725599	1	42.625	19.0708266								
3	4E+07	3_43979866	58	59	65	42	59	54	0.418093	1	5	29	9	0	17	5	0.05852787	1	33.375	26.3760579					4.4E+07	4.4E+07	0.14717	ID=promoter_33022;
3	4E+07	3_440874	13	40	12	11	27	12	0.014569	0.54886	2	19	16	6	11	11	0.98433491	1	14.875	11.4696805								
3	4E+07	3_441923	26	58	34	33	42	34	0.270679	1	13	40	34	39	27	37	0.29896222	1	34.625	12.7608497								
3	4E+07	3_442611	42	42	35	42	42	39	0.574402	1	16	40	16	15	28	16	0.29661245	1	31	12.9062554								
3	4E+07	3_44299162	105	76	63	73	91	68	0.017893	0.6473	22	42	35	65	32	50	0.06589199	1	60.125	26.3950076					4.4E+07	4.4E+07	0.12737	ID=promoter_33035;
3	4E+07	3_446013	3	12	22	3	8	13	0.766846	1	18	10	5	35	14	20	0.45746548	1	13.5	11.0969751								
3	4E+07	3_446140	49	31	48	34	40	41	0.831668	1	66	2	42	22	34	32	0.99811495	1	36.75	19.3667609								
3	4E+07	3_44664890	878	388	839	565	633	702	0.592608	1	215	263	274	98	239	186	0.20087921	1	440	291.755279					4.5E+07	4.5E+07	0.46767	ID=promoter_33045;
3	4E+07	3_448430	24	43	51	54	34	53	0.430266	1	53	4	13	31	29	22	0.77701989	1	34.125	19.2014694								
3	4E+07	3_44843859	2	88	12	14	45	13	8.88E-06	0.00108	2	28	51	33	15	42	5.71E-04	0.039558	28.75	29.1633331					4.5E+07	4.5E+07	0.15527	ID=promoter_33054;
3	4E+07	3_448919	30	3	13	15	17	14	0.615566	1	67	6	33	3	37	18	0.07385545	1	21.25	21.7567723								
3	5E+07	3_450157	9	0	0	0	5	0	0.05327	1	17	6	13	2	12	8	0.76853133	1	5.875	6.57783073								
3	5E+07	3_450467	14	42	26	1	28	14	0.027228	0.89953	21	35	25	74	28	50	0.01836895	0.6365951	29.75	21.7895781								
3	5E+07	3_452092	39	29	31	37	34	34	0.805115	1	85	1	12	5	43	9	5.12E-05	0.0051875	29.875	26.5837625								
3	5E+07	3_453759	184	101	107	60	143	84	1.91E-06	0.00027	67	94	32	32	81	32	1.42E-04	0.0123104	84.625	49.552101								
3	5E+07	3_456689	26	12	27	22	19	25	0.931133	1	9	6	23	3	8	13	0.4490005	1	16	9.56182887								
3	5E+07	3_461531	11	2	26	11	7	19	0.148295	1	1	0	13	0	1	7	0.08320466	1	8	9.1025899								
3	5E+07	3_464413	21	0	22	13	11	18	0.682529	1	33	0	5	0	17	3	0.01239331	0.4669004	11.75	12.5555907								
3	5E+07	3_464966	19	36	24	19	28	22	0.367037	1	19	10	9	0	15	5	0.11779525	1	17	10.8496215								
3	5E+07	3_468667	6	47	12	7	27	10	0.004472	0.21437	48	24	26	2	36	14	0.01744443	0.6140941	21.5	18.1265393								
3	5E+07	3_46936631	415	119	410	324	267	367	0.142611	1	186	107	146	101	147	124	0.74089338	1	226	135.444243					4.7E+07	4.7E+07	0.32983	ID=promoter_33068;
3	5E+07	3_482281	1	9	0	0	5	0	0.05327	1	58	0	0	0	29	0	1.45E-06	0.0002324	8.5	20.2414003								
3	5E+07	3_489217	17	22	11	9	20	10	0.076235	1	0	5	9	0	3	5	0.7175395	1	9.125	7.69856202								
3	5E+07	3_489286	0	0	12	19	0	16	0.001091	0.06822	11	10	10	18	11	14	0.73202476	1	10	7.07106781								
3	5E+07	3_489418	1	37	20	3	19	12	0.233425	1	46	0	5	0	23	3	9.46E-04	0.0600857	14	18.3303028								
3	5E+07	3_489629	1	51	2	11	26	7	7.99E-04	0.0525	0	0	15	12	0	14	5.26E-04	0.0367435	11.5	17.0629088								
3	5E+07	3_489772	24	36	2	14	30	8	2.50E-04	0.01962	2	37	48	11	20	30	0.24257255	1	21.75	17.2606075								
3	5E+07	3_494908	9	15	16	18	12	17	0.88186	1	26	23	17	14	25	16	0.50792242	1	17.25	5.28474895								
3	5E+07	3_49597451	262	13	91	93	138	92	1.18E-04	0.01037	106	56	44	8	81	26	5.30E-06	0.0007276	84.125	80.6215808					5E+07	5E+07	0.47953	ID=promoter_33120;
3	5E+07	3_49628450	142	35	50	32	89	41	1.89E-06	0.00027	94	79	38	71	87	55	0.08212693	1	67.625	37.5877545	5E+07	5E+07	ID=ENSP0000387309;CHST10;;	CHST10				
3	5E+07	3_49675907	450	111	374	493	281	434	0.001506	0.08902	189	137	161	47	163	104	0.01130161	0.4350704	245.25	168.686311					5E+07	5E+07	0.68632	ID=promoter_33122;
3	5E+07	3_504175	12	41	32	24	27	28	0.900886	1	22	48	67	21	35	44	0.40199623	1	33.375	17.8560714								
3	5E+07	3_50872946	9	5	23	1	7	12	0.724589	1	0	12	6	6	6	6	0.99142475	1	7.75	7.28501397	5E+07	5.1E+07	ID=ENSP0000386956;TSGA10;NM_182911;	TSGA10				
3	5E+07	3_51152536	467	237	417	479	352	448	0.518288	1	252	246	202	118	249	160	0.0013381	0.0796403	302.25	133.895215					5.1E+07	5.1E+07	0.73589	ID=promoter_33140;

3	5E+07	3_51194555	6	93	29	8	50	19	6.06E-05	0.00575	11	59	17	33	35	25	0.60746796	1	32	30.185143	5E+07	5.1E+07	ID=ENSP00000386841;MGAT4A;;	MGAT4A				
3	5E+07	3_51313262	0	0	2	0	0	1	0.652937	1	0	9	3	29	5	16	0.03625394	1	5.375	10.0276404	5E+07	5.1E+07	ID=ENSP00000366134;;					
3	5E+07	3_515544	109	128	135	105	119	120	0.504652	1	51	20	41	38	36	40	0.73226539	1	78.375	45.5127533								
3	5E+07	3_517632	2	1	15	21	2	18	0.005391	0.24994	16	10	28	1	13	15	0.84581615	1	11.75	10.0534287								
3	5E+07	3_526303	24	51	17	7	38	12	1.18E-04	0.01037	55	57	48	7	56	28	0.0254237	0.8237054	33.25	21.70418								
3	5E+07	3_528648	6	39	6	15	23	11	0.040732	1	53	0	18	3	27	11	0.06118138	1	17.5	18.9359824								
3	5E+07	3_537970	12	26	9	18	19	14	0.411928	1	2	41	19	16	22	18	0.92557456	1	17.875	11.7769448								
3	5E+07	3_538972	1413	1032	1423	1357	1223	1390	0.723699	1	156	61	105	34	109	70	0.05477388	1	697.63	662.760879								
3	5E+07	3_540916	122	88	70	141	105	106	0.551613	1	143	111	115	100	127	108	0.80453753	1	111.25	25.0470985								
3	5E+07	3_54583446	450	151	289	307	301	298	0.115631	1	165	120	109	46	143	78	6.98E-04	0.0468779	204.63	132.945679					5.5E+07	5.5E+07	0.18141	ID=promoter_33204;
3	5E+07	3_54659932	20	13	18	15	17	17	0.897282	1	22	10	6	5	16	6	0.14768941	1	13.625	6.30051018					5.5E+07	5.5E+07	0.16148	ID=promoter_33205;
3	6E+07	3_556985	4	8	3	17	6	10	0.79153	1	18	22	4	1	20	3	0.00346999	0.1723921	9.625	8.1229393								
3	6E+07	3_586706	29	108	30	57	69	44	0.005823	0.26582	71	42	45	24	57	35	0.15471957	1	50.75	27.9169687								
3	6E+07	3_608428	61	52	29	65	57	47	0.198617	1	32	57	47	53	45	50	0.67738509	1	49.5	12.983506								
3	6E+07	3_608814	36	64	25	59	50	42	0.276891	1	112	21	138	30	67	84	0.18064402	1	60.625	43.1341346								
3	6E+07	3_609173	1	8	4	1	5	3	0.645778	1	40	13	13	1	27	7	0.00575936	0.2548307	10.125	13.0978461								
3	6E+07	3_619081	4	0	11	18	2	15	0.01804	0.65084	101	8	26	1	55	14	2.27E-05	0.0025764	21.125	33.4511905								
3	6E+07	3_626461	10	43	21	13	27	17	0.124321	1	4	7	36	5	6	21	0.00855938	0.348087	17.375	14.7835575								
3	6E+07	3_62771489	22	26	4	13	24	9	0.00876	0.36763	80	30	24	61	55	43	0.70389935	1	32.5	25.3320802	6E+07	6.3E+07	ID=ENSP00000350547;SEMA4F;NM_004263;	SEMA4F				
3	6E+07	3_62899711	20	20	9	10	20	10	0.076235	1	3	1	15	0	2	8	0.1303879	1	9.75	8.06668634	6E+07	6.3E+07	ID=ENSP00000264094;LOXL3;NM_032603;	LOXL3				
3	6E+07	3_630321	28	0	32	21	14	27	0.312392	1	0	0	11	0	0	6	0.0393172	1	11.5	13.6904555								
3	6E+07	3_630707	340	226	309	301	283	305	0.590739	1	60	54	93	99	57	96	0.0014072	0.0833363	185.25	121.410461								
3	6E+07	3_63082718	1	6	3	8	4	6	0.929647	1	9	4	5	5	7	5	0.9051169	1	5.125	2.58774585					6.3E+07	6.3E+07	0.31965	ID=promoter_33246;
3	6E+07	3_632575	1	11	0	5	6	3	0.462004	1	0	0	16	44	0	30	9.40E-08	1.96E-05	9.625	15.1085359								
3	6E+07	3_633229	6	36	36	33	21	35	0.441201	1	9	10	25	30	10	28	0.006881	0.2937185	23.125	12.7888065								
3	6E+07	3_63395238	19	0	27	9	10	18	0.549586	1	5	12	3	10	9	7	0.94097808	1	10.625	8.83074985					6.3E+07	6.3E+07	0.50765	ID=promoter_33262;
3	6E+07	3_635198	50	14	53	41	32	47	0.616656	1	0	0	0	0	0	0	1	1	19.75	24.0994961								
3	6E+07	3_63532968	3	23	7	23	13	15	0.999061	1	2	33	14	3	18	9	0.30718372	1	13.5	11.6863779					6.4E+07	6.4E+07	0.68893	ID=promoter_33270;
3	6E+07	3_63567760	0	29	3	3	15	3	0.006447	0.2884	24	0	10	2	12	6	0.45526626	1	8.875	11.3947043	6E+07	6.4E+07	ID=ENSP00000264093;DGUOK;NM_080916;	DGUOK				
3	6E+07	3_636016	15	58	26	62	37	44	0.998103	1	12	47	61	6	30	34	0.72414385	1	35.875	23.6729955								
3	6E+07	3_637697	183	32	123	125	108	124	0.986899	1	48	32	48	22	40	35	0.96931568	1	76.625	59.0543031								
3	6E+07	3_639586	11	19	19	13	15	16	0.965652	1	4	52	12	16	28	14	0.15944813	1	18.25	14.4790489								
3	6E+07	3_63976142	8	0	2	4	4	3	0.840045	1	19	8	3	2	14	3	0.04246461	1	5.75	6.06512278	6E+07	6.4E+07	ID=ENSP00000258106;EMX1;NM_004097;	EMX1				
3	6E+07	3_640507	283	148	372	232	216	302	0.142692	1	149	100	120	79	125	100	0.54633398	1	185.38	101.511347								
3	6E+07	3_645010	47	60	49	28	54	39	0.06618	1	66	38	31	40	52	36	0.40124865	1	44.875	13.3356397								
3	6E+07	3_646671	21	35	5	7	28	6	1.19E-04	0.01043	13	20	15	31	17	23	0.4932031	1	18.375	10.6494467								
3	6E+07	3_64773200	0	22	5	15	11	10	0.842816	1	0	10	27	11	5	19	0.0096847	0.3824351	11.25	9.79431322	6E+07	6.5E+07	ID=ENSP00000001146;CYP26B1;NM_019885;	CYP26B1	6.5E+07	6.5E+07	0.97899	ID=promoter_33294;
3	6E+07	3_64773346	1465	832	1493	1682	1149	1588	1.24E-04	0.01084	655	623	602	347	639	475	0.00126734	0.0756817	962.38	505.29537	6E+07	6.5E+07	ID=ENSP00000001146;CYP26B1;NM_019885;	CYP26B1	6.5E+07	6.5E+07	0.97899	ID=promoter_33294;

3	6E+07	3_64776481	678	316	621	460	497	541	0.485306	1	161	174	241	34	168	138	0.5741259	1	335.63	230.236486					6.5E+07	6.5E+07	0.97899	ID=promoter_33294;
3	6E+07	3_648580	35	79	41	25	57	33	0.004384	0.2112	40	36	62	17	38	40	0.85429025	1	41.875	19.9028892								
3	6E+07	3_649052	108	56	92	55	82	74	0.260659	1	12	64	28	18	38	23	0.26660526	1	54.125	34.2279648								
3	6E+07	3_649240	394	215	125	188	305	157	7.10E-17	#######	31	27	54	23	29	39	0.31214516	1	132.13	129.784918								
3	7E+07	3_650654	25	46	22	23	36	23	0.069202	1	13	18	34	1	16	18	0.85288016	1	22.75	13.4350288								
3	7E+07	3_65293395	3	0	38	17	2	28	8.68E-05	0.00783	0	0	40	18	0	29	1.61E-07	3.22E-05	14.5	16.835337	7E+07	6.5E+07	ID=ENSP00000386881;DYSF;NM_001130987;	DYSF	6.5E+07	6.5E+07	0.18269	ID=promoter_33299;
3	7E+07	3_65380426	16	0	30	0	8	15	0.557319	1	0	0	9	0	0	5	0.06742467	1	6.875	11.076842	7E+07	6.5E+07	ID=ENSP00000386881;DYSF;NM_001130987;	DYSF				
3	7E+07	3_659436	6	5	53	144	6	99	1.23E-15	#######	29	3	15	2	16	9	0.48000706	1	32.125	48.4456618								
3	7E+07	3_65985361	23	2	20	14	13	17	0.957342	1	0	5	9	0	3	5	0.7175395	1	9.125	9.01486867					6.6E+07	6.6E+07	0.37612	ID=promoter_33327;
3	7E+07	3_660737	64	7	44	40	36	42	0.99972	1	74	22	30	17	48	24	0.04295976	1	37.25	23.0821514								
3	7E+07	3_661870	267	68	219	197	168	208	0.873036	1	39	56	32	16	48	24	0.04295976	1	111.75	99.0782519								
3	7E+07	3_666873	5	16	12	50	11	31	0.036004	1	108	33	31	39	71	35	0.00796123	0.3283441	36.75	32.4158251								
3	7E+07	3_66949581	87	69	82	85	78	84	0.862747	1	69	8	14	1	39	8	1.12E-04	0.0100767	51.875	37.3647561	7E+07	6.7E+07	ID=ENSP00000386456;AAK1;NM_014911;	AAK1				
3	7E+07	3_671593	88	15	71	52	52	62	0.99622	1	19	21	62	3	20	33	0.11912992	1	41.375	30.8773495								
3	7E+07	3_67252654	50	48	27	17	49	22	5.23E-04	0.0367	0	13	8	15	7	12	0.42229866	1	22.25	18.2032179	7E+07	6.7E+07	ID=ENSP00000301945;ANTXR1;NM_032208;	ANTXR1				
3	7E+07	3_67269427	38	39	62	52	39	57	0.569805	1	15	13	12	0	14	6	0.26891969	1	28.875	21.9768547	7E+07	6.7E+07	ID=ENSP00000301945;ANTXR1;NM_032208;	ANTXR1				
3	7E+07	3_677310	9	41	15	2	25	9	0.005635	0.25889	1	35	8	50	18	29	0.17554477	1	20.125	19.0520902								
3	7E+07	3_686763	30	34	31	13	32	22	0.148825	1	82	61	84	59	72	72	0.90181421	1	49.25	26.0863949								
3	7E+07	3_691239	50	62	35	14	56	25	1.65E-04	0.01374	38	12	19	28	25	24	0.99255599	1	32.25	17.6372171								
3	7E+07	3_692418	83	53	54	100	68	77	0.977841	1	110	40	87	99	75	93	0.17055736	1	78.25	25.9105605								
3	7E+07	3_69331596	1098	421	655	526	760	591	4.27E-13	#######	458	340	317	213	399	265	1.31E-04	0.0114541	503.5	275.595459					6.9E+07	6.9E+07	0.34533	ID=promoter_33364;
3	7E+07	3_697614	47	38	46	22	43	34	0.224092	1	17	21	45	12	19	29	0.23299331	1	31	14.4617723								
3	7E+07	3_698030	7	11	0	7	9	4	0.244237	1	21	0	9	14	11	12	0.92663959	1	8.625	6.98851098								
3	7E+07	3_701826	25	25	58	32	25	45	0.223918	1	41	5	22	37	23	30	0.4647018	1	30.625	15.5557752								
3	7E+07	3_704258	19	29	18	26	24	22	0.704268	1	7	2	54	7	5	31	3.00E-05	0.0032574	20.25	16.6454627								
3	7E+07	3_704528	286	224	175	202	255	189	8.82E-06	0.00107	95	138	138	82	117	110	0.99441092	1	167.5	68.4397545								
3	7E+07	3_708776	20	4	23	13	12	18	0.803442	1	57	4	43	4	31	24	0.80238053	1	21	19.6977156								
3	7E+07	3_710961	0	1	8	1	1	5	0.353258	1	2	13	18	0	8	9	0.92351511	1	5.375	6.88554386								
3	7E+07	3_721281	42	30	8	4	36	6	1.64E-06	0.00024	0	49	65	0	25	33	0.40760787	1	24.75	25.2685575								
3	7E+07	3_722348	455	239	381	302	347	342	0.073156	1	109	149	146	33	129	90	0.11577174	1	226.75	144.258449								
3	7E+07	3_72236243	447	185	423	462	316	443	0.052889	1	224	163	139	98	194	119	0.00169572	0.0953605	267.63	150.75613					7.2E+07	7.2E+07	0.45968	ID=promoter_33406;
3	7E+07	3_723006	7	17	11	6	12	9	0.592799	1	15	0	9	18	8	14	0.34554715	1	10.375	6.13974173								
3	7E+07	3_72631202	262	134	176	148	198	162	0.002901	0.15122	173	79	111	84	126	98	0.4266906	1	145.88	59.5109534					7.3E+07	7.3E+07	0.50472	ID=promoter_33410;
3	7E+07	3_72638156	5263	3697	6286	6206	4480	6246	1.11E-17	#######	5368	5152	10922	4224	5260	7573	1.81E-133	9.57E-130	5889.8	2215.94087					7.3E+07	7.3E+07	0.88702	ID=promoter_33413;
3	7E+07	3_72843093	2	57	13	22	30	18	0.072527	1	35	37	89	39	36	64	0.00635208	0.2749222	36.75	27.0911161	7E+07	7.3E+07	ID=ENSP00000263991;EHBP1;NM_015252;	EHBP1				
3	7E+07	3_728793	0	33	5	11	17	8	0.087954	1	1	0	11	9	1	10	0.01766414	0.6154554	8.75	10.8594922								
3	7E+07	3_731384	17	19	1	6	18	4	0.003432	0.17404	1	3	11	0	2	6	0.31385293	1	7.25	7.53562965								
3	7E+07	3_73573818	675	292	353	384	484	369	2.69E-09	#######	248	124	269	56	186	163	0.87159551	1	300.13	186.980089					7.4E+07	7.4E+07	0.50073	ID=promoter_33421;

3	7E+07	3_73678778	859	634	655	460	747	558	4.33E-15	#######	433	446	213	224	440	219	2.07E-13	9.38E-11	490.5	219.639315					7.4E+07	7.4E+07	0.48233	ID=promoter_33422;
3	7E+07	3_746870	0	1	2	3	1	3	0.700657	1	19	1	15	1	10	8	0.95278883	1	5.25	7.38241153								
3	7E+07	3_748807	17	0	12	1	9	7	0.713906	1	6	8	3	0	7	2	0.29886173	1	5.875	6.17454452								
3	7E+07	3_748813	6	1	27	14	4	21	0.010729	0.43446	19	5	8	0	12	4	0.18067673	1	10	9.35032467								
3	7E+07	3_74902424	31	43	19	32	37	26	0.128943	1	82	37	15	77	60	46	0.62428933	1	42	24.8710962					7.5E+07	7.5E+07	0.21723	ID=promoter_33435;
3	7E+07	3_749222	68	42	21	38	55	30	0.002546	0.13706	2	16	17	25	9	21	0.05613829	1	28.625	20.3254766								
3	8E+07	3_753254	8	36	11	13	22	12	0.091688	1	13	10	17	3	12	10	0.96947392	1	13.875	9.83343422								
3	8E+07	3_754418	3	40	5	17	22	11	0.058934	1	0	14	0	50	7	25	0.00310365	0.1565896	16.125	19.0445905								
3	8E+07	3_760265	0	27	10	4	14	7	0.165008	1	1	43	36	1	22	19	0.97472654	1	15.25	17.4499284								
3	8E+07	3_765538	28	58	24	38	43	31	0.113462	1	51	25	50	1	38	26	0.48792921	1	34.375	18.6772398								
3	8E+07	3_768563	17	6	14	18	12	16	0.945172	1	0	2	10	0	1	5	0.22510075	1	8.375	7.44383734								
3	8E+07	3_77422366	0	18	5	3	9	4	0.244237	1	0	5	10	29	3	20	9.56E-04	0.060345	8.75	10.0818082	8E+07	7.7E+07	ID=ENSP0000342381;VRK2;NM_006296;	VRK2				
3	8E+07	3_776682	2	90	3	11	46	7	1.99E-08	#######	8	27	46	85	18	66	1.66E-07	3.31E-05	34	36.0951125								
3	8E+07	3_792397	458	243	263	280	351	272	1.59E-06	0.00023	296	167	276	134	232	205	0.88226914	1	264.63	96.9593987								
3	8E+07	3_79733515	2594	1842	3426	3097	2218	3262	1.85E-15	#######	1778	1487	2259	758	1633	1509	0.99594956	1	2155.1	873.766304					8E+07	8E+07	0.13469	ID=promoter_33450;
3	8E+07	3_800898	20	28	4	0	24	2	1.35E-05	0.00155	17	1	15	12	9	14	0.47220951	1	12.125	9.87692115								
3	8E+07	3_801790	138	47	122	164	93	143	0.125227	1	501	156	142	229	329	186	3.63E-07	6.77E-05	187.38	136.329367								
3	8E+07	3_80704504	0	36	0	18	18	9	0.098614	1	11	8	24	3	10	14	0.60490119	1	12.5	12.7615494	8E+07	8.1E+07	ID=ENSP0000263373;SPTBN4;NM_020971;	SPTBN4				
3	8E+07	3_80704802	10	9	3	24	10	14	0.912507	1	0	10	5	0	5	3	0.8350419	1	7.625	7.79995421	8E+07	8.1E+07	ID=ENSP0000263373;SPTBN4;NM_020971;	SPTBN4				
3	8E+07	3_80741558	32	58	58	25	45	42	0.565697	1	43	52	71	2	48	37	0.69995349	1	42.625	22.1677855	8E+07	8.1E+07	ID=ENSP0000263373;SPTBN4;NM_020971;	SPTBN4				
3	8E+07	3_80744186	16	36	2	10	26	6	3.38E-04	0.02566	27	20	32	36	24	34	0.27690884	1	22.375	12.5349511	8E+07	8.1E+07	ID=ENSP0000263373;SPTBN4;NM_020971;	SPTBN4				
3	8E+07	3_808107	14	37	42	15	26	29	0.982786	1	1	11	13	13	6	13	0.20797135	1	18.25	13.8847295								
3	8E+07	3_826219	2	20	0	5	11	3	0.050503	1	0	12	5	30	6	18	0.03091566	0.954015	9.25	10.780273								
3	8E+07	3_830131	11	0	6	26	6	16	0.210908	1	80	13	65	42	47	54	0.55944892	1	30.375	29.3010848								
3	8E+07	3_830810	56	29	84	56	43	70	0.23605	1	39	9	18	44	24	31	0.47191283	1	41.875	23.9131613								
3	8E+07	3_842719	0	41	11	7	21	9	0.03105	0.99404	23	14	41	0	19	21	0.85675254	1	17.125	16.5221713								
3	8E+07	3_843078	321	173	349	228	247	289	0.999588	1	58	143	61	50	101	56	0.00771282	0.3222758	172.88	118.142091								
3	8E+07	3_844870	43	0	17	27	22	22	0.86913	1	0	1	49	35	1	42	6.46E-10	1.96E-07	21.5	19.9857092								
3	9E+07	3_85615463	6505	2858	7614	5477	4682	6546	4.73E-19	#######	3640	2676	2479	1871	3158	2175	1.32E-25	1.19E-22	4140	2117.73369					8.6E+07	8.6E+07	0.62461	ID=promoter_33479;
3	9E+07	3_862502	84	32	91	80	58	86	0.384512	1	85	23	44	21	54	33	0.16457707	1	57.5	30.3362113								
3	9E+07	3_863261	181	65	54	74	123	64	4.18E-07	#######	0	13	15	18	7	17	0.08351885	1	52.5	58.6100918								
3	9E+07	3_872715	0	20	10	0	10	5	0.276106	1	42	8	11	2	25	7	0.01215165	0.4601029	11.625	13.9993622								
3	9E+07	3_87354255	0	0	0	0	0	0	1	1	0	0	0	47	0	24	2.39E-06	0.0003623	5.875	16.6170094	9E+07	8.7E+07	ID=ENSP0000238738;RHOQ;NM_012249;	RHOQ				
3	9E+07	3_87357201	1	22	46	35	12	41	0.002936	0.15296	29	27	30	43	28	37	0.36354865	1	29.125	13.9328748	9E+07	8.7E+07	ID=ENSP0000238738;RHOQ;NM_012249;	RHOQ				
3	9E+07	3_87361389	12	14	5	0	13	3	0.018372	0.65917	25	0	11	1	13	6	0.35360803	1	8.5	8.73416935	9E+07	8.7E+07	ID=ENSP0000238738;RHOQ;NM_012249;	RHOQ	8.7E+07	8.7E+07	0.13387	ID=promoter_33504;
3	9E+07	3_874777	40	71	46	74	56	60	0.888	1	0	9	26	1	5	14	0.08295177	1	33.375	29.4712329								

3	9E+07	3_87553531	152	54	161	114	103	138	0.593092	1	11	33	19	0	22	10	0.16182597	1	68	64.9351325	9E+07	8.8E+07	ID=ENSP00000263734;EPAS1;NM_001430;	EPAS1				
3	9E+07	3_87768219	18	14	4	5	16	5	0.021336	0.74103	80	40	40	2	60	21	3.42E-04	0.0258917	25.375	26.763181	9E+07	8.8E+07	ID=ENSP00000305355-D8;PRKCB;NM_002738;	PRKCB				
3	9E+07	3_87920564	344	231	420	373	288	397	0.113026	1	320	160	164	94	240	129	2.83E-06	0.0004196	263.25	117.445124	9E+07	8.8E+07	ID=ENSP00000305355-D8;PRKCB;NM_002738;	PRKCB	8.8E+07	8.8E+07	0.11687	ID=promoter_33516;
3	9E+07	3_882586	0	1	7	2	1	5	0.353258	1	17	5	1	11	11	6	0.57179039	1	5.5	5.95219047								
3	9E+07	3_88529391	59	85	15	21	72	18	8.25E-10	#######	18	0	33	3	9	18	0.15524483	1	29.25	29.1535345	9E+07	8.9E+07	ID=ENSP00000263736;SRBD1;NM_018079;	SRBD1				
3	9E+07	3_886682	11	30	11	3	21	7	0.0088	0.36783	29	28	31	65	29	48	0.0440188	1	26	19.1012341								
3	9E+07	3_886969	3	11	7	15	7	11	0.831726	1	68	11	17	41	40	29	0.60017008	1	21.625	21.967102								
3	9E+07	3_888948	13	26	4	11	20	8	0.026297	0.87605	0	19	4	1	10	3	0.19568363	1	9.75	9.25434261								
3	9E+07	3_888991	245	101	194	151	173	173	0.331882	1	10	40	28	14	25	21	0.94599092	1	97.875	90.0054562								
3	9E+07	3_88940004	432	352	777	606	392	692	6.19E-10	#######	813	502	649	387	658	518	0.0206218	0.6985624	564.75	174.804176					8.9E+07	8.9E+07	0.18967	ID=promoter_33538;
3	9E+07	3_889987	36	108	20	32	72	26	3.51E-07	#######	72	63	33	49	68	41	0.09040999	1	51.625	28.550394								
3	9E+07	3_891095	141	107	77	108	124	93	0.004502	0.21519	58	84	119	35	71	77	0.63413428	1	91.125	34.4318685								
3	9E+07	3_90157624	9	12	6	12	11	9	0.722819	1	0	0	17	1	0	9	0.00779604	0.3222758	7.125	6.42400632	9E+07	9E+07	ID=ENSP00000260645;ABCG5;NM_022436;	ABCG5				
3	9E+07	3_90625434	8	39	12	25	24	19	0.435991	1	41	6	13	21	24	17	0.69596948	1	20.625	13.5112387					9.1E+07	9.1E+07	0.08947	ID=promoter_33563;
3	9E+07	3_90703416	93	22	104	87	58	96	0.114719	1	13	27	34	13	20	24	0.69398062	1	49.125	38.6095381					9.1E+07	9.1E+07	0.33646	ID=promoter_33565;
3	9E+07	3_908175	9	3	10	7	6	9	0.896349	1	3	7	1	4	5	3	0.8350419	1	5.5	3.2071349								
3	9E+07	3_908252	3	33	12	8	18	10	0.152558	1	54	23	53	1	39	27	0.50415049	1	23.375	21.3470473								
3	9E+07	3_908259	606	410	809	665	508	737	0.001091	0.06822	18	55	18	2	37	10	0.00114024	0.0700243	322.88	338.427641								
3	9E+07	3_914244	12	24	9	9	18	9	0.098614	1	1	17	6	1	9	4	0.4571786	1	9.875	7.82737869								
3	9E+07	3_914495	22	32	8	29	27	19	0.225928	1	3	20	12	4	12	8	0.76853133	1	16.25	11.1451463								
3	9E+07	3_91462286	290	109	367	280	200	324	0.001499	0.08861	179	114	67	17	147	42	9.66E-12	3.57E-09	177.88	122.922086					9.1E+07	9.1E+07	0.29695	ID=promoter_33583;
3	9E+07	3_91600134	15	114	20	10	65	15	2.09E-09	#######	113	28	30	8	71	19	1.97E-06	0.0003071	42.25	44.6534273	9E+07	9.2E+07	ID=ENSP00000320663;EML4;NM_019063;	EML4				
3	9E+07	3_916927	352	239	389	295	296	342	0.982134	1	203	156	112	81	180	97	7.45E-05	0.0071391	228.38	111.342377								
3	9E+07	3_922336	11	22	6	11	17	9	0.140533	1	0	8	30	13	4	22	9.67E-04	0.0606639	12.625	9.41029072								
3	9E+07	3_935304	5	9	8	6	7	7	0.956352	1	0	4	20	41	2	31	9.34E-07	0.0001565	11.625	13.2118724								
3	9E+07	3_935753	24	1	14	7	13	11	0.725732	1	0	4	9	0	2	5	0.46647814	1	7.375	8.31414457								
3	9E+07	3_936811	7	41	22	20	24	21	0.616071	1	31	17	38	65	24	52	0.00187074	0.1037321	30.125	18.0193349								
3	9E+07	3_94936085	505	313	330	315	409	323	6.12E-07	#######	300	245	141	140	273	141	7.80E-08	1.66E-05	286.13	116.915644	9E+07	9.5E+07	ID=ENSP00000333393;ATL2;NM_022374;	ATL2	9.5E+07	9.5E+07	0.56636	ID=promoter_33606;
3	9E+07	3_94962213	9	56	12	15	33	14	0.00394	0.1935	33	32	64	54	33	59	0.00881797	0.3552637	34.375	21.5733798	9E+07	9.5E+07	ID=ENSP00000333393;ATL2;NM_022374;	ATL2				
3	1E+08	3_950480	32	7	63	60	20	62	4.43E-04	0.03199	65	48	48	13	57	31	0.05372762	1	42	22.4626674								
3	1E+08	3_954265	30	18	32	36	24	34	0.777678	1	11	0	8	5	6	7	0.91781435	1	17.5	13.6486525								

3	1E+08	3_96284253	9	72	10	15	41	13	6.00E-05	0.00569	64	25	51	27	45	39	0.95370772	1	34.125	24.8563731	1E+08	9.6E+07	ID=ENSP000002330 99;HEATR5B;NM_019024;	HEATR5B				
3	1E+08	3_974435	9	27	6	9	18	8	0.059652	1	55	39	2	18	47	10	2.34E-05	0.002637	20.625	18.5082028								
3	1E+08	3_981863	12	51	14	4	32	9	2.11E-04	0.01699	20	1	7	17	11	12	0.92663959	1	15.75	15.6364958								
3	1E+08	3_985219	58	45	23	21	52	22	1.57E-04	0.01313	44	33	37	32	39	35	0.99032895	1	36.625	12.2233676								
3	1E+08	3_997337	19	87	23	28	53	26	8.61E-04	0.05619	15	35	71	38	25	55	0.00108314	0.0670948	39.5	25.9009101								
3	1E+08	3_999342	12	37	13	23	25	18	0.280499	1	10	4	26	0	7	13	0.3188207	1	15.625	12.2467197								
3	1E+08	3_100210412	74	72	112	76	73	94	0.836098	1	74	46	30	3	60	17	2.90E-05	0.0031754	60.875	33.4554608								
3	1E+08	3_100281894	58	37	97	73	48	85	0.071386	1	135	79	59	39	107	49	1.65E-04	0.0139983	72.125	32.3438557								
3	1E+08	3_100398265	223	124	459	425	174	442	9.77E-18	#######	336	401	439	182	369	311	0.46481099	1	323.63	129.883396								
3	1E+08	3_100410678	5	5	0	7	5	4	0.848147	1	11	1	0	27	6	14	0.1466131	1	7	8.92828571								
3	1E+08	3_100461341	35	22	67	62	29	65	0.012861	0.49778	0	6	0	1	3	1	0.65196691	1	24.125	27.833369								
3	1E+08	3_100596370	0	32	0	0	16	0	8.41E-05	0.00761	0	12	18	0	6	9	0.65381141	1	7.75	12.0208153								
3	1E+08	3_100916622	280	117	280	232	199	256	0.620172	1	93	79	70	41	86	56	0.11788216	1	149	98.6885433					1E+08	1E+08	0.62743	ID=promoter_33636;
3	1E+08	3_101444965	32	21	3	6	27	5	7.68E-05	0.00707	0	0	0	0	0	0	1	1	7.75	12.127301								
3	1E+08	3_101657380	159	131	93	56	145	75	2.47E-08	#######	81	78	107	14	80	61	0.51539745	1	89.875	44.5563447	1E+08	1E+08	ID=ENSP000003854 35;GALNT14;;	GALNT14				
3	1E+08	3_101659436	92	65	161	111	79	136	0.024147	0.81985	35	66	71	28	51	50	0.96124075	1	78.625	42.8983433	1E+08	1E+08	ID=ENSP000003854 35;GALNT14;;	GALNT14				
3	1E+08	3_101704667	9	30	9	19	20	14	0.325699	1	37	18	20	27	28	24	0.96104627	1	21.125	9.81889578								
3	1E+08	3_102083455	1	49	1	10	25	6	5.64E-04	0.03913	38	8	12	1	23	7	0.02502936	0.8114813	15	18.3458832								
3	1E+08	3_102226124	1	19	6	0	10	3	0.082185	1	52	33	65	2	43	34	0.78602279	1	22.25	25.2402739								
3	1E+08	3_102363123	20	11	20	7	16	14	0.724026	1	0	0	10	16	0	13	9.01E-04	0.0577931	10.5	7.96420564								
3	1E+08	3_102667692	0	9	6	0	5	3	0.645778	1	19	0	7	0	10	4	0.34296513	1	5.125	6.7281392								
3	1E+08	3_103092566	62	224	22	42	143	32	3.24E-20	#######	61	52	29	63	57	46	0.78263009	1	69.375	64.3648917	1E+08	1E+08	ID=ENSP000003737 00;ALK;NM_004304;	ALK				
3	1E+08	3_103122145	16	51	26	20	34	23	0.11872	1	1	52	15	63	27	39	0.20294831	1	30.5	22.0064925	1E+08	1E+08	ID=ENSP000003737 00;ALK;NM_004304;	ALK				
3	1E+08	3_103136746	148	92	133	118	120	126	0.685761	1	0	6	7	0	3	4	0.89184014	1	63	65.8070773	1E+08	1E+08	ID=ENSP000003737 00;ALK;NM_004304;	ALK				
3	1E+08	3_103182377	43	58	98	64	51	81	0.234905	1	0	25	32	0	13	16	0.74830213	1	40	33.2995066	1E+08	1E+08	ID=ENSP000003737 00;ALK;NM_004304;	ALK				
3	1E+08	3_103193751	38	62	11	18	50	15	3.75E-06	0.0005	29	26	31	8	28	20	0.67114862	1	27.875	17.1500729								
3	1E+08	3_103220247	35	31	37	15	33	26	0.311396	1	20	49	42	21	35	32	0.99839619	1	31.25	11.792855								
3	1E+08	3_103324560	275	128	205	267	202	236	0.999146	1	130	105	146	50	118	98	0.72324624	1	163.25	79.1558138					1E+08	1E+08	0.29516	ID=promoter_33667;
3	1E+08	3_103353142	51	16	35	16	34	26	0.254409	1	1	10	11	3	6	7	0.91781435	1	17.875	16.9574046								
3	1E+08	3_103362298	13	20	19	40	17	30	0.39938	1	33	14	37	10	24	24	0.96613769	1	23.25	11.7077508								

3	1E+08	3_103510865	4	14	8	15	9	12	0.95859	1	0	42	26	39	21	33	0.16057985	1	18.5	15.6934927								
3	1E+08	3_103684120	19	35	63	81	27	72	9.09E-04	0.05849	15	11	6	0	13	3	0.06317581	1	28.75	28.9963052								
3	1E+08	3_103724060	136	38	100	64	87	82	0.362881	1	126	17	38	1	72	20	2.73E-06	0.0004069	65	50.4918664								
3	1E+08	3_103724250	180	72	165	112	126	139	0.883073	1	185	51	38	0	118	19	1.07E-14	5.34E-12	100.38	70.7327718								
3	1E+08	3_103802559	0	22	8	1	11	5	0.189421	1	0	16	0	0	8	0	0.02452223	0.7977568	5.875	8.69215574								
3	1E+08	3_103882769	3	64	13	28	34	21	0.063114	1	93	28	30	32	61	31	0.02177598	0.7340029	36.375	28.858955								
3	1E+08	3_103914909	47	12	41	25	30	33	0.968109	1	0	15	1	2	8	2	0.20550608	1	17.875	18.2556254								
3	1E+08	3_103963528	5	22	6	9	14	8	0.253058	1	0	21	12	2	11	7	0.73613127	1	9.625	8.22778394								
3	1E+08	3_104257337	60	107	13	43	84	28	6.00E-09	#######	102	97	53	11	100	32	2.89E-07	5.48E-05	60.75	38.3321946					1E+08	1E+08	0.11834	ID=promoter_33702;
3	1E+08	3_104304896	3	23	20	17	13	19	0.82904	1	2	2	7	1	2	4	0.6610374	1	9.375	9.11729126								
3	1E+08	3_104366211	0	12	11	6	6	9	0.896349	1	30	5	14	15	18	15	0.95456211	1	11.625	8.991067								
3	1E+08	3_104428578	369	110	310	256	240	283	0.99819	1	111	107	76	79	109	78	0.21608221	1	177.25	116.093251					1E+08	1E+08	0.541	ID=promoter_33711;
3	1E+08	3_104442696	3463	2058	3546	3750	2761	3648	1.43E-05	0.00164	1851	1630	1825	1067	1741	1446	0.0084338	0.343665	2398.8	1027.10074					1E+08	1E+08	0.49641	ID=promoter_33713;
3	1E+08	3_104502384	172	49	203	134	111	169	0.10105	1	67	53	25	50	60	38	0.18101617	1	94.125	66.221355					1E+08	1E+08	0.59302	ID=promoter_33722;
3	1E+08	3_104517231	882	536	733	737	709	735	0.063539	1	534	502	299	189	518	244	7.06E-18	4.38E-15	551.5	231.375391					1E+08	1E+08	0.34267	ID=promoter_33723;
3	1E+08	3_104564426	43	35	44	22	39	33	0.382234	1	7	4	25	65	6	45	7.19E-08	1.54E-05	30.625	20.3746166								
3	1E+08	3_104733387	4	15	6	17	10	12	0.998584	1	0	10	6	25	5	16	0.03625394	1	10.375	8.15803723								
3	1E+08	3_104735490	342	329	316	287	336	302	0.003451	0.17438	190	181	153	139	186	146	0.32147682	1	242.13	84.5364461					1E+08	1E+08	0.42885	ID=promoter_33735;
3	1E+08	3_104829346	32	4	75	67	18	71	6.66E-06	0.00083	1	1	2	0	1	1	0.99856566	1	22.75	31.666792								
3	1E+08	3_104986371	11	18	12	9	15	11	0.4902	1	20	14	43	5	17	24	0.40932813	1	16.5	11.7229933								
3	1E+08	3_104998535	14	32	2	10	23	6	0.001551	0.09145	1	19	51	36	10	44	5.86E-06	0.000796	20.625	17.646833								
3	1E+08	3_105072267	18	10	9	13	14	11	0.605836	1	0	0	9	16	0	13	9.01E-04	0.0577931	9.375	6.63190341								
3	1E+08	3_105294873	35	85	33	53	60	43	0.044963	1	44	89	39	23	67	31	0.00485773	0.2226648	50.125	24.3629549								
3	1E+08	3_105468638	184	71	134	140	128	137	0.757556	1	28	84	41	10	56	26	0.01206885	0.4578174	86.5	61.1975723					1.1E+08	1.1E+08	0.63289	ID=promoter_33770;
3	1E+08	3_105589429	79	43	59	87	61	73	0.993336	1	26	16	13	0	21	7	0.05007015	1	40.375	32.0710706								
3	1E+08	3_105959664	5	11	8	14	8	11	0.943069	1	32	9	5	2	21	4	0.00573189	0.2548307	10.75	9.37702359								
3	1E+08	3_106068256	0	0	1	14	0	8	0.033035	1	15	19	18	0	17	9	0.38755874	1	8.375	8.83074985								
3	1E+08	3_106098398	1	17	1	0	9	1	0.020382	0.71452	25	1	0	1	13	1	0.00904697	0.3617342	5.75	9.66215001					1.1E+08	1.1E+08	0.23322	ID=promoter_33791;
3	1E+08	3_106099975	68	32	63	67	50	65	0.861076	1	24	13	18	8	19	13	0.6985193	1	36.625	25.3768032					1.1E+08	1.1E+08	0.62333	ID=promoter_33792;
3	1E+08	3_106130254	14	16	32	15	15	24	0.638589	1	93	20	37	37	57	37	0.23694992	1	33	26.1205994	1E+08	1.1E+08	ID=ENSP00000260600;ADCY3;NM_004036;	ADCY3				
3	1E+08	3_106304877	2	33	10	4	18	7	0.033499	1	12	24	6	0	18	3	0.00813808	0.3342859	11.375	11.5255926	1E+08	1.1E+08	ID=ENSP00000385216;NCOA1;;	NCOA1				

3	1E+08	3_106466806	5	4	12	13	5	13	0.299488	1	23	18	18	10	21	14	0.63082335	1	12.875	6.59951297					1.1E+08	1.1E+08	0.43242	ID=promoter_33807;
3	1E+08	3_107074449	213	104	237	127	159	182	0.975007	1	67	32	82	0	50	41	0.84384887	1	107.75	82.6693068					1.1E+08	1.1E+08	0.43845	ID=promoter_33827;
3	1E+08	3_107104107	47	63	24	27	55	26	4.07E-04	0.02994	107	44	71	13	76	42	0.02469969	0.8031639	49.5	30.4161611					1.1E+08	1.1E+08	0.14836	ID=promoter_33829;
3	1E+08	3_107173222	51	33	61	50	42	56	0.820895	1	88	106	163	44	97	104	0.58759656	1	74.5	43.0846343								
3	1E+08	3_107198101	14	14	119	83	14	101	3.05E-12	#######	2	45	40	7	24	24	0.96613769	1	40.5	41.4004831								
3	1E+08	3_107278447	38	39	35	8	39	22	0.019446	0.69119	32	24	12	0	28	6	0.00180324	0.1003399	23.5	15.0237907								
3	1E+08	3_107293517	1	7	9	7	4	8	0.677894	1	0	13	2	1	7	2	0.29886173	1	5	4.69041576								
3	1E+08	3_108095922	12	29	7	4	21	6	0.004166	0.20266	16	1	29	10	9	20	0.07989336	1	13.5	10.6234243								
3	1E+08	3_108380147	11	34	15	10	23	13	0.098913	1	54	67	67	63	61	65	0.73663297	1	40.125	25.5926189								
3	1E+08	3_108758259	14	13	0	2	14	1	0.001161	0.07164	1	0	22	0	1	11	0.01045793	0.4067594	6.5	8.5857024								
3	1E+08	3_109102891	34	11	11	12	23	12	0.065071	1	27	27	10	2	27	6	0.00268813	0.1381379	16.75	11.0808715								
3	1E+08	3_109256752	32	79	25	9	56	17	9.75E-07	0.00015	32	26	21	26	29	24	0.91882689	1	31.25	20.6172605								
3	1E+08	3_109299839	15	14	0	5	15	3	0.006447	0.2884	0	2	15	1	1	8	0.04988758	1	6.5	6.94879229								
3	1E+08	3_109463886	247	241	203	174	244	189	9.13E-05	0.00821	161	106	100	100	134	100	0.25859008	1	166.5	60.8910033					1.1E+08	1.1E+08	0.5853	ID=promoter_33858;
3	1E+08	3_109743516	223	146	278	149	185	214	0.990453	1	42	65	97	61	54	79	0.03349746	1	132.63	83.6095304					1.1E+08	1.1E+08	0.35776	ID=promoter_33866;
3	1E+08	3_109893562	0	25	6	6	13	6	0.146758	1	0	10	1	1	5	1	0.30486099	1	6.125	8.44202921								
3	1E+08	3_109896608	30	15	40	10	23	25	0.965853	1	12	26	41	0	19	21	0.85675254	1	21.75	14.8396188					1.1E+08	1.1E+08	0.16311	ID=promoter_33873;
3	1E+08	3_109897809	0	2	0	3	1	2	0.907382	1	109	138	114	43	124	79	0.03224386	0.9875313	51.125	59.6092934					1.1E+08	1.1E+08	0.11806	ID=promoter_33874;
3	1E+08	3_109931479	25	65	28	27	45	28	0.027695	0.91219	23	24	34	7	24	21	0.98147478	1	29.125	16.4180867								
3	1E+08	3_110053093	228	151	177	113	190	145	4.46E-04	0.03219	0	2	10	0	1	5	0.22510075	1	85.125	93.3632499								
3	1E+08	3_110362200	8	9	22	1	9	12	0.95859	1	13	5	30	15	9	23	0.02676526	0.8546242	12.875	9.43303466								
3	1E+08	3_110518837	3	16	10	7	10	9	0.84607	1	25	11	8	9	18	9	0.30718372	1	11.125	6.70687281								
3	1E+08	3_111564107	185	145	136	174	165	155	0.138297	1	112	46	56	9	79	33	4.11E-04	0.0300788	107.88	64.1458995								
3	1E+08	3_111790559	4	11	9	3	8	6	0.705675	1	19	35	14	2	27	8	0.01145524	0.4380166	12.125	10.9340556								
3	1E+08	3_112145657	88	70	97	82	79	90	0.982267	1	50	31	17	1	41	9	1.14E-04	0.0101988	54.5	35.3876492								
3	1E+08	3_112420508	3	4	10	32	4	21	0.010729	0.43446	3	10	47	0	7	24	0.00482107	0.2211976	13.625	16.8263187								
3	1E+08	3_112448965	0	9	0	1	5	1	0.186119	1	30	4	0	0	17	0	3.78E-04	0.0279647	5.5	10.3923048								
3	1E+08	3_112476899	101	100	89	97	101	93	0.24045	1	51	39	43	58	45	51	0.6162221	1	72.25	27.0066129								
3	1E+08	3_112564675	1	0	2	4	1	3	0.700657	1	4	0	20	24	2	22	1.09E-04	0.0098689	6.875	9.52346726								
3	1E+08	3_112642428	21	19	17	24	20	21	0.939065	1	20	11	16	0	16	8	0.35023049	1	16	7.52139805								
3	1E+08	3_112658502	45	56	89	41	51	65	0.906125	1	4	32	9	23	18	16	0.9925456	1	37.375	27.3544851								
3	1E+08	3_112786501	25	17	33	16	21	25	0.998746	1	16	26	11	9	21	10	0.21021716	1	19.125	8.16678814								
3	1E+08	3_112795527	209	78	224	193	144	209	0.143521	1	40	90	84	71	65	78	0.3050555	1	123.63	72.4291526	1E+08	1.1E+08	ID=ENSP00003793 56;MYCN;NM_005378;	MYCN	1.1E+08	1.1E+08	0.49924	ID=promoter_33922;

3	1E+08	3_112832698	12	13	11	27	13	19	0.82904	1	12	22	27	32	17	30	0.09758228	1	19.5	8.46842876								
3	1E+08	3_112848646	58	1	59	50	30	55	0.139689	1	0	1	1	0	1	1	0.99856566	1	21.25	28.6244152								
3	1E+08	3_112966056	122	102	76	14	112	45	1.60E-09	#######	1	76	19	0	39	10	5.37E-04	0.0374204	51.25	48.3816671								
3	1E+08	3_113353554	180	717	276	231	449	254	2.65E-20	#######	741	583	771	403	662	587	0.73497979	1	487.75	244.553675								
3	1E+08	3_113886253	5	24	26	21	15	24	0.638589	1	0	0	23	32	0	28	2.76E-07	5.26E-05	16.375	12.6822429								
3	1E+08	3_113991275	7	0	6	7	4	7	0.813458	1	13	0	29	0	7	15	0.16935974	1	7.75	9.73579551								
3	1E+08	3_114433675	62	1	6	6	32	6	1.43E-05	0.00164	56	12	17	0	34	9	0.00172782	0.09686	20	24.7444077								
3	1E+08	3_114799670	38	27	32	11	33	22	0.114927	1	1	2	5	8	2	7	0.20455692	1	15.5	14.5896245								
3	1E+08	3_114805086	20	44	36	30	32	33	0.87385	1	42	28	35	17	35	26	0.69250145	1	31.5	9.68061391					1.1E+08	1.1E+08	0.11373	ID=promoter_33952;
3	1E+08	3_114842242	38	34	4	27	36	16	0.003558	0.17915	60	41	14	2	51	8	7.49E-07	0.000129	27.5	19.9141012								
3	1E+08	3_115051160	4	36	2	2	20	2	1.33E-04	0.01141	0	18	5	0	9	3	0.27712561	1	8.375	12.5804554					1.2E+08	1.2E+08	0.14273	ID=promoter_33961;
3	1E+08	3_115056201	324	155	240	271	240	256	0.571647	1	168	97	79	50	133	65	9.54E-05	0.0088336	173	97.8219958					1.2E+08	1.2E+08	0.71371	ID=promoter_33963;
3	1E+08	3_115124776	2	5	14	1	4	8	0.677894	1	3	1	13	3	2	8	0.1303879	1	5.25	5.25764749								
3	1E+08	3_115148357	15	14	4	5	15	5	0.034023	1	24	31	27	5	28	16	0.29661245	1	15.625	10.6896145								
3	1E+08	3_115156404	15	46	6	10	31	8	1.52E-04	0.01277	24	8	58	41	16	50	4.08E-05	0.0042741	26	19.8350339								
3	1E+08	3_115229462	151	44	69	53	98	61	4.08E-04	0.02998	30	29	22	1	30	12	0.0403413	1	49.875	45.7210096								
3	1E+08	3_115541726	3	12	10	0	8	5	0.532535	1	0	10	25	2	5	14	0.08295177	1	7.75	8.46421037					1.2E+08	1.2E+08	0.14071	ID=promoter_33969;
3	1E+08	3_115712820	11	0	19	23	6	21	0.046061	1	7	0	8	2	4	5	0.90483023	1	8.75	8.58154165								
3	1E+08	3_115743051	15	17	16	6	16	11	0.385778	1	22	11	3	0	17	2	0.0046333	0.2138882	11.25	7.62982868								
3	1E+08	3_115789027	27	37	18	59	32	39	0.987189	1	42	23	52	4	33	28	0.94230752	1	32.75	18.2658933					1.2E+08	1.2E+08	0.16809	ID=promoter_33982;
3	1E+08	3_115791472	1105	901	1001	1044	1003	1023	0.007043	0.30636	348	468	256	270	408	263	2.32E-05	0.0026249	674.13	371.823859					1.2E+08	1.2E+08	0.31419	ID=promoter_33983;
3	1E+08	3_115798138	0	1	6	30	1	18	0.001742	0.10079	1	8	0	1	5	1	0.30486099	1	5.875	10.1901563								
3	1E+08	3_115835831	9	29	31	31	19	31	0.523429	1	71	14	39	5	43	22	0.0708179	1	28.625	20.9484913								
3	1E+08	3_115905429	130	175	164	106	153	135	0.053611	1	23	33	45	156	28	101	5.95E-11	2.04E-08	104	62.2299882								
3	1E+08	3_115991525	25	20	15	21	23	18	0.432563	1	20	11	25	14	16	20	0.67029873	1	18.875	5.11126208					1.2E+08	1.2E+08	0.17818	ID=promoter_34018;
3	1E+08	3_116020363	3205	760	2705	1544	1983	2125	0.015132	0.56724	294	264	346	235	279	291	0.3713762	1	1169.1	1192.04703					1.2E+08	1.2E+08	0.13947	ID=promoter_34025;
3	1E+08	3_116088390	251	107	220	231	179	226	0.76274	1	65	60	35	61	63	48	0.59100593	1	128.75	89.7373151					1.2E+08	1.2E+08	0.49295	ID=promoter_34034;
3	1E+08	3_116119493	7449	6412	9891	8558	6931	9225	1.80E-14	#######	5741	4669	8888	2579	5205	5734	2.08E-18	1.33E-15	6773.4	2421.63303					1.2E+08	1.2E+08	0.40601	ID=promoter_34038;
3	1E+08	3_116272769	38	174	61	25	106	43	6.04E-09	#######	62	51	127	94	57	111	1.92E-05	0.0022361	79	50.0685245	1E+08	1.2E+08	ID=ENSP0000382039;ROCK2;NM_004850;	ROCK2				
3	1E+08	3_116315640	1	21	0	11	11	6	0.3028	1	1	0	11	9	1	10	0.01766414	0.6154554	6.75	7.57344986								
3	1E+08	3_116391074	1	23	50	41	12	46	5.58E-04	0.03879	1	1	7	58	1	33	8.19E-08	1.74E-05	22.75	23.8612058								
3	1E+08	3_116440698	29	7	11	18	18	15	0.617094	1	21	18	3	0	20	2	0.00122165	0.0738454	13.375	9.84069539								

3	1E+08	3_116578510	125	44	57	60	85	59	0.007418	0.32037	55	44	52	5	50	29	0.12800836	1	55.25	33.1220686					1.2E+08	1.2E+08	0.60237	ID=promoter_34063;
3	1E+08	3_116597493	0	26	3	0	13	2	0.006819	0.29841	44	2	10	16	23	13	0.35430953	1	12.625	15.6107792	1E+08	1.2E+08	ID=ENSP0000385385;PDIA6;;	PDIA6	1.2E+08	1.2E+08	0.32521	ID=promoter_34069;
3	1E+08	3_116602636	19	48	28	27	34	28	0.378922	1	18	14	16	3	16	10	0.61601721	1	21.625	13.2118724	1E+08	1.2E+08	ID=ENSP0000272238;ATP6V1C2;NM_001039362;	ATP6V1C2	1.2E+08	1.2E+08	0.59454	ID=promoter_34070;
3	1E+08	3_116655408	108	206	130	95	157	113	3.38E-04	0.02566	77	45	91	18	61	55	0.98889628	1	96.25	56.6587529								
3	1E+08	3_116660293	57	19	43	95	38	69	0.092807	1	19	20	54	2	20	28	0.36590545	1	38.625	29.8325685								
3	1E+08	3_116687495	46	54	118	108	50	113	4.41E-04	0.03187	16	80	62	11	48	37	0.69995349	1	61.875	38.9374498					1.2E+08	1.2E+08	0.25645	ID=promoter_34077;
3	1E+08	3_116711437	2	10	17	4	6	11	0.674578	1	5	1	10	0	3	5	0.7175395	1	6.125	5.79254447	1E+08	1.2E+08	ID=ENSP0000371101;NOL10;;	NOL10				
3	1E+08	3_116730003	5	1	9	2	3	6	0.747087	1	3	5	4	11	4	8	0.43697044	1	5	3.42261387	1E+08	1.2E+08	ID=ENSP0000371101;NOL10;;	NOL10	1.2E+08	1.2E+08	0.32423	ID=promoter_34084;
3	1E+08	3_116741666	15	0	10	13	8	12	0.864244	1	1	16	27	33	9	30	0.00152443	0.0882297	14.375	11.4385002	1E+08	1.2E+08	ID=ENSP0000371101;NOL10;;	NOL10	1.2E+08	1.2E+08	0.17248	ID=promoter_34086;
3	1E+08	3_116764822	0	10	0	1	5	1	0.186119	1	25	15	8	0	20	4	0.00863789	0.3495868	7.375	9.10160896	1E+08	1.2E+08	ID=ENSP0000371101;NOL10;;	NOL10				
3	1E+08	3_116780756	294	200	202	210	247	206	0.001367	0.08174	84	57	115	23	71	69	0.96179373	1	148.13	92.4823342	1E+08	1.2E+08	ID=ENSP0000371101;NOL10;;	NOL10	1.2E+08	1.2E+08	0.34475	ID=promoter_34092;
3	1E+08	3_116822696	13	7	7	8	10	8	0.719353	1	0	19	14	0	10	7	0.84912739	1	8.5	6.65475126								
3	1E+08	3_116895990	197	164	205	287	181	246	0.321922	1	156	178	358	149	167	254	3.36E-06	0.000488	211.75	73.4647632								
3	1E+08	3_116899304	281	111	143	160	196	152	5.91E-04	0.04077	116	62	64	10	89	37	1.41E-04	0.012246	118.38	81.7014382								
3	1E+08	3_116915930	99	43	74	59	71	67	0.441021	1	41	11	35	34	26	35	0.3499771	1	49.5	27.223939					1.2E+08	1.2E+08	0.12682	ID=promoter_34104;
3	1E+08	3_116918613	705	397	764	721	551	743	0.045934	1	381	321	342	132	351	237	7.72E-04	0.0509844	470.38	230.136567								
3	1E+08	3_116932143	225	133	170	106	179	138	9.46E-04	0.06025	43	55	17	53	49	35	0.49765999	1	100.25	71.7351279					1.2E+08	1.2E+08	0.20818	ID=promoter_34105;
3	1E+08	3_116937165	58	18	24	28	38	26	0.100519	1	0	30	38	3	15	21	0.47046428	1	24.875	18.7116274								
3	1E+08	3_116943060	6	20	3	17	13	10	0.600182	1	0	0	2	0	0	1	0.58312783	1	6	8.01783726								
3	1E+08	3_116965187	209	282	116	158	246	137	7.92E-12	#######	230	134	171	65	182	118	0.00980063	0.3860309	170.63	68.7062848								
3	1E+08	3_116974977	8	23	13	9	16	11	0.385778	1	1	2	6	56	2	31	9.34E-07	0.0001565	14.75	18.0455772	1E+08	1.2E+08	ID=ENSP0000379542;TAF1B;;	TAF1B				
3	1E+08	3_116979796	10	105	22	69	58	46	0.136768	1	43	5	56	12	24	34	0.27690884	1	40.25	34.9765227	1E+08	1.2E+08	ID=ENSP0000379542;TAF1B;;	TAF1B	1.2E+08	1.2E+08	0.32266	ID=promoter_34110;
3	1E+08	3_117074999	0	0	5	0	0	3	0.278365	1	31	0	4	26	16	15	0.99951315	1	8.25	12.7251158								
3	1E+08	3_117135135	5	57	10	32	31	21	0.144504	1	33	22	40	27	28	34	0.56965937	1	28.25	16.5421539								
3	1E+08	3_117214239	18	7	11	18	13	15	0.999061	1	17	25	29	19	21	24	0.78178162	1	18	6.98978847								
3	1E+08	3_117230395	42	171	34	32	107	33	4.83E-12	#######	42	67	55	50	55	53	0.97997501	1	61.625	45.6412642					1.2E+08	1.2E+08	0.34706	ID=promoter_34141;

3	1E+08	3_117239366	1	8	11	5	5	8	0.86114	1	0	32	10	40	16	25	0.25669436	1	13.375	14.657397					1.2E+08	1.2E+08	0.23548	ID=promoter_34143;
3	1E+08	3_117317082	20	41	22	28	31	25	0.374336	1	1	4	35	9	3	22	3.46E-04	0.0261529	20	14.5012315					1.2E+08	1.2E+08	0.40958	ID=promoter_34148;
3	1E+08	3_117331663	58	25	39	45	42	42	0.76508	1	0	0	16	49	0	33	1.86E-08	4.40E-06	29	22.2453848								
3	1E+08	3_117342751	18	2	5	11	10	8	0.719353	1	48	5	22	4	27	13	0.14207	1	14.375	15.3524079					1.2E+08	1.2E+08	0.16493	ID=promoter_34156;
3	1E+08	3_117351233	25	39	47	21	32	34	0.922482	1	10	30	54	62	20	58	2.11E-05	0.0024277	36	17.6796897					1.2E+08	1.2E+08	0.15533	ID=promoter_34159;
3	1E+08	3_117387630	123	88	90	59	106	75	0.003241	0.16636	61	13	21	2	37	12	0.00419427	0.1971915	57.125	42.5657055	1E+08	1.2E+08	ID=ENSP0000316404;ASAP2;NM_001135191;	ASAP2	1.2E+08	1.2E+08	0.68704	ID=promoter_34168;
3	1E+08	3_117407229	32	56	38	31	44	35	0.225671	1	22	42	49	2	32	26	0.8825541	1	34	16.775833	1E+08	1.2E+08	ID=ENSP0000316404;ASAP2;NM_001135191;	ASAP2				
3	1E+08	3_117422654	7	19	13	28	13	21	0.660505	1	24	5	1	27	15	14	0.99983448	1	15.5	10.5017005	1E+08	1.2E+08	ID=ENSP0000316404;ASAP2;NM_001135191;	ASAP2	1.2E+08	1.2E+08	0.22239	ID=promoter_34177;
3	1E+08	3_117428306	57	25	94	126	41	110	2.00E-05	0.00219	408	146	23	17	277	20	6.01E-45	1.05E-41	112	129.190889	1E+08	1.2E+08	ID=ENSP0000316404;ASAP2;NM_001135191;	ASAP2				
3	1E+08	3_117429490	8	21	5	20	15	13	0.725173	1	4	10	7	3	7	5	0.9051169	1	9.75	7.00510018	1E+08	1.2E+08	ID=ENSP0000316404;ASAP2;NM_001135191;	ASAP2	1.2E+08	1.2E+08	0.85979	ID=promoter_34180;
3	1E+08	3_117430244	20	33	31	17	27	24	0.611598	1	63	23	75	32	43	54	0.32914056	1	36.75	20.9812842	1E+08	1.2E+08	ID=ENSP0000316404;ASAP2;NM_001135191;	ASAP2	1.2E+08	1.2E+08	0.85979	ID=promoter_34180;
3	1E+08	3_117433234	1207	508	1029	743	858	886	0.02893	0.94741	871	626	685	322	749	504	1.68E-07	3.35E-05	748.88	283.528501	1E+08	1.2E+08	ID=ENSP0000316404;ASAP2;NM_001135191;	ASAP2	1.2E+08	1.2E+08	0.85979	ID=promoter_34180;
3	1E+08	3_117439720	43	4	19	10	24	15	0.151024	1	19	16	10	9	18	10	0.42261869	1	16.25	12.0208153	1E+08	1.2E+08	ID=ENSP0000316404;ASAP2;NM_001135191;	ASAP2	1.2E+08	1.2E+08	0.54406	ID=promoter_34182;
3	1E+08	3_117457397	17	1	24	18	9	21	0.212308	1	0	0	12	1	0	7	0.02292696	0.7568963	9.125	9.77515071					1.2E+08	1.2E+08	0.21842	ID=promoter_34188;
3	1E+08	3_117534076	5	6	4	6	6	5	0.851367	1	1	3	19	3	2	11	0.03110435	0.956324	5.875	5.56616051					1.2E+08	1.2E+08	0.2128	ID=promoter_34206;
3	1E+08	3_117558033	8	29	0	8	19	4	0.002035	0.11403	1	19	14	14	10	14	0.60490119	1	11.625	9.57582522								
3	1E+08	3_117625120	44	31	20	18	38	19	0.007518	0.3239	11	6	27	5	9	16	0.2823118	1	20.25	13.3710775					1.2E+08	1.2E+08	0.22777	ID=promoter_34222;
3	1E+08	3_117642588	34	29	0	4	32	2	1.33E-07	#######	40	19	4	0	30	2	1.33E-05	0.0016203	16.25	16.377249								
3	1E+08	3_117705119	0	7	0	1	4	1	0.312866	1	0	0	11	28	0	20	2.07E-05	0.0023913	5.875	9.8479512								
3	1E+08	3_117714602	20	0	35	26	10	31	0.021049	0.7339	1	0	0	0	1	0	0.62906424	1	10.25	14.4494192								
3	1E+08	3_117729627	7	10	16	12	9	14	0.802715	1	4	13	43	8	9	26	0.0081957	0.3355909	14.125	12.2525508					1.2E+08	1.2E+08	0.25	ID=promoter_34240;
3	1E+08	3_117772861	49	27	55	41	38	48	0.943425	1	25	18	28	38	22	33	0.21053573	1	35.125	12.7776311								

3	1E+08	3_117869356	29	15	5	1	22	3	1.41E-04	0.01198	19	16	37	0	18	19	0.92496372	1	15.25	13.1881982								
3	1E+08	3_117898120	7	8	0	0	8	0	0.00917	0.37984	60	9	17	31	35	24	0.52090333	1	16.5	20.2343414								
3	1E+08	3_118040874	97	31	49	62	64	56	0.2748	1	28	8	11	0	18	6	0.0767986	1	35.75	32.4246379								
3	1E+08	3_118146309	3	35	10	0	19	5	0.004955	0.23359	20	8	2	0	14	1	0.00574567	0.2548307	9.75	12.197775								
3	1E+08	3_118147577	0	0	4	4	0	4	0.181755	1	10	0	7	29	5	18	0.01516798	0.5480081	6.75	9.69167537								
3	1E+08	3_118447771	24	95	47	42	60	45	0.073204	1	75	36	51	9	56	30	0.04920085	1	47.375	27.3126737								
3	1E+08	3_118490368	227	143	160	144	185	152	0.004778	0.22659	0	0	0	0	0	0	1	1	84.25	93.7546189					1.2E+08	1.2E+08	0.11207	ID=promoter_34259;
3	1E+08	3_118526793	1	0	13	7	1	10	0.048828	1	44	11	15	2	28	9	0.01512239	0.5480081	11.625	14.2622128								
3	1E+08	3_118698493	29	59	95	55	44	75	0.140892	1	982	694	858	312	838	585	8.72E-07	0.000148	385.5	397.664252								
3	1E+08	3_118701611	45	40	10	21	43	16	1.90E-04	0.01552	102	38	41	63	70	52	0.47955826	1	45	27.9387084					1.2E+08	1.2E+08	0.22045	ID=promoter_34263;
3	1E+08	3_118750287	20	40	9	7	30	8	2.50E-04	0.01962	0	22	8	31	11	20	0.1879092	1	17.125	13.5903064					1.2E+08	1.2E+08	0.127	ID=promoter_34269;
3	1E+08	3_118897034	10	15	4	6	13	5	0.083134	1	26	28	16	0	27	8	0.01145524	0.4380166	13.125	10.1056915								
3	1E+08	3_118993117	14	10	4	2	12	3	0.030625	0.98374	1	1	16	1	1	9	0.02974549	0.9232309	6.125	6.26640704								
3	1E+08	3_119162829	64	3	35	91	34	63	0.095468	1	54	6	36	2	30	19	0.42545995	1	36.375	32.244324								
3	1E+08	3_119171556	0	45	9	20	23	15	0.200003	1	3	32	17	34	18	26	0.34534801	1	20	15.9642458					1.2E+08	1.2E+08	0.12148	ID=promoter_34280;
3	1E+08	3_119205768	10	15	6	5	13	6	0.146758	1	0	0	10	0	0	5	0.06742467	1	5.75	5.62519841								
3	1E+08	3_119209690	15	87	21	42	51	32	0.018778	0.67185	41	75	29	26	58	28	0.01553926	0.5562856	42	25.93949								
3	1E+08	3_119246093	23	17	8	19	20	14	0.325699	1	0	16	32	19	8	26	0.00417605	0.1967234	16.75	9.55809306								
3	1E+08	3_119272666	8	43	6	7	26	7	7.99E-04	0.0525	0	48	34	2	24	18	0.79306625	1	18.5	19.7267042								
3	1E+08	3_119319708	84	52	73	51	68	62	0.355452	1	25	11	27	17	18	22	0.68341813	1	42.5	26.7368125								
3	1E+08	3_119326410	607	198	492	385	403	439	0.562719	1	151	200	162	87	176	125	0.07359077	1	285.25	186.553516					1.2E+08	1.2E+08	0.56692	ID=promoter_34288;
3	1E+08	3_119330509	272	77	172	148	175	160	0.073726	1	111	72	47	50	92	49	0.00649279	0.2797402	118.63	76.5691797	1E+08	1.2E+08	ID=ENSP0000244745-D2;SOX4;NM_003107;	SOX4	1.2E+08	1.2E+08	0.79498	ID=promoter_34289;
3	1E+08	3_119528455	80	100	94	118	90	106	0.99959	1	1	6	0	1	4	1	0.45332778	1	50	52.3695659					1.2E+08	1.2E+08	0.32391	ID=promoter_34306;
3	1E+08	3_119906160	6	9	11	31	8	21	0.137587	1	74	42	64	4	58	34	0.10116204	1	30.125	27.4925315								
3	1E+08	3_119937972	23	30	11	29	27	20	0.290795	1	1	46	32	77	24	55	6.60E-04	0.0445774	31.125	23.0244435								
3	1E+08	3_120165795	13	1	6	0	7	3	0.314307	1	27	10	32	2	19	17	0.99437087	1	11.375	12.1177261								
3	1E+08	3_120182035	27	24	22	18	26	20	0.360218	1	0	32	20	1	16	11	0.74569574	1	18	11.625096					1.2E+08	1.2E+08	0.13796	ID=promoter_34339;
3	1E+08	3_120218527	11	10	7	23	11	15	0.930409	1	0	44	21	3	22	12	0.32694425	1	14.875	14.2170672					1.2E+08	1.2E+08	0.13511	ID=promoter_34342;
3	1E+08	3_120262771	0	5	0	0	3	0	0.172143	1	44	8	8	35	26	22	0.95151398	1	12.5	17.1714048								
3	1E+08	3_120280892	270	119	235	242	195	239	0.901518	1	234	144	131	154	189	143	0.18533823	1	191.13	59.7481321								
3	1E+08	3_120326210	17	14	11	16	16	14	0.724026	1	7	2	2	0	5	1	0.30486099	1	8.625	6.80204801					1.2E+08	1.2E+08	0.27045	ID=promoter_34362;
3	1E+08	3_120372315	16	32	26	9	24	18	0.351411	1	50	46	31	0	48	16	0.00106564	0.0662694	26.25	17.3266929								

3	1E+08	3_120376103	18	29	26	24	24	25	0.917255	1	1	23	37	4	12	21	0.20312388	1	20.25	12.2561938				
3	1E+08	3_120390076	29	41	30	55	35	43	0.979504	1	15	1	7	26	8	17	0.13786384	1	25.5	17.7119814				
3	1E+08	3_120393643	11	69	19	22	40	21	0.009148	0.37984	76	63	26	35	70	31	0.00216709	0.1173835	40.125	25.3345473				
3	1E+08	3_120426496	11	21	42	47	16	45	0.008157	0.34662	4	7	12	0	6	6	0.99142475	1	18	17.5336411	1.2E+08	1.2E+08	0.27045	ID=promoter_34388;
3	1E+08	3_120495533	58	25	47	102	42	75	0.089533	1	24	19	16	45	22	31	0.31838102	1	42	28.55571	1.2E+08	1.2E+08	0.31534	ID=promoter_34406;
3	1E+08	3_120511098	9	15	8	3	12	6	0.213448	1	8	9	2	0	9	1	0.05455957	1	6.75	4.83292281				
3	1E+08	3_120517337	9	0	11	52	5	32	3.82E-04	0.02825	44	20	19	32	32	26	0.8825541	1	23.375	17.9518204				
3	1E+08	3_120520636	51	4	17	41	28	29	0.895448	1	0	0	6	0	0	3	0.19828566	1	14.875	20.1879561				
3	1E+08	3_120535053	172	136	142	83	154	113	6.73E-04	0.04537	70	105	156	28	88	92	0.7222777	1	111.5	48.8730133				
3	1E+08	3_120536350	1184	734	1205	1332	959	1269	0.018781	0.67185	205	172	230	193	189	212	0.16196909	1	656.88	517.789377				
3	1E+08	3_120554631	126	63	146	104	95	125	0.699935	1	24	69	66	65	47	66	0.0906189	1	82.875	39.5490202				
3	1E+08	3_120618072	2	1	3	5	2	4	0.823343	1	0	9	18	2	5	10	0.35527535	1	5	5.95219047				
3	1E+08	3_120645374	7	0	5	4	4	5	0.995512	1	14	10	5	0	12	3	0.09316162	1	5.625	4.74906006				
3	1E+08	3_120656469	4	27	4	14	16	9	0.196587	1	31	8	14	4	20	9	0.18397305	1	13.25	10.6200619	1.2E+08	1.2E+08	0.18336	ID=promoter_34455;
3	1E+08	3_120683650	80	77	55	71	79	63	0.071258	1	31	82	22	27	57	25	0.006106	0.2666968	55.625	25.466715	1.2E+08	1.2E+08	0.19038	ID=promoter_34461;
3	1E+08	3_120685526	52	58	23	20	55	22	4.52E-05	0.00446	58	54	35	59	56	47	0.88120181	1	44.875	16.3570649	1.2E+08	1.2E+08	0.45838	ID=promoter_34462;
3	1E+08	3_120689357	396	162	792	855	279	824	3.84E-42	#######	117	67	248	176	92	212	4.25E-13	1.84E-10	351.63	307.730372	1.2E+08	1.2E+08	0.37876	ID=promoter_34464;
3	1E+08	3_120710953	2	8	8	27	5	18	0.065263	1	11	0	3	23	6	13	0.20797135	1	10.25	9.85248337	1.2E+08	1.2E+08	0.345	ID=promoter_34471;
3	1E+08	3_120713862	158	8	93	86	83	90	0.875203	1	0	11	23	0	6	12	0.2888538	1	47.375	58.2701529				
3	1E+08	3_120739303	8	8	8	5	8	7	0.850897	1	0	1	16	26	1	21	5.13E-05	0.0051875	9	8.46842876	1.2E+08	1.2E+08	0.18848	ID=promoter_34480;
3	1E+08	3_120792471	6	21	17	16	14	17	0.995406	1	0	12	10	0	6	5	0.98461866	1	10.25	7.79651937				
3	1E+08	3_120862941	46	34	12	5	40	9	3.70E-06	0.00049	18	28	11	0	23	6	0.01269948	0.4762983	19.25	15.6090633				
3	1E+08	3_120869124	11	23	1	16	17	9	0.140533	1	1	13	15	0	7	8	0.92121674	1	10	8.46842876				
3	1E+08	3_120928533	24	30	14	44	27	29	0.94719	1	42	2	85	29	22	57	1.06E-04	0.0096524	33.75	24.8696602	1.2E+08	1.2E+08	0.12669	ID=promoter_34519;
3	1E+08	3_120935280	9925	2271	9966	9662	6098	9814	3.95E-83	#######	2566	1474	2998	1171	2020	2085	0.00266959	0.1376567	5004.1	4055.41273	1.2E+08	1.2E+08	0.12268	ID=promoter_34521;
3	1E+08	3_121003214	223	58	113	116	141	115	0.01459	0.54936	15	6	23	0	11	12	0.92663959	1	69.25	77.2227761	1.2E+08	1.2E+08	0.13894	ID=promoter_34540;
3	1E+08	3_121004679	5	14	3	13	10	8	0.719353	1	1	0	22	8	1	15	0.00126073	0.075413	8.25	7.59228932	1.2E+08	1.2E+08	0.32029	ID=promoter_34541;
3	1E+08	3_121015150	113	26	77	71	70	74	0.823208	1	21	0	11	4	11	8	0.87190251	1	40.375	41.3208266	1.2E+08	1.2E+08	0.93169	ID=promoter_34544;
3	1E+08	3_121027376	123	36	81	88	80	85	0.817324	1	28	33	68	38	31	53	0.0228348	0.7568963	61.875	33.8966391				
3	1E+08	3_121029516	106	89	110	104	98	107	0.876862	1	65	64	152	99	65	126	5.01E-06	0.0006925	98.625	27.9894751	1.2E+08	1.2E+08	0.24152	ID=promoter_34546;
3	1E+08	3_121036863	117	37	105	77	77	91	0.998819	1	37	30	27	37	34	32	0.99811495	1	58.375	36.090907	1.2E+08	1.2E+08	0.30789	ID=promoter_34548;

3	1E+08	3_121040714	157	74	96	79	116	88	0.008011	0.34287	14	40	37	3	27	20	0.74799297	1	62.5	50.0085707								
3	1E+08	3_121044891	32	48	22	35	40	29	0.13759	1	36	16	47	12	26	30	0.71540497	1	31	13.3844259								
3	1E+08	3_121068898	9	38	16	31	24	24	0.858118	1	22	22	36	5	22	21	0.99541892	1	22.375	12.1059312								
3	1E+08	3_121112885	13	0	11	6	7	9	0.983538	1	9	1	8	1	5	5	0.99284883	1	6.125	4.96955014					1.2E+08	1.2E+08	0.63693	ID=promoter_34575;
3	1E+08	3_121124825	22	18	8	32	20	20	0.880283	1	10	37	57	5	24	31	0.47191283	1	23.625	17.6225302					1.2E+08	1.2E+08	0.45498	ID=promoter_34578;
3	1E+08	3_121127821	12	45	7	25	29	16	0.046212	1	2	29	22	27	16	25	0.25669436	1	21.125	13.7885822					1.2E+08	1.2E+08	0.82753	ID=promoter_34579;
3	1E+08	3_121166305	388	122	275	248	255	262	0.3214	1	68	112	99	41	90	70	0.54424576	1	169.13	120.937336					1.2E+08	1.2E+08	0.35497	ID=promoter_34589;
3	1E+08	3_121182714	100	64	118	101	82	110	0.654489	1	22	8	8	8	15	8	0.44137572	1	53.625	47.6473279					1.2E+08	1.2E+08	0.65674	ID=promoter_34594;
3	1E+08	3_121190743	403	99	309	267	251	288	0.968013	1	74	140	199	17	107	108	0.82320927	1	188.5	130.760413								
3	1E+08	3_121250689	439	220	293	462	330	378	0.951626	1	339	256	229	66	298	148	2.29E-09	6.25E-07	288	127.680405	1E+08	1.2E+08	ID=ENSP0000337263;TPO;;	TPO	1.2E+08	1.2E+08	0.44381	ID=promoter_34612;
3	1E+08	3_121256937	57	43	89	104	50	97	0.014047	0.53368	0	39	9	0	20	5	0.01914528	0.6572777	42.625	39.4640433	1E+08	1.2E+08	ID=ENSP0000337263;TPO;;	TPO	1.2E+08	1.2E+08	0.39006	ID=promoter_34615;
3	1E+08	3_121269538	1	2	3	14	2	9	0.179815	1	4	20	22	23	12	23	0.11381058	1	11.125	9.62789845					1.2E+08	1.2E+08	0.95019	ID=promoter_34619;
3	1E+08	3_121271165	195	76	190	203	136	197	0.16562	1	230	146	248	220	188	234	0.01048729	0.4077902	188.5	54.7357287					1.2E+08	1.2E+08	0.95019	ID=promoter_34619;
3	1E+08	3_121277427	162	79	253	204	121	229	9.77E-05	0.00872	47	37	17	25	42	21	0.06366916	1	103	90.8137812					1.2E+08	1.2E+08	0.39791	ID=promoter_34620;
3	1E+08	3_121281032	110	273	176	164	192	170	0.027626	0.91036	199	197	321	179	198	250	0.00491623	0.2246966	202.38	65.8438358								
3	1E+08	3_121284055	7	5	8	8	6	8	0.9722	1	43	9	4	1	26	3	2.53E-04	0.0200383	10.625	13.3409948					1.2E+08	1.2E+08	0.74274	ID=promoter_34621;
3	1E+08	3_121301991	52	15	70	66	34	68	0.036717	1	54	13	46	14	34	30	0.98073068	1	41.25	23.8132616	1E+08	1.2E+08	ID=ENSP0000337263-D2;TPO;;	TPO	1.2E+08	1.2E+08	0.39006	ID=promoter_34627;
3	1E+08	3_121308239	167	46	106	97	107	102	0.324021	1	94	55	47	25	75	36	0.00424411	0.1992065	79.625	45.706947	1E+08	1.2E+08	ID=ENSP0000337263-D2;TPO;;	TPO	1.2E+08	1.2E+08	0.44381	ID=promoter_34630;
3	1E+08	3_121368226	265	78	235	133	172	184	0.685797	1	88	52	103	14	70	59	0.86485049	1	121	87.2991573								
3	1E+08	3_121388725	18	97	19	29	58	24	4.20E-05	0.00416	33	32	36	5	33	21	0.39890162	1	33.625	27.5781357					1.2E+08	1.2E+08	0.21242	ID=promoter_34653;
3	1E+08	3_121409636	59	50	80	46	55	63	0.99177	1	11	47	53	15	29	34	0.64824985	1	45.125	22.585947					1.2E+08	1.2E+08	0.35364	ID=promoter_34660;
3	1E+08	3_121426684	35	48	33	38	42	36	0.38232	1	121	30	88	69	76	79	0.77564863	1	57.75	32.5740914					1.2E+08	1.2E+08	0.61966	ID=promoter_34666;
3	1E+08	3_121432950	0	0	23	6	0	15	0.001671	0.09737	0	0	14	20	0	17	1.04E-04	0.0094884	7.875	9.74587825					1.2E+08	1.2E+08	0.65564	ID=promoter_34668;
3	1E+08	3_121621476	13	38	50	20	26	35	0.867873	1	8	17	42	25	13	34	0.00397338	0.1887956	26.625	15.0422025					1.2E+08	1.2E+08	0.52962	ID=promoter_34707;
3	1E+08	3_121631009	7	21	12	4	14	8	0.253058	1	84	0	11	16	42	14	0.00250702	0.1318429	19.375	26.9387533					1.2E+08	1.2E+08	0.53681	ID=promoter_34710;
3	1E+08	3_121635064	56	21	16	52	39	34	0.446846	1	9	25	23	16	17	20	0.769441	1	27.25	17.268882					1.2E+08	1.2E+08	0.18686	ID=promoter_34711;
3	1E+08	3_121646416	101	28	59	74	65	67	0.758939	1	55	104	134	74	80	104	0.07357561	1	78.625	33.2949481					1.2E+08	1.2E+08	0.68925	ID=promoter_34713;

3	1E+08	3_121681194	8	21	14	18	15	16	0.965652	1	7	6	5	2	7	4	0.73798468	1	10.125	6.74933859					1.2E+08	1.2E+08	0.3863	ID=promoter_34725;
3	1E+08	3_121922526	70	46	45	33	58	39	0.024629	0.83312	76	73	12	12	75	12	1.23E-09	3.55E-07	45.875	25.8757333								
3	1E+08	3_122020949	555	68	233	203	312	218	2.25E-08	#######	228	48	37	56	138	47	5.09E-09	1.33E-06	178.5	174.04515								
3	1E+08	3_122717842	3	15	3	13	9	8	0.848833	1	37	25	31	6	31	19	0.35951856	1	16.625	13.0486726								
3	1E+08	3_122728862	69	48	79	61	59	70	0.99791	1	133	38	48	39	86	44	0.00501518	0.2287068	64.375	31.2407129								
3	1E+08	3_122826102	40	34	41	43	37	42	0.98949	1	47	60	143	27	54	85	0.00876602	0.3537697	54.375	37.0711189								
3	1E+08	3_122854324	1	12	2	72	7	37	3.26E-04	0.02485	0	11	24	19	6	22	0.00549069	0.2478586	17.625	23.6216456					1.2E+08	1.2E+08	0.757	ID=promoter_34742;
3	1E+08	3_123192347	14	3	75	55	9	65	3.84E-08	#######	59	25	36	44	42	40	0.99257914	1	38.875	24.2100186								
3	1E+08	3_123604780	18	54	6	14	36	10	6.61E-05	0.0062	22	27	40	18	25	29	0.71264652	1	24.875	15.3942243								
4	43939	4_43939	142	43	57	98	93	78	0.08993	1	15	53	50	24	34	37	0.79599102	1	60.25	41.2925106								
4	44606	4_44606	2023	1239	1246	1671	1631	1459	4.43E-13	#######	709	282	1083	392	496	738	2.04E-15	1.07E-12	1080.6	602.276856					44433	45168	0.13269	ID=promoter_34763;
4	81094	4_81094	361	139	378	360	250	369	0.019202	0.68357	109	108	133	22	109	78	0.21608221	1	201.25	141.336225					80353	81463	0.42344	ID=promoter_34767;
4	142978	4_142978	0	37	3	6	19	5	0.004955	0.23359	1	3	6	1	2	4	0.6610374	1	7.125	12.2758474					142546	143321	0.08873	ID=promoter_34777;
4	144170	4_144170	2	3	8	2	3	5	0.88968	1	0	11	1	28	6	15	0.10150651	1	6.875	9.32642176					143574	145225	0.43345	ID=promoter_34778;
4	286612	4_286612	6	9	10	7	8	9	0.996316	1	5	5	5	18	5	12	0.17891578	1	8.125	4.42194204					286587	288150	0.54968	ID=promoter_34797;
4	365447	4_365447	25	9	13	2	17	8	0.087954	1	1	11	10	9	6	10	0.51486293	1	10	7.3872477	362496	390657	ID=ENSP000003665 22;;NM_025251;					
4	394601	4_394601	958	383	767	670	671	719	0.241659	1	248	173	379	96	211	238	0.11545504	1	459.25	306.745008					392321	395370	0.68764	ID=promoter_34807;
4	459776	4_459776	1	21	18	19	11	19	0.589612	1	78	15	33	1	47	17	0.00260618	0.1361074	23.25	24.4992711					459373	460891	0.28614	ID=promoter_34823;
4	462158	4_462158	261	169	228	219	215	224	0.462378	1	166	83	123	81	125	102	0.63268956	1	166.25	67.2410376	459792	462758	ID=ENSP000002925 43;CYHR1;;	CYHR1	461852	463741	0.5207	ID=promoter_34824;
4	510296	4_510296	5	1	15	42	3	29	1.75E-04	0.01448	2	1	32	14	2	23	6.46E-05	0.0062789	14	15.4550038					505871	510614	0.8053	ID=promoter_34833;
4	544738	4_544738	23	38	8	20	31	14	0.008767	0.36763	2	24	25	59	13	42	1.51E-04	0.0129335	24.875	17.6184765					542749	544749	0.45449	ID=promoter_34838;
4	573762	4_573762	6	15	11	16	11	14	0.979671	1	10	31	23	1	21	12	0.40192224	1	14.125	9.50845488								
4	592685	4_592685	5667	3519	5823	5050	4593	5437	0.900639	1	3974	2802	2948	1418	3388	2183	2.67E-39	3.85E-36	3900.1	1538.3473					591355	593597	0.68964	ID=promoter_34848;
4	597924	4_597924	175	38	148	133	107	141	0.661908	1	67	38	39	0	53	20	0.00189061	0.1047116	79.75	63.5109664					597102	598872	0.43091	ID=promoter_34851;
4	647978	4_647978	43	44	50	68	44	59	0.797703	1	37	29	42	21	33	32	0.98369494	1	41.75	14.0382132					643253	648343	0.77771	ID=promoter_34860;
4	656534	4_656534	389	219	306	360	304	333	0.689342	1	146	120	112	76	133	94	0.13011741	1	216	121.482509	656175	678778	ID=ENSP000003326 98;HSF1;NM_005526;	HSF1	655045	657268	0.66517	ID=promoter_34861;
4	669372	4_669372	78	51	53	14	65	34	4.60E-04	0.03302	2	0	29	7	1	18	2.55E-04	0.0200383	29.25	28.6343949	656175	678778	ID=ENSP000003326 98;HSF1;NM_005526;	HSF1				

4	678940	4_678940	18	39	3	23	29	13	0.011304	0.44898	28	34	19	5	31	12	0.02977149	0.9232309	21.125	12.755251					678544	679359	0.1888	ID=promoter_34866;
4	743811	4_743811	221	98	1	0	160	1	8.53E-41	#######	124	77	1	7	101	4	1.20E-18	7.72E-16	66.125	80.0186362					743662	745581	0.34717	ID=promoter_34883;
4	748171	4_748171	5241	1434	5286	2973	3338	4130	0.07203	1	2173	1125	1533	1120	1649	1327	6.28E-04	0.0425975	2610.6	1747.41041					746313	749016	0.58469	ID=promoter_34884;
4	838631	4_838631	28	1	20	20	15	20	0.931941	1	10	27	20	18	19	19	0.97309647	1	18	8.83176087								
4	848126	4_848126	217	76	178	99	147	139	0.189168	1	12	31	39	29	22	34	0.16818144	1	85.125	75.4480663	845524	852002	ID=ENSP0000325618;PARP10;NM_032789;	PARP10				
4	890337	4_890337	28	57	44	84	43	64	0.480537	1	73	28	61	36	51	49	0.98415652	1	51.375	20.8390944					889351	891621	0.39403	ID=promoter_34912;
4	915262	4_915262	6	28	9	6	17	8	0.087954	1	16	17	17	17	17	17	0.97589399	1	14.5	7.34846923								
4	923891	4_923891	133	244	634	387	189	511	7.30E-23	#######	95	179	122	94	137	108	0.45152152	1	236	188.288532								
4	934441	4_934441	6	4	21	16	5	19	0.046679	1	5	27	21	7	16	14	0.9876114	1	13.375	8.95923307					934130	935142	0.19965	ID=promoter_34917;
4	1E+06	4_103439	6	17	9	10	12	10	0.724827	1	23	16	0	6	20	3	0.00346999	0.1723921	10.875	7.37636186								
4	1E+06	4_115666	27	58	30	13	43	22	0.004975	0.23406	26	32	20	3	29	12	0.05423954	1	26.125	16.0840427								
4	1E+06	4_1166600	2	21	6	7	12	7	0.325249	1	4	3	1	0	4	1	0.45332778	1	5.5	6.69754753					1165393	1168331	0.39171	ID=promoter_34957;
4	1E+06	4_1167449	13	33	8	14	23	11	0.040732	1	0	18	15	0	9	8	0.99626583	1	12.625	10.6360237					1165393	1168331	0.39171	ID=promoter_34957;
4	1E+06	4_128724	1	12	10	4	7	7	0.956352	1	2	1	6	6	2	6	0.31385293	1	5.25	4.09703726								
4	1E+06	4_1288186	15	29	11	44	22	28	0.959756	1	12	47	46	19	30	33	0.79414776	1	27.875	15.751984					1287774	1288774	0.19267	ID=promoter_34982;
4	1E+06	4_132141	40	43	24	62	42	43	0.821371	1	12	71	60	18	42	39	0.99997259	1	41.25	21.9268264								
4	1E+06	4_136215	297	226	376	322	262	349	0.297496	1	267	227	93	79	247	86	4.17E-15	2.14E-12	235.88	104.851511								
4	1E+06	4_142970	34	36	52	49	35	51	0.612388	1	51	6	43	0	29	22	0.77701989	1	33.875	20.2233069								
4	1E+06	4_143223	149	152	124	96	151	110	6.38E-04	0.04361	165	49	67	27	107	47	7.19E-05	0.0069272	103.63	51.8733003								
4	1E+06	4_148730	3	31	16	40	17	28	0.540451	1	21	12	2	1	17	2	0.0046333	0.2138882	15.75	14.3003496								
4	1E+06	4_1493157	12	25	44	47	19	46	0.025024	0.84326	27	4	1	6	16	4	0.04223275	1	20.75	17.9344043					1490163	1493829	0.72758	ID=promoter_35009;
4	2E+06	4_1502787	12	33	11	16	23	14	0.143642	1	22	13	24	2	18	13	0.78939737	1	16.625	9.5009398					1501623	1507014	0.839	ID=promoter_35012;
4	2E+06	4_1503533	13	43	17	28	28	23	0.444971	1	13	30	20	24	22	22	0.96891521	1	23.5	10.1277554					1501623	1507014	0.839	ID=promoter_35012;
4	2E+06	4_1531552	58	46	25	23	52	24	4.64E-04	0.03323	0	21	19	4	11	12	0.92663959	1	24.5	19.4569121					1530817	1532043	0.27941	ID=promoter_35020;
4	2E+06	4_155257	11	27	10	0	19	5	0.004955	0.23359	0	14	0	0	7	0	0.03898208	1	7.75	9.75045786								
4	2E+06	4_1580010	2	15	17	3	9	10	0.993074	1	89	24	21	8	57	15	2.19E-05	0.0025036	22.375	28.0913688					1577875	1581085	0.61892	ID=promoter_35033;
4	2E+06	4_1586155	79	85	37	82	82	60	0.019644	0.69635	45	2	43	5	24	24	0.96613769	1	47.25	32.9534303					1585786	1587269	0.30367	ID=promoter_35036;
4	2E+06	4_166177	13	5	3	5	9	4	0.244237	1	13	5	0	11	9	6	0.8208167	1	6.875	4.85320218								
4	2E+06	4_168936	9	8	2	7	9	5	0.390586	1	14	0	10	29	7	20	0.02608553	0.8424716	9.875	8.90324981								
4	2E+06	4_176736	422	286	334	401	354	368	0.268	1	34	87	52	82	61	67	0.63117539	1	212.25	164.746342								
4	2E+06	4_181073	21	29	36	20	25	28	0.986003	1	36	12	26	1	24	14	0.38067267	1	22.625	11.9515391								
4	2E+06	4_1853993	0	22	0	0	11	0	0.001579	0.09256	9	14	20	0	12	10	0.96947392	1	8.125	9.50845488					1853951	1854474	0.14191	ID=promoter_35108;
4	2E+06	4_1900118	155	60	77	43	108	60	1.26E-05	0.00147	39	18	38	31	29	35	0.57403411	1	57.625	43.2399865					1898955	1902085	0.48495	ID=promoter_35122;
4	2E+06	4_190282	12	42	39	45	27	42	0.517231	1	35	28	33	24	32	29	0.99609288	1	32.25	10.7404443								
4	2E+06	4_191220	60	57	119	101	59	110	0.015027	0.5639	14	14	60	8	14	34	0.0069754	0.2965071	54.125	41.1111299								
4	2E+06	4_191935	0	8	0	4	4	2	0.597627	1	49	7	14	0	28	7	0.00393447	0.1875084	10.25	16.4121035								
4	2E+06	4_1923235	11	3	18	26	7	22	0.061117	1	7	21	32	9	14	21	0.37101303	1	15.875	10.0631932					1922788	1924134	0.1749	ID=promoter_35128;
4	2E+06	4_193041	7	11	5	18	9	12	0.95859	1	0	4	3	2	2	3	0.86792893	1	6.25	5.8002463								
4	2E+06	4_193321	13	7	6	9	10	8	0.719353	1	23	0	6	2	12	4	0.18067673	1	8.25	7.16638981								

4	2E+06	4_1957643	6	3	5	2	5	4	0.848147	1	2	5	20	10	4	15	0.026797	0.8546242	6.625	5.99851172					1955910	1957772	0.27488	ID=promoter_35139;
4	2E+06	4_1968367	52	222	62	75	137	69	2.78E-08	#######	150	38	65	139	94	102	0.54472517	1	100.38	63.6349803					1967790	1968654	0.16724	ID=promoter_35143;
4	2E+06	4_1984775	0	24	53	27	12	40	0.004048	0.19796	29	19	7	0	24	4	0.00163693	0.0924179	19.875	17.7477363					1984701	1985509	0.17498	ID=promoter_35148;
4	2E+06	4_2022586	17	33	6	37	25	22	0.614783	1	60	34	43	84	47	64	0.13161654	1	39.25	24.2590189					2022061	2023342	0.24539	ID=promoter_35161;
4	2E+06	4_2044447	10	50	9	22	30	16	0.032973	1	46	3	34	64	25	49	0.00774653	0.3222758	29.75	22.237356					2044378	2045216	0.26372	ID=promoter_35167;
4	2E+06	4_2044602	13	1	8	16	7	12	0.724589	1	0	0	8	2	0	5	0.06742467	1	6	6.21059003					2044378	2045216	0.26372	ID=promoter_35167;
4	2E+06	4_2060544	90	39	132	239	65	186	1.30E-09	#######	20	11	13	23	16	18	0.85288016	1	70.875	80.3553492					2060105	2060682	0.21363	ID=promoter_35173;
4	2E+06	4_206668	0	19	2	1	10	2	0.03464	1	23	0	0	0	12	0	0.00384008	0.1844256	5.625	9.57582522								
4	2E+06	4_2067607	39	59	39	53	49	46	0.553876	1	33	67	73	62	50	68	0.11742194	1	53.125	14.6622694					2067299	2069978	0.38384	ID=promoter_35175;
4	2E+06	4_2067990	57	26	34	33	42	34	0.270679	1	0	2	10	3	1	7	0.08320466	1	20.625	20.2832619					2067299	2069978	0.38384	ID=promoter_35175;
4	2E+06	4_2068951	1	0	11	21	1	16	0.004036	0.19749	0	0	5	3	0	4	0.11562589	1	5.125	7.43423548					2067299	2069978	0.38384	ID=promoter_35175;
4	2E+06	4_2072166	10	15	16	3	13	10	0.600182	1	65	0	32	0	33	16	0.09595749	1	17.625	21.8889241					2072110	2072945	0.16443	ID=promoter_35176;
4	2E+06	4_207352	9	40	6	7	25	7	0.001309	0.07852	16	76	12	2	46	7	2.43E-06	0.0003668	21	25.1225567								
4	2E+06	4_2104721	8	15	1	8	12	5	0.126784	1	0	12	26	0	6	13	0.20797135	1	8.75	8.95624283					2104362	2105014	0.15574	ID=promoter_35182;
4	2E+06	4_210699	0	2	9	16	1	13	0.014136	0.5348	13	14	12	8	14	10	0.81923661	1	9.25	5.72588109								
4	2E+06	4_2150159	16	48	43	9	32	26	0.376129	1	5	26	28	6	16	17	0.92610249	1	22.625	16.5437731					2150062	2150561	0.12683	ID=promoter_35195;
4	2E+06	4_2184758	100	86	115	90	93	103	0.922635	1	85	63	93	18	74	56	0.5174724	1	81.25	29.4606275					2183628	2184892	0.23211	ID=promoter_35207;
4	2E+06	4_2226427	55	0	49	67	28	58	0.043412	1	7	38	25	0	23	13	0.35430953	1	30.125	26.1120525					2226095	2226616	0.15876	ID=promoter_35217;
4	2E+06	4_2405252	6	24	8	3	15	6	0.065303	1	8	6	21	0	7	11	0.54353855	1	9.5	8.48528137					2404065	2405493	0.20677	ID=promoter_35237;
4	2E+06	4_240823	50	52	48	42	51	45	0.377067	1	64	58	106	43	61	75	0.25923598	1	57.875	20.7738675								
4	3E+06	4_268640	2135	1308	2240	3011	1722	2626	2.26E-16	#######	1676	948	1409	767	1312	1088	0.02476895	0.8050493	1686.8	743.837108								
4	3E+06	4_281119	0	9	3	0	5	2	0.402696	1	26	5	25	1	16	13	0.9394435	1	8.625	10.8356225								
4	3E+06	4_283674	15	77	21	27	46	24	0.004234	0.2055	33	87	43	21	60	32	0.03795192	1	40.5	27.1240536								
4	3E+06	4_291728	0	14	11	4	7	8	0.998739	1	59	3	32	19	31	26	0.931505	1	17.75	19.5940953								
4	3E+06	4_2919426	23	30	15	39	27	27	0.841861	1	0	27	14	4	14	9	0.68990751	1	19	13.2449451					2919021	2920075	0.24982	ID=promoter_35275;
4	3E+06	4_293117	344	108	234	333	226	284	0.74159	1	108	97	101	26	103	64	0.04069473	1	168.88	119.190649								
4	3E+06	4_297562	136	123	40	82	130	61	7.73E-09	#######	83	16	14	41	50	28	0.09740279	1	66.875	46.587667								
4	3E+06	4_297567	1239	439	915	1031	839	973	0.971388	1	353	191	161	153	272	157	1.14E-05	0.0014204	560.25	435.464858								
4	3E+06	4_299201	9	0	9	31	5	20	0.033145	1	30	4	2	2	17	2	0.0046333	0.2138882	10.875	12.5406482								
4	3E+06	4_3033525	68	48	60	45	58	53	0.419924	1	19	12	57	32	16	45	3.20E-04	0.0244334	42.625	19.9852624					3032949	3033575	0.11323	ID=promoter_35300;
4	3E+06	4_311081	2	62	10	6	32	8	9.18E-05	0.00825	22	15	15	54	19	35	0.04884304	1	23.25	22.3910058								
4	3E+06	4_312482	39	19	11	5	29	8	4.11E-04	0.03016	49	5	25	4	27	15	0.27474073	1	19.625	16.9616163								
4	3E+06	4_319450	166	74	124	136	120	130	0.821019	1	59	66	34	27	63	31	0.0134489	0.4972223	85.75	50.4288749								
4	3E+06	4_3315765	13	2	3	10	8	7	0.850897	1	0	1	7	18	1	13	0.0036419	0.1775497	6.75	6.4531277	3E+06	3358597	ID=ENSP0000354913-D2;FAM135A;;	FAM135A	3315679	3317441	0.18971	ID=promoter_35327;

4	3E+06	4_3320226	0	37	14	24	19	19	0.885913	1	52	23	35	24	38	30	0.80512437	1	26.125	15.6519054	3E+06	3358597	ID=ENSP0000354913-D2;FAM135A;;	FAM135A	3319047	3320721	0.20803	ID=promoter_35328;
4	3E+06	4_335937	6	16	15	1	11	8	0.5832	1	3	29	18	30	16	24	0.32201294	1	14.75	11.0291821								
4	3E+06	4_3440905	41	89	65	44	65	55	0.201317	1	207	73	98	34	140	66	2.36E-05	0.002647	81.375	55.7108286					3440545	3441305	0.11373	ID=promoter_35339;
4	3E+06	4_347235	106	35	94	80	71	87	0.963348	1	120	13	42	33	67	38	0.05046999	1	65.375	39.5110744								
4	4E+06	4_351888	68	120	12	12	94	12	1.38E-17	#######	33	14	6	1	24	4	0.00163693	0.0924179	33.25	41.0391799								
4	4E+06	4_352974	84	0	41	69	42	55	0.865381	1	30	71	23	0	51	12	2.36E-05	0.002647	39.75	32.354068								
4	4E+06	4_355414	0	50	7	10	25	9	0.005635	0.25889	70	21	88	121	46	105	9.30E-07	0.0001564	45.875	43.978688								
4	4E+06	4_369582	15	32	37	27	24	32	0.89335	1	48	40	17	32	44	25	0.1424362	1	31	11.1867268								
4	4E+06	4_377040	90	125	124	105	108	115	0.770486	1	184	67	52	68	126	60	8.63E-05	0.008155	101.88	42.7131211								
4	4E+06	4_380748	134	66	97	38	100	68	0.0021	0.11676	2	5	8	3	4	6	0.75330063	1	44.125	50.2719391								
4	4E+06	4_408217	205	27	128	137	116	133	0.98407	1	133	45	86	69	89	78	0.93650472	1	103.75	58.1445244								
4	4E+06	4_408294	34	73	21	16	54	19	1.03E-05	0.00123	20	45	13	4	33	9	0.00251067	0.1318429	28.25	22.0632209								
4	4E+06	4_418570	35	53	15	7	44	11	2.81E-06	0.00039	56	10	15	3	33	9	0.00251067	0.1318429	24.25	20.9540313								
4	4E+06	4_423428	50	65	26	36	58	31	0.001405	0.08377	54	56	50	70	55	60	0.68321005	1	50.875	14.357054								
4	4E+06	4_443502	23	61	29	11	42	20	0.002773	0.14678	29	56	64	10	43	37	0.94624198	1	35.375	21.9540917								
4	5E+06	4_464119	70	45	85	107	58	96	0.114719	1	14	7	25	1	11	13	0.83819512	1	44.25	39.2892206								
4	5E+06	4_476817	21	4	45	95	13	70	2.06E-07	#######	16	0	7	24	8	16	0.19094317	1	26.5	31.0989205								
4	5E+06	4_479426	3	8	7	9	6	8	0.9722	1	0	0	11	23	0	17	1.04E-04	0.0094884	7.625	7.44383734								
4	5E+06	4_483767	228	133	238	245	181	242	0.411215	1	57	44	100	36	51	68	0.14284968	1	135.13	90.0419545								
4	5E+06	4_485612	0	36	0	0	18	0	2.60E-05	0.00275	0	2	2	2	1	2	0.81304206	1	5.25	12.4642345								
4	5E+06	4_486277	28	35	0	9	32	5	5.06E-06	0.00065	35	29	15	0	32	8	0.00178361	0.0995187	18.875	14.7883883								
4	5E+06	4_5026892	23	20	5	6	22	6	0.002551	0.13706	66	9	10	32	38	21	0.15716135	1	21.375	20.3395287					5026566	5027275	0.13137	ID=promoter_35387;
4	5E+06	4_507423	16	21	31	21	19	26	0.877519	1	10	11	16	24	11	20	0.1879092	1	18.75	6.96419414								
4	5E+06	4_5134526	4	23	14	1	14	8	0.253058	1	22	1	20	0	12	10	0.96947392	1	10.625	10.1691059	5E+06	5208886	ID=ENSP00000348108;KHDRBS3;NM_006558;	KHDRBS3	5133790	5134606	0.12977	ID=promoter_35409;
4	5E+06	4_5135907	4	11	1	18	8	10	0.991043	1	0	17	6	0	9	3	0.27712561	1	7.125	7.37636186	5E+06	5208886	ID=ENSP00000348108;KHDRBS3;NM_006558;	KHDRBS3	5135512	5136212	0.12052	ID=promoter_35410;
4	5E+06	4_5146834	102	53	101	120	78	111	0.423811	1	21	106	34	35	64	35	0.03975055	1	71.5	39.5943719	5E+06	5208886	ID=ENSP00000348108;KHDRBS3;NM_006558;	KHDRBS3				
4	5E+06	4_5177804	8	5	28	24	7	26	0.016775	0.61417	3	41	33	0	22	17	0.8526593	1	17.75	15.6182128	5E+06	5208886	ID=ENSP00000348108;KHDRBS3;NM_006558;	KHDRBS3	5177466	5178373	0.21758	ID=promoter_35422;
4	5E+06	4_5184980	1	43	0	0	22	0	2.49E-06	0.00035	0	0	0	0	0	0	1	1	5.5	15.1563282	5E+06	5208886	ID=ENSP00000348108;KHDRBS3;NM_006558;	KHDRBS3	5184398	5185470	0.18559	ID=promoter_35423;
4	5E+06	4_5195739	29	19	9	21	24	15	0.151024	1	15	0	9	1	8	5	0.78486764	1	12.875	10.034761	5E+06	5208886	ID=ENSP00000348108;KHDRBS3;NM_006558;	KHDRBS3	5195407	5196072	0.11795	ID=promoter_35427;
4	5E+06	4_526976	39	37	21	21	38	21	0.018051	0.65084	66	12	34	6	39	20	0.09202448	1	29.5	18.9057814								
4	6E+06	4_552511	9	2	5	7	6	6	0.962469	1	58	5	17	1	32	9	0.00363228	0.1775497	13	18.8452343								
4	6E+06	4_557982	127	18	13	34	73	24	5.65E-08	#######	22	38	24	31	30	28	0.99966898	1	38.375	36.7654244								
4	6E+06	4_559304	0	23	0	6	12	3	0.030625	0.98374	6	5	6	1	6	4	0.87665919	1	5.875	7.43423548								
4	6E+06	4_560206	31	1	15	14	16	15	0.822707	1	11	0	20	3	6	12	0.2888538	1	11.875	10.5889093								
4	6E+06	4_562896	32	294	56	67	163	62	1.85E-14	#######	65	418	185	75	242	130	2.49E-06	0.0003742	149	139.527775								
4	6E+06	4_579053	21	33	31	30	27	31	0.996709	1	2	31	67	79	17	73	2.97E-09	8.05E-07	36.75	24.7314144								
4	6E+06	4_582152	25	57	21	26	41	24	0.022233	0.76438	24	60	14	1	42	8	3.29E-05	0.0035427	28.5	20.2343414								
4	6E+06	4_5830095	4	62	18	25	33	22	0.114927	1	18	46	55	30	32	43	0.2783992	1	32.25	20.1901673	6E+06	5954342	ID=ENSP00000326997;ZFAT;;	ZFAT				
4	6E+06	4_5868235	26	41	19	10	34	15	0.004584	0.21859	0	11	24	19	6	22	0.00549069	0.2478586	18.75	12.3259309	6E+06	5954342	ID=ENSP00000326997;ZFAT;;	ZFAT				

4	6E+06	4_5871474	80	40	51	37	60	44	0.057742	1	69	43	75	97	56	86	0.01212024	0.4594926	61.5	21.8958574	6E+06	5954342	ID=ENSP00000326997;ZFAT;;	ZFAT	5870735	5871916	0.20955	ID=promoter_35454;
4	6E+06	4_5871748	0	39	7	47	20	27	0.892511	1	19	0	5	0	10	3	0.19568363	1	14.625	18.7230759	6E+06	5954342	ID=ENSP00000326997;ZFAT;;	ZFAT	5870735	5871916	0.20955	ID=promoter_35454;
4	6E+06	4_5872084	0	18	17	15	9	16	0.603365	1	43	1	23	14	22	19	0.97472654	1	16.375	13.4582688	6E+06	5954342	ID=ENSP00000326997;ZFAT;;	ZFAT				
4	6E+06	4_5877691	16	59	14	22	38	18	0.004632	0.22041	49	43	37	8	46	23	0.04897987	1	31	18.5626353	6E+06	5954342	ID=ENSP00000326997;ZFAT;;	ZFAT				
4	6E+06	4_5890930	0	3	0	1	2	1	0.773064	1	1	18	0	27	10	14	0.60490119	1	6.25	10.3613292	6E+06	5954342	ID=ENSP00000326997;ZFAT;;	ZFAT	5890781	5892446	0.26254	ID=promoter_35458;
4	6E+06	4_5900246	39	68	19	19	54	19	1.03E-05	0.00123	76	23	51	11	50	31	0.20817627	1	38.25	24.4642596	6E+06	5954342	ID=ENSP00000326997;ZFAT;;	ZFAT				
4	6E+06	4_5928733	39	21	38	80	30	59	0.066164	1	0	20	11	3	10	7	0.84912739	1	26.5	25.96151	6E+06	5954342	ID=ENSP00000326997;ZFAT;;	ZFAT				
4	6E+06	4_5936126	147	89	187	122	118	155	0.650621	1	33	49	61	17	41	39	0.9933981	1	88.125	59.4677283	6E+06	5954342	ID=ENSP00000326997;ZFAT;;	ZFAT				
4	6E+06	4_5974868	4	56	93	112	30	103	3.97E-07	#######	88	43	70	77	66	74	0.53116459	1	67.875	33.6173318					5974495	5975401	0.21841	ID=promoter_35470;
4	6E+06	4_603341	70	90	194	231	80	213	1.07E-09	#######	29	8	27	5	19	16	0.96066018	1	81.75	86.347719								
4	6E+06	4_620427	20	101	28	32	61	30	3.07E-04	0.0236	1	53	42	16	27	29	0.85917478	1	36.625	30.4909237								
4	6E+06	4_620967	3	73	22	27	38	25	0.074872	1	23	67	33	17	45	25	0.11611057	1	33.125	24.4039663								
4	6E+06	4_628437	6	0	0	15	3	8	0.459247	1	0	8	21	46	4	34	2.21E-06	0.0003393	12	15.7207779								
4	6E+06	4_631368	53	20	18	24	37	21	0.025035	0.84326	95	0	17	1	48	9	6.63E-06	0.0008864	28.5	31.4551835								
4	6E+06	4_633110	38	13	29	20	26	25	0.776821	1	0	0	3	18	0	11	0.00265095	0.136745	15.125	13.9226178								
4	6E+06	4_642214	0	11	7	0	6	4	0.674789	1	71	31	41	0	51	21	0.00566618	0.2544931	20.125	25.6706475								
4	6E+06	4_648196	11	7	3	8	9	6	0.554309	1	20	9	1	20	15	11	0.83933961	1	9.875	7.01910658								
4	7E+06	4_659908	288	121	310	185	205	248	0.947849	1	71	57	67	2	64	35	0.03975055	1	137.63	112.863935								
4	7E+06	4_667681	38	52	72	32	45	52	0.99731	1	218	139	210	153	179	182	0.68024091	1	114.25	75.8621871								
4	7E+06	4_676460	25	36	21	23	31	22	0.19035	1	55	18	47	63	37	55	0.08111667	1	36	17.1130694								
4	7E+06	4_676686	9	76	18	16	43	17	3.55E-04	0.02675	100	36	82	3	68	43	0.14210593	1	42.5	37.8266724								
4	7E+06	4_688323	16	21	38	18	19	28	0.743706	1	0	12	3	17	6	10	0.51486293	1	15.625	11.6733824								
4	7E+06	4_6913396	24	0	10	8	12	9	0.592799	1	0	0	1	0	0	1	0.58312783	1	5.375	8.53459347					6913188	6913848	0.1379	ID=promoter_35498;
4	7E+06	4_700135	54	15	39	36	35	38	0.947014	1	0	14	4	8	7	6	0.99011599	1	21.25	19.3520025								
4	7E+06	4_7119641	55	26	87	15	41	51	0.961552	1	18	37	7	1	28	4	2.96E-04	0.0229072	30.75	28.4692817	7E+06	7329601	ID=ENSP00000220616;TG;NM_003235;	TG				
4	7E+06	4_7168883	39	31	17	37	35	27	0.257207	1	0	12	38	17	6	28	3.40E-04	0.0257676	23.875	14.426538	7E+06	7329601	ID=ENSP00000220616;TG;NM_003235;	TG				
4	7E+06	4_7204061	26	26	11	19	26	15	0.082345	1	26	43	67	22	35	45	0.34560916	1	30	17.4519749	7E+06	7329601	ID=ENSP00000220616;TG;NM_003235;	TG				
4	7E+06	4_7225762	9	18	13	14	14	14	0.914609	1	12	34	32	29	23	31	0.39238725	1	20.125	9.96332561	7E+06	7329601	ID=ENSP00000220616;TG;NM_003235;	TG				
4	7E+06	4_7312959	3	49	1	2	26	2	4.27E-06	0.00056	45	32	47	2	39	25	0.35043112	1	22.625	22.620708	7E+06	7329601	ID=ENSP00000220616;TG;NM_003235;	TG				
4	7E+06	4_7327208	664	338	602	575	501	589	0.999287	1	349	274	264	123	312	194	6.33E-05	0.0061905	398.63	192.226011	7E+06	7329601	ID=ENSP00000220616;TG;NM_003235;	TG	7326429	7327901	0.39239	ID=promoter_35553;
4	7E+06	4_743603	448	224	414	335	336	375	0.803317	1	289	196	182	57	243	120	7.06E-08	1.52E-05	268.13	129.660477								

4	8E+06	4_7501071	0	38	10	13	19	12	0.233425	1	36	38	85	45	37	65	0.00685049	0.2925913	33.125	26.5245843	7E+06	7549662	ID=ENSP0000250173;LRRC6;NM_012472;	LRRC6				
4	8E+06	4_7517731	223	35	0	0	129	0	1.39E-33	#######	0	0	0	0	0	0	1	1	32.25	78.0416555	7E+06	7549662	ID=ENSP0000250173;LRRC6;NM_012472;	LRRC6				
4	8E+06	4_7534658	38	15	6	24	27	15	0.059587	1	20	0	0	0	10	0	0.00970398	0.3824351	12.875	13.9123532	7E+06	7549662	ID=ENSP0000250173;LRRC6;NM_012472;	LRRC6				
4	8E+06	4_7636527	363	165	392	285	264	339	0.544139	1	1	20	75	3	11	39	1.30E-04	0.0113876	163	163.604925					7635829	7637061	0.15763	ID=promoter_35569;
4	8E+06	4_767626	559	273	463	443	416	453	0.549279	1	52	52	58	70	52	64	0.31355081	1	246.25	215.866195								
4	8E+06	4_783125	51	85	38	37	68	38	9.00E-04	0.05799	142	57	108	38	100	73	0.29806122	1	69.5	38.7630162								
4	8E+06	4_7901886	249	77	326	304	163	315	1.16E-06	0.00017	189	201	281	170	195	226	0.07277142	1	224.63	81.9005276	8E+06	7912375	ID=ENSP0000359244-D3;KCNQ2;;	KCNQ2				
4	8E+06	4_791329	36	64	26	20	50	23	5.99E-04	0.04108	82	68	40	36	75	38	0.00869927	0.3512743	46.5	22.0842542								
4	8E+06	4_798081	21	27	35	23	24	29	0.994218	1	0	36	35	11	18	23	0.59196476	1	23.5	12.8062485								
4	8E+06	4_799128	99	52	8	11	76	10	3.28E-14	#######	40	13	1	3	27	2	5.20E-05	0.0052176	28.375	33.8650157								
4	9E+06	4_864617	59	135	42	66	97	54	4.16E-05	0.00414	68	66	136	43	67	90	0.06912642	1	76.875	37.5554352								
4	9E+06	4_8666250	581	216	553	449	399	501	0.597189	1	258	144	170	71	201	121	7.85E-04	0.0517234	305.25	195.465561	9E+06	8896495	ID=ENSP0000286355;ADCY8;NM_001115;	ADCY8	8664534	8666856	0.57372	ID=promoter_35580;
4	9E+06	4_8879830	0	11	1	2	6	2	0.258649	1	22	0	5	1	11	3	0.13591373	1	5.25	7.70435869	9E+06	8896495	ID=ENSP0000286355;ADCY8;NM_001115;	ADCY8				
4	9E+06	4_895386	168	29	195	125	99	160	0.04206	1	51	59	51	7	55	29	0.04486397	1	85.625	68.4458858								
4	9E+06	4_896406	81	31	75	73	56	74	0.797171	1	71	17	41	34	44	38	0.95009183	1	52.875	24.718053								
4	9E+06	4_913448	29	31	18	11	30	15	0.021049	0.7339	5	36	41	5	21	23	0.85819415	1	22	14.1522335								
4	9E+06	4_915871	73	97	48	75	85	62	0.016216	0.59861	62	43	43	4	53	24	0.01197842	0.4547496	55.625	27.9588345								
4	9E+06	4_932908	0	0	11	0	0	6	0.077487	1	24	12	0	0	18	0	2.38E-04	0.0190698	5.875	8.98311909								
4	9E+06	4_938994	218	917	179	191	568	185	1.15E-57	#######	683	469	725	287	576	506	0.67694314	1	458.63	285.027286								
4	9E+06	4_940488	12	48	9	22	30	16	0.032973	1	28	38	42	58	33	50	0.08650441	1	32.125	17.3735225								
4	9E+06	4_9461539	51	18	36	51	35	44	0.954231	1	8	44	23	4	26	14	0.2525037	1	29.375	18.7611871	9E+06	9574063	ID=ENSP0000344591;ASAP1;;	ASAP1	9461047	9461853	0.13484	ID=promoter_35608;
4	1E+07	4_9538759	6	24	14	6	15	10	0.374041	1	6	45	51	0	26	26	0.96336814	1	19	19.3390796	9E+06	9574063	ID=ENSP0000344591;ASAP1;;	ASAP1				
4	1E+07	4_9560749	38	9	52	97	24	75	7.86E-05	0.00719	18	18	7	4	18	6	0.0767986	1	30.375	31.5546578	9E+06	9574063	ID=ENSP0000344591;ASAP1;;	ASAP1	9560330	9561278	0.16288	ID=promoter_35620;
4	1E+07	4_959402	40	91	15	7	66	11	2.48E-11	#######	42	19	41	50	31	46	0.12404634	1	38.125	26.2756677								
4	1E+07	4_963518	42	74	175	178	58	177	4.42E-10	#######	59	56	100	131	58	116	6.12E-06	0.0008251	101.88	53.8501559								
4	1E+07	4_9797795	32	10	22	13	21	18	0.61832	1	12	4	5	10	8	8	0.98858271	1	13.5	9.2890104					9797522	9799446	0.54572	ID=promoter_35628;
4	1E+07	4_9910283	4173	1238	2781	2119	2706	2450	1.51E-19	#######	2663	1278	1150	826	1971	988	1.78E-56	3.53E-53	2028.5	1132.74799					9909770	9910716	0.22408	ID=promoter_35632;
4	1E+07	4_9944496	674	140	481	248	407	365	9.35E-04	0.05976	60	154	154	22	107	88	0.70711476	1	241.63	223.98848					9944032	9945037	0.24748	ID=promoter_35635;
4	1E+07	4_10026897	7	19	2	18	13	10	0.600182	1	0	10	1	0	5	1	0.30486099	1	7.125	7.86379588					1E+07	1E+07	0.11597	ID=promoter_35643;
4	1E+07	4_100703	1	25	15	19	13	17	0.957342	1	2	21	40	11	12	26	0.04325208	1	16.75	12.7251158								
4	1E+07	4_101949	63	24	46	62	44	54	0.975459	1	22	48	31	100	35	66	0.00244904	0.1293678	49.5	25.7792829								
4	1E+07	4_105366	16	33	8	25	25	17	0.214068	1	58	38	50	11	48	31	0.28825568	1	29.875	18.2164565								
4	1E+07	4_106839	0	13	4	0	7	2	0.160905	1	0	31	18	25	16	22	0.48233468	1	11.375	12.3049061								
4	1E+07	4_111324	31	109	37	16	70	27	1.60E-06	0.00023	52	27	71	54	40	63	0.02967119	0.9232309	49.625	29.6548599								

4	1E+07	4_114217	83	19	42	71	51	57	0.969085	1	39	10	2	2	25	2	1.29E-04	0.0113348	33.5	30.9515751								
4	1E+07	4_11532484	40	7	5	23	24	14	0.105787	1	31	9	24	3	20	14	0.72101732	1	17.75	13.6774058					1.2E+07	1.2E+07	0.15333	ID=promoter_35664;
4	1E+07	4_115690	77	0	80	67	39	74	0.049214	1	0	24	23	0	12	12	0.98292303	1	33.875	35.3409415								
4	1E+07	4_115826	0	53	15	7	27	11	0.00829	0.35136	79	40	32	24	60	28	0.00931194	0.3713925	31.25	25.9436202								
4	1E+07	4_115922	16	44	67	68	30	68	0.009252	0.38259	58	17	46	5	38	26	0.48792921	1	40.125	24.5498763								
4	1E+07	4_11604210	919	355	765	668	637	717	0.750815	1	411	383	249	103	397	176	8.41E-16	4.48E-13	481.63	276.341888					1.2E+07	1.2E+07	0.13205	ID=promoter_35675;
4	1E+07	4_116410	16	4	14	21	10	18	0.549586	1	24	33	19	2	29	11	0.03330399	1	16.625	10.1971635								
4	1E+07	4_116485	6	18	6	17	12	12	0.926346	1	2	1	0	1	2	1	0.87709017	1	6.375	7.22965125								
4	1E+07	4_118199	42	10	42	32	26	37	0.751146	1	16	27	15	1	22	8	0.06315916	1	23.125	15.0469503								
4	1E+07	4_125988	35	42	22	24	39	23	0.028526	0.93503	44	28	88	15	36	52	0.11926525	1	37.25	22.7956763								
4	1E+07	4_138304	0	6	3	0	3	2	0.821455	1	13	9	13	0	11	7	0.73613127	1	5.5	5.63154381								
4	1E+07	4_139355	8	40	21	24	24	23	0.786129	1	3	33	50	44	18	47	4.88E-04	0.0348216	27.875	16.8983304								
4	1E+07	4_140973	4	40	9	3	22	6	0.002551	0.13706	1	31	15	29	16	22	0.48233468	1	16.5	14.9092493								
4	1E+07	4_141056	40	34	17	40	37	29	0.26207	1	38	31	44	53	35	49	0.17214544	1	37.125	10.4804239								
4	1E+07	4_142366	34	16	15	3	25	9	0.005635	0.25889	51	27	61	27	39	44	0.67035096	1	29.25	19.2037943								
4	1E+07	4_14630323	411	92	378	295	252	337	0.288988	1	100	101	148	0	101	74	0.30489802	1	190.63	150.568387					1.5E+07	1.5E+07	0.13068	ID=promoter_35715;
4	2E+07	4_15335241	8	39	10	9	24	10	0.016074	0.59429	11	16	56	36	14	46	5.53E-05	0.0055177	23.125	18.1142209	2E+07	1.5E+07	ID=ENSP00000262219;ANXA13;NM_001003954;	ANXA13				
4	2E+07	4_155728	93	24	58	29	59	44	0.072146	1	0	0	6	0	0	3	0.19828566	1	26.25	33.7077778								
4	2E+07	4_157777	45	58	36	31	52	34	0.027006	0.89569	9	79	36	54	44	45	0.89815416	1	43.5	20.8463769								
4	2E+07	4_158864	0	22	7	1	11	4	0.10516	1	0	53	41	48	27	45	0.0504211	1	21.5	22.7721635								
4	2E+07	4_158924	27	62	49	58	45	54	0.993642	1	1	43	44	1	22	23	0.92188352	1	35.625	23.8203844								
4	2E+07	4_161750	0	12	14	0	6	7	0.999953	1	0	5	19	0	3	10	0.11508955	1	6.25	7.68579395								
4	2E+07	4_162722	127	61	26	52	94	39	8.55E-08	#######	0	0	0	1	0	1	0.58312783	1	33.375	45.2293757								
4	2E+07	4_16332748	662	331	513	473	497	493	0.030671	0.98498	332	200	266	26	266	146	1.77E-06	0.0002793	350.38	197.938618					1.6E+07	1.6E+07	0.52516	ID=promoter_35758;
4	2E+07	4_163908	34	17	19	47	26	33	0.955738	1	0	0	1	0	0	1	0.58312783	1	14.75	18.0297373								
4	2E+07	4_164495	57	62	69	86	60	78	0.835698	1	22	10	25	18	16	22	0.48233468	1	43.625	28.1776887								
4	2E+07	4_164964	11	78	11	30	45	21	0.001467	0.08694	0	53	14	0	27	7	0.00575936	0.2548307	24.625	27.7227988								
4	2E+07	4_17179066	921	398	919	829	660	874	0.062629	1	341	360	388	74	351	231	2.59E-04	0.0203191	528.75	317.070407					1.7E+07	1.7E+07	0.37679	ID=promoter_35761;
4	2E+07	4_174931	95	86	69	69	91	69	0.022496	0.77278	0	24	16	28	12	22	0.15316566	1	48.375	35.5324299								
4	2E+07	4_178941	6	0	0	0	3	0	0.172143	1	23	0	11	1	12	6	0.45526626	1	5.125	8.25378701								
4	2E+07	4_187977	21	24	14	0	23	7	0.003444	0.1741	1	9	14	0	5	7	0.77775394	1	10.375	9.48589178								
4	2E+07	4_18910763	9	13	20	51	11	36	0.007918	0.3393	47	18	44	6	33	25	0.74763976	1	26	18.3147092	2E+07	1.9E+07	ID=ENSP00000367406;TAF2;NM_003184;	TAF2				
4	2E+07	4_19035268	0	15	0	5	8	3	0.206144	1	16	5	13	0	11	7	0.73613127	1	6.75	6.9230464	2E+07	1.9E+07	ID=ENSP00000259486;ENPP2;NM_006209;	ENPP2				
4	2E+07	4_193350	14	30	37	50	22	44	0.117866	1	13	3	16	21	8	19	0.06853691	1	23	15.1563282								
4	2E+07	4_19345868	24	48	2	9	36	6	1.64E-06	0.00024	27	2	12	1	15	7	0.31064188	1	15.625	16.4311333	2E+07	1.9E+07	ID=ENSP00000276681;MAL2;NM_052886;	MAL2				
4	2E+07	4_193490	24	52	30	23	38	27	0.13199	1	34	11	27	5	23	16	0.67590535	1	25.75	14.3203152								
4	2E+07	4_197482	35	2	21	7	19	14	0.411928	1	0	14	9	0	7	5	0.9051169	1	11	12.1184629								
4	2E+07	4_200092	1	12	5	0	7	3	0.314307	1	25	0	0	0	13	0	0.00241566	0.1280305	5.375	8.97516414								
4	2E+07	4_200866	1533	1128	1587	1474	1331	1531	0.872967	1	816	526	411	95	671	253	2.98E-35	3.80E-32	946.25	569.596787								
4	2E+07	4_200977	8	76	8	8	42	8	4.96E-07	#######	27	13	21	18	20	20	0.97170071	1	22.375	22.7592211								
4	2E+07	4_201147	4	5	22	32	5	27	0.002605	0.1394	42	9	35	25	26	30	0.71540497	1	21.75	14.4395291								
4	2E+07	4_202525	157	91	228	60	124	144	0.996678	1	49	61	115	108	55	112	6.01E-06	0.0008111	108.63	59.9164597								
4	2E+07	4_203684	97	69	195	112	83	154	0.003116	0.16069	36	27	99	9	32	54	0.02438444	0.7977568	80.5	59.5267047								
4	2E+07	4_204445	1	31	7	0	16	4	0.009581	0.39335	20	33	11	1	27	6	0.00268813	0.1381379	13	13.4695424								
4	2E+07	4_204502	311	182	218	205	247	212	0.003662	0.18279	77	72	51	69	75	60	0.69578479	1	148.13	94.3707544								
4	2E+07	4_204934	20	38	10	1	29	6	7.05E-05	0.00656	52	8	35	19	30	27	0.99379101	1	22.875	17.3735225								
4	2E+07	4_205551	127	55	101	52	91	77	0.106031	1	107	57	47	1	82	24	1.05E-06	0.0001733	68.375	40.6480978								
4	2E+07	4_206488	26	46	50	19	36	35	0.730868	1	54	47	60	23	51	42	0.85075386	1	40.625	15.6016254								
4	2E+07	4_216617	24	1	24	7	13	16	0.991731	1	1	5	48	29	3	39	4.83E-08	1.06E-05	17.375	16.7241271								
4	2E+07	4_219008	3673	1925	3730	2793	2799	3262	0.969178	1	532	226	379	264	379	322	0.51542704	1	1690.3	1540.04543								
4	2E+07	4_220168	19	41	6	17	30	12	0.004264	0.20626	133	4	1	1	69	1	5.41E-14	2.58E-11	27.75	44.5829884								

4	2E+07	4_220303	598	559	335	554	579	445	1.23E-10	#######	94	118	108	110	106	109	0.74900985	1	309.5	229.729033								
4	2E+07	4_224623	66	132	108	123	99	116	0.99997	1	167	176	91	43	172	67	4.20E-09	1.11E-06	113.25	46.23465								
4	2E+07	4_224999	57	114	51	67	86	59	0.005772	0.26393	194	144	126	127	169	127	0.20107533	1	110	49.1528229								
4	2E+07	4_225191	14	0	5	5	7	5	0.693374	1	15	25	6	0	20	3	0.00346999	0.1723921	8.75	8.61477137								
4	2E+07	4_226838	15	22	10	28	19	19	0.885913	1	0	2	31	13	1	22	3.00E-05	0.0032574	15.125	11.3192314								
4	2E+07	4_22798654	955	316	874	688	636	781	0.691742	1	467	277	307	202	372	255	9.62E-04	0.0605631	510.75	290.823339					2.3E+07	2.3E+07	0.29596	ID=promoter_35785;
4	2E+07	4_22887867	18	16	77	11	17	44	0.017506	0.63424	0	0	32	20	0	26	8.13E-07	0.0001389	21.75	24.696732	2E+07	2.3E+07	ID=ENSP00000379065;TRPS1;NM_014112;	TRPS1				
4	2E+07	4_231988	85	81	82	42	83	62	0.025356	0.85286	52	4	50	28	28	39	0.25425581	1	53	28.7998016								
4	3E+07	4_255369	5	42	19	41	24	30	0.97337	1	53	29	29	86	41	58	0.11335044	1	38	24.3955499								
4	3E+07	4_260488	1	0	26	10	1	18	0.001742	0.10079	22	44	29	0	33	15	0.0650987	1	16.5	16.3007449								
4	3E+07	4_265360	34	50	44	63	42	54	0.905206	1	108	91	182	61	100	122	0.12429748	1	79.125	48.2032527								
4	3E+07	4_283302	14	23	5	0	19	3	7.41E-04	0.04898	19	0	0	3	10	2	0.09294023	1	8	9.31971796								
4	3E+07	4_283831	0	23	2	4	12	3	0.030625	0.98374	27	17	28	2	22	15	0.65424549	1	12.875	12.1236192								
4	3E+07	4_299717	37	12	16	23	25	20	0.43889	1	92	48	36	18	70	27	3.41E-04	0.0258297	35.25	26.0370615								
4	3E+07	4_306678	39	71	50	17	55	34	0.011572	0.45508	59	101	60	65	80	63	0.62497735	1	57.75	24.429198								
4	3E+07	4_311520	309	87	915	844	198	880	8.77E-73	#######	140	160	183	153	150	168	0.24151303	1	348.88	334.022855								
4	3E+07	4_312301	2	0	5	6	1	6	0.241978	1	6	0	21	0	3	11	0.0740992	1	5	6.98978847								
4	3E+07	4_33768763	9	75	43	36	42	40	0.640089	1	90	35	167	30	63	99	0.00414069	0.1960929	60.625	50.0883149	3E+07	3.4E+07	ID=ENSP00002622 31;RIMS2;;	RIMS2				
4	3E+07	4_33984277	9364	9019	13406	12020	9192	####	3.02E-32	#######	8756	6308	13824	3542	7532	8683	1.69E-42	2.71E-39	9529.9	3521.10155					3.4E+07	3.4E+07	0.73478	ID=promoter_35819;
4	3E+07	4_342544	10	8	13	5	9	9	0.944235	1	0	4	8	19	2	14	0.00692302	0.294895	8.375	5.82942046								
4	3E+07	4_347744	21	27	29	13	24	21	0.616071	1	16	33	16	55	25	36	0.23362127	1	26.25	13.5935909								
4	3E+07	4_347747	44	12	37	41	28	39	0.785678	1	0	0	0	0	0	0	1	1	16.75	20.303061								
4	3E+07	4_348599	5	14	4	2	10	3	0.082185	1	0	12	25	13	6	19	0.02032977	0.6899793	9.375	8.24512843								
4	3E+07	4_34932506	15	12	31	12	14	22	0.691767	1	5	5	8	4	5	6	0.91271846	1	11.5	8.83176087					3.5E+07	3.5E+07	0.73659	ID=promoter_35833;
4	4E+07	4_351809	18	40	7	19	29	13	0.011304	0.44898	5	43	28	42	24	35	0.22621095	1	25.25	15.3785565								
4	4E+07	4_353440	20	21	13	25	21	19	0.713172	1	42	1	20	2	22	11	0.23631249	1	18	13.1583759								
4	4E+07	4_354775	29	14	1	0	22	1	1.12E-05	0.00132	1	0	12	3	1	8	0.04988758	1	7.5	10.2956301								
4	4E+07	4_364127	9	0	11	1	5	6	0.999292	1	0	30	15	0	15	8	0.44137572	1	8.25	10.57963								
4	4E+07	4_365065	9	46	14	7	28	11	0.005436	0.25174	20	26	42	22	23	32	0.32689554	1	23.25	14.3502115								
4	4E+07	4_366414	14	13	8	11	14	10	0.480768	1	0	6	21	0	3	11	0.0740992	1	9.125	7.18008158								
4	4E+07	4_368322	13	16	9	3	15	6	0.065303	1	0	7	24	12	4	18	0.00668749	0.2868305	10.5	7.57816412								
4	4E+07	4_369137	102	35	64	60	69	62	0.311447	1	19	8	6	19	14	13	0.99999086	1	39.125	33.5960776								
4	4E+07	4_37294771	58	17	36	53	38	45	0.999061	1	22	11	28	31	17	30	0.09758228	1	32	16.5443128					3.7E+07	3.7E+07	0.41364	ID=promoter_35864;
4	4E+07	4_38291937	3	77	13	23	40	18	0.002142	0.11834	18	48	32	53	33	43	0.33537098	1	33.375	24.4536734	4E+07	3.8E+07	ID=ENSP00003470 09;STK3;;	STK3				
4	4E+07	4_388873	4	3	9	20	4	15	0.092184	1	10	12	24	70	11	47	3.39E-06	0.0004904	19	21.8370589								
4	4E+07	4_38906189	125	115	181	47	120	114	0.271453	1	160	128	94	81	144	88	0.00813426	0.3342859	116.38	42.8550297					3.9E+07	3.9E+07	0.27693	ID=promoter_35883;
4	4E+07	4_38981513	143	48	108	118	96	113	0.99968	1	46	0	4	5	23	5	0.00590668	0.2600594	59	56.8833393	4E+07	3.9E+07	ID=ENSP00002548 98;MATN2;NM_002380;	MATN2				
4	4E+07	4_395016	10	36	6	2	23	4	2.40E-04	0.01893	2	0	9	2	1	6	0.13767373	1	8.375	11.7344122								
4	4E+07	4_399096	14	53	6	0	34	3	1.56E-07	#######	1	1	26	1	1	14	0.00214405	0.1161796	12.75	18.5914727								
4	4E+07	4_401406	0	19	0	1	10	1	0.011548	0.45484	24	8	2	1	16	2	0.00719486	0.3031284	6.875	9.47835881								
4	4E+07	4_404437	7	21	25	22	14	24	0.52503	1	50	0	9	0	25	5	0.00263335	0.136745	16.75	16.6282891								
4	4E+07	4_406999	463	216	442	254	340	348	0.202058	1	235	261	159	88	248	124	8.66E-08	1.83E-05	264.75	129.026852								
4	4E+07	4_408583	7	38	2	4	23	3	8.06E-05	0.00734	22	11	0	0	17	0	3.78E-04	0.0279647	10.5	13.2880182								
4	4E+07	4_410118	23	51	7	11	37	9	1.73E-05	0.00192	3	29	69	3	16	36	0.0095878	0.3813297	24.5	24.2546019								
4	4E+07	4_410618	44	21	26	16	33	21	0.084147	1	37	16	7	1	27	4	4.55E-04	0.0327286	21	14.4222051								
4	4E+07	4_411347	592	348	399	340	470	370	5.71E-08	#######	164	213	266	49	189	158	0.63101038	1	296.38	164.354093								
4	4E+07	4_419734	20	7	3	2	14	3	0.010924	0.43763	11	4	6	2	8	4	0.59180275	1	6.875	6.10474055								
4	4E+07	4_420827	21	10	26	1	16	14	0.724026	1	53	6	25	0	30	13	0.06382438	1	17.75	17.5966718								
4	4E+07	4_422445	30	31	25	19	31	22	0.19035	1	51	0	19	3	26	11	0.081859	1	22.25	16.2897865								
4	4E+07	4_425648	13	18	20	8	16	14	0.724026	1	86	49	57	26	68	42	0.11413963	1	34.625	26.9599571								
4	4E+07	4_42699194	2110	801	1725	1102	1456	1414	2.70E-06	0.00037	984	696	773	555	840	664	0.00892159	0.3589328	1093.3	545.159413					4.3E+07	4.3E+07	0.44861	ID=promoter_35915;

4	4E+07	4_427976	27	96	23	58	62	41	0.015531	0.57901	111	12	158	87	62	123	3.66E-06	0.0005231	71.5	50.6218473								
4	4E+07	4_437182	14	2	2	11	8	7	0.850897	1	80	24	14	36	52	25	0.02300235	0.7590344	22.875	25.6539471								
4	4E+07	4_443216	0	14	1	11	7	6	0.851931	1	1	2	11	0	2	6	0.31385293	1	5	5.90399381								
4	4E+07	4_443344	51	73	32	12	62	22	2.14E-06	0.0003	28	107	90	5	68	48	0.34938015	1	49.75	37.1396977								
4	4E+07	4_449358	25	36	0	1	31	1	5.83E-08	#######	42	34	26	25	38	26	0.48792921	1	23.625	15.4821326								
4	5E+07	4_450445	10	0	15	10	5	13	0.299488	1	78	25	36	3	52	20	0.00258053	0.1348666	22.125	25.418989								
4	5E+07	4_47205670	253	551	330	226	402	278	6.36E-11	#######	291	328	365	251	310	308	0.69008277	1	324.38	103.042207					4.7E+07	4.7E+07	0.99999	ID=promoter_35945;
4	5E+07	4_479939	9	31	8	15	20	12	0.173914	1	3	0	8	1	2	5	0.46647814	1	9.375	10.0133839								
4	5E+07	4_495800	30	42	5	12	36	9	2.87E-05	0.00298	0	4	33	2	2	18	8.85E-04	0.0572814	16	16.4490556								
4	5E+07	4_520429	9	46	0	5	28	3	4.80E-06	0.00062	33	1	6	21	17	14	0.9475482	1	15.125	16.7283976								
4	5E+07	4_527778	31	50	26	13	41	20	0.004028	0.19746	41	56	61	17	49	39	0.77645318	1	36.875	17.9478411								
4	6E+07	4_568112	0	40	12	0	20	6	0.006754	0.29841	0	10	17	0	5	9	0.48000906	1	9.875	13.902081								
4	6E+07	4_57418338	2	9	1	1	6	1	0.108618	1	22	0	5	1	11	3	0.13591373	1	5.125	7.43423548					5.7E+07	5.7E+07	0.75083	ID=promoter_35980;
4	6E+07	4_579554	15	20	30	10	18	20	0.986196	1	4	32	8	17	18	13	0.78939737	1	17	10.0427657								
4	6E+07	4_579692	2547	2123	5271	5415	2335	5343	########	#######	1974	1381	3589	1504	1678	2547	3.21E-55	6.19E-52	2975.5	1613.24766								
4	6E+07	4_579866	73	62	28	59	68	44	0.007694	0.3309	96	14	88	55	55	72	0.15333907	1	59.375	27.8153991								
4	6E+07	4_580411	38	73	40	62	56	51	0.423514	1	164	70	100	37	117	69	0.01093178	0.4226567	73	42.6781978								
4	6E+07	4_586152	251	171	201	168	211	185	0.014936	0.56136	1	4	0	3	3	2	0.9363008	1	99.875	107.632228								
4	6E+07	4_58930646	619	412	670	361	516	516	0.037261	1	258	262	288	147	260	218	0.54962199	1	377.13	182.874225					5.9E+07	5.9E+07	0.70819	ID=promoter_35998;
4	6E+07	4_59046743	1	11	7	0	6	4	0.674789	1	0	22	14	0	11	7	0.73613127	1	6.875	8.21909797	6E+07	5.9E+07	ID=ENSP0000220876;STMN2;NM_007029;	STMN2				
4	6E+07	4_595990	0	17	0	0	9	0	0.005101	0.2381	0	29	12	20	15	16	0.92652531	1	9.75	11.4236597								
4	6E+07	4_601796	0	13	0	0	7	0	0.016484	0.60584	78	8	7	0	43	4	3.89E-07	7.19E-05	13.25	26.6230298								
4	6E+07	4_603966	1	33	4	5	17	5	0.013234	0.50759	71	28	22	2	50	12	3.48E-05	0.0037136	20.75	23.9030781								
4	6E+07	4_606890	5	41	0	5	23	3	8.06E-05	0.00734	1	57	21	4	29	13	0.08411918	1	16.75	21.3591733								
4	6E+07	4_615787	4	1	9	0	3	5	0.88968	1	2	14	0	13	8	7	0.9937864	1	5.375	5.80486249								
4	6E+07	4_615956	2	28	8	29	15	19	0.975531	1	33	24	46	1	29	24	0.91882689	1	21.375	16.1239618								
4	6E+07	4_617912	35	77	29	15	56	22	2.95E-05	0.00306	57	50	22	1	54	12	7.23E-06	0.0009506	35.75	24.5807706								
4	6E+07	4_630559	6	13	5	0	10	3	0.082185	1	0	28	2	0	14	1	0.00574567	0.2548307	6.75	9.66215001								
4	6E+07	4_647004	6	17	2	5	12	4	0.066899	1	3	0	6	18	2	12	0.01895406	0.6519669	7.125	6.7281392								
4	6E+07	4_647682	152	108	222	129	130	176	0.462846	1	20	11	18	19	16	19	0.76520201	1	84.875	79.5243673								
4	7E+07	4_650157	67	33	69	92	50	81	0.196758	1	96	19	10	45	58	28	0.01553926	0.5562856	53.875	32.2598269								
4	7E+07	4_652345	68	60	126	67	64	97	0.279868	1	0	9	16	0	5	8	0.62517663	1	43.25	44.6598253								
4	7E+07	4_653573	7	2	13	15	5	14	0.226542	1	7	11	82	3	9	43	3.89E-06	0.0005505	17.5	26.4575131								
4	7E+07	4_656112	17	11	17	14	14	16	0.99748	1	0	0	9	1	0	5	0.06742467	1	8.625	7.38603895								
4	7E+07	4_65669220	5	28	8	20	17	14	0.615566	1	13	17	96	0	15	48	4.77E-05	0.0048963	23.375	30.6498077	7E+07	6.6E+07	ID=ENSP0000262209;TRPA1;NM_007332;	TRPA1				
4	7E+07	4_659014	40	20	15	8	30	12	0.004264	0.20626	22	12	0	5	17	3	0.01239331	0.4669004	15.25	12.4298029								
4	7E+07	4_659734	58	28	26	33	43	30	0.087253	1	152	57	58	15	105	37	9.87E-07	0.0001643	53.375	43.2003224								
4	7E+07	4_66101898	1274	665	1369	1259	970	1314	0.003038	0.15738	2015	1441	1449	846	1728	1148	1.65E-17	9.98E-15	1289.8	408.643313					6.6E+07	6.6E+07	0.49277	ID=promoter_36054;
4	7E+07	4_66104770	85	16	53	66	51	60	0.999876	1	86	63	70	69	75	70	0.99917266	1	63.5	22.0842542					6.6E+07	6.6E+07	0.2401	ID=promoter_36055;
4	7E+07	4_664981	142	56	102	103	99	103	0.695319	1	0	1	0	1	1	1	0.99856566	1	50.625	58.3240639								
4	7E+07	4_669157	102	62	24	19	82	22	1.60E-10	#######	34	28	49	43	31	46	0.12404634	1	45.125	26.9520076								
4	7E+07	4_670631	0	15	3	18	8	11	0.943069	1	0	32	17	2	16	10	0.61601721	1	10.875	11.5193936								
4	7E+07	4_672270	0	0	3	2	0	3	0.278365	1	4	18	20	0	11	10	0.99899888	1	5.875	8.25378701								
4	7E+07	4_672794	6	11	25	19	9	22	0.165185	1	15	9	0	2	12	1	0.01422424	0.5202299	10.875	8.50944853								
4	7E+07	4_672909	1	8	2	3	5	3	0.645778	1	43	7	9	2	25	6	0.00589984	0.2600594	9.375	13.9277472								
4	7E+07	4_67390301	317	148	200	209	233	205	0.010676	0.43305	38	68	57	2	53	30	0.09265438	1	129.88	107.207992	7E+07	6.7E+07	ID=ENSP0000267974;NCOA2;;	NCOA2				
4	7E+07	4_67451735	1033	582	944	1246	808	1095	0.007773	0.33377	581	575	575	280	578	428	0.00196174	0.1078562	727	315.876106					6.7E+07	6.7E+07	0.77395	ID=promoter_36065;
4	7E+07	4_677084	0	5	0	3	3	2	0.821455	1	40	0	9	0	20	5	0.01914528	0.6572777	7.125	13.6741414								
4	7E+07	4_679183	9	24	4	0	17	2	7.31E-04	0.04855	24	23	9	0	24	5	0.00395202	0.1881563	11.625	10.5415843								
4	7E+07	4_680003	21	12	24	24	17	24	0.842518	1	0	3	17	31	2	24	3.82E-05	0.0040135	16.5	10.8100482								
4	7E+07	4_688068	270	111	242	255	191	249	0.546704	1	177	113	146	49	145	98	0.05264122	1	170.38	79.6455317								
4	7E+07	4_689666	7	50	14	12	29	13	0.011304	0.44898	42	0	11	3	21	7	0.05007015	1	17.375	18.3920596								
4	7E+07	4_690825	47	16	30	39	32	35	0.959954	1	32	20	34	22	26	28	0.85926991	1	30	10.3509834								

4	7E+07	4_69315721	21	37	10	7	29	9	8.94E-04	0.05763	60	44	19	34	52	27	0.04641028	1	29	18.0633805	7E+07	6.9E+07	ID=ENSP0000379793;;					
4	7E+07	4_696160	6	39	21	18	23	20	0.617116	1	5	40	13	2	23	8	0.04560275	1	18	14.7744954								
4	7E+07	4_699222	28	13	32	28	21	30	0.785963	1	0	4	7	0	2	4	0.6610374	1	14	13.4057557								
4	7E+07	4_706229	654	1982	677	899	1318	788	3.16E-51	#######	1441	1182	2129	758	1312	1444	3.91E-05	0.004102	1215.3	584.162343								
4	7E+07	4_707911	562	278	694	647	420	671	4.00E-06	0.00053	57	38	195	51	48	123	3.62E-09	9.63E-07	315.25	278.678386								
4	7E+07	4_71131149	929	223	826	563	576	695	0.881662	1	216	243	259	86	230	173	0.11475424	1	418.13	314.771771					7.1E+07	7.1E+07	0.37358	ID=promoter_36102;
4	7E+07	4_712154	29	14	20	35	22	28	0.959756	1	95	1	32	57	48	45	0.99854017	1	35.375	29.1544532								
4	7E+07	4_714069	7	40	6	6	24	6	9.38E-04	0.05976	14	28	10	23	21	17	0.91718448	1	16.75	12.4527679								
4	7E+07	4_714484	96	36	65	39	66	52	0.096967	1	43	0	2	18	22	10	0.16182597	1	37.375	32.2044252								
4	7E+07	4_71549690	368	270	396	542	319	469	0.007716	0.33175	305	283	166	168	294	167	2.18E-06	0.0003351	312.25	124.095297					7.2E+07	7.2E+07	0.4753	ID=promoter_36105;
4	7E+07	4_719403	0	18	13	11	9	12	0.95859	1	0	10	4	35	5	20	0.00613894	0.2677261	11.375	11.4634512								
4	7E+07	4_722188	0	20	11	0	10	6	0.41703	1	12	1	8	1	7	5	0.9051169	1	6.625	7.36667205								
4	7E+07	4_722645	34	37	13	36	36	25	0.12572	1	24	11	25	23	18	24	0.50317852	1	25.375	9.92741514								
4	7E+07	4_724717	24	58	34	16	41	25	0.031944	1	58	8	39	57	33	48	0.13295864	1	36.75	19.8044007								
4	7E+07	4_728007	11	7	17	3	9	10	0.993074	1	19	28	20	11	24	16	0.5906404	1	14.5	8.03563492								
4	7E+07	4_729151	10	59	15	7	35	11	2.32E-04	0.01845	69	21	13	3	45	8	9.46E-06	0.0012039	24.625	25.0253443								
4	7E+07	4_731451	4	25	11	18	15	15	0.908797	1	26	45	6	4	36	5	2.52E-05	0.0028067	17.375	14.2622128								
4	7E+07	4_731799	10	9	2	16	10	9	0.84607	1	1	0	1	10	1	6	0.13767373	1	6.125	5.89036744								
4	7E+07	4_734534	16	159	57	28	88	43	7.50E-06	0.00093	127	42	83	68	85	76	0.9741013	1	72.5	49.1731634								
4	7E+07	4_73553917	801	308	567	392	555	480	5.89E-06	0.00075	218	269	256	54	244	155	0.00105695	0.0657578	358.13	231.123614					7.4E+07	7.4E+07	0.63199	ID=promoter_36122;
4	7E+07	4_740621	165	114	106	170	140	138	0.348663	1	30	18	28	25	24	27	0.78764771	1	82	64.5843856								
4	7E+07	4_743313	1	36	11	0	19	6	0.010861	0.43721	18	16	18	1	17	10	0.5154613	1	12.625	12.2350492								
4	7E+07	4_745671	8	26	6	18	17	12	0.395844	1	1	25	42	59	13	51	2.81E-06	0.0004184	23.125	19.6136796								
4	7E+07	4_749851	27	130	34	43	79	39	3.03E-05	0.00314	117	86	27	10	102	19	8.87E-12	3.31E-09	59.25	45.4556299								
4	8E+07	4_750029	27	0	10	13	14	12	0.725787	1	78	16	18	22	47	20	0.01141841	0.4373265	23	23.6462017								
4	8E+07	4_750970	110	85	77	84	98	81	0.064264	1	37	8	7	2	23	5	0.00590668	0.2600594	51.25	42.7342285								
4	8E+07	4_751157	292	89	221	303	191	262	0.257596	1	483	221	228	191	352	210	2.29E-06	0.0003501	253.5	113.59829								
4	8E+07	4_752003	33	9	40	25	21	33	0.575361	1	88	31	35	31	60	33	0.05140286	1	36.5	22.7658893								
4	8E+07	4_753120	7	17	18	14	12	16	0.945172	1	20	8	24	0	14	12	0.98032967	1	13.5	7.92824967								
4	8E+07	4_75473579	724	316	613	380	520	497	0.004767	0.22612	332	186	266	100	259	183	0.01870231	0.6456407	364.63	209.136276					7.5E+07	7.5E+07	0.75105	ID=promoter_36161;
4	8E+07	4_76630833	4317	4844	5391	6017	4581	5704	0.010753	0.4351	4917	3642	9777	2154	4280	5966	5.19E-92	1.61E-88	5132.4	2213.86243					7.7E+07	7.7E+07	0.71579	ID=promoter_36168;
4	8E+07	4_76871621	12	40	5	1	26	3	1.49E-05	0.00168	3	40	21	0	22	11	0.23631249	1	15.25	16.7310661	8E+07	7.7E+07	ID=ENSP0000354842;TOX;NM_014729;	TOX				
4	8E+07	4_769593	16	35	13	7	26	10	0.006911	0.3015	24	17	28	49	21	39	0.03240705	0.9910413	23.625	13.5429212								
4	8E+07	4_77104509	175	74	116	52	125	84	3.36E-04	0.02557	38	23	3	2	31	3	2.73E-05	0.0030031	60.375	59.8639827	8E+07	7.7E+07	ID=ENSP0000038176;NSMAF;NM_003580;	NSMAF				
4	8E+07	4_77117050	1	33	3	0	17	2	7.31E-04	0.04855	0	0	11	1	0	6	0.0393172	1	6.125	11.4696805	8E+07	7.7E+07	ID=ENSP0000038176;NSMAF;NM_003580;	NSMAF	7.7E+07	7.7E+07	0.17297	ID=promoter_36176;
4	8E+07	4_77117079	7	13	6	0	10	3	0.082185	1	1	19	10	0	10	5	0.51906437	1	7	6.80336051	8E+07	7.7E+07	ID=ENSP0000038176;NSMAF;NM_003580;	NSMAF	7.7E+07	7.7E+07	0.17297	ID=promoter_36176;
4	8E+07	4_771688	0	16	14	0	8	7	0.850897	1	33	9	5	2	21	4	0.00573189	0.2548307	9.875	11.1539551								
4	8E+07	4_773765	31	157	30	10	94	20	5.90E-14	#######	104	145	135	14	125	75	0.01103333	0.4260062	78.25	63.1183695								
4	8E+07	4_774567	44	95	126	164	70	145	3.93E-04	0.02898	66	40	50	67	53	59	0.62574856	1	81.5	43.9805152								
4	8E+07	4_77528011	154	195	94	95	175	95	4.60E-09	#######	159	103	87	180	131	134	0.7254721	1	133.38	43.3422839					7.8E+07	7.8E+07	0.12573	ID=promoter_36191;
4	8E+07	4_776542	40	32	15	6	36	11	1.44E-04	0.0122	9	36	30	4	23	17	0.77734386	1	21.5	14.5405836								
4	8E+07	4_776925	233	169	164	243	201	204	0.345786	1	130	63	24	20	97	22	8.29E-10	2.46E-07	130.75	87.7296985								
4	8E+07	4_780737	99	90	121	83	95	102	0.824941	1	1	13	0	0	7	0	0.03898208	1	50.875	51.9544787								
4	8E+07	4_781449	5	19	35	27	12	31	0.05845	1	0	0	11	1	0	6	0.0393172	1	12.25	13.4243914								
4	8E+07	4_783614	189	74	211	212	132	212	0.01742	0.6315	263	107	149	117	185	133	0.0809116	1	165.25	64.041839								
4	8E+07	4_783805	11	62	27	23	37	25	0.097534	1	66	19	63	104	43	84	2.55E-04	0.0200383	46.875	31.9528112								
4	8E+07	4_783990	2	0	2	29	1	16	0.004036	0.19749	11	35	13	20	23	17	0.77734386	1	14	13.0712334								

4	8E+07	4_78420971	23	29	160	225	26	193	4.75E-23	#######	0	30	54	9	15	32	0.02335039	0.7680341	66.25	81.3980519					7.8E+07	7.8E+07	0.67493	ID=promoter_36204;
4	8E+07	4_785370	25	4	5	22	15	14	0.827043	1	0	0	0	1	0	1	0.58312783	1	7.125	10.3155569								
4	8E+07	4_787910	8	31	6	6	20	6	0.006754	0.29841	55	56	35	1	56	18	2.29E-04	0.0185569	24.75	22.6258386								
4	8E+07	4_788087	273	104	202	356	189	279	0.050198	1	446	50	140	163	248	152	2.79E-04	0.0217092	216.75	133.430292								
4	8E+07	4_790283	40	14	10	21	27	16	0.087673	1	38	22	47	57	30	52	0.02131376	0.7194421	31.125	16.7880697								
4	8E+07	4_790287	10	30	13	23	20	18	0.715835	1	4	12	5	3	8	4	0.59180275	1	12.5	9.57675758								
4	8E+07	4_790469	42	29	33	28	36	31	0.4485	1	0	0	23	12	0	18	6.08E-05	0.0059624	20.875	15.422039								
4	8E+07	4_79062270	35	27	15	37	31	26	0.448024	1	77	46	55	52	62	54	0.94534839	1	43	19.0112749					7.9E+07	7.9E+07	0.17337	ID=promoter_36233;
4	8E+07	4_79129963	99	27	47	100	63	74	0.999966	1	29	1	12	1	15	7	0.31064188	1	39.5	40.0784944					7.9E+07	7.9E+07	0.09361	ID=promoter_36234;
4	8E+07	4_791336	12	0	4	11	6	8	0.9722	1	15	2	7	1	9	4	0.4571786	1	6.5	5.63154381								
4	8E+07	4_79203669	11	15	3	22	13	13	0.920459	1	41	62	62	3	52	33	0.23056449	1	27.375	24.5586034					7.9E+07	7.9E+07	0.12061	ID=promoter_36236;
4	8E+07	4_792569	84	13	57	77	49	67	0.716473	1	31	0	34	22	16	28	0.11940283	1	39.75	30.1508114								
4	8E+07	4_795373	29	30	10	13	30	12	0.004264	0.20626	50	28	62	44	39	53	0.18946288	1	33.25	17.8465683								
4	8E+07	4_79611368	37	41	36	10	39	23	0.028526	0.93503	70	6	53	15	38	34	0.98877343	1	33.5	22.1617431	8E+07	8E+07	ID=ENSP00000220676;RP1;NM_006269;	RP1	8E+07	8E+07	0.39894	ID=promoter_36246;
4	8E+07	4_79618901	124	42	34	42	83	38	4.07E-06	0.00054	12	38	12	12	25	12	0.16190573	1	39.5	36.7501215					8E+07	8E+07	0.09723	ID=promoter_36247;
4	8E+07	4_797415	18	0	9	13	9	11	0.995818	1	0	0	0	0	0	0	1	1	5	7.30948503								
4	8E+07	4_798212	22	14	27	20	18	24	0.918896	1	14	39	34	1	27	18	0.55301708	1	21.375	12.1177261								
4	8E+07	4_800575	1	8	4	0	5	2	0.402696	1	20	25	1	8	23	5	0.00590668	0.2600594	8.375	9.33407735								
4	8E+07	4_80210844	337	281	330	261	309	296	0.044579	1	108	137	175	57	123	116	0.99124837	1	210.75	105.920118					8E+07	8E+07	0.59224	ID=promoter_36273;
4	8E+07	4_80288396	648	238	484	433	443	459	0.176122	1	237	145	139	129	191	134	0.04605975	1	306.63	192.464422	8E+07	8E+07	ID=ENSP00000297313;RGS20;NM_170587;	RGS20	8E+07	8E+07	0.44811	ID=promoter_36279;
4	8E+07	4_80312370	7996	6001	9106	13735	6999	####	########	#######	8209	3891	19520	4701	6050	12111	0	0	9144.9	5184.38495					8E+07	8E+07	0.47231	ID=promoter_36280;
4	8E+07	4_80316379	17	33	9	15	25	12	0.031505	1	2	30	40	49	16	45	3.20E-04	0.0244334	24.375	16.1946861	8E+07	8E+07	ID=ENSP00000379995;ATP6V1H;NM_213620;	ATP6V1H				
4	8E+07	4_80358986	0	2	10	5	1	8	0.109969	1	20	0	6	0	10	3	0.19568363	1	5.375	6.9062601	8E+07	8E+07	ID=ENSP00000379995;ATP6V1H;NM_213620;	ATP6V1H				
4	8E+07	4_804884	34	53	59	11	44	35	0.225671	1	3	19	23	0	11	12	0.92663959	1	25.25	21.9268264								
4	8E+07	4_807192	19	41	12	25	30	19	0.102296	1	35	0	20	60	18	40	0.00638295	0.2759882	26.5	18.5857088								
4	8E+07	4_80737712	460	303	469	359	382	414	0.537108	1	212	258	246	108	235	177	0.11246772	1	301.88	123.583907					8.1E+07	8.1E+07	0.28317	ID=promoter_36291;
4	8E+07	4_807442	2	93	23	11	48	17	3.99E-05	0.00399	36	56	51	27	46	39	0.91919411	1	37.375	29.0070804								
4	8E+07	4_808007	19	1	11	28	10	20	0.378359	1	17	18	5	21	18	13	0.78939737	1	15	8.83176087								
4	8E+07	4_808407	35	0	25	15	18	20	0.986196	1	77	37	24	40	57	32	0.07191194	1	31.625	22.5130914								
4	8E+07	4_80878232	10	9	5	4	10	5	0.276106	1	0	10	27	0	5	14	0.08295177	1	8.125	8.64270956					8.1E+07	8.1E+07	0.16039	ID=promoter_36297;
4	8E+07	4_80881074	30	34	22	15	32	19	0.056624	1	3	25	44	0	14	22	0.29543416	1	21.625	15.0896133					8.1E+07	8.1E+07	0.13423	ID=promoter_36298;
4	8E+07	4_81036345	98	43	115	101	71	108	0.233338	1	74	93	175	26	84	101	0.21024049	1	90.625	45.6725535					8.1E+07	8.1E+07	0.40209	ID=promoter_36304;
4	8E+07	4_811907	56	122	11	68	89	40	1.12E-06	0.00017	58	133	35	14	96	25	1.18E-08	2.88E-06	62.125	45.2972326								
4	8E+07	4_813218	4	0	3	6	2	5	0.651182	1	19	8	0	26	14	13	0.99999086	1	8.25	9.39224604								
4	8E+07	4_817299	19	57	23	26	38	25	0.074872	1	69	12	20	35	41	28	0.4570143	1	32.625	20.1064133								
4	8E+07	4_81814870	25	48	19	33	37	26	0.128943	1	10	44	25	23	27	24	0.98883926	1	28.375	12.6935023					8.2E+07	8.2E+07	0.13251	ID=promoter_36315;

4	8E+07	4_81910877	590	251	484	341	421	413	0.035506	1	605	371	539	387	488	463	0.93823451	1	446	127.764292					8.2E+07	8.2E+07	0.26063	ID=promoter_36316;
4	8E+07	4_820092	8	57	20	17	33	19	0.041427	1	17	27	26	7	22	17	0.8526593	1	22.375	15.7655819								
4	8E+07	4_820804	31	0	22	34	16	28	0.437866	1	30	0	34	0	15	17	0.85096642	1	18.875	16.066269								
4	8E+07	4_820966	207	48	97	91	128	94	0.002355	0.12769	28	55	41	25	42	33	0.7761727	1	74	59.9404466								
4	8E+07	4_821467	0	15	32	32	8	32	0.004292	0.20739	1	0	36	0	1	18	2.55E-04	0.0200383	14.5	16.4229804								
4	8E+07	4_821471	30	8	8	0	19	4	0.002035	0.11403	4	1	5	12	3	9	0.17582851	1	8.5	9.53190132								
4	8E+07	4_82224044	79	42	53	83	61	68	0.959207	1	39	6	7	9	23	8	0.04560275	1	39.75	31.0518					8.2E+07	8.2E+07	0.16888	ID=promoter_36339;
4	8E+07	4_82230345	139	179	140	144	159	142	0.060544	1	51	30	108	53	41	81	2.83E-04	0.0220085	105.5	54.287594					8.2E+07	8.2E+07	0.54392	ID=promoter_36340;
4	8E+07	4_822651	39	17	88	82	28	85	3.49E-05	0.00356	20	10	4	3	15	4	0.06170633	1	32.875	34.1360199								
4	8E+07	4_82341569	40	2	24	35	21	30	0.785963	1	27	7	3	3	17	3	0.01239331	0.4669004	17.625	15.6564683					8.2E+07	8.2E+07	0.54491	ID=promoter_36354;
4	8E+07	4_82341686	9200	6624	10322	9620	7912	9971	1.16E-05	0.00136	7291	5873	12863	3702	6582	8283	1.08E-75	2.80E-72	8186.9	2883.90769					8.2E+07	8.2E+07	0.54491	ID=promoter_36354;
4	8E+07	4_823479	40	34	36	54	37	45	0.986769	1	21	5	3	48	13	26	0.0678374	1	30.125	18.8485505								
4	8E+07	4_82374627	886	706	939	703	796	821	0.035203	1	464	463	622	158	464	390	0.36131787	1	617.63	253.317947					8.2E+07	8.2E+07	0.14711	ID=promoter_36358;
4	8E+07	4_824246	13	19	7	4	16	6	0.042495	1	19	7	7	0	13	4	0.12786863	1	9.5	6.88684045								
4	8E+07	4_82442644	0	0	166	142	0	154	3.09E-29	#######	0	0	98	15	0	57	4.45E-14	2.15E-11	52.625	71.1074388					8.2E+07	8.2E+07	0.14641	ID=promoter_36365;
4	8E+07	4_824596	205	116	173	129	161	151	0.141298	1	149	111	94	98	130	96	0.23827452	1	134.38	38.8401026								
4	8E+07	4_825781	9	8	2	7	9	5	0.390586	1	17	0	8	30	9	19	0.11219606	1	10.125	9.49341877								
4	8E+07	4_82706634	213	42	227	158	128	193	0.087192	1	39	115	131	179	77	155	8.72E-08	1.84E-05	138	70.9345775	8E+07	8.3E+07	ID=ENSP0000313420;PRKDC;NM_006904;	PRKDC	8.3E+07	8.3E+07	0.18495	ID=promoter_36388;
4	8E+07	4_82715320	8	28	3	6	18	5	0.008132	0.34618	55	0	0	0	28	0	2.31E-06	0.0003528	12.5	19.5228803	8E+07	8.3E+07	ID=ENSP0000313420;PRKDC;NM_006904;	PRKDC				
4	8E+07	4_82788152	42	78	19	50	60	35	0.00364	0.18261	50	61	85	28	56	57	0.88387193	1	51.625	22.7340996	8E+07	8.3E+07	ID=ENSP0000313420;PRKDC;NM_006904;	PRKDC				
4	8E+07	4_846738	7	47	21	26	27	24	0.611598	1	31	40	73	44	36	59	0.02403133	0.7884333	36.125	19.8597763								
4	8E+07	4_846825	7	35	15	11	21	13	0.183347	1	34	116	14	10	75	12	1.23E-09	3.55E-07	30.25	36.2678527								
4	8E+07	4_847543	4	34	2	5	19	4	0.002035	0.11403	2	13	3	1	8	2	0.20550608	1	8	11.1611571								
4	9E+07	4_858495	0	9	0	0	5	0	0.05327	1	26	8	32	0	17	16	0.99905703	1	9.375	12.7720398								
4	9E+07	4_860137	55	26	56	47	41	52	0.932362	1	99	60	128	60	80	94	0.29532087	1	66.375	32.0398747								
4	9E+07	4_869557	8	13	3	8	11	6	0.3028	1	13	23	47	25	18	36	0.02410096	0.7905361	17.5	14.081396								
4	9E+07	4_87674991	4	16	9	11	10	10	0.938234	1	0	6	0	0	3	0	0.24893445	1	5.75	5.92211352	9E+07	8.8E+07	ID=ENSP0000236166-D6;FMO6P;;	FMO6P				
4	9E+07	4_87954555	0	21	6	1	11	4	0.10516	1	23	1	12	0	12	6	0.45526626	1	8	9.56182887					8.8E+07	8.8E+07	0.56959	ID=promoter_36441;
4	9E+07	4_88178963	441	160	418	288	301	353	0.999998	1	221	159	86	136	190	111	4.99E-04	0.0354295	238.63	132.14055					8.8E+07	8.8E+07	0.55198	ID=promoter_36442;
4	9E+07	4_88354570	646	175	514	358	411	436	0.343411	1	149	153	182	87	151	135	0.95424261	1	283	202.093331					8.8E+07	8.8E+07	0.55198	ID=promoter_36445;
4	9E+07	4_89013091	1	12	7	7	7	7	0.956352	1	25	1	5	6	13	6	0.35360803	1	8	7.72750191					8.9E+07	8.9E+07	0.65646	ID=promoter_36455;
4	9E+07	4_89013313	267	103	256	225	185	241	0.561839	1	105	71	41	71	88	56	0.08622677	1	142.38	91.5594069					8.9E+07	8.9E+07	0.65646	ID=promoter_36455;
4	9E+07	4_890150	87	46	51	29	67	40	0.002653	0.14147	14	37	40	2	26	21	0.89538361	1	38.25	25.6111025								
4	9E+07	4_89016172	2752	2109	3606	3238	2431	3422	5.05E-11	#######	1766	1774	1972	751	1770	1362	1.53E-06	0.000243	2246	915.872262					8.9E+07	8.9E+07	0.22547	ID=promoter_36456;

4	9E+07	4_89474812	12	57	14	9	35	12	4.73E-04	0.03365	0	17	19	0	9	10	0.92505409	1	16	18.015866	9E+07	9E+07	ID=ENSP0000362586-D2;PBX3;;	PBX3				
4	9E+07	4_896202	185	89	152	96	137	124	0.110897	1	71	51	28	41	61	35	0.07360558	1	89.125	54.8072923								
4	9E+07	4_896863	1	29	14	8	15	11	0.4902	1	19	13	1	2	16	2	0.00719486	0.3031284	10.875	9.93460762								
4	9E+07	4_897674	3	0	16	9	2	13	0.039726	1	0	10	7	10	5	9	0.48000906	1	6.875	5.56616051								
4	9E+07	4_908707	201	90	141	150	146	146	0.394224	1	148	115	85	15	132	50	1.82E-07	3.61E-05	118.13	55.8478481								
4	9E+07	4_908814	88	27	59	71	58	65	0.968656	1	122	58	75	48	90	62	0.19589823	1	68.5	28.3700043								
4	9E+07	4_910963	31	55	3	40	43	22	0.004975	0.23406	32	12	48	2	22	25	0.78399411	1	27.875	20.1950312								
4	9E+07	4_918251	15	29	18	40	22	29	0.918216	1	33	2	13	2	18	8	0.20901227	1	19	13.9795769								
4	9E+07	4_927973	352	139	269	411	246	340	0.145755	1	183	168	156	102	176	129	0.12701246	1	222.5	110.136279								
4	9E+07	4_92819523	203	55	201	164	129	183	0.253603	1	90	124	58	63	107	61	0.00924928	0.3692029	119.75	62.8143523					9.3E+07	9.3E+07	0.32936	ID=promoter_36499;
4	9E+07	4_92850551	1	13	10	8	7	9	0.983538	1	11	14	20	1	13	11	0.97545578	1	9.75	6.4531277	9E+07	9.3E+07	ID=ENSP0000356990;KLHDC9;NM_152366;	KLHDC9				
4	9E+07	4_933704	26	6	24	1	16	13	0.613293	1	9	24	15	0	17	8	0.27279187	1	13.125	10.6427373								
4	9E+07	4_935623	970	464	1215	801	717	1008	0.001017	0.06429	344	275	299	186	310	243	0.14642039	1	569.25	377.891802								
4	9E+07	4_935629	291	117	238	208	204	223	0.767457	1	117	50	171	62	84	117	0.01643663	0.5841568	156.75	85.3459347								
4	9E+07	4_93572920	4	7	9	2	6	6	0.962469	1	1	0	27	1	1	14	0.00214405	0.1161796	6.375	8.91126895	9E+07	9.4E+07	ID=ENSP0000294785;NCSTN;NM_015331;	NCSTN				
4	9E+07	4_93695845	7	40	5	15	24	10	0.016074	0.59429	20	20	29	7	20	18	0.99585638	1	17.875	12.1471631	9E+07	9.4E+07	ID=ENSP0000355257-D2;;					
4	9E+07	4_940044	70	81	84	49	76	67	0.231821	1	0	0	33	33	0	33	1.86E-08	4.40E-06	43.75	33.345593								
4	9E+07	4_943113	0	15	12	16	8	14	0.661714	1	1	1	12	0	1	6	0.13767373	1	7.125	7.21976454								
4	9E+07	4_948487	169	56	159	115	113	137	0.966707	1	36	2	4	0	19	2	0.00190863	0.1054641	67.625	70.6438502								
4	1E+08	4_951513	98	61	52	92	80	72	0.262573	1	6	15	28	11	11	20	0.1879092	1	45.375	36.2291812								
4	1E+08	4_957767	0	27	9	15	14	12	0.725737	1	9	10	15	0	10	8	0.95278883	1	10.625	8.76580206								
4	1E+08	4_958496	55	113	83	60	84	72	0.146169	1	104	40	32	5	72	19	1.35E-06	0.000219	61.5	36.8200256								
4	1E+08	4_959194	2239	1876	3106	3975	2058	3541	9.98E-43	#######	2071	1230	3828	1628	1651	2728	2.00E-77	5.60E-74	2494.1	1022.67093								
4	1E+08	4_964407	48	103	56	59	76	58	0.0457	1	44	67	73	6	56	40	0.45044047	1	57	27.5888176								
4	1E+08	4_965768	14	19	3	18	17	11	0.29617	1	0	9	14	0	5	7	0.77775394	1	9.625	7.79995421								
4	1E+08	4_966681	9	61	37	26	35	32	0.593642	1	26	32	68	15	29	42	0.17628028	1	34.25	20.7416076								
4	1E+08	4_966765	16	18	18	13	17	16	0.818283	1	1	16	40	0	9	20	0.07989336	1	15.25	12.3606519								
4	1E+08	4_96924938	112	39	81	112	76	97	0.858944	1	23	17	15	1	20	8	0.11762508	1	50	45.0618622	1E+08	9.7E+07	ID=ENSP0000351486;NTRK1;NM_002529;	NTRK1				
4	1E+08	4_97268042	921	511	826	665	716	746	0.076745	1	405	334	346	67	370	207	3.10E-08	7.10E-06	509.38	282.323491	1E+08	9.7E+07	ID=ENSP0000268182-D3;IQGAP1;NM_003870;	IQGAP1	9.7E+07	9.7E+07	0.12752	ID=promoter_36571;
4	1E+08	4_97367043	6265	4955	6376	6470	5610	6423	0.416351	1	4017	3608	8793	2894	3813	5844	4.59E-130	2.18E-126	5422.3	1925.62433					9.7E+07	9.7E+07	0.43437	ID=promoter_36579;
4	1E+08	4_973722	35	14	43	78	25	61	0.006919	0.30167	25	15	20	0	20	10	0.26942782	1	28.75	23.8791601								
4	1E+08	4_975114	274	126	184	246	200	215	0.674202	1	78	65	45	0	72	23	1.91E-05	0.0022262	127.25	98.6461932								
4	1E+08	4_97516618	789	195	552	507	492	530	0.396518	1	181	195	228	34	188	131	0.04210202	1	335.13	252.912993					9.8E+07	9.8E+07	0.441	ID=promoter_36585;
4	1E+08	4_97781896	40	10	52	46	25	49	0.10751	1	0	33	29	26	17	28	0.16600816	1	29.5	17.5824588	1E+08	9.8E+07	ID=ENSP0000357256;BGLAP;;	BGLAP				
4	1E+08	4_98114561	2792	1643	2658	2635	2218	2647	0.834848	1	2511	1477	2348	1105	1994	1727	0.11757961	1	2146.1	641.077316					9.8E+07	9.8E+07	0.15266	ID=promoter_36614;
4	1E+08	4_985171	37	179	41	41	108	41	7.57E-10	#######	99	61	46	62	80	54	0.19467416	1	70.75	48.1211269								
4	1E+08	4_98703672	2258	833	2483	2048	1546	2266	1.12E-10	#######	1131	658	855	420	895	638	2.42E-06	0.0003664	1335.8	801.585171					9.9E+07	9.9E+07	0.45687	ID=promoter_36637;
4	1E+08	4_988323	25	9	29	20	17	25	0.771176	1	0	0	0	0	0	0	1	1	10.375	12.4549187								
4	1E+08	4_989020	8	13	13	8	11	11	0.932271	1	0	20	19	18	10	19	0.1719433	1	12.375	6.82301777								

4	1E+08	4_98903161	6632	4688	9335	12923	5660	####	########	#######	6312	4177	12649	4333	5245	8491	2.91E-225	2.97E-221	7631.1	3591.56024					9.9E+07	9.9E+07	0.22561	ID=promoter_36654;
4	1E+08	4_98913191	131	108	104	126	120	115	0.300241	1	107	84	31	35	96	33	2.03E-06	0.0003157	90.75	38.4029389	1E+08	9.9E+07	ID=ENSP00000292180;FLAD1;NM_025207;	FLAD1				
4	1E+08	4_98926165	172	48	130	124	110	127	0.992652	1	96	55	72	58	76	65	0.89240132	1	94.375	44.2394055					9.9E+07	9.9E+07	0.3921	ID=promoter_36657;
4	1E+08	4_989388	215	56	163	176	136	170	0.858174	1	142	89	81	2	116	42	4.29E-07	7.84E-05	115.5	70.5427935								
4	1E+08	4_989849	40	17	84	95	29	90	1.33E-05	0.00154	84	99	36	27	92	32	4.32E-06	0.000603	60.25	33.4055171								
4	1E+08	4_991696	18	49	20	1	34	11	3.70E-04	0.02765	36	18	12	27	27	20	0.74799297	1	22.625	14.7545393								
4	1E+08	4_991938	30	1	30	19	16	25	0.667718	1	61	19	14	0	40	7	3.09E-05	0.003345	21.75	19.4770928								
4	1E+08	4_992345	8	17	0	0	13	0	4.88E-04	0.03443	15	7	4	0	11	2	0.06154634	1	6.375	6.73874723								
4	1E+08	4_994008	5	12	16	2	9	9	0.944235	1	46	14	17	0	30	9	0.00749313	0.313751	14	14.4518906								
4	1E+08	4_994216	398	270	277	313	334	295	0.001754	0.10104	148	114	99	29	131	64	1.09E-04	0.0098689	206	126.522725								
4	1E+08	4_994487	80	61	70	44	71	57	0.100263	1	96	17	62	42	57	52	0.99650227	1	59	24.5589669								
4	1E+08	4_99770934	0	1	1	0	1	1	0.993645	1	0	19	0	29	10	15	0.49251655	1	6.25	11.2852622					1E+08	1E+08	0.23401	ID=promoter_36690;
4	1E+08	4_100179278	43	17	42	37	30	40	0.868514	1	1	60	56	37	31	47	0.09966146	1	36.625	19.4417629								
4	1E+08	4_100362010	1	1	5	10	1	8	0.109969	1	26	0	11	1	13	6	0.35360803	1	6.875	8.83883476								
4	1E+08	4_100485987	1	0	13	9	1	11	0.032372	1	36	0	12	0	18	6	0.0767986	1	8.875	12.3107096								
4	1E+08	4_100534815	11	41	17	14	26	16	0.118485	1	14	11	15	1	13	8	0.65599869	1	15.5	11.389218								
4	1E+08	4_100660448	19	17	10	0	18	5	0.008132	0.34618	24	1	9	0	13	5	0.22538616	1	10	9.31971796								
4	1E+08	4_101236191	3	5	3	4	4	4	0.97482	1	1	1	6	100	1	53	1.73E-12	7.04E-10	15.375	34.2383974								
4	1E+08	4_101613013	10	41	16	0	26	8	0.001755	0.10104	24	15	5	27	20	16	0.90780655	1	17.25	13.1556615	1E+08	1E+08	ID=ENSP00000357842;TUFT1;NM_020127;	TUFT1				
4	1E+08	4_101700871	22	28	28	68	25	48	0.13045	1	0	0	18	1	0	10	0.00454609	0.2107253	20.625	22.6711743								
4	1E+08	4_102074552	477	151	462	491	314	477	0.001718	0.09971	294	169	114	154	232	134	6.21E-05	0.0060773	289	164.053998					1E+08	1E+08	0.60899	ID=promoter_36751;
4	1E+08	4_102327573	11	9	5	5	10	5	0.276106	1	38	10	21	2	24	12	0.20726737	1	12.625	11.74658								
4	1E+08	4_102340119	15	8	5	0	12	3	0.030625	0.98374	1	24	8	0	13	4	0.12786863	1	7.625	8.36553303								
4	1E+08	4_102775581	297	59	154	148	178	151	0.013214	0.50759	107	49	53	17	78	35	0.00130184	0.0776118	110.5	89.9015334					1E+08	1E+08	0.24158	ID=promoter_36769;
4	1E+08	4_102787286	277	171	191	187	224	189	0.003652	0.18279	237	101	126	4	169	65	3.87E-09	1.02E-06	161.75	84.8338713								
4	1E+08	4_103179587	180	93	87	93	137	90	8.40E-05	0.00761	27	48	51	2	38	27	0.5702351	1	72.625	54.3925875	1E+08	1E+08	ID=ENSP0000036431 0-D3;H2AFX;NM_002105;	H2AFX	1E+08	1E+08	0.67815	ID=promoter_36781;
4	1E+08	4_103235010	478	114	324	293	296	309	0.357773	1	140	154	189	97	147	143	0.91933862	1	223.63	131.304376					1E+08	1E+08	0.35985	ID=promoter_36787;
4	1E+08	4_103306521	2976	4682	3863	5765	3829	4814	0.00587	0.2677	3319	2457	5313	1351	2888	3332	7.22E-17	4.11E-14	3715.8	1492.22219					1E+08	1E+08	0.74964	ID=promoter_36792;
4	1E+08	4_103318968	1094	379	1059	627	737	843	0.883088	1	485	369	420	161	427	291	2.59E-04	0.0203191	574.25	336.088743					1E+08	1E+08	0.73463	ID=promoter_36794;
4	1E+08	4_103329381	8820	5855	8655	8558	7338	8607	1	1	6123	4150	4558	1680	5137	3119	2.46E-77	6.75E-74	6049.9	2555.81429					1E+08	1E+08	0.63393	ID=promoter_36796;
4	1E+08	4_103336486	417	208	397	174	313	286	0.009236	0.38203	100	151	73	49	126	61	1.26E-04	0.0111328	196.13	140.330668					1E+08	1E+08	0.56391	ID=promoter_36798;
4	1E+08	4_103361330	2099	544	1807	1321	1322	1564	0.974001	1	750	502	554	319	626	437	2.97E-05	0.0032446	987	669.952664					1E+08	1E+08	0.50365	ID=promoter_36805;

4	1E+08	4_103368448	1116	609	830	776	863	803	1.36E-05	0.00156	482	473	356	138	478	247	3.74E-13	1.64E-10	597.5	305.472935					1E+08	1E+08	0.62789	ID=promoter_36807;
4	1E+08	4_103379176	12750	8188	14463	15593	10469	####	1.43E-55	#######	8455	6589	8503	3520	7522	6012	1.02E-16	5.67E-14	9757.6	4137.44662					1E+08	1E+08	0.74727	ID=promoter_36809;
4	1E+08	4_103534510	11	39	6	13	25	10	0.01059	0.42992	0	9	1	0	5	1	0.30486099	1	9.875	12.811128	1E+08	1E+08	ID=ENSP00000347802;ANKRD35;NM_144698;	ANKRD35				
4	1E+08	4_103541240	130	43	138	128	87	133	0.156234	1	45	13	52	69	29	61	9.35E-04	0.0596362	77.25	47.9516125	1E+08	1E+08	ID=ENSP00000347802;ANKRD35;NM_144698;	ANKRD35	1E+08	1E+08	0.19083	ID=promoter_36818;
4	1E+08	4_103548868	275	111	151	196	193	174	0.041303	1	135	36	78	59	86	69	0.67011337	1	130.13	78.1837351								
4	1E+08	4_104573296	98	78	83	50	88	67	0.027109	0.89891	10	20	8	2	15	5	0.11779525	1	43.625	38.5817777	1E+08	1E+08	ID=ENSP00000359053-D2;NBPF6;;	NBPF6				
4	1E+08	4_105300374	20	12	10	18	16	14	0.724026	1	51	53	76	19	52	48	0.99977053	1	32.375	24.289548	1E+08	1.1E+08	ID=ENSP00000360765;;					
4	1E+08	4_105362003	59	57	37	13	58	25	7.41E-05	0.00687	14	54	9	14	34	12	0.01147783	0.4380166	32.125	22.0417623								
4	1E+08	4_105566442	19	15	0	0	17	0	4.68E-05	0.00457	0	7	7	1	4	4	0.99427496	1	6.125	7.41499446								
4	1E+08	4_105708542	40	56	17	51	48	34	0.075354	1	52	28	20	3	40	12	0.00146628	0.0860502	33.375	19.3164438								
4	1E+08	4_105791471	79	17	40	27	48	34	0.075354	1	58	10	15	5	34	10	0.00342551	0.1709558	31.375	25.9060804	1E+08	1.1E+08	ID=ENSP00000358428;HAO2;;	HAO2				
4	1E+08	4_106094434	18	45	41	57	32	49	0.500668	1	54	32	75	24	43	50	0.55103436	1	43.25	18.7292743								
4	1E+08	4_106253088	2	1	6	0	2	3	0.964182	1	19	11	1	0	15	1	0.00364468	0.1775497	5	6.80336051								
4	1E+08	4_107128211	16	47	29	34	32	32	0.815447	1	90	14	67	106	52	87	0.00291021	0.148297	50.375	34.1882916								
4	1E+08	4_107132022	74	25	31	43	50	37	0.100649	1	0	0	0	0	0	0	1	1	21.625	27.1763421								
4	1E+08	4_107188146	2	2	2	15	2	9	0.179815	1	89	18	22	20	54	21	0.00230401	0.1234682	21.25	28.6692967								
4	1E+08	4_107197542	0	0	11	5	0	8	0.033035	1	0	9	20	39	5	30	4.95E-05	0.0050484	10.5	13.4483138								
4	1E+08	4_107216312	32	78	43	61	55	52	0.535946	1	218	113	57	126	166	92	3.33E-04	0.0252908	91	60.7665321					1.1E+08	1.1E+08	0.58503	ID=promoter_36877;
4	1E+08	4_107621630	36	9	38	19	23	29	0.967038	1	45	31	34	22	38	28	0.65253165	1	29.25	11.7321293								
4	1E+08	4_107721490	0	0	72	32	0	52	2.36E-10	#######	0	0	5	0	0	3	0.19828566	1	13.625	26.0435761								
4	1E+08	4_107761582	34	31	17	16	33	17	0.018001	0.65052	1	32	82	20	17	51	5.27E-05	0.0052805	29.125	23.9787853	1E+08	1.1E+08	ID=ENSP00000358478;TTF2;NM_003594;	TTF2				
4	1E+08	4_107774168	55	33	61	44	44	53	0.991545	1	89	105	86	73	97	80	0.74077581	1	68.25	24.429198	1E+08	1.1E+08	ID=ENSP00000358478;TTF2;NM_003594;	TTF2				
4	1E+08	4_107788555	2434	1267	2072	1882	1851	1977	0.015041	0.56426	1293	1025	1183	397	1159	790	1.89E-10	6.09E-08	1444.1	650.312988					1.1E+08	1.1E+08	0.21542	ID=promoter_36884;
4	1E+08	4_107824890	451	189	324	281	320	303	0.027844	0.91436	32	18	17	22	25	20	0.88611817	1	166.75	170.182885	1E+08	1.1E+08	ID=ENSP00000358482;IGSF2;NM_004258;	IGSF2				

4	1E+08	4_107828079	9	52	63	37	31	50	0.375729	1	48	41	62	16	45	39	0.95370772	1	41	19.856629	1E+08	1.1E+08	ID=ENSP0000358482;IGSF2;NM_004258;	IGSF2				
4	1E+08	4_107858085	3	0	10	3	2	7	0.358464	1	0	17	33	1	9	17	0.21129426	1	8.375	11.5627172								
4	1E+08	4_107859339	198	79	231	196	139	214	0.04346	1	54	78	19	12	66	16	1.47E-06	0.0002348	108.38	86.8215534					1.1E+08	1.1E+08	0.34704	ID=promoter_36885;
4	1E+08	4_107903968	21	15	4	3	18	4	0.003432	0.17404	0	17	27	26	9	27	0.00543585	0.2460834	14.125	10.6158574	1E+08	1.1E+08	ID=ENSP0000376899;PTGFRN;NM_020440;	PTGFRN	1.1E+08	1.1E+08	0.2358	ID=promoter_36890;
4	1E+08	4_108229751	12	46	20	15	29	18	0.097648	1	65	8	41	19	37	30	0.86166638	1	28.25	20.1121854								
4	1E+08	4_108229901	491	226	404	368	359	386	0.494331	1	83	122	76	58	103	67	0.07620278	1	228.5	170.717812								
4	1E+08	4_108233071	943	182	692	715	563	704	0.527105	1	335	397	383	54	366	219	1.61E-06	0.0002543	462.63	297.543724					1.1E+08	1.1E+08	0.5993	ID=promoter_36896;
4	1E+08	4_108732751	22	36	6	21	29	14	0.018844	0.67198	19	0	7	0	10	4	0.34296513	1	13.875	12.6878739								
4	1E+08	4_109080694	41	59	111	65	50	88	0.069857	1	46	44	26	24	45	25	0.11611057	1	52	27.7231208								
4	1E+08	4_109283865	2	50	10	7	26	9	0.003595	0.18068	22	22	24	1	22	13	0.43054584	1	17.25	16.1134372	1E+08	1.1E+08	ID=ENSP0000261448;CASQ2;NM_001232;	CASQ2				
4	1E+08	4_109313905	145	142	113	60	144	87	4.20E-06	0.00055	119	17	101	27	68	64	0.99623345	1	90.5	49.8798557	1E+08	1.1E+08	ID=ENSP0000358522;VANGL1;NM_138959;	VANGL1	1.1E+08	1.1E+08	0.3288	ID=promoter_36943;
4	1E+08	4_109338017	65	84	79	85	75	82	0.90772	1	26	16	63	2	21	33	0.16057985	1	52.5	32.9545141					1.1E+08	1.1E+08	0.24335	ID=promoter_36945;
4	1E+08	4_109360981	4851	2009	2431	1419	3430	1925	########	#######	2271	1291	954	327	1781	641	2.15E-99	7.67E-96	1944.1	1367.78209					1.1E+08	1.1E+08	0.56913	ID=promoter_36948;
4	1E+08	4_110234017	81	52	41	33	67	37	8.34E-04	0.0545	0	1	5	0	1	3	0.56022579	1	26.625	30.2132303								
4	1E+08	4_110668260	100	120	76	28	110	52	1.66E-07	#######	73	56	37	1	65	19	1.79E-05	0.0021018	61.375	39.0235735								
4	1E+08	4_110685762	6	18	10	26	12	18	0.803442	1	0	10	2	0	5	1	0.30486099	1	9	9.19627254								
4	1E+08	4_110764355	6	59	11	0	33	6	8.35E-06	0.00102	28	7	14	5	18	10	0.42261869	1	16.25	19.1963538								
4	1E+08	4_110798592	0	0	0	0	0	0	1	1	19	10	14	2	15	8	0.44137572	1	5.625	7.63333853								
4	1E+08	4_110809859	71	26	62	40	49	51	0.836353	1	25	43	58	61	34	60	0.0095223	0.3791471	48.25	17.3019404					1.1E+08	1.1E+08	0.17432	ID=promoter_36967;
4	1E+08	4_110831235	22	90	23	50	56	37	0.023015	0.78841	91	33	85	8	62	47	0.58093458	1	50.25	33.9569055								
4	1E+08	4_110972363	0	25	2	5	13	4	0.041893	1	37	51	19	27	44	23	0.07826377	1	20.75	18.0059514								
4	1E+08	4_111057709	10	3	3	1	7	2	0.160905	1	0	11	14	0	6	7	0.91781435	1	5.25	5.54848757								
4	1E+08	4_111337216	4	11	11	10	8	11	0.943069	1	26	0	19	0	13	10	0.90601506	1	10.125	9.06228448	1E+08	1.1E+08	ID=ENSP0000304604;MAGI3;NM_001142782;	MAGI3				
4	1E+08	4_111401513	103	53	35	24	78	30	3.30E-07	#######	76	1	15	0	39	8	1.12E-04	0.0100767	38.375	36.7226575								
4	1E+08	4_111552585	11	55	27	3	33	15	0.006796	0.29841	44	20	7	3	32	5	1.41E-04	0.012246	21.25	19.5137314								
4	1E+08	4_111552911	45	156	69	102	101	86	0.090439	1	43	37	59	4	40	32	0.82411274	1	64.375	46.426401								
4	1E+08	4_111626610	8	22	6	9	15	8	0.181584	1	42	31	37	3	37	20	0.14440904	1	19.75	15.341354								
4	1E+08	4_111713789	0	55	6	12	28	9	0.001431	0.08503	78	0	8	4	39	6	1.85E-05	0.0021598	20.375	29.3935732								

4	1E+08	4_112403255	94	81	67	54	88	61	0.006099	0.27549	159	74	92	16	117	54	8.64E-05	0.008159	79.625	40.6340375								
4	1E+08	4_112668507	288	167	122	82	228	102	4.43E-16	#######	167	132	109	8	150	59	6.56E-08	1.42E-05	134.38	80.397117								
4	1E+08	4_112782952	48	19	39	12	34	26	0.254409	1	33	28	26	0	31	13	0.04801516	1	25.625	15.2403365								
4	1E+08	4_112893199	220	74	174	163	147	169	0.984307	1	30	20	15	3	25	9	0.04125862	1	87.375	85.5067875								
4	1E+08	4_112941189	29	58	91	50	44	71	0.248172	1	1	13	42	41	7	42	9.37E-07	0.0001567	40.625	27.7691375								
4	1E+08	4_112951693	41	24	22	12	33	17	0.018001	0.65052	31	5	11	2	18	7	0.13207736	1	18.5	13.4057557								
4	1E+08	4_113003155	78	50	145	59	64	102	0.155292	1	202	214	470	451	208	461	6.60E-26	6.04E-23	208.63	167.244848								
4	1E+08	4_113075704	14	7	19	6	11	13	0.999831	1	0	8	9	2	4	6	0.75330063	1	8.125	6.12809688								
4	1E+08	4_113163525	167	43	63	15	105	39	7.84E-10	#######	0	14	10	2	7	6	0.99011599	1	39.25	55.943211								
4	1E+08	4_113246375	1	10	9	1	6	5	0.851367	1	18	28	19	41	23	30	0.4647018	1	15.875	13.7158667								
4	1E+08	4_113426013	7	19	0	11	13	6	0.146758	1	0	27	38	16	14	27	0.07530308	1	14.75	13.1990259								
4	1E+08	4_113958098	47	0	67	23	24	45	0.173105	1	1	15	0	2	8	1	0.0848225	1	19.375	25.2243505								
4	1E+08	4_114050136	322	165	320	231	244	276	0.918236	1	40	44	49	35	42	42	0.9414956	1	150.75	126.506634								
4	1E+08	4_114052877	44	2	18	27	23	23	0.863606	1	0	0	6	1	0	4	0.11562589	1	12.25	16.1665439								
4	1E+08	4_114081031	47	10	3	7	29	5	2.61E-05	0.00275	0	0	0	1	0	1	0.58312783	1	8.5	15.9910689								
4	1E+08	4_114083005	47	35	63	54	41	59	0.60213	1	37	29	5	48	33	27	0.89046553	1	39.75	17.7984751								
4	1E+08	4_114217466	42	76	77	88	59	83	0.564772	1	68	50	140	30	59	85	0.03207467	0.9827712	71.375	33.9787223								
4	1E+08	4_114233958	79	191	66	137	135	102	0.003309	0.16955	122	54	39	35	88	37	1.89E-04	0.0156622	90.375	54.8398643								
4	1E+08	4_114409656	12	7	12	6	10	9	0.84607	1	0	0	9	0	0	5	0.06742467	1	5.75	5.2030211								
4	1E+08	4_114516515	1567	651	1512	1095	1109	1304	0.998199	1	639	459	721	190	549	456	0.22213229	1	854.25	493.016589	1E+08	1.1E+08	ID=ENSP000003588 10;FAM40A;NM_033088;	FAM40A				
4	1E+08	4_114520686	325	88	212	173	207	193	0.07114	1	51	96	51	1	74	26	5.61E-05	0.0055937	124.63	105.836717	1E+08	1.1E+08	ID=ENSP000003588 10;FAM40A;NM_033088;	FAM40A	1.1E+08	1.1E+08	0.37262	ID=promoter_37066;
4	1E+08	4_114782270	15	24	11	13	20	12	0.173914	1	0	12	0	0	6	0	0.06196836	1	9.375	8.71677365								
4	1E+08	4_114907079	15	0	13	21	8	17	0.371907	1	0	16	0	0	8	0	0.02452223	0.7977568	8.125	8.96720214								
4	1E+08	4_115136283	2155	1185	2337	2003	1670	2170	0.007083	0.30684	1148	1247	1187	603	1198	895	6.28E-06	0.0008443	1483.1	605.927963					1.2E+08	1.2E+08	0.57189	ID=promoter_37083;
4	1E+08	4_115151305	142	42	145	118	92	132	0.331473	1	78	33	54	28	56	41	0.51562687	1	80	48.6239212	1E+08	1.2E+08	ID=ENSP000003093 24;CYB561D1;NM_182580;	CYB561D1	1.2E+08	1.2E+08	0.22318	ID=promoter_37086;
4	1E+08	4_115307406	7	29	9	31	18	20	0.986196	1	74	5	37	17	40	27	0.44121895	1	26.125	22.7749079	1E+08	1.2E+08	ID=ENSP000003866 28;PSRC1;NM_001032290;	PSRC1				
4	1E+08	4_115896881	17	12	7	10	15	9	0.269309	1	0	0	5	1	0	3	0.19828566	1	6.5	6.21059003	1E+08	1.2E+08	ID=ENSP000003590 48;Clorf59;;	Clorf59				
4	1E+08	4_116187119	13	33	2	36	23	19	0.524147	1	22	22	76	86	22	81	4.44E-09	1.17E-06	36.25	29.7261309								
4	1E+08	4_116702853	150	126	166	144	138	155	0.933683	1	188	182	291	65	185	178	0.93921057	1	164	64.2250507								
4	1E+08	4_116717917	56	48	15	10	52	13	2.75E-07	#######	41	21	80	56	31	68	2.25E-04	0.0182743	40.875	24.0739189								
4	1E+08	4_116845247	0	20	1	0	10	1	0.011548	0.45484	0	7	0	28	4	14	0.04184353	1	7	10.9674844								

4	1E+08	4_116951469	165	67	155	180	116	168	0.216325	1	48	27	18	21	38	20	0.11575311	1	85.125	69.6592472								
4	1E+08	4_117033734	9	14	1	1	12	1	0.003675	0.18279	1	11	18	1	6	10	0.51486293	1	7	6.9075528	1E+08	1.2E+08	ID=ENSP00000359090;NTNG1;NM_001113226;	NTNG1				
4	1E+08	4_117307591	41	32	29	36	37	33	0.517593	1	76	18	13	38	47	26	0.10217262	1	35.375	19.0633343								
4	1E+08	4_117342454	9	55	38	35	32	37	0.998236	1	7	18	17	8	13	13	0.98151319	1	23.375	17.4432754								
4	1E+08	4_117355209	0	1	12	25	1	19	0.001144	0.07115	51	12	11	0	32	6	3.53E-04	0.0265555	14	17.1880357					1.2E+08	1.2E+08	0.20097	ID=promoter_37128;
4	1E+08	4_117410506	780	229	500	475	505	488	0.009338	0.38547	193	249	337	133	221	235	0.34188729	1	362	213.348139					1.2E+08	1.2E+08	0.52986	ID=promoter_37129;
4	1E+08	4_117636686	8	46	22	5	27	14	0.038924	1	47	0	14	44	24	29	0.62889415	1	23.25	19.6596033								
4	1E+08	4_117776650	24	49	20	10	37	15	0.001345	0.08048	42	64	65	22	53	44	0.86370907	1	37	21.023796								
4	1E+08	4_117790353	0	44	6	4	22	5	0.001077	0.06758	11	45	22	6	28	14	0.15944813	1	17.25	18.0218122								
4	1E+08	4_118576427	11	1	14	13	6	14	0.355886	1	0	18	4	0	9	2	0.1390433	1	7.625	7.19002285								
4	1E+08	4_118800369	5	16	8	0	11	4	0.10516	1	23	27	7	0	25	4	0.00107118	0.0664407	10.75	10.1945364								
4	1E+08	4_119135341	1	20	4	2	11	3	0.050503	1	26	0	2	3	13	3	0.06317581	1	7.25	9.92471662								
4	1E+08	4_119618699	90	78	69	49	84	59	0.009489	0.39157	108	88	44	61	98	53	0.00599286	0.2634475	73.375	21.8431912								
4	1E+08	4_119762473	1	14	4	6	8	5	0.532535	1	17	23	8	20	20	14	0.72101732	1	11.625	8.01672359								
4	1E+08	4_119923942	22	32	9	8	27	9	0.002276	0.124	20	13	37	0	17	19	0.85444859	1	17.625	12.5690948								
4	1E+08	4_122029359	129	64	98	129	97	114	0.999899	1	64	43	85	28	54	57	0.79136001	1	80	37.2941972					1.2E+08	1.2E+08	0.26557	ID=promoter_37142;
4	1E+08	4_122269539	27	3493	22	32	1760	27	0	0	71	3074	56	37	1573	47	1.08E-288	1.29E-284	851.5	1505.31867					1.2E+08	1.2E+08	0.49635	ID=promoter_37145;
4	1E+08	4_122307669	16	56	14	1	36	8	1.18E-05	0.00138	18	43	34	28	31	31	0.95647893	1	26.25	17.6857489								
4	1E+08	4_123228002	43	57	76	22	50	49	0.6698	1	0	0	0	0	0	0	1	1	24.75	30.3726851								
4	1E+08	4_123229156	8	31	0	6	20	3	4.27E-04	0.03093	26	23	6	1	25	4	0.00107118	0.0664407	12.625	12.1177261								
4	1E+08	4_123326897	189	72	170	148	131	159	0.958905	1	40	17	35	17	29	26	0.99236142	1	86	71.6579175								
4	1E+08	4_123909494	48	52	18	27	50	23	5.99E-04	0.04108	23	9	27	14	16	21	0.57439123	1	27.25	15.3785565								
4	1E+08	4_123915964	22	4	18	40	13	29	0.146708	1	1	0	0	0	1	0	0.62906424	1	10.625	14.7448538								
4	1E+08	4_123925339	39	76	24	24	58	24	4.20E-05	0.00416	33	25	44	26	29	35	0.57403411	1	36.375	17.6872634								
4	1E+08	4_124008514	430	289	376	270	360	323	0.002134	0.11812	263	179	253	183	221	218	0.80929733	1	280.38	86.6222299								
4	1E+08	4_124067539	17	27	13	20	22	17	0.428533	1	26	28	27	2	27	15	0.27474073	1	20	9.1025899								
4	1E+08	4_124078482	2	41	11	3	22	7	0.005528	0.25484	2	9	34	31	6	33	3.01E-05	0.0032658	16.625	16.0618225								
4	1E+08	4_124079458	0	38	15	1	19	8	0.039867	1	1	36	26	31	19	29	0.23299331	1	18.5	16.3270153								
4	1E+08	4_124341806	3	43	47	85	23	66	6.80E-04	0.04582	43	8	9	27	26	18	0.63344191	1	33.125	27.3518738								
4	1E+08	4_125035688	45	25	33	9	35	21	0.046836	1	0	1	25	6	1	16	7.41E-04	0.0492332	18	16.4316767								
4	1E+08	4_125311742	100	60	165	101	80	133	0.046441	1	61	42	127	33	52	80	0.01607851	0.5735039	86.125	45.3382131	1E+08	1.3E+08	ID=ENSP00000377715;DPYD;;	DPYD				
4	1E+08	4_125369557	1	0	11	34	1	23	2.11E-04	0.01699	21	27	2	20	24	11	0.1422748	1	14.5	12.9283741	1E+08	1.3E+08	ID=ENSP00000377715;DPYD;;	DPYD				

4	1E+08	4_125434371	3	40	21	29	22	25	0.99414	1	88	34	43	20	61	32	0.03034118	0.9401478	34.75	25.0128538	1E+08	1.3E+08	ID=ENSP00000377715;DPYD;;	DPYD				
4	1E+08	4_125475076	20	30	12	32	25	22	0.614783	1	25	15	34	22	20	28	0.36590545	1	23.75	7.97764734	1E+08	1.3E+08	ID=ENSP00000377715;DPYD;;	DPYD				
4	1E+08	4_125601630	27	120	0	41	74	21	3.66E-09	#######	9	78	62	77	44	70	0.01765503	0.6154554	51.75	40.2199311								
4	1E+08	4_125783159	3	32	8	13	18	11	0.22238	1	26	9	10	7	18	9	0.30718372	1	13.5	10.0995049								
4	1E+08	4_126050040	305	209	219	142	257	181	7.38E-07	0.00011	0	46	30	22	23	26	0.7859394	1	121.63	113.382964								
4	1E+08	4_126136297	21	62	12	37	42	25	0.023617	0.805	82	30	69	58	56	64	0.51867409	1	46.375	24.9051773								
4	1E+08	4_126245297	0	32	3	0	16	2	0.001284	0.07728	23	13	0	19	18	10	0.42261869	1	11.25	12.4183045								
4	1E+08	4_126386571	5152	4846	3071	3194	4999	3133	########	#######	4999	3787	4360	943	4393	2652	8.13E-68	1.84E-64	3794	1400.72573								
4	1E+08	4_126772453	39	65	11	37	52	24	4.64E-04	0.03323	10	7	24	31	9	28	0.00358036	0.1770746	28	19.4569121								
4	1E+08	4_126783162	2	12	3	0	7	2	0.160905	1	32	7	12	0	20	6	0.03829208	1	8.5	10.6636901								
4	1E+08	4_126848233	5	4	15	3	5	9	0.74134	1	2	13	31	1	8	16	0.19094317	1	9.25	10.1805136								
4	1E+08	4_126877465	0	0	13	5	0	9	0.02157	0.74463	0	37	18	0	19	9	0.23949138	1	9.125	13.2064649								
4	1E+08	4_126937189	14	7	26	27	11	27	0.107592	1	45	0	13	20	23	17	0.77734386	1	19	13.918128								
4	1E+08	4_126946018	11	0	21	0	6	11	0.674578	1	0	0	19	0	0	10	0.00454609	0.2107253	6.375	9.24179172					1.3E+08	1.3E+08	0.14627	ID=promoter_37214;
4	1E+08	4_127125695	66	51	67	31	59	49	0.199646	1	53	67	87	45	60	66	0.63067067	1	58.375	17.0623855					1.3E+08	1.3E+08	0.19289	ID=promoter_37239;
4	1E+08	4_127236830	8	24	1	4	16	3	0.003782	0.18716	1	3	11	5	2	8	0.1303879	1	7.125	7.62397534								
4	1E+08	4_127315015	0	21	7	6	11	7	0.438207	1	0	3	29	13	2	21	1.85E-04	0.0153843	9.875	10.4531267								
4	1E+08	4_127373479	5	2	18	5	4	12	0.241003	1	25	26	19	41	26	30	0.71540497	1	17.625	13.2873463								
4	1E+08	4_127374023	127	63	169	54	95	112	0.999333	1	98	40	36	25	69	31	0.0028472	0.1455015	76.5	50.5427683	1E+08	1.3E+08	ID=ENSP00000260526;ARHGAP29;NM_004815;	ARHGAP29				
4	1E+08	4_127396659	14	28	8	21	21	15	0.333352	1	56	0	23	1	28	12	0.0723178	1	18.875	18.129987	1E+08	1.3E+08	ID=ENSP00000260526;ARHGAP29;NM_004815;	ARHGAP29				
4	1E+08	4_127511925	58	27	47	35	43	41	0.636393	1	38	0	19	3	19	11	0.45547225	1	28.375	20.3535711	1E+08	1.3E+08	ID=ENSP00000359245;ABCA4;NM_000350;	ABCA4				
4	1E+08	4_127540270	94	75	49	75	85	62	0.016216	0.59861	35	21	52	41	28	47	0.04140707	1	55.25	24.2531294	1E+08	1.3E+08	ID=ENSP00000359245;ABCA4;NM_000350;	ABCA4	1.3E+08	1.3E+08	0.16553	ID=promoter_37270;
4	1E+08	4_127541379	14	42	8	16	28	12	0.009759	0.39939	15	5	27	21	10	24	0.03201086	0.981237	18.5	11.7229933	1E+08	1.3E+08	ID=ENSP00000359245;ABCA4;NM_000350;	ABCA4				
4	1E+08	4_127748670	63	42	61	35	53	48	0.428688	1	37	25	27	96	31	62	0.00163495	0.0924179	48.25	23.8851418								
4	1E+08	4_127752107	3	29	0	1	16	1	3.65E-04	0.02739	1	9	11	5	5	8	0.62517663	1	7.375	9.59073213					1.3E+08	1.3E+08	0.19418	ID=promoter_37291;
4	1E+08	4_127941838	12	1	36	11	7	24	0.032448	1	2	0	5	28	1	17	4.35E-04	0.0314643	11.875	13.3463478								
4	1E+08	4_127946368	3025	1122	2486	1151	2074	1819	1.23E-18	#######	666	655	817	326	661	572	0.48321099	1	1281	958.657096					1.3E+08	1.3E+08	0.57286	ID=promoter_37312;

4	1E+08	4_127949464	11	52	0	22	32	11	9.30E-04	0.05947	38	60	16	3	49	10	1.04E-05	0.0013129	25.25	22.4165118								
4	1E+08	4_127985266	0	6	0	0	3	0	0.172143	1	6	0	18	12	3	15	0.01140593	0.4370832	5.25	6.75594976	1E+08	1.3E+08	ID=ENSP00000359264;BCAR3;;	BCAR3	1.3E+08	1.3E+08	0.15984	ID=promoter_37322;
4	1E+08	4_128137778	17	0	12	5	9	9	0.944235	1	24	17	33	2	21	18	0.97064008	1	13.75	11.3609607								
4	1E+08	4_128163997	332	119	273	254	226	264	0.998971	1	119	158	184	102	139	143	0.68218745	1	192.63	84.498415	1E+08	1.3E+08	ID=ENSP000003592 95;DR1;;	DR1	1.3E+08	1.3E+08	0.31071	ID=promoter_37326;
4	1E+08	4_128317731	130	85	152	125	108	139	0.76952	1	174	117	92	73	146	83	0.00157393	0.0896055	118.5	34.3802269	1E+08	1.3E+08	ID=ENSP000003433 77;CCDC18;;	CCDC18				
4	1E+08	4_128471451	106	51	61	32	79	47	8.64E-04	0.05636	66	15	48	0	41	24	0.19624561	1	47.375	32.7585867					1.3E+08	1.3E+08	0.1159	ID=promoter_37331;
4	1E+08	4_128486149	397	232	302	203	315	253	3.78E-05	0.00381	472	166	428	90	319	259	0.28403138	1	286.25	136.254751								
4	1E+08	4_128556032	21	38	10	14	30	12	0.004264	0.20626	45	29	28	8	37	18	0.07385545	1	24.125	13.3034636								
4	1E+08	4_128729869	33	102	22	19	68	21	6.57E-08	#######	16	46	38	19	31	29	0.99938482	1	36.875	28.3772419	1E+08	1.3E+08	ID=ENSP000003593 56;EVI5;NM_005665;	EVI5				
4	1E+08	4_128809669	16	60	6	14	38	10	2.46E-05	0.00262	40	9	12	5	25	9	0.04125862	1	20.25	19.484426	1E+08	1.3E+08	ID=ENSP000003593 56;EVI5;NM_005665;	EVI5				
4	1E+08	4_128833180	27	66	38	18	47	28	0.015252	0.57129	92	29	86	67	61	77	0.19504521	1	52.875	28.4777482					1.3E+08	1.3E+08	0.20002	ID=promoter_37340;
4	1E+08	4_128964881	0	24	5	0	12	3	0.030625	0.98374	0	0	6	35	0	21	1.21E-05	0.0014894	8.75	13.3603892	1E+08	1.3E+08	ID=ENSP000003593 85;GLMN;NM_053274;	GLMN				
4	1E+08	4_129050143	0	17	8	9	9	9	0.944235	1	0	1	22	0	1	11	0.01045793	0.4067594	7.125	8.55966455					1.3E+08	1.3E+08	0.41198	ID=promoter_37360;
4	1E+08	4_129225890	110	63	82	100	87	91	0.74658	1	27	1	42	0	14	21	0.37101303	1	53.125	42.6093132								
4	1E+08	4_129236841	373	326	1025	786	350	906	6.55E-37	#######	169	240	266	56	205	161	0.28849285	1	405.13	329.690087					1.3E+08	1.3E+08	0.31824	ID=promoter_37368;
4	1E+08	4_129244037	15	43	27	5	29	16	0.046212	1	17	39	13	5	28	9	0.01512239	0.5480081	20.5	14.4913767								
4	1E+08	4_129403369	17	28	7	2	23	5	6.40E-04	0.04366	2	15	6	2	9	4	0.4571786	1	9.875	9.3722615								
4	1E+08	4_129404668	21	52	21	15	37	18	0.006738	0.29841	74	25	33	23	50	28	0.09740279	1	33	20.0641827					1.3E+08	1.3E+08	0.17007	ID=promoter_37386;
4	1E+08	4_129483864	9	4	0	20	7	10	0.922856	1	1	18	19	2	10	11	0.92604858	1	9.125	8.62616452								
4	1E+08	4_129486480	41	67	84	35	54	60	0.959156	1	46	8	23	0	27	12	0.09555594	1	38	28.3044671					1.3E+08	1.3E+08	0.24483	ID=promoter_37410;
4	1E+08	4_129498099	31	8	16	26	20	21	0.939065	1	0	0	27	2	0	15	3.07E-04	0.0235795	13.75	12.969745					1.3E+08	1.3E+08	0.15362	ID=promoter_37414;
4	1E+08	4_129507109	120	68	75	54	94	65	0.004122	0.20117	1	3	0	1	2	1	0.87709017	1	40.25	45.693857					1.3E+08	1.3E+08	0.29748	ID=promoter_37416;
4	1E+08	4_129520291	10	42	12	26	26	19	0.285911	1	1	1	29	13	1	21	5.13E-05	0.0051875	16.75	14.3999008								
4	1E+08	4_129566726	20	10	7	1	15	4	0.015825	0.58672	1	4	0	0	3	0	0.24893445	1	5.375	6.92691438								
4	1E+08	4_129584681	70	21	30	15	46	23	0.002685	0.14289	22	1	28	1	12	15	0.74080056	1	23.5	21.7715411					1.3E+08	1.3E+08	0.22011	ID=promoter_37429;
4	1E+08	4_129617552	2	9	5	4	6	5	0.851367	1	1	0	16	52	1	34	4.78E-08	1.05E-05	11.125	17.307616								
4	1E+08	4_129676942	750	387	541	423	569	482	8.80E-07	0.00013	214	107	152	81	161	117	0.13287639	1	331.88	235.936092								

4	1E+08	4_129685626	14	32	35	17	23	26	0.991715	1	44	21	17	33	33	25	0.74763976	1	26.625	10.8092223	1E+08	1.3E+08	ID=ENSP000002346 26;CDC7;NM_003503;	CDC7	1.3E+08	1.3E+08	0.36133	ID=promoter_37439;
4	1E+08	4_129747826	31	97	18	24	64	21	4.29E-07	#######	40	33	53	22	37	38	0.90627168	1	39.75	25.6668213								
4	1E+08	4_129796135	0	13	6	5	7	6	0.851931	1	0	11	11	0	6	6	0.99142475	1	5.75	5.44452543	1E+08	1.3E+08	ID=ENSP000003594 54;HFM1;NM_001017975;	HFM1				
4	1E+08	4_129862437	8	0	8	0	4	4	0.97482	1	2	0	29	1	1	15	0.00126073	0.075413	6	9.89949494								
4	1E+08	4_129981945	62	69	91	90	66	91	0.605873	1	80	33	22	47	57	35	0.15471957	1	61.75	25.8111272								
4	1E+08	4_130071867	11	1	0	6	6	3	0.462004	1	33	26	15	35	30	25	0.92542307	1	15.875	13.9226178	1E+08	1.3E+08	ID=ENSP000003780 79;ZNF644;;	ZNF644				
4	1E+08	4_130123684	13	90	32	46	52	39	0.103737	1	99	82	110	61	91	86	0.99184759	1	66.625	34.3175819					1.3E+08	1.3E+08	0.29051	ID=promoter_37465;
4	1E+08	4_130142344	59	42	22	6	51	14	1.05E-06	0.00016	53	19	13	4	36	9	8.09E-04	0.0530112	27.25	21.3123036								
4	1E+08	4_130179226	8	42	4	23	25	14	0.076795	1	38	34	16	31	36	24	0.45392565	1	24.5	14.081396								
4	1E+08	4_130182386	107	63	92	95	85	94	0.925518	1	97	41	121	27	69	74	0.68404299	1	80.375	33.1358136					1.3E+08	1.3E+08	0.11975	ID=promoter_37477;
4	1E+08	4_130356587	14	8	2	11	11	7	0.438207	1	27	11	0	18	19	9	0.23949138	1	11.375	8.65097022								
4	1E+08	4_130464075	154	39	66	78	97	72	0.012251	0.4774	54	27	34	1	41	18	0.02573717	0.8329163	56.625	46.024644								
4	1E+08	4_130776592	36	16	24	37	26	31	0.998108	1	33	0	2	1	17	2	0.0046333	0.2138882	18.625	16.1062322								
4	1E+08	4_130841842	19	47	13	28	33	21	0.084147	1	43	37	18	0	40	9	1.70E-04	0.0143289	25.625	16.088483					1.3E+08	1.3E+08	0.11681	ID=promoter_37528;
4	1E+08	4_130888675	21	68	22	22	45	22	0.002408	0.1301	4	42	3	23	23	13	0.35430953	1	25.625	21.0369573								
4	1E+08	4_130930996	39	57	31	39	48	35	0.09725	1	17	10	18	1	14	10	0.81923661	1	26.5	18.3147092								
4	1E+08	4_130986903	302	94	89	181	198	135	5.53E-06	0.0007	22	30	67	55	26	61	2.16E-04	0.0176336	105	93.6711573								
4	1E+08	4_131086522	230	101	166	201	166	184	0.86961	1	164	65	91	44	115	68	0.01235635	0.4667394	132.75	66.9920038								
4	1E+08	4_131121830	9	45	16	3	27	10	0.004472	0.21437	17	31	35	27	24	31	0.47191283	1	22.875	14.0858338								
4	1E+08	4_131166148	673	882	1228	1326	778	1277	1.08E-12	#######	542	433	1007	303	488	655	3.87E-09	1.02E-06	799.25	372.98401					1.3E+08	1.3E+08	0.19119	ID=promoter_37566;
4	1E+08	4_131180554	13	12	12	18	13	15	0.999061	1	7	17	36	4	12	20	0.26508384	1	14.875	9.7165176								
4	1E+08	4_131231688	24	0	10	27	12	19	0.716431	1	16	15	3	43	16	23	0.39752759	1	17.25	13.9565653								
4	1E+08	4_131330225	112	70	97	69	91	83	0.251436	1	124	37	19	4	81	12	9.43E-11	3.18E-08	66.5	43.6839297					1.3E+08	1.3E+08	0.16419	ID=promoter_37594;
4	1E+08	4_131430387	37	43	35	44	40	40	0.774898	1	30	5	13	13	18	13	0.78939737	1	27.5	15.0807351								
4	1E+08	4_131459636	39	172	40	72	106	56	4.61E-06	0.0006	43	152	131	72	98	102	0.71516036	1	90.125	53.7279855								
4	1E+08	4_131514944	211	74	182	174	143	178	0.86936	1	17	32	110	80	25	95	8.52E-11	2.88E-08	110	72.1209302	1E+08	1.3E+08	ID=ENSP000003780 93-D4;LRRC8D;NM_018103;	LRRC8D				
4	1E+08	4_131521328	4	26	3	4	15	4	0.015825	0.58672	1	18	28	5	10	17	0.30316389	1	11.125	11.0897314	1E+08	1.3E+08	ID=ENSP000003780 93-D4;LRRC8D;NM_018103;	LRRC8D	1.3E+08	1.3E+08	0.10374	ID=promoter_37630;

4	1E+08	4_131533423	7	0	7	0	4	4	0.97482	1	18	0	6	10	9	8	0.99626583	1	6	6.21059003	1E+08	1.3E+08	ID=ENSP00000378093-D4;LRRC8D;NM_018103;	LRRC8D	1.3E+08	1.3E+08	0.14631	ID=promoter_37634;
4	1E+08	4_131538900	2	15	0	0	9	0	0.005101	0.2381	0	1	6	29	1	18	2.55E-04	0.0200383	6.625	10.3914457	1E+08	1.3E+08	ID=ENSP00000378093-D4;LRRC8D;NM_018103;	LRRC8D	1.3E+08	1.3E+08	0.11198	ID=promoter_37635;
4	1E+08	4_131598087	7	14	25	10	11	18	0.682529	1	9	28	15	22	19	19	0.97309647	1	16.25	7.85129653								
4	1E+08	4_131721559	169	97	318	249	133	284	5.33E-08	#######	36	71	95	21	54	58	0.7381813	1	132	105.224929								
4	1E+08	4_131779037	0	6	46	32	3	39	2.82E-06	0.00039	18	1	33	43	10	38	9.38E-05	0.0087137	22.375	18.6466198	1E+08	1.3E+08	ID=ENSP00000378093-D6;LRRC8D;NM_018103;	LRRC8D				
4	1E+08	4_131837592	299	169	287	274	234	281	0.965334	1	190	161	155	100	176	128	0.11155445	1	204.38	72.9969422					1.3E+08	1.3E+08	0.33974	ID=promoter_37685;
4	1E+08	4_131857986	13	23	5	1	18	3	0.001281	0.07728	0	8	32	1	4	17	0.01069978	0.4139111	10.375	11.6611381								
4	1E+08	4_131867399	61	80	75	67	71	71	0.63593	1	18	25	7	48	22	28	0.53586024	1	47.625	27.7536999								
4	1E+08	4_131920509	40	12	66	44	26	55	0.042437	1	33	12	42	33	23	38	0.08548704	1	35.25	17.6534092								
4	1E+08	4_131927847	3	7	1	2	5	2	0.402696	1	25	7	3	1	16	2	0.00719486	0.3031284	6.125	7.98994904	1E+08	1.3E+08	ID=ENSP00000294671;GBP7;NM_207398;	GBP7	1.3E+08	1.3E+08	0.1254	ID=promoter_37690;
4	1E+08	4_132071928	34	13	12	14	24	13	0.071032	1	70	0	13	24	35	19	0.16432451	1	22.5	21.5804144								
4	1E+08	4_132153242	2	9	14	7	6	11	0.674578	1	1	26	21	19	14	20	0.45746548	1	12.375	9.13294664	1E+08	1.3E+08	ID=ENSP00000378170;CCBL2;NM_001008662;	CCBL2				
4	1E+08	4_132224294	17	23	13	9	20	11	0.118302	1	155	125	23	5	140	14	4.06E-21	2.91E-18	46.25	58.7482523					1.3E+08	1.3E+08	0.60154	ID=promoter_37717;
4	1E+08	4_132273086	29	95	38	35	62	37	0.004108	0.20053	92	30	27	68	61	48	0.69649502	1	51.75	28.8530761	1E+08	1.3E+08	ID=ENSP00000359552;PKN2;NM_006256;	PKN2				
4	1E+08	4_132454952	1	6	8	2	4	5	0.995512	1	0	14	6	24	7	15	0.16935974	1	7.625	7.99888385								
4	1E+08	4_132553032	83	212	163	166	148	165	0.90424	1	198	139	206	152	169	179	0.46173053	1	164.88	42.3469935								
4	1E+08	4_132561238	0	14	0	9	7	5	0.693374	1	27	21	12	15	24	14	0.38067267	1	12.25	9.37702359								
4	1E+08	4_132649931	62	7	36	79	35	58	0.268112	1	42	15	8	88	29	48	0.0440188	1	42.125	31.6924394								
4	1E+08	4_132654095	27	64	13	36	46	25	0.006513	0.28949	82	44	46	8	63	27	0.00271208	0.139047	40	24.8710962					1.3E+08	1.3E+08	0.27412	ID=promoter_37760;
4	1E+08	4_132677941	36	73	20	29	55	25	2.44E-04	0.01921	0	18	64	29	9	47	5.62E-07	9.91E-05	33.625	24.1479813								
4	1E+08	4_132682514	45	13	19	42	29	31	0.937432	1	24	0	14	2	12	8	0.76853133	1	19.875	16.6256042								
4	1E+08	4_132704971	173	159	186	148	166	167	0.374208	1	52	58	56	36	55	46	0.87561338	1	108.5	63.2816832					1.3E+08	1.3E+08	0.14109	ID=promoter_37770;
4	1E+08	4_132839807	354	252	236	197	303	217	1.51E-07	#######	458	365	503	243	412	373	0.94654377	1	326	112.226811								
4	1E+08	4_132842937	80	54	57	82	67	70	0.794947	1	83	56	69	32	70	51	0.42300951	1	64.125	17.7396851					1.3E+08	1.3E+08	0.35764	ID=promoter_37794;
4	1E+08	4_132914081	86	52	163	223	69	193	1.34E-09	#######	37	10	51	16	24	34	0.27690884	1	79.75	75.4448711								

4	1E+08	4_132932820	17	0	7	0	9	4	0.244237	1	39	12	7	3	26	5	0.00174818	0.0978457	10.625	12.8612097								
4	1E+08	4_132958012	16	30	20	14	23	17	0.346113	1	21	4	21	17	13	19	0.44319111	1	17.875	7.39570339					1.3E+08	1.3E+08	0.24316	ID=promoter_37823;
4	1E+08	4_133004354	43	7	249	56	25	153	1.23E-16	#######	17	5	10	39	11	25	0.03752473	1	53.25	81.315523								
4	1E+08	4_133108346	177	95	117	127	136	122	0.097672	1	42	45	0	0	44	0	1.39E-09	3.97E-07	75.375	63.8188004								
4	1E+08	4_133309285	1	10	3	8	6	6	0.962469	1	27	20	7	12	24	10	0.09254106	1	11	8.68496237								
4	1E+08	4_133367780	21	5	18	19	13	19	0.82904	1	42	7	10	20	25	15	0.40601431	1	17.75	11.609725								
4	1E+08	4_133494070	25	14	14	34	20	24	0.997169	1	31	54	26	21	43	24	0.13191129	1	27.375	12.9387292								
4	1E+08	4_133499531	15	65	17	0	40	9	3.70E-06	0.00049	28	51	22	3	40	13	0.00273506	0.1400212	25.125	22.5732933								
4	1E+08	4_133524129	9	13	4	11	11	8	0.5832	1	33	16	2	1	25	2	1.29E-04	0.0113348	11.125	10.3293963					1.3E+08	1.3E+08	0.32911	ID=promoter_37911;
4	1E+08	4_133546395	32	26	8	0	29	4	8.83E-06	0.00107	9	8	30	28	9	29	0.00234316	0.1251016	17.625	12.5804554								
4	1E+08	4_133546693	18	64	8	1	41	5	3.37E-08	#######	23	14	27	0	19	14	0.80779431	1	19.375	20.4795194								
4	1E+08	4_133769023	4	16	29	13	10	21	0.306738	1	72	24	43	6	48	25	0.06027309	1	25.875	22.585947								
4	1E+08	4_133869154	4	25	30	52	15	41	0.015451	0.57677	4	14	47	0	9	24	0.01819934	0.631944	22	19.9785599								
4	1E+08	4_133896284	7	0	4	10	4	7	0.813458	1	18	26	24	1	22	13	0.43054584	1	11.25	10.2085398								
4	1E+08	4_133932685	23	20	44	26	22	35	0.531372	1	31	39	18	6	35	12	0.00825069	0.3370693	25.875	12.1118301								
4	1E+08	4_133939251	35	89	32	19	62	26	2.56E-05	0.00271	33	47	47	24	40	36	0.99172993	1	40.75	21.8092379					1.3E+08	1.3E+08	0.32937	ID=promoter_37977;
4	1E+08	4_133960684	22	49	29	27	36	28	0.259755	1	69	18	81	80	44	81	9.34E-04	0.0596362	46.875	26.5191979					1.3E+08	1.3E+08	0.12685	ID=promoter_37983;
4	1E+08	4_133992499	16	6	11	0	11	6	0.3028	1	0	9	24	17	5	21	0.00386645	0.1846363	10.375	8.4335639					1.3E+08	1.3E+08	0.33221	ID=promoter_37990;
4	1E+08	4_133995301	16	10	17	23	13	20	0.747034	1	7	0	1	0	4	1	0.45332778	1	9.25	8.77903346					1.3E+08	1.3E+08	0.20945	ID=promoter_37991;
4	1E+08	4_134009694	8	32	16	29	20	23	0.997913	1	26	36	56	8	31	32	0.91294871	1	26.375	15.999442					1.3E+08	1.3E+08	0.28164	ID=promoter_37996;
4	1E+08	4_134015568	1	11	7	8	6	8	0.9722	1	1	37	53	19	19	36	0.03633239	1	17.125	18.6274567					1.3E+08	1.3E+08	0.14237	ID=promoter_37998;
4	1E+08	4_134034988	75	29	93	23	52	58	0.965868	1	40	30	15	9	35	12	0.00825069	0.3370693	39.25	29.5816063					1.3E+08	1.3E+08	0.2511	ID=promoter_38003;
4	1E+08	4_134038294	26	110	18	26	68	22	1.37E-07	#######	100	49	80	22	75	51	0.23004046	1	53.875	37.4945234					1.3E+08	1.3E+08	0.33047	ID=promoter_38004;
4	1E+08	4_134041164	173	102	113	163	138	138	0.414853	1	158	67	68	27	113	48	2.06E-05	0.0023874	108.88	52.9782637					1.3E+08	1.3E+08	0.2031	ID=promoter_38005;
4	1E+08	4_134047126	75	37	53	43	56	48	0.277483	1	6	1	34	41	4	38	2.77E-07	5.27E-05	36.25	23.9448772								
4	1E+08	4_134122651	0	24	1	11	12	6	0.213448	1	0	0	10	0	0	5	0.06742467	1	5.75	8.73007936	1E+08	1.3E+08	ID=ENSP00000359581;HS2ST1;NM_012262;	HS2ST1				
4	1E+08	4_134136256	36	20	61	20	28	41	0.662931	1	38	16	66	4	27	35	0.42118875	1	32.625	21.9540917								
4	1E+08	4_134148295	128	86	105	110	107	108	0.544649	1	100	119	105	115	110	110	0.85394297	1	108.5	12.7503501					1.3E+08	1.3E+08	0.18602	ID=promoter_38039;
4	1E+08	4_134167698	13	44	32	17	29	25	0.526429	1	27	43	52	30	35	41	0.59499216	1	32.25	13.5198267								
4	1E+08	4_134230806	0	0	4	3	0	4	0.181755	1	29	0	10	1	15	6	0.20085144	1	5.875	9.93460762					1.3E+08	1.3E+08	0.14508	ID=promoter_38052;

4	1E+08	4_134242734	55	20	61	67	38	64	0.206517	1	15	20	84	17	18	51	1.01E-04	0.0092979	42.375	27.3597071					1.3E+08	1.3E+08	0.45111	ID=promoter_38055;
4	1E+08	4_134244777	218	270	182	177	244	180	1.19E-05	0.00139	83	64	75	27	74	51	0.26270121	1	137	86.2421839								
4	1E+08	4_134267875	10	0	3	4	5	4	0.848147	1	0	18	4	14	9	9	0.98716474	1	6.625	6.65340944	1E+08	1.3E+08	ID=ENSP0000212369;SH3GLB1;;	SH3GLB1				
4	1E+08	4_134618880	9	24	8	13	17	11	0.29617	1	0	1	40	12	1	26	3.52E-06	0.0005061	13.375	13.1141962								
4	1E+08	4_134650826	18	13	9	45	16	27	0.510734	1	0	7	39	7	4	23	5.91E-04	0.0404335	17.25	16.2018517	1E+08	1.3E+08	ID=ENSP0000359596;CLCA2;NM_006536;	CLCA2	1.3E+08	1.3E+08	0.39616	ID=promoter_38085;
4	1E+08	4_134655663	10	39	3	23	25	13	0.050329	1	1	6	4	20	4	12	0.09850366	1	13.25	13.1339255	1E+08	1.3E+08	ID=ENSP0000359596;CLCA2;NM_006536;	CLCA2				
4	1E+08	4_134679082	386	216	423	364	301	394	0.357701	1	242	200	131	94	221	113	1.20E-06	0.0001963	257	121.447225					1.3E+08	1.3E+08	0.6548	ID=promoter_38092;
4	1E+08	4_134682569	1	18	2	10	10	6	0.41703	1	0	9	35	30	5	33	1.10E-05	0.0013789	13.125	13.3997601								
4	1E+08	4_134754415	8	32	6	3	20	5	0.002998	0.15536	2	14	14	1	8	8	0.98858271	1	10	10.2120377								
4	1E+08	4_134806493	1	5	3	0	3	2	0.821455	1	1	7	25	0	4	13	0.06462238	1	5.25	8.3623305					1.3E+08	1.3E+08	0.43451	ID=promoter_38121;
4	1E+08	4_134827480	111	29	92	82	70	87	0.937058	1	20	25	78	38	23	58	1.29E-04	0.0113348	59.375	35.2539258								
4	1E+08	4_134855250	12	2	3	13	7	8	0.998739	1	0	8	12	1	4	7	0.58783259	1	6.375	5.47559521	1E+08	1.3E+08	ID=ENSP0000378219-D2;ODF2L;NM_001007022;	ODF2L	1.3E+08	1.3E+08	0.25051	ID=promoter_38132;
4	1E+08	4_134934721	15	32	10	17	24	14	0.105787	1	33	21	24	25	27	25	0.99999055	1	22.125	8.04340901								
4	1E+08	4_134944041	0	1	3	4	1	4	0.505922	1	0	6	1	26	3	14	0.01844097	0.6375434	5.125	8.69215574					1.3E+08	1.3E+08	0.31938	ID=promoter_38155;
4	1E+08	4_134944212	215	38	114	150	127	132	0.622693	1	25	2	6	2	14	4	0.08931834	1	69	80.8402304					1.3E+08	1.3E+08	0.31938	ID=promoter_38155;
4	1E+08	4_134946336	18	22	12	6	20	9	0.046279	1	70	10	3	9	40	6	1.20E-05	0.001486	18.75	21.5986111								
4	1E+08	4_134958713	223	157	200	147	190	174	0.061062	1	100	65	79	13	83	46	0.01824981	0.6330806	123	71.3041774					1.3E+08	1.3E+08	0.77171	ID=promoter_38158;
4	1E+08	4_134961676	0	25	21	20	13	21	0.660505	1	16	15	0	0	16	0	6.01E-04	0.0409216	12.125	10.4940459								
4	1E+08	4_134977030	7	13	9	10	10	10	0.938234	1	29	12	2	27	21	15	0.7415049	1	13.625	9.5009398					1.3E+08	1.3E+08	0.20049	ID=promoter_38163;
4	1E+08	4_135026950	152	73	50	61	113	56	3.82E-07	#######	0	0	16	3	0	10	0.00454609	0.2107253	44.375	52.3257312								
4	1E+08	4_135214662	5	28	0	12	17	6	0.027279	0.89953	22	17	19	32	20	26	0.52081498	1	16.875	10.9078936	1E+08	1.4E+08	ID=ENSP0000359606;ZNHIT6;NM_017953;	ZNHIT6				
4	1E+08	4_135225171	7	102	15	28	55	22	4.52E-05	0.00446	3	75	59	64	39	62	0.02823855	0.8918846	44.125	36.0493214	1E+08	1.4E+08	ID=ENSP0000359606;ZNHIT6;NM_017953;	ZNHIT6				
4	1E+08	4_135247855	4	34	19	11	19	15	0.513973	1	0	17	12	33	9	23	0.02676526	0.8546242	16.25	12.3259309	1E+08	1.4E+08	ID=ENSP0000359606;ZNHIT6;NM_017953;	ZNHIT6	1.4E+08	1.4E+08	0.17999	ID=promoter_38192;

4	1E+08	4_135252354	12	62	8	9	37	9	1.73E-05	0.00192	3	27	24	3	15	14	0.99983448	1	18.5	19.7049667	1E+08	1.4E+08	ID=ENSP0000359606;ZNHIT6;NM_017953;	ZNHIT6				
4	1E+08	4_135339115	15	14	21	17	15	19	0.975531	1	14	28	14	5	21	10	0.21021716	1	16	6.59003577					1.4E+08	1.4E+08	0.17846	ID=promoter_38212;
4	1E+08	4_135348147	7	25	11	6	16	9	0.196587	1	40	1	16	109	21	63	5.20E-06	0.0007146	26.875	35.4378228								
4	1E+08	4_135356663	868	116	862	531	492	697	0.005773	0.26393	308	111	88	108	210	98	7.79E-08	1.66E-05	374	337.9497					1.4E+08	1.4E+08	0.68023	ID=promoter_38218;
4	1E+08	4_135372088	14	5	5	11	10	8	0.719353	1	0	7	27	2	4	15	0.026797	0.8546242	8.875	8.60958768					1.4E+08	1.4E+08	0.21844	ID=promoter_38223;
4	1E+08	4_135395977	31	36	30	29	34	30	0.52193	1	1	0	34	1	1	18	2.55E-04	0.0200383	20.25	16.3685238								
4	1E+08	4_135426137	38	49	85	41	44	63	0.596403	1	41	59	57	66	50	62	0.30754852	1	54.5	15.8384703					1.4E+08	1.4E+08	0.10161	ID=promoter_38235;
4	1E+08	4_135492350	21	50	22	8	36	15	0.002038	0.11411	66	33	53	29	50	41	0.84384887	1	35.25	19.4330352								
4	1E+08	4_135575308	166	206	142	131	186	137	1.67E-04	0.0139	67	143	130	49	105	90	0.86157107	1	129.25	50.5844414								
4	1E+08	4_135587901	12	146	27	10	79	19	5.62E-11	#######	2	39	42	1	21	22	0.92273004	1	34.875	47.5828225								
4	1E+08	4_135591997	10	14	25	37	12	31	0.05845	1	29	13	17	14	21	16	0.83919704	1	19.875	9.44816687					1.4E+08	1.4E+08	0.16957	ID=promoter_38273;
4	1E+08	4_135598972	30	44	78	118	37	98	8.16E-05	0.00741	105	57	62	45	81	54	0.16912805	1	67.375	30.8727226								
4	1E+08	4_135658150	13	29	6	14	21	10	0.052655	1	1	38	32	4	20	18	0.99585638	1	17.125	14.0452076								
4	1E+08	4_135691594	349	145	175	284	247	230	0.041289	1	113	110	66	66	112	66	0.01308773	0.4886778	163.5	102.699284					1.4E+08	1.4E+08	0.60852	ID=promoter_38286;
4	1E+08	4_135697991	0	50	1	0	25	1	1.94E-06	0.00028	38	9	24	21	24	23	0.99354957	1	17.875	18.8485505	1E+08	1.4E+08	ID=ENSP0000340594;SYDE2;NM_032184;	SYDE2				
4	1E+08	4_135820885	23	11	73	87	17	80	1.30E-07	#######	25	57	26	0	41	13	0.00193653	0.1067879	37.75	30.931953	1E+08	1.4E+08	ID=ENSP0000294664;WDR63;NM_145172;	WDR63	1.4E+08	1.4E+08	0.10479	ID=promoter_38300;
4	1E+08	4_135887542	52	9	21	51	31	36	0.999169	1	51	62	69	35	57	52	0.99650227	1	43.75	20.513062								
4	1E+08	4_135901033	124	25	78	73	75	76	0.667252	1	74	34	70	6	54	38	0.426257	1	60.5	37.2251835					1.4E+08	1.4E+08	0.51985	ID=promoter_38318;
4	1E+08	4_135942585	27	14	22	18	21	20	0.800056	1	17	30	1	21	24	11	0.1422748	1	18.75	8.87613172								
4	1E+08	4_135944698	191	75	176	471	133	324	3.69E-12	#######	50	21	97	43	36	70	0.00108542	0.0671641	140.5	146.969385					1.4E+08	1.4E+08	0.45122	ID=promoter_38326;
4	1E+08	4_135963794	25	67	15	40	46	28	0.020633	0.72153	73	11	34	82	42	58	0.14082722	1	43.375	27.3022108					1.4E+08	1.4E+08	0.26828	ID=promoter_38330;
4	1E+08	4_135977492	10	7	11	7	9	9	0.944235	1	29	11	11	0	20	6	0.03829208	1	10.75	8.25919401								
4	1E+08	4_136009913	22	14	18	6	18	12	0.307258	1	9	34	21	14	22	18	0.92557456	1	17.25	8.73007936					1.4E+08	1.4E+08	0.6519	ID=promoter_38343;
4	1E+08	4_136029289	117	61	157	88	89	123	0.498495	1	0	10	22	36	5	29	8.13E-05	0.0077285	61.375	55.48214								
4	1E+08	4_136033683	320	123	233	155	222	194	0.010919	0.43763	184	239	224	103	212	164	0.21909926	1	197.63	70.8719923								
4	1E+08	4_136044968	75	39	120	68	57	94	0.124978	1	104	90	116	71	97	94	0.95415268	1	85.375	27.443123					1.4E+08	1.4E+08	0.2903	ID=promoter_38356;
4	1E+08	4_136052109	21	77	56	27	49	42	0.325711	1	64	38	19	49	51	34	0.32663423	1	43.875	21.1689631					1.4E+08	1.4E+08	0.37599	ID=promoter_38358;

4	1E+08	4_136080456	16	30	7	2	23	5	6.40E-04	0.04366	1	16	20	19	9	20	0.07989336	1	13.875	9.90580638					1.4E+08	1.4E+08	0.57538	ID=promoter_38363;
4	1E+08	4_136080775	13	11	4	36	12	20	0.626521	1	35	0	19	0	18	10	0.42261869	1	14.75	14.3800457					1.4E+08	1.4E+08	0.57538	ID=promoter_38363;
4	1E+08	4_136082770	2	33	19	23	18	21	0.99986	1	31	55	44	1	43	23	0.09790572	1	26	18.8906628					1.4E+08	1.4E+08	0.57538	ID=promoter_38363;
4	1E+08	4_136106188	22	95	15	26	59	21	4.20E-06	0.00055	73	61	52	3	67	28	0.00135181	0.0804233	43.375	31.8968762								
4	1E+08	4_136129988	6726	2393	4028	4199	4560	4114	2.91E-33	#######	807	733	781	396	770	589	0.0020629	0.1128097	2507.9	2282.2836					1.4E+08	1.4E+08	0.58471	ID=promoter_38379;
4	1E+08	4_136146809	166	283	134	151	225	143	4.42E-08	#######	99	95	109	29	97	69	0.2411361	1	133.25	73.5871689					1.4E+08	1.4E+08	0.45269	ID=promoter_38382;
4	1E+08	4_136198505	10	72	19	7	41	13	6.00E-05	0.00569	31	36	58	8	34	33	0.98250989	1	30.125	24.2571786								
4	1E+08	4_136200390	947	213	651	648	580	650	0.72727	1	235	212	203	93	224	148	0.0057409	0.2548307	400.25	305.733194					1.4E+08	1.4E+08	0.46301	ID=promoter_38393;
4	1E+08	4_136215420	81	82	178	207	82	193	4.89E-07	#######	100	91	94	10	96	52	0.00681961	0.2916212	105.38	61.1834186					1.4E+08	1.4E+08	0.3536	ID=promoter_38399;
4	1E+08	4_136222440	31	42	57	57	37	57	0.432208	1	44	76	67	63	60	65	0.68425704	1	54.625	14.7448538					1.4E+08	1.4E+08	0.28918	ID=promoter_38400;
4	1E+08	4_136228550	48	26	35	29	37	32	0.448082	1	23	13	28	20	18	24	0.50317852	1	27.75	10.4710485					1.4E+08	1.4E+08	0.26971	ID=promoter_38403;
5	2344	5_2344	34	19	8	9	27	9	0.002276	0.124	1	24	22	24	13	23	0.16598565	1	17.625	10.7827574								
5	26055	5_26055	0	39	17	15	20	16	0.517469	1	25	20	20	26	23	23	0.96752545	1	20.25	11.055057								
5	51680	5_51680	20	13	8	5	17	7	0.051092	1	1	11	19	2	6	11	0.39136385	1	9.875	7.21976454					51302	52283	0.26732	ID=promoter_38412;
5	55077	5_55077	78	26	169	138	52	154	1.68E-08	#######	56	40	52	13	48	33	0.41465317	1	71.5	54.8712779								
5	60551	5_60551	7	205	5	15	106	10	1.37E-21	#######	226	164	0	21	195	11	1.68E-33	1.98E-30	80.375	99.3233356					58404	61299	0.40703	ID=promoter_38414;
5	63480	5_63480	607	222	480	524	415	502	0.89786	1	212	172	214	87	192	151	0.31801135	1	314.75	192.005022					62729	64357	0.62845	ID=promoter_38415;
5	78875	5_78875	16	2	29	11	9	20	0.269594	1	0	4	13	33	2	23	6.46E-05	0.0062789	13.5	12.1772622	67402	81537	ID=ENSP00000160874;GTSE1;;	GTSE1	78828	79780	0.33426	ID=promoter_38422;
5	98856	5_98856	43	81	23	45	62	34	0.001283	0.07728	65	48	46	75	57	61	0.73768767	1	53.25	19.084399	88567	109166	ID=ENSP00000370419;TTC38;NM_017931;	TTC38				
5	119423	5_119423	3	24	8	6	14	7	0.165008	1	17	1	25	8	9	17	0.21129426	1	11.5	9.30437685	115309	121707	ID=ENSP00000253255;PKDREJ;NM_006071;	PKDREJ	114292	123071	0.98184	ID=promoter_38431;
5	120595	5_120595	12	8	6	20	10	13	0.970528	1	26	11	47	4	19	26	0.43045343	1	16.75	14.2503133	115309	121707	ID=ENSP00000253255;PKDREJ;NM_006071;	PKDREJ	114292	123071	0.98184	ID=promoter_38431;
5	122868	5_122868	5	40	5	1	23	3	8.06E-05	0.00734	0	0	1	1	0	1	0.58312783	1	6.625	13.637527					114292	123071	0.98184	ID=promoter_38431;
5	149748	5_149748	122	27	92	42	75	67	0.267064	1	34	43	142	19	39	81	1.19E-04	0.0105864	65.125	46.9663633	142394	161039	ID=ENSP00000385523;PPARA;NM_005036;	PPARA	149500	150372	0.21472	ID=promoter_38444;
5	173965	5_173965	0	24	12	15	12	14	0.999907	1	10	26	12	2	18	7	0.13207736	1	12.625	9.21082438								
5	187241	5_187241	0	4	6	27	2	17	0.008084	0.3457	15	12	11	0	14	6	0.26891969	1	9.375	9.00694177	175685	187326	ID=ENSP00000290846;TRMU;NM_018006;	TRMU	186815	188259	0.18901	ID=promoter_38453;
5	219543	5_219543	0	5	3	21	3	12	0.129494	1	29	8	38	13	19	26	0.43045343	1	14.625	13.5323686								

5	220089	5_220089	2	22	6	3	12	5	0.126784	1	4	3	5	4	4	5	0.90483023	1	6.125	6.53425042								
5	223741	5_223741	3	36	1	25	20	13	0.243396	1	12	1	27	6	7	17	0.08351885	1	13.875	13.6322884								
5	260502	5_260502	1	40	10	19	21	15	0.333352	1	1	0	7	3	1	5	0.22510075	1	10.125	13.6322884								
5	263960	5_263960	8	35	11	11	22	11	0.058934	1	1	1	4	5	1	5	0.22510075	1	9.5	11.0324198					263610	264778	0.2441	ID=promoter_38469;
5	267000	5_267000	5	18	4	1	12	3	0.030625	0.98374	1	7	8	1	4	5	0.90483023	1	5.625	5.70557372								
5	276483	5_276483	8	6	11	6	7	9	0.983538	1	0	2	18	2	1	10	0.01766414	0.6154554	6.625	5.82942046					275881	277832	0.4122	ID=promoter_38472;
5	280896	5_280896	0	28	8	4	14	6	0.098789	1	0	1	5	0	1	3	0.56022579	1	5.75	9.45289071					278613	283669	0.77954	ID=promoter_38473;
5	286673	5_286673	23	67	55	26	45	41	0.502494	1	2	13	48	5	8	27	0.00271213	0.139047	29.875	24.1450231					285265	287455	0.35381	ID=promoter_38474;
5	290631	5_290631	106	47	91	70	77	81	0.791187	1	10	25	11	10	18	11	0.54801163	1	46.25	38.655068								
5	570115	5_570115	0	0	2	0	0	1	0.652937	1	0	0	3	35	0	19	3.54E-05	0.003758	5	12.1772622								
5	696836	5_696836	61	92	54	57	77	56	0.022947	0.78666	63	143	157	51	103	104	0.82787479	1	84.75	42.354794	696407	707326	ID=ENSP00000384836;FAM118A;NM_001104595;	FAM118A				
5	923465	5_923465	10	20	1	7	15	4	0.015825	0.58672	5	0	1	1	3	1	0.65196691	1	5.625	6.80204801					922994	924423	0.39616	ID=promoter_38545;
5	959467	5_959467	103	76	80	37	90	59	0.002022	0.11403	117	50	74	29	84	52	0.07033515	1	70.75	30.7420141								
5	964673	5_964673	87	55	65	46	71	56	0.082262	1	80	30	39	9	55	24	0.00692659	0.2949591	51.375	25.9721692					964119	965207	0.26002	ID=promoter_38553;
5	989509	5_989509	26	20	20	10	23	15	0.200003	1	17	14	20	0	16	10	0.61601721	1	15.875	7.97204939								
5	1E+06	5_1150468	1	0	4	7	1	6	0.241978	1	50	0	4	2	25	3	3.93E-04	0.0289246	8.5	16.9368575	1E+06	1244375	ID=ENSP00000337560;PRR5;;	PRR5				
5	1E+06	5_1192682	2764	1867	3311	2461	2316	2886	0.095124	1	1455	1000	1493	710	1228	1102	0.7357487	1	1882.6	897.66251	1E+06	1244375	ID=ENSP00000337560;PRR5;;	PRR5	1188073	1193226	0.9173	ID=promoter_38593;
5	1E+06	5_1204756	238	75	134	161	157	148	0.160924	1	90	5	28	25	48	27	0.11059688	1	94.5	79.4966307	1E+06	1244375	ID=ENSP00000337560;PRR5;;	PRR5	1204296	1205432	0.26486	ID=promoter_38600;
5	1E+06	5_1205157	36	7	13	10	22	12	0.091688	1	49	2	14	5	26	10	0.05079892	1	17	16.5960408	1E+06	1244375	ID=ENSP00000337560;PRR5;;	PRR5	1204296	1205432	0.26486	ID=promoter_38600;
5	1E+06	5_1206653	13	1	10	24	7	17	0.253572	1	17	21	0	23	19	12	0.57792516	1	13.625	9.41029072	1E+06	1244375	ID=ENSP00000337560;PRR5;;	PRR5	1206518	1207176	0.21969	ID=promoter_38601;
5	1E+06	5_1227588	32	24	32	10	28	21	0.295201	1	0	9	30	22	5	26	3.56E-04	0.0266828	19.875	12.1236192	1E+06	1244375	ID=ENSP00000337560;PRR5;;	PRR5	1227345	1228550	0.25526	ID=promoter_38604;
5	1E+06	5_1269546	25	10	23	37	18	30	0.49591	1	52	44	28	35	48	32	0.34885608	1	31.75	13.1339255					1268834	1269849	0.23446	ID=promoter_38608;
5	1E+06	5_134029	50	1	93	63	26	78	9.62E-05	0.00862	0	0	37	12	0	25	1.39E-06	0.0002238	32	34.7233968								
5	1E+06	5_136182	17	35	33	27	26	30	0.998123	1	1	36	20	38	19	29	0.23299331	1	25.875	12.6314517								
5	1E+06	5_142067	6	6	5	2	6	4	0.674789	1	19	5	13	7	12	10	0.96947392	1	7.875	5.46253473								
5	1E+06	5_1443381	195	135	157	210	165	184	0.894847	1	88	122	62	49	105	56	0.00326332	0.163777	127.25	58.9570061					1442483	1443950	0.50568	ID=promoter_38617;
5	1E+06	5_1481382	153	13	157	131	83	144	0.016811	0.6153	31	10	19	0	21	10	0.21021716	1	64.25	69.4730163					1481089	1482019	0.15838	ID=promoter_38622;
5	2E+06	5_1500724	126	25	114	87	76	101	0.712841	1	29	33	31	42	31	37	0.58195082	1	60.875	41.5329043					1500157	1501940	0.25043	ID=promoter_38625;
5	2E+06	5_151559	390	182	303	262	286	283	0.127184	1	80	78	96	114	79	105	0.05195393	1	188.13	117.864859								
5	2E+06	5_155295	18	12	25	30	15	28	0.341844	1	4	8	26	3	6	15	0.10150651	1	15.75	10.511898								
5	2E+06	5_1577158	0	2	78	82	1	80	6.29E-15	#######	0	15	17	1	8	9	0.92351511	1	24.375	34.997704					1576958	1577912	0.20659	ID=promoter_38633;

5	2E+06	5_1580331	3426	2473	3237	3489	2950	3363	0.528276	1	3060	2137	2213	1549	2599	1881	2.53E-15	1.31E-12	2698	706.904924					1579539	1580641	0.26722	ID=promoter_38634;
5	2E+06	5_158121	15	13	19	30	14	25	0.447365	1	13	12	26	18	13	22	0.2175897	1	18.25	6.5846141								
5	2E+06	5_159166	97	78	88	49	88	69	0.04161	1	0	39	32	27	20	30	0.24257255	1	51.25	33.5931541								
5	2E+06	5_1601135	25	0	32	29	13	31	0.09038	1	0	19	1	0	10	1	0.03495212	1	13.25	14.3800457					1600562	1601563	0.21863	ID=promoter_38636;
5	2E+06	5_160826	458	150	402	275	304	339	0.814777	1	38	10	3	19	24	11	0.1422748	1	169.38	185.897005								
5	2E+06	5_163426	213	185	179	118	199	149	1.62E-04	0.01352	78	115	120	66	97	93	0.97348576	1	134.25	52.6979262								
5	2E+06	5_165306	16	1	20	16	9	18	0.416978	1	31	1	14	0	16	7	0.23724011	1	12.375	10.9926924								
5	2E+06	5_166443	7	16	10	5	12	8	0.45534	1	13	45	8	4	29	6	0.00120521	0.0734459	13.5	13.3416641								
5	2E+06	5_175187	45	48	52	34	47	43	0.498196	1	35	13	23	14	24	19	0.87598349	1	33	15.0996689								
5	2E+06	5_1755255	371	159	377	368	265	373	0.075607	1	93	62	77	28	78	53	0.21543412	1	191.88	153.601002					1754496	1755450	0.20659	ID=promoter_38659;
5	2E+06	5_1821476	11	0	8	11	6	10	0.79153	1	0	12	1	0	6	1	0.20114407	1	5.375	5.60452623					1821445	1822187	0.22692	ID=promoter_38670;
5	2E+06	5_1841759	47	20	38	38	34	38	0.979283	1	0	8	18	0	4	9	0.31291743	1	21.125	18.1850606	2E+06	1882593	ID=ENSP00000384913;EFCAB6;;	EFCAB6	1841546	1843058	0.45127	ID=promoter_38676;
5	2E+06	5_188638	42	80	55	33	61	44	0.045895	1	34	17	27	25	26	26	0.96336814	1	39.125	20.1099655								
5	2E+06	5_1920563	10	2	5	14	6	10	0.79153	1	1	0	9	1	1	5	0.22510075	1	5.25	5.1754917					1919327	1922799	0.56261	ID=promoter_38693;
5	2E+06	5_1923379	13	24	0	12	19	6	0.010861	0.43721	0	3	34	25	2	30	1.59E-06	0.0002517	13.875	12.755251					1923209	1923874	0.14386	ID=promoter_38694;
5	2E+06	5_195059	15	29	43	40	22	42	0.174311	1	30	21	23	0	26	12	0.12503864	1	25.125	13.8092878								
5	2E+06	5_1953475	9	0	21	12	5	17	0.090491	1	36	12	8	0	24	4	0.00163693	0.0924179	12.25	11.7686023					1952170	1953683	0.4396	ID=promoter_38699;
5	2E+06	5_1975387	12	42	553	332	27	443	2.27E-67	#######	41	17	250	87	29	169	1.28E-24	1.09E-21	166.75	195.497716					1974421	1975389	0.16647	ID=promoter_38706;
5	2E+06	5_199474	7	16	8	5	12	7	0.325249	1	17	16	16	1	17	9	0.38755874	1	10.75	6.22781778								
5	2E+06	5_199768	6	3	8	26	5	17	0.090491	1	0	10	2	3	5	3	0.8350419	1	7.25	8.25919401								
5	2E+06	5_205022	127	37	91	81	82	86	0.768691	1	86	18	20	54	52	37	0.46718443	1	64.25	38.454983								
5	2E+06	5_205980	9	50	15	12	30	14	0.012913	0.49818	9	15	40	4	12	22	0.15316566	1	19.25	16.507574								
5	2E+06	5_213468	1	9	27	22	5	25	0.005501	0.25445	27	41	70	13	34	42	0.45863117	1	26.25	21.5853786								
5	2E+06	5_214031	391	147	206	136	269	171	1.63E-09	#######	262	171	318	134	217	226	0.47203155	1	220.63	94.8561881								
5	2E+06	5_2150051	28	32	7	39	30	23	0.302742	1	41	1	17	3	21	10	0.21021716	1	21	16.1687529	2E+06	2202276	ID=ENSP00000350833-D2;MPPED2;NM_001584;	MPPED2	2149763	2150549	0.17836	ID=promoter_38744;
5	2E+06	5_2179242	3	79	24	15	41	20	0.004028	0.19746	9	20	22	1	15	12	0.93002578	1	21.625	24.7151631	2E+06	2202276	ID=ENSP00000350833-D2;MPPED2;NM_001584;	MPPED2				
5	2E+06	5_2201799	46	47	56	91	47	74	0.285193	1	120	145	93	21	133	57	3.81E-06	0.0005402	77.375	42.0235903	2E+06	2202276	ID=ENSP00000350833-D2;MPPED2;NM_001584;	MPPED2				
5	2E+06	5_2210513	30	30	38	37	30	38	0.95166	1	61	24	18	0	43	9	5.12E-05	0.0051875	29.75	17.5397508					2210090	2210977	0.19958	ID=promoter_38754;
5	2E+06	5_2249603	17	90	27	13	54	20	2.00E-05	0.00219	69	41	55	24	55	40	0.50389454	1	42	27.3025378					2246050	2249806	0.53013	ID=promoter_38760;
5	2E+06	5_2251052	81	99	34	30	90	32	6.13E-09	#######	67	102	13	51	85	32	4.12E-05	0.0043065	59.625	33.0365058					2250545	2251106	0.16237	ID=promoter_38761;
5	2E+06	5_226198	9	1	11	9	5	10	0.615109	1	3	4	45	1	4	23	5.91E-04	0.0404335	10.375	14.5104642								
5	2E+06	5_2286505	0	12	3	11	6	7	0.999953	1	0	2	1	25	1	13	0.0036419	0.1775497	6.75	8.77903346					2286138	2287547	0.22535	ID=promoter_38769;
5	2E+06	5_2287521	83	20	74	89	52	82	0.245676	1	18	22	8	0	20	4	0.00863789	0.3495868	39.25	36.319023					2286138	2287547	0.22535	ID=promoter_38769;

5	2E+06	5_2321527	668	366	657	464	517	561	0.442346	1	461	500	322	225	481	274	6.78E-10	2.05E-07	457.88	154.346028					2320126	2321711	0.32264	ID=promoter_38772;
5	2E+06	5_2365754	14	4	3	10	9	7	0.713906	1	1	6	3	17	4	10	0.21760186	1	7.25	5.8002463					2365254	2366784	0.25992	ID=promoter_38780;
5	2E+06	5_236754	26	27	68	54	27	61	0.015497	0.57832	57	27	1	1	42	1	1.45E-08	3.49E-06	32.625	25.0994451								
5	3E+06	5_2501791	11	42	28	17	27	23	0.526863	1	32	11	29	10	22	20	0.99799876	1	22.5	11.9163753					2501128	2503202	0.37832	ID=promoter_38799;
5	3E+06	5_251089	7	11	6	2	9	4	0.244237	1	18	26	1	4	22	3	0.001462	0.085933	9.375	8.65097022								
5	3E+06	5_263091	6	0	1	7	3	4	0.986002	1	48	7	18	0	28	9	0.01512239	0.5480081	10.875	16.1106664								
5	3E+06	5_264907	80	64	94	204	72	149	3.24E-04	0.02475	121	80	124	69	101	97	0.96956292	1	104.5	45.9254054								
5	3E+06	5_2651619	14	1	5	26	8	16	0.45954	1	39	18	6	1	29	4	1.92E-04	0.0158648	13.75	13.4562784					2651285	2652525	0.14591	ID=promoter_38807;
5	3E+06	5_2716927	7	38	2	2	23	2	2.40E-05	0.00257	16	44	23	2	30	13	0.06382438	1	16.75	16.8247267	3E+06	2741739	ID=ENSP0000378835-D2;ARFGAP2;;	ARFGAP2	2716691	2717784	0.1382	ID=promoter_38817;
5	3E+06	5_2720600	3	13	11	4	8	8	0.950274	1	0	16	12	20	8	16	0.19094317	1	9.875	6.91659495	3E+06	2741739	ID=ENSP0000378835-D2;ARFGAP2;;	ARFGAP2				
5	3E+06	5_2727701	3	6	4	19	5	12	0.389128	1	0	18	0	3	9	2	0.1390433	1	6.625	7.59581652	3E+06	2741739	ID=ENSP0000378835-D2;ARFGAP2;;	ARFGAP2	2727359	2728170	0.17558	ID=promoter_38820;
5	3E+06	5_277648	27	77	25	27	52	26	0.00124	0.07563	55	55	89	30	55	60	0.68321005	1	48.125	24.9424337								
5	3E+06	5_3026545	12	16	24	19	14	22	0.691767	1	106	108	97	51	107	74	0.15086624	1	54.125	42.7799603	3E+06	3028950	ID=ENSP0000374620;CYP2D6;;	CYP2D6				
5	3E+06	5_303070	1748	1098	1442	1214	1423	1328	1.48E-08	#######	568	594	680	283	581	482	0.19668562	1	953.38	501.544169								
5	3E+06	5_304168	441	138	414	323	290	369	0.583833	1	141	160	102	33	151	68	2.87E-06	0.0004246	219	152.379226								
5	3E+06	5_3042222	27	1	15	34	14	25	0.447365	1	7	1	14	1	4	8	0.43697044	1	12.5	12.5811651					3041848	3043062	0.19585	ID=promoter_38847;
5	3E+06	5_330358	113	72	101	70	93	86	0.281093	1	35	21	58	39	28	49	0.02425357	0.7948115	63.625	32.1155837								
5	3E+06	5_332285	28	58	12	15	43	14	4.87E-05	0.00473	41	17	13	37	29	25	0.96515949	1	27.625	16.5610343								
5	3E+06	5_333928	111	11	95	58	61	77	0.91234	1	49	49	51	19	49	35	0.49765999	1	55.375	33.9408624								
5	3E+06	5_3364904	161	68	104	118	115	111	0.340796	1	66	60	24	2	63	13	4.43E-07	8.03E-05	75.375	51.2276083					3364425	3365422	0.19277	ID=promoter_38870;
5	4E+06	5_352239	11	26	24	10	19	17	0.718296	1	9	14	4	11	12	8	0.76853133	1	13.625	7.57698583								
5	4E+06	5_3553483	86	55	169	86	71	128	0.013788	0.52738	16	64	78	49	40	64	0.02333576	0.7680341	75.375	44.342619	4E+06	3554734	ID=ENSP0000385315;POLR3H;;	POLR3H				
5	4E+06	5_3568184	4554	3289	9992	10600	3922	####	0	0	3344	2255	11659	2622	2800	7141	0	0	6039.4	3982.79557	4E+06	3582373	ID=ENSP0000379769;ACO2;;	ACO2				
5	4E+06	5_3603247	137	92	108	106	115	107	0.224571	1	128	80	48	27	104	38	2.35E-06	0.0003566	90.75	37.9200286					3602177	3604045	0.3878	ID=promoter_38886;
5	4E+06	5_389990	6	36	15	6	21	11	0.084122	1	1	9	39	21	5	30	4.95E-05	0.0050484	16.625	14.2722258								
5	4E+06	5_400769	0	7	8	9	4	9	0.54411	1	64	29	23	52	47	38	0.82124279	1	24	23.1640115								
5	4E+06	5_4240917	26	60	10	25	43	18	6.41E-04	0.04369	38	22	36	56	30	46	0.09558038	1	34.125	17.0916646	4E+06	4243366	ID=ENSP0000345841-D2;MKL2;;	MKL2	4240678	4241294	0.1627	ID=promoter_38907;
5	4E+06	5_4313789	20	63	3	3	42	3	1.57E-09	#######	2	0	16	0	1	8	0.04988758	1	13.375	21.4338684	4E+06	4313978	ID=ENSP0000216194;ADSL;NM_000026;	ADSL	4313182	4314980	0.39822	ID=promoter_38913;
5	4E+06	5_4338608	17	4	40	55	11	48	1.36E-04	0.01165	14	14	8	3	14	6	0.26891969	1	19.375	18.4850326					4338341	4339391	0.17288	ID=promoter_38916;
5	4E+06	5_439451	223	167	86	108	195	97	9.98E-12	#######	127	87	39	71	107	55	0.0014998	0.0875137	113.5	58.4074359								

5	5E+06	5_4576330	19	67	40	20	43	30	0.087253	1	127	83	87	63	105	75	0.2241662	1	63.25	36.6479389	5E+06	4577008	ID=ENSP00000330432;FAM83F;NM_138435;	FAM83F	4576186	4577789	0.5648	ID=promoter_38928;
5	5E+06	5_492735	20	35	27	34	28	31	0.975765	1	17	21	26	42	19	34	0.06506114	1	27.75	8.61477137								
5	5E+06	5_4937387	11	0	21	20	6	21	0.046061	1	98	79	18	3	89	11	1.05E-12	4.37E-10	31.25	36.5034245					4936645	4937621	0.17773	ID=promoter_38944;
5	5E+06	5_500103	119	57	3	12	88	8	3.28E-18	#######	33	22	17	3	28	10	0.02694098	0.8576836	33.25	38.8945461								
5	5E+06	5_502866	11	1	19	22	6	21	0.046061	1	0	5	11	2	3	7	0.3829009	1	8.875	8.33987838								
5	5E+06	5_503924	21	22	18	14	22	16	0.340123	1	46	37	7	2	42	5	1.82E-06	0.0002865	20.875	14.6232447								
5	5E+06	5_5064355	13	4	12	14	9	13	0.89076	1	5	3	6	12	4	9	0.31291743	1	8.625	4.53360468					5063398	5065716	0.64672	ID=promoter_38951;
5	5E+06	5_508079	356	90	177	198	223	188	0.003648	0.18279	154	63	86	40	109	63	0.01067966	0.4139111	145.5	101.901914								
5	5E+06	5_513076	98	59	201	166	79	184	1.34E-06	0.0002	122	78	40	14	100	27	1.09E-08	2.67E-06	97.25	63.4096658								
5	5E+06	5_514204	7	7	7	2	7	5	0.693374	1	6	12	4	26	9	15	0.36943449	1	8.875	7.49166203								
5	5E+06	5_5215537	354	228	240	200	291	220	4.64E-06	0.0006	179	135	197	146	157	172	0.31750653	1	209.88	68.5678757					5215057	5217114	0.52026	ID=promoter_38963;
5	5E+06	5_538324	93	40	50	60	67	55	0.143123	1	55	14	9	0	35	5	3.88E-05	0.0040735	40.125	31.1009302								
5	5E+06	5_5387453	243	147	248	263	195	256	0.495054	1	76	42	49	58	59	54	0.99769331	1	140.75	97.2122126					5387100	5387884	0.09261	ID=promoter_38974;
5	5E+06	5_540248	137	27	49	91	82	70	0.146246	1	54	14	19	0	34	10	0.00342551	0.1709558	48.875	45.5801884								
5	5E+06	5_5410844	159	38	203	169	99	186	6.73E-04	0.04537	49	94	15	32	72	24	3.48E-05	0.0037136	94.875	72.6742979					5409022	5411130	0.71356	ID=promoter_38976;
5	5E+06	5_5443788	224	113	194	255	169	225	0.460529	1	137	94	201	58	116	130	0.33078549	1	159.5	69.131345					5443191	5444173	0.18406	ID=promoter_38978;
5	5E+06	5_5450065	4	18	3	10	11	7	0.438207	1	1	0	5	1	1	3	0.56022579	1	5.25	6.04152299	5E+06	5450262	ID=ENSP00000216068;DNAL4;NM_005740;	DNAL4	5449458	5451390	0.38851	ID=promoter_38980;
5	5E+06	5_546492	46	45	35	31	46	33	0.093519	1	85	59	42	15	72	29	4.96E-04	0.0353042	44.75	20.7071416								
5	6E+06	5_553729	0	9	0	5	5	3	0.645778	1	54	0	0	1	27	1	1.48E-05	0.0017848	8.625	18.6236217								
5	6E+06	5_6091630	37	39	40	75	38	58	0.449052	1	0	0	7	0	0	4	0.11562589	1	24.75	27.421316	6E+06	6136730	ID=ENSP00000333142-D2;PLA2G6;NM_003560;	PLA2G6				
5	6E+06	5_6238224	30	26	39	67	28	53	0.117855	1	1	5	15	0	3	8	0.26303803	1	22.875	22.8375474	6E+06	6283395	ID=ENSP00000333142;PLA2G6;NM_003560;	PLA2G6				
5	6E+06	5_6281710	247	101	195	191	174	193	0.866793	1	89	64	73	14	77	44	0.0353607	1	121.75	79.8869737	6E+06	6283395	ID=ENSP00000333142;PLA2G6;NM_003560;	PLA2G6	6281478	6281981	0.20061	ID=promoter_39030;
5	6E+06	5_6285773	382	187	311	268	285	290	0.233552	1	298	243	324	143	271	234	0.72831347	1	269.5	77.158464	6E+06	6303324	ID=ENSP00000371085;BAIAP2L2;NM_025045;	BAIAP2L2				
5	6E+06	5_638375	13	5	3	10	9	7	0.713906	1	0	3	1	5	2	3	0.86792893	1	5	4.44007722								
5	6E+06	5_638767	89	44	47	110	67	79	0.999504	1	0	7	14	0	4	7	0.58783259	1	38.875	41.9538437								
5	6E+06	5_6459549	16	3	7	4	10	6	0.41703	1	15	4	24	28	10	26	0.01509923	0.5480081	12.625	9.67969155					6459324	6460189	0.18612	ID=promoter_39051;
5	7E+06	5_6527247	24	63	3	7	44	5	6.20E-09	#######	1	11	16	20	6	18	0.03091566	0.954015	18.125	19.8309823	7E+06	6541601	ID=ENSP00000262832;EIF3L;;	EIF3L				
5	7E+06	5_702582	123	31	119	56	77	88	0.986221	1	12	23	22	1	18	12	0.67374005	1	48.375	47.5452792								
5	7E+06	5_7027358	7	13	2	2	10	2	0.03464	1	4	12	4	0	8	2	0.20550608	1	5.5	4.78091444					7026870	7027791	0.15664	ID=promoter_39092;
5	7E+06	5_7061621	35	10	22	26	23	24	0.922719	1	56	37	75	37	47	56	0.44534269	1	37.25	20.2960235	7E+06	7068670	ID=ENSP00000385828;TST;;	TST				
5	7E+06	5_711617	100	77	160	87	89	124	0.463304	1	23	31	32	20	27	26	0.99047429	1	66.25	49.1346546								

5	7E+06	5_713817	6	30	24	49	18	37	0.141403	1	21	34	37	4	28	21	0.76300557	1	25.625	15.3151419								
5	8E+06	5_763098	0	14	5	6	7	6	0.851931	1	14	24	3	14	19	9	0.23949138	1	10	7.87400787								
5	8E+06	5_7649729	223	77	230	158	150	194	0.667622	1	109	49	57	34	79	46	0.04072886	1	117.13	77.8853324					7649048	7651067	0.47005	ID=promoter_39121;
5	8E+06	5_776625	1	24	4	12	13	8	0.344217	1	16	0	9	0	8	5	0.78486764	1	8.25	8.69729022								
5	8E+06	5_781154	206	113	162	163	160	163	0.447816	1	91	45	68	22	68	45	0.21157912	1	108.75	64.1644094								
5	8E+06	5_787527	13	13	24	21	13	23	0.490912	1	18	9	5	9	14	7	0.39930956	1	14	6.52467843								
5	8E+06	5_789805	75	172	90	136	124	113	0.15054	1	212	192	204	84	202	144	0.05406399	1	145.63	56.8605751								
5	8E+06	5_790476	15	0	8	10	8	9	0.996316	1	0	23	7	1	12	4	0.18067673	1	8	8.07111251								
5	8E+06	5_798538	491	2572	524	684	1532	604	########	#######	1987	1986	2249	1517	1987	1883	0.79146943	1	1501.3	829.831265								
5	8E+06	5_805527	1	23	3	5	12	4	0.066899	1	2	0	6	2	1	4	0.36109265	1	5.25	7.44023809								
5	8E+06	5_819304	1	52	5	19	27	12	0.014569	0.54886	0	19	23	2	10	13	0.72167512	1	15.125	17.5453494								
5	8E+06	5_837852	14	17	13	3	16	8	0.127562	1	23	1	17	28	12	23	0.11381058	1	14.5	9.13392421								
5	9E+06	5_859160	12	9	4	7	11	6	0.3028	1	20	16	23	1	18	12	0.67374005	1	11.5	7.72750191								
5	9E+06	5_8904312	1387	987	845	624	1187	735	2.43E-42	#######	915	812	401	266	864	334	2.32E-43	3.85E-40	779.63	351.922044					8903585	8904738	0.19541	ID=promoter_39176;
5	9E+06	5_916332	177	123	155	148	150	152	0.445225	1	112	90	146	44	101	95	0.99485916	1	124.38	42.3081806								
5	9E+06	5_923804	144	114	154	143	129	149	0.991844	1	44	74	127	35	59	81	0.06955586	1	104.38	47.0711847								
5	1E+07	5_966056	2	4	6	7	3	7	0.596562	1	21	2	13	0	12	7	0.61698677	1	6.875	6.97828264								
5	1E+07	5_985895	10	19	1	1	15	1	6.51E-04	0.04418	3	17	8	3	10	6	0.69729498	1	7.75	7.1063352								
5	1E+07	5_987441	15	7	9	11	11	10	0.842816	1	18	13	12	5	16	9	0.48000706	1	11.25	4.23421438								
5	1E+07	5_995058	91	81	36	25	86	31	1.88E-08	#######	60	56	184	2	58	93	0.00409718	0.1941611	66.875	55.5246084								
5	1E+07	5_100246	4	3	15	4	4	10	0.424038	1	0	3	28	4	2	16	0.00248877	0.1311269	7.625	9.33407735								
5	1E+07	5_103457	10	5	9	1	8	5	0.532535	1	14	12	25	7	13	16	0.74830213	1	10.375	7.17012652								
5	1E+07	5_104965	300	166	292	215	233	254	0.721816	1	0	31	7	43	16	25	0.25669436	1	131.75	127.076748								
5	1E+07	5_108072	11	25	8	5	18	7	0.033499	1	5	10	6	2	8	4	0.59180275	1	9	7.09124208								
5	1E+07	5_11019661	5026	3908	5082	5084	4467	5083	0.33473	1	2987	2415	2894	1404	2701	2149	8.23E-07	0.0001402	3600	1395.28625					1.1E+07	1.1E+07	0.35533	ID=promoter_39214;
5	1E+07	5_112422	6	20	19	8	13	14	0.975695	1	39	8	20	37	24	29	0.62889415	1	19.625	12.7272205								
5	1E+07	5_11842965	1	0	8	5	1	7	0.163779	1	15	7	20	1	11	11	0.98433491	1	7.125	7.16015762	1E+07	1.2E+07	ID=ENSP00000376585;RFX4;NM_213594;	RFX4				
5	1E+07	5_119862	30	49	42	32	40	37	0.580086	1	38	19	131	24	29	78	1.91E-06	0.0002984	45.625	35.8127571								
5	1E+07	5_120362	10	30	20	16	20	18	0.715835	1	1	3	9	1	2	5	0.46647814	1	11.25	10.2504355								
5	1E+07	5_125302	23	21	11	10	22	11	0.058934	1	7	13	8	0	10	4	0.34296513	1	11.625	7.48212155								
5	1E+07	5_127210	28	0	6	6	14	6	0.098789	1	0	12	2	0	6	1	0.20114407	1	6.75	9.55809306								
5	1E+07	5_128181	19	20	11	9	20	10	0.076235	1	2	28	17	3	15	10	0.7195765	1	13.625	8.97516414								
5	1E+07	5_128365	13	0	19	18	7	19	0.148295	1	0	0	0	0	0	0	1	1	6.25	8.79529095								
5	1E+07	5_129002	36	17	23	14	27	19	0.225928	1	0	0	7	0	0	4	0.11562589	1	12.125	12.9993132								
5	1E+07	5_129558	1	52	1	6	27	4	2.68E-05	0.00281	9	22	18	12	16	15	0.99951315	1	15.125	16.6685118								
5	1E+07	5_129934	10	38	75	27	24	51	0.05144	1	0	5	12	1	3	7	0.3829009	1	21	25.4670656								
5	1E+07	5_134946	11	2	4	0	7	2	0.160905	1	30	1	10	8	16	9	0.48000706	1	8.25	9.72111105								
5	1E+07	5_136426	115	72	74	59	94	67	0.007052	0.30636	21	57	46	30	39	38	0.97643104	1	59.25	29.2464894								
5	1E+07	5_137263	1	33	0	4	17	2	7.31E-04	0.04855	19	0	9	1	10	5	0.51906437	1	8.375	11.8796044								
5	1E+07	5_13936589	397	137	329	323	267	326	0.888547	1	153	92	163	118	123	141	0.22617894	1	214	116.46336					1.4E+07	1.4E+07	0.25707	ID=promoter_39313;
5	1E+07	5_14034692	964	539	727	742	752	735	0.00203	0.11403	830	647	527	207	739	367	3.73E-22	2.88E-19	647.88	229.614856	1E+07	1.4E+07	ID=ENSP00000257860;PRPH;NM_006262;	PRPH	1.4E+07	1.4E+07	0.54308	ID=promoter_39316;
5	1E+07	5_141271	6	53	9	29	30	19	0.102296	1	30	39	52	10	35	31	0.98304591	1	28.5	18.8755322								
5	1E+07	5_141499	527	192	415	400	360	408	0.893221	1	208	140	70	43	174	57	9.29E-12	3.45E-09	249.38	176.848553								
5	1E+07	5_14218522	154	83	66	112	119	89	0.005282	0.24537	132	86	38	13	109	26	1.69E-10	5.46E-08	85.5	46.9893605					1.4E+07	1.4E+07	0.51663	ID=promoter_39326;
5	1E+07	5_14339603	198	30	117	69	114	93	0.032909	1	34	22	85	1	28	43	0.11009196	1	69.5	64.1181944					1.4E+07	1.4E+07	0.52013	ID=promoter_39329;
5	1E+07	5_14418257	68	68	68	30	68	49	0.031826	1	2	0	37	2	1	20	8.76E-05	0.0082396	34.375	30.9282003	1E+07	1.4E+07	ID=ENSP00000321951;FAIM2;NM_012306;	FAIM2				
5	1E+07	5_14666936	850	274	666	518	562	592	0.188604	1	280	306	194	126	293	160	3.61E-07	6.74E-05	401.75	251.787751					1.5E+07	1.5E+07	0.49353	ID=promoter_39343;
5	1E+07	5_14733675	126	29	73	100	78	87	0.949241	1	41	29	30	35	35	33	0.99757329	1	57.875	37.6010543					1.5E+07	1.5E+07	0.4588	ID=promoter_39346;

5	1E+07	5_14943813	186	139	296	380	163	338	1.09E-08	#######	146	59	135	176	103	156	4.88E-04	0.0348216	189.63	101.608404					1.5E+07	1.5E+07	0.56197	ID=promoter_39348;
5	1E+07	5_14975663	313	106	305	282	210	294	0.146203	1	109	91	101	45	100	73	0.29806122	1	169	110.617746					1.5E+07	1.5E+07	0.5015	ID=promoter_39349;
5	2E+07	5_15234657	37	13	16	81	25	49	0.10751	1	11	10	6	0	11	3	0.13591373	1	21.75	26.2610521	2E+07	1.5E+07	ID=ENSP0000376465-D2;GALNT3;NM_004482;	GALNT3				
5	2E+07	5_155009	22	7	21	15	15	18	0.997876	1	7	4	4	3	6	4	0.87665919	1	10.375	7.81824789								
5	2E+07	5_158650	93	49	50	49	71	50	0.020206	0.71359	14	20	30	1	17	16	0.99905703	1	38.25	28.6443712								
5	2E+07	5_158870	5	9	4	7	7	6	0.851931	1	3	20	24	22	12	23	0.11381058	1	11.75	8.74642784								
5	2E+07	5_159091	23	11	22	16	17	19	0.989587	1	0	1	0	0	1	0	0.62906424	1	9.125	10.1761275								
5	2E+07	5_159322	367	249	563	623	308	593	9.26E-12	#######	126	130	133	55	128	94	0.22818268	1	280.75	215.377907								
5	2E+07	5_161406	23	9	12	17	16	15	0.822707	1	0	0	10	4	0	7	0.02292696	0.7568963	9.375	8.0700062								
5	2E+07	5_165263	434	196	145	118	315	132	4.39E-24	#######	116	21	3	23	69	13	3.80E-08	8.61E-06	132	139.777374								
5	2E+07	5_167399	204	392	273	197	298	235	2.77E-05	0.00289	514	413	539	208	464	374	0.13145637	1	342.5	140.772359								
5	2E+07	5_169337	12	3	5	1	8	3	0.206144	1	0	2	18	15	1	17	4.35E-04	0.0314643	7	6.96932052								
5	2E+07	5_169929	48	12	29	90	30	60	0.054164	1	13	36	28	35	25	32	0.47866995	1	36.375	24.7209425								
5	2E+07	5_17053534	328	167	360	327	248	344	0.131224	1	167	166	105	29	167	67	1.91E-08	4.52E-06	206.13	119.17147	2E+07	1.7E+07	ID=ENSP0000334188;PFDN5;NM_002624;	PFDN5	1.7E+07	1.7E+07	0.25537	ID=promoter_39434;
5	2E+07	5_17084277	68	97	104	156	83	130	0.118512	1	42	9	30	15	26	23	0.98669459	1	65.125	51.0250289					1.7E+07	1.7E+07	0.20537	ID=promoter_39437;
5	2E+07	5_170898	51	46	55	55	49	55	0.97521	1	40	26	9	3	33	6	2.33E-04	0.0188551	35.625	20.6324121								
5	2E+07	5_173624	33	15	20	33	24	27	0.98899	1	0	14	0	10	7	5	0.9051169	1	15.625	12.7943905								
5	2E+07	5_17462576	244	97	113	115	171	114	1.35E-05	0.00155	92	92	67	5	92	36	3.58E-05	0.0037948	103.13	66.8718123					1.7E+07	1.7E+07	0.20254	ID=promoter_39448;
5	2E+07	5_174762	584	250	590	415	417	503	0.914625	1	306	186	259	165	246	212	0.73825794	1	344.38	168.041608								
5	2E+07	5_17521854	601	249	503	292	425	398	0.005343	0.24784	170	171	203	162	171	183	0.40143716	1	293.88	167.404077					1.8E+07	1.8E+07	0.2962	ID=promoter_39452;
5	2E+07	5_177972	29	62	36	30	46	33	0.093519	1	18	22	67	19	20	43	0.00596609	0.2624325	35.375	19.0032892								
5	2E+07	5_17804168	5	20	3	4	13	4	0.041893	1	0	19	51	1	10	26	0.01509923	0.5480081	12.875	17.2662966	2E+07	1.8E+07	ID=ENSP0000209875;CBX5;NM_012117;	CBX5				
5	2E+07	5_181054	5	17	15	9	11	12	0.985018	1	24	18	10	3	21	7	0.05007015	1	12.625	7.11010348								
5	2E+07	5_18107824	1088	836	984	845	962	915	3.23E-05	0.00332	785	467	256	384	626	320	1.66E-17	1.00E-14	705.63	299.755585					1.8E+07	1.8E+07	0.19335	ID=promoter_39489;
5	2E+07	5_182015	15	42	20	7	29	14	0.018844	0.67198	101	30	42	27	66	35	0.02569593	0.8317701	35.5	29.1547595								
5	2E+07	5_18292039	526	383	398	286	455	342	2.91E-09	#######	170	179	192	52	175	122	0.0528931	1	273.25	154.34169					1.8E+07	1.8E+07	0.255	ID=promoter_39495;
5	2E+07	5_187135	20	37	9	9	29	9	8.94E-04	0.05763	25	56	19	0	41	10	2.49E-04	0.0198398	21.875	17.8280477								
5	2E+07	5_195111	198	132	318	308	165	313	2.97E-06	0.0004	22	123	55	110	73	83	0.44393966	1	158.25	108.89674								
5	2E+07	5_21380876	20	29	24	11	25	18	0.280499	1	84	21	3	6	53	5	1.36E-08	3.29E-06	24.75	25.577613	2E+07	2.1E+07	ID=ENSP0000333667;SHMT2;NM_005412;	SHMT2				
5	2E+07	5_21387635	191	110	200	178	151	189	0.837726	1	139	29	66	26	84	46	0.01478111	0.5381157	117.38	70.9566014					2.1E+07	2.1E+07	0.14054	ID=promoter_39567;
5	2E+07	5_214410	106	84	123	114	95	119	0.892143	1	54	45	48	23	50	36	0.51063368	1	74.625	37.1096349								
5	2E+07	5_21526812	26	76	25	29	51	27	0.002775	0.14683	10	72	78	10	41	44	0.79631464	1	40.75	29.5235983	2E+07	2.2E+07	ID=ENSP0000300134;STAT6;NM_003153;	STAT6				
5	2E+07	5_21827617	994	718	526	447	856	487	1.41E-37	#######	842	626	356	69	734	213	8.97E-55	1.71E-51	572.25	290.964284					2.2E+07	2.2E+07	0.19538	ID=promoter_39600;
5	2E+07	5_21903424	286	158	200	133	222	167	7.06E-05	0.00657	82	78	101	3	80	52	0.13437556	1	130.13	86.1948581					2.2E+07	2.2E+07	0.2549	ID=promoter_39609;
5	2E+07	5_224529	745	266	431	362	506	397	1.21E-08	#######	16	0	18	1	8	10	0.81871774	1	229.88	272.665906								

5	2E+07	5_23129775	3	2	82	67	3	75	7.46E-13	#######	2	9	25	2	6	14	0.1466131	1	24	32.3640012					2.3E+07	2.3E+07	0.52665	ID=promoter_39626;
5	2E+07	5_232298	39	33	7	8	36	8	1.18E-05	0.00138	27	49	28	18	38	23	0.26660526	1	26.125	14.64277								
5	3E+07	5_256220	33	94	14	11	64	13	5.46E-10	#######	70	67	64	44	69	54	0.64692807	1	49.625	29.1838385								
5	3E+07	5_278486	74	97	62	62	86	62	0.012874	0.49814	123	74	77	10	99	44	1.82E-04	0.015179	72.375	32.2974457								
5	3E+07	5_279254	36	34	64	54	35	59	0.232101	1	48	48	36	33	48	35	0.5564231	1	44.125	11.2178111								
5	3E+07	5_285234	8	16	3	14	12	9	0.592799	1	45	4	9	0	25	5	0.00263335	0.136745	12.375	14.2521928								
5	3E+07	5_28767156	250	22	258	95	136	177	0.65965	1	81	70	66	16	76	41	0.0183238	0.635493	107.25	94.5859398					2.9E+07	2.9E+07	0.65625	ID=promoter_39664;
5	3E+07	5_29505106	179	209	156	179	194	168	0.015356	0.57396	147	129	226	132	138	179	0.0118292	0.4495627	169.63	35.1890304	3E+07	3E+07	ID=ENSP0000312274;MSRB3;NM_001031679;	MSRB3				
5	3E+07	5_296436	0	0	11	1	0	6	0.077487	1	40	8	14	1	24	8	0.03264408	0.9940318	9.375	13.5218078								
5	3E+07	5_310610	177	55	144	127	116	136	0.999993	1	19	3	20	43	11	32	0.00257396	0.1347203	73.5	66.1383831								
5	3E+07	5_311488	116	379	107	141	248	124	1.38E-14	#######	304	369	393	52	337	223	4.53E-04	0.0326835	232.63	142.047213								
5	3E+07	5_312318	19	28	38	22	24	30	0.97337	1	27	7	27	0	17	14	0.9475482	1	21	12.2591074								
5	3E+07	5_313761	24	79	8	17	52	13	2.75E-07	#######	11	18	10	16	15	13	0.98432481	1	22.875	23.25288								
5	3E+07	5_319013	35	28	11	29	32	20	0.080425	1	74	14	47	5	44	26	0.18536972	1	30.375	22.3474991								
5	3E+07	5_320789	73	32	28	37	53	33	0.014749	0.55477	16	51	24	22	34	23	0.5031903	1	35.375	18.5621543								
5	3E+07	5_32104385	9944	8761	10283	11720	9353	####	0.979613	1	9380	8466	17786	4435	8923	11111	5.82E-95	1.93E-91	10097	3757.47341					3.2E+07	3.2E+07	0.43572	ID=promoter_39699;
5	3E+07	5_32310230	178	92	120	87	135	104	0.005152	0.24041	42	36	39	0	39	20	0.09202448	1	74.25	56.8123478					3.2E+07	3.2E+07	0.44036	ID=promoter_39702;
5	3E+07	5_326338	29	28	19	37	29	28	0.762896	1	12	15	7	27	14	17	0.75475782	1	21.75	10.1242284								
5	3E+07	5_326635	39	115	83	87	77	85	0.928299	1	109	170	160	68	140	114	0.58909933	1	103.88	44.543518								
5	3E+07	5_329921	0	28	10	20	14	15	0.970741	1	47	26	8	19	37	14	0.01279999	0.4795643	19.75	14.5086181								
5	3E+07	5_33569614	38	25	10	9	32	10	4.55E-04	0.03273	0	56	12	2	28	7	0.00393447	0.1875084	19	19.4422221	3E+07	3.4E+07	ID=ENSP0000261266;PTPRB;NM_002837;	PTPRB				
5	3E+07	5_337225	178	67	166	122	123	144	0.999881	1	128	84	63	16	106	40	3.57E-06	0.0005113	103	55.2991863								
5	3E+07	5_337385	6	40	18	7	23	13	0.098913	1	21	8	7	40	15	24	0.24434768	1	18.375	14.4512728								
5	3E+07	5_337506	70	12	49	29	41	39	0.643788	1	50	20	31	26	35	29	0.90474267	1	35.875	18.9995301								
5	3E+07	5_343875	151	94	158	104	123	131	0.744029	1	20	26	45	37	23	41	0.03826354	1	79.375	55.3996841								
5	3E+07	5_345293	271	148	290	271	210	281	0.351996	1	13	40	22	0	27	11	0.06118138	1	131.88	128.736095								
5	3E+07	5_34660470	4	83	14	22	44	18	4.24E-04	0.03084	99	77	64	19	88	42	0.00150431	0.0877408	47.75	36.9004452	3E+07	3.5E+07	ID=ENSP0000368017;ZFC3H1;NM_144982;	ZFC3H1				
5	3E+07	5_34664738	6	40	11	23	23	17	0.346113	1	15	40	7	23	28	15	0.22181264	1	20.625	13.5534656	3E+07	3.5E+07	ID=ENSP0000368017;ZFC3H1;NM_144982;	ZFC3H1				
5	3E+07	5_34667430	5	43	5	2	24	4	1.39E-04	0.01187	23	9	0	2	16	1	0.00230968	0.1234682	11.125	14.7690555	3E+07	3.5E+07	ID=ENSP0000368017;ZFC3H1;NM_144982;	ZFC3H1				
5	3E+07	5_34675257	15	49	28	20	32	24	0.247982	1	0	14	13	1	7	7	0.99000271	1	17.5	15.7025931	3E+07	3.5E+07	ID=ENSP0000368017;ZFC3H1;NM_144982;	ZFC3H1				
5	3E+07	5_348117	12	16	20	22	14	21	0.774664	1	1	27	7	0	14	4	0.08931834	1	13.125	9.89137431								
5	4E+07	5_355387	76	41	91	56	59	74	0.928938	1	86	63	52	33	75	43	0.04008846	1	62.25	20.8103683								
5	4E+07	5_377127	10	1	6	5	6	6	0.962469	1	0	0	17	22	0	20	2.07E-05	0.0023913	7.625	8.1929848								
5	4E+07	5_387381	23	53	28	14	38	21	0.018051	0.65084	39	63	67	62	51	65	0.23221759	1	43.625	20.4096441								
5	4E+07	5_392627	21	33	37	21	27	29	0.94719	1	124	60	15	42	92	29	7.36E-07	0.0001271	44.125	35.3126343								
5	4E+07	5_40212444	43	0	29	45	22	37	0.404342	1	20	26	22	1	23	12	0.26207533	1	23.25	16.6968774					4E+07	4E+07	0.43274	ID=promoter_39738;
5	4E+07	5_409985	6	2	5	0	4	3	0.840045	1	23	8	10	2	16	6	0.14768941	1	7	7.2702918								
5	4E+07	5_41427269	349	204	307	328	277	318	0.966387	1	180	148	76	83	164	80	1.04E-05	0.0013129	209.38	107.91258					4.1E+07	4.1E+07	0.54593	ID=promoter_39745;
5	4E+07	5_434568	114	162	185	225	138	205	0.099056	1	82	52	104	2	67	53	0.68854646	1	115.75	72.8535713								
5	4E+07	5_443332	34	118	44	84	76	64	0.145865	1	90	116	186	144	103	165	5.98E-05	0.0059171	102	50.3246602								
5	4E+07	5_447152	14	0	9	16	7	13	0.61331	1	8	1	19	0	5	10	0.35527535	1	8.375	7.53918336								

5	4E+07	5_447357	8	18	17	28	13	23	0.490912	1	79	39	16	46	59	31	0.03456818	1	31.375	23.0523473								
5	5E+07	5_457728	48	19	36	27	34	32	0.669588	1	0	0	7	0	0	4	0.11562589	1	17.125	18.4888963								
5	5E+07	5_459464	1	0	0	0	1	0	0.556284	1	14	0	13	16	7	15	0.16935974	1	5.5	7.36788398								
5	5E+07	5_460438	2	73	13	18	38	16	0.001589	0.09301	39	18	11	31	29	21	0.68832436	1	25.625	22.3602805								
5	5E+07	5_461794	89	58	80	46	74	63	0.171656	1	16	9	5	0	13	3	0.06317581	1	37.875	35.2275765								
5	5E+07	5_473091	50	58	60	51	54	56	0.811596	1	22	36	1	40	29	21	0.68832436	1	39.75	20.0196332								
5	5E+07	5_476832	154	141	13	24	148	19	3.20E-27	#######	69	46	60	13	58	37	0.20262844	1	65	55.0168805								
5	5E+07	5_481775	9	5	9	12	7	11	0.831726	1	16	8	12	2	12	7	0.61698677	1	9.125	4.35685011								
5	5E+07	5_491736	2	26	6	2	14	4	0.02589	0.86655	6	59	26	24	33	25	0.74763976	1	18.875	19.416028								
5	5E+07	5_49232736	902	393	904	679	648	792	0.739542	1	357	350	470	94	354	282	0.1639819	1	518.63	286.411262					4.9E+07	4.9E+07	0.50073	ID=promoter_39790;
5	5E+07	5_496580	27	30	23	17	29	20	0.181062	1	177	102	77	75	140	76	7.29E-04	0.0487307	66	54.6233859								
5	5E+07	5_496788	14	2	9	18	8	14	0.661714	1	42	12	27	17	27	22	0.90387209	1	17.625	12.2233676								
5	5E+07	5_51575914	14	13	18	30	14	24	0.52503	1	35	12	18	20	24	19	0.87598349	1	20	8.29802042	5E+07	5.2E+07	ID=ENSP0000325155;PLEKHA5;;	PLEKHA5				
5	5E+07	5_517601	74	66	69	88	70	79	0.972764	1	87	38	39	24	63	32	0.01910095	0.6567034	60.625	24.0353163								
5	5E+07	5_51816583	271	186	309	356	229	333	0.043062	1	144	95	100	98	120	99	0.69122862	1	194.88	104.066514					5.2E+07	5.2E+07	0.57292	ID=promoter_39802;
5	5E+07	5_519022	0	37	6	0	19	3	7.41E-04	0.04898	30	1	20	1	16	11	0.74569574	1	11.875	15.018441								
5	5E+07	5_52240347	6	1	8	6	4	7	0.813458	1	17	1	21	22	9	22	0.0389747	1	10.25	8.54818276	5E+07	5.2E+07	ID=ENSP0000266497;PIK3C2G;;	PIK3C2G				
5	5E+07	5_532403	1	46	10	10	24	10	0.016074	0.59429	1	21	38	35	11	37	3.12E-04	0.0239121	20.25	17.5152994								
5	5E+07	5_535920	13	20	10	7	17	9	0.140533	1	17	6	13	17	12	15	0.74080056	1	12.875	4.99821397								
5	5E+07	5_536987	68	41	74	84	55	79	0.512893	1	18	61	78	30	40	54	0.19353425	1	56.75	24.2059615								
5	5E+07	5_539853	30	0	34	24	15	29	0.283564	1	1	11	36	2	6	19	0.02032977	0.6899793	17.25	15.4619348								
5	6E+07	5_55927654	17	17	7	13	17	10	0.210142	1	29	33	29	4	31	17	0.21261815	1	18.625	10.7429379	6E+07	5.6E+07	ID=ENSP0000279593;GRIN2B;NM_000834;	GRIN2B				
5	6E+07	5_55964400	25	8	27	2	17	15	0.722447	1	8	5	30	17	7	24	0.00482107	0.2211976	15.25	10.9511904	6E+07	5.6E+07	ID=ENSP0000279593;GRIN2B;NM_000834;	GRIN2B				
5	6E+07	5_560123	65	38	48	49	52	49	0.544924	1	78	42	35	21	60	28	0.00931194	0.3713925	47	17.7924543								
5	6E+07	5_562145	6	40	18	7	23	13	0.098913	1	3	3	15	53	3	34	6.76E-07	0.0001173	18.125	18.6427887								
5	6E+07	5_562435	1	25	10	13	13	12	0.835334	1	0	0	36	34	0	35	6.33E-09	1.62E-06	14.875	15.0089259								
5	6E+07	5_562615	10	36	3	0	23	2	2.40E-05	0.00257	1	19	6	0	10	3	0.19568363	1	9.375	12.5349511								
5	6E+07	5_563071	45	42	25	4	44	15	6.23E-05	0.00589	41	62	47	4	52	26	0.03304839	1	33.75	20.9335684								
5	6E+07	5_563978	15	56	14	10	36	12	2.99E-04	0.02308	41	21	42	0	31	21	0.53735114	1	24.875	19.2460571								
5	6E+07	5_56730192	336	78	254	206	207	230	0.852267	1	93	66	100	0	80	50	0.0887083	1	141.63	112.425134					5.7E+07	5.7E+07	0.67801	ID=promoter_39830;
5	6E+07	5_56924481	4659	4644	5638	5579	4652	5609	0.380945	1	3430	3196	6396	1227	3313	3812	9.19E-19	5.96E-16	4346.1	1667.69914					5.7E+07	5.7E+07	0.41806	ID=promoter_39833;
5	6E+07	5_56977240	515	173	428	361	344	395	0.959217	1	187	94	99	101	141	100	0.12110862	1	244.75	166.152039					5.7E+07	5.7E+07	0.15893	ID=promoter_39835;
5	6E+07	5_572125	567	221	415	335	394	375	0.014853	0.55839	159	104	194	47	132	121	0.99600807	1	255.25	172.999381								
5	6E+07	5_583168	974	337	581	562	656	572	1.32E-06	0.00019	217	181	236	172	199	204	0.60058167	1	407.5	280.899067								
5	6E+07	5_58329941	114	50	118	73	82	96	0.99992	1	8	15	21	36	12	29	0.01494463	0.5435394	54.375	43.296115					5.8E+07	5.8E+07	0.30992	ID=promoter_39854;
5	6E+07	5_58428155	1136	1504	1286	2011	1320	1649	0.23318	1	964	767	2204	641	866	1423	4.38E-40	6.45E-37	1314.1	563.29577					5.8E+07	5.8E+07	0.3114	ID=promoter_39855;
5	6E+07	5_584412	267	87	251	135	177	193	0.781912	1	43	100	71	64	72	68	0.99397127	1	127.25	85.7983516								
5	6E+07	5_585888	13	13	5	0	13	3	0.018372	0.65917	0	16	8	0	8	4	0.59180275	1	6.875	6.59951297								
5	6E+07	5_589515	0	21	0	0	11	0	0.001579	0.09256	0	22	0	0	11	0	0.00610443	0.2666968	5.375	9.95615388								
5	6E+07	5_595387	19	157	30	50	88	40	1.66E-06	0.00024	120	66	108	44	93	76	0.71675827	1	74.25	48.7874398								
5	6E+07	5_596082	17	10	4	10	14	7	0.165008	1	4	0	15	52	2	34	1.89E-07	3.74E-05	14	16.3968638								
5	6E+07	5_597623	60	52	289	237	56	263	2.54E-23	#######	117	54	318	142	86	230	1.82E-17	1.09E-14	158.63	108.622462								
5	6E+07	5_59788632	0	6	5	20	3	13	0.09118	1	0	4	0	16	2	8	0.1303879	1	6.375	7.63333853	6E+07	6E+07	ID=ENSP0000376264-D3;MASP1;NM_139125;	MASP1				

5	6E+07	5_59843371	835	259	752	622	547	687	0.490747	1	267	182	214	179	225	197	0.8423756	1	413.75	275.014675					6E+07	6E+07	0.48817	ID=promoter_39876;
5	6E+07	5_59880368	0	3	5	10	2	8	0.255957	1	15	0	7	2	8	5	0.78486764	1	5.25	5.23040561					6E+07	6E+07	0.14942	ID=promoter_39881;
5	6E+07	5_60055559	20	53	42	68	37	55	0.536039	1	32	21	50	17	27	34	0.49096177	1	37.875	18.4192252	6E+07	6E+07	ID=ENSP00000349364;IFFO1;NM_001039670;	IFFO1	6E+07	6E+07	0.17636	ID=promoter_39895;
5	6E+07	5_60150058	7	14	1	4	11	3	0.050503	1	49	43	8	0	46	4	1.01E-07	2.09E-05	15.75	19.2409533					6E+07	6E+07	0.22595	ID=promoter_39903;
5	6E+07	5_60150338	6	16	18	13	11	16	0.859437	1	15	2	24	4	9	14	0.47220951	1	12.25	7.61108215					6E+07	6E+07	0.22595	ID=promoter_39903;
5	6E+07	5_603414	258	190	299	308	224	304	0.25302	1	195	233	225	198	214	212	0.79088022	1	238.25	46.2408292								
5	6E+07	5_604623	6	23	1	1	15	1	6.51E-04	0.04418	10	15	0	1	13	1	0.00904697	0.3617342	7.125	8.33987838								
5	6E+07	5_605156	42	30	48	18	36	33	0.591026	1	44	27	17	0	36	9	8.09E-04	0.0530112	28.25	16.3073165								
5	6E+07	5_606185	29	29	3	14	29	9	8.94E-04	0.05763	15	28	12	2	22	7	0.03554381	1	16.5	11.1226924								
5	6E+07	5_606612	157	87	204	243	122	224	3.15E-04	0.02414	98	40	71	23	69	47	0.26227639	1	115.38	78.5274065								
5	6E+07	5_606768	34	3	5	16	19	11	0.163668	1	34	20	15	4	27	10	0.0371409	1	16.375	12.5235492								
5	6E+07	5_607020	45	131	44	77	88	61	0.006099	0.27549	118	72	98	67	95	83	0.92509272	1	81.5	31.8792364								
5	6E+07	5_610770	1	67	57	48	34	53	0.430266	1	181	68	49	89	125	69	0.00222021	0.1198973	70	51.4781507								
5	6E+07	5_611421	4	44	63	29	24	46	0.14369	1	8	11	28	11	10	20	0.12622058	1	24.75	20.533944								
5	6E+07	5_612601	55	42	64	45	49	55	0.97521	1	114	23	34	0	69	17	9.82E-07	0.0001638	47.125	33.4341036								
5	6E+07	5_612679	43	42	27	43	43	35	0.271897	1	32	1	11	3	17	7	0.1782776	1	25.25	17.9104917								
5	6E+07	5_613176	98	48	45	101	73	73	0.627873	1	91	17	84	42	54	63	0.46216133	1	65.75	31.4858245								
5	6E+07	5_61371421	1477	393	1127	1088	935	1108	0.973764	1	373	351	305	90	362	198	1.21E-08	2.95E-06	650.5	502.579631					6.1E+07	6.1E+07	0.7015	ID=promoter_39938;
5	6E+07	5_61663220	4	53	9	16	29	13	0.011304	0.44898	47	0	16	22	24	19	0.87598349	1	20.875	19.3644557	6E+07	6.2E+07	ID=ENSP00000384253;;					
5	6E+07	5_617768	255	216	206	209	236	208	0.010602	0.43029	66	73	77	131	70	104	0.0087339	0.3525734	154.13	76.0985029								
5	6E+07	5_618110	21	49	36	16	35	26	0.205248	1	30	35	8	15	33	12	0.01587283	0.5667339	26.25	13.604096								
5	6E+07	5_61853585	615	196	544	350	406	447	0.652687	1	227	58	230	16	143	123	0.83061094	1	279.5	213.194747					6.2E+07	6.2E+07	0.18964	ID=promoter_39949;
5	6E+07	5_619278	9	33	12	10	21	11	0.084122	1	7	1	9	0	4	5	0.90483023	1	10.125	10.1761275								
5	6E+07	5_61935981	21	44	7	21	33	14	0.00394	0.1935	35	11	13	31	23	22	0.99450571	1	22.875	12.8556547					6.2E+07	6.2E+07	0.77278	ID=promoter_39950;
5	6E+07	5_619746	25	7	21	29	16	25	0.667718	1	13	26	44	3	20	24	0.69398062	1	21	13.190906								
5	6E+07	5_619785	110	13	43	44	62	44	0.036233	1	1	23	4	1	12	3	0.09316162	1	29.875	36.7634815								
5	6E+07	5_622743	29	25	31	25	27	28	0.900886	1	79	33	92	0	56	46	0.83113017	1	39.25	30.5228252								
5	6E+07	5_622765	249	217	888	960	233	924	1.78E-68	#######	124	56	680	297	90	489	2.87E-67	6.30E-64	433.88	355.040214								
5	6E+07	5_62392061	2	29	2	6	16	4	0.009581	0.39335	6	2	8	0	4	4	0.99427496	1	6.875	9.34172667	6E+07	6.2E+07	ID=ENSP00000372067-D2;PRMT8;NM_019854;	PRMT8				
5	6E+07	5_62415648	11	25	4	12	18	8	0.059652	1	10	3	6	11	7	9	0.80856893	1	10.25	6.88165263	6E+07	6.2E+07	ID=ENSP00000372067-D2;PRMT8;NM_019854;	PRMT8				
5	6E+07	5_626051	9	29	5	1	19	3	7.41E-04	0.04898	26	9	3	22	18	13	0.78939737	1	13	10.9935046								
5	6E+07	5_62609488	283	146	226	192	215	209	0.153362	1	144	60	106	46	102	76	0.35218466	1	150.38	81.1804648					6.3E+07	6.3E+07	0.66926	ID=promoter_39974;
5	6E+07	5_62786249	1	5	2	13	3	8	0.459247	1	1	0	19	1	1	10	0.01766414	0.6154554	5.25	6.98467711	6E+07	6.3E+07	ID=ENSP00000261177;TSPAN11;NM_001080509;	TSPAN11				
5	6E+07	5_628050	2	1	0	1	2	1	0.773064	1	1	11	6	29	6	18	0.03091566	0.954015	6.375	9.85520167								
5	6E+07	5_62831063	15	488	23	37	252	30	4.95E-47	#######	67	420	16	6	244	11	2.89E-43	4.74E-40	134	199.212736	6E+07	6.3E+07	ID=ENSP00000011898;TSPAN9;NM_006675;	TSPAN9				

5	6E+07	5_62833262	94	108	67	63	101	65	6.48E-04	0.04414	11	51	50	5	31	28	0.99502871	1	56.125	35.8665682	6E+07	6.3E+07	ID=ENSP00000011898;TSPAN9;NM_006675;	TSPAN9				
5	6E+07	5_62879412	8	27	31	19	18	25	0.86091	1	0	9	0	1	5	1	0.30486099	1	11.875	12.3570396	6E+07	6.3E+07	ID=ENSP00000011898;TSPAN9;NM_006675;	TSPAN9				
5	6E+07	5_629138	14	3	22	6	9	14	0.802715	1	0	20	7	15	10	11	0.92604858	1	10.875	8.04340901								
5	6E+07	5_629789	29	28	16	8	29	12	0.006478	0.28949	57	30	22	17	44	20	0.02618764	0.844814	25.875	14.6914698								
5	6E+07	5_63019642	40	15	24	3	28	14	0.027228	0.89953	0	30	23	24	15	24	0.24434768	1	19.875	13.3890947	6E+07	6.3E+07	ID=ENSP00000349828-D3;TEAD3;;	TEAD3				
5	6E+07	5_63051090	0	11	36	2	6	19	0.087167	1	227	117	145	89	172	117	0.03462269	1	78.375	81.4545228					6.3E+07	6.3E+07	0.14437	ID=promoter_39990;
5	6E+07	5_63142100	10	5	33	8	8	21	0.137587	1	58	2	38	0	30	19	0.42545995	1	19.25	21.1305466	6E+07	6.3E+07	ID=ENSP00000372125;;;					
5	6E+07	5_63160503	73	20	69	64	47	67	0.590402	1	41	41	30	37	41	34	0.89086031	1	46.875	19.416028	6E+07	6.3E+07	ID=ENSP00000337501;NRIP2;NM_031474;	NRIP2				
5	6E+07	5_63163132	1	7	9	8	4	9	0.54411	1	15	10	5	7	13	6	0.35360803	1	7.75	4.02669663	6E+07	6.3E+07	ID=ENSP00000337501;NRIP2;NM_031474;	NRIP2				
5	6E+07	5_63165528	18	3	4	10	11	7	0.438207	1	9	0	16	1	5	9	0.48000906	1	7.625	6.78101341	6E+07	6.3E+07	ID=ENSP00000337501;NRIP2;NM_031474;	NRIP2				
5	6E+07	5_63181356	472	192	413	327	332	370	0.795309	1	255	131	608	165	193	387	3.27E-18	2.06E-15	320.38	166.947329					6.3E+07	6.3E+07	0.39618	ID=promoter_39997;
5	6E+07	5_632536	47	61	120	88	54	104	0.011557	0.45508	80	43	37	26	62	32	0.02413328	0.7914145	62.75	31.3220598								
5	6E+07	5_63296172	1064	202	630	788	633	709	0.700442	1	481	255	252	103	368	178	3.81E-12	1.49E-09	471.88	333.790969					6.3E+07	6.3E+07	0.46701	ID=promoter_40000;
5	6E+07	5_63401462	337	163	207	201	250	204	5.66E-04	0.03925	127	83	131	33	105	82	0.50729328	1	160.25	91.9747636	6E+07	6.3E+07	ID=ENSP00000315662;IQSEC3;;	IQSEC3				
5	6E+07	5_635340	1670	793	2059	1600	1232	1830	1.10E-09	#######	1192	582	619	379	887	499	2.17E-18	1.37E-15	1111.8	611.28851								
5	6E+07	5_63725178	540	270	498	547	405	523	0.347743	1	271	224	288	111	248	200	0.3406854	1	343.63	162.994248					6.4E+07	6.4E+07	0.74702	ID=promoter_40012;
5	6E+07	5_63925774	4787	1852	2739	2338	3320	2539	9.31E-59	#######	2990	1061	1239	811	2026	1025	1.30E-56	2.61E-53	2227.1	1303.38104					6.4E+07	6.4E+07	0.64704	ID=promoter_40013;
5	6E+07	5_64297980	4	9	3	4	7	4	0.503048	1	1	11	5	20	6	13	0.20797135	1	7.125	6.12809688	6E+07	6.4E+07	ID=ENSP00000380386;ERC1;;	ERC1				
5	6E+07	5_644117	2	4	4	12	3	8	0.459247	1	9	0	10	24	5	17	0.02356069	0.7742377	8.125	7.64269025								
5	6E+07	5_64699990	462	133	307	190	298	249	3.79E-04	0.02808	169	42	83	25	106	54	0.00135296	0.0804582	176.38	146.607286					6.5E+07	6.5E+07	0.43973	ID=promoter_40027;
5	6E+07	5_647074	13	42	15	35	28	25	0.609751	1	46	34	15	23	40	19	0.05034717	1	27.875	13.0541455								
5	6E+07	5_647634	145	53	209	141	99	175	0.004548	0.21732	28	116	45	27	72	36	0.00890416	0.3584328	95.5	67.0053303								
5	6E+07	5_647814	50	42	54	8	46	31	0.054078	1	7	20	21	11	14	16	0.84864027	1	26.625	19.2126261								
5	6E+07	5_64836194	55	23	54	33	39	44	0.984479	1	33	13	28	4	23	16	0.67590535	1	30.375	17.8720652					6.5E+07	6.5E+07	0.31348	ID=promoter_40032;
5	6E+07	5_649441	6	0	9	10	3	10	0.252243	1	0	1	14	25	1	20	8.76E-05	0.0082396	8.125	8.54295867								
5	6E+07	5_649930	6	5	4	0	6	2	0.258649	1	4	7	13	10	6	12	0.2888538	1	6.125	3.97986001								
5	7E+07	5_650110	25	14	40	38	20	39	0.174648	1	30	55	17	22	43	20	0.03412779	1	30.125	13.6427636								
5	7E+07	5_650561	29	15	68	26	22	47	0.062332	1	53	20	2	0	37	1	1.46E-07	2.94E-05	26.625	23.6276926								
5	7E+07	5_651580	17	3	19	16	10	18	0.549586	1	0	0	13	0	0	7	0.02292696	0.7568963	8.5	8.50210058								
5	7E+07	5_651704	14	29	8	28	22	18	0.522468	1	13	7	23	4	10	14	0.60490119	1	15.75	9.73579551								
5	7E+07	5_651955	110	16	125	124	63	125	0.002681	0.14283	191	68	18	123	130	71	0.00138925	0.0823758	96.875	59.5661397								

5	7E+07	5_652420	53	73	38	31	63	35	0.001387	0.08282	39	68	101	4	54	53	0.95737654	1	50.875	29.7582522								
5	7E+07	5_653325	1007	528	808	654	768	731	2.74E-04	0.02136	463	333	246	121	398	184	1.70E-14	8.40E-12	520	295.351606								
5	7E+07	5_658261	193	53	145	93	123	119	0.324613	1	26	55	30	41	41	36	0.97213063	1	79.5	60.2992537								
5	7E+07	5_658728	60	34	95	56	47	76	0.220987	1	51	8	28	3	30	16	0.19481252	1	41.875	30.1114003								
5	7E+07	5_67046870	4	13	1	2	9	2	0.058587	1	37	22	11	3	30	7	0.00181176	0.1006964	11.625	12.4892811	7E+07	6.7E+07	ID=ENSP00000310688;ABCD2;NM_005164;	ABCD2				
5	7E+07	5_672582	44	43	46	43	44	45	0.811179	1	44	31	53	16	38	35	0.99961713	1	40	11.4142768								
5	7E+07	5_673095	7	10	4	24	9	14	0.802715	1	76	0	19	66	38	43	0.66881858	1	25.75	29.1044916								
5	7E+07	5_673621	23	11	0	9	17	5	0.013234	0.50759	0	5	36	22	3	29	9.29E-06	0.0011901	13.25	12.7138844								
5	7E+07	5_678116	27	61	34	57	44	46	0.861515	1	86	22	64	8	54	36	0.30582789	1	44.875	26.1448575								
5	7E+07	5_679920	2	26	4	0	14	2	0.003923	0.19308	22	1	10	19	12	15	0.74080056	1	10.5	10.4197614								
5	7E+07	5_682599	15	70	15	7	43	11	4.66E-06	0.0006	23	53	30	18	38	24	0.33399749	1	28.875	21.6823661								
5	7E+07	5_688271	6	14	30	7	10	19	0.459788	1	37	36	42	42	37	42	0.6671639	1	26.75	15.3506631								
5	7E+07	5_695141	8	1	0	12	5	6	0.999292	1	16	10	0	2	13	1	0.00904697	0.3617342	6.125	6.1976378								
5	7E+07	5_69658511	0	31	4	36	16	20	0.982167	1	22	11	12	34	17	23	0.4932031	1	18.75	13.9667974	7E+07	7E+07	ID=ENSP00000378947;PPHLN1;NM_201515;	PPHLN1				
5	7E+07	5_69728364	2	59	5	3	31	4	2.89E-06	0.0004	104	28	32	1	66	17	3.09E-06	0.000453	29.25	36.4485939	7E+07	7E+07	ID=ENSP00000378947;PPHLN1;NM_201515;	PPHLN1				
5	7E+07	5_69806809	7101	6512	7339	6714	6807	7027	5.45E-13	#######	4792	4076	9689	2538	4434	6114	7.30E-90	2.22E-86	6095.1	2218.74729					7E+07	7E+07	0.7042	ID=promoter_40091;
5	7E+07	5_699875	7	18	36	18	13	27	0.229532	1	5	27	16	20	16	18	0.85288016	1	18.375	10.0133839								
5	7E+07	5_701012	108	85	136	62	97	99	0.621964	1	70	63	46	1	67	24	1.82E-04	0.015179	71.375	40.4614014								
5	7E+07	5_701964	10	20	1	3	15	2	0.002248	0.12273	12	0	12	1	6	7	0.91781435	1	7.375	7.20986427								
5	7E+07	5_702934	23	75	22	46	49	34	0.059003	1	98	53	74	25	76	50	0.16678193	1	52	28.3750393								
5	7E+07	5_70542638	0	13	5	0	7	3	0.314307	1	1	17	4	1	9	3	0.27712561	1	5.125	6.44620597	7E+07	7.1E+07	ID=ENSP00000309332;ADAMTS9;;	ADAMTS9				
5	7E+07	5_710053	39	5	23	14	22	19	0.617881	1	7	36	28	31	22	30	0.38408346	1	22.875	12.9552802								
5	7E+07	5_711242	3	0	7	0	2	4	0.823343	1	25	11	10	2	18	6	0.0767986	1	7.25	8.3452296								
5	7E+07	5_711763	29	8	33	28	19	31	0.523429	1	43	8	16	2	26	9	0.02974521	0.9232309	20.875	14.4660934								
5	7E+07	5_71835695	35	254	33	7	145	20	5.36E-26	#######	92	96	142	108	94	125	0.02931358	0.9184722	95.875	78.2403577	7E+07	7.2E+07	ID=ENSP00000378866;NELL2;NM_001145109;	NELL2				
5	7E+07	5_721022	1	15	0	1	8	1	0.03583	1	0	34	9	24	17	17	0.97589399	1	10.5	12.8619039								
5	7E+07	5_721229	7	10	2	5	9	4	0.244237	1	1	2	5	14	2	10	0.05067035	1	5.75	4.46414285								
5	7E+07	5_722586	33	43	3	28	38	16	0.001589	0.09301	112	61	53	1	87	27	1.22E-06	0.0001993	41.75	35.57989								
5	7E+07	5_725152	34	55	7	26	45	17	1.50E-04	0.01267	15	20	52	10	18	31	0.10487282	1	27.375	18.2829937								
5	7E+07	5_727804	12	26	4	7	19	6	0.010861	0.43721	33	1	18	60	17	39	0.00550599	0.2484707	20.125	19.489466								
5	7E+07	5_72859720	1	11	4	7	6	6	0.962469	1	0	16	12	4	8	8	0.98858271	1	6.875	5.66789202					7.3E+07	7.3E+07	0.73495	ID=promoter_40112;
5	7E+07	5_72859831	684	234	538	438	459	488	0.318936	1	236	287	198	35	262	117	1.61E-10	5.24E-08	331.25	208.594446					7.3E+07	7.3E+07	0.73495	ID=promoter_40112;
5	7E+07	5_72886574	257	81	175	200	169	188	0.882549	1	98	78	134	12	88	73	0.78144854	1	129.38	78.4855537					7.3E+07	7.3E+07	0.63342	ID=promoter_40115;
5	7E+07	5_73095426	1666	980	1905	1850	1323	1878	7.03E-07	0.00011	1261	792	970	441	1027	706	6.54E-09	1.66E-06	1233.1	531.23426					7.3E+07	7.3E+07	0.33822	ID=promoter_40117;
5	7E+07	5_734500	520	191	320	263	356	292	3.13E-05	0.00323	56	35	37	48	46	43	0.99923232	1	183.75	175.813009								
5	7E+07	5_73710777	2	40	1	0	21	1	2.00E-05	0.00219	3	9	13	18	6	16	0.069189	1	10.75	13.4350288	7E+07	7.4E+07	ID=ENSP00000004980;SENP1;;	SENP1				
5	7E+07	5_741919	2	15	0	1	9	1	0.020382	0.71452	0	13	1	17	7	9	0.80856893	1	6.125	7.45342682								
5	7E+07	5_744837	19	3	17	3	11	10	0.842816	1	3	1	9	0	2	5	0.46647814	1	6.875	7.37636186								
5	7E+07	5_74676629	41	72	28	47	57	38	0.023828	0.81152	167	66	76	26	117	51	2.49E-05	0.0027797	65.375	45.2988095	7E+07	7.5E+07	ID=ENSP00000367308;SLC41A2;;	SLC41A2				
5	7E+07	5_747973	2	11	1	5	7	3	0.314307	1	21	0	16	1	11	9	0.96206785	1	7.125	7.95410945								
5	8E+07	5_750135	4	5	12	10	5	11	0.495311	1	58	0	0	0	29	0	1.45E-06	0.0002324	11.125	19.489466								
5	8E+07	5_750627	39	28	54	41	34	48	0.709836	1	40	24	20	0	32	10	0.00696943	0.2965071	30.75	16.5335157								

5	8E+07	5_751403	17	7	10	21	12	16	0.945172	1	42	49	16	11	46	14	6.26E-04	0.0425022	21.625	15.491357								
5	8E+07	5_75319749	2028	1634	1075	990	1831	1033	4.84E-81	#######	1011	1186	869	292	1099	581	1.88E-27	1.83E-24	1135.6	516.921364					7.5E+07	7.5E+07	0.31772	ID=promoter_40149;
5	8E+07	5_75426093	228	36	169	135	132	152	0.988052	1	91	31	62	0	61	31	0.02177598	0.7340029	94	77.8863641					7.5E+07	7.5E+07	0.69743	ID=promoter_40154;
5	8E+07	5_754399	77	93	51	54	85	53	0.001186	0.07291	41	56	39	45	49	42	0.93283003	1	57	18.7692758								
5	8E+07	5_75458139	339	108	280	245	224	263	0.999942	1	83	89	99	48	86	74	0.89537388	1	161.38	109.276762					7.5E+07	7.5E+07	0.64822	ID=promoter_40155;
5	8E+07	5_757297	5	4	11	1	5	6	0.999292	1	17	0	5	2	9	4	0.4571786	1	5.625	5.70557372								
5	8E+07	5_757362	0	0	0	4	0	2	0.426327	1	63	40	23	36	52	30	0.11301787	1	20.75	23.8012605								
5	8E+07	5_758504	52	34	70	18	43	44	0.816262	1	45	21	7	50	33	29	0.97818147	1	37.125	20.9519007								
5	8E+07	5_75882038	22	0	15	15	11	15	0.930409	1	17	9	25	0	13	13	0.98151319	1	12.875	9.2803556	8E+07	7.6E+07	ID=ENSP00000376615;NT5DC3;NM_001031701;	NT5DC3				
5	8E+07	5_75882687	13	0	11	5	7	8	0.998739	1	223	89	27	3	156	15	9.68E-24	7.99E-21	46.375	76.9952457	8E+07	7.6E+07	ID=ENSP00000376615;NT5DC3;NM_001031701;	NT5DC3				
5	8E+07	5_75890062	0	10	3	9	5	6	0.999292	1	0	19	17	2	10	10	0.98574881	1	7.5	7.50238057	8E+07	7.6E+07	ID=ENSP00000376615;NT5DC3;NM_001031701;	NT5DC3				
5	8E+07	5_75899429	74	79	67	81	77	74	0.471587	1	76	29	83	21	53	52	0.95866627	1	63.75	24.4992711	8E+07	7.6E+07	ID=ENSP00000376615;NT5DC3;NM_001031701;	NT5DC3				
5	8E+07	5_759949	445	210	337	254	328	296	0.004616	0.21996	180	152	119	41	166	80	6.33E-06	0.0008495	217.25	127.60178								
5	8E+07	5_763711	1	6	9	24	4	17	0.046186	1	0	0	0	3	0	2	0.34003807	1	5.375	8.21040281								
5	8E+07	5_766241	28	48	34	47	38	41	0.93346	1	0	1	14	1	1	8	0.04988758	1	21.625	20.3886348								
5	8E+07	5_770206	0	10	5	7	5	6	0.999292	1	27	22	24	28	25	26	0.91912473	1	15.375	11.0574797								
5	8E+07	5_774170	9	52	47	29	31	38	0.983553	1	36	20	17	38	28	28	0.96060652	1	31	15.0047611								
5	8E+07	5_77656230	116	126	81	52	121	67	2.96E-06	0.0004	127	62	54	40	95	47	0.00165098	0.0930606	82.25	35.7880666	8E+07	7.8E+07	ID=ENSP00000258534;;NM_018370;					
5	8E+07	5_781904	0	4	3	0	2	2	0.98733	1	15	0	19	1	8	10	0.81871774	1	5.25	7.47854073								
5	8E+07	5_78921376	71	11	7	0	41	4	1.01E-08	#######	34	50	19	2	42	11	3.56E-04	0.0266828	24.25	25.4319821	8E+07	7.9E+07	ID=ENSP00000376705;;;					
5	8E+07	5_79097766	547	60	495	473	304	484	1.53E-04	0.01284	198	118	162	32	158	97	0.00564583	0.2538983	260.63	209.950972					7.9E+07	7.9E+07	0.22593	ID=promoter_40201;
5	8E+07	5_794597	39	43	40	81	41	61	0.49837	1	33	25	15	17	29	16	0.24173971	1	36.625	20.7979223								
5	8E+07	5_796851	59	71	38	17	65	28	2.33E-05	0.00251	51	53	84	45	52	65	0.27250363	1	52.25	20.3733019								
5	8E+07	5_801430	7	14	20	14	11	17	0.774447	1	28	20	10	1	24	6	0.00867909	0.3505674	14.25	8.46421037								
5	8E+07	5_802687	92	26	62	46	59	54	0.418093	1	54	81	20	37	68	29	0.0015925	0.0904103	52.25	25.3701174								
5	8E+07	5_804253	0	39	12	8	20	10	0.076235	1	31	12	23	7	22	15	0.65424549	1	16.5	13.2772631								
5	8E+07	5_80450548	39	32	13	28	36	21	0.034407	1	25	27	2	0	26	1	2.34E-05	0.002637	20.75	14.2001006					8E+07	8E+07	0.20266	ID=promoter_40209;
5	8E+07	5_804634	0	15	1	4	8	3	0.206144	1	10	26	6	1	18	4	0.01933304	0.6616555	7.875	8.93528319								
5	8E+07	5_804983	22	25	17	19	24	18	0.351411	1	51	25	28	15	38	22	0.20745497	1	25.25	11.2979138								
5	8E+07	5_80670101	25	21	15	1	23	8	0.007066	0.30636	1	28	27	2	15	15	0.97869956	1	15	12.0118989	8E+07	8.1E+07	ID=ENSP00000353059;APAF1;NM_181861;	APAF1				
5	8E+07	5_807756	432	164	285	261	298	273	0.012607	0.48953	82	105	74	97	94	86	0.99609906	1	187.5	127.755347								
5	8E+07	5_813039	0	29	24	6	15	15	0.908797	1	14	30	34	9	22	22	0.96891521	1	18.25	12.6575782								
5	8E+07	5_813405	63	84	83	92	74	88	0.996209	1	110	43	111	97	77	104	0.04199235	1	85.375	23.132769								
5	8E+07	5_813933	8	6	5	0	7	3	0.314307	1	9	0	24	2	5	13	0.12287893	1	6.75	7.75978645								
5	8E+07	5_81517961	3	0	1	3	2	2	0.98733	1	0	13	2	27	7	15	0.16935974	1	6.125	9.41787814					8.2E+07	8.2E+07	0.12907	ID=promoter_40223;
5	8E+07	5_81550288	12	12	11	4	12	8	0.45534	1	5	11	6	1	8	4	0.59180275	1	7.75	4.26781978					8.2E+07	8.2E+07	0.1215	ID=promoter_40224;
5	8E+07	5_816126	7	0	26	8	4	17	0.046186	1	0	16	17	0	8	9	0.92351511	1	9.25	9.63253416								
5	8E+07	5_816483	89	95	106	114	92	110	0.99084	1	152	171	465	150	162	308	3.86E-13	1.68E-10	167.75	123.709048								

5	8E+07	5_816758	17	9	18	1	13	10	0.600182	1	17	27	25	19	22	22	0.96891521	1	16.625	8.34843869								
5	8E+07	5_81837018	229	134	216	295	182	256	0.172089	1	110	161	152	49	136	101	0.23901773	1	168.25	76.6620599					8.2E+07	8.2E+07	0.16536	ID=promoter_40229;
5	8E+07	5_819023	87	75	59	54	81	57	0.011532	0.45484	60	65	104	22	63	63	0.91353974	1	65.75	24.2943262								
5	8E+07	5_820377	116	211	79	96	164	88	9.96E-09	#######	155	123	198	87	139	143	0.68218745	1	133.13	50.118253								
5	8E+07	5_822410	19	36	55	16	28	36	0.935761	1	1	16	25	1	9	13	0.58766318	1	21.125	17.9319149								
5	8E+07	5_822500	10	20	9	0	15	5	0.034023	1	52	38	15	0	45	8	9.46E-06	0.0012039	18	18.3692289								
5	8E+07	5_824676	49	25	54	51	37	53	0.64625	1	21	3	2	24	12	13	0.92692342	1	28.625	20.7635498								
5	8E+07	5_825993	11	43	10	10	27	10	0.004472	0.21437	10	13	52	20	12	36	9.56E-04	0.060345	21.125	16.7880697								
5	8E+07	5_82949879	67	30	22	24	49	23	8.79E-04	0.05707	3	35	37	18	19	28	0.28999502	1	29.5	18.5703296	8E+07	8.3E+07	ID=ENSP00000376806;USP44;;	USP44				
5	8E+07	5_83114673	1	18	1	12	10	7	0.570701	1	7	13	17	12	10	15	0.49251655	1	10.125	6.55607679	8E+07	8.3E+07	ID=ENSP00000344446;FGD6;NM_018351;	FGD6				
5	8E+07	5_83202149	7	22	40	10	15	25	0.557403	1	24	32	31	1	28	16	0.29661245	1	20.875	13.6741414	8E+07	8.3E+07	ID=ENSP00000376814;NR2C2;;	NR2C2				
5	8E+07	5_833473	6	13	7	9	10	8	0.719353	1	0	26	42	9	13	26	0.0678374	1	14	13.56466								
5	8E+07	5_833913	8	18	14	20	13	17	0.957342	1	26	15	37	12	21	25	0.69849299	1	18.75	9.1456469								
5	8E+07	5_83394251	57	57	78	53	57	66	0.99745	1	25	37	19	4	31	12	0.02977149	0.9232309	41.25	24.3471911					8.3E+07	8.3E+07	0.26129	ID=promoter_40270;
5	8E+07	5_83421162	1465	870	3194	2951	1168	3073	########	#######	752	608	1340	762	680	1051	1.15E-24	1.00E-21	1492.8	1021.07461					8.3E+07	8.3E+07	0.60972	ID=promoter_40274;
5	8E+07	5_834502	156	72	195	150	114	173	0.10106	1	101	89	117	36	95	77	0.68098723	1	114.5	51.0881591								
5	8E+07	5_83480983	1	13	51	29	7	40	1.05E-04	0.00931	0	0	46	70	0	58	2.60E-14	1.28E-11	26.25	27.0330486					8.3E+07	8.3E+07	0.14652	ID=promoter_40277;
5	8E+07	5_83527739	12	51	6	16	32	11	9.30E-04	0.05947	24	1	4	1	13	3	0.06317581	1	14.375	16.8093299	8E+07	8.4E+07	ID=ENSP00000330737;NDUFA12;NM_018838;	NDUFA12				
5	8E+07	5_835943	11	79	19	15	45	17	1.50E-04	0.01267	1	44	28	10	23	19	0.93310412	1	25.875	25.1079811								
5	8E+07	5_83623641	8	12	7	17	10	12	0.998584	1	18	0	2	22	9	12	0.70935077	1	10.75	7.86947085					8.4E+07	8.4E+07	0.12144	ID=promoter_40286;
5	8E+07	5_836261	274	95	251	153	185	202	0.78058	1	11	22	44	9	17	27	0.21252244	1	107.38	107.502741								
5	8E+07	5_837556	1	1	2	8	1	5	0.353258	1	15	8	22	6	12	14	0.84238319	1	7.875	7.39570339								
5	8E+07	5_837626	115	48	69	62	82	66	0.072527	1	87	160	119	57	124	88	0.15751174	1	89.625	38.6409757								
5	8E+07	5_837775	21	29	22	25	25	24	0.781475	1	62	30	13	0	46	7	2.43E-06	0.0003668	25.25	17.7422015								
5	8E+07	5_837853	106	83	116	131	95	124	0.735269	1	141	57	113	29	99	71	0.2542032	1	97	38.1912481								
5	8E+07	5_83864537	110	51	75	44	81	60	0.02459	0.832	77	36	43	32	57	38	0.2863469	1	58.5	26.6779738	8E+07	8.4E+07	ID=ENSP00000258526;PLXNC1;NM_005761;	PLXNC1				
5	8E+07	5_83867069	11	0	11	17	6	14	0.355886	1	0	0	11	59	0	35	6.33E-09	1.62E-06	13.625	19.4491094	8E+07	8.4E+07	ID=ENSP00000258526;PLXNC1;NM_005761;	PLXNC1	8.4E+07	8.4E+07	0.19581	ID=promoter_40305;
5	8E+07	5_839415	18	67	29	20	43	25	0.017316	0.62799	125	145	62	53	135	58	3.37E-06	0.000489	64.875	47.3390129								
5	8E+07	5_839968	92	41	93	101	67	97	0.414305	1	41	21	14	15	31	15	0.10937197	1	52.25	37.2203861								
5	8E+07	5_840760	192	49	358	313	121	336	5.53E-16	#######	72	0	99	40	36	70	0.00108542	0.0671641	140.38	133.278374								
5	8E+07	5_84086055	27	47	48	17	37	33	0.517593	1	57	15	42	41	36	42	0.59776306	1	36.75	15.3320393					8.4E+07	8.4E+07	0.14719	ID=promoter_40322;
5	8E+07	5_84087474	6	51	14	5	29	10	0.001829	0.10386	11	17	26	8	14	17	0.75475782	1	17.25	15.2479507					8.4E+07	8.4E+07	0.86051	ID=promoter_40323;
5	8E+07	5_84088308	330	102	336	325	216	331	0.009115	0.37984	121	69	93	38	95	66	0.19561503	1	176.75	129.502068					8.4E+07	8.4E+07	0.86051	ID=promoter_40323;
5	8E+07	5_841951	0	16	1	0	8	1	0.03583	1	10	2	5	19	6	12	0.2888538	1	6.625	7.52021086								
5	8E+07	5_843514	7	30	14	14	19	14	0.411928	1	34	23	39	36	29	38	0.36985231	1	24.625	11.9036309								
5	8E+07	5_843634	147	225	134	124	186	129	2.12E-05	0.00231	165	195	116	81	180	99	1.36E-04	0.0118261	148.38	45.8442628								
5	8E+07	5_844341	82	49	45	51	66	48	0.039347	1	0	18	7	0	9	4	0.4571786	1	31.5	29.7273323								
5	8E+07	5_844492	24	7	16	20	16	18	0.992646	1	60	18	11	0	39	6	1.85E-05	0.0021598	19.5	18.0633805								
5	8E+07	5_847495	26	136	26	21	81	24	1.29E-09	#######	112	30	90	42	71	66	0.99987261	1	60.375	45.4121994								

5	8E+07	5_84807646	0	12	25	0	6	13	0.45043	1	12	13	5	0	13	3	0.06317581	1	8.375	8.83074985	8E+07	8.5E+07	ID=ENSP00000317955;EEA1;NM_003566;	EEA1				
5	8E+07	5_849069	26	45	35	24	36	30	0.380805	1	34	32	40	8	33	24	0.66283249	1	30.5	11.3641039								
5	8E+07	5_849174	0	13	12	12	7	12	0.724589	1	1	1	9	3	1	6	0.13767373	1	6.375	5.65527567								
5	9E+07	5_850483	16	5	17	7	11	12	0.985018	1	15	9	13	9	12	11	0.99963644	1	11.375	4.47013902								
5	9E+07	5_851200	30	7	3	0	19	2	2.36E-04	0.01868	25	17	24	0	21	12	0.40192224	1	13.25	12.2094811								
5	9E+07	5_851822	43	33	29	4	38	17	0.002761	0.14629	29	1	20	30	15	25	0.19020812	1	23.625	14.5006158								
5	9E+07	5_853228	16	21	3	9	19	6	0.010861	0.43721	0	13	0	9	7	5	0.9051169	1	8.875	7.62397534								
5	9E+07	5_854140	18	26	18	29	22	24	0.97026	1	0	15	42	60	8	51	2.88E-08	6.63E-06	26	18.3069074								
5	9E+07	5_868089	0	0	0	8	0	4	0.181755	1	0	16	14	17	8	16	0.19094317	1	6.875	7.80910641								
5	9E+07	5_86899960	63	72	68	115	68	92	0.670931	1	57	79	16	26	68	21	2.23E-05	0.0025411	62	30.8730118	9E+07	8.7E+07	ID=ENSP00000261172;EPYC;NM_004950;	EPYC				
5	9E+07	5_86916151	1	16	7	5	9	6	0.554309	1	0	3	23	0	2	12	0.01895406	0.6519669	6.875	8.37406711	9E+07	8.7E+07	ID=ENSP00000261172;EPYC;NM_004950;	EPYC				
5	9E+07	5_869259	0	16	0	0	8	0	0.00917	0.37984	23	21	19	16	22	18	0.92557456	1	11.875	10.1056915								
5	9E+07	5_875688	14	27	6	7	21	7	0.0088	0.36783	22	12	5	23	17	14	0.9475482	1	14.5	8.53563957								
5	9E+07	5_875908	0	35	2	6	18	4	0.003432	0.17404	83	15	30	36	49	33	0.36223911	1	25.875	27.3048269								
5	9E+07	5_878806	12	27	6	24	20	15	0.418324	1	18	17	18	31	18	25	0.42027749	1	19.125	8.07885246								
5	9E+07	5_878816	82	75	53	39	79	46	5.96E-04	0.04099	0	3	6	1	2	4	0.6610374	1	32.375	34.5168178								
5	9E+07	5_879319	14	19	6	17	17	12	0.395844	1	0	12	6	1	6	4	0.87665919	1	9.375	7.17012652								
5	9E+07	5_882389	27	17	15	52	22	34	0.599769	1	0	26	23	2	13	13	0.98151319	1	20.25	16.3685238								
5	9E+07	5_886046	14	0	7	3	7	5	0.693374	1	18	0	6	15	9	11	0.82669107	1	7.875	6.99872437								
5	9E+07	5_901595	15	41	13	38	28	26	0.690999	1	43	30	91	9	37	50	0.21697517	1	35	26.2678511								
5	9E+07	5_90240104	10	22	0	10	16	5	0.021336	0.74103	18	29	3	31	24	17	0.69596948	1	15.375	11.5131912	9E+07	9E+07	ID=ENSP00000349358;C12orf29;NM_001009894;	C12orf29				
5	9E+07	5_908962	37	132	53	38	85	46	8.41E-05	0.00761	71	75	92	65	73	79	0.63434638	1	70.375	31.1215933								
5	9E+07	5_909404	14	0	13	9	7	11	0.831726	1	62	60	15	0	61	8	9.96E-09	2.46E-06	21.625	24.9967855								
5	9E+07	5_909899	1	1	2	2	1	2	0.907382	1	28	0	12	2	14	7	0.39930956	1	6	9.66584561								
5	9E+07	5_910699	8	4	16	19	6	18	0.118314	1	24	9	20	1	17	11	0.64636287	1	12.625	8.27970841								
5	9E+07	5_911192	0	10	16	28	5	22	0.016403	0.6047	29	19	13	45	24	29	0.62889415	1	20	13.8151056								
5	9E+07	5_911746	21	109	44	51	65	48	0.049348	1	15	11	38	20	13	29	0.02500319	0.8111854	38.625	31.8789563								
5	9E+07	5_912819	109	104	68	66	107	67	2.28E-04	0.01817	56	57	50	7	57	29	0.02830192	0.8918846	64.625	32.0710706								
5	9E+07	5_91540278	2462	1311	2337	1752	1887	2045	0.046278	1	1091	1050	1081	636	1071	859	0.00668618	0.2868305	1465	655.447067					9.2E+07	9.2E+07	0.35019	ID=promoter_40376;
5	9E+07	5_91692647	2652	1326	2599	1799	1989	2199	0.160768	1	976	967	1009	408	972	709	7.22E-06	0.0009506	1467	813.991927					9.2E+07	9.2E+07	0.48659	ID=promoter_40377;
5	9E+07	5_91762702	1544	716	1039	1075	1130	1057	7.95E-07	0.00012	769	465	534	104	617	319	9.62E-17	5.37E-14	780.75	440.572922					9.2E+07	9.2E+07	0.70929	ID=promoter_40378;
5	9E+07	5_91767325	54	59	30	51	57	41	0.054559	1	0	29	26	3	15	15	0.97869956	1	31.5	22.3159136					9.2E+07	9.2E+07	0.36027	ID=promoter_40379;
5	9E+07	5_919469	11	48	36	29	30	33	0.968109	1	29	47	57	5	38	31	0.86961204	1	32.75	18.1481994								
5	9E+07	5_925232	25	16	4	5	21	5	0.001802	0.10311	26	32	29	0	29	15	0.17706132	1	17.125	12.6314517								
5	9E+07	5_933505	9	26	15	8	18	12	0.307258	1	0	2	9	1	1	5	0.22510075	1	8.75	8.61477137								
5	9E+07	5_93722232	633	358	424	441	496	433	3.88E-05	0.00391	348	255	252	125	302	189	1.13E-04	0.0101411	354.5	152.599382					9.4E+07	9.4E+07	0.61273	ID=promoter_40391;
5	9E+07	5_937309	18	9	21	9	14	15	0.970741	1	33	30	15	5	32	10	0.00696943	0.2965071	17.5	10.11364								
5	9E+07	5_941140	1	0	7	1	1	4	0.505922	1	36	25	27	2	31	15	0.10937197	1	12.375	14.5399695								
5	9E+07	5_942423	40	28	0	6	34	3	1.56E-07	#######	200	147	0	4	174	2	1.68E-34	2.07E-31	53.125	76.9664816								
5	9E+07	5_942593	19	34	28	22	27	25	0.694422	1	35	0	18	6	18	12	0.67374005	1	20.25	12.4756906								
5	9E+07	5_943369	2	0	32	33	1	33	3.05E-06	0.00041	0	9	7	1	5	4	0.97610903	1	10.5	13.9693542								
5	9E+07	5_94402555	0	0	0	0	0	0	1	1	11	22	10	0	17	5	0.05852787	1	5.375	8.22778394					9.4E+07	9.4E+07	0.15058	ID=promoter_40406;
5	9E+07	5_94414999	0	8	19	23	4	21	0.010729	0.43446	39	1	1	23	20	12	0.48622029	1	14.25	14.0483858	9E+07	9.5E+07	ID=ENSP00000327416;PPFIA2;NM_003625;	PPFIA2				
5	9E+07	5_948172	28	11	2	0	20	1	3.58E-05	0.00364	42	11	2	22	27	12	0.09555594	1	14.75	14.8396188								

5	9E+07	5_949032	174	76	108	135	125	122	0.351332	1	9	29	28	1	19	15	0.89728723	1	70	63.7495098								
5	1E+08	5_956880	349	120	269	286	235	278	0.995399	1	207	82	142	32	145	87	0.00536463	0.2433219	185.88	110.014853								
5	1E+08	5_957237	129	51	81	60	90	71	0.042281	1	181	107	175	37	144	106	0.1957622	1	102.63	55.2292301								
5	1E+08	5_957247	598	366	456	441	482	449	0.00203	0.11403	130	233	197	182	182	190	0.5186988	1	325.38	164.814994								
5	1E+08	5_957641	13	19	1	12	16	7	0.076817	1	0	8	2	0	4	1	0.45332778	1	6.875	7.21976454								
5	1E+08	5_95895841	18	8	28	11	13	20	0.747034	1	47	42	11	1	45	6	1.38E-06	0.0002234	20.75	16.6626186					9.6E+07	9.6E+07	0.42886	ID=promoter_40439;
5	1E+08	5_959701	5	10	5	3	8	4	0.357158	1	15	8	27	1	12	14	0.84238319	1	9.25	8.39642781								
5	1E+08	5_961493	6	63	2	7	35	5	9.64E-07	0.00015	14	9	18	24	12	21	0.20312388	1	17.875	19.5626284								
5	1E+08	5_96151115	0	0	4	13	0	9	0.02157	0.74463	0	25	18	18	13	18	0.54030568	1	9.75	9.98212688					9.6E+07	9.6E+07	0.21769	ID=promoter_40450;
5	1E+08	5_962725	0	0	4	0	0	2	0.426327	1	0	50	15	0	25	8	0.02318551	0.7636665	8.625	17.5086713								
5	1E+08	5_96360755	79	127	101	90	103	96	0.267408	1	67	69	85	22	68	54	0.69683916	1	80	30.3079433	1E+08	9.6E+07	ID=ENSP0000376932;SYT1;NM_001135805;	SYT1				
5	1E+08	5_968259	12	15	8	7	14	8	0.253058	1	29	1	0	0	15	0	9.56E-04	0.060345	9	9.8270763								
5	1E+08	5_969698	6	27	14	3	17	9	0.140533	1	51	37	19	2	44	11	1.66E-04	0.0140498	19.875	17.5045912								
5	1E+08	5_970377	733	233	361	282	483	322	1.77E-14	#######	313	110	251	1	212	126	3.57E-04	0.0267297	285.5	214.613273								
5	1E+08	5_97058638	3820	2023	4003	2468	2922	3236	0.07925	1	1947	1553	1505	421	1750	963	2.94E-38	4.08E-35	2217.5	1201.57016					9.7E+07	9.7E+07	0.39717	ID=promoter_40480;
5	1E+08	5_970763	33	41	44	70	37	57	0.432208	1	69	58	61	90	64	76	0.3428502	1	58.25	18.5453113								
5	1E+08	5_971857	15	9	2	6	12	4	0.066899	1	25	3	5	2	14	4	0.08931834	1	8.375	7.99888385								
5	1E+08	5_97198655	473	172	329	332	323	331	0.224642	1	269	132	119	132	201	126	0.00246778	0.1303095	244.75	127.574908					9.7E+07	9.7E+07	0.63482	ID=promoter_40489;
5	1E+08	5_97225962	18	0	17	3	9	10	0.993074	1	10	0	21	2	5	12	0.17891578	1	8.875	8.75764972	1E+08	9.7E+07	ID=ENSP0000323246;E2F7;NM_203394;	E2F7	9.7E+07	9.7E+07	0.42789	ID=promoter_40495;
5	1E+08	5_972632	60	10	56	33	35	45	0.920357	1	16	3	32	11	10	22	0.06514386	1	27.625	21.4804729								
5	1E+08	5_973159	114	157	91	77	136	84	1.59E-05	0.00178	112	138	165	77	125	121	0.94415608	1	116.38	34.3342291								
5	1E+08	5_973780	0	26	3	2	13	3	0.018372	0.65917	9	21	9	47	15	28	0.0827714	1	14.625	15.9905106								
5	1E+08	5_975194	4	11	5	29	8	17	0.371907	1	18	30	47	2	24	25	0.92007618	1	18.25	15.9261689								
5	1E+08	5_977528	13	4	2	0	9	1	0.020382	0.71452	24	13	0	0	19	0	1.50E-04	0.0128633	7	8.79935063								
5	1E+08	5_979989	22	36	10	20	29	15	0.030102	0.97706	67	34	36	6	51	21	0.00566618	0.2544931	28.875	19.2237762								
5	1E+08	5_980407	0	10	14	1	5	8	0.86114	1	0	6	9	20	3	15	0.01140593	0.4370832	7.5	7.21110255								
5	1E+08	5_980818	18	22	12	18	20	15	0.418324	1	13	22	24	2	18	13	0.78939737	1	16.375	7.20986427								
5	1E+08	5_981176	50	147	22	12	99	17	2.09E-16	#######	354	199	30	54	277	42	1.33E-34	1.65E-31	108.5	119.098998								
5	1E+08	5_981937	0	0	6	8	0	7	0.050594	1	0	0	15	39	0	27	4.74E-07	8.50E-05	8.5	13.4589322								
5	1E+08	5_982648	8	41	2	17	25	10	0.01059	0.42992	12	21	29	31	17	30	0.09758228	1	20.125	13.0102981								
5	1E+08	5_98297911	51	42	66	37	47	52	0.958724	1	27	49	21	1	38	11	0.0015633	0.0891068	36.75	20.2043135					9.8E+07	9.8E+07	0.15947	ID=promoter_40525;
5	1E+08	5_983751	44	8	3	0	26	2	4.27E-06	0.00056	13	0	4	0	7	2	0.29886173	1	9	14.8612632								
5	1E+08	5_983874	46	39	11	15	43	13	2.33E-05	0.00251	94	53	47	52	74	50	0.22202085	1	44.625	25.6622986								
5	1E+08	5_984019	14	71	7	21	43	14	4.87E-05	0.00473	3	68	3	7	36	5	2.52E-05	0.0028067	24.25	28.5694642								
5	1E+08	5_985860	49	97	45	51	73	48	0.006824	0.29841	55	20	94	3	38	49	0.30854506	1	51.75	32.2523532								
5	1E+08	5_986486	12	12	2	10	12	6	0.213448	1	0	16	4	10	8	7	0.9937864	1	8.25	5.59974489								
5	1E+08	5_987081	8	25	18	15	17	17	0.897282	1	14	1	14	2	8	8	0.98858271	1	12.125	8.09651601								
5	1E+08	5_987352	68	67	37	23	68	30	2.10E-05	0.00229	49	39	40	22	44	31	0.50195989	1	43.125	17.4800907								
5	1E+08	5_98787587	9	43	29	32	26	31	0.998108	1	2	10	21	9	6	15	0.10150651	1	19.375	14.2321317					9.9E+07	9.9E+07	0.89573	ID=promoter_40539;
5	1E+08	5_98801164	21	26	13	13	24	13	0.071032	1	15	8	19	0	12	10	0.96947392	1	14.375	8.03452372					9.9E+07	9.9E+07	0.66499	ID=promoter_40543;
5	1E+08	5_98808690	154	86	126	148	120	137	0.976891	1	76	53	45	9	65	27	0.00152707	0.0883233	87.125	51.9022089					9.9E+07	9.9E+07	0.34292	ID=promoter_40544;
5	1E+08	5_98822734	10	25	17	10	18	14	0.509661	1	0	17	22	11	9	17	0.21129426	1	14	7.92824967					9.9E+07	9.9E+07	0.71806	ID=promoter_40548;
5	1E+08	5_98846694	59	17	24	37	38	31	0.320458	1	6	21	32	37	14	35	0.00480639	0.2209499	29.125	16.0306626					9.9E+07	9.9E+07	0.20794	ID=promoter_40552;
5	1E+08	5_98865927	17	27	20	9	22	15	0.260529	1	1	12	26	5	7	16	0.11989665	1	14.625	9.51596403					9.9E+07	9.9E+07	0.27562	ID=promoter_40557;
5	1E+08	5_98867131	16	25	18	18	21	18	0.61832	1	9	17	58	72	13	65	3.81E-09	1.01E-06	29.125	22.8688028					9.9E+07	9.9E+07	0.27562	ID=promoter_40557;

5	1E+08	5_98894666	75	23	18	41	49	30	0.017024	0.6223	0	6	0	1	3	1	0.65196691	1	20.5	26.2787258					9.9E+07	9.9E+07	0.37527	ID=promoter_40570;
5	1E+08	5_988990	836	584	1017	931	710	974	0.006429	0.2884	513	381	564	162	447	363	0.17232023	1	623.5	288.754468								
5	1E+08	5_989142	72	45	61	40	59	51	0.276863	1	75	24	46	31	50	39	0.72066795	1	49.25	18.5298986								
5	1E+08	5_989331	52	18	22	44	35	33	0.665929	1	32	11	15	1	22	8	0.06315916	1	24.375	17.1792025								
5	1E+08	5_994175	311	203	162	141	257	152	7.77E-11	#######	65	50	64	5	58	35	0.12965177	1	125.13	99.7503133								
5	1E+08	5_994830	34	22	5	0	28	3	4.80E-06	0.00062	0	9	10	0	5	5	0.99284883	1	10	12.224098								
5	1E+08	5_996434	21	0	15	23	11	19	0.589612	1	111	42	35	2	77	19	2.02E-07	3.97E-05	31.125	35.3732647								
5	1E+08	5_996752	0	12	2	4	6	3	0.462004	1	16	5	0	1	11	1	0.02232329	0.7455867	5	5.92814112								
5	1E+08	5_996839	62	43	62	115	53	89	0.115153	1	15	6	35	0	11	18	0.32230807	1	42.25	37.7349781								
5	1E+08	5_99715827	712	181	467	398	447	433	0.017619	0.63803	115	150	263	57	133	160	0.08376343	1	292.88	219.783944					1E+08	1E+08	0.61411	ID=promoter_40612;
5	1E+08	5_997243	115	38	110	86	77	98	0.866089	1	52	21	67	4	37	36	0.97888929	1	61.625	40.4154762								
5	1E+08	5_99746782	7	0	4	6	4	5	0.995512	1	17	0	9	9	9	9	0.98716474	1	6.5	5.52914357	1E+08	1E+08	ID=ENSP000003701 96;SBF1;;	SBF1	1E+08	1E+08	0.26078	ID=promoter_40619;
5	1E+08	5_997872	7	1	8	0	4	4	0.97482	1	18	9	14	0	14	7	0.39930956	1	7.125	6.64266513								
5	1E+08	5_998501	14	12	3	10	13	7	0.234546	1	4	21	19	1	13	10	0.90601506	1	10.5	7.42582367								
5	1E+08	5_99876859	7	38	29	33	23	31	0.880117	1	24	29	7	35	27	21	0.83317938	1	25.25	12.0326936	1E+08	1E+08	ID=ENSP000003791 83;PANX2;;	PANX2				
5	1E+08	5_99903910	69	97	112	59	83	86	0.722361	1	100	31	63	41	66	52	0.68004331	1	71.5	29.0516781	1E+08	1E+08	ID=ENSP000002627 94;MOV10L1;NM_018995;	MOV10L1	1E+08	1E+08	0.19692	ID=promoter_40655;
5	1E+08	5_99919942	28	49	26	14	39	20	0.008322	0.35239	18	27	31	25	23	28	0.62399108	1	27.25	10.3888677	1E+08	1E+08	ID=ENSP000002627 94;MOV10L1;NM_018995;	MOV10L1	1E+08	1E+08	0.69798	ID=promoter_40658;
5	1E+08	5_99922568	0	8	8	2	4	5	0.995512	1	0	6	11	9	3	10	0.11508955	1	5.5	4.27617987	1E+08	1E+08	ID=ENSP000002627 94;MOV10L1;NM_018995;	MOV10L1				
5	1E+08	5_99939254	148	190	52	78	169	65	9.06E-15	#######	81	64	39	141	73	90	0.19218681	1	99.125	53.7784808	1E+08	1E+08	ID=ENSP000002627 94;MOV10L1;NM_018995;	MOV10L1				
5	1E+08	5_99953256	20	27	23	28	24	26	0.961326	1	19	45	38	21	32	30	0.99902655	1	27.625	9.31875988	1E+08	1E+08	ID=ENSP000003792 16;MLC1;NM_139202;	MLC1	1E+08	1E+08	0.26376	ID=promoter_40669;
5	1E+08	5_99953925	56	9	27	55	33	41	0.970179	1	3	16	5	15	10	10	0.98574881	1	23.25	21.2518907	1E+08	1E+08	ID=ENSP000003792 16;MLC1;NM_139202;	MLC1	1E+08	1E+08	0.26376	ID=promoter_40669;
5	1E+08	5_999672	76	78	90	13	77	52	0.007815	0.3354	0	63	15	57	32	36	0.72740457	1	49	34.5667057								
5	1E+08	5_99978824	19	5	5	4	12	5	0.126784	1	3	6	5	0	5	3	0.8350419	1	5.875	5.61725657					1E+08	1E+08	0.44421	ID=promoter_40675;
5	1E+08	5_999882	20	0	11	14	10	13	0.970528	1	50	4	25	19	27	22	0.90387209	1	17.875	15.412773								
5	1E+08	5_99995267	113	101	242	252	107	247	1.91E-08	#######	40	35	39	52	38	46	0.47469033	1	109.25	89.9233801					1E+08	1E+08	0.92556	ID=promoter_40679;
5	1E+08	5_100011578	52	140	51	57	96	54	5.77E-05	0.0055	131	98	95	52	115	74	0.04809086	1	84.5	36.8897973					1E+08	1E+08	0.21895	ID=promoter_40684;
5	1E+08	5_100016601	6	8	9	1	7	5	0.693374	1	0	6	9	3	3	6	0.53745121	1	5.25	3.53553391	1E+08	1E+08	ID=ENSP000003839 38;CRELD2;NM_001135101;	CRELD2	1E+08	1E+08	0.39681	ID=promoter_40687;
5	1E+08	5_100031973	50	18	33	67	34	50	0.594713	1	0	0	0	2	0	1	0.58312783	1	21.25	26.1684106								

5	1E+08	5_100049127	20	3	22	47	12	35	0.018854	0.672	33	27	11	5	30	8	0.00380767	0.1842991	21	14.8227624	1E+08	1E+08	ID=ENSP000002661 82;TTLL8;NM_001080447;	TTLL8	1E+08	1E+08	0.46125	ID=promoter_40694;
5	1E+08	5_100050097	29	25	33	22	27	28	0.900886	1	50	6	28	0	28	14	0.15944813	1	24.125	15.5970464	1E+08	1E+08	ID=ENSP000002661 82;TTLL8;NM_001080447;	TTLL8	1E+08	1E+08	0.46125	ID=promoter_40694;
5	1E+08	5_100062854	13	13	5	3	13	4	0.041893	1	1	0	6	5	1	6	0.13767373	1	5.75	4.92080133	1E+08	1E+08	ID=ENSP000002661 82;TTLL8;NM_001080447;	TTLL8	1E+08	1E+08	0.44948	ID=promoter_40696;
5	1E+08	5_100063908	86	15	95	65	51	80	0.26579	1	16	32	20	5	24	13	0.28736897	1	41.75	35.1151168	1E+08	1E+08	ID=ENSP000002661 82;TTLL8;NM_001080447;	TTLL8	1E+08	1E+08	0.44948	ID=promoter_40696;
5	1E+08	5_100103468	6	3	1	2	5	2	0.402696	1	0	20	15	0	10	8	0.95278883	1	5.875	7.54865172								
5	1E+08	5_100105105	1856	665	1765	1451	1261	1608	0.08468	1	111	124	106	99	118	103	0.90512083	1	772.13	791.644299					1E+08	1E+08	0.38657	ID=promoter_40708;
5	1E+08	5_100143520	5	12	18	21	9	20	0.269594	1	21	12	31	22	17	27	0.21252244	1	17.75	7.95972002								
5	1E+08	5_100216398	7	1	2	0	4	1	0.312866	1	43	10	0	4	27	2	5.20E-05	0.0052176	8.375	14.4314884					1E+08	1E+08	0.76533	ID=promoter_40726;
5	1E+08	5_100219528	22	14	9	5	18	7	0.033499	1	0	0	6	9	0	8	0.01336935	0.4945372	8.125	7.31803057					1E+08	1E+08	0.76533	ID=promoter_40726;
5	1E+08	5_100255246	0	4	0	6	2	3	0.964182	1	23	4	7	0	14	4	0.08931834	1	5.5	7.59699189					1E+08	1E+08	0.30141	ID=promoter_40738;
5	1E+08	5_100270602	22	9	15	12	16	14	0.724026	1	32	43	22	21	38	22	0.20745497	1	22	11.0840941								
5	1E+08	5_100274922	34	59	41	42	47	42	0.437987	1	87	43	68	5	65	37	0.05725272	1	47.375	24.47703					1E+08	1E+08	0.31098	ID=promoter_40746;
5	1E+08	5_100288016	3	57	2	1	30	2	4.24E-07	#######	5	11	8	18	8	13	0.4490005	1	13.125	18.5890713					1E+08	1E+08	0.14785	ID=promoter_40750;
5	1E+08	5_100288287	156	78	92	105	117	99	0.055845	1	59	14	10	19	37	15	0.02098916	0.7098276	66.625	51.4585478					1E+08	1E+08	0.14785	ID=promoter_40750;
5	1E+08	5_100294498	192	54	126	80	123	103	0.040233	1	57	31	39	48	44	44	0.93879668	1	78.375	54.7068486					1E+08	1E+08	0.52171	ID=promoter_40752;
5	1E+08	5_100296016	9	22	11	2	16	7	0.076817	1	29	28	3	16	29	10	0.0194	0.6626766	15	10.5559733					1E+08	1E+08	0.52171	ID=promoter_40752;
5	1E+08	5_100309704	81	54	62	134	68	98	0.425599	1	0	0	0	0	0	0	1	1	41.375	50.1168278					1E+08	1E+08	0.49684	ID=promoter_40756;
5	1E+08	5_100310025	7	0	0	1	4	1	0.312866	1	14	11	6	13	13	10	0.90601506	1	6.5	5.78174467					1E+08	1E+08	0.49684	ID=promoter_40756;
5	1E+08	5_100319077	35	60	27	66	48	47	0.678251	1	31	35	56	42	33	49	0.10767024	1	44	14.6774852					1E+08	1E+08	0.46091	ID=promoter_40758;
5	1E+08	5_100340712	57	86	86	109	72	98	0.631157	1	111	43	60	93	77	77	0.89536519	1	80.625	24.8937026					1E+08	1E+08	0.11633	ID=promoter_40761;
5	1E+08	5_100344061	14	1	22	19	8	21	0.137587	1	0	6	10	23	3	17	0.00428697	0.2008879	11.875	9.09375767								
5	1E+08	5_100392769	26	26	18	33	26	26	0.847245	1	26	8	0	2	17	1	0.00146248	0.085933	17.375	12.5007143								
5	1E+08	5_100395150	10	31	23	27	21	25	0.998746	1	87	9	13	1	48	7	1.03E-06	0.0001704	25.125	26.9626064								
5	1E+08	5_100404889	20	90	25	23	55	24	1.43E-04	0.01212	51	65	49	17	58	33	0.0775783	1	42.5	25.967012								
5	1E+08	5_100410662	7	25	4	8	16	6	0.042495	1	12	7	15	0	10	8	0.95278883	1	9.75	7.66718425								
5	1E+08	5_100414387	95	69	172	179	82	176	2.68E-05	0.00281	33	64	27	28	49	28	0.11921514	1	83.375	61.5256915								

5	1E+08	5_100460066	66	175	55	82	121	69	6.58E-06	0.00082	141	93	147	79	117	113	0.95289901	1	104.75	43.6373038								
5	1E+08	5_100463414	6	9	20	0	8	10	0.991043	1	33	1	10	1	17	6	0.10713464	1	10	11.389218					1E+08	1E+08	0.28963	ID=promoter_40778;
5	1E+08	5_100505193	48	19	10	20	34	15	0.004584	0.21859	2	33	42	17	18	30	0.13648184	1	23.875	15.8153407								
6	5521	6_5521	136	49	105	110	93	108	0.997508	1	17	31	44	23	24	34	0.27690884	1	64.375	45.6443941					4822	6038	0.38755	ID=promoter_40784;
6	29033	6_29033	139	144	98	114	142	106	0.001829	0.10386	67	114	46	57	91	52	0.01925819	0.6608363	97.375	37.1327253								
6	30587	6_30587	14	18	0	0	16	0	8.41E-05	0.00761	10	0	14	2	5	8	0.62517663	1	7.25	7.55456343					30174	30979	0.1466	ID=promoter_40789;
6	53372	6_53372	13	1	5	15	7	10	0.922856	1	7	13	11	1	10	6	0.69729498	1	8.25	5.54848757					51438	54867	0.57824	ID=promoter_40796;
6	56698	6_56698	19	3	6	6	11	6	0.3028	1	16	11	11	19	14	15	0.92682316	1	11.375	6.16296543					55182	56810	0.26627	ID=promoter_40797;
6	79718	6_79718	16	38	4	14	27	9	0.002276	0.124	29	16	20	31	23	26	0.7859394	1	21	10.9674844					79212	80818	0.39391	ID=promoter_40806;
6	86835	6_86835	0	1	8	3	1	6	0.241978	1	25	19	11	9	22	10	0.16182597	1	9.5	8.78310066								
6	112520	6_112520	19	22	31	27	21	29	0.849881	1	17	8	15	19	13	17	0.64388579	1	19.75	7.1063352								
6	152354	6_152354	407	182	335	438	295	387	0.350082	1	185	102	117	64	144	91	0.01585269	0.5664395	228.75	144.737941					152042	153254	0.41096	ID=promoter_40821;
6	170428	6_170428	34	11	36	21	23	29	0.967038	1	0	19	2	2	10	2	0.09294023	1	15.625	14.2922307	159330	172123	ID=ENSP00003027 77;MC1R; NM_002386;	MC1R				
6	202003	6_202003	4	0	18	17	2	18	0.005391	0.24994	8	0	5	16	4	11	0.14780855	1	8.5	7.52139805					199003	202696	0.5758	ID=promoter_40830;
6	206453	6_206453	195	114	292	259	155	276	1.55E-04	0.01299	50	13	55	2	32	29	0.99609288	1	122.5	112.76651					205277	206625	0.28306	ID=promoter_40831;
6	215177	6_215177	131	58	43	65	95	54	7.98E-05	0.00729	21	40	31	0	31	16	0.15534889	1	48.625	39.0711348								
6	262804	6_262804	7	0	5	13	4	9	0.54411	1	19	18	10	1	19	6	0.05448017	1	9.125	7.1999504					243970	263648	0.99999	ID=promoter_40841;
6	327848	6_327848	49	105	94	57	77	76	0.564891	1	105	91	127	26	98	77	0.55365112	1	81.75	34.1373277					327444	328696	0.3566	ID=promoter_40854;
6	344606	6_344606	166	51	164	120	109	142	0.711811	1	19	60	56	28	40	42	0.85290898	1	83	58.8241933								
6	409293	6_409293	182	112	193	214	147	204	0.297944	1	134	93	147	36	114	92	0.61250986	1	138.88	58.4720629					407433	409872	0.46048	ID=promoter_40879;
6	414339	6_414339	59	8	18	4	34	11	3.70E-04	0.02765	15	13	3	1	14	2	0.01719769	0.6064547	15.125	18.7421412					413426	414628	0.31055	ID=promoter_40880;
6	427308	6_427308	103	59	101	72	81	87	0.849789	1	20	35	13	40	28	27	0.98939345	1	55.375	34.5126788								
6	459329	6_459329	83	92	86	84	88	85	0.441327	1	122	164	106	72	143	89	0.01212266	0.4594926	101.13	29.7486494								
6	461399	6_461399	36	22	18	20	29	19	0.13502	1	2	22	6	3	12	5	0.30420563	1	16.125	11.6917248					461107	461606	0.13874	ID=promoter_40892;
6	471017	6_471017	8	34	7	16	21	12	0.1273	1	2	0	13	0	1	7	0.08320466	1	10	11.3263284								
6	523879	6_523879	40	30	22	18	35	20	0.032214	1	5	4	14	1	5	8	0.62517663	1	16.75	13.6355627					523627	524834	0.28697	ID=promoter_40909;
6	532346	6_532346	43	40	23	23	42	23	0.010848	0.43721	0	6	0	0	3	0	0.24893445	1	16.875	17.9875949								
6	600488	6_600488	69	72	66	34	71	50	0.020206	0.71359	52	14	61	41	33	51	0.06897109	1	51.125	20.1737984	576275	662729	ID=ENSP00002842 62;JPH3; NM_020655;	JPH3	600248	601661	0.27485	ID=promoter_40933;
6	606075	6_606075	134	127	156	174	131	165	0.830862	1	166	53	62	28	110	45	1.24E-05	0.0015211	112.5	56.6190276	576275	662729	ID=ENSP00002842 62;JPH3; NM_020655;	JPH3	605513	606145	0.19791	ID=promoter_40934;

6	608514	6_608514	10	0	20	3	5	12	0.389128	1	0	0	20	0	0	10	0.00454609	0.2107253	6.625	8.92728562	576275	662729	ID=ENSP000002842 62;JPH3;NM_020655;	JPH3	608053	608770	0.21132	ID=promoter_40935;
6	701920	6_701920	2	0	13	8	1	11	0.032372	1	1	10	3	26	6	15	0.10150651	1	7.875	8.67570499					701382	703207	0.31993	ID=promoter_40950;
6	795429	6_795429	712	220	559	422	466	491	0.252383	1	259	286	187	126	273	157	9.35E-06	0.001193	346.38	202.069958	753439	796003	ID=ENSP0000310841;FBXO31;NM_024735;	FBXO31	794853	796723	0.54701	ID=promoter_40968;
6	827510	6_827510	14	11	9	16	13	13	0.920459	1	8	10	6	3	9	5	0.6500913	1	9.625	4.1726148					825829	828791	0.501	ID=promoter_40971;
6	855905	6_855905	14	30	16	8	22	12	0.091688	1	35	37	7	23	36	15	0.02815151	0.8902666	21.25	11.8049626	832132	866971	ID=ENSP0000268616;ZCCHC14;NM_015144;	ZCCHC14	853227	858149	0.79704	ID=promoter_40979;
6	858483	6_858483	25	94	47	25	60	36	0.00526	0.24489	17	23	28	3	20	16	0.90780655	1	32.75	27.5823442	832132	866971	ID=ENSP0000268616;ZCCHC14;NM_015144;	ZCCHC14				
6	932300	6_932300	405	212	479	323	309	401	0.409602	1	175	101	142	61	138	102	0.22063735	1	237.25	149.833002								
6	979578	6_979578	2278	1254	2027	1641	1766	1834	0.001261	0.07684	691	351	496	208	521	352	2.49E-05	0.0027797	1118.3	797.272807					979481	979983	0.12142	ID=promoter_40995;
6	1E+06	6_106829	8	0	0	0	4	0	0.09576	1	0	29	4	0	15	2	0.01114184	0.4293828	5.125	10.0773792								
6	1E+06	6_1113379	48	71	69	100	60	85	0.533334	1	56	50	66	31	53	49	0.99990602	1	61.375	20.4376229					1112884	1113667	0.15765	ID=promoter_41009;
6	1E+06	6_1126782	4	30	11	7	17	9	0.140533	1	23	0	13	2	12	8	0.76853133	1	11.25	10.5254793					1124897	1128687	0.60456	ID=promoter_41013;
6	1E+06	6_1128344	31	19	20	20	25	20	0.43889	1	10	2	4	23	6	14	0.1466131	1	16.125	9.93460762					1124897	1128687	0.60456	ID=promoter_41013;
6	1E+06	6_1130369	131	72	130	151	102	141	0.449211	1	99	98	51	34	99	43	1.19E-04	0.0105864	95.75	41.1677752					1129561	1131303	0.29149	ID=promoter_41014;
6	1E+06	6_1144469	20	2	3	6	11	5	0.189421	1	3	7	2	2	5	2	0.58563225	1	5.625	6.11642986					1140361	1145231	0.69068	ID=promoter_41018;
6	1E+06	6_1149683	97	51	82	50	74	66	0.267903	1	18	6	16	7	12	12	0.98292303	1	40.875	34.9139248					1149218	1149914	0.19386	ID=promoter_41020;
6	1E+06	6_1166373	78	89	103	132	84	118	0.449277	1	76	46	32	26	61	29	0.01058397	0.410878	72.75	36.4289916					1166205	1167811	0.32109	ID=promoter_41026;
6	1E+06	6_1170693	13	1	15	7	7	11	0.831726	1	0	13	7	1	7	4	0.73798468	1	7.125	6.05775772					1169334	1173096	0.56195	ID=promoter_41028;
6	1E+06	6_1172442	356	107	251	281	232	266	0.968395	1	19	25	53	1	22	27	0.61865829	1	136.63	138.673241					1169334	1173096	0.56195	ID=promoter_41028;
6	1E+06	6_1208364	3034	2791	3792	4639	2913	4216	2.59E-17	#######	546	437	762	472	492	617	3.41E-06	0.0004928	2059.1	1701.55261					1207871	1210203	0.43037	ID=promoter_41039;
6	1E+06	6_121319	40	61	38	22	51	30	0.00928	0.3835	73	169	149	59	121	104	0.8520635	1	76.375	53.6601408								
6	1E+06	6_123109	14	4	7	9	9	8	0.848833	1	0	20	27	17	10	22	0.06514386	1	12.25	8.94027804								
6	1E+06	6_1245039	39	24	35	27	32	31	0.749073	1	21	32	40	6	27	23	0.95651383	1	28	11.2122382					1244210	1247336	0.41569	ID=promoter_41051;
6	1E+06	6_1276028	0	40	5	0	20	3	4.27E-04	0.03093	1	0	5	19	1	12	0.00617711	0.2683255	8.75	14.13961					1274619	1276497	0.28524	ID=promoter_41062;
6	1E+06	6_1295223	7	7	19	2	7	11	0.831726	1	9	44	12	35	27	24	0.98883926	1	16.875	14.9803443					1294425	1295456	0.19969	ID=promoter_41070;
6	1E+06	6_129997	51	13	39	37	32	38	0.998681	1	8	17	14	0	13	7	0.50249747	1	22.375	17.7437112								
6	1E+06	6_130910	4	34	2	6	19	4	0.002035	0.11403	65	56	20	32	61	26	0.00307085	0.1550988	27.375	23.9101742								
6	1E+06	6_1317153	24	53	11	39	39	25	0.056901	1	40	45	80	28	43	54	0.32914056	1	40	20.8258082					1317013	1318535	0.1918	ID=promoter_41080;

6	1E+06	6_1347293	31	28	22	11	30	17	0.049763	1	28	0	8	4	14	6	0.26891969	1	16.5	12.1655251	1346999	1347773	0.15961	ID=promoter_41092;
6	1E+06	6_1365751	111	52	82	72	82	77	0.372886	1	24	61	66	38	43	52	0.43329818	1	63.25	26.7941891	1365085	1366176	0.22026	ID=promoter_41098;
6	1E+06	6_1410136	231	167	147	156	199	152	3.19E-04	0.0244	162	116	91	164	139	128	0.99851027	1	154.25	40.9311268	1409989	1411877	0.34663	ID=promoter_41112;
6	1E+06	6_1413123	549	205	520	681	377	601	1.69E-05	0.00188	299	189	466	204	244	335	1.63E-05	0.0019379	389.13	189.033208	1412369	1416942	0.86844	ID=promoter_41113;
6	1E+06	6_1415051	168	62	102	95	115	99	0.076861	1	32	52	32	30	42	31	0.627947	1	71.625	47.9700427	1412369	1416942	0.86844	ID=promoter_41113;
6	1E+06	6_1416351	696	234	781	655	465	718	2.18E-05	0.00237	328	232	402	139	280	271	0.8800014	1	433.38	244.359184	1412369	1416942	0.86844	ID=promoter_41113;
6	1E+06	6_1431738	22	34	22	25	28	24	0.526767	1	28	2	25	34	15	30	0.04484306	1	24	10.0711753	1431369	1432266	0.17155	ID=promoter_41117;
6	1E+06	6_1434469	1	55	6	10	28	8	6.70E-04	0.04525	7	7	25	51	7	38	6.79E-06	0.0009069	20.25	21.3992657	1434006	1434966	0.1496	ID=promoter_41118;
6	1E+06	6_144372	38	19	21	31	29	26	0.607762	1	5	14	28	13	10	21	0.0912903	1	21.125	10.7628661				
6	1E+06	6_1448037	71	35	56	32	53	44	0.234496	1	20	10	0	1	15	1	0.00364468	0.1775497	28.125	25.6316629	1446036	1448133	0.31698	ID=promoter_41122;
6	1E+06	6_147536	5	19	3	6	12	5	0.126784	1	0	8	6	0	4	3	0.96209564	1	5.875	6.03412912				
6	1E+06	6_147984	74	47	80	81	61	81	0.764855	1	60	34	48	16	47	32	0.40088139	1	55	23.1825057				
6	1E+06	6_1483553	40	19	35	12	30	24	0.372243	1	67	7	21	3	37	12	0.00419427	0.1971915	25.5	21.0984089	1483088	1484275	0.22692	ID=promoter_41134;
6	2E+06	6_1517980	38	21	437	463	30	450	4.32E-67	#######	38	14	167	43	26	105	1.99E-12	8.05E-10	152.63	189.8292	1516166	1518911	0.41602	ID=promoter_41144;
6	2E+06	6_152173	646	237	550	518	442	534	0.899724	1	217	196	136	143	207	140	0.01520064	0.5490489	330.38	205.488225				
6	2E+06	6_1562582	27	14	38	31	21	35	0.441201	1	15	13	11	1	14	6	0.26891969	1	18.75	12.1508377	1560905	1563645	0.41664	ID=promoter_41160;
6	2E+06	6_1573976	164	148	150	185	156	168	0.744333	1	92	31	49	19	62	34	0.04520648	1	104.75	65.3905847	1572904	1574449	0.3155	ID=promoter_41164;
6	2E+06	6_1594086	835	1203	0	0	1019	0	########	#######	85	210	5	2	148	4	4.55E-28	4.58E-25	292.5	464.656248	1592502	1597607	0.78057	ID=promoter_41170;
6	2E+06	6_1659331	25	8	19	10	17	15	0.722447	1	35	24	17	10	30	14	0.09649838	1	18.5	9.2736185	1659002	1659782	0.19252	ID=promoter_41186;
6	2E+06	6_1660929	7	11	13	22	9	18	0.416978	1	57	7	12	51	32	32	0.95510701	1	22.5	20.0570615	1660217	1661629	0.26582	ID=promoter_41187;
6	2E+06	6_1661030	9	1	20	12	5	16	0.124289	1	2	12	11	3	7	7	0.99000271	1	8.75	6.4531277	1660217	1661629	0.26582	ID=promoter_41187;
6	2E+06	6_1682114	117	31	133	67	74	100	0.652494	1	67	45	55	44	56	50	0.98152393	1	69.875	36.3374561	1678333	1682565	0.78796	ID=promoter_41189;
6	2E+06	6_178647	18	19	13	18	19	16	0.617999	1	0	6	20	3	3	12	0.04708211	1	12.125	7.98994904				
6	2E+06	6_1817675	360	185	265	231	273	248	0.014024	0.53325	164	98	93	10	131	52	6.72E-07	0.0001169	175.75	110.554124	1817168	1818737	0.48521	ID=promoter_41222;
6	2E+06	6_185938	103	36	100	108	70	104	0.309052	1	48	25	31	1	37	16	0.03310062	1	56.5	41.2691514				
6	2E+06	6_1881532	32	47	59	32	40	46	0.99583	1	40	48	26	24	44	25	0.1424362	1	38.5	12.1655251	1878919	1881918	0.51811	ID=promoter_41235;
6	2E+06	6_1942217	291	84	243	250	188	247	0.502899	1	153	120	85	70	137	78	0.00241906	0.1280458	162	87.2336763	1941761	1946234	0.832	ID=promoter_41252;
6	2E+06	6_196254	26	27	12	25	27	19	0.225928	1	49	23	41	4	36	23	0.37391478	1	25.875	14.3670009				
6	2E+06	6_196434	7	21	11	22	14	17	0.995406	1	27	11	16	0	19	8	0.15782218	1	14.375	8.84691229				
6	2E+06	6_197169	39	46	77	56	43	67	0.345384	1	69	75	218	126	72	172	2.32E-11	8.34E-09	88.25	58.7774252				
6	2E+06	6_1983567	137	61	220	158	99	189	3.85E-04	0.02845	139	6	68	41	73	55	0.50820884	1	103.75	71.1171066	1983186	1987308	0.65811	ID=promoter_41259;

6	2E+06	6_1983716	56	35	118	95	46	107	3.94E-04	0.02904	33	34	31	8	34	20	0.26570036	1	51.25	36.9700651					1983186	1987308	0.65811	ID=promoter_41259;
6	2E+06	6_1988338	101	55	52	42	78	47	0.001171	0.07205	10	8	24	35	9	30	0.00152443	0.0882297	40.875	29.9830309					1988108	1989200	0.22077	ID=promoter_41260;
6	2E+06	6_202238	0	34	13	1	17	7	0.051092	1	8	0	8	0	4	4	0.99427496	1	8	11.6004926								
6	2E+06	6_205348	14	30	3	11	22	7	0.005528	0.25484	16	3	10	34	10	22	0.06514386	1	15.125	11.444743								
6	2E+06	6_211616	4	30	22	9	17	16	0.818283	1	5	19	27	12	12	20	0.26508384	1	16	9.94269294								
6	2E+06	6_2123826	42	13	37	24	28	31	0.975765	1	17	11	4	1	14	3	0.04246461	1	18.625	14.7835575					2123544	2124281	0.11552	ID=promoter_41286;
6	2E+06	6_2133901	70	50	59	42	60	51	0.236466	1	13	54	47	14	34	31	0.99776322	1	43.625	20.3886348					2133453	2134129	0.12354	ID=promoter_41289;
6	2E+06	6_214148	5	0	8	6	3	7	0.596562	1	7	0	2	26	4	14	0.04184353	1	6.75	8.3623305								
6	2E+06	6_216231	3	17	9	8	10	9	0.84607	1	12	34	42	20	23	31	0.39238725	1	18.125	13.5165243								
6	2E+06	6_218052	11	13	9	4	12	7	0.325249	1	0	0	9	3	0	6	0.0393172	1	6.125	5.02671435								
6	2E+06	6_2202249	984	692	1331	886	838	1109	0.030825	0.98949	767	566	669	310	667	490	4.63E-04	0.033237	775.63	303.133604					2200850	2202642	0.29239	ID=promoter_41299;
6	2E+06	6_2266725	11	17	34	42	14	38	0.021991	0.75736	22	15	5	19	19	12	0.57792516	1	20.625	12.082307	2E+06	2272049	ID=ENSP00000258180;KIAA0513;NM_014732;	KIAA0513	2265891	2268658	0.52215	ID=promoter_41313;
6	2E+06	6_230448	32	30	55	46	31	51	0.328261	1	15	29	22	23	22	23	0.92188352	1	31.5	13.1257653								
6	2E+06	6_2374200	8	5	12	5	7	9	0.983538	1	5	0	4	2	3	3	0.99570314	1	5.125	3.64250699	2E+06	2380693	ID=ENSP00000341681;ZDHHC7;NM_001145548;	ZDHHC7	2373243	2375009	0.40736	ID=promoter_41332;
6	2E+06	6_2378916	539	485	515	671	512	593	0.978236	1	692	371	529	312	532	421	0.05238367	1	514.25	130.535983	2E+06	2380693	ID=ENSP00000341681;ZDHHC7;NM_001145548;	ZDHHC7	2377234	2382733	0.85782	ID=promoter_41333;
6	2E+06	6_239305	203	68	147	124	136	136	0.420177	1	21	46	14	33	34	24	0.59108388	1	82	68.5231973								
6	2E+06	6_2413973	93	82	118	262	88	190	9.66E-06	0.00116	92	92	180	128	92	154	3.19E-05	0.003448	130.88	61.6752729					2413919	2414466	0.1837	ID=promoter_41342;
6	2E+06	6_243335	90	69	72	32	80	52	0.00351	0.17713	96	31	33	22	64	28	0.00316776	0.1594859	55.625	29.437282								
6	2E+06	6_246397	21	7	46	28	14	37	0.029025	0.94901	0	7	17	1	4	9	0.31291743	1	15.875	15.6427756								
6	2E+06	6_2481077	405	218	545	485	312	515	1.07E-05	0.00127	287	118	112	102	203	107	1.05E-05	0.0013232	284	176.38918					2480637	2482292	0.24569	ID=promoter_41359;
6	3E+06	6_2569907	219	44	138	95	132	117	0.086296	1	32	5	12	0	19	6	0.05448017	1	68.125	77.4881327					2569319	2570532	0.27514	ID=promoter_41376;
6	3E+06	6_2622448	9	46	20	35	28	28	0.83651	1	46	39	12	3	43	8	2.17E-05	0.0024827	26.25	17.318446					2621948	2623503	0.19545	ID=promoter_41389;
6	3E+06	6_2717101	13	3	4	3	8	4	0.357158	1	35	10	6	8	23	7	0.02502936	0.8114813	10.25	10.6066017	3E+06	2734124	ID=ENSP00000262429;ATP2C2;NM_014861;	ATP2C2	2715823	2717555	0.23953	ID=promoter_41400;
6	3E+06	6_2752985	29	24	21	26	27	24	0.611598	1	7	0	2	19	4	11	0.14780855	1	16	11.3389342	3E+06	2765116	ID=ENSP00000343635;KIAA1609;NM_020947;	KIAA1609	2752709	2753685	0.21572	ID=promoter_41412;
6	3E+06	6_294982	16	17	12	3	17	8	0.087954	1	16	21	22	43	19	33	0.08582638	1	18.75	11.4361582								
6	3E+06	6_305764	11	25	6	19	18	13	0.404491	1	0	18	35	1	9	18	0.15524483	1	14.375	12.1882555								
6	3E+06	6_3129263	42	54	26	51	48	39	0.230677	1	13	40	37	28	27	33	0.56497566	1	36.375	13.5955613	3E+06	3140351	ID=ENSP00000344223;MBTPS1;NM_003791;	MBTPS1				
6	3E+06	6_3135374	13	58	19	42	36	31	0.4485	1	58	48	13	14	53	14	4.84E-05	0.004961	33.125	20.3991421	3E+06	3140351	ID=ENSP00000344223;MBTPS1;NM_003791;	MBTPS1				
6	3E+06	6_315493	60	34	17	50	47	34	0.095427	1	1	0	9	14	1	12	0.00617711	0.2683255	23.125	22.5035711								
6	3E+06	6_326454	11	11	7	5	11	6	0.3028	1	10	5	7	15	8	11	0.69450319	1	8.875	3.48209707								
6	3E+06	6_328792	7	4	5	4	6	5	0.851367	1	8	2	14	6	5	10	0.35527535	1	6.25	3.65474252								

6	3E+06	6_348952	121	142	112	106	132	109	0.024353	0.82541	97	36	71	53	67	62	0.99994762	1	92.25	35.9751899								
6	4E+06	6_407581	17	19	6	12	18	9	0.098614	1	0	8	8	0	4	4	0.99427496	1	8.75	7.02546389								
6	5E+06	6_458600	67	24	60	46	46	53	0.996073	1	12	17	45	13	15	29	0.06124724	1	35.5	21.7977718								
6	5E+06	6_4719325	29	18	69	58	24	64	0.001979	0.11184	55	45	12	35	50	24	0.02621347	0.8452651	40.125	20.1028605	5E+06	4815079	ID=ENSP00000362369-D2;PLCG1;NM_002660;	PLCG1	4719032	4719553	0.14767	ID=promoter_41491;
6	5E+06	6_4771088	5	15	1	5	10	3	0.082185	1	9	20	35	19	15	27	0.11059826	1	13.625	11.0703917	5E+06	4815079	ID=ENSP00000362369-D2;PLCG1;NM_002660;	PLCG1				
6	5E+06	6_4773987	24	32	20	12	28	16	0.064038	1	30	6	6	1	18	4	0.01933304	0.6616555	16.375	11.7830083	5E+06	4815079	ID=ENSP00000362369-D2;PLCG1;NM_002660;	PLCG1				
6	5E+06	6_4791393	58	96	23	29	77	26	3.67E-08	#######	60	78	95	37	69	66	0.98360752	1	59.5	28.5507068	5E+06	4815079	ID=ENSP00000362369-D2;PLCG1;NM_002660;	PLCG1				
6	5E+06	6_4846652	11	6	14	3	9	9	0.944235	1	7	8	10	13	8	12	0.56746184	1	9	3.7032804					4845867	4848163	0.45298	ID=promoter_41507;
6	5E+06	6_499090	3	12	18	7	8	13	0.766846	1	16	9	4	21	13	13	0.98151319	1	11.25	6.62786326								
6	5E+06	6_5105949	11	0	7	6	6	7	0.999953	1	120	50	30	2	85	16	7.14E-10	2.15E-07	28.25	40.7527387	5E+06	5122077	ID=ENSP00000248272;GAN;NM_022041;	GAN				
6	5E+06	6_514594	33	142	48	81	88	65	0.01708	0.62404	105	67	115	28	86	72	0.81566698	1	77.375	40.9631803								
6	5E+06	6_522160	5	7	3	27	6	15	0.276126	1	0	2	9	1	1	5	0.22510075	1	6.75	8.73007936								
6	5E+06	6_523898	11	9	5	18	10	12	0.998584	1	4	7	17	28	6	23	0.00349909	0.1735359	12.375	8.14050718								
6	5E+06	6_524111	7	27	12	11	17	12	0.395844	1	6	8	50	17	7	34	4.74E-05	0.004869	17.25	14.8876747								
6	5E+06	6_5267370	9	31	4	11	20	8	0.026297	0.87605	5	16	20	12	11	16	0.51039395	1	13.5	8.83176087					5253380	5282600	1	ID=promoter_41562;
6	5E+06	6_5291040	38	163	24	48	101	36	6.45E-10	#######	82	77	54	81	80	68	0.87071503	1	70.875	42.8233498					5290921	5302658	0.99783	ID=promoter_41564;
6	5E+06	6_5298395	26	83	27	57	55	42	0.107828	1	81	50	52	28	66	40	0.10191864	1	50.5	22.9595918					5290921	5302658	0.99783	ID=promoter_41564;
6	5E+06	6_530503	8	34	6	5	21	6	0.004166	0.20266	4	12	3	2	8	3	0.3843038	1	9.25	10.4846827								
6	5E+06	6_532629	7	28	26	34	18	30	0.49591	1	61	29	36	42	45	39	0.95370772	1	32.875	15.3104773								
6	5E+06	6_543643	32	8	16	15	20	16	0.517469	1	47	13	20	18	30	19	0.42545995	1	21.125	12.5406482								
6	6E+06	6_550592	24	51	8	7	38	8	4.14E-06	0.00055	14	4	27	33	9	30	0.00152443	0.0882297	21	16								
6	6E+06	6_554897	8	3	2	2	6	2	0.258649	1	16	0	16	0	8	8	0.98858271	1	5.875	6.7281392								
6	6E+06	6_559858	2	15	13	17	9	15	0.704209	1	20	6	22	2	13	12	0.99994263	1	12.125	7.88194139								
6	6E+06	6_566547	10	21	8	2	16	5	0.021336	0.74103	19	3	10	17	11	14	0.73202476	1	11.25	7.12640963								
6	6E+06	6_569658	0	29	2	3	15	3	0.006447	0.2884	23	0	18	0	12	9	0.89054267	1	9.375	11.9754213								
6	6E+06	6_585852	8	18	5	6	13	6	0.146758	1	0	1	0	2	1	1	0.99856566	1	5	6.02376247								
6	6E+06	6_5912638	81	25	49	42	53	46	0.324538	1	0	9	22	24	5	23	0.00150862	0.0878849	31.5	25.5063017					5912330	5912838	0.15768	ID=promoter_41598;
6	6E+06	6_595627	435	433	555	400	434	478	0.637006	1	363	335	377	158	349	268	0.06745411	1	382	112.398526								
6	6E+06	6_5963879	814	239	576	684	527	630	0.949553	1	348	258	200	198	303	199	7.42E-04	0.0492767	414.63	242.28374					5963379	5965713	0.54324	ID=promoter_41604;
6	6E+06	6_597197	685	253	380	254	469	317	1.59E-13	#######	50	111	74	52	81	63	0.57838304	1	232.38	218.16307								
6	6E+06	6_6140681	682	710	1253	1076	696	1165	8.12E-13	#######	528	481	468	432	505	450	0.83011458	1	703.75	304.886279					6139650	6140973	0.18958	ID=promoter_41615;
6	6E+06	6_6181028	13	15	7	8	14	8	0.253058	1	0	12	9	3	6	6	0.99142475	1	8.375	5.06916448					6181003	6181523	0.10922	ID=promoter_41618;
6	6E+06	6_628294	20	0	19	3	10	11	0.989255	1	8	0	0	2	4	1	0.45332778	1	6.5	8.45154255								
6	6E+06	6_632546	78	9	45	48	44	47	0.905244	1	5	1	8	1	3	5	0.7175395	1	24.375	28.8639048								
6	6E+06	6_6404336	46	35	65	24	41	45	0.953743	1	9	29	3	22	19	13	0.6985193	1	29.125	19.8957102					6402832	6404695	0.20667	ID=promoter_41631;

6	6E+06	6_6475634	35	74	37	38	55	38	0.039881	1	59	12	36	5	36	21	0.23487	1	37	22.3862203					6475273	6476083	0.10535	ID=promoter_41639;
6	7E+06	6_6529129	28	2	12	20	15	16	0.965652	1	14	8	0	0	11	0	0.00610443	0.2666968	10.5	10.0711753					6528490	6529545	0.19915	ID=promoter_41650;
6	7E+06	6_6554009	4	3	8	10	4	9	0.54411	1	0	1	18	16	1	17	4.35E-04	0.0314643	7.5	6.76123404					6552531	6554442	0.23273	ID=promoter_41652;
6	7E+06	6_655445	0	38	25	8	19	17	0.718296	1	13	5	26	4	9	15	0.36943449	1	14.875	13.3570474								
6	7E+06	6_659591	149	55	106	41	102	74	0.006439	0.2884	29	9	0	0	19	0	1.50E-04	0.0128633	48.625	53.6521535								
6	7E+06	6_6639020	0	9	0	1	5	1	0.186119	1	39	1	4	3	20	4	0.00863789	0.3495868	7.125	13.2172777					6638254	6639165	0.1761	ID=promoter_41667;
6	7E+06	6_6648908	9	16	5	22	13	14	0.975695	1	26	8	42	17	17	30	0.09758228	1	18.125	12.0171009					6648209	6649182	0.14147	ID=promoter_41668;
6	7E+06	6_6664828	17	24	1	7	21	4	7.04E-04	0.04721	31	0	8	23	16	16	0.97729577	1	13.875	11.5193936					6664623	6665132	0.14176	ID=promoter_41674;
6	7E+06	6_6676245	4	5	9	8	5	9	0.74134	1	5	1	12	44	3	28	1.56E-05	0.0018671	11	13.7529218					6675568	6676690	0.19523	ID=promoter_41677;
6	7E+06	6_6740471	2471	1608	1891	1697	2040	1794	4.84E-18	#######	204	286	257	103	245	180	0.05970358	1	1064.6	947.165235					6739224	6740480	0.25341	ID=promoter_41692;
6	7E+06	6_678097	57	34	39	90	46	65	0.625996	1	16	41	47	1	29	24	0.91882689	1	40.625	26.6454901								
6	7E+06	6_6895838	219	75	145	135	147	140	0.209818	1	73	45	44	60	59	52	0.96530969	1	99.5	61.556014					6895454	6896288	0.14247	ID=promoter_41719;
6	7E+06	6_7160285	115	96	110	82	106	96	0.183774	1	19	4	15	3	12	9	0.89054267	1	55.5	49.6185449	7E+06	7218463	ID=ENSP0000303129;VAT1L;NM_020927;	VAT1L				
6	7E+06	6_747963	1	56	30	39	29	35	0.993567	1	70	92	72	3	81	38	0.00195927	0.1077881	45.375	33.0451531								
6	8E+06	6_781573	6	39	12	4	23	8	0.007066	0.30636	5	12	29	49	9	39	2.59E-05	0.0028712	19.5	17.2626765								
6	8E+06	6_806321	527	307	426	333	417	380	0.001714	0.09966	343	233	294	143	288	219	0.08630719	1	325.75	116.042788								
6	9E+06	6_859688	20	145	29	53	83	41	1.81E-05	0.00201	83	69	84	83	76	84	0.53879257	1	70.75	38.9606028								
6	9E+06	6_864600	6	28	4	18	17	11	0.29617	1	2	1	8	30	2	19	5.26E-04	0.0367435	12.125	11.6672619								
6	9E+06	6_883388	12	19	8	16	16	12	0.497978	1	24	26	19	8	25	14	0.3120942	1	16.5	6.80336051								
6	9E+06	6_888075	1	20	3	3	11	3	0.050503	1	2	23	6	1	13	4	0.12786863	1	7.375	8.89522344								
6	9E+06	6_8897838	0	18	5	11	9	8	0.848833	1	0	16	29	2	8	16	0.19094317	1	10.125	10.3570197	9E+06	9010084	ID=ENSP0000283882;CFDP1;NM_006324;	CFDP1				
6	1E+07	6_953665	36	40	21	13	38	17	0.002761	0.14629	5	5	1	7	5	4	0.97610903	1	16	14.918828								
6	1E+07	6_991319	1	0	2	1	1	2	0.907382	1	41	1	0	1	21	1	2.34E-04	0.0188551	5.875	14.2070154								
6	1E+07	6_997980	155	17	130	128	86	129	0.208372	1	85	27	73	28	56	51	0.99579377	1	80.375	53.4306961								
6	1E+07	6_100696	56	78	40	56	67	48	0.031176	0.99786	77	61	27	69	69	48	0.30878603	1	58	17.6635217								
6	1E+07	6_105132	8	9	30	49	9	40	5.30E-04	0.03711	1	9	16	5	5	11	0.25523295	1	15.875	16.0039058								
6	1E+07	6_105670	12	11	10	17	12	14	0.999907	1	3	50	39	12	27	26	0.99047429	1	19.25	16.3160745								
6	1E+07	6_110519	14	47	25	4	31	15	0.014573	0.54886	33	9	20	12	21	16	0.83919704	1	20.5	14.1320305								
6	1E+07	6_11163668	3	15	16	25	9	21	0.212308	1	6	20	10	7	13	9	0.79589064	1	12.75	7.55456343	1E+07	1.1E+07	ID=ENSP0000268489;ZFHX3;NM_006885;	ZFHX3	1.1E+07	1.1E+07	0.15788	ID=promoter_41847;
6	1E+07	6_11195846	10	30	4	12	20	8	0.026297	0.87605	0	0	0	1	0	1	0.58312783	1	7.125	10.3845696	1E+07	1.1E+07	ID=ENSP0000268489;ZFHX3;NM_006885;	ZFHX3				
6	1E+07	6_11233788	35	34	17	19	35	18	0.013919	0.53027	19	8	13	0	14	7	0.39930956	1	18.125	11.933596	1E+07	1.1E+07	ID=ENSP0000268489;ZFHX3;NM_006885;	ZFHX3				
6	1E+07	6_11252067	147	101	198	138	124	168	0.475886	1	277	354	218	66	316	142	2.22E-12	8.93E-10	187.38	94.9495167	1E+07	1.1E+07	ID=ENSP0000268489;ZFHX3;NM_006885;	ZFHX3				
6	1E+07	6_11384102	20	0	23	4	10	14	0.912507	1	0	0	0	27	0	14	5.26E-04	0.0367435	9.25	11.889371					1.1E+07	1.1E+07	0.13989	ID=promoter_41872;

6	1E+07	6_114608	75	49	101	60	62	81	0.815289	1	68	64	53	28	66	41	0.1282584	1	62.25	21.1778186								
6	1E+07	6_114947	7	4	12	6	6	9	0.896349	1	0	16	0	22	8	11	0.69450319	1	8.375	7.78161753								
6	1E+07	6_117669	38	0	40	38	19	39	0.128333	1	0	0	4	6	0	5	0.06742467	1	15.75	19.1068425								
6	1E+07	6_118147	21	41	3	14	31	9	3.43E-04	0.02597	1	10	29	3	6	16	0.069189	1	15.25	14.2302495								
6	1E+07	6_118406	15	14	28	33	15	31	0.189122	1	1	32	23	36	17	30	0.09758228	1	22.75	11.9731843								
6	1E+07	6_12122408	427	150	339	323	289	331	0.957123	1	183	110	110	135	147	123	0.70324537	1	222.13	122.635858					1.2E+07	1.2E+07	0.35359	ID=promoter_41877;
6	1E+07	6_127880	39	42	31	17	41	24	0.022233	0.76438	52	59	64	13	56	39	0.38794685	1	39.625	18.6082739								
6	1E+07	6_12872970	39	11	21	44	25	33	0.905451	1	0	47	5	6	24	6	0.00867909	0.3505674	21.625	19.078316	1E+07	1.3E+07	ID=ENSP0000373485;TSNAXIP1;NM_018430;	TSNAXIP1				
6	1E+07	6_13082180	119	73	105	158	96	132	0.494904	1	71	58	57	26	65	42	0.18631934	1	83.375	41.7439046					1.3E+07	1.3E+07	0.3642	ID=promoter_41907;
6	1E+07	6_133627	90	11	36	76	51	56	0.943505	1	0	18	37	0	9	19	0.11219606	1	33.5	33.8230691								
6	1E+07	6_13393912	70	33	29	16	52	23	2.73E-04	0.02129	23	88	29	34	56	32	0.08797895	1	40.25	25.058503					1.3E+07	1.3E+07	0.32391	ID=promoter_41923;
6	1E+07	6_13397949	314	128	140	210	221	175	4.83E-04	0.03434	175	61	67	19	118	43	3.87E-07	7.16E-05	139.25	94.6810435	1E+07	1.3E+07	ID=ENSP0000264009;HSF4;NM_001040667;	HSF4	1.3E+07	1.3E+07	0.76315	ID=promoter_41924;
6	1E+07	6_134515	47	18	58	23	33	41	0.970179	1	46	12	32	17	29	25	0.96515949	1	31.625	16.8941029								
6	1E+07	6_135653	17	37	25	29	27	27	0.841861	1	9	2	20	3	6	12	0.2888538	1	17.75	12.5214102								
6	1E+07	6_140806	7	11	32	36	9	34	0.004304	0.20787	49	12	28	25	31	27	0.97229028	1	25	14.36265								
6	1E+07	6_142608	56	96	61	50	76	56	0.028793	0.94357	61	39	98	91	50	95	1.50E-04	0.0128633	69	22.7156334								
6	1E+07	6_142756	93	64	134	96	79	115	0.334111	1	80	82	61	30	81	46	0.02749004	0.8743833	80	30.3832661								
6	1E+07	6_144004	7	19	6	8	13	7	0.234546	1	7	12	18	3	10	11	0.92604858	1	10	5.80640041								
6	1E+07	6_144598	22	4	1	22	13	12	0.835334	1	31	45	8	2	38	5	1.05E-05	0.0013232	16.875	15.8604405								
6	1E+07	6_145336	26	86	44	50	56	47	0.235754	1	91	34	69	47	63	58	0.99927361	1	55.875	23.7212232								
6	1E+07	6_147727	44	56	20	30	50	25	0.001605	0.09378	48	25	50	2	37	26	0.55440389	1	34.375	18.3298157								
6	2E+07	6_159221	15	9	3	11	12	7	0.325249	1	0	12	3	1	6	2	0.42506085	1	6.75	5.67576301								
6	2E+07	6_160379	0	9	0	9	5	5	0.968625	1	9	6	10	2	8	6	0.92562801	1	5.625	4.30738568								
6	2E+07	6_160532	34	27	5	17	31	11	0.001462	0.08667	0	66	14	1	33	8	0.00121322	0.0738375	20.5	22.0064925								
6	2E+07	6_165596	4	19	29	16	12	23	0.379064	1	19	11	12	5	15	9	0.58227053	1	14.375	8.21040281								
6	2E+07	6_167491	0	26	0	6	13	3	0.018372	0.65917	4	0	10	4	2	7	0.20455692	1	6.25	8.71370021								
6	2E+07	6_180553	55	62	51	28	59	40	0.025416	0.8545	29	38	52	21	34	37	0.79599102	1	42	14.9857075								
6	2E+07	6_185473	38	94	38	70	66	54	0.142619	1	159	99	111	55	129	83	0.03317832	1	83	41.300294								
6	2E+07	6_187350	23	11	7	15	17	11	0.29617	1	10	0	5	1	5	3	0.8350419	1	9	7.57816412								
6	2E+07	6_192273	154	95	113	90	125	102	0.023788	0.81042	22	45	22	2	34	12	0.01147783	0.4380166	67.875	53.1182979								
6	2E+07	6_19334986	52	37	17	17	45	17	1.50E-04	0.01267	57	18	25	13	38	19	0.08276384	1	29.5	17.1380946	2E+07	1.9E+07	ID=ENSP0000377823;NDRG4;NM_001130487;	NDRG4	1.9E+07	1.9E+07	0.19588	ID=promoter_41945;
6	2E+07	6_19520008	87	52	145	152	70	149	1.65E-04	0.01374	96	26	56	23	61	40	0.23163204	1	79.625	49.6241229					2E+07	2E+07	0.18928	ID=promoter_41951;
6	2E+07	6_19598078	20	13	5	7	17	6	0.027279	0.89953	17	18	19	8	18	14	0.88543974	1	13.375	5.97464882					2E+07	2E+07	0.1763	ID=promoter_41954;
6	2E+07	6_196819	0	13	5	11	7	8	0.998739	1	1	9	11	1	5	6	0.91271846	1	6.375	5.26273965								
6	2E+07	6_197233	20	2	4	13	11	9	0.722819	1	21	3	0	1	12	1	0.01422424	0.5202299	8	8.68496237								
6	2E+07	6_197415	21	19	16	13	20	15	0.418324	1	23	0	31	22	12	27	0.03064003	0.9477626	18.125	9.04650682								
6	2E+07	6_197573	61	40	64	30	51	47	0.48921	1	4	6	30	13	5	22	0.00242132	0.1280458	31	23.1455025								
6	2E+07	6_198260	23	57	44	13	40	29	0.13759	1	69	3	9	3	36	6	6.62E-05	0.006417	27.625	25.740116								
6	2E+07	6_198368	67	1	39	12	34	26	0.254409	1	120	3	11	0	62	6	7.45E-10	2.23E-07	31.625	42.5673836								
6	2E+07	6_19855211	49	0	33	22	25	28	0.986003	1	0	0	10	13	0	12	0.00154584	0.0883233	15.875	17.836059					2E+07	2E+07	0.24621	ID=promoter_41962;
6	2E+07	6_200203	0	6	1	0	3	1	0.508575	1	25	21	18	13	23	16	0.67590535	1	10.5	10.0995049								
6	2E+07	6_200709	9	28	6	2	19	4	0.002035	0.11403	0	18	9	14	9	12	0.70935077	1	10.75	9.11435289								
6	2E+07	6_202669	10	56	7	9	33	8	5.52E-05	0.00528	13	15	16	23	14	20	0.45746548	1	18.625	15.9009209								
6	2E+07	6_20363841	162	60	130	149	111	140	0.849442	1	35	1	10	2	18	6	0.0767986	1	68.625	68.2431315					2E+07	2E+07	0.18281	ID=promoter_41976;
6	2E+07	6_206125	16	17	8	15	17	12	0.395844	1	18	13	26	19	16	23	0.39752759	1	16.5	5.15474816								
6	2E+07	6_206610	38	32	69	57	35	63	0.123062	1	17	37	55	17	27	36	0.35693007	1	40.25	18.8584956								
6	2E+07	6_20813926	43	33	18	13	38	16	0.001589	0.09301	16	30	44	56	23	50	0.00229649	0.1233633	31.625	15.3895837	2E+07	2.1E+07	ID=ENSP0000262134;LPCAT2;NM_017839;	LPCAT2				

6	2E+07	6_208324	6	18	3	8	12	6	0.213448	1	6	0	21	1	3	11	0.0740992	1	7.875	7.69856202							
6	2E+07	6_208504	42	30	68	104	36	86	0.001237	0.07553	0	11	9	14	6	12	0.2888538	1	34.75	35.5397162							
6	2E+07	6_208768	0	1	5	1	1	3	0.700657	1	11	0	11	17	6	14	0.1466131	1	5.75	6.47522752							
6	2E+07	6_210009	318	205	447	240	262	344	0.388014	1	137	125	101	32	131	67	3.12E-04	0.0239121	200.63	133.188307							
6	2E+07	6_211489	8	22	16	39	15	28	0.341844	1	44	32	47	75	38	61	0.02681985	0.855162	35.375	21.0708838							
6	2E+07	6_212449	10	26	5	6	18	6	0.017303	0.62786	28	38	26	1	33	14	0.04245489	1	17.5	13.5857068							
6	2E+07	6_214200	47	24	10	26	36	18	0.009725	0.39843	90	0	7	22	45	15	0.00163449	0.0924179	28.25	28.8084809							
6	2E+07	6_214618	10	10	13	5	10	9	0.84607	1	26	52	7	0	39	4	2.33E-06	0.0003543	15.375	16.6127103							
6	2E+07	6_214759	18	4	1	26	11	14	0.979671	1	0	0	12	2	0	7	0.02292696	0.7568963	7.875	9.77515071							
6	2E+07	6_215430	0	22	2	19	11	11	0.932271	1	0	26	10	1	13	6	0.35360803	1	10	10.8627805							
6	2E+07	6_216262	0	8	7	9	4	8	0.677894	1	20	1	7	1	11	4	0.25131486	1	6.625	6.47936284							
6	2E+07	6_21642114	16	14	6	18	15	12	0.610117	1	34	9	9	5	22	7	0.03554381	1	13.875	9.3722615				2.2E+07	2.2E+07	0.17191	ID=promoter_42029;
6	2E+07	6_222641	11	51	8	19	31	14	0.008767	0.36763	25	11	27	9	18	18	0.97449422	1	20.125	14.4562147							
6	2E+07	6_224043	1	8	0	1	5	1	0.186119	1	10	3	9	10	7	10	0.67636194	1	5.25	4.39967531							
6	2E+07	6_228294	28	23	16	25	26	21	0.441319	1	31	19	8	2	25	5	0.00263335	0.136745	19	9.97138764							
6	2E+07	6_231785	711	570	462	482	641	472	9.38E-14	#######	214	247	162	44	231	103	2.17E-09	5.97E-07	361.5	228.571964							
6	2E+07	6_232502	0	0	7	2	0	5	0.118674	1	25	0	6	0	13	3	0.06317581	1	5	8.56904729							
6	2E+07	6_234233	1893	1381	2142	1660	1637	1901	0.956885	1	1092	983	1398	574	1038	986	0.86067516	1	1390.4	508.761223							
6	2E+07	6_23435322	202	95	148	82	149	115	0.003125	0.16098	73	74	21	8	74	15	2.74E-08	6.33E-06	87.875	63.2352467				2.3E+07	2.3E+07	0.37774	ID=promoter_42064;
6	2E+07	6_234386	1	23	1	7	12	4	0.066899	1	0	5	18	4	3	11	0.0740992	1	7.375	8.53459347							
6	2E+07	6_236592	6	23	13	4	15	9	0.269309	1	3	13	52	14	8	33	1.82E-04	0.015179	16	15.9463386							
6	2E+07	6_236686	2	23	0	6	13	3	0.018372	0.65917	25	6	1	3	16	2	0.00719486	0.3031284	8.25	9.96780532							
6	2E+07	6_23812640	103	65	93	95	84	94	0.952083	1	10	32	45	41	21	43	0.00940319	0.3747181	60.5	33.9242854				2.4E+07	2.4E+07	0.4616	ID=promoter_42089;
6	2E+07	6_23893633	5	11	20	20	8	20	0.179808	1	3	10	0	4	7	2	0.29886173	1	9.125	7.60521438				2.4E+07	2.4E+07	0.16127	ID=promoter_42097;
6	2E+07	6_239032	85	30	46	28	58	37	0.013289	0.50938	1	26	20	23	14	22	0.29543416	1	32.375	24.6283083							
6	2E+07	6_23936214	70	29	45	13	50	29	0.008713	0.36691	15	4	21	29	10	25	0.022088	0.7420679	28.25	20.9540313	2E+07	2.4E+07	ID=ENSP00000378205;;	2.4E+07	2.4E+07	0.18468	ID=promoter_42101;
6	2E+07	6_23960033	1	17	5	0	9	3	0.131542	1	4	25	5	38	15	22	0.38478854	1	11.875	13.6113136	2E+07	2.4E+07	ID=ENSP00000378205;;				
6	2E+07	6_23971876	113	28	52	46	71	49	0.015386	0.57495	0	28	18	1	14	10	0.81923661	1	35.75	36.3661931	2E+07	2.4E+07	ID=ENSP00000378205;;				
6	2E+07	6_24011367	14	15	10	4	15	7	0.113635	1	7	9	7	3	8	5	0.78486764	1	8.625	4.30738568	2E+07	2.4E+07	ID=ENSP00000378205;;	2.4E+07	2.4E+07	0.58692	ID=promoter_42108;
6	2E+07	6_24102160	5	19	2	2	12	2	0.011798	0.46127	14	24	19	12	19	16	0.96066018	1	12.125	8.40811683				2.4E+07	2.4E+07	0.18228	ID=promoter_42120;
6	2E+07	6_241151	133	38	132	124	86	128	0.230916	1	38	34	32	17	36	25	0.53796732	1	68.5	51.1412609							
6	2E+07	6_242110	4435	3444	5358	6611	3940	5985	2.08E-35	#######	3286	2797	5536	1708	3042	3622	2.49E-23	2.01E-20	4146.9	1628.18185							
6	2E+07	6_249722	8	26	47	81	17	64	3.66E-05	0.00372	41	39	29	6	40	18	0.03384608	1	34.625	23.8982217							
6	3E+07	6_253050	36	22	10	9	29	10	0.001829	0.10386	1	9	25	0	5	13	0.12287893	1	14	12.5356634							
6	3E+07	6_256156	0	15	3	2	8	3	0.206144	1	17	9	28	0	13	14	0.92696813	1	9.25	10.0534287							
6	3E+07	6_263590	27	48	30	45	38	38	0.784842	1	40	32	20	16	36	18	0.09436184	1	32.25	11.4486431							
6	3E+07	6_266599	5	11	20	15	8	18	0.296056	1	13	17	28	2	15	15	0.97869956	1	13.875	8.25378701							
6	3E+07	6_266978	11	10	28	10	11	19	0.589612	1	2	6	24	22	4	23	5.91E-04	0.0404335	14.125	9.32642176							
6	3E+07	6_267694	0	12	8	0	6	4	0.674789	1	0	6	13	2	3	8	0.26303803	1	5.125	5.43631966							
6	3E+07	6_267986	39	56	44	63	48	54	0.978101	1	21	27	52	13	24	33	0.33498297	1	39.375	17.7356582							
6	3E+07	6_271700	26	0	4	3	13	4	0.041893	1	0	9	15	0	5	8	0.62517663	1	7.125	9.26494931							
6	3E+07	6_271737	32	3	32	17	18	25	0.86091	1	16	12	30	0	14	15	0.92682316	1	17.75	12.6801307							
6	3E+07	6_271886	145	25	112	138	85	125	0.272752	1	33	40	54	20	37	37	0.94827688	1	70.875	52.1767258							
6	3E+07	6_272010	73	37	15	21	55	18	3.26E-06	0.00044	21	8	7	23	15	15	0.97869956	1	25.625	21.3604274							
6	3E+07	6_272887	0	16	2	0	8	1	0.03583	1	2	13	9	2	8	6	0.92562801	1	5.5	6.27921742							
6	3E+07	6_27294663	3319	1823	3724	3330	2571	3527	1.15E-08	#######	2404	1345	1659	781	1875	1220	4.75E-21	3.37E-18	2298.1	1068.75234				2.7E+07	2.7E+07	0.54892	ID=promoter_42167;
6	3E+07	6_27308982	31	7	15	30	19	23	0.994841	1	30	5	10	32	18	21	0.77315138	1	20	11.8562822				2.7E+07	2.7E+07	0.20118	ID=promoter_42168;
6	3E+07	6_273943	19	70	72	61	45	67	0.463341	1	10	17	26	22	14	24	0.17833616	1	37.125	25.8812536							
6	3E+07	6_274111	9	36	0	0	23	0	1.39E-06	0.0002	30	18	7	16	24	12	0.20726737	1	14.5	13.223356							
6	3E+07	6_27664686	23	2	40	33	13	37	0.017709	0.64095	40	72	47	79	56	63	0.57293854	1	42	24.9112711				2.8E+07	2.8E+07	0.1367	ID=promoter_42193;
6	3E+07	6_280998	39	12	41	51	26	46	0.240995	1	14	12	15	18	13	17	0.64388579	1	25.25	15.7457477							

6	3E+07	6_28248326	8	20	8	3	14	6	0.098789	1	0	4	7	28	2	18	8.85E-04	0.0572814	9.75	9.45289071	3E+07	2.8E+07	ID=ENSP00000312277;ZNF507;NM_014910;	ZNF507				
6	3E+07	6_282849	24	61	29	5	43	17	3.55E-04	0.02675	49	21	40	8	35	24	0.52090333	1	29.625	19.4491094								
6	3E+07	6_282879	22	3	24	34	13	29	0.146708	1	64	17	24	9	41	17	0.01650079	0.5857858	24.625	18.5775402								
6	3E+07	6_28297360	143	148	114	156	146	135	0.13621	1	41	17	22	3	29	13	0.08411918	1	80.5	65.7983934					2.8E+07	2.8E+07	0.12925	ID=promoter_42229;
6	3E+07	6_28521417	426	96	261	182	261	222	0.001934	0.10953	128	64	90	40	96	65	0.14529553	1	160.88	128.21348					2.9E+07	2.9E+07	0.5317	ID=promoter_42240;
6	3E+07	6_28700049	10	3	4	11	7	8	0.998739	1	23	1	5	3	12	4	0.18067673	1	7.5	7.17137166	3E+07	2.9E+07	ID=ENSP00000253188;SLC7A10;NM_019849;	SLC7A10				
6	3E+07	6_28889528	774	256	640	536	515	588	0.905001	1	373	287	266	153	330	210	1.02E-04	0.0093839	410.63	216.553482					2.9E+07	2.9E+07	0.63166	ID=promoter_42259;
6	3E+07	6_29277629	4	7	5	0	6	3	0.462004	1	9	10	15	14	10	15	0.49251655	1	8	5.07092553					2.9E+07	2.9E+07	0.60425	ID=promoter_42273;
6	3E+07	6_29414546	1326	544	919	953	935	936	0.002782	0.14699	971	526	475	325	749	400	2.17E-18	1.37E-15	754.88	337.862794					2.9E+07	2.9E+07	0.32682	ID=promoter_42277;
6	3E+07	6_29563164	863	329	624	490	596	557	5.74E-04	0.03975	323	237	282	91	280	187	0.00228208	0.1227275	404.88	244.77362					3E+07	3E+07	0.32954	ID=promoter_42283;
6	3E+07	6_297928	354	133	307	185	244	246	0.245141	1	129	148	151	116	139	134	0.94912565	1	190.38	89.7137312								
6	3E+07	6_298577	35	21	12	21	28	17	0.092775	1	15	21	13	2	18	8	0.20901227	1	17.5	9.56182887								
6	3E+07	6_298751	28	42	33	28	35	31	0.520594	1	36	20	7	74	28	41	0.17053904	1	33.5	19.50824								
6	3E+07	6_29935240	275	79	204	184	177	194	0.808222	1	114	111	67	30	113	49	3.20E-05	0.0034562	133	81.5317642					3E+07	3E+07	0.26617	ID=promoter_42300;
6	3E+07	6_299385	10	33	33	41	22	37	0.404342	1	11	0	3	0	6	2	0.42506085	1	16.375	16.664226								
6	3E+07	6_300010	13	2	5	14	8	10	0.991043	1	26	41	10	21	34	16	0.07441541	1	16.5	12.6151836								
6	3E+07	6_300144	14	7	3	16	11	10	0.842816	1	4	5	20	27	5	24	9.36E-04	0.0596362	12	8.68496237								
6	3E+07	6_30228652	2279	1481	2388	2289	1880	2339	0.163644	1	1663	1065	636	579	1364	608	6.43E-52	1.19E-48	1547.5	737.829636	3E+07	3E+07	ID=ENSP00000262623;ATP4A;NM_000704;	ATP4A	3E+07	3E+07	0.26589	ID=promoter_42324;
6	3E+07	6_30324813	1785	833	2125	1707	1309	1916	4.84E-09	#######	1060	756	683	484	908	584	3.71E-11	1.31E-08	1179.1	607.42817					3E+07	3E+07	0.27157	ID=promoter_42328;
6	3E+07	6_304468	14	1	8	17	8	13	0.766846	1	2	11	10	3	7	7	0.99000271	1	8.25	5.84929788								
6	3E+07	6_30640520	61	1	65	62	31	64	0.032464	1	1	12	37	1	7	19	0.03889743	1	30	29.5344835	3E+07	3.1E+07	ID=ENSP00000368165-D2;;NM_001042631;		3.1E+07	3.1E+07	0.47797	ID=promoter_42352;
6	3E+07	6_30858921	149	64	93	58	107	76	0.003316	0.16983	21	37	6	35	29	21	0.68832436	1	57.875	45.645959					3.1E+07	3.1E+07	0.19639	ID=promoter_42359;
6	3E+07	6_311630	21	109	26	52	65	39	0.003397	0.17328	53	28	92	59	41	76	0.0012895	0.0769086	55	31.7129987								
6	3E+07	6_318439	30	45	13	19	38	16	0.001589	0.09301	32	18	18	9	25	14	0.3120942	1	23	11.8079876								
6	3E+07	6_321534	15	9	7	10	12	9	0.592799	1	4	7	0	7	6	4	0.87665919	1	7.375	4.37321392								
6	3E+07	6_324104	12	15	1	3	14	2	0.003923	0.19308	77	68	1	16	73	9	1.47E-10	4.79E-08	24.125	30.549433								
6	3E+07	6_32482711	188	71	230	168	130	199	0.060599	1	104	60	72	26	82	49	0.04952257	1	114.88	71.9274188	3E+07	3.2E+07	ID=ENSP00000386962;C19orf15;;	C19orf15	3.2E+07	3.2E+07	0.29464	ID=promoter_42412;
6	3E+07	6_32509339	1133	895	1699	1684	1014	1692	5.21E-18	#######	2000	1336	2734	989	1668	1862	2.44E-07	4.70E-05	1558.8	610.30806					3.3E+07	3.3E+07	0.68345	ID=promoter_42414;
6	3E+07	6_32589763	32	59	33	25	46	29	0.02902	0.94901	29	14	72	28	22	50	0.00140811	0.0833555	36.5	19.1460552					3.3E+07	3.3E+07	0.34159	ID=promoter_42425;
6	3E+07	6_32693925	45	51	47	48	48	48	0.736366	1	36	19	72	42	28	57	0.00221024	0.1194491	45	14.8516473	3E+07	3.3E+07	ID=ENSP00000248342;EIF3K;NM_013234;	EIF3K	3.3E+07	3.3E+07	0.1817	ID=promoter_42439;
6	3E+07	6_327207	8	13	8	29	11	19	0.589612	1	14	19	21	16	17	19	0.85444859	1	16	7.01019666								

6	3E+07	6_32733111	33	17	16	12	25	14	0.076795	1	49	16	20	0	33	10	0.00489842	0.2240264	20.375	14.7060483					3.3E+07	3.3E+07	0.14798	ID=promoter_42441;
6	3E+07	6_32864540	189	203	306	201	196	254	0.576815	1	11	77	50	16	44	33	0.65373342	1	131.63	107.684642	3E+07	3.3E+07	ID=ENSP00000292853;FBXO27;NM_178820;	FBXO27				
6	3E+07	6_32958419	114	35	155	78	75	117	0.153995	1	14	17	72	25	16	49	6.21E-05	0.0060773	63.75	50.8660706					3.3E+07	3.3E+07	0.34417	ID=promoter_42456;
6	3E+07	6_329849	123	96	114	155	110	135	0.939773	1	101	25	58	9	63	34	0.03646514	1	85.125	50.2378272								
6	3E+07	6_33015015	268	55	167	117	162	142	0.039431	1	79	34	51	39	57	45	0.72242782	1	101.25	81.0057317					3.3E+07	3.3E+07	0.46814	ID=promoter_42462;
6	3E+07	6_330272	13	21	11	10	17	11	0.29617	1	7	4	17	2	6	10	0.51486293	1	10.625	6.39056224								
6	3E+07	6_330922	31	63	21	38	47	30	0.030325	0.98285	56	22	34	25	39	30	0.74371451	1	36.25	15.5999084								
6	3E+07	6_332153	4	1	7	2	3	5	0.88968	1	0	17	18	1	9	10	0.92505409	1	6.25	7.28501397								
6	3E+07	6_332173	9	7	0	6	8	3	0.206144	1	12	21	22	6	17	14	0.9475482	1	10.375	7.65203054								
6	3E+07	6_33256862	104	111	45	46	108	46	1.50E-08	#######	91	152	64	25	122	45	3.13E-07	5.91E-05	79.75	42.2636284					3.3E+07	3.3E+07	0.51061	ID=promoter_42470;
6	3E+07	6_332864	16	15	13	20	16	17	0.960459	1	0	6	14	1	3	8	0.26303803	1	10.625	7.36667205								
6	3E+07	6_333724	1	5	20	8	3	14	0.063635	1	14	2	2	0	8	1	0.0848225	1	6.5	7.13141941								
6	3E+07	6_334368	108	43	101	65	76	83	0.903734	1	11	26	29	13	19	21	0.85675254	1	49.5	38.067609								
6	3E+07	6_335360	41	43	48	38	42	43	0.821371	1	59	28	67	20	44	44	0.93879668	1	43	15.3063946								
6	3E+07	6_33708663	2333	1670	2579	2251	2002	2415	0.650544	1	1714	1390	1267	659	1552	963	8.28E-22	6.16E-19	1732.9	636.581369					3.4E+07	3.4E+07	0.45625	ID=promoter_42500;
6	3E+07	6_338180	2	42	12	17	22	15	0.260529	1	43	8	15	5	26	10	0.05079892	1	18	15.9104638								
6	3E+07	6_33864110	29	78	28	69	54	49	0.426995	1	62	40	60	21	51	41	0.79361989	1	48.375	21.4937699	3E+07	3.4E+07	ID=ENSP0000332679-D20;CYP2A13;NM_000766;	CYP2A13				
6	3E+07	6_338885	1425	1030	1602	1416	1228	1509	0.480371	1	952	597	835	322	775	579	4.32E-04	0.0314643	1022.4	441.52364								
6	3E+07	6_339048	618	191	230	146	405	188	1.89E-26	#######	48	38	25	1	43	13	9.59E-04	0.0604009	162.13	202.385373								
6	3E+07	6_34053355	543	136	413	290	340	352	0.259384	1	94	98	169	52	96	111	0.28322278	1	224.38	175.241008					3.4E+07	3.4E+07	0.55274	ID=promoter_42514;
6	3E+07	6_34089146	22	13	26	22	18	24	0.918896	1	3	11	2	0	7	1	0.13113995	1	12.375	10.155048					3.4E+07	3.4E+07	0.14214	ID=promoter_42519;
6	3E+07	6_34100205	66	40	60	37	53	49	0.484571	1	14	35	43	17	25	30	0.63340892	1	39	18.1894789					3.4E+07	3.4E+07	0.21728	ID=promoter_42521;
6	3E+07	6_347810	4	11	22	24	8	23	0.078123	1	1	14	7	0	8	4	0.59180275	1	10.375	9.11729126								
6	3E+07	6_348652	31	33	61	52	32	57	0.16287	1	32	18	47	34	25	41	0.07455929	1	38.5	13.7840488								
6	3E+07	6_349505	7	11	18	4	9	11	0.995818	1	0	0	4	1	0	3	0.19828566	1	5.625	6.254998								
6	4E+07	6_350024	52	6	24	24	29	24	0.446278	1	40	17	1	0	29	1	5.87E-06	0.000796	20.5	18.5933936								
6	4E+07	6_35150834	298	130	280	315	214	298	0.159105	1	117	78	112	12	98	62	0.06046094	1	167.75	113.810306					3.5E+07	3.5E+07	0.19287	ID=promoter_42593;
6	4E+07	6_35169717	10	14	32	11	12	22	0.455056	1	33	20	24	2	27	13	0.14207	1	18.25	10.9902554					3.5E+07	3.5E+07	0.44369	ID=promoter_42596;
6	4E+07	6_353234	111	43	68	130	77	99	0.833112	1	28	57	23	1	43	12	4.94E-04	0.0351794	57.625	44.2264869								
6	4E+07	6_35461267	69	28	76	40	49	58	0.998901	1	36	0	26	12	18	19	0.92496372	1	35.875	26.0243704					3.5E+07	3.5E+07	0.10372	ID=promoter_42611;
6	4E+07	6_354857	228	125	213	175	177	194	0.808222	1	78	121	92	51	100	72	0.26074675	1	135.38	64.1225111								
6	4E+07	6_35487611	15	21	33	20	18	27	0.720164	1	28	23	49	38	26	44	0.04734528	1	28.375	11.1603571					3.5E+07	3.5E+07	0.51207	ID=promoter_42612;
6	4E+07	6_355131	19	34	25	42	27	34	0.962765	1	19	29	22	14	24	18	0.79306625	1	25.5	9.14955112								
6	4E+07	6_35569025	280	108	150	102	194	126	1.08E-06	0.00016	59	64	127	24	62	76	0.26158563	1	114.25	78.2171885					3.6E+07	3.6E+07	0.13679	ID=promoter_42617;
6	4E+07	6_357705	318	148	408	257	233	333	0.068263	1	282	124	69	65	203	67	1.81E-13	8.28E-11	208.88	125.582571								
6	4E+07	6_359388	15	19	14	12	17	13	0.50439	1	46	0	18	16	23	17	0.77734386	1	17.5	12.9394193								
6	4E+07	6_35948079	887	483	1040	1357	685	1199	4.24E-16	#######	559	318	492	369	439	431	0.69913376	1	688.13	367.774946					3.6E+07	3.6E+07	0.4855	ID=promoter_42653;
6	4E+07	6_35995161	812	431	713	705	622	709	0.873183	1	498	289	341	78	394	210	4.49E-10	1.38E-07	483.38	249.335653					3.6E+07	3.6E+07	0.49131	ID=promoter_42660;

6	4E+07	6_36031290	9	14	2	0	12	1	0.003675	0.18279	10	5	0	2	8	1	0.0848225	1	5.25	5.2030211	4E+07	3.6E+07	ID=ENSP00000245934;SYMPK;NM_004819;	SYMPK				
6	4E+07	6_36057730	55	59	55	77	57	66	0.99745	1	1	26	35	21	14	28	0.05515466	1	41.125	24.7064913	4E+07	3.6E+07	ID=ENSP00000263257;NOVA2;NM_002516;	NOVA2				
6	4E+07	6_36194604	47	9	42	47	28	45	0.42187	1	177	96	132	89	137	111	0.57287076	1	79.875	54.9192589	4E+07	3.6E+07	ID=ENSP00000012443;PPP5C;NM_006247;	PPP5C				
6	4E+07	6_362691	2	10	2	5	6	4	0.674789	1	13	8	15	1	11	8	0.87190251	1	7	5.34522484								
6	4E+07	6_363030	0	22	5	0	11	3	0.050503	1	16	10	17	6	13	12	0.99994263	1	9.5	8.17662173								
6	4E+07	6_363038	65	9	42	23	37	33	0.517593	1	0	7	0	0	4	0	0.15659576	1	18.25	23.8552181								
6	4E+07	6_36352078	91	51	86	97	71	92	0.81966	1	0	1	33	28	1	31	2.40E-07	4.64E-05	48.375	39.3516654	4E+07	3.6E+07	ID=ENSP00000375777;STRN4;NM_001039877;	STRN4				
6	4E+07	6_36405784	299	58	251	175	179	213	0.989993	1	119	75	104	19	97	62	0.07101452	1	137.5	97.1773048					3.6E+07	3.6E+07	0.51338	ID=promoter_42690;
6	4E+07	6_366106	716	203	622	424	460	523	0.887893	1	287	266	174	85	277	130	5.60E-10	1.70E-07	347.13	222.705724								
6	4E+07	6_366123	11	23	19	12	17	16	0.818283	1	17	8	19	35	13	27	0.0491434	1	18	8.46842876								
6	4E+07	6_366866	10	7	10	11	9	11	0.995818	1	21	8	7	40	15	24	0.24434768	1	14.25	11.335784								
6	4E+07	6_368738	13	9	23	10	11	17	0.774447	1	20	9	11	8	15	10	0.7195765	1	12.875	5.59176691								
6	4E+07	6_36893752	2	0	12	6	1	9	0.073427	1	0	10	4	21	5	13	0.12287893	1	6.875	7.1999504	4E+07	3.7E+07	ID=ENSP00000331907;DHX34;NM_014681;	DHX34	3.7E+07	3.7E+07	0.22393	ID=promoter_42727;
6	4E+07	6_36961015	324	134	267	225	229	246	0.63215	1	111	82	90	39	97	65	0.1264303	1	159	100.898818	4E+07	3.7E+07	ID=ENSP00000375772;SLC8A2;;	SLC8A2	3.7E+07	3.7E+07	0.30822	ID=promoter_42741;
6	4E+07	6_37015370	1015	241	744	443	628	594	8.29E-04	0.05425	197	176	272	71	187	172	0.99723653	1	394.88	325.025466					3.7E+07	3.7E+07	0.30734	ID=promoter_42749;
6	4E+07	6_37053060	2353	1286	2964	2006	1820	2485	5.31E-06	0.00068	1867	1331	1174	765	1599	970	1.16E-24	1.00E-21	1718.3	717.278936					3.7E+07	3.7E+07	0.68802	ID=promoter_42753;
6	4E+07	6_370677	40	15	24	10	28	17	0.092775	1	0	18	2	2	9	2	0.1390433	1	13.875	13.5903064								
6	4E+07	6_371788	20	9	30	6	15	18	0.997876	1	5	8	5	5	7	5	0.9051169	1	11	9.16515139								
6	4E+07	6_372821	88	73	83	155	81	119	0.293253	1	93	163	213	76	128	145	0.25397457	1	118	52.0411925								
6	4E+07	6_37328359	351	181	33	13	266	23	3.13E-54	#######	124	43	39	24	84	32	5.64E-05	0.0056196	101	116.456	4E+07	3.7E+07	ID=ENSP00000263274;LIG1;NM_000234;	LIG1				
6	4E+07	6_374111	124	106	158	159	115	159	0.404799	1	99	33	12	0	66	6	1.24E-10	4.10E-08	86.375	63.4776395								
6	4E+07	6_374152	15	8	5	1	12	3	0.030625	0.98374	8	0	17	0	4	9	0.31291743	1	6.75	6.5846141								
6	4E+07	6_37442167	26	6	25	0	16	13	0.613293	1	31	4	12	2	18	7	0.13207736	1	13.25	12.2911118	4E+07	3.7E+07	ID=ENSP00000263269;GRIN2D;NM_000836;	GRIN2D				
6	4E+07	6_37476001	2393	3139	2275	2712	2766	2494	1.52E-20	#######	7463	6093	11500	2960	6778	7230	1.05E-15	5.57E-13	4816.9	3302.04869					3.7E+07	3.7E+07	0.56665	ID=promoter_42789;
6	4E+07	6_37507124	83	21	17	0	52	9	6.25E-09	#######	6	28	35	37	17	36	0.01546861	0.5546463	28.375	25.6121483					3.8E+07	3.8E+07	0.29059	ID=promoter_42796;
6	4E+07	6_37520734	4	9	4	6	7	5	0.693374	1	11	3	4	9	7	7	0.99000271	1	6.25	3.01188123					3.8E+07	3.8E+07	0.20925	ID=promoter_42799;
6	4E+07	6_37733104	97	72	102	81	85	92	0.866901	1	59	107	29	3	83	16	1.63E-09	4.59E-07	68.75	36.8888291					3.8E+07	3.8E+07	0.20447	ID=promoter_42821;
6	4E+07	6_37763228	39	13	4	2	26	3	1.49E-05	0.00168	52	22	3	0	37	2	5.42E-07	9.60E-05	16.875	19.3792046	4E+07	3.8E+07	ID=ENSP00000221413;RUVBL2;NM_006666;	RUVBL2	3.8E+07	3.8E+07	0.35507	ID=promoter_42828;

6	4E+07	6_378100	21	8	31	6	15	19	0.975531	1	30	4	1	0	17	1	0.00146248	0.085933	12.625	12.78322								
6	4E+07	6_378738	11	22	12	3	17	8	0.087954	1	21	3	10	17	12	14	0.84238319	1	12.375	7.28868987								
6	4E+07	6_37898915	154	118	165	170	136	168	0.90412	1	74	88	66	42	81	54	0.16912805	1	109.63	49.2281496					3.8E+07	3.8E+07	0.55666	ID=promoter_42849;
6	4E+07	6_38038890	217	79	200	166	148	183	0.892382	1	57	126	55	0	92	28	3.92E-07	7.23E-05	112.5	77.2620772					3.8E+07	3.8E+07	0.32916	ID=promoter_42870;
6	4E+07	6_38092202	1	16	4	6	9	5	0.390586	1	7	3	5	5	5	5	0.99284883	1	5.875	4.48608961					3.8E+07	3.8E+07	0.42021	ID=promoter_42874;
6	4E+07	6_38207739	26	30	62	34	28	48	0.275656	1	39	31	31	22	35	27	0.77254999	1	34.375	12.2467197	4E+07	3.8E+07	ID=ENSP0000353769;SCAF1;NM_021228;	SCAF1	3.8E+07	3.8E+07	0.53815	ID=promoter_42891;
6	4E+07	6_38230826	5	0	4	12	3	8	0.459247	1	38	3	10	0	21	5	0.01300804	0.4860836	9	12.478553	4E+07	3.8E+07	ID=ENSP0000353769;SCAF1;NM_021228;	SCAF1				
6	4E+07	6_38317724	104	30	34	35	67	35	3.56E-04	0.02681	64	18	13	9	41	11	5.18E-04	0.0365242	38.375	31.5908554					3.8E+07	3.8E+07	0.52891	ID=promoter_42907;
6	4E+07	6_38318697	27	32	55	35	30	45	0.57861	1	30	22	35	22	26	29	0.79045324	1	32.25	10.5254793					3.8E+07	3.8E+07	0.52891	ID=promoter_42907;
6	4E+07	6_38342605	2	11	5	9	7	7	0.956352	1	10	8	12	3	9	8	0.99626583	1	7.5	3.74165739	4E+07	3.8E+07	ID=ENSP0000375726-D2;NOSIP;NM_015953;	NOSIP	3.8E+07	3.8E+07	0.42042	ID=promoter_42911;
6	4E+07	6_38416708	1	0	2	0	1	1	0.993645	1	54	1	6	8	28	7	0.00393447	0.1875084	9	18.4158317	4E+07	3.8E+07	ID=ENSP0000384465;CPT1C;NM_001136052;	CPT1C				
6	4E+07	6_38438589	1	0	1	12	1	7	0.163779	1	15	6	5	1	11	3	0.13591373	1	5.125	5.6426311	4E+07	3.8E+07	ID=ENSP0000246801;TSKS;NM_021733;	TSKS				
6	4E+07	6_38513890	7	7	7	15	7	11	0.831726	1	1	9	8	2	5	5	0.99284883	1	7	4.30945804					3.9E+07	3.9E+07	0.22197	ID=promoter_42940;
6	4E+07	6_386087	36	2	14	15	19	15	0.513973	1	23	0	5	18	12	12	0.98292303	1	14.125	11.9694552								
6	4E+07	6_387929	24	24	18	12	24	15	0.151024	1	23	4	9	0	14	5	0.16413709	1	14.25	9.42261717								
6	4E+07	6_38808085	407	198	543	473	303	508	4.84E-06	0.00063	180	187	142	186	184	164	0.93517402	1	289.5	158.149116					3.9E+07	3.9E+07	0.27038	ID=promoter_42968;
6	4E+07	6_388277	5	1	2	8	3	5	0.88968	1	8	1	11	5	5	8	0.62517663	1	5.125	3.68151754								
6	4E+07	6_38836487	13	19	13	16	16	15	0.822707	1	27	10	32	6	19	19	0.97309647	1	17	8.71779789					3.9E+07	3.9E+07	0.33143	ID=promoter_42972;
6	4E+07	6_38949133	538	176	368	283	357	326	0.004662	0.22164	213	90	101	44	152	73	1.58E-05	0.0018878	226.63	165.040201					3.9E+07	3.9E+07	0.34164	ID=promoter_42983;
6	4E+07	6_38966789	221	95	202	207	158	205	0.634764	1	128	63	33	67	96	50	0.00363285	0.1775497	127	74.0829651					3.9E+07	3.9E+07	0.2966	ID=promoter_42986;
6	4E+07	6_39038394	2	5	10	10	4	10	0.424038	1	1	1	1	11	1	6	0.13767373	1	5.125	4.51782185					3.9E+07	3.9E+07	0.277	ID=promoter_42998;
6	4E+07	6_390721	26	15	3	5	21	4	7.04E-04	0.04721	9	11	11	2	10	7	0.84912739	1	10.25	7.75978645								
6	4E+07	6_391966	17	17	17	18	17	18	0.955187	1	14	14	14	0	14	7	0.39930956	1	13.875	5.84166072								
6	4E+07	6_39223173	2	12	10	1	7	6	0.851931	1	16	5	23	19	11	21	0.13989775	1	11	8.03563492					3.9E+07	3.9E+07	0.16798	ID=promoter_43021;
6	4E+07	6_39300855	17	71	6	10	44	8	1.70E-07	#######	27	16	38	17	22	28	0.53586024	1	25.25	20.9880919	4E+07	3.9E+07	ID=ENSP0000345243-D3;SIGLEC12;NM_033130;	SIGLEC12	3.9E+07	3.9E+07	0.32499	ID=promoter_43026;

6	4E+07	6_39367688	14	32	26	34	23	30	0.929178	1	3	5	14	2	4	8	0.43697044	1	16.25	12.947697	4E+07	3.9E+07	ID=ENSP0000387087;;		3.9E+07	3.9E+07	0.1928	ID=promoter_43037;
6	4E+07	6_39368462	63	40	50	48	52	49	0.544924	1	36	60	43	28	48	36	0.62895407	1	46	11.7958831					3.9E+07	3.9E+07	0.1928	ID=promoter_43037;
6	4E+07	6_393699	5	42	16	28	24	22	0.704268	1	6	18	31	0	12	16	0.63262749	1	18.25	14.5675765								
6	4E+07	6_39552881	726	226	568	496	476	532	0.745955	1	318	154	195	89	236	142	2.22E-04	0.0180511	346.5	225.857098					4E+07	4E+07	0.40186	ID=promoter_43056;
6	4E+07	6_400580	3	7	2	18	5	10	0.615109	1	21	7	19	1	14	10	0.81923661	1	9.75	8.25919401								
6	4E+07	6_40192953	1193	812	790	916	1003	853	3.66E-11	#######	684	607	456	221	646	339	8.72E-17	4.90E-14	709.88	294.582241					4E+07	4E+07	0.09976	ID=promoter_43082;
6	4E+07	6_40351707	55	21	26	61	38	44	0.998299	1	23	4	11	23	14	17	0.75475782	1	28	19.9499373					4E+07	4E+07	0.63835	ID=promoter_43104;
6	4E+07	6_403575	78	53	82	85	66	84	0.884061	1	6	29	14	3	18	9	0.30718372	1	43.75	35.0744107								
6	4E+07	6_405016	6	14	6	14	10	10	0.938234	1	25	0	32	3	13	18	0.54030568	1	12.5	11.1611571								
6	4E+07	6_40538524	2	26	1	8	14	5	0.053576	1	0	0	1	17	0	9	0.00779604	0.3222758	6.875	9.68706796	4E+07	4.1E+07	ID=ENSP0000375639;TFPT;NM_013342;	TFPT				
6	4E+07	6_405583	32	54	8	23	43	16	1.90E-04	0.01552	14	16	46	16	15	31	0.03250921	0.9922526	26.125	16.4615353								
6	4E+07	6_405601	22	11	1	7	17	4	0.005755	0.26328	2	11	22	0	7	11	0.54353855	1	9.5	8.79935063								
6	4E+07	6_40564782	1123	405	972	517	764	745	0.001576	0.09256	494	209	326	102	352	214	6.01E-06	0.0008111	518.5	356.748411					4.1E+07	4.1E+07	0.65123	ID=promoter_43119;
6	4E+07	6_405662	689	617	683	905	653	794	0.792971	1	218	266	407	376	242	392	4.12E-11	1.44E-08	520.13	239.639275								
6	4E+07	6_40666246	447	288	430	323	368	377	0.181101	1	378	246	296	78	312	187	1.24E-05	0.0015211	310.75	117.447557	4E+07	4.1E+07	ID=ENSP0000365714;TTYH1;NM_001005367;	TTYH1				
6	4E+07	6_411414	46	13	34	88	30	61	0.044121	1	7	14	14	3	11	9	0.96206785	1	27.375	28.3847922								
6	4E+07	6_41212364	93	30	51	54	62	53	0.236471	1	44	33	31	2	39	17	0.02919595	0.915188	42.25	26.1192321					4.1E+07	4.1E+07	0.19537	ID=promoter_43188;
6	4E+07	6_412136	25	5	13	3	15	8	0.181584	1	1	3	13	1	2	7	0.20455692	1	8	8.41766849								
6	4E+07	6_41252257	33	73	36	57	53	47	0.37511	1	25	34	20	8	30	14	0.09649838	1	35.75	20.6449579	4E+07	4.1E+07	ID=ENSP0000320853;BRSK1;;	BRSK1				
6	4E+07	6_41257803	13	18	16	12	16	14	0.724026	1	13	2	2	0	8	1	0.0848225	1	9.5	7.05083582	4E+07	4.1E+07	ID=ENSP0000320853;BRSK1;;	BRSK1	4.1E+07	4.1E+07	0.18016	ID=promoter_43192;
6	4E+07	6_413588	1	28	3	0	15	2	0.002248	0.12273	1	9	0	0	5	0	0.09850879	1	5.25	9.67692395								
6	4E+07	6_413970	7	21	2	1	14	2	0.003923	0.19308	21	11	10	1	16	6	0.14768941	1	9.25	8.22452778								
6	4E+07	6_41403606	13	0	9	16	7	13	0.61331	1	19	41	54	42	30	48	0.05970579	1	24.25	18.9868375					4.1E+07	4.1E+07	0.27865	ID=promoter_43210;
6	4E+07	6_41550776	82	82	33	48	82	41	2.61E-05	0.00275	78	65	15	2	72	9	2.27E-10	7.25E-08	50.625	31.3411528					4.2E+07	4.2E+07	0.16421	ID=promoter_43222;
6	4E+07	6_415653	82	29	83	39	56	61	0.923132	1	31	25	20	16	28	18	0.47597237	1	40.625	26.763181								
6	4E+07	6_41591237	5	11	21	24	8	23	0.078123	1	42	24	7	0	33	4	3.34E-05	0.003583	16.75	13.666957	4E+07	4.2E+07	ID=ENSP0000299481;NLRP14;NM_176822;	NLRP14				
6	4E+07	6_41605999	11	35	5	14	23	10	0.024151	0.81985	39	8	12	1	24	7	0.01749745	0.6144624	15.625	13.8557621	4E+07	4.2E+07	ID=ENSP0000299481;NLRP14;NM_176822;	NLRP14				
6	4E+07	6_416889	35	36	30	40	36	35	0.730868	1	0	58	1	4	29	3	6.67E-05	0.0064567	25.5	21.3808994								
6	4E+07	6_41861351	31	52	50	76	42	63	0.464879	1	51	37	16	37	44	27	0.23585004	1	43.75	17.8225379					4.2E+07	4.2E+07	0.12759	ID=promoter_43233;
6	4E+07	6_418916	20	41	8	6	31	7	6.31E-05	0.00596	3	8	19	25	6	22	0.00549069	0.2478586	16.25	12.6913919								
6	4E+07	6_420484	93	91	72	52	92	62	0.002924	0.15239	40	49	29	50	45	40	0.98146806	1	59.5	23.4155016								
6	4E+07	6_421191	5	16	17	6	11	12	0.985018	1	7	1	14	8	4	11	0.14780855	1	9.25	5.75077635								
6	4E+07	6_421454	11	9	18	6	10	12	0.998584	1	88	18	14	11	53	13	2.32E-05	0.0026249	21.875	27.0419647								
6	4E+07	6_421600	2	13	2	0	8	1	0.03583	1	9	0	16	3	5	10	0.35527535	1	5.625	6.20915223								
6	4E+07	6_422528	19	6	33	55	13	44	0.002066	0.11504	19	9	5	0	14	3	0.04246461	1	18.25	18.179659								

6	4E+07	6_42261305	48	27	47	40	38	44	0.998299	1	25	31	8	11	28	10	0.02694098	0.8576836	29.625	15.080143					4.2E+07	4.2E+07	0.70867	ID=promoter_43256;
6	4E+07	6_42261791	56	31	51	50	44	51	0.998332	1	68	31	11	11	50	11	1.59E-05	0.001895	38.625	21.0097766					4.2E+07	4.2E+07	0.70867	ID=promoter_43256;
6	4E+07	6_422688	1	14	7	10	8	9	0.996316	1	3	7	12	13	5	13	0.12287893	1	8.375	4.71888304								
6	4E+07	6_425503	0	15	2	8	8	5	0.532535	1	10	28	24	40	19	32	0.1120603	1	15.875	13.8196289								
6	4E+07	6_426872	3	10	5	7	7	6	0.851931	1	1	8	15	0	5	8	0.62517663	1	6.125	4.96955014								
6	4E+07	6_427501	8	24	1	2	16	2	0.001284	0.07728	12	7	8	1	10	5	0.51906437	1	7.875	7.62397534								
6	4E+07	6_427653	148	91	126	113	120	120	0.465301	1	34	10	9	12	22	11	0.23631249	1	67.875	57.8852004								
6	4E+07	6_42798855	9	10	5	1	10	3	0.082185	1	15	8	5	3	12	4	0.18067673	1	7	4.44007722					4.3E+07	4.3E+07	0.16197	ID=promoter_43318;
6	4E+07	6_427991	28	0	28	46	14	37	0.029025	0.94901	3	1	1	0	2	1	0.87709017	1	13.375	17.9836235								
6	4E+07	6_428095	22	8	18	11	15	15	0.908797	1	28	1	19	0	15	10	0.7195765	1	13.375	10.0560927								
6	4E+07	6_42839988	306	128	308	240	217	274	0.719589	1	89	88	85	3	89	44	0.0024466	0.1292868	155.88	114.021849					4.3E+07	4.3E+07	0.69023	ID=promoter_43325;
6	4E+07	6_428443	14	5	32	37	10	35	0.005919	0.26931	0	0	0	0	0	0	1	1	11	15.3343685								
6	4E+07	6_428803	121	73	128	91	97	110	0.971057	1	57	40	59	49	49	54	0.68043536	1	77.25	33.0054108								
6	4E+07	6_42882282	11	6	23	20	9	22	0.165185	1	3	24	42	35	14	39	0.00101734	0.0634872	20.5	13.617216					4.3E+07	4.3E+07	0.17551	ID=promoter_43330;
6	4E+07	6_42897797	417	169	383	353	293	368	0.682788	1	100	133	130	3	117	67	0.00647628	0.279113	211	152.211319					4.3E+07	4.3E+07	0.46401	ID=promoter_43331;
6	4E+07	6_42972241	24	14	6	4	19	5	0.004955	0.23359	26	0	1	6	13	4	0.12786863	1	10.125	10.1198179					4.3E+07	4.3E+07	0.42129	ID=promoter_43352;
6	4E+07	6_43001295	8	16	4	16	12	10	0.724827	1	18	5	8	1	12	5	0.30420563	1	9.5	6.36957052	4E+07	4.3E+07	ID=ENSP000002913 86;SSU72 ;NM_0141 88;	SSU72				
6	4E+07	6_43056317	58	13	67	40	36	54	0.518636	1	14	16	16	7	15	12	0.93002578	1	28.875	23.0182381					4.3E+07	4.3E+07	0.58687	ID=promoter_43367;
6	4E+07	6_43056489	2417	1263	2302	2131	1840	2217	0.695697	1	1316	943	1087	638	1130	863	9.92E-05	0.0091558	1512.1	675.591262					4.3E+07	4.3E+07	0.58687	ID=promoter_43367;
6	4E+07	6_430678	132	27	149	124	80	137	0.025736	0.86401	18	0	6	18	9	12	0.70935077	1	59.25	63.6165971								
6	4E+07	6_430927	257	92	286	135	175	211	0.964354	1	19	29	35	13	24	24	0.96613769	1	108.25	109.143091								
6	4E+07	6_431149	1993	1374	2500	1754	1684	2127	0.076107	1	312	457	243	173	385	208	1.74E-09	4.84E-07	1100.8	917.170143								
6	4E+07	6_431359	196	158	181	214	177	198	0.900287	1	132	118	142	56	125	99	0.5035332	1	149.63	49.976959								
6	4E+07	6_431575	8	11	2	7	10	5	0.276106	1	3	4	19	18	4	19	0.00415462	0.1961166	9	6.54653671								
6	4E+07	6_431723	16	7	3	17	12	10	0.724827	1	44	13	20	1	29	11	0.03330399	1	15.125	13.5165243								
6	4E+07	6_431897	214	109	247	218	162	233	0.136194	1	76	33	37	81	55	59	0.73804812	1	126.88	86.3769769								
6	4E+07	6_431937	13	28	18	5	21	12	0.1273	1	5	3	23	3	4	13	0.06462238	1	12.25	9.8088881								
6	4E+07	6_43219090	83	18	59	41	51	50	0.665611	1	34	20	44	0	27	22	0.90387209	1	37.375	25.8895318					4.3E+07	4.3E+07	0.16477	ID=promoter_43390;
6	4E+07	6_43328640	44	66	86	102	55	94	0.08311	1	45	23	31	12	34	22	0.41778496	1	51.125	31.2612837					4.3E+07	4.3E+07	0.17981	ID=promoter_43412;
6	4E+07	6_433364	4783	4053	4839	6197	4418	5518	0.007905	0.33894	2791	1754	4272	1912	2273	3092	4.51E-43	7.32E-40	3825.1	1551.12415								
6	4E+07	6_43340540	17	18	3	20	18	12	0.307258	1	0	22	4	5	11	5	0.40227598	1	11.125	8.91928088					4.3E+07	4.3E+07	0.47137	ID=promoter_43414;
6	4E+07	6_433573	51	74	8	14	63	11	1.27E-10	#######	45	32	18	17	39	18	0.04421467	1	32.375	22.7215285								
6	4E+07	6_433784	38	11	15	51	25	33	0.905451	1	8	31	35	21	20	28	0.36590545	1	26.25	14.9737866								
6	4E+07	6_43414534	91	27	61	41	59	51	0.276863	1	6	14	2	1	10	2	0.09294023	1	30.375	32.2221641					4.3E+07	4.3E+07	0.31581	ID=promoter_43433;
6	4E+07	6_434722	1	9	8	7	5	8	0.86114	1	52	10	5	1	31	3	2.73E-05	0.0030031	11.625	16.664226								
6	4E+07	6_434771	74	23	34	103	49	69	0.618874	1	50	15	32	4	33	18	0.18694358	1	41.875	32.7476826								
6	4E+07	6_435066	7	1	6	9	4	8	0.677894	1	18	23	32	34	21	33	0.16057985	1	16.25	12.4642345								
6	4E+07	6_435108	91	27	61	105	59	83	0.564772	1	124	86	93	28	105	61	0.01369618	0.5055791	76.875	35.219465								
6	4E+07	6_43530897	5	44	12	22	25	17	0.214068	1	67	29	42	10	48	26	0.08258093	1	28.875	21.0878265					4.4E+07	4.4E+07	0.33776	ID=promoter_43465;
6	4E+07	6_43545763	24	6	4	13	15	9	0.269309	1	0	7	21	12	4	17	0.01069978	0.4139111	10.875	8.32273135					4.4E+07	4.4E+07	0.39033	ID=promoter_43471;
6	4E+07	6_43559887	10	5	18	3	8	11	0.943069	1	0	0	4	0	0	2	0.34003807	1	5	6.25642527					4.4E+07	4.4E+07	0.56119	ID=promoter_43475;

6	4E+07	6_43578072	15	34	9	32	25	21	0.526204	1	28	8	39	28	18	34	0.04464527	1	24.125	11.8495027					4.4E+07	4.4E+07	0.451	ID=promoter_43480;
6	4E+07	6_435848	10	2	10	16	6	13	0.45043	1	1	4	22	1	3	12	0.04708211	1	8.25	7.72287881								
6	4E+07	6_43598728	271	118	159	224	195	192	0.226348	1	173	69	162	73	121	118	0.92622144	1	156.13	69.7801394					4.4E+07	4.4E+07	0.41614	ID=promoter_43488;
6	4E+07	6_43601483	11	21	9	6	16	8	0.127562	1	3	11	15	4	7	10	0.67636194	1	10	5.97614305					4.4E+07	4.4E+07	0.1757	ID=promoter_43489;
6	4E+07	6_436461	1	33	8	18	17	13	0.50439	1	9	0	17	9	5	13	0.12287893	1	11.875	10.6962944								
6	4E+07	6_436685	1	6	14	12	4	13	0.177139	1	2	7	22	6	5	14	0.08295177	1	8.75	6.94365075								
6	4E+07	6_437051	35	74	17	21	55	19	6.58E-06	0.00082	49	26	38	22	38	30	0.80512437	1	35.25	18.8812076								
6	4E+07	6_437292	395	149	634	743	272	689	4.30E-27	#######	202	166	410	122	184	266	1.82E-05	0.0021335	352.63	235.681345								
6	4E+07	6_437319	133	65	132	88	99	110	0.926397	1	54	33	53	3	44	28	0.29364396	1	70.125	45.6146515								
6	4E+07	6_437412	242	74	254	182	158	218	0.298113	1	130	103	41	48	117	45	1.50E-06	0.000239	134.25	83.657038								
6	4E+07	6_437510	31	13	19	30	22	25	0.99414	1	24	1	12	3	13	8	0.65599869	1	16.625	11.4009711								
6	4E+07	6_437981	31	63	45	53	47	49	0.846376	1	36	70	52	3	53	28	0.05110576	1	44.125	21.0335616								
6	4E+07	6_439650	9	2	5	7	6	6	0.962469	1	0	9	10	2	5	6	0.91271846	1	5.5	3.81725406								
6	4E+07	6_440546	36	1	22	12	19	17	0.718296	1	11	19	16	0	15	8	0.44137572	1	14.625	11.6611381								
6	4E+07	6_440567	64	25	35	22	45	29	0.038455	1	5	22	11	30	14	21	0.37101303	1	26.75	17.8705664								
6	4E+07	6_441061	24	7	32	2	16	17	0.960459	1	48	15	9	18	32	14	0.05628287	1	19.375	15.0422025								
6	4E+07	6_442167	27	19	21	8	23	15	0.200003	1	28	29	36	28	29	32	0.79342549	1	24.5	8.43462253								
6	4E+07	6_442562	18	32	48	15	25	32	0.94785	1	8	15	26	21	12	24	0.08343652	1	22.875	12.5178444								
6	4E+07	6_442657	5	14	8	18	10	13	0.970528	1	18	3	17	21	11	19	0.24832023	1	13	6.76123404								
6	4E+07	6_443151	300	89	274	245	195	260	0.399991	1	108	101	79	64	105	72	0.14134206	1	157.5	97.6685357								
6	4E+07	6_445062	4	0	10	1	2	6	0.490921	1	0	7	22	3	4	13	0.06462238	1	5.875	7.39570339								
6	4E+07	6_447251	52	16	60	87	34	74	0.010104	0.41206	0	18	37	3	9	20	0.07989336	1	34.125	30.5073176								
6	4E+07	6_44733244	3	11	10	0	7	5	0.693374	1	15	8	12	0	12	6	0.45526626	1	7.375	5.70557372					4.5E+07	4.5E+07	0.23877	ID=promoter_43555;
6	4E+07	6_448028	72	24	22	16	48	19	1.43E-04	0.01212	21	27	3	2	24	3	6.10E-04	0.0415146	23.375	21.7514367								
6	4E+07	6_448792	0	9	59	51	5	55	3.55E-08	#######	24	40	38	64	32	51	0.05193594	1	35.625	23.070931								
6	4E+07	6_448927	4	0	14	25	2	20	0.002384	0.12916	13	10	21	4	12	13	0.92692342	1	11.375	8.68393426								
6	4E+07	6_44920254	1	83	67	50	42	59	0.670281	1	39	61	94	34	50	64	0.22912492	1	53.625	29.5390178					4.5E+07	4.5E+07	0.12849	ID=promoter_43559;
6	4E+07	6_449738	5	34	3	8	20	6	0.006754	0.29841	0	16	2	2	8	2	0.20550608	1	8.75	11.3735282								
6	4E+07	6_449914	44	28	35	33	36	34	0.662257	1	22	29	30	20	26	25	0.99152959	1	30.125	7.54865172								
6	5E+07	6_45064969	21	0	39	13	11	26	0.138574	1	0	11	2	14	6	8	0.79537884	1	12.5	13.1039798					4.5E+07	4.5E+07	0.10009	ID=promoter_43565;
6	5E+07	6_451508	15	90	32	33	53	33	0.014749	0.55477	108	30	53	64	69	59	0.90130219	1	53.125	32.3571034								
6	5E+07	6_452506	169	111	122	108	140	115	0.017445	0.63218	94	37	75	49	66	62	0.99720434	1	95.625	42.2710218								
6	5E+07	6_453207	13	17	10	25	15	18	0.997876	1	1	8	7	1	5	4	0.97610903	1	10.25	8.08437647								
6	5E+07	6_453818	24	6	16	15	15	16	0.965652	1	26	8	38	26	17	32	0.05485386	1	19.875	10.6427373								
6	5E+07	6_453896	9	16	11	5	13	8	0.344217	1	1	16	9	1	9	5	0.6500913	1	8.5	5.90399381								
6	5E+07	6_453909	2	11	0	7	7	4	0.503048	1	31	11	13	0	21	7	0.05007015	1	9.375	10.155048								
6	5E+07	6_45425960	15	53	29	22	34	26	0.254409	1	50	15	34	33	33	34	0.91076368	1	31.375	14.4116768	5E+07	4.5E+07	ID=ENSP00000343552;ICMT;NM_012405;	ICMT	4.5E+07	4.5E+07	0.6663	ID=promoter_43599;
6	5E+07	6_454715	0	19	6	5	10	6	0.41703	1	14	15	53	11	15	32	0.02335039	0.7680341	15.375	16.3963193								
6	5E+07	6_456057	6	20	11	13	13	12	0.835334	1	11	16	5	1	14	3	0.04246461	1	10.375	6.18610193								
6	5E+07	6_456064	11	13	22	16	12	19	0.716431	1	32	17	27	15	25	21	0.94599092	1	19.125	7.27888335								
6	5E+07	6_456234	0	7	3	14	4	9	0.54411	1	1	3	3	11	2	7	0.20455692	1	5.25	4.97852531								
6	5E+07	6_457636	0	2	2	3	1	3	0.700657	1	40	7	12	0	24	6	0.00867909	0.3505674	8.25	13.4456578								
6	5E+07	6_458365	7	17	12	12	12	12	0.926346	1	2	13	9	15	8	12	0.56746184	1	10.875	4.76407689								
6	5E+07	6_458558	18	11	43	34	15	39	0.026862	0.89217	46	5	6	38	26	22	0.95151398	1	25.125	16.9910691								
6	5E+07	6_459034	12	1	5	12	7	9	0.983538	1	2	8	0	53	5	27	2.18E-04	0.0177562	11.625	17.3611841								
6	5E+07	6_459693	60	0	33	40	30	37	0.979341	1	10	17	12	1	14	7	0.39930956	1	21.625	20.9825608								
6	5E+07	6_459775	30	48	11	20	39	16	0.001052	0.06629	10	8	16	20	9	18	0.15524483	1	20.375	13.2226807								
6	5E+07	6_461207	112	52	111	135	82	123	0.224141	1	32	33	22	35	33	29	0.97818147	1	66.5	45.0998891								
6	5E+07	6_461262	14	26	22	21	20	22	0.978625	1	15	9	3	1	12	2	0.04045894	1	13.875	9.04650682								
6	5E+07	6_461465	30	12	7	16	21	12	0.1273	1	11	8	14	0	10	7	0.84912739	1	12.25	8.69729022								
6	5E+07	6_46172032	7	6	29	20	7	25	0.023401	0.7984	17	10	6	25	14	16	0.84864027	1	15	9.07114735					4.6E+07	4.6E+07	0.14863	ID=promoter_43681;
6	5E+07	6_462557	3	1	14	7	2	11	0.085797	1	5	7	15	6	6	11	0.39136385	1	7.25	4.92080133								
6	5E+07	6_46267481	786	293	711	702	540	707	0.156927	1	471	274	391	166	373	279	0.0249754	0.8106522	474.25	232.998927					4.6E+07	4.6E+07	0.64528	ID=promoter_43692;
6	5E+07	6_46377168	84	40	56	80	62	68	0.929462	1	23	58	26	28	41	27	0.38241111	1	49.375	24.0293719					4.6E+07	4.6E+07	0.38683	ID=promoter_43698;

6	5E+07	6_46377908	367	201	390	355	284	373	0.356313	1	235	94	101	20	165	61	1.72E-09	4.80E-07	220.38	141.095649					4.6E+07	4.6E+07	0.38683	ID=promoter_43698;
6	5E+07	6_463976	58	88	163	202	73	183	1.39E-07	#######	95	51	104	93	73	99	0.04609138	1	106.75	51.2800435								
6	5E+07	6_464374	28	37	7	7	33	7	2.22E-05	0.00241	2	15	4	30	9	17	0.21129426	1	16.25	13.5409432								
6	5E+07	6_46465010	3	21	4	6	12	5	0.126784	1	2	1	6	2	2	4	0.6610374	1	5.625	6.47936284	5E+07	4.7E+07	ID=ENSP00000383700;RERE;NM_001042681;	RERE	4.6E+07	4.6E+07	0.32783	ID=promoter_43708;
6	5E+07	6_465352	8	26	8	12	17	10	0.210142	1	32	17	19	27	25	23	0.99965163	1	18.625	9.07016302								
6	5E+07	6_46638633	9	13	6	8	11	7	0.438207	1	47	8	25	7	28	16	0.29661245	1	15.375	14.1717778					4.7E+07	4.7E+07	0.11505	ID=promoter_43730;
6	5E+07	6_466475	18	1	12	1	10	7	0.570701	1	3	0	11	0	2	6	0.31385293	1	5.75	6.9230464								
6	5E+07	6_46750355	25	19	26	21	22	24	0.97026	1	70	17	8	2	44	5	7.53E-07	0.0001294	23.5	20.5008711	5E+07	4.7E+07	ID=ENSP00000270685;;					
6	5E+07	6_468019	6	39	14	10	23	12	0.065071	1	2	11	15	2	7	9	0.80856893	1	12.375	11.8434732								
6	5E+07	6_47040495	16	1	11	11	9	11	0.995818	1	0	16	5	14	8	10	0.81871774	1	9.25	6.4531277	5E+07	4.7E+07	ID=ENSP00000306328;SLC25A33;NM_032315;	SLC25A33	4.7E+07	4.7E+07	0.31071	ID=promoter_43747;
6	5E+07	6_472162	100	75	62	61	88	62	0.007998	0.34244	110	35	64	29	73	47	0.14644849	1	67	28.1323403								
6	5E+07	6_472749	4	20	8	10	12	9	0.592799	1	30	16	2	51	23	27	0.70626281	1	17.625	16.2826595								
6	5E+07	6_473017	5	2	6	0	4	3	0.840045	1	25	13	5	0	19	3	0.00532306	0.24159	7	8.38365757								
6	5E+07	6_47393781	81	29	98	64	55	81	0.426543	1	8	7	22	53	8	38	1.73E-05	0.0020431	45.25	34.0744982					4.7E+07	4.7E+07	0.27075	ID=promoter_43779;
6	5E+07	6_473945	1	0	2	10	1	6	0.241978	1	0	12	20	3	6	12	0.2888538	1	6	7.2702918								
6	5E+07	6_47499758	33	30	56	55	32	56	0.191727	1	111	115	55	90	113	73	0.05370768	1	68.125	33.1508996	5E+07	4.8E+07	ID=ENSP00000366262;PGD;NM_002631;	PGD	4.7E+07	4.8E+07	0.23758	ID=promoter_43788;
6	5E+07	6_47543371	0	6	29	20	3	25	8.89E-04	0.05749	4	0	3	0	2	2	0.99713337	1	7.75	10.8331502	5E+07	4.8E+07	ID=ENSP00000366237;DFFA;NM_004401;	DFFA				
6	5E+07	6_47576827	117	72	68	58	95	63	0.001785	0.10259	136	134	89	6	135	48	2.41E-08	5.62E-06	85	43.8666811					4.8E+07	4.8E+07	0.10475	ID=promoter_43795;
6	5E+07	6_47593126	123	89	146	96	106	121	0.979354	1	305	182	192	59	244	126	4.41E-07	8.01E-05	149	77.8900324					4.8E+07	4.8E+07	0.14166	ID=promoter_43798;
6	5E+07	6_477157	0	6	7	3	3	5	0.88968	1	0	13	13	0	7	7	0.99000271	1	5.25	5.49675229								
6	5E+07	6_47726423	4	0	2	0	2	1	0.773064	1	14	5	7	30	10	19	0.1719433	1	7.75	10.0676285					4.8E+07	4.8E+07	0.31071	ID=promoter_43813;
6	5E+07	6_478753	148	39	88	58	94	73	0.028919	0.94726	0	1	3	1	1	2	0.81304206	1	42.25	53.8032129								
6	5E+07	6_478884	278	91	150	194	185	172	0.089138	1	63	53	105	47	58	76	0.13679073	1	122.63	80.549785								
6	5E+07	6_479232	83	99	63	63	91	63	0.005014	0.23539	150	115	88	39	133	64	6.60E-05	0.0064063	87.5	34.5749703								
6	5E+07	6_48004923	569	151	380	220	360	300	6.41E-05	0.00605	243	139	123	44	191	84	4.19E-08	9.39E-06	233.63	168.248063					4.8E+07	4.8E+07	0.43529	ID=promoter_43838;
6	5E+07	6_48067954	0	0	0	0	0	0	1	1	31	0	10	0	16	5	0.08348313	1	5.125	11.0251336					4.8E+07	4.8E+07	0.2376	ID=promoter_43846;
6	5E+07	6_480725	87	56	59	41	72	50	0.015806	0.58672	53	52	23	48	53	36	0.35159657	1	52.375	17.999504								
6	5E+07	6_48073620	6	6	9	5	6	7	0.999953	1	23	20	2	1	22	2	4.98E-04	0.0353761	9	8.14160391					4.8E+07	4.8E+07	0.22146	ID=promoter_43848;
6	5E+07	6_482769	6	39	4	8	23	6	0.001551	0.09145	0	5	14	11	3	13	0.02959638	0.9232309	10.875	12.1471631								
6	5E+07	6_483888	7	5	4	7	6	6	0.962469	1	8	1	2	9	5	6	0.91271846	1	5.375	2.87538817								
6	5E+07	6_483997	367	365	293	335	366	314	2.40E-04	0.01893	200	65	91	68	133	80	0.0086793	0.3505674	223	133.783407								
6	5E+07	6_484247	67	54	19	35	61	27	6.68E-05	0.00626	18	0	22	24	9	23	0.02676526	0.8546242	29.875	21.4970928								
6	5E+07	6_484282	21	17	38	8	19	23	0.994841	1	10	9	24	4	10	14	0.60490119	1	16.375	11.1218896								
6	5E+07	6_485225	247	53	254	218	150	236	0.018627	0.6674	239	68	80	51	154	66	5.32E-07	9.44E-05	151.25	95.3126434								
6	5E+07	6_48626882	20	11	9	13	16	11	0.385778	1	15	16	28	14	16	21	0.57439123	1	15.75	5.94618725	5E+07	4.9E+07	ID=ENSP00000365944;FBXO6;NM_018438;	FBXO6				
6	5E+07	6_487290	10	34	2	0	22	1	1.12E-05	0.00132	3	10	19	2	7	11	0.54353855	1	10	11.5511286								

6	5E+07	6_48754728	333	66	290	273	200	282	0.137	1	150	82	102	19	116	61	0.00136139	0.0808579	164.38	118.146323					4.9E+07	4.9E+07	0.36677	ID=promoter_43891;
6	5E+07	6_48905696	29	2	15	11	16	13	0.613293	1	12	0	8	7	6	8	0.79537884	1	10.5	8.99205999					4.9E+07	4.9E+07	0.1514	ID=promoter_43906;
6	5E+07	6_489633	69	354	92	120	212	106	1.42E-12	#######	152	336	209	124	244	167	0.0100631	0.3953877	182	108.894444								
6	5E+07	6_49445384	0	38	4	15	19	10	0.108738	1	23	16	25	3	20	14	0.72101732	1	15.5	12.9945043	5E+07	5E+07	ID=ENSP0000011700;VPS13D;NM_015378;	VPS13D				
6	5E+07	6_50121672	652	170	415	307	411	361	3.05E-04	0.02346	56	146	150	22	101	86	0.84537677	1	239.75	209.786797	5E+07	5E+07	ID=ENSP0000365253;LRRC38;;	LRRC38	5E+07	5E+07	0.57845	ID=promoter_43952;
6	5E+07	6_501601	172	152	140	177	162	159	0.278679	1	231	204	195	208	218	202	0.99998828	1	184.88	30.3618064								
6	5E+07	6_501943	29	89	34	31	59	33	0.002304	0.12507	130	34	88	58	82	73	0.96881136	1	61.625	37.1519563								
6	5E+07	6_50294816	89	56	72	98	73	85	0.998945	1	23	58	34	36	41	35	0.93777492	1	58.25	26.8474527					5E+07	5E+07	0.17826	ID=promoter_43959;
6	5E+07	6_503108	16	16	11	22	16	17	0.960459	1	21	25	20	22	23	21	0.9987298	1	19.125	4.48608961								
6	5E+07	6_50326366	293	314	271	299	304	285	0.024528	0.83049	199	141	150	44	170	97	6.01E-04	0.0409216	213.88	96.55855					5E+07	5E+07	0.57214	ID=promoter_43960;
6	5E+07	6_50354693	104	65	55	39	85	47	1.27E-04	0.01105	28	55	21	10	42	16	0.0075077	0.3140846	47.125	29.6909678					5E+07	5E+07	0.77598	ID=promoter_43961;
6	5E+07	6_505520	50	27	40	30	39	35	0.514222	1	77	53	31	30	65	31	0.00815344	0.3345322	42.25	17.1026314								
6	5E+07	6_507560	156	85	116	140	121	128	0.717436	1	5	21	2	2	13	2	0.02644099	0.8480056	65.875	65.8731194								
6	5E+07	6_508228	7	0	35	22	4	29	4.84E-04	0.03438	49	20	31	21	35	26	0.69250145	1	23.125	15.5051835								
6	5E+07	6_508795	14	12	15	27	13	21	0.660505	1	0	16	23	1	8	12	0.56746184	1	13.5	9.42640668								
6	5E+07	6_50927890	18	13	28	22	16	25	0.667718	1	28	12	8	0	20	4	0.00863789	0.3495868	16.125	9.80433578	5E+07	5.1E+07	ID=ENSP0000365198;;NM_201628;					
6	5E+07	6_50954024	24	8	21	6	16	14	0.724026	1	0	0	0	0	0	0	1	1	7.375	9.86968664	5E+07	5.1E+07	ID=ENSP0000365198;;NM_201628;					
6	5E+07	6_51196961	202	51	159	143	127	151	0.993671	1	136	57	79	107	97	93	0.97348576	1	116.75	52.8143649	5E+07	5.1E+07	ID=ENSP0000365147;EFHD2;NM_024329;	EFHD2				
6	5E+07	6_51201233	129	33	64	66	81	65	0.072126	1	26	29	82	0	28	41	0.17053904	1	53.625	40.3871886	5E+07	5.1E+07	ID=ENSP0000365147;EFHD2;NM_024329;	EFHD2				
6	5E+07	6_51206563	13	24	17	19	19	18	0.809248	1	1	39	15	24	20	20	0.97170071	1	19	10.8890509					5.1E+07	5.1E+07	0.12971	ID=promoter_43986;
6	5E+07	6_513728	3	36	9	10	20	10	0.076235	1	23	32	42	17	28	30	0.85900118	1	21.5	14.1117985								
6	5E+07	6_51380040	34	88	42	64	61	53	0.276178	1	97	107	185	91	102	138	0.01439312	0.5251956	88.5	47.004559	5E+07	5.1E+07	ID=ENSP0000365007;DNAJC16;NM_015291;	DNAJC16				
6	5E+07	6_51512655	22	45	24	11	34	18	0.01975	0.69938	0	26	10	2	13	6	0.35360803	1	17.5	14.8131216					5.2E+07	5.2E+07	0.19599	ID=promoter_43998;
6	5E+07	6_51564824	803	181	576	471	492	524	0.306449	1	331	209	203	71	270	137	4.09E-08	9.21E-06	355.63	244.635553					5.2E+07	5.2E+07	0.70538	ID=promoter_44001;
6	5E+07	6_51675496	71	28	75	36	50	56	0.972202	1	34	8	38	1	21	20	0.99628266	1	36.375	26.2566318	5E+07	5.2E+07	ID=ENSP0000384858;EPHA2;;	EPHA2	5.2E+07	5.2E+07	0.1842	ID=promoter_44005;
6	5E+07	6_517450	2	72	7	14	37	11	8.94E-05	0.00805	2	16	24	13	9	19	0.11219606	1	18.75	22.7580441								
6	5E+07	6_52087875	12	16	7	22	14	15	0.970741	1	26	15	45	26	21	36	0.07531112	1	21.125	11.7404977	5E+07	5.2E+07	ID=ENSP0000327214;ATP13A2;NM_022089;	ATP13A2				
6	5E+07	6_523241	5	20	3	6	13	5	0.083134	1	21	2	8	18	12	13	0.92692342	1	10.375	7.94512429								
6	5E+07	6_525318	305	99	208	171	202	190	0.091111	1	74	134	143	5	104	74	0.21826129	1	142.38	90.2946565								

6	5E+07	6_526018	2	11	14	9	7	12	0.724589	1	20	3	27	2	12	15	0.74080056	1	11	9.07114735								
6	5E+07	6_52694905	9	23	4	1	16	3	0.003782	0.18716	12	1	4	10	7	7	0.99000271	1	8	7.32900305					5.3E+07	5.3E+07	0.08978	ID=promoter_44041;
6	5E+07	6_529291	0	57	20	5	29	13	0.011304	0.44898	26	3	22	44	15	33	0.01662686	0.5898213	22.125	20.2233069								
6	5E+07	6_530240	18	65	56	77	42	67	0.292238	1	46	33	65	11	40	38	0.9941866	1	46.375	23.7843736								
6	5E+07	6_531927	3	27	1	22	15	12	0.610117	1	8	15	23	53	12	38	4.13E-04	0.0302097	19	16.7417357								
6	5E+07	6_531997	30	31	10	13	31	12	0.002785	0.14699	18	45	18	26	32	22	0.55606214	1	23.875	11.4572186								
6	5E+07	6_535132	3	4	12	3	4	8	0.677894	1	10	11	2	0	11	1	0.02232329	0.7455867	5.625	4.6271713								
6	5E+07	6_536599	21	51	28	9	36	19	0.015282	0.57166	42	51	27	16	47	22	0.02632675	0.8479606	30.625	15.8288841								
6	5E+07	6_537378	131	93	95	76	112	86	0.011893	0.46411	37	14	8	2	26	5	0.00174818	0.0978457	57	48.1841705								
6	5E+07	6_53753928	90	25	73	83	58	78	0.732365	1	24	13	17	6	19	12	0.57792516	1	41.375	34.4712613	5E+07	5.4E+07	ID=ENSP0000364243-D2;PLA2G2F;NM_022819;	PLA2G2F				
6	5E+07	6_538132	14	4	15	11	9	13	0.89076	1	0	11	0	1	6	1	0.20114407	1	7	6.41426981								
6	5E+07	6_53932980	13	9	10	2	11	6	0.3028	1	3	6	6	1	5	4	0.97610903	1	6.25	4.20034012	5E+07	5.4E+07	ID=ENSP00000247992;PLA2G2C;NM_001105572;	PLA2G2C				
6	5E+07	6_54050156	1963	853	2066	1835	1408	1951	1.14E-05	0.00134	1015	833	955	276	924	616	1.57E-09	4.43E-07	1224.5	647.376243					5.4E+07	5.4E+07	0.77122	ID=promoter_44081;
6	5E+07	6_541629	19	26	8	9	23	9	0.013495	0.51644	0	0	20	32	0	26	8.13E-07	0.0001389	14.25	11.8412113								
6	5E+07	6_547739	27	36	49	44	32	47	0.616656	1	84	27	61	21	56	41	0.51562687	1	43.625	20.9893681								
6	5E+07	6_547780	0	6	17	1	3	9	0.343967	1	34	15	37	0	25	19	0.80752973	1	13.75	14.9451378								
6	5E+07	6_548440	4	14	4	10	9	7	0.713906	1	0	8	0	0	4	0	0.15659576	1	5	5.23722937								
6	6E+07	6_55007328	4	12	2	0	8	1	0.03583	1	0	4	17	1	2	9	0.08177705	1	5	6.21059003	5E+07	5.5E+07	ID=ENSP00003638 27;HSPG2;NM_005529;	HSPG2				
6	6E+07	6_550467	169	67	126	107	118	117	0.435451	1	50	32	26	24	41	25	0.25084268	1	75.125	53.4534176								
6	6E+07	6_551842	70	14	19	22	42	21	0.004493	0.21484	0	0	1	0	0	1	0.58312783	1	15.75	23.77724								
6	6E+07	6_552611	16	8	5	7	12	6	0.213448	1	4	2	8	2	3	5	0.7175395	1	6.5	4.53557368								
6	6E+07	6_552716	699	392	850	931	546	891	8.16E-09	#######	207	219	152	127	213	140	0.0064437	0.2777929	447.13	329.978977								
6	6E+07	6_553894	94	41	61	86	68	74	0.905175	1	33	35	39	89	34	64	0.00300895	0.1522956	59.75	26.2664914								
6	6E+07	6_55507607	2	4	7	15	3	11	0.181935	1	0	0	8	7	0	8	0.01336935	0.4945372	5.375	5.01248441					5.6E+07	5.6E+07	0.18849	ID=promoter_44108;
6	6E+07	6_555240	94	66	42	35	80	39	2.10E-05	0.00229	65	38	19	2	52	11	7.13E-06	0.0009418	45.125	29.0931386								
6	6E+07	6_557263	54	12	18	29	33	24	0.19836	1	31	29	12	45	30	29	0.98716634	1	28.75	15.0404217								
6	6E+07	6_561376	2	13	10	5	8	8	0.950274	1	7	9	9	24	8	17	0.13786384	1	9.875	6.59951297								
6	6E+07	6_56374294	103	41	47	92	72	70	0.534169	1	1	12	38	0	7	19	0.03889743	1	41.75	38.9275885	6E+07	5.6E+07	ID=ENSP00002362 55;GRHL3;NM_021180;	GRHL3				
6	6E+07	6_56386105	157	6	113	107	82	110	0.654489	1	5	18	17	0	12	9	0.89054267	1	52.875	62.3067011	6E+07	5.6E+07	ID=ENSP00002362 55;GRHL3;NM_021180;	GRHL3	5.6E+07	5.6E+07	0.22592	ID=promoter_44132;
6	6E+07	6_564458	54	42	61	41	48	51	0.886006	1	17	8	5	34	13	20	0.35609139	1	32.75	20.8240932								
6	6E+07	6_565096	8	20	12	18	14	15	0.970741	1	2	19	31	15	11	23	0.07426989	1	15.625	8.70036945								
6	6E+07	6_56805375	26	162	47	46	94	47	5.57E-06	0.00071	73	93	128	60	83	94	0.41214619	1	79.375	45.9873818	6E+07	5.7E+07	ID=ENSP00003635 10;SRRM1;;	SRRM1				
6	6E+07	6_570194	4	36	1	9	20	5	0.002998	0.15536	17	12	18	5	15	12	0.93002578	1	12.75	11.1835338								
6	6E+07	6_572070	315	109	761	870	212	816	1.09E-58	#######	66	106	301	96	86	199	2.03E-12	8.19E-10	328	316.648521								
6	6E+07	6_57294159	22	53	27	45	38	36	0.654883	1	22	47	62	76	35	69	9.79E-04	0.0613359	44.25	19.623237	6E+07	5.7E+07	ID=ENSP00003634 52;MAN1C1;NM_020379;	MAN1C1				
6	6E+07	6_574826	417	231	316	399	324	358	0.738202	1	284	127	132	46	206	89	5.54E-09	1.44E-06	244	134.347098								
6	6E+07	6_57670108	941	520	595	594	731	595	2.43E-10	#######	643	162	296	155	403	226	7.71E-09	1.94E-06	488.25	269.118004					5.8E+07	5.8E+07	0.21176	ID=promoter_44169;

6	6E+07	6_57793603	5	26	3	8	16	6	0.042495	1	3	5	13	5	4	9	0.31291743	1	8.5	7.78276484	6E+07	5.8E+07	ID=ENSP0000320485;ARID1A;NM_006015;	ARID1A				
6	6E+07	6_57845496	4	40	9	13	22	11	0.058934	1	6	32	67	25	19	46	0.0012482	0.0750721	24.5	21.5074738	6E+07	5.8E+07	ID=ENSP0000363257;ZDHHC18;NM_032283;	ZDHHC18	5.8E+07	5.8E+07	0.15575	ID=promoter_44174;
6	6E+07	6_57879215	30	49	9	11	40	10	8.99E-06	0.00109	20	65	24	4	43	14	0.00178346	0.0995187	26.5	21.0509586	6E+07	5.8E+07	ID=ENSP0000354645;GPATCH3;NM_022078;	GPATCH3				
6	6E+07	6_580220	1	19	15	21	10	18	0.549586	1	0	14	11	36	7	24	0.00482107	0.2211976	14.625	11.5255926								
6	6E+07	6_580297	1	0	4	11	1	8	0.109969	1	0	3	9	17	2	13	0.01148244	0.4380166	5.625	6.13974173								
6	6E+07	6_58135989	404	154	317	365	279	341	0.878175	1	247	144	141	45	196	93	4.20E-07	7.69E-05	227.13	126.271179					5.8E+07	5.8E+07	0.42171	ID=promoter_44187;
6	6E+07	6_58239538	975	320	752	619	648	686	0.173194	1	272	149	197	159	211	178	0.65064762	1	430.38	311.719626					5.8E+07	5.8E+07	0.45035	ID=promoter_44189;
6	6E+07	6_58272174	195	61	158	126	128	142	0.900719	1	152	37	47	19	95	33	2.82E-06	0.0004194	99.375	66.1447277					5.8E+07	5.8E+07	0.1948	ID=promoter_44193;
6	6E+07	6_58409774	27	32	43	89	30	66	0.014766	0.55527	7	16	9	21	12	15	0.74080056	1	30.5	26.4791023	6E+07	5.8E+07	ID=ENSP0000382126-D3;FGR;NM_001042747;	FGR				
6	6E+07	6_584343	228	127	211	118	178	165	0.092305	1	93	31	13	2	62	8	6.44E-09	1.64E-06	102.88	85.9192935								
6	6E+07	6_586303	30	47	14	5	39	10	1.49E-05	0.00168	14	14	49	17	14	33	0.01005158	0.3950437	23.75	16.4729215								
6	6E+07	6_587288	20	4	11	1	12	6	0.213448	1	1	6	11	1	4	6	0.75330063	1	6.875	6.74933859								
6	6E+07	6_587443	1	34	7	3	18	5	0.008132	0.34618	14	0	20	0	7	10	0.67636194	1	9.875	12.1353969								
6	6E+07	6_587815	4	48	10	3	26	7	7.99E-04	0.0525	5	4	6	2	5	4	0.97610903	1	10.25	15.4434452								
6	6E+07	6_58919608	51	28	77	38	40	58	0.58615	1	26	14	18	13	20	16	0.90780655	1	33.125	21.8595353					5.9E+07	5.9E+07	0.80669	ID=promoter_44208;
6	6E+07	6_58925357	51	14	34	34	33	34	0.868497	1	3	7	22	10	5	16	0.03625394	1	21.875	16.5739684					5.9E+07	5.9E+07	0.15037	ID=promoter_44209;
6	6E+07	6_58982927	29	40	31	63	35	47	0.832078	1	0	4	4	3	2	4	0.6610374	1	21.75	22.7643205					5.9E+07	5.9E+07	0.19615	ID=promoter_44212;
6	6E+07	6_59020088	699	162	468	376	431	422	0.030566	0.98374	477	221	169	85	349	127	9.81E-20	6.61E-17	332.13	208.718839					5.9E+07	5.9E+07	0.71345	ID=promoter_44214;
6	6E+07	6_592356	17	2	5	11	10	8	0.719353	1	18	27	36	2	23	19	0.93310412	1	14.75	12.2561938								
6	6E+07	6_595076	4	26	0	12	15	6	0.065303	1	2	26	29	38	14	34	0.0069754	0.2965071	17.125	14.4166323								
6	6E+07	6_595372	340	137	257	213	239	235	0.157488	1	126	96	133	20	111	77	0.14591256	1	165.25	100.361845								
6	6E+07	6_596185	13	11	14	6	12	10	0.724827	1	4	27	32	25	16	29	0.09020737	1	16.5	10.2678416								
6	6E+07	6_596812	8	4	20	4	6	12	0.558139	1	0	0	5	29	0	17	1.04E-04	0.0094884	8.75	10.3475325								
6	6E+07	6_597325	39	39	57	70	39	64	0.251797	1	20	12	32	39	16	36	0.0095878	0.3813297	38.5	18.6010752								
6	6E+07	6_59804063	4	87	11	8	46	10	4.10E-07	#######	14	5	26	2	10	14	0.60490119	1	19.625	28.2586902	6E+07	6E+07	ID=ENSP0000362854;PUM1;;	PUM1				
6	6E+07	6_599924	3	68	12	11	36	12	2.99E-04	0.02308	27	39	20	12	33	16	0.09595749	1	24	20.9897934								
6	6E+07	6_601097	12	17	6	13	15	10	0.374041	1	14	2	11	3	8	7	0.9937864	1	9.75	5.44452543								
6	6E+07	6_60435391	1834	858	1982	1193	1346	1588	0.987768	1	1097	609	722	277	853	500	2.31E-15	1.20E-12	1071.5	590.241839					6E+07	6E+07	0.28452	ID=promoter_44232;
6	6E+07	6_604551	64	68	37	76	66	57	0.235893	1	23	30	19	15	27	17	0.45364599	1	41.5	24.2074369								
6	6E+07	6_604726	105	96	80	65	101	73	0.006315	0.28437	0	63	29	36	32	33	0.9118622	1	59.25	35.6320482								
6	6E+07	6_611401	0	45	173	95	23	134	3.12E-14	#######	51	30	91	69	41	80	3.86E-04	0.0284975	69.25	52.3142974								
6	6E+07	6_611802	23	36	13	60	30	37	0.979341	1	73	36	140	16	55	78	0.05186876	1	49.625	42.0439821								
6	6E+07	6_611988	222	60	209	151	141	180	0.753075	1	0	57	17	32	29	25	0.96515949	1	93.5	87.8228737								
6	6E+07	6_61232432	31	7	41	18	19	30	0.595918	1	3	8	10	0	6	5	0.98461866	1	14.75	14.3800457	6E+07	6.1E+07	ID=ENSP0000324450;ZSCAN20;NM_145238;	ZSCAN20				
6	6E+07	6_613300	99	31	73	76	65	75	0.995314	1	27	13	12	15	20	14	0.72101732	1	43.25	34.1624271								
6	6E+07	6_613341	14	14	17	4	14	11	0.605836	1	22	10	4	0	16	2	0.00719486	0.3031284	10.625	7.50119038								
6	6E+07	6_614794	0	6	6	21	3	14	0.063635	1	4	0	4	1	2	3	0.86792893	1	5.25	6.81909085								

6	6E+07	6_619163	41	63	41	31	52	36	0.048511	1	25	25	19	25	25	22	0.9842524	1	33.75	14.220207								
6	6E+07	6_620280	0	30	1	4	15	3	0.006447	0.2884	12	1	16	17	7	17	0.08351885	1	10.125	10.6023919								
6	6E+07	6_62360819	222	83	171	152	153	162	0.661905	1	75	93	77	41	84	59	0.26099774	1	114.25	60.9185639					6.2E+07	6.2E+07	0.33688	ID=promoter_44286;
6	6E+07	6_62562505	151	27	55	69	89	62	0.006261	0.28201	31	25	42	34	28	38	0.30569567	1	54.25	41.8799304					6.3E+07	6.3E+07	0.38928	ID=promoter_44289;
6	6E+07	6_627608	10	148	53	45	79	49	0.001748	0.10098	136	138	165	18	137	92	0.05579967	1	89.125	63.6742547								
6	6E+07	6_629912	15	73	15	21	44	18	4.24E-04	0.03084	26	22	35	45	24	40	0.07026944	1	31.5	19.6104928								
6	6E+07	6_631112	20	9	15	12	15	14	0.827043	1	0	1	16	37	1	27	2.06E-06	0.0003194	13.75	11.7321293								
6	6E+07	6_632203	2	52	0	15	27	8	0.001088	0.0681	34	14	22	14	24	18	0.79306625	1	19.125	17.0665713								
6	6E+07	6_632283	53	14	34	35	34	35	0.863165	1	2	39	38	1	21	20	0.99628266	1	27	18.9887185								
6	6E+07	6_632898	14	11	12	2	13	7	0.234546	1	19	6	4	0	13	2	0.02644099	0.8480056	8.5	6.54653671								
6	6E+07	6_637675	5	26	9	3	16	6	0.042495	1	30	3	28	1	17	15	0.99031847	1	13.125	12.5747763								
6	6E+07	6_639047	3	22	0	5	13	3	0.018372	0.65917	0	1	18	8	1	13	0.0036419	0.1775497	7.125	8.45893441								
6	6E+07	6_643478	511	189	497	411	350	454	0.367257	1	20	23	31	2	22	17	0.8526593	1	210.5	226.836631								
6	7E+07	6_652071	11	4	5	15	8	10	0.991043	1	21	8	13	10	15	12	0.93002578	1	10.875	5.54043577								
6	7E+07	6_653672	12	3	11	2	8	7	0.850897	1	0	1	2	9	1	6	0.13767373	1	5	4.84030696								
6	7E+07	6_65407718	0	3	7	6	2	7	0.358464	1	19	10	11	1	15	6	0.20085144	1	7.125	6.22064535	7E+07	6.5E+07	ID=ENSP0000361949;PABPC4;NM_001135653;	PABPC4				
6	7E+07	6_656395	50	35	45	52	43	49	0.990477	1	0	7	21	2	4	12	0.09850366	1	26.5	21.8108754								
6	7E+07	6_66182934	143	92	123	123	118	123	0.65708	1	101	93	124	113	97	119	0.12110526	1	114	17.7200451					6.6E+07	6.6E+07	0.21263	ID=promoter_44387;
6	7E+07	6_661922	23	23	95	49	23	72	1.12E-04	0.00988	21	0	39	22	11	31	0.00385975	0.1846363	34	28.4906								
6	7E+07	6_662029	0	11	1	2	6	2	0.258649	1	19	6	17	18	13	18	0.54030568	1	9.25	8.0311892								
6	7E+07	6_66415461	4	21	17	15	13	16	0.991731	1	0	49	0	10	25	5	0.00263335	0.136745	14.5	15.9910689					6.6E+07	6.6E+07	0.13734	ID=promoter_44390;
6	7E+07	6_664761	177	198	240	227	188	234	0.832204	1	151	140	493	30	146	262	6.18E-10	1.88E-07	207	132.675759								
6	7E+07	6_665574	1	10	8	7	6	8	0.9722	1	6	11	6	5	9	6	0.8208167	1	6.75	3.10529502								
6	7E+07	6_665707	11	3	1	0	7	1	0.062633	1	39	18	7	1	29	4	1.92E-04	0.0158648	10	13.2341549								
6	7E+07	6_665946	90	150	66	69	120	68	6.13E-06	0.00077	69	39	44	37	54	41	0.62747256	1	70.5	36.9207646								
6	7E+07	6_667152	21	26	19	15	24	17	0.274507	1	47	25	33	9	36	21	0.23487	1	24.375	11.6733824								
6	7E+07	6_66890354	16	42	17	10	29	14	0.018844	0.67198	17	2	29	1	10	15	0.49251655	1	16.75	13.6250819	7E+07	6.7E+07	ID=ENSP0000303316;C18orf58;;	C18orf58				
6	7E+07	6_670370	68	26	43	44	47	44	0.559809	1	0	0	0	0	0	0	1	1	22.625	26.6990503								
6	7E+07	6_671179	69	65	71	72	67	72	0.87538	1	11	49	47	7	30	27	0.99379101	1	48.875	26.40583								
6	7E+07	6_67219045	4	13	1	10	9	6	0.554309	1	1	6	23	1	4	12	0.09850366	1	7.375	7.72634639	7E+07	6.7E+07	ID=ENSP0000372924;RALBP1;NM_006788;	RALBP1				
6	7E+07	6_677743	0	14	9	0	7	5	0.693374	1	25	0	14	1	13	8	0.65599869	1	7.875	9.2803556								
6	7E+07	6_678436	61	27	104	42	44	73	0.18882	1	15	30	27	7	23	17	0.77734386	1	39.125	30.9074078								
6	7E+07	6_678452	21	21	13	10	21	12	0.1273	1	43	11	6	11	27	9	0.02128286	0.7187388	17	11.7473401								
6	7E+07	6_679792	16	19	11	13	18	12	0.307258	1	2	9	9	26	6	18	0.03091566	0.954015	13.125	7.27888335								
6	7E+07	6_683912	8	5	10	0	7	5	0.693374	1	2	5	26	19	4	23	5.91E-04	0.0404335	9.375	8.87914893								
6	7E+07	6_684378	177	92	65	52	135	59	3.81E-10	#######	97	18	94	66	58	80	0.06793	1	82.625	46.3401631								
6	7E+07	6_685400	26	12	34	22	19	28	0.743706	1	0	20	16	20	10	18	0.2304087	1	18.75	10.0249688								
6	7E+07	6_686361	25	94	70	9	60	40	0.019574	0.69488	47	21	25	2	34	14	0.0317829	0.9755061	36.625	31.5998983								
6	7E+07	6_688326	7	16	1	6	12	4	0.066899	1	137	9	11	21	73	16	9.38E-08	1.96E-05	26	45.2706149								
6	7E+07	6_691137	61	110	55	33	86	44	2.48E-05	0.00264	126	76	94	36	101	65	0.06970605	1	73.875	33.9303383								
6	7E+07	6_692143	12	92	15	13	52	14	6.39E-07	0.0001	46	33	36	79	40	58	0.08997623	1	40.75	30.3773882								
6	7E+07	6_693854	14	60	1	12	37	7	2.66E-06	0.00037	0	22	16	28	11	22	0.10262264	1	19.125	19.0445905								
6	7E+07	6_69431057	1418	500	678	438	959	558	6.11E-40	#######	667	270	242	101	469	172	5.83E-26	5.37E-23	539.25	409.26441					6.9E+07	6.9E+07	0.21713	ID=promoter_44424;
6	7E+07	6_69548070	16	18	17	24	17	21	0.98748	1	17	5	20	4	11	12	0.92663959	1	15.125	7.01910658	7E+07	7E+07	ID=ENSP0000343158;EPB41L3;NM_012307;	EPB41L3				
6	7E+07	6_705227	0	41	8	16	21	12	0.1273	1	28	18	20	0	23	10	0.12305918	1	16.375	13.9584844								
6	7E+07	6_70932086	117	46	83	54	82	69	0.124202	1	2	1	11	0	2	6	0.31385293	1	39.25	43.7713213					7.1E+07	7.1E+07	0.20975	ID=promoter_44438;
6	7E+07	6_710183	0	0	0	0	0	0	1	1	50	28	19	1	39	10	5.37E-04	0.0374204	12.25	18.691098								
6	7E+07	6_711186	38	71	28	36	55	32	0.005632	0.25889	15	17	26	30	16	28	0.11940283	1	32.625	17.4841765								
6	7E+07	6_718693	0	16	15	14	8	15	0.557319	1	0	4	3	3	2	3	0.86792893	1	6.875	6.89590976								

6	7E+07	6_72280219	6	9	5	1	8	3	0.206144	1	11	10	16	27	11	22	0.10262264	1	10.625	7.98100423					7.2E+07	7.2E+07	0.15552	ID=promoter_44456;
6	7E+07	6_731687	8	24	14	12	16	13	0.613293	1	93	65	130	33	79	82	0.77315304	1	47.375	44.5419144								
6	7E+07	6_738177	14	73	14	21	44	18	4.24E-04	0.03084	42	21	55	26	32	41	0.38704929	1	33.25	21.4592637								
6	7E+07	6_738759	333	81	229	214	207	222	0.65054	1	16	93	94	67	55	81	0.0280935	0.8894169	140.88	106.335638								
6	7E+07	6_74164748	24	87	14	37	56	26	2.78E-04	0.02161	10	82	79	48	46	64	0.10686069	1	47.625	31.473061	7E+07	7.4E+07	ID=ENSP00000324532;LAMA3;NM_198129;	LAMA3				
6	7E+07	6_742461	0	8	3	4	4	4	0.97482	1	0	4	11	24	2	18	8.85E-04	0.0572814	6.75	7.90569415								
6	8E+07	6_753371	0	1	0	5	1	3	0.700657	1	1	0	0	33	1	17	4.35E-04	0.0314643	5	11.4392807								
6	8E+07	6_755407	22	40	16	48	31	32	0.879223	1	353	149	161	113	251	137	2.97E-06	0.0004381	112.75	112.404563								
6	8E+07	6_756087	5	15	17	9	10	13	0.970528	1	61	32	37	20	47	29	0.2216313	1	24.5	18.2522014								
6	8E+07	6_756919	2	10	14	2	6	8	0.9722	1	0	11	7	38	6	23	0.00349909	0.1735359	10.5	12.1655251								
6	8E+07	6_758991	0	11	5	6	6	6	0.962469	1	19	0	0	8	10	4	0.34296513	1	6.125	6.6211242								
6	8E+07	6_765947	19	59	27	11	39	19	0.00521	0.24279	74	51	56	1	63	29	0.00629125	0.2727856	37.25	26.201145								
6	8E+07	6_767518	33	24	8	10	29	9	8.94E-04	0.05763	21	6	3	23	14	13	0.99999086	1	16	10.6636901								
6	8E+07	6_769027	9	12	18	43	11	31	0.036004	1	6	23	34	40	15	37	0.00395829	0.1883294	23.125	14.3471003								
6	8E+07	6_769878	69	10	4	14	40	9	3.70E-06	0.00049	1	16	7	0	9	4	0.4571786	1	15.125	22.516264								
6	8E+07	6_772624	115	103	91	97	109	94	0.090143	1	19	55	37	13	37	25	0.47119089	1	66.25	40.2412368								
6	8E+07	6_781629	30	81	24	7	56	16	4.49E-07	#######	123	29	47	78	76	63	0.80600176	1	52.375	38.5521447								
6	8E+07	6_787795	29	69	66	66	49	66	0.763834	1	201	55	52	74	128	63	1.59E-04	0.0135699	76.5	52.2384642								
6	8E+07	6_787991	27	85	13	52	56	33	0.005995	0.27181	57	80	82	67	69	75	0.63380937	1	57.875	26.4382704								
6	8E+07	6_788933	1	0	0	4	1	2	0.907382	1	25	4	6	24	15	15	0.97869956	1	8	10.4060422								
6	8E+07	6_795732	5	4	1	3	5	2	0.402696	1	49	33	9	1	41	5	2.83E-06	0.0004196	13.125	17.907999								
6	8E+07	6_79663850	26	62	15	10	44	13	1.44E-05	0.00165	39	6	19	1	23	10	0.12305918	1	22.25	20.0124961	8E+07	8E+07	ID=ENSP00000257198-D2;DSC1;NM_024421;	DSC1				
6	8E+07	6_79677363	9	14	19	30	12	25	0.252846	1	1	0	28	12	1	20	8.76E-05	0.0082396	14.125	11.1539551	8E+07	8E+07	ID=ENSP00000257198-D2;DSC1;NM_024421;	DSC1				
6	8E+07	6_80797140	4	18	6	6	11	6	0.3028	1	76	1	26	0	39	13	0.00384532	0.1844291	17.125	25.4077463	8E+07	8.1E+07	ID=ENSP00000382165;FAM59A;;	FAM59A				
6	8E+07	6_80866600	0	47	13	18	24	16	0.207334	1	13	1	9	3	7	6	0.99011599	1	13	15.1657509					8.1E+07	8.1E+07	0.13129	ID=promoter_44532;
6	8E+07	6_80867027	52	50	47	78	51	63	0.962908	1	79	20	40	13	50	27	0.07256683	1	47.375	23.7603361					8.1E+07	8.1E+07	0.13129	ID=promoter_44532;
6	8E+07	6_81108757	91	55	133	67	73	100	0.601024	1	31	42	37	17	37	27	0.63815857	1	59.125	37.6086521					8.1E+07	8.1E+07	0.44782	ID=promoter_44533;
6	8E+07	6_813650	176	250	189	319	213	254	0.984322	1	134	194	325	250	164	288	2.63E-10	8.29E-08	229.63	68.5084718								
6	8E+07	6_81566358	6	15	0	0	11	0	0.001579	0.09256	11	19	14	0	15	7	0.31064188	1	8.125	7.66135945	8E+07	8.2E+07	ID=ENSP00000269197;ASXL3;NM_030632;	ASXL3				
6	8E+07	6_816688	44	36	28	23	40	26	0.059242	1	0	18	0	0	9	0	0.01542606	0.5535504	18.625	17.2952306								
6	8E+07	6_824635	181	356	139	182	269	161	5.25E-11	#######	368	469	419	205	419	312	0.01343113	0.496694	289.88	126.881764								
6	8E+07	6_824848	130	70	90	42	100	66	0.001163	0.07164	25	40	20	33	33	27	0.89046553	1	56.25	37.9200286								
6	8E+07	6_825333	1234	1357	2149	2161	1296	2155	2.39E-22	#######	373	306	1419	508	340	964	2.43E-76	6.55E-73	1188.4	742.470382								
6	8E+07	6_82838355	500	217	473	373	359	423	0.998014	1	232	134	219	116	183	168	0.99583031	1	283	147.566934					8.3E+07	8.3E+07	0.38939	ID=promoter_44540;
6	8E+07	6_829504	5	39	16	15	22	16	0.340123	1	28	39	32	6	34	19	0.20290377	1	22.5	13.8460928								
6	8E+07	6_835149	60	25	52	15	43	34	0.224092	1	0	18	24	0	9	12	0.70935077	1	24.25	21.8746429								
6	8E+07	6_836977	1	21	31	16	11	24	0.223145	1	21	32	22	3	27	13	0.14207	1	18.375	11.4385002								
6	8E+07	6_840432	3	17	1	0	10	1	0.011548	0.45484	9	5	6	1	7	4	0.73798468	1	5.25	5.62519841								
6	8E+07	6_844505	0	17	0	7	9	4	0.244237	1	26	0	12	0	13	6	0.35360803	1	7.75	9.83797308								
6	8E+07	6_847417	20	81	40	34	51	37	0.080244	1	26	37	44	14	32	29	0.99609288	1	37	20.5008711								
6	9E+07	6_853018	21	21	15	2	21	9	0.03105	0.99404	57	17	20	44	37	32	0.95947178	1	24.625	17.4596474								
6	9E+07	6_859935	15	9	19	30	12	25	0.252846	1	39	29	34	0	34	17	0.10758511	1	21.875	13.3997601								
6	9E+07	6_866156	2	22	10	4	12	7	0.325249	1	18	0	7	0	9	4	0.4571786	1	7.875	8.28833087								
6	9E+07	6_873316	9	0	8	0	5	4	0.848147	1	9	0	26	0	5	13	0.12287893	1	6.5	8.9761589								
6	9E+07	6_874473	0	22	5	20	11	13	0.999831	1	14	5	11	0	10	6	0.69729498	1	9.625	8.53459347								
6	9E+07	6_879274	2	13	6	7	8	7	0.850897	1	9	23	17	13	16	15	0.99951315	1	11.25	6.69221296								

6	9E+07	6_891690	7	2	6	8	5	7	0.955252	1	2	4	7	30	3	19	0.0015822	0.0898971	8.25	9.08295106								
6	9E+07	6_893006	9	15	0	8	12	4	0.066899	1	2	7	17	0	5	9	0.48000906	1	7.25	6.4531277								
6	9E+07	6_897443	0	0	16	0	0	8	0.033035	1	0	1	13	24	1	19	1.49E-04	0.0128238	6.75	9.54313515								
6	9E+07	6_920937	0	4	5	10	2	8	0.255957	1	0	26	0	1	13	1	0.00904697	0.3617342	5.75	8.89221168								
6	9E+07	6_927924	31	65	12	33	48	23	0.001283	0.07728	19	89	44	30	54	37	0.36386527	1	40.375	25.3430041								
6	9E+07	6_928166	25	19	15	12	22	14	0.192025	1	27	6	11	8	17	10	0.5154613	1	15.375	7.68927825								
6	9E+07	6_928504	39	25	32	15	32	24	0.247982	1	31	11	29	2	21	16	0.83919704	1	23	12.478553								
6	9E+07	6_942380	19	21	13	22	20	18	0.715835	1	69	51	9	41	60	25	0.00260517	0.1361046	30.625	20.9621257								
6	9E+07	6_94377293	0	23	1	1	12	1	0.003675	0.18279	0	1	15	9	1	12	0.00617711	0.2683255	6.25	8.66437699	9E+07	9.4E+07	ID=ENSP00000333938;NEXN;NM_144573;	NEXN				
6	9E+07	6_947158	11	27	24	5	19	15	0.513973	1	0	1	0	2	1	1	0.99856566	1	8.75	10.9772492								
6	9E+07	6_949986	155	482	163	234	319	199	1.08E-11	#######	504	419	617	197	462	407	0.75470206	1	346.38	180.057084								
6	1E+08	6_950263	3	27	1	10	15	6	0.065303	1	57	28	27	3	43	15	0.00318797	0.1603338	19.5	19.1535152								
6	1E+08	6_95292192	200	50	152	109	125	131	0.666159	1	42	70	64	2	56	33	0.11440163	1	86.125	64.2638256					9.5E+07	9.5E+07	0.49383	ID=promoter_44583;
6	1E+08	6_959646	13	34	24	19	24	22	0.704268	1	1	23	32	32	12	32	0.00478712	0.2203477	22.25	11.2090525								
6	1E+08	6_959992	16	24	6	3	20	5	0.002998	0.15536	31	0	2	1	16	2	0.00719486	0.3031284	10.375	11.8434732								
6	1E+08	6_963141	31	166	32	43	99	38	5.66E-09	#######	47	60	80	49	54	65	0.3636418	1	63.5	44.307368								
6	1E+08	6_986622	124	109	147	173	117	160	0.450462	1	220	206	287	47	213	167	0.2679627	1	164.13	74.3667312								
6	1E+08	6_100079620	48	60	23	23	54	23	1.22E-04	0.01067	31	20	38	79	26	59	4.41E-04	0.031866	40.25	20.8652137								
6	1E+08	6_100964281	15	21	17	5	18	11	0.22238	1	0	9	27	9	5	18	0.01516798	0.5480081	12.875	8.82265752								
6	1E+08	6_101223672	4	8	5	2	6	4	0.674789	1	0	0	2	25	0	14	5.26E-04	0.0367435	5.75	8.22452778								
6	1E+08	6_101356951	25	11	12	9	18	11	0.22238	1	2	13	8	1	8	5	0.78486764	1	10.125	7.45342682								
6	1E+08	6_102235132	41	20	43	40	31	42	0.830569	1	0	27	18	40	14	29	0.03997116	1	28.625	15.2497073	1E+08	1E+08	ID=ENSP00000262345;IL12RB2;NM_001559;	IL12RB2				
6	1E+08	6_102261596	9	47	32	13	28	23	0.444971	1	3	42	30	17	23	24	0.92099721	1	24.125	15.986043	1E+08	1E+08	ID=ENSP00000262345;IL12RB2;NM_001559;	IL12RB2				
6	1E+08	6_102599126	10	13	14	21	12	18	0.803442	1	2	21	33	2	12	18	0.42746936	1	14.5	10.4334626								
6	1E+08	6_102607119	9	0	3	10	5	7	0.955252	1	31	0	9	1	16	5	0.08348313	1	7.875	10.2600125								
6	1E+08	6_102944153	1576	1170	1857	2049	1373	1953	2.95E-07	#######	1138	838	2078	625	988	1352	9.21E-20	6.23E-17	1416.4	555.051848								
6	1E+08	6_103543823	27	32	11	9	30	10	0.001158	0.07164	4	17	27	6	11	17	0.4100993	1	16.625	10.7827574	1E+08	1E+08	ID=ENSP00000360108;DNAJC6;;	DNAJC6				
6	1E+08	6_103642739	852	219	645	622	536	634	0.989831	1	203	202	260	101	203	181	0.92996555	1	388	275.742116					1E+08	1E+08	0.54671	ID=promoter_44630;
6	1E+08	6_103881610	121	74	105	36	98	71	0.007658	0.32964	65	51	64	258	58	161	5.03E-13	2.17E-10	96.75	70.7505679					1E+08	1E+08	0.61414	ID=promoter_44635;
6	1E+08	6_103984554	12	33	14	19	23	17	0.346113	1	30	20	7	41	25	24	0.99255599	1	22	11.6373782								
6	1E+08	6_103985372	16	6	18	11	11	15	0.930409	1	51	30	20	6	41	13	0.00193653	0.1067879	19.75	14.8972672								
6	1E+08	6_104000668	4	29	16	13	17	15	0.722447	1	14	16	42	8	15	25	0.19020812	1	17.75	12.197775								
6	1E+08	6_104263230	42	82	17	24	62	21	1.08E-06	0.00016	116	34	50	34	75	42	0.03039885	0.9417302	49.875	33.211175								
6	1E+08	6_104521362	9	11	55	34	10	45	1.88E-04	0.01539	26	6	9	0	16	5	0.08348313	1	18.75	18.3750607								
6	1E+08	6_104720021	0	36	0	9	18	5	0.008132	0.34618	31	2	7	1	17	4	0.02867352	0.9010784	10.75	14.4790489								
6	1E+08	6_104990435	12	21	6	5	17	6	0.027279	0.89953	7	18	9	0	13	5	0.22538616	1	9.75	6.96419414								
6	1E+08	6_105311843	23	57	9	10	40	10	8.99E-06	0.00109	9	25	39	27	17	33	0.04051129	1	24.875	16.7539974								
6	1E+08	6_105648694	11	12	14	7	12	11	0.839207	1	2	17	5	5	10	5	0.51906437	1	9.125	5.16685868								
6	1E+08	6_105750357	2	20	10	1	11	6	0.3028	1	0	10	0	0	5	0	0.09850879	1	5.375	7.30826342								

6	1E+08	6_106098712	0	15	9	4	8	7	0.850897	1	21	15	9	19	18	14	0.88543974	1	11.5	7.28991476								
6	1E+08	6_106111191	1361	517	848	943	939	896	5.54E-05	0.0053	385	384	328	146	385	237	3.85E-06	0.0005454	614	402.949128					1.1E+08	1.1E+08	0.746	ID=promoter_44656;
6	1E+08	6_106181288	82	16	20	50	49	35	0.07705	1	38	41	38	16	40	27	0.44121895	1	37.625	21.9736043								
6	1E+08	6_106246855	21	39	17	26	30	22	0.240296	1	53	10	16	5	32	11	0.01265194	0.4751394	23.375	15.7927425								
6	1E+08	6_107221987	420	100	296	185	260	241	0.030759	0.98759	48	79	113	81	64	97	0.00862117	0.3495868	165.25	129.645395								
6	1E+08	6_108136154	17	6	0	6	12	3	0.030625	0.98374	34	21	18	15	28	17	0.38227241	1	14.625	10.6628527	1E+08	1.1E+08	ID=ENSP000003602 62;FGGY;NM_001113411;	FGGY				
6	1E+08	6_108277136	10	1	10	1	6	6	0.962469	1	0	0	11	17	0	14	5.26E-04	0.0367435	6.25	6.54107898	1E+08	1.1E+08	ID=ENSP000003602 62;FGGY;NM_001113411;	FGGY				
6	1E+08	6_108328192	62	29	104	121	46	113	8.73E-05	0.00787	159	111	58	20	135	39	1.02E-10	3.41E-08	83	48.3971664								
6	1E+08	6_108481923	37	36	46	33	37	40	0.938033	1	0	21	14	21	11	18	0.32230807	1	26	14.832397								
6	1E+08	6_108496515	990	941	1302	1251	966	1277	0.019511	0.69334	291	324	353	172	308	263	0.61912866	1	703	465.366829								
6	1E+08	6_108630252	469	133	305	229	301	267	0.003856	0.19063	182	39	104	63	111	84	0.37224664	1	190.5	142.54022								
6	1E+08	6_108831297	182	52	131	137	117	134	0.982377	1	28	20	41	2	24	22	0.99927301	1	74.125	66.1933046								
6	1E+08	6_108950297	22	32	5	5	27	5	7.68E-05	0.00707	5	14	22	53	10	38	9.38E-05	0.0087137	19.75	16.6968774								
6	1E+08	6_109075818	38	30	26	9	34	18	0.01975	0.69938	39	26	61	25	33	43	0.33537098	1	31.75	15.0214133								
6	1E+08	6_109162241	179	68	158	122	124	140	0.953234	1	75	17	70	49	46	60	0.21594777	1	92.25	55.6102509	1E+08	1.1E+08	ID=ENSP000003602 69;TACSTD2;NM_002353;	TACSTD2	1.1E+08	1.1E+08	0.6419	ID=promoter_44689;
6	1E+08	6_109465745	12	24	4	0	18	2	4.15E-04	0.03028	24	1	16	0	13	8	0.65599869	1	10.125	10.3432172								
6	1E+08	6_109516310	7	0	7	10	4	9	0.54411	1	17	11	4	0	14	2	0.01719769	0.6064547	7	5.75698334								
6	1E+08	6_109850069	8	19	17	18	14	18	0.967347	1	20	4	5	2	12	4	0.18067673	1	11.625	7.57698583								
6	1E+08	6_110158415	40	7	17	33	24	25	0.917255	1	3	0	2	2	2	2	0.99713337	1	13	15.5471633	1E+08	1.1E+08	ID=ENSP000003602 79;DAB1;;	DAB1				
6	1E+08	6_110202276	2005	1323	2254	1610	1664	1932	0.954611	1	1561	934	1017	396	1248	707	5.53E-25	4.87E-22	1387.5	602.60174	1E+08	1.1E+08	ID=ENSP000003602 79;DAB1;;	DAB1				
6	1E+08	6_110943521	42	34	15	4	38	10	2.46E-05	0.00262	13	58	28	2	36	15	0.02815151	0.8902666	24.5	19.5667356								
6	1E+08	6_111846713	35	59	20	31	47	26	0.007051	0.30636	41	32	71	50	37	61	0.01977671	0.6742538	42.375	16.6813626								
6	1E+08	6_112208788	128	6	52	74	67	63	0.450792	1	42	11	13	63	27	38	0.24763409	1	48.625	40.8968651					1.1E+08	1.1E+08	0.38597	ID=promoter_44707;
6	1E+08	6_112452743	10	30	27	19	20	23	0.997913	1	18	33	6	2	26	4	6.99E-04	0.0468789	18.125	11.4322539								
6	1E+08	6_112815101	686	673	1592	1786	680	1689	8.07E-63	#######	58	39	116	58	49	87	0.00105442	0.065658	626	710.581452	1E+08	1.1E+08	ID=ENSP000003036 34;LRP8;NM_004631;	LRP8	1.1E+08	1.1E+08	0.13186	ID=promoter_44733;
6	1E+08	6_112848788	7	5	7	5	6	6	0.962469	1	70	14	25	0	42	13	0.00136526	0.0810207	16.625	22.8594307	1E+08	1.1E+08	ID=ENSP000003036 34;LRP8;NM_004631;	LRP8				

6	1E+08	6_112936178	7	23	0	4	15	2	0.002248	0.12273	2	0	0	14	1	7	0.08320466	1	6.25	8.29371535	1E+08	1.1E+08	ID=ENSP000003605 41;CPT2; NM_000098;	CPT2				
6	1E+08	6_112974739	90	35	44	31	63	38	0.004347	0.20961	102	90	57	0	96	29	1.80E-07	3.58E-05	56.125	35.4055585								
6	1E+08	6_113014499	27	16	17	44	22	31	0.804879	1	26	1	6	27	14	17	0.75475782	1	20.5	13.5751874	1E+08	1.1E+08	ID=ENSP000003605 49;SLC1A 7;NM_006 671;	SLC1A7				
6	1E+08	6_113043424	45	10	19	25	28	22	0.367037	1	6	16	24	8	11	16	0.51039395	1	19.125	12.6314517								
6	1E+08	6_113599968	471	298	359	311	385	335	3.20E-04	0.02446	209	257	117	84	233	101	5.31E-10	1.63E-07	263.25	126.670269					1.1E+08	1.1E+08	0.32982	ID=promoter_44755;
6	1E+08	6_114202711	69	72	47	74	71	61	0.201291	1	58	95	54	14	77	34	0.00112478	0.0691941	60.375	23.8143864	1E+08	1.1E+08	ID=ENSP000003372 65;OSBPL 9;;	OSBPL9				
6	1E+08	6_115304767	12	9	2	0	11	1	0.006522	0.28949	2	15	19	0	9	10	0.92505409	1	7.375	7.40535521	1E+08	1.2E+08	ID=ENSP000003794 57;FAF1; NM_007051;	FAF1				
6	1E+08	6_115430674	7	11	6	10	9	8	0.848833	1	25	3	35	1	14	18	0.65378371	1	12.25	11.7442995								
6	1E+08	6_115491799	443	249	496	404	346	450	0.351965	1	81	111	109	35	96	72	0.39558322	1	241	183.155671								
6	1E+08	6_116151744	19	9	4	3	14	4	0.02589	0.86655	0	0	8	2	0	5	0.06742467	1	5.625	6.34569595								
6	1E+08	6_116413703	18	29	31	37	24	34	0.777678	1	23	24	12	8	24	10	0.09254106	1	22.75	9.7650982								
6	1E+08	6_116519348	21	4	7	11	13	9	0.46931	1	16	4	5	11	10	8	0.95278883	1	9.875	6.15136454					1.2E+08	1.2E+08	0.1586	ID=promoter_44791;
6	1E+08	6_116554772	28	17	24	19	23	22	0.790781	1	21	17	15	40	19	28	0.28999502	1	22.625	8.1929848								
6	1E+08	6_116670874	208	1070	301	316	639	309	1.27E-38	#######	852	459	795	540	656	668	0.23232073	1	567.63	307.34947								
6	1E+08	6_117032004	79	17	67	82	48	75	0.2977	1	11	25	49	13	18	31	0.10487282	1	42.875	30.1256298								
6	1E+08	6_117148104	20	40	11	11	30	11	0.002281	0.12403	5	3	18	2	4	10	0.21760186	1	13.75	12.4871362					1.2E+08	1.2E+08	0.19989	ID=promoter_44817;
6	1E+08	6_117208919	66	31	34	40	49	37	0.125191	1	1	45	12	22	23	17	0.77734386	1	31.375	20.1844176					1.2E+08	1.2E+08	0.14245	ID=promoter_44825;
6	1E+08	6_117244542	5	27	2	4	16	3	0.003782	0.18716	32	20	14	1	26	8	0.01635039	0.5814583	13.125	12.0527116								
6	1E+08	6_117316300	115	50	152	181	83	167	2.56E-04	0.02007	115	31	57	7	73	32	0.0014486	0.0855753	88.5	61.4305648					1.2E+08	1.2E+08	0.19096	ID=promoter_44843;
6	1E+08	6_117325196	6	26	36	59	16	48	0.003374	0.17222	65	29	35	38	47	37	0.75787008	1	36.75	18.5914727					1.2E+08	1.2E+08	0.16197	ID=promoter_44845;
6	1E+08	6_117330528	4	7	5	8	6	7	0.999953	1	15	0	16	12	8	14	0.34554715	1	8.375	5.5789784								
6	1E+08	6_117345314	14	38	24	16	26	20	0.360218	1	2	19	10	9	11	10	0.99899888	1	16.5	10.9544512								
6	1E+08	6_117421671	57	44	69	61	51	65	0.906125	1	10	0	0	31	5	16	0.03625394	1	34	27.9795844								
6	1E+08	6_117533880	122	187	108	125	155	117	0.001385	0.08272	9	69	110	52	39	81	1.19E-04	0.0105864	97.75	53.914615					1.2E+08	1.2E+08	0.47557	ID=promoter_44863;
6	1E+08	6_117606085	0	12	3	0	6	2	0.258649	1	14	9	1	3	12	2	0.04045894	1	5.25	5.59974489								
6	1E+08	6_117859335	1	14	7	5	8	6	0.705675	1	6	10	8	1	8	5	0.78486764	1	6.5	4.37525509								
6	1E+08	6_117897951	34	52	22	17	43	20	0.001895	0.10739	47	56	54	12	52	33	0.23056449	1	36.75	17.8625706								
6	1E+08	6_117999591	14	47	37	34	31	36	0.999169	1	36	59	58	29	48	44	0.99854654	1	39.25	15.0594062	1E+08	1.2E+08	ID=ENSP000003610 14;MKNK1 ;NM_003684;	MKNK1				

6	1E+08	6_118011387	217	39	87	64	128	76	1.03E-05	0.00123	94	83	98	4	89	51	0.02183351	0.7351944	85.75	61.870256								
6	1E+08	6_118077247	135	39	178	166	87	172	3.18E-04	0.02435	85	56	50	51	71	51	0.37870966	1	95	56.3762868					1.2E+08	1.2E+08	0.23189	ID=promoter_44889;
6	1E+08	6_118102321	82	94	371	406	88	389	2.01E-32	#######	88	31	114	78	60	96	0.00356478	0.1765486	158	144.474417								
6	1E+08	6_118113862	6	6	4	0	6	2	0.258649	1	23	11	36	0	17	18	0.92557631	1	10.75	12.5896783								
6	1E+08	6_118123496	58	6	27	18	32	23	0.194505	1	0	6	5	2	3	4	0.89184014	1	15.25	19.484426	1E+08	1.2E+08	ID=ENSP00000243167;FAAH;NM_001441;	FAAH				
6	1E+08	6_118256785	3	2	0	0	3	0	0.172143	1	29	1	9	0	15	5	0.11779525	1	5.5	9.95705062	1E+08	1.2E+08	ID=ENSP00000361048;Clorf190;NM_001013615;	Clorf190				
6	1E+08	6_118262477	213	39	161	88	126	125	0.413854	1	50	23	22	0	37	11	0.00224181	0.1207582	74.5	75.2766327	1E+08	1.2E+08	ID=ENSP00000361048;Clorf190;NM_001013615;	Clorf190				
6	1E+08	6_118273675	34	6	9	24	20	17	0.618382	1	14	1	3	1	8	2	0.20550608	1	11.5	11.9403278								
6	1E+08	6_118342312	56	55	43	102	56	73	0.837949	1	48	52	12	32	50	22	0.01176819	0.4476014	50	25.6069187								
6	1E+08	6_118361861	14	15	23	22	15	23	0.720508	1	25	4	14	2	15	8	0.44137572	1	14.875	8.49264388								
6	1E+08	6_118379271	0	6	0	5	3	3	0.981055	1	19	4	3	4	12	4	0.18067673	1	5.125	6.01040764	1E+08	1.2E+08	ID=ENSP00000361048-D2;Clorf190;NM_001013615;	Clorf190				
6	1E+08	6_118502270	24	16	16	33	20	25	0.977759	1	36	14	34	4	25	19	0.80752973	1	22.125	11.4945639					1.2E+08	1.2E+08	0.29024	ID=promoter_44931;
6	1E+08	6_118517886	6	10	27	6	8	17	0.371907	1	0	8	22	0	4	11	0.14780855	1	9.875	9.77515071					1.2E+08	1.2E+08	0.23383	ID=promoter_44937;
6	1E+08	6_118523575	71	131	32	50	101	41	1.50E-08	#######	110	72	108	81	91	95	0.72020747	1	81.875	32.9650789								
6	1E+08	6_118578702	22	59	21	8	41	15	2.40E-04	0.01893	2	16	33	24	9	29	0.00234316	0.1251016	23.125	17.3899602								
6	1E+08	6_118779979	24	10	15	13	17	14	0.615566	1	141	34	8	41	88	25	2.31E-07	4.49E-05	35.75	44.1223947	1E+08	1.2E+08	ID=ENSP00000361152;PRDX1;NM_181697;	PRDX1				
6	1E+08	6_119244792	1	3	20	5	2	13	0.039726	1	21	19	24	20	20	22	0.85756598	1	14.125	9.38749168								
6	1E+08	6_119272137	2	20	11	0	11	6	0.3028	1	57	36	23	25	47	24	0.05449507	1	21.75	18.6681547	1E+08	1.2E+08	ID=ENSP00000335580;RNF220;;	RNF220	1.2E+08	1.2E+08	0.2386	ID=promoter_44965;
6	1E+08	6_119296702	2	5	1	9	4	5	0.995512	1	2	13	7	2	8	5	0.78486764	1	5.125	4.25734659								
6	1E+08	6_119307561	15	3	6	1	9	4	0.244237	1	1	4	19	23	3	21	5.76E-04	0.0398271	9	8.70139562					1.2E+08	1.2E+08	0.19043	ID=promoter_44971;
6	1E+08	6_119344095	19	15	97	93	17	95	4.87E-10	#######	7	7	40	41	7	41	1.54E-06	0.000244	39.875	36.4552073	1E+08	1.2E+08	ID=ENSP00000386506;;;		1.2E+08	1.2E+08	0.54973	ID=promoter_44974;
6	1E+08	6_119356228	397	206	307	276	302	292	0.062084	1	114	101	139	38	108	89	0.7127769	1	197.25	121.403401					1.2E+08	1.2E+08	0.46613	ID=promoter_44976;
6	1E+08	6_119497675	10	12	2	6	11	4	0.10516	1	59	17	14	16	38	15	0.01554903	0.5562856	17	17.703914								
6	1E+08	6_119522700	28	27	42	28	28	35	0.969001	1	430	212	743	246	321	495	6.49E-12	2.45E-09	219.5	256.634259					1.2E+08	1.2E+08	0.23342	ID=promoter_44996;

6	1E+08	6_119578898	43	29	16	24	36	20	0.023274	0.79554	0	11	24	0	6	12	0.2888538	1	18.375	14.7254639								
6	1E+08	6_119583296	0	7	1	2	4	2	0.597627	1	28	6	6	12	17	9	0.38755874	1	7.75	9.0514403								
6	1E+08	6_119628060	18	39	12	7	29	10	0.001829	0.10386	0	18	23	17	9	20	0.07989336	1	16.75	11.5604004								
6	1E+08	6_119690696	11	74	1	13	43	7	1.03E-07	#######	16	27	27	31	22	29	0.4569972	1	25	22.1746316					1.2E+08	1.2E+08	0.10393	ID=promoter_45026;
6	1E+08	6_119749982	11	7	21	6	9	14	0.802715	1	11	33	10	2	22	6	0.01847254	0.6380171	12.625	9.89859297					1.2E+08	1.2E+08	0.29533	ID=promoter_45035;
6	1E+08	6_119757516	305	236	276	225	271	251	0.025949	0.86829	347	317	186	115	332	151	1.20E-12	4.97E-10	250.88	76.2841259					1.2E+08	1.2E+08	0.89194	ID=promoter_45036;
6	1E+08	6_119782449	4	0	0	5	2	3	0.964182	1	12	13	9	9	13	9	0.79589064	1	6.5	5.04267503								
6	1E+08	6_119820761	0	3	2	1	2	2	0.98733	1	11	21	3	12	16	8	0.35023049	1	6.625	7.3472541	1E+08	1.2E+08	ID=ENSP0000361331;ERI3;NM_024066;	ERI3				
6	1E+08	6_119848995	13	15	12	12	14	12	0.725787	1	51	14	26	5	33	16	0.09595749	1	18.5	14.3527001								
6	1E+08	6_119878874	32	30	51	67	31	59	0.088267	1	50	25	29	21	38	25	0.40838944	1	38.125	16.0039058								
6	1E+08	6_119886269	14	0	11	3	7	7	0.956352	1	2	10	5	9	6	7	0.91781435	1	6.75	4.94974747								
6	1E+08	6_119911408	31	55	18	24	43	21	0.003115	0.16067	10	10	23	15	10	19	0.1719433	1	23.25	14.713939								
6	1E+08	6_119937890	46	55	58	67	51	63	0.962908	1	86	59	44	24	73	34	0.00329337	0.1649373	54.875	18.0984411					1.2E+08	1.2E+08	0.15389	ID=promoter_45068;
6	1E+08	6_119949882	1	3	8	8	2	8	0.255957	1	15	13	5	1	14	3	0.04246461	1	6.75	5.25764749								
6	1E+08	6_119965950	576	609	605	1106	593	856	5.14E-04	0.0361	674	1058	1968	590	866	1279	1.73E-25	1.54E-22	898.25	482.827609								
6	1E+08	6_119969886	153	84	138	113	119	126	0.725469	1	39	37	30	23	38	27	0.5702351	1	77.125	52.1137149								
6	1E+08	6_119979837	118	42	101	95	80	98	0.959333	1	15	13	0	38	14	19	0.55289753	1	52.75	45.4996075					1.2E+08	1.2E+08	0.25429	ID=promoter_45080;
6	1E+08	6_119993108	44	19	30	12	32	21	0.11093	1	0	3	23	16	2	20	3.12E-04	0.0239121	18.375	14.3122076								
6	1E+08	6_120023828	2	16	7	3	9	5	0.390586	1	4	4	17	4	4	11	0.14780855	1	7.125	5.96268156								
6	1E+08	6_120033930	18	32	30	20	25	25	0.852664	1	25	6	57	45	16	51	2.67E-05	0.0029507	29.125	16.0217486								
6	1E+08	6_120066951	2	16	12	12	9	12	0.95859	1	19	2	1	36	11	19	0.24832023	1	12.5	11.7108009					1.2E+08	1.2E+08	0.72338	ID=promoter_45093;
6	1E+08	6_120072414	2	0	22	4	1	13	0.014136	0.5348	36	1	8	1	19	5	0.02799778	0.8875672	9.25	12.9917556					1.2E+08	1.2E+08	0.17872	ID=promoter_45095;
6	1E+08	6_120103410	1	11	11	2	6	7	0.999953	1	11	0	10	7	6	9	0.65381141	1	6.625	4.86789776					1.2E+08	1.2E+08	0.13131	ID=promoter_45098;
6	1E+08	6_120131423	104	61	112	64	83	88	0.803988	1	81	40	39	34	61	37	0.12204036	1	66.875	29.849324					1.2E+08	1.2E+08	0.49714	ID=promoter_45104;
6	1E+08	6_120220316	16	9	13	7	13	10	0.600182	1	7	3	4	9	5	7	0.77775394	1	8.5	4.34248119								
6	1E+08	6_120262120	15	58	29	16	37	23	0.052004	1	66	21	124	3	44	64	0.06786129	1	41.5	39.8030867								
6	1E+08	6_120299775	52	39	120	45	46	83	0.061575	1	412	336	366	251	374	309	0.32523247	1	202.63	156.657896								
6	1E+08	6_120313924	0	4	10	10	2	10	0.124792	1	0	11	5	0	6	3	0.67473421	1	5	4.81070235	1E+08	1.2E+08	ID=ENSP0000321175-D2;;		1.2E+08	1.2E+08	0.24563	ID=promoter_45125;
6	1E+08	6_120350695	5	28	10	11	17	11	0.29617	1	21	2	20	2	12	11	0.99963644	1	12.375	9.66492185								
6	1E+08	6_120364647	0	2	1	9	1	5	0.353258	1	24	14	20	16	19	18	0.99782856	1	10.75	9.17683419	1E+08	1.2E+08	ID=ENSP0000296394-D3;WDR65;NM_152498;	WDR65				

6	1E+08	6_120390731	1237	540	1013	686	889	850	1.11E-04	0.0098	515	339	377	182	427	280	3.58E-05	0.0037948	611.13	355.797469	1E+08	1.2E+08	ID=ENSP000002360 51;EBNA1BP2;NM_006824;	EBNA1BP2	1.2E+08	1.2E+08	0.51368	ID=promoter_45133;
6	1E+08	6_120735364	13	26	15	4	20	10	0.076235	1	0	17	7	2	9	5	0.6500913	1	10.5	8.83176087								
6	1E+08	6_120801838	9	11	2	31	10	17	0.644969	1	52	46	65	17	49	41	0.88927407	1	29.125	23.0368494								
6	1E+08	6_120888102	11	53	7	12	32	10	4.55E-04	0.03273	25	19	20	25	22	23	0.92188352	1	21.5	14.322809								
6	1E+08	6_120900187	14	4	27	19	9	23	0.127121	1	23	17	21	3	20	12	0.48622029	1	16	8.63547501								
6	1E+08	6_120935537	30	72	42	98	51	70	0.694167	1	401	150	32	251	276	142	5.43E-08	1.18E-05	134.5	130.961281					1.2E+08	1.2E+08	0.14738	ID=promoter_45158;
6	1E+08	6_120998121	33	23	20	66	28	43	0.538256	1	0	8	13	3	4	8	0.43697044	1	20.75	21.2854746	1E+08	1.2E+08	ID=ENSP000003403 78;;NM_001080850 ;					
6	1E+08	6_121001039	103	128	129	126	116	128	0.892643	1	43	54	35	83	49	59	0.39841053	1	87.625	39.5761021	1E+08	1.2E+08	ID=ENSP000003403 78;;NM_001080850 ;		1.2E+08	1.2E+08	0.1905	ID=promoter_45166;
6	1E+08	6_121098622	62	21	42	50	42	46	0.9497	1	24	15	29	6	20	18	0.99585638	1	31.125	18.8106163								
6	1E+08	6_121115740	1	19	14	6	10	10	0.938234	1	2	0	8	9	1	9	0.02974549	0.9232309	7.375	6.63190341	1E+08	1.2E+08	ID=ENSP000003870 64;RIMKLA;;	RIMKLA				
6	1E+08	6_121137467	13	1	19	21	7	20	0.111433	1	4	7	7	2	6	5	0.98461866	1	9.25	7.61108215								
6	1E+08	6_121152237	1	8	1	1	5	1	0.186119	1	16	16	22	4	16	13	0.9394435	1	8.625	8.31414457								
6	1E+08	6_121152498	4	15	5	4	10	5	0.276106	1	0	13	16	33	7	25	0.00310365	0.1565896	11.25	10.57963					1.2E+08	1.2E+08	0.13179	ID=promoter_45189;
6	1E+08	6_121181483	20	13	7	26	17	17	0.897282	1	0	0	40	1	0	21	1.21E-05	0.0014894	13.375	14.4611549								
6	1E+08	6_121251118	19	37	7	22	28	15	0.042594	1	43	45	43	0	44	22	0.0558436	1	27	17.5417868								
6	1E+08	6_121286578	17	30	8	10	24	9	0.00876	0.36763	1	11	6	0	6	3	0.67473421	1	10.375	9.63531451								
6	1E+08	6_121347190	1	6	10	13	4	12	0.241003	1	8	17	5	0	13	3	0.06317581	1	7.5	5.78174467								
6	1E+08	6_121353861	33	16	12	12	25	12	0.031505	1	1	24	28	4	13	16	0.74830213	1	16.25	11.3231747								
6	1E+08	6_121378795	271	41	357	243	156	300	2.76E-06	0.00038	35	17	64	10	26	37	0.24075912	1	129.75	137.65874								
6	1E+08	6_121416652	24	19	17	18	22	18	0.522468	1	7	17	18	6	12	12	0.98292303	1	15.75	6.13537751					1.2E+08	1.2E+08	0.30651	ID=promoter_45225;
6	1E+08	6_121418048	4	0	4	3	2	4	0.823343	1	13	8	13	1	11	7	0.73613127	1	5.75	5.06387768								
6	1E+08	6_121448674	184	98	99	97	141	98	3.04E-04	0.02341	36	34	10	11	35	11	0.00454855	0.2107253	71.125	59.6092934								
6	1E+08	6_121475299	19	38	12	11	29	12	0.006478	0.28949	13	1	16	9	7	13	0.3188207	1	14.875	10.7362869					1.2E+08	1.2E+08	0.33842	ID=promoter_45239;
6	1E+08	6_121508941	15	11	27	12	13	20	0.747034	1	27	0	33	4	14	19	0.55289753	1	16.125	11.7890688								
6	1E+08	6_121545127	96	27	117	85	62	101	0.123558	1	8	0	18	12	4	15	0.026797	0.8546242	45.375	46.1672039								
6	1E+08	6_121577201	102	19	53	63	61	58	0.518037	1	0	31	22	17	16	20	0.67029873	1	38.375	32.7236808					1.2E+08	1.2E+08	0.25509	ID=promoter_45254;
6	1E+08	6_121579127	27	14	9	12	21	11	0.084122	1	22	5	3	16	14	10	0.81923661	1	13.5	8.15913161					1.2E+08	1.2E+08	0.38343	ID=promoter_45255;
6	1E+08	6_121579446	13	28	6	7	21	7	0.0088	0.36783	32	10	17	10	21	14	0.63082335	1	15.375	9.70916357					1.2E+08	1.2E+08	0.38343	ID=promoter_45255;

6	1E+08	6_121619142	42	51	64	95	47	80	0.125401	1	88	5	34	9	47	22	0.02632675	0.8479606	48.5	33.1102056	1E+08	1.2E+08	ID=ENSP00000361704;CTPS;NM_001905;	CTPS	1.2E+08	1.2E+08	0.55183	ID=promoter_45267;
6	1E+08	6_121626352	0	0	15	13	0	14	0.00256	0.1373	0	10	2	1	5	2	0.58563225	1	5.125	6.42400632					1.2E+08	1.2E+08	0.42649	ID=promoter_45268;
6	1E+08	6_121700124	2	0	12	7	1	10	0.048828	1	16	0	5	10	8	8	0.98858271	1	6.5	5.85540044								
6	1E+08	6_121744052	6	6	10	49	6	30	0.001943	0.11002	27	17	16	3	22	10	0.16182597	1	16.75	15.172814	1E+08	1.2E+08	ID=ENSP00000262916;KCNQ4;NM_004700;	KCNQ4	1.2E+08	1.2E+08	0.37139	ID=promoter_45288;
6	1E+08	6_121749955	0	18	9	16	9	13	0.89076	1	35	27	40	12	31	26	0.931505	1	19.625	13.5112387	1E+08	1.2E+08	ID=ENSP00000262916;KCNQ4;NM_004700;	KCNQ4				
6	1E+08	6_121758836	468	249	260	427	359	344	0.027226	0.89953	48	18	41	22	33	32	0.98369494	1	191.63	185.92851	1E+08	1.2E+08	ID=ENSP00000262916;KCNQ4;NM_004700;	KCNQ4				
6	1E+08	6_121797066	146	68	154	133	107	144	0.559416	1	90	45	47	55	68	51	0.51845391	1	92.25	45.7063609					1.2E+08	1.2E+08	0.42609	ID=promoter_45298;
6	1E+08	6_121797614	449	225	423	478	337	451	0.186525	1	181	188	167	96	185	132	0.07029526	1	275.88	149.280596					1.2E+08	1.2E+08	0.42609	ID=promoter_45298;
6	1E+08	6_121867216	32	44	32	34	38	33	0.447527	1	19	28	25	7	24	16	0.5906404	1	27.625	11.0445526								
6	1E+08	6_121876007	102	67	44	43	85	44	3.53E-05	0.0036	88	41	37	10	65	24	3.47E-04	0.0262008	54	29.866369								
6	1E+08	6_121967032	48	47	16	48	48	32	0.042987	1	41	24	36	3	33	20	0.31924699	1	32.875	16.8560078								
6	1E+08	6_122127087	81	101	82	106	91	94	0.688034	1	26	19	42	25	23	34	0.21851877	1	60.25	36.0703677								
6	1E+08	6_122185511	363	63	332	302	213	317	0.026574	0.88446	181	108	117	12	145	65	4.21E-06	0.0005888	184.75	132.213842	1E+08	1.2E+08	ID=ENSP00000361803;SMAP2;NM_022733;	SMAP2	1.2E+08	1.2E+08	0.29153	ID=promoter_45327;
6	1E+08	6_122490841	65	34	70	77	50	74	0.444751	1	33	44	31	3	39	17	0.02919595	0.915188	44.625	24.7093823					1.2E+08	1.2E+08	0.28918	ID=promoter_45341;
6	1E+08	6_122729163	6	12	1	0	9	1	0.020382	0.71452	12	0	8	9	6	9	0.65381141	1	6	5.09901951								
6	1E+08	6_122742474	35	63	23	17	49	20	1.71E-04	0.01419	82	99	106	22	91	64	0.23632065	1	55.875	36.4120761								
6	1E+08	6_122833517	3	18	3	11	11	7	0.438207	1	5	11	3	1	8	2	0.20550608	1	6.875	5.86606463								
6	1E+08	6_122896982	630	502	888	405	566	647	0.90459	1	436	269	577	108	353	343	0.82504553	1	476.88	235.810116					1.2E+08	1.2E+08	0.18916	ID=promoter_45361;
6	1E+08	6_122946237	5	16	14	4	11	9	0.722819	1	1	10	10	32	6	21	0.00855938	0.348087	11.5	9.71008312								
6	1E+08	6_122948364	1083	643	1063	651	863	857	0.002544	0.13706	541	443	496	224	492	360	0.00288879	0.1473631	643	297.042085								
6	1E+08	6_122960282	14	8	6	1	11	4	0.10516	1	2	4	11	1	3	6	0.53745121	1	5.875	4.82367672								
6	1E+08	6_122961327	13	8	13	10	11	12	0.985018	1	25	0	13	2	13	8	0.65599869	1	10.5	7.72750191					1.2E+08	1.2E+08	0.36918	ID=promoter_45369;
6	1E+08	6_123024915	696	230	476	521	463	499	0.422782	1	252	274	217	183	263	200	0.10687784	1	356.13	185.121919					1.2E+08	1.2E+08	0.4131	ID=promoter_45375;
6	1E+08	6_123049896	84	40	54	41	62	48	0.093606	1	73	46	47	23	60	35	0.08940859	1	51	19.3759792					1.2E+08	1.2E+08	0.60193	ID=promoter_45377;
6	1E+08	6_123118549	286	75	201	145	181	173	0.155632	1	78	66	55	27	72	41	0.0422523	1	116.63	88.0177539					1.2E+08	1.2E+08	0.28272	ID=promoter_45383;
6	1E+08	6_123121857	19	15	8	29	17	19	0.989587	1	15	18	7	12	17	10	0.5154613	1	15.375	6.98851098								

6	1E+08	6_123158703	136	84	118	139	110	129	0.999999	1	45	44	90	8	45	49	0.73760641	1	83	47.5244298								
7	113524	7_113524	374	163	168	179	269	174	4.26E-09	#######	165	138	69	78	152	74	2.29E-05	0.002595	166.75	93.6402691	22169	129476	ID=ENSP000002304 49;EXOC2;NM_018303;	EXOC2	112698	114982	0.4509	ID=promoter_45438;
7	118463	7_118463	5	6	8	7	6	8	0.9722	1	2	16	7	0	9	4	0.4571786	1	6.375	4.74906006	22169	129476	ID=ENSP000002304 49;EXOC2;NM_018303;	EXOC2	115706	118902	0.4738	ID=promoter_45439;
7	173181	7_173181	19	9	9	13	14	11	0.605836	1	89	19	17	2	54	10	1.34E-06	0.0002179	22.125	27.6479785								
7	177021	7_177021	305	189	264	230	247	247	0.207055	1	85	110	114	31	98	73	0.36604617	1	166	95.7496736								
7	193026	7_193026	39	24	30	22	32	26	0.376129	1	17	6	9	1	12	5	0.30420563	1	18.5	12.8173989					191839	193158	0.17869	ID=promoter_45461;
7	236482	7_236482	0	7	5	1	4	3	0.840045	1	20	4	1	45	12	23	0.11381058	1	10.375	15.3802982								
7	255466	7_255466	8	47	23	43	28	33	0.999827	1	24	40	60	23	32	42	0.32992529	1	33.5	16.8098611					254008	255784	0.43361	ID=promoter_45478;
7	263585	7_263585	60	232	287	298	146	293	5.61E-07	#######	24	82	75	27	53	51	0.98209156	1	135.63	116.55647								
7	271700	7_271700	57	14	57	61	36	59	0.282954	1	0	9	4	0	5	2	0.58563225	1	25.25	27.8041621								
7	353662	7_353662	4	5	30	33	5	32	3.82E-04	0.02825	5	4	5	1	5	3	0.8350419	1	10.875	12.8222741								
7	354574	7_354574	10	11	7	4	11	6	0.3028	1	14	0	5	0	7	3	0.51857467	1	6.375	5.09726818					354170	355665	0.31764	ID=promoter_45500;
7	359990	7_359990	27	44	16	7	36	12	2.99E-04	0.02308	13	19	13	14	16	14	0.9876114	1	19.125	11.5812348					359804	361494	0.27384	ID=promoter_45501;
7	446853	7_446853	0	8	0	0	4	0	0.09576	1	0	14	22	4	7	13	0.3188207	1	6	8.21149021								
7	659933	7_659933	219	97	128	108	158	118	9.11E-04	0.05851	123	88	58	48	106	53	9.58E-04	0.0604009	108.63	52.8662396								
7	681244	7_681244	7	56	0	7	32	4	1.65E-06	0.00024	1	6	14	0	4	7	0.58783259	1	11.375	18.6389569					679663	681276	0.23837	ID=promoter_45549;
7	716328	7_716328	65	31	49	103	48	76	0.26345	1	52	12	6	27	32	17	0.17107071	1	43.125	31.4662518					715343	716954	0.27628	ID=promoter_45560;
7	721729	7_721729	2	23	7	18	13	13	0.920459	1	2	4	25	6	3	16	0.00701108	0.2971393	10.875	9.56836305					721216	722267	0.25144	ID=promoter_45563;
7	760413	7_760413	33	13	18	7	23	13	0.098913	1	19	7	12	15	13	14	0.92696813	1	15.5	8.34950811					759973	760892	0.27115	ID=promoter_45576;
7	784221	7_784221	18	2	5	9	10	7	0.570701	1	10	2	5	7	6	6	0.99142475	1	7.25	5.23040561					782921	785355	0.6827	ID=promoter_45583;
7	797853	7_797853	411	169	507	474	290	491	4.04E-06	0.00054	274	144	126	77	209	102	4.57E-07	8.25E-05	272.75	169.740769					795916	800195	0.6944	ID=promoter_45589;
7	827050	7_827050	7	6	16	23	7	20	0.111433	1	0	0	7	0	0	4	0.11562589	1	7.375	8.31414457					826650	828223	0.29246	ID=promoter_45598;
7	914453	7_914453	14	6	9	12	10	11	0.989255	1	11	11	13	3	11	8	0.87190251	1	9.875	3.72011905					912858	916852	0.81176	ID=promoter_45622;
7	938429	7_938429	19	39	24	6	29	15	0.030102	0.97706	2	1	39	5	2	22	1.09E-04	0.0098689	16.875	15.8874389					937569	940580	0.61665	ID=promoter_45628;
7	951988	7_951988	11	84	24	45	48	35	0.09725	1	48	66	54	21	57	38	0.2863469	1	44.125	24.5498763								
7	953876	7_953876	0	0	0	0	0	0	1	1	16	5	10	14	11	12	0.92663959	1	5.625	6.80204801					952591	955301	0.55012	ID=promoter_45634;
7	953996	7_953996	49	46	38	54	48	46	0.618	1	23	8	22	1	16	12	0.85678722	1	30.125	19.6209618					952591	955301	0.55012	ID=promoter_45634;
7	989722	7_989722	63	6	52	33	35	43	0.979504	1	65	11	4	21	38	13	0.0053803	0.2438003	31.875	25.2780961					989164	992512	0.6789	ID=promoter_45643;
7	990198	7_990198	32	8	29	2	20	16	0.517469	1	0	16	9	0	8	5	0.78486764	1	12	12.6378117					989164	992512	0.6789	ID=promoter_45643;
7	991787	7_991787	798	287	685	378	543	532	0.012719	0.4931	693	422	487	266	558	377	1.17E-05	0.0014539	502	200.537848					989164	992512	0.6789	ID=promoter_45643;

7	999098	7_999098	97	83	109	150	90	130	0.314489	1	47	67	49	55	57	52	0.99650227	1	82.125	35.5505174					997534	1003968	0.886	ID=promoter_45646;
7	1E+06	7_1002867	3	28	15	7	16	11	0.385778	1	42	11	13	0	27	7	0.00575936	0.2548307	14.875	13.8918013					997534	1003968	0.886	ID=promoter_45646;
7	1E+06	7_100439	57	77	73	42	67	58	0.235639	1	97	44	64	23	71	44	0.10720797	1	59.625	23.2989117								
7	1E+06	7_1008422	237	58	156	115	148	136	0.119829	1	27	15	17	24	21	21	0.97030696	1	81.125	81.2956465					1007051	1008572	0.23841	ID=promoter_45647;
7	1E+06	7_1012725	34	18	53	28	26	41	0.495657	1	19	16	34	46	18	40	0.00638295	0.2759882	31	13.4907376					1009242	1014178	0.81639	ID=promoter_45648;
7	1E+06	7_1017776	10	21	12	6	16	9	0.196587	1	40	5	27	1	23	14	0.45771946	1	15.25	13.1773615					1015758	1018237	0.44512	ID=promoter_45650;
7	1E+06	7_103301	24	11	19	5	18	12	0.307258	1	9	1	7	3	5	5	0.99284883	1	9.875	7.95410945								
7	1E+06	7_1040749	5	26	11	16	16	14	0.724026	1	12	27	32	15	20	24	0.69398062	1	18	9.31971796					1038815	1043903	0.7767	ID=promoter_45658;
7	1E+06	7_1078100	4	19	10	3	12	7	0.325249	1	1	0	6	1	1	4	0.36109265	1	5.5	6.34710283					1075479	1078169	0.4741	ID=promoter_45670;
7	1E+06	7_111783	54	73	18	22	64	20	2.07E-07	#######	67	31	39	15	49	27	0.0898846	1	39.875	22.4527281								
7	1E+06	7_114167	14	23	14	20	19	17	0.718296	1	7	12	26	15	10	21	0.0912903	1	16.375	6.20915223								
7	1E+06	7_1201795	54	101	47	47	78	47	0.001171	0.07205	142	57	38	19	100	29	4.28E-08	9.53E-06	63.125	39.4151439					1200350	1202609	0.46906	ID=promoter_45707;
7	1E+06	7_122061	3	29	20	27	16	24	0.746917	1	2	36	51	21	19	36	0.03633239	1	23.625	16.2826595								
7	1E+06	7_122783	7	31	13	11	19	12	0.233425	1	50	11	32	21	31	27	0.97229028	1	22	14.7066554								
7	1E+06	7_126326	252	157	177	181	205	179	0.015107	0.56646	62	40	45	34	51	40	0.73048207	1	118.5	83.2637805								
7	1E+06	7_1281014	62	30	35	18	46	27	0.014364	0.54261	22	43	5	1	33	3	1.11E-05	0.0013866	27	20.0713015					1280113	1283661	0.71662	ID=promoter_45729;
7	1E+06	7_1312523	0	11	11	1	6	6	0.962469	1	0	13	26	1	7	14	0.23485029	1	7.875	9.20306626					1310181	1312611	0.4616	ID=promoter_45738;
7	1E+06	7_1315696	71	31	58	112	51	85	0.13708	1	44	11	6	0	28	3	1.04E-04	0.0094884	41.625	38.0636216					1313852	1316294	0.39593	ID=promoter_45739;
7	1E+06	7_132205	8	21	2	8	15	5	0.034023	1	13	1	16	0	7	8	0.92121674	1	8.625	7.59581652								
7	1E+06	7_1356738	43	41	45	69	42	57	0.772843	1	118	13	24	9	66	17	3.09E-06	0.000453	45.25	35.2247883					1354433	1357594	0.52194	ID=promoter_45750;
7	1E+06	7_1370874	13	21	11	13	17	12	0.395844	1	95	20	31	42	58	37	0.20262844	1	30.75	27.9936217					1370083	1371871	0.30169	ID=promoter_45754;
7	1E+06	7_138389	3	0	5	2	2	4	0.823343	1	9	17	12	0	13	6	0.35360803	1	6	6.14119579								
7	1E+06	7_1416506	1	11	7	2	6	5	0.851367	1	34	13	5	7	24	6	0.00867909	0.3505674	10	10.515295	1E+06	1425993	ID=ENSP000003179 98-D2;RPP40;NM_006638;	RPP40				
7	1E+06	7_145133	3	8	5	3	6	4	0.674789	1	7	19	25	9	13	17	0.64388579	1	9.875	7.95410945								
7	1E+06	7_146371	13	0	6	10	7	8	0.998739	1	30	4	18	0	17	9	0.38755874	1	10.125	10.1761275								
7	1E+06	7_1482666	46	18	29	34	32	32	0.815447	1	15	9	18	2	12	10	0.96947392	1	21.375	14.2220905					1482551	1483268	0.17942	ID=promoter_45774;
7	2E+06	7_153044	21	23	25	11	22	18	0.522468	1	6	5	7	8	6	8	0.79537884	1	13.25	8.32809359								
7	2E+06	7_154923	10	14	10	0	12	5	0.126784	1	38	30	58	0	34	29	0.94710596	1	20	20.3680422								
7	2E+06	7_155957	4	60	17	9	32	13	0.003339	0.17064	35	56	15	8	46	12	1.61E-04	0.0137078	25.5	22.1488471								
7	2E+06	7_1563148	4	52	21	18	28	20	0.231138	1	13	38	47	12	26	30	0.71540497	1	25.625	17.7195413					1562964	1563682	0.69388	ID=promoter_45798;
7	2E+06	7_159661	163	29	142	94	96	118	0.943643	1	1	42	11	2	22	7	0.03554381	1	60.5	64.3850027								
7	2E+06	7_1622730	68	22	51	38	45	45	0.750586	1	32	15	37	3	24	20	0.93987967	1	33.25	20.4782114					1622615	1623332	0.17942	ID=promoter_45811;
7	2E+06	7_1637577	988	435	672	816	712	744	0.087855	1	369	343	248	79	356	164	3.83E-13	1.67E-10	493.75	305.672911					1636494	1640246	0.84717	ID=promoter_45816;
7	2E+06	7_165881	3	29	12	16	16	14	0.724026	1	26	22	23	31	24	27	0.78764771	1	20.25	9.40744386								

7	2E+06	7_1693630	7	12	6	7	10	7	0.570701	1	10	12	21	7	11	14	0.73202476	1	10.25	4.94974747	2E+06	1696258	ID=ENSP00000317998;RPP40;NM_006638;	RPP40				
7	2E+06	7_1728595	13	9	4	10	11	7	0.438207	1	8	5	0	6	7	3	0.51857467	1	6.875	4.0155946					1728124	1728905	0.20944	ID=promoter_45831;
7	2E+06	7_1737823	35	35	52	39	35	46	0.879175	1	54	66	41	30	60	36	0.11494742	1	44	12.188988					1736026	1740490	0.74463	ID=promoter_45833;
7	2E+06	7_174217	91	67	143	137	79	140	0.012759	0.49423	0	11	10	3	6	7	0.91781435	1	57.75	60.4004494								
7	2E+06	7_1753492	41	34	23	24	38	24	0.054487	1	9	16	10	46	13	28	0.03522609	1	25.375	13.8557621					1752801	1753779	0.19044	ID=promoter_45837;
7	2E+06	7_1756212	1	23	11	10	12	11	0.839207	1	6	30	12	23	18	18	0.97449422	1	14.5	9.84160266					1755634	1756393	0.29566	ID=promoter_45838;
7	2E+06	7_177701	15	22	20	37	19	29	0.670114	1	2	10	9	23	6	16	0.069189	1	17.25	10.7670131								
7	2E+06	7_1784898	216	121	193	218	169	206	0.933661	1	153	31	53	63	92	58	0.0687376	1	131	75.5267219					1784242	1785010	0.15978	ID=promoter_45844;
7	2E+06	7_1810439	0	0	0	9	0	5	0.118674	1	22	9	1	0	16	1	0.00230968	0.1234682	5.125	7.90004521					1810038	1811275	0.25399	ID=promoter_45851;
7	2E+06	7_1860506	388	138	231	222	263	227	0.003099	0.16017	109	161	157	8	135	83	0.01233035	0.4658807	176.75	110.080166					1860223	1861001	0.11502	ID=promoter_45859;
7	2E+06	7_1881440	2029	2226	2919	3041	2128	2980	3.25E-09	#######	1716	1466	1805	596	1591	1201	5.07E-07	9.05E-05	1974.8	788.317512					1881375	1882177	0.12887	ID=promoter_45865;
7	2E+06	7_1936239	364	81	264	325	223	295	0.398623	1	55	37	120	70	46	95	2.79E-05	0.0030667	164.5	131.795296					1935739	1936517	0.11502	ID=promoter_45881;
7	2E+06	7_198690	9	48	10	17	29	14	0.018844	0.67198	6	9	8	0	8	4	0.59180275	1	13.375	14.7545393								
7	2E+06	7_2024671	299	174	177	176	237	177	2.75E-05	0.00288	47	15	33	6	31	20	0.44595779	1	115.88	105.644197					2023959	2025411	0.23879	ID=promoter_45894;
7	2E+06	7_210533	1	0	6	5	1	6	0.241978	1	13	8	22	20	11	21	0.13989775	1	9.375	8.24512843								
7	2E+06	7_210896	10	1	12	16	6	14	0.355886	1	40	20	7	5	30	6	8.03E-04	0.0527633	13.875	12.1589179								
7	2E+06	7_2109889	2976	2120	2866	3598	2548	3232	0.012701	0.49265	809	759	1773	448	784	1111	5.68E-19	3.74E-16	1918.6	1173.29547					2109349	2110085	0.183	ID=promoter_45907;
7	2E+06	7_2112397	32	54	3	4	43	4	3.21E-09	#######	98	64	9	31	81	20	9.15E-08	1.92E-05	36.875	33.4426481					2111658	2113146	0.27215	ID=promoter_45908;
7	2E+06	7_211703	0	12	14	3	6	9	0.896349	1	9	37	23	3	23	13	0.35430953	1	12.625	12.3165104								
7	2E+06	7_216850	117	91	100	77	104	89	0.090412	1	56	15	28	0	36	14	0.01744443	0.6140941	60.5	42.698277								
7	2E+06	7_218660	1	12	0	2	7	1	0.062633	1	12	7	15	1	10	8	0.95278883	1	6.25	6.04152299								
7	2E+06	7_2191673	54	34	94	65	44	80	0.062614	1	80	47	71	0	64	36	0.05308728	1	55.625	29.3497992					2191039	2191934	0.27356	ID=promoter_45925;
7	2E+06	7_224494	0	3	0	5	2	3	0.964182	1	25	2	5	0	14	3	0.04246461	1	5	8.34950811								
7	2E+06	7_226767	2	14	3	2	8	3	0.206144	1	6	3	10	3	5	7	0.77775394	1	5.375	4.40575922								
7	2E+06	7_2298589	2	5	6	4	4	5	0.995512	1	2	6	6	25	4	16	0.01700101	0.6008545	7	7.46420027	2E+06	2311233	ID=ENSP00000369461;PECI;;	PECI				
7	2E+06	7_2309000	4	21	2	19	13	11	0.725732	1	35	11	3	0	23	2	3.18E-04	0.0243067	11.875	12.2408858	2E+06	2311233	ID=ENSP00000369461;PECI;;	PECI	2308648	2309261	0.14353	ID=promoter_45948;
7	2E+06	7_231841	8	48	14	5	28	10	0.00287	0.14985	11	12	15	42	12	29	0.01494463	0.5435394	19.375	16.2123189								
7	2E+06	7_2359147	91	69	40	62	80	51	0.002552	0.13708	47	35	40	18	41	29	0.53508799	1	50.25	22.8144691					2358308	2360228	0.33252	ID=promoter_45961;
7	2E+06	7_241320	9	23	13	9	16	11	0.385778	1	21	45	22	9	33	16	0.09595749	1	18.875	12.1941145								
7	2E+06	7_242326	19	10	5	14	15	10	0.374041	1	30	19	18	4	25	11	0.10848128	1	14.875	8.55966455								
7	2E+06	7_245728	16	27	16	5	22	11	0.058934	1	64	23	56	13	44	35	0.79543197	1	27.5	21.1997305								
7	2E+06	7_2462112	17	48	18	6	33	12	0.001163	0.07164	14	11	18	1	13	10	0.90601506	1	16.625	14.0604562					2461850	2463601	0.26288	ID=promoter_45984;
7	3E+06	7_2503109	24	11	12	23	18	18	0.89158	1	11	10	12	12	11	12	0.92663959	1	14.375	5.68048036					2502813	2503802	0.15628	ID=promoter_45994;

7	3E+06	7_2516193	60	44	35	29	52	32	0.014039	0.53368	12	20	5	30	16	18	0.85288016	1	29.375	17.5981939	3E+06	2525087	ID=ENSP0000369636;C6orf145;NM_183373;	C6orf145				
7	3E+06	7_2520929	4	25	2	7	15	5	0.034023	1	28	0	47	36	14	42	3.00E-04	0.023179	18.625	17.7598061	3E+06	2525087	ID=ENSP0000369636;C6orf145;NM_183373;	C6orf145	2520018	2521247	0.36799	ID=promoter_45998;
7	3E+06	7_252705	11	60	12	5	36	9	2.87E-05	0.00298	16	13	10	26	15	18	0.76034467	1	19.125	17.5860131								
7	3E+06	7_253240	22	0	7	24	11	16	0.859437	1	58	28	34	1	43	18	0.01460712	0.5325962	21.75	19.3815081								
7	3E+06	7_257055	106	37	77	76	72	77	0.85308	1	19	6	6	18	13	12	0.99994263	1	43.125	38.1517178								
7	3E+06	7_260116	38	31	40	19	35	30	0.448769	1	20	7	0	0	14	0	0.0015196	0.0882005	19.375	16.088483								
7	3E+06	7_260191	70	63	76	23	67	50	0.050914	1	72	26	50	39	49	45	0.99896345	1	52.375	21.0776625								
7	3E+06	7_2626048	100	50	168	133	75	151	5.57E-04	0.03874	41	41	43	42	41	43	0.85218106	1	77.25	50.2102721	3E+06	2766956	ID=ENSP0000385028;SLC22A23;NM_015482;	SLC22A23	2624200	2627755	0.76078	ID=promoter_46020;
7	3E+06	7_2627315	689	389	574	389	539	482	8.09E-05	0.00736	340	481	273	135	411	204	1.16E-12	4.81E-10	408.75	173.180954	3E+06	2766956	ID=ENSP0000385028;SLC22A23;NM_015482;	SLC22A23	2624200	2627755	0.76078	ID=promoter_46020;
7	3E+06	7_2639851	23	68	43	38	46	41	0.439359	1	71	69	82	47	70	65	0.99995432	1	55.125	20.2515432	3E+06	2766956	ID=ENSP0000385028;SLC22A23;NM_015482;	SLC22A23	2639410	2639960	0.14998	ID=promoter_46023;
7	3E+06	7_2674199	287	65	285	324	176	305	1.82E-04	0.01496	14	5	13	1	10	7	0.84912739	1	124.25	146.229516	3E+06	2766956	ID=ENSP0000385028;SLC22A23;NM_015482;	SLC22A23	2673284	2674840	0.27241	ID=promoter_46034;
7	3E+06	7_2719749	94	15	87	96	55	92	0.111804	1	1	11	0	19	6	10	0.51486293	1	40.375	43.5691159	3E+06	2766956	ID=ENSP0000385028;SLC22A23;NM_015482;	SLC22A23	2719247	2721129	0.23452	ID=promoter_46050;
7	3E+06	7_2723403	36	47	62	54	42	58	0.722298	1	30	34	62	27	32	45	0.19260481	1	44	14.1724078	3E+06	2766956	ID=ENSP0000385028;SLC22A23;NM_015482;	SLC22A23	2723286	2724682	0.2172	ID=promoter_46051;
7	3E+06	7_2724448	27	15	38	55	21	47	0.043438	1	41	50	53	6	46	30	0.32156472	1	35.625	18.1102773	3E+06	2766956	ID=ENSP0000385028;SLC22A23;NM_015482;	SLC22A23	2723286	2724682	0.2172	ID=promoter_46051;
7	3E+06	7_2727241	58	28	26	30	43	28	0.048681	1	0	0	19	17	0	18	6.08E-05	0.0059624	22.25	18.5684063	3E+06	2766956	ID=ENSP0000385028;SLC22A23;NM_015482;	SLC22A23	2726910	2728354	0.29846	ID=promoter_46052;
7	3E+06	7_2734596	20	42	13	18	31	16	0.023279	0.79554	26	56	28	4	41	16	0.01022012	0.4008669	25.875	16.5308154	3E+06	2766956	ID=ENSP0000385028;SLC22A23;NM_015482;	SLC22A23	2732610	2735176	0.42449	ID=promoter_46054;
7	3E+06	7_2737971	7	0	4	14	4	9	0.54411	1	2	1	8	27	2	18	8.85E-04	0.0572814	7.875	8.96720214	3E+06	2766956	ID=ENSP0000385028;SLC22A23;NM_015482;	SLC22A23				
7	3E+06	7_2767630	73	53	16	12	63	14	2.37E-09	#######	21	6	14	21	14	18	0.65378371	1	27	23.3605284					2766301	2768214	0.38853	ID=promoter_46063;
7	3E+06	7_279209	88	32	74	73	60	74	0.959109	1	46	70	38	26	58	32	0.05843809	1	55.875	23.0925931								
7	3E+06	7_2858196	178	174	128	201	176	165	0.116869	1	314	182	208	148	248	178	0.03300093	1	191.63	55.8977063	3E+06	2871886	ID=ENSP0000369739;BPHL;;	BPHL	2858149	2860229	0.31356	ID=promoter_46083;

7	3E+06	7_2883488	27	0	42	14	14	28	0.256487	1	16	11	1	0	14	1	0.00574567	0.2548307	13.875	14.7690555	3E+06	2903083	ID=ENSP0000369773;RIPK1;NM_003804;	RIPK1	2882795	2883771	0.31097	ID=promoter_46090;
7	3E+06	7_292724	38	1	8	1	20	5	0.002998	0.15536	0	2	11	0	1	6	0.13767373	1	7.625	12.9276835								
7	3E+06	7_298273	282	187	206	189	235	198	0.002645	0.14125	181	96	138	87	139	113	0.58373892	1	170.75	63.2404708								
7	3E+06	7_299088	1	17	0	0	9	0	0.005101	0.2381	11	1	14	1	6	8	0.79537884	1	5.625	7.1301673								
7	3E+06	7_3021375	22	28	22	26	25	24	0.781475	1	62	83	50	22	73	36	0.00698312	0.296747	39.375	23.1204146					3020244	3022454	0.2903	ID=promoter_46121;
7	3E+06	7_3023633	37	69	28	28	53	28	0.002148	0.11858	114	80	57	28	97	43	2.07E-04	0.0169862	55.125	31.2063982					3023104	3023962	0.25429	ID=promoter_46122;
7	3E+06	7_3165139	17	28	8	26	23	17	0.346113	1	64	8	8	0	36	4	8.86E-06	0.0011428	19.875	20.2726945					3163801	3167975	0.70641	ID=promoter_46168;
7	3E+06	7_3221781	18	47	36	22	33	29	0.523141	1	77	39	34	13	58	24	0.00294193	0.1496995	35.75	20.2255143					3221619	3222512	0.13093	ID=promoter_46180;
7	3E+06	7_3227114	27	67	28	41	47	35	0.121692	1	69	34	25	28	52	27	0.04641028	1	39.875	18.0826476					3226745	3227291	0.11442	ID=promoter_46182;
7	3E+06	7_325893	18	4	17	13	11	15	0.930409	1	2	18	3	3	10	3	0.19568363	1	9.75	7.40173725								
7	3E+06	7_329261	20	49	22	15	35	19	0.02151	0.74463	27	6	21	1	17	11	0.64636287	1	20.125	14.5252244								
7	3E+06	7_3295612	2	24	15	9	13	12	0.835334	1	7	3	22	2	5	12	0.17891578	1	10.5	8.8640526					3293695	3296706	0.52716	ID=promoter_46197;
7	3E+06	7_330770	16	91	59	109	54	84	0.267527	1	61	92	134	24	77	79	0.81821933	1	73.25	40.8682552								
7	3E+06	7_3310181	15	16	8	2	16	5	0.021336	0.74103	0	6	1	4	3	3	0.99570314	1	6.5	6.14119579					3309621	3310691	0.21878	ID=promoter_46201;
7	3E+06	7_3358836	11	5	4	14	8	9	0.996316	1	13	8	11	2	11	7	0.73613127	1	8.5	4.44007722					3357711	3361663	0.6986	ID=promoter_46218;
7	3E+06	7_3370051	25	24	45	26	25	36	0.732372	1	0	0	4	0	0	2	0.34003807	1	15.5	16.8861735					3369390	3370313	0.27538	ID=promoter_46221;
7	3E+06	7_3382558	80	47	84	57	64	71	0.949062	1	62	36	68	55	49	62	0.26318652	1	61.125	16.0929221					3381768	3383051	0.24599	ID=promoter_46226;
7	3E+06	7_3391682	67	113	77	67	90	72	0.05158	1	92	8	84	27	50	56	0.62274969	1	66.875	34.2488269					3391603	3392498	0.17413	ID=promoter_46231;
7	3E+06	7_3404160	412	464	374	471	438	423	0.016946	0.61978	328	384	197	112	356	155	9.07E-15	4.56E-12	342.75	127.23965					3403673	3404174	0.11661	ID=promoter_46236;
7	3E+06	7_3445773	31	69	26	10	50	18	3.18E-05	0.00328	7	3	12	50	5	31	3.00E-05	0.0032574	26	23.2747564					3445456	3446158	0.17273	ID=promoter_46249;
7	4E+06	7_3546834	13	9	3	2	11	3	0.050503	1	29	22	16	0	26	8	0.01635039	0.5814583	11.75	10.2782711					3546415	3547276	0.14756	ID=promoter_46275;
7	4E+06	7_3546905	336	418	266	340	377	303	5.30E-06	0.00068	314	216	254	145	265	200	0.08902131	1	286.13	84.3706745					3546415	3547276	0.14756	ID=promoter_46275;
7	4E+06	7_356895	18	0	11	1	9	6	0.554309	1	0	5	11	0	3	6	0.53745121	1	5.75	6.84000835								
7	4E+06	7_358666	5	30	8	17	18	13	0.404491	1	82	35	42	1	59	22	8.10E-04	0.0530524	27.5	26.5706605								
7	4E+06	7_3605083	12	2	7	10	7	9	0.983538	1	0	11	15	0	6	8	0.79537884	1	7.125	5.81715443					3604229	3608335	0.56989	ID=promoter_46289;
7	4E+06	7_3615080	41	15	25	16	28	21	0.295201	1	104	47	10	1	76	6	1.36E-12	5.61E-10	32.375	32.7934554					3612681	3616019	0.52974	ID=promoter_46291;
7	4E+06	7_3628307	92	38	43	61	65	52	0.117524	1	23	51	54	52	37	53	0.12301229	1	51.75	20.0124961					3627472	3628683	0.20921	ID=promoter_46295;
7	4E+06	7_3629646	5719	3851	4866	6544	4785	5705	0.706765	1	1705	1510	1363	324	1608	844	1.12E-40	1.68E-37	3235.3	2313.13589					3629221	3631093	0.22246	ID=promoter_46296;
7	4E+06	7_3653478	17	25	7	33	21	20	0.800056	1	45	2	41	0	24	21	0.98147478	1	21.25	17.5234537					3652888	3653667	0.21192	ID=promoter_46303;
7	4E+06	7_3745180	42	40	23	29	41	26	0.044828	1	36	18	44	43	27	44	0.06498379	1	34.375	9.89859297					3743611	3745761	0.38793	ID=promoter_46321;
7	4E+06	7_376856	23	39	17	10	31	14	0.008767	0.36763	17	28	19	27	23	23	0.96752545	1	22.5	8.88015444								

7	4E+06	7_382112	32	16	47	20	24	34	0.777678	1	13	26	49	48	20	49	7.09E-04	0.047505	31.375	14.9469299								
7	4E+06	7_3930303	5	22	9	11	14	10	0.480768	1	13	38	14	19	26	17	0.53107524	1	16.375	10.2530483					3929231	3930435	0.14283	ID=promoter_46347;
7	4E+06	7_3943055	35	9	22	44	22	33	0.669499	1	19	31	0	21	25	11	0.10848128	1	22.625	14.1111658	4E+06	4101357	ID=ENSP00000264870;F13A1;NM_000129;	F13A1				
7	4E+06	7_3947394	17	8	12	8	13	10	0.600182	1	0	1	8	13	1	11	0.01045793	0.4067594	8.375	5.78020019	4E+06	4101357	ID=ENSP00000264870;F13A1;NM_000129;	F13A1	3947160	3948240	0.23178	ID=promoter_46352;
7	4E+06	7_3953902	18	10	1	0	14	1	0.001161	0.07164	0	19	10	36	10	23	0.04592065	1	11.75	12.4067954	4E+06	4101357	ID=ENSP00000264870;F13A1;NM_000129;	F13A1	3953537	3954471	0.20173	ID=promoter_46353;
7	4E+06	7_3989370	36	19	19	13	28	16	0.064038	1	65	13	33	10	39	22	0.17007728	1	26	18.3692289	4E+06	4101357	ID=ENSP00000264870;F13A1;NM_000129;	F13A1				
7	4E+06	7_4047987	573	500	1058	1113	537	1086	1.86E-24	#######	502	404	762	120	453	441	0.76544199	1	629	334.15095	4E+06	4101357	ID=ENSP00000264870;F13A1;NM_000129;	F13A1	4046432	4049944	0.66456	ID=promoter_46367;
7	4E+06	7_4070358	10	1	1	9	6	5	0.851367	1	24	17	10	5	21	8	0.08664698	1	9.625	7.85470741	4E+06	4101357	ID=ENSP00000264870;F13A1;NM_000129;	F13A1	4069845	4070596	0.22257	ID=promoter_46372;
7	4E+06	7_4085102	6	24	8	18	15	13	0.725173	1	48	10	20	18	29	19	0.49734269	1	19	13.3095025	4E+06	4101357	ID=ENSP00000264870;F13A1;NM_000129;	F13A1				
7	4E+06	7_413409	20	25	6	10	23	8	0.007066	0.30636	4	16	23	44	10	34	5.55E-04	0.0385056	18.5	12.8730061								
7	4E+06	7_420439	1	17	0	4	9	2	0.058587	1	1	14	10	0	8	5	0.78486764	1	5.875	6.83347851								
7	4E+06	7_4291752	28	41	34	14	35	24	0.122315	1	20	25	30	18	23	24	0.92099721	1	26.25	8.89221168					4291309	4292194	0.13351	ID=promoter_46385;
7	4E+06	7_4368998	4	43	12	9	24	11	0.027804	0.91326	2	2	10	1	2	6	0.31385293	1	10.375	13.8247966					4368844	4369696	0.17812	ID=promoter_46403;
7	4E+06	7_438489	213	88	121	163	151	142	0.166287	1	136	67	107	26	102	67	0.08858616	1	115.13	57.8209984								
7	4E+06	7_4391067	25	30	5	30	28	18	0.129829	1	3	28	20	15	16	18	0.85288016	1	19.5	10.8627805					4390720	4391466	0.10502	ID=promoter_46404;
7	4E+06	7_4451849	22	3	6	13	13	10	0.600182	1	51	54	50	15	53	33	0.1954656	1	26.75	21.4326187					4451696	4452384	0.16435	ID=promoter_46414;
7	5E+06	7_4522995	41	25	30	22	33	26	0.311396	1	31	14	26	18	23	22	0.99450571	1	25.875	8.37406711					4521588	4523942	0.32111	ID=promoter_46424;
7	5E+06	7_4534616	12	0	11	16	6	14	0.355886	1	1	0	0	0	1	0	0.62906424	1	5	6.78232998					4532178	4534686	0.34877	ID=promoter_46427;
7	5E+06	7_4554304	470	166	270	246	318	258	5.62E-05	0.00537	126	44	14	43	85	29	8.03E-06	0.0010461	172.38	153.075087	5E+06	4605875	ID=ENSP00000369265;RREB1;NM_001003699;	RREB1				
7	5E+06	7_467816	31	115	46	34	73	40	3.89E-04	0.0287	155	104	82	71	130	77	0.0071983	0.3031557	79.75	43.3976629								
7	5E+06	7_469838	25	3	4	5	14	5	0.053576	1	0	6	1	3	3	2	0.9363008	1	5.875	7.97204939								
7	5E+06	7_471858	5	36	21	25	21	23	0.974526	1	19	7	15	23	13	19	0.44319111	1	18.875	10.006248								
7	5E+06	7_4784142	30	31	22	6	31	14	0.008767	0.36763	12	30	57	20	21	39	0.03240705	0.9910413	26	15.4272486					4783747	4785248	0.61247	ID=promoter_46475;
7	5E+06	7_4784266	15	7	16	19	11	18	0.682529	1	8	5	7	12	7	10	0.67636194	1	11.125	5.11126208					4783747	4785248	0.61247	ID=promoter_46475;
7	5E+06	7_479074	16	47	26	18	32	22	0.148825	1	49	35	104	5	42	55	0.23814509	1	37.5	30.8961394								
7	5E+06	7_483740	16	7	4	21	12	13	0.980472	1	14	45	27	12	30	20	0.51779033	1	18.25	13.0466197								
7	5E+06	7_486678	67	331	34	62	199	48	1.71E-26	#######	254	167	108	85	211	97	4.03E-08	9.09E-06	138.5	104.803217								

7	5E+06	7_487813	37	25	20	3	31	12	0.002785	0.14699	65	23	19	2	44	11	1.66E-04	0.0140498	24.25	20.0624026								
7	5E+06	7_4961402	1	1	8	5	1	7	0.163779	1	1	20	9	2	11	6	0.57179039	1	5.875	6.55607679	5E+06	4962999	ID=ENSP00000339834;SNRNP48;NM_152551;	SNRNP48	4961044	4963622	0.56393	ID=promoter_46493;
7	5E+06	7_497397	0	8	12	3	4	8	0.677894	1	0	5	20	0	3	10	0.11508955	1	6	7.11135912								
7	5E+06	7_4989080	21	41	36	23	31	30	0.753666	1	18	31	32	12	25	22	0.9842524	1	26.75	9.82344135					4989054	4989703	0.1266	ID=promoter_46497;
7	5E+06	7_499284	63	23	59	24	43	42	0.699784	1	54	36	19	43	45	31	0.44260393	1	40.125	17.307616								
7	5E+06	7_501456	7	35	13	9	21	11	0.084122	1	25	16	7	0	21	4	0.00573189	0.2548307	14	11.2503968								
7	5E+06	7_504928	190	83	142	129	137	136	0.385886	1	41	17	9	3	29	6	0.00120521	0.0734459	76.75	70.4369019								
7	5E+06	7_508538	171	120	196	165	146	181	0.883477	1	71	21	21	15	46	18	0.00598711	0.263276	97.5	75.0047618								
7	5E+06	7_510186	1	0	5	6	1	6	0.241978	1	13	18	12	0	16	6	0.14768941	1	6.875	6.7704716								
7	5E+06	7_512139	5	7	6	8	6	7	0.999953	1	10	9	1	8	10	5	0.51906437	1	6.75	2.81577191								
7	5E+06	7_5431948	7	28	16	28	18	22	0.991654	1	49	21	22	4	35	13	0.01427485	0.52128	21.875	14.0756883					5431893	5432457	0.11741	ID=promoter_46551;
7	6E+06	7_5513535	544	318	687	612	431	650	2.67E-04	0.0209	18	3	25	1	11	13	0.83819512	1	276	301.217529					5511974	5515684	0.54091	ID=promoter_46570;
7	6E+06	7_5553820	34	57	12	38	46	25	0.006513	0.28949	72	35	32	19	54	26	0.02018388	0.6864966	37.375	19.3164438					5553621	5554504	0.12676	ID=promoter_46576;
7	6E+06	7_5633710	340	171	192	240	256	216	0.001638	0.09564	169	162	109	76	166	93	4.44E-04	0.0320666	182.38	80.7835512					5633178	5634647	0.34253	ID=promoter_46579;
7	6E+06	7_568934	8	39	8	29	24	19	0.435991	1	39	55	47	13	47	30	0.2752571	1	29.75	18.2580706								
7	6E+06	7_571576	29	7	18	5	18	12	0.307258	1	4	0	3	0	2	2	0.99713337	1	8.25	10.138329								
7	6E+06	7_5741909	147	62	102	104	105	103	0.434769	1	61	47	43	2	54	23	0.00592153	0.2605525	71	44.9253349					5740852	5742401	0.42953	ID=promoter_46584;
7	6E+06	7_591768	0	25	17	11	13	14	0.975695	1	6	3	9	19	5	14	0.08295177	1	11.25	8.53145441								
7	6E+06	7_612581	23	52	48	41	38	45	0.999061	1	47	32	54	4	40	29	0.60017008	1	37.625	17.1459075								
7	6E+06	7_639723	10	76	11	16	43	14	4.87E-05	0.00473	44	14	38	17	29	28	0.98828997	1	28.25	23.0945263								
7	6E+06	7_643419	58	20	66	59	39	63	0.288156	1	68	0	7	30	34	19	0.20290377	1	38.5	27.5577316								
7	7E+06	7_660912	0	8	4	14	4	9	0.54411	1	1	3	13	0	2	7	0.20455692	1	5.375	5.65527567								
7	7E+06	7_667100	11	8	2	16	10	9	0.84607	1	0	37	30	1	19	16	0.96066018	1	13.125	13.8196289								
7	7E+06	7_674446	13	0	13	8	7	11	0.831726	1	0	0	0	7	0	4	0.11562589	1	5.125	5.86606463								
7	7E+06	7_734190	102	39	27	14	71	21	1.57E-08	#######	26	79	37	55	53	46	0.94791873	1	47.375	29.7750495								
7	7E+06	7_740075	12	37	33	62	25	48	0.13045	1	172	98	143	129	135	136	0.79116085	1	85.75	58.4312783								
7	7E+06	7_7457550	13	50	18	46	32	32	0.815447	1	91	68	55	1	80	28	2.39E-05	0.0026765	42.75	30.2831872					7456736	7457729	0.1432	ID=promoter_46605;
7	8E+06	7_7637232	1179	653	1163	1153	916	1158	0.23798	1	1472	917	1031	468	1195	750	3.81E-16	2.05E-13	1004.5	319.804405	8E+06	7642522	ID=ENSP00000377265;TFAP2B;NM_003221;	TFAP2B	7635522	7637530	0.67457	ID=promoter_46612;
7	8E+06	7_766781	21	8	26	31	15	29	0.283564	1	1	10	6	1	6	4	0.87665919	1	13	11.5139667								
7	8E+06	7_7775241	0	21	1	6	11	4	0.10516	1	43	13	9	1	28	5	7.63E-04	0.0504367	11.75	14.5086181					7774823	7775730	0.13971	ID=promoter_46621;
7	8E+06	7_8025960	12	16	6	4	14	5	0.053576	1	0	5	0	18	3	9	0.17582851	1	7.625	6.92691438	8E+06	8055249	ID=ENSP00000346693;ELOVL2;NM_017770;	ELOVL2				
7	8E+06	7_807064	32	37	4	10	35	7	7.74E-06	0.00096	49	36	46	14	43	30	0.4873798	1	28.5	16.9873903								
7	8E+06	7_810191	12	44	18	18	28	18	0.129829	1	4	2	27	14	3	21	5.76E-04	0.0398271	17.375	13.4050897								
7	8E+06	7_8145524	485	146	281	174	316	228	1.19E-07	#######	91	188	138	42	140	90	0.02442917	0.7977568	193.13	137.234354					8144030	8145584	0.55554	ID=promoter_46646;
7	8E+06	7_820610	37	21	76	46	29	61	0.031783	1	86	30	26	49	58	38	0.24734975	1	46.375	23.5125347								
7	8E+06	7_827885	36	54	38	26	45	32	0.091522	1	36	21	56	34	29	45	0.09145238	1	37.625	12.1647911								
7	8E+06	7_830594	87	26	50	48	57	49	0.277335	1	0	0	9	13	0	11	0.00265095	0.136745	29.125	30.5026345								
7	8E+06	7_8370805	3	51	12	16	27	14	0.038924	1	7	5	27	50	6	39	1.51E-06	0.0002404	21.375	19.4858007					8370109	8370885	0.12937	ID=promoter_46663;
7	8E+06	7_842896	21	41	31	36	31	34	0.964086	1	43	48	63	2	46	33	0.5299474	1	35.625	18.3298157								
7	9E+06	7_8766494	156	71	181	174	114	178	0.057111	1	38	57	56	9	48	33	0.41465317	1	92.75	67.085766					8766118	8768919	0.75018	ID=promoter_46669;
7	9E+06	7_879613	37	36	11	12	37	12	1.88E-04	0.01539	48	2	57	18	25	38	0.15239423	1	27.625	19.6609657								
7	9E+06	7_881124	19	64	23	69	42	46	0.9497	1	78	35	56	41	57	49	0.9276628	1	48.125	21.8595353								

7	9E+06	7_8898548	36	22	36	12	29	24	0.446278	1	0	1	5	0	1	3	0.56022579	1	14	15.4827092	9E+06	8914503	ID=ENSP0000382395;HIVEP1;NM_002114;	HIVEP1				
7	9E+06	7_904505	1	35	5	4	18	5	0.008132	0.34618	11	40	31	7	26	19	0.73188825	1	16.75	15.8271918								
7	9E+06	7_908138	8	55	11	30	32	21	0.11093	1	0	33	29	21	17	25	0.33405521	1	23.375	17.3611841								
7	9E+06	7_924499	114	57	58	63	86	61	0.009946	0.40658	40	41	40	5	41	23	0.14981223	1	52.25	30.8070029								
7	9E+06	7_924981	31	87	56	44	59	50	0.236381	1	9	19	26	77	14	52	3.94E-06	0.000557	43.625	27.8359352								
7	9E+06	7_931980	14	0	5	15	7	10	0.922856	1	29	0	0	0	15	0	9.56E-04	0.060345	7.875	10.6293059								
7	9E+06	7_9374991	673	577	613	669	625	641	0.057506	1	633	361	370	253	497	312	3.93E-07	7.24E-05	518.63	164.46097					9374693	9375814	0.17231	ID=promoter_46688;
7	9E+06	7_9425796	66	82	74	62	74	68	0.346164	1	34	64	105	6	49	56	0.56303155	1	61.625	30.0757971					9425158	9425825	0.10499	ID=promoter_46689;
7	1E+07	7_953426	89	13	88	34	51	61	0.99472	1	142	81	49	30	112	40	5.27E-07	9.36E-05	65.75	42.1417675								
7	1E+07	7_955756	99	31	56	119	65	88	0.680328	1	0	9	15	8	5	12	0.17891578	1	42.125	45.0917715								
7	1E+07	7_961029	2	35	12	17	19	15	0.513973	1	39	40	21	82	40	52	0.27124824	1	31	24.6692405								
7	1E+07	7_962200	5	4	11	1	5	6	0.999292	1	5	12	9	16	9	13	0.58766318	1	7.875	4.96955014								
7	1E+07	7_974728	15	0	10	8	8	9	0.996316	1	0	11	0	1	6	1	0.20114407	1	5.625	6.06953752								
7	1E+07	7_977521	22	12	4	2	17	3	0.002206	0.1213	20	0	2	1	10	2	0.09294023	1	7.875	8.91928088								
7	1E+07	7_982138	51	17	35	36	34	36	0.912382	1	36	14	0	0	25	0	9.28E-06	0.0011901	23.625	18.6619361								
7	1E+07	7_984718	46	42	25	23	44	24	0.008407	0.35544	15	11	24	38	13	31	0.01226355	0.4638477	28	12.6942057								
7	1E+07	7_991853	4	0	5	3	2	4	0.823343	1	2	13	17	4	8	11	0.69450319	1	6	5.85540044								
7	1E+07	7_995494	2	9	13	1	6	7	0.999953	1	30	27	17	10	29	14	0.12465658	1	13.625	10.6091268								
7	1E+07	7_100330	10	27	7	12	19	10	0.108738	1	0	5	39	1	3	20	9.56E-04	0.060345	12.625	13.5955613								
7	1E+07	7_103075	2	7	3	4	5	4	0.848147	1	45	30	7	0	38	4	3.64E-06	0.0005208	12.25	16.263456								
7	1E+07	7_10328974	1	21	24	4	11	14	0.979671	1	1	15	19	1	8	10	0.81871774	1	10.75	9.98212688	1E+07	1E+07	ID=ENSP0000368468;CCDC90A;NM_001031713;	CCDC90A				
7	1E+07	7_104188	138	118	74	145	128	110	0.055825	1	119	97	147	115	108	131	0.11627235	1	119.13	24.9195133								
7	1E+07	7_105162	15	8	30	8	12	19	0.716431	1	11	8	10	5	10	8	0.95278883	1	11.875	7.88194139								
7	1E+07	7_10589400	8	1	16	10	5	13	0.299488	1	1	6	2	0	4	1	0.45332778	1	5.5	5.60611911	1E+07	1.1E+07	ID=ENSP0000368450;CD83;NM_004233;	CD83	1.1E+07	1.1E+07	0.12384	ID=promoter_46728;
7	1E+07	7_108269	3	16	4	18	10	11	0.989255	1	0	0	0	13	0	7	0.02292696	0.7568963	6.75	7.64852927								
7	1E+07	7_11022222	171	196	211	132	184	172	0.100298	1	154	54	71	78	104	75	0.25119925	1	133.38	59.8186247					1.1E+07	1.1E+07	0.11231	ID=promoter_46749;
7	1E+07	7_110294	13	54	32	18	34	25	0.201935	1	2	50	51	29	26	40	0.12680108	1	31.125	19.3792046								
7	1E+07	7_110622	21	32	40	14	27	27	0.841861	1	0	13	9	19	7	14	0.23485029	1	18.5	12.7279221								
7	1E+07	7_110831	5	22	2	7	14	5	0.053576	1	10	0	1	1	5	1	0.30486099	1	6	7.32900305								
7	1E+07	7_110989	57	12	80	54	35	67	0.059993	1	0	0	0	0	0	0	1	1	25.375	32.8587344								
7	1E+07	7_111184	20	45	20	25	33	23	0.152883	1	3	5	14	2	4	8	0.43697044	1	16.75	14.340253								
7	1E+07	7_111329	5	35	13	23	20	18	0.715835	1	62	53	37	20	58	29	0.02229888	0.7455867	31	19.5740353								
7	1E+07	7_111567	8	22	18	6	15	12	0.610117	1	83	40	94	26	62	60	0.97224477	1	37.125	33.557786								
7	1E+07	7_111931	107	27	63	30	67	47	0.023969	0.81581	20	17	13	4	19	9	0.23949138	1	35.125	33.9134929								
7	1E+07	7_11251774	38	22	23	34	30	29	0.758275	1	49	33	29	1	41	15	0.00610396	0.2666968	28.625	14.0909039					1.1E+07	1.1E+07	0.12777	ID=promoter_46765;
7	1E+07	7_112865	23	56	22	4	40	13	9.58E-05	0.00859	11	30	33	17	21	25	0.69849299	1	24.5	15.8655062								
7	1E+07	7_113349	15	0	8	4	8	6	0.705675	1	0	14	13	0	7	7	0.99000271	1	6.75	6.60627407								
7	1E+07	7_113633	19	16	25	17	18	21	0.99986	1	43	4	31	37	24	34	0.27690884	1	24	12.6378117								
7	1E+07	7_114165	161	32	136	89	97	113	0.998777	1	34	49	24	4	42	14	0.00250702	0.1318429	66.125	56.7335565								
7	1E+07	7_114195	13	76	36	21	45	29	0.038455	1	42	39	77	58	41	68	0.01167006	0.4445799	45.25	23.5417562								
7	1E+07	7_114348	9	35	3	11	22	7	0.005528	0.25484	5	13	15	49	9	32	6.35E-04	0.0429702	17.5	16.0623784								
7	1E+07	7_114671	23	17	2	8	20	5	0.002998	0.15536	2	20	20	0	11	10	0.99899888	1	11.5	9.50187951								
7	1E+07	7_115567	76	39	59	77	58	68	0.999997	1	96	41	29	10	69	20	8.18E-06	0.0010647	53.375	28.670231								
7	1E+07	7_116529	11	28	23	31	20	27	0.892511	1	30	4	4	2	17	3	0.01239331	0.4669004	16.625	12.6484048								
7	1E+07	7_117172	357	74	256	228	216	242	0.886859	1	113	78	88	27	96	58	0.03456711	1	152.63	114.277779								
7	1E+07	7_117384	78	33	37	76	56	57	0.752336	1	63	45	40	11	54	26	0.02018388	0.6864966	47.875	22.9809704								
7	1E+07	7_117792	16	59	1	6	38	4	5.55E-08	#######	48	11	27	0	30	14	0.09649838	1	21	22.0389266								
7	1E+07	7_118246	10	9	13	1	10	7	0.570701	1	0	0	16	0	0	8	0.01336935	0.4945372	6.125	6.6211242								
7	1E+07	7_11925056	20	3	33	29	12	31	0.05845	1	1	0	11	2	1	7	0.08320466	1	12.375	13.3088317	1E+07	1.2E+07	ID=ENSP0000380478;JARID2;;	JARID2	1.2E+07	1.2E+07	0.16031	ID=promoter_46796;
7	1E+07	7_11948881	10	8	22	31	9	27	0.040696	1	3	27	20	13	15	17	0.85096642	1	16.75	9.79431322	1E+07	1.2E+07	ID=ENSP0000380478;JARID2;;	JARID2				

7	1E+07	7_11991352	103	67	61	81	85	71	0.105302	1	18	18	19	0	18	10	0.42261869	1	45.875	36.9417979	1E+07	1.2E+07	ID=ENSP00000380478;JARID2;;	JARID2				
7	1E+07	7_12000086	4	40	14	34	22	24	0.97026	1	89	17	26	7	53	17	3.51E-04	0.0264748	28.875	27.3414259	1E+07	1.2E+07	ID=ENSP00000380478;JARID2;;	JARID2	1.2E+07	1.2E+07	0.22477	ID=promoter_46810;
7	1E+07	7_12017263	8	23	6	25	16	16	0.903021	1	1	2	19	24	2	22	1.09E-04	0.0098689	13.5	10.2678416	1E+07	1.2E+07	ID=ENSP00000348183;DTNBP1;;	DTNBP1	1.2E+07	1.2E+07	0.40362	ID=promoter_46816;
7	1E+07	7_121462	10	91	18	3	51	11	7.64E-08	#######	0	18	10	19	9	15	0.36943449	1	21.125	29.1029576								
7	1E+07	7_125037	260	270	324	179	265	252	0.05762	1	231	203	230	31	217	131	4.85E-04	0.0346769	216	86.7673408								
7	1E+07	7_12545828	2136	1215	2394	1801	1676	2098	0.139192	1	1152	1070	1036	349	1111	693	1.81E-15	9.54E-13	1394.1	668.899189					1.3E+07	1.3E+07	0.49077	ID=promoter_46824;
7	1E+07	7_125687	21	18	7	9	20	8	0.026297	0.87605	1	9	28	33	5	31	3.00E-05	0.0032574	15.75	11.119481								
7	1E+07	7_12698736	69	28	46	39	49	43	0.378786	1	11	6	34	31	9	33	4.07E-04	0.0298473	33	19.7989899	1E+07	1.3E+07	ID=ENSP00000259727;GMPR;NM_006877;	GMPR				
7	1E+07	7_12735201	24	27	40	22	26	31	0.998108	1	1	0	0	5	1	3	0.56022579	1	14.875	15.3291273					1.3E+07	1.3E+07	0.26455	ID=promoter_46831;
7	1E+07	7_127719	8	14	33	46	11	40	0.002161	0.11921	0	2	6	4	1	5	0.22510075	1	14.125	16.5825855								
7	1E+07	7_12792734	2	4	10	6	3	8	0.459247	1	4	11	31	7	8	19	0.06853691	1	9.375	9.25723655					1.3E+07	1.3E+07	0.11947	ID=promoter_46835;
7	1E+07	7_128877	4	20	1	4	12	3	0.030625	0.98374	36	4	4	9	20	7	0.06990458	1	10.25	11.9612469								
7	1E+07	7_131718	145	46	117	84	96	101	0.747258	1	46	64	77	0	55	39	0.43845264	1	72.375	44.9155954								
7	1E+07	7_133058	1	0	4	0	1	2	0.907382	1	21	0	14	0	11	7	0.73613127	1	5	8.05339325								
7	1E+07	7_134403	3	39	14	17	21	16	0.42382	1	10	32	64	25	21	45	0.0048577	0.2226648	25.5	19.4862589								
7	1E+07	7_134751	9	9	25	3	9	14	0.802715	1	0	20	8	0	10	4	0.34296513	1	9.25	9.06720937								
7	1E+07	7_13567930	599	242	467	446	421	457	0.517897	1	204	135	244	128	170	186	0.29458773	1	308.13	173.453524	1E+07	1.4E+07	ID=ENSP00000368341;RBM24;NM_001143942;	RBM24				
7	1E+07	7_13605791	719	204	99	100	462	100	4.71E-65	#######	294	219	33	28	257	31	1.16E-35	1.51E-32	212	225.228773					1.4E+07	1.4E+07	0.30991	ID=promoter_46852;
7	1E+07	7_136115	39	89	25	19	64	22	8.66E-07	0.00013	89	26	86	5	58	46	0.73126527	1	47.25	35.021422								
7	1E+07	7_139355	3	33	2	7	18	5	0.008132	0.34618	1	35	15	2	18	9	0.30718372	1	12.25	14.1698876								
7	1E+07	7_14482611	40	29	47	29	35	38	0.947014	1	11	15	32	2	13	17	0.64388579	1	25.625	15.1745793					1.4E+07	1.4E+07	0.557	ID=promoter_46869;
7	1E+07	7_146594	34	10	21	15	22	18	0.522468	1	1	33	18	6	17	12	0.76881979	1	17.25	11.9013805								
7	2E+07	7_157790	1	52	1	31	27	16	0.087673	1	19	7	11	18	13	15	0.84581615	1	17.5	17.2046505								
7	2E+07	7_158904	421	210	209	221	316	215	3.57E-09	#######	105	160	221	12	133	117	0.91801578	1	194.88	116.902203								
7	2E+07	7_16505261	607	265	499	384	436	442	0.096433	1	280	210	168	87	245	128	6.75E-07	0.0001172	312.5	174.099315					1.7E+07	1.7E+07	0.53098	ID=promoter_46885;
7	2E+07	7_165414	3	62	31	36	33	34	0.868497	1	24	56	17	14	40	16	0.01383566	0.5095422	30.375	20.4446116								
7	2E+07	7_166867	6	23	10	17	15	14	0.827043	1	20	17	12	1	19	7	0.09662636	1	13.25	7.40173725								
7	2E+07	7_167695	9	0	6	6	5	6	0.999292	1	2	16	32	1	9	17	0.21129426	1	9	10.6234243								
7	2E+07	7_173200	2	2	9	1	2	5	0.651182	1	1	1	13	15	1	14	0.00214405	0.1161796	5.5	5.90399381								
7	2E+07	7_17602565	181	92	169	137	137	153	0.916649	1	150	78	80	96	114	88	0.4339217	1	122.88	41.350203					1.8E+07	1.8E+07	0.85801	ID=promoter_46890;
7	2E+07	7_17603807	756	272	682	475	514	579	0.80032	1	244	300	203	63	272	133	6.14E-09	1.58E-06	374.38	241.93561	2E+07	1.8E+07	ID=ENSP00000244745;SOX4;NM_003107;	SOX4	1.8E+07	1.8E+07	0.85801	ID=promoter_46890;
7	2E+07	7_176277	29	28	16	10	29	13	0.011304	0.44898	15	17	16	41	16	29	0.09020737	1	21.5	10.2399777								
7	2E+07	7_17654924	5666	5735	297	225	5701	261	0	0	8599	4943	189	109	6771	149	0	0	3220.4	3393.05479					1.8E+07	1.8E+07	0.63416	ID=promoter_46891;
7	2E+07	7_178682	7	14	33	8	11	21	0.417506	1	18	21	31	16	20	24	0.69398062	1	18.5	9.57675758								
7	2E+07	7_178895	13	29	23	24	21	24	0.996223	1	40	30	30	11	35	21	0.28311174	1	25	9.53190132								
7	2E+07	7_20079749	5	33	20	23	19	22	0.999149	1	44	6	3	40	25	22	0.9842524	1	21.75	16.2106667	2E+07	2E+07	ID=ENSP00000367715;DCDC2;NM_016356;	DCDC2				

7	2E+07	7_213494	9	3	1	4	6	3	0.462004	1	0	6	29	19	3	24	1.24E-04	0.0109901	8.875	10.1056915								
7	2E+07	7_21443690	35	37	26	51	36	39	0.942553	1	11	69	28	17	40	23	0.18311734	1	34.25	18.6758056	2E+07	2.2E+07	ID=ENSP0000331983;LRRC16A;;	LRRC16A				
7	2E+07	7_21648139	3	14	11	8	9	10	0.993074	1	8	32	20	17	20	19	0.99708906	1	14.125	9.0307016	2E+07	2.2E+07	ID=ENSP0000367062-D15;HIST1H3D;;	HIST1H3D	2.2E+07	2.2E+07	0.20381	ID=promoter_46919;
7	2E+07	7_218730	1	29	5	1	15	3	0.006447	0.2884	0	1	1	2	1	2	0.81304206	1	5	9.81252843								
7	2E+07	7_21924596	673	230	469	385	452	427	0.005728	0.26298	212	190	203	43	201	123	0.00125874	0.075413	300.63	198.247275					2.2E+07	2.2E+07	0.27445	ID=promoter_46926;
7	2E+07	7_21932745	10	5	19	4	8	12	0.864244	1	2	12	10	3	7	7	0.99000271	1	8.125	5.74300817					2.2E+07	2.2E+07	0.38886	ID=promoter_46927;
7	2E+07	7_220001	235	30	199	117	133	158	0.994174	1	87	56	34	39	72	37	0.01254306	0.4715466	99.625	78.7363367								
7	2E+07	7_22314951	389	92	326	280	241	303	0.723622	1	244	217	103	51	231	77	4.96E-15	2.53E-12	212.75	120.8278					2.2E+07	2.2E+07	0.55184	ID=promoter_46952;
7	2E+07	7_22557218	324	103	279	220	214	250	0.999059	1	256	95	88	84	176	86	4.76E-06	0.0006599	181.13	99.0936606					2.3E+07	2.3E+07	0.95159	ID=promoter_46965;
7	2E+07	7_22559190	2883	1546	2468	2316	2215	2392	0.019643	0.69635	1293	859	1166	848	1076	1007	0.97689834	1	1672.4	781.058794					2.3E+07	2.3E+07	0.95159	ID=promoter_46965;
7	2E+07	7_22560334	6071	2263	5656	3617	4167	4637	0.047687	1	1910	1449	1807	1075	1680	1441	0.09605978	1	2981	1932.02048					2.3E+07	2.3E+07	0.95159	ID=promoter_46965;
7	2E+07	7_225673	299	176	304	198	238	251	0.500866	1	113	126	104	88	120	96	0.5597059	1	176	85.6854713								
7	2E+07	7_228204	246	109	136	103	178	120	1.27E-05	0.00148	142	70	71	22	106	47	9.47E-05	0.0087745	112.38	66.6181818								
7	2E+07	7_228790	1	29	5	1	15	3	0.006447	0.2884	3	17	55	21	10	38	9.38E-05	0.0087137	16.5	18.7235528								
7	2E+07	7_228940	16	43	15	16	30	16	0.032973	1	28	15	26	26	22	26	0.70257106	1	23.125	9.77515071								
7	2E+07	7_235421	32	26	25	25	29	25	0.526429	1	28	37	41	2	33	22	0.4848075	1	27	11.6863779								
7	2E+07	7_23618711	22	7	16	18	15	17	0.995306	1	17	0	3	4	9	4	0.4571786	1	10.875	8.28833087					2.4E+07	2.4E+07	0.21665	ID=promoter_47018;
7	2E+07	7_23912983	175	58	127	137	117	132	0.954157	1	63	25	60	21	44	41	0.99972002	1	83.25	56.1598484					2.4E+07	2.4E+07	0.36336	ID=promoter_47037;
7	2E+07	7_23919119	77	24	44	44	51	44	0.325273	1	33	5	23	19	19	21	0.85675254	1	33.625	21.830107					2.4E+07	2.4E+07	0.17797	ID=promoter_47038;
7	2E+07	7_23949317	15	25	9	27	20	18	0.715835	1	34	17	5	0	26	3	2.53E-04	0.0200383	16.5	11.6619038					2.4E+07	2.4E+07	0.13846	ID=promoter_47041;
7	2E+07	7_24114646	16181	4484	13307	8621	10333	####	2.40E-12	#######	7398	3546	4145	1832	5472	2989	6.18E-121	2.60E-117	7439.3	5048.77852					2.4E+07	2.4E+07	0.5183	ID=promoter_47048;
7	2E+07	7_244753	0	28	5	0	14	3	0.010924	0.43763	24	9	9	3	17	6	0.10713464	1	9.75	10.6603404								
7	2E+07	7_245842	13	15	17	18	14	18	0.967347	1	20	20	16	0	20	8	0.11762508	1	14.875	6.46832944								
7	2E+07	7_24835331	254	146	221	248	200	235	0.999836	1	81	79	96	57	80	77	0.97273869	1	147.75	81.7692747					2.5E+07	2.5E+07	0.17935	ID=promoter_47091;
7	2E+07	7_24842383	968	690	1105	994	829	1050	0.254907	1	519	511	856	266	515	561	0.03016923	0.9350227	738.63	290.719274					2.5E+07	2.5E+07	0.3176	ID=promoter_47092;
7	2E+07	7_249317	101	95	91	71	98	81	0.064264	1	99	85	76	4	92	40	2.29E-04	0.0185569	77.75	31.6351974								
7	3E+07	7_256085	31	40	24	16	36	20	0.023274	0.79554	19	12	5	3	16	4	0.04223275	1	18.75	12.6462869								
7	3E+07	7_257067	87	42	17	41	65	29	4.02E-05	0.00402	39	10	38	36	25	37	0.18968026	1	38.75	22.8395021								
7	3E+07	7_26706745	42	0	12	2	21	7	0.0088	0.36783	0	9	0	0	5	0	0.09850879	1	8.125	14.4660934	3E+07	2.7E+07	ID=ENSP0000365694;PPP1R10;NM_002714;	PPP1R10				
7	3E+07	7_26720201	275	171	204	272	223	238	0.598312	1	107	85	41	26	96	34	3.55E-06	0.0005089	147.63	97.9444996					2.7E+07	2.7E+07	0.45618	ID=promoter_47115;
7	3E+07	7_26819052	349	83	286	280	216	283	0.472089	1	82	82	90	31	82	61	0.42715115	1	160.38	122.843614					2.7E+07	2.7E+07	0.38856	ID=promoter_47125;
7	3E+07	7_26944763	351	143	173	122	247	148	3.86E-10	#######	146	110	54	23	128	39	1.26E-09	3.63E-07	140.25	98.5346205					2.7E+07	2.7E+07	0.47622	ID=promoter_47133;

7	3E+07	7_27027321	305	145	229	171	225	200	0.016617	0.61009	153	92	124	28	123	76	0.01985888	0.6765708	155.88	83.9973426					2.7E+07	2.7E+07	0.45618	ID=promoter_47143;
7	3E+07	7_270436	1	10	9	9	6	9	0.896349	1	0	0	25	8	0	17	1.04E-04	0.0094884	7.75	8.20713975								
7	3E+07	7_27080482	188	70	163	157	129	160	0.894618	1	118	66	62	0	92	31	2.44E-06	0.0003679	103	64.0468578					2.7E+07	2.7E+07	0.38453	ID=promoter_47148;
7	3E+07	7_27205278	375	176	195	175	276	185	1.86E-08	#######	206	114	73	16	160	45	6.32E-13	2.69E-10	166.25	107.008344					2.7E+07	2.7E+07	0.48048	ID=promoter_47159;
7	3E+07	7_273795	16	20	8	15	18	12	0.307258	1	0	0	14	17	0	16	1.79E-04	0.0149638	11.25	7.72287881								
7	3E+07	7_274452	3	1	9	1	2	5	0.651182	1	21	34	8	0	28	4	2.96E-04	0.0229072	9.625	12.0230434								
7	3E+07	7_27459159	48	67	95	79	58	87	0.347226	1	133	80	70	22	107	46	4.66E-05	0.0048006	74.25	32.5740914					2.7E+07	2.7E+07	0.29767	ID=promoter_47166;
7	3E+07	7_276466	39	19	35	38	29	37	0.9441	1	0	10	3	0	5	2	0.58563225	1	18	17.1880357								
7	3E+07	7_27733404	13	18	15	42	16	29	0.370878	1	40	56	88	3	48	46	0.98714075	1	34.375	28.0761083	3E+07	2.8E+07	ID=ENSP00000365076;;NM_021184;					
7	3E+07	7_27873254	16	32	5	9	24	7	0.00213	0.11798	4	20	17	2	12	10	0.96947392	1	13.125	10.1198179	3E+07	2.8E+07	ID=ENSP00000364543;;NM_006929;		2.8E+07	2.8E+07	0.17341	ID=promoter_47196;
7	3E+07	7_27903340	21	23	73	42	22	58	0.003994	0.19603	31	19	29	31	25	30	0.63340892	1	33.625	17.4923453	3E+07	2.8E+07	ID=ENSP00000364444;C4A;NM_001002029;	C4A				
7	3E+07	7_28153814	96	58	89	68	77	79	0.704691	1	68	65	47	42	67	45	0.24411887	1	66.625	18.6542795	3E+07	2.8E+07	ID=ENSP00000355238-D3;BTN3A3;NM_006994;	BTN3A3				
7	3E+07	7_283717	22	32	9	15	27	12	0.014569	0.54886	22	25	19	14	24	17	0.69596948	1	19.75	7.16638981								
7	3E+07	7_288618	42	0	0	0	21	0	4.48E-06	0.00059	37	3	10	1	20	6	0.03829208	1	11.625	17.5738239								
7	3E+07	7_290885	74	62	55	76	68	66	0.547503	1	133	73	97	17	103	57	0.00678663	0.2903848	73.375	33.3078176								
7	3E+07	7_293980	8	75	17	8	42	13	3.75E-05	0.00379	32	34	101	29	33	65	0.00148024	0.0866909	38	33.1920817								
7	3E+07	7_29579237	85	38	535	310	62	423	1.47E-47	#######	63	36	153	33	50	93	2.73E-04	0.0212771	156.63	179.087319					3E+07	3E+07	0.25911	ID=promoter_47241;
7	3E+07	7_29618027	260	105	240	200	183	220	0.970185	1	86	80	101	48	83	75	0.98717408	1	140	80.822557	3E+07	3E+07	ID=ENSP00000363817;;;		3E+07	3E+07	0.39301	ID=promoter_47244;
7	3E+07	7_296836	7	30	6	1	19	4	0.002035	0.11403	11	13	4	1	12	3	0.09316162	1	9.125	9.46327488								
7	3E+07	7_298885	69	29	74	45	49	60	0.97537	1	16	19	14	3	18	9	0.30718372	1	33.625	26.4031248								
7	3E+07	7_30027815	32	45	35	31	39	33	0.382234	1	4	32	26	38	18	32	0.07969995	1	30.375	12.0349194	3E+07	3E+07	ID=ENSP00000381654-D4;HMGCLL1;NM_019036;	HMGCLL1				
7	3E+07	7_30071649	19	37	19	26	28	23	0.444971	1	57	5	33	34	31	34	0.79475598	1	28.75	15.4619348	3E+07	3E+07	ID=ENSP00000381654-D4;HMGCLL1;NM_019036;	HMGCLL1				
7	3E+07	7_31317270	0	16	0	13	8	7	0.850897	1	1	10	3	16	6	10	0.51486293	1	7.375	7.1301673	3E+07	3.1E+07	ID=ENSP00000359925;LRRC1;NM_018214;	LRRC1				
7	3E+07	7_318581	109	71	104	45	90	75	0.089486	1	102	50	76	71	76	74	0.95582697	1	78.5	24.4540385								
7	3E+07	7_320611	1	19	6	0	10	3	0.082185	1	60	52	24	0	56	12	3.25E-06	0.0004735	20.25	23.8971008								
7	3E+07	7_324197	2221	1325	2548	2486	1773	2517	5.57E-09	#######	1544	1111	2235	825	1328	1530	4.77E-08	1.05E-05	1786.9	666.72921								
7	3E+07	7_328628	166	93	152	145	130	149	0.981625	1	46	19	24	6	33	15	0.0650987	1	81.375	65.9522284								
7	3E+07	7_330730	36	13	18	23	25	21	0.526204	1	1	39	40	40	20	40	0.01593163	0.5684069	26.25	14.7720779								
7	3E+07	7_33074497	11	69	12	22	40	17	0.001238	0.07553	9	0	52	24	5	38	8.63E-07	0.0001467	24.875	23.6669601					3.3E+07	3.3E+07	0.15605	ID=promoter_47272;
7	3E+07	7_33330528	420	122	217	233	271	225	6.04E-04	0.04138	91	67	98	100	79	99	0.13382452	1	168.5	118.260004					3.3E+07	3.3E+07	0.24583	ID=promoter_47278;
7	3E+07	7_334003	25	74	60	19	50	40	0.192624	1	326	231	315	108	279	212	0.09162737	1	144.75	127.036272								

7	3E+07	7_33474073	88	91	87	73	90	80	0.194187	1	43	97	99	12	70	56	0.71281462	1	73.75	30.7373667	3E+07	3.4E+07	ID=ENSP00000359805;DST;;	DST				
7	3E+07	7_33518906	22	16	20	22	19	21	0.982527	1	0	50	22	19	25	21	0.94599092	1	21.375	13.7002346	3E+07	3.4E+07	ID=ENSP00000359805;DST;;	DST				
7	3E+07	7_33523628	8	9	0	5	9	3	0.131542	1	0	3	3	18	2	11	0.03110435	0.956324	5.75	5.94618725	3E+07	3.4E+07	ID=ENSP00000359805;DST;;	DST				
7	3E+07	7_33550242	97	70	67	49	84	58	0.007221	0.31244	43	11	62	36	27	49	0.01695111	0.6008545	54.375	25.7900174	3E+07	3.4E+07	ID=ENSP00000359805;DST;;	DST	3.4E+07	3.4E+07	0.38266	ID=promoter_47289;
7	3E+07	7_33562674	13	27	22	27	20	25	0.977759	1	22	6	9	1	14	5	0.16413709	1	15.875	9.9776536	3E+07	3.4E+07	ID=ENSP00000359805;DST;;	DST	3.4E+07	3.4E+07	0.34936	ID=promoter_47291;
7	3E+07	7_33638007	0	0	5	0	0	3	0.278365	1	1	35	0	0	18	0	2.38E-04	0.0190698	5.125	12.1941145	3E+07	3.4E+07	ID=ENSP00000359805;DST;;	DST				
7	3E+07	7_338940	834	767	923	1242	801	1083	0.009496	0.39165	484	506	1339	228	495	784	4.39E-20	3.00E-17	790.38	380.975041								
7	3E+07	7_34042185	2	16	25	16	9	21	0.212308	1	1	22	36	3	12	20	0.26508384	1	15.125	12.5406482	3E+07	3.4E+07	ID=ENSP00000293739;;					
7	3E+07	7_34054728	9	30	22	27	20	25	0.977759	1	100	44	44	34	72	39	0.0237312	0.7793027	38.75	27.2802912	3E+07	3.4E+07	ID=ENSP00000363746;;NM_005452;					
7	3E+07	7_34059310	53	74	15	24	64	20	2.07E-07	#######	208	38	46	62	123	54	1.70E-05	0.0020144	65	60.8910033	3E+07	3.4E+07	ID=ENSP00000363746;;NM_005452;					
7	3E+07	7_34121527	279	181	276	215	230	246	0.602668	1	150	93	89	11	122	50	3.78E-06	0.0005365	161.75	94.7850953					3.4E+07	3.4E+07	0.37655	ID=promoter_47334;
7	3E+07	7_341837	19	34	6	5	27	6	2.01E-04	0.01628	1	6	17	0	4	9	0.31291743	1	11	11.5634893								
7	3E+07	7_344542	20	27	13	6	24	10	0.016074	0.59429	0	21	10	2	11	6	0.57179039	1	12.375	9.66492185								
7	3E+07	7_344899	138	96	195	191	117	193	0.013898	0.52987	25	41	33	36	33	35	0.85720038	1	94.375	71.9164942								
7	3E+07	7_34613449	149	30	215	198	90	207	3.86E-07	#######	47	21	30	31	34	31	0.99776322	1	90.125	82.8603602	3E+07	3.5E+07	ID=ENSP00000363296;GRM4;NM_000841;	GRM4	3.5E+07	3.5E+07	0.2052	ID=promoter_47375;
7	3E+07	7_34674061	743	388	626	450	566	538	0.0022	0.12121	371	258	286	84	315	185	4.31E-06	0.0006021	400.75	208.813485	3E+07	3.5E+07	ID=ENSP00000363296;GRM4;NM_000841;	GRM4				
7	3E+07	7_348933	8	28	10	1	18	6	0.017303	0.62786	8	42	21	14	25	18	0.71458637	1	16.5	13.2880182								
7	4E+07	7_35070900	9	20	31	24	15	28	0.341844	1	14	41	11	14	28	13	0.1098583	1	20.5	11.0194633					3.5E+07	3.5E+07	0.47026	ID=promoter_47396;
7	4E+07	7_35275381	1320	426	1314	943	873	1129	0.095561	1	552	441	427	212	497	320	2.09E-06	0.0003237	704.38	430.452565					3.5E+07	3.5E+07	0.39096	ID=promoter_47403;
7	4E+07	7_35421231	30	109	51	25	70	38	4.63E-04	0.0332	80	300	23	27	190	25	1.30E-25	1.17E-22	80.625	93.8554169	4E+07	3.5E+07	ID=ENSP00000367540;;					
7	4E+07	7_35426893	87	85	54	86	86	70	0.073928	1	56	62	106	73	59	90	0.01097962	0.4242766	76.125	18.0905461	4E+07	3.5E+07	ID=ENSP00000367540;;					
7	4E+07	7_355537	4	19	2	16	12	9	0.592799	1	8	14	10	2	11	6	0.57179039	1	9.375	6.52330985								
7	4E+07	7_355538	17	1	20	9	9	15	0.704209	1	0	12	6	0	6	3	0.67473421	1	8.125	7.77243297								
7	4E+07	7_356631	69	15	49	81	42	65	0.372885	1	58	7	5	0	33	3	1.11E-05	0.0013866	35.5	32.302145								
7	4E+07	7_357650	245	12	2	22	129	12	3.30E-26	#######	77	7	23	13	42	18	0.01944768	0.6639875	50.125	82.1434938								
7	4E+07	7_36118588	17	33	34	29	25	32	0.94785	1	15	1	36	15	8	26	0.00417605	0.1967234	22.5	12.3750902	4E+07	3.6E+07	ID=ENSP00000349811;FKBP5;NM_004117;	FKBP5				
7	4E+07	7_36201959	474	171	406	320	323	363	0.855229	1	81	93	116	21	87	69	0.62533455	1	210.25	167.596241					3.6E+07	3.6E+07	0.28829	ID=promoter_47450;
7	4E+07	7_362168	7	0	13	0	4	7	0.813458	1	0	16	10	2	8	6	0.92562801	1	6	6.43650304								
7	4E+07	7_362206	8	5	5	5	7	5	0.693374	1	21	0	16	0	11	8	0.87190251	1	7.5	7.42582367								
7	4E+07	7_366955	94	34	94	95	64	95	0.344729	1	27	20	52	9	24	31	0.47191283	1	53.125	36.2311527								
7	4E+07	7_36776687	7	23	5	7	15	6	0.065303	1	24	46	11	2	35	7	2.45E-04	0.0195648	15.625	14.7157593					3.7E+07	3.7E+07	0.61217	ID=promoter_47462;

7	4E+07	7_367772	462	338	323	424	400	374	0.006809	0.29841	185	245	235	75	215	155	0.05712104	1	285.88	127.108994								
7	4E+07	7_37176964	8	31	12	8	20	10	0.076235	1	18	4	14	2	11	8	0.87190251	1	12.125	9.23405962					3.7E+07	3.7E+07	0.44336	ID=promoter_47470;
7	4E+07	7_373212	152	123	191	164	138	178	0.703932	1	82	80	36	25	81	31	8.60E-05	0.008132	106.63	60.5968823								
7	4E+07	7_37482037	84	146	204	186	115	195	0.006954	0.30307	125	60	119	2	93	61	0.10787957	1	115.75	66.3211882	4E+07	3.8E+07	ID=ENSP0000377964;TBC1D22A;;	TBC1D22A				
7	4E+07	7_37516595	4	21	4	13	13	9	0.46931	1	7	21	5	1	14	3	0.04246461	1	9.5	7.8921299	4E+07	3.8E+07	ID=ENSP0000377964;TBC1D22A;;	TBC1D22A				
7	4E+07	7_377409	157	32	115	125	95	120	0.864507	1	18	9	30	23	14	27	0.07530308	1	63.625	58.5196853								
7	4E+07	7_38670887	239	106	222	254	173	238	0.279078	1	73	70	27	42	72	35	0.00621759	0.269925	129.13	93.7267114					3.9E+07	3.9E+07	0.18961	ID=promoter_47495;
7	4E+07	7_386842	8	13	7	10	11	9	0.722819	1	20	6	4	31	13	18	0.54030568	1	12.375	9.02278861								
7	4E+07	7_386923	34	32	14	32	33	23	0.152883	1	21	42	26	27	32	27	0.9371194	1	28.5	8.55235974								
7	4E+07	7_387273	0	23	0	15	12	8	0.45534	1	1	6	3	3	4	3	0.96209564	1	6.375	8.31414457								
7	4E+07	7_389512	34	107	55	46	71	51	0.026233	0.87605	69	28	49	97	49	73	0.03468232	1	60.625	28.5303723								
7	4E+07	7_389593	34	31	21	32	33	27	0.37765	1	36	39	59	50	38	55	0.10359486	1	37.75	11.8291408								
7	4E+07	7_39360405	2	12	10	3	7	7	0.956352	1	0	3	7	8	2	8	0.1303879	1	5.625	4.24053568	4E+07	3.9E+07	ID=ENSP0000333363;DNAH8;;	DNAH8				
7	4E+07	7_39391416	0	43	6	0	22	3	1.41E-04	0.01198	48	12	18	2	30	10	0.01387572	0.5103594	16.125	19.2014694	4E+07	3.9E+07	ID=ENSP0000333363;DNAH8;;	DNAH8				
7	4E+07	7_394437	1	3	8	8	2	8	0.255957	1	14	3	3	0	9	2	0.1390433	1	5	4.65985898								
7	4E+07	7_395338	3	25	22	6	14	14	0.914609	1	0	16	3	2	8	3	0.3843038	1	9.625	9.86968664								
7	4E+07	7_397642	3	20	6	7	12	7	0.325249	1	5	13	8	5	9	7	0.94097808	1	8.375	5.55331304								
7	4E+07	7_397998	16	2	14	8	9	11	0.995818	1	1	13	4	10	7	7	0.99000271	1	8.5	5.70713839								
7	4E+07	7_398743	83	19	17	32	51	25	0.001113	0.06946	27	14	5	9	21	7	0.05007015	1	25.75	24.754509								
7	4E+07	7_399410	11	25	1	16	18	9	0.098614	1	1	0	17	4	1	11	0.01045793	0.4067594	9.375	9.30341719								
7	4E+07	7_402898	142	52	64	60	97	62	7.56E-04	0.04981	0	0	15	5	0	10	0.00454609	0.2107253	42.25	48.5996767								
7	4E+07	7_40339158	0	0	4	0	0	2	0.426327	1	28	7	3	1	18	2	0.00297675	0.1507727	5.375	9.47081985	4E+07	4E+07	ID=ENSP0000381876;DAAM2;NM_015345;	DAAM2				
7	4E+07	7_408009	19	11	36	33	15	35	0.075557	1	40	46	22	27	43	25	0.17340376	1	29.25	11.6588409								
7	4E+07	7_412636	15	11	40	47	13	44	0.002066	0.11504	0	5	2	1	3	2	0.9363008	1	15.125	18.3415025								
7	4E+07	7_414051	5	0	3	24	3	14	0.063635	1	1	8	1	1	5	1	0.30486099	1	5.375	7.98100423								
7	4E+07	7_41473037	7	16	8	5	12	7	0.325249	1	9	11	18	8	10	13	0.72167512	1	10.25	4.52769257					4.1E+07	4.1E+07	0.11934	ID=promoter_47542;
7	4E+07	7_416211	494	226	516	400	360	458	0.514006	1	313	221	302	123	267	213	0.26210206	1	324.38	137.714962								
7	4E+07	7_41635959	52	74	65	31	63	48	0.076128	1	55	31	60	50	43	55	0.28335822	1	52.25	15.172814					4.2E+07	4.2E+07	0.11934	ID=promoter_47548;
7	4E+07	7_416530	17	94	19	24	56	22	2.95E-05	0.00306	51	56	64	80	54	72	0.12739908	1	50.625	28.7647478								
7	4E+07	7_417025	237	106	221	196	172	209	0.942858	1	219	64	67	83	142	75	3.44E-04	0.0260293	149.13	75.7730399								
7	4E+07	7_419817	12	2	11	8	7	10	0.922856	1	0	2	11	2	1	7	0.08320466	1	6	4.98569382								
7	4E+07	7_421189	33	47	13	8	40	11	2.07E-05	0.00227	35	38	20	38	37	29	0.79484841	1	29	13.7113092								
7	4E+07	7_42133202	2941	1487	5461	4807	2214	5134	########	#######	1612	1157	2283	1176	1385	1730	1.09E-15	5.76E-13	2615.5	1673.14742					4.2E+07	4.2E+07	0.39755	ID=promoter_47565;
7	4E+07	7_42301486	400	105	346	286	253	316	0.757751	1	109	59	91	43	84	67	0.65562346	1	179.88	141.011081					4.2E+07	4.2E+07	0.48871	ID=promoter_47575;
7	4E+07	7_42335961	23	13	1	23	18	12	0.307258	1	0	12	7	1	6	4	0.87665919	1	10	9.42640668					4.2E+07	4.2E+07	0.35578	ID=promoter_47576;
7	4E+07	7_424843	2	28	11	11	15	11	0.4902	1	0	2	8	2	1	5	0.22510075	1	8	9.18072515								
7	4E+07	7_426772	73	3	37	51	38	44	0.998299	1	12	18	13	7	15	10	0.7195765	1	26.75	24.696732								
7	4E+07	7_42788294	1594	713	1951	1269	1154	1610	3.95E-05	0.00396	811	578	739	320	695	530	0.00317758	0.1599237	996.88	555.005132					4.3E+07	4.3E+07	0.42059	ID=promoter_47593;
7	4E+07	7_429058	12	21	3	6	17	5	0.013234	0.50759	44	22	21	5	33	13	0.02654774	0.8508557	16.75	13.4774945								
7	4E+07	7_429258	2	20	20	1	11	11	0.932271	1	37	21	7	1	29	4	1.92E-04	0.0158648	13.625	12.9828183								
7	4E+07	7_43028489	364	105	275	207	235	241	0.341353	1	72	133	61	58	103	60	0.01550452	0.5553889	159.38	112.599083					4.3E+07	4.3E+07	0.42169	ID=promoter_47607;
7	4E+07	7_431383	7	12	7	1	10	4	0.162164	1	0	9	11	0	5	6	0.91271846	1	5.875	4.91172068								
7	4E+07	7_433611	100	81	75	55	91	65	0.008562	0.36158	147	105	107	55	126	81	0.03540934	1	90.625	30.7428854								

7	4E+07	7_43447041	16	22	25	37	19	31	0.523429	1	21	39	54	40	30	47	0.07584163	1	31.75	12.8034594	4E+07	4.4E+07	ID=ENSP00000361992;UBR2;;	UBR2				
7	4E+07	7_43818177	199	518	163	214	359	189	6.35E-19	#######	441	416	415	329	429	372	0.6409531	1	336.88	131.156872	4E+07	4.4E+07	ID=ENSP00000375440-D5351;;					
7	4E+07	7_43983141	437	287	371	312	362	342	0.016049	0.59429	280	119	121	129	200	125	0.00234375	0.1251016	257	121.75737					4.4E+07	4.4E+07	0.49665	ID=promoter_47638;
7	4E+07	7_440152	107	60	74	59	84	67	0.060438	1	21	14	32	1	18	17	0.9984894	1	46	35.3351302								
7	4E+07	7_44104438	45	6	37	16	26	27	0.906335	1	62	16	23	0	39	12	0.0020907	0.1142865	25.625	20.9143662	4E+07	4.4E+07	ID=ENSP00000361730-D2;CUL9;;	CUL9				
7	4E+07	7_44113212	105	28	89	74	67	82	0.967241	1	22	12	28	26	17	27	0.21252244	1	48	35.5808937	4E+07	4.4E+07	ID=ENSP00000230431-D2;C6orf108;NM_006443;	C6orf108	4.4E+07	4.4E+07	0.30626	ID=promoter_47646;
7	4E+07	7_44318097	5256	2924	5082	5550	4090	5316	5.08E-06	0.00065	3561	1922	2537	1426	2742	1982	2.84E-16	1.54E-13	3532.3	1596.19483	4E+07	4.4E+07	ID=ENSP00000230431;C6orf108;NM_006443;	C6orf108	4.4E+07	4.4E+07	0.30626	ID=promoter_47659;
7	4E+07	7_44326871	3	0	7	1	2	4	0.823343	1	8	17	14	0	13	7	0.50249747	1	6.25	6.49725217	4E+07	4.4E+07	ID=ENSP00000361730;CUL9;;	CUL9				
7	4E+07	7_44377615	4264	3025	5206	5464	3645	5335	7.15E-24	#######	3241	2283	5249	2053	2762	3651	3.67E-44	6.31E-41	3848.1	1378.84553					4.4E+07	4.4E+07	0.16783	ID=promoter_47667;
7	4E+07	7_444813	66	18	56	23	42	40	0.640089	1	21	11	11	20	16	16	0.97729577	1	28.25	20.8515159								
7	4E+07	7_446752	38	74	22	40	56	31	0.002754	0.14618	14	49	85	23	32	54	0.02438444	0.7977568	43.125	25.2893964								
7	5E+07	7_452010	24	66	39	27	45	33	0.117793	1	73	77	95	14	75	55	0.41657057	1	51.875	29.5994088								
7	5E+07	7_454057	0	3	1	9	2	5	0.651182	1	0	3	5	26	2	16	0.00248877	0.1311269	5.875	8.65922299								
7	5E+07	7_454440	421	245	533	554	333	544	1.08E-05	0.00128	124	112	122	75	118	99	0.7646226	1	273.25	199.77684								
7	5E+07	7_45471069	37	56	37	34	47	36	0.152702	1	85	65	63	5	75	34	0.00193848	0.1068241	47.75	24.5923914					4.5E+07	4.5E+07	0.0906	ID=promoter_47706;
7	5E+07	7_457153	2	1	3	10	2	7	0.358464	1	38	6	31	2	22	17	0.8526593	1	11.625	14.5301411								
7	5E+07	7_45764318	93	893	2103	1336	493	1720	########	#######	80	631	703	320	356	512	1.16E-09	3.37E-07	769.88	683.803739					4.6E+07	4.6E+07	0.4904	ID=promoter_47715;
7	5E+07	7_466263	24	104	25	36	64	31	1.64E-04	0.01368	57	66	73	60	62	67	0.6844175	1	55.625	26.975849								
7	5E+07	7_46734732	21	49	29	47	35	38	0.947014	1	63	42	41	67	53	54	0.88746565	1	44.875	15.5143942	5E+07	4.7E+07	ID=ENSP00000360510;SUPT3H;;	SUPT3H				
7	5E+07	7_46962912	382	187	51	58	285	55	3.37E-43	#######	353	137	36	26	245	31	2.27E-33	2.66E-30	153.75	142.976272	5E+07	4.7E+07	ID=ENSP00000352514;RUNX2;NM_001024630;	RUNX2	4.7E+07	4.7E+07	0.43934	ID=promoter_47733;
7	5E+07	7_47010270	733	299	440	315	516	378	2.03E-11	#######	253	281	284	223	267	254	0.95747088	1	353.5	166.120438	5E+07	4.7E+07	ID=ENSP00000352514;RUNX2;NM_001024630;	RUNX2	4.7E+07	4.7E+07	0.73392	ID=promoter_47736;
7	5E+07	7_47026183	12	23	10	12	18	11	0.22238	1	2	22	18	30	12	24	0.08343652	1	16.125	8.85498246	5E+07	4.7E+07	ID=ENSP00000352514;RUNX2;NM_001024630;	RUNX2				
7	5E+07	7_471698	639	450	1466	992	545	1229	5.08E-37	#######	1095	716	796	383	906	590	1.67E-10	5.42E-08	817.13	357.467456								
7	5E+07	7_471900	15	33	7	13	24	10	0.016074	0.59429	3	3	8	6	3	7	0.3829009	1	11	9.87059124								
7	5E+07	7_47228956	114	33	154	100	74	127	0.032469	1	0	26	30	6	13	18	0.54030568	1	57.875	56.8417289					4.7E+07	4.7E+07	0.11483	ID=promoter_47744;
7	5E+07	7_472686	22	7	17	12	15	15	0.908797	1	4	17	17	3	11	10	0.99899888	1	12.375	7.00892288								
7	5E+07	7_474998	0	10	23	22	5	23	0.011447	0.45401	0	4	10	1	2	6	0.31385293	1	8.75	9.39224604								
7	5E+07	7_478466	8	65	48	22	37	35	0.658574	1	47	79	79	11	63	45	0.40770129	1	44.875	28.7324481								
7	5E+07	7_478971	24	17	10	17	21	14	0.252399	1	1	6	12	1	4	7	0.58783259	1	11	8.17662173								

7	5E+07	7_48360658	10	7	3	11	9	7	0.713906	1	1	32	14	34	17	24	0.40932813	1	14	12.4670996	5E+07	4.8E+07	ID=ENSP0000329454;RCAN2;NM_005822;	RCAN2				
7	5E+07	7_484481	16	37	12	22	27	17	0.124321	1	41	15	10	8	28	9	0.01512239	0.5480081	20.125	12.4377019								
7	5E+07	7_48626698	448	238	353	448	343	401	0.998997	1	273	168	194	100	221	147	0.00737686	0.3103384	277.75	128.668733					4.9E+07	4.9E+07	0.49847	ID=promoter_47780;
7	5E+07	7_486721	22	62	37	5	42	21	0.004493	0.21484	89	14	80	27	52	54	0.84299297	1	42	31.2958235								
7	5E+07	7_48873030	8	0	4	21	4	13	0.177139	1	14	4	26	1	9	14	0.47220951	1	9.75	9.63253416	5E+07	4.9E+07	ID=ENSP0000346065;TDRD6;NM_001010870;	TDRD6				
7	5E+07	7_490903	12	7	39	21	10	30	0.028514	0.93503	40	19	4	13	30	9	0.00749313	0.313751	19.375	13.6165603								
7	5E+07	7_491164	77	40	46	128	59	87	0.396679	1	17	70	32	0	44	16	0.00398908	0.1893524	51.25	40.0526439								
7	5E+07	7_498121	20	16	32	30	18	31	0.427258	1	0	68	26	24	34	25	0.67804059	1	27	19.3612278								
7	5E+07	7_500832	80	22	7	13	51	10	2.93E-08	#######	38	52	12	44	45	28	0.24903664	1	33.5	24.9341991								
7	5E+07	7_502644	36	72	41	48	54	45	0.234992	1	0	14	26	1	7	14	0.23485029	1	29.75	24.6620008								
7	5E+07	7_51039528	18	41	7	7	30	7	1.06E-04	0.00938	70	5	14	2	38	8	1.68E-04	0.0141854	20.5	23.5007599					5.1E+07	5.1E+07	0.09658	ID=promoter_47800;
7	5E+07	7_514731	88	45	6	21	67	14	2.92E-10	#######	70	58	28	6	64	17	6.58E-06	0.0008813	40.25	30.1650223								
7	5E+07	7_517426	3	47	15	21	25	18	0.280499	1	8	1	7	37	5	22	0.00242132	0.1280458	17.375	16.6984815								
7	5E+07	7_51804626	788	327	703	571	558	637	0.896419	1	427	200	216	117	314	167	3.17E-08	7.24E-06	418.63	247.508117	5E+07	5.2E+07	ID=ENSP0000008391;TFAP2D;NM_172238;	TFAP2D	5.2E+07	5.2E+07	0.34922	ID=promoter_47806;
7	5E+07	7_519206	6	53	2	3	30	3	1.54E-06	0.00022	18	31	43	17	25	30	0.63340892	1	21.625	19.0483408								
7	5E+07	7_520071	19	50	28	40	35	34	0.735392	1	41	7	16	12	24	14	0.38067267	1	26.625	15.6016254								
7	5E+07	7_525281	0	2	11	1	1	6	0.241978	1	22	0	5	1	11	3	0.13591373	1	5.25	7.70435869								
7	5E+07	7_526906	2	9	2	2	6	2	0.258649	1	3	12	11	2	8	7	0.9937864	1	5.375	4.47013902								
7	5E+07	7_530739	117	178	182	164	148	173	0.999527	1	68	70	86	20	69	53	0.58764732	1	110.63	59.5337539								
7	5E+07	7_534906	400	50	8	15	225	12	2.68E-50	#######	1	36	9	29	19	19	0.97309647	1	68.5	134.944433								
7	5E+07	7_53542806	687	330	551	526	509	539	0.254134	1	210	273	225	150	242	188	0.19074046	1	369	194.058901					5.4E+07	5.4E+07	0.37191	ID=promoter_47822;
7	5E+07	7_53649964	216	41	164	99	129	132	0.543861	1	64	71	71	44	68	58	0.89700969	1	96.25	62.1467158					5.4E+07	5.4E+07	0.34785	ID=promoter_47825;
7	5E+07	7_53698628	1005	341	613	619	673	616	4.81E-05	0.00469	272	209	170	55	241	113	8.56E-09	2.14E-06	410.5	313.219229	5E+07	5.4E+07	ID=ENSP0000314030-D4;DNAJA2;NM_005880;	DNAJA2	5.4E+07	5.4E+07	0.63604	ID=promoter_47827;
7	5E+07	7_53811381	413	135	448	173	274	311	0.924361	1	94	108	53	54	101	54	0.00423089	0.1986514	184.75	157.022064					5.4E+07	5.4E+07	0.47359	ID=promoter_47831;
7	5E+07	7_538353	47	20	25	9	34	17	0.012579	0.48874	21	21	30	26	21	28	0.4487548	1	24.875	10.8422125								
7	5E+07	7_538356	0	4	6	9	2	8	0.255957	1	5	14	15	1	10	8	0.95278883	1	6.75	5.54848757								
7	5E+07	7_53934435	16	9	26	6	13	16	0.991731	1	8	1	11	5	5	8	0.62517663	1	10.25	7.74135462	5E+07	5.4E+07	ID=ENSP0000258883;ADAMTS7;;	ADAMTS7				
7	5E+07	7_54127227	127	72	104	67	100	86	0.105971	1	19	31	56	12	25	34	0.34266885	1	61	40.4474968	5E+07	5.4E+07	ID=ENSP0000378240;CTSH;NM_148979;	CTSH				
7	5E+07	7_54197009	27	13	18	12	20	15	0.418324	1	0	5	11	1	3	6	0.53745121	1	10.875	8.99900788	5E+07	5.4E+07	ID=ENSP0000261752;RASGRF1;NM_002891;	RASGRF1				
7	5E+07	7_54263596	2	10	3	7	6	5	0.851367	1	2	27	26	3	15	15	0.97869956	1	10	10.5559733	5E+07	5.4E+07	ID=ENSP0000261752;RASGRF1;NM_002891;	RASGRF1				
7	5E+07	7_54273344	53	33	44	61	43	53	0.971253	1	0	15	15	4	8	10	0.81871774	1	28.125	22.9685344	5E+07	5.4E+07	ID=ENSP0000261752;RASGRF1;NM_002891;	RASGRF1				

7	5E+07	7_545835	45	50	76	39	48	58	0.988469	1	77	39	75	33	58	54	0.99974204	1	54.25	18.6758056								
7	5E+07	7_545965	21	28	14	23	25	19	0.35609	1	16	26	14	19	21	17	0.91718448	1	20.125	5.33017019								
7	5E+07	7_545980	68	39	28	31	54	30	0.003551	0.17889	45	0	22	38	23	30	0.4647018	1	33.875	19.5041204								
7	5E+07	7_546614	33	47	30	45	40	38	0.647488	1	102	55	53	19	79	36	0.00149905	0.0875056	48	25.0428205								
7	5E+07	7_549030	48	13	36	9	31	23	0.244309	1	1	0	36	0	1	18	2.55E-04	0.0200383	17.875	19.238633								
7	6E+07	7_55318874	145	122	87	64	134	76	1.55E-06	0.00022	54	47	34	1	51	18	0.00122967	0.074208	69.25	47.0432931	6E+07	5.5E+07	ID=ENSP0000378190;ARNT;;	ARNT				
7	6E+07	7_555027	23	37	15	35	30	25	0.447286	1	24	30	55	41	27	48	0.02250024	0.7507942	32.5	12.4211801								
7	6E+07	7_55762647	14	20	7	6	17	7	0.051092	1	27	9	9	1	18	5	0.0406475	1	11.625	8.38259251					5.6E+07	5.6E+07	0.61692	ID=promoter_47870;
7	6E+07	7_558552	82	48	42	45	65	44	0.017156	0.6252	50	21	30	8	36	19	0.13186357	1	40.75	22.1601315								
7	6E+07	7_55905890	4	31	14	13	18	14	0.509661	1	48	59	14	18	54	16	1.31E-04	0.0114541	25.125	19.2608967	6E+07	5.6E+07	ID=ENSP0000378177;KIAA1199;;	KIAA1199				
7	6E+07	7_56286140	0	0	9	0	0	5	0.118674	1	7	0	15	36	4	26	1.32E-04	0.0115204	8.375	12.4778375	6E+07	5.6E+07	ID=ENSP0000352413;TMC3;NM_001080532;	TMC3				
7	6E+07	7_569066	7	4	10	29	6	20	0.063627	1	0	3	24	30	2	27	7.81E-06	0.0010203	13.375	12.3049061								
7	6E+07	7_569810	14	27	15	15	21	15	0.333352	1	23	16	27	21	20	24	0.69398062	1	19.75	5.47070118								
7	6E+07	7_572444	101	64	117	86	83	102	0.951504	1	41	31	76	26	36	51	0.14571533	1	67.75	33.285561								
7	6E+07	7_576082	1	25	10	2	13	6	0.146758	1	13	33	32	31	23	32	0.32689554	1	18.375	13.4794818								
7	6E+07	7_576455	23	17	16	0	20	8	0.026297	0.87605	30	36	26	2	33	14	0.04245489	1	18.75	12.7475488								
7	6E+07	7_57874626	19	9	21	27	14	24	0.52503	1	19	24	15	3	22	9	0.10444527	1	17.125	7.90004521	6E+07	5.8E+07	ID=ENSP0000368696;TM6SF1;;	TM6SF1				
7	6E+07	7_578845	56	17	44	21	37	33	0.517593	1	0	7	30	2	4	16	0.01700101	0.6008545	22.125	20.1383607								
7	6E+07	7_58122890	42	26	25	15	34	20	0.04416	1	22	9	2	1	16	2	0.00719486	0.3031284	17.75	13.8331899	6E+07	5.8E+07	ID=ENSP0000305632;HOMER2;NM_199330;	HOMER2				
7	6E+07	7_58138966	141	107	132	91	124	112	0.13258	1	25	52	65	15	39	40	0.90397927	1	78.5	47.0804782					5.8E+07	5.8E+07	0.28926	ID=promoter_47906;
7	6E+07	7_581524	134	97	60	51	116	56	1.26E-07	#######	26	0	17	38	13	28	0.03522609	1	52.875	44.0922329								
7	6E+07	7_59051081	27	14	24	28	21	26	0.983171	1	10	19	9	25	15	17	0.85096642	1	19.5	7.65319728	6E+07	5.9E+07	ID=ENSP0000353655;MAN2A2;NM_006122;	MAN2A2				
7	6E+07	7_59090112	198	36	139	97	117	118	0.51111	1	138	109	181	27	124	104	0.75290602	1	115.63	61.7505061	6E+07	5.9E+07	ID=ENSP0000377801;RCCD1;NM_033544;	RCCD1	5.9E+07	5.9E+07	0.34764	ID=promoter_47926;
7	6E+07	7_59161361	2	18	5	9	10	7	0.570701	1	1	1	7	23	1	15	0.00126073	0.075413	8.25	8.1897148	6E+07	5.9E+07	ID=ENSP0000373073;UROC1;;	UROC1				
7	6E+07	7_592481	79	45	74	47	62	61	0.621083	1	102	16	39	17	59	28	0.01205788	0.4575229	52.375	30.4158676								
7	6E+07	7_592867	218	129	138	158	174	148	0.015522	0.57882	99	48	76	29	74	53	0.35569714	1	111.88	61.8371999								
7	6E+07	7_595666	1983	2013	3257	3732	1998	3495	2.07E-45	#######	2370	1543	3910	1324	1957	2617	9.94E-34	1.18E-30	2516.5	992.614009								
7	6E+07	7_59879484	12	1	20	11	7	16	0.324814	1	1	7	22	35	4	29	2.88E-05	0.0031583	13.625	11.5874008	6E+07	6E+07	ID=ENSP0000268134;ACAN;;	ACAN				
7	6E+07	7_604270	5	14	19	22	10	21	0.306738	1	58	16	26	5	37	16	0.03310062	1	20.625	16.8348066								
7	6E+07	7_607597	22	2	1	5	12	3	0.030625	0.98374	17	5	5	2	11	4	0.25131486	1	7.375	7.76323754								
7	6E+07	7_61075910	13	13	17	16	13	17	0.957342	1	1	12	8	0	7	4	0.73798468	1	10	6.45865975	6E+07	6.1E+07	ID=ENSP0000331897-D2;IDH2;NM_002168;	IDH2				
7	6E+07	7_612769	245	245	296	225	245	261	0.556561	1	609	585	377	108	597	243	1.33E-27	1.31E-24	336.25	177.553815								
7	6E+07	7_612800	78	18	43	26	48	35	0.09725	1	3	12	9	7	8	8	0.98858271	1	24.5	25.1111813								
7	6E+07	7_613488	129	36	157	108	83	133	0.081478	1	75	26	67	39	51	53	0.84389715	1	79.625	47.5693103								
7	6E+07	7_615282	27	19	11	1	23	6	0.001551	0.09145	1	14	5	2	8	4	0.59180275	1	10	9.54687683								

7	6E+07	7_62038316	3	10	1	2	7	2	0.160905	1	9	4	11	5	7	8	0.92121674	1	5.625	3.85217935	6E+07	6.2E+07	ID=ENSP0000326924;SCAPER;NM_001145923;	SCAPER				
7	6E+07	7_62247897	65	81	66	84	73	75	0.72239	1	147	70	31	40	109	36	1.46E-07	2.94E-05	73	35.1242692	6E+07	6.2E+07	ID=ENSP0000326924;SCAPER;NM_001145923;	SCAPER				
7	6E+07	7_62305481	256	148	338	248	202	293	0.066756	1	153	85	102	33	119	68	0.00571222	0.2548307	170.38	102.186017					6.2E+07	6.2E+07	0.46191	ID=promoter_47991;
7	6E+07	7_62434539	54	14	53	46	34	50	0.594713	1	35	47	26	22	41	24	0.19624561	1	37.125	15.1415936	6E+07	6.2E+07	ID=ENSP0000368914;PSTPIP1;NM_003978;	PSTPIP1				
7	6E+07	7_62466251	24	51	37	44	38	41	0.93346	1	106	30	46	19	68	33	0.00807829	0.3327873	44.625	27.1605675	6E+07	6.2E+07	ID=ENSP0000267970;TSPAN3;NM_005724;	TSPAN3				
7	6E+07	7_624728	246	149	285	492	198	389	1.78E-08	#######	10	21	23	11	16	17	0.92610249	1	154.63	175.631869								
7	6E+07	7_625105	14	30	1	11	22	6	0.002551	0.13706	0	1	24	32	1	28	1.20E-06	0.0001963	14.125	13.2172777								
7	6E+07	7_628346	12	0	3	16	6	10	0.79153	1	0	6	4	1	3	3	0.99570314	1	5.25	5.87367006								
7	6E+07	7_630287	89	37	90	118	63	104	0.098942	1	88	29	45	20	59	33	0.06332576	1	64.5	35.9404269								
7	6E+07	7_634193	11	12	13	13	12	13	0.980472	1	0	8	16	19	4	18	0.00668749	0.2868305	11.5	5.68205194								
7	6E+07	7_634204	10	80	44	22	45	33	0.117793	1	48	29	29	38	39	34	0.96627933	1	37.5	21.0441712								
7	6E+07	7_63433250	431	177	410	309	304	360	0.992511	1	273	207	184	61	240	123	4.10E-07	7.52E-05	256.5	124.950276					6.3E+07	6.3E+07	0.5167	ID=promoter_48009;
7	6E+07	7_646865	27	32	11	9	30	10	0.001158	0.07164	2	28	23	26	15	25	0.19020812	1	19.75	10.8726393								
7	6E+07	7_646894	10	57	26	51	34	39	0.995484	1	25	54	45	11	40	28	0.51986598	1	34.875	19.2089079								
7	6E+07	7_64813680	1727	562	1482	1214	1145	1348	0.995789	1	732	563	761	334	648	548	0.28547321	1	921.88	494.705522	6E+07	6.5E+07	ID=ENSP0000355402;ISLR2;NM_020851;	ISLR2	6.5E+07	6.5E+07	0.74638	ID=promoter_48045;
7	6E+07	7_64845860	87	21	69	37	54	53	0.653183	1	1	1	6	0	1	3	0.56022579	1	27.75	33.8431255	6E+07	6.5E+07	ID=ENSP0000268058;PML;NM_033238;	PML				
7	6E+07	7_649584	1	6	2	7	4	5	0.995512	1	9	10	27	3	10	15	0.49251655	1	8.125	8.28833087								
7	7E+07	7_650874	299	186	399	425	243	412	2.78E-05	0.0029	132	207	157	112	170	135	0.40535111	1	239.63	120.669487								
7	7E+07	7_65162834	97	138	119	173	118	146	0.911145	1	99	58	123	54	79	89	0.45034009	1	107.63	39.7992731	7E+07	6.5E+07	ID=ENSP0000320084;CD276;NM_001024736;	CD276	6.5E+07	6.5E+07	0.18073	ID=promoter_48059;
7	7E+07	7_65164228	244	96	245	229	170	237	0.227515	1	38	4	22	11	21	17	0.91718448	1	111.13	109.865421	7E+07	6.5E+07	ID=ENSP0000320084;CD276;NM_001024736;	CD276				
7	7E+07	7_651835	132	103	153	127	118	140	0.995824	1	15	53	35	5	34	20	0.26570036	1	77.875	57.7418825								
7	7E+07	7_65501930	582	235	527	441	409	484	0.991367	1	229	105	167	52	167	110	0.02002508	0.6815826	292.25	198.903243					6.5E+07	6.6E+07	0.71452	ID=promoter_48063;
7	7E+07	7_655506	36	77	62	27	57	45	0.135775	1	43	58	50	46	51	48	0.99717376	1	49.875	15.7065728								
7	7E+07	7_658170	105	69	86	56	87	71	0.07423	1	8	15	18	1	12	10	0.96947392	1	44.75	39.4814604								
7	7E+07	7_659273	10	3	2	6	7	4	0.503048	1	9	0	10	11	5	11	0.25523295	1	6.375	4.24053568								
7	7E+07	7_661004	5	5	16	17	5	17	0.090491	1	4	24	48	2	14	25	0.13553211	1	15.125	15.422039								
7	7E+07	7_66373843	9633	10469	10965	13905	10051	####	3.70E-04	0.02765	9619	7471	17833	4698	8545	11266	5.66E-133	2.89E-129	10574	3962.43141					6.6E+07	6.6E+07	0.33404	ID=promoter_48073;
7	7E+07	7_67643170	51	44	63	69	48	66	0.703494	1	59	41	39	45	50	42	0.89489882	1	51.375	11.0832886					6.8E+07	6.8E+07	0.63445	ID=promoter_48082;
7	7E+07	7_679227	32	81	47	40	57	44	0.110223	1	48	62	162	138	55	150	6.29E-12	2.39E-09	76.25	48.2811705								
7	7E+07	7_696630	7	55	29	4	31	17	0.035825	1	15	35	48	15	25	32	0.47866995	1	26	18.9057814								
7	7E+07	7_703061	6	38	5	11	22	8	0.011059	0.44213	0	20	13	1	10	7	0.84912739	1	11.75	12.4871362								
7	7E+07	7_70928662	1396	516	1017	983	956	1000	0.040401	1	536	317	363	212	427	288	1.55E-04	0.0132523	667.5	416.738699					7.1E+07	7.1E+07	0.51598	ID=promoter_48121;

7	7E+07	7_70944642	749	294	596	520	522	558	0.31255	1	168	236	172	121	202	147	0.08165416	1	357	233.484781					7.1E+07	7.1E+07	0.62656	ID=promoter_48123;
7	7E+07	7_714003	31	1	14	19	16	17	0.960459	1	0	5	3	0	3	2	0.9363008	1	9.125	11.2559508								
7	7E+07	7_716737	18	11	9	4	15	7	0.113635	1	0	4	17	24	2	21	1.85E-04	0.0153843	10.875	8.25378701								
7	7E+07	7_717802	25	30	19	20	28	20	0.231138	1	19	47	27	4	33	16	0.09595749	1	23.875	12.1941145								
7	7E+07	7_718798	16	14	27	27	15	27	0.40743	1	43	13	20	27	28	24	0.96104627	1	23.375	9.89859297								
7	7E+07	7_71984130	41	34	122	72	38	97	1.70E-04	0.01413	18	1	8	2	10	5	0.51906437	1	37.25	41.7432629	7E+07	7.2E+07	ID=ENSP0000350446;NPAS3;NM_173159;	NPAS3				
7	7E+07	7_728182	4	52	9	15	28	12	0.009759	0.39939	61	18	29	21	40	25	0.29783938	1	26.125	20.3289906								
7	7E+07	7_73077881	46	23	30	20	35	25	0.160265	1	25	43	34	19	34	27	0.83476892	1	30	10.2539191	7E+07	7.3E+07	ID=ENSP0000280979;AKAP6;NM_004274;	AKAP6				
7	7E+07	7_74951974	1	2	6	2	2	4	0.823343	1	26	7	4	1	17	3	0.01239331	0.4669004	6.125	8.33987838	7E+07	7.5E+07	ID=ENSP0000206595;G2E3;NM_017769;	G2E3				
7	8E+07	7_750567	54	66	84	67	60	76	0.905657	1	19	42	24	47	31	36	0.65410506	1	50.375	22.122629								
7	8E+07	7_752324	21	34	6	5	28	6	1.19E-04	0.01043	36	39	22	20	38	21	0.15716135	1	22.875	12.9442486								
7	8E+07	7_760432	16	22	14	7	19	11	0.163668	1	15	20	7	36	18	22	0.68341813	1	17.125	9.32642176								
7	8E+07	7_763945	4	19	9	12	12	11	0.839207	1	18	65	26	9	42	18	0.01944768	0.6639875	20.25	19.3741359								
7	8E+07	7_76669906	215	113	151	139	164	145	0.045283	1	126	57	92	41	92	67	0.32158127	1	116.75	55.2158104					7.7E+07	7.7E+07	0.3053	ID=promoter_48157;
7	8E+07	7_773627	43	90	19	39	67	29	1.81E-05	0.00201	54	39	21	28	47	25	0.07550832	1	41.625	22.7780439								
7	8E+07	7_80545249	128	68	121	78	98	100	0.618075	1	57	45	14	0	51	7	2.80E-07	5.32E-05	63.875	45.6115195					8.1E+07	8.1E+07	0.49519	ID=promoter_48163;
7	8E+07	7_809363	6	4	3	0	5	2	0.402696	1	28	8	4	0	18	2	0.00297675	0.1507727	6.625	9.05439909								
7	8E+07	7_811332	7	8	10	12	8	11	0.943069	1	19	0	3	0	10	2	0.09294023	1	7.375	6.45727718								
7	8E+07	7_81263862	333	40	301	298	187	300	0.003365	0.17188	95	27	93	59	61	76	0.2255143	1	155.75	130.774342	8E+07	8.1E+07	ID=ENSP0000250373;NFATC4;NM_004554;	NFATC4				
7	8E+07	7_81315443	218	96	253	179	157	216	0.313789	1	38	57	29	23	48	26	0.08258093	1	111.63	91.9408777	8E+07	8.1E+07	ID=ENSP0000380008;LTB4R;NM_001143919;	LTB4R	8.1E+07	8.1E+07	0.28927	ID=promoter_48173;
7	8E+07	7_81327610	1	9	7	5	5	6	0.999292	1	26	1	3	0	14	2	0.01719769	0.6064547	6.5	8.48528137	8E+07	8.1E+07	ID=ENSP0000267425;C14orf21;NM_174913;	C14orf21				
7	8E+07	7_81366405	17	16	26	8	17	17	0.897282	1	56	18	18	21	37	20	0.14440904	1	22.5	14.4420022	8E+07	8.1E+07	ID=ENSP0000382341;;NM_004581;					
7	8E+07	7_81450899	572	199	357	436	386	397	0.184025	1	246	222	132	105	234	119	4.33E-07	7.88E-05	283.63	159.92582					8.1E+07	8.1E+07	0.26943	ID=promoter_48185;
7	8E+07	7_81528264	4	16	4	2	10	3	0.082185	1	12	6	9	0	9	5	0.6500913	1	6.625	5.37022213	8E+07	8.2E+07	ID=ENSP0000216780;PCK2;NM_004563;	PCK2				
7	8E+07	7_816223	131	157	100	72	144	86	2.94E-06	0.0004	67	39	28	3	53	16	1.88E-04	0.0155974	74.625	52.4348235								
7	8E+07	7_818221	23	16	6	15	20	11	0.118302	1	25	17	5	5	21	5	0.01300804	0.4860836	14	7.94624799								
7	8E+07	7_819321	26	2	34	19	14	27	0.312392	1	3	10	2	1	7	2	0.29886173	1	12.125	12.7328316								
7	8E+07	7_819551	507	628	408	553	568	481	8.80E-07	0.00013	1225	1109	508	315	1167	412	2.73E-67	6.09E-64	656.63	329.98915								
7	8E+07	7_82026426	769	391	545	595	580	570	0.01099	0.44006	543	378	247	221	461	234	2.50E-13	1.12E-10	461.13	185.851275					8.2E+07	8.2E+07	0.25658	ID=promoter_48204;
7	8E+07	7_82460319	1	10	10	0	6	5	0.851367	1	0	21	13	12	11	13	0.83819512	1	8.375	7.50119038	8E+07	8.2E+07	ID=ENSP0000380666;SLC7A7;;	SLC7A7				
7	8E+07	7_826054	7	15	10	17	11	14	0.979671	1	25	10	7	0	18	4	0.01933304	0.6616555	11.375	7.57698583								
7	8E+07	7_826211	55	4	28	28	30	28	0.684	1	52	28	80	27	40	54	0.19353425	1	37.75	23.3773395								
7	8E+07	7_826422	22	0	34	11	11	23	0.278506	1	1	0	1	0	1	1	0.99856566	1	8.625	12.9166283								

7	8E+07	7_839072	3	5	32	3	4	18	0.032333	1	34	19	15	19	27	17	0.45364599	1	16.25	12.2911118								
7	8E+07	7_84488037	145	136	2	1	141	2	2.76E-35	#######	33	107	6	3	70	5	6.26E-12	2.38E-09	54.125	64.0009766					8.4E+07	8.4E+07	0.12764	ID=promoter_48242;
7	8E+07	7_84511071	939	694	913	729	817	821	0.007401	0.31975	480	475	575	205	478	390	0.17311527	1	626.25	244.811735					8.5E+07	8.5E+07	0.23408	ID=promoter_48244;
7	8E+07	7_847350	0	11	6	10	6	8	0.9722	1	23	26	21	0	25	11	0.10848128	1	12.125	10.1901563								
7	8E+07	7_84749258	326	83	243	177	205	210	0.385683	1	124	28	76	45	76	61	0.70330309	1	137.75	103.994162					8.5E+07	8.5E+07	0.17016	ID=promoter_48249;
7	8E+07	7_84763932	187	58	213	162	123	188	0.07275	1	33	74	64	99	54	82	0.01749793	0.6144624	111.25	66.9706759					8.5E+07	8.5E+07	0.20541	ID=promoter_48253;
7	8E+07	7_84872559	365	197	153	130	281	142	3.96E-16	#######	120	140	68	0	130	34	2.04E-11	7.37E-09	146.63	106.21802					8.5E+07	8.5E+07	0.21388	ID=promoter_48254;
7	9E+07	7_875678	0	31	2	2	16	2	0.001284	0.07728	32	31	17	1	32	9	0.00363228	0.1775497	14.5	14.937967								
7	9E+07	7_87748054	1	11	2	20	6	11	0.674578	1	16	8	0	0	12	0	0.00384008	0.1844256	7.25	7.79651937	9E+07	8.8E+07	ID=ENSP0000354548-D2;;;		8.8E+07	8.8E+07	0.17703	ID=promoter_48268;
7	9E+07	7_882295	43	58	31	23	51	27	0.002775	0.14683	0	26	28	5	13	17	0.64388579	1	26.75	18.7597594								
7	9E+07	7_888182	54	23	57	60	39	59	0.465705	1	11	71	41	3	41	22	0.11146441	1	40	24.9170051								
7	9E+07	7_899113	38	1	24	25	20	25	0.977759	1	1	8	0	0	5	0	0.09850879	1	12.125	14.8076959								
7	9E+07	7_901182	190	75	136	76	133	106	0.011569	0.45508	116	36	21	5	76	13	2.05E-09	5.65E-07	81.875	62.7772422								
7	9E+07	7_905469	19	38	11	36	29	24	0.446278	1	27	25	8	34	26	21	0.89538361	1	24.75	11.3105513								
7	9E+07	7_90577952	6	17	29	18	12	24	0.311519	1	45	10	32	0	28	16	0.29661245	1	19.625	14.9182295					9.1E+07	9.1E+07	0.09229	ID=promoter_48286;
7	9E+07	7_90700869	137	91	84	134	114	109	0.311052	1	0	52	37	88	26	63	1.04E-04	0.0094884	77.875	46.8475872					9.1E+07	9.1E+07	0.19847	ID=promoter_48287;
7	9E+07	7_910369	23	56	10	19	40	15	3.72E-04	0.02775	71	38	48	8	55	28	0.03226495	0.9878091	34.125	22.8750488								
7	9E+07	7_912091	16	36	46	47	26	47	0.203847	1	71	47	81	30	59	56	0.99205173	1	46.75	21.0560477								
7	9E+07	7_92511115	686	262	585	436	474	511	0.416621	1	364	235	176	86	300	131	1.73E-12	7.04E-10	353.75	205.822913					9.3E+07	9.3E+07	0.53184	ID=promoter_48304;
7	9E+07	7_925623	0	1	0	4	1	2	0.907382	1	26	7	2	1	17	2	0.0046333	0.2138882	5.125	8.75764972								
7	9E+07	7_927911	29	17	40	30	23	35	0.623149	1	216	160	204	39	188	122	0.00859922	0.3494083	91.875	85.7695209								
7	9E+07	7_932555	35	112	26	31	74	29	9.90E-07	0.00015	62	56	50	20	59	35	0.10798462	1	49	29.4666882								
7	9E+07	7_933180	0	17	9	2	9	6	0.554309	1	24	36	16	8	30	12	0.0403413	1	14	11.9403278								
7	9E+07	7_934007	24	37	41	49	31	45	0.658002	1	0	1	10	0	1	5	0.22510075	1	20.25	20.1689294								
7	9E+07	7_938601	8	4	10	18	6	14	0.355886	1	0	12	16	5	6	11	0.39136385	1	9.125	6.12809688								
7	1E+08	7_953407	516	330	611	474	423	543	0.379775	1	16	26	13	16	21	15	0.7415049	1	250.25	260.085014								
7	1E+08	7_955739	654	760	426	501	707	464	8.05E-22	#######	534	472	646	225	503	436	0.59011153	1	527.25	164.635311								
7	1E+08	7_956679	5	14	13	14	10	14	0.912507	1	12	7	29	4	10	17	0.30316389	1	12.25	7.88760329								
7	1E+08	7_95726664	1825	889	1762	1869	1357	1816	0.00116	0.07164	1086	891	953	420	989	687	4.14E-08	9.31E-06	1211.9	538.185958					9.6E+07	9.6E+07	0.54439	ID=promoter_48321;
7	1E+08	7_96277350	8	38	0	3	23	2	2.40E-05	0.00257	35	23	49	3	29	26	0.99236142	1	19.875	18.9769597	1E+08	9.6E+07	ID=ENSP0000377747;CHD2;NM_001271;	CHD2				
7	1E+08	7_966856	14	20	0	0	17	0	4.68E-05	0.00457	26	10	7	19	18	13	0.78939737	1	12	9.48683298								
7	1E+08	7_96835833	101	31	85	73	66	79	0.992641	1	20	42	38	16	31	27	0.97229028	1	50.75	31.5673882	1E+08	9.7E+07	ID=ENSP0000216605;MTHFD1;NM_005956;	MTHFD1				
7	1E+08	7_97087729	434	240	423	358	337	391	0.989304	1	336	276	196	185	306	191	8.96E-05	0.0084056	306	96.8607248					9.7E+07	9.7E+07	0.80453	ID=promoter_48340;
7	1E+08	7_97350386	6	13	3	2	10	3	0.082185	1	0	0	9	9	0	9	0.00779604	0.3222758	5.25	4.77343841	1E+08	9.7E+07	ID=ENSP0000263373-D3;SPTBN4;NM_020971;	SPTBN4				
7	1E+08	7_975026	38	109	77	48	74	63	0.171656	1	67	29	77	54	48	66	0.11221359	1	62.375	25.6567312								
7	1E+08	7_97536569	347	155	350	265	251	308	0.868657	1	201	57	92	39	129	66	3.55E-04	0.0266828	188.25	123.80255					9.8E+07	9.8E+07	0.25032	ID=promoter_48351;
7	1E+08	7_990689	9	0	2	7	5	5	0.968625	1	0	8	24	0	4	12	0.09850366	1	6.25	8.11964109								
7	1E+08	7_993143	0	19	0	15	10	8	0.719353	1	0	2	7	2	1	5	0.22510075	1	5.625	7.46300399								

7	1E+08	7_99698200	25	20	25	12	23	19	0.524147	1	0	11	14	2	6	8	0.79537884	1	13.625	9.48589178	1E+08	1E+08	ID=ENSP00000330278;PLEKHH1;NM_020715;	PLEKHH1				
7	1E+08	7_100365644	26	41	25	2	34	14	0.00261	0.13949	65	69	61	0	67	31	0.00485773	0.2226648	36.125	27.3936254					1E+08	1E+08	0.11616	ID=promoter_48370;
7	1E+08	7_100367360	97	55	55	40	76	48	0.002969	0.15437	98	41	85	28	70	57	0.76752806	1	62.375	27.3283605								
7	1E+08	7_100386471	1	32	9	4	17	7	0.051092	1	1	14	45	27	8	36	4.47E-05	0.0046182	16.625	16.308959								
7	1E+08	7_100684694	9	4	4	1	7	3	0.314307	1	16	20	4	17	18	11	0.54801163	1	9.375	7.28868987								
7	1E+08	7_100831637	26	60	28	34	43	31	0.113462	1	132	93	78	11	113	45	5.09E-06	0.0007008	57.75	41.0809306								
7	1E+08	7_100848510	0	22	10	7	11	9	0.722819	1	20	0	12	13	10	13	0.72167512	1	10.5	8.14160391								
7	1E+08	7_100956471	7	30	6	39	19	23	0.994841	1	13	6	20	19	10	20	0.12622058	1	17.5	12.083046	1E+08	1E+08	ID=ENSP00000377941;ACTN1;NM_001130004;	ACTN1	1E+08	1E+08	0.22615	ID=promoter_48375;
7	1E+08	7_101014582	167	46	158	117	107	138	0.762163	1	56	42	69	10	49	40	0.83663943	1	83.125	57.4964285					1E+08	1E+08	0.84653	ID=promoter_48379;
7	1E+08	7_101068439	7	13	1	0	10	1	0.011548	0.45484	0	7	21	22	4	22	9.67E-04	0.0606639	8.875	8.96720214								
7	1E+08	7_101228685	0	6	0	0	3	0	0.172143	1	19	16	0	2	18	1	9.25E-04	0.05912	5.375	7.79995421	1E+08	1E+08	ID=ENSP00000387331-D2;EXD2;;	EXD2				
7	1E+08	7_101480513	482	218	431	400	350	416	0.98351	1	104	88	131	19	96	75	0.53909375	1	234.13	178.555664								
7	1E+08	7_101677125	11	9	3	4	10	4	0.162164	1	0	7	17	0	4	9	0.31291743	1	6.375	5.8538754								
7	1E+08	7_101685027	99	65	102	96	82	99	0.981475	1	88	35	69	52	62	61	0.9470222	1	75.75	24.4233495								
7	1E+08	7_101705277	38	24	28	21	31	25	0.374336	1	39	25	38	41	32	40	0.44930039	1	31.75	8.0311892	1E+08	1E+08	ID=ENSP00000344424;KIAA0247;NM_014734;	KIAA0247	1E+08	1E+08	0.17175	ID=promoter_48393;
7	1E+08	7_101902670	1	8	6	2	5	4	0.848147	1	51	27	7	14	39	11	0.00108567	0.0671641	14.5	16.8946314								
7	1E+08	7_101993539	4	0	9	13	2	11	0.085797	1	11	13	6	1	12	4	0.18067673	1	7.125	5.16685868	1E+08	1E+08	ID=ENSP00000370680;SMOC1;NM_022137;	SMOC1				
7	1E+08	7_102028374	596	322	427	633	459	530	0.970144	1	239	192	246	112	216	179	0.5400541	1	345.88	189.709129								
7	1E+08	7_102590314	4	14	8	16	9	12	0.95859	1	0	8	11	10	4	11	0.14780855	1	8.875	5.16685868	1E+08	1E+08	ID=ENSP00000358407-D10;ADAM30;NM_021794;	ADAM30				
7	1E+08	7_102682504	260	125	245	122	193	184	0.131228	1	78	35	73	19	57	46	0.78263009	1	119.63	89.9617974								
7	1E+08	7_102825949	15	25	12	6	20	9	0.046279	1	6	4	1	20	5	11	0.25523295	1	11.125	8.39110924								
7	1E+08	7_103157344	548	6	351	297	277	324	0.999415	1	323	6	212	149	165	181	0.29363783	1	236.5	183.537462								
7	1E+08	7_103270106	96	64	56	116	80	86	0.854114	1	116	74	83	15	95	49	0.00328925	0.1648745	77.5	33.5942172								
7	1E+08	7_103408976	8	1	1	0	5	1	0.186119	1	0	5	23	4	3	14	0.01844097	0.6375434	5.25	7.70435869	1E+08	1E+08	ID=ENSP00000351352;SIPA1L1;NM_015556;	SIPA1L1				
7	1E+08	7_103645769	4	59	21	0	32	11	9.30E-04	0.05947	157	17	45	45	87	45	0.005551	0.2498699	43.5	50.4097496								
7	1E+08	7_103678782	14	52	14	13	33	14	0.00394	0.1935	59	35	20	57	47	39	0.87718245	1	33	20.3820649								

7	1E+08	7_103712297	1	11	0	0	6	0	0.029633	0.9649	8	3	16	1	6	9	0.65381141	1	5	6								
7	1E+08	7_103841826	44	51	59	42	48	51	0.886006	1	9	26	31	3	18	17	0.9984894	1	33.125	19.7515822								
7	1E+08	7_104070325	19	30	19	14	25	17	0.214068	1	11	19	15	8	15	12	0.93002578	1	16.875	6.66413642								
7	1E+08	7_104144337	73	73	52	58	73	55	0.044	1	35	1	1	0	18	1	9.25E-04	0.05912	36.625	32.1200316	1E+08	1E+08	ID=ENSP00000351147-D2;WDR21A;NM_015604;	WDR21A				
7	1E+08	7_104233426	769	189	608	577	479	593	0.679792	1	290	172	207	121	231	164	0.03226677	0.9878091	366.63	246.558563					1E+08	1E+08	0.29831	ID=promoter_48427;
7	1E+08	7_104328130	1015	314	728	435	665	582	1.51E-06	0.00022	52	31	16	26	42	21	0.06366916	1	327.13	377.310194	1E+08	1E+08	ID=ENSP00000351147;WDR21A;NM_015604;	WDR21A				
7	1E+08	7_104936861	28	0	5	5	14	5	0.053576	1	20	2	0	1	11	1	0.02232329	0.7455867	7.625	10.5144458					1E+08	1E+08	0.26525	ID=promoter_48438;
7	1E+08	7_105137699	1607	502	1637	1554	1055	1596	1.05E-09	#######	1178	548	661	382	863	522	8.43E-14	3.95E-11	1008.6	542.662204					1.1E+08	1.1E+08	0.53684	ID=promoter_48446;
7	1E+08	7_105852342	2	6	3	6	4	5	0.995512	1	8	3	8	7	6	8	0.79537884	1	5.375	2.38671921								
7	1E+08	7_105854237	4	3	1	2	4	2	0.597627	1	28	11	9	9	20	9	0.18397305	1	8.375	8.74948978								
7	1E+08	7_105945781	4	16	8	28	10	18	0.549586	1	0	21	13	0	11	7	0.73613127	1	11.25	10.1242284								
7	1E+08	7_106085002	213	67	176	149	140	163	0.99793	1	18	28	44	19	23	32	0.32689554	1	89.25	78.116671	1E+08	1.1E+08	ID=ENSP00000261978;LTBP2;NM_000428;	LTBP2	1.1E+08	1.1E+08	0.58737	ID=promoter_48475;
7	1E+08	7_106239711	13	10	6	14	12	10	0.724827	1	0	23	12	6	12	9	0.89054267	1	10.5	6.84522774	1E+08	1.1E+08	ID=ENSP00000324463;YLPM1;NM_019589;	YLPM1				
7	1E+08	7_106380037	194	109	142	143	152	143	0.165386	1	72	38	83	46	55	65	0.41373823	1	103.38	53.8143303								
7	1E+08	7_106403880	86	35	101	79	61	90	0.382534	1	16	0	28	22	8	25	0.00638831	0.2759882	45.875	37.322676								
7	1E+08	7_106439035	13	27	14	17	20	16	0.517469	1	14	17	14	1	16	8	0.35023049	1	14.625	7.11010348								
7	1E+08	7_107212288	6	36	15	15	21	15	0.333352	1	11	0	8	7	6	8	0.79537884	1	12.25	10.7935166								
7	1E+08	7_107421555	88	46	90	71	67	81	0.983382	1	59	54	32	11	57	22	0.00151246	0.0879967	56.375	27.0393232								
7	1E+08	7_107504708	16	7	2	9	12	6	0.213448	1	2	0	30	3	1	17	4.35E-04	0.0314643	8.625	10.0560927								
7	1E+08	7_107529552	32	34	27	26	33	27	0.37765	1	2	52	45	21	27	33	0.56497566	1	29.875	15.2074935								
7	1E+08	7_107592525	622	324	465	398	473	432	8.22E-04	0.05386	370	173	225	173	272	199	0.03933743	1	343.75	155.177088					1.1E+08	1.1E+08	0.44677	ID=promoter_48509;
7	1E+08	7_107611598	220	45	89	80	133	85	5.23E-05	0.00504	34	33	28	13	34	21	0.33784557	1	67.75	66.7869748								
7	1E+08	7_107775626	61	39	91	58	50	75	0.401769	1	48	48	26	16	48	21	0.01335255	0.4945372	48.375	23.0089268								
7	1E+08	7_107921814	341	102	328	295	222	312	0.11934	1	50	180	95	25	115	60	0.00123632	0.0745461	177	128.292077					1.1E+08	1.1E+08	0.60327	ID=promoter_48513;
7	1E+08	7_108048349	0	7	1	0	4	1	0.312866	1	13	8	24	1	11	13	0.83819512	1	6.75	8.41342465								
7	1E+08	7_108098229	18	2	5	6	10	6	0.41703	1	53	9	5	4	31	5	2.16E-04	0.0176336	12.75	16.9852878								
7	1E+08	7_108149949	103	10	108	38	57	73	0.883659	1	13	7	0	0	10	0	0.00970398	0.3824351	34.875	45.2056807					1.1E+08	1.1E+08	0.13885	ID=promoter_48524;
7	1E+08	7_108233599	118	77	94	90	98	92	0.306778	1	71	106	47	9	89	28	1.13E-06	0.0001861	76.5	34.9244082								

7	1E+08	7_108371215	1	24	8	9	13	9	0.46931	1	1	43	3	1	22	2	4.98E-04	0.0353761	11.25	14.9737866	1E+08	1.1E+08	ID=ENSP00000298351;TMEM63C;NM_020431;	TMEM63C				
7	1E+08	7_108440652	1	1	4	14	1	9	0.073427	1	50	8	11	4	29	8	0.00552548	0.2488783	11.625	16.1858624								
7	1E+08	7_108471500	2	4	9	7	3	8	0.459247	1	9	17	12	0	13	6	0.35360803	1	7.5	5.52914357	1E+08	1.1E+08	ID=ENSP00000262445-D2;MAP2K4;NM_003010;	MAP2K4				
7	1E+08	7_108506736	2	13	7	5	8	6	0.705675	1	23	7	25	13	15	19	0.66253156	1	11.875	8.37406711	1E+08	1.1E+08	ID=ENSP00000262445-D2;MAP2K4;NM_003010;	MAP2K4				
7	1E+08	7_108555178	39	16	38	25	28	32	0.994967	1	5	5	22	14	5	18	0.01516798	0.5480081	20.5	13.1692282					1.1E+08	1.1E+08	0.19626	ID=promoter_48546;
7	1E+08	7_108911934	62	59	92	76	61	84	0.634026	1	34	15	19	19	25	19	0.80752973	1	47	29.2672416								
7	1E+08	7_109607772	11	74	42	49	43	46	0.910016	1	29	53	56	31	41	44	0.79631464	1	43.125	19.3865749								
7	1E+08	7_110253027	22	18	29	32	20	31	0.621699	1	1	31	12	0	16	6	0.14768941	1	18.125	12.8222741								
7	1E+08	7_110283534	12	67	13	1	40	7	5.29E-07	#######	18	10	32	38	14	35	0.00480639	0.2209499	23.875	21.1622136								
7	1E+08	7_110444476	20	96	30	22	58	26	1.28E-04	0.01112	78	79	136	33	79	85	0.63438772	1	61.75	42.0976755								
7	1E+08	7_110620526	99	259	190	224	179	207	0.990379	1	58	72	104	174	65	139	7.60E-08	1.62E-05	147.5	74.4004608								
7	1E+08	7_111655506	10882	10916	13312	16024	10899	####	1.99E-26	#######	13306	10331	23901	7013	11819	15457	1.20E-174	8.57E-171	13211	5065.41726					1.1E+08	1.1E+08	0.49014	ID=promoter_48569;
7	1E+08	7_111766611	16	172	33	37	94	35	7.45E-09	#######	56	92	62	45	74	54	0.40724635	1	64.125	49.0930239								
7	1E+08	7_113545329	1	17	4	8	9	6	0.554309	1	0	0	9	1	0	5	0.06742467	1	5	6								
7	1E+08	7_113602285	17	60	5	28	39	17	0.001855	0.10518	59	94	53	48	77	51	0.17368211	1	45.5	28.1982776								
7	1E+08	7_113717065	4	10	4	9	7	7	0.956352	1	0	2	12	0	1	6	0.13767373	1	5.125	4.64258241								
7	1E+08	7_114114045	27	84	65	56	56	61	0.923132	1	90	122	134	141	106	138	0.03050944	0.9445414	89.875	40.2614225								
7	1E+08	7_114867214	23	14	9	11	19	10	0.108738	1	68	66	45	51	67	48	0.39298026	1	35.875	24.6021921								
7	1E+08	7_114868014	8	17	11	27	13	19	0.82904	1	47	2	15	3	25	9	0.04125862	1	16.25	14.8203528								
7	1E+08	7_115054756	22	9	7	7	16	7	0.076817	1	28	6	1	29	17	15	0.99031847	1	13.625	10.9536361								
7	1E+08	7_115605142	45	37	15	24	41	20	0.004028	0.19746	13	7	30	5	10	18	0.2304087	1	22	14.4716472								
7	1E+08	7_115836242	1	9	5	7	5	6	0.999292	1	25	23	17	2	24	10	0.09254106	1	11.125	9.35700654								
7	1E+08	7_117224265	0	9	14	6	5	10	0.615109	1	16	28	7	1	22	4	0.00378795	0.1837142	10.125	9.12512231								
7	1E+08	7_117549657	127	196	52	53	162	53	6.83E-17	#######	155	121	177	29	138	103	0.24879344	1	113.75	62.543128								
7	1E+08	7_117556897	10	24	18	8	17	13	0.50439	1	15	24	22	35	20	29	0.29996038	1	19.5	8.71779789								
7	1E+08	7_118050341	0	2	18	17	1	18	0.001742	0.10079	16	1	4	2	9	3	0.27712561	1	7.5	7.96420564	1E+08	1.2E+08	ID=ENSP00000343104;KCNK10;NM_021161;	KCNK10				
7	1E+08	7_118360567	39	68	11	8	54	10	5.89E-09	#######	47	58	58	13	53	36	0.35159657	1	37.75	24.0104144	1E+08	1.2E+08	ID=ENSP00000384682;ZC3H14;;	ZC3H14				
7	1E+08	7_118758384	19	10	0	8	15	4	0.015825	0.58672	40	22	1	3	31	2	8.42E-06	0.0010929	12.875	13.5903064								
7	1E+08	7_118943826	24	8	18	5	16	12	0.497978	1	16	11	1	0	14	1	0.00574567	0.2548307	10.375	8.50105036								

7	1E+08	7_118950510	17	12	2	7	15	5	0.034023	1	66	14	27	0	40	14	0.00488665	0.2236314	18.125	21.1757105								
7	1E+08	7_119085654	33	35	58	42	34	50	0.594713	1	98	44	32	40	71	36	0.01130697	0.4351594	47.75	21.9268264								
7	1E+08	7_119094817	189	158	162	155	174	159	0.074048	1	121	94	85	13	108	49	1.26E-04	0.0111328	122.13	56.7058513								
7	1E+08	7_119125027	40	33	37	45	37	41	0.969029	1	55	35	17	1	45	9	2.27E-05	0.0025764	32.875	16.80508					1.2E+08	1.2E+08	0.13618	ID=promoter_48609;
7	1E+08	7_119189205	100	94	11	18	97	15	7.46E-17	#######	69	41	50	2	55	26	0.01564739	0.5592438	48.125	37.2230247								
7	1E+08	7_119258865	153	229	164	209	191	187	0.212952	1	128	149	176	75	139	126	0.9835005	1	160.38	47.5873257								
7	1E+08	7_119308124	21	18	29	17	20	23	0.997913	1	53	0	4	0	27	2	5.20E-05	0.0052176	17.75	17.7260905					1.2E+08	1.2E+08	0.13369	ID=promoter_48620;
7	1E+08	7_119319973	8	16	8	4	12	6	0.213448	1	12	8	6	1	10	4	0.34296513	1	7.875	4.6117087								
7	1E+08	7_119646143	8	1	21	19	5	20	0.033145	1	22	5	1	5	14	3	0.04246461	1	10.25	8.95624283	1E+08	1.2E+08	ID=ENSP00000377099;TDP1;;	TDP1				
7	1E+08	7_119647576	4	11	2	9	8	6	0.705675	1	0	12	13	12	6	13	0.20797135	1	7.875	5.11126208	1E+08	1.2E+08	ID=ENSP00000377099;TDP1;;	TDP1				
7	1E+08	7_119690049	1	1	2	1	1	2	0.907382	1	45	8	25	11	27	18	0.55301708	1	11.75	15.7548179	1E+08	1.2E+08	ID=ENSP00000377099;TDP1;;	TDP1				
7	1E+08	7_119748971	35	91	34	18	63	26	1.69E-05	0.00188	43	10	39	7	27	23	0.95651383	1	34.625	26.4949456								
7	1E+08	7_119991256	94	73	67	49	84	58	0.007221	0.31244	77	63	74	9	70	42	0.08015226	1	63.25	25.3701174								
7	1E+08	7_120027767	72	28	42	43	50	43	0.325532	1	79	54	122	48	67	85	0.15585174	1	61	29.6599778								
7	1E+08	7_120106562	6	18	12	7	12	10	0.724827	1	13	19	31	0	16	16	0.97729577	1	13.25	9.55809306								
7	1E+08	7_120134330	13	34	12	20	24	16	0.207334	1	21	2	4	2	12	3	0.09316162	1	13.5	11.1867268	1E+08	1.2E+08	ID=ENSP00000336127;TTC7B;NM_001010854;	TTC7B				
7	1E+08	7_120177872	32	0	16	25	16	21	0.944185	1	1	0	1	0	1	1	0.99856566	1	9.375	13.1141962	1E+08	1.2E+08	ID=ENSP00000336127;TTC7B;NM_001010854;	TTC7B	1.2E+08	1.2E+08	0.1345	ID=promoter_48655;
7	1E+08	7_120280705	2	9	4	12	6	8	0.9722	1	0	3	11	0	2	6	0.31385293	1	5.125	4.85320218	1E+08	1.2E+08	ID=ENSP00000336127;TTC7B;NM_001010854;	TTC7B				
7	1E+08	7_120381356	42	5	46	21	24	34	0.777678	1	5	3	23	1	4	12	0.09850366	1	18.25	17.9104917								
7	1E+08	7_120465099	65	27	58	42	46	50	0.932872	1	15	8	3	1	12	2	0.04045894	1	27.375	25.0310521	1E+08	1.2E+08	ID=ENSP00000261991;RPS6KA5;NM_004755;	RPS6KA5				
7	1E+08	7_120874294	0	1	4	5	1	5	0.353258	1	23	1	3	20	12	12	0.98292303	1	7.125	9.06228448	1E+08	1.2E+08	ID=ENSP00000374507;CCDC88C;NM_001080414;	CCDC88C				
7	1E+08	7_120908580	12	33	18	17	23	18	0.432563	1	33	7	30	12	20	21	0.92353071	1	20.25	10.3337174					1.2E+08	1.2E+08	0.63658	ID=promoter_48697;
7	1E+08	7_121300505	61	26	23	55	44	39	0.441907	1	3	33	12	5	18	9	0.30718372	1	27.25	21.6382598								
7	1E+08	7_121443488	13	16	14	1	15	8	0.181584	1	21	18	6	4	20	5	0.01914528	0.6572777	11.625	7.15017482								
7	1E+08	7_121450717	23	5	4	0	14	2	0.003923	0.19308	7	4	13	0	6	7	0.91781435	1	7	7.6718409					1.2E+08	1.2E+08	0.10887	ID=promoter_48703;

7	1E+08	7_121474265	207	67	58	92	137	75	3.87E-07	#######	28	22	47	24	25	36	0.23362127	1	68.125	61.0513543					1.2E+08	1.2E+08	0.11996	ID=promoter_48705;
7	1E+08	7_121529924	588	200	397	366	394	382	0.029354	0.95954	138	160	166	32	149	99	0.03579778	1	255.88	179.982886					1.2E+08	1.2E+08	0.24074	ID=promoter_48708;
7	1E+08	7_121714930	35	89	21	37	62	29	1.29E-04	0.01118	78	29	24	3	54	14	3.32E-05	0.0035696	39.5	29.2379401								
7	1E+08	7_121736093	170	21	48	76	96	62	9.99E-04	0.06326	60	29	23	7	45	15	0.00163449	0.0924179	54.25	51.955338								
7	1E+08	7_121777375	67	32	49	30	50	40	0.192624	1	25	19	34	2	22	18	0.92557456	1	32.25	19.4183271								
7	1E+08	7_121818233	18	59	42	10	39	26	0.077552	1	22	56	38	14	39	26	0.42501095	1	32.375	19.0633343								
7	1E+08	7_121848435	406	129	388	371	268	380	0.058715	1	126	143	133	168	135	151	0.28363456	1	233	129.608201					1.2E+08	1.2E+08	0.53088	ID=promoter_48713;
7	1E+08	7_121852940	0	52	11	4	26	8	0.001755	0.10104	35	8	35	2	22	19	0.97472654	1	18.375	19.4858007								
7	1E+08	7_121920456	80	56	43	64	68	54	0.098398	1	14	20	10	2	17	6	0.10713464	1	36.125	28.6378246								
7	1E+08	7_121951097	69	33	62	62	51	62	0.982243	1	2	79	11	22	41	17	0.01650079	0.5857858	42.5	29.1253449								
7	1E+08	7_122130693	160	51	152	105	106	129	0.961263	1	0	0	8	21	0	15	3.07E-04	0.0235795	62.125	67.5688802	1E+08	1.2E+08	ID=ENSP00002164 87;RIN3; NM_024832;	RIN3				
7	1E+08	7_122154993	34	9	3	17	22	10	0.03588	1	0	0	1	0	0	1	0.58312783	1	8	12.0948631	1E+08	1.2E+08	ID=ENSP00002164 87;RIN3; NM_024832;	RIN3				
7	1E+08	7_122217859	30	9	42	24	20	33	0.480879	1	2	15	6	4	9	5	0.6500913	1	16.5	14.2628388	1E+08	1.2E+08	ID=ENSP00003769 11;LGMN; ;	LGMN				
7	1E+08	7_122255910	16	9	10	1	13	6	0.146758	1	0	16	7	0	8	4	0.59180275	1	7.375	6.63190341								
7	1E+08	7_122322405	8	21	15	14	15	15	0.908797	1	1	13	4	2	7	3	0.51857467	1	9.75	7.12640963	1E+08	1.2E+08	ID=ENSP00002164 87-D2;RIN3; NM_024832;	RIN3				
7	1E+08	7_122347446	1	15	3	4	8	4	0.357158	1	9	4	11	1	7	6	0.99011599	1	6	5.09901951								
7	1E+08	7_122412071	11	14	16	11	13	14	0.975695	1	0	10	0	1	5	1	0.30486099	1	7.875	6.53425042								
7	1E+08	7_122560934	10	34	0	2	22	1	1.12E-05	0.00132	13	12	0	3	13	2	0.02644099	0.8480056	9.25	11.3231747	1E+08	1.2E+08	ID=ENSP00003462 72;ITPK1 ;NM_001142594;	ITPK1				
7	1E+08	7_122613915	19	54	36	31	37	34	0.588357	1	10	19	40	2	15	21	0.47046428	1	26.375	17.045632								
7	1E+08	7_122635912	28	18	6	11	23	9	0.013495	0.51644	0	8	5	1	4	3	0.96209564	1	9.625	9.36463713					1.2E+08	1.2E+08	0.15616	ID=promoter_48764;
7	1E+08	7_122691164	12	12	18	17	12	18	0.803442	1	14	15	7	1	15	4	0.06170633	1	12	5.60611911	1E+08	1.2E+08	ID=ENSP00003843 99-D2;ITPK1 ;NM_001142593;	ITPK1	1.2E+08	1.2E+08	0.31416	ID=promoter_48770;
7	1E+08	7_122720006	21	20	13	19	21	16	0.42382	1	9	4	16	2	7	9	0.80856893	1	13	7.32900305	1E+08	1.2E+08	ID=ENSP00003843 99-D2;ITPK1 ;NM_001142593;	ITPK1				
7	1E+08	7_122758673	2	19	6	2	11	4	0.10516	1	3	6	12	1	5	7	0.77775394	1	6.375	6.20915223								
7	1E+08	7_123314280	45	19	8	36	32	22	0.148825	1	37	23	38	26	30	32	0.85844914	1	29	12.1655251								

7	1E+08	7_123374132	0	31	10	2	16	6	0.042495	1	12	9	13	0	11	7	0.73613127	1	9.625	10.1268737					1.2E+08	1.2E+08	0.64199	ID=promoter_48786;
7	1E+08	7_123417341	25	2	48	39	14	44	0.003735	0.1857	30	0	12	1	15	7	0.31064188	1	19.625	18.5852284								
7	1E+08	7_123445644	106	46	53	36	76	45	0.001043	0.06583	56	49	51	0	53	26	0.02589825	0.8377494	49.625	29.0169286					1.2E+08	1.2E+08	0.63659	ID=promoter_48791;
7	1E+08	7_123499260	81	67	44	42	74	43	9.22E-04	0.05911	8	21	9	19	15	14	0.99983448	1	36.375	27.0234686	1E+08	1.2E+08	ID=ENSP00000320675;ASB2;NM_016150;	ASB2				
7	1E+08	7_123512193	397	99	305	200	248	253	0.294733	1	71	36	46	61	54	54	0.92541771	1	151.88	135.267394	1E+08	1.2E+08	ID=ENSP00000320675;ASB2;NM_016150;	ASB2				
7	1E+08	7_123523527	0	76	8	0	38	4	5.55E-08	#######	35	7	8	17	21	13	0.51498325	1	18.875	25.6817751								
7	1E+08	7_123565713	117	35	116	122	76	119	0.142486	1	54	42	25	75	48	50	0.84654018	1	73.25	40.1061093								
7	1E+08	7_123683493	3	39	17	19	21	18	0.61832	1	17	27	20	15	22	18	0.92557456	1	19.625	10.2947629					1.2E+08	1.2E+08	0.43265	ID=promoter_48799;
7	1E+08	7_123696268	12	73	7	22	43	15	9.81E-05	0.00874	0	56	28	66	28	47	0.04140707	1	33	28.2084081								
7	1E+08	7_123723778	43	4	19	15	24	17	0.274507	1	16	14	24	0	15	12	0.93002578	1	16.875	13.0978461								
7	1E+08	7_123852431	14	51	11	19	33	15	0.006796	0.29841	29	44	20	4	37	12	0.00419427	0.1971915	24	16.3182632	1E+08	1.2E+08	ID=ENSP00000305924-D2;PPP4R4;NM_058237;	PPP4R4				
7	1E+08	7_123872815	20	40	17	19	30	18	0.072527	1	30	22	51	56	26	54	0.00241507	0.1280305	31.875	15.3198051	1E+08	1.2E+08	ID=ENSP00000305924-D2;PPP4R4;NM_058237;	PPP4R4				
7	1E+08	7_123908120	93	62	94	69	78	82	0.786659	1	12	36	13	20	24	17	0.69596948	1	49.875	34.2237908								
7	1E+08	7_123937830	11	37	4	1	24	3	4.60E-05	0.00451	5	5	25	0	5	13	0.12287893	1	11	13.1692282								
7	1E+08	7_124003279	27	3	14	0	15	7	0.113635	1	6	0	15	1	3	8	0.26303803	1	8.25	9.64735345								
7	1E+08	7_124101115	8	4	2	3	6	3	0.462004	1	18	6	15	3	12	9	0.89054267	1	7.375	5.99851172	1E+08	1.2E+08	ID=ENSP00000305924;PPP4R4;NM_058237;	PPP4R4				
7	1E+08	7_124196857	13	0	37	53	7	45	1.52E-05	0.00171	1	0	6	0	1	3	0.56022579	1	13.75	20.2537474								
7	1E+08	7_124263660	93	83	42	42	88	42	4.60E-06	0.0006	46	24	29	9	35	19	0.16432451	1	46	28.6655592								
7	1E+08	7_124303480	40	58	40	15	49	28	0.008152	0.34652	1	43	39	4	22	22	0.96891521	1	30	20.6189926								
7	1E+08	7_124381341	47	22	22	34	35	28	0.315717	1	37	27	21	1	32	11	0.01265194	0.4751394	26.375	13.6689168	1E+08	1.2E+08	ID=ENSP00000337133-D9;SERPINA9;NM_175739;	SERPINA9				
7	1E+08	7_124458013	22	1	8	9	12	9	0.592799	1	2	0	4	11	1	8	0.04988758	1	7.125	7.21976454								
7	1E+08	7_124546160	17	16	9	1	17	5	0.013234	0.50759	0	1	0	0	1	0	0.62906424	1	5.5	7.42582367								
7	1E+08	7_124991629	12	6	5	9	9	7	0.713906	1	3	0	6	0	2	3	0.86792893	1	5.125	4.15546112								
7	1E+08	7_125108370	0	27	18	1	14	10	0.480768	1	0	12	10	4	6	7	0.91781435	1	9	9.75412001					1.3E+08	1.3E+08	0.14183	ID=promoter_48850;
7	1E+08	7_125108418	67	37	66	20	52	43	0.233918	1	22	0	1	0	11	1	0.02232329	0.7455867	26.625	27.8461975					1.3E+08	1.3E+08	0.14183	ID=promoter_48850;

7	1E+08	7_125176123	0	10	0	18	5	9	0.74134	1	22	9	41	5	16	23	0.39752759	1	13.125	13.7158667								
7	1E+08	7_125197091	8	12	23	18	10	21	0.306738	1	33	20	7	31	27	19	0.6528836	1	19	9.79795897								
7	1E+08	7_125229064	8	11	8	2	10	5	0.276106	1	0	19	22	2	10	12	0.83305499	1	9	8.05339325	1E+08	1.3E+08	ID=ENSP00000376783;DICER1;;	DICER1	1.3E+08	1.3E+08	0.51588	ID=promoter_48866;
7	1E+08	7_125239021	22	42	35	38	32	37	0.998236	1	79	21	38	19	50	29	0.12800836	1	36.75	19.270629	1E+08	1.3E+08	ID=ENSP00000376783;DICER1;;	DICER1				
7	1E+08	7_125239841	18	107	8	26	63	17	3.23E-08	#######	71	32	51	22	52	37	0.46718443	1	41.875	33.0256935	1E+08	1.3E+08	ID=ENSP00000376783;DICER1;;	DICER1	1.3E+08	1.3E+08	0.33612	ID=promoter_48868;
7	1E+08	7_125280911	6	68	36	23	37	30	0.319086	1	25	52	47	53	39	50	0.31296206	1	38.75	20.069522								
7	1E+08	7_125327342	45	26	53	32	36	43	0.996776	1	0	0	7	0	0	4	0.11562589	1	20.375	21.5866195	1E+08	1.3E+08	ID=ENSP00000298912;CLMN;NM_024734;	CLMN	1.3E+08	1.3E+08	0.13964	ID=promoter_48885;
7	1E+08	7_125333107	5	10	3	1	8	2	0.097885	1	0	23	17	15	12	16	0.63262749	1	9.25	8.39642781	1E+08	1.3E+08	ID=ENSP00000298912;CLMN;NM_024734;	CLMN				
7	1E+08	7_125536780	4	15	4	2	10	3	0.082185	1	21	13	9	10	17	10	0.5154613	1	9.75	6.4531277	1E+08	1.3E+08	ID=ENSP00000334308;C14orf49;NM_152592;	C14orf49				
7	1E+08	7_125661943	5	13	4	6	9	5	0.390586	1	0	19	19	15	10	17	0.30316389	1	10.125	7.29848321								
7	1E+08	7_125756589	428	465	394	456	447	425	0.008056	0.34472	520	214	431	114	367	273	0.02218175	0.7448668	377.75	139.37283								
7	1E+08	7_125964309	1	2	3	14	2	9	0.179815	1	17	0	9	0	9	5	0.6500913	1	5.75	6.71352579					1.3E+08	1.3E+08	0.14057	ID=promoter_48921;
7	1E+08	7_126116235	86	77	154	153	82	154	0.002386	0.12916	69	106	67	128	88	98	0.45756909	1	105	36.0951125								
7	1E+08	7_126127306	1	3	10	21	2	16	0.012093	0.47152	9	1	21	27	5	24	9.36E-04	0.0596362	11.625	10.155048								
7	1E+08	7_126203400	5	0	0	0	3	0	0.172143	1	36	21	7	0	29	4	1.92E-04	0.0158648	8.625	13.1793941					1.3E+08	1.3E+08	0.11553	ID=promoter_48926;
7	1E+08	7_126254650	4	1	4	11	3	8	0.459247	1	0	14	6	12	7	9	0.80856893	1	6.5	5.23722937					1.3E+08	1.3E+08	0.13592	ID=promoter_48928;
7	1E+08	7_126294541	19	8	15	59	14	37	0.029025	0.94901	2	9	36	3	6	20	0.0132456	0.49354	18.875	19.5333963					1.3E+08	1.3E+08	0.13001	ID=promoter_48932;
7	1E+08	7_126300901	36	2	6	13	19	10	0.108738	1	51	31	10	3	41	7	2.03E-05	0.0023546	19	18.0791909								
7	1E+08	7_126323777	449	128	326	268	289	297	0.277429	1	290	190	119	149	240	134	1.25E-05	0.0015294	239.88	114.845782								
7	1E+08	7_126325042	1	26	4	31	14	18	0.967347	1	19	3	10	0	11	5	0.40227598	1	11.75	12.0682582								
7	1E+08	7_126348998	57	0	18	8	29	13	0.011304	0.44898	49	41	49	5	45	27	0.1974124	1	28.375	23.0027172					1.3E+08	1.3E+08	0.12857	ID=promoter_48936;
7	1E+08	7_126429578	1	8	14	7	5	11	0.495311	1	1	0	16	4	1	10	0.01766414	0.6154554	6.375	6.06953752								
7	1E+08	7_126834550	8	50	9	5	29	7	1.77E-04	0.01461	18	6	41	5	12	23	0.11381058	1	17.75	17.7904469								
7	1E+08	7_126851336	187	94	83	80	141	82	1.69E-06	0.00024	325	129	150	92	227	121	4.13E-06	0.0005798	142.5	82.6991104								
7	1E+08	7_126866546	17	47	10	26	32	18	0.038646	1	95	30	16	22	63	19	3.67E-05	0.0038816	32.875	27.4977272								
7	1E+08	7_126877946	18	14	44	28	16	36	0.087649	1	37	16	13	0	27	7	0.00575936	0.2548307	21.25	14.2503133								
7	1E+08	7_126970898	69	145	70	102	107	86	0.031836	1	209	92	150	71	151	111	0.17740634	1	113.5	50.3104647								
7	1E+08	7_126983525	13	28	15	12	21	14	0.252399	1	16	8	41	43	12	42	7.33E-05	0.0070383	22	13.6277029								

7	1E+08	7_126990614	0	12	15	17	6	16	0.210908	1	1	8	11	2	5	7	0.77775394	1	8.25	6.5846141					1.3E+08	1.3E+08	0.38921	ID=promoter_48965;
7	1E+08	7_127046735	41	58	22	14	50	18	3.18E-05	0.00328	51	13	16	30	32	23	0.64682985	1	30.625	17.5168286								
7	1E+08	7_127057632	90	70	86	79	80	83	0.735585	1	24	20	34	37	22	36	0.10388249	1	55	29.1253449								
7	1E+08	7_127093357	3	6	17	16	5	17	0.090491	1	0	0	8	17	0	13	9.01E-04	0.0577931	8.375	7.38603895					1.3E+08	1.3E+08	0.22051	ID=promoter_48974;
7	1E+08	7_127347222	154	85	60	49	120	55	1.66E-08	#######	147	35	55	24	91	40	3.03E-04	0.0233982	76.125	49.3049911								
7	1E+08	7_127373525	8	2	10	11	5	11	0.495311	1	4	14	6	0	9	3	0.27712561	1	6.875	4.76407689								
7	1E+08	7_127458105	440	160	239	238	300	239	4.20E-05	0.00416	55	26	37	17	41	27	0.38241111	1	151.5	148.66357								
7	1E+08	7_127486422	2	35	6	1	19	4	0.002035	0.11403	9	19	12	15	14	14	0.98010536	1	12.375	11.0316104								
7	1E+08	7_127495107	6	11	0	11	9	6	0.554309	1	12	0	0	0	6	0	0.06196836	1	5	5.63154381								
7	1E+08	7_128492775	1168	951	1010	846	1060	928	5.50E-10	#######	1330	1121	759	367	1226	563	8.47E-44	1.44E-40	944	295.602049								
7	1E+08	7_128542850	12	9	21	24	11	23	0.278506	1	21	0	14	0	11	7	0.73613127	1	12.625	9.28804916								
7	1E+08	7_128551578	50	50	20	23	50	22	3.52E-04	0.02658	0	0	12	0	0	6	0.0393172	1	19.375	20.9416707								
7	1E+08	7_128652011	44	54	26	23	49	25	0.0023	0.12489	84	5	28	1	45	15	0.00163449	0.0924179	33.125	27.1105541								
7	1E+08	7_128720006	33	60	39	34	47	37	0.188658	1	31	21	16	20	26	18	0.63344191	1	31.75	13.9360786								
7	1E+08	7_128771758	78	224	58	103	151	81	4.25E-08	#######	137	117	215	80	127	148	0.16587584	1	126.5	62.4476924								
7	1E+08	7_128773371	33	30	19	22	32	21	0.11093	1	39	0	34	20	20	27	0.43992527	1	24.625	12.3049061								
7	1E+08	7_128824216	6	9	6	4	8	5	0.532535	1	9	1	11	7	5	9	0.48000906	1	6.625	3.15945294								
7	1E+08	7_128902256	3	12	5	19	8	12	0.864244	1	0	3	7	30	2	19	5.26E-04	0.0367435	9.875	10.1198179								
7	1E+08	7_128922040	8	33	3	8	21	6	0.004166	0.20266	9	43	26	0	26	13	0.18179217	1	16.25	15.6729977								
7	1E+08	7_129331236	5	21	0	22	13	11	0.725732	1	9	52	40	9	31	25	0.87406593	1	19.75	18.1087982								
7	1E+08	7_129388375	306	58	220	126	182	173	0.139821	1	43	68	30	15	56	23	0.0033198	0.1660864	108.25	103.599159								
7	1E+08	7_129472476	62	37	26	19	50	23	5.99E-04	0.04108	1	16	7	1	9	4	0.4571786	1	21.125	20.6566316								
7	1E+08	7_129478020	16	20	9	9	18	9	0.098614	1	18	17	3	1	18	2	0.00297675	0.1507727	11.625	7.17012652								
7	1E+08	7_129488244	12	7	3	0	10	2	0.03464	1	0	15	0	3	8	2	0.20550608	1	5	5.80640041	1E+08	1.3E+08	ID=ENSP00000349723;BCL11B;NM_138576;	BCL11B	1.3E+08	1.3E+08	0.86016	ID=promoter_49035;
7	1E+08	7_129512055	152	285	522	734	219	628	7.87E-31	#######	137	100	190	121	119	156	0.016692	0.5919849	280.13	229.072314	1E+08	1.3E+08	ID=ENSP00000349723;BCL11B;NM_138576;	BCL11B				
7	1E+08	7_129525882	10	37	27	8	24	18	0.351411	1	33	0	9	37	17	23	0.4932031	1	20.125	14.9325866	1E+08	1.3E+08	ID=ENSP00000349723;BCL11B;NM_138576;	BCL11B	1.3E+08	1.3E+08	0.1685	ID=promoter_49045;
7	1E+08	7_129570255	0	20	15	22	10	19	0.459788	1	36	10	13	16	23	15	0.56706676	1	16.5	10.3647755					1.3E+08	1.3E+08	0.16643	ID=promoter_49055;
7	1E+08	7_129662623	27	17	44	26	22	35	0.531372	1	14	1	0	0	8	0	0.02452223	0.7977568	16.125	15.7972647								
7	1E+08	7_129710264	157	34	129	129	96	129	0.600894	1	138	39	28	37	89	33	1.97E-05	0.0022887	86.375	56.2137185	1E+08	1.3E+08	ID=ENSP00000327436;SETD3;NM_032233;	SETD3	1.3E+08	1.3E+08	0.28641	ID=promoter_49075;

7	1E+08	7_129723994	1	21	2	14	11	8	0.5832	1	16	7	7	4	12	6	0.45526626	1	9	7.21110255	1E+08	1.3E+08	ID=ENSP00000327436;SETD3;NM_032233;	SETD3				
7	1E+08	7_129725337	203	145	326	378	174	352	2.00E-08	#######	298	492	539	175	395	357	0.94132011	1	319.5	144.798382	1E+08	1.3E+08	ID=ENSP00000327436;SETD3;NM_032233;	SETD3	1.3E+08	1.3E+08	0.13052	ID=promoter_49078;
7	1E+08	7_129833740	72	10	129	125	41	127	1.38E-07	#######	11	16	70	15	14	43	1.98E-04	0.0163228	56	50.6585206								
7	1E+08	7_129890670	8	18	6	3	13	5	0.083134	1	0	5	8	2	3	5	0.7175395	1	6.25	5.52268051								
7	1E+08	7_129915727	7	10	5	11	9	8	0.848833	1	4	2	3	4	3	4	0.89184014	1	5.75	3.28416112					1.3E+08	1.3E+08	0.43802	ID=promoter_49107;
7	1E+08	7_130244347	7	4	5	7	6	6	0.962469	1	18	14	8	15	16	12	0.85678722	1	9.75	5.1754917					1.3E+08	1.3E+08	0.34806	ID=promoter_49139;
7	1E+08	7_130254985	30	29	36	15	30	26	0.525876	1	1	18	11	4	10	8	0.95278883	1	18	12.7166932					1.3E+08	1.3E+08	0.12034	ID=promoter_49140;
7	1E+08	7_130279123	3	42	35	18	23	27	0.999996	1	14	23	16	2	19	9	0.23949138	1	19.125	14.0452076					1.3E+08	1.3E+08	0.16562	ID=promoter_49146;
7	1E+08	7_130310455	8	15	5	11	12	8	0.45534	1	23	3	16	6	13	11	0.97545578	1	10.875	6.74933859					1.3E+08	1.3E+08	0.4155	ID=promoter_49156;
7	1E+08	7_130326570	633	214	383	425	424	404	0.011281	0.44898	200	191	267	80	196	174	0.91719051	1	299.13	174.09475					1.3E+08	1.3E+08	0.46831	ID=promoter_49161;
7	1E+08	7_130330705	144	67	65	84	106	75	0.003241	0.16636	111	78	62	32	95	47	0.00165098	0.0930606	80.375	34.0123402					1.3E+08	1.3E+08	0.12753	ID=promoter_49162;
7	1E+08	7_130410634	0	24	9	9	12	9	0.592799	1	19	8	8	1	14	5	0.16413709	1	9.75	8.17225271								
7	1E+08	7_130410903	28	27	29	11	28	20	0.231138	1	6	11	62	4	9	33	4.07E-04	0.0298473	22.25	19.0169098								
7	1E+08	7_130433452	264	101	180	163	183	172	0.112557	1	71	21	25	0	46	13	3.24E-04	0.0246729	103.13	92.5008687					1.3E+08	1.3E+08	0.58869	ID=promoter_49174;
7	1E+08	7_130471667	27	6	27	30	17	29	0.467377	1	0	0	10	3	0	7	0.02292696	0.7568963	12.875	12.9663025					1.3E+08	1.3E+08	0.11245	ID=promoter_49183;
7	1E+08	7_130473619	31	7	60	25	19	43	0.05234	1	0	0	16	4	0	10	0.00454609	0.2107253	17.875	20.5317698					1.3E+08	1.3E+08	0.20176	ID=promoter_49184;
7	1E+08	7_130585119	53	7	18	25	30	22	0.240296	1	0	6	7	11	3	9	0.17582851	1	15.875	16.8898744					1.3E+08	1.3E+08	0.20176	ID=promoter_49208;
7	1E+08	7_130587080	16	21	45	31	19	38	0.157767	1	16	14	5	7	15	6	0.20085144	1	19.375	13.125085					1.3E+08	1.3E+08	0.11933	ID=promoter_49209;
7	1E+08	7_130587782	432	199	1220	1564	316	1392	########	#######	70	14	188	25	42	107	5.21E-08	1.14E-05	464	595.149201								
7	1E+08	7_130625302	265	25	240	340	145	290	7.58E-07	0.00012	91	21	71	3	56	37	0.27526121	1	132	130.071409					1.3E+08	1.3E+08	0.59384	ID=promoter_49219;
7	1E+08	7_130651874	22	14	6	27	18	17	0.813792	1	12	0	12	21	6	17	0.046525	1	14.25	8.86002257								
7	1E+08	7_130653500	43	82	29	29	63	29	8.79E-05	0.00792	95	99	121	77	97	99	0.79715025	1	71.875	34.4816204					1.3E+08	1.3E+08	0.15942	ID=promoter_49226;
7	1E+08	7_130659162	2	32	12	12	17	12	0.395844	1	16	25	32	1	21	17	0.91718448	1	16.5	12.2124059								
7	1E+08	7_130707142	7	20	16	7	14	12	0.725787	1	0	2	17	4	1	11	0.01045793	0.4067594	9.125	7.52970309								
7	1E+08	7_130838756	5	63	3	12	34	8	3.31E-05	0.00339	33	10	21	18	22	20	0.99799876	1	20.625	19.63188					1.3E+08	1.3E+08	0.41555	ID=promoter_49247;
7	1E+08	7_130840985	10	38	44	7	24	26	0.961326	1	6	31	64	7	19	36	0.03633239	1	25.875	21.7612861								
7	1E+08	7_130860056	5	20	23	7	13	15	0.999061	1	10	7	8	3	9	6	0.8208167	1	10.375	7.20986427								

7	1E+08	7_130870996	12	10	8	14	11	11	0.932271	1	4	35	22	7	20	15	0.82431124	1	14	10.0711753	1E+08	1.3E+08	ID=ENSP0000355237;CDC42BPB;NM_006035;	CDC42BPB	1.3E+08	1.3E+08	0.8144	ID=promoter_49253;
7	1E+08	7_130872009	4	28	4	3	16	4	0.009581	0.39335	5	7	4	4	6	4	0.87665919	1	7.375	8.41660773	1E+08	1.3E+08	ID=ENSP0000355237;CDC42BPB;NM_006035;	CDC42BPB	1.3E+08	1.3E+08	0.8144	ID=promoter_49253;
7	1E+08	7_130894115	12	41	6	8	27	7	4.86E-04	0.03443	0	26	9	0	13	5	0.22538616	1	12.75	14.0483858	1E+08	1.3E+08	ID=ENSP0000355237;CDC42BPB;NM_006035;	CDC42BPB	1.3E+08	1.3E+08	0.72054	ID=promoter_49259;
7	1E+08	7_130903954	107	103	118	120	105	119	0.967601	1	56	29	43	65	43	54	0.32914056	1	80.125	36.0017361	1E+08	1.3E+08	ID=ENSP0000355237;CDC42BPB;NM_006035;	CDC42BPB	1.3E+08	1.3E+08	0.43322	ID=promoter_49262;
7	1E+08	7_130953579	22	6	16	2	14	9	0.360318	1	5	14	26	14	10	20	0.12622058	1	13.125	8.40811683					1.3E+08	1.3E+08	0.23801	ID=promoter_49275;
7	1E+08	7_130956239	7	0	12	3	4	8	0.677894	1	47	51	28	27	49	28	0.11921514	1	21.875	19.6282413					1.3E+08	1.3E+08	0.25902	ID=promoter_49276;
7	1E+08	7_131020925	18	10	8	12	14	10	0.480768	1	8	6	26	11	7	19	0.03889743	1	12.375	6.58868077								
7	1E+08	7_131033217	27	94	43	29	61	36	0.003872	0.19137	110	63	73	100	87	87	0.88260517	1	67.375	32.3769649								
7	1E+08	7_131081905	2	26	27	31	14	29	0.208355	1	0	28	10	2	14	6	0.26891969	1	15.75	13.48809								
7	1E+08	7_131095483	24	14	29	6	19	18	0.809248	1	22	16	40	49	19	45	0.00179902	0.1003004	25	14.0915375					1.3E+08	1.3E+08	0.43376	ID=promoter_49290;
7	1E+08	7_131103212	0	2	9	12	1	11	0.032372	1	21	12	0	1	17	1	0.00146248	0.085933	7.125	7.64269025					1.3E+08	1.3E+08	0.18976	ID=promoter_49293;
7	1E+08	7_131192299	12	17	2	12	15	7	0.113635	1	3	5	11	1	4	6	0.75330063	1	7.875	5.86606463	1E+08	1.3E+08	ID=ENSP0000262241;RCOR1;NM_015156;	RCOR1				
7	1E+08	7_131236101	328	146	351	201	237	276	0.996714	1	108	125	57	22	117	40	1.02E-07	2.11E-05	167.25	119.369474					1.3E+08	1.3E+08	0.73776	ID=promoter_49303;
7	1E+08	7_131239884	44	35	13	22	40	18	0.002142	0.11834	13	14	32	2	14	17	0.75475782	1	21.875	14.0350327								
7	1E+08	7_131282866	16	70	39	79	43	59	0.736066	1	36	24	23	6	30	15	0.13985038	1	36.625	25.7012368								
7	1E+08	7_131395350	13	38	15	14	26	15	0.082345	1	27	11	24	3	19	14	0.80779431	1	18.125	10.9601551					1.3E+08	1.3E+08	0.4712	ID=promoter_49330;
7	1E+08	7_131402867	10	21	10	21	16	16	0.903021	1	21	4	27	0	13	14	0.92696813	1	14.25	9.58793885								
7	1E+08	7_131441285	5116	3239	6259	5833	4178	6046	1.76E-24	#######	3630	1805	2475	1318	2718	1897	2.08E-20	1.43E-17	3709.4	1854.93619								
7	1E+08	7_131459699	122	124	86	64	123	75	3.26E-05	0.00335	129	115	128	49	122	89	0.22660209	1	102.13	31.5478661	1E+08	1.3E+08	ID=ENSP0000216756-D2;;NM_032630;					
7	1E+08	7_131595269	161	82	203	137	122	170	0.347731	1	0	13	13	4	7	9	0.80856893	1	76.625	81.0695248	1E+08	1.3E+08	ID=ENSP0000262236;ZNF839;;	ZNF839	1.3E+08	1.3E+08	0.27333	ID=promoter_49368;
7	1E+08	7_131627985	83	46	44	49	65	47	0.0386	1	24	13	31	0	19	16	0.96066018	1	36.25	25.4544411					1.3E+08	1.3E+08	0.51715	ID=promoter_49373;
7	1E+08	7_131684099	20	16	2	3	18	3	0.001281	0.07728	13	6	16	5	10	11	0.92604858	1	10.125	6.91659495								
7	1E+08	7_131694717	1	5	1	0	3	1	0.508575	1	19	0	12	2	10	7	0.84912739	1	5	6.92820323					1.3E+08	1.3E+08	0.61006	ID=promoter_49389;

7	1E+08	7_131700793	3	20	6	2	12	4	0.066899	1	20	16	7	1	18	4	0.01933304	0.6616555	9.375	8.03452372	1E+08	1.3E+08	ID=ENSP00000341037;WDR20;NM_144574;	WDR20				
7	1E+08	7_131778689	5	1	1	3	3	2	0.821455	1	19	55	9	6	37	8	2.51E-04	0.0199657	12.375	18.1811323								
7	1E+08	7_131841267	1	19	6	4	10	5	0.276106	1	22	9	28	10	16	19	0.76520201	1	12.375	9.54594155					1.3E+08	1.3E+08	0.35759	ID=promoter_49408;
7	1E+08	7_131844472	2	8	1	18	5	10	0.615109	1	28	6	17	4	17	11	0.64636287	1	10.5	9.53190132					1.3E+08	1.3E+08	0.59862	ID=promoter_49409;
7	1E+08	7_131884231	0	18	5	7	9	6	0.554309	1	32	6	5	1	19	3	0.00532306	0.24159	9.25	10.6871084					1.3E+08	1.3E+08	0.99674	ID=promoter_49419;
7	1E+08	7_131890526	7	3	15	3	5	9	0.74134	1	1	9	41	9	5	25	5.78E-04	0.0398304	11	12.9173195					1.3E+08	1.3E+08	0.99674	ID=promoter_49419;
7	1E+08	7_131903888	23	54	2	26	39	14	3.02E-04	0.02327	0	3	13	45	2	29	2.70E-06	0.0004028	20.75	20.2960235					1.3E+08	1.3E+08	0.44105	ID=promoter_49420;
7	1E+08	7_131905521	52	79	39	25	66	32	1.27E-04	0.01105	37	60	28	46	49	37	0.64071519	1	45.75	17.7904469					1.3E+08	1.3E+08	0.54787	ID=promoter_49421;
7	1E+08	7_131927586	127	68	99	59	98	79	0.044492	1	138	77	79	14	108	47	5.44E-05	0.0054393	82.625	39.3335099					1.3E+08	1.3E+08	0.74637	ID=promoter_49428;
7	1E+08	7_131937691	8	20	21	18	14	20	0.851664	1	23	10	31	31	17	31	0.07354669	1	20.25	8.44731572					1.3E+08	1.3E+08	0.585	ID=promoter_49429;
7	1E+08	7_131944005	21	3	8	8	12	8	0.45534	1	1	3	10	3	2	7	0.20455692	1	7.125	6.44620597								
7	1E+08	7_131948855	8	0	2	0	4	1	0.312866	1	37	20	4	0	29	2	2.09E-05	0.0024085	8.875	13.2172777								
7	1E+08	7_131962652	1	13	1	7	7	4	0.503048	1	22	0	4	2	11	3	0.13591373	1	6.25	7.66718425								
7	1E+08	7_132007672	12	14	0	16	13	8	0.344217	1	0	0	0	0	0	0	1	1	5.25	7.32412842					1.3E+08	1.3E+08	0.16124	ID=promoter_49455;
7	1E+08	7_132026921	11	15	4	5	13	5	0.083134	1	1	11	3	2	6	3	0.67473421	1	6.5	5.12695956								
7	1E+08	7_132053953	1	15	8	27	8	18	0.296056	1	51	7	15	2	29	9	0.01067645	0.4139111	15.75	16.5335157								
7	1E+08	7_132071744	49	88	19	30	69	25	6.89E-07	0.00011	47	78	33	57	63	45	0.40770129	1	50.125	23.7152001					1.3E+08	1.3E+08	0.36136	ID=promoter_49464;
7	1E+08	7_132244865	129	66	132	160	98	146	0.185562	1	21	38	12	19	30	16	0.19481252	1	72.125	59.5157542								
7	1E+08	7_132258519	351	134	328	249	243	289	0.987437	1	162	104	98	88	133	93	0.11185389	1	189.25	105.982141					1.3E+08	1.3E+08	0.20895	ID=promoter_49479;
7	1E+08	7_132269360	16	40	12	7	28	10	0.00287	0.14985	223	151	93	132	187	113	0.00143087	0.0846326	84.25	79.1630325								
7	1E+08	7_132276448	62	7	64	24	35	44	0.954231	1	125	13	27	3	69	15	2.06E-07	4.03E-05	40.625	41.220444								
7	1E+08	7_132368482	219	137	289	215	178	252	0.157035	1	177	91	127	55	134	91	0.07093535	1	163.75	76.27722								
7	1E+08	7_132415983	10	2	6	30	6	18	0.118314	1	6	3	0	0	5	0	0.09850879	1	7.125	9.8479512					1.3E+08	1.3E+08	0.21199	ID=promoter_49509;
7	1E+08	7_132496652	17	7	11	5	12	8	0.45534	1	18	12	9	3	15	6	0.20085144	1	10.25	5.36523199								
7	1E+08	7_132549552	8	35	29	23	22	26	0.999662	1	34	35	51	38	35	45	0.34560916	1	31.625	12.4434435								
7	1E+08	7_132605918	0	16	3	4	8	4	0.357158	1	2	33	12	0	18	6	0.0767986	1	8.75	11.3735282								
7	1E+08	7_132715820	10	31	8	6	21	7	0.0088	0.36783	1	6	27	4	4	16	0.01700101	0.6008545	11.625	11.0961705								
7	1E+08	7_132769960	19	31	28	0	25	14	0.076795	1	30	35	16	20	33	18	0.18694358	1	22.375	11.2241767	1E+08	1.3E+08	ID=ENSP0000375751-D2;TP53BP2;;	TP53BP2	1.3E+08	1.3E+08	0.1355	ID=promoter_49543;

7	1E+08	7_132786589	35	12	12	38	24	25	0.917255	1	13	18	26	1	16	14	0.9876114	1	19.375	12.6709736	1E+08	1.3E+08	ID=ENSP0000375751-D2;TP53BP2;;	TP53BP2	1.3E+08	1.3E+08	0.87413	ID=promoter_49547;
7	1E+08	7_132788179	9	3	2	1	6	2	0.258649	1	0	4	2	24	2	13	0.01148244	0.4380166	5.625	7.90908158					1.3E+08	1.3E+08	0.87413	ID=promoter_49547;
7	1E+08	7_132799572	28	29	15	38	29	27	0.687522	1	0	14	8	18	7	13	0.3188207	1	18.75	12.3375154					1.3E+08	1.3E+08	0.24865	ID=promoter_49549;
7	1E+08	7_132801509	91	33	98	91	62	95	0.259915	1	97	19	8	8	58	8	3.68E-08	8.35E-06	55.625	42.0881303					1.3E+08	1.3E+08	0.46374	ID=promoter_49550;
7	1E+08	7_132856915	8	5	21	6	7	14	0.506445	1	0	2	4	0	1	2	0.81304206	1	5.75	6.77706215	1E+08	1.3E+08	ID=ENSP0000341154;KLC1;NM_182923;	KLC1	1.3E+08	1.3E+08	0.1312	ID=promoter_49564;
7	1E+08	7_132899946	1	31	8	0	16	4	0.009581	0.39335	27	12	0	27	20	14	0.72101732	1	13.25	13.2206548					1.3E+08	1.3E+08	0.15572	ID=promoter_49568;
7	1E+08	7_132985903	587	297	524	541	442	533	0.911547	1	265	217	268	82	241	175	0.04786553	1	347.63	180.930877								
7	1E+08	7_133001268	1282	634	1521	1088	958	1305	0.002045	0.11447	1615	425	553	707	1020	630	7.27E-15	3.69E-12	978.13	460.332935					1.3E+08	1.3E+08	0.39773	ID=promoter_49581;
7	1E+08	7_133017738	178	62	92	130	120	111	0.195199	1	57	79	71	32	68	52	0.57827579	1	87.625	46.3247465	1E+08	1.3E+08	ID=ENSP0000387303;TDRD9;;	TDRD9	1.3E+08	1.3E+08	0.48693	ID=promoter_49583;
7	1E+08	7_133070454	67	90	66	56	79	61	0.047234	1	58	69	53	62	64	58	0.99220102	1	65.125	11.5193936	1E+08	1.3E+08	ID=ENSP0000387303;TDRD9;;	TDRD9	1.3E+08	1.3E+08	0.24003	ID=promoter_49598;
7	1E+08	7_133085077	4	8	22	39	6	31	0.00134	0.08025	2	44	25	21	23	23	0.96752545	1	20.625	15.5282002	1E+08	1.3E+08	ID=ENSP0000387303;TDRD9;;	TDRD9				
7	1E+08	7_133093753	187	162	295	145	175	220	0.791165	1	105	81	79	36	93	58	0.05822081	1	136.25	80.9528081								
7	1E+08	7_133099782	4	18	13	7	11	10	0.842816	1	1	6	6	28	4	17	0.01069978	0.4139111	10.375	8.89522344								
7	1E+08	7_133105461	10	4	9	7	7	8	0.998739	1	9	35	19	2	22	11	0.23631249	1	11.875	10.6158574					1.3E+08	1.3E+08	0.44295	ID=promoter_49607;
7	1E+08	7_133131137	59	73	63	61	66	62	0.453236	1	63	49	64	25	56	45	0.77467809	1	57.125	14.5645117	1E+08	1.3E+08	ID=ENSP0000376372;PLD4;NM_138790;	PLD4	1.3E+08	1.3E+08	0.30702	ID=promoter_49613;
7	1E+08	7_133135729	10	4	1	4	7	3	0.314307	1	25	5	15	4	15	10	0.7195765	1	8.5	7.98212288					1.3E+08	1.3E+08	0.85266	ID=promoter_49614;
7	1E+08	7_133139990	4	9	0	6	7	3	0.314307	1	10	0	17	0	5	9	0.48000906	1	5.75	6.06512278					1.3E+08	1.3E+08	0.85266	ID=promoter_49614;
7	1E+08	7_133140506	30	38	48	126	34	87	4.01E-04	0.02951	49	36	68	17	43	43	0.94014517	1	51.5	33.6282194					1.3E+08	1.3E+08	0.85266	ID=promoter_49614;
7	1E+08	7_133167730	28	9	12	16	19	14	0.411928	1	14	15	31	18	15	25	0.19020812	1	17.875	7.69856202	1E+08	1.3E+08	ID=ENSP0000328169;JAG2;NM_002226;	JAG2	1.3E+08	1.3E+08	0.41992	ID=promoter_49622;
7	1E+08	7_133194858	0	7	4	7	4	6	0.929647	1	13	1	12	2	7	7	0.99000271	1	5.75	4.89168391								
7	1E+08	7_133197687	16	5	35	31	11	33	0.019979	0.70661	1	0	3	0	1	2	0.81304206	1	11.375	14.352078					1.3E+08	1.3E+08	0.21705	ID=promoter_49628;
7	1E+08	7_133227450	344	119	490	288	232	389	8.47E-05	0.00765	151	135	169	60	143	115	0.52458581	1	219.5	143.071411					1.3E+08	1.3E+08	0.88301	ID=promoter_49634;
7	1E+08	7_133256967	51	9	33	31	30	32	0.932483	1	0	3	0	1	2	1	0.87709017	1	16	19.6177762					1.3E+08	1.3E+08	0.24538	ID=promoter_49642;

7	1E+08	7_133275085	13	37	19	15	25	17	0.214068	1	27	9	13	52	18	33	0.0599435	1	23.125	14.7787251								
7	1E+08	7_133287602	20	29	34	13	25	24	0.781475	1	28	33	19	27	31	23	0.7197219	1	25.375	7.3472541								
7	1E+08	7_133812192	19	60	24	50	40	37	0.580086	1	60	26	46	5	43	26	0.2226398	1	36.25	20.4991289					1.3E+08	1.3E+08	0.12744	ID=promoter_49654;
7	1E+08	7_133854589	22	64	27	36	43	32	0.144857	1	63	62	56	34	63	45	0.40770129	1	45.5	17.5173383								
7	1E+08	7_133855992	4	25	8	1	15	5	0.034023	1	36	1	17	22	19	20	0.92427823	1	14.25	12.8257553								
7	1E+08	7_133951272	5	0	6	11	3	9	0.343967	1	19	5	8	17	12	13	0.92692342	1	8.875	6.44620597					1.3E+08	1.3E+08	0.18333	ID=promoter_49676;
7	1E+08	7_133962832	8	6	12	2	7	7	0.956352	1	11	9	0	25	10	13	0.72167512	1	9.125	7.64269025								
7	1E+08	7_134005089	129	30	105	101	80	103	0.822338	1	153	68	98	19	111	59	0.00220978	0.1194491	87.875	46.2738356								
7	1E+08	7_134054131	11	0	3	13	6	8	0.9722	1	26	10	17	0	18	9	0.30718372	1	10	8.9761589								
7	1E+08	7_134076925	5	5	2	15	5	9	0.74134	1	8	1	24	3	5	14	0.08295177	1	7.875	7.86379588								
7	1E+08	7_134144411	16	8	14	0	12	7	0.325249	1	4	14	4	21	9	13	0.58766318	1	10.125	7.21976454								
7	1E+08	7_134186736	29	18	39	15	24	27	0.98899	1	0	6	11	3	3	7	0.3829009	1	15.125	13.3463478					1.3E+08	1.3E+08	0.1791	ID=promoter_49717;
7	1E+08	7_134244976	67	0	16	12	34	14	0.00261	0.13949	15	16	9	0	16	5	0.08348313	1	16.875	21.2900882								
7	1E+08	7_135068680	16	11	5	6	14	6	0.098789	1	0	41	6	0	21	3	0.0022553	0.1213334	10.625	13.3730806								
7	1E+08	7_135422641	11	13	0	1	12	1	0.003675	0.18279	12	3	28	0	8	14	0.34554715	1	8.5	9.63624112	1E+08	1.4E+08	ID=ENSP0000360107;EFHC1;NM_018100;	EFHC1				
7	1E+08	7_135452030	13	23	5	16	18	11	0.22238	1	52	28	25	0	40	13	0.00273506	0.1400212	20.25	16.086818								
7	1E+08	7_135535953	15	5	12	8	10	10	0.938234	1	0	9	22	19	5	21	0.00386645	0.1846363	11.25	7.28501397	1E+08	1.4E+08	ID=ENSP0000182527;TRAM2;NM_012288;	TRAM2	1.4E+08	1.4E+08	0.68381	ID=promoter_49743;
7	1E+08	7_136323277	109	23	58	57	66	58	0.273677	1	52	64	47	42	58	45	0.66857123	1	56.5	24.663449								
8	47897	8_47897	0	13	10	0	7	5	0.693374	1	8	9	2	1	9	2	0.1390433	1	5.375	5.1806646								
8	98721	8_98721	13	38	25	11	26	18	0.220252	1	75	32	64	22	54	43	0.75792271	1	35	23.2624775	96202	111076	ID=ENSP0000305653;LETM1;NM_012318;	LETM1	97878	100319	0.47553	ID=promoter_49771;
8	104447	8_104447	6	9	14	8	8	11	0.943069	1	111	57	39	4	84	22	1.26E-07	2.58E-05	31	37.4089371	96202	111076	ID=ENSP0000305653;LETM1;NM_012318;	LETM1	101188	105110	0.66877	ID=promoter_49772;
8	106484	8_106484	9	12	9	7	11	8	0.5832	1	2	6	14	4	4	9	0.31291743	1	7.875	3.97986001	96202	111076	ID=ENSP0000305653;LETM1;NM_012318;	LETM1	105480	108458	0.58328	ID=promoter_49773;
8	163770	8_163770	26	3	12	0	15	6	0.065303	1	0	11	2	0	6	1	0.20114407	1	6.75	9.17683419					163640	165239	0.35831	ID=promoter_49786;
8	168027	8_168027	127	24	105	114	76	110	0.369051	1	76	50	48	23	63	36	0.06492712	1	70.875	40.7936882					167538	168384	0.40137	ID=promoter_49787;
8	212582	8_212582	15	46	20	23	31	22	0.19035	1	28	24	53	58	26	56	0.00124088	0.0746948	33.375	16.4398253								
8	251695	8_251695	2	39	3	6	21	5	0.001802	0.10311	21	1	16	2	11	9	0.96206785	1	11.25	13.4137456					251236	251954	0.14074	ID=promoter_49809;
8	310269	8_310269	168	24	75	69	96	72	0.01525	0.57129	26	17	9	16	22	13	0.43054584	1	50.5	53.5670474								
8	325067	8_325067	21	26	13	3	24	8	0.004476	0.21437	11	4	10	21	8	16	0.19094317	1	13.625	8.34843869	324513	330560	ID=ENSP0000372335;WHSC2;;	WHSC2	323873	339741	0.9997	ID=promoter_49820;

8	330558	8_330558	38	37	20	16	38	18	0.004632	0.22041	16	0	12	2	8	7	0.9937864	1	17.625	14.080762	324513	330560	ID=ENSP00000372335;WHSC2;;	WHSC2	323873	339741	0.9997	ID=promoter_49820;
8	336986	8_336986	519	363	594	724	441	659	4.16E-04	0.03032	58	33	38	33	46	36	0.74800413	1	295.25	289.765299					323873	339741	0.9997	ID=promoter_49820;
8	346093	8_346093	1	3	1	1	2	1	0.773064	1	17	9	9	45	13	27	0.0491434	1	10.75	14.9451378					344211	346534	0.55257	ID=promoter_49822;
8	347462	8_347462	18	44	29	32	31	31	0.820663	1	2	7	10	22	5	16	0.03625394	1	20.5	14.1421356					347079	350652	0.65494	ID=promoter_49823;
8	354335	8_354335	3	39	13	23	21	18	0.61832	1	15	15	17	1	15	9	0.58227053	1	15.75	11.8532696					351230	356461	0.82318	ID=promoter_49824;
8	418181	8_418181	2	23	0	3	13	2	0.006819	0.29841	1	7	19	6	4	13	0.06462238	1	7.625	8.65097022	407446	534569	ID=ENSP00000372316;POLN;NM_181808;	POLN				
8	521792	8_521792	7	6	6	3	7	5	0.693374	1	0	0	5	22	0	14	5.26E-04	0.0367435	6.125	6.95778084	407446	534569	ID=ENSP00000372316;POLN;NM_181808;	POLN	521264	522626	0.26525	ID=promoter_49861;
8	526587	8_526587	19	34	11	3	27	7	4.86E-04	0.03443	0	31	1	2	16	2	0.00719486	0.3031284	12.625	13.8247966	407446	534569	ID=ENSP00000372316;POLN;NM_181808;	POLN	526431	527097	0.20071	ID=promoter_49862;
8	546811	8_546811	648	211	551	415	430	483	0.807975	1	223	200	164	87	212	126	3.57E-04	0.0267297	312.38	201.35962					546396	548212	0.58897	ID=promoter_49864;
8	552958	8_552958	11	3	13	11	7	12	0.724589	1	38	16	23	24	27	24	0.98883926	1	17.375	10.7562274					552876	554045	0.33138	ID=promoter_49865;
8	561097	8_561097	475	104	449	306	290	378	0.400963	1	203	101	93	48	152	71	7.35E-06	0.0009646	222.38	168.36859					559198	563157	0.7872	ID=promoter_49866;
8	561900	8_561900	1401	450	1684	1105	926	1395	2.47E-08	#######	656	403	496	170	530	333	1.57E-07	3.16E-05	795.63	537.944484					559198	563157	0.7872	ID=promoter_49866;
8	564916	8_564916	348	164	306	401	256	354	0.133062	1	167	144	231	107	156	169	0.37288087	1	233.5	106.699846					564231	565967	0.36209	ID=promoter_49867;
8	580256	8_580256	8	16	8	8	12	8	0.45534	1	13	9	10	9	11	10	0.99899888	1	10.125	2.90012315								
8	596632	8_596632	13	17	6	6	15	6	0.065303	1	31	1	11	32	16	22	0.48233468	1	14.625	11.5007764					596162	597648	0.28172	ID=promoter_49875;
8	605893	8_605893	0	11	2	7	6	5	0.851367	1	2	13	1	4	8	3	0.3843038	1	5	4.84030696								
8	611278	8_611278	716	174	683	591	445	637	0.005443	0.25191	259	199	230	104	229	167	0.06117174	1	369.5	249.816504					610278	611870	0.62175	ID=promoter_49878;
8	669708	8_669708	160	77	216	347	119	282	4.33E-10	#######	40	36	71	52	38	62	0.02094562	0.7090268	124.88	109.86022	619965	702869	ID=ENSP00000381254;;					
8	687594	8_687594	100	38	39	26	69	33	6.76E-05	0.00633	15	16	11	2	16	7	0.23724011	1	30.875	30.7545003	619965	702869	ID=ENSP00000381254;;					
8	698645	8_698645	43	7	2	7	25	5	2.23E-04	0.01779	0	25	2	1	13	2	0.02644099	0.8480056	10.875	15.2824596	619965	702869	ID=ENSP00000381254;;		697019	698719	0.31288	ID=promoter_49901;
8	737945	8_737945	285	125	322	171	205	247	0.960413	1	73	131	145	25	102	85	0.76834646	1	159.63	100.052754					737254	738440	0.36353	ID=promoter_49906;
8	834450	8_834450	101	36	25	27	69	26	1.32E-06	0.00019	47	24	6	2	36	4	8.86E-06	0.0011428	33.5	30.9331076					833789	836911	0.60336	ID=promoter_49919;
8	835874	8_835874	15	18	18	5	17	12	0.395844	1	26	6	13	3	16	8	0.35023049	1	13	7.8921299					833789	836911	0.60336	ID=promoter_49919;
8	836420	8_836420	4	12	0	1	8	1	0.03583	1	1	0	8	27	1	18	2.55E-04	0.0200383	6.625	9.28804916					833789	836911	0.60336	ID=promoter_49919;

8	851553	8_851553	197	77	149	134	137	142	0.586269	1	85	36	67	12	61	40	0.23163204	1	94.625	61.4327449					850771	852333	0.34679	ID=promoter_49923;
8	869625	8_869625	4	3	1	7	4	4	0.97482	1	6	5	20	0	6	10	0.51486293	1	5.75	6.22781778								
8	883147	8_883147	29	50	20	35	40	28	0.10608	1	21	16	19	25	19	22	0.7764064	1	26.875	11.1539551								
8	888976	8_888976	14	12	17	1	13	9	0.46931	1	6	11	19	13	9	16	0.2823118	1	11.625	5.80486249					888436	889591	0.29443	ID=promoter_49933;
8	929088	8_929088	1	15	15	1	8	8	0.950274	1	13	0	25	2	7	14	0.23485029	1	9	9.2736185					928589	929729	0.22418	ID=promoter_49939;
8	944043	8_944043	25	10	7	3	18	5	0.008132	0.34618	1	12	31	8	7	20	0.02608553	0.8424716	12.125	10.5348063					943468	946458	0.47956	ID=promoter_49942;
8	951966	8_951966	37	2	14	2	20	8	0.026297	0.87605	79	76	72	19	78	46	0.04925617	1	37.625	33.4020423								
8	970147	8_970147	33	16	14	14	25	14	0.076795	1	80	21	44	47	51	46	0.99094219	1	33.625	22.9218392					968614	971862	0.49098	ID=promoter_49947;
8	1E+06	8_1068902	25	0	4	3	13	4	0.041893	1	0	13	0	1	7	1	0.13113995	1	5.75	8.90826262					1068064	1071712	0.50453	ID=promoter_49977;
8	1E+06	8_1076922	15	13	29	34	14	32	0.105504	1	50	22	28	4	36	16	0.04368613	1	24.375	14.2521928					1075796	1077793	0.41804	ID=promoter_49980;
8	1E+06	8_1077751	134	74	90	65	104	78	0.010761	0.4351	24	8	21	22	16	22	0.48233468	1	54.75	43.6569418					1075796	1077793	0.41804	ID=promoter_49980;
8	1E+06	8_116191	9	2	8	4	6	6	0.962469	1	1	10	4	4	6	4	0.87665919	1	5.25	3.32737563								
8	1E+06	8_1186063	13	16	9	18	15	14	0.827043	1	3	1	10	5	2	8	0.1303879	1	9.375	6.11642986	1E+06	1282488	ID=ENSP0000347184;HTT;NM_002111;	HTT	1185743	1187035	0.20705	ID=promoter_50008;
8	1E+06	8_1259886	11	16	24	6	14	15	0.970741	1	1	11	7	17	6	12	0.2888538	1	11.625	7.24938421	1E+06	1282488	ID=ENSP0000347184;HTT;NM_002111;	HTT	1258994	1263004	0.6285	ID=promoter_50029;
8	1E+06	8_1273506	28	53	18	40	41	29	0.108664	1	0	0	0	1	0	1	0.58312783	1	17.5	20.9352744	1E+06	1282488	ID=ENSP0000347184;HTT;NM_002111;	HTT	1272974	1278509	0.85843	ID=promoter_50033;
8	1E+06	8_1275366	22	45	65	95	34	80	0.002433	0.13134	66	22	72	59	44	66	0.0443253	1	55.75	25.0699023	1E+06	1282488	ID=ENSP0000347184;HTT;NM_002111;	HTT	1272974	1278509	0.85843	ID=promoter_50033;
8	1E+06	8_1282878	2	8	7	15	5	11	0.495311	1	3	7	20	22	5	21	0.00386645	0.1846363	10.5	7.57816412					1281077	1282933	0.38397	ID=promoter_50034;
8	1E+06	8_1293401	13	52	19	22	33	21	0.084147	1	68	18	49	9	43	29	0.41314796	1	31.25	21.82888					1291057	1294087	0.61124	ID=promoter_50036;
8	1E+06	8_1306648	43	33	17	10	38	14	4.70E-04	0.03357	14	12	11	1	13	6	0.35360803	1	17.625	13.6479983	1E+06	1370923	ID=ENSP0000372238;RGS12;;	RGS12	1302818	1309724	0.947	ID=promoter_50041;
8	1E+06	8_1313764	51	44	74	47	48	61	0.917298	1	45	18	17	4	32	11	0.01265194	0.4751394	37.5	22.7596134	1E+06	1370923	ID=ENSP0000372238;RGS12;;	RGS12	1312488	1314115	0.22004	ID=promoter_50043;
8	1E+06	8_1316322	121	54	52	44	88	48	7.07E-05	0.00657	40	38	6	2	39	4	2.33E-06	0.0003543	44.625	36.5471809	1E+06	1370923	ID=ENSP0000372238;RGS12;;	RGS12	1315640	1318352	0.42983	ID=promoter_50044;
8	1E+06	8_1324539	22	7	6	10	15	8	0.181584	1	1	3	5	3	2	4	0.6610374	1	7.125	6.6211242	1E+06	1370923	ID=ENSP0000372238;RGS12;;	RGS12	1323319	1324754	0.40571	ID=promoter_50045;
8	1E+06	8_1346674	126	104	189	148	115	169	0.174402	1	27	16	21	14	22	18	0.92557456	1	80.625	69.6274525	1E+06	1370923	ID=ENSP0000372238;RGS12;;	RGS12	1345806	1347100	0.26994	ID=promoter_50051;

8	1E+06	8_1349067	35	43	34	26	39	30	0.216196	1	127	10	60	23	69	42	0.09589548	1	44.75	36.3386846	1E+06	1370923	ID=ENSP00000372238;RGS12;;	RGS12				
8	1E+06	8_1398622	23	21	31	55	22	43	0.143846	1	7	6	9	1	7	5	0.9051169	1	19.125	17.7155098					1397523	1398791	0.23333	ID=promoter_50061;
8	1E+06	8_141358	16	10	9	20	13	15	0.999061	1	0	6	5	0	3	3	0.99570314	1	8.25	7.1063352								
8	1E+06	8_142811	242	125	344	389	184	367	2.10E-08	#######	237	224	247	248	231	248	0.27515966	1	257	79.5630927								
8	2E+06	8_152168	17	20	1	2	19	2	2.36E-04	0.01868	20	14	11	34	17	23	0.4932031	1	14.875	10.6695495								
8	2E+06	8_160049	85	23	11	14	54	13	9.96E-08	#######	84	20	21	37	52	29	0.08575664	1	36.875	30.3759186								
8	2E+06	8_161195	50	7	54	62	29	58	0.059693	1	15	6	5	1	11	3	0.13591373	1	25	25.6236498								
8	2E+06	8_162142	45	39	4	29	42	17	5.42E-04	0.03781	8	25	27	12	17	20	0.769441	1	23.625	14.6378911								
8	2E+06	8_1627874	18	27	16	11	23	14	0.143642	1	33	16	15	7	25	11	0.10848128	1	17.875	8.39110924					1626479	1628136	0.25754	ID=promoter_50119;
8	2E+06	8_165890	100	25	84	59	63	72	0.988712	1	35	75	100	144	55	122	2.12E-07	4.14E-05	77.75	38.5217935								
8	2E+06	8_1677986	69	38	70	62	54	66	0.97517	1	40	18	57	1	29	29	0.95922868	1	44.375	24.9853529					1676914	1678220	0.1686	ID=promoter_50133;
8	2E+06	8_1706317	29	15	83	67	22	75	2.40E-05	0.00257	18	40	50	11	29	31	0.858757	1	39.125	26.0298592					1705767	1706802	0.26456	ID=promoter_50140;
8	2E+06	8_1720757	1092	510	1606	1533	801	1570	9.21E-32	#######	523	382	778	244	453	511	0.0096756	0.3824351	833.5	522.136271					1719145	1722621	0.91147	ID=promoter_50142;
8	2E+06	8_174442	37	0	47	32	19	40	0.103616	1	0	0	0	0	0	0	1	1	14.5	20.4240754								
8	2E+06	8_175992	100	54	37	35	77	36	1.47E-05	0.00167	15	38	61	11	27	36	0.35693007	1	43.875	28.3369395								
8	2E+06	8_1777949	18	2	29	34	10	32	0.015448	0.57677	0	15	23	1	8	12	0.56746184	1	15.25	13.1990259					1777851	1779472	0.2537	ID=promoter_50152;
8	2E+06	8_1822505	16	14	15	11	15	13	0.725173	1	5	9	4	175	7	90	1.44E-17	8.82E-15	31.125	58.3032405					1821852	1823461	0.42768	ID=promoter_50157;
8	2E+06	8_182676	9	12	12	10	11	11	0.932271	1	0	5	10	23	3	17	0.00428697	0.2008879	10.125	6.57783073								
8	2E+06	8_1858052	4	0	1	26	2	14	0.026823	0.8911	0	0	9	5	0	7	0.02292696	0.7568963	5.625	8.83074985					1857425	1858411	0.14387	ID=promoter_50161;
8	2E+06	8_1955784	15	49	15	7	32	11	9.30E-04	0.05947	42	36	53	11	39	32	0.87710631	1	28.5	18.4855156					1955714	1956365	0.12929	ID=promoter_50176;
8	2E+06	8_2098914	125	56	125	164	91	145	0.071062	1	40	48	46	0	44	23	0.07826377	1	75.5	55.6776436	2E+06	2099065	ID=ENSP00000321606-D3;CRMP1;NM_001014809;	CRMP1	2098171	2100209	0.75803	ID=promoter_50191;
8	2E+06	8_2120783	7	25	24	35	16	30	0.310604	1	0	2	11	23	1	17	4.35E-04	0.0314643	15.875	12.6088121					2120603	2121346	0.13684	ID=promoter_50192;
8	2E+06	8_2120856	113	74	130	109	94	120	0.827737	1	75	33	40	24	54	32	0.12930039	1	74.75	40.027669					2120603	2121346	0.13684	ID=promoter_50192;
8	2E+06	8_214298	3	0	11	4	2	8	0.255957	1	17	0	2	5	9	4	0.4571786	1	5.25	5.89794153								
8	2E+06	8_2164906	0	18	32	19	9	26	0.054763	1	37	13	18	35	25	27	0.8592775	1	21.5	12.5242622	2E+06	2177315	ID=ENSP00000380985;C4orf50;;	C4orf50				
8	2E+06	8_219393	166	37	135	132	102	134	0.68908	1	78	87	72	22	83	47	0.02422951	0.7942054	91.125	49.9497962								
8	2E+06	8_2194807	98	77	106	97	88	102	0.996667	1	42	126	75	42	84	59	0.26099774	1	82.875	29.9067002	2E+06	2246561	ID=ENSP00000386711;JAKMIP1;NM_001099433;	JAKMIP1	2194558	2195187	0.17591	ID=promoter_50200;
8	2E+06	8_2198204	5	15	12	6	10	9	0.84607	1	20	15	0	3	18	2	0.00297675	0.1507727	9.5	6.98978847	2E+06	2246561	ID=ENSP00000386711;JAKMIP1;NM_001099433;	JAKMIP1	2197383	2198337	0.16933	ID=promoter_50202;
8	2E+06	8_2202122	31	115	43	47	73	45	0.002574	0.13802	101	83	67	21	92	44	0.00115864	0.0709106	63.5	33.8188451	2E+06	2246561	ID=ENSP00000386711;JAKMIP1;NM_001099433;	JAKMIP1				

8	2E+06	8_2210460	404	190	298	227	297	263	0.003898	0.19244	17	29	34	63	23	49	0.00321925	0.1617361	157.75	144.826744	2E+06	2246561	ID=ENSP0000386711;JAKMIP1;NM_001099433;	JAKMIP1	2209621	2211340	0.29218	ID=promoter_50207;
8	2E+06	8_2213554	8	17	3	16	13	10	0.600182	1	31	5	0	1	18	1	9.25E-04	0.05912	10.125	10.5889093	2E+06	2246561	ID=ENSP0000386711;JAKMIP1;NM_001099433;	JAKMIP1				
8	2E+06	8_2215730	0	18	12	0	9	6	0.554309	1	0	0	16	2	0	9	0.00779604	0.3222758	6	7.92824967	2E+06	2246561	ID=ENSP0000386711;JAKMIP1;NM_001099433;	JAKMIP1	2214892	2216079	0.26155	ID=promoter_50209;
8	2E+06	8_2217340	117	52	78	71	85	75	0.196803	1	23	23	38	10	23	24	0.92099721	1	51.5	35.6611033	2E+06	2246561	ID=ENSP0000386711;JAKMIP1;NM_001099433;	JAKMIP1	2216932	2217865	0.15545	ID=promoter_50210;
8	2E+06	8_2237984	17	21	12	11	19	12	0.233425	1	4	3	12	1	4	7	0.58783259	1	10.125	7.01910658	2E+06	2246561	ID=ENSP0000386711;JAKMIP1;NM_001099433;	JAKMIP1				
8	2E+06	8_2241101	9	16	1	8	13	5	0.083134	1	1	21	5	0	11	3	0.13591373	1	7.625	7.59581652	2E+06	2246561	ID=ENSP0000386711;JAKMIP1;NM_001099433;	JAKMIP1	2240856	2242295	0.477	ID=promoter_50215;
8	2E+06	8_229115	48	9	24	13	29	19	0.13502	1	0	1	5	0	1	3	0.56022579	1	12.5	16.5010822								
8	2E+06	8_2352418	483	308	439	457	396	448	0.87168	1	357	212	252	99	285	176	1.11E-04	0.0100183	325.88	134.092119					2351469	2352422	0.19734	ID=promoter_50229;
8	2E+06	8_238533	128	59	108	116	94	112	0.993738	1	55	101	69	82	78	76	0.95341757	1	89.75	27.4004692								
8	2E+06	8_239219	941	528	565	526	735	546	3.61E-15	#######	198	269	237	117	234	177	0.12342412	1	422.63	270.632559								
8	2E+06	8_241230	184	76	145	137	130	141	0.812656	1	4	24	15	4	14	10	0.81923661	1	73.625	72.5985783								
8	2E+06	8_2454605	151	41	85	129	96	107	0.936191	1	80	71	89	43	76	66	0.92737739	1	86.125	38.2078432					2453790	2455397	0.41623	ID=promoter_50243;
8	3E+06	8_252309	30	17	40	16	24	28	0.999813	1	16	41	23	1	29	12	0.05423954	1	23	13.5224681								
8	3E+06	8_253370	104	110	100	97	107	99	0.233674	1	53	1	39	61	27	50	0.01268586	0.4762881	70.625	38.6816217								
8	3E+06	8_2534186	154	83	123	104	119	114	0.302023	1	6	22	35	0	14	18	0.65378371	1	65.875	58.0380109					2534017	2534586	0.12279	ID=promoter_50251;
8	3E+06	8_253934	111	55	109	155	83	132	0.092576	1	57	27	39	10	42	25	0.20942954	1	70.375	49.6126064								
8	3E+06	8_256890	70	49	80	52	60	66	0.937235	1	0	10	0	1	5	1	0.30486099	1	32.75	33.648392								
8	3E+06	8_2705667	216	144	157	137	180	147	0.004694	0.22295	276	131	107	51	204	79	9.20E-11	3.11E-08	152.38	68.088047					2705144	2706113	0.18457	ID=promoter_50277;
8	3E+06	8_2715226	0	0	18	7	0	13	0.00392	0.19308	1	16	0	27	9	14	0.47220951	1	8.625	10.4462912					2714869	2715836	0.23821	ID=promoter_50279;
8	3E+06	8_273861	237	87	122	129	162	126	0.00232	0.12592	53	62	37	28	58	33	0.0775783	1	94.375	68.4688407								
8	3E+06	8_2746023	5	18	7	4	12	6	0.213448	1	1	6	35	1	4	18	0.00668749	0.2868305	9.625	11.5627172					2745534	2746335	0.12167	ID=promoter_50284;
8	3E+06	8_2750872	8	31	20	37	20	29	0.765605	1	22	12	43	21	17	32	0.05485386	1	24.25	11.9970234					2750233	2750887	0.15753	ID=promoter_50286;
8	3E+06	8_2788466	5	16	14	13	11	14	0.979671	1	3	6	7	4	5	6	0.91271846	1	8.5	5.04267503					2788020	2789623	0.27612	ID=promoter_50294;
8	3E+06	8_279665	42	8	54	60	25	57	0.018651	0.66809	0	0	6	1	0	4	0.11562589	1	21.375	25.9831676								
8	3E+06	8_2845928	19	3	22	10	11	16	0.859437	1	5	13	17	11	9	14	0.47220951	1	12.5	6.63324958					2845385	2846330	0.14146	ID=promoter_50310;
8	3E+06	8_285850	2	5	13	5	4	9	0.54411	1	4	2	0	11	3	6	0.53745121	1	5.25	4.52769257								
8	3E+06	8_2862573	0	19	7	10	10	9	0.84607	1	1	10	29	0	6	15	0.10150651	1	9.5	10.2120377					2861850	2862666	0.1884	ID=promoter_50316;
8	3E+06	8_2870696	8	5	10	13	7	12	0.724589	1	23	4	21	9	14	15	0.92682316	1	11.625	7.00892288					2870125	2871147	0.17153	ID=promoter_50319;
8	3E+06	8_2893354	103	36	76	33	70	55	0.081616	1	0	0	0	2	0	1	0.58312783	1	31.25	39.5718154					2893017	2896843	0.52365	ID=promoter_50322;

8	3E+06	8_2894665	66	21	72	37	44	55	0.951721	1	1	9	14	0	5	7	0.77775394	1	27.5	28.2286582					2893017	2896843	0.52365	ID=promoter_50322;
8	3E+06	8_299224	4	5	7	2	5	5	0.968625	1	6	4	0	15	5	8	0.62517663	1	5.375	4.47013902								
8	3E+06	8_300582	0	10	2	4	5	3	0.645778	1	0	1	0	43	1	22	3.00E-05	0.0032574	7.5	14.735768								
8	3E+06	8_301433	3	5	2	11	4	7	0.813458	1	13	21	3	4	17	4	0.02867352	0.9010784	7.75	6.69221296								
8	3E+06	8_302500	19	48	25	63	34	44	0.911425	1	40	45	86	27	43	57	0.20516024	1	44.125	22.1452188								
8	3E+06	8_3048085	9	0	0	4	5	2	0.402696	1	0	15	40	0	8	20	0.04735057	1	8.5	13.8564065					3047552	3048346	0.2029	ID=promoter_50355;
8	3E+06	8_3110684	2	0	0	1	1	1	0.993645	1	4	3	11	24	4	18	0.00668749	0.2868305	5.625	8.22778394	3E+06	3222773	ID=ENSP00000353402;AFAP1;;	AFAP1	3109988	3110897	0.14801	ID=promoter_50374;
8	3E+06	8_3115632	15	54	12	21	35	17	0.008714	0.36691	37	4	49	27	21	38	0.04330269	1	27.375	17.9119872	3E+06	3222773	ID=ENSP00000353402;AFAP1;;	AFAP1	3113894	3115657	0.29894	ID=promoter_50375;
8	3E+06	8_3123086	174	65	135	68	120	102	0.055916	1	15	24	15	29	20	22	0.85756598	1	65.625	59.4785375	3E+06	3222773	ID=ENSP00000353402;AFAP1;;	AFAP1				
8	3E+06	8_3141197	2	0	10	0	1	5	0.353258	1	0	10	38	0	5	19	0.0096847	0.3824351	7.5	13.082158	3E+06	3222773	ID=ENSP00000353402;AFAP1;;	AFAP1	3140931	3141725	0.2029	ID=promoter_50379;
8	3E+06	8_3186792	90	48	43	42	69	43	0.004183	0.20335	0	0	4	10	0	7	0.02292696	0.7568963	29.625	31.8744748	3E+06	3222773	ID=ENSP00000353402;AFAP1;;	AFAP1	3185896	3188083	0.32414	ID=promoter_50389;
8	3E+06	8_3188251	41	9	33	38	25	36	0.732372	1	85	7	15	2	46	9	1.51E-05	0.0018118	28.75	27.2173789	3E+06	3222773	ID=ENSP00000353402;AFAP1;;	AFAP1				
8	3E+06	8_3210039	1	25	3	0	13	2	0.006819	0.29841	15	25	10	5	20	8	0.11762508	1	10.5	10.198039	3E+06	3222773	ID=ENSP00000353402;AFAP1;;	AFAP1	3209363	3214481	0.75867	ID=promoter_50397;
8	3E+06	8_322335	7	12	0	15	10	8	0.719353	1	2	5	3	2	4	3	0.96209564	1	5.75	5.28474895								
8	3E+06	8_330039	66	32	47	75	49	61	0.952982	1	104	60	68	79	82	74	0.98602478	1	66.375	21.5468758								
8	3E+06	8_330653	67	32	42	22	50	32	0.024936	0.84252	26	17	27	7	22	17	0.8526593	1	30	18.1422947								
8	3E+06	8_332548	26	36	17	24	31	21	0.144504	1	0	17	32	2	9	17	0.21129426	1	19.25	13.0356654								
8	3E+06	8_3336065	227	74	106	71	151	89	1.03E-06	0.00015	41	20	60	38	31	49	0.06280227	1	79.625	65.1217267	3E+06	3412541	ID=ENSP00000358266-D3;ABLIM1;;	ABLIM1	3335187	3336528	0.28417	ID=promoter_50413;
8	3E+06	8_3338556	18	32	11	14	25	13	0.050329	1	37	2	8	29	20	19	0.99708906	1	18.875	12.494999	3E+06	3412541	ID=ENSP00000358266-D3;ABLIM1;;	ABLIM1	3337763	3339103	0.33922	ID=promoter_50414;
8	3E+06	8_3351710	60	13	52	65	37	59	0.339357	1	120	32	54	43	76	49	0.13721142	1	54.875	31.1605864	3E+06	3412541	ID=ENSP00000358266-D3;ABLIM1;;	ABLIM1				
8	3E+06	8_343059	23	75	22	39	49	31	0.023879	0.81295	35	27	50	48	31	49	0.06280227	1	39.875	17.7155098								
8	3E+06	8_348027	18	14	4	15	16	10	0.283594	1	1	35	21	1	18	11	0.54801163	1	13.625	11.6120319								
8	3E+06	8_3499821	23	0	15	8	12	12	0.926346	1	0	16	0	0	8	0	0.02452223	0.7977568	7.75	9.20791585					3499280	3499948	0.17843	ID=promoter_50444;
8	4E+06	8_354762	48	39	35	43	44	39	0.441907	1	66	33	48	33	50	41	0.84384887	1	43.125	11.076842								
8	4E+06	8_355215	5	0	7	16	3	12	0.129494	1	0	8	16	0	4	8	0.43697044	1	6.5	6.67618368								
8	4E+06	8_357567	1	1	4	1	1	3	0.700657	1	14	9	22	1	12	12	0.98292303	1	6.625	7.83649885								
8	4E+06	8_3597649	1	30	5	14	16	10	0.283594	1	24	31	36	2	28	19	0.57381461	1	17.875	14.1566491					3596914	3598179	0.35783	ID=promoter_50465;
8	4E+06	8_3608259	8	0	8	1	4	5	0.995512	1	28	13	23	13	21	18	0.97064008	1	11.75	9.82344135					3608179	3608748	0.19001	ID=promoter_50469;
8	4E+06	8_363914	15	11	6	6	13	6	0.146758	1	2	2	4	3	2	4	0.6610374	1	6.125	4.64258241								
8	4E+06	8_364813	38	32	16	23	35	20	0.032214	1	69	2	29	47	36	38	0.85555789	1	32	20.2696113								
8	4E+06	8_368468	63	30	65	68	47	67	0.590402	1	83	80	68	37	82	53	0.1204305	1	61.75	18.8812076								
8	4E+06	8_3693672	6	5	6	5	6	6	0.962469	1	1	12	9	11	7	10	0.67636194	1	6.875	3.60307408					3693233	3694163	0.22963	ID=promoter_50492;

8	4E+06	8_369585	54	20	55	41	37	48	0.89929	1	0	24	13	18	12	16	0.63262749	1	28.125	19.8669683								
8	4E+06	8_3740489	1912	1167	2130	2592	1540	2361	2.52E-15	#######	1159	843	899	614	1001	757	1.27E-04	0.0112073	1414.5	707.67648					3739806	3741452	0.35537	ID=promoter_50499;
8	4E+06	8_3749319	113	160	138	161	137	150	0.843766	1	54	78	114	24	66	69	0.78348043	1	105.25	49.6926266					3748499	3749385	0.21326	ID=promoter_50501;
8	4E+06	8_3756700	689	201	527	473	445	500	0.80443	1	252	160	182	87	206	135	0.00704823	0.2985552	321.38	213.849705					3755745	3758340	0.51403	ID=promoter_50504;
8	4E+06	8_384632	14	15	9	22	15	16	0.965652	1	0	5	20	27	3	24	1.24E-04	0.0109901	14	9.03959544								
8	4E+06	8_392292	17	3	3	11	10	7	0.570701	1	0	12	0	0	6	0	0.06196836	1	5.75	6.62786326								
8	4E+06	8_393293	20	9	5	11	15	8	0.181584	1	0	9	17	23	5	20	0.00613894	0.2677261	11.75	7.75978645								
8	4E+06	8_3946081	0	7	12	30	4	21	0.010729	0.43446	0	0	1	0	0	1	0.58312783	1	6.25	10.5661184					3945532	3946162	0.15413	ID=promoter_50517;
8	4E+06	8_412076	2	58	12	23	30	18	0.072527	1	0	12	36	8	6	22	0.00549069	0.2478586	18.875	19.6209618								
8	4E+06	8_4307434	440	95	447	284	268	366	0.166369	1	45	27	38	5	36	22	0.30031675	1	172.63	188.572788					4307320	4308285	0.18002	ID=promoter_50538;
8	4E+06	8_443089	19	6	12	16	13	14	0.975695	1	19	0	19	1	10	10	0.98574881	1	11.5	8.1240384								
8	5E+06	8_4521470	412	260	164	175	336	170	4.21E-19	#######	190	194	100	142	192	121	0.00370878	0.1804258	204.63	95.4641855	5E+06	4531876	ID=ENSP00000345917;LYAR;NM_017816;	LYAR				
8	5E+06	8_485641	33	42	56	28	38	42	0.965359	1	53	10	20	24	32	22	0.55606214	1	33.25	16.095696								
8	5E+06	8_4867321	28	47	16	18	38	17	0.002761	0.14629	13	19	48	2	16	25	0.25669436	1	23.875	16.2607108					4866939	4867776	0.10756	ID=promoter_50571;
8	5E+06	8_502731	33	71	25	20	52	23	2.73E-04	0.02129	60	38	38	60	49	49	0.93208319	1	43.125	18.3804049								
8	5E+06	8_508870	126	72	116	103	99	110	0.926397	1	159	102	240	24	131	132	0.79567163	1	117.75	63.3194847								
8	5E+06	8_533605	5	15	10	3	10	7	0.570701	1	9	0	16	1	5	9	0.48000906	1	7.375	6.11642986								
8	6E+06	8_570790	33	10	19	2	22	11	0.058934	1	1	13	22	21	7	22	0.01139713	0.4370832	15.125	10.8158283								
8	6E+06	8_594549	0	68	8	1	34	5	1.68E-06	0.00024	1	9	26	1	5	14	0.08295177	1	14.25	23.3712277								
8	6E+06	8_611182	165	47	116	110	106	113	0.778799	1	170	40	49	2	105	26	7.94E-10	2.36E-07	87.375	61.8983902								
8	6E+06	8_616755	10	8	0	39	9	20	0.269594	1	0	0	5	0	0	3	0.19828566	1	7.75	13.253032								
8	6E+06	8_633731	70	63	52	83	67	68	0.70189	1	68	58	62	33	63	48	0.59100593	1	61.125	14.6036933								
8	7E+06	8_660241	43	6	28	23	25	26	0.911793	1	23	1	13	21	12	17	0.52622129	1	19.75	13.1881982								
8	7E+06	8_666888	11	0	5	9	6	7	0.999953	1	16	0	11	0	8	6	0.92562801	1	6.5	6.164414								
8	7E+06	8_668391	72	125	95	64	99	80	0.044719	1	40	73	63	43	57	53	0.99987682	1	71.875	27.642811								
8	7E+06	8_673078	9	0	9	5	5	7	0.955252	1	10	9	15	0	10	8	0.95278883	1	7.125	5.16685868								
8	7E+06	8_700282	18	5	6	15	12	11	0.839207	1	4	0	14	1	2	8	0.1303879	1	7.875	6.83347851								
8	7E+06	8_714869	19	54	29	39	37	34	0.588357	1	2	13	67	20	8	44	9.36E-07	0.0001567	30.375	21.7776917								
8	7E+06	8_734348	12	10	11	0	11	6	0.3028	1	20	1	7	0	11	4	0.25131486	1	7.625	7.06980501								
8	8E+06	8_7559589	82	40	64	73	61	69	0.978901	1	66	0	43	0	33	22	0.4848075	1	46	31.6814502	7E+06	7564054	ID=ENSP00000371838;CPEB2;;	CPEB2				
8	8E+06	8_7944892	11	20	36	37	16	37	0.068995	1	23	11	14	12	17	13	0.87203467	1	20.5	10.7835854					7944756	7945270	0.12192	ID=promoter_50582;
8	8E+06	8_8115097	9	24	3	13	17	8	0.087954	1	64	1	29	24	33	27	0.89046553	1	20.875	20.2233069	8E+06	8131721	ID=ENSP00000374303;;NM_020785;					
8	8E+06	8_844227	2	0	6	6	1	6	0.241978	1	24	10	10	8	17	9	0.38755874	1	8.25	7.28501397								
8	8E+06	8_8499450	3	20	28	7	12	18	0.803442	1	62	38	31	4	50	18	0.00170227	0.0956535	24.125	20.1808785	8E+06	8571249	ID=ENSP00000265014;;NM_001145852;					
8	9E+06	8_8548082	3	52	22	18	28	20	0.231138	1	39	39	56	0	39	28	0.58548315	1	28.625	21.1925695	8E+06	8571249	ID=ENSP00000265014;;NM_001145852;					
8	9E+06	8_8563538	17	29	23	6	23	15	0.200003	1	21	27	22	14	24	18	0.79306625	1	19.875	7.41499446	8E+06	8571249	ID=ENSP00000265014;;NM_001145852;					
8	9E+06	8_8861824	661	400	742	658	531	700	0.127234	1	315	245	416	74	280	245	0.80400117	1	438.88	232.176311	9E+06	8896334	ID=ENSP00000306921;LDB2;;	LDB2				
8	9E+06	8_893104	0	0	0	1	0	1	0.652937	1	53	8	21	24	31	23	0.7197219	1	13.375	18.7459519								

8	9E+06	8_9048581	10	35	15	5	23	10	0.024151	0.81985	15	21	24	1	18	13	0.78939737	1	15.75	10.9119855					9048113	9048934	0.09799	ID=promoter_50622;
8	9E+06	8_905337	0	13	12	2	7	7	0.956352	1	9	3	15	3	6	9	0.65381141	1	7.125	5.79254447								
8	9E+06	8_907924	90	49	35	54	70	45	0.006073	0.27451	105	48	45	31	77	38	0.0053726	0.2435789	57.125	26.324554								
8	9E+06	8_912131	0	9	5	5	5	5	0.968625	1	1	0	2	29	1	16	7.41E-04	0.0492332	6.375	9.65012953								
8	9E+06	8_933393	36	21	18	9	29	14	0.018844	0.67198	10	7	30	2	9	16	0.2823118	1	16.625	11.8555292								
8	9E+06	8_944034	0	6	22	27	3	25	8.89E-04	0.05749	18	5	41	3	12	22	0.15316566	1	15.25	14.2803561								
8	1E+07	8_957637	39	22	18	14	31	16	0.023279	0.79554	25	37	40	27	31	34	0.79475598	1	27.75	9.91031209								
8	1E+07	8_967709	29	52	39	78	41	59	0.60213	1	36	71	53	79	54	66	0.3191975	1	54.625	19.515104								
8	1E+07	8_9702803	2	6	2	7	4	5	0.995512	1	39	0	14	1	20	8	0.11762508	1	8.875	12.9883189					9702133	9703965	0.46639	ID=promoter_50628;
8	1E+07	8_9703468	172	101	161	35	137	98	8.01E-04	0.05261	123	72	29	63	98	46	5.34E-04	0.0372477	94.5	54.2639054					9702133	9703965	0.46639	ID=promoter_50628;
8	1E+07	8_977466	26	86	24	44	56	34	0.008607	0.36317	94	47	67	32	71	50	0.32772295	1	52.5	26.9708838								
8	1E+07	8_124865	9	11	0	7	10	4	0.162164	1	1	10	9	5	6	7	0.91781435	1	6.5	4.14039336								
8	1E+07	8_130080	10	12	25	20	11	23	0.278506	1	64	19	9	5	42	7	1.33E-05	0.0016203	20.5	18.7844921								
8	1E+07	8_134421	15	7	7	8	11	8	0.5832	1	38	16	18	4	27	11	0.06118138	1	14.125	10.8947891								
8	1E+07	8_147474	35	54	16	15	45	16	7.83E-05	0.00718	2	44	25	24	23	25	0.85898626	1	26.875	16.839049								
8	1E+07	8_148088	390	187	419	243	289	331	0.957123	1	207	99	124	91	153	108	0.09384713	1	220	125.803702								
8	1E+07	8_148789	8	8	11	16	8	14	0.661714	1	11	4	18	3	8	11	0.69450319	1	9.875	5.2762947								
8	1E+07	8_149372	20	23	19	11	22	15	0.260529	1	0	0	8	0	0	4	0.11562589	1	10.125	9.67230952								
8	2E+07	8_15008344	0	0	1	4	0	3	0.278365	1	12	0	24	21	6	23	0.00349909	0.1735359	7.75	9.98212688					1.5E+07	1.5E+07	0.1917	ID=promoter_50654;
8	2E+07	8_150715	1	3	0	8	2	4	0.823343	1	64	40	5	1	52	3	1.98E-09	5.47E-07	15.25	23.7110583								
8	2E+07	8_150937	36	11	5	2	24	4	1.39E-04	0.01187	50	24	15	0	37	8	2.51E-04	0.0199657	17.875	17.6913013								
8	2E+07	8_15282916	59	42	69	40	51	55	0.910781	1	32	24	18	10	28	14	0.15944813	1	36.75	20.090865					1.5E+07	1.5E+07	0.75334	ID=promoter_50661;
8	2E+07	8_15344159	9	14	10	1	12	6	0.213448	1	8	6	1	2	7	2	0.29886173	1	6.375	4.74906006	2E+07	1.5E+07	ID=ENSP0000336741-D3;DHX15;NM_001358;	DHX15	1.5E+07	1.5E+07	0.82809	ID=promoter_50662;
8	2E+07	8_154017	0	20	5	0	10	3	0.082185	1	1	0	12	21	1	17	4.35E-04	0.0314643	7.375	9.07016302								
8	2E+07	8_154305	40	52	30	31	46	31	0.054078	1	88	26	91	55	57	73	0.18526955	1	51.625	25.5507199								
8	2E+07	8_155753	18	47	29	24	33	27	0.37765	1	193	119	228	130	156	179	0.14910265	1	98.5	81.6140744								
8	2E+07	8_15643665	20	27	21	4	24	13	0.071032	1	25	25	44	2	25	23	0.99965163	1	21	13.3523674					1.6E+07	1.6E+07	0.23843	ID=promoter_50668;
8	2E+07	8_157714	22	10	42	17	16	30	0.310604	1	1	0	15	15	1	15	0.00126073	0.075413	15.25	13.2422484								
8	2E+07	8_157866	23	43	39	11	33	25	0.251341	1	40	13	54	18	27	36	0.35693007	1	30.125	15.8964282								
8	2E+07	8_157981	33	30	53	77	32	65	0.036332	1	61	6	14	35	34	25	0.67804059	1	38.625	23.8143864								
8	2E+07	8_163303	2	13	2	3	8	3	0.206144	1	0	16	10	1	8	6	0.92562801	1	5.875	6.17454452								
8	2E+07	8_164801	32	53	5	29	43	17	3.55E-04	0.02675	20	12	24	1	16	13	0.9394435	1	22	16.6818978								
8	2E+07	8_166064	3	35	13	3	19	8	0.039867	1	21	34	35	19	28	27	0.98939345	1	20.375	13.4900757								
8	2E+07	8_166771	24	61	8	19	43	14	4.87E-05	0.00473	154	36	49	22	95	36	1.42E-05	0.0017226	46.625	46.6412984								
8	2E+07	8_168117	87	49	98	80	68	89	0.793131	1	23	15	12	0	19	6	0.05448017	1	45.5	38.377821								
8	2E+07	8_169052	84	57	115	92	71	104	0.351416	1	43	8	70	15	26	43	0.06134398	1	60.5	37.3210014								
8	2E+07	8_169256	1	19	2	5	10	4	0.162164	1	9	1	17	1	5	9	0.48000906	1	6.875	7.41499446								
8	2E+07	8_184423	28	16	39	28	22	34	0.599769	1	16	27	30	57	22	44	0.01053141	0.4092826	30.125	13.1956432								
8	2E+07	8_185588	12	10	7	18	11	13	0.999831	1	30	18	35	6	24	21	0.98147478	1	17	10.6234243								
8	2E+07	8_188704	4	11	11	18	8	15	0.557319	1	38	40	36	57	39	47	0.47823362	1	26.875	18.4888963								
8	2E+07	8_189755	80	11	64	62	46	63	0.726232	1	16	8	20	39	12	30	0.01030357	0.4032822	37.5	27.9080122								
8	2E+07	8_190669	1	5	1	13	3	7	0.596562	1	24	20	17	0	22	9	0.10444527	1	10.125	9.56836305								
8	2E+07	8_198140	3	29	0	22	16	11	0.385778	1	13	0	13	0	7	7	0.99000271	1	10	11.1611571								
8	2E+07	8_206959	7	6	5	5	7	5	0.693374	1	65	1	19	15	33	17	0.1362937	1	15.375	20.8938644								
8	2E+07	8_222876	2	6	9	10	4	10	0.424038	1	6	31	23	1	19	12	0.57792516	1	11	10.5559733								
8	2E+07	8_23190771	23	125	32	39	74	36	4.54E-05	0.00448	28	76	81	32	52	57	0.68205932	1	54.5	36.0198358	2E+07	2.3E+07	ID=ENSP0000302895;ARAP2;NM_015230;	ARAP2				
8	2E+07	8_24150277	660	272	549	437	466	493	0.278911	1	281	193	192	88	237	140	1.13E-04	0.0101411	334	196.513904					2.4E+07	2.4E+07	0.6703	ID=promoter_50720;
8	2E+07	8_246323	35	97	70	65	66	68	0.754287	1	106	100	129	54	103	92	0.96695608	1	82	31.0759438								
8	2E+07	8_246679	0	5	19	11	3	15	0.044084	1	1	23	15	3	12	9	0.89054267	1	9.625	8.70036945								
8	2E+07	8_248559	20	59	16	18	40	17	0.001238	0.07553	0	0	11	39	0	25	1.39E-06	0.0002238	20.375	19.9351628								
8	3E+07	8_250156	5	1	8	10	3	9	0.343967	1	27	0	20	13	14	17	0.75475782	1	10.5	9.30437685								
8	3E+07	8_25051591	30	4	25	12	17	19	0.989587	1	3	40	46	1	22	24	0.85866092	1	20.125	17.6103663					2.5E+07	2.5E+07	0.1868	ID=promoter_50740;

8	3E+07	8_251181	0	25	14	18	13	16	0.991731	1	25	37	17	53	31	35	0.72585335	1	23.625	15.9099026								
8	3E+07	8_251859	471	145	346	264	308	305	0.110603	1	39	36	34	48	38	41	0.79653724	1	172.88	168.80626								
8	3E+07	8_253022	10	22	2	5	16	4	0.009581	0.39335	0	0	1	40	0	21	1.21E-05	0.0014894	10	14.2126704								
8	3E+07	8_255958	0	13	14	5	7	10	0.922856	1	32	27	1	0	30	1	3.70E-06	0.0005267	11.5	12.4556356								
8	3E+07	8_256967	47	7	3	19	27	11	0.00829	0.35136	9	15	4	37	12	21	0.20312388	1	17.625	16.1858624								
8	3E+07	8_259422	1749	1136	1311	968	1443	1140	1.31E-22	#######	494	712	744	160	603	452	0.0028687	0.1464956	909.25	495.908042								
8	3E+07	8_263572	3	28	6	22	16	14	0.724026	1	26	21	19	22	24	21	0.98147478	1	18.375	9.05439909								
8	3E+07	8_265288	10	10	11	7	10	9	0.84607	1	9	6	15	6	8	11	0.69450319	1	9.25	3.01188123								
8	3E+07	8_268790	177	74	79	29	126	54	8.96E-10	#######	52	16	2	2	34	2	2.14E-06	0.0003293	53.875	58.1068904								
8	3E+07	8_269979	766	54	112	95	410	104	6.81E-52	#######	164	36	103	33	100	68	0.14095235	1	170.38	244.630881								
8	3E+07	8_270629	10	23	3	5	17	4	0.005755	0.26328	15	0	7	2	8	5	0.78486764	1	8.125	7.67998326								
8	3E+07	8_270967	5	29	8	2	17	5	0.013234	0.50759	28	38	12	1	33	7	5.50E-04	0.0381958	15.375	14.2220905								
8	3E+07	8_27124509	0	14	3	1	7	2	0.160905	1	15	19	1	2	17	2	0.0046333	0.2138882	6.875	7.73558567					2.7E+07	2.7E+07	0.14033	ID=promoter_50772;
8	3E+07	8_271344	38	11	43	85	25	64	0.003154	0.16233	23	8	35	24	16	30	0.06738755	1	33.375	24.2660109								
8	3E+07	8_271582	116	49	82	67	83	75	0.25968	1	32	23	18	23	28	21	0.76300557	1	51.25	34.7717044								
8	3E+07	8_272769	10	25	11	13	18	12	0.307258	1	21	3	0	1	12	1	0.01422424	0.5202299	10.5	9.13392421								
8	3E+07	8_277767	8	0	11	26	4	19	0.022499	0.77278	15	7	14	5	11	10	0.99899888	1	10.75	7.85129653								
8	3E+07	8_278618	493	193	341	308	343	325	0.02203	0.75833	249	155	162	95	202	129	0.00396094	0.1883926	249.5	128.026783								
8	3E+07	8_278884	8	15	0	2	12	1	0.003675	0.18279	37	19	3	2	28	3	1.04E-04	0.0094884	10.75	12.6010204								
8	3E+07	8_279146	13	1	5	18	7	12	0.724589	1	12	23	8	8	18	8	0.20901227	1	11	7.09124208								
8	3E+07	8_28078210	4083	2636	4618	4500	3360	4559	1.19E-09	#######	2630	2026	1923	900	2328	1412	1.35E-35	1.74E-32	2914.5	1350.67877					2.8E+07	2.8E+07	0.74509	ID=promoter_50794;
8	3E+07	8_28609901	1	6	3	4	4	4	0.97482	1	8	23	17	2	16	10	0.61601721	1	8	7.8921299	3E+07	2.9E+07	ID=ENSP0000382670;GRXCR1;NM_001080476;	GRXCR1				
8	3E+07	8_290708	28	111	23	3	70	13	2.23E-11	#######	22	56	59	36	39	48	0.41901633	1	42.25	33.2941436								
8	3E+07	8_299420	0	11	15	0	6	8	0.9722	1	29	11	7	1	20	4	0.00863789	0.3495868	9.25	9.8088881								
8	3E+07	8_30600623	100	99	55	73	100	64	6.22E-04	0.0426	73	98	70	52	86	61	0.27621808	1	77.5	19.427521					3.1E+07	3.1E+07	0.3176	ID=promoter_50801;
8	3E+07	8_323946	34	15	17	26	25	22	0.614783	1	0	2	0	0	1	0	0.62906424	1	11.75	13.3389869								
8	3E+07	8_32414851	54	112	32	46	83	39	6.80E-06	0.00085	86	18	41	69	52	55	0.79243147	1	57.25	30.5695927	3E+07	3.2E+07	ID=ENSP0000333113;NFXL1;;	NFXL1				
8	3E+07	8_32435477	187	228	119	137	208	128	3.88E-08	#######	79	86	122	108	83	115	0.01935309	0.6621829	133.25	50.7423464	3E+07	3.2E+07	ID=ENSP0000333113;NFXL1;;	NFXL1	3.2E+07	3.2E+07	0.26855	ID=promoter_50809;
8	3E+07	8_32600998	54	442	65	132	248	99	2.26E-20	#######	158	203	229	122	181	176	0.90329084	1	175.63	123.446851	3E+07	3.3E+07	ID=ENSP0000350224-D3;BMX;;	BMX				
8	3E+07	8_33603159	27	1	44	41	14	43	0.005077	0.23773	62	6	51	0	34	26	0.76044447	1	29	24.2015348	3E+07	3.4E+07	ID=ENSP0000334625;DCUN1D4;NM_001040402;	DCUN1D4				
8	3E+07	8_336258	4	36	0	4	20	2	1.33E-04	0.01141	0	3	10	0	2	5	0.46647814	1	7.125	12.1353969								
8	3E+07	8_33676515	70	185	127	137	128	132	0.583106	1	147	138	200	68	143	134	0.99601107	1	134	47.2863617	3E+07	3.4E+07	ID=ENSP0000341944;LRRC66;NM_001024611;	LRRC66				
8	3E+07	8_33681992	38	2	19	7	20	13	0.243396	1	28	0	18	0	14	9	0.68990751	1	14	14.1320305	3E+07	3.4E+07	ID=ENSP0000341944;LRRC66;NM_001024611;	LRRC66				
8	3E+07	8_340512	0	11	2	1	6	2	0.258649	1	20	22	9	7	21	8	0.08664698	1	9	8.38365757								
8	3E+07	8_341201	116	99	114	112	108	113	0.697037	1	61	39	18	3	50	11	1.59E-05	0.001895	70.25	46.123282								
8	3E+07	8_34737602	698	403	965	645	551	805	3.64E-04	0.02737	174	134	113	92	154	103	0.03620954	1	403	331.027837	3E+07	3.5E+07	ID=ENSP0000370787;;					
8	3E+07	8_348903	11	129	33	52	70	43	0.003122	0.16094	46	31	86	53	39	70	0.00345432	0.172273	55.125	36.8914915								
8	4E+07	8_35142096	294	109	310	230	202	270	0.37259	1	108	114	156	50	111	103	0.99997573	1	171.38	95.5120598					3.5E+07	3.5E+07	0.55864	ID=promoter_50843;
8	4E+07	8_351585	22	74	30	34	48	32	0.042987	1	12	7	14	0	10	7	0.84912739	1	24.125	23.1420304								
8	4E+07	8_351605	8	0	6	1	4	4	0.97482	1	39	7	6	4	23	5	0.00590668	0.2600594	8.875	12.494999								

8	4E+07	8_35194808	3285	2984	4375	5883	3135	5129	6.08E-48	#######	2885	2451	4979	1447	2668	3213	1.62E-22	1.27E-19	3536.1	1445.54641					3.5E+07	3.5E+07	0.55864	ID=promoter_50848;
8	4E+07	8_352945	9	45	11	8	27	10	0.004472	0.21437	30	16	27	20	23	24	0.92099721	1	20.75	12.7138844								
8	4E+07	8_356802	106	21	121	94	64	108	0.068042	1	25	8	15	23	17	19	0.85444859	1	51.625	46.7147499								
8	4E+07	8_358284	47	16	77	43	32	60	0.096475	1	0	33	18	0	17	9	0.38755874	1	29.25	26.2174969								
8	4E+07	8_359054	14	30	25	11	22	18	0.522468	1	1	59	28	25	30	27	0.99379101	1	24.125	17.2248782								
8	4E+07	8_359425	67	134	37	18	101	28	1.80E-12	#######	104	20	106	30	62	68	0.63163707	1	64.5	45.0523505								
8	4E+07	8_364351	131	310	139	93	221	116	5.39E-12	#######	221	369	585	432	295	509	6.91E-17	3.95E-14	285	171.248358								
8	4E+07	8_36750286	78	119	86	49	99	68	0.002709	0.14407	59	54	38	93	57	66	0.46802313	1	72	26.8860027					3.7E+07	3.7E+07	0.19729	ID=promoter_50860;
8	4E+07	8_37143252	435	157	409	420	296	415	0.063517	1	231	199	192	45	215	119	2.99E-05	0.0032574	261	143.645397					3.7E+07	3.7E+07	0.59737	ID=promoter_50861;
8	4E+07	8_372787	119	57	49	56	88	53	4.88E-04	0.03443	119	84	74	89	102	82	0.62983253	1	80.875	27.3466504								
8	4E+07	8_376909	6	8	3	12	7	8	0.998739	1	13	21	8	0	17	4	0.02867352	0.9010784	8.875	6.5123509								
8	4E+07	8_397512	1	13	7	13	7	10	0.922856	1	1	12	14	1	7	8	0.92121674	1	7.75	5.97016391								
8	4E+07	8_399774	149	170	94	160	160	127	0.004222	0.20495	171	180	190	58	176	124	0.06348572	1	146.5	46.2045143								
8	4E+07	8_410104	13	61	6	14	37	10	4.04E-05	0.00403	28	26	42	7	27	25	0.99999055	1	24.625	19.0633343								
8	4E+07	8_412057	0	16	0	2	8	1	0.03583	1	60	24	7	10	42	9	7.66E-05	0.0073207	14.875	20.0530546								
8	4E+07	8_41217957	4	84	7	5	44	6	1.98E-08	#######	23	9	7	10	16	9	0.48000706	1	18.625	27.0710045	4E+07	4.1E+07	ID=ENSP0000303552;ETFDH;NM_004453;	ETFDH				
8	4E+07	8_41926952	275	140	142	229	208	186	0.026348	0.87745	153	202	123	62	178	93	3.29E-05	0.0035427	165.75	66.7955302	4E+07	4.2E+07	ID=ENSP0000264431;RAPGEF2;NM_014247;	RAPGEF2				
8	4E+07	8_419381	6	36	2	7	21	5	0.001802	0.10311	0	7	5	0	4	3	0.96209564	1	7.875	11.7283235								
8	4E+07	8_420811	11	12	12	16	12	14	0.999907	1	0	14	15	2	7	9	0.80856893	1	10.25	5.97016391								
8	4E+07	8_421755	36	22	24	12	29	18	0.097648	1	1	17	25	18	9	22	0.0389747	1	19.375	10.2530483								
8	4E+07	8_42199787	437	187	318	317	312	318	0.210882	1	152	139	115	59	146	87	0.00446715	0.2090568	215.5	128.263122					4.2E+07	4.2E+07	0.30857	ID=promoter_50895;
8	4E+07	8_449397	3	49	12	20	26	16	0.118485	1	6	17	23	2	12	13	0.92692342	1	16.5	15.2783694								
8	4E+07	8_449482	72	36	55	59	54	57	0.856981	1	46	34	21	20	40	21	0.10160257	1	42.875	18.4888963								
8	5E+07	8_454344	14	25	13	10	20	12	0.173914	1	9	0	31	19	5	25	5.78E-04	0.0398304	15.125	9.731209								
8	5E+07	8_455398	288	124	281	255	206	268	0.535031	1	87	95	100	30	91	65	0.27362816	1	157.5	100.959681								
8	5E+07	8_462780	31	281	72	64	156	68	1.18E-11	#######	124	132	146	57	128	102	0.52152822	1	113.38	79.0441784								
8	5E+07	8_464776	30	0	8	30	15	19	0.975531	1	42	27	28	52	35	40	0.66345137	1	27.125	16.7283976								
8	5E+07	8_466704	54	65	49	36	60	43	0.044963	1	32	38	45	25	35	35	0.95100306	1	43	12.8951818								
8	5E+07	8_470768	74	46	79	60	60	70	0.999533	1	37	13	14	2	25	8	0.02318551	0.7636665	40.625	29.19852								
8	5E+07	8_471018	1	6	6	4	4	5	0.995512	1	17	16	7	9	17	8	0.27279187	1	8.25	5.59974489								
8	5E+07	8_472312	18	2	10	4	10	7	0.570701	1	48	9	14	0	29	7	0.00267552	0.137714	13.125	15.3384438								
8	5E+07	8_474386	8	2	7	23	5	15	0.168861	1	26	14	10	1	20	6	0.03829208	1	11.375	9.13294664								
8	5E+07	8_47617981	22	51	6	7	37	7	2.66E-06	0.00037	39	7	25	4	23	15	0.56706676	1	20.125	17.4882614					4.8E+07	4.8E+07	0.2685	ID=promoter_50927;
8	5E+07	8_47618653	490	258	286	303	374	295	1.94E-06	0.00028	136	84	68	63	110	66	0.01894881	0.6519669	211	150.354818					4.8E+07	4.8E+07	0.2685	ID=promoter_50927;
8	5E+07	8_476326	223	35	139	160	129	150	0.997377	1	60	27	41	45	44	43	0.9701733	1	91.25	72.9946182								
8	5E+07	8_47735114	192	61	164	153	127	159	0.860633	1	79	49	61	5	64	33	0.02117326	0.7157147	95.5	65.8092482					4.8E+07	4.8E+07	0.69068	ID=promoter_50929;
8	5E+07	8_477664	7	65	46	8	36	27	0.208316	1	35	36	62	70	36	66	0.00359322	0.1775262	41.125	24.4624348								
8	5E+07	8_478403	60	31	31	58	46	45	0.686795	1	7	3	5	0	5	3	0.8350419	1	24.375	24.5411462								
8	5E+07	8_479431	35	16	27	12	26	20	0.360218	1	2	12	3	1	7	2	0.29886173	1	13.5	12.270755								
8	5E+07	8_48046343	653	303	765	614	478	690	0.002233	0.12265	281	190	310	107	236	209	0.89049028	1	402.88	240.411218					4.8E+07	4.8E+07	1	ID=promoter_50935;
8	5E+07	8_481008	168	73	155	101	121	128	0.717436	1	0	3	0	25	2	13	0.01148244	0.4380166	65.625	69.652069								
8	5E+07	8_481274	1	8	9	10	5	10	0.615109	1	2	1	11	12	2	12	0.01895406	0.6519669	6.75	4.65218843								
8	5E+07	8_48146936	323	78	283	226	201	255	0.707575	1	62	78	142	38	70	90	0.11944569	1	153.75	109.54549					4.8E+07	4.8E+07	0.59972	ID=promoter_50937;
8	5E+07	8_482989	16	41	4	7	29	6	7.05E-05	0.00656	5	3	23	1	4	12	0.09850366	1	12.5	13.7113092								
8	5E+07	8_484473	1	14	6	1	8	4	0.357158	1	12	14	8	10	13	9	0.79589064	1	8.25	5.25764749								
8	5E+07	8_492982	0	15	7	7	8	7	0.850897	1	0	0	6	22	0	14	5.26E-04	0.0367435	7.125	7.90004521								
8	5E+07	8_511197	22	43	4	13	33	9	1.29E-04	0.01118	21	17	17	6	19	12	0.57792516	1	17.875	12.0527116								
8	5E+07	8_522975	17	53	2	12	35	7	7.74E-06	0.00096	12	8	58	3	10	31	0.00200647	0.1098505	20.625	22.1161706								
8	5E+07	8_536432	17	39	4	8	28	6	1.19E-04	0.01043	3	17	32	47	10	40	3.77E-05	0.0039639	20.875	16.6256042								
8	5E+07	8_543087	176	235	194	196	206	195	0.099137	1	244	134	133	124	189	129	0.02673137	0.8546242	179.5	46.3650114								

8	5E+07	8_54789058	173	150	175	153	162	164	0.412635	1	215	109	153	96	162	125	0.30372945	1	153	37.6487526					5.5E+07	5.5E+07	0.67704	ID=promoter_50956;
8	6E+07	8_554291	0	3	7	8	2	8	0.255957	1	41	15	35	1	28	18	0.47597237	1	13.75	15.7729425								
8	6E+07	8_565026	2	12	17	8	7	13	0.61331	1	38	19	45	31	29	38	0.36985231	1	21.5	15.090205								
8	6E+07	8_566259	22	26	9	30	24	20	0.525373	1	75	55	67	20	65	44	0.27039156	1	38	24.2840336								
8	6E+07	8_56712735	70	75	137	91	73	114	0.159571	1	58	23	42	14	41	28	0.4570143	1	63.75	39.4886964	6E+07	5.7E+07	ID=ENSP0000379484;TMPRSS11F;;	TMPRSS11F				
8	6E+07	8_57469297	856	441	768	735	649	752	0.974318	1	336	265	300	146	301	223	0.03945903	1	480.88	267.735711					5.7E+07	5.7E+07	0.41256	ID=promoter_50963;
8	6E+07	8_579578	76	60	66	100	68	83	0.970815	1	27	2	12	44	15	28	0.0827714	1	48.375	33.4319672								
8	6E+07	8_593262	224	299	180	217	262	199	1.94E-05	0.00214	231	396	392	301	314	347	0.07804503	1	280	81.3352849								
8	6E+07	8_593711	1	22	5	0	12	3	0.030625	0.98374	2	15	39	1	9	20	0.07989336	1	10.625	13.9277472								
8	6E+07	8_596524	16	27	34	26	22	30	0.865661	1	81	20	29	2	51	16	3.80E-04	0.0280836	29.375	23.0523473								
8	6E+07	8_601558	12	14	13	5	13	9	0.46931	1	0	0	4	1	0	3	0.19828566	1	6.125	5.98659216								
8	6E+07	8_602552	387	226	337	439	307	388	0.620307	1	204	121	165	104	163	135	0.62524147	1	247.88	125.297859								
8	6E+07	8_602730	103	35	42	35	69	39	9.68E-04	0.06148	7	3	18	4	5	11	0.25523295	1	30.875	32.9390671								
8	6E+07	8_605841	14	35	11	16	25	14	0.076795	1	25	25	42	39	25	41	0.07455929	1	25.875	11.8132795								
8	6E+07	8_60636288	1752	967	1836	1091	1360	1464	0.074347	1	571	575	380	240	573	310	1.05E-13	4.90E-11	926.5	604.414475					6.1E+07	6.1E+07	0.12841	ID=promoter_50983;
8	6E+07	8_607278	1	61	13	9	31	11	0.001462	0.08667	18	12	51	2	15	27	0.11059826	1	20.875	22.5479647								
8	6E+07	8_609749	20	27	11	10	24	11	0.027804	0.91326	25	0	11	34	13	23	0.16598565	1	17.25	11.1579568								
8	6E+07	8_615705	14	25	2	13	20	8	0.026297	0.87605	39	19	1	0	29	1	5.87E-06	0.000796	14.125	13.5270892								
8	6E+07	8_617959	0	0	0	15	0	8	0.033035	1	26	12	28	49	19	39	0.01421979	0.5202299	16.25	17.3925271								
8	6E+07	8_619764	121	114	188	186	118	187	0.036885	1	55	8	40	17	32	29	0.99609288	1	91.125	71.8379874								
8	6E+07	8_621201	29	40	2	10	35	6	2.83E-06	0.00039	33	20	36	25	27	31	0.7179117	1	24.375	13.1032984								
8	6E+07	8_632848	165	57	106	121	111	114	0.608306	1	71	40	61	21	56	41	0.51562687	1	80.25	47.2584082								
8	6E+07	8_633337	0	3	12	10	2	11	0.085797	1	18	4	20	2	11	11	0.98433491	1	8.625	7.57698583								
8	6E+07	8_634864	19	28	11	3	24	7	0.00213	0.11798	4	17	7	6	11	7	0.73613127	1	11.875	8.75764972								
8	6E+07	8_637506	6	21	2	6	14	4	0.02589	0.86655	2	10	36	27	6	32	4.92E-05	0.0050322	13.75	12.7251158								
8	6E+07	8_638250	29	20	26	24	25	25	0.852664	1	3	19	28	34	11	31	0.00385975	0.1846363	22.875	9.38749168								
8	6E+07	8_644919	136	45	91	101	91	96	0.768833	1	84	75	62	40	80	51	0.10980414	1	79.25	31.3585258								
8	6E+07	8_645747	55	25	80	67	40	74	0.064412	1	79	78	46	26	79	36	0.00149905	0.0875056	57	22.8785614								
8	6E+07	8_646775	10	2	15	15	6	15	0.276126	1	28	3	18	5	16	12	0.85678722	1	12	8.81557064								
8	6E+07	8_647880	0	16	1	0	8	1	0.03583	1	9	10	11	0	10	6	0.69729498	1	5.875	6.35694221								
8	6E+07	8_648356	444	290	381	336	367	359	0.049812	1	144	106	137	28	125	83	0.06060465	1	233.25	149.079796								
8	6E+07	8_649627	9	39	13	4	24	9	0.00876	0.36763	0	30	29	3	15	16	0.92652531	1	15.875	14.7400087								
8	7E+07	8_650123	169	59	135	145	114	140	0.935891	1	79	18	52	28	49	40	0.83663943	1	85.625	56.9358975								
8	7E+07	8_654691	73	35	35	63	54	49	0.426995	1	0	44	44	1	22	23	0.92188352	1	36.875	26.0298592								
8	7E+07	8_65568606	2267	841	2390	3585	1554	2988	4.56E-56	#######	962	871	1763	925	917	1344	5.35E-26	4.96E-23	1700.5	994.640782					6.6E+07	6.6E+07	0.67166	ID=promoter_51064;
8	7E+07	8_656451	11	46	6	22	29	14	0.018844	0.67198	102	33	49	25	68	37	0.03072133	0.9498655	36.75	30.4055916								
8	7E+07	8_660069	25	35	29	19	30	24	0.372243	1	1	27	19	1	14	10	0.81923661	1	19.5	12.5470543								
8	7E+07	8_663322	417	53	396	433	235	415	2.94E-06	0.0004	227	36	150	64	132	107	0.58678422	1	222	171.610855								
8	7E+07	8_66356941	12	6	9	17	9	13	0.89076	1	15	1	8	40	8	24	0.00970297	0.3824351	13.5	11.844227	7E+07	6.6E+07	ID=ENSP0000263985;PET112L;NM_004564;	PET112L				
8	7E+07	8_66386595	38	57	41	32	48	37	0.154373	1	25	35	25	27	30	26	0.96889533	1	35	10.7038044	7E+07	6.6E+07	ID=ENSP0000263985;PET112L;NM_004564;	PET112L				
8	7E+07	8_66428184	28	10	91	100	19	96	1.79E-09	#######	21	27	160	13	24	87	1.42E-09	4.05E-07	56.25	54.4996723					6.6E+07	6.6E+07	0.24088	ID=promoter_51076;
8	7E+07	8_674220	1	25	1	24	13	13	0.920459	1	14	16	42	3	15	23	0.30916076	1	15.75	14.3601632								
8	7E+07	8_705374	19	51	14	14	35	14	0.001717	0.09971	13	40	32	37	27	35	0.42118875	1	27.5	14.4716472								
8	7E+07	8_708070	25	23	20	29	24	25	0.917255	1	47	28	17	4	38	11	0.0015633	0.0891068	24.125	12.1706614								
8	7E+07	8_709713	3	59	5	1	31	3	8.71E-07	0.00013	20	5	41	13	13	27	0.0491434	1	18.375	21.0097766								
8	7E+07	8_709952	0	15	0	5	8	3	0.206144	1	30	25	0	1	28	1	9.31E-06	0.0011901	9.5	12.270755								
8	7E+07	8_711966	5	4	7	17	5	12	0.389128	1	18	21	1	5	20	3	0.00346999	0.1723921	9.75	7.64852927								
8	7E+07	8_71355034	8	0	7	7	4	7	0.813458	1	19	1	15	16	10	16	0.39084582	1	9.125	6.95778084	7E+07	7.1E+07	ID=ENSP0000368761;ZNF827;NM_178835;	ZNF827				
8	7E+07	8_71887896	513	128	320	282	321	301	0.020072	0.70957	147	145	77	26	146	52	6.05E-09	1.56E-06	204.75	158.265374					7.2E+07	7.2E+07	0.76605	ID=promoter_51125;
8	7E+07	8_720390	0	37	1	6	19	4	0.002035	0.11403	1	2	5	0	2	3	0.86792893	1	6.5	12.5242622								

8	7E+07	8_720936	0	10	8	9	5	9	0.74134	1	1	18	19	4	10	12	0.83305499	1	8.625	7.08998287								
8	7E+07	8_725068	316	122	242	202	219	222	0.308578	1	109	91	92	138	100	115	0.2873623	1	164	81.60007								
8	7E+07	8_736711	0	23	0	2	12	1	0.003675	0.18279	0	19	5	1	10	3	0.19568363	1	6.25	9.31588505								
8	7E+07	8_73775176	10	75	11	15	43	13	2.33E-05	0.00251	35	26	51	4	31	28	0.99502871	1	28.375	24.3424233	7E+07	7.4E+07	ID=ENSP00000323505;IL15;NM_172174;	IL15				
8	7E+07	8_745002	12	4	18	10	8	14	0.661714	1	17	3	13	3	10	8	0.95278883	1	10	6.094494								
8	7E+07	8_747656	32	0	2	9	16	6	0.042495	1	10	11	0	3	11	2	0.06154634	1	8.375	10.5415843								
8	7E+07	8_747674	9	22	0	13	16	7	0.076817	1	19	11	24	1	15	13	0.98432481	1	12.375	9.00694177								
8	7E+07	8_74931494	189	103	257	189	146	223	0.045339	1	67	102	85	3	85	44	0.00632665	0.2740706	124.38	81.480826					7.5E+07	7.5E+07	0.79561	ID=promoter_51150;
8	7E+07	8_749317	288	142	297	239	215	268	0.801928	1	125	90	67	27	108	47	5.44E-05	0.0054393	159.38	102.94095								
8	8E+07	8_751562	2	9	5	18	6	12	0.558139	1	12	45	25	26	29	26	0.99236142	1	17.75	14.058551								
8	8E+07	8_751956	9	35	5	25	22	15	0.260529	1	17	48	28	6	33	17	0.1362937	1	21.625	15.2497073								
8	8E+07	8_777308	1	21	8	3	11	6	0.3028	1	2	16	40	22	9	31	9.86E-04	0.0617473	14.125	13.4317056								
8	8E+07	8_811628	30	43	8	43	37	26	0.128943	1	12	21	38	14	17	26	0.26839536	1	26.125	14.2772296								
8	8E+07	8_82449686	419	306	294	219	363	257	3.12E-09	#######	155	192	175	2	174	89	2.21E-05	0.0025244	220.25	123.653606					8.2E+07	8.2E+07	0.42522	ID=promoter_51176;
8	8E+07	8_82618600	1177	610	876	827	894	852	7.96E-05	0.00728	425	281	358	226	353	292	0.35387144	1	597.5	336.263077					8.3E+07	8.3E+07	0.42522	ID=promoter_51177;
8	9E+07	8_855680	1	19	3	12	10	8	0.719353	1	27	5	12	4	16	8	0.35023049	1	10.375	9.00694177								
8	9E+07	8_857262	12	47	33	11	30	22	0.240296	1	91	13	30	60	52	45	0.94444197	1	37.125	27.99713								
8	9E+07	8_865560	55	131	53	61	93	57	4.49E-04	0.03238	103	155	210	97	129	154	0.10468836	1	108.13	55.2020639								
8	9E+07	8_86568961	307	120	283	204	214	244	0.955267	1	102	113	66	19	108	43	8.68E-06	0.0011226	151.75	102.830999					8.7E+07	8.7E+07	0.73704	ID=promoter_51201;
8	9E+07	8_870242	16	16	10	2	16	6	0.042495	1	43	11	1	3	27	2	5.20E-05	0.0052176	12.75	13.604096								
8	9E+07	8_87704141	16	0	4	2	8	3	0.206144	1	18	0	1	1	9	1	0.05455957	1	5.25	7.38241153	9E+07	8.8E+07	ID=ENSP00000368966;TRPC3;NM_001130698;	TRPC3				
8	9E+07	8_877414	19	46	13	11	33	12	0.001163	0.07164	13	78	22	5	46	14	6.26E-04	0.0425022	25.875	24.4156712								
8	9E+07	8_883112	0	47	6	0	24	3	4.60E-05	0.00451	43	12	13	0	28	7	0.00393447	0.1875084	15.125	19.1865839								
8	9E+07	8_885107	1	25	3	0	13	2	0.006819	0.29841	7	12	9	2	10	6	0.69729498	1	7.375	8.26243651								
8	9E+07	8_894423	10	10	12	19	10	16	0.741563	1	23	23	14	35	23	25	0.85898626	1	18.25	8.61477137								
8	9E+07	8_903649	62	57	40	48	60	44	0.057742	1	58	49	48	17	54	33	0.16457707	1	47.375	14.1415043								
8	9E+07	8_912506	57	51	62	39	54	51	0.538939	1	0	7	1	0	4	1	0.45332778	1	27.125	27.7202221								
8	9E+07	8_922807	1	22	12	24	12	18	0.803442	1	9	67	59	22	38	41	0.79653724	1	27	23.6280705								
8	9E+07	8_926430	16	24	0	5	20	3	4.27E-04	0.03093	14	17	13	5	16	9	0.48000706	1	11.75	7.85129653								
8	9E+07	8_926804	9	30	1	7	20	4	0.0012	0.07347	8	8	23	26	8	25	0.00638831	0.2759882	14	10.6636901								
8	9E+07	8_928135	1	30	14	3	16	9	0.196587	1	87	26	51	2	57	27	0.0137363	0.5068396	26.75	29.836459								
8	9E+07	8_930355	1	23	0	19	12	10	0.724827	1	55	11	28	2	33	15	0.0650987	1	17.375	18.5544565								
8	9E+07	8_937891	51	5	30	27	28	29	0.895448	1	69	7	24	33	38	29	0.73182863	1	30.75	21.2653306								
8	1E+08	8_954929	14	1	9	26	8	18	0.296056	1	0	42	22	21	21	22	0.92273004	1	16.875	13.9636006								
8	1E+08	8_956733	9	49	1	9	29	5	2.61E-05	0.00275	34	25	26	10	30	18	0.33903897	1	20.375	16.0351177								
8	1E+08	8_958944	4	5	8	6	5	7	0.955252	1	15	21	24	0	18	12	0.67374005	1	10.375	8.634441								
8	1E+08	8_964618	48	31	30	45	40	38	0.647488	1	87	18	64	4	53	34	0.24176884	1	40.875	26.2647918								
8	1E+08	8_969067	23	25	21	17	24	19	0.435991	1	18	10	17	0	14	9	0.68990751	1	16.375	8.03452372								
8	1E+08	8_969156	132	61	52	70	97	61	5.45E-04	0.038	117	26	116	78	72	97	0.05388558	1	81.5	36.8704295								
8	1E+08	8_975023	84	23	92	38	54	65	0.990172	1	27	21	31	28	24	30	0.54878546	1	43	28.3246485								
8	1E+08	8_975745	22	7	28	8	15	18	0.997876	1	19	14	6	0	17	3	0.01239331	0.4669004	13	9.42640668								
8	1E+08	8_977821	19	14	21	11	17	16	0.818283	1	6	11	9	4	9	7	0.94097808	1	11.875	5.91457039								
8	1E+08	8_980425	165	29	124	42	97	83	0.106125	1	52	12	42	0	32	21	0.46573568	1	58.25	56.9128406								
8	1E+08	8_986499	13	20	7	38	17	23	0.905043	1	0	5	10	14	3	12	0.04708211	1	13.375	11.6611381								
8	1E+08	8_992815	9	32	4	11	21	8	0.017144	0.62492	0	25	20	1	13	11	0.97545578	1	12.75	11.7564572								
8	1E+08	8_100500578	14	42	9	21	28	15	0.042594	1	41	41	31	43	41	37	0.9929878	1	30.25	13.8021737								
8	1E+08	8_100916439	66	132	38	51	99	45	3.14E-07	#######	52	47	47	32	50	40	0.78520541	1	58.125	31.4934801	1E+08	1E+08	ID=ENSP00000303325;TACR3;NM_001059;	TACR3				
8	1E+08	8_101012408	6	17	1	7	12	4	0.066899	1	17	27	1	2	22	2	4.98E-04	0.0353761	9.75	9.54313515								
8	1E+08	8_101056637	731	267	323	268	499	296	3.23E-20	#######	174	118	78	15	146	47	3.36E-10	1.05E-07	246.75	222.077046	1E+08	1E+08	ID=ENSP00000378265;NHEDC2;NM_178833;	NHEDC2	1E+08	1E+08	0.33702	ID=promoter_51292;

8	1E+08	8_101400869	130	66	75	65	98	70	0.005937	0.27004	71	36	16	10	54	13	1.58E-05	0.0018878	58.625	38.3664195					1E+08	1E+08	0.60517	ID=promoter_51296;
8	1E+08	8_101521481	2	40	6	13	21	10	0.052655	1	0	7	1	9	4	5	0.90483023	1	9.75	12.980755	1E+08	1E+08	ID=ENSP0000226578-D2;MANBA;NM_005908;	MANBA				
8	1E+08	8_101644467	0	1	2	0	1	1	0.993645	1	38	9	1	1	24	1	5.88E-05	0.0058344	6.5	13.0602997								
8	1E+08	8_102366253	109	50	82	38	80	60	0.030623	0.98374	6	13	43	2	10	23	0.04592065	1	42.875	37.6086521								
8	1E+08	8_102415915	3	72	14	5	38	10	2.46E-05	0.00262	13	36	53	56	25	55	0.00108314	0.0670948	31.5	26.4196896								
8	1E+08	8_102416704	47	71	43	55	59	49	0.199646	1	10	28	32	20	19	26	0.43045343	1	38.25	19.7393733								
8	1E+08	8_102490146	4	32	16	18	18	17	0.813792	1	72	3	23	13	38	18	0.05735612	1	22.625	22.0903179								
8	1E+08	8_102999554	301	91	80	13	196	47	3.27E-26	#######	117	74	42	26	96	34	3.55E-06	0.0005089	93	90.9191163								
8	1E+08	8_103467714	27	26	44	10	27	27	0.841861	1	145	19	14	1	82	8	9.10E-13	3.81E-10	35.75	45.980586								
8	1E+08	8_103468771	174	35	117	43	105	80	0.013674	0.52317	0	8	16	0	4	8	0.43697044	1	49.125	63.3052413								
8	1E+08	8_103866829	26	34	35	17	30	26	0.525876	1	10	3	20	0	7	10	0.67636194	1	18.125	13.2388767								
8	1E+08	8_104018967	10	2	19	18	6	19	0.087167	1	14	2	19	31	8	25	0.00638831	0.2759882	14.375	9.69443876	1E+08	1E+08	ID=ENSP0000296411;METAP1;NM_015143;	METAP1	1E+08	1E+08	0.43654	ID=promoter_51314;
8	1E+08	8_104343560	23	0	19	14	12	17	0.88186	1	62	22	0	0	42	0	3.51E-09	9.37E-07	17.5	20.5773801								
8	1E+08	8_104352027	134	53	110	90	94	100	0.793582	1	0	13	13	1	7	7	0.99000271	1	51.75	53.2909802								
8	1E+08	8_104368075	43	31	19	14	37	17	0.004081	0.19947	9	21	29	25	15	27	0.11059826	1	23.875	10.6561518								
8	1E+08	8_104385639	18	10	10	14	14	12	0.725787	1	7	10	8	2	9	5	0.6500913	1	9.875	4.73399559								
8	1E+08	8_104454229	5	10	4	0	8	2	0.097885	1	0	1	11	20	1	16	7.41E-04	0.0492332	6.375	6.94750726								
8	1E+08	8_104734131	0	7	5	13	4	9	0.54411	1	1	5	6	3	3	5	0.7175395	1	5	4.03555625								
8	1E+08	8_105075232	23	20	27	54	22	41	0.209664	1	140	53	86	19	97	53	0.00743129	0.3120589	52.75	42.2230472								
8	1E+08	8_106114444	12	12	10	15	12	13	0.980472	1	2	19	30	23	11	27	0.01818526	0.631944	15.375	8.5846624								
8	1E+08	8_106207219	48	6	23	19	27	21	0.36385	1	9	25	5	19	17	12	0.76881979	1	19.25	13.9258239								
8	1E+08	8_106377718	242	51	135	129	147	132	0.082355	1	87	54	104	44	71	74	0.77967049	1	105.75	65.2506158								
8	1E+08	8_106509371	0	6	0	26	3	13	0.09118	1	22	17	25	16	20	21	0.92353071	1	14	10.6770783								
8	1E+08	8_106551368	1	8	13	5	5	9	0.74134	1	1	10	7	1	6	4	0.87665919	1	5.75	4.55913526								
8	1E+08	8_107112634	37	52	32	32	45	32	0.091522	1	100	80	54	14	90	34	2.42E-05	0.0027058	50.125	28.114244								
8	1E+08	8_107113882	21	11	26	18	16	22	0.889379	1	0	15	0	0	8	0	0.02452223	0.7977568	11.375	10.363914								
8	1E+08	8_107172903	17	20	36	7	19	22	0.999149	1	19	31	25	3	25	14	0.3120942	1	19.75	11.1451463	1E+08	1.1E+08	ID=ENSP0000321746;PDLIM5;NM_006457;	PDLIM5				
8	1E+08	8_107346316	0	5	4	0	3	2	0.821455	1	26	28	24	2	27	13	0.14207	1	11.125	12.4835606								
8	1E+08	8_107412504	20	45	15	9	33	12	0.001163	0.07164	40	41	52	10	41	31	0.69213115	1	29	17.27095								
8	1E+08	8_107863130	3655	2085	4341	2847	2870	3594	0.032818	1	2370	1613	1679	1288	1992	1484	1.37E-09	3.92E-07	2484.8	1066.41814					1.1E+08	1.1E+08	0.15387	ID=promoter_51336;
8	1E+08	8_108166513	18	33	18	15	26	17	0.164334	1	56	25	9	9	41	9	1.14E-04	0.0101988	22.875	15.5787172								
8	1E+08	8_108598585	1	47	4	9	24	7	0.00213	0.11798	15	9	19	31	12	25	0.0604166	1	16.875	15.3849416								

8	1E+08	8_110060591	21	14	15	6	18	11	0.22238	1	61	9	16	23	35	20	0.21889545	1	20.625	17.2455999								
8	1E+08	8_110138770	35	50	40	27	43	34	0.224092	1	42	22	40	2	32	21	0.46573568	1	32.25	15.0499169								
8	1E+08	8_110256335	1	33	26	19	17	23	0.905043	1	0	55	26	3	28	15	0.22181264	1	20.375	18.9581306								
8	1E+08	8_111031589	1	13	21	10	7	16	0.324814	1	43	16	19	23	30	21	0.61223556	1	18.25	12.197775								
8	1E+08	8_111099813	31	47	10	6	39	8	2.44E-06	0.00034	28	47	30	27	38	29	0.73182863	1	28.25	14.8492424								
8	1E+08	8_111559619	92	13	21	12	53	17	4.03E-06	0.00053	15	13	13	0	14	7	0.39930956	1	22.375	28.7249891								
8	1E+08	8_111673403	45	24	14	28	35	21	0.046836	1	12	40	21	15	26	18	0.63344191	1	24.875	12.1941145								
8	1E+08	8_111861854	4	8	12	5	6	9	0.896349	1	4	0	5	23	2	14	0.00692302	0.294895	7.625	7.11010348					1.1E+08	1.1E+08	0.18768	ID=promoter_51348;
8	1E+08	8_112076628	6	31	11	4	19	8	0.039867	1	35	9	6	3	22	5	0.00878788	0.3541514	13.125	12.5747763	1E+08	1.1E+08	ID=ENSP0000378624-D2;HERC4;NM_022079;	HERC4	1.1E+08	1.1E+08	0.15984	ID=promoter_51366;
8	1E+08	8_112081259	38	44	54	43	41	49	0.996095	1	2	23	10	19	13	15	0.84581615	1	29.125	18.3337121	1E+08	1.1E+08	ID=ENSP0000378624-D2;HERC4;NM_022079;	HERC4				
8	1E+08	8_112233605	78	40	75	52	59	64	0.910405	1	125	46	116	64	86	90	0.7236218	1	74.5	31.4051861								
8	1E+08	8_112812514	3	58	5	9	31	7	6.31E-05	0.00596	37	50	39	2	44	21	0.03878541	1	25.375	23.0585435								
8	1E+08	8_112956111	109	222	142	115	166	129	0.001951	0.11038	126	153	347	60	140	204	1.78E-04	0.0149638	159.25	88.6111086	1E+08	1.1E+08	ID=ENSP0000303575;NUDT9;NM_024047;	NUDT9				
8	1E+08	8_113237636	28	42	7	2	35	5	9.64E-07	0.00015	12	19	14	0	16	7	0.23724011	1	15.5	14.040757								
8	1E+08	8_113268531	7	28	4	11	18	8	0.059652	1	14	8	2	13	11	8	0.87190251	1	10.875	8.07885246					1.1E+08	1.1E+08	0.29172	ID=promoter_51437;
8	1E+08	8_113327639	35	59	55	39	47	47	0.741076	1	61	51	18	86	56	52	0.99996345	1	50.5	20.3259159								
8	1E+08	8_113378345	42	35	84	63	39	74	0.049214	1	51	23	23	28	37	26	0.55440389	1	43.625	21.4871224								
8	1E+08	8_113781717	8	8	0	3	8	2	0.097885	1	0	0	2	24	0	13	9.01E-04	0.0577931	5.625	8.14050718								
8	1E+08	8_113786063	5	56	6	6	31	6	2.44E-05	0.00261	0	20	23	11	10	17	0.30316389	1	15.875	17.9955352								
8	1E+08	8_113905304	67	50	72	108	59	90	0.290187	1	20	54	74	34	37	54	0.10025585	1	59.875	27.0049599	1E+08	1.1E+08	ID=ENSP0000352157;MAPK10;NM_138982;	MAPK10				
8	1E+08	8_113918340	2	12	11	7	7	9	0.983538	1	9	32	36	55	21	46	0.00345893	0.1723921	20.5	18.4468348	1E+08	1.1E+08	ID=ENSP0000352157;MAPK10;NM_138982;	MAPK10				
8	1E+08	8_113918704	20	7	15	4	14	10	0.480768	1	50	48	5	1	49	3	7.80E-09	1.96E-06	18.75	19.6668684	1E+08	1.1E+08	ID=ENSP0000352157;MAPK10;NM_138982;	MAPK10				
8	1E+08	8_114329296	4	11	15	8	8	12	0.864244	1	82	0	13	16	41	15	0.00610396	0.2666968	18.625	26.1858059								
8	1E+08	8_114337875	940	526	2315	2686	733	2501	########	#######	884	581	1402	680	733	1041	5.59E-18	3.49E-15	1251.8	823.415665								
8	1E+08	8_114632842	17	1	22	7	9	15	0.704209	1	0	0	17	0	0	9	0.00779604	0.3222758	8	9.258201								
8	1E+08	8_114683699	21	11	0	2	16	1	3.65E-04	0.02739	23	8	13	0	16	7	0.23724011	1	9.75	9.00396738								
8	1E+08	8_114696040	42	60	51	35	51	43	0.277188	1	6	22	18	25	14	22	0.29543416	1	32.375	18.0232984								

8	1E+08	8_114767705	6	48	3	19	27	11	0.00829	0.35136	5	20	2	0	13	1	0.00904697	0.3617342	12.875	16.0929221								
8	1E+08	8_114783979	2586	2194	3452	3033	2390	3243	4.44E-07	#######	2053	1896	2371	882	1975	1627	0.00197734	0.1086302	2308.4	774.306498					1.1E+08	1.1E+08	0.25888	ID=promoter_51473;
8	1E+08	8_115063724	38	0	39	40	19	40	0.103616	1	0	8	13	6	4	10	0.21760186	1	18	17.896528					1.2E+08	1.2E+08	0.18916	ID=promoter_51490;
8	1E+08	8_115070740	12	170	36	21	91	29	4.75E-10	#######	65	183	91	59	124	75	0.01321528	0.4933113	79.625	64.9569913								
8	1E+08	8_115075737	34	25	10	7	30	9	5.55E-04	0.03862	40	38	12	1	39	7	4.70E-05	0.0048349	20.875	15.273109								
8	1E+08	8_115125109	8	43	1	19	26	10	0.006911	0.3015	69	54	48	19	62	34	0.04520648	1	32.625	24.207068					1.2E+08	1.2E+08	0.35397	ID=promoter_51498;
8	1E+08	8_115138143	361	133	299	345	247	322	0.45811	1	135	81	122	91	108	107	0.8880583	1	195.88	117.989633								
8	1E+08	8_115410797	294	119	267	153	207	210	0.332916	1	105	60	75	48	83	62	0.43572071	1	140.13	93.1733216					1.2E+08	1.2E+08	0.10095	ID=promoter_51520;
8	1E+08	8_115412225	2	24	13	5	13	9	0.46931	1	15	8	35	3	12	19	0.33990144	1	13.125	11.4572186					1.2E+08	1.2E+08	0.4811	ID=promoter_51521;
8	1E+08	8_115417538	38	27	36	6999	33	3518	0	0	24	27	8335	1577	26	4956	0	0	2132.9	3475.56127	1E+08	1.2E+08	ID=ENSP00000295886;NKX6-1;NM_006168;	NKX6-1	1.2E+08	1.2E+08	0.83322	ID=promoter_51522;
8	1E+08	8_115437180	2	12	3	10	7	7	0.956352	1	23	11	21	20	17	21	0.67722313	1	12.75	7.99553447					1.2E+08	1.2E+08	0.14195	ID=promoter_51523;
8	1E+08	8_115462536	9	87	27	24	48	26	0.005046	0.23667	43	40	47	19	42	33	0.7761727	1	37	23.9702196					1.2E+08	1.2E+08	0.15863	ID=promoter_51526;
8	1E+08	8_115464983	194	146	206	111	170	159	0.120638	1	148	90	106	77	119	92	0.42412141	1	134.75	47.2342188					1.2E+08	1.2E+08	0.1879	ID=promoter_51527;
8	1E+08	8_115481638	119	116	167	170	118	169	0.249082	1	1	0	12	0	1	6	0.13767373	1	73.125	77.2499422								
8	1E+08	8_115498090	6	11	17	16	9	17	0.506269	1	6	36	9	11	21	10	0.21021716	1	14	9.76875486								
8	1E+08	8_115519885	12	6	6	4	9	5	0.390586	1	32	48	22	3	40	13	0.00273506	0.1400212	16.625	16.2211282								
8	1E+08	8_115575233	12	1	10	7	7	9	0.983538	1	69	21	6	14	45	10	5.20E-05	0.0052176	17.5	21.6399102								
8	1E+08	8_115582770	0	0	6	3	0	5	0.118674	1	7	9	9	18	8	14	0.34554715	1	6.5	5.87974732								
8	1E+08	8_115839769	10	9	9	16	10	13	0.970528	1	24	4	11	11	14	11	0.91900834	1	11.75	5.94618725								
8	1E+08	8_115860129	103	60	82	87	82	85	0.726748	1	226	55	39	54	141	47	1.86E-09	5.15E-07	88.25	59.4444519								
8	1E+08	8_116119763	46	11	46	40	29	43	0.62009	1	26	40	72	6	33	39	0.58889164	1	35.875	21.2228819					1.2E+08	1.2E+08	0.67825	ID=promoter_51558;
8	1E+08	8_116166821	0	14	11	7	7	9	0.983538	1	0	9	4	1	5	3	0.8350419	1	5.75	5.33853913								
8	1E+08	8_116245725	189	74	201	119	132	160	0.96173	1	33	46	49	14	40	32	0.82411274	1	90.625	71.5720167	1E+08	1.2E+08	ID=ENSP00000310873;;NM_015697;		1.2E+08	1.2E+08	0.63184	ID=promoter_51564;
8	1E+08	8_116310509	13	0	1	6	7	4	0.503048	1	0	33	5	0	17	3	0.01239331	0.4669004	7.25	11.335784								
8	1E+08	8_116314181	43	9	5	0	26	3	1.49E-05	0.00168	0	0	5	14	0	10	0.00454609	0.2107253	9.5	14.4122963								
8	1E+08	8_116434631	7	23	24	0	15	12	0.610117	1	43	18	54	4	31	29	0.99938482	1	21.625	18.9656645								
8	1E+08	8_116477261	0	28	2	10	14	6	0.098789	1	0	32	0	10	16	5	0.08348313	1	10.25	12.9366589	1E+08	1.2E+08	ID=ENSP00000341947;LIN54;NM_194282;	LIN54				
8	1E+08	8_116527094	137	65	73	67	101	70	0.002862	0.14985	108	84	33	44	96	39	4.60E-05	0.0047457	76.375	33.5769372	1E+08	1.2E+08	ID=ENSP00000341947;LIN54;NM_194282;	LIN54	1.2E+08	1.2E+08	0.42625	ID=promoter_51585;

8	1E+08	8_116589278	1	39	22	20	20	21	0.939065	1	113	83	38	1	98	20	1.09E-10	3.63E-08	39.625	39.5472141	1E+08	1.2E+08	ID=ENSP00000378721;SEC31A;NM_001077207;	SEC31A				
8	1E+08	8_116617277	111	34	3	3	73	3	2.34E-17	#######	33	69	12	21	51	17	6.95E-04	0.0467204	35.75	37.2357355	1E+08	1.2E+08	ID=ENSP00000378721;SEC31A;NM_001077207;	SEC31A				
8	1E+08	8_116632560	67	23	15	29	45	22	0.002408	0.1301	46	38	20	2	42	11	3.56E-04	0.0266828	30	20.1707001	1E+08	1.2E+08	ID=ENSP00000378721;SEC31A;NM_001077207;	SEC31A	1.2E+08	1.2E+08	0.1676	ID=promoter_51593;
8	1E+08	8_116648419	516	269	504	547	393	526	0.140647	1	331	149	169	101	240	135	1.65E-05	0.00196	323.25	179.857682					1.2E+08	1.2E+08	0.62327	ID=promoter_51596;
8	1E+08	8_116801962	3	0	6	16	2	11	0.085797	1	25	1	0	23	13	12	0.99994263	1	9.25	10.4982992					1.2E+08	1.2E+08	0.23019	ID=promoter_51626;
8	1E+08	8_116819215	111	154	105	148	133	127	0.251803	1	158	144	95	116	151	106	0.08859478	1	128.88	24.718053					1.2E+08	1.2E+08	0.18425	ID=promoter_51628;
8	1E+08	8_116854501	38	33	24	31	36	28	0.259755	1	27	51	25	11	39	18	0.04421467	1	30	11.625096	1E+08	1.2E+08	ID=ENSP00000273920;ENOPH1;NM_021204;	ENOPH1	1.2E+08	1.2E+08	0.20435	ID=promoter_51636;
8	1E+08	8_116854779	3	18	14	9	11	12	0.985018	1	1	0	0	0	1	0	0.62906424	1	5.625	7.15017482	1E+08	1.2E+08	ID=ENSP00000273920;ENOPH1;NM_021204;	ENOPH1	1.2E+08	1.2E+08	0.20435	ID=promoter_51636;
8	1E+08	8_116884434	64	42	48	29	53	39	0.083184	1	86	44	53	30	65	42	0.18631934	1	49.5	18.6853648					1.2E+08	1.2E+08	0.1412	ID=promoter_51640;
8	1E+08	8_116924497	7	21	0	11	14	6	0.098789	1	43	1	9	1	22	5	0.00878788	0.3541514	11.625	14.4512728					1.2E+08	1.2E+08	0.38334	ID=promoter_51642;
8	1E+08	8_117021062	53	89	45	23	71	34	5.23E-05	0.00504	103	43	96	125	73	111	0.00407734	0.1932847	72.125	35.7908109								
8	1E+08	8_117102677	17	16	26	24	17	25	0.771176	1	19	52	34	1	36	18	0.09436184	1	23.625	14.8990652					1.2E+08	1.2E+08	0.19553	ID=promoter_51661;
8	1E+08	8_117189884	5	25	19	12	15	16	0.965652	1	21	7	8	8	14	8	0.54462139	1	13.125	7.51070664					1.2E+08	1.2E+08	0.13255	ID=promoter_51674;
8	1E+08	8_117235298	85	145	154	143	115	149	0.725073	1	180	183	160	107	182	134	0.12774182	1	144.63	33.852358					1.2E+08	1.2E+08	0.12959	ID=promoter_51687;
8	1E+08	8_117292356	16	47	16	31	32	24	0.247982	1	34	32	37	23	33	30	0.99699964	1	29.5	10.6770783								
8	1E+08	8_117307138	38	48	9	32	43	21	0.003115	0.16067	32	16	15	25	24	20	0.93987967	1	26.875	13.1196418								
8	1E+08	8_117340471	4	3	0	0	4	0	0.09576	1	19	9	5	10	14	8	0.54462139	1	6.25	6.31890587					1.2E+08	1.2E+08	0.39959	ID=promoter_51702;
8	1E+08	8_117384728	39	13	35	19	26	27	0.906335	1	2	3	2	0	3	1	0.65196691	1	14.125	15.5695444								
8	1E+08	8_117392409	4	84	11	26	44	19	7.50E-04	0.04944	84	43	41	27	64	34	0.02926519	0.917157	40	30.1993377								
8	1E+08	8_117432400	376	219	294	384	298	339	0.928351	1	191	132	258	117	162	188	0.11035632	1	246.38	101.26477					1.2E+08	1.2E+08	0.29605	ID=promoter_51718;
8	1E+08	8_117433355	146	45	139	138	96	139	0.282551	1	143	36	91	102	90	97	0.58813093	1	105	44.6190222								
8	1E+08	8_117522468	74	136	111	64	105	88	0.06519	1	63	38	26	22	51	24	0.02029992	0.6899501	66.75	40.209274					1.2E+08	1.2E+08	0.33323	ID=promoter_51729;
8	1E+08	8_117526907	0	9	0	4	5	2	0.402696	1	0	64	0	1	32	1	1.47E-06	0.0002348	9.75	22.1472346								
8	1E+08	8_117536365	267	101	136	132	184	134	1.27E-04	0.01105	211	68	101	28	140	65	1.60E-05	0.0019054	130.5	76.8095976								
8	1E+08	8_117549374	4699	2727	5773	6134	3713	5954	1.11E-49	#######	3489	2483	5886	1638	2986	3762	5.17E-35	6.48E-32	4103.6	1749.25126					1.2E+08	1.2E+08	0.67591	ID=promoter_51738;

8	1E+08	8_117580690	15	2	9	6	9	8	0.848833	1	44	19	18	0	32	9	0.00363228	0.1775497	14.125	13.994259								
8	1E+08	8_117614517	19	37	20	7	28	14	0.027228	0.89953	4	36	19	5	20	12	0.48622029	1	18.375	12.9607925								
8	1E+08	8_117714858	28	55	36	22	42	29	0.084977	1	18	31	22	4	25	13	0.22997911	1	27	14.8420253								
8	1E+08	8_117744279	124	44	100	124	84	112	0.673869	1	260	77	184	60	169	122	0.10775515	1	121.63	71.0451315					1.2E+08	1.2E+08	0.17844	ID=promoter_51771;
8	1E+08	8_117755915	10	18	10	6	14	8	0.253058	1	3	16	24	24	10	24	0.03201086	0.981237	13.875	7.90004521								
8	1E+08	8_117797325	0	32	6	25	16	16	0.903021	1	122	69	36	6	96	21	5.54E-10	1.69E-07	37	40.9005763	1E+08	1.2E+08	ID=ENSP0000378945;PRKG2;NM_006259;	PRKG2				
8	1E+08	8_117799527	66	17	42	22	42	32	0.179623	1	7	38	49	19	23	34	0.21851877	1	32.5	19.6323348	1E+08	1.2E+08	ID=ENSP0000378945;PRKG2;NM_006259;	PRKG2	1.2E+08	1.2E+08	0.14606	ID=promoter_51782;
8	1E+08	8_117808784	64	77	41	33	71	37	2.13E-04	0.01712	59	64	34	7	62	21	1.76E-04	0.0148084	47.375	22.7466638	1E+08	1.2E+08	ID=ENSP0000378945;PRKG2;NM_006259;	PRKG2	1.2E+08	1.2E+08	0.38162	ID=promoter_51785;
8	1E+08	8_117852995	14	12	26	18	13	22	0.573969	1	12	1	2	0	7	1	0.13113995	1	10.625	9.14857522								
8	1E+08	8_117875504	7	21	7	2	14	5	0.053576	1	46	33	11	3	40	7	3.09E-05	0.003345	16.25	15.8812558					1.2E+08	1.2E+08	0.40528	ID=promoter_51798;
8	1E+08	8_117882794	0	17	14	26	9	20	0.269594	1	17	0	13	19	9	16	0.2823118	1	13.25	9.06720937								
8	1E+08	8_117899806	5	0	27	11	3	19	0.009621	0.39464	1	5	13	0	3	7	0.3829009	1	7.75	9.17683419								
8	1E+08	8_117919522	11	80	7	2	46	5	2.00E-09	#######	1	48	43	3	25	23	0.99965163	1	24.375	29.2376347								
8	1E+08	8_117990261	0	8	13	14	4	14	0.128496	1	10	0	7	7	5	7	0.77775394	1	7.375	5.23552426								
8	1E+08	8_118036085	2	19	2	27	11	15	0.930409	1	0	1	13	1	1	7	0.08320466	1	8.125	10.2878222								
8	1E+08	8_118046844	40	21	14	33	31	24	0.30595	1	70	18	24	1	44	13	6.70E-04	0.0451887	27.625	20.7773056								
8	1E+08	8_118179848	40	92	101	80	66	91	0.605873	1	33	82	21	49	58	35	0.12965177	1	62.25	30.0463927								
8	1E+08	8_118278967	10	28	5	1	19	3	7.41E-04	0.04898	18	21	14	7	20	11	0.37181688	1	13	9.00793301								
8	1E+08	8_118284600	27	21	5	11	24	8	0.004476	0.21437	0	0	17	26	0	22	7.03E-06	0.0009311	13.375	11.018653								
8	1E+08	8_118293191	35	56	27	23	46	25	0.006513	0.28949	0	1	7	0	1	4	0.36109265	1	18.625	20.3325039								
8	1E+08	8_118297149	9	6	1	0	8	1	0.03583	1	0	0	14	21	0	18	6.08E-05	0.0059624	6.375	7.83649885								
8	1E+08	8_118333941	10	21	7	6	16	7	0.076817	1	21	5	8	0	13	4	0.12786863	1	9.75	7.51664819					1.2E+08	1.2E+08	0.43178	ID=promoter_51829;
8	1E+08	8_118389977	62	22	24	38	42	31	0.142577	1	0	14	1	1	7	1	0.13113995	1	20.25	21.5986111								
8	1E+08	8_118398752	16	4	1	10	10	6	0.41703	1	0	23	9	9	12	9	0.89054267	1	9	7.70899289								
8	1E+08	8_118427234	16	24	17	35	20	26	0.941925	1	34	53	21	21	44	21	0.03878541	1	27.625	12.4663834								
8	1E+08	8_118451738	32	30	35	17	31	26	0.448024	1	45	23	12	0	34	6	1.54E-04	0.0131747	24.25	14.3003496								
8	1E+08	8_118465709	6	23	37	28	15	33	0.121516	1	8	29	10	10	19	10	0.34022508	1	18.875	11.7890688								
8	1E+08	8_118483261	1533	742	1478	1010	1138	1244	0.228519	1	788	638	551	257	713	404	1.42E-14	7.04E-12	874.63	444.509983					1.2E+08	1.2E+08	0.51109	ID=promoter_51837;
8	1E+08	8_118517519	22	4	1	0	13	1	0.002067	0.11504	16	3	17	1	10	9	0.99792604	1	8	8.81557064					1.2E+08	1.2E+08	0.22358	ID=promoter_51841;
8	1E+08	8_118543271	11	13	19	18	12	19	0.716431	1	8	11	23	30	10	27	0.01023344	0.4008669	16.625	7.3472541					1.2E+08	1.2E+08	0.10303	ID=promoter_51847;
8	1E+08	8_118581470	22	22	24	35	22	30	0.865661	1	8	24	17	14	16	16	0.97729577	1	20.75	8.01338167								

8	1E+08	8_118634203	1246	383	932	767	815	850	0.056453	1	232	296	411	111	264	261	0.76213748	1	547.25	393.326382	1E+08	1.2E+08	ID=ENSP0000311697;FGF5;NM_004464;	FGF5	1.2E+08	1.2E+08	0.77087	ID=promoter_51863;
8	1E+08	8_118660261	1	57	6	12	29	9	8.94E-04	0.05763	14	3	7	6	9	7	0.94097808	1	13.25	18.1875153					1.2E+08	1.2E+08	0.30691	ID=promoter_51868;
8	1E+08	8_118670304	115	74	63	38	95	51	2.34E-05	0.00252	19	55	57	15	37	36	0.97888929	1	54.5	32.098064					1.2E+08	1.2E+08	0.32635	ID=promoter_51869;
8	1E+08	8_118684719	305	113	275	154	209	215	0.401022	1	175	55	112	53	115	83	0.21905318	1	155.25	93.5929637					1.2E+08	1.2E+08	0.13546	ID=promoter_51873;
8	1E+08	8_118689754	1989	5790	4792	11750	3890	8271	########	#######	6595	4776	14295	3300	5686	8798	6.97E-202	6.22E-198	6660.9	4227.61609					1.2E+08	1.2E+08	0.71502	ID=promoter_51874;
8	1E+08	8_118719344	258	79	172	119	169	146	0.024934	0.84252	50	43	139	81	47	110	2.61E-07	5.00E-05	117.63	71.8091469					1.2E+08	1.2E+08	0.59823	ID=promoter_51879;
8	1E+08	8_118738101	1	5	7	13	3	10	0.252243	1	0	13	10	0	7	5	0.9051169	1	6.125	5.51459104								
8	1E+08	8_118792940	7	0	5	1	4	3	0.840045	1	17	22	27	1	20	14	0.72101732	1	10	10.5424313								
8	1E+08	8_118809282	8	9	3	24	9	14	0.802715	1	63	34	28	18	49	23	0.02311888	0.762351	23.375	19.2423454					1.2E+08	1.2E+08	0.1077	ID=promoter_51888;
8	1E+08	8_118811391	57	65	48	61	61	55	0.365476	1	28	72	116	19	50	68	0.11742194	1	58.25	29.6153918					1.2E+08	1.2E+08	0.1537	ID=promoter_51889;
8	1E+08	8_118882521	4	26	22	21	15	22	0.799617	1	3	5	18	19	4	19	0.00415462	0.1961166	14.75	9.2234174								
8	1E+08	8_118883647	23	2	29	31	13	30	0.115644	1	26	27	36	3	27	20	0.74799297	1	22.125	12.6991282								
8	1E+08	8_118889786	21	42	11	16	32	14	0.005901	0.26867	86	48	32	21	67	27	8.47E-04	0.0552224	34.625	24.3658866					1.2E+08	1.2E+08	0.11202	ID=promoter_51899;
8	1E+08	8_118897854	20	1	24	24	11	24	0.223145	1	51	56	34	37	54	36	0.30582789	1	30.875	17.6832245								
8	1E+08	8_118949570	31	11	27	24	21	26	0.983171	1	62	50	70	0	56	35	0.18347418	1	34.375	24.4302944								
8	1E+08	8_119026305	42	61	47	42	52	45	0.32494	1	13	18	13	30	16	22	0.48233468	1	33.25	17.6291155					1.2E+08	1.2E+08	0.25695	ID=promoter_51911;
8	1E+08	8_119036294	21	17	10	1	19	6	0.010861	0.43721	5	0	9	22	3	16	0.00701108	0.2971393	10.625	8.60128728					1.2E+08	1.2E+08	0.26053	ID=promoter_51912;
8	1E+08	8_119089662	10	7	5	19	9	12	0.95859	1	0	0	0	0	0	0	1	1	5.125	6.81254096								
8	1E+08	8_119105976	76	19	45	46	48	46	0.618	1	78	20	2	1	49	2	2.18E-09	5.99E-07	35.875	30.4041233					1.2E+08	1.2E+08	0.16759	ID=promoter_51918;
8	1E+08	8_119107156	35	57	82	20	46	51	0.962331	1	28	27	37	9	28	23	0.91166341	1	36.875	22.9374148								
8	1E+08	8_119234516	172	84	296	205	128	251	1.07E-05	0.00127	27	40	51	59	34	55	0.03539654	1	116.75	96.8411807								
8	1E+08	8_119276517	5	12	7	13	9	10	0.993074	1	25	6	23	1	16	12	0.85678722	1	11.5	8.61891607								
8	1E+08	8_119309419	10	7	14	18	9	16	0.603365	1	0	0	1	0	0	1	0.58312783	1	6.25	7.14642768								
8	1E+08	8_119317248	10	12	0	12	11	6	0.3028	1	25	13	11	2	19	7	0.09662636	1	10.625	7.59581652								
8	1E+08	8_119325979	33	68	26	8	51	17	1.02E-05	0.00122	31	28	74	32	30	53	0.01619749	0.5774592	37.5	22.1939503								
8	1E+08	8_119331810	0	15	15	10	8	13	0.766846	1	1	3	10	2	2	6	0.31385293	1	7	6.23354978					1.2E+08	1.2E+08	0.35676	ID=promoter_51927;
8	1E+08	8_119374808	157	90	257	217	124	237	5.03E-05	0.00486	148	95	63	88	122	76	0.0235369	0.7739903	139.38	68.791481								
8	1E+08	8_119507798	7	42	24	22	25	23	0.701069	1	0	0	17	0	0	9	0.00779604	0.3222758	14	15.090205					1.2E+08	1.2E+08	0.19344	ID=promoter_51932;
8	1E+08	8_119521739	3	17	11	5	10	8	0.719353	1	17	16	0	0	17	0	3.78E-04	0.0279647	8.625	7.50119038								
8	1E+08	8_119549134	39	72	31	32	56	32	0.004101	0.20025	56	77	118	36	67	77	0.43596115	1	57.625	30.2604763								

8	1E+08	8_119576571	3	13	4	6	8	5	0.532535	1	2	6	8	13	4	11	0.14780855	1	6.875	4.22365786								
8	1E+08	8_119616890	53	51	67	67	52	67	0.87877	1	17	10	9	4	14	7	0.39930956	1	34.75	27.2907624	1E+08	1.2E+08	ID=ENSP00000378959;PAQR3;NM_001040202;	PAQR3	1.2E+08	1.2E+08	0.11722	ID=promoter_51939;
8	1E+08	8_119618886	21	9	6	3	15	5	0.034023	1	1	22	3	1	12	2	0.04045894	1	8.25	8.59817256	1E+08	1.2E+08	ID=ENSP00000378959;PAQR3;NM_001040202;	PAQR3	1.2E+08	1.2E+08	0.17364	ID=promoter_51940;
8	1E+08	8_119921600	22	62	7	4	42	6	6.04E-08	#######	24	18	21	0	21	11	0.29862283	1	19.75	19.3224518								
9	3368	9_3368	114	90	174	206	102	190	7.27E-04	0.04855	1	10	17	8	6	13	0.20797135	1	77.5	81.2509341					1984	3859	0.24747	ID=promoter_51946;
9	19123	9_19123	16	21	15	29	19	22	0.999149	1	54	6	36	29	30	33	0.79414776	1	25.75	14.868471					18571	20254	0.40483	ID=promoter_51950;
9	20519	9_20519	12	27	11	7	20	9	0.046279	1	41	43	25	21	42	23	0.12157761	1	23.375	13.4582688								
9	36788	9_36788	53	70	31	29	62	30	2.12E-04	0.01705	81	19	51	3	50	27	0.07256683	1	42.125	26.3136982					35558	38129	0.41467	ID=promoter_51956;
9	37543	9_37543	169	130	67	87	150	77	1.04E-08	#######	79	69	8	39	74	24	1.76E-05	0.00207	81	50.1682882					35558	38129	0.41467	ID=promoter_51956;
9	55336	9_55336	14	1	0	0	8	0	0.00917	0.37984	17	2	6	0	10	3	0.19568363	1	5	6.82432624								
9	75139	9_75139	29	70	60	56	50	58	0.998346	1	88	27	44	46	58	45	0.66857123	1	52.5	20.5356832					73214	79121	0.82873	ID=promoter_51964;
9	77276	9_77276	9	3	8	7	6	8	0.9722	1	10	7	1	1	9	1	0.05455957	1	5.75	3.57571172					73214	79121	0.82873	ID=promoter_51964;
9	105672	9_105672	57	35	81	60	46	71	0.347009	1	13	0	6	3	7	5	0.9051169	1	31.875	30.9858839					104750	105952	0.20067	ID=promoter_51973;
9	110992	9_110992	59	32	39	19	46	29	0.02902	0.94901	44	30	43	3	37	23	0.31728647	1	33.625	17.0540108								
9	132339	9_132339	5	15	22	14	10	18	0.549586	1	4	8	24	5	6	15	0.10150651	1	12.125	7.88194139					128521	132495	0.70136	ID=promoter_51983;
9	159978	9_159978	0	27	4	11	14	8	0.253058	1	0	4	21	11	2	16	0.00248877	0.1311269	9.75	9.88144003								
9	163239	9_163239	6	26	0	0	16	0	8.41E-05	0.00761	0	22	19	14	11	17	0.4100993	1	10.875	10.7362869								
9	177247	9_177247	43	48	63	44	46	54	0.999992	1	25	44	51	27	35	39	0.73123094	1	43.125	12.368595								
9	206004	9_206004	6	4	8	18	5	13	0.299488	1	10	14	9	6	12	8	0.76853133	1	9.375	4.6271713					203812	207109	0.64366	ID=promoter_52000;
9	276155	9_276155	628	216	609	465	422	537	0.456304	1	151	79	53	7	115	30	3.30E-10	1.03E-07	276	253.725949					275798	277755	0.33736	ID=promoter_52021;
9	278918	9_278918	5	23	29	17	14	23	0.607375	1	34	29	23	9	32	16	0.1226614	1	21.125	10.1480118					278237	279519	0.23326	ID=promoter_52022;
9	302580	9_302580	14	61	22	21	38	22	0.026787	0.89052	32	68	39	25	50	32	0.25798519	1	35.25	19.623237								
9	303943	9_303943	0	24	20	17	12	19	0.716431	1	6	29	32	17	18	25	0.42027749	1	18.125	10.8685326					303725	304280	0.15874	ID=promoter_52028;
9	317322	9_317322	8	10	16	16	9	16	0.603365	1	0	18	1	4	9	3	0.27712561	1	9.125	7.07990113								
9	317645	9_317645	160	132	340	331	146	336	3.76E-11	#######	63	85	32	35	74	34	0.00253133	0.13283	147.25	124.308085								
9	336893	9_336893	65	43	33	40	54	37	0.03878	1	33	19	14	25	26	20	0.8208633	1	34	15.9731918					334336	338215	0.47223	ID=promoter_52034;
9	338642	9_338642	1	27	32	80	14	56	7.20E-05	0.00668	0	9	23	1	5	12	0.17891578	1	21.625	26.7898568								
9	344526	9_344526	35	36	41	34	36	38	0.902223	1	55	21	23	4	38	14	0.00933665	0.3721703	31.125	15.2074935					341916	344936	0.58748	ID=promoter_52036;
9	391305	9_391305	0	30	2	7	15	5	0.034023	1	3	14	10	9	9	10	0.92505409	1	9.375	9.53096457					391011	391999	0.52387	ID=promoter_52042;
9	425149	9_425149	0	24	19	8	12	14	0.999907	1	0	19	11	3	10	7	0.84912739	1	10.5	9.33503386					424182	427408	0.5478	ID=promoter_52053;
9	449860	9_449860	9	7	6	16	8	11	0.943069	1	6	7	9	3	7	6	0.99011599	1	7.875	3.79614466					447835	451134	0.57865	ID=promoter_52060;

9	450027	9_450027	7	4	8	15	6	12	0.558139	1	11	6	10	7	9	9	0.98716474	1	8.5	3.42261387					447835	451134	0.57865	ID=promoter_52060;
9	451052	9_451052	11	10	3	4	11	4	0.10516	1	6	11	5	0	9	3	0.27712561	1	6.25	4.0620192					447835	451134	0.57865	ID=promoter_52060;
9	587918	9_587918	130	81	27	21	106	24	4.62E-15	#######	148	102	39	17	125	28	1.50E-12	6.15E-10	70.625	51.8870683					587644	589597	0.388	ID=promoter_52092;
9	613725	9_613725	21	16	7	27	19	17	0.718296	1	0	29	7	42	15	25	0.19020812	1	18.625	13.9072181					613230	613771	0.10972	ID=promoter_52096;
9	700274	9_700274	3	6	3	12	5	8	0.86114	1	41	13	4	24	27	14	0.20179887	1	13.25	13.2853303	689292	702985	ID=ENSP000002995 96;TMEM41B;NM_015012;	TMEM41B				
9	714727	9_714727	23	16	11	31	20	21	0.939065	1	27	31	17	2	29	10	0.0194	0.6626766	19.75	10.2085398					714120	715028	0.21062	ID=promoter_52105;
9	730949	9_730949	25	1	16	30	13	23	0.490912	1	17	8	2	4	13	3	0.06317581	1	12.875	10.8816687								
9	766641	9_766641	23	0	13	0	12	7	0.325249	1	7	11	16	16	9	16	0.2823118	1	10.75	8.06668634								
9	774047	9_774047	6	10	12	2	8	7	0.850897	1	6	14	12	1	10	7	0.84912739	1	7.875	4.85320218					773128	774690	0.18662	ID=promoter_52111;
9	790815	9_790815	71	56	30	46	64	38	0.003206	0.16476	75	87	40	27	81	34	3.64E-04	0.0271967	54	22	776735	821579	ID=ENSP000003088 20;RIC3;;	RIC3	790266	793447	0.49046	ID=promoter_52117;
9	821881	9_821881	15	0	19	8	8	14	0.661714	1	0	5	10	0	3	5	0.7175395	1	7.125	7.25923058					819792	824255	0.74715	ID=promoter_52121;
9	822688	9_822688	349	124	204	240	237	222	0.054401	1	32	47	25	1	40	13	0.00273506	0.1400212	127.75	125.180726					819792	824255	0.74715	ID=promoter_52121;
9	826521	9_826521	42	36	20	32	39	26	0.077552	1	19	27	14	17	23	16	0.67590535	1	25.875	10.0205147					824716	828769	0.643	ID=promoter_52122;
9	827612	9_827612	15	46	18	12	31	15	0.014573	0.54886	5	38	7	28	22	18	0.92557456	1	21.125	14.8366101					824716	828769	0.643	ID=promoter_52122;
9	836936	9_836936	0	48	4	18	24	11	0.027804	0.91326	39	15	42	0	27	21	0.83317938	1	20.75	19.6741309					836397	837088	0.19116	ID=promoter_52125;
9	840335	9_840335	39	22	50	49	31	50	0.375729	1	0	8	3	0	4	2	0.76928717	1	21.375	21.7908074					839829	840826	0.25624	ID=promoter_52126;
9	845392	9_845392	11	23	19	20	17	20	0.999959	1	27	11	17	18	19	18	0.99782856	1	18.25	5.47070118					843444	845553	0.33452	ID=promoter_52127;
9	852871	9_852871	6	2	12	13	4	13	0.177139	1	6	9	7	1	8	4	0.59180275	1	7	4.27617987								
9	862464	9_862464	29	5	25	13	17	19	0.989587	1	0	27	9	12	14	11	0.91900834	1	15	10.7835854								
9	964541	9_964541	0	14	0	5	7	3	0.314307	1	29	6	3	3	18	3	0.00813808	0.3342859	7.5	9.75412001								
9	1E+06	9_128256	19	92	47	35	56	41	0.068706	1	102	114	132	98	108	115	0.5853991	1	79.875	40.7901861								
9	1E+06	9_135385	24	19	22	6	22	14	0.192025	1	38	8	29	13	23	21	0.9987298	1	19.875	10.7893797								
9	1E+06	9_137654	1624	1459	2108	2312	1542	2210	1.13E-08	#######	1414	1140	2587	810	1277	1699	1.29E-21	9.45E-19	1681.8	606.684844								
9	1E+06	9_1382735	52	113	64	60	83	62	0.025356	0.85286	136	105	74	5	121	40	2.64E-08	6.11E-06	76.125	41.1111299					1382084	1383673	0.25989	ID=promoter_52166;
9	1E+06	9_1391360	2	16	1	6	9	4	0.244237	1	38	8	28	14	23	21	0.9987298	1	14.125	13.0322402					1390330	1391717	0.2189	ID=promoter_52168;
9	1E+06	9_1400731	16	21	18	32	19	25	0.931133	1	29	7	29	8	18	19	0.92496372	1	20	9.56182887					1399517	1402515	0.41369	ID=promoter_52172;
9	1E+06	9_1411625	28	57	18	24	43	21	0.003115	0.16067	49	17	8	19	33	14	0.04245489	1	27.5	16.8946314					1411472	1412974	0.23082	ID=promoter_52175;
9	1E+06	9_1417045	28	79	40	38	54	39	0.06618	1	66	79	89	54	73	72	0.93276295	1	59.125	22.478164					1416104	1417497	0.35211	ID=promoter_52177;
9	1E+06	9_145450	8	19	9	9	14	9	0.360318	1	3	4	13	4	4	9	0.31291743	1	8.625	5.37022213								
9	1E+06	9_146949	8	9	3	3	9	3	0.131542	1	24	10	2	7	17	5	0.05852787	1	8.25	7.04576874								
9	2E+06	9_151304	19	22	9	14	21	12	0.1273	1	42	42	38	2	42	20	0.04419125	1	23.5	15.4919334								
9	2E+06	9_153230	25	1	6	0	13	3	0.018372	0.65917	0	20	0	0	10	0	0.00970398	0.3824351	6.5	10.1699838								
9	2E+06	9_155983	31	65	31	10	48	21	4.53E-04	0.03263	40	24	9	3	32	6	3.53E-04	0.0265555	26.625	20.1489986								
9	2E+06	9_157696	55	63	90	101	59	96	0.138811	1	38	31	62	33	35	48	0.20763434	1	59.125	25.8204653								

9	2E+06	9_158949	22	12	25	18	17	22	0.954686	1	26	7	20	1	17	11	0.64636287	1	16.375	8.92728562								
9	2E+06	9_1642019	16	21	13	27	19	20	0.944476	1	92	34	72	24	63	48	0.59100593	1	37.375	28.7846065	2E+06	1700323	ID=ENSP00000384655-D4;;					
9	2E+06	9_1687751	10	9	6	19	10	13	0.970528	1	18	8	10	2	13	6	0.35360803	1	10.25	5.72588109	2E+06	1700323	ID=ENSP00000384655-D4;;		1687638	1688566	0.14257	ID=promoter_52220;
9	2E+06	9_1691003	21	14	23	47	18	35	0.21203	1	2	21	32	23	12	28	0.0214954	0.7254018	22.875	13.0212738	2E+06	1700323	ID=ENSP00000384655-D4;;					
9	2E+06	9_1725423	19	20	14	18	20	16	0.517469	1	0	0	0	0	0	0	1	1	8.875	9.64272487					1725133	1726106	0.17864	ID=promoter_52226;
9	2E+06	9_1813251	8	6	22	13	7	18	0.195146	1	37	24	10	16	31	13	0.04801516	1	17	10.2956301					1812587	1813599	0.25668	ID=promoter_52235;
9	2E+06	9_186133	28	48	21	25	38	23	0.038697	1	30	45	48	77	38	63	0.01626363	0.5795279	40.25	18.3283543								
9	2E+06	9_1892248	237	112	60	113	175	87	1.31E-10	#######	169	87	66	54	128	60	5.16E-05	0.0052142	112.25	62.887087					1892093	1893683	0.36026	ID=promoter_52243;
9	2E+06	9_192099	29	23	25	23	26	24	0.697783	1	2	20	1	10	11	6	0.57179039	1	16.625	10.8092223								
9	2E+06	9_201622	6	35	8	2	21	5	0.001802	0.10311	3	19	14	2	11	8	0.87190251	1	11.125	11.3947043								
9	2E+06	9_2115077	0	8	1	5	4	3	0.840045	1	0	7	0	31	4	16	0.01700101	0.6008545	6.5	10.4334626					2114496	2115867	0.20824	ID=promoter_52256;
9	2E+06	9_226362	33	6	26	26	20	26	0.941925	1	13	10	1	6	12	4	0.18067673	1	15.125	11.6672619								
9	2E+06	9_2318000	4	32	9	6	18	8	0.059652	1	18	19	10	2	19	6	0.05448017	1	12.5	9.97138764					2317505	2318790	0.21092	ID=promoter_52262;
9	2E+06	9_245331	5	10	3	7	8	5	0.532535	1	6	7	4	0	7	2	0.29886173	1	5.25	3.01188123								
9	3E+06	9_2546501	71	45	105	62	58	84	0.465107	1	118	33	40	0	76	20	6.14E-07	0.0001072	59.25	38.6624587	3E+06	2548438	ID=ENSP00000346716;DNHD1L;NM_173589;	DNHD1L				
9	3E+06	9_2563446	18	35	21	34	27	28	0.900886	1	60	60	40	65	60	53	0.96770353	1	41.625	18.1654106	3E+06	2564168	ID=ENSP00000379998;ARFIP2;NM_012402;	ARFIP2				
9	3E+06	9_2571024	13	16	6	17	15	12	0.610117	1	31	0	4	18	16	11	0.74569574	1	13.125	9.80433578					2570529	2571351	0.13954	ID=promoter_52273;
9	3E+06	9_2582098	19	22	12	7	21	10	0.052655	1	26	22	21	5	24	13	0.28736897	1	16.75	7.74135462					2581577	2583938	0.52977	ID=promoter_52275;
9	3E+06	9_2599863	355	146	255	172	251	214	0.002665	0.14204	107	56	34	57	82	46	0.0224437	0.749258	147.75	111.033779	3E+06	2606050	ID=ENSP00000265983;HPX;NM_000613;	HPX	2599251	2601280	0.27743	ID=promoter_52278;
9	3E+06	9_2602124	65	20	28	23	43	26	0.024991	0.84297	0	2	0	0	1	0	0.62906424	1	17.25	22.5562788	3E+06	2606050	ID=ENSP00000265983;HPX;NM_000613;	HPX	2602011	2602550	0.1626	ID=promoter_52279;
9	3E+06	9_262514	29	70	28	27	50	28	0.005898	0.26867	31	17	23	40	24	32	0.40021755	1	33.125	16.2782721								
9	3E+06	9_2737485	0	31	3	4	16	4	0.009581	0.39335	0	25	37	3	13	20	0.35609139	1	12.875	15.412773	3E+06	2742994	ID=ENSP00000265978;FAM160A2;NM_032127;	FAM160A2				
9	3E+06	9_2825509	2	14	0	9	8	5	0.532535	1	0	0	14	4	0	9	0.00779604	0.3222758	5.375	6.11642986					2824652	2826835	0.33853	ID=promoter_52321;
9	3E+06	9_304640	30	49	6	17	40	12	4.56E-05	0.00449	16	14	24	1	15	13	0.98432481	1	19.625	14.9946419								
9	3E+06	9_310826	581	159	122	124	370	123	4.80E-37	#######	131	141	32	0	136	16	2.12E-19	1.42E-16	161.25	178.547913								
9	3E+06	9_311787	18	11	0	10	15	5	0.034023	1	19	3	7	6	11	7	0.73613127	1	9.25	6.71352579								
9	3E+06	9_316306	117	86	73	97	102	85	0.064862	1	0	22	8	1	11	5	0.40227598	1	50.5	47.7643023								
9	3E+06	9_3256875	41	95	77	153	68	115	0.055432	1	24	56	34	0	40	17	0.02201768	0.7404021	60	47.9761846	3E+06	3281915	ID=ENSP00000338711;TRIM6;NM_001003819;	TRIM6				
9	3E+06	9_3428524	14	15	5	22	15	14	0.827043	1	18	58	31	59	38	45	0.53763968	1	27.75	20.3522551					3428255	3429924	0.20025	ID=promoter_52355;

9	4E+06	9_364646	5	0	8	3	3	6	0.747087	1	0	17	32	2	9	17	0.21129426	1	8.375	11.0445526								
9	4E+06	9_368848	0	9	14	6	5	10	0.615109	1	43	2	1	0	23	1	9.32E-05	0.0086863	9.375	14.4611549								
9	4E+06	9_374088	25	0	12	41	13	27	0.229532	1	20	11	17	0	16	9	0.48000706	1	15.75	13.4986772								
9	4E+06	9_382685	25	3	3	2	14	3	0.010924	0.43763	21	11	6	2	16	4	0.04223275	1	9.125	9.12512231								
9	4E+06	9_387320	33	46	19	30	40	25	0.042829	1	23	33	63	36	28	50	0.01836895	0.6365951	35.375	13.8247966								
9	4E+06	9_394114	37	18	32	33	28	33	0.999827	1	11	14	34	31	13	33	0.00582644	0.2576386	26.25	10.1945364								
9	4E+06	9_4017808	18	30	20	4	24	12	0.04556	1	0	24	19	34	12	27	0.03064003	0.9477626	18.625	11.6978325					4017329	4017864	0.13408	ID=promoter_52410;
9	4E+06	9_4025866	34	67	30	35	51	33	0.025979	0.86911	21	27	31	7	24	19	0.87598349	1	31.5	16.9537185					4025409	4025983	0.12082	ID=promoter_52413;
9	4E+06	9_422130	0	7	3	5	4	4	0.97482	1	16	15	0	0	16	0	6.01E-04	0.0409216	5.75	6.54107898								
9	4E+06	9_423759	14	18	2	5	16	4	0.009581	0.39335	50	0	12	0	25	6	0.00589984	0.2600594	12.625	16.552406								
9	4E+06	9_435820	93	38	94	105	66	100	0.26986	1	3	12	36	29	8	33	1.82E-04	0.015179	51.25	40.0562105								
9	4E+06	9_443836	2	8	1	0	5	1	0.186119	1	1	15	16	0	8	8	0.98858271	1	5.375	6.75991336								
9	5E+06	9_464406	11	5	3	7	8	5	0.532535	1	8	11	4	0	10	2	0.09294023	1	6.125	3.87067731								
9	5E+06	9_464424	151	70	72	118	111	95	0.077025	1	65	67	65	15	66	40	0.10191864	1	77.875	40.456105								
9	5E+06	9_523151	2	15	4	15	9	10	0.993074	1	8	9	3	11	9	7	0.94097808	1	8.375	5.12521777								
9	5E+06	9_530441	110	74	37	45	92	41	5.86E-07	#######	77	80	29	33	79	31	1.59E-04	0.0135699	60.625	28.849053								
9	5E+06	9_5449477	50	29	47	28	40	38	0.647488	1	1	22	10	3	12	7	0.61698677	1	23.75	18.5760983	5E+06	5477234	ID=ENSP00000300737;STIM1;NM_003156;	STIM1	5449296	5449827	0.12879	ID=promoter_52477;
9	6E+06	9_550612	84	20	25	41	52	33	0.019645	0.69635	2	17	20	0	10	10	0.98574881	1	26.125	26.7124235								
9	6E+06	9_552717	9	26	37	17	18	27	0.720164	1	0	26	42	19	13	31	0.01226355	0.4638477	22	13.8564065								
9	6E+06	9_5726290	1	13	2	4	7	3	0.314307	1	0	8	10	21	4	16	0.01700101	0.6008545	7.375	7.17012652	6E+06	5824091	ID=ENSP00000316032;NUP98;NM_016320;	NUP98				
9	6E+06	9_5872649	28	29	42	28	29	35	0.993567	1	34	12	48	28	23	38	0.08548704	1	31.125	10.7628661	6E+06	5873864	ID=ENSP00000368966-D5;TRPC3;NM_001130698;	TRPC3				
9	6E+06	9_5886348	8	18	2	3	13	3	0.018372	0.65917	21	3	5	19	12	12	0.98292303	1	9.875	8.07885246					5885135	5888150	0.45958	ID=promoter_52518;
9	6E+06	9_597702	2	7	4	1	5	3	0.645778	1	17	12	26	8	15	17	0.85096642	1	9.625	8.46737436								
9	6E+06	9_610740	9	5	27	7	7	17	0.253572	1	0	23	14	2	12	8	0.76853133	1	10.875	9.76052545								
9	6E+06	9_616621	0	11	7	5	6	6	0.962469	1	9	8	0	1	9	1	0.05455957	1	5.125	4.3239367								
9	6E+06	9_626200	8	0	1	3	4	2	0.597627	1	22	15	0	5	19	3	0.00532306	0.24159	6.75	7.95972002								
9	6E+06	9_634581	17	28	20	9	23	15	0.200003	1	1	0	14	49	1	32	1.40E-07	2.84E-05	17.25	15.9082189								
9	6E+06	9_636372	0	7	19	2	4	11	0.322758	1	0	16	0	7	8	4	0.59180275	1	6.375	7.50119038								
9	6E+06	9_637961	223	132	232	205	178	219	0.894024	1	128	175	85	73	152	79	1.30E-04	0.0113876	156.63	61.3117036								
9	6E+06	9_6390289	5	6	23	27	6	25	0.011836	0.46238	1	8	8	4	5	6	0.91271846	1	10.25	9.43776608					6389389	6391672	0.47563	ID=promoter_52583;
9	6E+06	9_639216	239	39	139	140	139	140	0.444371	1	0	11	17	1	6	9	0.65381141	1	73.25	88.6804053								
9	6E+06	9_6400459	19	11	32	24	15	28	0.341844	1	1	9	2	27	5	15	0.05518429	1	15.625	11.6120319					6399981	6401126	0.21249	ID=promoter_52585;
9	7E+06	9_665228	22	21	42	18	22	30	0.865661	1	1	7	0	1	4	1	0.45332778	1	14	14.6189701								
9	7E+06	9_675004	9	20	25	14	15	20	0.931941	1	2	7	25	2	5	14	0.08295177	1	13	9.50187951								
9	7E+06	9_6798682	1255	404	1929	1731	830	1830	8.92E-52	#######	455	411	556	414	433	485	0.01648644	0.5857131	894.38	645.076726					6798207	6799662	0.24313	ID=promoter_52603;
9	7E+06	9_6828563	313	294	399	374	304	387	0.564997	1	111	35	106	21	73	64	0.94824117	1	206.63	154.528719					6828493	6829054	0.15846	ID=promoter_52608;
9	7E+06	9_686980	23	15	11	24	19	18	0.809248	1	0	1	2	1	1	2	0.81304206	1	9.625	10.1127571								
9	7E+06	9_6876174	3	21	4	0	12	2	0.011798	0.46127	0	0	10	11	0	11	0.00265095	0.136745	6.125	7.43423548	7E+06	6910802	ID=ENSP00000377233;ARAP1;NM_001040118;	ARAP1	6875687	6876187	0.15832	ID=promoter_52616;
9	7E+06	9_6894600	26	32	71	64	29	68	0.006248	0.2817	1	28	16	27	15	22	0.38478854	1	33.125	23.3876494	7E+06	6910802	ID=ENSP00000377233;ARAP1;NM_001040118;	ARAP1				

9	7E+06	9_6942257	7	56	2	5	32	4	1.65E-06	0.00024	0	29	2	4	15	3	0.02833385	0.8918846	13.125	19.6282413	7E+06	6958801	ID=ENSP00000335247;STARD10;NM_006645;	STARD10				
9	7E+06	9_6991742	4	19	6	6	12	6	0.213448	1	23	14	27	1	19	14	0.80779431	1	12.5	9.66584561	7E+06	6993129	ID=ENSP00000326340;ATG16L2;NM_033388;	ATG16L2				
9	7E+06	9_725128	10	28	8	13	19	11	0.163668	1	29	9	17	4	19	11	0.45547225	1	14.75	9.28516482								
9	7E+06	9_735205	18	19	20	14	19	17	0.718296	1	12	3	31	4	8	18	0.09791732	1	15.125	9.10945349								
9	7E+06	9_7359263	517	195	376	233	356	305	2.82E-04	0.02189	147	126	223	85	137	154	0.25907129	1	237.75	143.037208					7358763	7359582	0.13832	ID=promoter_52643;
9	7E+06	9_7360802	405	105	286	189	255	238	0.039626	1	134	84	84	10	109	47	4.11E-05	0.0043023	162.13	127.872076					7360519	7361827	0.39454	ID=promoter_52644;
9	7E+06	9_738504	26	28	14	18	27	16	0.087673	1	0	0	9	0	0	5	0.06742467	1	11.875	11.5441698								
9	7E+06	9_7461482	55	22	34	16	39	25	0.056901	1	63	30	26	16	47	21	0.01758384	0.6154554	32.75	17.4908139					7461153	7462547	0.27308	ID=promoter_52656;
9	7E+06	9_7471265	259	45	106	100	152	103	7.67E-05	0.00707	32	12	12	24	22	18	0.92557456	1	73.75	83.424474					7471182	7472777	0.3525	ID=promoter_52658;
9	8E+06	9_7502098	13	16	3	13	15	8	0.181584	1	36	26	12	31	31	22	0.62998213	1	18.75	11.132321	8E+06	7509735	ID=ENSP00000348852-D2;FAM168A;NM_015159;	FAM168A				
9	8E+06	9_7594236	484	312	532	273	398	403	0.114335	1	294	253	187	118	274	153	2.53E-06	0.0003791	306.63	139.560471					7593736	7594555	0.13102	ID=promoter_52668;
9	8E+06	9_7891093	357	200	292	291	279	292	0.395406	1	49	116	146	41	83	94	0.41214619	1	186.5	118.315559					7890705	7891264	0.13133	ID=promoter_52675;
9	8E+06	9_7963611	310	84	111	102	197	107	4.22E-10	#######	45	59	35	46	52	41	0.73995351	1	99	89.6979057					7962925	7964108	0.26662	ID=promoter_52677;
9	8E+06	9_802509	13	23	4	7	18	6	0.017303	0.62786	16	7	1	3	12	2	0.04045894	1	9.25	7.49761867								
9	8E+06	9_8037299	99	36	58	39	68	49	0.031826	1	9	50	20	46	30	33	0.79414776	1	44.625	27.1184571	8E+06	8037746	ID=ENSP00000344431;DNAJB13;NM_153614;	DNAJB13				
9	8E+06	9_805782	12	8	3	8	10	6	0.41703	1	90	6	21	26	48	24	0.04295976	1	21.75	28.6593291								
9	8E+06	9_8083794	3	12	5	7	8	6	0.705675	1	3	8	26	15	6	21	0.00855938	0.348087	9.875	7.75403121	8E+06	8095807	ID=ENSP00000334379;C2CD3;;	C2CD3	8083273	8084148	0.15217	ID=promoter_52689;
9	8E+06	9_8085246	0	15	2	1	8	2	0.097885	1	0	8	0	19	4	10	0.21760186	1	5.625	7.57698583	8E+06	8095807	ID=ENSP00000334379;C2CD3;;	C2CD3				
9	8E+06	9_820722	5	21	15	12	13	14	0.975695	1	4	29	29	3	17	16	0.99905703	1	14.75	10.7004673								
9	9E+06	9_8715775	51	10	0	1	31	1	5.83E-08	#######	20	13	1	0	17	1	0.00146248	0.085933	12	17.4027912	9E+06	8717765	ID=ENSP00000365510;CHRDL2;;	CHRDL2				
9	9E+06	9_871858	72	38	10	36	55	23	8.13E-05	0.00739	194	117	95	96	156	96	0.00635091	0.2749222	82.25	57.9032444								
9	9E+06	9_887263	24	40	37	35	32	36	0.985347	1	82	25	64	29	54	47	0.95121577	1	42	20.5078382								
9	9E+06	9_892039	707	673	279	282	690	281	6.02E-54	#######	845	918	529	205	882	367	8.19E-39	1.16E-35	554.75	274.324495								
9	9E+06	9_894119	107	63	103	142	85	123	0.328739	1	36	68	61	34	52	48	0.99977053	1	76.75	37.5376002								
9	9E+06	9_895269	27	17	44	27	22	36	0.465846	1	0	18	13	0	9	7	0.94097808	1	18.25	14.6945082								
9	9E+06	9_898665	0	27	9	21	14	15	0.970741	1	31	26	38	9	29	24	0.91882689	1	20.125	12.9442486								
9	9E+06	9_9055128	690	337	610	346	514	478	0.001227	0.07505	510	258	282	124	384	203	4.58E-10	1.41E-07	394.63	191.690925					9054212	9055618	0.38109	ID=promoter_52755;
9	9E+06	9_914192	2060	2021	2582	3329	2041	2956	1.99E-12	#######	1911	1674	3651	1298	1793	2475	5.52E-37	7.58E-34	2315.8	814.530847								
9	1E+07	9_965989	38	88	11	25	63	18	7.23E-08	#######	37	80	84	50	59	67	0.52305481	1	51.625	29.1250383								
9	1E+07	9_970088	15	13	19	4	14	12	0.725787	1	119	10	51	31	65	41	0.15152082	1	32.75	37.8219513								
9	1E+07	9_987735	2	32	8	12	17	10	0.210142	1	8	10	45	2	9	24	0.01819934	0.631944	14.875	15.3942243								
9	1E+07	9_997424	13	44	12	16	29	14	0.018844	0.67198	40	13	20	20	27	20	0.74799297	1	22.25	12.6123522								
9	1E+07	9_101460	151	53	133	97	102	115	0.958566	1	19	10	29	1	15	15	0.97869956	1	61.625	58.1007438								
9	1E+07	9_102281	0	37	12	12	19	12	0.233425	1	38	16	15	16	27	16	0.35958363	1	18.25	12.947697								

9	1E+07	9_10385369	2616	2585	3397	5048	2601	4223	5.33E-38	#######	2931	2140	5680	1839	2536	3760	1.37E-74	3.43E-71	3279.5	1379.50892					1E+07	1E+07	0.4727	ID=promoter_52830;
9	1E+07	9_104062	40	17	54	33	29	44	0.558719	1	13	9	3	20	11	12	0.92663959	1	23.625	17.270433								
9	1E+07	9_10422373	15	15	8	27	15	18	0.997876	1	31	28	56	23	30	40	0.31832457	1	25.375	14.6476473					1E+07	1E+07	0.13801	ID=promoter_52833;
9	1E+07	9_10448961	569	249	478	431	409	455	0.739388	1	171	122	176	18	147	97	0.03304961	1	276.75	193.72569					1E+07	1E+07	0.58028	ID=promoter_52834;
9	1E+07	9_104967	339	141	259	268	240	264	0.771602	1	191	124	116	39	158	78	2.22E-05	0.0025318	184.63	98.4522183								
9	1E+07	9_105640	11	26	5	8	19	7	0.021688	0.74727	48	3	35	22	26	29	0.79045324	1	19.75	15.9798981								
9	1E+07	9_105897	71	21	25	48	46	37	0.228422	1	74	23	23	88	49	56	0.56303155	1	46.625	27.5107122								
9	1E+07	9_10638065	139	96	216	186	118	201	0.005326	0.24715	65	59	91	20	62	56	0.99008213	1	109	66.6847594					1.1E+07	1.1E+07	0.22728	ID=promoter_52846;
9	1E+07	9_108539	2	21	2	9	12	6	0.213448	1	9	30	5	5	20	5	0.01914528	0.6572777	10.375	9.9991071								
9	1E+07	9_109796	21	32	22	13	27	18	0.170316	1	0	1	5	10	1	8	0.04988758	1	13	11.2884264								
9	1E+07	9_110268	4	6	18	10	5	14	0.226542	1	56	11	18	1	34	10	0.00342551	0.1709558	15.5	17.4683387								
9	1E+07	9_11107513	76	20	17	10	48	14	4.54E-06	0.00059	28	19	19	11	24	15	0.48349198	1	25	21.3541565	1E+07	1.1E+07	ID=ENSP0000354087;CAPN5;;	CAPN5				
9	1E+07	9_121174	12	2	6	9	7	8	0.998739	1	32	12	17	33	22	25	0.78399411	1	15.375	11.4634512								
9	1E+07	9_123473	9	25	2	12	17	7	0.051092	1	29	19	28	2	24	15	0.48349198	1	15.75	11.0808715								
9	1E+07	9_12640119	9	48	22	14	29	18	0.097648	1	17	22	40	22	20	31	0.1936646	1	24.25	13.1881982	1E+07	1.3E+07	ID=ENSP0000354952;GAB2;;	GAB2				
9	1E+07	9_126788	1	33	4	2	17	3	0.002206	0.1213	46	6	21	5	26	13	0.18179217	1	14.75	16.8501802								
9	1E+07	9_13062271	0	12	12	6	6	9	0.896349	1	12	0	9	6	6	8	0.79537884	1	7.125	5.05505405					1.3E+07	1.3E+07	0.16367	ID=promoter_52923;
9	1E+07	9_130770	24	24	14	17	24	16	0.207334	1	34	42	25	3	38	14	0.00933665	0.3721703	22.875	11.9813844								
9	1E+07	9_130776	27	62	48	16	45	32	0.091522	1	0	29	22	23	15	23	0.30916076	1	28.375	19.0708266								
9	1E+07	9_13244728	82	35	50	55	59	53	0.368108	1	34	17	39	73	26	56	0.00124088	0.0746948	48.125	21.5170198					1.3E+07	1.3E+07	0.15997	ID=promoter_52936;
9	1E+07	9_132753	62	21	94	77	42	86	0.011173	0.44648	32	15	32	6	24	19	0.87598349	1	42.375	31.6089386								
9	1E+07	9_132950	20	91	29	17	56	23	5.37E-05	0.00515	41	64	48	5	53	27	0.03678266	1	39.375	28.06593								
9	1E+07	9_133852	29	44	87	58	37	73	0.033525	1	190	126	201	96	158	149	0.98884329	1	103.88	64.3460233								
9	1E+07	9_134047	326	194	339	325	260	332	0.590866	1	158	135	212	160	147	186	0.01823018	0.632861	231.13	85.2231315								
9	1E+07	9_135655	1	0	5	3	1	4	0.505922	1	15	7	6	8	11	7	0.73613127	1	5.625	4.71888304								
9	1E+07	9_135743	6	13	3	2	10	3	0.082185	1	0	9	15	10	5	13	0.12287893	1	7.25	5.3917927								
9	1E+07	9_135797	128	97	138	134	113	136	0.979758	1	94	93	52	17	94	35	1.16E-05	0.0014428	94.125	42.0796439								
9	1E+07	9_135882	39	60	28	51	50	40	0.192624	1	53	40	41	23	47	32	0.40088139	1	41.875	12.5178444								
9	1E+07	9_137115	12	23	31	21	18	26	0.793453	1	55	32	20	7	44	14	0.00126336	0.0755071	25.125	14.7496973								
9	1E+07	9_138397	7	31	3	7	19	5	0.004955	0.23359	11	0	2	1	6	2	0.42506085	1	7.75	10.0959681								
9	1E+07	9_143634	29	8	7	12	19	10	0.108738	1	18	1	21	0	10	11	0.92604858	1	12	10.0853501								
9	1E+07	9_145421	0	2	6	2	1	4	0.505922	1	21	3	9	0	12	5	0.30420563	1	5.375	7.00892288								
9	1E+07	9_147422	7	49	6	8	28	7	2.93E-04	0.0227	57	41	27	1	49	14	2.13E-04	0.0174485	24.5	22.0648395								
9	1E+07	9_148238	28	0	19	27	14	23	0.607375	1	1	14	0	0	8	0	0.02452223	0.7977568	11.125	12.4262108								
9	1E+07	9_149342	13	11	36	18	12	27	0.161087	1	19	0	8	9	10	9	0.99792604	1	14.25	10.633505								
9	2E+07	9_17147416	1	8	5	18	5	12	0.389128	1	19	21	1	3	20	2	0.00122165	0.0738454	9.5	8.48528137	2E+07	1.7E+07	ID=ENSP0000317362;PRCP;NM_005040;	PRCP				
9	2E+07	9_172362	68	266	122	132	167	127	0.001028	0.06498	335	201	258	118	268	188	0.01327592	0.4945372	187.5	92.2357229								
9	2E+07	9_17554897	188	65	191	154	127	173	0.440069	1	80	68	54	10	74	32	0.00109565	0.0677139	101.25	67.3938319					1.8E+07	1.8E+07	0.34205	ID=promoter_52958;
9	2E+07	9_17653991	281	189	228	354	235	291	0.826054	1	67	133	114	148	100	131	0.03257613	0.9938703	189.25	94.5179499					1.8E+07	1.8E+07	0.34205	ID=promoter_52959;
9	2E+07	9_177908	14	5	0	0	10	0	0.002838	0.14869	0	17	31	25	9	28	0.00358036	0.1770746	11.5	12.1772622								
9	2E+07	9_190948	6	24	4	7	15	6	0.065303	1	0	10	19	1	5	10	0.35527535	1	8.875	8.52622006								
9	2E+07	9_195375	88	348	121	131	218	126	8.84E-10	#######	196	280	400	250	238	325	2.94E-05	0.0032143	226.75	112.530313								
9	2E+07	9_199469	43	53	5	23	48	14	4.54E-06	0.00059	232	67	57	43	150	50	5.14E-10	1.58E-07	65.375	70.1344372								
9	2E+07	9_20502411	11	14	8	5	13	7	0.234546	1	18	5	5	1	12	3	0.09316162	1	8.375	5.60452623	2E+07	2.1E+07	ID=ENSP0000346800;CCDC81;NM_021827;	CCDC81				
9	2E+07	9_207426	12	4	31	28	8	30	0.008498	0.35909	0	7	17	15	4	16	0.01700101	0.6008545	14.25	10.9772492								
9	2E+07	9_209975	0	22	0	5	11	3	0.050503	1	0	12	7	1	6	4	0.87665919	1	5.875	7.80910641								
9	2E+07	9_213332	29	56	17	12	43	15	9.81E-05	0.00874	15	78	76	74	47	75	0.012691	0.4762983	44.625	29.3741261								

9	2E+07	9_213607	15	65	20	19	40	20	0.005812	0.26547	21	22	39	23	22	31	0.31838102	1	28	16.5356757								
9	2E+07	9_216797	24	54	13	7	39	10	1.49E-05	0.00168	24	16	28	0	20	14	0.72101732	1	20.75	16.3946856								
9	2E+07	9_217914	221	55	249	58	138	154	0.913598	1	181	48	29	18	115	24	3.16E-12	1.25E-09	107.38	93.5230109								
9	2E+07	9_219702	0	3	11	19	2	15	0.01804	0.65084	1	9	22	14	5	18	0.01516798	0.5480081	9.875	8.21909797								
9	2E+07	9_219735	28	21	15	18	25	17	0.214068	1	2	11	38	9	7	24	0.00482107	0.2211976	17.75	11.3609607								
9	2E+07	9_223949	98	132	99	177	115	138	0.983833	1	281	153	103	64	217	84	2.07E-11	7.46E-09	138.38	67.6671212								
9	2E+07	9_22977550	26	50	29	26	38	28	0.169685	1	54	50	42	20	52	31	0.14604732	1	37.125	13.3463478	2E+07	2.3E+07	ID=ENSP0000366404-D57;;NM_006510;					
9	2E+07	9_241055	53	105	61	37	79	49	0.001748	0.10098	107	64	130	89	86	110	0.08029188	1	80.75	31.9049929								
9	2E+07	9_249016	0	38	3	6	19	5	0.004955	0.23359	10	0	0	17	5	9	0.48000906	1	9.25	13.0575649								
9	3E+07	9_251490	6	37	11	22	22	17	0.428533	1	30	13	7	3	22	5	0.00878788	0.3541514	16.125	12.2874791								
9	3E+07	9_253805	28	28	19	21	28	20	0.231138	1	46	21	54	12	34	33	0.98250989	1	28.625	14.3022226								
9	3E+07	9_254602	11	8	24	6	10	15	0.833288	1	8	30	6	15	19	11	0.45547225	1	13.5	8.9761589								
9	3E+07	9_254880	0	6	6	8	3	7	0.596562	1	1	11	10	1	6	6	0.99142475	1	5.375	4.27409138								
9	3E+07	9_25696067	2	11	33	7	7	20	0.111433	1	15	1	12	1	8	7	0.9937864	1	10.25	10.6469311					2.6E+07	2.6E+07	0.12282	ID=promoter_53021;	
9	3E+07	9_257579	13	0	0	39	7	20	0.111433	1	33	10	33	0	22	17	0.8526593	1	16	16.5615734								
9	3E+07	9_260862	76	26	15	27	51	21	1.33E-04	0.01141	52	45	49	15	49	32	0.30115968	1	38.125	21.155462								
9	3E+07	9_263794	23	3	24	19	13	22	0.573969	1	0	9	0	1	5	1	0.30486099	1	9.875	10.5348063								
9	3E+07	9_26487667	288	109	200	188	199	194	0.185385	1	145	142	105	41	144	73	1.12E-04	0.0100767	152.25	74.2808378	3E+07	2.7E+07	ID=ENSP0000316681;KIAA1731;NM_033395;	KIAA1731	2.6E+07	2.6E+07	0.41561	ID=promoter_53042;	
9	3E+07	9_266342	21	42	8	14	32	11	9.30E-04	0.05947	76	14	21	68	45	45	0.93745013	1	33	26.1588554								
9	3E+07	9_267882	153	26	101	97	90	99	0.907345	1	84	67	125	19	76	72	0.99134247	1	84	45.9471746								
9	3E+07	9_268187	0	25	8	1	13	5	0.083134	1	17	11	1	18	14	10	0.81923661	1	10.125	9.29573635								
9	3E+07	9_27041831	98	24	41	50	61	46	0.07422	1	27	26	26	1	27	14	0.20179887	1	36.625	28.5654011					2.7E+07	2.7E+07	0.17784	ID=promoter_53060;	
9	3E+07	9_27071979	234	135	240	178	185	209	0.933157	1	55	50	70	69	53	70	0.14818593	1	128.88	79.8041129	3E+07	2.7E+07	ID=ENSP0000299001;PIWIL4;NM_152431;	PIWIL4					
9	3E+07	9_27258745	9	28	4	1	19	3	7.41E-04	0.04898	0	13	5	19	7	12	0.42229866	1	9.875	9.68706796	3E+07	2.7E+07	ID=ENSP0000320968;AMOTL1;;	AMOTL1					
9	3E+07	9_27260050	81	35	56	48	58	52	0.369374	1	29	10	12	1	20	7	0.06990458	1	34	26.8966275	3E+07	2.7E+07	ID=ENSP0000320968;AMOTL1;;	AMOTL1					
9	3E+07	9_27576231	44	0	13	10	22	12	0.091688	1	0	5	5	0	3	3	0.99570314	1	9.625	14.7060483					2.8E+07	2.8E+07	0.1967	ID=promoter_53092;	
9	3E+07	9_276977	15	24	26	16	20	21	0.939065	1	58	50	23	1	54	12	7.23E-06	0.0009506	26.625	18.7307044								
9	3E+07	9_277705	6	29	28	10	18	19	0.949854	1	26	18	3	24	22	14	0.54188925	1	18	10.3785493								
9	3E+07	9_281012	11	31	6	15	21	11	0.084122	1	39	29	2	2	34	2	2.14E-06	0.0003293	16.875	14.3171975								
9	3E+07	9_287123	10	56	6	17	33	12	0.001163	0.07164	17	41	37	13	29	25	0.96515949	1	24.625	17.7999799								
9	3E+07	9_316442	19	77	19	12	48	16	2.00E-05	0.00219	61	63	31	47	62	39	0.16153279	1	41.125	24.27484								
9	3E+07	9_32025971	91	27	61	69	59	65	0.941045	1	29	56	50	3	43	27	0.2794817	1	48.25	27.6547593					3.2E+07	3.2E+07	0.70576	ID=promoter_53109;	
9	3E+07	9_327800	10	19	5	21	15	13	0.725173	1	19	14	16	1	17	9	0.38755874	1	13.125	7.1999504								
9	3E+07	9_330320	15	19	22	1	17	12	0.395844	1	0	18	18	0	9	9	0.98716474	1	11.625	9.54594155								
9	3E+07	9_33368909	333	153	312	360	243	336	0.147662	1	214	159	158	57	187	108	4.06E-04	0.0298045	218.25	106.548111					3.3E+07	3.3E+07	0.23218	ID=promoter_53116;	
9	3E+07	9_334617	5	68	20	21	37	21	0.025035	0.84326	12	36	41	17	24	29	0.62889415	1	27.5	20.1777813								
9	3E+07	9_337123	50	41	34	56	46	45	0.686795	1	5	38	28	9	22	19	0.97472654	1	32.625	18.094494								
9	3E+07	9_339393	5	43	9	16	24	13	0.071032	1	0	12	11	16	6	14	0.1466131	1	14	12.8951818								
9	3E+07	9_339506	0	10	4	6	5	5	0.968625	1	33	6	27	9	20	18	0.99585638	1	11.875	11.7039371								
9	3E+07	9_346451	11	18	12	0	15	6	0.065303	1	63	15	9	0	39	5	6.81E-06	0.0009075	16	20.0570615								
9	4E+07	9_35480990	495	147	365	416	321	391	0.882003	1	215	220	253	111	218	182	0.58012343	1	277.75	134.541284					3.5E+07	3.5E+07	0.33254	ID=promoter_53122;	
9	4E+07	9_35618133	1235	1047	1811	2651	1141	2231	1.79E-44	#######	1377	952	2046	1370	1165	1708	7.02E-33	7.96E-30	1561.1	572.901746					3.6E+07	3.6E+07	0.1546	ID=promoter_53124;	

9	4E+07	9_36186224	135	29	35	39	82	37	3.56E-06	0.00048	46	59	61	52	53	57	0.73828082	1	57	33.4706695	4E+07	3.6E+07	ID=ENSP0000265843;EXPH5;NM_015065;	EXPH5				
9	4E+07	9_36212516	110	140	106	80	125	93	0.003618	0.18171	86	139	139	68	113	104	0.99858066	1	108.5	28.8345031	4E+07	3.6E+07	ID=ENSP0000265843;EXPH5;NM_015065;	EXPH5	3.6E+07	3.6E+07	0.39644	ID=promoter_53130;
9	4E+07	9_363003	11	14	18	10	13	14	0.975695	1	18	1	23	4	10	14	0.60490119	1	12.375	7.4246212								
9	4E+07	9_365162	17	99	26	36	58	31	0.001405	0.08377	23	25	65	37	24	51	0.0026203	0.1364955	41	27.676705								
9	4E+07	9_372024	21	0	8	4	11	6	0.3028	1	27	7	10	3	17	7	0.1782776	1	10	9.31971796								
9	4E+07	9_372878	15	26	11	24	21	18	0.61832	1	15	10	33	18	13	26	0.0678374	1	19	8								
9	4E+07	9_373684	33	110	33	46	72	40	5.42E-04	0.03781	45	63	75	29	54	52	0.98103932	1	54.25	27.5408787								
9	4E+07	9_375151	7	20	7	6	14	7	0.165008	1	23	3	17	16	13	17	0.64388579	1	12.375	7.48212155								
9	4E+07	9_379904	15	23	1	10	19	6	0.010861	0.43721	6	15	15	3	11	9	0.96206785	1	11	7.3872477								
9	4E+07	9_380850	15	11	32	25	13	29	0.146708	1	69	112	57	37	91	47	0.00427313	0.2005027	44.75	33.5889012								
9	4E+07	9_381017	4	15	0	8	10	4	0.162164	1	1	2	10	1	2	6	0.31385293	1	5.125	5.35690476								
9	4E+07	9_381315	12	10	8	12	11	10	0.842816	1	0	14	10	0	7	5	0.9051169	1	8.25	5.3917927								
9	4E+07	9_38706188	475	163	309	263	319	286	0.004179	0.20323	160	87	120	64	124	92	0.2683236	1	205.13	137.268702					3.9E+07	3.9E+07	0.21296	ID=promoter_53161;
9	4E+07	9_389965	7	6	6	4	7	5	0.693374	1	1	24	21	22	13	22	0.2175897	1	11.375	9.28804916								
9	4E+07	9_390056	38	24	80	27	31	54	0.210087	1	18	15	2	0	17	1	0.00146248	0.085933	25.5	25.3546276								
9	4E+07	9_395305	0	14	7	25	7	16	0.324814	1	1	28	15	2	15	9	0.58227053	1	11.5	10.8627805								
9	4E+07	9_397380	23	7	11	1	15	6	0.065303	1	75	44	26	36	60	31	0.02750657	0.8747143	27.875	23.9549428								
9	4E+07	9_399600	2	4	12	0	3	6	0.747087	1	20	6	16	0	13	8	0.65599869	1	7.5	7.61577311								
9	4E+07	9_402361	9	22	25	10	16	18	0.992646	1	10	36	0	5	23	3	9.46E-04	0.0600857	14.625	11.9515391								
9	4E+07	9_404264	20	30	11	6	25	9	0.005635	0.25889	0	21	9	0	11	5	0.40227598	1	12.125	10.7096419								
9	4E+07	9_406080	11	9	16	6	10	11	0.989255	1	1	3	15	4	2	10	0.05067035	1	8.125	5.56616051								
9	4E+07	9_406174	35	9	8	10	22	9	0.020582	0.72012	0	0	12	1	0	7	0.02292696	0.7568963	9.375	11.4134945								
9	4E+07	9_406431	2	29	26	20	16	23	0.822157	1	27	5	46	10	16	28	0.11940283	1	20.625	14.5792171								
9	4E+07	9_417840	30	37	25	13	34	19	0.029997	0.97543	19	29	44	50	24	47	0.00952662	0.3792134	30.875	12.3916735								
9	4E+07	9_418717	9	21	8	9	15	9	0.269309	1	0	23	1	26	12	14	0.84238319	1	12.125	9.9776536								
9	4E+07	9_421586	117	80	69	54	99	62	4.28E-04	0.03099	122	61	32	13	92	23	1.25E-08	3.04E-06	68.5	37.8455508								
9	4E+07	9_422710	32	34	38	43	33	41	0.970179	1	322	393	366	335	358	351	0.75725883	1	195.38	170.886711								
9	4E+07	9_42363271	264	171	193	223	218	208	0.102578	1	214	35	128	27	125	78	0.02209391	0.7420918	156.88	87.1541123					4.2E+07	4.2E+07	0.19255	ID=promoter_53217;
9	4E+07	9_426069	9	25	2	9	17	6	0.027279	0.89953	3	14	16	23	9	20	0.07989336	1	12.625	8.50105036								
9	4E+07	9_428039	7	2	24	29	5	27	0.002605	0.1394	14	23	12	1	19	7	0.09662636	1	14	10.5017005								
9	4E+07	9_428102	4	14	10	20	9	15	0.704209	1	0	16	20	17	8	19	0.06853691	1	12.625	7.38603895								
9	4E+07	9_429130	0	8	11	28	4	20	0.015573	0.58011	46	26	12	1	36	7	1.63E-04	0.0138451	16.5	15.7116881								
9	4E+07	9_43288298	43	27	25	25	35	25	0.160265	1	67	23	10	2	45	6	1.38E-06	0.0002234	27.75	19.9624648	4E+07	4.3E+07	ID=ENSP0000325917;PCSK7;NM_004716;	PCSK7				
9	4E+07	9_43303990	17	24	14	14	21	14	0.252399	1	29	8	1	1	19	1	5.85E-04	0.0402157	13.5	10.0427657	4E+07	4.3E+07	ID=ENSP0000278951;SIDT2;;	SIDT2				
9	4E+07	9_43662700	22	27	22	37	25	30	0.996463	1	5	9	12	15	7	14	0.23485029	1	18.625	10.4599577	4E+07	4.4E+07	ID=ENSP0000278935-D2;CEP164;NM_014956;	CEP164				
9	4E+07	9_43855876	16	45	21	13	31	17	0.035825	1	10	26	9	16	18	13	0.78939737	1	19.5	11.7229933					4.4E+07	4.4E+07	0.10728	ID=promoter_53248;
9	4E+07	9_439608	282	118	408	381	200	395	9.86E-09	#######	72	69	71	29	71	50	0.32772295	1	178.75	153.663594								
9	4E+07	9_439911	27	38	25	11	33	18	0.027763	0.91318	41	20	8	5	31	7	0.00122211	0.0738454	21.875	13.4210655								
9	4E+07	9_43991853	11	21	9	1	16	5	0.021336	0.74103	19	37	19	0	28	10	0.02694098	0.8576836	14.625	12.0941249					4.4E+07	4.4E+07	0.10481	ID=promoter_53253;
9	4E+07	9_440477	41	17	14	8	29	11	0.003535	0.17827	21	16	16	2	19	9	0.23949138	1	16.875	11.3947043								
9	4E+07	9_442123	2	9	4	4	6	4	0.674789	1	2	0	14	28	1	21	5.13E-05	0.0051875	7.875	9.29573635								
9	4E+07	9_444633	1	14	1	1	8	1	0.03583	1	1	12	14	3	7	9	0.80856893	1	5.875	6.24356812								
9	4E+07	9_444845	16	21	18	27	19	23	0.994841	1	76	57	24	1	67	13	8.67E-08	1.83E-05	30	24.3545244								
9	4E+07	9_447382	21	35	26	30	28	28	0.83651	1	59	19	31	18	39	25	0.35043112	1	29.875	13.2496631								
9	4E+07	9_44832819	964	679	1101	723	822	912	0.512201	1	673	426	868	879	550	874	1.55E-22	1.22E-19	789.13	208.392101					4.5E+07	4.5E+07	0.41322	ID=promoter_53274;

9	4E+07	9_44842714	0	0	5	6	0	6	0.077487	1	26	58	11	0	42	6	5.09E-06	0.0007008	13.25	20.0624026	4E+07	4.5E+07	ID=ENSP00000339048;SLC37A4;;	SLC37A4				
9	5E+07	9_450430	5	23	7	5	14	6	0.098789	1	9	44	32	47	27	40	0.16464486	1	21.5	17.6473389								
9	5E+07	9_451247	158	49	85	108	104	97	0.266036	1	21	13	0	36	17	18	0.92557631	1	58.75	54.1789363								
9	5E+07	9_45215897	3262	1322	3494	2161	2292	2828	0.197449	1	1328	1193	897	502	1261	700	5.32E-27	5.10E-24	1769.9	1098.70514					4.5E+07	4.5E+07	0.28914	ID=promoter_53298;
9	5E+07	9_454966	34	26	4	14	30	9	5.55E-04	0.03862	36	24	5	1	30	3	4.27E-05	0.004447	18	13.8873015								
9	5E+07	9_45593440	11	40	31	39	26	35	0.867873	1	16	17	11	5	17	8	0.27279187	1	21.25	13.5303891					4.6E+07	4.6E+07	0.14962	ID=promoter_53311;
9	5E+07	9_45992704	19	45	51	20	32	36	0.985347	1	28	28	30	22	28	26	0.99998173	1	30.375	11.6978325	5E+07	4.6E+07	ID=ENSP00000343129;TRIM29;NM_012101;	TRIM29				
9	5E+07	9_460193	1	10	10	9	6	10	0.79153	1	0	0	9	3	0	6	0.0393172	1	5.25	4.65218843								
9	5E+07	9_464113	60	30	16	61	45	39	0.381351	1	20	18	19	0	19	10	0.34022508	1	28	21.6794834								
9	5E+07	9_46703423	24	32	8	19	28	14	0.027228	0.89953	25	18	46	4	22	25	0.78399411	1	22	13.2772631	5E+07	4.7E+07	ID=ENSP00000278723;GRIK4;NM_014619;	GRIK4				
9	5E+07	9_468575	12	15	7	2	14	5	0.053576	1	0	3	6	0	2	3	0.86792893	1	5.625	5.52752851								
9	5E+07	9_469758	8	16	10	10	12	10	0.724827	1	0	11	13	0	6	7	0.91781435	1	8.5	5.75698334								
9	5E+07	9_47188305	37	139	44	46	88	45	1.91E-05	0.00211	123	151	159	29	137	94	0.07859753	1	91	56.7525959	5E+07	4.7E+07	ID=ENSP00000260197;SORL1;NM_003105;	SORL1				
9	5E+07	9_47190280	38	6	20	8	22	14	0.192025	1	0	0	0	32	0	16	1.79E-04	0.0149638	13	15.1939838	5E+07	4.7E+07	ID=ENSP00000260197;SORL1;NM_003105;	SORL1				
9	5E+07	9_47218232	135	39	125	58	87	92	0.786325	1	59	47	26	4	53	15	9.70E-05	0.0089702	61.625	45.8816412					4.7E+07	4.7E+07	0.09526	ID=promoter_53336;
9	5E+07	9_479855	4	64	15	5	34	10	1.75E-04	0.01448	43	46	17	13	45	15	0.00163449	0.0924179	25.875	22.1323132								
9	5E+07	9_482704	16	2	10	31	9	21	0.212308	1	37	5	7	5	21	6	0.02669383	0.8538132	14.125	13.0541455								
9	5E+07	9_48298599	9	25	25	0	17	13	0.50439	1	12	2	24	7	7	16	0.11989665	1	13	10.3647755	5E+07	4.8E+07	ID=ENSP00000284273;UBASH3B;NM_032873;	UBASH3B				
9	5E+07	9_483262	0	19	14	40	10	27	0.068117	1	43	21	18	3	32	11	0.01265194	0.4751394	19.75	15.397124								
9	5E+07	9_48334479	22	21	14	23	22	19	0.617881	1	13	3	13	14	8	14	0.34554715	1	15.375	6.56696277	5E+07	4.8E+07	ID=ENSP00000227348;CRTAM;NM_019604;	CRTAM				
9	5E+07	9_48424471	14	17	1	0	16	1	3.65E-04	0.02739	16	0	9	0	8	5	0.78486764	1	7.125	7.71709605	5E+07	4.8E+07	ID=ENSP00000227349;C11orf63;NM_024806;	C11orf63				
9	5E+07	9_48452518	4464	4024	5756	6968	4244	6362	3.68E-34	#######	3421	2981	6871	1919	3201	4395	1.44E-63	3.07E-60	4550.5	1837.34412					4.8E+07	4.8E+07	0.5452	ID=promoter_53351;
9	5E+07	9_498284	74	40	66	53	57	60	0.842506	1	0	74	43	24	37	34	0.99931372	1	46.75	25.7945177								
9	5E+07	9_50434529	400	203	300	263	302	282	0.022185	0.76346	134	95	92	40	115	66	0.00734117	0.308928	190.88	123.031282					5E+07	5E+07	0.39486	ID=promoter_53381;
9	5E+07	9_50486154	148	53	115	90	101	103	0.606547	1	37	31	43	30	34	37	0.79599102	1	68.375	44.2652314					5E+07	5E+07	0.58177	ID=promoter_53384;
9	5E+07	9_50508282	589	300	380	425	445	403	8.13E-04	0.05337	173	229	200	61	201	131	0.0069124	0.294895	294.63	166.306891					5.1E+07	5.1E+07	0.19238	ID=promoter_53386;
9	5E+07	9_50554843	26	11	65	44	19	55	0.002102	0.11683	0	13	2	0	7	1	0.13113995	1	20.125	23.5762441	5E+07	5.1E+07	ID=ENSP00000304945;ROBO4;NM_019055;	ROBO4				
9	5E+07	9_511501	226	61	91	81	144	86	2.94E-06	0.0004	64	28	25	16	46	21	0.02302622	0.7594708	74	67.179929								

9	5E+07	9_51701763	1142	479	826	613	811	720	3.41E-07	#######	253	277	237	226	265	232	0.81653048	1	506.63	335.150686	5E+07	5.2E+07	ID=ENSP0000328023;SRPR;NM_003139;	SRPR	5.2E+07	5.2E+07	0.36679	ID=promoter_53410;
9	5E+07	9_51768365	65	92	80	43	79	62	0.058276	1	46	53	63	60	50	62	0.30754852	1	62.75	16.6110978	5E+07	5.2E+07	ID=ENSP0000263579;DCPS;NM_014026;	DCPS	5.2E+07	5.2E+07	0.30631	ID=promoter_53413;
9	5E+07	9_519702	3	10	17	5	7	11	0.831726	1	28	10	36	27	19	32	0.1120603	1	17	12.0712172								
9	5E+07	9_522795	44	0	27	25	22	26	0.999662	1	0	0	0	0	0	0	1	1	12	17.4765149								
9	5E+07	9_524074	15	12	23	12	14	18	0.967347	1	11	12	18	0	12	9	0.89054267	1	12.875	6.59951297								
9	5E+07	9_525287	97	65	77	65	81	71	0.198572	1	3	9	15	1	6	8	0.79537884	1	41.5	38.4075885								
9	5E+07	9_528263	8	24	14	9	16	12	0.497978	1	1	29	23	19	15	21	0.47046428	1	15.875	9.50845488								
9	5E+07	9_52854268	30	41	23	24	36	24	0.094407	1	56	61	74	34	59	54	0.99769331	1	42.875	18.7802138					5.3E+07	5.3E+07	0.11954	ID=promoter_53428;
9	5E+07	9_529721	24	42	22	14	33	18	0.027763	0.91318	20	19	12	18	20	15	0.82431124	1	21.375	9.21082438								
9	5E+07	9_534843	24	73	12	19	49	16	1.26E-05	0.00147	27	25	16	1	26	9	0.02974521	0.9232309	24.625	21.2800208								
9	5E+07	9_535173	20	7	12	13	14	13	0.831264	1	48	23	19	6	36	13	0.01036973	0.4053038	18.5	13.3844259								
9	5E+07	9_535935	5	0	14	0	3	7	0.596562	1	58	0	3	18	29	11	0.03330399	1	12.25	19.7031542								
9	5E+07	9_538225	12	8	2	13	10	8	0.719353	1	0	6	9	2	3	6	0.53745121	1	6.5	4.84030696								
9	5E+07	9_540674	7	14	491	549	11	520	2.23E-90	#######	23	21	666	757	22	712	2.28E-153	1.48E-149	316	329.816832								
9	5E+07	9_541010	15	26	12	26	21	19	0.713172	1	9	10	17	2	10	10	0.98574881	1	14.625	8.31414457								
9	5E+07	9_541687	16	29	22	14	23	18	0.432563	1	3	1	11	1	2	6	0.31385293	1	12.125	10.2321273								
9	5E+07	9_542955	10	2	14	5	6	10	0.79153	1	1	2	8	0	2	4	0.6610374	1	5.25	4.97852531								
9	5E+07	9_543630	41	63	285	285	52	285	2.72E-28	#######	62	47	109	12	55	61	0.62742724	1	113	109.561464								
9	5E+07	9_54379155	4140	1893	5028	3992	3017	4510	6.33E-24	#######	2256	1539	1662	786	1898	1224	3.04E-22	2.36E-19	2662	1516.01178					5.4E+07	5.4E+07	0.25647	ID=promoter_53446;
9	5E+07	9_543877	377	100	292	189	239	241	0.253059	1	64	25	19	29	45	24	0.08598552	1	136.88	135.835444								
9	5E+07	9_54631471	66	90	178	169	78	174	9.97E-06	0.0012	43	34	76	80	39	78	3.12E-04	0.0239121	92	53.6789397					5.5E+07	5.5E+07	0.78168	ID=promoter_53450;
9	5E+07	9_54734607	35	172	41	67	104	54	3.84E-06	0.00051	126	87	93	27	107	60	0.00700462	0.2971393	81	49.7335759	5E+07	5.5E+07	ID=ENSP0000281437;BARX2;NM_003658;	BARX2				
9	5E+07	9_54771980	12	7	6	5	10	6	0.41703	1	7	0	0	11	4	6	0.75330063	1	6	4.40778532	5E+07	5.5E+07	ID=ENSP0000281437;BARX2;NM_003658;	BARX2				
9	5E+07	9_548834	15	21	9	6	18	8	0.059652	1	39	2	12	1	21	7	0.05007015	1	13.125	12.3916735								
9	6E+07	9_553267	13	262	50	54	138	52	1.73E-12	#######	143	135	389	127	139	258	1.45E-10	4.74E-08	146.63	124.419725								
9	6E+07	9_555150	111	67	68	54	89	61	0.004743	0.22508	7	15	21	1	11	11	0.98433491	1	43	38.3107594								
9	6E+07	9_55557150	0	13	16	8	7	12	0.724589	1	12	32	14	2	22	8	0.06315916	1	12.125	9.86244681	6E+07	5.6E+07	ID=ENSP0000282849-D2;ADAMTS18;NM_199355;	ADAMTS18				
9	6E+07	9_556307	1830	1697	2242	3040	1764	2641	1.79E-14	#######	1429	1179	3026	673	1304	1850	2.97E-31	3.26E-28	1889.5	843.197825								
9	6E+07	9_558251	9	21	3	0	15	2	0.002248	0.12273	0	6	18	1	3	10	0.11508955	1	7.25	8.20713975								
9	6E+07	9_560728	12	28	31	12	20	22	0.978625	1	6	2	16	1	4	9	0.31291743	1	13.5	11.1611571								
9	6E+07	9_561462	15	4	3	1	10	2	0.03464	1	15	6	8	0	11	4	0.25131486	1	6.5	5.83095189								
9	6E+07	9_562493	12	52	16	7	32	12	0.001806	0.10314	0	35	20	1	18	11	0.54801163	1	17.875	17.8200329								
9	6E+07	9_563543	5	49	47	48	27	48	0.219655	1	65	29	36	33	47	35	0.61680793	1	39	17.8165252								
9	6E+07	9_566355	35	15	26	13	25	20	0.43889	1	41	29	55	10	35	33	0.99757329	1	28	15.4457576								
9	6E+07	9_568216	32	64	59	43	48	51	0.886006	1	64	21	39	59	43	49	0.61296897	1	47.625	16.2299327								
9	6E+07	9_568682	24	24	48	65	24	57	0.012526	0.4876	17	23	21	4	20	13	0.60545249	1	28.25	19.1665631								
9	6E+07	9_569990	6	28	0	0	17	0	4.68E-05	0.00457	21	27	0	0	24	0	1.47E-05	0.0017742	10.25	12.8146122								
9	6E+07	9_570543	0	7	9	7	4	8	0.677894	1	7	18	13	2	13	8	0.65599869	1	7.875	5.7180791								
9	6E+07	9_57077456	6	8	0	0	7	0	0.016484	0.60584	21	34	6	12	28	9	0.01512239	0.5480081	10.875	11.5317884					5.7E+07	5.7E+07	0.14334	ID=promoter_53494;
9	6E+07	9_57096480	1	29	3	21	15	12	0.610117	1	3	9	13	0	6	7	0.91781435	1	9.875	10.4940459					5.7E+07	5.7E+07	0.1113	ID=promoter_53496;
9	6E+07	9_572263	66	11	61	36	39	49	0.949978	1	16	8	43	0	12	22	0.15316566	1	30.125	25.0795164								
9	6E+07	9_576203	165	90	118	88	128	103	0.016567	0.6084	267	144	126	37	206	82	2.20E-10	7.04E-08	129.38	67.9914648								
9	6E+07	9_581709	11	13	11	8	12	10	0.724827	1	1	12	0	0	7	0	0.03898208	1	7	5.70713839								
9	6E+07	9_581762	44	47	24	33	46	29	0.02902	0.94901	0	10	20	1	5	11	0.25523295	1	22.375	18.1496753								
9	6E+07	9_584867	4	3	1	14	4	8	0.677894	1	6	7	15	0	7	8	0.92121674	1	6.25	5.59974489								
9	6E+07	9_585333	5	9	5	9	7	7	0.956352	1	17	0	9	1	9	5	0.6500913	1	6.875	5.40997755								

9	6E+07	9_589175	38	9	3	8	24	6	9.38E-04	0.05976	0	9	0	0	5	0	0.09850879	1	8.375	12.6371052								
9	6E+07	9_58976683	50	45	42	26	48	34	0.075354	1	41	35	30	35	38	33	0.96300436	1	38	7.96420564					5.9E+07	5.9E+07	0.56181	ID=promoter_53507;
9	6E+07	9_59274630	23	9	26	8	16	17	0.960459	1	0	17	12	0	9	6	0.8208167	1	11.875	9.67230952	6E+07	5.9E+07	ID=ENSP0000374531;GLB1L2;;	GLB1L2				
9	6E+07	9_59275419	100	36	41	62	68	52	0.064724	1	31	23	26	29	27	28	0.91714458	1	43.5	25.8843582	6E+07	5.9E+07	ID=ENSP0000374531;GLB1L2;;	GLB1L2				
9	6E+07	9_598453	71	10	65	97	41	81	0.022731	0.78018	59	30	41	52	45	47	0.84906089	1	53.125	26.6267186								
9	6E+07	9_60027860	126	125	104	108	126	106	0.04039	1	86	80	64	24	83	44	0.00995874	0.3918263	89.625	34.200825					6E+07	6E+07	0.30978	ID=promoter_53518;
9	6E+07	9_617148	91	54	3	8	73	6	1.39E-15	#######	7	15	2	2	11	2	0.06154634	1	22.75	32.5389876								
9	6E+07	9_619067	0	56	5	0	28	3	4.80E-06	0.00062	0	0	0	0	0	0	1	1	7.625	19.6246019								
9	6E+07	9_620299	3	17	6	0	10	3	0.082185	1	20	0	1	1	10	1	0.03495212	1	6	8								
9	6E+07	9_623774	132	112	127	87	122	107	0.088396	1	29	23	44	2	26	23	0.98669459	1	69.5	51.177562								
9	6E+07	9_62483821	54	1	21	12	28	17	0.092775	1	17	12	21	11	15	16	0.92652531	1	18.625	15.6838179	6E+07	6.3E+07	ID=ENSP0000356159;PLEKHA6;;	PLEKHA6	6.2E+07	6.2E+07	0.15774	ID=promoter_53535;
9	6E+07	9_62490016	65	32	33	37	49	35	0.07705	1	11	35	25	12	23	19	0.93310412	1	31.25	16.9431824	6E+07	6.3E+07	ID=ENSP0000356159;PLEKHA6;;	PLEKHA6				
9	6E+07	9_62686139	1	1	6	0	1	3	0.700657	1	1	26	15	4	14	10	0.81923661	1	6.75	9.19238816	6E+07	6.3E+07	ID=ENSP0000356155;PIK3C2B;NM_002646;	PIK3C2B				
9	6E+07	9_62692657	36	44	34	25	40	30	0.174995	1	17	3	50	7	10	29	0.00459313	0.2126524	27	17.0209954	6E+07	6.3E+07	ID=ENSP0000356155;PIK3C2B;NM_002646;	PIK3C2B				
9	6E+07	9_633073	26	19	13	16	23	15	0.200003	1	1	26	15	17	14	16	0.84864027	1	16.625	7.94512429								
9	6E+07	9_633146	217	100	96	106	159	101	6.24E-06	0.00079	21	8	7	16	15	12	0.93002578	1	71.375	73.2860102								
9	6E+07	9_63318361	252	118	199	266	185	233	0.770141	1	91	79	41	41	85	41	0.0022077	0.1194024	135.88	91.0046113					6.3E+07	6.3E+07	0.22724	ID=promoter_53549;
9	6E+07	9_634627	41	58	56	35	50	46	0.491498	1	50	119	85	51	85	68	0.66293597	1	61.875	27.4248974								
9	6E+07	9_63852788	31	38	14	23	35	19	0.02151	0.74463	17	19	33	13	18	23	0.59196476	1	23.5	9.41123948	6E+07	6.4E+07	ID=ENSP0000265727;ADAM22;NM_021723;	ADAM22				
9	6E+07	9_638755	2	24	12	5	13	9	0.46931	1	83	9	28	0	46	14	6.26E-04	0.0425022	20.375	27.2078639								
9	7E+07	9_650642	0	6	11	0	3	6	0.747087	1	0	12	15	0	6	8	0.79537884	1	5.5	6.36957052								
9	7E+07	9_661906	16	20	11	11	18	11	0.22238	1	32	1	48	1	17	25	0.33405521	1	17.5	15.9373775								
9	7E+07	9_667485	29	2	24	20	16	22	0.889379	1	11	16	32	18	14	25	0.13553211	1	19	9.69535971								
9	7E+07	9_679938	23	67	21	14	45	18	2.78E-04	0.02161	61	51	15	4	56	10	5.82E-07	0.0001023	32	23.9821362								
9	7E+07	9_690963	7	0	9	9	4	9	0.54411	1	0	0	18	40	0	29	1.61E-07	3.22E-05	10.375	13.4900757								
9	7E+07	9_703301	15	28	5	1	22	3	1.41E-04	0.01198	63	24	40	19	44	30	0.42803716	1	24.375	19.9566494								
9	7E+07	9_707721	15	24	26	37	20	32	0.549915	1	36	0	14	14	18	14	0.88543974	1	20.75	12.4756906								
9	7E+07	9_71649160	1829	1146	1977	1468	1488	1723	0.935807	1	1135	895	1093	493	1015	793	0.00145934	0.085933	1254.5	486.811198					7.2E+07	7.2E+07	0.2681	ID=promoter_53599;
9	7E+07	9_728071	21	59	18	25	40	22	0.013995	0.53231	40	32	65	59	36	62	0.01099831	0.424822	39.875	18.8257992								
9	8E+07	9_760020	116	58	66	49	87	58	0.003334	0.17061	19	0	19	1	10	10	0.98574881	1	41	39.3736678								
9	8E+07	9_762235	603	297	835	1180	450	1008	5.30E-30	#######	908	537	130	389	723	260	7.84E-41	1.19E-37	609.88	347.833473								
9	8E+07	9_763003	502	238	374	424	370	399	0.507239	1	195	192	235	31	194	133	0.0267899	0.8546242	273.88	150.861751								
9	8E+07	9_763380	219	99	74	75	159	75	1.46E-10	#######	459	407	193	95	433	144	1.26E-27	1.25E-24	202.63	152.602132								
9	8E+07	9_765304	0	21	23	10	11	17	0.774447	1	24	8	24	27	16	26	0.20160145	1	17.125	9.77515071								
9	8E+07	9_767130	27	15	49	62	21	56	0.004308	0.20804	57	30	24	7	44	16	0.00398908	0.1893524	33.875	19.9745374								
9	8E+07	9_769481	37	119	21	43	78	32	1.11E-06	0.00016	100	56	82	34	78	58	0.4439538	1	61.5	35.0061219								
9	8E+07	9_777922	28	75	30	35	52	33	0.019645	0.69635	158	67	70	58	113	64	0.0063361	0.2743971	65.125	41.9538437								
9	8E+07	9_781503	18	12	4	22	15	13	0.725173	1	2	1	11	26	2	19	5.26E-04	0.0367435	12	9.3960478								
9	8E+07	9_78269693	186	41	142	101	114	122	0.780387	1	0	34	0	0	17	0	3.78E-04	0.0279647	63	71.759519	8E+07	7.8E+07	ID=ENSP0000385686;ETV1;;	ETV1				
9	8E+07	9_784978	17	28	9	6	23	8	0.007066	0.30636	64	34	15	11	49	13	1.07E-04	0.0097311	23	19.1087116								

9	8E+07	9_79532487	675	211	617	421	443	519	0.99983	1	489	346	507	172	418	340	0.20074747	1	429.75	179.708295					8E+07	8E+07	0.33712	ID=promoter_53625;
9	8E+07	9_808131	15	29	26	6	22	16	0.340123	1	27	62	42	66	45	54	0.43957352	1	34.125	21.2296121								
9	8E+07	9_815258	2	10	2	1	6	2	0.258649	1	0	4	13	9	2	11	0.03110435	0.956324	5.125	4.85320218								
9	8E+07	9_823133	2	50	3	26	26	15	0.082345	1	28	29	3	6	29	5	5.02E-04	0.0355894	18.375	17.5575584								
9	8E+07	9_833247	2	12	7	1	7	4	0.503048	1	21	9	9	2	15	6	0.20085144	1	7.875	6.64266513								
9	8E+07	9_838659	95	26	118	64	61	91	0.346143	1	26	3	0	0	15	0	9.56E-04	0.060345	41.5	45.7133928								
9	8E+07	9_843207	12	36	19	10	24	15	0.151024	1	18	25	51	1	22	26	0.70257106	1	21.5	15.8294481								
9	9E+07	9_850666	6	1	6	3	4	5	0.995512	1	10	6	2	12	8	7	0.9937864	1	5.75	3.80788655								
9	9E+07	9_852193	132	105	41	50	119	46	1.49E-10	#######	63	141	48	18	102	33	2.66E-07	5.08E-05	74.75	45.3675466								
9	9E+07	9_85307941	86	65	119	105	76	112	0.305918	1	100	67	54	30	84	42	0.00405376	0.1922949	78.25	29.5864351	9E+07	8.5E+07	ID=ENSP0000343656;RAPGEF5;NM_012294;	RAPGEF5				
9	9E+07	9_85327387	2	9	20	6	6	13	0.45043	1	1	2	17	1	2	9	0.08177705	1	7.25	7.51664819	9E+07	8.5E+07	ID=ENSP0000343656;RAPGEF5;NM_012294;	RAPGEF5				
9	9E+07	9_860616	13	23	27	17	18	22	0.991654	1	13	33	41	12	23	27	0.70626281	1	22.375	10.6494467								
9	9E+07	9_862563	3	5	1	7	4	4	0.97482	1	2	12	9	3	7	6	0.99011599	1	5.25	3.80788655								
9	9E+07	9_86415707	770	367	687	757	569	722	0.373829	1	486	374	377	122	430	250	2.46E-08	5.72E-06	492.5	228.423792					8.6E+07	8.6E+07	0.51605	ID=promoter_53681;
9	9E+07	9_865134	0	7	13	4	4	9	0.54411	1	32	0	11	18	16	15	0.99951315	1	10.625	10.6896145								
9	9E+07	9_869915	198	121	191	195	160	193	0.966309	1	192	141	96	82	167	89	1.09E-04	0.0098689	152	48.1129623								
9	9E+07	9_87356123	7	67	14	24	37	19	0.010763	0.4351	2	34	10	2	18	6	0.0767986	1	20	21.9544984	9E+07	8.7E+07	ID=ENSP0000234923-D2;KIAA1324;NM_020775;	KIAA1324				
9	9E+07	9_876333	48	42	75	61	45	68	0.41748	1	0	23	30	14	12	22	0.15316566	1	36.625	24.807473								
9	9E+07	9_88816411	687	279	413	243	483	328	1.03E-13	#######	194	205	292	79	200	186	0.99951168	1	299	183.375493					8.9E+07	8.9E+07	0.59729	ID=promoter_53695;
9	9E+07	9_890185	39	53	36	45	46	41	0.439359	1	128	36	43	90	82	67	0.74501545	1	58.75	33.0961802								
9	9E+07	9_89691190	38	17	22	33	28	28	0.83651	1	10	32	28	5	21	17	0.91718448	1	23.125	11.7161366	9E+07	9E+07	ID=ENSP0000265362;SEMA3A;NM_006080;	SEMA3A				
9	9E+07	9_897730	22	18	17	20	20	19	0.804666	1	1	24	32	2	13	17	0.64388579	1	17	10.6234243								
9	9E+07	9_905423	9	16	10	4	13	7	0.234546	1	1	3	6	1	2	4	0.6610374	1	6.25	5.1754917								
9	9E+07	9_906411	16	4	18	5	10	12	0.998584	1	10	17	13	0	14	7	0.39930956	1	10.375	6.73874723								
9	9E+07	9_906560	7	12	5	7	10	6	0.41703	1	34	1	7	2	18	5	0.0406475	1	9.375	10.5144458								
9	9E+07	9_939050	5	0	11	12	3	12	0.129494	1	18	15	6	0	17	3	0.01239331	0.4669004	8.375	6.69621428								
9	1E+08	9_95419246	348	114	261	251	231	256	0.822263	1	126	81	95	42	104	69	0.09606481	1	164.75	107.774566					9.5E+07	9.5E+07	0.53567	ID=promoter_53706;
9	1E+08	9_956741	8	14	5	8	11	7	0.438207	1	5	6	5	1	6	3	0.67473421	1	6.5	3.74165739								
9	1E+08	9_95698757	26	85	38	18	56	28	7.41E-04	0.04898	35	27	49	13	31	31	0.95647893	1	36.375	22.7026587	1E+08	9.6E+07	ID=ENSP0000285871;CCDC146;NM_020879;	CCDC146				
9	1E+08	9_959245	16	34	10	26	25	18	0.280499	1	45	30	18	12	38	15	0.01554903	0.5562856	23.875	12.0763937								
9	1E+08	9_96120259	18	24	15	6	21	11	0.084122	1	15	9	27	0	12	14	0.84238319	1	14.25	9.0356437					9.6E+07	9.6E+07	0.22103	ID=promoter_53713;
9	1E+08	9_96376732	0	16	2	11	8	7	0.850897	1	1	5	12	0	3	6	0.53745121	1	5.875	6.26640704	1E+08	9.6E+07	ID=ENSP0000368565;DNAJC2;NM_014377;	DNAJC2				
9	1E+08	9_976661	0	33	5	2	17	4	0.005755	0.26328	27	11	20	1	19	11	0.45547225	1	12.375	12.78322								
9	1E+08	9_98160831	201	151	186	154	176	170	0.194769	1	100	96	128	219	98	174	1.11E-06	0.000183	154.38	45.3901736					9.8E+07	9.8E+07	0.59235	ID=promoter_53727;
9	1E+08	9_98580200	2	15	3	0	9	2	0.058587	1	1	1	21	30	1	26	3.52E-06	0.0005061	9.125	11.4322539	1E+08	9.9E+07	ID=ENSP0000297416;ATXN7L1;NM_020725;	ATXN7L1	9.9E+07	9.9E+07	0.19533	ID=promoter_53735;

9	1E+08	9_98767110	17	30	9	25	24	17	0.274507	1	49	33	57	30	41	44	0.79631464	1	31.25	15.6821099	1E+08	9.9E+07	ID=ENSP0000326344;ATXN7L1;NM_152749;	ATXN7L1				
9	1E+08	9_993248	2	10	13	5	6	9	0.896349	1	0	5	21	7	3	14	0.01844097	0.6375434	7.875	6.7281392								
9	1E+08	9_100002447	11	7	30	34	9	32	0.00839	0.35516	0	6	30	0	3	15	0.01140593	0.4370832	14.75	14.2503133								
9	1E+08	9_100154914	1331	522	1512	1029	927	1271	0.001441	0.08558	496	371	498	101	434	300	4.55E-04	0.0327286	732.5	498.346122					1E+08	1E+08	0.49473	ID=promoter_53755;
9	1E+08	9_100652676	14	15	11	0	15	6	0.065303	1	0	0	0	2	0	1	0.58312783	1	5.25	6.81909085								
9	1E+08	9_100771809	5	14	8	11	10	10	0.938234	1	5	6	6	21	6	14	0.1466131	1	9.5	5.63154381								
9	1E+08	9_100806199	1	27	40	74	14	57	5.09E-05	0.00491	41	7	12	18	24	15	0.48349198	1	27.5	23.7667234								
9	1E+08	9_100914021	9	11	11	11	10	11	0.989255	1	13	21	26	2	17	14	0.9475482	1	13	7.3872477								
9	1E+08	9_100931479	0	4	5	8	2	7	0.358464	1	19	7	11	1	13	6	0.35360803	1	6.875	6.08129451								
9	1E+08	9_100978242	11	0	10	27	6	19	0.087167	1	1	0	15	0	1	8	0.04988758	1	8	9.73946317								
9	1E+08	9_100986851	93	64	76	68	79	72	0.299586	1	96	52	94	24	74	59	0.68809469	1	70.875	24.7584762								
9	1E+08	9_101211721	6	8	0	3	7	2	0.160905	1	38	27	15	0	33	8	0.00121322	0.0738375	12.125	13.7885822								
9	1E+08	9_102249936	4	50	25	45	27	35	0.926583	1	0	11	26	0	6	13	0.20797135	1	20.125	19.7226302								
9	1E+08	9_102590163	4	32	1	4	18	3	0.001281	0.07728	2	9	15	13	6	14	0.1466131	1	10	10.2539191								
9	1E+08	9_105911184	147	64	130	76	106	103	0.395256	1	194	57	74	19	126	47	2.52E-07	4.84E-05	95.125	56.9622369					1.1E+08	1.1E+08	0.54546	ID=promoter_53784;
9	1E+08	9_106716886	172	373	142	182	273	162	2.22E-11	#######	280	298	356	309	289	333	0.02539768	0.8230489	264	87.7121917								
9	1E+08	9_106747348	1038	455	685	559	747	622	1.83E-09	#######	341	276	221	195	309	208	0.00162619	0.0922497	471.25	285.077809					1.1E+08	1.1E+08	0.13619	ID=promoter_53791;
9	1E+08	9_108024357	351	177	294	353	264	324	0.8615	1	171	124	203	102	148	153	0.63942966	1	221.88	98.5443156					1.1E+08	1.1E+08	0.47277	ID=promoter_53799;
9	1E+08	9_108514687	137	68	147	120	103	134	0.731458	1	8	30	31	21	19	26	0.43045343	1	70.25	56.426311	1E+08	1.1E+08	ID=ENSP0000281834;TNFSF4;NM_003326;	TNFSF4				
9	1E+08	9_109082646	52	104	47	45	78	46	8.14E-04	0.05341	132	62	117	32	97	75	0.49740947	1	73.875	37.9376839								
9	1E+08	9_109965041	9	80	12	8	45	10	6.90E-07	0.00011	47	86	49	47	67	48	0.39298026	1	42.25	30.7605963								
9	1E+08	9_110040890	48	40	90	87	44	89	0.011325	0.44968	22	13	30	32	18	31	0.10487282	1	45.25	28.7091324					1.1E+08	1.1E+08	0.17722	ID=promoter_53810;
9	1E+08	9_110071096	78	30	64	88	54	76	0.591207	1	0	18	18	11	9	15	0.36943449	1	38.375	33.4063189	1E+08	1.1E+08	ID=ENSP0000375916;RABGAP1L;;	RABGAP1L				
9	1E+08	9_110251476	386	3725	432	252	2056	342	0	0	222	3206	150	153	1714	152	6.37E-260	7.00E-256	1065.8	1491.01085					1.1E+08	1.1E+08	0.26496	ID=promoter_53815;
9	1E+08	9_110325040	19	83	34	22	51	28	0.004238	0.20555	24	60	61	22	42	42	0.9414956	1	40.625	24.1065344								
9	1E+08	9_110590288	0	25	9	9	13	9	0.46931	1	13	0	12	16	7	14	0.23485029	1	10.5	8.22886904								
9	1E+08	9_111604419	32	57	12	17	45	15	3.94E-05	0.00396	51	48	15	1	50	8	1.15E-06	0.0001892	29.125	20.8767643								
9	1E+08	9_111659959	6	0	15	14	3	15	0.044084	1	0	10	1	0	5	1	0.30486099	1	5.75	6.47522752								
9	1E+08	9_111791950	2	3	1	10	3	6	0.747087	1	21	8	6	58	15	32	0.02335039	0.7680341	13.625	19.0258283								
9	1E+08	9_112373085	38	26	22	9	32	16	0.016272	0.6002	0	0	1	0	0	1	0.58312783	1	12	14.8034745								
9	1E+08	9_112570474	1	0	19	4	1	12	0.021411	0.74203	10	25	2	14	18	8	0.20901227	1	9.375	9.25723655								
9	1E+08	9_112600743	20	53	33	41	37	37	0.789862	1	43	53	41	43	48	42	0.96329274	1	40.875	10.6962944								

9	1E+08	9_112805205	21	84	15	42	53	29	0.003285	0.16835	20	64	75	40	42	58	0.14082722	1	45.125	26.5030322								
9	1E+08	9_113065438	238	116	108	106	177	107	2.52E-07	#######	3	99	15	0	51	8	7.49E-07	0.000129	85.625	79.6257989								
9	1E+08	9_113118202	0	30	13	7	15	10	0.374041	1	0	13	1	1	7	1	0.13113995	1	8.125	10.4257579								
9	1E+08	9_113304981	16	19	3	0	18	2	4.15E-04	0.03028	1	7	2	0	4	1	0.45332778	1	6	7.48331477								
9	1E+08	9_113367214	23	37	10	10	30	10	0.001158	0.07164	36	11	32	23	24	28	0.70960943	1	22.75	11.5108644	1E+08	1.1E+08	ID=ENSP00000356601;ANGPTL1;NM_004673;	ANGPTL1				
9	1E+08	9_113367969	1	1	6	10	1	8	0.109969	1	0	1	8	39	1	24	1.03E-05	0.0013049	8.25	12.980755	1E+08	1.1E+08	ID=ENSP00000356601;ANGPTL1;NM_004673;	ANGPTL1				
9	1E+08	9_113995908	12	21	7	25	17	16	0.818283	1	21	5	17	12	13	15	0.84581615	1	15	7.15142343								
9	1E+08	9_114395418	17	4	7	1	11	4	0.10516	1	0	8	21	0	4	11	0.14780855	1	7.25	7.9237437								
9	1E+08	9_114526099	12	11	13	1	12	7	0.325249	1	15	0	5	0	8	3	0.3843038	1	7.125	6.31183695								
9	1E+08	9_114639236	619	299	546	307	459	427	0.002556	0.13727	145	138	137	83	142	110	0.36703875	1	284.25	201.519053								
9	1E+08	9_114657453	45	38	28	31	42	30	0.111123	1	10	19	25	20	15	23	0.30916076	1	27	11.1355287								
9	1E+08	9_114668058	0	66	7	10	33	9	1.29E-04	0.01118	5	21	17	27	13	22	0.2175897	1	19.125	20.9177812								
9	1E+08	9_114921104	3362	1844	3731	4137	2603	3934	1.07E-22	#######	2621	1964	2427	1167	2293	1797	6.48E-07	0.0001128	2656.6	1018.15056								
9	1E+08	9_115023753	15	30	10	17	23	14	0.143642	1	13	8	15	4	11	10	0.99899888	1	14	7.74596669								
9	1E+08	9_115279269	6	0	13	12	3	13	0.09118	1	0	5	8	5	3	7	0.3829009	1	6.125	4.82367672								
9	1E+08	9_115393069	4	3	7	1	4	4	0.97482	1	0	9	26	1	5	14	0.08295177	1	6.375	8.51783842								
9	1E+08	9_115399425	38	54	24	2	46	13	5.45E-06	0.00069	52	21	43	0	37	22	0.25078535	1	29.25	21.0085017								
9	1E+08	9_115431974	40	4	4	5	22	5	0.001077	0.06758	2	1	11	3	2	7	0.20455692	1	8.75	12.980755								
9	1E+08	9_115568952	0	17	0	4	9	2	0.058587	1	19	1	9	0	10	5	0.51906437	1	6.25	7.88760329								
9	1E+08	9_115665350	4	14	13	5	9	9	0.944235	1	34	0	28	5	17	17	0.97589399	1	12.875	12.2175225								
9	1E+08	9_115675507	1	57	3	1	29	2	7.56E-07	0.00012	23	1	21	1	12	11	0.99963644	1	13.5	19.9069263								
9	1E+08	9_116285922	134	155	66	44	145	55	5.52E-13	#######	32	31	25	20	32	23	0.64682985	1	63.375	52.3066166								
9	1E+08	9_116353964	34	100	16	36	67	26	3.13E-06	0.00042	40	45	69	32	43	51	0.49081674	1	46.5	26.2406446	1E+08	1.2E+08	ID=ENSP000003565 29;RGS16;NM_002928;	RGS16				
9	1E+08	9_116651598	47	20	26	48	34	37	0.951407	1	2	0	0	14	1	7	0.08320466	1	19.625	19.6682304								
9	1E+08	9_116968813	16	22	2	3	19	3	7.41E-04	0.04898	29	3	11	15	16	13	0.9394435	1	12.625	9.81161848								
9	1E+08	9_117015691	8	3	1	0	6	1	0.108618	1	8	13	10	6	11	8	0.87190251	1	6.125	4.51782185								
9	1E+08	9_117220925	14	39	9	3	27	6	2.01E-04	0.01628	29	12	15	3	21	9	0.13945494	1	15.5	12.5584348								
9	1E+08	9_117223396	40	47	27	33	44	30	0.067875	1	36	4	25	35	20	30	0.24257255	1	30.875	12.8889488								
9	1E+08	9_117486431	38	27	11	8	33	10	2.83E-04	0.02195	24	7	4	1	16	3	0.01878845	0.6479877	15	13.0930734	1E+08	1.2E+08	ID=ENSP000003540 97;RGL1;;	RGL1				
9	1E+08	9_117990501	16	33	25	32	25	29	0.999172	1	22	47	59	18	35	39	0.73123094	1	31.5	14.8996644								
9	1E+08	9_118166033	0	5	13	10	3	12	0.129494	1	0	9	0	17	5	9	0.48000906	1	6.75	6.54107898								
9	1E+08	9_118221813	298	164	224	201	231	213	0.03978	1	83	51	57	39	67	48	0.39298026	1	139.63	96.0475143					1.2E+08	1.2E+08	0.37222	ID=promoter_53922;

9	1E+08	9_118227055	67	24	30	37	46	34	0.119794	1	0	26	11	56	13	34	0.00397338	0.1887956	31.375	22.0125776								
9	1E+08	9_118346499	2	11	3	6	7	5	0.693374	1	2	21	15	6	12	11	0.99963644	1	8.25	6.88165263	1E+08	1.2E+08	ID=ENSP000003564 82;EDEM3;NM_025191;	EDEM3				
9	1E+08	9_118401527	64	5	48	12	35	30	0.448769	1	0	5	8	2	3	5	0.7175395	1	18	24.11283	1E+08	1.2E+08	ID=ENSP00000375868;FAM129A;;	FAM129A				
9	1E+08	9_118917868	18	12	12	5	15	9	0.269309	1	29	23	32	23	26	28	0.85926991	1	19.25	9.2234174	1E+08	1.2E+08	ID=ENSP00000356468;IVNS1ABP;NM_006469;	IVNS1ABP				
9	1E+08	9_118929174	150	92	134	187	121	161	0.577026	1	114	109	171	59	112	115	0.74341017	1	127	42.1832736								
9	1E+08	9_119435277	2	46	20	15	24	18	0.351411	1	87	21	64	10	54	37	0.36386527	1	33.125	29.6957789								
9	1E+08	9_119671761	635	2262	784	737	1449	761	1.59E-74	#######	1807	1408	1792	920	1608	1356	0.0368481	1	1293.1	610.116251								
9	1E+08	9_120321638	350	115	25	73	233	49	9.39E-34	#######	261	259	295	119	260	207	0.26248629	1	187.13	118.285898								
9	1E+08	9_120448815	5	0	10	0	3	5	0.88968	1	10	15	0	0	13	0	0.00241566	0.1280305	5	5.97614305								
9	1E+08	9_121494353	40	106	25	34	73	30	2.75E-06	0.00038	72	15	61	27	44	44	0.93879668	1	47.5	30.2985148								
9	1E+08	9_121541814	43	24	3	1	34	2	4.18E-08	#######	2	6	1	4	4	3	0.96209564	1	10.5	15.1657509								
9	1E+08	9_121674703	10	0	6	4	5	5	0.968625	1	14	7	2	6	11	4	0.25131486	1	6.125	4.42194204								
9	1E+08	9_121676497	45	32	6	1	39	4	3.14E-08	#######	4	13	5	3	9	4	0.4571786	1	13.625	16.1239618								
9	1E+08	9_121713700	24	27	27	22	26	25	0.776821	1	23	16	11	5	20	8	0.11762508	1	19.375	7.98100423	1E+08	1.2E+08	ID=ENSP00000355923;PTPN14;NM_005401;	PTPN14	1.2E+08	1.2E+08	0.15182	ID=promoter_53956;
9	1E+08	9_121785856	20	44	6	19	32	13	0.003339	0.17064	1	0	7	27	1	17	4.35E-04	0.0314643	15.5	15.0712593								
9	1E+08	9_121793694	52	107	18	31	80	25	4.42E-09	#######	68	39	70	2	54	36	0.30582789	1	48.375	33.3078176								
9	1E+08	9_121825068	133	21	107	47	77	77	0.612063	1	26	0	22	0	13	11	0.97545578	1	44.5	49.4339386								
9	1E+08	9_121859082	4	0	2	5	2	4	0.823343	1	0	1	23	11	1	17	4.35E-04	0.0314643	5.75	7.85129653								
9	1E+08	9_121980922	612	506	895	731	559	813	4.58E-04	0.03293	66	99	240	130	83	185	6.24E-11	2.13E-08	409.88	318.622678								
9	1E+08	9_122030835	23	18	19	42	21	31	0.717186	1	67	15	26	0	41	13	0.00193653	0.1067879	26.25	20.1972417	1E+08	1.2E+08	ID=ENSP00000355925;PROX1;;	PROX1	1.2E+08	1.2E+08	0.1574	ID=promoter_53969;
9	1E+08	9_122065393	43	5	32	5	24	19	0.435991	1	63	24	30	15	44	23	0.07826377	1	27.125	19.6645984								
9	1E+08	9_122115271	32	53	30	22	43	26	0.024991	0.84297	21	26	32	3	24	18	0.79306625	1	27.375	13.9993622								
9	1E+08	9_122132562	22	79	47	50	51	49	0.607071	1	44	41	27	25	43	26	0.2226398	1	41.875	18.4269794								
9	1E+08	9_122186076	3	8	0	1	6	1	0.108618	1	36	0	22	42	18	32	0.07969995	1	14	17.1130694								
9	1E+08	9_122276481	200	115	299	205	158	252	0.009835	0.40238	106	109	44	82	108	63	0.01295635	0.4849326	145	83.0043028								
9	1E+08	9_122414534	40	56	22	36	48	29	0.016139	0.59639	138	100	42	26	119	34	1.21E-09	3.50E-07	57.5	40.620192								
9	1E+08	9_122465633	5	59	11	32	32	22	0.148825	1	31	19	18	7	25	13	0.22997911	1	22.75	17.7502515								
9	1E+08	9_122579231	10	17	1	12	14	7	0.165008	1	0	0	18	11	0	15	3.07E-04	0.0235795	8.625	7.40535521					1.2E+08	1.2E+08	0.13131	ID=promoter_53985;
9	1E+08	9_122586044	14	16	57	14	15	36	0.058922	1	32	0	20	4	16	12	0.85678722	1	19.625	17.9518204								
9	1E+08	9_122909102	2627	1719	3116	3261	2173	3189	7.09E-15	#######	1459	1109	1756	624	1284	1190	0.99997708	1	1958.9	951.210573								
9	1E+08	9_123014683	36	18	23	10	27	17	0.124321	1	18	40	26	46	29	36	0.50182289	1	27.125	12.414709								

9	1E+08	9_123093392	126	56	108	104	91	106	0.99882	1	57	49	38	2	53	20	0.00189061	0.1047116	67.5	41.6447561					1.2E+08	1.2E+08	0.39299	ID=promoter_53995;
9	1E+08	9_123166398	123	111	127	60	117	94	0.022903	0.7857	15	47	27	22	31	25	0.87406593	1	66.5	46.9771981					1.2E+08	1.2E+08	0.61689	ID=promoter_53996;
9	1E+08	9_123367231	19	26	76	53	23	65	9.09E-04	0.05849	43	15	18	2	29	10	0.0194	0.6626766	31.5	24.1601798								
9	1E+08	9_123699389	10	28	9	11	19	10	0.108738	1	52	19	46	1	36	24	0.45392565	1	22	18.5009652								
9	1E+08	9_123967905	7	19	3	0	13	2	0.006819	0.29841	19	16	30	4	18	17	0.9984894	1	12.25	10.3613292					1.2E+08	1.2E+08	0.19755	ID=promoter_54022;
9	1E+08	9_123977995	276	107	229	204	192	217	0.932205	1	59	51	65	56	55	61	0.62742724	1	130.88	91.1034538								
9	1E+08	9_124023220	0	17	9	5	9	7	0.713906	1	2	16	11	2	9	7	0.94097808	1	7.75	6.54107898	1E+08	1.2E+08	ID=ENSP00003559 66;NEK2;NM_002497;	NEK2				
9	1E+08	9_124027550	17	14	1	9	16	5	0.021336	0.74103	15	24	20	29	20	25	0.60650656	1	16.125	8.69215574					1.2E+08	1.2E+08	0.22615	ID=promoter_54027;
9	1E+08	9_124072438	26	22	16	8	24	12	0.04556	1	20	28	21	32	24	27	0.78764771	1	21.625	7.44383734								
9	1E+08	9_124157085	0	16	0	8	8	4	0.357158	1	0	0	22	14	0	18	6.08E-05	0.0059624	7.5	8.8640526								
9	1E+08	9_124211642	11	39	13	7	25	10	0.01059	0.42992	8	64	38	39	36	39	0.79640057	1	27.375	20.6670027								
9	1E+08	9_124216480	360	124	255	189	242	222	0.029749	0.96847	63	28	48	31	46	40	0.95710435	1	137.25	121.325713					1.2E+08	1.2E+08	0.30978	ID=promoter_54038;
9	1E+08	9_124217944	6	10	83	141	8	112	5.69E-17	#######	1	6	51	0	4	26	1.32E-04	0.0115204	37.25	51.3301638								
9	1E+08	9_124253464	0	15	7	3	8	5	0.532535	1	26	3	4	0	15	2	0.01114184	0.4293828	7.25	8.97217922								
9	1E+08	9_124335181	6	31	5	1	19	3	7.41E-04	0.04898	20	0	7	2	10	5	0.51906437	1	9	10.8759236								
9	1E+08	9_124500666	0	0	0	0	0	0	1	1	0	13	10	36	7	23	0.00743961	0.3120589	7.375	12.7047516					1.2E+08	1.2E+08	0.1565	ID=promoter_54045;
9	1E+08	9_124601047	26	24	11	26	25	19	0.35609	1	38	17	1	2	28	2	3.30E-05	0.0035507	18.125	12.8667623								
9	1E+08	9_124603451	23	55	12	42	39	27	0.103366	1	87	20	33	15	54	24	0.00913094	0.3646829	35.875	25.2101878								
9	1E+08	9_124692504	0	9	1	10	5	6	0.999292	1	0	20	17	2	10	10	0.98574881	1	7.375	7.92712342								
9	1E+08	9_124765629	7	15	7	0	11	4	0.10516	1	0	0	11	20	0	16	1.79E-04	0.0149638	7.5	7.50238057					1.2E+08	1.2E+08	0.11711	ID=promoter_54055;
9	1E+08	9_124800758	7	8	2	10	8	6	0.705675	1	2	12	12	12	7	12	0.42229866	1	8.125	4.22365786					1.2E+08	1.2E+08	0.10636	ID=promoter_54056;
9	1E+08	9_124876030	1	26	15	12	14	14	0.914609	1	2	0	16	26	1	21	5.13E-05	0.0051875	12.25	10.5661184								
9	1E+08	9_124889416	37	26	1	13	32	7	3.75E-05	0.00379	16	18	10	0	17	5	0.05852787	1	15.125	12.3338964					1.2E+08	1.2E+08	0.10214	ID=promoter_54065;
9	1E+08	9_125420224	76	50	86	127	63	107	0.064063	1	27	48	45	29	38	37	0.97766404	1	61	33.6621873								
9	1E+08	9_125422063	6	2	0	8	4	4	0.97482	1	0	7	16	2	4	9	0.31291743	1	5.125	5.38350656								
9	1E+08	9_125570300	1020	523	705	761	772	733	2.18E-04	0.01746	83	62	74	90	73	82	0.48961889	1	414.75	385.124006								
9	1E+08	9_125662381	14	17	28	32	16	30	0.310604	1	10	13	38	1	12	20	0.26508384	1	19.125	12.4262108					1.3E+08	1.3E+08	0.11303	ID=promoter_54109;
9	1E+08	9_125821007	265	130	194	133	198	164	0.004151	0.20244	113	54	68	31	84	50	0.04386073	1	123.5	77.1843804								
9	1E+08	9_125838729	218	65	122	105	142	114	0.010053	0.41045	99	56	74	24	78	49	0.09951654	1	95.375	58.4023911								
9	1E+08	9_125874658	0	15	13	0	8	7	0.850897	1	11	0	10	0	6	5	0.98461866	1	6.125	6.70687281								
9	1E+08	9_125937049	537	258	384	235	398	310	3.70E-07	#######	230	175	250	34	203	142	0.03560128	1	262.88	147.342592								
9	1E+08	9_126194600	6	21	6	8	14	7	0.165008	1	17	13	15	1	15	8	0.44137572	1	10.875	6.70687281								

9	1E+08	9_126225793	0	2	2	10	1	6	0.241978	1	19	7	15	0	13	8	0.65599869	1	6.875	7.21976454				
9	1E+08	9_126317185	25	4	74	32	15	53	3.87E-04	0.02857	51	15	17	3	33	10	0.00489842	0.2240264	27.625	24.3776098				
9	1E+08	9_126395217	29	6	34	31	18	33	0.306971	1	19	8	8	0	14	4	0.08931834	1	16.875	13.1196418				
9	1E+08	9_126500957	141	75	206	106	108	156	0.249532	1	64	29	87	27	47	57	0.39249424	1	91.875	59.623671				
9	1E+08	9_126700736	2	21	11	7	12	9	0.592799	1	29	2	34	16	16	25	0.25669436	1	15.25	12.0208153				
9	1E+08	9_126778630	38	16	19	25	27	22	0.443332	1	18	0	10	12	9	11	0.82669107	1	17.25	11.1707526	1.3E+08	1.3E+08	0.1239	ID=promoter_54151;
9	1E+08	9_126818767	1	8	31	9	5	20	0.033145	1	0	6	25	2	3	14	0.01844097	0.6375434	10.25	11.535659				
9	1E+08	9_126855485	10	13	9	6	12	8	0.45534	1	13	12	8	0	13	4	0.12786863	1	8.875	4.35685011				
9	1E+08	9_126935272	17	13	39	31	15	35	0.075557	1	49	25	9	10	37	10	0.00114024	0.0700243	24.125	14.5939077	1.3E+08	1.3E+08	0.10944	ID=promoter_54164;
9	1E+08	9_126946729	187	157	73	102	172	88	6.13E-10	#######	69	24	33	20	47	27	0.13514307	1	83.125	61.9894864				
9	1E+08	9_126958184	262	71	199	191	167	195	0.999082	1	31	31	31	14	31	23	0.7197219	1	103.75	97.638912				
9	1E+08	9_126968422	100	14	116	139	57	128	1.82E-04	0.01496	5	15	8	0	10	4	0.34296513	1	49.625	58.0466253				
9	1E+08	9_126985007	18	16	27	18	17	23	0.905043	1	44	30	31	17	37	24	0.39135592	1	25.125	9.77515071				
9	1E+08	9_127017095	13	9	12	7	11	10	0.842816	1	22	19	8	16	21	12	0.40192224	1	13.25	5.3917927				
9	1E+08	9_127052714	199	56	179	167	128	173	0.47702	1	115	90	87	52	103	70	0.1320029	1	118.13	56.8668558	1.3E+08	1.3E+08	0.34629	ID=promoter_54188;
9	1E+08	9_127104626	8	6	15	5	7	10	0.922856	1	0	8	0	9	4	5	0.90483023	1	6.375	4.92624169				
9	1E+08	9_127127834	45	83	53	80	64	67	0.809072	1	117	64	158	129	91	144	2.85E-04	0.0221519	91.125	39.7902985	1.3E+08	1.3E+08	0.27541	ID=promoter_54195;
9	1E+08	9_127162615	261	117	321	229	189	275	0.073457	1	15	10	32	16	13	24	0.12479476	1	125.13	127.417691	1.3E+08	1.3E+08	0.42252	ID=promoter_54207;
9	1E+08	9_127168222	13	22	9	15	18	12	0.307258	1	56	24	45	25	40	35	0.96931568	1	26.125	16.3045787	1.3E+08	1.3E+08	0.34872	ID=promoter_54208;
9	1E+08	9_127249121	12	7	17	9	10	13	0.970528	1	53	18	34	16	36	25	0.53796732	1	20.75	15.4156692	1.3E+08	1.3E+08	0.14731	ID=promoter_54225;
9	1E+08	9_127295598	6	0	19	6	3	13	0.09118	1	85	20	16	21	53	19	0.00111164	0.0685927	21.625	26.763181				
9	1E+08	9_127304639	16	38	3	7	27	5	7.68E-05	0.00707	0	0	0	1	0	1	0.58312783	1	8.125	13.2604406	1.3E+08	1.3E+08	0.31071	ID=promoter_54232;
9	1E+08	9_127311254	7	13	14	21	10	18	0.549586	1	3	23	18	28	13	23	0.16598565	1	15.875	8.32273135				
9	1E+08	9_127322906	167	79	168	166	123	167	0.468036	1	67	43	86	11	55	49	0.97972456	1	98.375	61.3466439				
9	1E+08	9_127453084	3	0	11	12	2	12	0.058554	1	8	1	25	11	5	18	0.01516798	0.5480081	8.875	8.06115022	1.3E+08	1.3E+08	0.20198	ID=promoter_54255;
9	1E+08	9_127457592	87	34	48	36	61	42	0.026947	0.89396	69	73	35	10	71	23	2.70E-05	0.0029792	49	25.4446177	1.3E+08	1.3E+08	0.14731	ID=promoter_54257;
9	1E+08	9_127515598	6	57	12	5	32	9	2.11E-04	0.01699	69	2	36	3	36	20	0.17860656	1	23.75	26.7568416	1.3E+08	1.3E+08	0.14908	ID=promoter_54272;
9	1E+08	9_127530309	20	2	7	13	11	10	0.842816	1	19	11	0	25	15	13	0.98432481	1	12.125	8.88718982				
9	1E+08	9_127535612	331	56	157	105	194	131	4.89E-06	0.00063	112	62	60	35	87	48	0.01412681	0.5194607	114.75	95.8000149	1.3E+08	1.3E+08	0.10909	ID=promoter_54274;
9	1E+08	9_127561743	9	6	3	8	8	6	0.705675	1	13	0	0	1	7	1	0.13113995	1	5	4.78091444				
9	1E+08	9_127598386	4	20	1	3	12	2	0.011798	0.46127	24	0	9	32	12	21	0.20312388	1	11.625	12.1059312				
9	1E+08	9_127608417	17	16	21	13	17	17	0.897282	1	3	13	5	2	8	4	0.59180275	1	11.25	7.06601524	1.3E+08	1.3E+08	0.13318	ID=promoter_54282;

9	1E+08	9_127611966	15	11	23	1	13	12	0.835334	1	16	22	14	13	19	14	0.80779431	1	14.375	6.84392327								
9	1E+08	9_127721059	32	5	10	47	19	29	0.670114	1	0	29	16	15	15	16	0.92652531	1	19.25	15.6547574								
9	1E+08	9_127744188	72	41	63	72	57	68	0.995565	1	0	27	22	10	14	16	0.84864027	1	38.375	28.157402					1.3E+08	1.3E+08	0.13527	ID=promoter_54292;
9	1E+08	9_127757223	0	0	4	2	0	3	0.278365	1	0	0	23	21	0	22	7.03E-06	0.0009311	6.25	9.83797308					1.3E+08	1.3E+08	0.14889	ID=promoter_54293;
9	1E+08	9_127793483	0	12	0	7	6	4	0.674789	1	25	8	9	3	17	6	0.10713464	1	8	8.10643483								
9	1E+08	9_127902234	0	5	3	2	3	3	0.981055	1	7	8	5	12	8	9	0.92351511	1	5.25	3.77018378	1E+08	1.3E+08	ID=ENSP00000341819;VWC2;NM_198570;	VWC2	1.3E+08	1.3E+08	0.77753	ID=promoter_54302;
9	1E+08	9_127902519	463	186	326	268	325	297	0.007751	0.33296	163	77	122	7	120	65	0.00195974	0.1077881	201.5	146.227807	1E+08	1.3E+08	ID=ENSP00000341819;VWC2;NM_198570;	VWC2	1.3E+08	1.3E+08	0.77753	ID=promoter_54302;
9	1E+08	9_128008840	3	45	20	7	24	14	0.105787	1	25	58	25	6	42	16	0.0075077	0.3140846	23.625	19.522423								
9	1E+08	9_128155511	6	10	4	4	8	4	0.357158	1	27	18	22	1	23	12	0.26207533	1	11.5	9.62140471								
9	1E+08	9_128163266	36	34	49	12	35	31	0.520594	1	32	36	59	16	34	38	0.73008239	1	34.25	15.4434452					1.3E+08	1.3E+08	0.15353	ID=promoter_54321;
9	1E+08	9_128163401	40	5	11	5	23	8	0.007066	0.30636	17	49	27	0	33	14	0.04245489	1	19.25	17.8305516					1.3E+08	1.3E+08	0.15353	ID=promoter_54321;
9	1E+08	9_128171389	12	12	0	1	12	1	0.003675	0.18279	1	12	2	1	7	2	0.29886173	1	5.125	5.7180791					1.3E+08	1.3E+08	0.20371	ID=promoter_54323;
9	1E+08	9_128225385	14	45	24	27	30	26	0.525876	1	65	54	69	98	60	84	0.04898991	1	49.5	27.8516478	1E+08	1.3E+08	ID=ENSP00000262032;IKZF4;NM_022465;	IKZF4	1.3E+08	1.3E+08	0.25897	ID=promoter_54334;
9	1E+08	9_128227097	21	3	16	14	12	15	0.986595	1	33	0	19	17	17	18	0.92557631	1	15.375	10.3501208	1E+08	1.3E+08	ID=ENSP00000262032;IKZF4;NM_022465;	IKZF4	1.3E+08	1.3E+08	0.18266	ID=promoter_54335;
9	1E+08	9_128249511	59	7	37	42	33	40	0.990292	1	20	10	30	4	15	17	0.85096642	1	26.125	19.3423111								
9	1E+08	9_128326953	219	332	226	328	276	277	0.185693	1	206	201	290	83	204	187	0.99383738	1	235.63	81.5193886								
9	1E+08	9_128384420	0	13	5	1	7	3	0.314307	1	8	15	16	0	12	8	0.76853133	1	7.25	6.75594976					1.3E+08	1.3E+08	0.14737	ID=promoter_54364;
9	1E+08	9_128503124	183	85	229	156	134	193	0.187685	1	110	17	24	21	64	23	2.79E-04	0.0217092	103.13	80.9045955	1E+08	1.3E+08	ID=ENSP00000263915-D5;GRB14;NM_004490;	GRB14				
9	1E+08	9_128512265	66	40	75	83	53	79	0.400483	1	16	58	15	45	37	30	0.86166638	1	49.75	25.4656632					1.3E+08	1.3E+08	0.23065	ID=promoter_54392;
9	1E+08	9_128523896	117	20	95	66	69	81	0.999988	1	6	4	8	25	5	17	0.02356069	0.7742377	42.625	44.2006383								
9	1E+08	9_128531703	14	19	10	14	17	12	0.395844	1	0	18	10	15	9	13	0.58766318	1	12.5	6					1.3E+08	1.3E+08	0.42643	ID=promoter_54400;
9	1E+08	9_128559805	181	64	135	119	123	127	0.601249	1	20	42	48	41	31	45	0.15310101	1	81.25	56.7947432					1.3E+08	1.3E+08	0.54666	ID=promoter_54407;
9	1E+08	9_128559901	90	87	89	89	89	89	0.566982	1	87	42	46	17	65	32	0.01179676	0.4484493	68.375	28.9034724					1.3E+08	1.3E+08	0.54666	ID=promoter_54407;
9	1E+08	9_128580619	16	65	8	26	41	17	8.21E-04	0.05385	42	21	40	26	32	33	0.9118622	1	30.5	17.9682259					1.3E+08	1.3E+08	0.24392	ID=promoter_54411;

9	1E+08	9_128629935	12	23	18	28	18	23	0.963662	1	74	53	17	25	64	21	8.94E-05	0.0083924	31.25	21.2720474	1.3E+08	1.3E+08	0.26373	ID=promoter_54421;
9	1E+08	9_128648746	362	200	177	184	281	181	1.42E-09	#######	32	36	37	9	34	23	0.5031903	1	129.63	122.811048				
9	1E+08	9_128759931	78	30	37	23	54	30	0.003551	0.17889	41	1	8	18	21	13	0.51498325	1	29.5	23.8746728	1.3E+08	1.3E+08	0.26042	ID=promoter_54431;
9	1E+08	9_128773607	51	19	62	65	35	64	0.103601	1	38	11	49	20	25	35	0.2845844	1	39.375	20.6808227	1.3E+08	1.3E+08	0.13835	ID=promoter_54435;
9	1E+08	9_128782303	78	53	162	155	66	159	3.34E-06	0.00045	7	10	71	65	9	68	1.49E-11	5.44E-09	75.125	57.850639	1.3E+08	1.3E+08	0.62999	ID=promoter_54436;
9	1E+08	9_128805236	166	104	85	113	135	99	0.001634	0.09548	45	10	37	4	28	21	0.76300557	1	70.5	56.2062528				
9	1E+08	9_128815258	29	63	20	14	46	17	9.70E-05	0.00867	48	42	42	31	45	37	0.86385836	1	36.125	15.8514308	1.3E+08	1.3E+08	0.20892	ID=promoter_54442;
9	1E+08	9_128825358	57	12	32	36	35	34	0.735392	1	15	17	18	2	16	10	0.61601721	1	23.625	17.2787028				
9	1E+08	9_128848991	4	3	8	10	4	9	0.54411	1	10	2	6	1	6	4	0.87665919	1	5.5	3.54562104				
9	1E+08	9_128862092	106	58	47	16	82	32	2.04E-07	#######	108	89	53	64	99	59	0.02554244	0.8269892	67.625	31.5546578	1.3E+08	1.3E+08	0.09577	ID=promoter_54452;
9	1E+08	9_128868847	254	128	216	242	191	229	0.975283	1	2	55	13	1	29	7	0.00267552	0.137714	113.88	110.569225				
9	1E+08	9_128911149	447	21	155	78	234	117	8.34E-14	#######	59	39	24	18	49	21	0.01008507	0.3959241	105.13	145.332562	1.3E+08	1.3E+08	0.2389	ID=promoter_54467;
9	1E+08	9_129120609	10	19	51	46	15	49	0.001403	0.08373	19	60	84	44	40	64	0.02333576	0.7680341	41.625	24.6978166				
9	1E+08	9_129192054	5	26	23	32	16	28	0.437866	1	60	28	59	25	44	42	0.99085882	1	32.25	18.6068344				
9	1E+08	9_129270552	5	27	10	2	16	6	0.042495	1	10	1	11	20	6	16	0.069189	1	10.75	8.90826262				
9	1E+08	9_129534950	6	15	3	6	11	5	0.189421	1	0	27	12	30	14	21	0.37101303	1	12.375	11.0445526				
9	1E+08	9_129614860	12	19	16	27	16	22	0.889379	1	41	18	6	6	30	6	8.03E-04	0.0527633	18.125	11.5812348				
9	1E+08	9_129660097	2	7	8	7	5	8	0.86114	1	3	16	13	1	10	7	0.84912739	1	7.125	5.2762947				
9	1E+08	9_129696481	1	33	1	3	17	2	7.31E-04	0.04855	8	20	1	0	14	1	0.00574567	0.2548307	8.375	11.9754213	1.3E+08	1.3E+08	0.19875	ID=promoter_54549;
9	1E+08	9_129750123	4	43	5	10	24	8	0.004476	0.21437	5	11	8	29	8	19	0.06853691	1	14.375	14.080762	1.3E+08	1.3E+08	0.28894	ID=promoter_54560;
9	1E+08	9_129759435	2	0	6	14	1	10	0.048828	1	20	10	8	23	15	16	0.92652531	1	10.375	8.17552969				
9	1E+08	9_129846643	85	36	62	11	61	37	0.005559	0.2562	0	0	5	0	0	3	0.19828566	1	24.875	32.8652552	1.3E+08	1.3E+08	0.25482	ID=promoter_54573;
9	1E+08	9_129867375	27	47	21	18	37	20	0.016661	0.61076	26	12	19	52	19	36	0.03633239	1	27.75	14.2803561				
9	1E+08	9_130039329	4	9	9	22	7	16	0.324814	1	3	6	8	1	5	5	0.99284883	1	7.75	6.4531277				
9	1E+08	9_130074526	7	9	3	0	8	2	0.097885	1	0	5	24	0	3	12	0.04708211	1	6	8.03563492				
9	1E+08	9_130075137	5	10	6	10	8	8	0.950274	1	33	4	25	1	19	13	0.6985193	1	11.75	11.2599163	1.3E+08	1.3E+08	0.30323	ID=promoter_54597;
9	1E+08	9_130086395	12	23	12	3	18	8	0.059652	1	24	9	21	63	17	42	0.00184663	0.1024348	20.875	18.5429039				
9	1E+08	9_130097570	3	8	12	16	6	14	0.355886	1	20	33	7	14	27	11	0.06118138	1	14.125	9.34172667	1.3E+08	1.3E+08	0.23001	ID=promoter_54600;
9	1E+08	9_130097598	0	21	2	2	11	2	0.020291	0.71411	0	1	2	30	1	16	7.41E-04	0.0492332	7.25	11.5480363	1.3E+08	1.3E+08	0.23001	ID=promoter_54600;
9	1E+08	9_130337209	2	72	21	96	37	59	0.339357	1	3	37	108	46	20	77	5.59E-09	1.45E-06	48.125	40.487873				
9	1E+08	9_130399890	30	35	15	13	33	14	0.00394	0.1935	18	12	31	3	15	17	0.85096642	1	19.625	11.1859287				

9	1E+08	9_130439639	30	30	12	9	30	11	0.002281	0.12403	38	9	14	0	24	7	0.01749745	0.6144624	17.75	13.231456					1.3E+08	1.3E+08	0.14978	ID=promoter_54660;
9	1E+08	9_130444315	6	47	4	17	27	11	0.00829	0.35136	77	17	14	5	47	10	2.34E-05	0.002637	23.375	25.6845562								
9	1E+08	9_130489435	43	11	22	15	27	19	0.225928	1	9	7	3	2	8	3	0.3843038	1	14	13.3844259								
9	1E+08	9_130551860	15	0	36	15	8	26	0.031378	1	15	24	31	19	20	25	0.60650656	1	19.375	11.1218896								
9	1E+08	9_130556413	1	21	4	6	11	5	0.189421	1	21	8	15	10	15	13	0.98432481	1	10.75	7.55456343					1.3E+08	1.3E+08	0.18326	ID=promoter_54674;
9	1E+08	9_130590018	98	139	104	59	119	82	8.83E-04	0.05718	28	7	56	5	18	31	0.10487282	1	62	48.4974226								
9	1E+08	9_130616624	40	18	55	37	29	46	0.442059	1	18	39	47	27	29	37	0.43334569	1	35.125	13.2604406					1.3E+08	1.3E+08	0.43618	ID=promoter_54682;
9	1E+08	9_130661432	451	265	481	431	358	456	0.505706	1	250	217	227	113	234	170	0.05286225	1	304.38	132.81667								
9	1E+08	9_130666367	40	46	13	16	43	15	9.81E-05	0.00874	23	24	36	53	24	45	0.01749321	0.6144624	31.375	14.520306	1E+08	1.3E+08	ID=ENSP00000384701;VSTM2A;;	VSTM2A	1.3E+08	1.3E+08	0.40822	ID=promoter_54690;
9	1E+08	9_130667242	19	14	32	32	17	32	0.282172	1	4	8	18	2	6	10	0.51486293	1	16.125	11.5441698	1E+08	1.3E+08	ID=ENSP00000384701;VSTM2A;;	VSTM2A	1.3E+08	1.3E+08	0.40822	ID=promoter_54690;
9	1E+08	9_130701221	36	30	49	38	33	44	0.856357	1	21	19	4	21	20	13	0.60545249	1	27.25	13.9360786								
9	1E+08	9_130737778	28	9	29	23	19	26	0.877519	1	14	13	4	2	14	3	0.04246461	1	15.25	10.4163333					1.3E+08	1.3E+08	0.21666	ID=promoter_54699;
9	1E+08	9_130762127	1	5	4	8	3	6	0.747087	1	6	0	2	27	3	15	0.01140593	0.4370832	6.625	8.65097022					1.3E+08	1.3E+08	0.29356	ID=promoter_54704;
9	1E+08	9_130767293	40	49	29	28	45	29	0.038455	1	38	20	26	35	29	31	0.858757	1	33.125	9.20306626					1.3E+08	1.3E+08	0.43678	ID=promoter_54705;
9	1E+08	9_130788002	1	42	47	58	22	53	0.014944	0.56151	22	22	59	20	22	40	0.03530987	1	33.875	20.7256742								
9	1E+08	9_130860873	14	17	8	17	16	13	0.613293	1	5	26	11	40	16	26	0.20160145	1	17.25	11.2090525					1.3E+08	1.3E+08	0.58619	ID=promoter_54715;
9	1E+08	9_130964416	0	20	2	0	10	1	0.011548	0.45484	1	21	14	4	11	9	0.96206785	1	7.75	9.08295106								
9	1E+08	9_131113161	30	13	11	11	22	11	0.058934	1	1	0	4	17	1	11	0.01045793	0.4067594	10.875	9.78975412								
9	1E+08	9_131283224	109	60	127	111	85	119	0.459204	1	0	0	0	0	0	0	1	1	50.875	57.6006882								
9	1E+08	9_131494958	10	21	16	14	16	15	0.822707	1	19	19	41	23	19	32	0.1120603	1	20.375	9.28804916								
9	1E+08	9_131561341	9	25	5	13	17	9	0.140533	1	31	21	31	20	26	26	0.96336814	1	19.375	9.70916357								
9	1E+08	9_131741609	1042	423	712	667	733	690	1.78E-04	0.01468	573	370	380	187	472	284	4.94E-08	1.08E-05	544.25	264.968866					1.3E+08	1.3E+08	0.35156	ID=promoter_54754;
9	1E+08	9_131875353	137	20	65	56	79	61	0.047234	1	38	15	32	34	27	33	0.56497566	1	49.625	39.0565067	1E+08	1.3E+08	ID=ENSP00000356070;MAPKAPK2;NM_032960;	MAPKAPK2				
9	1E+08	9_131875651	1	24	15	5	13	10	0.600182	1	0	14	11	0	7	6	0.99011599	1	8.75	8.71370021	1E+08	1.3E+08	ID=ENSP00000356070;MAPKAPK2;NM_032960;	MAPKAPK2				
9	1E+08	9_131945200	62	46	70	68	54	69	0.8949	1	92	10	28	27	51	28	0.07905636	1	50.375	27.4118718	1E+08	1.3E+08	ID=ENSP00000356066;IL10;NM_000572;	IL10				
9	1E+08	9_131956435	23	28	14	12	26	13	0.035221	1	54	31	32	4	43	18	0.01460712	0.5325962	24.75	15.4434452								
9	1E+08	9_131960191	45	19	44	34	32	39	0.987189	1	39	26	31	20	33	26	0.82464739	1	32.25	10.1101081								

9	1E+08	9_132211923	129	68	90	66	99	78	0.030195	0.97963	9	59	29	0	34	15	0.04959965	1	56.25	42.7810038					1.3E+08	1.3E+08	0.22499	ID=promoter_54764;
9	1E+08	9_132378716	31	65	17	48	48	33	0.057413	1	25	8	35	44	17	40	0.00384905	0.1844907	34.125	18.1772031								
10	36899	10_36899	82	178	73	90	130	82	4.59E-05	0.00451	147	185	232	141	166	187	0.18729533	1	141	56.4775556	19541	43696	ID=ENSP000002615 84;PALB2;NM_024675;	PALB2				
10	579956	10_57995	1	0	25	9	1	17	0.002653	0.14147	36	17	24	36	27	30	0.79159197	1	18.5	14.2728514								
10	583938	10_58393	4	0	1	0	2	1	0.773064	1	0	15	35	0	8	18	0.09791732	1	6.875	12.4721117								
10	676154	10_67615	1	12	0	15	7	8	0.998739	1	0	6	8	3	3	6	0.53745121	1	5.625	5.68048036								
10	838511	10_83851	21	16	7	28	19	18	0.809248	1	30	17	17	0	24	9	0.0567576	1	17	9.97138764								
10	870956	10_87095	262	205	243	270	234	257	0.767202	1	115	71	66	44	93	55	0.0286992	0.9011995	159.5	95.3594703								
10	1E+06	10_12410	12	24	7	1	18	4	0.003432	0.17404	45	15	30	24	30	27	0.99379101	1	19.75	14.0178458								
10	1E+06	10_1290492	0	0	2	0	0	1	0.652937	1	45	19	2	0	32	1	1.47E-06	0.0002348	8.5	16.1067865					1289904	1290864	0.15192	ID=promoter_05246;
10	1E+06	10_1354238	12	3	0	1	8	1	0.03583	1	28	0	5	0	14	3	0.04246461	1	6.125	9.731209					1353861	1354821	0.15192	ID=promoter_05249;
10	1E+06	10_14598	26	153	55	35	90	45	9.33E-06	0.00113	74	75	121	69	75	95	0.12765354	1	76	42.4768845								
10	2E+06	10_1625335	23	27	14	9	25	12	0.031505	1	17	8	7	22	13	15	0.84581615	1	15.875	7.60521438					1622440	1625684	0.52116	ID=promoter_05273;
10	2E+06	10_16262	5	49	18	23	27	21	0.36385	1	0	30	65	2	15	34	0.01174317	0.4468879	24	23.2992949								
10	2E+06	10_16913	6	38	42	18	22	30	0.865661	1	42	24	20	39	33	30	0.99699964	1	28.625	13.4900757								
10	2E+06	10_1808999	7	33	24	2	20	13	0.243396	1	42	0	19	23	21	21	0.97030696	1	18.75	14.926008	2E+06	1818022	ID=ENSP000003564 16;TROVE2;;	TROVE2				
10	2E+06	10_18307	33	68	19	14	51	17	1.02E-05	0.00122	6	36	69	19	21	44	0.00678032	0.2902019	33	23.9404022								
10	2E+06	10_1832816	2	1	3	18	2	11	0.085797	1	6	6	4	0	6	2	0.42506085	1	5	5.68205194					1832316	1833496	0.22669	ID=promoter_05288;
10	2E+06	10_1844147	9	3	10	12	6	11	0.674578	1	0	19	18	0	10	9	0.99792604	1	8.875	7.45342682					1843779	1844281	0.12142	ID=promoter_05289;
10	2E+06	10_19369	63	0	37	15	32	26	0.376129	1	14	11	22	1	13	12	0.99994263	1	20.375	20.8528004								
10	2E+06	10_19584	12	44	6	14	28	10	0.00287	0.14985	32	22	31	39	27	35	0.42118875	1	25	13.6381817								
10	2E+06	10_19745	15	4	0	11	10	6	0.41703	1	1	0	23	2	1	13	0.0036419	0.1775497	7	8.48528137								
10	2E+06	10_19994	17	18	4	3	18	4	0.003432	0.17404	0	14	5	3	7	4	0.73798468	1	8	7.13141941								
10	2E+06	10_21644	149	282	92	114	216	103	7.58E-14	#######	265	197	297	131	231	214	0.99997377	1	190.88	81.1725455								
10	2E+06	10_22232	14	29	3	25	22	14	0.192025	1	23	11	44	31	17	38	0.00782445	0.3231698	22.5	12.9173195								
10	2E+06	10_23921	1	12	5	3	7	4	0.503048	1	13	12	6	0	13	3	0.06317581	1	6.5	5.20988072								
10	2E+06	10_2393948	47	35	34	5026	41	2530	0	0	10	48	31	9	29	20	0.5935331	1	655	1766.21112					2393725	2394676	0.19002	ID=promoter_05309;
10	2E+06	10_2395784	1	5	10	6	3	8	0.459247	1	21	17	20	51	19	36	0.03633239	1	16.375	15.8017856					2395483	2396137	0.22896	ID=promoter_05310;
10	3E+06	10_25127	3	45	0	8	24	4	1.39E-04	0.01187	1	16	19	1	9	10	0.92505409	1	11.625	15.2871328								
10	3E+06	10_25142	4	3	30	26	4	28	7.19E-04	0.04817	13	9	16	3	11	10	0.99899888	1	13	10.4471459								
10	3E+06	10_27587	24	19	17	16	22	17	0.428533	1	52	20	26	34	36	30	0.91118883	1	26	12.0118989								
10	3E+06	10_31861	2	8	1	6	5	4	0.848147	1	4	8	12	8	6	10	0.51486293	1	6.125	3.64250699								
10	3E+06	10_31941	1	4	0	9	3	5	0.88968	1	0	0	5	36	0	21	1.21E-05	0.0014894	6.875	12.1941145								
10	3E+06	10_34942	8	22	9	11	15	10	0.374041	1	3	10	6	0	7	3	0.51857467	1	8.625	6.5451727								
10	4E+06	10_43406	16	21	3	4	19	4	0.002035	0.11403	85	23	3	7	54	5	8.69E-09	2.17E-06	20.25	27.3952551								
10	5E+06	10_50171	16	13	16	8	15	12	0.610117	1	0	40	25	38	20	32	0.15276046	1	19.5	14								
10	5E+06	10_50983	3	20	11	8	12	10	0.724827	1	0	27	36	40	14	38	0.00151301	0.0879967	18.125	15.0754057								
10	5E+06	10_54435	56	67	68	40	62	54	0.275762	1	48	76	49	76	62	63	0.87666535	1	60	13.6591152								
10	6E+06	10_55715	27	0	20	20	14	20	0.851664	1	68	1	21	14	35	18	0.11957198	1	21.375	21.1655886								
10	6E+06	10_56106	0	17	8	10	9	9	0.944235	1	29	0	7	1	15	4	0.06170633	1	9	9.97138764								
10	6E+06	10_56117	5	1	8	17	3	13	0.09118	1	1	0	23	4	1	14	0.00214405	0.1161796	7.375	8.36553303								
10	6E+06	10_58510	8	7	5	5	8	5	0.532535	1	12	0	20	0	6	10	0.51486293	1	7.125	6.55607679								
10	6E+06	10_5955125	1	53	16	5	27	11	0.00829	0.35136	1	33	35	21	17	28	0.16600816	1	20.625	18.6848869	6E+06	5955451	ID=ENSP000002591 54;KCTD3;NM_016121;	KCTD3				
10	6E+06	10_6022208	0	3	16	3	2	10	0.124792	1	10	8	12	0	9	6	0.8208167	1	6.5	5.90399381	6E+06	6714140	ID=ENSP000003559 10;USH2A;;	USH2A	6021951	6022715	0.12446	ID=promoter_05361;

10	6E+06	10_6200241	46	27	37	15	37	26	0.128943	1	14	8	0	3	11	2	0.06154634	1	18.75	16.4555679	6E+06	6714140	ID=ENSP00000355910;USH2A;;	USH2A				
10	6E+06	10_6233835	7	3	0	3	5	2	0.402696	1	80	24	8	57	52	33	0.23056449	1	22.75	29.7981303	6E+06	6714140	ID=ENSP00000355910;USH2A;;	USH2A				
10	6E+06	10_6246860	37	25	24	50	31	37	0.997448	1	6	12	14	3	9	9	0.98716474	1	21.375	16.0351177	6E+06	6714140	ID=ENSP00000355910;USH2A;;	USH2A	6246522	6247446	0.15883	ID=promoter_05373;
10	6E+06	10_6445953	8	27	16	10	18	13	0.404491	1	36	6	8	4	21	6	0.02669383	0.8538132	14.375	11.388434	6E+06	6714140	ID=ENSP00000355910;USH2A;;	USH2A				
10	7E+06	10_6595872	0	0	0	4	0	2	0.426327	1	0	9	0	28	5	14	0.08295177	1	5.125	9.78975412	6E+06	6714140	ID=ENSP00000355910;USH2A;;	USH2A				
10	7E+06	10_68259	485	257	495	446	371	471	0.522002	1	269	271	167	131	270	149	1.83E-06	0.0002875	315.13	142.2457								
10	7E+06	10_6972556	29	71	40	42	50	41	0.232493	1	69	28	26	18	49	22	0.01547602	0.5546463	40.375	19.8201737					6972220	6972992	0.19926	ID=promoter_05397;
10	7E+06	10_69830	18	49	8	27	34	18	0.01975	0.69938	10	49	23	20	30	22	0.70449098	1	25.5	15.7933078								
10	7E+06	10_70457	0	18	3	6	9	5	0.390586	1	0	34	16	23	17	20	0.769441	1	12.5	12.3056317								
10	7E+06	10_70549	23	25	56	49	24	53	0.032766	1	61	78	63	35	70	49	0.31828044	1	48.75	19.5576072								
10	7E+06	10_70592	10	15	9	2	13	6	0.146758	1	6	12	8	12	9	10	0.92505409	1	9.25	4.02669663								
10	7E+06	10_7123515	10	4	7	2	7	5	0.693374	1	6	10	8	0	8	4	0.59180275	1	5.875	3.64250699					7123045	7123896	0.28409	ID=promoter_05407;
10	7E+06	10_72296	3	17	9	4	10	7	0.570701	1	0	9	28	4	5	16	0.03625394	1	9.25	9.19238816								
10	7E+06	10_73445	19	6	4	8	13	6	0.146758	1	60	23	8	2	42	5	1.82E-06	0.0002865	16.25	19.1292596								
10	7E+06	10_73631	45	32	22	16	39	19	0.00521	0.24279	100	19	30	21	60	26	0.00407355	0.1931695	35.625	27.6143726								
10	7E+06	10_7438333	75	17	42	45	46	44	0.625335	1	0	12	19	31	6	25	0.00139684	0.0827572	30.125	23.6307155					7437672	7438512	0.13851	ID=promoter_05422;
10	7E+06	10_74827	20	25	6	15	23	11	0.040732	1	1	17	7	27	9	17	0.21129426	1	14.75	9.36177639								
10	8E+06	10_76759	5	18	5	8	12	7	0.325249	1	0	0	0	10	0	5	0.06742467	1	5.75	6.25071424								
10	8E+06	10_77977	19	26	14	8	23	11	0.040732	1	8	41	14	6	25	10	0.06888504	1	17	11.7229933								
10	8E+06	10_78358	12	47	10	16	30	13	0.007592	0.32689	18	10	33	55	14	44	1.30E-04	0.0113876	25.125	17.7155098								
10	8E+06	10_7837833	3	12	9	7	8	8	0.950274	1	20	9	25	1	15	13	0.98432481	1	10.75	8.15475322					7837277	7838101	0.22128	ID=promoter_05438;
10	8E+06	10_78705	0	10	0	1	5	1	0.186119	1	0	11	7	33	6	20	0.0132456	0.49354	7.75	11.2090525								
10	8E+06	10_79682	13	30	12	16	22	14	0.192025	1	33	19	28	21	26	25	0.99152959	1	21.5	7.98212288								
10	8E+06	10_80061	36	22	29	32	29	31	0.937432	1	8	9	1	6	9	4	0.4571786	1	17.875	13.4741817								
10	8E+06	10_80975	5	43	16	15	24	16	0.207334	1	12	9	9	0	11	5	0.40227598	1	13.625	12.9607925								
10	8E+06	10_81645	14	5	10	2	10	6	0.41703	1	18	0	6	24	9	15	0.36943449	1	9.875	8.28833087								
10	8E+06	10_82057	22	61	25	39	42	32	0.179623	1	51	7	24	0	29	12	0.05423954	1	28.625	20.7773056								
10	8E+06	10_83222	1	0	5	2	1	4	0.505922	1	18	7	21	4	13	13	0.98151319	1	7.25	7.9237437								
10	8E+06	10_83989	6	22	56	69	14	63	6.08E-06	0.00077	55	19	8	0	37	4	5.68E-06	0.0007768	29.375	26.629401								
10	8E+06	10_84143	9	48	5	24	29	15	0.030102	0.97706	22	24	34	39	23	37	0.10975248	1	25.625	14.4907606								
10	8E+06	10_84881	10	55	4	29	33	17	0.018001	0.65052	2	0	56	16	1	36	1.63E-08	3.89E-06	21.5	22.9284602								
10	9E+06	10_85207	16	57	14	45	37	30	0.319086	1	22	58	62	22	40	42	0.85290898	1	37	20.5287255								
10	9E+06	10_85459	11	25	22	14	18	18	0.89158	1	128	28	25	1	78	13	8.82E-10	2.61E-07	31.75	39.9204566								
10	9E+06	10_85482	24	37	8	3	31	6	2.44E-05	0.00261	54	31	14	9	43	12	4.94E-04	0.0351794	22.5	17.4109982								
10	9E+06	10_85653	0	8	1	1	4	1	0.312866	1	0	1	18	16	1	17	4.35E-04	0.0314643	5.625	7.50119038								
10	9E+06	10_85866	2	14	10	4	8	7	0.850897	1	2	15	8	4	9	6	0.8208167	1	7.375	5.20816666								
10	9E+06	10_86427	57	95	69	71	76	70	0.342974	1	93	63	67	30	78	49	0.09951654	1	68.125	20.5456808								
10	9E+06	10_86957	19	29	9	8	24	9	0.00876	0.36763	27	1	35	14	14	25	0.13553211	1	17.75	11.8170579								
10	9E+06	10_8737887	6	10	8	5	8	7	0.850897	1	1	7	15	14	4	15	0.026797	0.8546242	8.25	4.65218843					8737640	8738391	0.12819	ID=promoter_05473;
10	9E+06	10_87640	26	37	0	5	32	3	4.92E-07	#######	10	0	3	2	5	3	0.8350419	1	10.375	13.7418807								
10	9E+06	10_91249	16	40	16	9	28	13	0.016679	0.61094	4	9	12	12	7	12	0.42229866	1	14.75	10.9381377								
10	9E+06	10_93425	22	43	46	67	33	57	0.204975	1	26	26	41	36	26	39	0.1585967	1	38.375	14.5694151								
10	1E+07	10_95568	9	13	13	1	11	7	0.438207	1	25	23	8	4	24	6	0.00867909	0.3505674	12	8.46842876								
10	1E+07	10_95738	6	58	15	20	32	18	0.038646	1	41	46	43	4	44	24	0.10689254	1	29.125	20.3570802								
10	1E+07	10_96166	91	534	151	160	313	156	2.53E-18	#######	444	511	522	331	478	427	0.85641735	1	343	185.529358								
10	1E+07	10_96192	2	55	14	11	29	13	0.011304	0.44898	0	18	15	0	9	8	0.99626583	1	14.375	17.8960291								
10	1E+07	10_9694901	381	116	460	324	249	392	0.001305	0.07843	140	140	144	29	140	87	0.0129119	0.4836301	216.75	151.245071					9693495	9695167	0.63654	ID=promoter_05498;

10	1E+07	10_9752823	1	12	10	22	7	16	0.324814	1	89	32	57	13	61	35	0.07360558	1	29.5	29.5924701	1E+07	9780168	ID=ENSP0000303279;IARS2;NM_018060;	IARS2				
10	1E+07	10_9755762	75	68	85	42	72	64	0.269511	1	52	57	34	23	55	29	0.04486397	1	54.5	21.0916368	1E+07	9780168	ID=ENSP0000303279;IARS2;NM_018060;	IARS2				
10	1E+07	10_98138	3535	2255	3751	3214	2895	3483	0.600887	1	2089	1458	1808	876	1774	1342	1.31E-07	2.67E-05	2373.3	1031.53474								
10	1E+07	10_99193	102	130	90	129	116	110	0.277703	1	236	85	164	63	161	114	0.0873739	1	124.88	54.8776236								
10	1E+07	10_9942534	302	265	292	304	284	298	0.405216	1	49	94	92	122	72	107	0.0075808	0.3168642	190	110.125902					9941981	9942704	0.1316	ID=promoter_05527;
10	1E+07	10_99565	13	27	17	13	20	15	0.418324	1	19	79	35	35	49	35	0.49765999	1	29.75	21.8026866								
10	1E+07	10_9971893	0	13	15	0	7	8	0.998739	1	0	12	1	0	6	1	0.20114407	1	5.125	6.85435211					9971234	9972331	0.13527	ID=promoter_05528;
10	1E+07	10_10096003	15	31	2	11	23	7	0.003444	0.1741	1	18	9	0	10	5	0.51906437	1	10.875	10.4940459								
10	1E+07	10_10193900	2	23	12	14	13	13	0.920459	1	23	6	35	4	15	20	0.56420259	1	14.875	11.3947043								
10	1E+07	10_10241194	28	188	36	20	108	28	5.56E-14	#######	79	56	79	41	68	60	0.96183266	1	65.875	53.9932205								
10	1E+07	10_10297322	3677	2631	1869	1882	3154	1876	########	#######	3711	2533	3037	1109	3122	2073	3.90E-31	4.25E-28	2556.1	914.036173					1E+07	1E+07	0.44665	ID=promoter_05559;
10	1E+07	10_10468553	51	51	33	59	51	46	0.431965	1	94	36	71	13	65	42	0.18631934	1	51	24.8020736								
10	1E+07	10_10545931	12	49	69	56	31	63	0.040037	1	33	8	42	39	21	41	0.01771749	0.6168629	38.5	20.7777353								
10	1E+07	10_10689728	143	88	149	96	116	123	0.737612	1	53	7	37	3	30	20	0.51779033	1	72	56.535703								
10	1E+07	10_10741674	36	70	51	35	53	43	0.195695	1	57	69	55	41	63	48	0.59100593	1	51.75	13.6982793								
10	1E+07	10_10886761	71	95	32	47	83	40	1.12E-05	0.00132	128	159	109	34	144	72	7.93E-05	0.0075686	84.375	46.3648189								
10	1E+07	10_10982761	36	14	41	49	25	45	0.223918	1	14	21	17	5	18	11	0.54801163	1	24.625	15.4636671								
10	1E+07	10_11124395	0	42	4	5	21	5	0.001802	0.10311	9	0	11	15	5	13	0.12287893	1	10.75	13.666957								
10	1E+07	10_11180457	11	6	10	3	9	7	0.713906	1	41	5	8	0	23	4	0.00249416	0.131314	10.5	12.8396707								
10	1E+07	10_11214050	12	1	11	6	7	9	0.983538	1	21	16	5	32	19	19	0.97309647	1	13	9.97138764								
10	1E+07	10_11230774	33	16	10	32	25	21	0.526204	1	33	47	18	11	40	15	0.0083825	0.3419228	25	13.1583759								
10	1E+07	10_11291446	8	40	12	3	24	8	0.004476	0.21437	0	14	42	40	7	41	1.54E-06	0.000244	19.875	17.7959666								
10	1E+07	10_11339085	13	56	40	26	35	33	0.665929	1	21	16	64	20	19	42	0.0052053	0.236772	32	19.2056539								
10	1E+07	10_11436749	11	1	7	4	6	6	0.962469	1	1	8	2	20	5	11	0.25523295	1	6.75	6.4531277					1.1E+07	1.1E+07	0.48371	ID=promoter_05625;
10	1E+07	10_11440393	15	29	2	4	22	3	1.41E-04	0.01198	26	1	14	31	14	23	0.23132274	1	15.25	12.3027291								
10	1E+07	10_11509792	168	74	151	215	121	183	0.093904	1	100	83	53	23	92	38	9.35E-05	0.0087085	108.38	64.3560132					1.2E+07	1.2E+07	0.3529	ID=promoter_05635;
10	1E+07	10_11577923	4	30	1	7	17	4	0.005755	0.26328	25	12	19	7	19	13	0.6985193	1	13.125	10.4667842	1E+07	1.2E+07	ID=ENSP0000339161;AIDA;NM_022831;	AIDA	1.2E+07	1.2E+07	0.18446	ID=promoter_05646;
10	1E+07	10_11644543	126	70	82	120	98	101	0.659137	1	71	21	11	7	46	9	1.51E-05	0.0018118	63.5	46.7424555								
10	1E+07	10_11655140	6	20	13	3	13	8	0.344217	1	0	23	31	3	12	17	0.52622129	1	12.375	11.2622949					1.2E+07	1.2E+07	0.20614	ID=promoter_05651;
10	1E+07	10_11714715	8	8	4	5	8	5	0.532535	1	21	6	6	10	14	8	0.54462139	1	8.5	5.39841247								
10	1E+07	10_11717965	699	349	594	502	524	548	0.171144	1	406	255	435	131	331	283	0.60669646	1	421.38	181.67234					1.2E+07	1.2E+07	0.27515	ID=promoter_05654;
10	1E+07	10_11729507	90	68	48	86	79	67	0.146178	1	114	69	25	21	92	23	1.25E-08	3.04E-06	65.125	32.3306025								

10	1E+07	10_11776949	7	0	0	9	4	5	0.995512	1	28	1	0	0	15	0	9.56E-04	0.060345	5.625	9.72386607					1.2E+07	1.2E+07	0.62471	ID=promoter_05660;
10	1E+07	10_11779188	268	50	141	146	159	144	0.078782	1	7	27	12	7	17	10	0.5154613	1	82.25	94.3939314								
10	1E+07	10_11801184	17	27	25	17	22	21	0.795425	1	0	4	5	19	2	12	0.01895406	0.6519669	14.25	10.0676285								
10	1E+07	10_11847932	14	10	29	7	12	18	0.803442	1	5	0	24	7	3	16	0.00701108	0.2971393	12	9.88505365					1.2E+07	1.2E+07	0.16733	ID=promoter_05666;
10	1E+07	10_11921197	27	129	25	36	78	31	6.10E-07	#######	114	70	155	52	92	104	0.3815542	1	76	50.2962651								
10	1E+07	10_12085315	4	10	13	0	7	7	0.956352	1	56	17	42	38	37	40	0.79650037	1	22.5	20.2414003								
10	1E+07	10_12146165	1	5	6	18	3	12	0.129494	1	21	4	19	1	13	10	0.90601506	1	9.375	8.46737436								
10	1E+07	10_12168214	14	43	7	26	29	17	0.068354	1	2	10	38	14	6	26	8.76E-04	0.0569571	19.25	14.8780759								
10	1E+07	10_12243380	3	7	7	0	5	4	0.848147	1	46	13	2	1	30	2	1.33E-05	0.0016203	9.875	15.1980967								
10	1E+07	10_12249369	7	8	13	11	8	12	0.864244	1	18	11	0	4	15	2	0.01114184	0.4293828	9	5.5549206								
10	1E+07	10_13334688	255	91	211	260	173	236	0.319681	1	55	53	99	1	54	50	0.9999811	1	128.13	99.8104453	1E+07	1.3E+07	ID=ENSP0000310003-D2;CNIH2;NM_182553;	CNIH2	1.3E+07	1.3E+07	0.19321	ID=promoter_05695;
10	1E+07	10_13343971	21	52	8	16	37	12	1.88E-04	0.01539	4	5	6	8	5	7	0.77775394	1	15	16.0623784	1E+07	1.3E+07	ID=ENSP0000310003-D2;CNIH2;NM_182553;	CNIH2				
10	1E+07	10_13359620	22	1	7	7	12	7	0.325249	1	29	2	4	1	16	3	0.01878845	0.6479877	9.125	10.548358	1E+07	1.3E+07	ID=ENSP0000310003-D2;CNIH2;NM_182553;	CNIH2				
10	1E+07	10_13499056	0	20	5	6	10	6	0.41703	1	5	2	10	5	4	8	0.43697044	1	6.625	6.13974173					1.3E+07	1.3E+07	0.11016	ID=promoter_05700;
10	1E+07	10_13805117	22	50	24	10	36	17	0.005986	0.27181	9	54	27	5	32	16	0.1226614	1	25.125	18.3648539								
10	1E+07	10_13841550	6	20	15	16	13	16	0.991731	1	58	17	20	16	38	18	0.05735612	1	21	15.5747048								
10	1E+07	10_13980023	1	6	6	6	4	6	0.929647	1	17	3	11	11	10	11	0.92604858	1	7.625	5.12521777	1E+07	1.4E+07	ID=ENSP0000355800;TMEM63A;NM_014698;	TMEM63A				
10	1E+07	10_13995873	7	7	7	5	7	6	0.851931	1	16	1	14	3	9	9	0.98716474	1	7.5	5.12695956	1E+07	1.4E+07	ID=ENSP0000355800;TMEM63A;NM_014698;	TMEM63A				
10	1E+07	10_14127173	71	80	154	95	76	125	0.065448	1	0	0	8	0	0	4	0.11562589	1	51	57.8643734								
10	1E+07	10_14151806	15	5	32	16	10	24	0.15142	1	13	0	23	34	7	29	5.05E-04	0.0357488	17.25	11.9731843								
10	1E+07	10_14253252	118	28	40	26	73	33	1.46E-05	0.00166	64	39	31	9	52	20	0.00258053	0.1348666	44.375	33.5726823								
10	1E+07	10_14279960	10	5	7	7	8	7	0.850897	1	0	2	10	9	1	10	0.01766414	0.6154554	6.25	3.69362385								
10	1E+07	10_14340540	27	45	88	27	36	58	0.323582	1	38	0	0	0	19	0	1.50E-04	0.0128633	28.125	30.1019102								
10	1E+07	10_14410297	7	11	15	8	9	12	0.95859	1	15	19	11	15	17	13	0.87203467	1	12.625	4.06860805								
10	1E+07	10_14792504	30	75	57	36	53	47	0.37511	1	0	18	8	2	9	5	0.6500913	1	28.25	26.9324552								
10	1E+07	10_14992528	1	15	4	7	8	6	0.705675	1	21	4	5	19	13	12	0.99994263	1	9.5	7.6718409								
10	2E+07	10_15093604	268	1496	335	367	882	351	7.03E-71	#######	1424	1050	1932	794	1237	1363	6.13E-05	0.0060073	958.25	621.535598								
10	2E+07	10_15119533	5	17	9	21	11	15	0.930409	1	11	8	12	2	10	7	0.84912739	1	10.625	6.16296543								

10	2E+07	10_15303498	18	60	25	20	39	23	0.028526	0.93503	108	34	71	25	71	48	0.23709981	1	45.125	31.94834								
10	2E+07	10_15559564	98	15	39	63	57	51	0.370605	1	17	10	13	54	14	34	0.0069754	0.2965071	38.625	31.3001027								
10	2E+07	10_15713731	46	30	53	32	38	43	0.987075	1	41	5	35	19	23	27	0.70626281	1	32.625	15.2403365								
10	2E+07	10_15810634	13	16	19	11	15	15	0.908797	1	35	0	4	4	18	4	0.01933304	0.6616555	12.75	11.1066261								
10	2E+07	10_15912058	23	84	23	30	54	27	9.59E-04	0.06099	41	63	69	9	52	39	0.60511397	1	42.75	26.4183378								
10	2E+07	10_16086515	16	29	20	18	23	19	0.524147	1	59	61	37	1	60	19	1.06E-04	0.0096524	30.125	21.155462								
10	2E+07	10_16120213	41	52	46	34	47	40	0.325806	1	49	55	31	74	52	53	0.8886612	1	47.75	13.5198267	2E+07	1.6E+07	ID=ENSP00000375719;AKT3;;	AKT3				
10	2E+07	10_16177065	3368	1820	3605	2458	2594	3032	0.991455	1	2142	1475	1572	869	1809	1221	8.39E-17	4.73E-14	2163.6	943.667759	2E+07	1.6E+07	ID=ENSP00000375719;AKT3;;	AKT3				
10	2E+07	10_16339243	5	27	21	8	16	15	0.822707	1	0	21	6	31	11	19	0.24832023	1	14.875	11.5069855								
10	2E+07	10_16425412	62	88	50	65	75	58	0.056186	1	35	19	64	47	27	56	0.00196435	0.107958	53.75	21.0153005								
10	2E+07	10_16517617	48	42	2	0	45	1	1.62E-11	#######	56	29	9	0	43	5	1.17E-06	0.000192	23.25	23.3161501								
10	2E+07	10_16647483	0	25	6	9	13	8	0.344217	1	13	6	27	33	10	30	0.00304531	0.1540813	14.875	11.921619								
10	2E+07	10_16659626	61	61	18	19	61	19	4.07E-07	#######	24	34	16	28	29	22	0.77701989	1	32.625	18.4540936								
10	2E+07	10_16685277	14	10	5	28	12	17	0.88186	1	7	0	6	0	4	3	0.96209564	1	8.75	9.08295106								
10	2E+07	10_16761543	6	29	10	3	18	7	0.033499	1	0	6	3	1	3	2	0.9363008	1	7.25	9.34650431								
10	2E+07	10_16797100	1	44	0	10	23	5	6.40E-04	0.04366	8	2	6	18	5	12	0.17891578	1	11.125	14.5153859	2E+07	1.7E+07	ID=ENSP00000331260;ADSSL1;NM_152328;	ADSSL1				
10	2E+07	10_16903828	20	0	0	1	10	1	0.011548	0.45484	3	16	15	21	10	18	0.2304087	1	9.5	9.33503386	2E+07	1.7E+07	ID=ENSP00000355489;C1orf101;;	C1orf101				
10	2E+07	10_16938423	194	109	229	159	152	194	0.737785	1	132	54	74	0	93	37	4.32E-05	0.0044892	118.88	75.5975009					1.7E+07	1.7E+07	0.52863	ID=promoter_05788;
10	2E+07	10_16961966	11	8	14	5	10	10	0.938234	1	0	10	0	0	5	0	0.09850879	1	6	5.58057857								
10	2E+07	10_17049954	18	15	18	22	17	20	0.999959	1	19	0	21	16	10	19	0.1719433	1	16.125	6.91659495								
10	2E+07	10_17194997	226	144	177	167	185	172	0.089138	1	69	47	43	36	58	40	0.35228154	1	113.63	73.5428689					1.7E+07	1.7E+07	0.58401	ID=promoter_05797;
10	2E+07	10_17374387	438	224	822	523	331	673	1.12E-15	#######	370	289	521	145	330	333	0.5508112	1	416.5	212.583429								
10	2E+07	10_17443450	34	43	10	16	39	13	1.52E-04	0.01277	99	75	88	34	87	61	0.24474733	1	49.875	33.3528217								
10	2E+07	10_17475638	22	25	34	38	24	36	0.645518	1	26	45	34	44	36	39	0.79640057	1	33.5	8.65200225								
10	2E+07	10_17488574	0	14	16	19	7	18	0.195146	1	4	1	10	14	3	12	0.04708211	1	9.75	7.22594532								
10	2E+07	10_17771870	8	10	5	0	9	3	0.131542	1	0	4	20	1	2	11	0.03110435	0.956324	6	6.74007206								
10	2E+07	10_17837665	19	34	3	34	27	19	0.225928	1	50	6	35	15	28	25	0.9907189	1	24.5	16.3270153	2E+07	1.8E+07	ID=ENSP00000385545;KIF26B;NM_018012;	KIF26B				
10	2E+07	10_17856105	2	56	12	13	29	13	0.011304	0.44898	16	34	9	35	25	22	0.9842524	1	22.125	17.9478411	2E+07	1.8E+07	ID=ENSP00000385545;KIF26B;NM_018012;	KIF26B				
10	2E+07	10_18146270	93	8	136	56	51	96	0.022467	0.77206	586	15	391	275	301	333	0.083997	1	195	206.42259								
10	2E+07	10_18309040	50	55	145	202	53	174	6.31E-11	#######	45	76	137	49	61	93	0.00953103	0.3792832	94.875	58.9368609								

10	2E+07	10_18348572	280	87	261	175	184	218	0.99499	1	0	8	2	0	4	1	0.45332778	1	101.63	120.80792								
10	2E+07	10_18424427	1	2	9	1	2	5	0.651182	1	14	8	1	20	11	11	0.98433491	1	7	7.13141941								
10	2E+07	10_18433118	13	13	0	0	13	0	4.88E-04	0.03443	1	0	7	19	1	13	0.0036419	0.1775497	6.625	7.53918336								
10	2E+07	10_18462396	0	5	15	6	3	11	0.181935	1	1	13	25	2	7	14	0.23485029	1	8.375	8.65097022								
10	2E+07	10_18486329	22	36	3	10	29	7	1.77E-04	0.01461	0	0	8	0	0	4	0.11562589	1	9.875	12.9442486								
10	2E+07	10_18616987	28	54	6	15	41	11	1.27E-05	0.00148	42	19	22	1	31	12	0.02977149	0.9232309	23.375	17.7276016								
10	2E+07	10_18649347	4	1	0	0	3	0	0.172143	1	31	1	29	0	16	15	0.99951315	1	8.25	13.4986772								
10	2E+07	10_18689726	2	49	27	10	26	19	0.285911	1	43	24	31	21	34	26	0.76044447	1	25.875	15.606203								
10	2E+07	10_18998179	0	0	1	8	0	5	0.118674	1	20	2	4	23	11	14	0.73202476	1	7.25	9.20791585	2E+07	1.9E+07	ID=ENSP0000355843;SUSD4;;	SUSD4				
10	2E+07	10_19220314	259	152	101	134	206	118	1.85E-09	#######	94	128	50	79	111	65	0.01224932	0.4636779	124.63	63.3041129								
10	2E+07	10_19225404	1665	608	527	493	1137	510	5.57E-77	#######	684	382	192	124	533	158	5.06E-39	7.22E-36	584.38	477.603224					1.9E+07	1.9E+07	0.19511	ID=promoter_05867;
10	2E+07	10_19236711	1539	347	483	453	943	468	3.79E-54	#######	455	276	157	47	366	102	6.55E-29	6.78E-26	469.63	458.741427					1.9E+07	1.9E+07	0.20476	ID=promoter_05868;
10	2E+07	10_20170356	5	59	20	35	32	28	0.524213	1	67	46	19	1	57	10	3.83E-07	7.10E-05	31.5	24.377976								
10	2E+07	10_20439869	50	62	44	7	56	26	2.78E-04	0.02161	69	35	2	9	52	6	6.39E-08	1.38E-05	34.75	26.0205962								
10	2E+07	10_20822045	2	59	20	4	31	12	0.002785	0.14699	7	19	34	1	13	18	0.54030568	1	18.25	20.0124961								
10	2E+07	10_20898670	5290	5405	4576	5568	5348	5072	2.37E-26	#######	4366	3889	7728	2578	4128	5153	4.10E-45	7.23E-42	4925	1495.2243					2.1E+07	2.1E+07	0.45842	ID=promoter_05892;
10	2E+07	10_21017199	22	28	30	40	25	35	0.795408	1	50	6	26	14	28	20	0.67114862	1	27	13.8564065	2E+07	2.1E+07	ID=ENSP0000375751;TP53BP2;;	TP53BP2	2.1E+07	2.1E+07	0.11579	ID=promoter_05894;
10	2E+07	10_21051775	26	106	30	51	66	41	0.005078	0.23773	89	113	138	45	101	92	0.99258978	1	74.75	42.2095115								
10	2E+07	10_21123834	500	513	544	611	507	578	0.896364	1	419	350	266	91	385	179	7.15E-14	3.38E-11	411.75	170.385907	2E+07	2.1E+07	ID=ENSP0000355827;FBXO28;NM_015176;	FBXO28	2.1E+07	2.1E+07	0.38053	ID=promoter_05901;
10	2E+07	10_21163785	12	28	23	16	20	20	0.880283	1	0	0	18	1	0	10	0.00454609	0.2107253	12.25	10.9381377								
10	2E+07	10_21208071	5	16	7	11	11	9	0.722819	1	64	17	23	41	41	32	0.76585638	1	23	20.0499377								
10	2E+07	10_21274563	45	52	17	16	49	17	2.54E-05	0.00269	121	48	72	53	85	63	0.402684	1	53	33.2522824								
10	2E+07	10_21374328	46	147	33	21	97	27	5.72E-12	#######	64	51	60	38	58	49	0.89170578	1	57.5	38.8145775								
10	2E+07	10_21449068	18	40	17	11	29	14	0.018844	0.67198	46	25	39	3	36	21	0.23487	1	24.875	15.3756533	2E+07	2.2E+07	ID=ENSP0000356370;CRB1;NM_201253;	CRB1	2.1E+07	2.1E+07	0.17257	ID=promoter_05913;
10	2E+07	10_21468473	3	38	10	20	21	15	0.333352	1	12	28	32	44	20	38	0.0295645	0.9232309	23.375	14.4907606	2E+07	2.2E+07	ID=ENSP0000356370;CRB1;NM_201253;	CRB1				
10	2E+07	10_21808951	5	46	15	37	26	26	0.847245	1	20	51	38	45	36	42	0.59776306	1	32.125	16.685644								
10	2E+07	10_21873306	2	26	30	1	14	16	0.99748	1	35	9	35	31	22	33	0.21053573	1	21.125	14.6525229								
10	2E+07	10_21987530	5	53	13	26	29	20	0.181062	1	114	66	33	44	90	39	2.60E-04	0.0203864	44.25	34.623485					2.2E+07	2.2E+07	0.47745	ID=promoter_05935;
10	2E+07	10_21987760	195	40	154	198	118	176	0.128877	1	96	106	40	44	101	42	4.30E-05	0.004475	109.13	66.8333482					2.2E+07	2.2E+07	0.47745	ID=promoter_05935;

10	2E+07	10_22767821	46	60	101	26	53	64	0.987828	1	195	99	143	79	147	111	0.2630238	1	93.625	54.7512231								
10	2E+07	10_22774377	0	10	3	1	5	2	0.402696	1	14	12	9	0	13	5	0.22538616	1	6.125	5.74300817								
10	2E+07	10_22864516	44	69	8	0	57	4	1.02E-12	#######	50	25	34	2	38	18	0.05735612	1	29	24.8366089								
10	2E+07	10_22992923	22	15	4	5	19	5	0.004955	0.23359	63	25	31	1	44	16	0.00398908	0.1893524	20.75	20.2184498								
10	2E+07	10_23386889	127	70	108	102	99	105	0.77221	1	17	23	28	0	20	14	0.72101732	1	59.375	48.5443464								
10	2E+07	10_23968806	6	26	3	1	16	2	0.001284	0.07728	0	19	10	20	10	15	0.49251655	1	10.625	9.85520167								
10	2E+07	10_24046441	34	107	25	29	71	27	1.04E-06	0.00016	59	95	44	42	77	43	0.02676542	0.8546242	54.375	30.7614671					2.4E+07	2.4E+07	0.19441	ID=promoter_05986;
10	2E+07	10_24143851	40	11	31	7	26	19	0.285911	1	31	1	6	16	16	11	0.74569574	1	17.875	14.2872321								
10	2E+07	10_24160292	2	2	4	1	2	3	0.964182	1	0	21	9	2	11	6	0.57179039	1	5.125	6.97828264								
10	2E+07	10_24218900	0	30	6	2	15	4	0.015825	0.58672	32	29	29	9	31	19	0.35951856	1	17.125	14.0452076								
10	2E+07	10_24353732	15	10	16	1	13	9	0.46931	1	2	0	25	2	1	14	0.00214405	0.1161796	8.875	9.14076427	2E+07	2.4E+07	ID=ENSP00000356256;TIMM17A;NM_006335;	TIMM17A				
10	2E+07	10_24463431	408	124	382	242	266	312	1	1	42	59	30	7	51	19	0.00210847	0.1150505	161.75	161.783586								
10	2E+07	10_24467429	8	11	7	5	10	6	0.41703	1	28	20	0	14	24	7	0.01749745	0.6144624	11.625	8.92728562								
10	2E+07	10_24471465	17	0	26	15	9	21	0.212308	1	0	0	13	0	0	7	0.02292696	0.7568963	8.875	10.2041658	2E+07	2.5E+07	ID=ENSP00000375825;LGR6;;	LGR6				
10	2E+07	10_24594711	10	0	9	2	5	6	0.999292	1	3	27	10	0	15	5	0.11779525	1	7.625	8.92728562								
10	2E+07	10_24641224	1	23	3	4	12	4	0.066899	1	28	15	9	2	22	6	0.01847254	0.6380171	10.625	10.3224789								
10	2E+07	10_24670344	35	11	16	34	23	25	0.965853	1	0	3	16	29	2	23	6.46E-05	0.0062789	18	13.4801441								
10	2E+07	10_24703931	5	24	16	22	15	19	0.975531	1	30	51	51	17	41	34	0.89086031	1	27	16.4750894								
10	2E+07	10_24718063	68	22	36	43	45	40	0.440667	1	26	0	10	0	13	5	0.22538616	1	25.625	23.2252172								
10	2E+07	10_24719281	12	0	15	23	6	19	0.087167	1	17	0	0	22	9	11	0.82669107	1	11.125	9.86244681								
10	2E+07	10_24759578	99	30	74	73	65	74	0.984703	1	30	11	33	20	21	27	0.52862919	1	46.25	31.4040489					2.5E+07	2.5E+07	0.6427	ID=promoter_06044;
10	2E+07	10_24811264	176	53	89	78	115	84	0.003897	0.19244	63	26	54	59	45	57	0.29081695	1	74.75	44.9308993								
10	2E+07	10_24812483	41	5	19	21	23	20	0.617116	1	22	32	19	0	27	10	0.0371409	1	19.875	13.1848127								
10	3E+07	10_25376258	6	15	6	4	11	5	0.189421	1	2	6	10	1	4	6	0.75330063	1	6.25	4.49602999								
10	3E+07	10_26101997	11	7	7	16	9	12	0.95859	1	131	30	58	43	81	51	0.09335794	1	37.875	41.8720415								
10	3E+07	10_26126209	11	0	3	7	6	5	0.851367	1	11	0	11	2	6	7	0.91781435	1	5.625	4.95515604					2.6E+07	2.6E+07	0.26836	ID=promoter_06071;
10	3E+07	10_26181142	699	500	432	544	600	488	1.20E-08	#######	292	294	116	108	293	112	1.83E-15	9.61E-13	373.13	208.464754								
10	3E+07	10_26188763	341	146	284	300	244	292	0.973608	1	105	108	139	85	107	112	0.6677108	1	188.5	102.281125					2.6E+07	2.6E+07	0.1426	ID=promoter_06077;
10	3E+07	10_26290299	16	2	24	15	9	20	0.269594	1	1	21	10	1	11	6	0.57179039	1	11.25	9.19238816								
10	3E+07	10_26333549	7	21	3	6	14	5	0.053576	1	14	7	14	3	11	9	0.96206785	1	9.375	6.34569595								
10	3E+07	10_26862637	14	27	1	12	21	7	0.0088	0.36783	0	26	20	28	13	24	0.12479476	1	16	11.2249722								
10	3E+07	10_27166089	7	6	10	5	7	8	0.998739	1	1	9	13	0	5	7	0.77775394	1	6.375	4.40575922								
10	3E+07	10_27189759	322	274	316	308	298	312	0.373509	1	104	29	24	41	67	33	0.01034754	0.4047811	177.25	139.413824					2.7E+07	2.7E+07	0.15387	ID=promoter_06096;

10	3E+07	10_27196880	5	0	19	15	3	17	0.020784	0.72593	0	1	0	3	1	2	0.81304206	1	5.375	7.46300399								
10	3E+07	10_27343750	52	42	62	62	47	62	0.831405	1	35	24	9	1	30	5	3.29E-04	0.0250403	35.875	23.1235038								
10	3E+07	10_27916242	242	147	247	301	195	274	0.155206	1	113	65	132	27	89	80	0.98014325	1	159.25	95.652272	3E+07	2.8E+07	ID=ENSP00000365159-D2;ISCA1;NM_030940;	ISCA1	2.8E+07	2.8E+07	0.52847	ID=promoter_06102;
10	3E+07	10_28167939	10	47	1	5	29	3	2.72E-06	0.00038	34	33	39	29	34	34	0.95236908	1	24.75	17.0775542								
10	3E+07	10_28503954	68	58	95	68	63	82	0.824426	1	36	47	49	45	42	47	0.67429171	1	58.25	18.6068344								
10	3E+07	10_28559177	13	28	2	0	21	1	2.00E-05	0.00219	28	6	2	20	17	11	0.64636287	1	12.375	11.6856139								
10	3E+07	10_28958111	10	5	5	11	8	8	0.950274	1	31	42	11	19	37	15	0.02098916	0.7098276	16.75	13.2745729								
10	3E+07	10_29119946	80	69	27	39	75	33	6.61E-06	0.00083	206	129	210	94	168	152	0.97665878	1	106.75	69.9586613								
10	3E+07	10_29126274	7	8	4	19	8	12	0.864244	1	14	6	7	18	10	13	0.72167512	1	10.375	5.78020019								
10	3E+07	10_29319305	71	36	47	42	54	45	0.234992	1	13	7	8	1	10	5	0.51906437	1	28.125	24.6833518					2.9E+07	2.9E+07	0.72505	ID=promoter_06115;
10	3E+07	10_29448241	10	30	2	1	20	2	1.33E-04	0.01141	0	0	8	0	0	4	0.11562589	1	6.375	10.3086302								
10	3E+07	10_30306128	25	142	29	39	84	34	2.93E-07	#######	145	88	64	98	117	81	0.12813878	1	78.75	47.8203483								
10	3E+07	10_30632489	9	6	31	31	8	31	0.006053	0.27365	15	44	16	41	30	29	0.98716634	1	24.125	14.5448813								
10	3E+07	10_30655790	196	97	196	131	147	164	0.907309	1	159	37	34	1	98	18	1.92E-11	6.96E-09	106.38	76.2363571					3.1E+07	3.1E+07	0.71538	ID=promoter_06135;
10	3E+07	10_30656456	75	25	79	80	50	80	0.224253	1	19	47	27	10	33	19	0.24811784	1	45.25	29.0553659					3.1E+07	3.1E+07	0.71538	ID=promoter_06135;
10	3E+07	10_30669646	14	8	0	2	11	1	0.006522	0.28949	29	23	6	0	26	3	2.53E-04	0.0200383	10.25	10.8857705								
10	3E+07	10_31074272	1084	411	1577	937	748	1257	4.93E-14	#######	391	449	365	165	420	265	5.15E-06	0.0007084	672.38	479.151314					3.1E+07	3.1E+07	0.38378	ID=promoter_06146;
10	3E+07	10_31177238	167	106	169	143	137	156	0.968443	1	15	53	85	19	34	52	0.07203607	1	94.625	62.2482071					3.1E+07	3.1E+07	0.25627	ID=promoter_06147;
10	3E+07	10_34127814	0	30	15	7	15	11	0.4902	1	6	4	20	5	5	13	0.12287893	1	10.875	10.034761								
10	4E+07	10_35700111	45	54	16	26	50	21	2.02E-04	0.01635	22	28	15	10	25	13	0.22997911	1	27	15.2783694								
10	4E+07	10_36253355	0	6	19	12	3	16	0.03035	0.98285	1	11	4	1	6	3	0.67473421	1	6.75	6.71352579								
10	4E+07	10_36388238	34	35	19	14	35	17	0.008714	0.36691	41	40	2	10	41	6	7.83E-06	0.001021	24.375	14.9755754								
10	4E+07	10_36435278	48	30	14	11	39	13	1.52E-04	0.01277	0	0	2	0	0	1	0.58312783	1	13.125	17.5127505								
10	4E+07	10_36622706	68	68	143	85	68	114	0.064199	1	25	28	48	35	27	42	0.10529414	1	62.5	38.9321755								
10	4E+07	10_37528259	238	95	227	206	167	217	0.609568	1	98	51	52	13	75	33	0.00127663	0.0762043	122.5	88.3677381					3.8E+07	3.8E+07	0.48213	ID=promoter_06167;
10	4E+07	10_37548643	11	23	23	10	17	17	0.897282	1	15	20	20	38	18	29	0.17554477	1	20	8.84792147	4E+07	3.8E+07	ID=ENSP00000369339;C9orf72;NM_018325;	C9orf72				
10	4E+07	10_37783578	0	22	5	4	11	5	0.189421	1	5	18	1	8	12	5	0.30420563	1	7.875	7.95410945								
10	4E+07	10_37896967	28	29	18	21	29	20	0.181062	1	0	4	1	0	2	1	0.87709017	1	12.625	12.7159911								
10	4E+07	10_38105249	41	86	104	118	64	111	0.043077	1	53	131	108	42	92	75	0.71046483	1	85.375	35.6648587								
10	4E+07	10_38295868	0	11	3	10	6	7	0.999953	1	1	15	10	7	8	9	0.92351511	1	7.125	5.33017019								
10	4E+07	10_38346462	11	6	6	7	9	7	0.713906	1	0	35	24	23	18	24	0.50317852	1	14	11.9761668					3.8E+07	3.8E+07	0.15006	ID=promoter_06175;

10	4E+07	10_38413977	13	0	8	13	7	11	0.831726	1	38	17	17	0	28	9	0.01512239	0.5480081	13.25	12.0682582								
10	4E+07	10_38445687	98	45	14	11	72	13	7.59E-12	#######	67	17	9	4	42	7	1.33E-05	0.0016203	33.125	33.8966391					3.8E+07	3.8E+07	0.16438	ID=promoter_06179;
10	4E+07	10_38458351	27	162	17	38	95	28	3.34E-11	#######	159	113	105	74	136	90	0.04476573	1	86.875	57.1700159								
10	4E+07	10_38686169	39	45	8	28	42	18	9.64E-04	0.06126	40	20	18	0	30	9	0.00749313	0.313751	24.75	16.1134372								
10	4E+07	10_38868038	16	9	1	8	13	5	0.083134	1	0	16	7	1	8	4	0.59180275	1	7.25	6.40869944								
10	4E+07	10_38899126	0	15	6	1	8	4	0.357158	1	12	0	10	23	6	17	0.046525	1	8.375	8.22778394	4E+07	3.9E+07	ID=ENSP00002630 63;MTPAP ;NM_018109;	MTPAP				
10	4E+07	10_38991570	149	86	174	48	118	111	0.246996	1	117	90	31	27	104	29	9.92E-09	2.45E-06	90.25	54.110337								
10	4E+07	10_39166270	0	11	3	5	6	4	0.674789	1	28	27	7	7	28	7	0.00393447	0.1875084	11	10.6770783								
10	4E+07	10_39336902	4	29	35	56	17	46	0.010102	0.41206	11	33	28	10	22	19	0.97472654	1	25.75	16.9347487					3.9E+07	3.9E+07	0.72541	ID=promoter_06188;
10	4E+07	10_39518615	40	29	42	31	35	37	0.907308	1	84	5	15	2	45	9	2.27E-05	0.0025764	31	26.1533937								
10	4E+07	10_39647260	39	27	30	24	33	27	0.37765	1	89	36	64	76	63	70	0.5796554	1	48.125	24.7469334	4E+07	4E+07	ID=ENSP00003645 47;SVIL; NM_021738;	SVIL				
10	4E+07	10_39737888	274	79	218	176	177	197	0.879523	1	72	39	99	40	56	70	0.24656952	1	124.63	87.5637523					4E+07	4E+07	0.4057	ID=promoter_06198;
10	4E+07	10_39834146	12	17	5	2	15	4	0.015825	0.58672	19	27	1	0	23	1	9.32E-05	0.0086863	10.375	9.94179489					4E+07	4E+07	0.13275	ID=promoter_06200;
10	4E+07	10_39890241	37	84	20	34	61	27	6.68E-05	0.00626	60	88	49	57	74	53	0.35569714	1	53.625	23.8143864								
10	4E+07	10_39917960	43	131	32	69	87	51	3.19E-04	0.0244	106	60	104	104	83	104	0.12108928	1	81.125	35.0772108								
10	4E+07	10_40263451	13	7	17	9	10	13	0.970528	1	10	5	11	2	8	7	0.9937864	1	9.25	4.68279526								
10	4E+07	10_40522077	1	37	1	2	19	2	2.36E-04	0.01868	1	0	6	0	1	3	0.56022579	1	6	12.6716782								
10	4E+07	10_40597858	3	29	8	2	16	5	0.021336	0.74103	17	6	34	1	12	18	0.42746936	1	12.5	12.8173989								
10	4E+07	10_40623245	0	11	10	4	6	7	0.999953	1	12	15	12	7	14	10	0.81923661	1	8.875	4.91172068								
10	4E+07	10_40718178	31	17	64	65	24	65	0.001498	0.08861	16	0	7	13	8	10	0.81871774	1	26.625	24.9967855								
10	4E+07	10_41259187	16	48	4	15	32	10	4.55E-04	0.03273	28	0	7	12	14	10	0.81923661	1	16.25	15.4249334	4E+07	4.1E+07	ID=ENSP00002630 62;EPC1; NM_025209;	EPC1				
10	4E+07	10_41367607	113	87	54	25	100	40	1.26E-08	#######	70	78	47	48	74	48	0.15315945	1	65.25	27.5564356	4E+07	4.2E+07	ID=ENSP00003670 64;CUBN; NM_001081;	CUBN				
10	4E+07	10_41390813	42	94	42	39	68	41	0.002807	0.14801	42	74	24	24	58	24	0.00294193	0.1496995	47.625	24.2954287	4E+07	4.2E+07	ID=ENSP00003670 64;CUBN; NM_001081;	CUBN	4.1E+07	4.1E+07	0.35857	ID=promoter_06231;
10	4E+07	10_41498667	3	30	8	21	17	15	0.722447	1	25	6	36	13	16	25	0.25669436	1	17.75	12.0682582	4E+07	4.2E+07	ID=ENSP00003670 64;CUBN; NM_001081;	CUBN				
10	4E+07	10_41760786	4	0	0	0	2	0	0.309451	1	89	2	7	0	46	4	1.01E-07	2.09E-05	12.75	30.9134737								
10	4E+07	10_41938867	1	5	10	7	3	9	0.343967	1	80	11	21	5	46	13	3.24E-04	0.0246729	17.5	25.9449968								
10	4E+07	10_42001660	158	44	90	126	101	108	0.799706	1	22	35	67	34	29	51	0.01982417	0.6755494	72	48.9285193					4.2E+07	4.2E+07	0.59565	ID=promoter_06239;

10	4E+07	10_42063625	8	44	24	15	26	20	0.360218	1	1	16	34	14	9	24	0.01819934	0.631944	19.5	13.9795769								
10	4E+07	10_42229838	569	361	299	252	465	276	7.32E-19	#######	271	188	179	1	230	90	7.69E-12	2.89E-09	265	163.013584	4E+07	4.2E+07	ID=ENSP0000366746;STAM;NM_003473;	STAM				
10	4E+07	10_42288096	20	10	10	0	15	5	0.034023	1	40	16	11	20	28	16	0.29661245	1	15.875	11.7161366								
10	4E+07	10_42412491	46	58	15	24	52	20	4.78E-05	0.00467	15	33	42	6	24	24	0.96613769	1	29.875	17.9478411	4E+07	4.2E+07	ID=ENSP0000366586;SLC39A12;NM_001145195;	SLC39A12				
10	4E+07	10_42535710	26	16	3	15	21	9	0.03105	0.99404	25	1	4	23	13	14	0.92696813	1	14.125	10.2878222								
10	4E+07	10_42649314	5	1	1	5	3	3	0.981055	1	17	8	3	0	13	2	0.02644099	0.8480056	5	5.52914357								
10	4E+07	10_43132578	65	24	44	48	45	46	0.806124	1	74	43	25	39	59	32	0.04722228	1	45.25	17.4499284					4.3E+07	4.3E+07	0.27207	ID=promoter_06247;
10	4E+07	10_43963249	9	34	8	15	22	12	0.091688	1	74	14	59	22	44	41	0.99972002	1	29.375	24.6688786	4E+07	4.4E+07	ID=ENSP0000277632;C10orf97;NM_024948;	C10orf97				
10	4E+07	10_44204305	69	47	48	34	58	41	0.043015	1	18	40	39	11	29	25	0.96515949	1	38.25	18.0930136								
10	4E+07	10_44219230	1	37	7	0	19	4	0.002035	0.11403	64	35	120	44	50	82	0.00555909	0.2501552	38.5	40.03213								
10	4E+07	10_44244761	13	51	24	13	32	19	0.056624	1	53	89	76	41	71	59	0.82449967	1	45	28.0356915								
10	4E+07	10_44450207	36	27	27	10	32	19	0.056624	1	7	7	25	13	7	19	0.03889743	1	19	11.0711982								
10	4E+07	10_44520737	89	62	62	86	76	74	0.521112	1	86	26	19	41	56	30	0.04920085	1	58.875	27.7459778								
10	4E+07	10_44614974	41	44	13	5	43	9	7.71E-07	0.00012	33	32	56	2	33	29	0.97818147	1	28.25	19.5649103								
10	4E+07	10_44705524	77	33	129	80	55	105	0.012686	0.49233	76	58	48	17	67	33	0.01034754	0.4047811	64.75	34.3334489	4E+07	4.5E+07	ID=ENSP0000367623;HSPA14;NM_016299;	HSPA14				
10	4E+07	10_44919118	689	703	584	653	696	619	3.28E-06	0.00044	492	408	402	266	450	334	0.00857716	0.3486112	524.63	158.308864					4.5E+07	4.5E+07	0.64265	ID=promoter_06276;
10	4E+07	10_44944874	18	7	11	25	13	18	0.901125	1	24	17	8	1	21	5	0.01300804	0.4860836	13.875	8.52622006								
10	5E+07	10_45254482	8	4	6	17	6	12	0.558139	1	1	1	11	5	1	8	0.04988758	1	6.625	5.37022213								
10	5E+07	10_45277908	523	150	143	179	337	161	3.94E-21	#######	510	52	55	109	281	82	2.76E-21	1.99E-18	215.13	191.236157								
10	5E+07	10_45369997	81	22	27	69	52	48	0.486901	1	0	22	7	1	11	4	0.25131486	1	28.625	30.5049762								
10	5E+07	10_45452906	43	71	41	90	57	66	0.99745	1	52	27	44	9	40	27	0.44121895	1	47.125	24.9424337								
10	5E+07	10_46019229	29	14	2	0	22	1	1.12E-05	0.00132	32	8	18	2	20	10	0.26942782	1	13.125	12.414709								
10	5E+07	10_46024612	73	25	88	35	49	62	0.924371	1	0	14	19	25	7	22	0.01139713	0.4370832	34.875	30.1777472								
10	5E+07	10_46359224	57	32	69	68	45	69	0.374038	1	39	27	21	2	33	12	0.01587283	0.5667339	39.375	23.7543229								
10	5E+07	10_46808856	30	31	32	24	31	28	0.60342	1	31	2	20	3	17	12	0.76881979	1	21.625	12.5007143								
10	5E+07	10_46816898	15	37	25	30	26	28	0.95198	1	23	0	11	0	12	6	0.45526626	1	17.625	13.5429212								
10	5E+07	10_47206702	6	51	14	5	29	10	0.001829	0.10386	12	15	10	4	14	7	0.39930956	1	14.625	15.2684315								
10	5E+07	10_47248048	1	21	3	1	11	2	0.020291	0.71411	21	7	12	1	14	7	0.39930956	1	8.375	8.66746791								
10	5E+07	10_47263196	415	101	337	233	258	285	0.783657	1	91	74	80	11	83	46	0.01824981	0.6330806	167.75	144.14353					4.7E+07	4.7E+07	0.85239	ID=promoter_06309;
10	5E+07	10_47315318	8	1	16	25	5	21	0.023384	0.79837	37	37	19	2	37	11	0.00224181	0.1207582	18.125	14.2471802								

10	5E+07	10_47885224	9	82	28	9	46	19	3.31E-04	0.02521	36	41	44	3	39	24	0.28229872	1	31.5	25.7848239	5E+07	4.8E+07	ID=ENSP0000364884;MPP7;NM_173496;	MPP7				
10	5E+07	10_48161386	645	526	540	510	586	525	4.05E-05	0.00403	344	288	394	164	316	279	0.83836997	1	426.38	157.411143					4.8E+07	4.8E+07	0.38132	ID=promoter_06318;
10	5E+07	10_48453694	24	18	11	20	21	16	0.42382	1	97	30	59	29	64	44	0.30920511	1	36	28.4956137								
10	5E+07	10_48509592	11	62	17	46	37	32	0.448082	1	16	45	53	26	31	40	0.38158628	1	34.5	19.3243	5E+07	4.9E+07	ID=ENSP0000365604;ARHGAP21;NM_020824;	ARHGAP21				
10	5E+07	10_48522386	8	17	5	8	13	7	0.234546	1	1	0	12	0	1	6	0.13767373	1	6.375	6.11642986	5E+07	4.9E+07	ID=ENSP0000365604;ARHGAP21;NM_020824;	ARHGAP21				
10	5E+07	10_48651274	16	163	24	65	90	45	9.33E-06	0.00113	37	51	65	78	44	72	0.01079157	0.4173488	62.375	45.9500894								
10	5E+07	10_48881474	25	50	11	44	38	28	0.169685	1	36	10	54	21	23	38	0.08548704	1	31.375	17.1542373								
10	5E+07	10_48918015	142	31	37	28	87	33	4.42E-08	#######	75	30	10	30	53	20	0.00189061	0.1047116	47.875	42.2016841								
10	5E+07	10_48918782	11	19	5	22	15	14	0.827043	1	1	1	13	25	1	19	1.49E-04	0.0128238	12.125	9.34172667								
10	5E+07	10_48955570	175	104	193	148	140	171	0.938825	1	124	78	125	91	101	108	0.58696868	1	129.75	40.1417133								
10	5E+07	10_48996663	112	90	93	83	101	88	0.123224	1	76	12	36	22	44	29	0.35808073	1	65.5	36.9555718								
10	5E+07	10_49180347	0	10	11	25	5	18	0.065263	1	37	19	8	18	28	13	0.1098583	1	16	11.4392807								
10	5E+07	10_49286131	16	2	20	14	9	17	0.506269	1	1	10	10	3	6	7	0.91781435	1	9.5	7.01019666					4.9E+07	4.9E+07	0.79833	ID=promoter_06332;
10	5E+07	10_49348497	13	27	12	3	20	8	0.026297	0.87605	27	22	30	23	25	27	0.8592775	1	19.625	9.34936973					4.9E+07	4.9E+07	0.42873	ID=promoter_06333;
10	5E+07	10_49353303	26	43	62	38	35	50	0.669376	1	59	90	124	41	75	83	0.53820028	1	60.375	32.2708958								
10	5E+07	10_49818589	24	32	21	45	28	33	0.999827	1	1	0	16	15	1	16	7.41E-04	0.0492332	19.25	15.0404217								
10	5E+07	10_49892028	31	43	11	29	37	20	0.016661	0.61076	70	29	51	43	50	47	0.99766929	1	38.375	17.6549265	5E+07	5E+07	ID=ENSP0000365757;PIP4K2A;NM_005028;	PIP4K2A	5E+07	5E+07	0.15944	ID=promoter_06344;
10	5E+07	10_50162797	8	13	1	24	11	13	0.999831	1	2	4	19	16	3	18	0.00260948	0.1361302	10.875	8.45893441								
10	5E+07	10_50831478	3748	4167	4934	6728	3958	5831	1.94E-27	#######	4433	4019	8951	3014	4226	5983	1.35E-98	4.70E-95	4999.3	1930.58746					5.1E+07	5.1E+07	0.7818	ID=promoter_06358;
10	5E+07	10_50835751	101	31	47	63	66	55	0.170329	1	110	80	83	15	95	49	0.00328925	0.1648745	66.25	33.4696024					5.1E+07	5.1E+07	0.41701	ID=promoter_06359;
10	5E+07	10_50895086	20	6	18	27	13	23	0.490912	1	29	3	39	0	16	20	0.67029873	1	17.75	13.8331899								
10	5E+07	10_51035259	18	65	16	17	42	17	5.42E-04	0.03781	47	54	23	47	51	35	0.38844108	1	35.875	19.4967946								
10	5E+07	10_51257080	13	51	4	20	32	12	0.001806	0.10314	0	35	18	10	18	14	0.88543974	1	18.875	16.839049								
10	5E+07	10_51367160	5	22	23	6	14	15	0.970741	1	17	16	11	0	17	6	0.10713464	1	12.5	8.36660027								
10	5E+07	10_51384259	6	21	7	18	14	13	0.831264	1	47	33	49	0	40	25	0.29783938	1	22.625	18.7230759								
10	5E+07	10_51424359	7	13	14	8	10	11	0.989255	1	4	43	22	2	24	12	0.20726737	1	14.125	13.2819695								
10	5E+07	10_51955396	37	105	50	21	71	36	1.36E-04	0.01165	47	70	55	63	59	59	0.9187999	1	56	24.9284691	5E+07	5.2E+07	ID=ENSP0000366460;PLXDC2;NM_032812;	PLXDC2				
10	5E+07	10_52120963	298	132	235	175	215	205	0.104382	1	152	102	127	38	127	83	0.04510956	1	157.38	80.1996393					5.2E+07	5.2E+07	0.6041	ID=promoter_06378;

10	5E+07	10_53173616	11	39	29	5	25	17	0.214068	1	39	50	38	9	45	24	0.08598552	1	27.5	16.9199797								
10	5E+07	10_53212086	32	6	8	9	19	9	0.06806	1	2	3	21	32	3	27	2.63E-05	0.0029133	14.125	12.4606524								
10	5E+07	10_53300132	19	71	13	17	45	15	3.94E-05	0.00396	17	19	23	16	18	20	0.85572507	1	24.375	19.055839								
10	5E+07	10_53381741	1	39	7	27	20	17	0.618382	1	54	14	25	2	34	14	0.0317829	0.9755061	21.125	18.787819	5E+07	5.4E+07	ID=ENSP0000364001;NRP1;;	NRP1				
10	5E+07	10_53448063	54	62	109	59	58	84	0.465107	1	335	160	203	27	248	115	3.02E-09	8.17E-07	126.13	103.250649	5E+07	5.4E+07	ID=ENSP0000364001;NRP1;;	NRP1				
10	5E+07	10_53460292	0	20	10	5	10	8	0.719353	1	22	0	12	0	11	6	0.57179039	1	8.625	8.92728562	5E+07	5.4E+07	ID=ENSP0000364001;NRP1;;	NRP1	5.3E+07	5.3E+07	0.12769	ID=promoter_06397;
10	5E+07	10_53768511	11	78	17	16	45	17	1.50E-04	0.01267	39	44	29	35	42	32	0.70425168	1	33.625	21.4738213								
10	5E+07	10_53803123	59	7	38	47	33	43	0.901814	1	0	12	0	0	6	0	0.06196836	1	20.375	23.9221207								
10	5E+07	10_54063497	5	0	8	7	3	8	0.459247	1	1	12	13	1	7	7	0.99000271	1	5.875	5.02671435								
10	5E+07	10_54088748	11	25	6	6	18	6	0.017303	0.62786	22	5	16	15	14	16	0.84864027	1	13.25	7.59228932								
10	5E+07	10_54094643	6	20	8	2	13	5	0.083134	1	24	41	5	2	33	4	3.34E-05	0.003583	13.5	13.7944088								
10	5E+07	10_54210041	102	149	37	55	126	46	7.04E-12	#######	43	63	94	19	53	57	0.73828082	1	70.25	42.3143002								
10	5E+07	10_54282455	26	24	21	14	25	18	0.280499	1	41	15	29	1	28	15	0.22181264	1	21.375	11.8434732								
10	5E+07	10_54290173	5	33	3	17	19	10	0.108738	1	18	0	42	2	9	22	0.0389747	1	15	15.6021976								
10	5E+07	10_54337721	107	55	142	144	81	143	0.01249	0.48632	3	9	6	0	6	3	0.67473421	1	58.25	63.6457832					5.4E+07	5.4E+07	0.13851	ID=promoter_06426;
10	5E+07	10_54675304	19	18	2	23	19	13	0.317047	1	48	28	12	4	38	8	1.68E-04	0.0141854	19.25	14.6458185								
10	5E+07	10_54678438	34	3	16	11	19	14	0.411928	1	112	26	71	2	69	37	0.02472439	0.8037839	34.375	38.4593942					5.5E+07	5.5E+07	0.15184	ID=promoter_06449;
10	5E+07	10_54910217	384	190	252	289	287	271	0.037207	1	101	94	90	28	98	59	0.0306552	0.9480263	178.5	121.657599								
10	5E+07	10_54952797	203	91	197	172	147	185	0.816231	1	55	40	42	72	48	57	0.44805189	1	109	70.1101175								
10	5E+07	10_54982314	157	80	142	184	119	163	0.436483	1	111	99	190	115	105	153	0.00153913	0.0883233	134.75	40.0989847					5.5E+07	5.5E+07	0.24965	ID=promoter_06470;
10	6E+07	10_55011312	34	56	26	20	45	23	0.003847	0.19025	26	24	10	28	25	19	0.80752973	1	28	13.2664992	5E+07	5.5E+07	ID=ENSP0000363836;CCNY;NM_145012;	CCNY				
10	6E+07	10_55078580	21	56	16	12	39	14	3.02E-04	0.02327	39	23	68	11	31	40	0.38158628	1	30.75	21.4059404								
10	6E+07	10_55185792	79	108	69	76	94	73	0.028919	0.94726	12	31	40	52	22	46	0.00551602	0.2487658	58.375	30.682417								
10	6E+07	10_55449371	0	9	5	0	5	3	0.645778	1	26	0	0	0	13	0	0.00241566	0.1280305	5	9.11827051					5.5E+07	5.5E+07	0.11583	ID=promoter_06496;
10	6E+07	10_55870476	12	41	17	27	27	22	0.443332	1	92	19	57	36	56	47	0.88120181	1	37.625	26.4247584								
10	6E+07	10_55901128	1	13	10	8	7	9	0.983538	1	0	11	18	1	6	10	0.51486293	1	7.75	6.54107898								
10	6E+07	10_55982136	6	25	9	15	16	12	0.497978	1	24	20	10	23	22	17	0.8526593	1	16.5	7.50238057								
10	6E+07	10_56027683	62	52	93	74	57	84	0.411343	1	4	14	26	16	9	21	0.05613829	1	42.625	32.2531837								
10	6E+07	10_56028210	38	23	30	17	31	24	0.30595	1	7	17	7	10	12	9	0.89054267	1	18.625	11.1731502					5.6E+07	5.6E+07	0.68413	ID=promoter_06499;
10	6E+07	10_56068709	42	24	50	59	33	55	0.276422	1	47	0	12	18	24	15	0.48349198	1	31.5	20.9079616	6E+07	5.6E+07	ID=ENSP0000368102;CCDC3;NM_031455;	CCDC3				

10	6E+07	10_56070755	131	8	48	12	70	30	9.41E-06	0.00114	1	21	7	1	11	4	0.25131486	1	28.625	44.0938528	6E+07	5.6E+07	ID=ENSP0000368102;CCDC3;NM_031455;	CCDC3				
10	6E+07	10_56151583	49	7	58	68	28	63	0.014119	0.53474	0	0	0	0	0	0	1	1	22.75	29.994047								
10	6E+07	10_56167945	31	42	42	30	37	36	0.726365	1	173	45	86	103	109	95	0.9078275	1	69	49.6559592								
10	6E+07	10_56219860	16	31	53	24	24	39	0.450945	1	28	4	27	1	16	14	0.9876114	1	23	16.4750894	6E+07	5.6E+07	ID=ENSP0000368122;CAMK1D;NM_020397;	CAMK1D				
10	6E+07	10_56364902	1	11	3	4	6	4	0.674789	1	0	13	28	3	7	16	0.11989665	1	7.875	9.35700654	6E+07	5.6E+07	ID=ENSP0000368122;CAMK1D;NM_020397;	CAMK1D	5.6E+07	5.6E+07	0.17877	ID=promoter_06513;
10	6E+07	10_56371648	3243	1185	2320	2229	2214	2275	5.32E-05	0.00511	1739	1053	1335	689	1396	1012	1.80E-08	4.28E-06	1724.1	835.047892	6E+07	5.6E+07	ID=ENSP0000368122;CAMK1D;NM_020397;	CAMK1D				
10	6E+07	10_56581310	19	87	19	33	53	26	8.61E-04	0.05619	47	68	91	26	58	59	0.88147173	1	48.75	29.6587737					5.7E+07	5.7E+07	0.39228	ID=promoter_06530;
10	6E+07	10_56810005	12	49	7	5	31	6	2.44E-05	0.00261	1	9	15	4	5	10	0.35527535	1	12.75	15.3133928								
10	6E+07	10_56953502	55	19	154	28	37	91	5.28E-04	0.03699	19	4	4	5	12	5	0.30420563	1	36	50.6190252								
10	6E+07	10_57069909	1485	710	1677	1539	1098	1608	9.78E-08	#######	729	726	558	226	728	392	1.83E-17	1.09E-14	956.25	533.963549					5.7E+07	5.7E+07	0.57346	ID=promoter_06559;
10	6E+07	10_57486387	6	20	3	2	13	3	0.018372	0.65917	0	2	13	3	1	8	0.04988758	1	6.125	6.87516234	6E+07	5.7E+07	ID=ENSP0000368539;USP6NL;NM_014688;	USP6NL				
10	6E+07	10_57651318	1	30	4	8	16	6	0.042495	1	12	26	12	5	19	9	0.23949138	1	12.25	10.4846827	6E+07	5.8E+07	ID=ENSP0000368539-D3;USP6NL;NM_014688;	USP6NL	5.8E+07	5.8E+07	0.59576	ID=promoter_06624;
10	6E+07	10_57719016	53	146	59	47	100	53	9.95E-06	0.0012	170	85	145	54	128	100	0.43875196	1	94.875	50.5270437								
10	6E+07	10_57750110	65	32	66	36	49	51	0.836353	1	2	14	16	2	8	9	0.92351511	1	29.125	25.5646938					5.8E+07	5.8E+07	0.34175	ID=promoter_06645;
10	6E+07	10_57770419	89	135	143	143	112	143	0.797661	1	88	72	88	114	80	101	0.11694345	1	109	28.4454365					5.8E+07	5.8E+07	0.14448	ID=promoter_06650;
10	6E+07	10_57831267	42	30	22	37	36	30	0.380805	1	70	60	74	23	65	49	0.54902067	1	44.75	20.7071416								
10	6E+07	10_57903688	14	9	16	17	12	17	0.88186	1	1	28	11	13	15	12	0.93002578	1	13.625	7.670677								
10	6E+07	10_57933469	25	1	9	9	13	9	0.46931	1	0	26	0	27	13	14	0.92696813	1	12.125	12.0527116								
10	6E+07	10_57938325	30	0	9	10	15	10	0.374041	1	0	0	0	0	0	0	1	1	6.125	10.5618924								
10	6E+07	10_57972374	8	36	0	6	22	3	1.41E-04	0.01198	21	29	26	3	25	15	0.40601431	1	16.125	13.5376459					5.8E+07	5.8E+07	0.16285	ID=promoter_06682;
10	6E+07	10_57998770	0	27	11	5	14	8	0.253058	1	16	59	15	1	38	8	1.68E-04	0.0141854	16.75	19.2483766					5.8E+07	5.8E+07	0.24442	ID=promoter_06688;
10	6E+07	10_58010409	37	31	5	27	34	16	0.007736	0.3324	33	20	4	59	27	32	0.64141452	1	27	17.8805561								
10	6E+07	10_58040467	31	8	11	55	20	33	0.480879	1	1	14	36	0	8	18	0.09791732	1	19.5	19.3538479								
10	6E+07	10_58042815	13	0	28	4	7	16	0.324814	1	42	0	11	22	21	17	0.91718448	1	15	14.8227624								
10	6E+07	10_58345347	10	13	6	0	12	3	0.030625	0.98374	0	22	12	30	11	21	0.13989775	1	11.625	10.363914								
10	6E+07	10_58727516	26	27	14	13	27	14	0.038924	1	39	120	70	56	80	63	0.62497735	1	45.625	36.00372								

10	6E+07	10_58732826	640	368	633	538	504	586	0.989606	1	362	358	374	153	360	264	0.01494532	0.5435394	428.25	164.952591								
10	6E+07	10_59118750	24	45	17	23	35	20	0.032214	1	27	25	43	15	26	29	0.79045324	1	27.375	11.0316104								
10	6E+07	10_59135481	6	37	18	17	22	18	0.522468	1	27	33	45	31	30	38	0.43894807	1	26.75	12.5214102								
10	6E+07	10_59193585	0	1	7	2	1	5	0.353258	1	0	27	15	1	14	8	0.54462139	1	6.625	9.69443876								
10	6E+07	10_59592032	15	33	3	21	24	12	0.04556	1	16	32	38	2	24	20	0.93987967	1	20	13.5857068								
10	6E+07	10_59786875	13	25	25	50	19	38	0.157767	1	10	5	18	0	8	9	0.92351511	1	18.25	15.5999084								
10	6E+07	10_59895442	107	72	84	91	90	88	0.477621	1	47	52	57	33	50	45	0.98967312	1	67.875	24.9710547	6E+07	6E+07	ID=ENSP000003456 81-D2;GATA2;NM_032638;	GATA2				
10	6E+07	10_59955078	67	47	65	46	57	56	0.640968	1	33	8	23	31	21	27	0.52862919	1	40	20.2907438	6E+07	6E+07	ID=ENSP00003402 71;TAF3;NM_031923;	TAF3	6E+07	6E+07	0.15823	ID=promoter_06725;
10	6E+07	10_59988891	67	124	188	258	96	223	8.46E-08	#######	10	32	50	6	21	28	0.4487548	1	91.875	90.9512232								
10	6E+07	10_59995765	0	10	19	35	5	27	0.002605	0.1394	0	19	2	1	10	2	0.09294023	1	10.75	12.6913919								
10	6E+07	10_60024754	4	30	3	1	17	2	7.31E-04	0.04855	8	2	6	0	5	3	0.8350419	1	6.75	9.75045786					6E+07	6E+07	0.17247	ID=promoter_06738;
10	6E+07	10_60104312	11	51	1	23	31	12	0.002785	0.14699	39	11	20	11	25	16	0.50792242	1	20.875	16.5998064								
10	6E+07	10_60144152	9	0	8	4	5	6	0.999292	1	11	4	28	0	8	14	0.34554715	1	8	9.02377811								
10	6E+07	10_60179518	31	42	27	16	37	22	0.03657	1	9	73	32	15	41	24	0.19624561	1	30.625	20.2339001								
10	6E+07	10_60194242	26	2	18	9	14	14	0.914609	1	28	6	17	9	17	13	0.87203467	1	14.375	9.42545944								
10	6E+07	10_60228014	14	32	33	53	23	43	0.190522	1	36	62	37	24	49	31	0.24588806	1	36.375	15.2027958								
10	6E+07	10_60294917	9	0	7	5	5	6	0.999292	1	11	6	7	0	9	4	0.4571786	1	5.625	3.92564826								
10	6E+07	10_60304510	14	44	21	5	29	13	0.011304	0.44898	61	46	44	12	54	28	0.04072278	1	30.875	20.3149312					6E+07	6E+07	0.14428	ID=promoter_06786;
10	6E+07	10_60306718	19	38	43	35	29	39	0.855783	1	65	44	56	31	55	44	0.76644609	1	41.375	14.352078								
10	6E+07	10_60315298	13	56	5	21	35	13	9.22E-04	0.05911	38	26	47	6	32	27	0.9371194	1	26.5	18.9661352								
10	6E+07	10_60337649	9	41	34	8	25	21	0.526204	1	7	5	6	62	6	34	1.84E-05	0.0021552	21.5	21.5074738					6E+07	6E+07	0.58964	ID=promoter_06794;
10	6E+07	10_60344487	0	38	6	7	19	7	0.021688	0.74727	41	41	16	26	41	21	0.0807285	1	21.875	16.8814141					6E+07	6E+07	0.17165	ID=promoter_06796;
10	6E+07	10_60348716	11	10	4	6	11	5	0.189421	1	28	4	18	12	16	15	0.99951315	1	11.625	8.10533334					6E+07	6E+07	0.35719	ID=promoter_06797;
10	6E+07	10_60400471	23	89	30	14	56	22	2.95E-05	0.00306	1	12	36	5	7	21	0.01731975	0.610307	26.25	28.0293214								
10	6E+07	10_60450852	45	50	19	27	48	23	0.001283	0.07728	3	44	22	63	24	43	0.0312312	0.959604	34.125	19.6282413								
10	6E+07	10_60480109	18	4	13	23	11	18	0.682529	1	0	16	0	0	8	0	0.02452223	0.7977568	9.25	9.33120724								
10	6E+07	10_60541726	0	39	4	0	20	2	1.33E-04	0.01141	8	0	8	2	4	5	0.90483023	1	7.625	13.1141962								
10	6E+07	10_60633606	83	37	53	91	60	72	0.991532	1	544	195	299	92	370	196	1.14E-09	3.32E-07	174.25	172.489958								
10	6E+07	10_60644028	536	380	801	498	458	650	0.007491	0.32295	192	101	166	95	147	131	0.94743823	1	346.13	252.565939								
10	6E+07	10_60879279	7	23	15	15	15	15	0.908797	1	0	28	9	22	14	16	0.84864027	1	14.875	9.3110917								
10	6E+07	10_60951309	44	114	72	49	79	61	0.047234	1	75	93	70	89	84	80	0.98521335	1	75.75	23.0139709								
10	6E+07	10_61120302	0	6	13	1	3	7	0.596562	1	15	15	1	0	15	1	0.00364468	0.1775497	6.375	6.88554386								

10	6E+07	10_61375232	20	50	103	130	35	117	9.44E-08	#######	45	188	273	119	117	196	1.84E-06	0.0002887	116	83.512189				
10	6E+07	10_61382483	127	230	171	265	179	218	0.932926	1	165	182	234	112	174	173	0.80850657	1	185.75	53.6116991				
10	6E+07	10_61426161	17	1	12	20	9	16	0.603365	1	16	3	0	15	10	8	0.95278883	1	10.5	7.94624799				
10	6E+07	10_61479440	0	20	2	0	10	1	0.011548	0.45484	41	5	3	0	23	2	3.18E-04	0.0243067	8.875	14.5841156	6.1E+07	6.1E+07	0.1947	ID=promoter_06911;
10	6E+07	10_61482326	22	18	16	20	20	18	0.715835	1	1	36	9	24	19	17	0.99437087	1	18.25	10.3751076	6.1E+07	6.1E+07	0.63165	ID=promoter_06912;
10	6E+07	10_61548856	14	0	4	3	7	4	0.503048	1	3	7	20	4	5	12	0.17891578	1	6.875	6.7281392				
10	6E+07	10_61592364	1022	663	983	1086	843	1035	0.616019	1	712	352	442	226	532	334	1.40E-07	2.84E-05	685.75	326.23425	6.2E+07	6.2E+07	0.78635	ID=promoter_06926;
10	6E+07	10_61597934	0	2	0	11	1	6	0.241978	1	0	1	5	30	1	18	2.55E-04	0.0200383	6.125	10.3570197				
10	6E+07	10_61603378	5	28	9	3	17	6	0.027279	0.89953	0	22	13	19	11	16	0.51039395	1	12.375	9.91301453	6.2E+07	6.2E+07	0.14716	ID=promoter_06928;
10	6E+07	10_61711259	82	27	150	111	55	131	3.98E-05	0.00399	5	11	27	30	8	29	0.00112412	0.0691941	55.375	52.8040515				
10	6E+07	10_61716173	51	31	6	22	41	14	1.22E-04	0.01067	0	4	0	0	2	0	0.39572182	1	14.25	18.7977962				
10	6E+07	10_61760774	26	48	67	51	37	59	0.339357	1	28	29	15	24	29	20	0.5935331	1	36	17.4355958				
10	6E+07	10_61829312	9	10	10	5	10	8	0.719353	1	15	7	38	28	11	33	0.00170606	0.0957908	15.25	11.6343089				
10	6E+07	10_61890525	7	7	3	2	7	3	0.314307	1	2	12	9	2	7	6	0.99011599	1	5.5	3.81725406				
10	6E+07	10_62100550	34	25	29	12	30	21	0.185877	1	200	71	111	65	136	88	0.0304657	0.943392	68.375	62.0505266				
10	6E+07	10_62186727	20	73	28	61	47	45	0.621663	1	62	125	167	64	94	116	0.11779074	1	75	48.8262225				
10	6E+07	10_62282118	8	14	2	0	11	1	0.006522	0.28949	8	8	9	7	8	8	0.98858271	1	7	4.30945804				
10	6E+07	10_62352742	19	104	41	26	62	34	0.001283	0.07728	74	80	109	62	77	86	0.49310804	1	64.375	33.7678524				
10	6E+07	10_62532942	97	208	157	289	153	223	0.116599	1	261	256	364	185	259	275	0.29232056	1	227.13	83.2679625				
10	6E+07	10_62583664	1	12	3	22	7	13	0.61331	1	1	18	3	31	10	17	0.30316389	1	11.375	11.32554	6.3E+07	6.3E+07	0.41321	ID=promoter_07017;
10	6E+07	10_62613317	44	36	26	27	40	27	0.080128	1	9	27	11	2	18	7	0.13207736	1	22.75	14.3003496	6.3E+07	6.3E+07	0.18931	ID=promoter_07021;
10	6E+07	10_62669996	87	160	75	49	124	62	1.17E-07	#######	110	205	218	174	158	196	0.02381955	0.7818446	134.75	63.0866751				
10	6E+07	10_62683693	0	8	3	2	4	3	0.840045	1	73	36	24	2	55	13	1.07E-05	0.0013461	18.5	25.4782821	6.3E+07	6.3E+07	0.11902	ID=promoter_07039;
10	6E+07	10_62693699	29	15	30	51	22	41	0.209664	1	1	0	7	10	1	9	0.02974549	0.9232309	17.875	17.5697589				
10	6E+07	10_62705301	13	0	16	5	7	11	0.831726	1	0	16	7	0	8	4	0.59180275	1	7.125	7.05969445	6.3E+07	6.3E+07	0.23411	ID=promoter_07045;
10	6E+07	10_62726934	1	20	3	14	11	9	0.722819	1	1	9	14	0	5	7	0.77775394	1	7.75	7.59228932	6.3E+07	6.3E+07	0.09848	ID=promoter_07050;
10	6E+07	10_62744058	13	14	15	10	14	13	0.831264	1	68	38	136	7	53	72	0.10475411	1	37.625	44.6860077	6.3E+07	6.3E+07	0.26372	ID=promoter_07054;
10	6E+07	10_62781906	14	24	16	23	19	20	0.944476	1	4	7	13	11	6	12	0.2888538	1	14	7.01019666	6.3E+07	6.3E+07	0.1759	ID=promoter_07061;
10	6E+07	10_62807862	2	13	10	4	8	7	0.850897	1	11	0	2	1	6	2	0.42506085	1	5.375	5.12521777	6.3E+07	6.3E+07	0.28109	ID=promoter_07066;
10	6E+07	10_62808032	10	21	18	14	16	16	0.903021	1	43	4	6	16	24	11	0.1422748	1	16.5	12.188988	6.3E+07	6.3E+07	0.28109	ID=promoter_07066;
10	6E+07	10_62810611	60	84	115	90	72	103	0.432601	1	16	25	22	1	21	12	0.40192224	1	51.625	41.4485481				
10	6E+07	10_62835202	31	27	27	14	29	21	0.235916	1	49	36	16	36	43	26	0.2226398	1	29.5	11.351526				

10	6E+07	10_62873552	6	23	6	18	15	12	0.610117	1	46	47	22	2	47	12	1.10E-04	0.0099406	21.25	17.425351				
10	6E+07	10_62912258	3	12	3	15	8	9	0.996316	1	62	13	16	1	38	9	3.73E-04	0.0277241	15.625	19.6536983				
10	6E+07	10_62936801	12	7	9	2	10	6	0.41703	1	0	32	1	18	16	10	0.61601721	1	10.125	10.7362869				
10	6E+07	10_62943519	48	63	5	6	56	6	2.31E-11	#######	2	34	23	2	18	13	0.78939737	1	22.875	23.4242701				
10	6E+07	10_62992034	0	14	2	0	7	1	0.062633	1	8	0	15	2	4	9	0.31291743	1	5.125	6.35694221				
10	6E+07	10_63131632	10	7	1	8	9	5	0.390586	1	1	11	13	2	6	8	0.79537884	1	6.625	4.74906006	6.3E+07	6.3E+07	0.44509	ID=promoter_07111;
10	6E+07	10_63163320	14	23	34	25	19	30	0.595918	1	9	26	38	13	18	26	0.34534801	1	22.75	10.2504355	6.3E+07	6.3E+07	0.24686	ID=promoter_07121;
10	6E+07	10_63165939	15	21	33	12	18	23	0.963662	1	8	33	19	7	21	13	0.51498325	1	18.5	10.1699838				
10	6E+07	10_63184163	8	2	8	7	5	8	0.86114	1	57	10	6	15	34	11	0.0064318	0.2773637	14.125	17.707444	6.3E+07	6.3E+07	0.28709	ID=promoter_07126;
10	6E+07	10_63187888	7	5	3	6	6	5	0.851367	1	25	19	19	1	22	10	0.16182597	1	10.625	8.97516414	6.3E+07	6.3E+07	0.4993	ID=promoter_07128;
10	6E+07	10_63189181	11	18	15	5	15	10	0.374041	1	55	25	42	1	40	22	0.13824779	1	21.5	18.5626353	6.3E+07	6.3E+07	0.4993	ID=promoter_07128;
10	6E+07	10_63190606	11	40	10	15	26	13	0.035221	1	72	87	76	29	80	53	0.16262252	1	42.5	31.5458849	6.3E+07	6.3E+07	0.4993	ID=promoter_07128;
10	6E+07	10_63195594	18	27	36	24	23	30	0.929178	1	8	32	4	0	20	2	0.00122165	0.0738454	18.625	13.3837588	6.3E+07	6.3E+07	0.3011	ID=promoter_07130;
10	6E+07	10_63261198	53	43	74	22	48	48	0.736366	1	17	6	0	17	12	9	0.89054267	1	29	25.3658939	6.3E+07	6.3E+07	0.27329	ID=promoter_07158;
10	6E+07	10_63269227	2	12	10	10	7	10	0.922856	1	12	13	17	1	13	9	0.79589064	1	9.625	5.47559521	6.3E+07	6.3E+07	0.23086	ID=promoter_07161;
10	6E+07	10_63272590	372	194	538	344	283	441	9.10E-04	0.05851	61	38	32	5	50	19	0.00288576	0.1472614	198	198.703656	6.3E+07	6.3E+07	0.24353	ID=promoter_07163;
10	6E+07	10_63284902	4	12	7	1	8	4	0.357158	1	0	0	19	0	0	10	0.00454609	0.2107253	5.375	6.96803928	6.3E+07	6.3E+07	0.12533	ID=promoter_07166;
10	6E+07	10_63290672	59	25	34	28	42	31	0.142577	1	6	5	1	38	6	20	0.0132456	0.49354	24.5	19.8206242	6.3E+07	6.3E+07	0.98137	ID=promoter_07167;
10	6E+07	10_63294837	6	27	0	26	17	13	0.50439	1	1	0	27	0	1	14	0.00214405	0.1161796	10.875	13.2280816	6.3E+07	6.3E+07	0.98137	ID=promoter_07167;
10	6E+07	10_63298007	338	119	246	247	229	247	0.658316	1	185	97	109	60	141	85	0.00682574	0.2917962	175.13	95.3900826	6.3E+07	6.3E+07	0.28522	ID=promoter_07168;
10	6E+07	10_63300438	2	6	7	4	4	6	0.929647	1	23	2	16	21	13	19	0.44319111	1	10.125	8.57633788	6.3E+07	6.3E+07	0.65456	ID=promoter_07169;
10	6E+07	10_63328745	152	110	122	87	131	105	0.013894	0.52987	40	12	21	31	26	26	0.96336814	1	71.875	52.7458529				
10	6E+07	10_63420279	6	12	4	7	9	6	0.554309	1	10	28	58	23	19	41	0.00732633	0.3083947	18.5	18.0633805				
10	6E+07	10_63432433	11	25	38	28	18	33	0.306971	1	13	17	8	8	15	8	0.44137572	1	18.5	10.8364464	6.3E+07	6.3E+07	0.12745	ID=promoter_07190;
10	6E+07	10_63506310	16	14	2	3	15	3	0.006447	0.2884	9	3	2	44	6	23	0.00349909	0.1735359	11.625	14.2120422				
10	6E+07	10_63532116	361	220	386	570	291	478	3.19E-05	0.00329	207	171	168	42	189	105	1.18E-04	0.0105237	265.63	164.855727				
10	6E+07	10_63536805	2	55	29	12	29	21	0.235916	1	32	18	24	49	25	37	0.18968026	1	27.625	17.8640701	6.4E+07	6.4E+07	0.16686	ID=promoter_07212;
10	6E+07	10_63557818	14	30	8	10	22	9	0.020582	0.72012	0	11	4	21	6	13	0.20797135	1	12.25	9.54313515	6.4E+07	6.4E+07	0.13848	ID=promoter_07217;
10	6E+07	10_63563067	8	18	1	5	13	3	0.018372	0.65917	0	0	21	0	0	11	0.00265095	0.136745	6.625	8.48422907				

10	6E+07	10_63567138	14	42	3	5	28	4	1.54E-05	0.00173	0	7	9	1	4	5	0.90483023	1	10.125	13.6532309								
10	6E+07	10_63599882	14	14	12	5	14	9	0.360318	1	49	27	15	2	38	9	3.73E-04	0.0277241	17.25	14.8299889								
10	6E+07	10_63603961	5	18	2	3	12	3	0.030625	0.98374	0	0	14	7	0	11	0.00265095	0.136745	6.125	6.6211242								
10	6E+07	10_63649613	7	15	12	17	11	15	0.930409	1	15	26	23	25	21	24	0.78178162	1	17.5	6.67618368								
10	6E+07	10_63674779	10	2	12	20	6	16	0.210908	1	49	6	35	1	28	18	0.47597237	1	16.875	17.0414411					6.4E+07	6.4E+07	0.22206	ID=promoter_07236;
10	6E+07	10_63675493	22	11	2	23	17	13	0.50439	1	15	40	23	21	28	22	0.84457634	1	19.625	11.0316104					6.4E+07	6.4E+07	0.22206	ID=promoter_07236;
10	6E+07	10_63747918	22	46	17	23	34	20	0.04416	1	86	73	10	16	80	13	3.78E-10	1.18E-07	36.625	28.7050891								
10	6E+07	10_63751303	21	104	71	55	63	63	0.669207	1	32	22	67	62	27	65	8.56E-05	0.0080995	54.25	28.3334734								
10	6E+07	10_63751401	91	31	62	81	61	72	0.999352	1	29	9	29	24	19	27	0.35594749	1	44.5	29.6262432								
10	6E+07	10_63778391	3	17	3	0	10	2	0.03464	1	28	11	9	11	20	10	0.26942782	1	10.25	9.0514403					6.4E+07	6.4E+07	0.21069	ID=promoter_07259;
10	6E+07	10_63778715	11	20	11	16	16	14	0.724026	1	28	0	2	10	14	6	0.26891969	1	12.25	9.1456469					6.4E+07	6.4E+07	0.21069	ID=promoter_07259;
10	6E+07	10_63784421	2506	1681	2644	2221	2094	2433	0.950723	1	1541	1263	1544	392	1402	968	1.33E-11	4.88E-09	1724	732.843386								
10	6E+07	10_63792021	6	6	10	3	6	7	0.999953	1	0	11	2	7	6	5	0.98461866	1	5.625	3.81491434								
10	6E+07	10_63796991	0	5	0	0	3	0	0.172143	1	28	11	0	1	20	1	3.70E-04	0.0275298	5.625	9.84069539					6.4E+07	6.4E+07	0.13804	ID=promoter_07264;
10	6E+07	10_63845244	11	0	10	1	6	6	0.962469	1	0	0	21	5	0	13	9.01E-04	0.0577931	6	7.55928946					6.4E+07	6.4E+07	0.15595	ID=promoter_07274;
10	6E+07	10_63851634	42	0	9	0	21	5	0.001802	0.10311	0	0	0	0	0	0	1	1	6.375	14.735162					6.4E+07	6.4E+07	0.17415	ID=promoter_07276;
10	6E+07	10_63875393	73	93	25	34	83	30	3.69E-08	#######	19	46	30	29	33	30	0.99699964	1	43.625	26.0545444					6.4E+07	6.4E+07	0.23463	ID=promoter_07281;
10	6E+07	10_63885438	3	3	1	1	3	1	0.508575	1	0	8	2	32	4	17	0.01069978	0.4139111	6.25	10.6871084								
10	6E+07	10_64015627	59	18	21	70	39	46	0.999674	1	43	14	23	6	29	15	0.17706132	1	31.75	22.9642579					6.4E+07	6.4E+07	0.48434	ID=promoter_07299;
10	6E+07	10_64020561	490	117	550	412	304	481	2.23E-04	0.01779	117	61	97	104	89	101	0.37877484	1	243.5	203.304838					6.4E+07	6.4E+07	0.22922	ID=promoter_07301;
10	6E+07	10_64037736	135	62	64	75	99	70	0.004674	0.22217	53	20	22	6	37	14	0.01279999	0.4795643	54.625	40.7919372					6.4E+07	6.4E+07	0.28836	ID=promoter_07306;
10	6E+07	10_64043797	0	19	8	11	10	10	0.938234	1	0	11	2	1	6	2	0.42506085	1	6.5	6.9075528					6.4E+07	6.4E+07	0.19168	ID=promoter_07310;
10	6E+07	10_64051000	3	18	7	2	11	5	0.189421	1	27	12	18	0	20	9	0.18397305	1	10.875	9.538456								
10	6E+07	10_64065278	137	68	120	122	103	121	0.999934	1	44	19	33	12	32	23	0.64682985	1	69.375	50.2904066								
10	6E+07	10_64097379	38	16	17	27	27	22	0.443332	1	0	0	1	0	0	1	0.58312783	1	12.375	14.6085836					6.4E+07	6.4E+07	0.1691	ID=promoter_07326;
10	6E+07	10_64169616	41	11	33	55	26	44	0.32944	1	0	32	12	1	16	7	0.23724011	1	23.125	20.0245385					6.4E+07	6.4E+07	0.95057	ID=promoter_07345;
10	6E+07	10_64170447	4	7	13	2	6	8	0.9722	1	0	7	6	3	4	5	0.90483023	1	5.25	3.99106144								
10	6E+07	10_64175184	1	10	10	0	6	5	0.851367	1	15	0	7	2	8	5	0.78486764	1	5.625	5.68048036								
10	6E+07	10_64182278	239	128	221	183	184	202	0.809752	1	138	70	138	103	104	121	0.23476918	1	152.5	57.7952049	6E+07	6.4E+07	ID=ENSP00000374513-D2;ADARB1;NM_015834;	ADARB1	6.4E+07	6.4E+07	0.71771	ID=promoter_07349;

10	6E+07	10_64184497	340	184	477	497	262	487	1.05E-08	#######	41	90	91	10	66	51	0.61990708	1	216.25	195.683235	6E+07	6.4E+07	ID=ENSP0000374513-D2;ADARB1;NM_015834;	ADARB1	6.4E+07	6.4E+07	0.39	ID=promoter_07350;
10	6E+07	10_64223240	75	43	42	14	59	28	2.44E-04	0.01921	0	0	0	0	0	0	1	1	21.75	28.4090026	6E+07	6.4E+07	ID=ENSP0000374513-D2;ADARB1;NM_015834;	ADARB1	6.4E+07	6.4E+07	0.32386	ID=promoter_07362;
10	6E+07	10_64231136	1	35	0	2	18	1	1.14E-04	0.01003	11	0	0	1	6	1	0.20114407	1	6.25	12.1860576	6E+07	6.4E+07	ID=ENSP0000374513-D2;ADARB1;NM_015834;	ADARB1	6.4E+07	6.4E+07	0.41019	ID=promoter_07366;
10	6E+07	10_64250607	9	10	14	5	10	10	0.938234	1	1	24	38	31	13	35	0.00269206	0.1382662	16.5	13.1257653	6E+07	6.4E+07	ID=ENSP0000374513-D2;ADARB1;NM_015834;	ADARB1	6.4E+07	6.4E+07	0.59183	ID=promoter_07374;
10	6E+07	10_64251269	8	6	7	18	7	13	0.61331	1	15	1	12	17	8	15	0.25959112	1	10.5	5.97614305	6E+07	6.4E+07	ID=ENSP0000374513-D2;ADARB1;NM_015834;	ADARB1	6.4E+07	6.4E+07	0.59183	ID=promoter_07374;
10	6E+07	10_64270892	117	82	142	138	100	140	0.400277	1	143	205	417	130	174	274	2.29E-07	4.45E-05	171.75	104.812417					6.4E+07	6.4E+07	0.85127	ID=promoter_07380;
10	6E+07	10_64275593	15	5	2	1	10	2	0.03464	1	16	11	9	0	14	5	0.16413709	1	7.375	6.30051018					6.4E+07	6.4E+07	0.35175	ID=promoter_07381;
10	6E+07	10_64276325	64	30	165	162	47	164	3.98E-11	#######	1	13	21	2	7	12	0.42229866	1	57.25	68.5143364					6.4E+07	6.4E+07	0.35175	ID=promoter_07381;
10	6E+07	10_64300262	2	31	15	18	17	17	0.897282	1	0	5	6	16	3	11	0.0740992	1	11.625	10.3501208					6.4E+07	6.4E+07	0.32596	ID=promoter_07389;
10	6E+07	10_64308599	28	40	21	25	34	23	0.11872	1	21	39	25	77	30	51	0.02786329	0.8848986	34.5	18.6853648					6.4E+07	6.4E+07	0.54854	ID=promoter_07393;
10	6E+07	10_64335460	249	79	236	161	164	199	0.949609	1	60	74	57	41	67	49	0.44961427	1	119.63	83.9930907					6.4E+07	6.4E+07	0.3611	ID=promoter_07397;
10	6E+07	10_64336822	23	22	30	35	23	33	0.758773	1	6	28	30	7	17	19	0.85444859	1	22.625	10.7695006					6.4E+07	6.4E+07	0.6554	ID=promoter_07398;
10	6E+07	10_64354736	22	31	9	4	27	7	4.86E-04	0.03443	21	8	17	23	15	20	0.56420259	1	16.875	9.15637951								
10	6E+07	10_64360863	9	40	9	55	25	32	0.94785	1	88	31	22	35	60	29	0.01363409	0.5034172	36.125	26.0792063					6.4E+07	6.4E+07	0.68621	ID=promoter_07404;
10	6E+07	10_64430934	3	25	2	0	14	1	0.001161	0.07164	1	8	19	2	5	11	0.25523295	1	7.5	9.3960478	6E+07	6.4E+07	ID=ENSP0000263154;LARP5;;	LARP5	6.4E+07	6.4E+07	0.23565	ID=promoter_07413;
10	6E+07	10_64443521	12	11	1	15	12	8	0.45534	1	0	1	0	1	1	1	0.99856566	1	5.125	6.35694221	6E+07	6.4E+07	ID=ENSP0000263154;LARP5;;	LARP5	6.4E+07	6.4E+07	0.49345	ID=promoter_07419;
10	6E+07	10_64467953	48	35	47	40	42	44	0.871664	1	57	33	30	16	45	23	0.06212307	1	38.25	12.6913919								
10	6E+07	10_64477661	6	15	18	13	11	16	0.859437	1	15	8	12	3	12	8	0.76853133	1	11.25	5.11998884					6.4E+07	6.4E+07	0.31599	ID=promoter_07427;
10	6E+07	10_64531090	9	1	4	8	5	6	0.999292	1	0	4	4	12	2	8	0.1303879	1	5.25	4.09703726					6.5E+07	6.5E+07	0.19049	ID=promoter_07434;
10	6E+07	10_64595755	3	37	15	16	20	16	0.517469	1	36	4	7	1	20	4	0.00863789	0.3495868	14.875	14.3968002					6.5E+07	6.5E+07	0.2429	ID=promoter_07442;

10	6E+07	10_64599620	470	619	438	311	545	375	8.86E-15	#######	801	870	928	584	836	756	0.88478296	1	627.63	221.056513					6.5E+07	6.5E+07	0.86958	ID=promoter_07445;
10	6E+07	10_64618258	23	42	33	8	33	21	0.084147	1	57	23	45	31	40	38	0.9941866	1	32.75	15.2573167								
10	6E+07	10_64679385	8	25	14	12	17	13	0.50439	1	3	37	18	1	20	10	0.26942782	1	14.75	11.9013805					6.5E+07	6.5E+07	0.15011	ID=promoter_07458;
10	6E+07	10_64858655	25	13	17	1	19	9	0.06806	1	0	0	11	6	0	9	0.00779604	0.3222758	9.125	9.06228448	6E+07	6.5E+07	ID=ENSP0000381053;ZMYND11;;	ZMYND11	6.5E+07	6.5E+07	0.14756	ID=promoter_07466;
10	7E+07	10_65055782	22	15	23	18	19	21	0.982527	1	21	50	22	1	36	12	0.00589803	0.2600594	21.5	13.5751874								
10	7E+07	10_65327104	36	27	12	12	32	12	0.001806	0.10314	8	19	40	49	14	45	8.50E-05	0.0080481	25.375	15.0611847					6.5E+07	6.5E+07	0.38492	ID=promoter_07479;
10	7E+07	10_65521311	0	15	0	8	8	4	0.357158	1	0	10	4	19	5	12	0.17891578	1	7	7.30948503								
11	71136	11_71136	51	100	51	20	76	36	2.15E-05	0.00234	0	7	29	7	4	18	0.00668749	0.2868305	33.125	33.3013835	38976	101247	ID=ENSP0000372262;CRYL1;;	CRYL1				
11	82451	11_82451	28	1	5	19	15	12	0.610117	1	17	25	8	11	21	10	0.21021716	1	14.25	9.60282696	38976	101247	ID=ENSP0000372262;CRYL1;;	CRYL1	81777	82810	0.21625	ID=promoter_07514;
11	89093	11_89093	201	60	110	134	131	122	0.184792	1	0	18	2	2	9	2	0.1390433	1	65.875	75.283156	38976	101247	ID=ENSP0000372262;CRYL1;;	CRYL1	88861	89518	0.1604	ID=promoter_07518;
11	91105	11_91105	26	1	8	6	14	7	0.165008	1	1	1	10	1	1	6	0.13767373	1	6.75	8.58154165	38976	101247	ID=ENSP0000372262;CRYL1;;	CRYL1	90201	92052	0.51708	ID=promoter_07519;
11	98486	11_98486	4	0	38	4	2	21	0.001582	0.09271	0	27	13	3	14	8	0.54462139	1	11.125	14.106103	38976	101247	ID=ENSP0000372262;CRYL1;;	CRYL1	97662	102890	0.75763	ID=promoter_07522;
11	142583	11_142583	2	1	0	10	2	5	0.651182	1	1	7	11	23	4	17	0.01069978	0.4139111	6.875	7.80910641					141073	142653	0.30934	ID=promoter_07530;
11	151693	11_151693	1	4	2	12	3	7	0.596562	1	10	26	20	7	18	14	0.88543974	1	10.25	8.86002257					151331	152295	0.17993	ID=promoter_07533;
11	153708	11_15370	15	6	3	2	11	3	0.050503	1	3	0	16	2	2	9	0.08177705	1	5.875	6.17454452								
11	170322	11_17032	28	5	4	8	17	6	0.027279	0.89953	1	51	4	29	26	17	0.53107524	1	16.25	17.8705664								
11	171616	11_171616	83	50	32	27	67	30	3.12E-05	0.00323	42	32	35	34	37	35	0.99634264	1	41.875	18.0589313					170901	171825	0.19929	ID=promoter_07539;
11	178675	11_17867	61	36	64	35	49	50	0.786177	1	0	7	0	0	4	0	0.15659576	1	25.375	27.3492622								
11	233748	11_23374	260	608	196	189	434	193	3.20E-30	#######	319	412	540	207	366	374	0.41573776	1	341.38	162.594972								
11	330601	11_330601	1	48	18	2	25	10	0.01059	0.42992	0	14	30	3	7	17	0.08351885	1	14.5	17.1380946					330393	332125	0.49307	ID=promoter_07574;
11	331171	11_331171	4	15	7	11	10	9	0.84607	1	0	0	0	6	0	3	0.19828566	1	5.375	5.55331304					330393	332125	0.49307	ID=promoter_07574;
11	344719	11_344719	68	25	42	60	47	51	0.928531	1	11	54	13	24	33	19	0.24811784	1	37.125	21.937818					344495	346256	0.34262	ID=promoter_07579;
11	345112	11_345112	44	15	20	36	30	28	0.684	1	0	0	0	0	0	0	1	1	14.375	17.7437112					344495	346256	0.34262	ID=promoter_07579;
11	349345	11_349345	58	34	40	59	46	50	0.932872	1	0	0	3	0	0	2	0.34003807	1	24.25	26.4723576					348960	349665	0.10413	ID=promoter_07581;
11	471183	11_471183	5	26	4	0	16	2	0.001284	0.07728	0	11	6	1	6	4	0.87665919	1	6.625	8.65097022					470685	472778	0.28803	ID=promoter_07613;
11	476338	11_476338	111	132	179	170	122	175	0.232119	1	6	5	16	2	6	9	0.65381141	1	77.625	78.1956109					475903	477153	0.1823	ID=promoter_07615;
11	548241	11_548241	22	36	13	7	29	10	0.001829	0.10386	18	32	43	3	25	23	0.99965163	1	21.75	14.2402849					546979	548662	0.25761	ID=promoter_07632;

11	557664	11_557664	692	409	695	715	551	705	0.310514	1	593	355	415	142	474	279	9.88E-09	2.45E-06	502	205.087438	556396	559658	0.50711	ID=promoter_07635;
11	561220	11_561220	23	29	5	11	26	8	0.001755	0.10104	2	20	27	5	11	16	0.51039395	1	15.25	10.780273	561030	562164	0.21463	ID=promoter_07636;
11	563854	11_563854	8	14	4	3	11	4	0.10516	1	9	5	24	10	7	17	0.08351885	1	9.625	6.82301777	563650	564184	0.14449	ID=promoter_07638;
11	576803	11_576803	10	37	11	19	24	15	0.151024	1	61	14	37	12	38	25	0.40838944	1	25.125	18.2477983	576303	578627	0.37261	ID=promoter_07641;
11	581803	11_581803	60	65	115	88	63	102	0.129963	1	16	13	20	20	15	20	0.56420259	1	49.625	38.3924751	581470	582484	0.17079	ID=promoter_07642;
11	601007	11_60100	14	38	54	20	26	37	0.751146	1	12	16	43	0	14	22	0.29543416	1	24.625	18.3376077				
11	612182	11_61218	75	57	120	98	66	109	0.088033	1	155	57	130	37	106	84	0.56114486	1	91.125	41.3881884				
11	615890	11_61589	106	30	76	98	68	87	0.865782	1	35	19	18	25	27	22	0.90387209	1	50.875	36.5374074				
11	625090	11_62509	0	45	24	25	23	25	0.965853	1	50	30	22	21	40	22	0.13824779	1	27.125	15.422039				
11	633908	11_633908	113	121	92	63	117	78	4.66E-04	0.03334	57	130	126	42	94	84	0.97052768	1	93	34.7233968	633406	634569	0.20976	ID=promoter_07657;
11	636632	11_636632	15	14	22	20	15	21	0.871674	1	25	10	14	2	18	8	0.20901227	1	15.25	7.26537778	635122	636859	0.31393	ID=promoter_07658;
11	637700	11_637700	4	20	0	0	12	0	8.78E-04	0.05704	18	0	17	0	9	9	0.98716474	1	7.375	9.21082438	637237	638195	0.14085	ID=promoter_07659;
11	637879	11_637879	12	1	24	4	7	14	0.506445	1	0	0	10	20	0	15	3.07E-04	0.0235795	8.875	9.3110917	637237	638195	0.14085	ID=promoter_07659;
11	679115	11_679115	0	13	8	15	7	12	0.724589	1	19	7	6	6	13	6	0.35360803	1	9.25	6.04152299	676727	679844	0.63677	ID=promoter_07670;
11	694686	11_69468	0	0	2	5	0	4	0.181755	1	47	1	14	1	24	8	0.03264408	0.9940318	8.75	16.1400124				
11	696504	11_696504	15	66	24	11	41	18	0.001442	0.08558	1	57	25	23	29	24	0.91882689	1	27.75	22.4292539	695482	697193	0.34495	ID=promoter_07677;
11	712708	11_71270	16	25	12	7	21	10	0.052655	1	32	7	16	1	20	9	0.18397305	1	14.5	10.1559272				
11	717807	11_71780	18	44	33	55	31	44	0.717763	1	84	63	60	7	74	34	0.00253133	0.13283	45.5	25.3151564				
11	736543	11_736543	0	0	7	0	0	4	0.181755	1	22	0	0	36	11	18	0.32230807	1	8.125	13.6322884	736435	737909	0.25477	ID=promoter_07689;
11	737396	11_737396	11	72	22	40	42	31	0.142577	1	45	35	29	60	40	45	0.67176911	1	39.25	19.8404349	736435	737909	0.25477	ID=promoter_07689;
11	757113	11_757113	3	19	0	11	11	6	0.3028	1	0	44	12	3	22	8	0.06315916	1	11.5	14.7454593	755358	758223	0.53415	ID=promoter_07694;
11	829351	11_829351	0	35	20	30	18	25	0.86091	1	33	0	17	27	17	22	0.58360552	1	20.25	13.8950145	828819	831148	0.30369	ID=promoter_07717;
11	845363	11_845363	16	20	2	20	18	11	0.22238	1	0	20	10	4	10	7	0.84912739	1	11.5	8.60232527	845051	848170	0.48477	ID=promoter_07723;
11	846312	11_846312	321	185	213	236	253	225	0.010133	0.41316	81	67	135	37	74	86	0.36043726	1	159.38	96.9328744	845051	848170	0.48477	ID=promoter_07723;
11	858308	11_858308	27	5	15	6	16	11	0.385778	1	19	16	10	0	18	5	0.0406475	1	12.25	8.71370021	855862	858593	0.4551	ID=promoter_07727;
11	896281	11_89628	32	50	30	20	41	25	0.031944	1	51	16	50	34	34	42	0.45863117	1	35.375	13.7626565				
11	906076	11_90607	5	6	10	0	6	5	0.851367	1	31	0	4	4	16	4	0.04223275	1	7.5	10.0285307				
11	941110	11_941110	16	31	29	45	24	37	0.578559	1	20	10	13	0	15	7	0.31064188	1	20.5	14.0915375	939897	941398	0.32169	ID=promoter_07746;
11	945338	11_94533	41	25	46	63	33	55	0.276422	1	20	20	26	0	20	13	0.60545249	1	30.125	19.2979459				
11	955264	11_955264	178	92	154	197	135	176	0.652432	1	34	0	50	1	17	26	0.26839536	1	88.25	79.3054673	954489	955452	0.19173	ID=promoter_07751;
11	975601	11_97560	0	0	11	19	0	15	0.001671	0.09737	2	0	0	34	1	17	4.35E-04	0.0314643	8.25	12.5214102				
11	993462	11_99346	10	0	0	7	5	4	0.848147	1	0	4	20	0	2	10	0.05067035	1	5.125	7.12014245				
11	1E+06	11_1000849	0	16	9	2	8	6	0.705675	1	0	0	6	11	0	9	0.00779604	0.3222758	5.5	6.04743157	1000410	1003694	0.58096	ID=promoter_07756;

11	1E+06	11_1003528	9	38	20	25	24	23	0.786129	1	37	53	29	31	45	30	0.37258975	1	30.25	13.144798					1000410	1003694	0.58096	ID=promoter_07756;
11	1E+06	11_1056936	0	1	13	18	1	16	0.004036	0.19749	0	5	5	10	3	8	0.26303803	1	6.5	6.61167798					1055543	1057967	0.32404	ID=promoter_07767;
11	1E+06	11_1079225	92	33	36	82	63	59	0.460562	1	35	11	28	6	23	17	0.77734386	1	40.375	30.9328189					1078787	1080042	0.20198	ID=promoter_07774;
11	1E+06	11_1130533	9	8	28	7	9	18	0.416978	1	9	3	9	12	6	11	0.39136385	1	10.625	7.46300399					1129497	1131922	0.31557	ID=promoter_07788;
11	1E+06	11_1164931	16	23	8	8	20	8	0.026297	0.87605	24	1	32	20	13	26	0.0678374	1	16.5	10.2539191					1163876	1165421	0.21245	ID=promoter_07793;
11	1E+06	11_1168232	21	38	95	169	30	132	1.95E-11	#######	31	31	45	26	31	36	0.65410506	1	57	50.8527285					1168028	1168993	0.18638	ID=promoter_07794;
11	1E+06	11_1172419	14	32	23	27	23	25	0.965853	1	0	13	21	2	7	12	0.42229866	1	16.5	11.4267856					1171681	1174140	0.44395	ID=promoter_07796;
11	1E+06	11_1237658	5	0	10	21	3	16	0.03035	0.98285	1	0	11	2	1	7	0.08320466	1	6.25	7.36303509					1237350	1239418	0.58901	ID=promoter_07809;
11	1E+06	11_1242591	123	64	117	101	94	109	0.996684	1	95	10	33	33	53	33	0.1954656	1	72	43.0049831					1240804	1243218	0.40286	ID=promoter_07810;
11	1E+06	11_12841	12	11	13	12	12	13	0.980472	1	27	0	7	1	14	4	0.08931834	1	10.375	8.41660773								
11	1E+06	11_1362503	28	18	18	26	23	22	0.790781	1	1	0	0	0	1	0	0.62906424	1	11.375	12.3859079	1E+06	1368440	ID=ENSP0000218867;SGCG;NM_000231;	SGCG				
11	1E+06	11_1363799	13	19	35	9	16	22	0.889379	1	1	4	0	0	3	0	0.24893445	1	10.125	12.1471631	1E+06	1368440	ID=ENSP0000218867;SGCG;NM_000231;	SGCG	1363167	1365689	0.40526	ID=promoter_07835;
11	1E+06	11_1368840	7	23	14	21	15	18	0.997876	1	9	12	20	29	11	25	0.03752473	1	16.875	7.58640702					1367075	1369827	0.65665	ID=promoter_07836;
11	1E+06	11_14003	497	189	258	228	343	243	9.57E-09	#######	49	49	32	38	49	35	0.49765999	1	167.5	162.171867								
11	1E+06	11_1477598	15	0	10	27	8	19	0.232241	1	0	0	54	27	0	41	2.49E-10	7.88E-08	16.625	18.8523398	1E+06	1507566	ID=ENSP0000385408;TNFRSF19;;	TNFRSF19	1477324	1479814	0.38295	ID=promoter_07859;
11	2E+06	11_1561769	38	16	18	28	27	23	0.526863	1	30	1	26	0	16	13	0.9394435	1	19.625	13.6479983					1561690	1562189	0.12117	ID=promoter_07878;
11	2E+06	11_1605053	10	9	27	31	10	29	0.038384	1	40	0	6	1	20	4	0.00863789	0.3495868	15.5	15.0522898					1603737	1606234	0.35759	ID=promoter_07886;
11	2E+06	11_16572	5	9	3	6	7	5	0.693374	1	7	9	6	3	8	5	0.78486764	1	6	2.32992949								
11	2E+06	11_1741402	18	4	29	34	11	32	0.026902	0.89309	0	8	16	0	4	8	0.43697044	1	13.625	12.9387292					1740111	1742713	0.41727	ID=promoter_07921;
11	2E+06	11_1785501	146	33	117	147	90	132	0.261392	1	0	8	11	27	4	19	0.00415462	0.1961166	61.125	64.0612151	2E+06	1799207	ID=ENSP0000385408-D2;TNFRSF19;;	TNFRSF19	1785497	1786286	0.22837	ID=promoter_07933;
11	2E+06	11_1792669	4	34	16	6	19	11	0.163668	1	33	2	5	2	18	4	0.01933304	0.6616555	12.75	13.5514891	2E+06	1799207	ID=ENSP0000385408-D2;TNFRSF19;;	TNFRSF19	1790448	1792938	0.38295	ID=promoter_07935;
11	2E+06	11_1824814	4	9	5	1	7	3	0.314307	1	14	2	6	1	8	4	0.59180275	1	5.25	4.46414285					1824232	1825024	0.11165	ID=promoter_07944;
11	2E+06	11_1861392	5	7	15	6	6	11	0.674578	1	27	61	17	4	44	11	1.66E-04	0.0140498	17.75	19.1591082					1861059	1863394	0.43109	ID=promoter_07951;
11	2E+06	11_18859	14	64	15	21	39	18	0.003161	0.16258	28	18	36	13	23	25	0.85898626	1	26.125	17.1999792								
11	2E+06	11_1902828	7	4	0	2	6	1	0.108618	1	11	1	3	23	6	13	0.20797135	1	6.375	7.59581652					1901108	1903655	0.60009	ID=promoter_07959;

11	2E+06	11_1913570	18	10	17	12	14	15	0.970741	1	14	4	13	13	9	13	0.58766318	1	12.625	4.3404246					1906182	1913932	0.94977	ID=promoter_07961;
11	2E+06	11_1919635	11	8	8	3	10	6	0.41703	1	6	0	9	1	3	5	0.7175395	1	5.75	3.99106144					1919278	1921172	0.53847	ID=promoter_07963;
11	2E+06	11_1951263	54	21	30	22	38	26	0.100519	1	18	35	38	15	27	27	0.96198634	1	29.125	12.9442486					1948370	1952257	0.5316	ID=promoter_07972;
11	2E+06	11_19581	6	20	12	24	13	18	0.901125	1	15	4	12	14	10	13	0.72167512	1	13.375	6.61032742								
11	2E+06	11_19823	12	56	25	28	34	27	0.313685	1	53	31	16	50	42	33	0.7761727	1	33.875	17.049822								
11	2E+06	11_2006167	110	170	162	264	140	213	0.056463	1	60	71	147	28	66	88	0.08059616	1	126.5	75.5513071					2004987	2006508	0.28817	ID=promoter_07981;
11	2E+06	11_2013835	20	14	7	23	17	15	0.722447	1	4	11	18	5	8	12	0.56746184	1	12.75	7.16638981					2013516	2014263	0.16568	ID=promoter_07982;
11	2E+06	11_20890	2	14	11	6	8	9	0.996316	1	3	6	11	0	5	6	0.91271846	1	6.625	4.95515604								
11	2E+06	11_2111459	36	53	75	81	45	78	0.110382	1	133	38	62	58	86	60	0.23753907	1	67	30.9838668	2E+06	2136172	ID=ENSP00000382830;C1QTNF9;;	C1QTNF9				
11	2E+06	11_2138364	1	12	3	5	7	4	0.503048	1	0	9	10	17	5	14	0.08295177	1	7.125	5.89036744					2137481	2138859	0.21744	ID=promoter_08013;
11	2E+06	11_2144531	38	6	13	19	22	16	0.340123	1	1	29	47	33	15	40	0.00125919	0.075413	23.25	16.1488611					2144066	2146108	0.23682	ID=promoter_08015;
11	2E+06	11_22083	79	50	102	117	65	110	0.062293	1	7	0	0	1	4	1	0.45332778	1	44.5	49.3413764								
11	2E+06	11_2215249	71	53	90	84	62	87	0.558147	1	33	51	36	16	42	26	0.26520775	1	54.25	25.921585					2214903	2216424	0.28817	ID=promoter_08022;
11	2E+06	11_2392096	20	79	45	35	50	40	0.192624	1	111	80	124	20	96	72	0.39558322	1	64.25	40.2909065					2391211	2392771	0.62996	ID=promoter_08059;
11	2E+06	11_2401007	289	111	637	443	200	540	4.62E-24	#######	192	96	187	84	144	136	0.98797889	1	254.88	195.036581					2399965	2402064	0.31694	ID=promoter_08060;
11	2E+06	11_2416722	25	28	7	17	27	12	0.014569	0.54886	62	40	30	7	51	19	0.00210847	0.1150505	27	18.1580364					2416122	2417081	0.26405	ID=promoter_08064;
11	3E+06	11_25084	28	19	8	6	24	7	0.00213	0.11798	10	12	0	49	11	25	0.03752473	1	16.5	15.6387796								
11	3E+06	11_25209	79	74	150	130	77	140	0.007838	0.33626	13	34	15	5	24	10	0.09254106	1	62.5	55.345667								
11	3E+06	11_25748	110	95	116	152	103	134	0.731458	1	80	38	54	31	59	43	0.48515274	1	84.5	41.8910151								
11	3E+06	11_25811	2	51	13	13	27	13	0.024366	0.82541	11	2	24	72	7	48	4.59E-08	1.01E-05	23.5	25.0998008								
11	3E+06	11_2621035	3	40	10	27	22	19	0.617881	1	28	44	11	22	36	17	0.06533483	1	23.125	14.5645117					2620683	2621644	0.22171	ID=promoter_08105;
11	3E+06	11_2646661	13	40	14	14	27	14	0.038924	1	2	4	21	0	3	11	0.0740992	1	13.5	12.8951818					2646187	2646747	0.16389	ID=promoter_08108;
11	3E+06	11_2668765	1	1	4	0	1	2	0.907382	1	26	1	10	0	14	5	0.16413709	1	5.375	8.97516414					2665950	2668776	0.34141	ID=promoter_08112;
11	3E+06	11_26932	42	9	38	21	26	30	0.998123	1	12	10	8	2	11	5	0.40227598	1	17.75	14.7430371								
11	3E+06	11_27291	32	47	16	35	40	26	0.059242	1	57	21	27	18	39	23	0.22193731	1	31.625	14.421586								
11	3E+06	11_27367	2	45	5	6	24	6	9.38E-04	0.05976	13	0	6	9	7	8	0.92121674	1	10.75	14.3999008								
11	3E+06	11_2744759	8	69	145	121	39	133	6.39E-09	#######	65	39	20	22	52	21	0.00421666	0.1980481	61.125	49.6140461					2744325	2745309	0.16549	ID=promoter_08133;
11	3E+06	11_27523	82	286	123	95	184	109	6.16E-08	#######	303	280	344	219	292	282	0.88687619	1	216.5	102.957689								
11	3E+06	11_27929	11	37	21	6	24	14	0.105787	1	79	46	35	7	63	21	1.26E-04	0.0111328	30.25	24.719859								
11	3E+06	11_28263	24	37	7	8	31	8	1.52E-04	0.01277	1	5	15	2	3	9	0.17582851	1	12.375	12.4663834								
11	3E+06	11_2864749	47	23	34	39	35	37	0.907308	1	0	46	32	5	23	19	0.93310412	1	28.25	17.7099649					2864278	2865254	0.12529	ID=promoter_08152;
11	3E+06	11_28912	21	30	23	34	26	29	0.982786	1	20	82	22	1	51	12	2.36E-05	0.002647	29.125	23.4486521								
11	3E+06	11_29307	32	17	37	19	25	28	0.986003	1	36	43	15	53	40	34	0.93312112	1	31.5	13.5435805								
11	3E+06	11_2948793	21	21	6	34	21	20	0.800056	1	7	26	9	27	17	18	0.92557631	1	18.875	10.4120466					2946597	2949352	0.46201	ID=promoter_08167;
11	3E+06	11_30005	26	54	15	2	40	9	3.70E-06	0.00049	3	80	70	63	42	67	0.02029964	0.6899501	39.125	31.2886903								
11	3E+06	11_3061423	7	4	13	32	6	23	0.02363	0.80523	21	12	3	11	17	7	0.1782776	1	12.875	9.61304917					3059607	3061865	0.30047	ID=promoter_08190;
11	3E+06	11_30687	3	0	2	13	2	8	0.255957	1	20	6	17	0	13	9	0.79589064	1	7.625	7.94512429								

11	3E+06	11_3078559	223	116	217	256	170	237	0.227515	1	77	45	146	47	61	97	0.00375213	0.1824105	140.88	83.257668					3076668	3078987	0.33336	ID=promoter_08195;
11	3E+06	11_31016	5	24	5	0	15	3	0.006447	0.2884	38	48	17	60	43	39	0.99511464	1	24.625	22.0773801								
11	3E+06	11_3116845	21	30	84	80	26	82	2.84E-05	0.00296	47	68	72	53	58	63	0.68395617	1	56.875	23.129681					3115826	3117183	0.26276	ID=promoter_08200;
11	3E+06	11_3124954	6	10	5	9	8	7	0.850897	1	0	0	7	4	0	6	0.0393172	1	5.125	3.72011905					3124158	3125149	0.15435	ID=promoter_08201;
11	3E+06	11_31527	2	36	7	1	19	4	0.002035	0.11403	31	41	25	3	36	14	0.01744443	0.6140941	18.25	16.7396023								
11	3E+06	11_3161648	5	5	15	14	5	15	0.168861	1	21	23	9	38	22	24	0.85866092	1	16.25	11.0421271					3160858	3161795	0.1488	ID=promoter_08210;
11	3E+06	11_31642	14	59	56	32	37	44	0.998103	1	29	27	30	31	28	31	0.79257784	1	34.75	15.1539717								
11	3E+06	11_3268523	94	90	68	74	92	71	0.02835	0.93034	59	1	23	25	30	24	0.86494794	1	54.25	34.0744982					3267931	3268684	0.17239	ID=promoter_08230;
11	3E+06	11_3270277	146	106	44	159	126	102	0.019832	0.70195	26	40	37	49	33	43	0.33537098	1	75.875	53.1290545					3269777	3271164	0.15911	ID=promoter_08231;
11	3E+06	11_33001	13	23	28	26	18	27	0.720164	1	13	32	18	12	23	15	0.56706676	1	20.625	7.7078346								
11	3E+06	11_3321195	46	29	4	7	38	6	5.50E-07	#######	17	7	7	8	12	8	0.76853133	1	15.625	14.735162					3320246	3322334	0.42096	ID=promoter_08242;
11	3E+06	11_33380	115	57	39	77	86	58	0.004333	0.20916	1	33	49	45	17	47	2.68E-04	0.0209332	52	33.3980324								
11	3E+06	11_3342208	498	91	283	284	295	284	0.058895	1	30	33	66	68	32	67	5.04E-04	0.0357134	169.13	168.680965					3340227	3342902	0.35329	ID=promoter_08246;
11	3E+06	11_33709	214	108	206	174	161	190	0.998383	1	104	94	112	47	99	80	0.65840374	1	132.38	59.0301073								
11	3E+06	11_3373589	51	97	87	61	74	74	0.623882	1	51	84	63	52	68	58	0.89700969	1	68.25	18.3828336					3373089	3376422	0.49139	ID=promoter_08252;
11	3E+06	11_3411455	306	187	224	128	247	176	2.19E-06	0.00031	43	112	85	82	78	84	0.63443746	1	145.88	87.2360304	3E+06	3425871	ID=ENSP00000376368-D3;WASF1;;	WASF1				
11	3E+06	11_3421809	1606	673	1536	1131	1140	1334	0.998273	1	911	653	869	159	782	514	9.06E-09	2.25E-06	942.25	478.79514	3E+06	3425871	ID=ENSP00000376368-D3;WASF1;;	WASF1	3420575	3422333	0.77476	ID=promoter_08260;
11	3E+06	11_34391	1	0	1	6	1	4	0.505922	1	38	1	5	0	20	3	0.00346999	0.1723921	6.5	12.9283741								
11	3E+06	11_34422	7	27	6	26	17	16	0.818283	1	69	39	31	25	54	28	0.04072278	1	28.75	19.8044007								
11	4E+06	11_3522100	130	100	228	190	115	209	7.27E-04	0.04855	109	149	210	24	129	117	0.9854154	1	142.5	66.8601953					3521149	3522138	0.21905	ID=promoter_08282;
11	4E+06	11_3560179	511	328	560	837	420	699	9.01E-08	#######	903	308	449	595	606	522	0.46908734	1	561.38	216.345582					3559130	3560845	0.29764	ID=promoter_08290;
11	4E+06	11_35669	0	16	3	8	8	6	0.705675	1	0	14	5	2	7	4	0.73798468	1	6	6.164414								
11	4E+06	11_35880	32	29	32	27	31	30	0.753666	1	0	14	26	9	7	18	0.05735679	1	21.125	11.9575141								
11	4E+06	11_3598642	164	38	61	87	101	74	0.008093	0.34587	1165	901	832	313	1033	573	2.64E-22	2.06E-19	445.13	449.405778					3595802	3599582	0.62256	ID=promoter_08298;
11	4E+06	11_3609391	27	32	28	25	30	27	0.605646	1	1	30	15	10	16	13	0.9394435	1	21	11.0840941					3609067	3610164	0.26938	ID=promoter_08303;
11	4E+06	11_36284	20	36	5	11	28	8	6.70E-04	0.04525	0	4	4	4	2	4	0.6610374	1	10.5	12								
11	4E+06	11_36868	16	33	6	19	25	13	0.050329	1	2	19	21	44	11	33	0.00170606	0.0957908	20	13.5435805								
11	4E+06	11_3693709	0	10	2	1	5	2	0.402696	1	2	17	22	2	10	12	0.83305499	1	7	8.40068024					3693418	3694760	0.40922	ID=promoter_08324;
11	4E+06	11_3694492	47	28	54	22	38	38	0.784842	1	56	4	70	29	30	50	0.03617913	1	38.75	21.6250516					3693418	3694760	0.40922	ID=promoter_08324;
11	4E+06	11_3706939	5	48	12	73	27	43	0.457603	1	50	32	50	9	41	30	0.61431761	1	34.875	24.4273705					3706028	3707486	0.24062	ID=promoter_08327;
11	4E+06	11_3747006	121	38	114	118	80	116	0.343572	1	25	43	40	30	34	35	0.90965436	1	66.125	43.0927157					3746038	3747348	0.25193	ID=promoter_08337;
11	4E+06	11_3806073	49	22	23	34	36	29	0.317511	1	10	16	11	10	13	11	0.97545578	1	21.875	13.7262782					3805901	3807346	0.65436	ID=promoter_08344;

11	4E+06	11_3807310	6	20	4	0	13	2	0.006819	0.29841	49	12	0	4	31	2	8.42E-06	0.0010929	11.875	16.4093832					3805901	3807346	0.65436	ID=promoter_08344;
11	4E+06	11_38150	82	133	83	116	108	100	0.232539	1	44	73	58	18	59	38	0.21227106	1	75.875	37.1384956								
11	4E+06	11_3974931	459	187	464	305	323	385	0.977587	1	192	133	128	86	163	107	0.02051983	0.6956031	244.25	148.823721	4E+06	4051813	ID=ENSP0000370532;GTF3A;NM_002097;	GTF3A	3974234	3975527	0.46493	ID=promoter_08368;
11	4E+06	11_4022513	0	2	36	54	1	45	1.86E-08	#######	2	6	9	3	4	6	0.75330063	1	14	19.9212736	4E+06	4051813	ID=ENSP0000370532;GTF3A;NM_002097;	GTF3A	4022220	4022721	0.19363	ID=promoter_08373;
11	4E+06	11_4042738	6	9	7	15	8	11	0.943069	1	35	15	24	6	25	15	0.40601431	1	14.625	10.2947629	4E+06	4051813	ID=ENSP0000370532;GTF3A;NM_002097;	GTF3A	4041985	4043884	0.32391	ID=promoter_08377;
11	4E+06	11_4053574	0	0	0	0	0	0	1	1	33	29	23	30	31	27	0.97229028	1	14.375	15.6107792	4E+06	4055403	ID=ENSP0000384659-D2;MTIF3;NM_152912;	MTIF3				
11	4E+06	11_4153592	12	40	18	2	26	10	0.006911	0.3015	11	12	41	1	12	21	0.20312388	1	17.125	15.4590473	4E+06	4180482	ID=ENSP0000370787-D2;;					
11	4E+06	11_4161094	0	20	6	6	10	6	0.41703	1	15	0	9	0	8	5	0.78486764	1	7	7.42582367	4E+06	4180482	ID=ENSP0000370787-D2;;					
11	4E+06	11_4172070	5	16	0	9	11	5	0.189421	1	0	10	4	2	5	3	0.8350419	1	5.75	5.57417515	4E+06	4180482	ID=ENSP0000370787-D2;;		4171563	4172238	0.19351	ID=promoter_08400;
11	4E+06	11_42240	1	1	5	7	1	6	0.241978	1	56	1	2	0	29	1	5.87E-06	0.000796	9.125	19.0895446								
11	4E+06	11_42934	7	0	5	11	4	8	0.677894	1	0	9	7	1	5	4	0.97610903	1	5	4.24264069								
11	4E+06	11_43859	32	3	12	16	18	14	0.509661	1	15	1	21	0	8	11	0.69450319	1	12.5	11.0194633								
11	4E+06	11_4427786	5	1	9	19	3	14	0.063635	1	4	3	18	8	4	13	0.06462238	1	8.375	6.75991336					4427735	4428383	0.15282	ID=promoter_08424;
11	4E+06	11_44959	0	12	17	11	6	14	0.355886	1	7	7	0	0	7	0	0.03898208	1	6.75	6.40869944								
11	5E+06	11_46925	18	1	7	23	10	15	0.833288	1	14	7	3	0	11	2	0.06154634	1	9.125	8.37406711								
11	5E+06	11_4853325	212	75	183	164	144	174	0.965806	1	51	36	33	4	44	19	0.01716617	0.6063917	94.75	79.4044799					4850779	4855874	0.80624	ID=promoter_08480;
11	5E+06	11_4872342	31	11	7	14	21	11	0.084122	1	17	11	12	1	14	7	0.39930956	1	13	8.70139562	5E+06	4872818	ID=ENSP0000333490;PRHOXNB;NM_001105577;	PRHOXNB	4871590	4873693	0.7093	ID=promoter_08482;
11	5E+06	11_4875104	128	17	134	123	73	129	0.018871	0.67244	54	16	14	15	35	15	0.03750218	1	62.625	56.0304445					4874421	4875687	0.23534	ID=promoter_08483;
11	5E+06	11_49375	3	30	6	0	17	3	0.002206	0.1213	0	5	2	0	3	1	0.65196691	1	5.75	10.0676285								
11	5E+06	11_49610	10	0	3	3	5	3	0.645778	1	16	0	4	27	8	16	0.19094317	1	7.875	9.43303466								
11	5E+06	11_5001634	56	6	116	87	31	102	1.01E-06	0.00015	3	11	16	5	7	11	0.54353855	1	37.5	43.6839297	5E+06	5092279	ID=ENSP0000382529;PAN3;;	PAN3	5001319	5002529	0.31092	ID=promoter_08492;
11	5E+06	11_5013040	1	35	1	1	18	1	1.14E-04	0.01003	11	29	0	2	20	1	3.70E-04	0.0275298	10	14.1117985	5E+06	5092279	ID=ENSP0000382529;PAN3;;	PAN3	5012223	5013520	0.17108	ID=promoter_08493;
11	5E+06	11_5013199	13	13	19	12	13	16	0.991731	1	19	8	20	3	14	12	0.98032967	1	13.375	5.9266348	5E+06	5092279	ID=ENSP0000382529;PAN3;;	PAN3	5012223	5013520	0.17108	ID=promoter_08493;
11	5E+06	11_5042294	33	14	36	8	24	22	0.704268	1	9	11	6	0	10	3	0.19568363	1	14.625	12.9387292	5E+06	5092279	ID=ENSP0000382529;PAN3;;	PAN3	5042175	5042893	0.12547	ID=promoter_08495;
11	5E+06	11_5089075	4	30	9	32	17	21	0.98748	1	67	19	67	29	43	48	0.67540917	1	32.125	23.7392833	5E+06	5092279	ID=ENSP0000382529;PAN3;;	PAN3	5088175	5089433	0.31415	ID=promoter_08499;

11	5E+06	11_5161047	402	98	332	306	250	319	0.60824	1	33	60	20	33	47	27	0.13514307	1	160.5	158.187953					5160569	5161354	0.18454	ID=promoter_08520;
11	5E+06	11_51666	38	22	31	9	30	20	0.139907	1	40	34	17	69	37	43	0.60036427	1	32.5	18.2287058								
11	5E+06	11_51802	6	3	5	4	5	5	0.968625	1	16	1	8	0	9	4	0.4571786	1	5.375	5.01248441								
11	5E+06	11_5217492	135	74	69	54	105	62	7.13E-05	0.00662	102	1	42	11	52	27	0.04641028	1	61	44.5613542					5217291	5217976	0.13189	ID=promoter_08533;
11	5E+06	11_52636	3063	1425	2617	2314	2244	2466	0.082315	1	0	48	27	19	24	23	0.99354957	1	1189.1	1325.95663								
11	5E+06	11_53011	16	41	13	6	29	10	0.001829	0.10386	0	3	27	7	2	17	0.00148567	0.0868666	14.125	13.7989389								
11	5E+06	11_53731	15	0	10	6	8	8	0.950274	1	0	11	12	31	6	22	0.00549069	0.2478586	10.625	9.88415037								
11	5E+06	11_53913	4	0	14	1	2	8	0.255957	1	25	11	1	0	18	1	9.25E-04	0.05912	7	9.00793301								
11	5E+06	11_54015	1	0	1	0	1	1	0.993645	1	24	5	5	8	15	7	0.31064188	1	5.5	8.01783726								
11	5E+06	11_54105	3	118	24	6	61	15	1.62E-08	#######	43	32	46	4	38	25	0.40838944	1	34.5	37.8493253								
11	5E+06	11_54478	23	12	10	8	18	9	0.098614	1	0	0	6	0	0	3	0.19828566	1	7.375	7.90908158								
11	5E+06	11_5458584	317	101	182	202	209	192	0.049654	1	11	73	34	65	42	50	0.48789246	1	123.13	102.936093					5458409	5458977	0.13887	ID=promoter_08555;
11	6E+06	11_55807	47	109	78	28	78	53	0.00806	0.34477	86	30	79	56	58	68	0.42022787	1	64.125	28.6228555								
11	6E+06	11_5588862	43	399	17	18	221	18	8.46E-46	#######	17	335	25	29	176	27	4.05E-22	3.11E-19	110.38	159.544832					5586691	5588936	0.25344	ID=promoter_08566;
11	6E+06	11_56103	32	33	2	11	33	7	2.22E-05	0.00241	155	72	101	87	114	94	0.70192571	1	61.625	51.8733003								
11	6E+06	11_5675063	103	70	265	229	87	247	2.26E-12	#######	75	51	37	49	63	43	0.29900176	1	109.88	87.497653					5674383	5675545	0.27361	ID=promoter_08573;
11	6E+06	11_5691814	0	25	22	22	13	22	0.573969	1	0	9	10	2	5	6	0.91271846	1	11.25	10.4573966					5690868	5692168	0.17963	ID=promoter_08578;
11	6E+06	11_57556	2	22	0	6	12	3	0.030625	0.98374	1	11	11	1	6	6	0.99142475	1	6.75	7.59228932								
11	6E+06	11_57831	18	2	8	12	10	10	0.938234	1	7	2	1	22	5	12	0.17891578	1	9	7.80109882								
11	6E+06	11_57913	15	24	3	4	20	4	0.0012	0.07347	0	12	2	1	6	2	0.42506085	1	7.625	8.53459347								
11	6E+06	11_58559	154	21	156	189	88	173	3.52E-04	0.02658	39	8	36	2	24	19	0.87598349	1	75.625	76.8541058								
11	6E+06	11_5874614	46	35	53	50	41	52	0.932362	1	4	22	25	1	13	13	0.98151319	1	29.5	20.0499377					5872681	5875891	0.45015	ID=promoter_08597;
11	6E+06	11_5911756	123	25	86	80	74	83	0.96158	1	64	10	70	10	37	40	0.79650037	1	58.5	40.2988834					5911373	5912926	0.24229	ID=promoter_08603;
11	6E+06	11_60237	100	103	162	81	102	122	0.989469	1	31	14	1	0	23	1	9.32E-05	0.0086863	61.5	58.9358004								
11	6E+06	11_6030278	383	289	668	481	336	575	2.24E-07	#######	242	181	198	45	212	122	1.27E-04	0.0112073	310.88	195.376148					6029965	6031595	0.23609	ID=promoter_08617;
11	6E+06	11_6106968	125	41	86	83	83	85	0.678869	1	28	13	31	24	21	28	0.4487548	1	53.875	39.3897648					6105648	6107276	0.23519	ID=promoter_08637;
11	6E+06	11_61974	344	193	354	309	269	332	0.824874	1	168	187	244	57	178	151	0.7247361	1	232	101.254982								
11	6E+06	11_6198780	324	69	244	395	197	320	0.001473	0.08724	154	122	183	128	138	156	0.23568631	1	202.38	110.922029					6197591	6199846	0.7406	ID=promoter_08642;
11	6E+06	11_62404	49	17	51	65	33	58	0.174905	1	1	7	41	7	4	24	3.59E-04	0.0268372	29.75	24.5400547								
11	6E+06	11_6302917	8	56	35	7	32	21	0.11093	1	68	53	19	7	61	13	9.92E-07	0.0001649	31.625	24.8304968					6302853	6303893	0.168	ID=promoter_08653;
11	6E+06	11_6374316	7	24	3	0	16	2	0.001284	0.07728	14	16	35	3	15	19	0.66253156	1	12.75	12.0682582					6373148	6376055	0.40953	ID=promoter_08667;
11	6E+06	11_6375542	90	53	31	40	72	36	9.46E-05	0.00849	38	26	29	2	32	16	0.1226614	1	38.625	25.3542755					6373148	6376055	0.40953	ID=promoter_08667;
11	6E+06	11_63850	162	76	140	98	119	119	0.468277	1	36	47	32	19	42	26	0.26520775	1	76.25	52.917051								
11	7E+06	11_65225	325	146	352	237	236	295	0.766891	1	140	160	180	63	150	122	0.56164897	1	200.38	98.1120897								
11	7E+06	11_66055	457	166	297	222	312	260	2.32E-04	0.01845	398	117	208	70	258	139	1.20E-06	0.0001963	241.88	134.23161								
11	7E+06	11_66220	37	17	57	34	27	46	0.301451	1	1	0	14	2	1	8	0.04988758	1	20.25	20.6449579								
11	7E+06	11_6623272	521	222	548	490	372	519	0.038045	1	58	109	81	79	84	80	0.98521335	1	263.5	218.343766					6623115	6624272	0.24204	ID=promoter_08694;
11	7E+06	11_6865625	22	56	32	27	39	30	0.216196	1	42	16	46	4	29	25	0.96515949	1	30.625	16.9952725					6865267	6865767	0.11467	ID=promoter_08709;
11	7E+06	11_6940174	137	117	208	224	127	216	0.003755	0.18662	51	88	82	90	70	86	0.21432426	1	124.63	61.8937742	7E+06	6952440	ID=ENSP00000369849;C13orf33;NM_032849;	C13orf33				
11	7E+06	11_69590	92	93	75	117	93	96	0.679669	1	25	12	19	2	19	11	0.45547225	1	54.375	44.5739754								
11	7E+06	11_72706	1	8	5	3	5	4	0.848147	1	0	19	6	32	10	19	0.1719433	1	9.25	10.9250695								

11	7E+06	11_7333173	0	31	1	8	16	5	0.021336	0.74103	13	16	11	22	15	17	0.85096642	1	12.75	10.3888677	7E+06	7372105	ID=ENSP00000343002;B3GALTL;NM_194318;	B3GALTL	7332116	7333668	0.22456	ID=promoter_08732;
11	7E+06	11_7465355	872	263	534	620	568	577	0.051576	1	249	181	260	60	215	160	0.1082823	1	379.88	270.087519					7464747	7466052	0.19775	ID=promoter_08745;
11	8E+06	11_76346	10	57	36	41	34	39	0.995484	1	25	9	29	1	17	15	0.99031847	1	26	18.7692758								
11	8E+06	11_76717	12	64	16	25	38	21	0.018051	0.65084	71	26	41	9	49	25	0.04780316	1	33	23.5675321								
11	8E+06	11_77570	258	110	310	344	184	327	3.33E-05	0.00341	94	95	83	91	95	87	0.9966182	1	173.13	111.06811								
11	8E+06	11_78924	23	13	8	15	18	12	0.307258	1	91	15	60	25	53	43	0.80949557	1	31.25	29.0455308								
11	8E+06	11_79025	62	23	38	55	43	47	0.945585	1	38	0	8	26	19	17	0.99437087	1	31.25	21.4259522								
11	8E+06	11_7955459	9	48	14	12	29	13	0.011304	0.44898	2	2	10	4	2	7	0.20455692	1	12.625	14.9946419					7954536	7955570	0.18887	ID=promoter_08773;
11	8E+06	11_81206	12	6	9	2	9	6	0.554309	1	3	7	13	1	5	7	0.77775394	1	6.625	4.50198369								
11	8E+06	11_81389	13	34	3	12	24	8	0.004476	0.21437	0	16	22	8	8	15	0.25959112	1	13.5	10.8496215								
11	8E+06	11_81843	131	59	102	105	95	104	0.888453	1	24	36	24	0	30	12	0.0403413	1	60.125	47.2060453								
11	8E+06	11_82389	8	17	5	8	13	7	0.234546	1	27	12	38	10	20	24	0.69398062	1	15.625	11.375883								
11	8E+06	11_82716	48	68	37	10	58	24	4.20E-05	0.00416	19	106	63	76	63	70	0.5796554	1	53.375	31.5048182								
11	9E+06	11_85701	55	79	61	82	67	72	0.87538	1	22	23	16	0	23	8	0.04560275	1	42.25	30.9365711								
11	9E+06	11_93933	0	11	7	7	6	7	0.999953	1	25	15	1	28	20	15	0.82431124	1	11.75	10.3475325								
11	9E+06	11_9399403	16	0	23	7	8	15	0.557319	1	39	48	29	1	44	15	0.00228772	0.1229844	20.375	17.646833					9399026	9399597	0.12719	ID=promoter_08828;
11	9E+06	11_94582	13	54	33	79	34	56	0.292087	1	56	10	32	16	33	24	0.66283249	1	36.625	24.4945329								
11	9E+06	11_94591	292	212	385	265	252	325	0.52769	1	73	73	50	0	73	25	4.44E-05	0.0045939	168.75	138.359521								
11	9E+06	11_94852	32	56	73	57	44	65	0.496003	1	13	4	85	6	9	46	9.14E-07	0.0001543	40.75	31.3949495								
11	1E+07	11_97486	11	0	6	7	6	7	0.999953	1	9	2	7	22	6	15	0.10150651	1	8	6.67618368								
11	1E+07	11_10147093	11	8	4	0	10	2	0.03464	1	0	23	11	22	12	17	0.52622129	1	9.875	8.90324981								
11	1E+07	11_10223421	4	48	24	37	26	31	0.998108	1	71	43	50	7	57	29	0.02830192	0.8918846	35.5	22.7470563								
11	1E+07	11_10753489	1	16	8	10	9	9	0.944235	1	18	0	13	17	9	15	0.36943449	1	10.375	6.98851098								
11	1E+07	11_11220785	1	7	5	11	4	8	0.677894	1	9	3	14	0	6	7	0.91781435	1	6.25	4.92080133								
11	1E+07	11_11362835	32	40	52	74	36	63	0.155962	1	165	88	70	15	127	43	2.02E-08	4.75E-06	67	46.2693975	1E+07	1.1E+07	ID=ENSP00000353846;DCLK1;;	DCLK1				
11	1E+07	11_11494517	643	196	843	712	420	778	2.61E-13	#######	936	414	106	399	675	253	8.49E-36	1.11E-32	531.13	300.151599					1.1E+07	1.1E+07	0.54865	ID=promoter_08867;
11	1E+07	11_11534523	20	8	11	39	14	25	0.447365	1	77	18	33	62	48	48	0.93342204	1	33.5	24.8251025								
11	1E+07	11_11623008	9	0	4	0	5	2	0.402696	1	3	16	12	0	10	6	0.69729498	1	5.5	6.14119579								
11	1E+07	11_11729451	118	104	90	113	111	102	0.203668	1	134	110	83	97	122	90	0.25730864	1	106.13	16.3133381					1.2E+07	1.2E+07	0.48028	ID=promoter_08879;
11	1E+07	11_11780707	68	48	27	33	58	30	9.06E-04	0.05835	20	36	67	34	28	51	0.01382034	0.5095422	41.625	17.8320538								
11	1E+07	11_12093708	19	28	20	28	24	24	0.858118	1	14	17	15	33	16	24	0.32201294	1	21.75	7.00510018								
11	1E+07	11_12610216	3	24	16	10	14	13	0.831264	1	1	23	23	41	12	32	0.00478712	0.2203477	17.625	13.0705503								
11	1E+07	11_12971665	8	16	11	29	12	20	0.626521	1	0	0	11	3	0	7	0.02292696	0.7568963	9.75	9.64735345	1E+07	1.3E+07	ID=ENSP00000369003;TRPC4;NM_003306;	TRPC4				
11	1E+07	11_12999516	26	75	44	44	51	44	0.325273	1	74	92	75	25	83	50	0.0526458	1	56.875	25.3119819	1E+07	1.3E+07	ID=ENSP00000369003;TRPC4;NM_003306;	TRPC4	1.3E+07	1.3E+07	0.12266	ID=promoter_08894;
11	1E+07	11_13592183	55	45	18	63	50	41	0.232493	1	25	15	17	1	20	9	0.18397305	1	29.875	21.8529991								
11	1E+07	11_13681358	41	69	22	10	55	16	7.30E-07	0.00011	0	24	2	26	12	14	0.84238319	1	24.25	22.606889								
11	1E+07	11_13692052	1	42	1	0	22	1	1.12E-05	0.00132	0	14	4	10	7	7	0.99000271	1	9	14.2928554								
11	1E+07	11_14264592	6	9	31	8	8	20	0.179808	1	36	21	53	0	29	27	0.99987101	1	20.5	18.2287058								

11	1E+07	11_14721029	0	17	12	23	9	18	0.416978	1	0	0	9	0	0	5	0.06742467	1	7.625	9.08589959								
11	1E+07	11_14820809	14	13	7	9	14	8	0.253058	1	0	2	0	1	1	1	0.99856566	1	5.75	5.8002463					1.5E+07	1.5E+07	0.17438	ID=promoter_08920;
11	1E+07	11_14855516	53	37	29	36	45	33	0.117793	1	5	11	24	2	8	13	0.4490005	1	24.625	17.6872634					1.5E+07	1.5E+07	0.13897	ID=promoter_08925;
11	1E+07	11_14879389	14	0	7	24	7	16	0.324814	1	2	27	35	30	15	33	0.01662686	0.5898213	17.375	13.4370224					1.5E+07	1.5E+07	0.09434	ID=promoter_08930;
11	1E+07	11_14895637	504	167	365	368	336	367	0.640355	1	35	51	75	33	43	54	0.32914056	1	199.75	185.893018					1.5E+07	1.5E+07	0.19519	ID=promoter_08931;
11	1E+07	11_14949718	1	54	26	22	28	24	0.526767	1	11	49	29	4	30	17	0.26150314	1	24.5	19.471591								
11	1E+07	11_14966516	12	5	5	2	9	4	0.244237	1	84	35	1	11	60	6	1.82E-09	5.06E-07	19.375	28.2789043								
11	2E+07	11_15081619	38	14	56	20	26	38	0.687307	1	38	46	42	20	42	31	0.627947	1	34.25	14.713939								
11	2E+07	11_15178108	128	11	94	105	70	100	0.448064	1	0	1	1	0	1	1	0.99856566	1	42.5	55.9566158								
11	2E+07	11_15207982	445	164	336	321	305	329	0.573623	1	59	195	145	36	127	91	0.17062042	1	212.63	142.933489					1.5E+07	1.5E+07	0.87712	ID=promoter_08943;
11	2E+07	11_15492309	14	6	12	8	10	10	0.938234	1	8	6	7	4	7	6	0.99011599	1	8.125	3.31393163								
11	2E+07	11_15506820	42	12	32	14	27	23	0.526863	1	4	4	1	1	4	1	0.45332778	1	13.75	15.3506631	2E+07	1.6E+07	ID=ENSP00000343109;TPTE2;;	TPTE2				
11	2E+07	11_15759227	13	54	31	22	34	27	0.313685	1	1	36	37	4	19	21	0.85675254	1	24.75	18.1717992								
11	2E+07	11_15769520	596	296	665	567	446	616	0.031387	1	295	259	188	133	277	161	1.24E-05	0.0015211	374.88	203.341681					1.6E+07	1.6E+07	0.44873	ID=promoter_08963;
11	2E+07	11_15832360	0	25	14	5	13	10	0.600182	1	28	14	10	3	21	7	0.05007015	1	12.375	10.0702887	2E+07	1.6E+07	ID=ENSP00000347767;NEK5;NM_199289;	NEK5	1.6E+07	1.6E+07	0.13052	ID=promoter_08967;
11	2E+07	11_15832443	47	176	37	22	112	30	4.01E-14	#######	60	65	93	28	63	61	0.97110735	1	66	49.925659	2E+07	1.6E+07	ID=ENSP00000347767;NEK5;NM_199289;	NEK5	1.6E+07	1.6E+07	0.13052	ID=promoter_08967;
11	2E+07	11_15905929	7	31	16	0	19	8	0.039867	1	60	20	14	20	40	17	0.02201768	0.7404021	21	18.2600266								
11	2E+07	11_16005081	1	8	21	19	5	20	0.033145	1	0	12	18	18	6	18	0.03091566	0.954015	12.125	8.30554892								
11	2E+07	11_16060554	15	1	13	3	8	8	0.950274	1	31	25	74	25	28	50	0.01836895	0.6365951	23.375	23.0523473								
11	2E+07	11_16070148	1183	741	881	881	962	881	7.12E-07	0.00011	485	495	406	336	490	371	0.01301563	0.4862398	676	293.517096								
11	2E+07	11_16171280	318	65	107	87	192	97	2.96E-11	#######	65	31	50	1	48	26	0.08258093	1	90.5	97.4913916	2E+07	1.6E+07	ID=ENSP00000298125;WDFY2;NM_052950;	WDFY2				
11	2E+07	11_16377688	4	28	6	0	16	3	0.003782	0.18716	20	3	10	18	12	14	0.84238319	1	11.125	9.8479512								
11	2E+07	11_16404543	9	30	6	13	20	10	0.076235	1	1	23	7	2	12	5	0.30420563	1	11.375	10.2391057								
11	2E+07	11_16940951	6	2	2	20	4	11	0.322758	1	0	13	10	0	7	5	0.9051169	1	6.625	7.19002285								
11	2E+07	11_17665673	7	22	0	13	15	7	0.113635	1	2	21	11	17	12	14	0.84238319	1	11.625	8.24512843								
11	2E+07	11_17673142	16	17	2	1	17	2	7.31E-04	0.04855	20	11	12	0	16	6	0.14768941	1	9.875	7.88194139								
11	2E+07	11_17926681	65	52	26	7	59	17	2.30E-07	#######	35	0	30	9	18	20	0.85572507	1	28	22.6400404								
11	2E+07	11_17932194	2	6	8	8	4	8	0.677894	1	2	1	12	8	2	10	0.05067035	1	5.875	3.87067731								
11	2E+07	11_18057839	20	78	9	22	49	16	1.26E-05	0.00147	46	31	20	27	39	24	0.28229872	1	31.625	21.5601319								
11	2E+07	11_18428700	1	8	9	8	5	9	0.74134	1	5	5	5	16	5	11	0.25523295	1	7.125	4.38951673								

11	2E+07	11_18531894	0	19	7	24	10	16	0.741563	1	8	9	11	5	9	8	0.99626583	1	10.375	7.7078346								
11	2E+07	11_18635815	4	1	5	13	3	9	0.343967	1	66	13	2	4	40	3	4.70E-07	8.45E-05	13.5	21.7058254								
11	2E+07	11_18839216	40	19	78	50	30	64	0.023183	0.79379	21	15	40	0	18	20	0.85572507	1	32.875	24.3922558								
11	2E+07	11_18868517	10	31	16	13	21	15	0.333352	1	25	1	21	1	13	11	0.97545578	1	14.75	10.780273								
11	2E+07	11_19113089	0	0	6	8	0	7	0.050594	1	18	12	21	19	15	20	0.56420259	1	10.5	8.34950811								
11	2E+07	11_19174282	39	21	42	34	30	38	0.95166	1	29	17	37	2	23	20	0.9783171	1	27.625	13.5218078								
11	2E+07	11_19281898	19	79	2	14	49	8	1.12E-08	#######	59	49	58	33	54	46	0.9149187	1	39.125	26.4328664								
11	2E+07	11_19290385	2	15	4	15	9	10	0.993074	1	0	7	26	1	4	14	0.04184353	1	8.75	9.1300133								
11	2E+07	11_19319409	30	15	13	15	23	14	0.143642	1	28	33	24	35	31	30	0.98602473	1	24.125	8.75764972								
11	2E+07	11_19793436	8	7	16	0	8	8	0.950274	1	0	0	17	0	0	9	0.00779604	0.3222758	6	7.2702918								
11	2E+07	11_20135379	11	13	7	17	12	12	0.926346	1	44	2	7	3	23	5	0.00590668	0.2600594	13	13.4907376					2E+07	2E+07	0.1929	ID=promoter_09057;
11	2E+07	11_20275992	737	507	2213	1639	622	1926	########	#######	963	739	1213	604	851	909	0.01204432	0.4571298	1076.9	587.525668								
11	2E+07	11_20362399	5	56	0	5	31	3	8.71E-07	0.00013	33	20	14	0	27	7	0.00575936	0.2548307	16.625	19.4931307								
11	2E+07	11_20491721	46	37	32	54	42	43	0.821371	1	0	0	10	1	0	6	0.0393172	1	22.5	22.2838827	2E+07	2.1E+07	ID=ENSP0000368082;LRRC63;;	LRRC63				
11	2E+07	11_20567787	44	134	46	51	89	49	7.65E-05	0.00706	70	48	123	52	59	88	0.01708352	0.6036215	71	36.4848305								
11	2E+07	11_20675877	1351	649	877	822	1000	850	3.65E-11	#######	258	249	382	170	254	276	0.18792184	1	594.75	407.918672								
11	2E+07	11_20918799	17	9	5	8	13	7	0.234546	1	50	15	29	4	33	17	0.1362937	1	17.125	15.5603663								
11	2E+07	11_20924824	7	0	11	6	4	9	0.54411	1	24	15	12	14	20	13	0.60545249	1	11.125	7.14017807								
11	2E+07	11_20980277	14	14	1	1	14	1	0.001161	0.07164	61	1	19	2	31	11	0.01758669	0.6154554	14.125	20.3008621					2.1E+07	2.1E+07	0.12512	ID=promoter_09094;
11	2E+07	11_21009405	0	3	7	10	2	9	0.179815	1	11	10	22	5	11	14	0.73202476	1	8.5	6.65475126								
11	2E+07	11_21204132	0	0	3	18	0	11	0.009196	0.38047	0	0	14	18	0	16	1.79E-04	0.0149638	6.625	8.46737436								
11	2E+07	11_21296413	95	79	170	170	87	170	4.72E-04	0.03365	2	10	8	20	6	14	0.1466131	1	69.25	70.9823922								
11	2E+07	11_21840053	7	4	4	7	6	6	0.962469	1	18	17	18	0	18	9	0.30718372	1	9.375	7.20986427								
11	2E+07	11_21876881	97	93	117	68	95	93	0.462914	1	42	45	42	20	44	31	0.50195989	1	65.5	33.8103957								
11	2E+07	11_21964391	111	66	81	110	89	96	0.850179	1	41	9	6	0	25	3	3.93E-04	0.0289246	53	45.7696094								
11	2E+07	11_22173177	21	57	27	12	39	20	0.008322	0.35239	36	15	3	47	26	25	0.99152959	1	27.25	18.3672846								
11	2E+07	11_22473561	16	25	3	9	21	6	0.004166	0.20266	1	1	10	26	1	18	2.55E-04	0.0200383	11.375	10.0986208								
11	2E+07	11_22488572	4	27	3	0	16	2	0.001284	0.07728	0	10	5	4	5	5	0.99284883	1	6.625	8.81455776								
11	2E+07	11_22679326	16	65	39	15	41	27	0.06151	1	41	51	85	4	46	45	0.96763695	1	39.5	27.4330354								
11	2E+07	11_22820251	17	41	9	11	29	10	0.001829	0.10386	52	24	24	17	38	21	0.15716135	1	24.375	14.9469299					2.3E+07	2.3E+07	0.13537	ID=promoter_09138;
11	2E+07	11_23064105	0	24	0	9	12	5	0.126784	1	2	7	0	0	5	0	0.09850879	1	5.25	8.3623305								
11	2E+07	11_23235919	12	75	19	25	44	22	0.003473	0.17542	108	54	40	48	81	44	0.01544033	0.5539234	47.625	31.8341124								
11	2E+07	11_23283519	2	26	12	22	14	17	0.995406	1	25	15	4	13	20	9	0.18397305	1	14.875	9.04650682								
11	2E+07	11_23462242	57	76	36	27	67	32	8.74E-05	0.00788	107	74	120	28	91	74	0.7040516	1	65.625	35.2660804	2E+07	2.3E+07	ID=ENSP0000239849;TNFSF11;NM_003701;	TNFSF11				

11	2E+07	11_23689204	0	20	11	0	10	6	0.41703	1	1	13	11	29	7	20	0.02608553	0.8424716	10.625	10.3501208	2E+07	2.4E+07	ID=ENSP00000025301;AKAP11;NM_016248;	AKAP11	2.4E+07	2.4E+07	0.67629	ID=promoter_09149;
11	2E+07	11_23689375	0	11	3	1	6	2	0.258649	1	22	2	0	1	12	1	0.01422424	0.5202299	5	7.74596669	2E+07	2.4E+07	ID=ENSP00000025301;AKAP11;NM_016248;	AKAP11	2.4E+07	2.4E+07	0.67629	ID=promoter_09149;
11	2E+07	11_23849137	2	41	24	3	22	14	0.192025	1	1	7	23	13	4	18	0.00668749	0.2868305	14.25	14.1092675								
11	2E+07	11_23862341	21	37	9	17	29	13	0.011304	0.44898	54	31	13	24	43	19	0.02267705	0.755811	25.75	14.6262973								
11	2E+07	11_23878073	15	67	15	2	41	9	2.20E-06	0.00031	7	39	14	4	23	9	0.07737643	1	20.375	22.051482								
11	2E+07	11_23907845	8	47	24	10	28	17	0.092775	1	50	8	2	72	29	37	0.43334569	1	27.625	25.6233013								
11	2E+07	11_23931744	18	26	23	12	22	18	0.522468	1	16	14	10	2	15	6	0.20085144	1	15.125	7.54865172								
11	2E+07	11_23941144	8	25	5	9	17	7	0.051092	1	11	0	31	0	6	16	0.069189	1	11.125	11.2559508					2.4E+07	2.4E+07	0.5462	ID=promoter_09152;
11	2E+07	11_24452972	0	9	0	5	5	3	0.645778	1	32	9	1	2	21	2	7.81E-04	0.0515074	7.25	10.6603404					2.4E+07	2.4E+07	0.56582	ID=promoter_09155;
11	2E+07	11_24669400	130	71	108	61	101	85	0.076757	1	0	10	3	3	5	3	0.8350419	1	48.25	51.8507473								
11	2E+07	11_24673215	178	65	249	186	122	218	8.82E-04	0.05714	150	61	101	2	106	52	6.71E-04	0.0452348	124	80.9973545								
11	2E+07	11_24770668	13	48	29	28	31	29	0.68044	1	81	24	29	15	53	22	0.00501977	0.2287702	33.375	22.0060868								
11	3E+07	11_25024732	245	88	302	231	167	267	0.00696	0.30326	45	44	84	86	45	85	4.21E-04	0.0307791	140.63	101.800838					2.5E+07	2.5E+07	0.37679	ID=promoter_09168;
11	3E+07	11_25701725	3	22	16	7	13	12	0.835334	1	0	3	96	40	2	68	2.29E-15	1.19E-12	23.375	32.1866765								
11	3E+07	11_26181310	208	149	177	133	179	155	0.021175	0.73796	136	103	91	38	120	65	0.00195974	0.1077881	129.38	52.6577562								
11	3E+07	11_26458185	98	235	167	132	167	150	0.058954	1	421	175	300	74	298	187	1.42E-04	0.0123104	200.25	115.067372								
11	3E+07	11_26497938	19	10	12	10	15	11	0.4902	1	10	11	10	12	11	11	0.98433491	1	11.75	3.05894473								
11	3E+07	11_26574711	4	37	0	0	21	0	4.48E-06	0.00059	0	6	0	3	3	2	0.9363008	1	6.25	12.6349854								
11	3E+07	11_26646919	0	43	2	1	22	2	4.25E-05	0.00421	3	2	5	2	3	4	0.89184014	1	7.25	14.5184612								
11	3E+07	11_26708524	37	10	34	25	24	30	0.97337	1	37	28	17	12	33	15	0.0650987	1	25	10.928338					2.7E+07	2.7E+07	0.63657	ID=promoter_09173;
11	3E+07	11_26743922	13	24	17	14	19	16	0.617999	1	8	8	9	7	8	8	0.98858271	1	12.5	5.83095189								
11	3E+07	11_26775233	197	236	217	242	217	230	0.563342	1	114	132	191	91	123	141	0.22617894	1	177.5	57.6813909								
11	3E+07	11_26952806	1	4	0	2	3	1	0.508575	1	24	2	3	23	13	13	0.98151319	1	7.375	10.0276404	3E+07	2.7E+07	ID=ENSP00000219022;OLFM4;NM_006418;	OLFM4				
11	3E+07	11_27047572	1438	351	989	795	895	892	0.002634	0.14068	366	240	366	83	303	225	0.04111755	1	578.5	456.116213								
11	3E+07	11_28703120	1	62	18	27	32	23	0.194505	1	40	20	64	77	30	71	4.72E-05	0.0048519	38.625	26.6936991								
11	3E+07	11_30362312	16	90	21	50	53	36	0.037651	1	50	94	70	37	72	54	0.49880225	1	53.5	29.2867596								
11	3E+07	11_30650022	1	27	8	17	14	13	0.831264	1	24	6	13	0	15	7	0.31064188	1	12	10.0853501								
11	3E+07	11_32208849	107	527	70	132	317	101	3.28E-33	#######	152	296	345	161	224	253	0.09781829	1	223.75	154.298181								
11	3E+07	11_32376582	177	95	149	65	136	107	0.007914	0.33925	98	77	64	34	88	49	0.01531749	0.5531297	94.875	47.1394209								
11	3E+07	11_34681726	1	4	8	0	3	4	0.986002	1	15	6	6	2	11	4	0.25131486	1	5.25	4.80327269								
11	3E+07	11_34719405	0	28	18	2	14	10	0.480768	1	0	8	18	0	4	9	0.31291743	1	9.25	10.7935166					3.5E+07	3.5E+07	0.72381	ID=promoter_09189;

11	3E+07	11_34873165	51	229	21	33	140	27	1.34E-21	#######	339	225	45	40	282	43	4.11E-35	5.19E-32	122.88	122.449334					3.5E+07	3.5E+07	1	ID=promoter_09190;
11	3E+07	11_34875159	10	24	12	23	17	18	0.955187	1	32	0	13	23	16	18	0.85288016	1	17.125	10.1761275					3.5E+07	3.5E+07	1	ID=promoter_09190;
11	4E+07	11_36056750	66	183	66	90	125	78	5.08E-05	0.0049	174	147	192	118	161	155	0.94520672	1	129.5	51.8404145								
11	4E+07	11_36289331	0	9	15	8	5	12	0.389128	1	28	19	11	38	24	25	0.92007618	1	16	12.1420168								
11	4E+07	11_40379268	2	5	14	12	4	13	0.177139	1	14	19	33	15	17	24	0.40932813	1	14.25	9.37702359								
11	4E+07	11_42027923	19	67	37	14	43	26	0.024991	0.84297	98	12	36	3	55	20	0.00100251	0.0626991	35.75	32.1680853								
11	4E+07	11_43855879	13	15	15	8	14	12	0.725787	1	0	29	5	0	15	3	0.02833385	0.8918846	10.625	9.60561592								
11	4E+07	11_43989176	8	8	8	1	8	5	0.532535	1	3	9	12	0	6	6	0.99142475	1	6.125	4.25734659								
11	4E+07	11_44479397	14	25	6	16	20	11	0.118302	1	0	0	0	0	0	0	1	1	7.625	9.62047667								
11	4E+07	11_44596087	7	47	1	33	27	17	0.124321	1	14	3	13	22	9	18	0.15524483	1	17.5	15.8384703					4.5E+07	4.5E+07	0.69316	ID=promoter_09204;
11	5E+07	11_45192330	63	44	39	45	54	42	0.132387	1	108	39	38	80	74	59	0.68809469	1	57	25.3207989								
11	5E+07	11_45326266	117	172	255	150	145	203	0.265108	1	100	72	77	124	86	101	0.27107026	1	133.38	59.7325886								
11	5E+07	11_45485420	6	30	0	9	18	5	0.008132	0.34618	12	10	12	25	11	19	0.24832023	1	13	9.84160266								
11	5E+07	11_45580497	2	9	1	0	6	1	0.108618	1	1	15	4	12	8	8	0.98858271	1	5.5	5.73211504								
11	5E+07	11_45596232	13	44	1	0	29	1	1.88E-07	#######	0	8	8	19	4	14	0.04184353	1	11.625	14.7060483								
11	5E+07	11_46384723	2	9	1	2	6	2	0.258649	1	27	2	1	1	15	1	0.00364468	0.1775497	5.625	9.03860767								
11	5E+07	11_46404229	12	0	3	0	6	2	0.258649	1	4	12	24	3	8	14	0.34554715	1	7.25	8.25919401								
11	5E+07	11_46428005	103	68	88	60	86	74	0.146002	1	1	33	28	5	17	17	0.97589399	1	48.25	37.514759								
11	5E+07	11_46682748	4500	4401	2365	2338	4451	2352	########	#######	722	890	774	256	806	515	2.75E-10	8.65E-08	2030.8	1676.07967								
11	5E+07	11_47034795	134	66	96	53	100	75	0.01281	0.49596	73	71	20	54	72	37	0.01254306	0.4715466	70.875	33.4597306								
11	5E+07	11_47458006	80	120	67	35	100	51	4.09E-06	0.00054	185	138	185	122	162	154	0.9754152	1	116.5	53.8702144								
11	5E+07	11_48356620	80	24	11	15	52	13	2.75E-07	#######	29	14	11	5	22	8	0.06315916	1	23.625	24.0234261								
11	5E+07	11_49085417	13	2	5	1	8	3	0.206144	1	0	15	19	26	8	23	0.01462253	0.532614	10.125	9.56836305	5E+07	4.9E+07	ID=ENSP00000366416-D2;EDNRB;;	EDNRB				
11	5E+07	11_49250841	5	49	7	19	27	13	0.024366	0.82541	66	16	27	20	41	24	0.19624561	1	26.125	21.101371								
11	5E+07	11_49281913	18	32	20	20	25	20	0.43889	1	50	36	38	36	43	37	0.94624198	1	31.25	11.1579568								
11	5E+07	11_49608228	112	99	88	108	106	98	0.234809	1	96	80	107	127	88	117	0.0368544	1	102.13	14.6914698								
11	5E+07	11_49632431	693	253	511	429	473	470	0.038728	1	124	252	229	109	188	169	0.95869173	1	325	202.703866					5E+07	5E+07	0.18187	ID=promoter_09250;
11	5E+07	11_49636069	664	214	546	429	439	488	0.716476	1	177	188	171	93	183	132	0.08830301	1	310.25	208.179696					5E+07	5E+07	0.51468	ID=promoter_09251;
11	5E+07	11_49841333	17	0	4	15	9	10	0.993074	1	2	34	27	0	18	14	0.88543974	1	12.375	13.0596161								
11	5E+07	11_49991108	15	9	6	7	12	7	0.325249	1	11	3	10	22	7	16	0.11989665	1	10.375	5.90248132								
11	5E+07	11_50160882	67	78	27	9	73	18	4.97E-10	#######	101	31	32	6	66	19	1.25E-05	0.0015294	43.875	34.2363111					5E+07	5E+07	0.12743	ID=promoter_09259;
11	5E+07	11_50270988	5	2	9	22	4	16	0.065514	1	12	12	10	1	12	6	0.45526626	1	9.125	6.7281392								
11	5E+07	11_50725017	19	27	1	24	23	13	0.098913	1	30	27	17	9	29	13	0.08411918	1	19.25	10.0107086								
11	5E+07	11_50794099	714	435	265	251	575	258	2.86E-39	#######	204	82	78	48	143	63	3.14E-06	0.0004589	259.63	223.521771								

11	5E+07	11_50849951	0	11	12	22	6	17	0.158911	1	0	0	22	0	0	11	0.00265095	0.136745	8.375	9.79704766								
11	5E+07	11_51224173	3	14	9	1	9	5	0.390586	1	21	24	29	19	23	24	0.92099721	1	15	10.0427657								
11	5E+07	11_51257739	196	106	147	107	151	127	0.021254	0.74035	49	58	91	26	54	59	0.68287454	1	97.5	55.2681774								
11	5E+07	11_51558639	29	7	16	11	18	14	0.509661	1	51	8	31	63	30	47	0.07584163	1	27	20.8052191								
11	5E+07	11_51598431	8	48	18	31	28	25	0.609751	1	76	32	26	4	54	15	6.72E-05	0.0065007	30.375	23.1882821								
11	5E+07	11_51606719	24	12	18	24	18	21	0.99986	1	63	15	28	32	39	30	0.74371451	1	27	15.9910689								
11	5E+07	11_51680741	235	60	217	218	148	218	0.10063	1	110	38	34	2	74	18	3.03E-07	5.73E-05	114.25	95.4070228								
11	5E+07	11_53463340	7	39	1	3	23	2	2.40E-05	0.00257	27	13	37	5	20	21	0.92353071	1	16.5	15.5563492								
11	5E+07	11_53961054	121	184	95	47	153	71	1.89E-10	#######	110	133	124	102	122	113	0.99997735	1	114.5	38.5783063								
11	5E+07	11_54629900	7	43	16	29	25	23	0.701069	1	38	24	61	58	31	60	0.00305092	0.1542015	34.5	19.1907716								
11	5E+07	11_54925860	10	40	24	30	25	27	0.956696	1	80	57	35	20	69	28	7.52E-04	0.0497787	37	22.3542901								
11	6E+07	11_56201796	23	9	10	13	16	12	0.497978	1	41	38	37	0	40	19	0.05034717	1	21.375	15.6655901								
11	6E+07	11_60124972	0	4	5	0	2	3	0.964182	1	18	23	0	0	21	0	5.92E-05	0.0058659	6.25	9.11435289								
11	6E+07	11_60792690	2	18	0	0	10	0	0.002838	0.14869	0	58	14	0	29	7	0.00267552	0.137714	11.5	20.1068574								
11	6E+07	11_61030213	257	94	179	187	176	183	0.520801	1	120	76	74	28	98	51	0.0031879	0.1603338	126.88	75.1692139					6.1E+07	6.1E+07	0.43323	ID=promoter_09293;
11	6E+07	11_61362334	829	346	565	588	588	577	0.009589	0.39354	132	82	118	43	107	81	0.38688513	1	337.88	292.421143								
11	6E+07	11_61666856	9	29	16	12	19	14	0.411928	1	19	15	35	9	17	22	0.58360552	1	18	9.42640668								
11	6E+07	11_61769377	222	104	200	176	163	188	0.987706	1	137	152	226	117	145	172	0.09047669	1	166.75	46.7050013								
11	6E+07	11_61859825	40	29	20	11	35	16	0.005267	0.24489	73	27	38	5	50	22	0.01176819	0.4476014	30.375	21.0708838								
11	6E+07	11_62691848	42	62	17	21	52	19	2.52E-05	0.00268	61	33	36	73	47	55	0.50123132	1	43.125	20.3360166								
11	6E+07	11_62752934	19	62	28	20	41	24	0.022233	0.76438	52	61	32	19	57	26	0.0092631	0.3696513	36.625	18.77641								
11	6E+07	11_62944927	0	22	7	6	11	7	0.438207	1	2	31	19	24	17	22	0.58360552	1	13.875	11.5317884								
11	6E+07	11_62954815	80	94	56	39	87	48	9.87E-05	0.00879	144	58	31	30	101	31	1.09E-07	2.25E-05	66.5	38.6855455								
11	6E+07	11_62956352	8	28	1	26	18	14	0.509661	1	0	16	4	0	8	2	0.20550608	1	10.375	11.5627172								
11	6E+07	11_62966371	27	51	5	7	39	6	3.17E-07	#######	0	15	22	4	8	13	0.4490005	1	16.375	16.8348066								
11	6E+07	11_63440040	24	34	24	22	29	23	0.369822	1	6	24	4	16	15	10	0.7195765	1	19.25	10.0818082								
11	6E+07	11_63523605	0	66	30	5	33	18	0.027763	0.91318	19	52	23	1	36	12	0.00589803	0.2600594	24.5	24.1246762								
11	6E+07	11_63766302	144	630	454	393	387	424	0.624409	1	195	501	386	107	348	247	0.00561488	0.2525862	351.25	185.418253								
11	6E+07	11_63802636	39	30	8	17	35	13	9.22E-04	0.05911	19	0	27	2	10	15	0.49251655	1	17.75	13.8744369								
11	6E+07	11_63820648	137	146	80	61	142	71	1.16E-08	#######	205	196	54	196	201	125	0.001981	0.1087897	134.38	62.7077974					6.4E+07	6.4E+07	0.11128	ID=promoter_09301;
11	6E+07	11_63904152	0	1	9	6	1	8	0.109969	1	18	10	0	12	14	6	0.26891969	1	7	6.4807407								
11	6E+07	11_63915684	0	13	1	0	7	1	0.062633	1	80	13	7	17	47	12	1.10E-04	0.0099406	16.375	26.543428								
11	6E+07	11_63917329	4	6	9	22	5	16	0.124289	1	23	25	18	2	24	10	0.09254106	1	13.625	9.36463713								
11	6E+07	11_63957906	81	40	55	28	61	42	0.026947	0.89396	39	74	109	29	57	69	0.32705107	1	56.875	28.8118899								
11	6E+07	11_64272793	4	0	10	19	2	15	0.01804	0.65084	6	0	12	26	3	19	0.0015822	0.0898971	9.625	9.1953016								
11	6E+07	11_64351684	442	149	324	333	296	329	0.797894	1	152	139	133	36	146	85	0.0026886	0.1381379	213.5	136.372389	6E+07	6.4E+07	ID=ENSP00000366144;SOX21;NM_007084;	SOX21	6.4E+07	6.4E+07	0.80008	ID=promoter_09320;

11	6E+07	11_64357602	115	26	51	31	71	41	0.00111	0.06932	78	8	32	55	43	44	0.89932795	1	49.5	33.9284962								
11	6E+07	11_64422976	25	6	20	11	16	16	0.903021	1	33	34	17	17	34	17	0.10758511	1	20.375	9.88415037								
11	6E+07	11_64477667	34	30	36	1	32	19	0.056624	1	1	0	12	0	1	6	0.13767373	1	14.25	16.3597939								
11	6E+07	11_64634695	0	21	11	29	11	20	0.500229	1	26	2	40	26	14	33	0.01005158	0.3950437	19.375	13.9174864								
11	6E+07	11_64717311	13	16	4	14	15	9	0.269309	1	0	10	0	1	5	1	0.30486099	1	7.25	6.73477118								
11	7E+07	11_65498952	82	262	91	92	172	92	3.58E-09	#######	285	95	302	141	190	222	0.0637096	1	168.75	96.8323293								
11	7E+07	11_65639597	26	2	11	5	14	8	0.253058	1	2	24	19	15	13	17	0.64388579	1	13	9.56182887								
11	7E+07	11_65802629	103	41	55	82	72	69	0.485828	1	15	23	24	22	19	23	0.68897774	1	45.625	32.0710706								
11	7E+07	11_65819832	39	0	19	13	20	16	0.517469	1	19	11	7	25	15	16	0.92652531	1	16.625	11.927609								
11	7E+07	11_65822317	30	60	88	68	45	78	0.110382	1	15	6	18	27	11	23	0.07426989	1	39	29.3111388								
11	7E+07	11_65872020	1	27	17	13	14	15	0.970741	1	6	31	13	3	19	8	0.15782218	1	13.875	10.8422125								
11	7E+07	11_66087124	18	4	14	13	11	14	0.979671	1	7	6	9	1	7	5	0.9051169	1	9	5.65685425					6.6E+07	6.6E+07	0.50682	ID=promoter_09350;
11	7E+07	11_66087156	57	100	90	129	79	110	0.507689	1	80	54	77	79	67	78	0.39103705	1	83.25	23.9985119					6.6E+07	6.6E+07	0.50682	ID=promoter_09350;
11	7E+07	11_66575842	29	61	29	55	45	42	0.565697	1	92	25	42	58	59	50	0.89664128	1	48.875	22.4718077								
11	7E+07	11_66740809	310	147	197	213	229	205	0.018803	0.67198	104	58	15	25	81	20	9.15E-08	1.92E-05	133.63	102.867373								
11	7E+07	11_66743728	993	499	899	1075	746	987	0.045766	1	839	550	590	463	695	527	0.00230562	0.1234682	738.5	240.448391								
11	7E+07	11_66999353	20	39	60	78	30	69	0.007281	0.31495	58	27	16	10	43	13	9.59E-04	0.0604009	38.5	24.4832304								
11	7E+07	11_67226889	3833	1784	3995	3043	2809	3519	0.03387	1	730	696	659	337	713	498	7.28E-06	0.0009563	1884.6	1523.39348								
11	7E+07	11_67234332	8	56	7	20	32	14	0.005901	0.26867	21	11	28	1	16	15	0.99951315	1	19	17.3534682								
11	7E+07	11_67245380	19	1	5	7	10	6	0.41703	1	27	8	20	19	18	20	0.85572507	1	13.25	9.1456469								
11	7E+07	11_67248473	3	29	0	2	16	1	3.65E-04	0.02739	0	9	34	16	5	25	5.78E-04	0.0398304	11.625	13.4476498								
11	7E+07	11_67332854	12	1	5	6	7	6	0.851931	1	7	8	1	3	8	2	0.20550608	1	5.375	3.73927036					6.7E+07	6.7E+07	0.13472	ID=promoter_09363;
11	7E+07	11_67441544	3	19	1	10	11	6	0.3028	1	22	10	15	2	16	9	0.48000706	1	10.25	7.95972002								
11	7E+07	11_67450377	8	10	19	5	9	12	0.95859	1	29	19	30	4	24	17	0.69596948	1	15.5	10.3233439								
11	7E+07	11_67480005	16	11	12	0	14	6	0.098789	1	1	27	17	29	14	23	0.23132274	1	14.125	10.5889093	7E+07	6.8E+07	ID=ENSP00000365875;MBNL2;;	MBNL2				
11	7E+07	11_67484463	29	13	4	18	21	11	0.084122	1	175	21	223	59	98	141	0.00342875	0.1710576	67.75	83.5852858	7E+07	6.8E+07	ID=ENSP00000365875;MBNL2;;	MBNL2				
11	7E+07	11_67516507	75	60	133	58	68	96	0.503867	1	1	21	37	5	11	21	0.13989775	1	48.75	43.2624879	7E+07	6.8E+07	ID=ENSP00000365875;MBNL2;;	MBNL2				
11	7E+07	11_67546642	9	15	3	2	12	3	0.030625	0.98374	27	9	28	0	18	14	0.88543974	1	11.625	10.9013433								
11	7E+07	11_67685289	43	10	29	44	27	37	0.827591	1	77	30	24	13	54	19	8.02E-04	0.0527633	33.75	21.3390989					6.8E+07	6.8E+07	0.31389	ID=promoter_09381;
11	7E+07	11_67688881	118	58	86	119	88	103	0.999892	1	113	28	50	55	71	53	0.48925215	1	78.375	35.4196777					6.8E+07	6.8E+07	0.16844	ID=promoter_09382;
11	7E+07	11_67866905	14	39	12	9	27	11	0.00829	0.35136	1	2	22	44	2	33	3.22E-07	6.07E-05	17.875	16.0840427								
11	7E+07	11_68225280	4	21	16	8	13	12	0.835334	1	46	1	22	24	24	23	0.99354957	1	17.75	14.3103359								
11	7E+07	11_68227480	38	2	21	20	20	21	0.939065	1	4	12	34	6	8	20	0.04735057	1	17.125	13.6008141								

11	7E+07	11_68240265	55	23	65	24	39	45	0.997186	1	25	12	43	24	19	34	0.06506114	1	33.875	18.3959429								
11	7E+07	11_68377898	16	18	7	16	17	12	0.395844	1	8	8	9	1	8	5	0.78486764	1	10.375	5.78020019								
11	7E+07	11_68440758	8	20	25	8	14	17	0.995406	1	21	19	8	19	20	14	0.72101732	1	16	6.88684045								
11	7E+07	11_68499888	1	72	9	9	37	9	1.73E-05	0.00192	9	6	45	0	8	23	0.01462253	0.532614	18.875	25.7428908								
11	7E+07	11_68511123	13	50	19	20	32	20	0.080425	1	31	1	2	35	16	19	0.76520201	1	21.375	16.7582602								
11	7E+07	11_68537185	183	117	183	168	150	176	0.999996	1	78	77	76	1	78	39	0.00600794	0.2640292	110.38	64.6085742								
11	7E+07	11_68555132	1	21	10	10	11	10	0.842816	1	0	6	36	1	3	19	0.0015822	0.0898971	10.625	12.3743687								
11	7E+07	11_68564471	24	1	14	36	13	25	0.34413	1	38	30	73	47	34	60	0.0095223	0.3791471	32.875	21.7284376								
11	7E+07	11_68725784	7	4	7	10	6	9	0.896349	1	0	6	0	6	3	3	0.99570314	1	5	3.50509833								
11	7E+07	11_68761450	0	1	10	10	1	10	0.048828	1	19	1	2	23	10	13	0.72167512	1	8.25	8.87613172								
11	7E+07	11_68779627	13	36	2	32	25	17	0.214068	1	23	24	5	19	24	12	0.20726737	1	19.25	12.0682582								
11	7E+07	11_68781295	2	6	6	4	4	5	0.995512	1	0	15	11	0	8	6	0.92562801	1	5.5	5.29150262								
11	7E+07	11_68793248	14	4	23	13	9	18	0.416978	1	2	10	39	4	6	22	0.00549069	0.2478586	13.625	12.3396863								
11	7E+07	11_68816876	174	126	108	84	150	96	1.55E-05	0.00174	75	27	40	17	51	29	0.1051192	1	81.375	53.6388385					6.9E+07	6.9E+07	0.13898	ID=promoter_09449;
11	7E+07	11_68843264	5	0	5	3	3	4	0.986002	1	6	2	0	22	4	11	0.14780855	1	5.375	7.08998287								
11	7E+07	11_68865383	200	67	178	202	134	190	0.242312	1	66	86	144	8	76	76	0.8966513	1	118.88	72.1891118								
11	7E+07	11_68871877	136	30	94	70	83	82	0.54382	1	19	0	15	1	10	8	0.95278883	1	45.625	49.3759268								
11	7E+07	11_68894067	24	83	19	23	54	21	3.76E-05	0.0038	1233	1016	609	417	1125	513	6.96E-41	1.07E-37	428	484.059914								
11	7E+07	11_68917594	261	247	293	193	254	243	0.075174	1	20	0	0	0	10	0	0.00970398	0.3824351	126.75	133.150559								
11	7E+07	11_68964276	76	37	77	106	57	92	0.16392	1	10	70	35	1	40	18	0.03384608	1	51.5	36.4848305								
11	7E+07	11_68968084	116	44	71	58	80	65	0.086764	1	46	50	18	33	48	26	0.08258093	1	54.5	29.432732								
11	7E+07	11_68994047	0	9	16	8	5	12	0.389128	1	78	1	14	18	40	16	0.01383566	0.5095422	18	25.1111813								
11	7E+07	11_69189024	23	2	11	9	13	10	0.600182	1	2	5	2	3	4	3	0.96209564	1	7.125	7.27888335					6.9E+07	6.9E+07	0.31445	ID=promoter_09474;
11	7E+07	11_69244938	183	84	155	116	134	136	0.49265	1	3	20	83	8	12	46	1.22E-05	0.0015004	81.5	67.8759372								
11	7E+07	11_69276580	75	98	36	42	87	39	1.44E-06	0.00021	438	244	71	133	341	102	5.72E-25	5.01E-22	142.13	136.697202								
11	7E+07	11_69477228	2	21	29	11	12	20	0.626521	1	35	23	45	2	29	24	0.91882689	1	21	15.4087174								
11	7E+07	11_69877907	1	21	8	3	11	6	0.3028	1	3	4	14	16	4	15	0.026797	0.8546242	8.75	7.36303509								
11	7E+07	11_70134525	15	24	14	37	20	26	0.941925	1	40	51	32	25	46	29	0.26217857	1	29.75	12.7363372								
11	7E+07	11_70143228	21	12	22	11	17	17	0.897282	1	0	0	0	0	0	0	1	1	8.25	9.60282696								
11	7E+07	11_70165762	25	79	63	36	52	50	0.603451	1	111	53	68	62	82	65	0.64058412	1	62.125	26.3299802					7E+07	7E+07	0.19879	ID=promoter_09529;
11	7E+07	11_70251004	9	47	16	58	28	37	0.892844	1	63	36	25	23	50	24	0.02621347	0.8452651	34.625	19.7768804	7E+07	7E+07	ID=ENSP0000343871-D2;TMTC4;NM_032813;	TMTC4				
11	7E+07	11_70254569	35	35	38	13	35	26	0.205248	1	22	50	19	21	36	20	0.17860656	1	29.125	12.3223085	7E+07	7E+07	ID=ENSP0000343871-D2;TMTC4;NM_032813;	TMTC4				

11	7E+07	11_70264699	1	16	10	20	9	15	0.704209	1	46	25	17	21	36	19	0.13186357	1	19.5	13.0164731	7E+07	7E+07	ID=ENSP00000343871-D2;TMTC4;NM_032813;	TMTC4	7E+07	7E+07	0.26179	ID=promoter_09538;
11	7E+07	11_70276029	40	35	60	44	38	52	0.77004	1	0	11	13	2	6	8	0.79537884	1	25.625	22.0450027	7E+07	7E+07	ID=ENSP00000343871-D2;TMTC4;NM_032813;	TMTC4				
11	7E+07	11_70301206	63	108	52	77	86	65	0.026435	0.88004	45	36	52	39	41	46	0.67308043	1	59	23.7967585	7E+07	7E+07	ID=ENSP00000343871-D2;TMTC4;NM_032813;	TMTC4	7E+07	7E+07	0.26518	ID=promoter_09541;
11	7E+07	11_70311525	2	17	0	0	10	0	0.002838	0.14869	0	17	4	0	9	2	0.1390433	1	5	7.54036755					7E+07	7E+07	0.13073	ID=promoter_09544;
11	7E+07	11_70530105	6	4	6	3	5	5	0.968625	1	19	17	29	5	18	17	0.9984894	1	11.125	9.43303466								
11	7E+07	11_70589032	24	23	16	19	24	18	0.351411	1	44	99	13	31	72	22	1.02E-05	0.0012946	33.625	28.1421773					7.1E+07	7.1E+07	0.11728	ID=promoter_09553;
11	7E+07	11_70591271	54	53	20	33	54	27	9.59E-04	0.06099	66	14	19	4	40	12	0.00146628	0.0860502	32.875	22.3506951					7.1E+07	7.1E+07	0.19637	ID=promoter_09554;
11	7E+07	11_70620488	15	38	6	19	27	13	0.024366	0.82541	12	18	24	10	15	17	0.85096642	1	17.75	9.92471662								
11	7E+07	11_70862032	25	61	13	11	43	12	1.06E-05	0.00126	45	30	21	2	38	12	0.00296809	0.1507727	26	19.2650683								
11	7E+07	11_70875086	1	10	7	10	6	9	0.896349	1	1	12	25	27	7	26	0.00198609	0.1088597	11.625	9.76784082								
11	7E+07	11_70915878	988	442	2132	1678	715	1905	4.51E-81	#######	251	328	640	292	290	466	9.04E-13	3.80E-10	843.88	707.214342					7.1E+07	7.1E+07	0.47564	ID=promoter_09569;
11	7E+07	11_70961485	4	31	31	11	18	21	0.99986	1	26	65	39	20	46	30	0.32156472	1	28.375	18.6695895								
11	7E+07	11_70979063	11	57	11	11	34	11	3.70E-04	0.02765	0	6	13	21	3	17	0.00428697	0.2008879	16.25	17.5071414								
11	7E+07	11_71016254	47	25	51	122	36	87	9.61E-04	0.06109	102	98	122	121	100	122	0.12429748	1	86	39.0677068								
11	7E+07	11_71180657	31	48	31	13	40	22	0.013995	0.53231	64	19	27	21	42	24	0.16154149	1	31.75	16.7225254								
11	7E+07	11_71196154	26	9	2	8	18	5	0.008132	0.34618	8	2	6	0	5	3	0.8350419	1	7.625	8.14050718								
11	7E+07	11_71354317	0	19	3	12	10	8	0.719353	1	12	9	0	2	11	1	0.02232329	0.7455867	7.125	6.93721846								
11	7E+07	11_71396900	20	2	39	20	11	30	0.047869	1	4	12	3	51	8	27	0.00271213	0.139047	18.875	17.907999								
11	7E+07	11_71403845	38	56	18	33	47	26	0.007051	0.30636	67	52	42	59	60	51	0.90137816	1	45.625	15.9368172								
11	7E+07	11_71528506	13	16	7	16	15	12	0.610117	1	32	25	13	1	29	7	0.00267552	0.137714	15.375	9.69443876								
11	7E+07	11_71832005	2	37	33	1	20	17	0.618382	1	29	48	25	1	39	13	0.00384532	0.1844291	22	18.3692289								
11	7E+07	11_71868859	8	8	22	27	8	25	0.042844	1	0	0	0	0	0	0	1	1	8.125	10.776131					7.2E+07	7.2E+07	0.2165	ID=promoter_09602;
11	7E+07	11_71868969	19	0	19	5	10	12	0.998584	1	0	0	0	0	0	0	1	1	5.375	8.5846624					7.2E+07	7.2E+07	0.2165	ID=promoter_09602;
11	7E+07	11_71872118	248	73	249	249	161	249	0.023233	0.79532	47	16	68	6	32	37	0.65671368	1	119.5	109.360479					7.2E+07	7.2E+07	0.11873	ID=promoter_09603;
11	7E+07	11_71889794	350	241	665	316	296	491	1.37E-05	0.00157	213	226	168	129	220	149	0.01211396	0.4594066	288.5	168.231983								
11	7E+07	11_71960945	1559	1320	941	770	1440	856	1.11E-56	#######	1233	894	488	268	1064	378	7.56E-61	1.59E-57	934.13	430.952247					7.2E+07	7.2E+07	0.70741	ID=promoter_09612;
11	7E+07	11_72027178	35	140	60	45	88	53	4.88E-04	0.03443	88	57	49	69	73	59	0.73533013	1	67.875	33.2713412								
11	7E+07	11_72033805	176	123	166	134	150	150	0.384298	1	8	62	65	2	35	34	0.98131319	1	92	67.6566964								

11	7E+07	11_72041524	5	33	1	5	19	3	7.41E-04	0.04898	0	9	15	3	5	9	0.48000906	1	8.875	10.8553805								
11	7E+07	11_72127400	10	13	14	3	12	9	0.592799	1	19	30	15	14	25	15	0.40601431	1	14.75	7.70435869								
11	7E+07	11_72218821	923	399	432	355	661	394	3.05E-26	#######	167	104	75	70	136	73	6.96E-04	0.0467656	315.63	286.868779								
11	7E+07	11_72339819	25	11	0	0	18	0	2.60E-05	0.00275	10	0	9	0	5	5	0.99284883	1	6.875	8.85498246								
11	7E+07	11_72551044	47	27	41	24	37	33	0.517593	1	20	24	7	15	22	11	0.23631249	1	25.625	13.0486726								
11	7E+07	11_72569133	2	47	9	13	25	11	0.018765	0.67168	44	17	28	5	31	17	0.21261815	1	20.625	17.2952306					7.3E+07	7.3E+07	0.15042	ID=promoter_09625;
11	7E+07	11_72689825	18	18	7	5	18	6	0.017303	0.62786	28	11	36	10	20	23	0.77926641	1	16.625	10.7695006								
11	7E+07	11_73056803	341	326	377	323	334	350	0.337174	1	527	376	330	451	452	391	0.60504429	1	381.38	72.5513365								
11	7E+07	11_73171078	248	149	230	299	199	265	0.399982	1	132	77	23	2	105	13	7.59E-15	3.84E-12	145	108.029097								
11	7E+07	11_73207599	0	52	18	5	26	12	0.021538	0.74463	65	9	22	45	37	34	0.99931372	1	27	24.0119018								
11	7E+07	11_73267064	7	0	0	1	4	1	0.312866	1	23	0	2	22	12	12	0.98292303	1	6.875	9.92021745								
11	7E+07	11_73396601	9	27	13	16	18	15	0.617094	1	60	44	33	16	52	25	0.02300235	0.7590344	27.25	17.6291155								
11	7E+07	11_73912280	9	0	10	7	5	9	0.74134	1	3	12	5	0	8	3	0.3843038	1	5.75	4.52769257								
11	7E+07	11_74025874	29	45	4	25	37	15	0.001345	0.08048	44	5	30	28	25	29	0.71264652	1	26.25	15.3040611								
11	7E+07	11_74084443	579	336	415	321	458	368	3.82E-07	#######	108	62	85	12	85	49	0.02804214	0.888382	239.75	202.286748								
11	7E+07	11_74098617	4	53	4	43	29	24	0.446278	1	17	21	60	3	19	32	0.1120603	1	25.625	23.2252172								
11	7E+07	11_74182180	25	22	29	40	24	35	0.712535	1	1	0	17	13	1	15	0.00126073	0.075413	18.375	13.6689168								
11	7E+07	11_74195283	30	10	11	3	20	7	0.013885	0.52965	18	0	2	25	9	14	0.47220951	1	12.375	11.0703917								
11	7E+07	11_74213250	12	1	9	8	7	9	0.983538	1	1	13	5	16	7	11	0.54353855	1	8.125	5.51459104								
11	7E+07	11_74241973	25	45	13	11	35	12	4.73E-04	0.03365	66	31	29	5	49	17	0.0013565	0.0806014	28.125	19.981688								
11	7E+07	11_74285747	156	477	84	60	317	72	1.65E-43	#######	272	159	258	126	216	192	0.91412507	1	199	134.916906								
11	7E+07	11_74322406	4	0	4	0	2	2	0.98733	1	54	12	0	25	33	13	0.02654774	0.8508557	12.375	18.867489								
11	7E+07	11_74358125	66	49	44	38	58	41	0.043015	1	76	106	66	0	91	33	1.04E-05	0.0013129	55.625	31.0572628								
11	7E+07	11_74428597	11	51	19	20	31	20	0.106722	1	70	52	66	21	61	44	0.44530595	1	38.75	23.5174707								
11	7E+07	11_74435072	28	60	23	35	44	29	0.050532	1	26	43	72	23	35	48	0.20763434	1	38.75	18.3750607								
11	7E+07	11_74460921	24	7	13	30	16	22	0.889379	1	52	11	31	9	32	20	0.37947721	1	22.125	15.3291273								
11	7E+07	11_74463955	10	25	6	0	18	3	0.001281	0.07728	0	0	0	15	0	8	0.01336935	0.4945372	7	9.21179368								
11	7E+07	11_74654161	192	91	67	71	142	69	4.46E-09	#######	2	21	40	1	12	21	0.20312388	1	60.625	62.4704216					7.5E+07	7.5E+07	0.23043	ID=promoter_09667;
11	7E+07	11_74726103	0	71	9	11	36	10	6.61E-05	0.0062	26	37	31	2	32	17	0.17107071	1	23.375	23.5489612								
11	7E+07	11_74855198	0	0	7	9	0	8	0.033035	1	1	2	0	28	2	14	0.00692302	0.294895	5.875	9.58328157								
11	7E+07	11_74909544	0	35	4	10	18	7	0.033499	1	0	15	25	0	8	13	0.4490005	1	11.125	13.0978461	7E+07	7.5E+07	ID=ENSP0000245323;EFNB2;NM_004093;	EFNB2				
11	7E+07	11_74953279	9	21	6	9	15	8	0.181584	1	10	7	21	42	9	32	6.35E-04	0.0429702	15.625	12.1882555	7E+07	7.5E+07	ID=ENSP0000353844;ARGLU1;NM_018011;	ARGLU1				
11	7E+07	11_74982468	0	6	4	0	3	2	0.821455	1	19	7	32	0	13	16	0.74830213	1	8.5	11.389218								
11	8E+07	11_75059706	46	46	36	65	46	51	0.962331	1	55	55	14	4	55	9	3.53E-07	6.60E-05	40.125	21.1622136								

11	8E+07	11_75351966	39	57	30	18	48	24	0.002076	0.11546	0	36	53	21	18	37	0.01748595	0.6144624	31.75	18.8053944								
11	8E+07	11_75352086	1651	687	2705	1928	1169	2317	3.11E-48	#######	394	236	261	144	315	203	2.61E-04	0.0204535	1000.8	965.087968								
11	8E+07	11_75377253	53	38	33	28	46	31	0.054078	1	44	25	25	1	35	13	0.01427485	0.52128	30.875	15.5235995								
11	8E+07	11_75408289	1	0	4	7	1	6	0.241978	1	22	27	17	18	25	18	0.71458637	1	12	10.2817453								
11	8E+07	11_75416201	5	12	9	5	9	7	0.713906	1	22	17	37	47	20	42	0.00833392	0.3402748	19.25	15.4063994								
11	8E+07	11_75597255	29	3	36	46	16	41	0.024808	0.83898	44	25	18	37	35	28	0.8442835	1	29.75	14.3203152								
11	8E+07	11_75623928	20	24	28	20	22	24	0.97026	1	25	15	10	4	20	7	0.06990458	1	18.25	8.11964109								
11	8E+07	11_75664796	23	110	65	81	67	73	0.909306	1	95	164	163	55	130	109	0.74136495	1	94.5	49.9828542								
11	8E+07	11_75713669	4	7	17	14	6	16	0.210908	1	1	15	3	3	8	3	0.3843038	1	8	6.34710283					7.6E+07	7.6E+07	0.13603	ID=promoter_09733;
11	8E+07	11_75736966	0	7	2	7	4	5	0.995512	1	16	0	10	22	8	16	0.19094317	1	8	7.83763813								
11	8E+07	11_75756664	2	79	6	3	41	5	3.37E-08	#######	13	0	12	3	7	8	0.92121674	1	14.75	26.3804581								
11	8E+07	11_75790595	5	0	8	0	3	4	0.986002	1	33	11	13	0	22	7	0.03554381	1	8.75	11.055057								
11	8E+07	11_75843893	16	12	8	14	14	11	0.605836	1	15	0	28	28	8	28	0.00175088	0.0978457	15.125	9.43303466								
11	8E+07	11_76016937	423	639	43	49	531	46	########	#######	42	41	14	19	42	17	0.01229307	0.4645951	158.75	237.212834								
11	8E+07	11_76087939	2	53	5	7	28	6	1.19E-04	0.01043	24	39	62	26	32	44	0.23264674	1	27.25	22.5626113								
11	8E+07	11_76109375	317	269	221	153	293	187	3.41E-10	#######	60	17	24	1	39	13	0.00384532	0.1844291	132.75	124.54804					7.6E+07	7.6E+07	0.10371	ID=promoter_09753;
11	8E+07	11_76140365	18	81	15	19	50	17	1.61E-05	0.00181	49	10	41	3	30	22	0.70449098	1	29.5	25.9119388								
11	8E+07	11_76149807	11	63	32	9	37	21	0.025035	0.84326	15	5	3	49	10	26	0.01509923	0.5480081	23.375	22.3091109								
11	8E+07	11_76204871	48	63	50	43	56	47	0.235754	1	33	121	58	4	77	31	2.91E-04	0.0225937	52.5	33.1274595					7.6E+07	7.6E+07	0.13395	ID=promoter_09759;
11	8E+07	11_76212702	12	17	18	42	15	30	0.232732	1	0	3	8	0	2	4	0.6610374	1	12.5	13.8564065								
11	8E+07	11_76377687	42	27	14	10	35	12	4.73E-04	0.03365	84	95	28	47	90	38	1.67E-04	0.014126	43.375	31.1949515	8E+07	7.7E+07	ID=ENSP00000380438;MYO16;;	MYO16	7.6E+07	7.6E+07	0.12393	ID=promoter_09770;
11	8E+07	11_76408879	19	66	313	326	43	320	8.74E-38	#######	2	12	111	123	7	117	8.52E-24	7.07E-21	121.5	130.088981	8E+07	7.7E+07	ID=ENSP00000380438;MYO16;;	MYO16	7.6E+07	7.6E+07	0.1644	ID=promoter_09773;
11	8E+07	11_76414115	106	14	77	43	60	60	0.68213	1	0	8	8	0	4	4	0.99427496	1	32	39.9034549	8E+07	7.7E+07	ID=ENSP00000380438;MYO16;;	MYO16	7.6E+07	7.6E+07	0.15241	ID=promoter_09775;
11	8E+07	11_76472632	28	19	24	1	24	13	0.071032	1	0	12	0	0	6	0	0.06196836	1	10.5	11.8562822	8E+07	7.7E+07	ID=ENSP00000380438;MYO16;;	MYO16				
11	8E+07	11_76490153	15	16	1	7	16	4	0.009581	0.39335	14	7	20	7	11	14	0.73202476	1	10.875	6.31183695	8E+07	7.7E+07	ID=ENSP00000380438;MYO16;;	MYO16	7.6E+07	7.6E+07	0.3452	ID=promoter_09788;
11	8E+07	11_76557463	13	32	10	9	23	10	0.024151	0.81985	0	1	13	20	1	17	4.35E-04	0.0314643	12.25	10.3060315								
11	8E+07	11_76628975	55	31	48	42	43	45	0.866585	1	14	18	24	18	16	21	0.57439123	1	31.25	15.4063994								
11	8E+07	11_76652139	3	15	3	9	9	6	0.554309	1	5	27	28	1	16	15	0.99951315	1	11.375	10.8751026								
11	8E+07	11_76753456	6	74	9	25	40	17	0.001238	0.07553	69	33	16	29	51	23	0.01361705	0.5029183	32.625	25.7733944					7.7E+07	7.7E+07	0.23591	ID=promoter_09825;
11	8E+07	11_76779280	57	25	72	60	41	66	0.278666	1	0	4	19	30	2	25	2.25E-05	0.0025619	33.375	26.7898568								
11	8E+07	11_76780952	19	0	14	0	10	7	0.570701	1	1	3	22	4	2	13	0.01148244	0.4380166	7.875	9.0307016					7.7E+07	7.7E+07	0.28477	ID=promoter_09832;

11	8E+07	11_76802328	9	9	13	1	9	7	0.713906	1	12	4	0	6	8	3	0.3843038	1	6.75	4.83292281								
11	8E+07	11_76806075	441	226	274	291	334	283	2.82E-04	0.02189	9	58	52	5	34	29	0.94710596	1	169.5	161.088263								
11	8E+07	11_76809274	1	48	5	14	25	10	0.01059	0.42992	14	25	10	1	20	6	0.03829208	1	14.75	15.6182128								
11	8E+07	11_76898522	47	34	88	134	41	111	1.51E-05	0.0017	0	58	56	26	29	41	0.21540236	1	55.375	40.900358					7.7E+07	7.7E+07	0.32709	ID=promoter_09860;
11	8E+07	11_76908631	133	45	104	123	89	114	0.823814	1	48	23	24	15	36	20	0.17860656	1	64.375	48.0176679								
11	8E+07	11_76948862	0	21	1	0	11	1	0.006522	0.28949	14	7	0	0	11	0	0.00610443	0.2666968	5.375	8.0700062								
11	8E+07	11_76981255	72	14	34	45	43	40	0.571521	1	38	29	17	31	34	24	0.59108388	1	35	18.1107703					7.7E+07	7.7E+07	0.19749	ID=promoter_09877;
11	8E+07	11_76983210	5	59	17	32	32	25	0.308825	1	37	20	37	29	29	33	0.72226159	1	29.5	16.1952374					7.7E+07	7.7E+07	0.331	ID=promoter_09878;
11	8E+07	11_77002811	13	32	67	72	23	70	2.07E-04	0.01673	30	13	33	19	22	26	0.70257106	1	34.875	22.8500547								
11	8E+07	11_77045863	216	68	234	235	142	235	0.00501	0.23539	0	20	40	30	10	35	3.58E-04	0.0267905	105.38	103.73722								
11	8E+07	11_77129268	86	98	72	100	92	86	0.316699	1	50	112	73	4	81	39	0.00286752	0.1464878	74.375	34.4754053								
11	8E+07	11_77154716	12	0	25	25	6	25	0.011836	0.46238	18	0	21	0	9	11	0.82669107	1	12.625	11.2368971					7.7E+07	7.7E+07	0.21548	ID=promoter_09921;
11	8E+07	11_77169668	21	83	30	45	52	38	0.081745	1	37	4	39	22	21	31	0.25173633	1	35.125	23.2221415	8E+07	7.7E+07	ID=ENSP00000354105;COL9A1;;	COL9A1	7.7E+07	7.7E+07	0.20348	ID=promoter_09926;
11	8E+07	11_77196427	13	13	14	3	13	9	0.46931	1	17	5	12	43	11	28	0.01248827	0.4699819	15	12.2474487	8E+07	7.7E+07	ID=ENSP00000354105;COL9A1;;	COL9A1	7.7E+07	7.7E+07	0.15514	ID=promoter_09935;
11	8E+07	11_77198847	135	148	115	171	142	143	0.435969	1	61	58	39	121	60	80	0.10133053	1	106	47.7403693	8E+07	7.7E+07	ID=ENSP00000354105;COL9A1;;	COL9A1	7.7E+07	7.7E+07	0.27342	ID=promoter_09937;
11	8E+07	11_77223760	109	83	63	78	96	71	0.012057	0.47038	247	167	130	46	207	88	2.68E-09	7.27E-07	115.38	65.7330913					7.7E+07	7.7E+07	0.11403	ID=promoter_09947;
11	8E+07	11_77226452	236	115	186	115	176	151	0.018193	0.65566	42	7	16	5	25	11	0.10848128	1	90.25	87.5667093					7.7E+07	7.7E+07	0.21238	ID=promoter_09949;
11	8E+07	11_77244708	68	44	105	89	56	97	0.065085	1	18	19	27	34	19	31	0.144726	1	50.5	33.1102056					7.7E+07	7.7E+07	0.12676	ID=promoter_09955;
11	8E+07	11_77297277	72	34	66	77	53	72	0.719101	1	19	28	28	24	24	26	0.85918702	1	43.5	23.8806557								
11	8E+07	11_77306850	108	60	130	177	84	154	0.004041	0.19765	65	42	29	4	54	17	2.48E-04	0.0197712	76.875	57.3969574								
11	8E+07	11_77309904	16	20	32	21	18	27	0.720164	1	10	9	2	3	10	3	0.19568363	1	14.125	10.1339246								
11	8E+07	11_77313223	0	15	9	10	8	10	0.991043	1	0	17	11	21	9	16	0.2823118	1	10.375	7.52021086					7.7E+07	7.7E+07	0.16239	ID=promoter_09976;
11	8E+07	11_77347756	18	28	13	38	23	26	0.991715	1	44	44	41	18	44	30	0.42803716	1	30.5	12.8507921					7.7E+07	7.7E+07	0.2776	ID=promoter_09984;
11	8E+07	11_77354848	169	292	212	194	231	203	0.010723	0.43446	235	167	163	110	201	137	0.02056872	0.6970948	192.75	54.729856					7.7E+07	7.7E+07	0.32731	ID=promoter_09987;
11	8E+07	11_77388105	16	26	26	37	21	32	0.646214	1	18	1	4	3	10	4	0.34296513	1	16.375	12.9938172								
11	8E+07	11_77400985	1	19	18	2	10	10	0.938234	1	17	7	13	32	12	23	0.11381058	1	13.625	10.2530483					7.7E+07	7.7E+07	0.21999	ID=promoter_09997;
11	8E+07	11_77437843	27	0	13	11	14	12	0.725787	1	20	10	26	1	15	14	0.99983448	1	13.5	10.2678416								
11	8E+07	11_77493345	5	10	12	15	8	14	0.661714	1	36	13	45	23	25	34	0.34266885	1	19.875	13.902081					7.7E+07	7.7E+07	0.63119	ID=promoter_10027;
11	8E+07	11_77499287	42	58	49	62	50	56	0.972202	1	43	12	11	1	28	6	0.00180324	0.1003399	34.75	23.3834496					7.7E+07	7.8E+07	0.19598	ID=promoter_10029;

11	8E+07	11_77513666	11	11	5	4	11	5	0.189421	1	9	4	6	11	7	9	0.80856893	1	7.625	3.20434972					7.8E+07	7.8E+07	0.71989	ID=promoter_10034;
11	8E+07	11_77555992	66	84	24	55	75	40	1.97E-04	0.01603	119	81	125	38	100	82	0.71208091	1	74	35.8488892					7.8E+07	7.8E+07	0.38715	ID=promoter_10043;
11	8E+07	11_77558254	25	11	13	6	18	10	0.152558	1	76	46	8	0	61	4	1.12E-10	3.72E-08	23.125	25.715129								
11	8E+07	11_77593650	59	8	7	5	34	6	4.87E-06	0.00063	31	14	11	26	23	19	0.93310412	1	20.125	18.2556254								
11	8E+07	11_77605467	548	192	458	371	370	415	0.822105	1	271	130	190	120	201	155	0.22590244	1	285	158.391017								
11	8E+07	11_77702673	29	6	43	46	18	45	0.021053	0.7339	22	0	4	5	11	5	0.40227598	1	19.375	18.3765184								
11	8E+07	11_77710294	10	92	404	342	51	373	1.50E-43	#######	1	32	190	49	17	120	2.64E-19	1.76E-16	140	156.511068					7.8E+07	7.8E+07	0.16169	ID=promoter_10085;
11	8E+07	11_77719142	46	34	12	6	40	9	3.70E-06	0.00049	29	7	11	4	18	8	0.20901227	1	18.625	15.6016254								
11	8E+07	11_77723813	0	13	5	3	7	4	0.503048	1	10	7	6	1	9	4	0.4571786	1	5.625	4.40575922								
11	8E+07	11_77900348	3	0	1	4	2	3	0.964182	1	15	7	1	18	11	10	0.99899888	1	6.125	6.81254096	8E+07	7.8E+07	ID=ENSP0000364893;ARHGEF7;NM_001113511;	ARHGEF7	7.8E+07	7.8E+07	0.63085	ID=promoter_10130;
11	8E+07	11_77919861	0	17	9	1	9	5	0.390586	1	1	1	9	2	1	6	0.13767373	1	5	6.07100839					7.8E+07	7.8E+07	0.99355	ID=promoter_10136;
11	8E+07	11_77977333	148	26	180	178	87	179	7.56E-05	0.00699	37	27	61	29	32	45	0.19260481	1	85.75	70.2032763								
11	8E+07	11_77979205	1	22	6	6	12	6	0.213448	1	23	31	36	5	27	21	0.83317938	1	16.25	13.3924286					7.8E+07	7.8E+07	0.47834	ID=promoter_10149;
11	8E+07	11_78068713	711	435	1793	1496	573	1645	9.92E-80	#######	301	207	437	298	254	368	2.43E-07	4.70E-05	709.75	601.293071								
11	8E+07	11_78093914	11	7	7	10	9	9	0.944235	1	14	0	10	1	7	6	0.99011599	1	7.5	4.86973159					7.8E+07	7.8E+07	0.13091	ID=promoter_10185;
11	8E+07	11_78094438	76	150	161	189	113	175	0.069394	1	180	74	118	77	127	98	0.39315165	1	128.13	48.2832047								
11	8E+07	11_78117927	43	93	60	54	68	57	0.1709	1	15	9	4	39	12	22	0.15316566	1	39.625	29.990177					7.8E+07	7.8E+07	0.42533	ID=promoter_10191;
11	8E+07	11_78131748	163	85	206	248	124	227	3.10E-04	0.02381	55	108	107	81	82	94	0.37122292	1	131.63	67.434492					7.8E+07	7.8E+07	0.61672	ID=promoter_10197;
11	8E+07	11_78137365	16	7	21	21	12	21	0.538277	1	0	1	25	4	1	15	0.00126073	0.075413	11.875	10.006248					7.8E+07	7.8E+07	0.66332	ID=promoter_10199;
11	8E+07	11_78146323	20	53	23	15	37	19	0.010763	0.4351	99	49	66	26	74	46	0.1006707	1	43.875	28.7771611					7.8E+07	7.8E+07	0.39141	ID=promoter_10202;
11	8E+07	11_78149108	12	14	3	15	13	9	0.46931	1	13	7	7	4	10	6	0.69729498	1	9.375	4.6885118					7.8E+07	7.8E+07	0.44208	ID=promoter_10203;
11	8E+07	11_78154086	1	26	6	2	14	4	0.02589	0.86655	9	23	9	14	16	12	0.85678722	1	11.25	9.19238816					7.8E+07	7.8E+07	0.50365	ID=promoter_10204;
11	8E+07	11_78156527	15	2	15	6	9	11	0.995818	1	15	13	6	26	14	16	0.84864027	1	12.25	7.51664819					7.8E+07	7.8E+07	0.45172	ID=promoter_10205;
11	8E+07	11_78157199	20	22	16	19	21	18	0.61832	1	3	12	12	6	8	9	0.92351511	1	13.75	6.77706215					7.8E+07	7.8E+07	0.45172	ID=promoter_10205;
11	8E+07	11_78163792	15	21	11	20	18	16	0.720517	1	16	18	13	23	17	18	0.92557631	1	17.125	4.12093956					7.8E+07	7.8E+07	0.91267	ID=promoter_10206;
11	8E+07	11_78168418	12	20	11	22	16	17	0.960459	1	4	17	16	3	11	10	0.99899888	1	13.125	6.97828264								
11	8E+07	11_78171041	35	17	51	29	26	40	0.558059	1	2	2	40	13	2	27	7.81E-06	0.0010203	23.625	17.999504								
11	8E+07	11_78184702	7	19	8	8	13	8	0.344217	1	0	0	4	25	0	15	3.07E-04	0.0235795	8.875	8.85498246								
11	8E+07	11_78193225	5	27	9	10	16	10	0.283594	1	32	5	9	1	19	5	0.02799778	0.8875672	12.25	11.119481					7.8E+07	7.8E+07	0.48442	ID=promoter_10215;

11	8E+07	11_78195094	49	33	83	46	41	65	0.316708	1	11	0	55	15	6	35	1.12E-05	0.0013967	36.5	27.2763634								
11	8E+07	11_78208565	12	1	10	25	7	18	0.195146	1	0	0	2	0	0	1	0.58312783	1	6.25	8.95624283								
11	8E+07	11_78218149	123	29	113	88	76	101	0.712841	1	64	16	76	4	40	40	0.94420228	1	64.125	44.2894296					7.8E+07	7.8E+07	0.22449	ID=promoter_10222;
11	8E+07	11_78230632	144	104	167	165	124	166	0.537422	1	22	0	31	30	11	31	0.00385975	0.1846363	82.875	69.7596639					7.8E+07	7.8E+07	0.58965	ID=promoter_10227;
11	8E+07	11_78270481	64	34	58	57	49	58	0.998901	1	21	75	24	3	48	14	3.06E-04	0.0235795	42	25.0827203					7.8E+07	7.8E+07	0.69179	ID=promoter_10239;
11	8E+07	11_78274377	4	18	5	5	11	5	0.189421	1	24	0	6	3	12	5	0.30420563	1	8.125	8.30554892					7.8E+07	7.8E+07	0.35731	ID=promoter_10241;
11	8E+07	11_78301204	15	0	17	78	8	48	1.18E-05	0.00138	0	8	7	7	4	7	0.58783259	1	16.5	25.5845936					7.8E+07	7.8E+07	0.6535	ID=promoter_10248;
11	8E+07	11_78361803	16	38	20	6	27	13	0.024366	0.82541	17	9	7	7	13	7	0.50249747	1	15	10.7171425					7.8E+07	7.8E+07	0.75558	ID=promoter_10266;
11	8E+07	11_78374493	39	55	40	45	47	43	0.498196	1	97	53	24	2	75	13	3.12E-09	8.42E-07	44.375	27.2969779								
11	8E+07	11_78380525	0	26	5	1	13	3	0.018372	0.65917	19	0	15	0	10	8	0.95278883	1	8.25	10.3060315					7.8E+07	7.8E+07	0.55705	ID=promoter_10275;
11	8E+07	11_78404045	29	101	34	30	65	32	1.84E-04	0.01511	40	0	20	0	20	10	0.26942782	1	31.75	31.6983551								
11	8E+07	11_78408974	358	142	204	224	250	214	0.003125	0.16098	152	150	140	33	151	87	0.00172636	0.09686	175.38	92.8607829					7.8E+07	7.8E+07	0.24843	ID=promoter_10283;
11	8E+07	11_78472352	30	3	13	27	17	20	0.999959	1	8	1	10	0	5	5	0.99284883	1	11.5	11.4267856					7.8E+07	7.8E+07	0.65596	ID=promoter_10302;
11	8E+07	11_78496003	58	30	10	30	44	20	0.001287	0.0774	144	7	25	3	76	14	5.07E-09	1.33E-06	38.375	46.1393464					7.8E+07	7.8E+07	0.44678	ID=promoter_10311;
11	8E+07	11_78528223	283	226	254	374	255	314	0.846835	1	235	173	299	89	204	194	0.96820386	1	241.63	85.3328567					7.9E+07	7.9E+07	0.1441	ID=promoter_10321;
11	8E+07	11_78531782	125	55	138	148	90	143	0.077018	1	96	14	39	15	55	27	0.02271744	0.7568038	78.75	54.9590757					7.9E+07	7.9E+07	0.10164	ID=promoter_10323;
11	8E+07	11_78539595	90	85	30	20	88	25	9.47E-11	#######	17	16	15	3	17	9	0.38755874	1	34.5	33.5559235					7.9E+07	7.9E+07	0.6653	ID=promoter_10327;
11	8E+07	11_78584235	21	6	24	26	14	25	0.447365	1	19	30	21	2	25	12	0.16190573	1	18.625	9.70916357	8E+07	7.9E+07	ID=ENSP00000261965;TUBGCP3;NM_006322;	TUBGCP3				
11	8E+07	11_78603987	517	138	394	310	328	352	0.510944	1	222	129	191	92	176	142	0.46795421	1	249.13	147.373614					7.9E+07	7.9E+07	0.5472	ID=promoter_10342;
11	8E+07	11_78610760	1	1	10	20	1	15	0.006136	0.2768	25	0	12	14	13	13	0.98151319	1	10.375	9.30341719								
11	8E+07	11_78644630	623	211	101	126	417	114	8.72E-50	#######	123	120	34	42	122	38	5.54E-09	1.44E-06	172.5	190.160083					7.9E+07	7.9E+07	0.45081	ID=promoter_10353;
11	8E+07	11_78661686	7	52	9	22	30	16	0.032973	1	46	4	60	20	25	40	0.09550227	1	27.5	22.0389266					7.9E+07	7.9E+07	0.61426	ID=promoter_10358;
11	8E+07	11_78672480	16	55	26	22	36	24	0.094407	1	63	51	51	47	57	49	0.9276628	1	41.375	17.4268881					7.9E+07	7.9E+07	0.99704	ID=promoter_10359;
11	8E+07	11_78697128	173	146	113	122	160	118	5.85E-04	0.04039	3	37	16	39	20	28	0.36590545	1	81.125	64.8105756					7.9E+07	7.9E+07	0.10782	ID=promoter_10366;
11	8E+07	11_78745920	0	3	9	10	2	10	0.124792	1	13	11	9	14	12	12	0.98292303	1	8.625	4.80884602					7.9E+07	7.9E+07	0.38098	ID=promoter_10384;
11	8E+07	11_78755678	174	109	194	145	142	170	0.983957	1	62	11	26	15	37	21	0.1929982	1	92	73.6516899								
11	8E+07	11_78806132	37	47	19	37	42	28	0.063705	1	31	31	72	0	31	36	0.65410506	1	34.25	20.8035025					7.9E+07	7.9E+07	0.35773	ID=promoter_10405;

11	8E+07	11_78808082	0	32	18	1	16	10	0.283594	1	20	82	27	2	51	15	2.00E-04	0.0164781	22.75	26.9006107								
11	8E+07	11_78809505	18	35	9	3	27	6	2.01E-04	0.01628	51	33	50	19	42	35	0.89717353	1	27.25	17.926437					7.9E+07	7.9E+07	0.12674	ID=promoter_10406;
11	8E+07	11_78818417	11	7	12	17	9	15	0.704209	1	0	17	1	1	9	1	0.05455957	1	8.25	7.06601524					7.9E+07	7.9E+07	0.43983	ID=promoter_10410;
11	8E+07	11_78842894	30	23	7	13	27	10	0.004472	0.21437	1	12	41	1	7	21	0.01731975	0.610307	16	14.2728514	8E+07	7.9E+07	ID=ENSP0000364589;CUL4A;NM_001008895;	CUL4A	7.9E+07	7.9E+07	0.89529	ID=promoter_10415;
11	8E+07	11_78845736	0	21	0	3	11	2	0.020291	0.71411	15	18	19	2	17	11	0.64636287	1	9.75	9.28516482	8E+07	7.9E+07	ID=ENSP0000364589;CUL4A;NM_001008895;	CUL4A				
11	8E+07	11_78851208	2	40	7	5	21	6	0.004166	0.20266	18	7	12	2	13	7	0.50249747	1	11.625	12.6371052	8E+07	7.9E+07	ID=ENSP0000364589;CUL4A;NM_001008895;	CUL4A	7.9E+07	7.9E+07	0.90559	ID=promoter_10418;
11	8E+07	11_78876924	217	131	112	106	174	109	1.21E-06	0.00018	177	68	110	57	123	84	0.09545295	1	122.25	53.1460253					7.9E+07	7.9E+07	0.23775	ID=promoter_10426;
11	8E+07	11_78878450	9	26	2	6	18	4	0.003432	0.17404	145	82	95	24	114	60	0.00155095	0.0885797	48.625	52.4157487					7.9E+07	7.9E+07	0.3662	ID=promoter_10427;
11	8E+07	11_78883948	77	114	96	90	96	93	0.420312	1	79	73	105	371	76	238	1.15E-21	8.46E-19	125.63	100.159783					7.9E+07	7.9E+07	0.41188	ID=promoter_10429;
11	8E+07	11_78922081	3319	3237	4864	6666	3278	5765	3.37E-76	#######	3341	2602	5867	1602	2972	3735	2.96E-34	3.58E-31	3937.3	1711.02557					7.9E+07	7.9E+07	0.5808	ID=promoter_10439;
11	8E+07	11_78927099	47	45	54	61	46	58	0.935162	1	27	54	21	7	41	14	0.0035084	0.1738771	39.5	18.9887185					7.9E+07	7.9E+07	0.42177	ID=promoter_10440;
11	8E+07	11_78927710	157	149	149	175	153	162	0.661905	1	147	82	103	4	115	54	1.46E-04	0.0126341	120.75	56.0478112					7.9E+07	7.9E+07	0.42177	ID=promoter_10440;
11	8E+07	11_78961966	143	57	85	55	100	70	0.003665	0.18279	1	4	13	0	3	7	0.3829009	1	44.75	50.8688791								
11	8E+07	11_78975882	94	47	60	43	71	52	0.03368	1	62	13	35	31	38	33	0.96300436	1	48.125	24.3922558					7.9E+07	7.9E+07	0.52151	ID=promoter_10459;
11	8E+07	11_78978273	207	107	185	158	157	172	0.825812	1	130	82	148	18	106	83	0.51421579	1	129.38	60.2801495					7.9E+07	7.9E+07	0.13223	ID=promoter_10460;
11	8E+07	11_78989359	12	13	6	11	13	9	0.46931	1	0	19	1	18	10	10	0.98574881	1	10	7.13141941								
11	8E+07	11_79000798	23	11	9	8	17	9	0.140533	1	1	9	3	1	5	2	0.58563225	1	8.125	7.16015762					7.9E+07	7.9E+07	0.67257	ID=promoter_10466;
11	8E+07	11_79039457	13	3	8	11	8	10	0.991043	1	2	23	20	3	13	12	0.99994263	1	10.375	7.96308447	8E+07	7.9E+07	ID=ENSP0000264952-D2;GRK7;NM_139209;	GRK7	7.9E+07	7.9E+07	0.2988	ID=promoter_10476;
11	8E+07	11_79088407	33	17	63	29	25	46	0.188315	1	36	52	30	10	44	20	0.02618764	0.844814	33.75	17.2191754	8E+07	7.9E+07	ID=ENSP0000364502;FAM70B;NM_182614;	FAM70B	7.9E+07	7.9E+07	0.46472	ID=promoter_10487;
11	8E+07	11_79097634	232	107	117	131	170	124	2.63E-04	0.02061	63	58	48	4	61	26	0.00307085	0.1550988	95	69.2572843	8E+07	7.9E+07	ID=ENSP0000364502;FAM70B;NM_182614;	FAM70B				
11	8E+07	11_79104527	51	13	35	27	32	31	0.749073	1	16	12	9	2	14	6	0.26891969	1	20.625	16.0618225					7.9E+07	7.9E+07	0.33964	ID=promoter_10491;
11	8E+07	11_79115534	6	35	15	4	21	10	0.052655	1	13	18	47	35	16	41	0.00153524	0.0883233	21.625	15.5282002	8E+07	7.9E+07	ID=ENSP0000349962;GAS6;;	GAS6	7.9E+07	7.9E+07	0.42361	ID=promoter_10494;

11	8E+07	11_79135315	16	38	27	32	27	30	0.979365	1	0	50	51	1	25	26	0.91912473	1	26.875	19.9172395	8E+07	7.9E+07	ID=ENSP0000349962;GAS6;;	GAS6	7.9E+07	7.9E+07	0.22866	ID=promoter_10500;
11	8E+07	11_79152963	208	85	160	238	147	199	0.418897	1	37	35	38	0	36	19	0.13186357	1	100.13	89.8974614								
11	8E+07	11_79184969	12	26	30	16	19	23	0.994841	1	29	30	33	0	30	17	0.26150314	1	22	11.5511286								
11	8E+07	11_79216377	74	89	10	7	82	9	3.45E-16	#######	0	35	37	1	18	19	0.92496372	1	31.625	34.1004294	8E+07	7.9E+07	ID=ENSP0000374195;RASA3;;	RASA3	7.9E+07	7.9E+07	0.2504	ID=promoter_10517;
11	8E+07	11_79227187	13	0	7	6	7	7	0.956352	1	1	27	5	0	14	3	0.04246461	1	7.375	9.05439909	8E+07	7.9E+07	ID=ENSP0000374195;RASA3;;	RASA3				
11	8E+07	11_79228234	73	41	71	87	57	79	0.629981	1	40	63	45	26	52	36	0.40124865	1	55.75	20.789764	8E+07	7.9E+07	ID=ENSP0000374195;RASA3;;	RASA3				
11	8E+07	11_79237233	73	27	65	60	50	63	0.931036	1	0	0	6	0	0	3	0.19828566	1	28.875	32.1800182	8E+07	7.9E+07	ID=ENSP0000374195;RASA3;;	RASA3	7.9E+07	7.9E+07	0.12906	ID=promoter_10522;
11	8E+07	11_79238120	6	6	17	12	6	15	0.276126	1	11	2	13	1	7	7	0.99000271	1	8.5	5.63154381	8E+07	7.9E+07	ID=ENSP0000374195;RASA3;;	RASA3				
11	8E+07	11_79244655	31	13	28	26	22	27	0.987654	1	29	2	20	44	16	32	0.03645929	1	24.125	12.620137	8E+07	7.9E+07	ID=ENSP0000374195;RASA3;;	RASA3	7.9E+07	7.9E+07	0.16562	ID=promoter_10526;
11	8E+07	11_79245235	2	23	18	8	13	13	0.920459	1	17	14	24	2	16	13	0.9394435	1	13.5	8.68496237	8E+07	7.9E+07	ID=ENSP0000374195;RASA3;;	RASA3				
11	8E+07	11_79286263	1106	559	967	950	833	959	0.925262	1	229	135	141	170	182	156	0.77239228	1	532.13	418.75613								
11	8E+07	11_79291953	130	75	103	71	103	87	0.076897	1	90	78	74	109	84	92	0.54242813	1	91.25	21.0560477								
11	8E+07	11_79296954	23	27	5	11	25	8	0.002813	0.14803	6	22	12	31	14	22	0.29543416	1	17.125	9.87692115								
11	8E+07	11_79481797	4	29	7	1	17	4	0.005755	0.26328	24	8	15	2	16	9	0.48000706	1	11.25	10.4437268	8E+07	7.9E+07	ID=ENSP0000355388;MPHOSPH8;NM_017520;	MPHOSPH8	7.9E+07	7.9E+07	0.38179	ID=promoter_10548;
11	8E+07	11_79578495	1	10	8	15	6	12	0.558139	1	41	0	19	4	21	12	0.40192224	1	12.25	13.3496923					8E+07	8E+07	0.69722	ID=promoter_10554;
11	8E+07	11_79578844	4	0	7	15	2	11	0.085797	1	0	20	6	20	10	13	0.72167512	1	9	8.26351707					8E+07	8E+07	0.69722	ID=promoter_10554;
11	8E+07	11_79597456	0	18	15	3	9	9	0.944235	1	0	0	2	5	0	4	0.11562589	1	5.375	7.1301673					8E+07	8E+07	0.24244	ID=promoter_10557;
11	8E+07	11_79654721	33	18	18	24	26	21	0.441319	1	10	17	55	11	14	33	0.01005158	0.3950437	23.25	14.7527237	8E+07	8E+07	ID=ENSP0000371308;CENPJ;NM_018451;	CENPJ	8E+07	8E+07	0.37371	ID=promoter_10563;
11	8E+07	11_79758317	22	9	22	30	16	26	0.588052	1	23	15	50	16	19	33	0.08582638	1	23.375	12.4663834								
11	8E+07	11_79769944	35	61	9	1	48	5	6.41E-10	#######	32	38	41	38	35	40	0.66345137	1	31.875	18.8712745					8E+07	8E+07	0.42292	ID=promoter_10579;
11	8E+07	11_79795065	0	1	9	0	1	5	0.353258	1	25	13	8	19	19	14	0.80779431	1	9.375	9.24179172					8E+07	8E+07	0.43491	ID=promoter_10581;
12	69458	12_69458	14	7	10	41	11	26	0.138574	1	36	1	7	2	19	5	0.02799778	0.8875672	14.75	15.2853805								
12	75324	12_75324	181	84	86	80	133	83	2.71E-05	0.00284	39	2	3	3	21	3	0.0022553	0.1213334	59.75	61.6389255					75252	79965	0.78366	ID=promoter_10599;
12	100335	12_100335	0	0	218	241	0	230	2.63E-43	#######	1	7	94	24	4	59	4.30E-12	1.66E-09	73.125	101.62176					99375	100800	0.49459	ID=promoter_10604;

12	125871	12_125871	1	15	4	8	8	6	0.705675	1	11	7	5	0	9	3	0.27712561	1	6.375	5.01248441	123745	146211	ID=ENSP0000248139-D4;RAB40C;NM_021168;	RAB40C	122759	126897	0.6755	ID=promoter_10612;
12	138272	12_138272	15	43	27	4	29	16	0.046212	1	24	25	14	4	25	9	0.04125862	1	19.5	13.0384048	123745	146211	ID=ENSP0000248139-D4;RAB40C;NM_021168;	RAB40C				
12	217841	12_217841	42	71	32	33	57	33	0.004384	0.2112	39	28	75	82	34	79	2.12E-05	0.0024372	50.25	21.9463632					216809	218004	0.48792	ID=promoter_10637;
12	227043	12_22704	67	37	27	20	52	24	4.64E-04	0.03323	10	26	38	5	18	22	0.68341813	1	28.75	19.344619								
12	233786	12_23378	3	7	3	9	5	6	0.999292	1	175	77	19	14	126	17	4.82E-17	2.80E-14	38.375	60.3298966								
12	267888	12_267888	16	3	8	14	10	11	0.989255	1	25	5	3	0	15	2	0.01114184	0.4293828	9.25	8.44731572					267829	269155	0.14282	ID=promoter_10648;
12	295514	12_295514	5	3	10	11	4	11	0.322758	1	18	7	3	7	13	5	0.22538616	1	8	4.98569382					293939	297851	0.70865	ID=promoter_10652;
12	296724	12_296724	25	2	7	2	14	5	0.053576	1	1	15	47	35	8	41	4.06E-06	0.0005723	16.75	17.3101952					293939	297851	0.70865	ID=promoter_10652;
12	346109	12_346109	47	20	16	19	34	18	0.01975	0.69938	22	10	11	10	16	11	0.74569574	1	19.375	12.1177261					345627	348051	0.55212	ID=promoter_10668;
12	367745	12_367745	102	72	101	167	87	134	0.139549	1	15	21	15	50	18	33	0.0599435	1	67.875	53.7359616	365696	369091	ID=ENSP0000300784;FN3K;NM_022158;	FN3K	367104	367920	0.21676	ID=promoter_10672;
12	369691	12_369691	0	64	23	13	32	18	0.038646	1	1	8	12	39	5	26	3.56E-04	0.0266828	20	21.7912171					368518	369995	0.22836	ID=promoter_10673;
12	371134	12_371134	11	69	24	29	40	27	0.080128	1	49	24	22	1	37	12	0.00419427	0.1971915	28.625	21.4005174					370965	372459	0.24486	ID=promoter_10674;
12	384301	12_38430	196	86	257	217	141	237	0.003078	0.15918	127	97	81	23	112	52	1.46E-04	0.0126341	135.5	79.9070889								
12	387972	12_38797	19	0	7	7	10	7	0.570701	1	12	0	3	2	6	3	0.67473421	1	6.25	6.5846141								
12	399794	12_399794	0	29	2	0	15	1	6.51E-04	0.04418	19	5	13	0	12	7	0.61698677	1	8.5	10.8364464					397229	399951	0.43705	ID=promoter_10681;
12	402738	12_402738	16	33	10	7	25	9	0.005635	0.25889	18	1	17	35	10	26	0.01509923	0.5480081	17.125	11.8735901					400256	402812	0.52456	ID=promoter_10682;
12	441812	12_44181	25	34	29	14	30	22	0.240296	1	29	14	48	44	22	46	0.00551602	0.2487658	29.625	12.3859079								
12	447120	12_44712	9	40	5	20	25	13	0.050329	1	28	30	14	22	29	18	0.40423266	1	21	11.6004926								
12	449335	12_44933	74	67	106	105	71	106	0.288458	1	46	17	28	0	32	14	0.05628287	1	55.375	39.4314505								
12	461586	12_461586	20	17	14	30	19	22	0.999149	1	15	25	17	32	20	25	0.60650656	1	21.25	6.9230464					460712	462134	0.37553	ID=promoter_10700;
12	490345	12_490345	15	19	5	6	17	6	0.027279	0.89953	19	33	15	20	26	18	0.63344191	1	16.5	8.81557064					489545	490759	0.34692	ID=promoter_10707;
12	545518	12_545518	29	10	45	26	20	36	0.302044	1	62	26	7	48	44	28	0.29364396	1	31.625	18.9505937	501027	549559	ID=ENSP0000333932;TBCD;;	TBCD	543992	547501	0.61888	ID=promoter_10721;
12	547169	12_547169	18	56	54	93	37	74	0.027431	0.90413	21	31	31	34	26	33	0.48500888	1	42.25	24.6793725	501027	549559	ID=ENSP0000333932;TBCD;;	TBCD	543992	547501	0.61888	ID=promoter_10721;
12	553104	12_553104	25	49	4	8	37	6	9.51E-07	0.00014	23	8	28	40	16	34	0.01900644	0.6536112	23.125	16.0573748					549016	558308	0.98578	ID=promoter_10723;
12	562663	12_562663	48	52	288	373	50	331	9.08E-37	#######	24	15	200	36	20	118	1.62E-17	9.84E-15	129.5	138.881244	557531	565011	ID=ENSP0000219542-D2;METRN;NM_024042;	METRN				
12	571597	12_571597	800	409	874	589	605	732	0.852617	1	175	57	126	147	116	137	0.15715071	1	397.13	322.439557					571256	571996	0.19504	ID=promoter_10729;

12	577305	12_57730	6	20	6	6	13	6	0.146758	1	13	0	20	7	7	14	0.23485029	1	9.75	7.22594532								
12	595541	12_59554	74	50	36	61	62	49	0.115178	1	17	15	35	1	16	18	0.85288016	1	36.125	24.7642455								
12	611140	12_61114	7	1	4	0	4	2	0.597627	1	37	2	4	1	20	3	0.00346999	0.1723921	7	12.328828								
12	616334	12_616334	14	18	5	5	16	5	0.021336	0.74103	0	1	8	1	1	5	0.22510075	1	6.5	6.52467843					616048	618231	0.44842	ID=promoter_10741;
12	624611	12_624611	0	1	1	0	1	1	0.993645	1	36	0	6	1	18	4	0.01933304	0.6616555	5.625	12.4319577					620716	628212	0.94637	ID=promoter_10743;
12	645568	12_645568	19	3	8	9	11	9	0.722819	1	11	8	15	2	10	9	0.99792604	1	9.375	5.68048036					642227	650034	0.94724	ID=promoter_10747;
12	654527	12_654527	382	121	203	115	252	159	3.95E-09	#######	64	50	62	20	57	41	0.46221979	1	127.13	117.444744					652409	654858	0.45473	ID=promoter_10749;
12	671021	12_671021	26	13	8	15	20	12	0.173914	1	26	1	13	2	14	8	0.54462139	1	13	9.50187951					667379	673382	0.88641	ID=promoter_10752;
12	699865	12_699865	2	8	11	6	5	9	0.74134	1	0	16	7	0	8	4	0.59180275	1	6.25	5.57417515					696501	700412	0.62507	ID=promoter_10761;
12	701418	12_701418	60	43	63	41	52	52	0.717824	1	25	38	32	16	32	24	0.73408433	1	39.75	16.0690474					700627	707847	0.92805	ID=promoter_10762;
12	706713	12_706713	342	134	275	298	238	287	0.951355	1	79	118	56	79	99	68	0.16107082	1	172.63	113.701285					700627	707847	0.92805	ID=promoter_10762;
12	740383	12_740383	22	23	10	9	23	10	0.024151	0.81985	3	1	0	0	2	0	0.39572182	1	8.5	9.45666809					739787	741964	0.39243	ID=promoter_10770;
12	748933	12_74893	31	22	24	10	27	17	0.124321	1	16	15	17	58	16	38	0.00469731	0.2166328	24.125	15.1132629								
12	779415	12_779415	35	27	23	23	31	23	0.244309	1	27	35	42	16	31	29	0.99938482	1	28.5	8.34950811	778874	782605	ID=ENSP00000324196;AATK;NM_001080395;	AATK	779179	780511	0.37376	ID=promoter_10783;
12	781546	12_781546	114	128	145	187	121	166	0.422866	1	83	27	53	25	55	39	0.43845264	1	95.25	58.3652294	778874	782605	ID=ENSP00000324196;AATK;NM_001080395;	AATK	781541	782923	0.42499	ID=promoter_10785;
12	792586	12_792586	1471	550	1611	1075	1011	1343	0.011428	0.45336	10	41	29	12	26	21	0.89538361	1	599.88	690.884823					792037	793191	0.25446	ID=promoter_10788;
12	799287	12_79928	11	14	0	2	13	1	0.002067	0.11504	43	19	4	1	31	3	2.73E-05	0.0030031	11.75	14.3601632								
12	810360	12_81036	77	11	11	27	44	19	7.50E-04	0.04944	0	0	8	1	0	5	0.06742467	1	16.875	25.8757333								
12	811696	12_811696	17	19	20	23	18	22	0.991654	1	6	17	34	17	12	26	0.04325208	1	19.125	7.77243297					811429	812387	0.25284	ID=promoter_10794;
12	812894	12_81289	18	26	1	6	22	4	4.12E-04	0.03018	0	3	7	2	2	5	0.46647814	1	7.875	9.2803556								
12	816426	12_81642	24	3	23	34	14	29	0.208355	1	7	12	6	1	10	4	0.34296513	1	13.75	11.8773494								
12	828422	12_82842	18	17	14	6	18	10	0.152558	1	24	5	20	3	15	12	0.93002578	1	13.375	7.78161753								
12	866288	12_86628	85	87	143	83	86	113	0.729811	1	46	87	109	51	67	80	0.30911866	1	86.375	30.7382382								
12	868438	12_86843	181	85	136	123	133	130	0.334234	1	51	57	36	13	54	25	0.01373743	0.5068396	85.25	57.0532333								
12	904364	12_904364	6	76	26	28	41	27	0.06151	1	44	59	18	35	52	27	0.04641028	1	36.5	22.6147866					903791	906906	0.55879	ID=promoter_10821;
12	906352	12_906352	55	48	20	23	52	22	1.57E-04	0.01313	125	83	32	40	104	36	7.83E-07	0.0001342	53.25	35.2774716					903791	906906	0.55879	ID=promoter_10821;
12	910922	12_910922	0	0	2	14	0	8	0.033035	1	42	0	0	3	21	2	7.81E-04	0.0515074	7.625	14.6768769					907694	911954	0.62943	ID=promoter_10822;
12	919426	12_91942	90	99	72	83	95	78	0.063686	1	64	63	52	39	64	46	0.41838885	1	70.25	19.9409844								
12	980619	12_98061	88	74	124	94	81	109	0.644582	1	18	7	14	36	13	25	0.09257925	1	56.875	43.7833546								
12	993663	12_99366	0	0	37	27	0	32	1.19E-06	0.00018	0	0	0	0	0	0	1	1	8	15.0522898								
12	1E+06	12_10285	0	35	17	16	18	17	0.813792	1	37	15	8	3	26	6	0.00399118	0.1893894	16.375	13.5850496								
12	1E+06	12_1061161	11	17	29	20	14	25	0.447365	1	0	0	0	0	0	0	1	1	9.625	11.4009711					1060968	1063122	0.32114	ID=promoter_10850;
12	1E+06	12_1104558	1	25	1	0	13	1	0.002067	0.11504	25	20	0	2	23	1	9.32E-05	0.0086863	9.25	11.7807349	1E+06	1115819	ID=ENSP00000338218;RNF213;NM_020914;	RNF213	1104045	1104648	0.18121	ID=promoter_10865;

12	1E+06	12_1108044	244	87	278	196	166	237	0.150815	1	112	121	74	1	117	38	3.14E-08	7.18E-06	139.13	93.0597926	1E+06	1115819	ID=ENSP0000338218;RNF213;NM_020914;	RNF213	1107470	1108518	0.19924	ID=promoter_10866;
12	1E+06	12_1147186	8	25	17	9	17	13	0.50439	1	86	83	40	23	85	32	4.12E-05	0.0043065	36.375	31.3684896	1E+06	1161509	ID=ENSP0000324392;KIAA1618;NM_020954;	KIAA1618	1146980	1148465	0.16394	ID=promoter_10877;
12	1E+06	12_11870	3	25	5	1	14	3	0.010924	0.43763	0	2	2	6	1	4	0.36109265	1	5.5	8.1240384								
12	1E+06	12_12000	48	20	20	35	34	28	0.378922	1	22	0	10	0	11	5	0.40227598	1	19.375	16.5178475								
12	1E+06	12_12788	85	68	70	83	77	77	0.612063	1	7	81	86	29	44	58	0.20884644	1	63.625	29.5196666								
12	1E+06	12_1285626	3684	2368	4247	5299	3026	4773	2.87E-36	#######	2834	2015	4906	1803	2425	3355	2.22E-50	4.06E-47	3394.5	1337.6992	1E+06	1310450	ID=ENSP0000269349;EIF4A3;NM_014740;	EIF4A3	1284446	1286087	0.55664	ID=promoter_10905;
12	1E+06	12_1439484	13	34	7	3	24	5	3.79E-04	0.02808	21	6	10	16	14	13	0.99999086	1	13.75	10.0249688	1E+06	1447030	ID=ENSP0000374665;GAA;NM_001079804;	GAA				
12	2E+06	12_15058	0	0	7	10	0	9	0.02157	0.74463	10	0	13	17	5	15	0.05518429	1	7.125	6.55607679								
12	2E+06	12_1560399	16	31	19	5	24	12	0.04556	1	26	2	13	13	14	13	0.99999086	1	15.625	9.76784082	2E+06	1565942	ID=ENSP0000309794;TBC1D16;NM_019020;	TBC1D16				
12	2E+06	12_16910	12	12	11	14	12	13	0.980472	1	0	1	0	1	1	1	0.99856566	1	6.375	6.34569595								
12	2E+06	12_16964	4	7	0	0	6	0	0.029633	0.9649	31	0	1	0	16	1	0.00230968	0.1234682	5.375	10.6628527								
12	2E+06	12_16965	19	4	14	14	12	14	0.999907	1	15	1	5	0	8	3	0.3843038	1	9	7.28991476								
12	2E+06	12_17112	1	19	2	4	10	3	0.082185	1	0	15	9	52	8	31	4.57E-04	0.0328393	12.75	17.2854191								
12	2E+06	12_1738941	2	5	1	16	4	9	0.54411	1	10	1	22	15	6	19	0.02032977	0.6899793	9	8					1738711	1739436	0.08898	ID=promoter_10960;
12	2E+06	12_18800	116	78	68	65	97	67	0.003389	0.17294	41	101	28	34	71	31	0.00164328	0.0927035	66.375	31.5456019								
12	2E+06	12_1907868	7	26	11	5	17	8	0.087954	1	36	0	9	7	18	8	0.20901227	1	12.625	12.082307	2E+06	1917551	ID=ENSP0000262768;TIMP2;NM_003255;	TIMP2	1907150	1908164	0.20129	ID=promoter_10982;
12	2E+06	12_1915597	17	1	9	2	9	6	0.554309	1	20	6	0	1	13	1	0.00904697	0.3617342	7	7.74596669	2E+06	1917551	ID=ENSP0000262768;TIMP2;NM_003255;	TIMP2				
12	2E+06	12_19881	85	58	43	48	72	46	0.004789	0.22701	162	103	12	41	133	27	2.66E-14	1.30E-11	69	46.8004884								
12	2E+06	12_19889	55	49	30	30	52	30	0.006781	0.29841	38	0	0	0	19	0	1.50E-04	0.0128633	25.25	22.5815981								
12	2E+06	12_2010104	23	47	21	11	35	16	0.005267	0.24489	36	20	39	41	28	40	0.20927466	1	29.75	12.6349854					2009672	2010917	0.31845	ID=promoter_11008;
12	2E+06	12_2030468	41	47	33	26	44	30	0.067875	1	26	30	15	16	28	16	0.29661245	1	29.25	11.132321					2029965	2031133	0.28167	ID=promoter_11015;
12	2E+06	12_2040660	0	26	5	1	13	3	0.018372	0.65917	36	26	22	0	31	11	0.01758669	0.6154554	14.5	14.5209209					2038276	2042024	0.59023	ID=promoter_11018;
12	2E+06	12_21589	3	1	0	1	2	1	0.773064	1	30	10	1	1	20	1	3.70E-04	0.0275298	5.875	10.2600125								
12	2E+06	12_21844	6	28	4	2	17	3	0.002206	0.1213	0	1	5	0	1	3	0.56022579	1	5.75	9.26976653								
12	2E+06	12_22005	29	74	16	5	52	11	4.52E-08	#######	13	23	39	3	18	21	0.77315138	1	25.25	23.06977								
12	2E+06	12_22015	24	18	25	14	21	20	0.800056	1	0	4	3	0	2	2	0.99713337	1	11	10.5424313								
12	2E+06	12_2208804	780	270	614	350	525	482	5.11E-04	0.03592	12	21	62	40	17	51	5.27E-05	0.0052805	268.63	295.090755					2207875	2208990	0.2629	ID=promoter_11035;
12	2E+06	12_22833	172	67	42	51	120	47	1.80E-10	#######	37	66	64	23	52	44	0.90537885	1	65.25	45.8686011								
12	2E+06	12_2385520	2	5	12	15	4	14	0.128496	1	25	5	39	6	15	23	0.30916076	1	13.625	12.6484048					2384299	2385803	0.1702	ID=promoter_11065;
12	2E+06	12_2436663	462	401	295	221	432	258	2.53E-17	#######	277	254	145	75	266	110	2.38E-12	9.52E-10	266.25	125.864723					2436521	2437464	0.19552	ID=promoter_11073;
12	2E+06	12_2484176	6	10	2	4	8	3	0.206144	1	15	3	0	0	9	0	0.01542606	0.5535504	5	5.20988072					2484172	2484901	0.18955	ID=promoter_11080;

12	2E+06	12_2496920	267	92	266	190	180	228	0.742833	1	34	21	41	0	28	21	0.76300557	1	113.88	110.935551					2496134	2497075	0.16837	ID=promoter_11083;
12	2E+06	12_24982	12	3	9	1	8	5	0.532535	1	2	11	5	2	7	4	0.73798468	1	5.625	4.40575922								
12	3E+06	12_25664	38	8	28	11	23	20	0.617116	1	79	16	35	25	48	30	0.23376449	1	30	22.5515283								
12	3E+06	12_26088	15	4	17	8	10	13	0.970528	1	27	13	36	29	20	33	0.11912992	1	18.625	11.0445526								
12	3E+06	12_26431	30	0	14	7	15	11	0.4902	1	16	0	0	0	8	0	0.02452223	0.7977568	8.375	10.9536361								
12	3E+06	12_2677108	19	11	1	5	15	3	0.006447	0.2884	0	1	17	8	1	13	0.0036419	0.1775497	7.75	7.38241153					2676771	2677566	0.16071	ID=promoter_11122;
12	3E+06	12_26786	250	141	264	213	196	239	0.92204	1	83	113	73	15	98	44	2.40E-04	0.0192085	144	90.0555384								
12	3E+06	12_27225	85	52	40	29	69	35	1.75E-04	0.01448	40	55	34	31	48	33	0.41465317	1	45.75	18.3906032								
12	3E+06	12_27640	0	3	7	7	2	7	0.358464	1	8	3	20	13	6	17	0.046525	1	7.625	6.36816861								
12	3E+06	12_2796114	104	121	89	55	113	72	2.15E-04	0.01726	37	70	101	38	54	70	0.17739996	1	76.875	31.7104647					2794641	2799230	0.76451	ID=promoter_11144;
12	3E+06	12_2820221	11	8	7	17	10	12	0.998584	1	79	54	101	70	67	86	0.13377528	1	43.375	37.2824643					2818730	2821257	0.40815	ID=promoter_11150;
12	3E+06	12_28302	103	118	79	93	111	86	0.014597	0.54949	67	31	67	54	49	61	0.30440624	1	76.5	27.9284801								
12	3E+06	12_28390	166	118	119	97	142	108	0.002888	0.15067	178	104	135	41	141	88	0.01361032	0.5027997	119.75	42.6539229								
12	3E+06	12_28670	83	20	47	80	52	64	0.967331	1	51	8	19	0	30	10	0.01387572	0.5103594	38.5	31.7715057								
12	3E+06	12_2880209	777	303	628	470	540	549	0.061543	1	547	97	214	112	322	163	1.35E-09	3.87E-07	393.5	250.440184					2879494	2880949	0.33117	ID=promoter_11162;
12	3E+06	12_28934	33	46	19	12	40	16	6.92E-04	0.04656	38	36	111	25	37	68	0.00292801	0.1490973	40	30.6966727								
12	3E+06	12_29584	106	72	69	50	89	60	0.00356	0.17916	141	42	42	2	92	22	5.89E-09	1.52E-06	65.5	42.7150024								
12	3E+06	12_29687	881	357	1474	845	619	1160	2.54E-20	#######	200	143	141	140	172	141	0.556179	1	522.63	493.550674								
12	3E+06	12_30229	33	10	22	19	22	21	0.795425	1	21	4	6	2	13	4	0.12786863	1	14.625	10.8224304								
12	3E+06	12_3035016	478	149	599	518	314	559	1.94E-08	#######	173	133	201	82	153	142	0.99995184	1	291.63	204.307847					3033863	3035172	0.27562	ID=promoter_11175;
12	3E+06	12_30557	7	67	5	4	37	5	3.17E-07	#######	32	32	11	0	32	6	3.53E-04	0.0265555	19.75	22.7643205								
12	3E+06	12_3200983	76	25	46	30	51	38	0.102231	1	8	11	26	2	10	14	0.60490119	1	28	23.9344342					3199428	3201478	0.30911	ID=promoter_11193;
12	3E+06	12_32253	37	36	85	87	37	86	0.001856	0.10518	0	25	28	0	13	14	0.92696813	1	37.25	33.3113022								
12	3E+06	12_32462	135	27	62	88	81	75	0.334866	1	4	6	7	21	5	14	0.08295177	1	43.75	47.4665296								
12	3E+06	12_32956	11	3	8	0	7	4	0.503048	1	11	0	9	0	6	5	0.98461866	1	5.25	5.00713776								
12	3E+06	12_3425014	16	35	3	1	26	2	4.27E-06	0.00056	10	21	24	24	16	24	0.32201294	1	16.75	11.5850889					3424816	3426071	0.18865	ID=promoter_11237;
12	3E+06	12_3480923	933	273	1205	1253	603	1229	3.13E-28	#######	318	206	402	196	262	299	0.04844557	1	598.25	454.722443					3478093	3482319	0.8599	ID=promoter_11256;
12	3E+06	12_34868	83	44	59	97	64	78	0.974542	1	17	46	17	3	32	10	0.00696943	0.2965071	45.75	33.1091269								
12	4E+06	12_3528772	15	27	18	47	21	33	0.575361	1	7	27	11	23	17	17	0.97589399	1	21.875	12.4606524	4E+06	3533281	ID=ENSP0000314973;PRPSAP1;NM_002766;	PRPSAP1	3528291	3529357	0.17818	ID=promoter_11265;
12	4E+06	12_36229	15	24	50	19	20	35	0.356186	1	5	60	55	34	33	45	0.23803368	1	32.75	20.310096								
12	4E+06	12_3628566	9	31	14	19	20	17	0.618382	1	77	4	30	3	41	17	0.01650079	0.5857858	23.375	24.1479813					3628302	3629270	0.18616	ID=promoter_11282;
12	4E+06	12_3649629	0	29	9	19	15	14	0.827043	1	52	0	5	3	26	4	6.99E-04	0.0468789	14.625	18.1654106					3647814	3649982	0.37317	ID=promoter_11285;
12	4E+06	12_36625	550	488	599	620	519	610	0.999423	1	594	595	178	363	595	271	5.07E-22	3.81E-19	498.38	154.521323								
12	4E+06	12_36775	9	21	9	32	15	21	0.871674	1	12	19	5	10	16	8	0.35023049	1	14.625	8.83074985								
12	4E+06	12_3737162	8	33	4	9	21	7	0.0088	0.36783	0	49	18	11	25	15	0.40601431	1	16.5	16.5529454	4E+06	3760425	ID=ENSP0000312066;SRP68;NM_014230;	SRP68				
12	4E+06	12_3747417	31	77	19	52	54	36	0.029007	0.94901	5	66	65	14	36	40	0.73226539	1	41.125	27.3257466	4E+06	3760425	ID=ENSP0000312066;SRP68;NM_014230;	SRP68	3746940	3747825	0.16633	ID=promoter_11302;
12	4E+06	12_3759181	1	21	11	3	11	7	0.438207	1	25	1	13	1	13	7	0.50249747	1	9.5	9.60654539	4E+06	3760425	ID=ENSP0000312066;SRP68;NM_014230;	SRP68	3757293	3759928	0.47423	ID=promoter_11306;

12	4E+06	12_3762313	22	4	6	14	13	10	0.600182	1	0	6	3	0	3	2	0.9363008	1	6.875	7.54865172					3762048	3764497	0.38134	ID=promoter_11308;
12	4E+06	12_3764032	760	311	653	343	536	498	8.74E-04	0.05696	248	168	293	171	208	232	0.1524086	1	368.38	219.608702					3762048	3764497	0.38134	ID=promoter_11308;
12	4E+06	12_38414	98	52	104	85	75	95	0.883496	1	22	46	40	14	34	27	0.83476892	1	57.625	34.1841129								
12	4E+06	12_38665	63	44	22	91	54	57	0.856981	1	358	68	54	112	213	83	4.29E-11	1.49E-08	101.5	107.238053								
12	4E+06	12_3896958	261	97	160	201	179	181	0.370466	1	104	87	56	32	96	44	4.10E-04	0.0300518	124.75	77.0672248					3895914	3897726	0.60189	ID=promoter_11327;
12	4E+06	12_3919609	318	241	213	240	280	227	1.75E-04	0.01448	81	62	48	52	72	50	0.28940884	1	156.88	107.377089	4E+06	3923278	ID=ENSP0000254806;WBP2;NM_012478;	WBP2				
12	4E+06	12_3921786	390	175	335	294	283	315	0.814891	1	964	577	677	284	771	481	5.99E-11	2.05E-08	462	260.506101	4E+06	3923278	ID=ENSP0000254806;WBP2;NM_012478;	WBP2				
12	4E+06	12_40639	734	142	238	246	438	242	7.84E-21	#######	102	124	58	9	113	34	1.05E-08	2.58E-06	206.63	227.957977								
12	4E+06	12_41347	93	169	68	128	131	98	0.003132	0.16124	114	175	185	80	145	133	0.99608821	1	126.5	45.4281222								
12	4E+06	12_4184545	933	433	895	504	683	700	0.04276	1	111	103	103	76	107	90	0.79341382	1	394.75	359.704939					4184301	4185033	0.12671	ID=promoter_11357;
12	4E+06	12_42078	14	26	48	83	20	66	1.27E-04	0.01105	3	26	9	4	15	7	0.31064188	1	26.625	27.1921076								
12	4E+06	12_4214767	79	34	31	65	57	48	0.236028	1	0	22	10	0	11	5	0.40227598	1	30.125	29.048912					4214368	4215655	0.38403	ID=promoter_11362;
12	4E+06	12_4214788	3186	1918	2867	2074	2552	2471	8.76E-11	#######	1768	1228	1493	585	1498	1039	5.11E-12	1.97E-09	1889.9	843.677907					4214368	4215655	0.38403	ID=promoter_11362;
12	4E+06	12_4380676	642	342	569	726	492	648	0.15259	1	375	299	374	160	337	267	0.15855521	1	435.88	191.076228					4380369	4381740	0.34503	ID=promoter_11376;
12	5E+06	12_45201	10	18	2	7	14	5	0.053576	1	9	0	0	1	5	1	0.30486099	1	5.875	6.35694221								
12	5E+06	12_4663087	269	124	270	282	197	276	0.162167	1	79	107	84	71	93	78	0.80826835	1	160.75	95.0364592	5E+06	4673674	ID=ENSP0000334849;TMEM104;NM_017728;	TMEM104	4662729	4663930	0.22621	ID=promoter_11414;
12	5E+06	12_4667416	54	35	61	57	45	59	0.853486	1	5	55	64	2	30	33	0.79414776	1	41.625	25.0709707	5E+06	4673674	ID=ENSP0000334849;TMEM104;NM_017728;	TMEM104				
12	5E+06	12_4931046	188	50	91	105	119	98	0.033491	1	104	41	51	12	73	32	0.0014486	0.0855753	80.25	54.6044216	5E+06	4934413	ID=ENSP0000269346;TTYH2;NM_032646;	TTYH2				
12	5E+06	12_4943452	644	133	350	244	389	297	1.42E-07	#######	151	80	121	33	116	77	0.07383236	1	219.5	197.827486					4943236	4944684	0.41205	ID=promoter_11432;
12	5E+06	12_50126	145	40	113	114	93	114	0.951465	1	25	86	68	7	56	38	0.3292632	1	74.75	48.3787143								
12	5E+06	12_50197	10	24	6	0	17	3	0.002206	0.1213	0	1	29	48	1	39	3.25E-09	8.76E-07	14.75	17.3925271								
12	5E+06	12_5047118	56	43	22	22	50	22	3.52E-04	0.02658	54	17	9	1	36	5	2.52E-05	0.0028067	28	20.5912603					5046653	5047448	0.0928	ID=promoter_11437;
12	5E+06	12_50487	2	24	9	22	13	16	0.991731	1	1	32	11	3	17	7	0.1782776	1	13	11.6373782								
12	5E+06	12_51981	24	38	19	15	31	17	0.035825	1	60	3	19	8	32	14	0.05628287	1	23.25	18.1875153								
12	5E+06	12_5299846	430	197	364	357	314	361	0.966839	1	150	119	128	101	135	115	0.80067498	1	230.75	131.4401					5299415	5300071	0.11811	ID=promoter_11450;
12	5E+06	12_53293	484	282	291	352	383	322	5.60E-05	0.00535	137	117	148	38	127	93	0.22315773	1	231.13	146.78501								
12	5E+06	12_5338229	50	8	20	5	29	13	0.011304	0.44898	36	15	12	7	26	10	0.05079892	1	19.125	15.9323346					5337687	5338380	0.14156	ID=promoter_11455;
12	5E+06	12_54985	9	14	0	3	12	2	0.011798	0.46127	26	0	3	0	13	2	0.02644099	0.8480056	6.875	9.20306626								
12	6E+06	12_5580678	4	11	8	19	8	14	0.661714	1	3	14	11	1	9	6	0.8208167	1	8.875	6.08129451	6E+06	5644637	ID=ENSP0000376421;SDK2;NM_001144952;	SDK2				

12	6E+06	12_5596000	99	49	21	22	74	22	8.00E-09	#######	19	22	11	4	21	8	0.08664698	1	30.875	30.4323019	6E+06	5644637	ID=ENSP00000376421;SDK2;NM_001144952;	SDK2				
12	6E+06	12_5611849	127	40	92	140	84	116	0.521714	1	34	31	32	27	33	30	0.99699964	1	65.375	47.0134859	6E+06	5644637	ID=ENSP00000376421;SDK2;NM_001144952;	SDK2	5611187	5612336	0.28333	ID=promoter_11481;
12	6E+06	12_5612637	4	12	3	12	8	8	0.950274	1	21	25	7	16	23	12	0.26207533	1	12.5	7.87400787	6E+06	5644637	ID=ENSP00000376421;SDK2;NM_001144952;	SDK2				
12	6E+06	12_57714	3	5	2	2	4	2	0.597627	1	31	4	0	1	18	1	9.25E-04	0.05912	6	10.2260172								
12	6E+06	12_60399	102	106	67	66	104	67	5.33E-04	0.03729	25	46	46	6	36	26	0.6231482	1	58	34.7768396								
12	6E+06	12_60713	12	31	12	19	22	16	0.340123	1	0	10	40	70	5	55	1.25E-10	4.12E-08	24.25	22.378242								
12	6E+06	12_6094397	544	254	491	327	399	409	0.159295	1	116	29	53	27	73	40	0.02584975	0.8363702	230.13	207.927486					6093910	6094840	0.24773	ID=promoter_11518;
12	6E+06	12_6166730	7	7	6	3	7	5	0.693374	1	16	11	10	3	14	7	0.39930956	1	7.875	4.35685011					6166445	6167096	0.14225	ID=promoter_11520;
12	6E+06	12_62583	12	5	2	0	9	1	0.020382	0.71452	19	8	6	0	14	3	0.04246461	1	6.5	6.50274667								
12	6E+06	12_62651	110	35	114	54	73	84	0.99285	1	31	37	10	26	34	18	0.15020178	1	52.125	38.9264416								
12	6E+06	12_63599	24	79	40	22	52	31	0.00985	0.40287	23	64	50	72	44	61	0.12269521	1	46.75	23.06977								
12	6E+06	12_6379492	589	390	612	508	490	560	0.915491	1	334	281	171	122	308	147	1.32E-10	4.32E-08	375.88	183.620289					6379223	6381095	0.44462	ID=promoter_11531;
12	6E+06	12_6481856	17	16	7	3	17	5	0.013234	0.50759	2	5	9	2	4	6	0.75330063	1	7.625	5.99851172					6481851	6482351	0.12613	ID=promoter_11536;
12	6E+06	12_6496241	0	21	7	2	11	5	0.189421	1	0	1	17	21	1	19	1.49E-04	0.0128238	8.625	9.48589178					6495741	6496745	0.15987	ID=promoter_11540;
12	7E+06	12_65019	2	5	6	4	4	5	0.995512	1	20	10	13	21	15	17	0.85096642	1	10.125	7.27888335								
12	7E+06	12_65207	5	7	19	15	6	17	0.158911	1	7	3	23	9	5	16	0.03625394	1	11	7.17137166								
12	7E+06	12_6538204	418	58	14	30	238	22	8.15E-48	#######	71	2	26	35	37	31	0.9172203	1	81.75	137.667041					6537488	6538359	0.15086	ID=promoter_11544;
12	7E+06	12_65709	25	28	27	35	27	31	0.996709	1	48	19	17	4	34	11	0.0064318	0.2773637	25.375	12.9938172								
12	7E+06	12_66246	17	57	4	15	37	10	4.04E-05	0.00403	0	13	13	4	7	9	0.80856893	1	15.375	17.8960291								
12	7E+06	12_66706	0	5	112	204	3	158	3.62E-28	#######	9	0	229	142	5	186	4.58E-41	7.27E-38	87.625	96.6923509								
12	7E+06	12_66710	55	79	36	41	67	39	0.001832	0.10396	31	24	55	33	28	44	0.08728134	1	44.25	17.8465683								
12	7E+06	12_67051	0	31	1	6	16	4	0.009581	0.39335	8	14	11	3	11	7	0.73613127	1	9.25	10.0249688								
12	7E+06	12_68659	9	14	32	30	12	31	0.05845	1	54	14	13	17	34	15	0.04959965	1	22.875	15.0469503								
12	7E+06	12_69843	196	79	102	73	138	88	3.39E-05	0.00346	0	0	16	18	0	17	1.04E-04	0.0094884	60.5	67.2819229								
12	7E+06	12_70518	6	4	1	5	5	3	0.645778	1	28	0	15	3	14	9	0.68990751	1	7.75	9.37702359								
12	7E+06	12_71684	39	73	36	35	56	36	0.016856	0.61678	35	23	46	59	29	53	0.0112655	0.4337974	43.25	15.8452336								
12	7E+06	12_72920	0	32	7	6	16	7	0.076817	1	4	2	12	20	3	16	0.00701108	0.2971393	10.375	10.7695006								
12	7E+06	12_74070	0	12	4	5	6	5	0.851367	1	3	2	8	7	3	8	0.26303803	1	5.125	3.79614466								
12	7E+06	12_74454	15	37	0	9	26	5	1.31E-04	0.01132	30	7	22	0	19	11	0.45547225	1	15	13.66957								
12	8E+06	12_75168	0	0	6	7	0	7	0.050594	1	0	2	4	37	1	21	5.13E-05	0.0051875	7	12.4326758								
12	8E+06	12_7530910	11	12	0	8	12	4	0.066899	1	0	1	14	18	1	16	7.41E-04	0.0492332	8	6.94879229					7530478	7531391	0.10572	ID=promoter_11587;
12	8E+06	12_75872	26	32	22	31	29	27	0.687522	1	41	43	17	4	42	11	3.56E-04	0.0266828	27	12.8062485								
12	8E+06	12_76533	11	0	5	12	6	9	0.896349	1	1	19	14	2	10	8	0.95278883	1	8	6.96932052								
12	8E+06	12_76808	6	54	6	22	30	14	0.012913	0.49818	15	10	33	3	13	18	0.54030568	1	18.625	17.4022782								
12	8E+06	12_79098	16	31	6	7	24	7	0.00213	0.11798	22	8	2	25	15	14	0.99983448	1	14.625	10.4736063								
12	8E+06	12_84803	4	13	7	32	9	20	0.269594	1	33	50	12	14	42	13	0.00136526	0.0810207	20.625	15.9636865								
12	8E+06	12_84803	1	13	3	0	7	2	0.160905	1	2	26	0	19	14	10	0.81923661	1	8	10.0569805								
12	9E+06	12_85991	107	102	120	111	105	116	0.905844	1	41	63	27	16	52	22	0.0067009	0.287233	73.375	41.6411112								
12	9E+06	12_88066	11	34	1	12	23	7	0.003444	0.1741	27	19	75	19	23	47	0.00621772	0.269925	24.75	22.6699928								
12	9E+06	12_93798	21	37	16	17	29	17	0.068354	1	24	33	57	26	29	42	0.17628028	1	28.875	13.4953696								
12	9E+06	12_94613	26	44	27	42	35	35	0.799998	1	55	52	22	37	54	30	0.07548247	1	38.125	12.2990999								
12	1E+07	12_9521348	21	175	55	68	98	62	5.70E-04	0.03951	95	71	98	24	83	61	0.38588418	1	75.875	49.1424678	9E+06	9546909	ID=ENSP00000376444;ABCA5;;	ABCA5				
12	1E+07	12_97422	117	85	131	116	101	124	0.943677	1	35	19	19	1	27	10	0.0371409	1	65.375	52.4920062								
12	1E+07	12_10001628	3	36	38	75	20	57	0.001973	0.11159	35	9	80	4	22	42	0.01957138	0.6677319	35	29.9904747								

12	1E+07	12_10021842	5	2	13	10	4	12	0.241003	1	27	10	35	1	19	18	0.99782856	1	12.875	12.1118301	1E+07	1E+07	ID=ENSP000002621 39;WIPI1;NM_017983;	WIPI1				
12	1E+07	12_10031530	2	57	10	8	30	9	5.55E-04	0.03862	36	19	5	45	28	25	0.9907189	1	22.75	20.6449579					1E+07	1E+07	0.26937	ID=promoter_11635;
12	1E+07	12_10095216	115	123	133	140	119	137	0.988964	1	1597	1106	1273	709	1352	991	1.31E-07	2.67E-05	649.5	608.005639	1E+07	1E+07	ID=ENSP000003473 10;ARSG;NM_014960;	ARSG				
12	1E+07	12_10114578	17	76	48	52	47	50	0.890832	1	83	52	73	16	68	45	0.21157912	1	52.125	25.3964986	1E+07	1E+07	ID=ENSP000003473 10;ARSG;NM_014960;	ARSG				
12	1E+07	12_10157594	17	3	16	5	10	11	0.989255	1	12	1	5	12	7	9	0.80856893	1	8.875	6.12809688								
12	1E+07	12_10217792	13	51	16	12	32	14	0.005901	0.26867	49	30	22	55	40	39	0.97519103	1	31	18.0949875								
12	1E+07	12_10241878	17	15	44	31	16	38	0.053936	1	25	7	40	11	16	26	0.20160145	1	23.75	13.5935909								
12	1E+07	12_10282630	29	10	2	1	20	2	1.33E-04	0.01141	33	55	44	29	44	37	0.90878977	1	25.375	19.5881706								
12	1E+07	12_10284342	19	25	5	6	22	6	0.002551	0.13706	1	3	8	6	2	7	0.20455692	1	9.125	8.37406711								
12	1E+07	12_10302488	54	23	30	12	39	21	0.012903	0.49818	23	9	3	7	16	5	0.08348313	1	20.125	16.5480901								
12	1E+07	12_10348716	189	1384	188	184	787	186	########	#######	806	770	1185	431	788	808	0.13144205	1	642.13	471.214673					1E+07	1E+07	0.1046	ID=promoter_11654;
12	1E+07	12_10429543	176	180	147	230	178	189	0.634635	1	93	110	55	86	102	71	0.17723387	1	134.63	58.6318721								
12	1E+07	12_10627642	4	23	7	4	14	6	0.098789	1	0	0	8	0	0	4	0.11562589	1	5.75	7.64852927								
12	1E+07	12_10681592	11	32	22	25	22	24	0.97026	1	53	27	39	0	40	20	0.07259135	1	26.125	16.2694938								
12	1E+07	12_10956450	0	33	6	2	17	4	0.005755	0.26328	43	14	40	20	29	30	0.9150801	1	19.75	17.1276718								
12	1E+07	12_11056733	27	48	25	23	38	24	0.054487	1	21	33	50	40	27	45	0.0504211	1	33.375	11.375883								
12	1E+07	12_11158412	11	24	3	16	18	10	0.152558	1	21	16	31	23	19	27	0.35594749	1	18.125	8.62616452								
12	1E+07	12_11193074	12	26	21	37	19	29	0.670114	1	32	15	23	18	24	21	0.98147478	1	23	8.45154255								
12	1E+07	12_11251402	47	5	35	38	26	37	0.751146	1	38	9	0	0	24	0	1.47E-05	0.0017742	21.5	19.7484177								
12	1E+07	12_11257270	39	23	24	12	31	18	0.053237	1	17	19	31	46	18	39	0.00899519	0.3617342	26.375	11.5379808					1.1E+07	1.1E+07	0.14781	ID=promoter_11703;
12	1E+07	12_11289060	31	15	50	26	23	38	0.427862	1	0	11	0	0	6	0	0.06196836	1	16.625	18.0153704					1.1E+07	1.1E+07	0.16693	ID=promoter_11710;
12	1E+07	12_11290888	63	23	69	68	43	69	0.269417	1	27	34	6	9	31	8	0.00261167	0.1361949	37.375	25.9391458								
12	1E+07	12_11294532	1	22	0	4	12	2	0.011798	0.46127	0	4	17	36	2	27	7.81E-06	0.0010203	10.5	13.2017315								
12	1E+07	12_11321060	94	85	80	121	90	101	0.954539	1	27	34	42	88	31	65	6.19E-04	0.042107	71.375	33.2520139								
12	1E+07	12_11329484	1362	894	1289	2014	1128	1652	6.25E-08	#######	816	663	1534	383	740	959	5.32E-11	1.83E-08	1119.4	528.240188								
12	1E+07	12_11330343	1	5	2	2	3	2	0.821455	1	32	8	11	3	20	7	0.06990458	1	8	10.2817453								
12	1E+07	12_11433103	9	22	30	17	16	24	0.746917	1	3	21	22	12	12	17	0.52622129	1	17	8.61891607	1E+07	1.1E+07	ID=ENSP000002621 38;CACNG4;NM_014405;	CACNG4				
12	1E+07	12_11773278	0	39	3	2	20	3	4.27E-04	0.03093	0	32	34	2	16	18	0.85288016	1	14	17.5254916								
12	1E+07	12_11884356	25	115	7	23	70	15	1.56E-10	#######	62	68	63	19	65	41	0.15152082	1	47.75	35.8279619								
12	1E+07	12_12123121	7	59	18	41	33	30	0.598685	1	88	14	49	25	51	37	0.52329888	1	37.625	27.1921076								

12	1E+07	12_12175784	33	28	39	39	31	39	0.958494	1	101	37	76	21	69	49	0.35923969	1	46.75	27.3012297					1.2E+07	1.2E+07	0.1116	ID=promoter_11737;
12	1E+07	12_12191928	21	18	44	42	20	43	0.075496	1	18	26	11	5	22	8	0.06315916	1	23.125	13.7989389								
12	1E+07	12_12474711	105	39	100	73	72	87	0.982754	1	83	74	47	45	79	46	0.04072886	1	70.75	25.1552323								
12	1E+07	12_12578289	28	115	24	24	72	24	8.96E-08	#######	89	51	73	62	70	68	0.96297557	1	58.25	33.1910057								
12	1E+07	12_12794134	22	19	24	31	21	28	0.906033	1	0	8	0	28	4	14	0.04184353	1	16.5	12.2591074								
12	1E+07	12_12840150	0	10	4	8	5	6	0.999292	1	1	10	26	28	6	27	5.47E-04	0.0380615	10.875	10.6561518	1E+07	1.3E+07	ID=ENSP00000354927;MAP3K3;NM_203351;	MAP3K3				
12	1E+07	12_12898068	230	94	162	136	162	149	0.099643	1	217	49	67	8	133	38	1.08E-10	3.60E-08	120.38	79.8193272					1.3E+07	1.3E+07	0.24617	ID=promoter_11778;
12	1E+07	12_12961575	9	10	4	1	10	3	0.082185	1	24	1	4	4	13	4	0.12786863	1	7.125	7.56755292								
12	1E+07	12_12989856	42	18	38	38	30	38	0.95166	1	15	68	12	52	42	32	0.70425168	1	35.375	19.5004579	1E+07	1.3E+07	ID=ENSP00000290866;ACE;NM_000789;	ACE	1.3E+07	1.3E+07	0.13028	ID=promoter_11790;
12	1E+07	12_13059150	62	74	65	49	68	57	0.1709	1	73	35	16	38	54	27	0.02898642	0.9098185	51.5	20.6397674	1E+07	1.3E+07	ID=ENSP00000374171;TANC2;;	TANC2				
12	1E+07	12_13109594	0	14	2	15	7	9	0.983538	1	51	1	1	4	26	3	2.53E-04	0.0200383	11	17.2212493	1E+07	1.3E+07	ID=ENSP00000374171;TANC2;;	TANC2				
12	1E+07	12_13227262	11	59	12	34	35	23	0.091136	1	41	1	12	0	21	6	0.02669383	0.8538132	21.25	21.0967161								
12	1E+07	12_13654689	1	26	2	0	14	1	0.001161	0.07164	0	13	23	1	7	12	0.42229866	1	8.25	10.9250695								
12	1E+07	12_13684453	118	66	117	72	92	95	0.68384	1	26	27	11	2	27	7	0.00575936	0.2548307	54.875	45.658476	1E+07	1.4E+07	ID=ENSP00000311496;MARCH10;NM_001100875;	10-Mar	1.4E+07	1.4E+07	0.18399	ID=promoter_11803;
12	1E+07	12_13716732	31	15	32	35	23	34	0.691592	1	1	23	34	18	12	26	0.04325208	1	23.625	11.8314049	1E+07	1.4E+07	ID=ENSP00000307513;MRC2;NM_006039;	MRC2				
12	1E+07	12_14045871	9	7	12	3	8	8	0.950274	1	63	42	65	20	53	43	0.80949557	1	27.625	25.4554934	1E+07	1.4E+07	ID=ENSP00000302649;EFCAB3;NM_173503;	EFCAB3				
12	1E+07	12_14729132	273	68	174	146	171	160	0.120005	1	89	58	72	42	74	57	0.57457512	1	115.25	78.1184997					1.5E+07	1.5E+07	0.41262	ID=promoter_11818;
12	1E+07	12_14807597	188	71	112	143	130	128	0.371408	1	122	60	75	32	91	54	0.03242805	0.9914713	100.38	50.5821468					1.5E+07	1.5E+07	0.457	ID=promoter_11819;
12	2E+07	12_15001036	59	75	72	69	67	71	0.837068	1	15	24	34	57	20	46	0.00210871	0.1150505	50.625	23.1574579	1E+07	1.5E+07	ID=ENSP00000340820;MAPT;NM_001123066;	MAPT				
12	2E+07	12_15338159	1438	801	1687	1366	1120	1527	6.58E-04	0.04461	801	679	443	358	740	401	2.11E-17	1.26E-14	946.63	489.844564					1.5E+07	1.5E+07	0.52634	ID=promoter_11833;
12	2E+07	12_15368254	1	11	12	16	6	14	0.355886	1	27	25	20	0	26	10	0.05079892	1	14	10.0569805								
12	2E+07	12_15509550	28	57	35	14	43	25	0.017316	0.62799	178	116	102	5	147	54	1.29E-08	3.13E-06	66.875	60.0533989					1.6E+07	1.6E+07	0.29354	ID=promoter_11835;
12	2E+07	12_15562297	201	132	255	247	167	251	0.04514	1	98	83	85	104	91	95	0.72020747	1	150.63	72.5946427								
12	2E+07	12_15852527	10	5	11	22	8	17	0.371907	1	0	0	0	0	0	0	1	1	6	7.94624799								

12	2E+07	12_15891308	20	25	8	11	23	10	0.024151	0.81985	1	0	13	4	1	9	0.02974549	0.9232309	10.25	8.90826262	2E+07	1.6E+07	ID=ENSP00000311682;;					
12	2E+07	12_15906501	12	6	14	27	9	21	0.212308	1	4	0	21	0	2	11	0.03110435	0.956324	10.5	9.85610761	2E+07	1.6E+07	ID=ENSP00000311682;;					
12	2E+07	12_15921994	228	98	270	183	163	227	0.246224	1	47	101	82	38	74	60	0.74246895	1	130.88	85.7978313					1.6E+07	1.6E+07	0.1775	ID=promoter_11866;
12	2E+07	12_15945560	2	10	4	8	6	6	0.962469	1	22	12	19	0	17	10	0.5154613	1	9.625	7.85470741								
12	2E+07	12_16019755	124	119	128	98	122	113	0.193306	1	37	58	32	21	48	27	0.11059688	1	77.125	44.9394434								
12	2E+07	12_16091326	91	44	90	67	68	79	0.998323	1	11	26	30	21	19	26	0.43045343	1	47.5	31.3824337	2E+07	1.6E+07	ID=ENSP00000329219;FMNL1;NM_005892;	FMNL1	1.6E+07	1.6E+07	0.53374	ID=promoter_11884;
12	2E+07	12_16120916	48	19	39	48	34	44	0.911425	1	23	27	64	13	25	39	0.12121754	1	35.125	17.4964282								
12	2E+07	12_16135772	266	64	249	184	165	217	0.541022	1	225	127	195	78	176	137	0.30752159	1	173.5	76.3020314					1.6E+07	1.6E+07	0.5498	ID=promoter_11886;
12	2E+07	12_16165256	339	104	167	150	222	159	1.01E-05	0.00121	438	332	221	66	385	144	8.47E-21	5.96E-18	227.13	130.222269	2E+07	1.6E+07	ID=ENSP00000313731;PLCD3;NM_133373;	PLCD3	1.6E+07	1.6E+07	0.25564	ID=promoter_11891;
12	2E+07	12_16221918	21	3	8	8	12	8	0.45534	1	1	0	9	24	1	17	4.35E-04	0.0314643	9.25	8.87613172	2E+07	1.6E+07	ID=ENSP00000341504;DCAKD;NM_024819;	DCAKD				
12	2E+07	12_16377484	35	5	37	30	20	34	0.415985	1	6	33	27	0	20	14	0.72101732	1	21.625	15.2684315								
12	2E+07	12_16389853	905	307	681	588	606	635	0.138885	1	355	126	114	189	241	152	9.12E-04	0.0584724	408.13	288.064694								
12	2E+07	12_16760068	13	29	31	9	21	20	0.800056	1	5	2	6	6	4	6	0.75330063	1	12.625	11.1986925								
12	2E+07	12_16808347	23	8	7	20	16	14	0.724026	1	1	9	13	1	5	7	0.77775394	1	10.25	8.04895734								
12	2E+07	12_16829168	16	4	19	12	10	16	0.741563	1	1	2	10	13	2	12	0.01895406	0.6519669	9.625	6.65340944	2E+07	1.7E+07	ID=ENSP00000262407;ITGA2B;NM_000419;	ITGA2B	1.7E+07	1.7E+07	0.22936	ID=promoter_11931;
12	2E+07	12_16840238	18	8	23	13	13	18	0.901125	1	1	11	1	1	6	1	0.20114407	1	9.5	8.34950811	2E+07	1.7E+07	ID=ENSP00000262407;ITGA2B;NM_000419;	ITGA2B	1.7E+07	1.7E+07	0.39044	ID=promoter_11932;
12	2E+07	12_16867035	5	7	15	10	6	13	0.45043	1	25	7	24	21	16	23	0.39752759	1	14.25	8.15475322	2E+07	1.7E+07	ID=ENSP00000377587-D7;SLC4A5;NM_133478;	SLC4A5				
12	2E+07	12_16941970	349	287	328	354	318	341	0.515639	1	230	183	168	102	207	135	0.00606328	0.2662974	250.13	93.7983209	2E+07	1.7E+07	ID=ENSP00000302640;UBTF;NM_014233;	UBTF				
12	2E+07	12_16967098	22	66	31	22	44	27	0.026351	0.87745	61	10	35	39	36	37	0.90740746	1	35.75	19.3741359					1.7E+07	1.7E+07	0.48057	ID=promoter_11945;
12	2E+07	12_17032695	126	43	104	134	85	119	0.459204	1	40	20	23	3	30	13	0.06382438	1	61.625	51.6220814					1.7E+07	1.7E+07	0.38722	ID=promoter_11949;
12	2E+07	12_17215772	31	32	13	8	32	11	9.30E-04	0.05947	23	28	15	3	26	9	0.02974521	0.9232309	19.125	10.9471131								
12	2E+07	12_17453632	4	9	11	4	7	8	0.998739	1	0	7	9	1	4	5	0.90483023	1	5.625	3.99776723								
12	2E+07	12_17789052	0	16	0	1	8	1	0.03583	1	8	0	4	16	4	10	0.21760186	1	5.625	6.96803928	2E+07	1.8E+07	ID=ENSP00000309432;FAM134C;NM_178126;	FAM134C					

12	2E+07	12_17800175	333	107	280	264	220	272	0.844839	1	152	13	70	76	83	73	0.94762438	1	161.88	116.346082								
12	2E+07	12_17801785	98	48	83	47	73	65	0.268719	1	26	11	14	19	19	17	0.99437087	1	43.25	32.5038459	2E+07	1.8E+07	ID=ENSP000002537 89;PSMC3IP;NM_013290;	PSMC3IP				
12	2E+07	12_17817351	13	46	8	6	30	7	1.06E-04	0.00938	11	7	10	2	9	6	0.8208167	1	12.875	13.7989389	2E+07	1.8E+07	ID=ENSP000003774 06;COASY;;	COASY				
12	2E+07	12_17827190	493	281	817	757	387	787	3.21E-18	#######	99	100	129	51	100	90	0.97945289	1	340.88	309.844402	2E+07	1.8E+07	ID=ENSP000002259 29;HSD17B1;NM_000413;	HSD17B1	1.8E+07	1.8E+07	0.47697	ID=promoter_12007;
12	2E+07	12_18154192	2042	614	1899	1593	1328	1746	0.007316	0.31625	757	581	606	289	669	448	6.06E-07	0.000106	1047.6	683.669916					1.8E+07	1.8E+07	0.51934	ID=promoter_12022;
12	2E+07	12_18183843	18	12	26	9	15	18	0.997876	1	14	0	5	0	7	3	0.51857467	1	10.5	8.9761589	2E+07	1.8E+07	ID=ENSP000003412 08-D2;STAT5A;NM_003152;	STAT5A				
12	2E+07	12_18325550	225	20	196	151	123	174	0.281307	1	33	63	43	15	48	29	0.18592233	1	93.25	84.3542699					1.8E+07	1.8E+07	0.46456	ID=promoter_12034;
12	2E+07	12_18325959	196	61	154	145	129	150	0.997377	1	99	66	42	1	83	22	1.84E-07	3.64E-05	95.5	65.2883713					1.8E+07	1.8E+07	0.46456	ID=promoter_12034;
12	2E+07	12_18355057	155	63	82	96	109	89	0.038784	1	104	47	34	2	76	18	1.39E-07	2.83E-05	72.875	47.2574635								
12	2E+07	12_18394980	99	47	92	142	73	117	0.109872	1	69	20	50	24	45	37	0.86385836	1	67.875	41.4364832								
12	2E+07	12_18524385	57	1	29	16	29	23	0.369822	1	56	60	54	18	58	36	0.16340404	1	36.375	23.1080536	2E+07	1.9E+07	ID=ENSP000003667 66;TTC25;NM_031421;	TTC25				
12	2E+07	12_18698600	75	30	52	64	53	58	0.935505	1	58	24	28	64	41	46	0.67308043	1	49.375	19.4270614					1.9E+07	1.9E+07	0.31871	ID=promoter_12069;
12	2E+07	12_18710449	833	273	564	523	553	544	0.014192	0.53665	224	208	146	76	216	111	1.97E-06	0.0003071	355.88	258.40137					1.9E+07	1.9E+07	0.16256	ID=promoter_12071;
12	2E+07	12_18896732	22	16	24	39	19	32	0.454442	1	48	24	36	6	36	21	0.23487	1	26.875	13.4741817								
12	2E+07	12_18990489	67	52	71	50	60	61	0.733608	1	0	40	20	3	20	12	0.48622029	1	37.875	27.430106								
12	2E+07	12_19290715	8	23	8	0	16	4	0.009581	0.39335	24	9	4	0	17	2	0.0046333	0.2138882	9.5	9.31971796					1.9E+07	1.9E+07	0.18834	ID=promoter_12110;
12	2E+07	12_19292078	23	31	17	28	27	23	0.526863	1	42	36	35	23	39	29	0.66629858	1	29.375	8.22778394								
12	2E+07	12_19641737	37	61	66	45	49	56	0.991209	1	86	14	59	19	50	39	0.72066795	1	48.375	24.4478308								
12	2E+07	12_19666706	1	40	23	19	21	21	0.874689	1	47	27	22	21	37	22	0.25078535	1	25	13.9078601								
12	2E+07	12_19721041	17	23	29	11	20	20	0.880283	1	27	9	7	18	18	13	0.78939737	1	17.625	8.26243651								
12	2E+07	12_19776283	81	73	107	132	77	120	0.148607	1	37	32	46	51	35	49	0.17214544	1	69.875	35.4821384	2E+07	2E+07	ID=ENSP000003670 47;TNS4;NM_032865;	TNS4				
12	2E+07	12_19806386	594	247	669	537	421	603	0.006993	0.30452	936	772	716	353	854	535	7.73E-12	2.90E-09	603	223.686004	2E+07	2E+07	ID=ENSP000002695 93;IGFBP4;NM_001552;	IGFBP4				
12	2E+07	12_19986568	93	65	72	69	79	71	0.263507	1	80	57	86	42	69	64	0.99999532	1	70.5	16.3270153	2E+07	2E+07	ID=ENSP000002646 44;RAPGEFL1;NM_016339;	RAPGEFL1				

12	2E+07	12_20065949	59	48	55	26	54	41	0.106533	1	0	0	9	1	0	5	0.06742467	1	24.75	25.8221942								
12	2E+07	12_20081190	217	155	290	193	186	242	0.568016	1	145	81	71	40	113	56	5.07E-04	0.0358548	149	83.7359796	2E+07	2E+07	ID=ENSP0000377679;THRA;NM_199334;	THRA	2E+07	2E+07	0.17837	ID=promoter_12154;
12	2E+07	12_20368943	27	11	37	37	19	37	0.192436	1	9	26	12	15	18	14	0.88543974	1	21.75	11.5232684								
12	2E+07	12_20632814	5	39	22	28	22	25	0.99414	1	35	13	38	2	24	20	0.93987967	1	22.75	14.713939	2E+07	2.1E+07	ID=ENSP0000300658;PERLD1;NM_033419;	PERLD1				
12	2E+07	12_20648159	15	10	0	14	13	7	0.234546	1	2	4	0	19	3	10	0.11508955	1	8	7.46420027					2.1E+07	2.1E+07	0.39126	ID=promoter_12201;
12	2E+07	12_20651108	16	21	16	20	19	18	0.809248	1	7	11	3	0	9	2	0.1390433	1	11.75	7.81482108								
12	2E+07	12_20710756	3	1	6	18	2	12	0.058554	1	13	7	1	0	10	1	0.03495212	1	6.125	6.42400632								
12	2E+07	12_20730095	60	51	75	62	56	69	0.963227	1	0	7	28	4	4	16	0.01700101	0.6008545	35.875	29.8158036								
12	2E+07	12_20792804	286	86	212	198	186	205	0.82772	1	111	42	67	48	77	58	0.4889536	1	131.25	89.7659656					2.1E+07	2.1E+07	0.29912	ID=promoter_12209;
12	2E+07	12_21030900	2	1	2	12	2	7	0.358464	1	8	6	3	7	7	5	0.9051169	1	5.125	3.79614466								
12	2E+07	12_21044050	84	72	95	141	78	118	0.216881	1	72	25	51	47	49	49	0.93208319	1	73.375	35.241767								
12	2E+07	12_21275982	66	55	51	65	61	58	0.518037	1	0	6	7	0	3	4	0.89184014	1	31.25	30.4243793								
12	2E+07	12_21395059	9	40	15	15	25	15	0.11231	1	9	15	25	16	12	21	0.20312388	1	18	10.1840211								
12	2E+07	12_21441607	13	16	5	0	15	3	0.006447	0.2884	43	0	7	1	22	4	0.00378795	0.1837142	10.625	14.3719717	2E+07	2.1E+07	ID=ENSP0000290158;KPNB1;NM_002265;	KPNB1				
12	2E+07	12_21456314	302	93	398	242	198	320	0.001769	0.10181	77	138	84	69	108	77	0.21045584	1	175.38	123.958446					2.1E+07	2.1E+07	0.44807	ID=promoter_12226;
12	2E+07	12_21593971	11	18	12	13	15	13	0.725173	1	22	1	0	0	12	0	0.00384008	0.1844256	9.625	8.46737436	2E+07	2.2E+07	ID=ENSP0000269025;LRRC46;NM_033413;	LRRC46				
12	2E+07	12_21687458	614	319	519	367	467	443	0.005847	0.26681	230	160	180	147	195	164	0.65528001	1	317	173.783116					2.2E+07	2.2E+07	0.26398	ID=promoter_12245;
12	2E+07	12_21744049	90	28	65	99	59	82	0.609575	1	39	47	30	1	43	16	0.00548623	0.2478586	49.875	33.0386678								
12	2E+07	12_22257469	15	59	9	9	37	9	1.73E-05	0.00192	14	0	15	30	7	23	0.00743961	0.3120589	18.875	18.2790864								
12	2E+07	12_22441404	2961	2866	3197	4935	2914	4066	7.29E-12	#######	2896	1850	3555	1469	2373	2512	2.13E-05	0.0024467	2966.1	1054.1505	2E+07	2.2E+07	ID=ENSP0000249499-D2;HOXD9;NM_014213;	HOXD9	2.2E+07	2.2E+07	0.57448	ID=promoter_12276;
12	2E+07	12_22827491	10	34	22	3	22	13	0.135741	1	16	13	30	23	15	27	0.11059826	1	18.875	10.3708038					2.3E+07	2.3E+07	0.35761	ID=promoter_12304;
12	2E+07	12_23124087	316	241	246	176	279	211	7.83E-06	0.00096	318	198	157	94	258	126	1.54E-08	3.70E-06	218.25	77.623174								
12	2E+07	12_23233995	5	15	7	16	10	12	0.998584	1	11	9	29	2	10	16	0.39084582	1	11.75	8.43038721	2E+07	2.3E+07	ID=ENSP0000240364;FAM117A;NM_030802;	FAM117A				
12	2E+07	12_23255940	9	3	6	11	6	9	0.896349	1	20	16	38	90	18	64	4.03E-07	7.42E-05	24.125	28.7622645	2E+07	2.3E+07	ID=ENSP0000240364;FAM117A;NM_030802;	FAM117A				

12	2E+07	12_24025906	44	0	21	2	22	12	0.091688	1	0	0	0	0	0	0	1	1	8.375	16.1239618	2E+07	2.4E+07	ID=ENSP00000285238;ABCC3;NM_003786;	ABCC3				
12	2E+07	12_24281013	4	2	6	2	3	4	0.986002	1	0	14	4	8	7	6	0.99011599	1	5	4.40778532								
12	2E+07	12_24341474	3	34	6	4	19	5	0.004955	0.23359	10	6	32	3	8	18	0.09791732	1	12.25	13.013729								
12	2E+07	12_24464456	69	26	48	74	48	61	0.917298	1	17	8	4	5	13	5	0.22538616	1	31.375	28.6253509								
12	2E+07	12_24486785	3	16	9	3	10	6	0.41703	1	1	7	8	13	4	11	0.14780855	1	7.5	5.18238776								
12	2E+07	12_24547015	23	37	17	36	30	27	0.605646	1	70	37	26	21	54	24	0.00913094	0.3646829	33.375	16.7241271								
12	2E+07	12_24610867	541	168	85	49	355	67	2.78E-54	#######	203	126	64	19	165	42	1.17E-14	5.82E-12	156.88	166.98797								
12	2E+07	12_24713090	494	182	370	285	338	328	0.049805	1	231	180	84	74	206	79	4.96E-11	1.72E-08	237.5	142.592326					2.5E+07	2.5E+07	0.16845	ID=promoter_12378;
12	2E+07	12_24766531	24	12	14	15	18	15	0.617094	1	63	5	18	4	34	11	0.0064318	0.2773637	19.375	18.7916205								
12	2E+07	12_24852930	18	63	71	47	41	59	0.60213	1	7	46	70	24	27	47	0.02965987	0.9232309	43.25	24.5516947								
12	2E+07	12_24937272	5	102	12	38	54	25	3.59E-04	0.02701	7	36	39	34	22	37	0.08041438	1	34.125	30.9904939								
12	3E+07	12_25452492	3	26	4	4	15	4	0.015825	0.58672	6	13	19	1	10	10	0.98574881	1	9.5	8.96022959								
12	3E+07	12_26800979	3	15	10	5	9	8	0.848833	1	25	0	26	14	13	20	0.35609139	1	12.25	9.67692395								
12	3E+07	12_28127914	1	10	0	7	6	4	0.674789	1	21	1	6	42	11	24	0.05309113	1	11	14.2628388								
12	3E+07	12_28408283	13	33	5	17	23	11	0.040732	1	46	17	19	32	32	26	0.8825541	1	22.75	13.2098448								
12	3E+07	12_28749985	27	59	42	28	43	35	0.271897	1	75	56	68	4	66	36	0.0349479	1	44.875	24.0501708								
12	3E+07	12_29149999	17	52	28	4	35	16	0.005267	0.24489	24	62	58	5	43	32	0.64107892	1	31.25	23.2670828								
12	3E+07	12_29267516	3	14	9	0	9	5	0.390586	1	55	23	7	1	39	4	2.33E-06	0.0003543	14	18.2130252	3E+07	2.9E+07	ID=ENSP00000262065;MMD;NM_012329;	MMD				
12	3E+07	12_29295445	18	10	1	0	14	1	0.001161	0.07164	62	5	15	4	34	10	0.00342551	0.1709558	14.375	20.2903038								
12	3E+07	12_29307136	12	2	28	13	7	21	0.082897	1	0	0	8	12	0	10	0.00454609	0.2107253	9.375	9.30341719								
12	3E+07	12_30170089	68	164	71	58	116	65	6.84E-06	0.00085	152	137	199	77	145	138	0.97588803	1	115.75	53.6436656								
12	3E+07	12_30727599	167	56	169	226	112	198	0.002233	0.12265	173	36	44	0	105	22	3.40E-11	1.20E-08	108.88	83.6599196	3E+07	3.1E+07	ID=ENSP00000328181;NOG;NM_005450;	NOG	3.1E+07	3.1E+07	0.72222	ID=promoter_12419;
12	3E+07	12_31196457	4	0	7	14	2	11	0.085797	1	12	4	4	0	8	2	0.20550608	1	5.625	5.12521777								
12	3E+07	12_31303985	418	239	495	373	329	434	0.274591	1	249	148	188	121	199	155	0.2660008	1	278.88	135.076103								
12	3E+07	12_31370227	117	47	77	77	82	77	0.372886	1	102	73	56	22	88	39	4.53E-04	0.0326835	71.375	30.1280007								
12	3E+07	12_31383598	9	42	23	60	26	42	0.436267	1	20	27	50	1	24	26	0.85918702	1	29	20.2696113								
12	3E+07	12_31556586	0	0	6	36	0	21	1.30E-04	0.01126	0	0	1	0	0	1	0.58312783	1	5.375	12.5463427								
12	3E+07	12_31581375	3	0	19	3	2	11	0.085797	1	42	1	39	2	22	21	0.99541892	1	13.625	17.6630162								
12	3E+07	12_31678286	78	41	48	30	60	39	0.014419	0.54423	69	14	10	37	42	24	0.16154149	1	40.875	23.9906976								
12	3E+07	12_31813813	41	77	18	29	59	24	2.77E-05	0.00289	89	21	45	42	55	44	0.76644609	1	45.25	25.482487								
12	3E+07	12_31996165	3	8	1	14	6	8	0.9722	1	28	5	10	0	17	5	0.05852787	1	8.625	9.13294664								
12	3E+07	12_32035067	395	136	392	264	266	328	0.832284	1	188	150	148	79	169	114	0.03111522	0.9564522	219	119.631577								
12	3E+07	12_32137847	391	143	339	272	267	306	0.962388	1	199	155	145	58	177	102	5.80E-04	0.0399489	212.75	112.217582								

12	3E+07	12_32268561	10	6	4	5	8	5	0.532535	1	10	11	9	3	11	6	0.57179039	1	7.25	3.10529502								
12	3E+07	12_32535278	1	30	23	3	16	13	0.613293	1	25	12	12	4	19	8	0.15782218	1	13.75	11.055057								
12	3E+07	12_32622804	171	68	193	112	120	153	0.791249	1	45	61	66	34	53	50	0.99607449	1	93.75	59.2856282	3E+07	3.3E+07	ID=ENSP0000347929;BZRAP1;;	BZRAP1	3.3E+07	3.3E+07	0.35237	ID=promoter_12519;
12	3E+07	12_32748976	626	269	472	283	448	378	1.33E-05	0.00154	102	127	146	13	115	80	0.14106829	1	254.75	205.616668								
12	3E+07	12_32778208	18	27	24	26	23	25	0.965853	1	13	1	20	8	7	14	0.23485029	1	17.125	9.20306626	3E+07	3.3E+07	ID=ENSP0000325285;MTMR4;NM_004687;	MTMR4				
12	3E+07	12_32927976	6	26	12	1	16	7	0.076817	1	1	0	16	4	1	10	0.01766414	0.6154554	8.25	9.1456469	3E+07	3.3E+07	ID=ENSP0000240361;TEX14;;	TEX14				
12	3E+07	12_33568846	61	18	21	7	40	14	1.93E-04	0.01576	2	0	13	0	1	7	0.08320466	1	15.25	20.1689294					3.4E+07	3.4E+07	0.31085	ID=promoter_12538;
12	3E+07	12_33918012	266	155	243	246	211	245	0.994206	1	124	152	66	13	138	40	6.69E-11	2.27E-08	158.13	90.492995	3E+07	3.4E+07	ID=ENSP0000251241;DHX40;NM_024612;	DHX40	3.4E+07	3.4E+07	0.41109	ID=promoter_12542;
12	4E+07	12_35173662	9	24	32	4	17	18	0.955187	1	29	8	8	2	19	5	0.02799778	0.8875672	14.5	11.8803559								
12	4E+07	12_35193293	0	27	2	1	14	2	0.003923	0.19308	0	0	21	2	0	12	0.00154584	0.0883233	6.625	10.8751026								
12	4E+07	12_35371020	8	5	25	19	7	22	0.061117	1	1	0	1	0	1	1	0.99856566	1	7.375	9.57582522								
12	4E+07	12_36162242	2	17	3	5	10	4	0.162164	1	0	7	12	2	4	7	0.58783259	1	6	5.80640041	4E+07	3.6E+07	ID=ENSP00000367577-D2;;NM_001033580;					
12	4E+07	12_36515750	19	0	8	27	10	18	0.549586	1	0	0	9	1	0	5	0.06742467	1	8	10.1699838					3.7E+07	3.7E+07	0.09111	ID=promoter_12588;
12	4E+07	12_36549478	1397	554	1108	1118	976	1113	0.813516	1	711	412	502	271	562	387	3.70E-05	0.0039104	759.13	401.186967					3.7E+07	3.7E+07	0.1519	ID=promoter_12591;
12	4E+07	12_36594412	17	17	21	5	17	13	0.50439	1	13	8	2	1	11	2	0.06154634	1	10.5	7.55928946								
12	4E+07	12_36701452	147	32	61	72	90	67	0.017628	0.63818	1	2	16	49	2	33	3.22E-07	6.07E-05	47.5	48.0327269								
12	4E+07	12_36815666	27	14	24	21	21	23	0.974526	1	35	32	41	20	34	31	0.99776322	1	26.75	8.84388409	4E+07	3.7E+07	ID=ENSP0000225402;AATF;NM_012138;	AATF				
12	4E+07	12_36851014	1	17	6	2	9	4	0.244237	1	0	4	21	17	2	19	5.26E-04	0.0367435	8.5	8.43462253	4E+07	3.7E+07	ID=ENSP0000225402;AATF;NM_012138;	AATF				
12	4E+07	12_36874884	3	23	14	4	13	9	0.46931	1	18	15	22	33	17	28	0.16600816	1	16.5	9.95705062					3.7E+07	3.7E+07	0.16197	ID=promoter_12612;
12	4E+07	12_36920681	89	15	3	2	52	3	4.78E-12	#######	12	23	15	0	18	8	0.20901227	1	19.875	29.0243125								
12	4E+07	12_36989887	5	6	7	8	6	8	0.9722	1	71	29	84	4	50	44	0.96875279	1	26.75	32.5214215								
12	4E+07	12_36998056	4	5	9	4	5	7	0.955252	1	12	1	3	4	7	4	0.73798468	1	5.25	3.53553391								
12	4E+07	12_37157182	11	20	21	9	16	15	0.822707	1	11	1	12	14	6	13	0.20797135	1	12.375	6.32314343								
12	4E+07	12_37437304	6	0	2	1	3	2	0.821455	1	1	11	26	9	6	18	0.03091566	0.954015	7	8.68496237	4E+07	3.7E+07	ID=ENSP0000309560;C17orf66;NM_152781;	C17orf66				

12	4E+07	12_37542609	37	0	32	44	19	38	0.157767	1	22	0	15	11	11	13	0.83819512	1	20.125	16.5567207					3.8E+07	3.8E+07	0.33454	ID=promoter_12625;
12	4E+07	12_37560481	20	46	21	27	33	24	0.19836	1	51	34	37	31	43	34	0.78602279	1	33.375	11.0961705	4E+07	3.8E+07	ID=ENSP00000340652;MMP28;NM_024302;	MMP28				
12	4E+07	12_37588801	15	0	7	2	8	5	0.532535	1	1	0	13	10	1	12	0.00617711	0.2683255	6	6.094494	4E+07	3.8E+07	ID=ENSP00000268864;RASL10B;NM_033315;	RASL10B	3.8E+07	3.8E+07	0.39004	ID=promoter_12630;
12	4E+07	12_37789663	32	43	61	58	38	60	0.355118	1	37	17	24	18	27	21	0.83317938	1	36.25	16.9178688	4E+07	3.8E+07	ID=ENSP00000378067;SLFN11;;	SLFN11				
12	4E+07	12_37818849	6	10	11	18	8	15	0.557319	1	14	9	8	27	12	18	0.42746936	1	12.875	6.81254096								
12	4E+07	12_37952254	12	0	6	0	6	3	0.462004	1	0	35	22	13	18	18	0.97449422	1	11	12.478553								
12	4E+07	12_38029122	26	76	67	168	51	118	1.96E-04	0.01596	191	156	316	98	174	207	0.05203867	1	137.25	91.6059106								
12	4E+07	12_38030684	19	0	17	34	10	26	0.089693	1	22	0	28	3	11	16	0.51039395	1	15.375	13.0486726								
12	4E+07	12_38116468	2	9	4	4	6	4	0.674789	1	3	33	30	0	18	15	0.95456211	1	10.625	13.1576974					3.8E+07	3.8E+07	0.15391	ID=promoter_12641;
12	4E+07	12_38123015	61	79	59	72	70	66	0.443462	1	5	17	31	25	11	28	0.01248827	0.4699819	43.625	27.5210958					3.8E+07	3.8E+07	0.1313	ID=promoter_12642;
12	4E+07	12_38148899	69	88	33	59	79	46	5.96E-04	0.04099	47	74	33	25	61	29	0.01058397	0.410878	53.5	22.6021491								
12	4E+07	12_38208064	164	57	135	181	111	158	0.293794	1	47	47	36	35	47	36	0.68902234	1	87.75	61.4927406								
12	4E+07	12_38237556	31	0	17	13	16	15	0.822707	1	0	0	14	0	0	7	0.02292696	0.7568963	9.375	11.4134945								
12	4E+07	12_38335666	10	0	0	0	5	0	0.05327	1	15	13	1	28	14	15	0.92682316	1	8.375	10.1268737								
12	4E+07	12_38337533	8	16	7	0	12	4	0.066899	1	12	0	20	5	6	13	0.20797135	1	8.5	7.17137166								
12	4E+07	12_38442828	6	9	17	16	8	17	0.371907	1	48	6	28	1	27	15	0.27474073	1	16.375	15.3151419								
12	4E+07	12_38446333	25	0	44	59	13	52	1.42E-04	0.01206	0	10	12	0	5	6	0.91271846	1	18.75	22.2758678								
12	4E+07	12_38743462	1	11	7	8	6	8	0.9722	1	41	6	20	3	24	12	0.20726737	1	12.125	13.0102981								
12	4E+07	12_38747075	7	9	23	7	8	15	0.557319	1	1	14	26	1	8	14	0.34554715	1	11	9.36559052								
12	4E+07	12_38767241	87	36	62	41	62	52	0.200726	1	77	65	43	42	71	43	0.08511339	1	56.625	18.920416								
12	4E+07	12_38781258	4	17	29	13	11	21	0.417506	1	22	19	44	0	21	22	0.92273004	1	18.5	13.9283883								
12	4E+07	12_38899395	10	54	18	8	32	13	0.003339	0.17064	47	23	19	42	35	31	0.98304591	1	27.625	17.5738239								
12	4E+07	12_38923923	0	1	0	1	1	1	0.993645	1	4	25	16	0	15	8	0.44137572	1	5.875	9.43303466								
12	4E+07	12_39131548	10	68	24	17	39	21	0.012903	0.49818	8	11	41	35	10	38	9.38E-05	0.0087137	26.75	20.5478536								
12	4E+07	12_39139941	39	24	45	52	32	49	0.500668	1	16	37	18	0	27	9	0.02128286	0.7187388	28.875	17.3406006								
12	4E+07	12_39149200	10	8	8	8	9	8	0.848833	1	50	30	11	52	40	32	0.82411274	1	22.125	19.2757249								
12	4E+07	12_39223251	4	34	17	6	19	12	0.233425	1	15	54	20	0	35	10	0.00238425	0.1270261	18.75	17.8305516								
12	4E+07	12_39299512	68	24	60	90	46	75	0.210106	1	91	78	59	0	85	30	1.41E-05	0.0017119	58.75	31.9228982								
12	4E+07	12_39417443	2	0	2	21	1	12	0.021411	0.74203	1	26	12	1	14	7	0.39930956	1	8.125	10.3016989								
12	4E+07	12_39436191	6	0	33	5	3	19	0.009621	0.39464	12	2	6	0	7	3	0.51857467	1	8	10.8364464								
12	4E+07	12_39530657	1	27	13	7	14	10	0.480768	1	0	21	22	4	11	13	0.83819512	1	11.875	10.4257579								
12	4E+07	12_40402354	36	5	18	4	21	11	0.084122	1	25	10	16	25	18	21	0.77315138	1	17.375	11.0316104								

12	4E+07	12_40479992	6	19	0	4	13	2	0.006819	0.29841	0	9	19	1	5	10	0.35527535	1	7.25	7.88760329	4E+07	4E+07	ID=ENSP00000351132;RHOT1;NM_001033568;	RHOT1				
12	4E+07	12_40719216	2	13	8	9	8	9	0.996316	1	52	6	17	17	29	17	0.3180598	1	15.5	15.6479118								
12	4E+07	12_40906853	0	6	9	10	3	10	0.252243	1	9	24	18	11	17	15	0.99031847	1	10.875	7.29848321								
12	4E+07	12_40968009	9	0	19	2	5	11	0.495311	1	0	0	14	46	0	30	9.40E-08	1.96E-05	11.25	15.7910463								
12	4E+07	12_41038346	134	19	105	83	77	94	0.966759	1	97	87	35	25	92	30	1.35E-06	0.000219	73.125	41.8856863								
12	4E+07	12_41098629	118	83	74	118	101	96	0.335424	1	55	48	67	14	52	41	0.73995351	1	72.125	35.0731379								
12	4E+07	12_41160335	19	60	33	23	40	28	0.10608	1	78	45	58	36	62	47	0.58093458	1	44	20.2131499	4E+07	4.1E+07	ID=ENSP00000378227;RAB11FIP4;;	RAB11FIP4				
12	4E+07	12_41196963	22	43	16	15	33	16	0.011269	0.44898	13	9	40	2	11	21	0.13989775	1	20	14.4815153	4E+07	4.1E+07	ID=ENSP00000378227;RAB11FIP4;;	RAB11FIP4				
12	4E+07	12_41251500	40	0	31	40	20	36	0.302044	1	56	33	21	0	45	11	1.13E-04	0.0101411	27.625	19.7190082	4E+07	4.1E+07	ID=ENSP00000378227;RAB11FIP4;;	RAB11FIP4				
12	4E+07	12_41642855	7	14	8	8	11	8	0.5832	1	14	4	47	4	9	26	0.0081957	0.3355909	13.25	14.1698876								
12	4E+07	12_41694967	11	41	1	8	26	5	1.31E-04	0.01132	1	0	5	1	1	3	0.56022579	1	8.5	13.7113092								
12	4E+07	12_42054183	15	11	5	24	13	15	0.999061	1	20	0	7	1	10	4	0.34296513	1	10.375	8.74948978								
12	4E+07	12_42078164	14	11	24	13	13	19	0.82904	1	17	5	9	0	11	5	0.40227598	1	11.625	7.3277847					4.2E+07	4.2E+07	0.12977	ID=promoter_12778;
12	4E+07	12_42170315	131	41	71	105	86	88	0.666283	1	115	17	75	55	66	65	0.94183458	1	76.25	38.839413								
12	4E+07	12_42182405	453	215	202	186	334	194	1.93E-14	#######	66	110	46	1	88	24	1.16E-07	2.38E-05	159.88	141.888727								
12	4E+07	12_42858574	12	33	17	30	23	24	0.922719	1	15	22	14	9	19	12	0.57792516	1	19	8.61891607								
12	4E+07	12_42920038	12	19	9	17	16	13	0.613293	1	1	26	22	2	14	12	0.98032967	1	13.5	9.11827051	4E+07	4.3E+07	ID=ENSP00000312942;SEZ6;;	SEZ6				
12	4E+07	12_43059685	1	12	3	13	7	8	0.998739	1	0	17	10	1	9	6	0.8208167	1	7.125	6.6211242					4.3E+07	4.3E+07	0.19929	ID=promoter_12820;
12	4E+07	12_43441313	6	5	6	4	6	5	0.851367	1	13	13	4	10	13	7	0.50249747	1	7.625	3.81491434								
12	4E+07	12_43638486	26	8	13	20	17	17	0.897282	1	1	0	2	0	1	1	0.99856566	1	8.75	10.0107086	4E+07	4.4E+07	ID=ENSP00000378338-D2;GIT1;;	GIT1				
12	4E+07	12_44149634	24	65	68	39	45	54	0.993642	1	51	19	25	7	35	16	0.05723775	1	37.25	22.3015054								
12	4E+07	12_44253985	290	36	175	147	163	161	0.301417	1	61	64	77	67	63	72	0.47781125	1	114.63	85.1501825					4.4E+07	4.4E+07	0.44205	ID=promoter_12844;
12	4E+07	12_44254431	234	55	235	175	145	205	0.226437	1	84	41	39	33	63	36	0.06492712	1	112	88.3386342					4.4E+07	4.4E+07	0.44205	ID=promoter_12844;
12	4E+07	12_44539257	34	124	26	39	79	33	1.31E-06	0.00019	88	56	25	48	72	37	0.01254306	0.4715466	55	34.5129375								
12	4E+07	12_44729099	6	6	13	20	6	17	0.158911	1	13	17	15	2	15	9	0.58227053	1	11.5	6.21059003								
12	4E+07	12_44733863	2	38	30	11	20	21	0.939065	1	55	30	44	35	43	40	0.99987783	1	30.625	17.1208937								
12	4E+07	12_44917583	259	85	275	169	172	222	0.641635	1	258	245	214	155	252	185	0.05409824	1	207.5	65.9566958								
12	4E+07	12_44978998	311	179	229	170	245	200	6.67E-04	0.04514	212	65	144	16	139	80	0.00279709	0.1430946	165.75	92.9512161								
12	5E+07	12_45006014	43	89	49	82	66	66	0.656529	1	58	107	125	42	83	84	0.85149188	1	74.375	31.2681376								

12	5E+07	12_45026420	0	23	13	2	12	8	0.45534	1	1	9	7	16	5	12	0.17891578	1	8.875	8.09651601	5E+07	4.5E+07	ID=ENSP00000321706;GEMIN4;NM_015721;	GEMIN4	4.5E+07	4.5E+07	0.53132	ID=promoter_12885;
12	5E+07	12_45027158	18	36	18	2	27	10	0.004472	0.21437	0	3	17	1	2	9	0.08177705	1	11.875	12.6427563	5E+07	4.5E+07	ID=ENSP00000321706;GEMIN4;NM_015721;	GEMIN4	4.5E+07	4.5E+07	0.53132	ID=promoter_12885;
12	5E+07	12_45076682	639	623	979	759	631	869	0.008922	0.37269	102	181	127	69	142	98	0.07861646	1	434.88	354.98428	5E+07	4.5E+07	ID=ENSP00000373692;VPS53;;	VPS53				
12	5E+07	12_45095062	0	36	17	24	18	21	0.99986	1	0	10	5	12	5	9	0.48000906	1	13	12.4096736	5E+07	4.5E+07	ID=ENSP00000373692;VPS53;;	VPS53	4.5E+07	4.5E+07	0.12391	ID=promoter_12892;
12	5E+07	12_45280310	81	14	30	35	48	33	0.057413	1	1	1	19	0	1	10	0.01766414	0.6154554	22.625	27.1237245								
12	5E+07	12_45286651	4	32	3	10	18	7	0.033499	1	11	1	18	4	6	11	0.39136385	1	10.375	10.3501208								
12	5E+07	12_45328585	287	65	187	180	176	184	0.550874	1	62	63	54	46	63	50	0.7138487	1	118	88.9751169								
12	5E+07	12_45383686	4	18	17	10	11	14	0.979671	1	1	0	19	7	1	13	0.0036419	0.1775497	9.5	7.72750191	5E+07	4.5E+07	ID=ENSP00000304360;SMYD4;NM_052928;	SMYD4	4.5E+07	4.5E+07	0.12169	ID=promoter_12929;
12	5E+07	12_45465770	10	46	2	23	28	13	0.016679	0.61094	28	11	34	23	20	29	0.29996038	1	22.125	14.2572037	5E+07	4.5E+07	ID=ENSP00000254719;RPA1;NM_002945;	RPA1	4.5E+07	4.5E+07	0.15906	ID=promoter_12933;
12	5E+07	12_45486426	13	7	23	30	10	27	0.068117	1	23	11	28	1	17	15	0.99031847	1	17	10.4880885					4.5E+07	4.5E+07	0.25271	ID=promoter_12939;
12	5E+07	12_45554448	134	78	131	104	106	118	0.926513	1	24	8	51	23	16	37	0.00673411	0.288397	69.125	50.0868888								
12	5E+07	12_45560224	46	34	55	46	40	51	0.92492	1	233	146	144	21	190	83	3.61E-08	8.21E-06	90.625	75.111227					4.6E+07	4.6E+07	0.30525	ID=promoter_12946;
12	5E+07	12_45578229	171	46	106	111	109	109	0.499105	1	46	90	60	12	68	36	0.02256057	0.7526317	80.25	49.7902744					4.6E+07	4.6E+07	0.66541	ID=promoter_12947;
12	5E+07	12_45587642	3	11	6	15	7	11	0.831726	1	4	0	0	3	2	2	0.99713337	1	5.25	5.28474895	5E+07	4.6E+07	ID=ENSP00000263083;DPH1;NM_001383;	DPH1				
12	5E+07	12_45600932	585	209	437	362	397	400	0.099654	1	230	180	142	88	205	115	7.55E-05	0.0072204	279.13	168.115978					4.6E+07	4.6E+07	0.58757	ID=promoter_12950;
12	5E+07	12_45616367	22	52	47	29	37	38	0.847307	1	7	10	10	8	9	9	0.98716474	1	23.125	18.0193349								
12	5E+07	12_45628995	463	185	504	432	324	468	0.015537	0.57909	173	173	189	99	173	144	0.63354898	1	277.25	160.211244								
12	5E+07	12_45638373	155	61	146	65	108	106	0.426648	1	100	39	8	2	70	5	6.26E-12	2.38E-09	72	57.8223882								
12	5E+07	12_45640089	19	0	12	19	10	16	0.741563	1	0	0	11	0	0	6	0.0393172	1	7.625	8.634441								
12	5E+07	12_45653299	16	25	46	21	21	34	0.506531	1	18	20	8	16	19	12	0.57792516	1	21.25	11.1451463								
12	5E+07	12_45660959	157	78	122	132	118	127	0.797354	1	0	39	3	0	20	2	0.00122165	0.0738454	66.375	64.7256573								
12	5E+07	12_45998774	1053	774	637	640	914	639	4.38E-23	#######	499	335	219	192	417	206	5.49E-13	2.35E-10	543.63	294.006772								
12	5E+07	12_46151547	69	39	28	34	54	31	0.005274	0.24515	12	12	4	1	12	3	0.09316162	1	24.875	22.6049141	5E+07	4.6E+07	ID=ENSP00000320468;KIAA0664;;	KIAA0664				
12	5E+07	12_46321049	19	5	18	10	12	14	0.999907	1	1	0	25	1	1	13	0.0036419	0.1775497	9.875	9.68706796								
12	5E+07	12_46398428	12	5	1	36	9	19	0.337786	1	2	0	14	4	1	9	0.02974549	0.9232309	9.25	11.9373364								
12	5E+07	12_46455053	217	56	131	89	137	110	0.011852	0.46288	6	35	14	1	21	8	0.08664698	1	68.625	74.727959								

12	5E+07	12_46994984	60	75	80	104	68	92	0.670931	1	17	10	14	43	14	29	0.03997116	1	50.375	34.997704	5E+07	4.7E+07	ID=ENSP000003826 59;;;					
12	5E+07	12_47019733	597	291	543	710	444	627	0.011224	0.44828	156	71	193	43	114	118	0.70279554	1	325.5	256.601971	5E+07	4.7E+07	ID=ENSP000002255 19;SHPK;NM_01327 6;	SHPK				
12	5E+07	12_47047617	96	1	82	62	49	72	0.475553	1	45	9	22	22	27	22	0.90387209	1	42.375	34.8340453								
12	5E+07	12_47077062	19	6	35	30	13	33	0.053925	1	33	37	54	32	35	43	0.46295168	1	30.75	13.9155617								
12	5E+07	12_47128061	19	46	16	14	33	15	0.006796	0.29841	10	2	6	10	6	8	0.79537884	1	15.375	13.5112387	5E+07	4.7E+07	ID=ENSP000002630 87;ITGAE;NM_0022 08;	ITGAE				
12	5E+07	12_47323590	74	98	143	154	86	149	0.014977	0.56219	77	62	91	102	70	97	0.0358187	1	100.13	32.7389567								
12	5E+07	12_47390356	4	74	12	4	39	8	2.44E-06	0.00034	26	49	46	32	38	39	0.90512872	1	30.875	24.619606								
12	5E+07	12_47405506	26	32	7	0	29	4	8.83E-06	0.00107	31	37	46	2	34	24	0.59108388	1	22.625	17.3364809								
12	5E+07	12_47654606	24	10	22	8	17	15	0.722447	1	27	40	40	35	34	38	0.73008239	1	25.75	12.3837451								
12	5E+07	12_47752067	310	98	182	230	204	206	0.316098	1	90	36	109	56	63	83	0.10700487	1	138.88	93.9991451	5E+07	4.8E+07	ID=ENSP000002547 18;MYBBP1A;NM_01 4520;	MYBBP1A	4.8E+07	4.8E+07	0.36749	ID=promoter_13033;
12	5E+07	12_47827282	15	59	27	26	37	27	0.166749	1	51	52	72	16	52	44	0.90537885	1	39.75	21.4326187					4.8E+07	4.8E+07	0.11258	ID=promoter_13036;
12	5E+07	12_47880420	4	1	24	5	3	15	0.044084	1	0	0	8	3	0	6	0.0393172	1	5.625	7.90908158								
12	5E+07	12_47886866	4	30	1	1	17	1	2.04E-04	0.01649	11	0	8	1	6	5	0.98461866	1	7	10.0853501	5E+07	4.8E+07	ID=ENSP000003213 86;FBXO39;NM_153 230;	FBXO39	4.8E+07	4.8E+07	0.14324	ID=promoter_13040;
12	5E+07	12_47975339	219	171	137	81	195	109	1.87E-09	#######	0	15	52	20	8	36	4.47E-05	0.0046182	86.875	80.6074039					4.8E+07	4.8E+07	0.59046	ID=promoter_13044;
12	5E+07	12_47975935	2407	1582	2607	2250	1995	2429	0.466505	1	1267	1087	1310	516	1177	913	2.78E-04	0.021655	1628.3	728.496642					4.8E+07	4.8E+07	0.59046	ID=promoter_13044;
12	5E+07	12_47993745	0	6	3	6	3	5	0.88968	1	0	3	15	27	2	21	1.85E-04	0.0153843	7.5	9.21179368					4.8E+07	4.8E+07	0.17424	ID=promoter_13046;
12	5E+07	12_48073223	19	61	17	63	40	40	0.774898	1	38	17	33	2	28	18	0.47597237	1	31.25	21.8877004								
12	5E+07	12_48107920	44	37	45	25	41	35	0.382421	1	17	40	55	10	29	33	0.72226159	1	34.125	15.3663594	5E+07	4.8E+07	ID=ENSP000002624 83;PITPNM3;NM_03 1220;	PITPNM3				
12	5E+07	12_48287063	7	3	21	32	5	27	0.002605	0.1394	1	8	35	1	5	18	0.01516798	0.5480081	13.5	13.918128								
12	5E+07	12_48428357	11	1	2	15	6	9	0.896349	1	30	3	3	13	17	8	0.27279187	1	9.75	9.83797308	5E+07	4.8E+07	ID=ENSP000003230 87;WSCD1;NM_0152 53;	WSCD1	4.8E+07	4.8E+07	0.22938	ID=promoter_13055;
12	5E+07	12_48443942	220	37	199	230	129	215	0.006562	0.29117	26	35	45	2	31	24	0.80238053	1	99.25	98.1220086	5E+07	4.8E+07	ID=ENSP000003230 87;WSCD1;NM_0152 53;	WSCD1				
12	5E+07	12_48454455	9	24	10	18	17	14	0.615566	1	3	32	7	13	18	10	0.42261869	1	14.5	9.63624112	5E+07	4.8E+07	ID=ENSP000003230 87;WSCD1;NM_0152 53;	WSCD1				
12	5E+07	12_48470295	49	31	75	47	40	61	0.43304	1	1	23	12	0	12	6	0.45526626	1	29.75	26.15749								
12	5E+07	12_48575786	27	34	16	19	31	18	0.053237	1	27	13	31	24	20	28	0.36590545	1	23.875	7.33752586								

12	5E+07	12_49034128	14	1	30	3	8	17	0.371907	1	2	5	5	2	4	4	0.99427496	1	7.75	9.88144003								
12	5E+07	12_49088119	6	0	7	8	3	8	0.459247	1	26	13	13	10	20	12	0.48622029	1	10.375	7.57698583					4.9E+07	4.9E+07	0.13391	ID=promoter_13077;
12	5E+07	12_49139377	6	9	7	14	8	11	0.943069	1	50	19	12	9	35	11	0.00454855	0.2107253	15.75	14.4593025								
12	5E+07	12_49144296	186	88	111	132	137	122	0.085069	1	116	78	85	23	97	54	0.00995783	0.3918263	102.38	47.1075896					4.9E+07	4.9E+07	0.4675	ID=promoter_13087;
12	5E+07	12_49156722	963	361	695	765	662	730	0.515807	1	315	216	320	97	266	209	0.2009415	1	466.5	302.997407					4.9E+07	4.9E+07	0.75027	ID=promoter_13088;
12	5E+07	12_49260012	133	72	206	167	103	187	0.001598	0.09347	47	50	79	63	49	71	0.05287735	1	102.13	59.3787781								
12	5E+07	12_49443495	82	26	37	26	54	32	0.007687	0.33078	4	22	27	14	13	21	0.2807652	1	29.75	23.2670828								
12	5E+07	12_49483875	2	59	11	15	31	13	0.005055	0.23696	48	12	6	2	30	4	1.24E-04	0.0109901	19.375	21.7645682								
12	5E+07	12_49496297	513	114	341	564	314	453	0.018566	0.66539	176	164	197	109	170	153	0.96532163	1	272.25	179.739295	5E+07	5E+07	ID=ENSP00000251535-D2;ALOX12;NM_000697;	ALOX12	4.9E+07	4.9E+07	0.58528	ID=promoter_13123;
12	5E+07	12_49499174	31	16	14	51	24	33	0.838746	1	1	16	6	0	9	3	0.27712561	1	16.875	17.024667	5E+07	5E+07	ID=ENSP00000251535-D2;ALOX12;NM_000697;	ALOX12				
12	5E+07	12_49592558	54	34	39	11	44	25	0.012594	0.48919	37	44	45	12	41	29	0.53508799	1	34.5	15.4272486	5E+07	5E+07	ID=ENSP0000251535;ALOX12;NM_000697;	ALOX12				
12	5E+07	12_49748705	97	82	105	99	90	102	0.972164	1	25	50	22	19	38	21	0.15716135	1	62.375	37.4239705	5E+07	5E+07	ID=ENSP00000379869;C17orf81;NM_203415;	C17orf81	5E+07	5E+07	0.27806	ID=promoter_13138;
12	5E+07	12_49791033	45	14	44	21	30	33	0.968109	1	27	7	2	14	17	8	0.27279187	1	21.75	15.9977677					5E+07	5E+07	0.68087	ID=promoter_13148;
12	5E+07	12_49811016	398	140	726	743	269	735	3.59E-33	#######	152	112	218	61	132	140	0.53992001	1	318.75	275.476081					5E+07	5E+07	0.44056	ID=promoter_13152;
12	5E+07	12_49832657	33	40	13	4	37	9	1.73E-05	0.00192	16	54	62	7	35	35	0.95100306	1	28.625	21.9866031								
12	5E+07	12_49844976	185	48	146	144	117	145	0.906572	1	78	55	73	48	67	61	0.99480379	1	97.125	53.2606462					5E+07	5E+07	0.21489	ID=promoter_13156;
12	5E+07	12_49845638	179	191	67	138	185	103	4.43E-09	#######	37	73	72	54	55	63	0.5170734	1	101.38	59.3270295					5E+07	5E+07	0.21489	ID=promoter_13156;
12	5E+07	12_49911246	32	1	28	14	17	21	0.98748	1	1	10	1	27	6	14	0.1466131	1	14.25	13.1556615								
12	5E+07	12_49966948	184	64	131	139	124	135	0.835602	1	112	34	49	10	73	30	5.92E-04	0.0404825	90.375	60.1116521								
12	5E+07	12_50114157	23	22	10	5	23	8	0.007066	0.30636	14	10	2	23	12	13	0.92692342	1	13.625	8.29694436								
12	5E+07	12_50153813	256	134	235	202	195	219	0.907193	1	127	96	102	20	112	61	0.00328397	0.1647555	146.5	79.2572665					5E+07	5E+07	0.59765	ID=promoter_13201;
12	5E+07	12_50179185	18	6	12	8	12	10	0.724827	1	5	1	0	2	3	1	0.65196691	1	6.5	6.094494								
12	5E+07	12_50189711	15	31	60	21	23	41	0.269933	1	0	8	4	0	4	2	0.76928717	1	17.375	20.3254766								
12	5E+07	12_50205592	47	35	63	47	41	55	0.809005	1	45	11	12	20	28	16	0.29661245	1	35	18.9057814	5E+07	5E+07	ID=ENSP00000254854-D2;GUCY2D;NM_000180;	GUCY2D	5E+07	5E+07	0.43337	ID=promoter_13210;
12	5E+07	12_50299647	184	30	164	178	107	171	0.041853	1	84	35	45	25	60	35	0.08940859	1	93.125	70.5983357					5E+07	5E+07	0.59765	ID=promoter_13222;

12	5E+07	12_50356541	9	19	18	12	14	15	0.970741	1	9	14	20	4	12	12	0.98292303	1	13.125	5.66789202					5E+07	5E+07	0.69573	ID=promoter_13232;
12	5E+07	12_50458117	62	43	181	112	53	147	2.18E-07	#######	22	25	36	42	24	39	0.09051915	1	65.375	54.7407853					5E+07	5E+07	0.43337	ID=promoter_13249;
12	5E+07	12_50485943	9	3	3	7	6	5	0.851367	1	21	5	1	0	13	1	0.00904697	0.3617342	6.125	6.70687281								
12	5E+07	12_50543413	57	8	52	68	33	60	0.125172	1	0	21	15	31	11	23	0.07426989	1	31.5	24.8710962								
12	5E+07	12_50563762	799	295	608	764	547	686	0.505927	1	383	196	323	126	290	225	0.13357908	1	436.75	255.815642					5.1E+07	5.1E+07	0.69468	ID=promoter_13260;
12	5E+07	12_50566377	1916	1019	1877	1540	1468	1709	0.977931	1	1130	611	782	505	871	644	7.40E-05	0.0071008	1172.5	550.696442					5.1E+07	5.1E+07	0.37833	ID=promoter_13261;
12	5E+07	12_50613782	16	11	17	13	14	15	0.970741	1	13	1	35	3	7	19	0.03889743	1	13.625	10.377689								
12	5E+07	12_50703256	35	52	21	26	44	24	0.008407	0.35544	17	16	16	4	17	10	0.5154613	1	23.375	14.5988013					5.1E+07	5.1E+07	0.10879	ID=promoter_13276;
12	5E+07	12_50708391	13	12	5	1	13	3	0.018372	0.65917	19	24	0	0	22	0	3.73E-05	0.0039305	9.25	9.19238816					5.1E+07	5.1E+07	0.59292	ID=promoter_13279;
12	5E+07	12_50714738	5	26	2	6	16	4	0.009581	0.39335	27	50	26	50	39	38	0.97643104	1	24	19.0413085								
12	5E+07	12_50719613	76	58	92	59	67	76	0.980234	1	48	10	29	0	29	15	0.17706132	1	46.5	31.6949748					5.1E+07	5.1E+07	0.43995	ID=promoter_13282;
12	5E+07	12_50764301	4	53	6	4	29	5	2.61E-05	0.00275	1	5	25	8	3	17	0.00428697	0.2008879	13.25	17.6776695					5.1E+07	5.1E+07	0.18007	ID=promoter_13296;
12	5E+07	12_50780271	20	21	28	16	21	22	0.933629	1	1	1	6	2	1	4	0.36109265	1	11.875	10.6561518								
12	5E+07	12_50793795	402	123	393	273	263	333	0.650657	1	268	73	104	125	171	115	0.02808582	0.8893712	220.13	131.835869								
12	5E+07	12_50906489	8	5	6	8	7	7	0.956352	1	10	0	11	4	5	8	0.62517663	1	6.5	3.54562104								
12	5E+07	12_51101976	38	41	46	36	40	41	0.831668	1	37	21	20	17	29	19	0.49734269	1	32	10.9805022								
12	5E+07	12_51242533	30	50	52	61	40	57	0.639748	1	17	5	8	16	11	12	0.92663959	1	29.875	21.7744116								
12	5E+07	12_51271718	70	20	66	32	45	49	0.937165	1	38	18	11	13	28	12	0.0723178	1	33.5	23.1763426					5.1E+07	5.1E+07	0.90675	ID=promoter_13348;
12	5E+07	12_51282063	24	14	15	2	19	9	0.06806	1	9	0	10	0	5	5	0.99284883	1	9.25	8.43038721								
12	5E+07	12_51317934	15	82	28	27	49	28	0.008152	0.34652	17	75	68	36	46	52	0.61770074	1	43.5	27.1451127								
12	5E+07	12_51324896	22	33	14	4	28	9	0.001431	0.08503	2	12	30	5	7	18	0.05735679	1	15.25	11.9373364								
12	5E+07	12_51349355	14	1	1	0	8	1	0.03583	1	22	0	6	20	11	13	0.83819512	1	8	9.30437685								
12	5E+07	12_51371811	313	114	342	266	214	304	0.099209	1	175	98	152	37	137	95	0.09242867	1	187.13	109.0615					5.1E+07	5.1E+07	0.47381	ID=promoter_13364;
12	5E+07	12_51417560	20	8	24	10	14	17	0.995406	1	0	8	5	2	4	4	0.99427496	1	9.625	8.38259251								
12	5E+07	12_51463323	7	18	0	0	13	0	4.88E-04	0.03443	0	0	13	28	0	21	1.21E-05	0.0014894	8.25	10.5661184								
12	5E+07	12_51509623	111	138	27	30	125	29	2.34E-17	#######	32	69	40	2	51	21	0.00566618	0.2544931	56.125	46.5723324								
12	5E+07	12_51532536	34	51	20	16	43	18	6.41E-04	0.04369	48	50	41	21	49	31	0.24588806	1	35.125	14.4858305								
12	5E+07	12_51539145	560	57	5	25	309	15	1.06E-69	#######	83	7	41	14	45	28	0.24903664	1	99	188.189115					5.2E+07	5.2E+07	0.20763	ID=promoter_13391;
12	5E+07	12_51561654	37	35	16	50	36	33	0.591026	1	9	25	30	1	17	16	0.99905703	1	25.375	16.0618225					5.2E+07	5.2E+07	0.30166	ID=promoter_13398;
12	5E+07	12_51623313	0	22	2	3	11	3	0.050503	1	1	16	10	0	9	5	0.6500913	1	6.75	8.3623305								
12	5E+07	12_51663282	0	16	3	8	8	6	0.705675	1	0	10	28	1	5	15	0.05518429	1	8.25	9.77971662	5E+07	5.2E+07	ID=ENSP00000285199;USP43;NM_153210;	USP43				

12	5E+07	12_51670880	23	43	14	21	33	18	0.027763	0.91318	26	8	19	10	17	15	0.99031847	1	20.5	11.045361	5E+07	5.2E+07	ID=ENSP0000285199;USP43;NM_153210;	USP43	5.2E+07	5.2E+07	0.28255	ID=promoter_13413;
12	5E+07	12_51699454	7	27	17	6	17	12	0.395844	1	1	1	11	13	1	12	0.00617711	0.2683255	10.375	8.73314704	5E+07	5.2E+07	ID=ENSP0000327975;DHRS7C;NM_001105571;	DHRS7C				
12	5E+07	12_51743479	9	6	20	18	8	19	0.232241	1	43	22	33	1	33	17	0.1362937	1	19	14.0203933								
12	5E+07	12_51762804	40	64	24	33	52	29	0.004577	0.21855	66	36	49	24	51	37	0.52329888	1	42	16.3794296					5.2E+07	5.2E+07	0.28943	ID=promoter_13423;
12	5E+07	12_51781485	70	81	57	73	76	65	0.171631	1	139	60	78	33	100	56	0.0094873	0.3779644	73.875	30.4088216					5.2E+07	5.2E+07	0.16701	ID=promoter_13427;
12	5E+07	12_51801642	69	9	43	30	39	37	0.651187	1	52	42	8	48	47	28	0.17489115	1	37.625	21.0505344								
12	5E+07	12_51844527	269	90	17	9	180	13	2.21E-38	#######	22	109	17	16	66	17	3.09E-06	0.000453	68.625	89.4953111	5E+07	5.2E+07	ID=ENSP0000322608-D2;GAS7;NM_201433;	GAS7				
12	5E+07	12_51901438	12	26	24	19	19	22	0.999149	1	28	19	0	4	24	2	2.02E-04	0.0165855	16.5	10.2817453	5E+07	5.2E+07	ID=ENSP0000322608-D2;GAS7;NM_201433;	GAS7				
12	5E+07	12_52069800	5	15	1	20	10	11	0.989255	1	18	3	7	13	11	10	0.99899888	1	10.25	7.18629648								
12	5E+07	12_52163676	1	22	2	1	12	2	0.011798	0.46127	2	5	5	27	4	16	0.01700101	0.6008545	8.125	10.3155569								
12	5E+07	12_52351470	13	62	27	23	38	25	0.074872	1	89	60	39	30	75	35	0.00289268	0.1475089	42.875	25.3739884								
12	5E+07	12_52407726	34	1	16	15	18	16	0.720517	1	0	0	5	2	0	4	0.11562589	1	9.125	11.9575141								
12	5E+07	12_52429422	7	13	13	9	10	11	0.989255	1	21	33	27	12	27	20	0.74799297	1	16.875	9.20306626					5.2E+07	5.2E+07	0.46057	ID=promoter_13481;
12	5E+07	12_52493005	1081	395	874	725	738	800	0.302952	1	281	349	317	186	315	252	0.21796898	1	526	324.373242					5.2E+07	5.2E+07	0.49585	ID=promoter_13491;
12	5E+07	12_52566239	1	0	0	0	1	0	0.556284	1	0	24	23	0	12	12	0.98292303	1	6	10.8100482								
12	5E+07	12_52673100	2	25	5	1	14	3	0.010924	0.43763	25	5	5	27	15	16	0.92652531	1	11.875	11.5317884								
12	5E+07	12_52683814	3794	1959	4459	3729	2877	4094	1.80E-14	#######	2636	1552	1637	1034	2094	1336	9.66E-26	8.79E-23	2600	1256.2054								
12	5E+07	12_52710696	44	2	62	9	23	36	0.555384	1	0	3	0	0	2	0	0.39572182	1	15	24.1246762								
12	5E+07	12_52769230	500	123	355	300	312	328	0.382954	1	148	149	144	81	149	113	0.27253698	1	225	145.187563					5.3E+07	5.3E+07	0.55904	ID=promoter_13511;
12	5E+07	12_52784003	18	14	16	26	16	21	0.944185	1	45	5	10	17	25	14	0.3120942	1	18.875	12.1941145								
12	5E+07	12_52874433	1912	1460	2173	1921	1686	2047	0.576963	1	119	106	135	20	113	78	0.13245458	1	980.75	967.21777								
12	5E+07	12_52917857	0	24	22	16	12	19	0.716431	1	20	16	9	4	18	7	0.13207736	1	13.875	8.69215574								
12	5E+07	12_52969397	12	16	4	6	14	5	0.053576	1	0	0	6	0	0	3	0.19828566	1	5.5	5.92814112								
12	5E+07	12_52971908	1	47	14	31	24	23	0.786129	1	0	14	32	69	7	51	9.97E-09	2.46E-06	26	23.7246105					5.3E+07	5.3E+07	0.25901	ID=promoter_13518;
12	5E+07	12_53006182	40	27	41	31	34	36	0.912382	1	36	27	27	14	32	21	0.46573568	1	30.375	8.78208404								
12	5E+07	12_53010910	20	29	20	6	25	13	0.050329	1	34	36	60	3	35	32	0.99839619	1	26	18.2443729								
12	5E+07	12_53225895	10	25	5	9	18	7	0.033499	1	0	5	0	1	3	1	0.65196691	1	6.875	8.27107697								
12	5E+07	12_53228975	8	4	10	7	6	9	0.896349	1	0	0	19	5	0	12	0.00154584	0.0883233	6.625	6.13974173					5.3E+07	5.3E+07	0.17538	ID=promoter_13529;

12	5E+07	12_53274790	43	31	33	17	37	25	0.097534	1	62	28	25	21	45	23	0.06212307	1	32.5	14.3028469								
12	5E+07	12_53333454	3	21	9	3	12	6	0.213448	1	15	1	7	2	8	5	0.78486764	1	7.625	7.11010348	5E+07	5.3E+07	ID=ENSP00000374490;DNAH17;;	DNAH17	5.3E+07	5.3E+07	0.17655	ID=promoter_13540;
12	5E+07	12_53585834	23	23	26	23	23	25	0.965853	1	28	29	30	27	29	29	0.95922868	1	26.125	2.85043856					5.4E+07	5.4E+07	0.16185	ID=promoter_13578;
12	5E+07	12_53697304	20	11	1	11	16	6	0.042495	1	31	12	9	1	22	5	0.00878788	0.3541514	12	9.84160266								
12	5E+07	12_54006488	8	11	10	5	10	8	0.719353	1	36	15	30	9	26	20	0.8208633	1	15.5	11.2757641								
12	5E+07	12_54067142	13	26	2	10	20	6	0.006754	0.29841	23	12	29	8	18	19	0.92496372	1	15.375	9.53096457					5.4E+07	5.4E+07	0.11218	ID=promoter_13632;
12	5E+07	12_54080292	18	0	6	0	9	3	0.131542	1	36	0	0	0	18	0	2.38E-04	0.0190698	7.5	13.1257653								
12	5E+07	12_54163379	4	15	18	13	10	16	0.741563	1	16	13	40	10	15	25	0.19020812	1	16.125	10.548358								
12	5E+07	12_54165527	29	2	5	26	16	16	0.903021	1	20	22	13	0	21	7	0.05007015	1	14.625	11.2622949					5.4E+07	5.4E+07	0.2391	ID=promoter_13643;
12	5E+07	12_54183120	10	35	4	10	23	7	0.003444	0.1741	25	25	28	0	25	14	0.3120942	1	17.125	12.6991282								
12	5E+07	12_54191721	4	15	9	0	10	5	0.276106	1	0	7	5	0	4	3	0.96209564	1	5	5.29150262								
12	5E+07	12_54212125	23	28	33	18	26	26	0.847245	1	33	45	40	31	39	36	0.99982789	1	31.375	8.66746791								
12	5E+07	12_54213023	1	13	0	27	7	14	0.506445	1	0	1	1	1	1	1	0.99856566	1	5.5	9.71008312					5.4E+07	5.4E+07	0.28394	ID=promoter_13650;
12	5E+07	12_54240693	7	18	7	4	13	6	0.146758	1	16	0	0	1	8	1	0.0848225	1	6.625	7.00892288								
12	5E+07	12_54259599	22	5	39	7	14	23	0.607375	1	38	27	26	47	33	37	0.72881039	1	26.375	14.9851117								
12	5E+07	12_54260610	8	7	8	2	8	5	0.532535	1	0	12	5	11	6	8	0.79537884	1	6.625	4.13823634								
12	5E+07	12_54308465	17	61	21	38	39	30	0.216196	1	36	18	17	53	27	35	0.42118875	1	32.625	17.3117425	5E+07	5.4E+07	ID=ENSP00000262444;;;					
12	5E+07	12_54319706	48	34	20	57	41	39	0.643788	1	26	6	10	32	16	21	0.57439123	1	29.125	17.5616262	5E+07	5.4E+07	ID=ENSP00000262444;;;					
12	5E+07	12_54362851	0	16	2	0	8	1	0.03583	1	0	0	2	34	0	18	6.08E-05	0.0059624	6.75	12.2794835	5E+07	5.4E+07	ID=ENSP00000262444;;;		5.4E+07	5.4E+07	0.17343	ID=promoter_13666;
12	5E+07	12_54384563	117	79	100	228	98	164	0.019951	0.70581	23	86	45	4	55	25	0.01053466	0.4092977	85.25	69.4257054	5E+07	5.4E+07	ID=ENSP00000262444;;;					
12	5E+07	12_54397531	5	9	12	6	7	9	0.983538	1	0	1	0	9	1	5	0.22510075	1	5.25	4.59036258	5E+07	5.4E+07	ID=ENSP00000262444;;;		5.4E+07	5.4E+07	0.17317	ID=promoter_13675;
12	5E+07	12_54414711	41	65	185	170	53	178	1.77E-11	#######	29	21	71	9	25	40	0.09550227	1	73.875	67.3740889	5E+07	5.4E+07	ID=ENSP00000262444;;;					
12	5E+07	12_54512633	11	20	2	1	16	2	0.001284	0.07728	3	1	11	0	2	6	0.31385293	1	6.125	7.14017807								
12	5E+07	12_54517717	21	52	23	21	37	22	0.03657	1	15	12	14	55	14	35	0.00480639	0.2209499	26.625	17.045632					5.5E+07	5.5E+07	0.09948	ID=promoter_13693;
12	5E+07	12_54518240	74	38	52	70	56	61	0.923132	1	59	35	22	0	47	11	5.20E-05	0.0052176	43.75	25.0756								
12	5E+07	12_54534567	20	5	26	16	13	21	0.660505	1	62	27	20	25	45	23	0.06212307	1	25.125	16.4962117					5.5E+07	5.5E+07	0.11436	ID=promoter_13695;
12	5E+07	12_54563338	34	22	19	24	28	22	0.367037	1	55	21	12	9	38	11	0.0015633	0.0891068	24.5	14.4716472								
12	5E+07	12_54620726	2	21	10	21	12	16	0.945172	1	56	20	16	19	38	18	0.05735612	1	20.625	15.7474488					5.5E+07	5.5E+07	0.15625	ID=promoter_13710;
12	5E+07	12_54653375	19	14	5	3	17	4	0.005755	0.26328	19	8	46	5	14	26	0.10164664	1	14.875	14.0756883					5.5E+07	5.5E+07	0.17834	ID=promoter_13717;
12	5E+07	12_54654771	6	8	7	17	7	12	0.724589	1	14	13	19	1	14	10	0.81923661	1	10.625	6.11642986								

12	5E+07	12_54666581	586	846	976	959	716	968	0.015639	0.5824	141	167	202	203	154	203	0.00416289	0.1962976	510	374.23293					5.5E+07	5.5E+07	0.28584	ID=promoter_13718;
12	5E+07	12_54700629	0	9	4	0	5	2	0.402696	1	15	11	14	13	13	14	0.92696813	1	8.25	6.13537751					5.5E+07	5.5E+07	0.13517	ID=promoter_13724;
12	5E+07	12_54713710	31	18	25	6	25	16	0.157912	1	59	20	9	23	40	16	0.01383566	0.5095422	23.875	16.3745229					5.5E+07	5.5E+07	0.28975	ID=promoter_13730;
12	5E+07	12_54714793	7	18	30	15	13	23	0.490912	1	10	44	31	4	27	18	0.55301708	1	19.875	13.8918013					5.5E+07	5.5E+07	0.28975	ID=promoter_13730;
12	5E+07	12_54757208	21	49	14	8	35	11	2.32E-04	0.01845	17	41	44	51	29	48	0.0440188	1	30.625	17.3447192	5E+07	5.5E+07	ID=ENSP000028410;HS3ST3A1;NM_006042;	HS3ST3A1	5.5E+07	5.5E+07	0.3324	ID=promoter_13739;
12	5E+07	12_54804936	54	19	51	38	37	45	0.986769	1	11	11	4	11	11	8	0.87190251	1	24.875	19.8237772	5E+07	5.5E+07	ID=ENSP000028410;HS3ST3A1;NM_006042;	HS3ST3A1	5.5E+07	5.5E+07	0.16403	ID=promoter_13749;
12	5E+07	12_54814742	9	24	3	14	17	9	0.140533	1	18	28	39	6	23	23	0.96752545	1	17.625	12.1999707	5E+07	5.5E+07	ID=ENSP000028410;HS3ST3A1;NM_006042;	HS3ST3A1				
12	5E+07	12_54823292	1	20	17	7	11	12	0.985018	1	47	63	4	4	55	4	1.72E-09	4.80E-07	20.375	22.7654971	5E+07	5.5E+07	ID=ENSP000028410;HS3ST3A1;NM_006042;	HS3ST3A1	5.5E+07	5.5E+07	0.20909	ID=promoter_13754;
12	5E+07	12_54847800	1	22	3	4	12	4	0.066899	1	26	0	1	0	13	1	0.00904697	0.3617342	7.125	10.5618924								
12	6E+07	12_55009343	16	40	18	6	28	12	0.009759	0.39939	21	26	43	1	24	22	0.99927301	1	21.375	14.7738911					5.5E+07	5.5E+07	0.14904	ID=promoter_13794;
12	6E+07	12_55066996	69	51	51	18	60	35	0.00364	0.18261	39	7	51	1	23	26	0.7859394	1	35.875	24.3863984								
12	6E+07	12_55068408	7	13	4	9	10	7	0.570701	1	15	6	7	2	11	5	0.40227598	1	7.875	4.35685011					5.5E+07	5.5E+07	0.23801	ID=promoter_13811;
12	6E+07	12_55111016	1	24	1	18	13	10	0.600182	1	19	61	14	17	40	16	0.01383566	0.5095422	19.375	18.7688001								
12	6E+07	12_55274897	32	1	20	14	17	17	0.897282	1	3	1	1	0	2	1	0.87709017	1	9	11.8562822					5.5E+07	5.5E+07	0.14961	ID=promoter_13840;
12	6E+07	12_55321023	27	23	31	15	25	23	0.701069	1	21	12	25	15	17	20	0.769441	1	21.125	6.64266513								
12	6E+07	12_55356142	15	25	17	11	20	14	0.325699	1	0	9	15	16	5	16	0.03625394	1	13.5	7.21110255					5.5E+07	5.5E+07	0.24139	ID=promoter_13852;
12	6E+07	12_55361209	1	47	18	6	24	12	0.04556	1	0	15	8	2	8	5	0.78486764	1	12.125	15.5235995								
12	6E+07	12_55384210	10	46	3	2	28	3	4.80E-06	0.00062	7	19	20	26	13	23	0.16598565	1	16.625	14.657397								
12	6E+07	12_55457659	0	19	3	7	10	5	0.276106	1	0	2	10	0	1	5	0.22510075	1	5.125	6.68553876								
12	6E+07	12_55607026	0	17	11	3	9	7	0.713906	1	0	0	0	13	0	7	0.02292696	0.7568963	5.5	7.0305456								
12	6E+07	12_55643524	0	6	9	3	3	6	0.747087	1	34	0	10	0	17	5	0.05852787	1	7.75	11.3483794								
12	6E+07	12_55644959	70	17	18	0	44	9	4.55E-07	#######	29	22	8	1	26	5	0.00174818	0.0978457	20.625	22.3602805								
12	6E+07	12_55700522	1	8	16	26	5	21	0.023384	0.79837	22	1	19	42	12	31	0.00704867	0.2985552	16.875	13.7782178								
12	6E+07	12_55711094	21	2	8	14	12	11	0.839207	1	4	0	3	1	2	2	0.99713337	1	6.625	7.36667205								
12	6E+07	12_55784815	21	30	28	24	26	26	0.847245	1	34	28	36	18	31	27	0.97229028	1	27.375	6.16296543								
12	6E+07	12_55847776	11	7	10	6	9	8	0.848833	1	0	41	18	1	21	10	0.21021716	1	11.75	13.1339255								
12	6E+07	12_55865965	31	12	12	24	22	18	0.522468	1	26	8	15	2	17	9	0.38755874	1	16.25	9.86697232					5.6E+07	5.6E+07	0.18343	ID=promoter_13911;

12	6E+07	12_55866227	17	30	15	31	24	23	0.786129	1	68	21	27	6	45	17	0.00491937	0.2246966	26.875	18.6197859					5.6E+07	5.6E+07	0.18343	ID=promoter_13911;
12	6E+07	12_55884070	19	3	22	4	11	13	0.999831	1	7	0	0	4	4	2	0.76928717	1	7.375	8.45048604								
12	6E+07	12_55889867	44	159	52	53	102	53	4.96E-06	0.00064	103	14	49	47	59	48	0.79773703	1	65.125	45.0632492					5.6E+07	5.6E+07	0.39397	ID=promoter_13914;
12	6E+07	12_55895495	189	15	164	125	102	145	0.329624	1	55	62	35	12	59	24	0.00219251	0.1186258	82.125	68.3989087					5.6E+07	5.6E+07	0.33862	ID=promoter_13916;
12	6E+07	12_55921874	7	20	7	4	14	6	0.098789	1	10	0	14	22	5	18	0.01516798	0.5480081	10.5	7.6718409					5.6E+07	5.6E+07	0.19566	ID=promoter_13922;
12	6E+07	12_55924113	44	68	75	107	56	91	0.155939	1	61	96	12	24	79	18	4.29E-08	9.54E-06	60.875	33.0473146								
12	6E+07	12_55963422	22	2	14	11	12	13	0.980472	1	36	20	7	13	28	10	0.02694098	0.8576836	15.625	10.4599577					5.6E+07	5.6E+07	0.13741	ID=promoter_13931;
12	6E+07	12_55965222	32	50	35	79	41	57	0.708057	1	58	12	65	46	35	56	0.03732666	1	47.125	20.9519007								
12	6E+07	12_55995797	0	16	0	0	8	0	0.00917	0.37984	0	14	0	10	7	5	0.9051169	1	5	7.09124208	6E+07	5.6E+07	ID=ENSP0000379264;TEKT3;;	TEKT3				
12	6E+07	12_56008016	1115	300	157	154	708	156	3.41E-98	#######	124	72	65	22	98	44	2.40E-04	0.0192085	251.13	359.036185								
12	6E+07	12_56183220	79	38	30	39	59	35	0.004964	0.23382	40	35	17	3	38	10	7.84E-04	0.0516813	35.125	21.9052342								
12	6E+07	12_56215048	648	226	611	554	437	583	0.125885	1	285	249	307	109	267	208	0.16974201	1	373.63	201.355363					5.6E+07	5.6E+07	0.66444	ID=promoter_13953;
12	6E+07	12_56256068	28	24	13	35	26	24	0.697783	1	19	90	59	79	55	69	0.24384105	1	43.375	28.9972289					5.6E+07	5.6E+07	0.14146	ID=promoter_13960;
12	6E+07	12_56329453	12	15	9	3	14	6	0.098789	1	11	13	7	10	12	9	0.89054267	1	10	3.74165739								
12	6E+07	12_56358306	57	134	77	96	96	87	0.217186	1	120	86	94	25	103	60	0.01550452	0.5553889	86.125	34.3654207					5.6E+07	5.6E+07	0.29358	ID=promoter_13977;
12	6E+07	12_56378860	15	5	4	21	10	13	0.970528	1	19	12	5	22	16	14	0.9876114	1	12.875	7.51070664	6E+07	5.6E+07	ID=ENSP0000261647;TTC19;NM_017775;	TTC19				
12	6E+07	12_56382243	5	19	1	1	12	1	0.003675	0.18279	14	0	0	3	7	2	0.29886173	1	5.375	7.19002285	6E+07	5.6E+07	ID=ENSP0000261647;TTC19;NM_017775;	TTC19	5.6E+07	5.6E+07	0.37138	ID=promoter_13984;
12	6E+07	12_56384437	9	41	24	42	25	33	0.905451	1	2	41	23	84	22	54	3.31E-04	0.0251523	33.25	25.4319821	6E+07	5.6E+07	ID=ENSP0000261647;TTC19;NM_017775;	TTC19				
12	6E+07	12_56386570	47	105	58	79	76	69	0.303315	1	42	61	64	47	52	56	0.73834479	1	62.875	20.7118841	6E+07	5.6E+07	ID=ENSP0000261647;TTC19;NM_017775;	TTC19	5.6E+07	5.6E+07	0.48627	ID=promoter_13985;
12	6E+07	12_56390005	133	110	141	299	122	220	6.29E-04	0.04305	75	90	102	31	83	67	0.70075609	1	122.63	79.1615663	6E+07	5.6E+07	ID=ENSP0000261647;TTC19;NM_017775;	TTC19	5.6E+07	5.6E+07	0.92758	ID=promoter_13986;
12	6E+07	12_56392069	11	20	28	4	16	16	0.903021	1	0	5	9	31	3	20	9.56E-04	0.060345	13.5	11.5263673					5.6E+07	5.6E+07	0.92758	ID=promoter_13986;
12	6E+07	12_56399315	47	84	93	79	66	86	0.813268	1	23	38	53	23	31	38	0.51145443	1	55	27.4278274					5.6E+07	5.6E+07	0.99651	ID=promoter_13987;
12	6E+07	12_56405502	7	12	7	2	10	5	0.276106	1	11	3	17	5	7	11	0.54353855	1	8	5.04267503					5.6E+07	5.6E+07	0.99651	ID=promoter_13987;
12	6E+07	12_56405586	1	28	11	6	15	9	0.269309	1	3	0	1	0	2	1	0.87709017	1	6.25	9.55809306					5.6E+07	5.6E+07	0.99651	ID=promoter_13987;

12	6E+07	12_56406844	109	20	139	58	65	99	0.260224	1	0	1	22	2	1	12	0.00617711	0.2683255	43.875	53.525528					5.6E+07	5.6E+07	0.99651	ID=promoter_13987;
12	6E+07	12_56413954	18	88	20	38	53	29	0.003285	0.16835	72	26	51	23	49	37	0.64071519	1	42	26.1588554					5.6E+07	5.6E+07	0.41223	ID=promoter_13989;
12	6E+07	12_56435834	23	0	7	19	12	13	0.980472	1	16	0	4	0	8	2	0.20550608	1	8.625	9.37987968					5.6E+07	5.6E+07	0.242	ID=promoter_13993;
12	6E+07	12_56498586	4	23	7	8	14	8	0.253058	1	20	0	14	1	10	8	0.95278883	1	9.625	8.56800527								
12	6E+07	12_56506041	88	32	73	137	60	105	0.045189	1	2	21	32	3	12	18	0.42746936	1	48.5	47.0956474					5.7E+07	5.7E+07	0.21805	ID=promoter_14008;
12	6E+07	12_56536013	24	1	11	7	13	9	0.46931	1	13	16	7	4	15	6	0.20085144	1	10.375	7.3277847	6E+07	5.7E+07	ID=ENSP00000385699;ALDH3A2;;	ALDH3A2	5.7E+07	5.7E+07	0.19453	ID=promoter_14015;
12	6E+07	12_56541457	285	169	214	170	227	192	0.00366	0.18279	94	64	93	88	79	91	0.36747292	1	147.13	76.1960582					5.7E+07	5.7E+07	0.57281	ID=promoter_14017;
12	6E+07	12_56558314	9	48	22	30	29	26	0.607762	1	13	33	12	29	23	21	0.9987298	1	24.5	13.1475147								
12	6E+07	12_56601538	47	97	57	78	72	68	0.438584	1	71	48	60	55	60	58	0.97449738	1	64.125	17.0078763					5.7E+07	5.7E+07	0.79867	ID=promoter_14033;
12	6E+07	12_56607239	18	33	11	1	26	6	3.38E-04	0.02566	2	0	15	5	1	10	0.01766414	0.6154554	10.625	11.2496032					5.7E+07	5.7E+07	0.43418	ID=promoter_14035;
12	6E+07	12_56611408	83	51	14	42	67	28	1.03E-05	0.00123	14	44	12	4	29	8	0.00552548	0.2488783	33	26.8061827					5.7E+07	5.7E+07	0.85332	ID=promoter_14036;
12	6E+07	12_56615011	19	50	7	20	35	14	0.001717	0.09971	5	30	28	86	18	57	8.40E-06	0.0010923	30.625	26.4895532					5.7E+07	5.7E+07	0.85332	ID=promoter_14036;
12	6E+07	12_56666998	184	126	86	71	155	79	4.35E-09	#######	214	85	116	154	150	135	0.9693382	1	129.5	50.9901951					5.7E+07	5.7E+07	0.1792	ID=promoter_14046;
12	6E+07	12_56681421	5	47	18	6	26	12	0.021538	0.74463	260	59	88	56	160	72	1.31E-06	0.0002135	67.375	83.0145211								
12	6E+07	12_56697641	13	11	27	10	12	19	0.716431	1	0	0	8	15	0	12	0.00154584	0.0883233	10.5	8.66849798					5.7E+07	5.7E+07	0.55378	ID=promoter_14051;
12	6E+07	12_56697715	156	27	169	167	92	168	0.002686	0.1429	25	25	63	62	25	63	6.01E-05	0.0059427	86.75	65.8716284					5.7E+07	5.7E+07	0.55378	ID=promoter_14051;
12	6E+07	12_56718180	19	11	8	11	15	10	0.374041	1	54	14	19	3	34	11	0.0064318	0.2773637	17.375	15.7383744					5.7E+07	5.7E+07	0.23857	ID=promoter_14054;
12	6E+07	12_56718962	245	101	276	161	173	219	0.754924	1	377	120	323	188	249	256	0.50843894	1	223.88	98.2452289					5.7E+07	5.7E+07	0.23857	ID=promoter_14054;
12	6E+07	12_56736875	25	39	17	22	32	20	0.080425	1	0	18	14	27	9	21	0.05613829	1	20.25	11.2599163	6E+07	5.7E+07	ID=ENSP00000373647;FAM83G;;	FAM83G	5.7E+07	5.7E+07	0.55579	ID=promoter_14059;
12	6E+07	12_56742884	50	16	49	58	33	54	0.317953	1	5	0	1	0	3	1	0.65196691	1	22.375	25.4723239					5.7E+07	5.7E+07	0.57073	ID=promoter_14060;
12	6E+07	12_56749450	5	23	126	144	14	135	3.39E-18	#######	0	2	10	21	1	16	7.41E-04	0.0492332	41.375	58.5733667								
12	6E+07	12_56779933	0	19	0	1	10	1	0.011548	0.45484	29	14	7	1	22	4	0.00378795	0.1837142	8.875	10.8158283	6E+07	5.7E+07	ID=ENSP00000379017;PRPSAP2;;	PRPSAP2	5.7E+07	5.7E+07	0.46763	ID=promoter_14067;
12	6E+07	12_56780493	0	13	15	0	7	8	0.998739	1	0	11	6	2	6	4	0.87665919	1	5.875	6.31183695	6E+07	5.7E+07	ID=ENSP00000379017;PRPSAP2;;	PRPSAP2	5.7E+07	5.7E+07	0.46763	ID=promoter_14067;
12	6E+07	12_56783500	32	15	21	16	24	19	0.435991	1	0	10	1	0	5	1	0.30486099	1	11.875	11.4572186	6E+07	5.7E+07	ID=ENSP00000379017;PRPSAP2;;	PRPSAP2				
12	6E+07	12_56810952	11	0	8	6	6	7	0.999953	1	66	29	2	1	48	2	3.46E-09	9.29E-07	15.375	22.46227					5.7E+07	5.7E+07	0.30146	ID=promoter_14074;

12	6E+07	12_56824032	3	30	15	10	17	13	0.50439	1	1	24	26	40	13	33	0.00582644	0.2576386	18.625	13.6898033								
12	6E+07	12_56830115	26	16	26	18	21	22	0.933629	1	69	24	34	67	47	51	0.73805499	1	35	21.0916368								
12	6E+07	12_56856176	208	191	153	123	200	138	7.76E-06	0.00096	366	403	140	83	385	112	5.38E-29	5.61E-26	208.38	115.743482								
12	6E+07	12_56867772	0	0	2	1	0	2	0.426327	1	14	8	25	1	11	13	0.83819512	1	6.375	8.991067								
12	6E+07	12_56901374	28	64	22	16	46	19	3.31E-04	0.02521	3	39	62	58	21	60	1.78E-05	0.0020918	36.5	22.9906813	6E+07	5.7E+07	ID=ENSP0000268717;COPS3;NM_003653;	COPS3				
12	6E+07	12_56906300	11	36	27	16	24	22	0.704268	1	31	25	35	20	28	28	0.96060652	1	25.125	8.96720214	6E+07	5.7E+07	ID=ENSP0000268717;COPS3;NM_003653;	COPS3	5.7E+07	5.7E+07	0.71667	ID=promoter_14092;
12	6E+07	12_56914550	11	9	14	3	10	9	0.84607	1	1	11	0	20	6	10	0.51486293	1	8.625	6.9062601	6E+07	5.7E+07	ID=ENSP0000268717;COPS3;NM_003653;	COPS3	5.7E+07	5.7E+07	0.82086	ID=promoter_14094;
12	6E+07	12_56975786	0	16	2	2	8	2	0.097885	1	28	5	10	0	17	5	0.05852787	1	7.875	9.83343422								
12	6E+07	12_56987357	156	90	130	84	123	107	0.076163	1	38	53	36	24	46	30	0.32156472	1	76.375	47.5873257					5.7E+07	5.7E+07	0.62672	ID=promoter_14116;
12	6E+07	12_56995297	587	654	851	796	621	824	0.067485	1	510	413	1046	240	462	643	1.69E-10	5.46E-08	637.13	257.754278					5.7E+07	5.7E+07	0.25923	ID=promoter_14119;
12	6E+07	12_56996241	29	7	22	55	18	39	0.091386	1	13	15	31	2	14	17	0.75475782	1	21.75	16.7907287					5.7E+07	5.7E+07	0.25923	ID=promoter_14119;
12	6E+07	12_56997233	181	59	141	68	120	105	0.088747	1	66	50	40	41	58	41	0.41139034	1	80.75	51.7128058					5.7E+07	5.7E+07	0.77475	ID=promoter_14120;
12	6E+07	12_57032576	1449	1421	2132	3114	1435	2623	5.08E-41	#######	599	437	653	327	518	490	0.94999652	1	1266.5	971.904757								
12	6E+07	12_57034353	31	14	86	74	23	80	8.85E-06	0.00108	108	15	70	118	62	94	0.00993241	0.3910056	64.5	40.461622					5.7E+07	5.7E+07	0.22117	ID=promoter_14132;
12	6E+07	12_57053700	171	161	206	114	166	160	0.206926	1	53	17	31	12	35	22	0.35607393	1	95.625	77.1305711					5.7E+07	5.7E+07	0.57464	ID=promoter_14137;
12	6E+07	12_57056734	14	37	25	23	26	24	0.697783	1	46	55	29	7	51	18	0.00122967	0.074208	29.5	15.9821329								
12	6E+07	12_57061151	23	3	28	42	13	35	0.031286	1	49	1	72	34	25	53	0.0021326	0.1161796	31.5	23.573593								
12	6E+07	12_57167769	461	170	396	279	316	338	0.499087	1	93	72	81	2	83	42	0.00514922	0.234296	194.25	166.738247								
12	6E+07	12_57176167	2	21	2	3	12	3	0.030625	0.98374	14	32	22	14	23	18	0.86487232	1	13.75	10.9902554					5.7E+07	5.7E+07	0.16932	ID=promoter_14145;
12	6E+07	12_57180033	98	37	98	63	68	81	0.995617	1	16	2	30	9	9	20	0.07989336	1	44.125	38.1928845								
12	6E+07	12_57192532	26	21	27	41	24	34	0.777678	1	5	26	12	3	16	8	0.35023049	1	20.125	12.766446	6E+07	5.7E+07	ID=ENSP0000264659;;NM_025248;		5.7E+07	5.7E+07	0.45031	ID=promoter_14147;
13	215991	13_215991	360	180	173	264	270	219	2.43E-04	0.01915	133	196	164	101	165	133	0.48615586	1	196.38	81.347469					214786	216375	0.48851	ID=promoter_14165;
13	900840	13_900840	441	265	374	275	353	325	0.006833	0.29872	167	100	112	95	134	104	0.39578858	1	228.63	131.751864	899846	901242	ID=ENSP0000306670-D3;NUDT16L1;NM_032349;	NUDT16L1	900374	901888	0.38078	ID=promoter_14167;
13	901642	13_901642	252	106	256	260	179	258	0.105261	1	76	60	87	29	68	58	0.89700969	1	140.75	97.9631855					900374	901888	0.38078	ID=promoter_14167;
13	2E+06	13_1891436	38	5	22	81	22	52	0.019203	0.68357	3	18	20	9	11	15	0.61974467	1	24.5	25.4277688					1890857	1892120	0.27112	ID=promoter_14173;
13	2E+06	13_20823	0	28	2	12	14	7	0.165008	1	1	8	2	2	5	2	0.58563225	1	6.875	9.46327488								
13	3E+06	13_25198	16	80	16	25	48	21	4.53E-04	0.03263	11	19	32	19	15	26	0.14599233	1	27.25	22.2437792								

13	3E+06	13_26886	7	23	0	1	15	1	6.51E-04	0.04418	63	32	0	0	48	0	2.17E-10	6.98E-08	15.75	22.6384628								
13	3E+06	13_27273	118	90	34	10	104	22	2.05E-15	#######	20	18	34	15	19	25	0.5123552	1	42.375	39.6770445								
13	3E+06	13_2820513	409	275	200	202	342	201	2.20E-14	#######	190	91	162	128	141	145	0.6805022	1	207.13	98.2074153					2819265	2820846	0.47401	ID=promoter_14179;
13	4E+06	13_37365	2	44	42	26	23	34	0.691592	1	29	6	38	4	18	21	0.77315138	1	23.875	17.5534897								
13	4E+06	13_37760	21	105	29	21	63	25	9.34E-06	0.00113	121	164	111	127	143	119	0.6845972	1	87.375	55.6261436								
13	4E+06	13_4314605	1004	550	956	818	777	887	0.858704	1	792	431	450	237	612	344	5.99E-13	2.55E-10	654.75	276.635268					4313493	4315918	0.67621	ID=promoter_14185;
13	5E+06	13_47802	56	22	53	56	39	55	0.678132	1	0	41	72	29	21	51	5.83E-04	0.0401168	41.125	23.117325								
13	5E+06	13_5139384	13	4	9	4	9	7	0.713906	1	1	19	9	6	10	8	0.95278883	1	8.125	5.7678295					5138688	5141336	0.49654	ID=promoter_14186;
13	5E+06	13_5141093	22	6	11	36	14	24	0.52503	1	7	3	5	0	5	3	0.8350419	1	11.25	11.9970234					5138688	5141336	0.49654	ID=promoter_14186;
13	5E+06	13_52303	1	50	11	13	26	12	0.021538	0.74463	55	33	7	1	44	4	2.48E-07	4.77E-05	21.375	21.7119947								
13	7E+06	13_67675	11	13	9	12	12	11	0.839207	1	41	26	6	1	34	4	2.15E-05	0.0024657	14.875	12.755251								
13	7E+06	13_7066442	8	9	16	30	9	23	0.127121	1	24	15	29	2	20	16	0.90780655	1	16.625	10.2530483	7E+06	7196603	ID=ENSP00000281523;ZNF385D;NM_024697;	ZNF385D				
13	8E+06	13_78659	1	10	11	14	6	13	0.45043	1	0	2	12	2	1	7	0.08320466	1	6.5	5.75698334								
13	8E+06	13_78749	2	16	7	8	9	8	0.848833	1	20	11	20	2	16	11	0.74569574	1	10.75	7.30459738								
13	8E+06	13_81580	1	28	11	0	15	6	0.065303	1	27	54	5	13	41	9	1.14E-04	0.0101988	17.375	18.2438835								
13	9E+06	13_86367	18	46	29	13	32	21	0.11093	1	131	23	39	9	77	24	6.21E-06	0.0008357	38.5	39.4316769								
13	1E+07	13_96376	139	567	136	191	353	164	4.08E-23	#######	412	304	500	232	358	366	0.42013691	1	310.13	165.766994								
13	1E+07	13_96467	21	29	11	9	25	10	0.01059	0.42992	30	36	41	0	33	21	0.39890162	1	22.125	14.3670009								
13	1E+07	13_98123	8	10	20	45	9	33	0.006022	0.27245	47	19	13	17	33	15	0.0650987	1	22.375	15.1745793								
13	1E+07	13_99007	34	19	18	30	27	24	0.611598	1	95	28	49	11	62	30	0.01196223	0.4544974	35.5	26.6993847								
13	1E+07	13_10409111	1130	465	1016	1709	798	1363	2.41E-16	#######	601	411	760	422	506	591	6.70E-04	0.0451887	814.25	451.638841					1E+07	1E+07	0.6963	ID=promoter_14205;
13	1E+07	13_10780247	7	36	11	5	22	8	0.011059	0.44213	29	6	0	27	18	14	0.88543974	1	15.125	13.4529604								
13	1E+07	13_11121170	59	57	46	60	58	53	0.419924	1	51	27	44	15	39	30	0.74371451	1	44.875	16.1549417								
13	1E+07	13_11404416	0	3	10	6	2	8	0.255957	1	2	13	8	29	8	19	0.06853691	1	8.875	9.20306626								
13	1E+07	13_11587117	5	106	43	50	56	47	0.235754	1	53	60	67	75	57	71	0.24923067	1	57.375	28.7796431	1E+07	1.2E+07	ID=ENSP00000295720;NEK10;;	NEK10				
13	1E+07	13_12046890	10	55	4	0	33	2	7.46E-08	#######	61	13	30	4	37	17	0.05031232	1	22.125	23.9966516								
13	1E+07	13_12088756	4978	1059	4382	2143	3019	3263	0.005249	0.2445	1030	711	935	650	871	793	0.93451569	1	1986	1732.29476					1.2E+07	1.2E+07	0.62691	ID=promoter_14209;
13	1E+07	13_12885667	3	13	5	4	8	5	0.532535	1	51	17	10	9	34	10	0.00342551	0.1709558	14	15.6843871								
13	1E+07	13_13238325	6	0	14	0	3	7	0.596562	1	0	17	6	0	9	3	0.27712561	1	5.375	6.82301777								
13	1E+07	13_13318119	2	63	13	15	33	14	0.00394	0.1935	15	14	33	21	15	27	0.11059826	1	22	18.6930086								
13	1E+07	13_13633062	1509	1036	1404	1469	1273	1437	0.608785	1	650	607	488	259	629	374	6.14E-11	2.10E-08	927.75	491.34909								
13	1E+07	13_13814983	96	15	57	27	56	42	0.087144	1	60	23	22	17	42	20	0.04419125	1	39.625	28.6851754								
13	1E+07	13_14621234	109	69	120	72	89	96	0.850179	1	22	35	18	12	29	15	0.17706132	1	57.125	41.9129625								
13	2E+07	13_15050638	1577	367	1615	1362	972	1489	6.97E-10	#######	723	424	704	361	574	533	0.99962545	1	891.63	541.607116								
13	2E+07	13_15747275	4	2	0	5	3	3	0.981055	1	23	0	11	18	12	15	0.74080056	1	7.875	8.64270956								
13	2E+07	13_15747321	1	1	4	5	1	5	0.353258	1	34	19	12	1	27	7	0.00575936	0.2548307	9.625	11.7344122								
13	2E+07	13_15848233	76	89	43	8	83	26	2.25E-09	#######	83	33	37	5	58	21	6.55E-04	0.0442607	46.75	32.7272276					1.6E+07	1.6E+07	0.54084	ID=promoter_14223;
13	2E+07	13_15943409	169	224	217	223	197	220	0.88222	1	225	272	353	229	249	291	0.02690094	0.8575559	239	53.779975								
13	2E+07	13_16105218	5	28	27	22	17	25	0.771176	1	38	15	19	0	27	10	0.0371409	1	19.25	12.4642345								

13	2E+07	13_16411275	2	69	8	12	36	10	6.61E-05	0.0062	7	13	9	77	10	43	9.37E-06	0.0011945	24.625	30.1185159	2E+07	1.6E+07	ID=ENSP0000304743;CLASP2;;	CLASP2				
13	2E+07	13_18043011	5	19	4	8	12	6	0.213448	1	9	8	41	12	9	27	0.00543585	0.2460834	13.25	12.139075								
13	2E+07	13_18050194	1388	485	1005	980	937	993	0.083377	1	569	276	453	114	423	284	1.34E-04	0.0116593	658.75	427.40471					1.8E+07	1.8E+07	0.40759	ID=promoter_14234;
13	2E+07	13_18155052	56	26	23	68	41	46	0.978803	1	24	21	65	90	23	78	3.55E-08	8.08E-06	46.625	26.5003369								
13	2E+07	13_18640258	52	28	29	15	40	22	0.013995	0.53231	0	0	0	0	0	0	1	1	15.5	19.3907194								
13	2E+07	13_18874264	18	50	11	16	34	14	0.00261	0.13949	27	32	11	3	30	7	0.00181176	0.1006964	21	14.9092493	2E+07	1.9E+07	ID=ENSP0000308597;DLEC1;NM_007335;	DLEC1				
13	2E+07	13_18951045	17	25	20	12	21	16	0.42382	1	27	17	28	41	22	35	0.13287223	1	23.375	9.02278861					1.9E+07	1.9E+07	0.49087	ID=promoter_14245;
13	2E+07	13_19116770	23	75	7	8	49	8	1.12E-08	#######	0	27	4	2	14	3	0.04246461	1	18.25	24.9327667	2E+07	1.9E+07	ID=ENSP0000273173;SLC22A14;NM_004803;	SLC22A14				
13	2E+07	13_19268323	6	6	7	17	6	12	0.558139	1	0	12	4	0	6	2	0.42506085	1	6.5	5.75698334					1.9E+07	1.9E+07	0.35174	ID=promoter_14251;
13	2E+07	13_19272934	24	11	80	45	18	63	9.77E-05	0.00872	104	4	43	13	54	28	0.04072278	1	40.5	35.5567956	2E+07	1.9E+07	ID=ENSP0000328968;SCN5A;NM_198056;	SCN5A				
13	2E+07	13_19285767	499	161	407	416	330	412	0.699834	1	56	19	21	10	38	16	0.02490784	0.8088272	198.63	207.725738	2E+07	1.9E+07	ID=ENSP0000328968;SCN5A;NM_198056;	SCN5A				
13	2E+07	13_19343614	0	6	3	2	3	3	0.981055	1	44	0	6	1	22	4	0.00378795	0.1837142	7.75	14.8396188	2E+07	1.9E+07	ID=ENSP0000328968;SCN5A;NM_198056;	SCN5A				
13	2E+07	13_19369302	5	19	1	10	12	6	0.213448	1	21	6	5	2	14	4	0.08931834	1	8.625	7.53918336					1.9E+07	1.9E+07	0.35817	ID=promoter_14252;
13	2E+07	13_19582180	7	18	2	15	13	9	0.46931	1	13	16	36	0	15	18	0.76034467	1	13.375	11.2876355								
13	2E+07	13_20469618	3	13	13	0	8	7	0.850897	1	25	10	6	0	18	3	0.00813808	0.3342859	8.75	8.41342465								
13	2E+07	13_20731544	48	62	24	13	55	19	6.58E-06	0.00082	15	42	61	0	29	31	0.858757	1	33.125	23.3754297								
13	2E+07	13_21079937	29	5	19	42	17	31	0.33747	1	43	55	20	0	49	10	1.04E-05	0.0013129	26.625	19.2497681								
13	2E+07	13_21622167	30	60	21	33	45	27	0.019532	0.69375	9	24	1	10	17	6	0.10713464	1	23.5	18.4003106								
13	2E+07	13_21675575	3	3	17	6	3	12	0.129494	1	18	30	15	18	24	17	0.69596948	1	13.75	9.28516482	2E+07	2.2E+07	ID=ENSP0000340702;TRAK1;NM_014965;	TRAK1	2.2E+07	2.2E+07	0.14664	ID=promoter_14275;
13	2E+07	13_22009414	1	11	1	10	6	6	0.962469	1	10	14	12	3	12	8	0.76853133	1	7.75	5.23040561								
13	2E+07	13_22052421	262	87	257	157	175	207	0.996654	1	63	86	78	4	75	41	0.02272922	0.7568963	124.25	93.2519628					2.2E+07	2.2E+07	0.4438	ID=promoter_14283;
13	2E+07	13_22223332	306	313	256	230	310	243	1.35E-05	0.00155	154	176	80	21	165	51	5.32E-12	2.04E-09	192	104.650439								
13	2E+07	13_22432907	11	15	20	17	13	19	0.82904	1	1	2	9	0	2	5	0.46647814	1	9.375	7.72634639								
13	2E+07	13_22490499	39	13	31	17	26	24	0.697783	1	0	0	14	0	0	7	0.02292696	0.7568963	14.25	14.6945082								
13	2E+07	13_22506038	40	149	43	58	95	51	2.34E-05	0.00252	173	18	80	43	96	62	0.08309133	1	75.5	55.9515096								

13	2E+07	13_22617428	600	330	436	477	465	457	0.02692	0.89347	368	237	245	112	303	179	9.00E-06	0.0011587	350.63	154.542585					2.3E+07	2.3E+07	0.59922	ID=promoter_14297;
13	2E+07	13_23106139	6	28	23	18	17	21	0.98748	1	31	1	36	22	16	29	0.09020737	1	20.625	12.0230434								
13	2E+07	13_23259653	13	2	17	20	8	19	0.232241	1	13	0	0	5	7	3	0.51857467	1	8.75	7.95972002								
13	2E+07	13_23325896	72	43	112	79	58	96	0.114719	1	115	136	130	4	126	67	9.75E-04	0.0611121	86.375	45.9345419								
13	2E+07	13_23530836	16	2	0	6	9	3	0.131542	1	0	8	0	15	4	8	0.43697044	1	5.875	6.64266513					2.4E+07	2.4E+07	0.2071	ID=promoter_14304;
13	2E+07	13_24585090	10	14	1	0	12	1	0.003675	0.18279	22	0	25	0	11	13	0.83819512	1	9	10.4060422								
13	2E+07	13_24694777	152	59	63	63	106	63	7.57E-05	0.00699	47	44	49	0	46	25	0.09396206	1	59.625	42.4733109					2.5E+07	2.5E+07	0.21924	ID=promoter_14322;
13	3E+07	13_25647508	7	31	33	11	19	22	0.999149	1	21	15	36	12	18	24	0.50317852	1	20.75	11.22179	3E+07	2.6E+07	ID=ENSP00000343442;DHX30;NM_138615;	DHX30				
13	3E+07	13_25752520	0	9	10	3	5	7	0.955252	1	0	28	7	0	14	4	0.08931834	1	7.125	9.38749168	3E+07	2.6E+07	ID=ENSP00000379083;MAP4;NM_002375;	MAP4				
13	3E+07	13_26082125	4	18	0	0	11	0	0.001579	0.09256	4	6	8	2	5	5	0.99284883	1	5.25	5.84929788					2.6E+07	2.6E+07	0.23539	ID=promoter_14348;
13	3E+07	13_26400774	0	7	2	4	4	3	0.840045	1	32	2	3	1	17	2	0.0046333	0.2138882	6.375	10.5686531					2.6E+07	2.6E+07	0.37756	ID=promoter_14357;
13	3E+07	13_26431616	0	15	19	26	8	23	0.078123	1	19	30	62	37	25	50	0.00566012	0.2544609	26	18.236541								
13	3E+07	13_26478116	434	122	291	185	278	238	0.001646	0.0961	67	86	94	52	77	73	0.99063529	1	166.38	133.162562					2.6E+07	2.6E+07	0.24645	ID=promoter_14360;
13	3E+07	13_26848392	51	22	63	89	37	76	0.018147	0.65414	22	19	22	9	21	16	0.83919704	1	37.125	27.6686388					2.7E+07	2.7E+07	0.42611	ID=promoter_14374;
13	3E+07	13_26977357	1	12	8	0	7	4	0.503048	1	0	1	13	11	1	12	0.00617711	0.2683255	5.75	5.8002463					2.7E+07	2.7E+07	0.50099	ID=promoter_14378;
13	3E+07	13_27007690	110	79	97	112	95	105	0.915634	1	0	18	12	2	9	7	0.94097808	1	53.75	50.2188069	3E+07	2.7E+07	ID=ENSP00000328287;RNF123;NM_022064;	RNF123	2.7E+07	2.7E+07	0.2456	ID=promoter_14380;
13	3E+07	13_27078946	2946	1338	3151	2761	2142	2956	7.18E-08	#######	1682	1059	1048	821	1371	935	3.42E-12	1.35E-09	1850.8	951.827378					2.7E+07	2.7E+07	0.35015	ID=promoter_14382;
13	3E+07	13_27209909	0	11	8	0	6	4	0.674789	1	1	26	0	1	14	1	0.00574567	0.2548307	5.875	9.15637951	3E+07	2.7E+07	ID=ENSP00000378603;RBM5;;	RBM5				
13	3E+07	13_27418755	27	4	30	16	16	23	0.822157	1	13	22	36	1	18	19	0.92496372	1	18.625	12.3974364								
13	3E+07	13_27524979	7	19	15	3	13	9	0.46931	1	1	7	7	1	4	4	0.99427496	1	7.5	6.4807407	3E+07	2.8E+07	ID=ENSP00000354228;CACNA2D2;;	CACNA2D2				
13	3E+07	13_27750906	375	153	262	228	264	245	0.030155	0.97856	111	111	122	32	111	77	0.14591256	1	174.25	108.4353					2.8E+07	2.8E+07	0.28473	ID=promoter_14410;
13	3E+07	13_28412075	14	10	0	9	12	5	0.126784	1	0	8	11	1	4	6	0.75330063	1	6.625	5.50162314	3E+07	2.8E+07	ID=ENSP00000381764;VPRBP;;	VPRBP				
13	3E+07	13_28751576	35	1	25	5	18	15	0.617094	1	0	2	1	1	1	1	0.99856566	1	8.75	13.4668906	3E+07	2.9E+07	ID=ENSP00000378421;WDR51A;;	WDR51A				
13	3E+07	13_28862314	2	15	5	11	9	8	0.848833	1	13	13	3	0	13	2	0.02644099	0.8480056	7.75	5.87367006								

13	3E+07	13_29210448	162	8	222	172	85	197	6.10E-07	#######	11	25	93	31	18	62	9.73E-07	0.0001625	90.5	84.5238766					2.9E+07	2.9E+07	0.33213	ID=promoter_14436;
13	3E+07	13_29941069	13	9	0	11	11	6	0.3028	1	19	3	12	0	11	6	0.57179039	1	8.375	6.80204801								
13	3E+07	13_30126845	16	17	7	10	17	9	0.140533	1	47	14	6	3	31	5	2.16E-04	0.0176336	15	13.8564065	3E+07	3E+07	ID=ENSP0000382494-D4;CACNA1C;NM_001129827;	CACNA1C				
13	3E+07	13_30988628	56	29	39	52	43	46	0.910016	1	111	17	53	57	64	55	0.91850499	1	51.75	27.8913709								
13	3E+07	13_31227107	162	134	185	185	148	185	0.84667	1	130	103	117	54	117	86	0.26164544	1	133.75	44.1612306								
13	3E+07	13_31302198	7	37	19	33	22	26	0.999662	1	46	16	38	5	31	22	0.62998213	1	25.125	15.3942243								
13	3E+07	13_31421174	60	49	88	64	55	76	0.650894	1	96	71	99	75	84	87	0.76888162	1	75.25	17.8225379								
13	3E+07	13_31472032	56	77	61	52	67	57	0.201474	1	0	49	32	9	25	21	0.94599092	1	42	26.4142819								
13	3E+07	13_31544764	283	98	286	204	191	245	0.650666	1	62	72	84	34	67	59	0.95939407	1	140.38	101.913041					3.2E+07	3.2E+07	0.63416	ID=promoter_14454;
13	3E+07	13_31755957	17	27	22	27	22	25	0.99414	1	20	9	27	12	15	20	0.56420259	1	20.125	7.01910658								
13	3E+07	13_32191555	24	107	16	37	66	27	8.72E-06	0.00107	0	28	17	25	14	21	0.37101303	1	31.75	32.2567822								
13	3E+07	13_33346964	0	13	6	0	7	3	0.314307	1	23	11	18	0	17	9	0.38755874	1	8.875	8.85498246								
13	3E+07	13_33377180	4378	2928	5327	5548	3653	5438	8.29E-28	#######	2727	1733	2974	1142	2230	2058	0.98916246	1	3344.6	1603.2925					3.3E+07	3.3E+07	0.52367	ID=promoter_14461;
13	3E+07	13_33501772	1	78	24	19	40	22	0.013995	0.53231	6	29	21	73	18	47	4.88E-04	0.0348216	31.375	28.7697137								
13	3E+07	13_33695173	202	121	203	216	162	210	0.632858	1	162	60	94	15	111	55	5.78E-04	0.0398304	134.13	73.9680192					3.4E+07	3.4E+07	0.51986	ID=promoter_14465;
13	3E+07	13_33826204	6	1	5	8	4	7	0.813458	1	0	10	16	5	5	11	0.25523295	1	6.375	5.09726818								
13	3E+07	13_33834775	5	31	6	8	18	7	0.033499	1	8	61	17	39	35	28	0.8442835	1	21.875	20.1879561								
13	3E+07	13_34161640	0	22	13	1	11	7	0.438207	1	55	0	8	1	28	5	7.63E-04	0.0504367	12.5	18.8755322								
13	4E+07	13_35051913	12	32	24	12	22	18	0.522468	1	29	3	32	3	16	18	0.85288016	1	18.375	12.3628187								
13	4E+07	13_35192270	15	56	19	47	36	33	0.591026	1	22	49	28	27	36	28	0.78400353	1	32.875	15.5051835								
13	4E+07	13_35339424	31	49	26	14	40	20	0.005812	0.26547	50	18	75	79	34	77	4.32E-05	0.0044892	42.75	24.7948727								
13	4E+07	13_35443231	49	82	25	36	66	31	7.73E-05	0.0071	26	26	3	39	26	21	0.89538361	1	35.75	23.0015527								
13	4E+07	13_35503276	1	59	15	9	30	12	0.004264	0.20626	20	61	47	83	41	65	0.02455403	0.7986094	36.875	29.5897545								
13	4E+07	13_35691588	20	94	49	37	57	43	0.08835	1	134	70	101	10	102	56	0.00624991	0.2712401	64.375	43.1109449								
13	4E+07	13_35856635	20	25	9	23	23	16	0.267873	1	0	60	39	14	30	27	0.99379101	1	23.75	18.6681547								
13	4E+07	13_36484206	135	83	62	43	109	53	3.96E-07	#######	36	26	13	14	31	14	0.07401504	1	51.5	41.276074								
13	4E+07	13_37529566	75	49	51	49	62	50	0.140127	1	91	27	34	7	59	21	4.74E-04	0.0339584	47.875	26.4760674								
13	4E+07	13_37851903	28	134	22	60	81	41	3.75E-05	0.00379	61	77	106	61	69	84	0.2429602	1	68.625	37.3322438								
13	4E+07	13_39938792	12	12	0	6	12	3	0.030625	0.98374	3	6	27	5	5	16	0.03625394	1	8.875	8.39110924								
13	4E+07	13_40326306	54	14	35	38	34	37	0.951407	1	15	11	16	0	13	8	0.65599869	1	22.875	17.7155098								
13	4E+07	13_41206547	5	19	15	10	12	13	0.980472	1	11	6	14	1	9	8	0.99626583	1	10.125	5.91457039								
13	4E+07	13_41549396	9	34	7	16	22	12	0.091688	1	24	0	22	32	12	27	0.03064003	0.9477626	18	12.1537766								
13	4E+07	13_42455289	8	0	4	2	4	3	0.840045	1	34	0	0	26	17	13	0.87203467	1	9.25	13.2638068								
13	4E+07	13_43482392	43	53	20	61	48	41	0.325804	1	45	102	62	37	74	50	0.22202085	1	52.875	24.0679841								

13	4E+07	13_43666043	415	280	283	246	348	265	6.43E-07	0.0001	141	256	200	41	199	121	0.00112238	0.0691941	232.75	110.160597								
13	4E+07	13_44454009	15	30	3	1	23	2	2.40E-05	0.00257	57	46	38	2	52	20	0.00258053	0.1348666	24	21.8435999								
13	4E+07	13_44741232	3	96	15	7	50	11	1.29E-07	#######	19	4	8	2	12	5	0.30420563	1	19.25	31.5764379								
13	4E+07	13_44884489	12	26	16	18	19	17	0.718296	1	48	21	25	4	35	15	0.03750218	1	21.25	12.947697								
13	4E+07	13_44947679	14	17	11	34	16	23	0.822157	1	39	13	28	34	26	31	0.63757172	1	23.75	11.2090525								
13	5E+07	13_45064753	13	43	8	0	28	4	1.54E-05	0.00173	8	28	8	8	18	8	0.20901227	1	14.5	14.0203933								
13	5E+07	13_45514535	2920	2315	4032	3556	2618	3794	7.93E-16	#######	327	289	530	97	308	314	0.49516618	1	1758.3	1627.31152								
13	5E+07	13_45977998	233	63	139	113	148	126	0.029466	0.96276	58	33	37	7	46	22	0.03403886	1	85.375	73.5894742								
13	5E+07	13_46329973	549	230	394	415	390	405	0.228736	1	671	560	384	162	616	273	3.91E-24	3.28E-21	420.63	170.670893								
13	5E+07	13_46551087	49	93	20	37	71	29	3.51E-06	0.00047	79	29	108	36	54	72	0.12739908	1	56.375	32.5924509								
13	5E+07	13_47263979	5	33	30	10	19	20	0.944476	1	38	66	22	24	52	23	0.01036781	0.4053038	28.5	18.8072934								
13	5E+07	13_47408929	9	11	0	1	10	1	0.011548	0.45484	29	7	13	2	18	8	0.20901227	1	9	9.3960478								
13	5E+07	13_49470724	4	11	21	27	8	24	0.058083	1	26	6	1	3	16	2	0.00719486	0.3031284	12.375	10.7163093								
13	5E+07	13_50176502	72	66	147	110	69	129	0.006916	0.3016	49	39	39	46	44	43	0.9701733	1	71	38.6929303	5E+07	5E+07	ID=ENSP00000380602-D2;CNTN4;NM_175607;	CNTN4				
13	5E+07	13_50206367	224	50	115	89	137	102	0.002145	0.11849	35	25	23	0	30	12	0.0403413	1	70.125	72.5661029								
13	5E+07	13_50428999	38	43	48	105	41	77	0.048848	1	24	16	3	0	20	2	0.00122165	0.0738454	34.625	33.534364								
13	5E+07	13_51133938	0	27	4	1	14	3	0.010924	0.43763	79	116	65	59	98	62	0.06046094	1	43.875	42.6763149								
13	5E+07	13_51194562	1	16	0	21	9	11	0.995818	1	4	5	1	0	5	1	0.30486099	1	6	8.03563492								
13	5E+07	13_51408686	129	50	109	100	90	105	0.9993	1	42	24	0	28	33	14	0.04245489	1	60.25	46.4535098								
13	5E+07	13_52350978	17	22	11	10	20	11	0.118302	1	34	35	7	41	35	24	0.52090333	1	22.125	13.0322402								
13	5E+07	13_53650686	9	41	11	20	25	16	0.157912	1	2	22	12	3	12	8	0.76853133	1	15	12.6716782								
13	5E+07	13_54280775	6	1	0	11	4	6	0.929647	1	68	21	9	0	45	5	4.83E-07	8.64E-05	14.5	22.7470563								
13	6E+07	13_55123452	8	49	29	39	29	34	0.999998	1	19	35	18	39	27	29	0.85917478	1	29.5	13.606721								
13	6E+07	13_55171902	1	7	0	19	4	10	0.424038	1	19	0	11	0	10	6	0.69729498	1	7.125	8.33987838	6E+07	5.5E+07	ID=ENSP00000296003;MTMR14;NM_001077525;	MTMR14				
13	6E+07	13_55364608	12	19	5	7	16	6	0.042495	1	20	10	9	2	15	6	0.20085144	1	10.5	6.34710283	6E+07	5.5E+07	ID=ENSP00000373325;IL17RE;NM_153483;	IL17RE	5.5E+07	5.5E+07	0.37201	ID=promoter_14578;
13	6E+07	13_55459643	0	7	0	7	4	4	0.97482	1	0	8	4	23	4	14	0.04184353	1	6.125	7.62397534								
13	6E+07	13_55627759	5	2	1	8	4	5	0.995512	1	0	20	8	27	10	18	0.2304087	1	8.875	9.68706796	6E+07	5.6E+07	ID=ENSP00000287652;GHRLOS;NM_014760;	GHRLOS				
13	6E+07	13_56071347	15	168	37	38	92	38	1.10E-07	#######	40	132	93	35	86	64	0.41098218	1	69.75	55.0837025								
13	6E+07	13_56128821	5	18	3	5	12	4	0.066899	1	0	2	8	2	1	5	0.22510075	1	5.375	5.65527567					5.6E+07	5.6E+07	0.19334	ID=promoter_14596;
13	6E+07	13_56247499	10	22	9	10	16	10	0.283594	1	28	26	25	20	27	23	0.95651383	1	18.75	7.90569415								
13	6E+07	13_56466090	19	76	34	22	48	28	0.011191	0.44708	32	22	70	22	27	46	0.03881692	1	37.125	22.8125122								

13	6E+07	13_56972205	630	289	736	488	460	612	0.124093	1	297	182	220	69	240	145	2.22E-04	0.0180511	363.88	231.613864					5.7E+07	5.7E+07	0.50716	ID=promoter_14608;
13	6E+07	13_57487854	9	17	7	4	13	6	0.146758	1	1	0	2	10	1	6	0.13767373	1	6.25	5.70087713					5.7E+07	5.7E+07	0.74311	ID=promoter_14617;
13	6E+07	13_57711345	63	51	37	44	57	41	0.054559	1	44	47	14	47	46	31	0.38685872	1	43.375	14.0095631								
13	6E+07	13_58194314	184	46	176	134	115	155	0.528083	1	21	73	49	18	47	34	0.5433701	1	87.625	67.5847352	6E+07	5.8E+07	ID=ENSP0000352780-D3;GRIP1;;	GRIP1				
13	6E+07	13_58343867	88	45	50	65	67	58	0.235639	1	0	67	46	41	34	44	0.3405954	1	50.25	25.577613								
13	6E+07	13_58565140	52	188	76	102	120	89	0.00424	0.20559	125	173	148	70	149	109	0.16967851	1	116.75	50.0592506								
13	6E+07	13_58786237	289	135	216	167	212	192	0.033773	1	305	188	234	250	247	242	0.82946032	1	223	58.6563844	6E+07	5.9E+07	ID=ENSP0000258411-D7;WNT10A;NM_025216;	WNT10A				
13	6E+07	13_58975914	104	8	95	81	56	88	0.229003	1	34	17	0	0	26	0	5.84E-06	0.0007957	42.375	43.9900557					5.9E+07	5.9E+07	0.38375	ID=promoter_14640;
13	6E+07	13_59138153	54	38	83	96	46	90	0.017041	0.62279	64	11	8	6	38	7	7.12E-05	0.006869	45	35.0142828								
13	6E+07	13_59175130	32	13	45	30	23	38	0.427862	1	1	30	6	1	16	4	0.04223275	1	19.75	16.6282891								
13	6E+07	13_59248961	159	75	181	177	117	179	0.081111	1	66	92	21	44	79	33	4.11E-04	0.0300788	101.88	62.295236					5.9E+07	5.9E+07	0.66023	ID=promoter_14645;
13	6E+07	13_59302421	1702	786	1691	1260	1244	1476	0.956504	1	627	529	696	301	578	499	0.50695054	1	949	535.171535					5.9E+07	5.9E+07	0.60248	ID=promoter_14647;
13	6E+07	13_59513773	61	50	64	105	56	85	0.323738	1	17	27	47	14	22	31	0.31838102	1	48.125	29.849324					6E+07	6E+07	0.45844	ID=promoter_14653;
13	6E+07	13_59513929	115	81	190	207	98	199	3.95E-05	0.00396	93	83	38	53	88	46	0.00612522	0.2674545	107.5	61.0339484					6E+07	6E+07	0.45844	ID=promoter_14653;
13	6E+07	13_59591375	14	9	10	21	12	16	0.945172	1	30	9	16	9	20	13	0.60545249	1	14.75	7.51664819								
13	6E+07	13_60063455	203	59	154	159	131	157	0.983594	1	36	44	88	58	40	73	0.00211702	0.1154161	100.13	63.0497253								
13	6E+07	13_60376333	2	12	1	0	7	1	0.062633	1	8	8	10	1	8	6	0.92562801	1	5.25	4.74341649								
13	6E+07	13_60388861	316	138	199	297	227	248	0.74138	1	207	117	117	85	162	101	0.00701063	0.2971393	184.5	86.0564599					6E+07	6E+07	0.28223	ID=promoter_14672;
13	6E+07	13_61275031	0	72	22	5	36	14	0.001122	0.06988	48	12	11	1	30	6	8.03E-04	0.0527633	21.375	25.6567312								
13	6E+07	13_61518422	443	199	420	244	321	332	0.274239	1	154	83	156	61	119	109	0.99589784	1	220	143.052438	6E+07	6.2E+07	ID=ENSP0000273406;SRPRB;NM_021203;	SRPRB	6.2E+07	6.2E+07	0.22169	ID=promoter_14680;
13	6E+07	13_61536223	233	81	116	132	157	124	0.004131	0.20152	24	19	48	73	22	61	2.22E-05	0.0025318	90.75	70.1177581								
13	6E+07	13_61584187	41	1	73	84	21	79	3.37E-06	0.00045	0	4	10	2	2	6	0.31385293	1	26.875	34.6593113					6.2E+07	6.2E+07	0.16017	ID=promoter_14682;
13	6E+07	13_61641487	0	60	11	27	30	19	0.102296	1	21	65	63	8	43	36	0.90314284	1	31.875	26.7818569								
13	6E+07	13_61760006	1	6	4	0	4	2	0.597627	1	21	0	21	25	11	23	0.07426989	1	9.75	10.6871084	6E+07	6.2E+07	ID=ENSP0000296084;RYK;NM_002958;	RYK				
13	6E+07	13_61951016	159	115	180	140	137	160	0.999308	1	191	52	114	49	122	82	0.07753186	1	125	53.5697143					6.2E+07	6.2E+07	0.3102	ID=promoter_14687;
13	6E+07	13_62062910	125	95	156	108	110	132	0.98453	1	138	31	99	86	85	93	0.54275936	1	104.75	37.9313289								

13	6E+07	13_62175445	67	85	100	75	76	88	0.996601	1	69	30	66	75	50	71	0.06667316	1	70.875	19.981688	6E+07	6.2E+07	ID=ENSP00000309520;KY;;	KY				
13	6E+07	13_62186986	10	6	17	2	8	10	0.991043	1	22	6	1	0	14	1	0.00574567	0.2548307	8	7.9102104	6E+07	6.2E+07	ID=ENSP00000309520;KY;;	KY	6.2E+07	6.2E+07	0.42143	ID=promoter_14690;
13	6E+07	13_62188282	409	112	239	197	261	218	9.94E-04	0.06302	64	108	81	121	86	101	0.27107026	1	166.38	114.187737								
13	6E+07	13_62214963	79	38	83	62	59	73	0.954539	1	40	10	10	0	25	5	0.00263335	0.136745	40.25	32.199157								
13	6E+07	13_62387674	6	14	7	4	10	6	0.41703	1	1	30	10	5	16	8	0.35023049	1	9.625	9.11729126								
13	6E+07	13_62387762	96	54	97	112	75	105	0.503234	1	23	33	26	0	28	13	0.1098583	1	55.125	41.5225842								
13	6E+07	13_62531803	14	7	3	0	11	2	0.020291	0.71411	10	27	4	0	19	2	0.00190863	0.1054641	8.125	9.0307016								
13	6E+07	13_62629552	60	10	40	11	35	26	0.205248	1	3	19	14	0	11	7	0.73613127	1	19.625	20.3746166								
13	6E+07	13_62964046	24	2	23	4	13	14	0.975695	1	20	0	5	1	10	3	0.19568363	1	9.875	10.4940459								
13	6E+07	13_63243476	15	55	2	6	35	4	3.04E-07	#######	18	31	19	68	25	44	0.03372306	1	26.75	23.4078741								
13	6E+07	13_64465408	83	131	178	184	107	181	0.010482	0.4265	55	51	94	43	53	69	0.17466951	1	102.38	56.1323373								
13	6E+07	13_64512045	15	38	15	10	27	13	0.024366	0.82541	16	1	9	9	9	9	0.98716474	1	14.125	10.8026121	6E+07	6.5E+07	ID=ENSP00000366144-D2;SOX21;NM_007084;	SOX21	6.5E+07	6.5E+07	0.49352	ID=promoter_14703;
13	6E+07	13_64514384	489	196	301	341	343	321	0.014326	0.54141	98	85	176	41	92	109	0.22119263	1	215.88	151.699362					6.5E+07	6.5E+07	0.49352	ID=promoter_14703;
13	7E+07	13_65266782	11	0	0	0	6	0	0.029633	0.9649	35	0	0	0	18	0	2.38E-04	0.0190698	5.75	12.4298029								
13	7E+07	13_65840714	48	44	57	68	46	63	0.726232	1	132	61	66	1	97	34	2.56E-06	0.0003831	59.625	36.2015686								
13	7E+07	13_66638012	516	222	381	336	369	359	0.041137	1	155	192	156	50	174	103	0.00133439	0.0794529	251	149.911402								
13	7E+07	13_66818550	2	13	3	0	8	2	0.097885	1	18	4	23	1	11	12	0.92663959	1	8	8.78310066								
13	7E+07	13_67507431	2	2	5	2	2	4	0.823343	1	19	1	17	0	10	9	0.99792604	1	6	7.55928946	7E+07	6.8E+07	ID=ENSP00000286371-D2;ATP1B3;NM_001679;	ATP1B3				
13	7E+07	13_68223639	23	83	17	25	53	21	5.76E-05	0.00549	28	12	33	5	20	19	0.99708906	1	28.25	23.8731768								
13	7E+07	13_68349840	15	6	146	222	11	184	1.61E-28	#######	30	28	68	37	29	53	0.0112655	0.4337974	69	76.0169154								
13	7E+07	13_68529569	22	0	18	25	11	22	0.343316	1	17	21	37	5	19	21	0.85675254	1	18.125	11.5193936								
13	7E+07	13_70865026	3	68	22	24	36	23	0.069202	1	17	20	50	58	19	54	5.65E-05	0.0056257	32.75	22.883244								
13	7E+07	13_71952214	58	34	69	85	46	77	0.158759	1	171	109	109	24	140	67	3.46E-05	0.0037061	82.375	47.5092096					7.2E+07	7.2E+07	0.24065	ID=promoter_14752;
13	7E+07	13_71960286	136	57	147	65	97	106	0.880744	1	313	228	170	77	271	124	2.52E-10	7.96E-08	149.13	88.2001579					7.2E+07	7.2E+07	0.16731	ID=promoter_14754;
13	7E+07	13_72174460	9	9	10	0	9	5	0.390586	1	1	52	45	51	27	48	0.02250024	0.7507942	22.125	22.9187229								
13	7E+07	13_72582254	6	1	7	9	4	8	0.677894	1	35	9	17	3	22	10	0.16182597	1	10.875	10.8553805								
13	7E+07	13_72904523	13	12	9	6	13	8	0.344217	1	46	2	25	17	24	21	0.98147478	1	16.25	13.8950145								
13	7E+07	13_73992853	38	30	8	26	34	17	0.012579	0.48874	25	35	1	1	30	1	3.70E-06	0.0005267	20.5	14.9952373								
13	8E+07	13_75464731	1855	1445	4094	3749	1650	3922	########	#######	1199	889	3381	1036	1044	2209	1.19E-111	4.59E-108	2206	1317.23618					7.5E+07	7.5E+07	0.38286	ID=promoter_14779;
13	8E+07	13_76653892	11	41	15	32	26	24	0.697783	1	14	11	44	7	13	26	0.0678374	1	21.875	14.7593796								
13	8E+07	13_76826650	41	20	12	3	31	8	1.52E-04	0.01277	48	7	15	7	28	11	0.04532452	1	19.125	16.6256042								

13	8E+07	13_77774792	38	37	41	79	38	60	0.355118	1	91	52	45	60	72	53	0.44245022	1	55.375	20.0494923								
13	8E+07	13_77882418	58	66	44	57	62	51	0.168613	1	121	130	188	49	126	119	0.98947359	1	89.125	51.6096267								
13	8E+07	13_78169749	23	55	26	23	39	25	0.056901	1	12	31	17	1	22	9	0.10444527	1	23.5	15.7480157								
13	8E+07	13_78182531	956	357	630	472	657	551	4.05E-08	#######	137	249	206	247	193	227	0.05137251	1	406.75	273.004317	8E+07	7.8E+07	ID=ENSP00000337239;SCHIP1;NM_014575;	SCHIP1	7.8E+07	7.8E+07	0.57161	ID=promoter_14789;
13	8E+07	13_78402086	2	28	0	5	15	3	0.006447	0.2884	27	14	0	2	21	1	2.34E-04	0.0188551	9.75	11.8412113								
13	8E+07	13_78477788	1698	851	1439	1475	1275	1457	0.793365	1	1129	789	921	446	959	684	1.02E-06	0.0001692	1093.5	419.546013					7.8E+07	7.8E+07	0.52074	ID=promoter_14792;
13	8E+07	13_79701632	32	10	17	15	21	16	0.42382	1	10	28	24	12	19	18	0.99782856	1	18.5	8.48528137								
13	8E+07	13_80902423	14	28	1	8	21	5	0.001802	0.10311	20	13	23	53	17	38	0.00782445	0.3231698	20	15.8204208								
13	8E+07	13_81590214	1	37	1	2	19	2	2.36E-04	0.01868	6	13	33	2	10	18	0.2304087	1	11.875	14.855855								
13	8E+07	13_81853424	16	21	4	9	19	7	0.021688	0.74727	19	1	20	0	10	10	0.98574881	1	11.25	8.81151844								
13	8E+07	13_81976192	4	6	3	2	5	3	0.645778	1	6	22	3	22	14	13	0.99999086	1	8.5	8.45154255								
13	8E+07	13_82054229	79	4	66	41	42	54	0.905206	1	17	49	35	50	33	43	0.33537098	1	42.625	24.4185965								
13	8E+07	13_82355348	0	3	4	17	2	11	0.085797	1	1	8	26	44	5	35	3.99E-06	0.0005635	12.875	15.422039	8E+07	8.2E+07	ID=ENSP00000264674;EVI1;NM_001105078;	EVI1				
13	8E+07	13_82385061	238	79	195	182	159	189	0.992372	1	127	45	81	41	86	61	0.27621808	1	123.5	74.0771798	8E+07	8.2E+07	ID=ENSP00000376493;EVI1;;	EVI1	8.2E+07	8.2E+07	0.27934	ID=promoter_14802;
13	8E+07	13_82385503	226	58	216	406	142	311	2.98E-09	#######	103	70	109	97	87	103	0.24228825	1	160.63	117.307822					8.2E+07	8.2E+07	0.27934	ID=promoter_14802;
13	8E+07	13_83037466	0	3	0	8	2	4	0.823343	1	33	16	0	3	25	2	1.29E-04	0.0113348	7.875	11.5317884								
13	8E+07	13_83435896	615	985	1382	726	800	1054	0.046214	1	297	172	495	213	235	354	4.25E-08	9.48E-06	610.63	415.886982								
13	8E+07	13_83443403	133	79	138	99	106	119	0.947541	1	0	9	0	30	5	15	0.05518429	1	61	58.539364								
13	8E+07	13_83462686	418	216	355	246	317	301	0.031426	1	246	174	170	67	210	119	8.36E-05	0.0079261	236.5	109.96363					8.3E+07	8.3E+07	0.42558	ID=promoter_14812;
13	8E+07	13_83967026	893	377	668	563	635	616	0.0035	0.1767	312	260	326	192	286	259	0.9634428	1	448.88	238.730235					8.4E+07	8.4E+07	0.54745	ID=promoter_14815;
13	8E+07	13_84259874	31	21	21	17	26	19	0.285911	1	29	5	20	3	17	12	0.76881979	1	18.375	10.0418766	8E+07	8.4E+07	ID=ENSP00000338523;FNDC3B;NM_022763;	FNDC3B				
13	8E+07	13_84285102	2	48	9	3	25	6	5.64E-04	0.03913	28	3	3	4	16	4	0.04223275	1	12.5	16.758793	8E+07	8.4E+07	ID=ENSP00000338523;FNDC3B;NM_022763;	FNDC3B				
13	8E+07	13_84572648	29	55	15	17	42	16	2.94E-04	0.02276	69	26	24	35	48	30	0.23376449	1	33.75	18.9190003								
13	8E+07	13_84750092	25	34	18	19	30	19	0.102296	1	5	4	15	4	5	10	0.35527535	1	15.5	10.8627805					8.5E+07	8.5E+07	0.18248	ID=promoter_14819;
13	8E+07	13_84750359	19	2	24	11	11	18	0.682529	1	18	2	10	7	10	9	0.99792604	1	11.625	8.08768906								
13	9E+07	13_86133842	0	47	13	15	24	14	0.105787	1	11	1	25	19	6	22	0.00549069	0.2478586	16.375	14.9564845								
13	9E+07	13_86170221	591	577	977	1063	584	1020	1.12E-13	#######	656	467	1175	384	562	780	1.96E-12	7.95E-10	736.25	294.40048								
13	9E+07	13_87515167	0	50	11	5	25	8	0.002813	0.14803	1	11	29	1	6	15	0.10150651	1	13.5	17.5336411								
13	9E+07	13_88062392	4	39	6	4	22	5	0.001077	0.06758	2	19	6	25	11	16	0.51039395	1	13.125	13.2927209								

13	9E+07	13_88206285	50	26	37	73	38	55	0.607495	1	58	58	78	50	58	64	0.62952401	1	53.75	17.210877								
13	9E+07	13_89849232	10	40	20	24	25	22	0.614783	1	58	18	21	86	38	54	0.12669861	1	34.625	25.6622986								
13	9E+07	13_90461850	46	13	30	67	30	49	0.357262	1	62	10	0	11	36	6	6.62E-05	0.006417	29.875	25.6316629								
13	9E+07	13_90843439	1	11	4	1	6	3	0.462004	1	0	3	22	7	2	15	0.00415768	0.1961166	6.125	7.37636186								
13	9E+07	13_90916423	2644	2005	3185	3527	2325	3356	1.29E-13	#######	1662	1385	2012	1099	1524	1556	0.02759348	0.876892	2189.9	859.128202					9.1E+07	9.1E+07	0.19634	ID=promoter_14837;
13	9E+07	13_90926348	487	141	295	338	314	317	0.168913	1	162	127	169	129	145	149	0.67712223	1	231	130.387335					9.1E+07	9.1E+07	0.16415	ID=promoter_14839;
13	9E+07	13_91157811	15	22	26	9	19	18	0.809248	1	18	30	37	9	24	23	0.99354957	1	20.75	9.96780532								
13	9E+07	13_91700835	5	18	5	38	12	22	0.455056	1	30	17	8	0	24	4	0.00163693	0.0924179	15.125	13.3141976					9.2E+07	9.2E+07	0.54578	ID=promoter_14840;
13	9E+07	13_92114642	17	84	19	4	51	12	1.91E-07	#######	47	24	25	21	36	23	0.37391478	1	30.125	24.8218654								
13	9E+07	13_92223713	78	37	43	39	58	41	0.043015	1	0	39	1	2	20	2	0.00122165	0.0738454	29.875	27.2786547								
13	9E+07	13_92545902	3710	1795	3452	2858	2753	3155	0.672586	1	1854	1585	1610	1160	1720	1385	4.88E-04	0.0348216	2253	952.422625					9.3E+07	9.3E+07	0.22453	ID=promoter_14848;
13	9E+07	13_92709219	36	0	54	16	18	35	0.21203	1	11	30	65	4	21	35	0.09793111	1	27	23.5978207								
13	9E+07	13_93411046	21	51	41	19	36	30	0.380805	1	35	42	18	0	39	9	2.52E-04	0.020023	28.375	16.715583								
13	9E+07	13_93649485	130	55	47	45	93	46	5.01E-06	0.00064	54	32	38	2	43	20	0.03412779	1	50.375	36.3433527								
13	9E+07	13_93740340	686	232	573	537	459	555	0.89044	1	253	182	141	30	218	86	4.12E-11	1.44E-08	329.25	236.579041					9.4E+07	9.4E+07	0.48438	ID=promoter_14866;
13	9E+07	13_93741885	581	167	382	329	374	356	0.018458	0.66186	229	142	164	124	186	144	0.26685927	1	264.75	157.548859					9.4E+07	9.4E+07	0.48438	ID=promoter_14866;
13	9E+07	13_94202358	352	178	380	201	265	291	0.739659	1	167	102	97	88	135	93	0.08670533	1	195.63	113.188765					9.4E+07	9.4E+07	0.43782	ID=promoter_14871;
13	9E+07	13_94256337	20	45	19	16	33	18	0.027763	0.91318	85	11	50	55	48	53	0.67977483	1	37.625	25.6009905								
13	9E+07	13_94295290	16	20	30	9	18	20	0.986196	1	0	1	25	3	1	14	0.00214405	0.1161796	13	11.4642301					9.4E+07	9.4E+07	0.16473	ID=promoter_14873;
13	9E+07	13_94962225	72	51	78	47	62	63	0.72441	1	0	0	10	2	0	6	0.0393172	1	32.5	33.2350933								
13	1E+08	13_95436384	192	87	129	296	140	213	0.056463	1	73	38	32	9	56	21	0.00123801	0.0745848	107	96.3446195								
13	1E+08	13_95794201	2	8	13	1	5	7	0.955252	1	31	0	6	0	16	3	0.01878845	0.6479877	7.625	10.4872371								
13	1E+08	13_97588315	21	36	11	16	29	14	0.018844	0.67198	63	37	28	50	50	39	0.72066795	1	32.75	17.5478936								
13	1E+08	13_98114824	59	81	48	51	70	50	0.025685	0.8625	70	96	100	33	83	67	0.70075609	1	67.25	23.8192599								
13	1E+08	13_99066459	0	13	1	7	7	4	0.503048	1	66	1	12	2	34	7	3.68E-04	0.0274525	12.75	22.1149594								
13	1E+08	13_99172133	1	3	7	9	2	8	0.255957	1	16	8	19	1	12	10	0.96947392	1	8	6.65475126	1E+08	9.9E+07	ID=ENSP0000376227;TMEM44;;	TMEM44				
13	1E+08	13_99754172	3	37	10	3	20	7	0.013885	0.52965	84	13	31	26	49	29	0.15481988	1	25.875	26.707075								
13	1E+08	13_100729425	35	26	35	15	31	25	0.374336	1	0	0	7	12	0	10	0.00454609	0.2107253	16.25	14.3003496								
13	1E+08	13_100906375	165	54	81	63	110	72	4.92E-04	0.03466	78	32	47	11	55	29	0.04486397	1	66.375	46.0804808								
13	1E+08	13_101606537	21	4	5	6	13	6	0.146758	1	1	7	26	60	4	43	2.02E-08	4.75E-06	16.25	19.7538423								
13	1E+08	13_101683841	18	17	2	2	18	2	4.15E-04	0.03028	42	40	36	15	41	26	0.31320499	1	21.5	16.0712698								
13	1E+08	13_101741913	31	9	27	13	20	20	0.880283	1	0	0	0	0	0	0	1	1	10	12.7615494								
13	1E+08	13_101850720	13	78	22	23	46	23	0.002685	0.14289	68	18	20	17	43	19	0.02267705	0.755811	32.375	25.4049348								

13	1E+08	13_101933731	108	72	96	60	90	78	0.145429	1	12	32	42	1	22	22	0.96891521	1	52.875	38.2302704								
13	1E+08	13_102142644	186	36	111	140	111	126	0.968801	1	68	51	25	34	60	30	0.01955813	0.6674393	81.375	58.3632406								
13	1E+08	13_102697477	42	16	13	41	29	27	0.687522	1	8	0	8	0	4	4	0.99427496	1	16	16.6904592	1E+08	1E+08	ID=ENSP00000353452;MYLK;NM_053025;	MYLK				
13	1E+08	13_102789391	181	84	97	114	133	106	0.011569	0.45508	50	18	62	17	34	40	0.59203945	1	77.875	54.3308318	1E+08	1E+08	ID=ENSP00000353452;MYLK;NM_053025;	MYLK				
13	1E+08	13_102836871	185	30	45	47	108	46	1.50E-08	#######	46	26	24	15	36	20	0.17860656	1	52.25	54.9174705	1E+08	1E+08	ID=ENSP00000353452;MYLK;NM_053025;	MYLK				
13	1E+08	13_103000941	0	0	0	12	0	6	0.077487	1	2	16	15	3	9	9	0.98716474	1	6	7.07106781	1E+08	1E+08	ID=ENSP00000373153;PTPLB;NM_198402;	PTPLB				
13	1E+08	13_103088232	17	39	18	28	28	23	0.444971	1	61	36	41	21	49	31	0.24588806	1	32.625	14.8414237	1E+08	1E+08	ID=ENSP00000373153;PTPLB;NM_198402;	PTPLB				
13	1E+08	13_103179769	8	24	14	13	16	14	0.724026	1	37	20	40	1	29	21	0.68832436	1	19.625	13.5955613								
13	1E+08	13_103185058	24	20	5	6	22	6	0.002551	0.13706	0	0	11	2	0	7	0.02292696	0.7568963	8.5	9.13392421								
13	1E+08	13_103286806	18	133	14	13	76	14	2.43E-12	#######	0	86	18	19	43	19	0.02267705	0.755811	37.625	46.4971197								
13	1E+08	13_103310139	6	11	6	6	9	6	0.554309	1	17	4	4	0	11	2	0.06154634	1	6.75	5.14781507								
13	1E+08	13_103471632	46	22	60	81	34	71	0.019613	0.69609	27	59	27	4	43	16	0.00548623	0.2478586	40.75	25.1609107								
13	1E+08	13_103473795	113	337	96	81	225	89	9.16E-19	#######	344	259	318	153	302	236	0.14399692	1	212.63	113.634675								
13	1E+08	13_103617829	78	118	59	48	98	54	2.99E-05	0.0031	123	41	54	16	82	35	4.28E-04	0.0312589	67.125	37.2805484					1E+08	1E+08	0.3451	ID=promoter_14949;
13	1E+08	13_103677220	30	4	21	2	17	12	0.395844	1	0	2	0	1	1	1	0.99856566	1	7.5	11.4392807								
13	1E+08	13_105462590	0	24	3	0	12	2	0.011798	0.46127	14	0	11	0	7	6	0.99011599	1	6.5	8.9761589								
13	1E+08	13_105715276	13	13	22	2	13	12	0.835334	1	0	46	15	0	23	8	0.04560275	1	13.875	15.2450226								
13	1E+08	13_105915499	124	68	180	222	96	201	1.35E-05	0.00155	49	71	99	47	60	73	0.29388178	1	107.5	64.0156231								
13	1E+08	13_106061641	11	45	23	31	28	27	0.767529	1	49	21	20	4	35	12	0.00825069	0.3370693	25.5	15.5471633								
13	1E+08	13_107141121	29	30	31	21	30	26	0.525876	1	84	40	81	49	62	65	0.7863365	1	45.625	24.224766								
13	1E+08	13_107311987	4	54	11	7	29	9	8.94E-04	0.05763	39	58	51	32	49	42	0.93283003	1	32	22.1036519								
13	1E+08	13_107320628	16	74	45	46	45	46	0.806124	1	75	36	55	33	56	44	0.71330919	1	47.5	20.2060811								
13	1E+08	13_108449008	37	75	8	30	56	19	4.17E-06	0.00055	39	40	45	16	40	31	0.75504669	1	36.25	20.1689294								
13	1E+08	13_108659054	6	0	8	0	3	4	0.986002	1	24	0	13	1	12	7	0.61698677	1	6.5	8.51888658								
13	1E+08	13_109086848	12	37	14	0	25	7	0.001309	0.07852	33	11	56	13	22	35	0.13287223	1	22	18.3303028	1E+08	1.1E+08	ID=ENSP00000273398;ATP6V1A;NM_001690;	ATP6V1A				
13	1E+08	13_110365701	0	27	12	12	14	12	0.725787	1	38	19	21	26	29	24	0.91882689	1	19.375	11.5627172								
13	1E+08	13_111031780	1	28	13	12	15	13	0.725173	1	15	27	8	20	21	14	0.63082335	1	15.5	9.21179368								
13	1E+08	13_111181359	0	39	10	0	20	5	0.002998	0.15536	32	6	7	13	19	10	0.34022508	1	13.375	14.4808987								

13	1E+08	13_112357257	3732	2238	4140	4139	2985	4140	2.51E-11	#######	3029	2050	2251	1367	2540	1809	9.16E-17	5.13E-14	2868.3	1049.18713					1.1E+08	1.1E+08	0.29754	ID=promoter_15002;
13	1E+08	13_112679920	14	24	15	35	19	25	0.931133	1	47	31	41	7	39	24	0.28229872	1	26.75	14.1295031								
13	1E+08	13_112698875	0	6	9	4	3	7	0.596562	1	26	0	12	13	13	13	0.98151319	1	8.75	8.53145441								
13	1E+08	13_112972169	16	97	39	56	57	48	0.236028	1	51	139	88	66	95	77	0.68098723	1	69	38.2771846								
13	1E+08	13_113903441	7	2	5	11	5	8	0.86114	1	15	0	37	2	8	20	0.04735057	1	9.875	12.0527116	1E+08	1.1E+08	ID=ENSP000003059 88;ALCAM ;NM_001627;	ALCAM				
13	1E+08	13_115029692	2	10	2	7	6	5	0.851367	1	0	7	14	0	4	7	0.58783259	1	5.25	5.09201055								
13	1E+08	13_116056588	40	9	54	46	25	50	0.088056	1	68	60	120	40	64	80	0.20187084	1	54.625	31.7712246								
13	1E+08	13_116745997	65	149	51	81	107	66	1.62E-04	0.01352	223	79	161	97	151	129	0.79264345	1	113.25	58.8988237								
13	1E+08	13_118530336	13	41	13	10	27	12	0.014569	0.54886	62	24	36	38	43	37	0.94624198	1	29.625	17.9597765								
13	1E+08	13_119862045	0	10	5	37	5	21	0.023384	0.79837	17	9	6	1	13	4	0.12786863	1	10.625	11.93958								
13	1E+08	13_120402739	28	13	32	17	21	25	0.998746	1	56	15	30	0	36	15	0.02815151	0.8902666	23.875	16.762522								
13	1E+08	13_120509231	15	44	8	4	30	6	4.16E-05	0.00414	25	5	6	1	15	4	0.06170633	1	13.5	14.4913767								
13	1E+08	13_120792728	1	39	8	0	20	4	0.0012	0.07347	0	0	8	0	0	4	0.11562589	1	7	13.4057557								
13	1E+08	13_121521270	0	12	0	17	6	9	0.896349	1	1	10	4	14	6	9	0.65381141	1	7.25	6.81909085								
13	1E+08	13_121545130	13	9	12	5	11	9	0.722819	1	17	13	18	43	15	31	0.03250921	0.9922526	16.25	11.5727512					1.2E+08	1.2E+08	0.13627	ID=promoter_15041;
13	1E+08	13_123419516	18	18	25	3	18	14	0.509661	1	37	24	28	72	31	50	0.04928762	1	28.125	20.23743								
13	1E+08	13_125012967	4	72	16	27	38	22	0.026787	0.89052	1	16	64	0	9	32	6.35E-04	0.0429702	25	28.1272618								
13	1E+08	13_126479616	508	186	459	358	347	409	0.997781	1	217	110	117	34	164	76	2.28E-06	0.0003489	248.63	173.626806					1.3E+08	1.3E+08	0.24954	ID=promoter_15051;
13	1E+08	13_126732853	1	14	3	30	8	17	0.371907	1	15	8	17	7	12	12	0.98292303	1	11.875	9.29573635								
13	1E+08	13_127565115	149	154	83	93	152	88	5.15E-07	#######	45	87	62	17	66	40	0.10191864	1	86.25	47.312789								
13	1E+08	13_128014313	92	154	189	138	123	164	0.561029	1	237	124	396	199	181	298	5.12E-09	1.34E-06	191.13	94.6247287								
13	1E+08	13_128224203	6	19	9	6	13	8	0.344217	1	17	0	0	15	9	8	0.99626583	1	9	7.36788398								
13	1E+08	13_128870525	23	0	22	25	12	24	0.311519	1	9	0	5	1	5	3	0.8350419	1	10.625	10.9666703								
13	1E+08	13_129513533	4	37	7	9	21	8	0.017144	0.62492	3	0	5	19	2	12	0.01895406	0.6519669	10.5	12.1184629								
13	1E+08	13_130742017	17	40	16	24	29	20	0.181062	1	64	6	2	5	35	4	1.38E-05	0.0016769	21.75	21.035684								
13	1E+08	13_131427748	16	26	16	8	21	12	0.1273	1	29	7	5	0	18	3	0.00813808	0.3342859	13.375	10.2530483								
13	1E+08	13_133785824	33	25	14	16	29	15	0.030102	0.97706	20	23	45	37	22	41	0.02639021	0.8480056	26.625	10.8092223								
13	1E+08	13_133989381	4	35	6	9	20	8	0.026297	0.87605	4	20	34	5	12	20	0.26508384	1	14.625	13.3302824								
13	1E+08	13_134934358	5	33	13	22	19	18	0.809248	1	34	0	17	96	17	57	4.14E-06	0.0005807	27.5	30.185143								
13	1E+08	13_135225734	3045	1587	3430	2190	2316	2810	0.483819	1	1496	1243	1345	725	1370	1035	4.16E-06	0.0005823	1882.6	934.657605					1.4E+08	1.4E+08	0.18093	ID=promoter_15073;
13	1E+08	13_135305456	7	3	4	10	5	7	0.955252	1	1	31	1	12	16	7	0.23724011	1	8.625	9.89859297								
13	1E+08	13_135416856	35	31	33	35	33	34	0.868497	1	45	47	32	5	46	19	0.00967	0.3824351	32.875	12.7440462								
13	1E+08	13_135530588	65	99	35	51	82	43	6.47E-05	0.0061	8	86	94	16	47	55	0.50123132	1	56.75	35.131386								

13	1E+08	13_136023991	15	62	12	18	39	15	5.75E-04	0.03976	23	18	15	9	21	12	0.40192224	1	21.5	16.8946314	1E+08	1.4E+08	ID=ENSP0000284987;ADAMTS5;NM_007038;	ADAMTS5	1.4E+08	1.4E+08	0.66862	ID=promoter_15078;
13	1E+08	13_136052884	0	19	13	23	10	18	0.549586	1	94	41	48	38	68	43	0.14210593	1	34.5	28.7898792	1E+08	1.4E+08	ID=ENSP0000284987;ADAMTS5;NM_007038;	ADAMTS5				
13	1E+08	13_136991231	1	2	8	0	2	4	0.823343	1	1	29	16	31	15	24	0.24434768	1	11	12.8730061								
13	1E+08	13_137531192	47	96	45	45	72	45	0.003445	0.1741	80	22	65	43	51	54	0.79293336	1	55.375	23.6095471								
13	1E+08	13_137673125	0	35	11	2	18	7	0.033499	1	22	14	0	2	18	1	9.25E-04	0.05912	10.75	12.5896783								
13	1E+08	13_137724225	29	23	20	30	26	25	0.776821	1	0	7	18	1	4	10	0.21760186	1	16	11.9283576								
13	1E+08	13_137943193	2462	1410	2347	2061	1936	2204	0.631291	1	1447	1096	1435	655	1272	1045	0.0151123	0.5480081	1614.1	626.298979								
13	1E+08	13_138166727	4	1	14	4	3	9	0.343967	1	8	7	0	2	8	1	0.0848225	1	5	4.5669621								
13	1E+08	13_138167393	7	4	19	8	6	14	0.355886	1	10	2	0	0	6	0	0.06196836	1	6.25	6.34147347					1.4E+08	1.4E+08	0.11953	ID=promoter_15085;
13	1E+08	13_138289819	76	16	41	40	46	41	0.439359	1	55	27	9	29	41	19	0.03884844	1	36.625	21.5733798	1E+08	1.4E+08	ID=ENSP0000286827;TIAM1;NM_003253;	TIAM1	1.4E+08	1.4E+08	0.27973	ID=promoter_15087;
13	1E+08	13_138445207	6	13	0	8	10	4	0.162164	1	28	9	9	1	19	5	0.02799778	0.8875672	9.25	8.71370021								
13	1E+08	13_138469843	33	73	46	30	53	38	0.064845	1	15	33	26	1	24	14	0.38067267	1	32.125	21.2766639					1.4E+08	1.4E+08	0.70838	ID=promoter_15091;
13	1E+08	13_138477981	41	49	12	13	45	13	8.88E-06	0.00108	23	26	22	16	25	19	0.80752973	1	25.25	13.3068189								
13	1E+08	13_138641476	208	58	170	107	133	139	0.635674	1	21	0	0	2	11	1	0.02232329	0.7455867	70.75	82.2691576	1E+08	1.4E+08	ID=ENSP0000371931;SYNJ1;NM_003895;	SYNJ1				
13	1E+08	13_138804049	9	22	4	0	16	2	0.001284	0.07728	3	9	17	0	6	9	0.65381141	1	8	8								
13	1E+08	13_139342082	5	8	5	8	7	7	0.956352	1	12	4	6	7	8	7	0.9937864	1	6.875	2.53193884					1.4E+08	1.4E+08	0.78329	ID=promoter_15118;
13	1E+08	13_139343677	686	352	537	501	519	519	0.036555	1	519	263	224	71	391	148	1.02E-20	7.10E-18	394.13	201.756882					1.4E+08	1.4E+08	0.78329	ID=promoter_15118;
13	1E+08	13_139399525	131	93	146	125	112	136	0.963837	1	216	157	186	31	187	109	5.28E-04	0.0368652	135.63	56.6440956								
13	1E+08	13_139929062	17	6	9	16	12	13	0.980472	1	32	28	19	7	30	13	0.06382438	1	16.75	9.52815377								
13	1E+08	13_140007062	7	19	0	1	13	1	0.002067	0.11504	16	11	0	26	14	13	0.99999086	1	10	9.73946317								
13	1E+08	13_140040142	0	4	6	7	2	7	0.358464	1	0	16	0	16	8	8	0.98858271	1	6.125	6.68553876								
13	1E+08	13_140310879	1	0	7	6	1	7	0.163779	1	10	9	7	1	10	4	0.34296513	1	5.125	3.90741054	1E+08	1.4E+08	ID=ENSP0000382104;DOPEY2;NM_005128;	DOPEY2				
13	1E+08	13_140332338	2	23	0	14	13	7	0.234546	1	25	2	16	0	14	8	0.54462139	1	10.25	10.511898	1E+08	1.4E+08	ID=ENSP0000382104;DOPEY2;NM_005128;	DOPEY2				
13	1E+08	13_140482396	48	26	5	27	37	16	0.002386	0.12916	5	12	28	46	9	37	6.58E-05	0.0063912	24.625	16.6984815	1E+08	1.4E+08	ID=ENSP0000315700;CHAF1B;NM_005441;	CHAF1B				
13	1E+08	13_140730519	17	41	23	13	29	18	0.097648	1	62	7	28	14	35	21	0.28311174	1	25.625	18.078697								

13	1E+08	13_140939763	789	331	0	0	560	0	########	#######	365	194	0	2	280	1	1.85E-56	3.62E-53	210.13	280.236348	1.4E+08	1.4E+08	0.47287	ID=promoter_15163;
13	1E+08	13_140958946	8	1	0	2	5	1	0.186119	1	24	12	15	4	18	10	0.42261869	1	8.25	8.32809359				
13	1E+08	13_140959391	6	9	11	0	8	6	0.705675	1	18	30	15	1	24	8	0.03264408	0.9940318	11.25	9.82344135				
13	1E+08	13_141297028	19	7	11	1	13	6	0.146758	1	3	7	7	11	5	9	0.48000906	1	8.25	5.54848757				
13	1E+08	13_141357090	1552	697	1579	1561	1125	1570	4.92E-05	0.00477	1227	638	891	361	933	626	2.65E-09	7.22E-07	1063.3	480.661078				
13	1E+08	13_141460380	655	227	487	320	441	404	0.001545	0.09128	319	190	123	88	255	106	9.28E-12	3.45E-09	301.13	191.029869	1.4E+08	1.4E+08	0.65861	ID=promoter_15179;
13	1E+08	13_141625093	41	51	17	9	46	13	5.45E-06	0.00069	48	0	2	50	24	26	0.85918702	1	27.25	22.4228837				
13	1E+08	13_142118109	2	11	9	12	7	11	0.831726	1	0	35	26	0	18	13	0.78939737	1	11.875	12.6653351				
13	1E+08	13_142427414	65	68	43	40	67	42	0.005325	0.24715	128	93	107	115	111	111	0.85271812	1	82.375	33.2090242				
13	1E+08	13_142429273	91	38	77	97	65	87	0.722838	1	28	5	13	0	17	7	0.1782776	1	43.625	39.3080781				
13	1E+08	13_142579600	1	34	1	2	18	2	4.15E-04	0.03028	0	8	15	19	4	17	0.01069978	0.4139111	10	12.023786				
13	1E+08	13_142597035	1	1	2	10	1	6	0.241978	1	0	1	0	25	1	13	0.0036419	0.1775497	5	8.71779789				
13	1E+08	13_142649344	28	108	76	108	68	92	0.670931	1	95	45	238	187	70	213	6.98E-19	4.57E-16	110.63	70.354079				
13	1E+08	13_142653284	288	160	204	236	224	220	0.173094	1	96	40	87	33	68	60	0.96183266	1	143	93.9133339	1.4E+08	1.4E+08	0.15472	ID=promoter_15199;
13	1E+08	13_142850074	18	59	5	5	39	5	1.03E-07	#######	5	14	25	5	10	15	0.49251655	1	17	18.5703296				
13	1E+08	13_142931764	55	48	37	33	52	35	0.036495	1	63	1	7	1	32	4	5.18E-05	0.0052176	30.625	24.795953				
13	1E+08	13_142960120	2318	3999	2629	2385	3159	2507	1.20E-47	#######	5757	2608	3360	1784	4183	2572	7.44E-60	1.52E-56	3105	1267.94322	1.4E+08	1.4E+08	0.17476	ID=promoter_15215;
13	1E+08	13_142981524	68	70	42	49	69	46	0.01085	0.43721	22	69	34	19	46	27	0.16395114	1	46.625	20.9075345				
13	1E+08	13_143183655	6	34	7	1	20	4	0.0012	0.07347	2	3	15	29	3	22	3.46E-04	0.0261529	12.125	12.7888065	1.4E+08	1.4E+08	0.11662	ID=promoter_15227;
13	1E+08	13_143239597	76	76	37	71	76	54	0.017421	0.6315	80	61	51	25	71	38	0.02171687	0.7323563	59.625	20.2409592				
13	1E+08	13_143272726	191	66	120	100	129	110	0.047651	1	62	10	12	2	36	7	1.63E-04	0.0138451	70.375	65.1458528				
13	1E+08	13_143274185	512	237	407	351	375	379	0.12321	1	69	50	101	3	60	52	0.93875817	1	216.25	189.179242	1.4E+08	1.4E+08	0.15846	ID=promoter_15230;
13	1E+08	13_143323547	0	13	1	0	7	1	0.062633	1	0	17	18	11	9	15	0.36943449	1	7.5	8.05339325				
13	1E+08	13_143447646	3	33	14	24	18	19	0.949854	1	28	19	14	21	24	18	0.79306625	1	19.5	9.33503386	1.4E+08	1.4E+08	0.17551	ID=promoter_15253;
13	1E+08	13_143456481	16	49	32	14	33	23	0.152883	1	88	30	18	17	59	18	8.01E-05	0.0076297	33	25.133927				
13	1E+08	13_143514219	0	0	0	0	0	0	1	1	58	11	52	20	35	36	0.90853531	1	17.625	24.2129688				
13	1E+08	13_143522463	576	492	458	592	534	525	0.015998	0.59301	537	256	272	204	397	238	5.79E-07	0.0001019	423.38	155.656157				
13	1E+08	13_143569990	4	35	8	30	20	19	0.804666	1	25	21	22	23	23	23	0.96752545	1	21	10.3923048				
13	1E+08	13_143583495	9	26	10	9	18	10	0.152558	1	0	0	3	1	0	2	0.34003807	1	7.25	8.6808492				
13	1E+08	13_143642259	76	4	29	20	40	25	0.042829	1	55	42	11	22	49	17	0.0013565	0.0806014	32.375	24.0293719				
13	1E+08	13_143689881	4	3	10	6	4	8	0.677894	1	33	29	8	1	31	5	2.16E-04	0.0176336	11.75	12.2561938	1.4E+08	1.4E+08	0.1265	ID=promoter_15275;
13	1E+08	13_143731546	3	0	10	13	2	12	0.058554	1	0	0	5	25	0	15	3.07E-04	0.0235795	7	8.75051019				
13	1E+08	13_143786623	34	44	38	14	39	26	0.077552	1	78	20	40	66	49	53	0.73830238	1	41.75	21.4725739				
13	1E+08	13_143846265	59	132	80	29	96	55	8.55E-05	0.00772	162	119	113	72	141	93	0.03769473	1	95.75	43.3581431				

13	1E+08	13_144040591	24	40	15	24	32	20	0.080425	1	0	0	34	1	0	18	6.08E-05	0.0059624	17.25	15.8271918								
13	1E+08	13_144106233	2	17	5	8	10	7	0.570701	1	36	0	12	3	18	8	0.20901227	1	10.375	11.7708781					1.4E+08	1.4E+08	0.13107	ID=promoter_15300;
13	1E+08	13_144222591	0	0	0	4	0	2	0.426327	1	0	0	14	23	0	19	3.54E-05	0.003758	5.125	8.70857541	1E+08	1.4E+08	ID=ENSP00000332979;BACE2;NM_012105;	BACE2	1.4E+08	1.4E+08	0.15537	ID=promoter_15307;
13	1E+08	13_144296184	36	31	20	17	34	19	0.029997	0.97543	25	50	35	41	38	38	0.94691673	1	31.875	11.0121686					1.4E+08	1.4E+08	0.11807	ID=promoter_15316;
13	1E+08	13_144308886	127	43	116	109	85	113	0.683339	1	56	47	84	19	52	52	0.92807817	1	75.125	39.5092665	1E+08	1.4E+08	ID=ENSP00000381639;;					
13	1E+08	13_144445298	329	125	228	173	227	201	0.014382	0.54297	91	81	154	35	86	95	0.49897439	1	152	93.1435145	1E+08	1.4E+08	ID=ENSP00000381588;TMPRSS2;;	TMPRSS2				
13	1E+08	13_144515170	39	19	16	22	29	19	0.13502	1	28	17	70	31	23	51	0.00162933	0.0923909	30.25	17.8705664								
13	1E+08	13_144588043	3	14	0	10	9	5	0.390586	1	35	0	7	0	18	4	0.01933304	0.6616555	8.625	11.8555292								
13	1E+08	13_144717484	11	10	10	21	11	16	0.859437	1	27	17	3	3	22	3	0.001462	0.085933	12.75	8.43038721	1E+08	1.4E+08	ID=ENSP00000330161;RIPK4;;	RIPK4	1.4E+08	1.4E+08	0.44662	ID=promoter_15361;
13	1E+08	13_144757121	308	206	203	221	257	212	6.95E-04	0.04672	95	21	26	27	58	27	0.0106017	0.4114546	138.38	110.220477					1.4E+08	1.4E+08	0.48637	ID=promoter_15375;
13	1E+08	13_144758645	14	9	4	14	12	9	0.592799	1	2	13	2	3	8	3	0.3843038	1	7.625	5.47559521					1.4E+08	1.4E+08	0.48637	ID=promoter_15375;
13	1E+08	13_144767246	36	0	43	44	18	44	0.027286	0.89956	0	9	0	1	5	1	0.30486099	1	16.625	20.5352484	1E+08	1.4E+08	ID=ENSP00000269844;PRDM15;NM_022115;	PRDM15	1.4E+08	1.4E+08	0.26927	ID=promoter_15378;
13	1E+08	13_144777958	11	11	6	1	11	4	0.10516	1	0	1	14	5	1	10	0.01766414	0.6154554	6.125	5.35690476	1E+08	1.4E+08	ID=ENSP00000269844;PRDM15;NM_022115;	PRDM15	1.4E+08	1.4E+08	0.24029	ID=promoter_15383;
13	1E+08	13_144832310	25	22	9	7	24	8	0.004476	0.21437	1	6	0	0	4	0	0.15659576	1	8.75	9.73579551								
13	1E+08	13_144858494	42	0	5	18	21	12	0.1273	1	0	0	0	0	0	0	1	1	8.125	15.0469503								
13	1E+08	13_144860977	16	43	8	9	30	9	5.55E-04	0.03862	9	2	9	2	6	6	0.99142475	1	12.25	13.1990259					1.4E+08	1.4E+08	0.26179	ID=promoter_15407;
13	1E+08	13_144905385	18	38	27	21	28	24	0.526767	1	5	4	11	31	5	21	0.00386645	0.1846363	19.375	12.2932909								
13	1E+08	13_144907819	31	60	42	47	46	45	0.686795	1	52	38	68	39	45	54	0.43957352	1	47.125	12.3107096					1.4E+08	1.4E+08	0.17642	ID=promoter_15418;
13	1E+08	13_144908954	144	28	71	114	86	93	0.862734	1	13	0	8	2	7	5	0.9051169	1	47.5	55.7340624								
13	1E+08	13_144913583	50	72	106	93	61	100	0.117303	1	6	8	17	8	7	13	0.3188207	1	45	41.0957071								
13	1E+08	13_144943504	18	27	15	4	23	10	0.024151	0.81985	40	13	32	16	27	24	0.98883926	1	20.625	11.5874008					1.4E+08	1.4E+08	0.16583	ID=promoter_15430;
13	1E+08	13_144959791	83	74	77	64	79	71	0.263507	1	16	28	30	49	22	40	0.03530987	1	52.625	25.5674877					1.4E+08	1.4E+08	0.21246	ID=promoter_15433;
13	1E+08	13_144968481	81	121	72	70	101	71	0.003756	0.18662	4	42	40	49	23	45	0.01171564	0.4461969	59.875	34.6139423					1.4E+08	1.4E+08	0.47968	ID=promoter_15435;
13	1E+08	13_144981905	4	11	1	17	8	9	0.996316	1	0	0	10	2	0	6	0.0393172	1	5.625	6.30051018	1E+08	1.5E+08	ID=ENSP00000381385;SLC37A1;;	SLC37A1				
13	1E+08	13_145030182	5	43	7	18	24	13	0.071032	1	72	54	15	16	63	16	4.66E-06	0.0006466	28.75	24.5400547								

13	1E+08	13_145048375	4	0	0	7	2	4	0.823343	1	46	34	12	0	40	6	1.20E-05	0.001486	12.875	17.5453494					1.5E+08	1.5E+08	0.89363	ID=promoter_15458;
13	1E+08	13_145054041	182	187	231	269	185	250	0.343907	1	488	478	456	462	483	459	0.93024448	1	344.13	138.588226					1.5E+08	1.5E+08	0.52372	ID=promoter_15460;
13	1E+08	13_145060755	180	64	149	133	122	141	0.992875	1	85	39	31	23	62	27	0.00359287	0.1775262	88	59.3656948					1.5E+08	1.5E+08	0.76241	ID=promoter_15462;
13	1E+08	13_145081365	0	25	10	8	13	9	0.46931	1	6	15	27	21	11	24	0.05309113	1	14	9.65105472					1.5E+08	1.5E+08	0.1481	ID=promoter_15467;
13	1E+08	13_145086430	250	61	95	122	156	109	1.50E-04	0.01267	89	48	70	50	69	60	0.93635155	1	98.125	66.2537061								
13	1E+08	13_145090797	10	0	1	13	5	7	0.955252	1	16	8	5	1	12	3	0.09316162	1	6.75	5.99404466					1.5E+08	1.5E+08	0.5761	ID=promoter_15469;
13	1E+08	13_145100610	37	19	39	39	28	39	0.785678	1	0	12	15	9	6	12	0.2888538	1	21.25	15.1633958								
13	1E+08	13_145102185	100	40	66	85	70	76	0.896835	1	72	39	103	20	56	62	0.62817963	1	65.625	30.3359169					1.5E+08	1.5E+08	0.17374	ID=promoter_15473;
13	1E+08	13_145114654	561	180	284	288	371	286	5.37E-07	#######	304	153	236	132	229	184	0.35155452	1	267.25	135.375404								
13	1E+08	13_145132038	735	315	797	616	525	707	0.056259	1	282	163	218	153	223	186	0.56737708	1	409.88	263.807526					1.5E+08	1.5E+08	0.6149	ID=promoter_15479;
13	1E+08	13_145133786	41	53	33	34	47	34	0.095427	1	17	27	26	21	22	24	0.85866092	1	31.5	11.5387546					1.5E+08	1.5E+08	0.6149	ID=promoter_15479;
13	1E+08	13_145134070	17	7	12	13	12	13	0.980472	1	10	10	46	3	10	25	0.022088	0.7420679	14.75	13.2853303					1.5E+08	1.5E+08	0.6149	ID=promoter_15479;
13	1E+08	13_145139182	9	9	6	0	9	3	0.131542	1	12	1	4	1	7	3	0.51857467	1	5.25	4.46414285					1.5E+08	1.5E+08	0.63624	ID=promoter_15481;
13	1E+08	13_145141266	280	74	120	121	177	121	2.19E-05	0.00238	101	68	82	22	85	52	0.0591703	1	108.5	76.3188799					1.5E+08	1.5E+08	0.63624	ID=promoter_15481;
13	1E+08	13_145170412	15	15	21	3	15	12	0.610117	1	3	0	15	3	2	9	0.08177705	1	9.375	7.92712342					1.5E+08	1.5E+08	0.58787	ID=promoter_15487;
13	1E+08	13_145177772	5	20	6	12	13	9	0.46931	1	0	0	2	19	0	11	0.00265095	0.136745	8	8.08879295					1.5E+08	1.5E+08	0.50252	ID=promoter_15490;
13	1E+08	13_145183507	1	7	8	17	4	13	0.177139	1	13	0	12	3	7	8	0.92121674	1	7.625	6.0930288					1.5E+08	1.5E+08	0.82661	ID=promoter_15492;
13	1E+08	13_145184265	28	27	4	11	28	8	6.70E-04	0.04525	32	0	9	0	16	5	0.08348313	1	13.875	13.1739733					1.5E+08	1.5E+08	0.82661	ID=promoter_15492;
13	1E+08	13_145188090	961	1023	840	1026	992	933	7.71E-06	0.00095	45	45	34	32	45	33	0.59130718	1	500.75	496.926482					1.5E+08	1.5E+08	0.82661	ID=promoter_15492;
13	1E+08	13_145205047	32	5	33	28	19	31	0.523429	1	7	25	14	2	16	8	0.35023049	1	18.25	12.7138844								
13	1E+08	13_145219906	0	12	17	26	6	22	0.0331	1	0	1	0	0	1	0	0.62906424	1	7	10.1277554								
14	1E+06	14_12075	85	25	18	6	55	12	2.40E-08	#######	12	0	18	0	6	9	0.65381141	1	20.5	27.5577316								
14	1E+06	14_12228	1	25	12	10	13	11	0.725732	1	0	8	11	1	4	6	0.75330063	1	8.5	8.26351707								
14	1E+06	14_12239	79	32	33	64	56	49	0.322953	1	99	58	7	4	79	6	3.48E-13	1.54E-10	47	33.8230691								
14	1E+06	14_12263	0	6	1	0	3	1	0.508575	1	12	0	19	2	6	11	0.39136385	1	5	7.0305456								
14	1E+06	14_12543	5	27	9	2	16	6	0.042495	1	1	16	6	1	9	4	0.4571786	1	8.375	9.03860767								
14	1E+06	14_1366312	5077	4560	4968	6207	4819	5588	0.843478	1	4460	4043	7596	2350	4252	4973	9.97E-28	9.95E-25	4907.6	1538.009	1E+06	1366401	ID=ENSP00000252506;GADD45G;NM_006705;	GADD45G	1363588	1367170	0.76926	ID=promoter_15530;
14	1E+06	14_14235	1	31	7	7	16	7	0.076817	1	1	0	1	3	1	2	0.81304206	1	6.375	10.3224789								
14	1E+06	14_14235	47	38	64	61	43	63	0.530095	1	36	71	65	42	54	54	0.92541771	1	53	13.7529218								
14	2E+06	14_15254	32	11	27	15	22	21	0.795425	1	0	0	9	0	0	5	0.06742467	1	11.75	12.3952756								
14	2E+06	14_15348	10	13	7	10	12	9	0.592799	1	2	35	19	22	19	21	0.85675254	1	14.75	10.3613292								
14	2E+06	14_22232	0	37	7	2	19	5	0.004955	0.23359	9	32	32	18	21	25	0.69849299	1	17.125	14.7787251								
14	2E+06	14_24790	3	4	1	5	4	3	0.840045	1	19	13	7	0	16	4	0.04223275	1	6.5	6.45865975								

14	3E+06	14_2644526	148	84	62	56	116	59	5.22E-07	#######	0	5	10	3	3	7	0.3829009	1	46	52.2985933	3E+06	2671856	ID=ENSP0000364907;SYK;NM_003177;	SYK	2644465	2645074	0.12711	ID=promoter_15535;
14	3E+06	14_29505	1	10	14	24	6	19	0.087167	1	52	9	18	3	31	11	0.01758669	0.6154554	16.375	16.2387324								
14	3E+06	14_30557	2	45	7	21	24	14	0.105787	1	30	12	19	35	21	27	0.52862919	1	21.375	14.6085836								
14	3E+06	14_30567	10	1	11	9	6	10	0.79153	1	16	24	17	30	20	24	0.69398062	1	14.75	9.1300133								
14	3E+06	14_3439740	262	198	321	226	230	274	0.985047	1	260	89	120	47	175	84	2.92E-06	0.0004312	190.38	95.7331671					3438054	3440906	0.68111	ID=promoter_15547;
14	3E+06	14_34448	2	13	7	1	8	4	0.357158	1	48	3	7	25	26	16	0.43033428	1	13.25	16.0601548								
14	4E+06	14_36888	7	0	1	0	4	1	0.312866	1	31	0	4	0	16	2	0.00719486	0.3031284	5.375	10.6628527								
14	4E+06	14_37168	49	39	19	41	44	30	0.067875	1	0	19	11	0	10	6	0.69729498	1	22.25	18.8433391								
14	4E+06	14_38637	28	26	27	13	27	20	0.290795	1	38	2	17	2	20	10	0.26942782	1	19.125	12.9442486								
14	4E+06	14_42961	62	34	38	59	48	49	0.791123	1	1	33	40	28	17	34	0.02964294	0.9232309	36.875	18.9769597								
14	5E+06	14_47205	19	82	7	17	51	12	1.91E-07	#######	46	36	28	20	41	24	0.19624561	1	31.875	23.5762441								
14	5E+06	14_47295	38	53	22	18	46	20	5.83E-04	0.04027	135	25	84	39	80	62	0.5700955	1	51.75	39.7555028								
14	6E+06	14_61039	10	81	86	88	46	87	0.030208	0.97981	12	48	165	177	30	171	1.28E-24	1.09E-21	83.375	62.3261869								
14	6E+06	14_61287	210	126	170	135	168	153	0.075963	1	198	129	135	32	164	84	4.25E-05	0.0044359	141.88	54.989447								
14	6E+06	14_62280	0	17	2	1	9	2	0.058587	1	9	11	1	3	10	2	0.09294023	1	5.5	6.14119579								
14	6E+06	14_6336768	2741	975	2522	2289	1858	2406	0.005884	0.26823	1211	1084	1031	527	1148	779	1.29E-10	4.24E-08	1547.5	835.628762					6335948	6337458	0.4476	ID=promoter_15586;
14	7E+06	14_65039	50	54	45	43	52	44	0.277399	1	135	42	52	1	89	27	6.01E-07	0.0001053	52.75	37.2702486								
14	7E+06	14_69026	120	53	95	119	87	107	0.947523	1	99	12	39	4	56	22	0.00205431	0.1123834	67.625	46.7392081								
14	7E+06	14_6916202	557	199	396	377	378	387	0.170158	1	325	213	145	201	269	173	8.11E-04	0.0530935	301.63	138.044442					6915450	6916688	0.24769	ID=promoter_15611;
14	7E+06	14_69237	164	61	465	280	113	373	9.45E-23	#######	0	49	78	29	25	54	0.0015237	0.0882297	140.75	158.602603								
14	7E+06	14_69574	29	129	46	51	79	49	0.001748	0.10098	3	47	78	73	25	76	3.38E-07	6.36E-05	57	37.489046								
14	7E+06	14_69914	9	16	24	16	13	20	0.747034	1	57	13	40	32	35	36	0.90853531	1	25.875	16.2782721								
14	7E+06	14_70093	19	3	26	30	11	28	0.082798	1	12	17	18	0	15	9	0.58227053	1	15.625	10.3501208								
14	7E+06	14_70869	38	42	27	56	40	42	0.881842	1	41	49	27	7	45	17	0.00491937	0.2246966	35.875	15.2918045								
14	7E+06	14_71185	47	90	121	112	69	117	0.050737	1	169	133	101	138	151	120	0.45167935	1	113.88	36.3492484								
14	7E+06	14_71361	8	5	8	2	7	5	0.693374	1	34	7	11	7	21	9	0.13945494	1	10.25	9.93910028								
14	8E+06	14_75058	12	32	24	13	22	19	0.617881	1	17	13	16	1	15	9	0.58227053	1	16	9.1025899								
14	9E+06	14_90839	54	13	18	31	34	25	0.201935	1	17	9	15	3	13	9	0.79589064	1	20	15.91944								
14	9E+06	14_90999	332	255	241	224	294	233	4.03E-05	0.00403	180	73	212	152	127	182	7.40E-04	0.0492332	208.63	76.6139441								
14	9E+06	14_9120820	63	43	51	82	53	67	0.920361	1	45	34	62	3	40	33	0.88417976	1	47.875	23.4242701	9E+06	9125218	ID=ENSP0000311997-D2;NEFH;NM_021076;	NEFH	9119128	9122073	0.62965	ID=promoter_15647;
14	9E+06	14_92010	11	24	9	5	18	7	0.033499	1	0	16	7	1	8	4	0.59180275	1	9.125	7.95410945								
14	9E+06	14_93995	10	27	7	5	19	6	0.010861	0.43721	0	9	14	0	5	7	0.77775394	1	9	8.71779789								
14	1E+07	14_98643	38	79	17	86	59	52	0.320882	1	78	16	45	24	47	35	0.61680793	1	47.875	29.2156392								
14	1E+07	14_10101710	20	77	53	22	49	38	0.155939	1	1	15	11	29	8	20	0.04735057	1	28.5	24.8193473								
14	1E+07	14_10488225	5	14	17	4	10	11	0.989255	1	22	0	8	4	11	6	0.57179039	1	9.25	7.61108215								
14	1E+07	14_10601569	15	26	9	7	21	8	0.017144	0.62492	0	3	20	1	2	11	0.03110435	0.956324	10.125	9.41787814	1E+07	1.1E+07	ID=ENSP0000343690-D2;DPYSL3;;	DPYSL3				
14	1E+07	14_10996310	11	25	19	19	18	19	0.949854	1	47	26	24	2	37	13	0.00748968	0.313751	21.625	13.0486726								
14	1E+07	14_11225761	48	117	17	19	83	18	2.87E-12	#######	94	128	88	75	111	82	0.29496272	1	73.25	41.9310659								
14	1E+07	14_11231021	10	4	12	5	7	9	0.983538	1	0	22	0	29	11	15	0.61974467	1	10.25	10.4846827								
14	1E+07	14_11391383	6	15	1	11	11	6	0.3028	1	9	0	12	0	5	6	0.91271846	1	6.75	5.89794153	1E+07	1.1E+07	ID=ENSP0000369843;EPHX2;NM_001979;	EPHX2				
14	1E+07	14_11416372	15	40	23	21	28	22	0.367037	1	43	39	15	1	41	8	4.96E-05	0.005055	24.625	14.831795								
14	1E+07	14_11499193	1419	726	848	1075	1073	962	9.96E-09	#######	766	687	270	259	727	265	3.22E-40	4.79E-37	756.25	385.219696					1.1E+07	1.1E+07	0.28654	ID=promoter_15684;
14	1E+07	14_11752796	503	312	283	355	408	319	3.28E-07	#######	247	249	178	124	248	151	2.21E-04	0.0179903	281.38	115.363943								

14	1E+07	14_11821673	4	33	8	0	19	4	0.002035	0.11403	49	5	15	15	27	15	0.27474073	1	16.125	16.7539974	1E+07	1.2E+07	ID=ENSP00000345031;C8orf80;;	C8orf80				
14	1E+07	14_11841633	794	423	444	537	609	491	4.04E-09	#######	440	295	343	117	368	230	1.45E-05	0.0017545	424.13	195.585887					1.2E+07	1.2E+07	0.29946	ID=promoter_15688;
14	1E+07	14_12088326	58	9	74	74	34	74	0.010104	0.41206	9	16	27	4	13	16	0.74830213	1	33.875	29.9973213					1.2E+07	1.2E+07	0.14532	ID=promoter_15690;
14	1E+07	14_12151012	129	57	61	52	93	57	4.49E-04	0.03238	0	1	6	2	1	4	0.36109265	1	38.5	45.5098106								
14	1E+07	14_12181292	459	129	312	232	294	272	0.018374	0.65917	168	130	168	47	149	108	0.14880554	1	205.63	128.615637					1.2E+07	1.2E+07	0.3628	ID=promoter_15693;
14	1E+07	14_12236405	1	30	4	30	16	17	0.960459	1	50	16	43	0	33	22	0.4848075	1	21.75	19.4109689	1E+07	1.2E+07	ID=ENSP00000369604;FBXO16;;	FBXO16				
14	1E+07	14_12419118	597	197	13	26	397	20	6.67E-89	#######	255	205	60	38	230	49	2.28E-23	1.85E-20	173.88	195.044638					1.2E+07	1.2E+07	0.19915	ID=promoter_15696;
14	1E+07	14_12772138	763	591	657	502	677	580	1.78E-07	#######	377	367	406	22	372	214	1.43E-07	2.90E-05	460.63	226.727613					1.3E+07	1.3E+07	0.34432	ID=promoter_15700;
14	1E+07	14_13290559	52	15	96	37	34	67	0.044866	1	1	18	48	33	10	41	2.38E-05	0.0026674	37.5	29.2330537								
14	1E+07	14_13851013	135	23	71	67	79	69	0.199329	1	0	0	9	3	0	6	0.0393172	1	38.5	48.5739495								
14	1E+07	14_14029769	12	19	28	21	16	25	0.667718	1	0	15	17	53	8	35	7.17E-05	0.0069126	20.625	15.3524079								
14	1E+07	14_14308196	17	48	26	25	33	26	0.311396	1	25	13	9	2	19	6	0.05448017	1	20.625	13.989154								
14	1E+07	14_14334652	55	55	66	65	55	66	0.992235	1	18	7	10	1	13	6	0.35360803	1	34.625	28.06593								
14	2E+07	14_15154527	15	51	11	13	33	12	0.001163	0.07164	0	22	34	4	11	19	0.24832023	1	18.75	16.7310661								
14	2E+07	14_15768210	11	52	24	16	32	20	0.080425	1	46	41	49	4	44	27	0.23585004	1	30.375	18.8599159								
14	2E+07	14_16082285	23	30	34	43	27	39	0.706776	1	31	11	1	0	21	1	2.34E-04	0.0188551	21.625	15.9278507								
14	2E+07	14_17079589	1	1	42	38	1	40	1.56E-07	#######	76	8	12	0	42	6	5.09E-06	0.0007008	22.25	27.3430587								
14	2E+07	14_17102035	79	19	70	29	49	50	0.786177	1	92	53	67	19	73	43	0.05897004	1	53.5	28.2033433								
14	2E+07	14_17113784	126	109	110	103	118	107	0.154282	1	183	96	149	30	140	90	0.02442917	0.7977568	113.25	44.1871022								
14	2E+07	14_17491005	69	79	44	71	74	58	0.06868	1	78	17	74	9	48	42	0.96329274	1	55.125	28.2814301								
14	2E+07	14_18182734	20	132	27	63	76	45	0.001043	0.06583	216	82	78	67	149	73	3.34E-05	0.003583	85.625	63.0440606								
14	2E+07	14_19281439	646	254	81	99	450	90	1.91E-66	#######	332	370	45	14	351	30	2.27E-54	4.26E-51	230.13	215.100931								
14	2E+07	14_19412876	47	25	32	1	36	17	0.005986	0.27181	46	37	2	4	42	3	1.90E-07	3.75E-05	24.25	19.4917565								
14	2E+07	14_19562095	80	127	8	51	104	30	2.04E-12	#######	63	49	60	71	56	66	0.41598055	1	63.625	33.4234199								
14	2E+07	14_19588452	0	19	0	1	10	1	0.011548	0.45484	1	28	11	15	15	13	0.98432481	1	9.375	10.6225838								
14	2E+07	14_19624496	8	14	4	22	11	13	0.999831	1	0	0	0	2	0	1	0.58312783	1	6.25	8.0311892								
14	2E+07	14_20352037	0	41	28	10	21	19	0.713172	1	0	6	0	0	3	0	0.24893445	1	10.625	15.5741315								
14	2E+07	14_20519474	2574	1883	2441	2930	2229	2686	0.641977	1	1596	1452	2676	707	1524	1692	2.10E-06	0.0003249	2032.4	754.652794								
14	2E+07	14_20571882	9	26	2	19	18	11	0.22238	1	24	0	11	2	12	7	0.61698677	1	11.625	10.2947629								
14	2E+07	14_20734059	4234	2051	3488	3552	3143	3520	0.169386	1	4758	2660	4031	1984	3709	3008	3.47E-07	6.50E-05	3344.8	1022.37577					2.1E+07	2.1E+07	0.46459	ID=promoter_15770;
14	2E+07	14_20871696	0	33	1	0	17	1	2.04E-04	0.01649	0	14	0	0	7	0	0.03898208	1	6	11.9403278								
14	2E+07	14_22394085	1120	598	770	857	859	814	7.14E-05	0.00662	388	316	403	147	352	275	0.10377732	1	574.88	322.915485	2E+07	2.2E+07	ID=ENSP00000266880;CHFR;;	CHFR	2.2E+07	2.2E+07	0.61093	ID=promoter_15789;

14	2E+07	14_22446469	34	52	52	27	43	40	0.571521	1	59	84	43	28	72	36	0.00890416	0.3584328	47.375	18.9279649	2E+07	2.2E+07	ID=ENSP0000204726;GOLGA3;NM_005895;	GOLGA3				
14	2E+07	14_22468840	0	6	8	2	3	5	0.88968	1	25	27	13	49	26	31	0.63757172	1	16.25	16.5421539	2E+07	2.2E+07	ID=ENSP0000204726;GOLGA3;NM_005895;	GOLGA3				
14	2E+07	14_22708788	89	73	3	0	81	2	5.07E-20	#######	10	24	1	1	17	1	0.00146248	0.085933	25.125	35.6307953					2.3E+07	2.3E+07	0.28953	ID=promoter_15804;
14	2E+07	14_22951557	23	27	30	34	25	32	0.94785	1	53	48	32	45	51	39	0.66313395	1	36.5	10.8100482	2E+07	2.3E+07	ID=ENSP0000329846;GALNT9;NM_001122636;	GALNT9				
14	2E+07	14_22963184	0	0	15	0	0	8	0.033035	1	17	27	0	8	22	4	0.00378795	0.1837142	8.375	10.3224789	2E+07	2.3E+07	ID=ENSP0000329846;GALNT9;NM_001122636;	GALNT9				
14	2E+07	14_23195878	8	0	0	11	4	6	0.929647	1	0	0	11	13	0	12	0.00154584	0.0883233	5.375	5.90248132	2E+07	2.3E+07	ID=ENSP0000333602-D2;EP400;;	EP400				
14	2E+07	14_23277052	79	35	36	38	57	37	0.017546	0.63555	40	14	39	18	27	29	0.85917478	1	37.375	19.5370527					2.3E+07	2.3E+07	0.60137	ID=promoter_15823;
14	2E+07	14_23364465	0	28	12	5	14	9	0.360318	1	0	0	3	1	0	2	0.34003807	1	6.125	9.731209								
14	2E+07	14_23608600	92	56	101	93	74	97	0.771495	1	136	113	51	0	125	26	2.94E-13	1.31E-10	80.25	42.7542813								
14	2E+07	14_23657782	223	134	309	262	179	286	0.004988	0.23452	24	11	29	3	18	16	0.9925456	1	124.38	125.130717								
14	2E+07	14_23685045	10	12	12	23	11	18	0.682529	1	26	1	22	93	14	58	2.55E-07	4.89E-05	24.875	28.7274756								
14	2E+07	14_24279770	8	22	2	2	15	2	0.002248	0.12273	33	6	15	11	20	13	0.60545249	1	12.375	10.7029702								
14	2E+07	14_24800810	176	15	74	59	96	67	0.004345	0.20959	19	8	7	28	14	18	0.65378371	1	48.25	57.0607446								
14	2E+07	14_24843791	8	26	1	18	17	10	0.210142	1	0	40	23	0	20	12	0.48622029	1	14.5	14.6969385								
14	3E+07	14_25384399	67	10	26	16	39	21	0.012903	0.49818	25	43	17	0	34	9	0.00172782	0.09686	25.5	20.9829863								
14	3E+07	14_25713146	594	527	227	270	561	249	5.78E-39	#######	214	390	337	205	302	271	0.92710475	1	345.5	148.0936								
14	3E+07	14_25837009	14	23	5	6	19	6	0.010861	0.43721	3	13	1	0	8	1	0.0848225	1	8.125	7.90004521					2.6E+07	2.6E+07	0.11399	ID=promoter_15860;
14	3E+07	14_26047571	114	41	71	77	78	74	0.424044	1	52	33	43	14	43	29	0.41314796	1	55.625	31.0112307								
14	3E+07	14_26096208	6	0	14	14	3	14	0.063635	1	22	20	2	0	21	1	2.34E-04	0.0188551	9.75	8.90826262								
14	3E+07	14_26560304	130	105	87	97	118	92	0.012628	0.49022	74	51	83	60	63	72	0.47781125	1	85.875	25.2780961								
14	3E+07	14_26693373	10	1	22	4	6	13	0.45043	1	30	12	11	1	21	6	0.02669383	0.8538132	11.375	10.2251441								
14	3E+07	14_26699900	26	47	42	19	37	31	0.381451	1	34	33	21	0	34	11	0.0064318	0.2773637	27.75	14.8107104								
14	3E+07	14_26764754	12	22	0	5	17	3	0.002206	0.1213	0	22	0	1	11	1	0.02232329	0.7455867	7.75	9.69167537								
14	3E+07	14_26802685	55	38	50	25	47	38	0.229608	1	68	12	26	41	40	34	0.93312112	1	39.375	18.188988					2.7E+07	2.7E+07	0.1411	ID=promoter_15868;
14	3E+07	14_26903178	1	18	3	4	10	4	0.162164	1	32	0	38	0	16	19	0.76520201	1	12	15.4272486								
14	3E+07	14_26994122	58	36	76	109	47	93	0.01269	0.49236	42	31	5	1	37	3	1.83E-06	0.0002875	44.75	35.951356								
14	3E+07	14_27392962	39	9	1	0	24	1	3.48E-06	0.00047	4	22	17	1	13	9	0.79589064	1	11.625	13.6898033								
14	3E+07	14_27461132	12	20	22	26	16	24	0.746917	1	1	1	17	1	1	9	0.02974549	0.9232309	12.5	10.3233439								
14	3E+07	14_27529223	59	44	47	60	52	54	0.821444	1	118	67	36	35	93	36	2.64E-05	0.0029221	58.25	26.7675176								

14	3E+07	14_27848508	7	18	4	15	13	10	0.600182	1	27	37	11	0	32	6	3.53E-04	0.0265555	14.875	12.3223085								
14	3E+07	14_27949131	203	332	112	169	268	141	2.50E-14	#######	243	177	70	12	210	41	9.12E-23	7.23E-20	164.75	100.583654								
14	3E+07	14_28008502	108	70	64	100	89	82	0.286502	1	57	95	165	69	76	117	0.00234812	0.1252416	91	35.1974025								
14	3E+07	14_28167559	10	0	0	0	5	0	0.05327	1	23	12	4	0	18	2	0.00297675	0.1507727	6.125	8.35699023								
14	3E+07	14_28770044	14	38	18	8	26	13	0.035221	1	35	14	31	1	25	16	0.50792242	1	19.875	13.3677383	3E+07	2.9E+07	ID=ENSP00000348551;NCOR2;NM_006312;	NCOR2				
14	3E+07	14_28817162	26	9	3	1	18	2	4.15E-04	0.03028	17	18	2	1	18	2	0.00297675	0.1507727	9.625	9.59073213	3E+07	2.9E+07	ID=ENSP00000348551;NCOR2;NM_006312;	NCOR2				
14	3E+07	14_28868727	61	36	29	39	49	34	0.059003	1	52	49	27	4	51	16	3.80E-04	0.0280836	37.125	17.7396851	3E+07	2.9E+07	ID=ENSP00000348551;NCOR2;NM_006312;	NCOR2				
14	3E+07	14_28986006	20	62	16	45	41	31	0.17739	1	14	69	21	41	42	31	0.627947	1	36	21.5008305								
14	3E+07	14_29031008	1625	268	1448	1048	947	1248	0.025828	0.86655	138	133	182	135	136	159	0.13784513	1	622.13	643.564058								
14	3E+07	14_29149505	0	21	11	0	11	6	0.3028	1	25	0	7	7	13	7	0.50249747	1	8.875	9.67230952								
14	3E+07	14_29322872	1	21	1	16	11	9	0.722819	1	20	6	23	0	13	12	0.99994263	1	11	9.97138764								
14	3E+07	14_29708755	121	243	41	51	182	46	1.68E-23	#######	31	61	51	13	46	32	0.45684804	1	76.5	74.2640078								
14	3E+07	14_29847151	308	402	2802	3180	355	2991	0	0	125	324	1067	662	225	865	2.54E-93	8.06E-90	1108.8	1200.16627								
14	3E+07	14_30212347	0	0	7	13	0	10	0.014084	0.53368	0	24	0	2	12	1	0.01422424	0.5202299	5.75	8.73007936								
14	3E+07	14_30213594	9	53	5	15	31	10	7.28E-04	0.04855	0	6	1	2	3	2	0.9363008	1	11.375	17.5086713								
14	3E+07	14_30288995	2	21	18	9	12	14	0.999907	1	39	13	16	18	26	17	0.53107524	1	17	10.743769								
14	3E+07	14_30534189	9	23	14	4	16	9	0.196587	1	31	22	18	2	27	10	0.0371409	1	15.375	10.0276404								
14	3E+07	14_30573024	15	22	8	5	19	7	0.021688	0.74727	18	8	50	24	13	37	0.00121371	0.0738375	18.75	14.3899767								
14	3E+07	14_30613605	14	1	10	23	8	17	0.371907	1	0	0	2	8	0	5	0.06742467	1	7.25	8.22452778								
14	3E+07	14_30700207	698	459	588	622	579	605	0.138207	1	248	235	201	106	242	154	0.00118308	0.0722825	394.63	224.578295					3.1E+07	3.1E+07	0.47285	ID=promoter_15948;
14	3E+07	14_30917563	13	63	13	3	38	8	4.14E-06	0.00055	48	45	31	54	47	43	0.99804762	1	33.75	22.0696948								
14	3E+07	14_30932033	294	112	295	251	203	273	0.336161	1	131	32	135	66	82	101	0.15890525	1	164.5	102.323018	3E+07	3.1E+07	ID=ENSP00000262519-D3;SETD1A;NM_014712;	SETD1A				
14	3E+07	14_31210269	298	120	171	158	209	165	6.61E-04	0.04479	164	97	125	72	131	99	0.3069352	1	150.63	68.6813293								
14	3E+07	14_31306132	146	79	116	115	113	116	0.60081	1	46	7	25	46	27	36	0.35693007	1	72.5	49.4165964	3E+07	3.1E+07	ID=ENSP00000261819;ANAPC5;NM_016237;	ANAPC5				
14	3E+07	14_31837772	554	351	499	543	453	521	0.954215	1	160	120	223	5	140	114	0.58909933	1	306.88	210.55806	3E+07	3.2E+07	ID=ENSP00000228827;GPN3;NM_016301;	GPN3				
14	3E+07	14_31876604	87	46	116	107	67	112	0.07005	1	26	44	45	16	35	31	0.98304591	1	60.875	37.4869025					3.2E+07	3.2E+07	0.29542	ID=promoter_15981;
14	3E+07	14_31877080	432	86	436	324	259	380	0.020776	0.72593	232	127	122	43	180	83	5.44E-07	9.63E-05	225.25	155.782587					3.2E+07	3.2E+07	0.29542	ID=promoter_15981;

14	3E+07	14_32239380	40	11	31	32	26	32	0.98353	1	35	22	43	28	29	36	0.50182289	1	30.25	10.1945364	3E+07	3.2E+07	ID=ENSP00000228841;MYL2;NM_000432;	MYL2				
14	3E+07	14_32426886	0	0	6	6	0	6	0.077487	1	0	10	2	19	5	11	0.25523295	1	5.375	6.61032742					3.2E+07	3.2E+07	0.14051	ID=promoter_15989;
14	3E+07	14_32671230	581	399	401	319	490	360	9.03E-11	#######	266	104	141	105	185	123	0.01625055	0.5792063	289.5	169.587735					3.3E+07	3.3E+07	0.18425	ID=promoter_15997;
14	3E+07	14_34765966	10	8	14	4	9	9	0.944235	1	77	70	124	25	74	75	0.86225073	1	41.5	43.7982387								
14	3E+07	14_34840457	18	43	10	5	31	8	1.52E-04	0.01277	44	18	17	20	31	19	0.35951856	1	21.875	14.2371496					3.5E+07	3.5E+07	0.53523	ID=promoter_16028;
14	3E+07	14_34923842	13	11	6	2	12	4	0.066899	1	18	7	26	0	13	13	0.98151319	1	10.375	8.60128728								
14	4E+07	14_35013355	48	0	35	6	24	21	0.616071	1	2	0	23	19	1	21	5.13E-05	0.0051875	16.625	17.9199609	3E+07	3.5E+07	ID=ENSP00000339952;KSR2;;	KSR2				
14	4E+07	14_35210527	114	59	125	125	87	125	0.346687	1	88	31	32	0	60	16	1.45E-05	0.0017545	71.75	48.2130688	3E+07	3.5E+07	ID=ENSP00000339952;KSR2;;	KSR2				
14	4E+07	14_35423269	3646	2234	3661	3032	2940	3347	0.497427	1	2177	1668	1979	1439	1923	1709	0.44632177	1	2479.5	862.336527					3.5E+07	3.5E+07	0.55957	ID=promoter_16034;
14	4E+07	14_35958766	7	28	4	26	18	15	0.617094	1	17	48	39	1	33	20	0.31924699	1	21.25	17.0188971	4E+07	3.6E+07	ID=ENSP00000384926;RNFT2;NM_032814;	RNFT2				
14	4E+07	14_36221704	19	49	20	23	34	22	0.087717	1	2	23	42	45	13	44	6.39E-05	0.0062406	27.875	16.0217486								
14	4E+07	14_36669860	133	254	174	122	194	148	3.75E-04	0.02793	179	196	377	138	188	258	2.09E-04	0.0171405	196.63	84.2647825								
14	4E+07	14_37633894	302	237	361	330	270	346	0.552017	1	206	40	400	93	123	247	6.40E-12	2.42E-09	246.13	127.983746								
14	4E+07	14_38162920	22	0	11	7	11	9	0.722819	1	0	5	4	0	3	2	0.9363008	1	6.125	7.51070664								
14	4E+07	14_38173932	1702	2426	2477	3317	2064	2897	3.50E-09	#######	2078	1787	3468	886	1933	2177	3.56E-09	9.48E-07	2267.6	854.131629					3.8E+07	3.8E+07	0.48322	ID=promoter_16075;
14	4E+07	14_38563266	62	55	59	37	59	48	0.166754	1	0	50	67	12	25	40	0.09550227	1	42.75	24.5865817	4E+07	3.9E+07	ID=ENSP00000376344;RBM19;NM_016196;	RBM19				
14	4E+07	14_38683571	25	31	17	21	28	19	0.175883	1	57	7	13	1	32	7	8.21E-04	0.0536252	21.5	17.2626765								
14	4E+07	14_38885356	25	17	12	9	21	11	0.084122	1	17	26	14	23	22	19	0.97472654	1	17.875	6.24356812	4E+07	3.9E+07	ID=ENSP00000280800;PLBD2;NM_173542;	PLBD2				
14	4E+07	14_38914748	1123	341	818	766	732	792	0.289169	1	576	315	314	91	446	203	1.03E-16	5.68E-14	543	340.666573					3.9E+07	3.9E+07	0.40573	ID=promoter_16105;
14	4E+07	14_38920745	3693	1660	3975	3753	2677	3864	1.46E-15	#######	534	508	837	666	521	752	4.83E-14	2.32E-11	1953.3	1578.66777								
14	4E+07	14_39270851	27	17	9	1	22	5	0.001077	0.06758	55	22	10	1	39	6	1.85E-05	0.0021598	17.75	17.6857489								
14	4E+07	14_40404843	14	104	2	41	59	22	8.08E-06	0.00099	104	32	59	9	68	34	0.01157455	0.4411768	45.625	40.4366789								
14	4E+07	14_40529861	1	3	5	0	2	3	0.964182	1	16	4	11	0	10	6	0.69729498	1	5	5.70713839								
14	4E+07	14_40664868	6152	5359	6406	9447	5756	7927	2.02E-19	#######	5872	5283	11170	4776	5578	7973	1.96E-136	1.17E-132	6808.1	2268.21676					4.1E+07	4.1E+07	0.41058	ID=promoter_16150;
14	4E+07	14_41232378	123	8	8	7	66	8	8.82E-13	#######	0	16	6	1	8	4	0.59180275	1	21.125	41.4537177	4E+07	4.1E+07	ID=ENSP00000384520;TCHP;NM_001143852;	TCHP				

14	4E+07	14_41309869	24	66	56	26	45	41	0.502494	1	55	58	92	55	57	74	0.15831276	1	54	21.6926584								
14	4E+07	14_41898321	36	41	3	10	39	7	9.09E-07	0.00014	0	0	23	2	0	13	9.01E-04	0.0577931	14.375	16.7582602					4.2E+07	4.2E+07	0.14554	ID=promoter_16188;
14	4E+07	14_41974550	66	144	105	51	105	78	0.008646	0.36461	52	35	58	20	44	39	0.97939611	1	66.375	39.939016								
14	4E+07	14_42117133	14	25	13	23	20	18	0.715835	1	13	0	10	44	7	27	0.00126401	0.0755142	17.75	13.1121536								
14	4E+07	14_42276217	171	84	193	133	128	163	0.784798	1	0	38	15	16	19	16	0.96066018	1	81.25	75.9205412	4E+07	4.2E+07	ID=ENSP00000315713;SSH1;NM_018984;	SSH1				
14	4E+07	14_42370372	8	39	146	198	24	172	2.85E-20	#######	8	14	61	36	11	49	1.32E-06	0.0002149	63.75	70.5787301	4E+07	4.2E+07	ID=ENSP00000261401;CORO1C;NM_014325;	CORO1C				
14	4E+07	14_42398113	19	31	4	6	25	5	2.23E-04	0.01779	1	6	13	0	4	7	0.58783259	1	10	10.5559733	4E+07	4.2E+07	ID=ENSP00000261401;CORO1C;NM_014325;	CORO1C				
14	4E+07	14_42469768	82	62	103	65	72	84	0.99944	1	71	26	32	62	49	47	0.98616252	1	62.875	24.9596102								
14	4E+07	14_42625511	28	39	10	13	34	12	7.44E-04	0.04914	65	35	26	0	50	13	7.31E-05	0.0070333	27	20.2414003								
14	4E+07	14_42912279	23	0	15	16	12	16	0.945172	1	0	10	31	0	5	16	0.03625394	1	11.875	11.6058791								
14	4E+07	14_43409065	0	30	4	7	15	6	0.065303	1	11	16	9	42	14	26	0.10164664	1	14.875	14.2271119								
14	4E+07	14_43984193	19	0	15	6	10	11	0.989255	1	0	0	2	0	0	1	0.58312783	1	5.25	7.61108215	4E+07	4.4E+07	ID=ENSP00000334563;MYO18B;NM_032608;	MYO18B				
14	4E+07	14_44436317	16	5	7	0	11	4	0.10516	1	9	0	2	22	5	12	0.17891578	1	7.625	7.87287386								
14	4E+07	14_44810288	8	15	6	0	12	3	0.030625	0.98374	22	22	17	29	22	23	0.92188352	1	14.875	9.65752852	4E+07	4.5E+07	ID=ENSP00000381210;HPS4;;	HPS4				
14	4E+07	14_44868879	0	13	3	3	7	3	0.314307	1	24	1	0	17	13	9	0.79589064	1	7.625	9.16417715								
14	5E+07	14_45337603	8	7	7	2	8	5	0.532535	1	2	12	30	1	7	16	0.11989665	1	8.625	9.41029072								
14	5E+07	14_45458371	16	43	4	5	30	5	1.51E-05	0.0017	20	15	11	15	18	13	0.78939737	1	16.125	12.1706614								
14	5E+07	14_45472865	68	35	90	49	52	70	0.753203	1	0	7	3	0	4	2	0.76928717	1	31.5	34.7850543								
14	5E+07	14_45914226	11	15	12	10	13	11	0.725732	1	40	39	34	2	40	18	0.03384608	1	20.375	14.8798762	5E+07	4.6E+07	ID=ENSP00000381003;TTC28;NM_001145418;	TTC28				
14	5E+07	14_46418236	6	14	0	17	10	9	0.84607	1	1	8	12	6	5	9	0.48000906	1	8	6.02376247								
14	5E+07	14_46559699	0	39	0	0	20	0	8.05E-06	0.00099	19	14	15	25	17	20	0.769441	1	14	13.938641								
14	5E+07	14_46824717	28	38	29	24	33	27	0.37765	1	23	32	66	1	28	34	0.56965937	1	30.125	18.1063328								
14	5E+07	14_46836854	9	24	10	22	17	16	0.818283	1	37	0	29	47	19	38	0.01959509	0.6683811	22.25	15.5815825								
14	5E+07	14_47483108	6	10	1	5	8	3	0.206144	1	0	16	11	1	8	6	0.92562801	1	6.25	5.70087713								
14	5E+07	14_48462083	73	11	1	1	42	1	9.36E-11	#######	49	4	12	1	27	7	0.00575936	0.2548307	19	27.0607782	5E+07	4.9E+07	ID=ENSP00000371747;OSBP2;;	OSBP2	4.8E+07	4.8E+07	0.32808	ID=promoter_16310;
14	5E+07	14_49090645	9	16	33	31	13	32	0.070074	1	36	18	65	2	27	34	0.49096177	1	26.25	19.7248936								
14	5E+07	14_49177809	7	11	16	4	9	10	0.993074	1	1	6	1	0	4	1	0.45332778	1	5.75	5.54848757	5E+07	4.9E+07	ID=ENSP00000371597;SFI1;NM_001007467;	SFI1				

14	5E+07	14_49184559	28	26	18	47	27	33	0.987507	1	10	22	26	2	16	14	0.9876114	1	22.375	13.3730806	5E+07	4.9E+07	ID=ENSP00000371597;SFI1;NM_001007467;	SFI1				
14	5E+07	14_49226816	268	170	252	237	219	245	0.878454	1	108	121	61	31	115	46	4.52E-06	0.0006278	156	89.9968253					4.9E+07	4.9E+07	0.22434	ID=promoter_16330;
14	5E+07	14_49643408	33	35	36	10	34	23	0.11872	1	29	16	18	12	23	15	0.56706676	1	23.625	10.7562274	5E+07	5E+07	ID=ENSP00000266088;SLC5A1;NM_000343;	SLC5A1				
14	5E+07	14_49781264	54	52	19	28	53	24	3.15E-04	0.02414	60	80	44	28	70	36	0.01429746	0.5219719	45.625	20.1135171								
14	5E+07	14_49839164	446	158	291	333	302	312	0.289893	1	148	147	92	24	148	58	7.36E-08	1.57E-05	204.88	139.495456					5E+07	5E+07	0.34911	ID=promoter_16336;
14	5E+07	14_49959524	5	45	12	7	25	10	0.01059	0.42992	29	32	36	1	31	19	0.35951856	1	20.875	16.5567207								
14	5E+07	14_49968033	204	85	114	154	145	134	0.136868	1	141	107	92	31	124	62	2.94E-04	0.0228018	116	51.6305557								
14	5E+07	14_50072463	20	21	10	18	21	14	0.252399	1	25	8	14	32	17	23	0.4932031	1	18.5	7.8921299					5E+07	5E+07	0.24251	ID=promoter_16339;
14	5E+07	14_50176994	48	4	43	37	26	40	0.558059	1	41	7	37	22	24	30	0.54878546	1	29.875	16.8220731								
14	5E+07	14_50598735	16	4	19	19	10	19	0.459788	1	18	9	3	24	14	14	0.98010536	1	14	7.70899289								
14	5E+07	14_50650540	583	121	332	324	352	328	0.010929	0.43771	166	147	99	81	157	90	0.00118602	0.0724308	231.63	171.570841					5.1E+07	5.1E+07	0.47253	ID=promoter_16347;
14	5E+07	14_50717443	427	77	331	307	252	319	0.664101	1	148	145	87	18	147	53	7.55E-09	1.91E-06	192.5	144.637478					5.1E+07	5.1E+07	0.57321	ID=promoter_16348;
14	5E+07	14_50734317	5	7	0	10	6	5	0.851367	1	6	0	8	6	3	7	0.3829009	1	5.25	3.57571172								
14	5E+07	14_50862310	84	70	109	36	77	73	0.426455	1	21	31	44	5	26	25	0.99152959	1	50	34.8384025	5E+07	5.1E+07	ID=ENSP00000381399;MIF;;	MIF	5.1E+07	5.1E+07	0.18441	ID=promoter_16355;
14	5E+07	14_50885704	355	115	305	297	235	301	0.599713	1	151	132	123	52	142	88	0.01149355	0.4382061	191.25	110.7955					5.1E+07	5.1E+07	0.36175	ID=promoter_16358;
14	5E+07	14_50917626	0	36	13	3	18	8	0.059652	1	129	27	70	2	78	36	0.00195494	0.1076481	35	44.6382283	5E+07	5.1E+07	ID=ENSP00000340883;SMARCB1;;	SMARCB1				
14	5E+07	14_50944540	24	13	28	19	19	24	0.971301	1	23	62	25	0	43	13	9.59E-04	0.0604009	24.25	17.6614997								
14	5E+07	14_51334580	2090	1187	2908	2682	1639	2795	1.64E-32	#######	2971	1707	4065	1080	2339	2573	1.56E-08	3.73E-06	2336.3	1013.72436	5E+07	5.1E+07	ID=ENSP00000292778;YDJC;NM_001017964;	YDJC	5.1E+07	5.1E+07	0.4815	ID=promoter_16376;
14	5E+07	14_51427622	106	98	168	125	102	147	0.277652	1	50	31	46	42	41	44	0.79631464	1	83.25	48.6760428								
14	5E+07	14_51465135	66	22	98	76	44	87	0.016984	0.621	25	43	37	0	34	19	0.20290377	1	45.875	32.1311487					5.1E+07	5.1E+07	0.13989	ID=promoter_16382;
14	5E+07	14_51478422	1189	422	1254	926	806	1090	0.009087	0.37919	476	316	441	110	396	276	0.00127816	0.076264	641.75	424.275096					5.1E+07	5.1E+07	0.7581	ID=promoter_16384;
14	5E+07	14_51490987	0	0	3	8	0	6	0.077487	1	0	19	36	18	10	27	0.01023344	0.4008669	10.5	12.9394193	5E+07	5.2E+07	ID=ENSP00000255882;PI4KA;NM_058004;	PI4KA				
14	5E+07	14_51571120	13	20	14	5	17	10	0.210142	1	2	9	2	0	6	1	0.20114407	1	8.125	7.07990113								
14	5E+07	14_51637416	0	6	2	9	3	6	0.747087	1	0	28	1	0	14	1	0.00574567	0.2548307	5.75	9.57302758	5E+07	5.2E+07	ID=ENSP00000346300;CRKL;NM_005207;	CRKL				
14	5E+07	14_52067386	432	135	362	261	284	312	0.726878	1	309	31	113	43	170	78	1.04E-06	0.0001719	210.75	151.067393					5.2E+07	5.2E+07	0.51708	ID=promoter_16415;

14	5E+07	14_52213147	162	66	121	117	114	119	0.672846	1	69	58	48	0	64	24	4.76E-04	0.0340675	80.125	50.7076706					5.2E+07	5.2E+07	0.83066	ID=promoter_16421;
14	5E+07	14_52273083	216	133	253	202	175	228	0.578893	1	32	48	153	15	40	84	6.95E-05	0.0067095	131.5	90.854672	5E+07	5.2E+07	ID=ENSP00000286808-D4;CLDN17;NM_012131;	CLDN17	5.2E+07	5.2E+07	0.56523	ID=promoter_16424;
14	5E+07	14_52437254	37	93	39	40	65	40	0.004832	0.229	45	79	19	57	62	38	0.12925359	1	51.125	24.2159186								
14	5E+07	14_52615203	144	51	135	187	98	161	0.030493	0.98374	22	35	4	40	29	22	0.77701989	1	77.25	67.7342496								
14	5E+07	14_52657934	957	353	1274	1050	655	1162	1.61E-16	#######	499	364	409	299	432	354	0.22703889	1	650.63	381.296636								
14	5E+07	14_52693791	2450	1288	2241	2067	1869	2154	0.856387	1	1181	733	1075	422	957	749	0.00238986	0.1272773	1432.1	738.136348					5.3E+07	5.3E+07	0.53091	ID=promoter_16450;
14	5E+07	14_52697905	1079	407	758	610	743	684	2.75E-05	0.00288	166	318	238	112	242	175	0.04288248	1	461	332.577381					5.3E+07	5.3E+07	0.42295	ID=promoter_16451;
14	5E+07	14_52781197	217	94	210	253	156	232	0.071368	1	87	54	84	15	71	50	0.32772295	1	126.75	87.2398173								
14	5E+07	14_52868095	157	34	163	129	96	146	0.140212	1	75	4	35	28	40	32	0.82411274	1	78.125	63.0610532								
14	5E+07	14_53305049	211	97	141	161	154	151	0.293045	1	46	117	53	21	82	37	0.00100787	0.0629514	105.88	64.4879557					5.3E+07	5.3E+07	0.47047	ID=promoter_16463;
14	5E+07	14_54811828	1	10	5	17	6	11	0.674578	1	88	20	28	14	54	21	0.00230401	0.1234682	22.875	27.653145								
14	6E+07	14_55178384	232	193	333	216	213	275	0.575227	1	127	81	85	61	104	73	0.1881909	1	166	93.9650391								
14	6E+07	14_55564695	1	12	1	0	7	1	0.062633	1	1	0	36	0	1	18	2.55E-04	0.0200383	6.375	12.6371052								
14	6E+07	14_55691764	23	55	37	13	39	25	0.056901	1	56	28	39	11	42	25	0.20942954	1	32.75	17.210877								
14	6E+07	14_55749662	15	60	36	46	38	41	0.93346	1	1	49	88	82	25	85	7.26E-09	1.84E-06	47.125	30.0971641								
14	6E+07	14_55815698	11151	7568	12856	11687	9360	####	4.88E-15	#######	9197	6558	13960	3680	7878	8820	5.35E-33	6.16E-30	9582.1	3483.86786								
14	6E+07	14_56449353	19	39	23	13	29	18	0.097648	1	1	12	48	24	7	36	1.80E-05	0.0021118	22.375	15.1179883								
14	6E+07	14_56857362	15	22	3	15	19	9	0.06806	1	67	12	15	5	40	10	3.67E-04	0.0274065	19.25	20.2184498								
14	6E+07	14_56891018	216	112	177	168	164	173	0.622309	1	79	56	70	54	68	62	0.99553145	1	116.5	62.5277081								
14	6E+07	14_57103364	282	195	302	223	239	263	0.77476	1	130	154	84	49	142	67	2.08E-05	0.0024009	177.38	90.1267956					5.7E+07	5.7E+07	0.26833	ID=promoter_16496;
14	6E+07	14_57432163	30	19	24	7	25	16	0.157912	1	18	24	33	2	21	18	0.97064008	1	19.625	10.676242								
14	6E+07	14_57505131	159	33	90	31	96	61	7.24E-04	0.04849	1	2	10	2	2	6	0.31385293	1	41	56.1782876								
14	6E+07	14_57807176	433	194	225	251	314	238	2.02E-06	0.00029	22	2	3	4	12	4	0.18067673	1	141.75	159.695916					5.8E+07	5.8E+07	0.10116	ID=promoter_16509;
14	6E+07	14_57910731	139	391	104	87	265	96	2.15E-24	#######	312	521	224	71	417	148	2.53E-24	2.14E-21	231.13	163.549414	6E+07	5.8E+07	ID=ENSP00000040877;TARBP1;NM_005646;	TARBP1				
14	6E+07	14_57929719	10	12	1	0	11	1	0.006522	0.28949	17	27	17	20	22	19	0.97472654	1	13	9.258201	6E+07	5.8E+07	ID=ENSP00000040877;TARBP1;NM_005646;	TARBP1				
14	6E+07	14_58000788	30	55	7	25	43	16	1.90E-04	0.01552	15	20	48	40	18	44	0.00151903	0.0882005	30	16.5960408	6E+07	5.8E+07	ID=ENSP00000355576;SLC35F3;;	SLC35F3				
14	6E+07	14_58104286	18	1	8	7	10	8	0.719353	1	15	0	17	1	8	9	0.92351511	1	8.375	7.48212155	6E+07	5.8E+07	ID=ENSP00000355576;SLC35F3;;	SLC35F3				
14	6E+07	14_58555330	1	0	8	2	1	5	0.353258	1	17	0	11	20	9	16	0.2823118	1	7.375	7.96308447								

14	6E+07	14_58770500	1	6	3	1	4	2	0.597627	1	14	18	21	17	16	19	0.76520201	1	10.125	8.25378701								
14	6E+07	14_58850995	0	26	9	28	13	19	0.82904	1	15	57	41	53	36	47	0.29921943	1	28.625	20.5282906	6E+07	5.9E+07	ID=ENSP0000355580;KCNK1;NM_002245;	KCNK1				
14	6E+07	14_58994144	24	43	6	31	34	19	0.029997	0.97543	7	55	25	1	31	13	0.04801516	1	24	18.9510648								
14	6E+07	14_59076019	2	25	7	2	14	5	0.053576	1	0	0	4	3	0	4	0.11562589	1	5.375	8.24512843								
14	6E+07	14_59760353	87	57	87	28	72	58	0.100813	1	30	34	13	0	32	7	8.21E-04	0.0536252	42	32.2578894								
14	6E+07	14_60502434	52	87	251	328	70	290	5.95E-23	#######	15	10	38	14	13	26	0.0678374	1	99.375	121.784279								
14	6E+07	14_60719444	50	85	252	339	68	296	1.65E-24	#######	29	12	54	28	21	41	0.01771749	0.6168629	106.13	121.146237								
14	6E+07	14_60929836	127	79	85	94	103	90	0.122803	1	68	29	56	0	49	28	0.11921514	1	67.25	39.3727607	6E+07	6.1E+07	ID=ENSP0000355598;DISC1;NM_001012957;	DISC1				
14	6E+07	14_60933798	25	70	23	2	48	13	2.04E-06	0.00029	26	0	14	29	13	22	0.2175897	1	23.625	21.7317247	6E+07	6.1E+07	ID=ENSP0000355598;DISC1;NM_001012957;	DISC1				
14	6E+07	14_60950450	58	77	83	59	68	71	0.790271	1	1	27	19	6	14	13	0.99999086	1	41.25	32.0211983	6E+07	6.1E+07	ID=ENSP0000355598;DISC1;NM_001012957;	DISC1				
14	6E+07	14_61416856	34	17	31	34	26	33	0.955738	1	0	5	5	18	3	12	0.04708211	1	18	13.8564065					6.1E+07	6.1E+07	0.40693	ID=promoter_16546;
14	6E+07	14_61462176	1	17	3	10	9	7	0.713906	1	3	30	14	1	17	8	0.27279187	1	9.875	10.1761275								
14	6E+07	14_61504525	3	38	10	13	21	12	0.1273	1	20	16	32	21	18	27	0.27948435	1	19.125	11.444743								
14	6E+07	14_61524630	681	367	326	198	524	262	5.16E-30	#######	398	253	252	47	326	150	3.97E-12	1.54E-09	315.25	184.001359	6E+07	6.2E+07	ID=ENSP0000295050;C1orf124;NM_032018;	C1orf124	6.2E+07	6.2E+07	0.62441	ID=promoter_16549;
14	6E+07	14_61805304	10	29	0	3	20	2	1.33E-04	0.01141	1	1	4	0	1	2	0.81304206	1	6	9.85610761	6E+07	6.2E+07	ID=ENSP0000355621;TTC13;NM_024525;	TTC13				
14	6E+07	14_61848251	1071	551	9	33	811	21	########	#######	877	424	55	7	651	31	2.68E-113	1.06E-109	378.38	424.003011	6E+07	6.2E+07	ID=ENSP0000355623;C1orf198;NM_032800;	C1orf198				
14	6E+07	14_61928182	35	45	20	22	40	21	0.009148	0.37984	55	13	3	3	34	3	7.09E-06	0.0009373	24.5	19.0638026	6E+07	6.2E+07	ID=ENSP0000271971;CAPN9;NM_006615;	CAPN9				
14	6E+07	14_61939343	0	10	12	12	5	12	0.389128	1	1	1	17	2	1	10	0.01766414	0.6154554	6.875	6.59951297	6E+07	6.2E+07	ID=ENSP0000271971;CAPN9;NM_006615;	CAPN9				
14	6E+07	14_62018822	13	6	0	1	10	1	0.011548	0.45484	19	16	2	2	18	2	0.00297675	0.1507727	7.375	7.52021086	6E+07	6.2E+07	ID=ENSP0000355629;COG2;NM_007357;	COG2				
14	6E+07	14_62173109	66	45	36	24	56	30	0.001814	0.10354	29	9	10	22	19	16	0.96066018	1	30.125	18.8939711								
14	6E+07	14_62716532	286	65	238	183	176	211	0.977356	1	56	97	31	81	77	56	0.38306768	1	129.63	93.960383					6.3E+07	6.3E+07	0.79688	ID=promoter_16574;
14	6E+07	14_63268992	19	19	14	43	19	29	0.670114	1	70	64	84	72	67	78	0.39103705	1	48.125	27.9664978								

14	6E+07	14_63277268	94	45	104	172	70	138	0.001658	0.09668	7	31	52	18	19	35	0.04884304	1	65.375	54.9127555					6.3E+07	6.3E+07	0.4851	ID=promoter_16584;
14	6E+07	14_63454450	15	57	23	16	36	20	0.023274	0.79554	44	27	47	1	36	24	0.45392565	1	28.75	18.979312	6E+07	6.3E+07	ID=ENSP00000363642;BMS1;NM_014753;	BMS1				
14	6E+07	14_63660900	255	189	268	311	222	290	0.482068	1	250	136	194	92	193	143	0.12605379	1	211.88	72.9568121					6.4E+07	6.4E+07	0.66161	ID=promoter_16589;
14	6E+07	14_63738981	30	72	23	35	51	29	0.006336	0.28511	18	17	33	28	18	31	0.10487282	1	32	17.4519749	6E+07	6.4E+07	ID=ENSP00000344798;RET;NM_020975;	RET				
14	6E+07	14_63883446	46	93	5	44	70	25	4.41E-07	#######	43	22	19	28	33	24	0.66283249	1	37.5	26.6028999								
14	6E+07	14_64064025	18	36	25	38	27	32	0.999212	1	30	19	20	14	25	17	0.61272849	1	25	8.83176087								
14	6E+07	14_64201099	262	101	205	96	182	151	0.006759	0.29841	48	87	49	68	68	59	0.93306523	1	114.5	77.5536681					6.4E+07	6.4E+07	0.60901	ID=promoter_16603;
14	7E+07	14_65651843	22	84	63	39	53	51	0.599843	1	218	68	92	72	143	82	0.00217764	0.1179102	82.25	59.4083929								
14	7E+07	14_66087802	15	20	26	29	18	28	0.644351	1	99	2	61	76	51	69	0.11997131	1	41	33.7977176	7E+07	6.6E+07	ID=ENSP00000280772;ANK3;NM_020987;	ANK3				
14	7E+07	14_66921272	2	18	3	12	10	8	0.719353	1	20	18	24	1	19	13	0.6985193	1	12.25	9.11435289					6.7E+07	6.7E+07	0.1374	ID=promoter_16621;
14	7E+07	14_68042405	25	61	11	16	43	14	4.87E-05	0.00473	30	32	30	36	31	33	0.85808364	1	30.125	15.0374532	7E+07	6.8E+07	ID=ENSP00000279873;ARID5B;NM_032199;	ARID5B				
14	7E+07	14_68804616	1	6	0	1	4	1	0.312866	1	16	3	19	1	10	10	0.98574881	1	5.875	7.45342682								
14	7E+07	14_69631035	23	48	19	25	36	22	0.049453	1	30	22	73	58	26	66	3.39E-05	0.0036339	37.25	19.9839221								
14	7E+07	14_69954791	41	106	63	108	74	86	0.998302	1	59	44	98	113	52	106	1.12E-05	0.0013967	79	30.2654919					7E+07	7E+07	0.37067	ID=promoter_16640;
14	7E+07	14_71911812	93	27	52	91	60	72	0.991532	1	31	16	44	4	24	24	0.96613769	1	44.75	32.757769								
14	8E+07	14_75452947	361	210	226	241	286	234	2.16E-04	0.01733	109	120	134	59	115	97	0.79060677	1	182.5	96.2481911	8E+07	7.5E+07	ID=ENSP00000346643;HKDC1;NM_025130;	HKDC1				
14	8E+07	14_75531492	16	25	19	46	21	33	0.575361	1	60	23	46	8	42	27	0.32837691	1	30.375	18.0707933					7.6E+07	7.6E+07	0.49639	ID=promoter_16661;
14	8E+07	14_75555662	14	44	20	13	29	17	0.068354	1	91	15	51	21	53	36	0.35159657	1	33.625	27.2655591								
14	8E+07	14_75570787	26	0	19	12	13	16	0.991731	1	0	0	2	22	0	12	0.00154584	0.0883233	10.125	11.0121686								
14	8E+07	14_75620181	1	70	26	9	36	18	0.009725	0.39843	63	22	66	80	43	73	0.0060631	0.2662974	42.125	30.8704089	8E+07	7.6E+07	ID=ENSP00000362403;TACR2;NM_001057;	TACR2				
14	8E+07	14_75967521	128	149	175	177	139	176	0.796703	1	70	44	65	30	57	48	0.88656252	1	104.75	59.4252231	8E+07	7.6E+07	ID=ENSP00000362352;H2AFY2;NM_018649;	H2AFY2				
14	8E+07	14_76236879	36	0	27	9	18	18	0.89158	1	21	0	8	0	11	4	0.25131486	1	12.625	13.8144593								
14	8E+07	14_76254161	144	30	130	83	87	107	0.947523	1	0	0	49	21	0	35	6.33E-09	1.62E-06	57.125	56.2657914								
14	8E+07	14_76323608	449	178	405	271	314	338	0.548515	1	255	116	174	47	186	111	0.00105635	0.0657495	236.88	137.756968								
14	8E+07	14_76340910	50	8	28	10	29	19	0.13502	1	1	26	0	1	14	1	0.00574567	0.2548307	15.5	17.7442143								

14	8E+07	14_76944622	44	258	77	82	151	80	2.76E-08	#######	105	213	197	142	159	170	0.43286845	1	139.75	75.8565375								
14	8E+07	14_76958773	149	67	118	133	108	126	0.99916	1	35	75	74	41	55	58	0.79079296	1	86.5	42.1764999					7.7E+07	7.7E+07	0.36261	ID=promoter_16694;
14	8E+07	14_77307548	245	287	411	643	266	527	1.62E-11	#######	638	551	706	236	595	471	0.03739788	1	464.63	193.660187								
14	8E+07	14_77358215	14	11	12	17	13	15	0.999061	1	27	18	17	21	23	19	0.93310412	1	17.125	5.16685868								
14	8E+07	14_77477386	1423	820	1734	1828	1122	1781	1.64E-14	#######	840	657	565	267	749	416	2.64E-16	1.44E-13	1016.8	573.608565					7.7E+07	7.7E+07	0.62872	ID=promoter_16705;
14	8E+07	14_77478053	595	160	479	349	378	414	0.628462	1	281	149	182	81	215	132	8.74E-04	0.0568789	284.5	178.962087					7.7E+07	7.7E+07	0.62872	ID=promoter_16705;
14	8E+07	14_78351722	29	35	22	10	32	16	0.016272	0.6002	51	13	9	14	32	12	0.02181216	0.7347023	22.875	14.6914698								
14	8E+07	14_78516489	50	32	63	45	41	54	0.854989	1	20	20	9	0	20	5	0.01914528	0.6572777	29.875	21.5766905								
14	8E+07	14_78736615	8	39	17	12	24	15	0.151024	1	36	37	41	25	37	33	0.98705072	1	26.875	13.1522676								
14	8E+07	14_79406339	24	54	9	36	39	23	0.028526	0.93503	12	0	19	3	6	11	0.39136385	1	19.625	18.1339264	8E+07	7.9E+07	ID=ENSP00000384479;TTC18;;	TTC18				
14	8E+07	14_80110207	1	8	4	6	5	5	0.968625	1	23	0	7	0	12	4	0.18067673	1	6.125	7.51070664	8E+07	8E+07	ID=ENSP00000211998;VCL;NM_014000;	VCL				
14	8E+07	14_80382982	1	30	6	9	16	8	0.127562	1	22	35	3	0	29	2	2.09E-05	0.0024085	13.25	13.7918195	8E+07	8.1E+07	ID=ENSP00000361819;ADK;NM_001123;	ADK				
14	8E+07	14_80864501	23	38	17	26	31	22	0.19035	1	124	26	91	5	75	48	0.13103646	1	43.75	41.3754586								
14	8E+07	14_80945923	0	0	1	7	0	4	0.181755	1	15	2	16	2	9	9	0.98716474	1	5.375	6.63190341	8E+07	8.1E+07	ID=ENSP00000361775;SAMD8;;	SAMD8				
14	8E+07	14_81144479	894	325	811	694	610	753	0.644194	1	334	288	328	146	311	237	0.07492884	1	477.5	278.66211					8.1E+07	8.1E+07	0.96331	ID=promoter_16762;
14	8E+07	14_81180408	5	1	6	5	3	6	0.747087	1	0	12	24	5	6	15	0.10150651	1	7.25	7.66718425								
14	8E+07	14_81181258	78	35	50	74	57	62	0.918924	1	82	7	38	80	45	59	0.21244158	1	55.5	27.4121975								
14	8E+07	14_81403836	23	25	16	22	24	19	0.435991	1	9	42	51	0	26	26	0.96336814	1	23.5	16.570197								
14	8E+07	14_82414058	22	101	36	39	62	38	0.00586	0.26733	31	61	119	90	46	105	9.30E-07	0.0001564	62.375	36.4885011								
14	8E+07	14_82634414	5	15	2	0	10	1	0.011548	0.45484	0	16	20	7	8	14	0.34554715	1	8.125	7.84560842								
14	8E+07	14_83211389	16	11	0	8	14	4	0.02589	0.86655	0	5	15	21	3	18	0.00260948	0.1361302	9.5	7.65319728								
14	8E+07	14_83317730	32	17	13	14	25	14	0.076795	1	24	16	22	4	20	13	0.60545249	1	17.75	8.3623305								
14	8E+07	14_83328885	109	73	207	88	91	148	0.048002	1	23	1	5	2	12	4	0.18067673	1	63.5	71.8172283								
14	8E+07	14_83472152	53	18	58	260	36	159	1.13E-13	#######	18	23	109	36	21	73	7.23E-08	1.55E-05	71.875	81.7652345					8.3E+07	8.3E+07	0.67044	ID=promoter_16780;
14	8E+07	14_84348617	56	82	38	77	69	58	0.171122	1	6	9	23	32	8	28	0.00175088	0.0978457	40.375	28.9380496								
14	8E+07	14_84613142	1	14	7	13	8	10	0.991043	1	61	11	22	33	36	28	0.78400353	1	20.25	19.084399								
14	8E+07	14_84675284	40	36	41	15	38	28	0.169685	1	39	12	27	23	26	25	0.99152959	1	29.125	11.5812348								
14	9E+07	14_85148995	130	19	124	114	75	119	0.120576	1	26	30	54	23	28	39	0.25425581	1	65	49.0684653	9E+07	8.5E+07	ID=ENSP00000361422;ZMIZ1;NM_020338;	ZMIZ1				
14	9E+07	14_85275503	14	0	13	26	7	20	0.111433	1	5	0	6	23	3	15	0.01140593	0.4370832	10.875	9.89137431								

14	9E+07	14_85297445	324	74	299	238	199	269	0.315623	1	214	112	103	101	163	102	0.00738506	0.3105919	183.13	98.1026248								
14	9E+07	14_85399308	890	603	832	866	747	849	0.820736	1	622	416	486	300	519	393	0.01011362	0.3969357	626.88	220.469264					8.5E+07	8.5E+07	0.16934	ID=promoter_16803;
14	9E+07	14_86180390	107	126	56	84	117	70	3.35E-05	0.00343	115	50	25	84	83	55	0.15226692	1	80.875	35.0446144								
14	9E+07	14_86504506	324	185	149	170	255	160	2.33E-09	#######	88	125	168	6	107	87	0.66141812	1	151.88	90.5561191								
14	9E+07	14_87644116	1	31	4	20	16	12	0.497978	1	0	12	17	5	6	11	0.39136385	1	11.25	10.8463292								
14	9E+07	14_88748567	10	7	15	19	9	17	0.506269	1	1	11	7	18	6	13	0.20797135	1	11	6.11788946								
14	9E+07	14_88956440	1	7	0	0	4	0	0.09576	1	41	8	5	1	25	3	3.93E-04	0.0289246	7.875	13.7574655								
14	9E+07	14_89584897	6	22	28	12	14	20	0.851664	1	6	8	12	46	7	29	5.05E-04	0.0357488	17.5	13.9283883								
14	9E+07	14_89993610	85	28	84	48	57	66	0.99745	1	1	24	33	62	13	48	1.09E-05	0.00137	45.625	29.8229897								
14	9E+07	14_90415879	27	6	19	40	17	30	0.39938	1	0	5	13	16	3	15	0.01140593	0.4370832	15.75	13.0466197								
14	9E+07	14_90802325	230	44	171	141	137	156	0.968443	1	0	40	58	0	20	29	0.29996038	1	85.5	84.8864788					9.1E+07	9.1E+07	0.68942	ID=promoter_16824;
14	9E+07	14_91094921	0	11	2	0	6	1	0.108618	1	5	19	5	0	12	3	0.09316162	1	5.25	6.71352579								
14	9E+07	14_91713124	35	636	55	110	336	83	1.72E-43	#######	202	2375	382	134	1289	258	1.19E-133	6.54E-130	491.13	787.08548	9E+07	9.2E+07	ID=ENSP0000361097;MMRN2;NM_024756;	MMRN2	9.2E+07	9.2E+07	0.34394	ID=promoter_16836;
14	9E+07	14_91798428	6	26	4	9	16	7	0.076817	1	0	64	12	2	32	7	8.21E-04	0.0536252	15.375	21.253151	9E+07	9.2E+07	ID=ENSP0000361097-D2;MMRN2;NM_024756;	MMRN2	9.2E+07	9.2E+07	0.36557	ID=promoter_16837;
14	9E+07	14_91993970	363	154	288	242	259	265	0.293944	1	70	119	77	81	95	79	0.77495543	1	174.25	110.608383					9.2E+07	9.2E+07	0.39461	ID=promoter_16841;
14	9E+07	14_92177705	216	534	180	236	375	208	8.50E-18	#######	611	743	826	530	677	678	0.36394693	1	484.5	248.001152								
14	9E+07	14_92290984	86	14	60	30	50	45	0.433543	1	33	40	12	28	37	20	0.14440904	1	37.875	24.590576								
14	9E+07	14_92337873	1	22	2	5	12	4	0.066899	1	21	16	21	3	19	12	0.57792516	1	11.375	9.45572389								
14	9E+07	14_92347379	9	5	5	0	7	3	0.314307	1	31	30	0	1	31	1	2.33E-06	0.0003543	10.125	12.9442486								
14	9E+07	14_92445862	0	35	12	0	18	6	0.017303	0.62786	18	1	39	8	10	24	0.03201086	0.981237	14.125	15.5051835								
14	9E+07	14_92535902	0	10	1	8	5	5	0.968625	1	1	15	3	6	8	5	0.78486764	1	5.5	5.26443594								
14	9E+07	14_92924258	125	94	134	121	110	128	0.99812	1	161	154	153	242	158	198	0.017909	0.6232266	148	43.8112836								
14	9E+07	14_92983109	39	5	4	16	22	10	0.03588	1	18	2	6	1	10	4	0.34296513	1	11.375	12.8055513								
14	9E+07	14_93169694	19	2	19	7	11	13	0.999831	1	12	9	14	29	11	22	0.10262264	1	13.875	8.42509008								
14	9E+07	14_93370014	163	237	153	171	200	162	0.002019	0.114	110	96	58	16	103	37	1.89E-06	0.0002956	125.5	70.0224454	9E+07	9.3E+07	ID=ENSP0000309314;WDFY4;;	WDFY4				
14	9E+07	14_93966250	12	12	34	19	12	27	0.161087	1	45	24	17	8	35	13	0.01427485	0.52128	21.375	12.5577239	9E+07	9.4E+07	ID=ENSP0000378929;CHAT;NM_001142934;	CHAT				
14	9E+07	14_94849982	16	37	7	5	27	6	2.01E-04	0.01628	2	33	41	2	18	22	0.68341813	1	17.875	16.5653468								
14	9E+07	14_94931235	1027	212	815	768	620	792	0.281835	1	290	99	344	157	195	251	0.00256965	0.1346431	464	352.185016					9.5E+07	9.5E+07	0.23706	ID=promoter_16879;
14	1E+08	14_98047227	0	10	7	15	5	11	0.495311	1	55	23	11	29	39	20	0.09202448	1	18.75	17.2274698								
14	1E+08	14_99136807	22	132	30	67	77	49	0.003103	0.16029	53	100	110	88	77	99	0.09658007	1	75.25	39.0045785								

14	1E+08	14_100753299	0	64	11	4	32	8	9.18E-05	0.00825	11	45	18	43	28	31	0.79257784	1	24.5	23.1455025								
14	1E+08	14_101603030	1	5	0	8	3	4	0.986002	1	22	21	7	21	22	14	0.54188925	1	10.625	9.27265565								
14	1E+08	14_104028080	16	24	10	14	20	12	0.173914	1	11	0	10	0	6	5	0.98461866	1	10.625	7.98100423								
14	1E+08	14_104186041	64	25	54	45	45	50	0.965846	1	2	0	1	3	1	2	0.81304206	1	24.25	26.6498459								
14	1E+08	14_104976491	6	21	7	9	14	8	0.253058	1	36	8	14	1	22	8	0.06315916	1	12.75	11.1066261								
14	1E+08	14_105826851	537	304	585	369	421	477	0.87442	1	285	186	226	109	236	168	0.03197455	0.9811772	325.13	165.744586					1.1E+08	1.1E+08	0.29365	ID=promoter_16920;
14	1E+08	14_106155502	168	45	162	85	107	124	0.995823	1	105	31	55	27	68	41	0.09040999	1	84.75	56.0580057					1.1E+08	1.1E+08	0.5035	ID=promoter_16924;
14	1E+08	14_106244116	11	8	4	3	10	4	0.162164	1	3	12	17	58	8	38	1.73E-05	0.0020431	14.5	18.2600266								
14	1E+08	14_107783847	87	44	80	70	66	75	0.982523	1	0	11	0	0	6	0	0.06196836	1	36.5	38.2995711								
14	1E+08	14_108292106	19	54	15	23	37	19	0.010763	0.4351	75	14	14	21	45	18	0.00810261	0.3335968	29.375	22.6333351								
14	1E+08	14_108380561	258	123	234	149	191	192	0.319136	1	51	91	74	35	71	55	0.60583723	1	126.88	82.3363095								
14	1E+08	14_108637202	63	52	63	56	58	60	0.792142	1	1	12	14	2	7	8	0.92121674	1	32.875	27.9716055					1.1E+08	1.1E+08	0.34932	ID=promoter_16939;
14	1E+08	14_108955947	3627	2415	4182	4102	3021	4142	5.52E-10	#######	2443	1731	2633	947	2087	1790	0.05423502	1	2760	1143.69014					1.1E+08	1.1E+08	0.51846	ID=promoter_16943;
14	1E+08	14_109125575	8	20	10	5	14	8	0.253058	1	0	6	14	1	3	8	0.26303803	1	8	6.65475126	1E+08	1.1E+08	ID=ENSP00000265986;IDE;NM_004969;	IDE				
14	1E+08	14_109215595	153	100	160	153	127	157	0.907646	1	47	36	30	36	42	33	0.7761727	1	89.375	58.8167554					1.1E+08	1.1E+08	0.58158	ID=promoter_16946;
14	1E+08	14_110612766	57	29	56	32	43	44	0.816262	1	7	45	45	84	26	65	4.95E-05	0.0050484	44.375	22.8406749								
14	1E+08	14_110710887	9	19	15	10	14	13	0.831264	1	38	0	1	0	19	1	5.85E-04	0.0402157	11.5	12.8396707								
14	1E+08	14_111459078	41	42	80	29	42	55	0.865381	1	0	12	16	13	6	15	0.10150651	1	29.125	25.2667907								
14	1E+08	14_111578420	8	0	3	9	4	6	0.929647	1	24	23	6	10	24	8	0.03264408	0.9940318	10.375	8.73314704								
14	1E+08	14_111852767	211	35	170	114	123	142	0.991721	1	46	80	91	9	63	50	0.7138487	1	94.5	68.7417528					1.1E+08	1.1E+08	0.14488	ID=promoter_16978;
14	1E+08	14_112092967	839	688	898	811	764	855	0.640467	1	272	134	254	121	203	188	0.99995026	1	502.13	337.099153								
14	1E+08	14_112172826	24	15	11	19	20	15	0.418324	1	192	121	209	45	157	127	0.520378	1	79.5	82.7491908								
14	1E+08	14_112823052	29	82	55	19	56	37	0.023015	0.78841	69	29	14	58	49	36	0.56911506	1	44.375	24.9567483	1E+08	1.1E+08	ID=ENSP00000360216;DNTT;NM_004088;	DNTT				
14	1E+08	14_112934991	25	28	11	23	27	17	0.124321	1	20	33	28	36	27	32	0.64141452	1	25.5	7.80109882	1E+08	1.1E+08	ID=ENSP00000350630;TLL2;NM_012465;	TLL2				
14	1E+08	14_112969079	66	37	52	25	52	39	0.103737	1	52	21	37	45	37	41	0.73319416	1	41.875	14.951708	1E+08	1.1E+08	ID=ENSP00000350630;TLL2;NM_012465;	TLL2				
14	1E+08	14_113061853	32	144	96	91	88	94	0.819467	1	119	120	137	125	120	131	0.43232865	1	108	35.6570971								
14	1E+08	14_113170246	22	18	14	12	20	13	0.243396	1	10	20	17	6	15	12	0.93002578	1	14.875	5.38350656	1E+08	1.1E+08	ID=ENSP00000339826;PIK3AP1;NM_152309;	PIK3AP1				
14	1E+08	14_113395938	256	135	221	177	196	199	0.356886	1	100	72	89	25	86	57	0.14257053	1	134.38	78.5656004								

14	1E+08	14_113533448	108	51	89	103	80	96	0.988725	1	44	25	14	20	35	17	0.08418543	1	56.75	38.1491434					1.1E+08	1.1E+08	0.21136	ID=promoter_17000;
14	1E+08	14_113740227	53	83	25	17	68	21	6.57E-08	#######	115	60	39	17	88	28	1.60E-06	0.000253	51.125	34.5106091								
14	1E+08	14_114034182	2	4	25	30	3	28	2.64E-04	0.02068	0	22	3	0	11	2	0.06154634	1	10.75	12.6123522								
14	1E+08	14_114044391	28	35	25	22	32	24	0.247982	1	0	13	47	0	7	24	0.00482107	0.2211976	21.25	16.4033969								
14	1E+08	14_114294480	14	18	245	225	16	235	3.27E-35	#######	17	33	178	42	25	110	8.32E-14	3.91E-11	96.5	101.064336					1.1E+08	1.1E+08	0.30633	ID=promoter_17024;
14	1E+08	14_114331806	31	29	28	28	30	28	0.684	1	0	33	15	1	17	8	0.27279187	1	20.625	13.5323686								
14	1E+08	14_114401274	6	1	8	3	4	6	0.929647	1	22	0	30	21	11	26	0.02624811	0.8458091	11.375	11.3381467								
14	1E+08	14_114411632	10	20	5	3	15	4	0.015825	0.58672	27	0	0	3	14	2	0.01719769	0.6064547	8.5	9.95705062	1E+08	1.1E+08	ID=ENSP00000359607;PYROXD2;NM_032709;	PYROXD2				
14	1E+08	14_115480931	517	133	349	382	325	366	0.867	1	160	140	168	162	150	165	0.31586869	1	251.38	144.84271					1.2E+08	1.2E+08	0.62145	ID=promoter_17043;
14	1E+08	14_115486162	282	152	375	231	217	303	0.145758	1	55	108	108	105	82	107	0.06461608	1	177	109.126401					1.2E+08	1.2E+08	0.75442	ID=promoter_17044;
14	1E+08	14_115694822	22	83	6	32	53	19	1.62E-05	0.00182	5	23	16	10	14	13	0.99999086	1	24.625	25.3317277								
14	1E+08	14_115795882	2	23	8	9	13	9	0.46931	1	0	1	5	10	1	8	0.04988758	1	7.25	7.40173725								
14	1E+08	14_116250147	0	92	9	32	46	21	9.98E-04	0.06325	42	27	22	27	35	25	0.60746796	1	31.375	27.7742815	1E+08	1.2E+08	ID=ENSP00000326411;CWF19L1;NM_018294;	CWF19L1				
14	1E+08	14_116329903	793	280	862	672	537	767	0.002141	0.11834	187	234	272	84	211	178	0.65064762	1	423	302.657279					1.2E+08	1.2E+08	0.45899	ID=promoter_17062;
14	1E+08	14_116658351	392	273	425	480	333	453	0.120978	1	153	117	79	112	135	96	0.13750526	1	253.88	160.125342								
14	1E+08	14_116701636	327	182	257	215	255	236	0.031521	1	106	138	81	59	122	70	0.00541778	0.2454208	170.63	92.3857402					1.2E+08	1.2E+08	0.41428	ID=promoter_17069;
14	1E+08	14_116709735	329	48	317	323	189	320	3.05E-04	0.02346	118	93	148	28	106	88	0.74599335	1	175.5	127.739691					1.2E+08	1.2E+08	0.31686	ID=promoter_17070;
14	1E+08	14_116756508	331	115	277	208	223	243	0.729494	1	137	68	44	31	103	38	3.24E-06	0.000473	151.38	110.682219					1.2E+08	1.2E+08	0.44399	ID=promoter_17079;
14	1E+08	14_116850364	4	35	2	19	20	11	0.118302	1	22	18	7	21	20	14	0.72101732	1	16	11.0582871								
14	1E+08	14_116881310	1105	354	915	625	730	770	0.12071	1	409	202	278	92	306	185	2.26E-05	0.0025712	497.5	356.16489					1.2E+08	1.2E+08	0.75697	ID=promoter_17090;
14	1E+08	14_116918239	4	19	1	15	12	8	0.45534	1	2	9	21	18	6	20	0.0132456	0.49354	11.125	8.13172798					1.2E+08	1.2E+08	0.3673	ID=promoter_17093;
14	1E+08	14_116989391	789	280	544	437	535	491	4.33E-04	0.0313	219	179	171	156	199	164	0.53617324	1	346.88	226.408883					1.2E+08	1.2E+08	0.53161	ID=promoter_17097;
14	1E+08	14_117143448	34	17	31	20	26	26	0.847245	1	25	27	33	7	26	20	0.8208633	1	24.25	9.20791585					1.2E+08	1.2E+08	0.53811	ID=promoter_17109;
14	1E+08	14_117206513	1	26	15	5	14	10	0.480768	1	26	40	16	1	33	9	0.00251067	0.1318429	16.25	13.8744369								
14	1E+08	14_117245539	0	37	10	0	19	5	0.004955	0.23359	34	18	26	0	26	13	0.18179217	1	15.625	15.4544261								
14	1E+08	14_117718816	34	98	47	34	66	41	0.005078	0.23773	301	82	136	26	192	81	8.48E-09	2.13E-06	94.75	91.6526829								
14	1E+08	14_118052477	290	93	264	206	192	235	0.908703	1	54	80	61	2	67	32	0.00715583	0.302645	131.25	106.977635					1.2E+08	1.2E+08	0.3617	ID=promoter_17128;
14	1E+08	14_118135366	398	195	410	352	297	381	0.511953	1	241	190	101	41	216	71	2.33E-14	1.15E-11	241	136.197336					1.2E+08	1.2E+08	0.3228	ID=promoter_17133;

14	1E+08	14_118446623	46	3	43	2	25	23	0.701069	1	1	32	16	0	17	8	0.27279187	1	17.875	19.6500636					1.2E+08	1.2E+08	0.28187	ID=promoter_17139;
14	1E+08	14_118548017	8	28	5	4	18	5	0.008132	0.34618	54	30	9	3	42	6	5.09E-06	0.0007008	17.625	18.1654106	1E+08	1.2E+08	ID=ENSP0000358918;SUFU;NM_016169;	SUFU				
14	1E+08	14_118660751	2547	979	2300	1720	1763	2010	0.684553	1	1206	846	897	385	1026	641	3.05E-14	1.48E-11	1360	757.838656					1.2E+08	1.2E+08	0.77242	ID=promoter_17146;
14	1E+08	14_118717958	148	48	171	162	98	167	0.012803	0.49582	60	40	93	0	50	47	0.99766929	1	90.25	63.6749558					1.2E+08	1.2E+08	0.16017	ID=promoter_17149;
14	1E+08	14_118800733	155	31	105	144	93	125	0.609393	1	49	0	65	1	25	33	0.40760787	1	68.75	60.4288247					1.2E+08	1.2E+08	0.15469	ID=promoter_17152;
14	1E+08	14_118876750	23	49	16	30	36	23	0.069202	1	2	22	30	4	12	17	0.52622129	1	22	15.2033831					1.2E+08	1.2E+08	0.20065	ID=promoter_17154;
14	1E+08	14_119130141	1	18	3	2	10	3	0.082185	1	14	12	0	1	13	1	0.00904697	0.3617342	6.375	7.11010348								
14	1E+08	14_119442044	220	142	228	234	181	231	0.696544	1	103	95	104	25	99	65	0.09460062	1	143.88	76.3253141					1.2E+08	1.2E+08	0.33977	ID=promoter_17166;
14	1E+08	14_119466066	2	1	8	13	2	11	0.085797	1	1	0	17	19	1	18	2.55E-04	0.0200383	7.625	7.78161753								
14	1E+08	14_119616987	363	202	276	269	283	273	0.069674	1	249	147	193	155	198	174	0.87602519	1	231.75	72.0411589								
14	1E+08	14_119660490	21	44	28	37	33	33	0.810265	1	20	10	7	0	15	4	0.06170633	1	20.875	15.0848789	1E+08	1.2E+08	ID=ENSP0000358789;SH3PXD2A;;	SH3PXD2A				
14	1E+08	14_119743933	262	126	2884	2968	194	2926	0	0	116	154	742	508	135	625	9.89E-78	2.82E-74	970	1226.46996								
14	1E+08	14_120000417	13	75	19	4	44	12	6.51E-06	0.00082	25	43	23	16	34	20	0.26570036	1	27.25	22.3271136								
14	1E+08	14_120326238	316	133	269	263	225	266	0.996232	1	12	154	109	39	83	74	0.97065186	1	161.88	111.29682					1.2E+08	1.2E+08	0.32415	ID=promoter_17185;
14	1E+08	14_120470713	446	171	477	352	309	415	0.196645	1	165	182	191	62	174	127	0.12138449	1	255.75	149.761477	1E+08	1.2E+08	ID=ENSP0000358718;CCDC147;NM_001008723;	CCDC147	1.2E+08	1.2E+08	0.32415	ID=promoter_17193;
14	1E+08	14_121358430	13	40	5	7	27	6	2.01E-04	0.01628	50	30	21	13	40	17	0.02201768	0.7404021	22.375	16.2475273	1E+08	1.2E+08	ID=ENSP0000376876;SORCS3;;	SORCS3				
14	1E+08	14_121836458	22	47	35	4	35	20	0.032214	1	4	38	80	19	21	50	8.41E-04	0.0548813	31.125	25.0852119								
14	1E+08	14_122223947	3	30	8	0	17	4	0.005755	0.26328	1	0	1	0	1	1	0.99856566	1	5.375	10.3086302								
14	1E+08	14_123417196	21	31	9	45	26	27	0.906335	1	0	18	35	0	9	18	0.15524483	1	19.875	16.4615353								
14	1E+08	14_123694703	0	12	0	17	6	9	0.896349	1	0	2	19	13	1	16	7.41E-04	0.0492332	7.875	8.20169843								
14	1E+08	14_123715041	2	50	18	9	26	14	0.055012	1	0	4	36	1	2	19	5.26E-04	0.0367435	15	18.6010752								
14	1E+08	14_125141126	12	35	7	6	24	7	0.00213	0.11798	5	1	14	1	3	8	0.26303803	1	10.125	11.0639376								
14	1E+08	14_125600082	24	61	5	11	43	8	2.90E-07	#######	5	21	28	22	13	25	0.09257925	1	22.125	17.9717039								
14	1E+08	14_126055261	276	90	165	176	183	171	0.100824	1	111	128	102	11	120	57	1.28E-04	0.0112886	132.38	77.0175073								
14	1E+08	14_126193541	48	3	15	0	26	8	0.001755	0.10104	0	0	8	37	0	23	4.10E-06	0.0005762	13.875	18.6351243								
14	1E+08	14_126249008	3346	1438	3266	2783	2392	3025	0.022725	0.78018	1689	1290	1436	454	1490	945	4.88E-19	3.23E-16	1962.8	1044.80603	1E+08	1.3E+08	ID=ENSP0000331152;MXI1;NM_130439;	MXI1	1.3E+08	1.3E+08	0.75387	ID=promoter_17205;
14	1E+08	14_126251137	4632	3425	5155	6185	4029	5670	9.65E-18	#######	2999	3040	3377	994	3020	2186	1.14E-17	7.05E-15	3725.9	1584.96998	1E+08	1.3E+08	ID=ENSP0000331152;MXI1;NM_130439;	MXI1				

14	1E+08	14_126421923	0	6	2	9	3	6	0.747087	1	25	13	5	9	19	7	0.09662636	1	8.625	7.79995421								
14	1E+08	14_126432017	27	16	22	0	22	11	0.058934	1	37	21	1	4	29	3	6.67E-05	0.0064567	16	13.3523674								
14	1E+08	14_127060652	23	15	17	12	19	15	0.513973	1	24	16	15	14	20	15	0.82431124	1	17	4.27617987								
14	1E+08	14_127229589	48	9	5	2	29	4	8.83E-06	0.00107	23	0	10	0	12	5	0.30420563	1	12.125	16.3308429								
14	1E+08	14_128839372	133	27	54	28	80	41	5.36E-05	0.00515	14	30	19	17	22	18	0.92557456	1	40.25	39.4669844								
14	1E+08	14_128949025	20	65	15	37	43	26	0.024991	0.84297	79	124	98	188	102	143	0.00570837	0.2548307	78.25	58.3089065								
14	1E+08	14_129037237	3	22	32	17	13	25	0.34413	1	53	20	23	7	37	15	0.02098916	0.7098276	22.125	15.4775183								
14	1E+08	14_129166207	957	249	652	557	603	605	0.024914	0.84237	202	230	249	82	216	166	0.19100985	1	397.25	295.966094					1.3E+08	1.3E+08	0.66318	ID=promoter_17230;
14	1E+08	14_129259108	455	228	476	463	342	470	0.083034	1	40	39	36	32	40	34	0.93312112	1	221.13	211.870875								
14	1E+08	14_129270004	17	89	39	24	53	32	0.010423	0.42436	18	18	25	5	18	15	0.95456211	1	29.375	25.9171069					1.3E+08	1.3E+08	0.12317	ID=promoter_17233;
14	1E+08	14_129281330	7	17	6	0	12	3	0.030625	0.98374	0	10	15	0	5	8	0.62517663	1	6.875	6.7704716	1E+08	1.3E+08	ID=ENSP00000344823;TCF7L2;;	TCF7L2				
14	1E+08	14_129321799	38	5	35	19	22	27	0.987654	1	16	21	10	0	19	5	0.02799778	0.8875672	18	13.4164079	1E+08	1.3E+08	ID=ENSP00000344823;TCF7L2;;	TCF7L2				
14	1E+08	14_129351671	26	11	11	5	19	8	0.039867	1	9	18	35	57	14	46	5.53E-05	0.0055177	21.5	17.4192012	1E+08	1.3E+08	ID=ENSP00000344823;TCF7L2;;	TCF7L2				
14	1E+08	14_129364190	12	13	15	9	13	12	0.835334	1	39	2	6	13	21	10	0.21021716	1	13.625	11.1090375	1E+08	1.3E+08	ID=ENSP00000344823;TCF7L2;;	TCF7L2				
14	1E+08	14_129380846	0	23	13	5	12	9	0.592799	1	26	8	14	5	17	10	0.5154613	1	11.75	9.09866552	1E+08	1.3E+08	ID=ENSP00000344823;TCF7L2;;	TCF7L2	1.3E+08	1.3E+08	0.24886	ID=promoter_17242;
14	1E+08	14_129498717	24	57	29	22	41	26	0.044828	1	13	19	50	12	16	31	0.04981266	1	28.25	16.6454627								
14	1E+08	14_129793151	15	14	7	22	15	15	0.908797	1	1	1	8	15	1	12	0.00617711	0.2683255	10.375	7.40535521	1E+08	1.3E+08	ID=ENSP00000277903;HABP2;NM_004132;	HABP2				
14	1E+08	14_129827080	24	45	24	49	35	37	0.907308	1	13	82	69	90	48	80	0.00493779	0.225322	49.5	28.6106873	1E+08	1.3E+08	ID=ENSP00000358365;NRAP;;	NRAP	1.3E+08	1.3E+08	0.16624	ID=promoter_17254;
14	1E+08	14_129835114	408	550	603	575	479	589	0.745039	1	21	17	10	15	19	13	0.6985193	1	274.88	282.792927	1E+08	1.3E+08	ID=ENSP00000358365;NRAP;;	NRAP				
14	1E+08	14_129878455	71	65	59	51	68	55	0.119458	1	26	66	57	18	46	38	0.8706833	1	51.625	19.390259	1E+08	1.3E+08	ID=ENSP00000358365;NRAP;;	NRAP				
14	1E+08	14_129889715	5382	2737	5255	5364	4060	5310	1.18E-06	0.00017	3417	2493	3606	1302	2955	2454	2.97E-04	0.0229721	3694.5	1523.12414					1.3E+08	1.3E+08	0.56174	ID=promoter_17256;
14	1E+08	14_130059856	0	20	23	2	10	13	0.970528	1	0	2	22	27	1	25	6.01E-06	0.0008111	12	11.9403278	1E+08	1.3E+08	ID=ENSP00000358307;NHLRC2;NM_198514;	NHLRC2				
14	1E+08	14_130142674	12	12	10	5	12	8	0.45534	1	24	26	6	2	25	4	0.00107118	0.0664407	12.125	8.69215574								
14	1E+08	14_130174669	87	108	181	164	98	173	0.004988	0.23452	105	61	140	69	83	105	0.10454861	1	114.38	43.6608275								
14	1E+08	14_130325700	9	45	28	25	27	27	0.841861	1	0	14	18	0	7	9	0.80856893	1	17.375	15.2121897								

14	1E+08	14_130492391	19	12	7	20	16	14	0.724026	1	3	20	44	35	12	40	1.75E-04	0.0147329	20	13.7529218					1.3E+08	1.3E+08	0.22516	ID=promoter_17267;
14	1E+08	14_130517635	8	21	12	4	15	8	0.181584	1	0	29	7	17	15	12	0.93002578	1	12.25	9.58793885	1E+08	1.3E+08	ID=ENSP000003767 00;AFAP1L2;;	AFAP1L2				
14	1E+08	14_130524383	55	19	61	100	37	81	0.006052	0.27365	0	0	18	0	0	9	0.00779604	0.3222758	31.625	36.7032015	1E+08	1.3E+08	ID=ENSP000003767 00;AFAP1L2;;	AFAP1L2				
14	1E+08	14_130660007	141	53	147	125	97	136	0.405125	1	55	53	51	53	54	52	0.98103932	1	84.75	44.2517151								
14	1E+08	14_131232608	6	1	4	0	4	2	0.597627	1	19	9	6	0	14	3	0.04246461	1	5.625	6.30051018								
14	1E+08	14_131329143	0	16	1	2	8	2	0.097885	1	26	9	0	0	18	0	2.38E-04	0.0190698	6.75	9.66215001								
14	1E+08	14_131440616	1	93	41	23	47	32	0.055772	1	20	55	52	2	38	27	0.5702351	1	35.875	30.9305097								
14	1E+08	14_132129779	37	7	35	30	22	33	0.669499	1	0	0	15	0	0	8	0.01336935	0.4945372	15.5	16.2392822								
14	1E+08	14_132174736	17	10	15	3	14	9	0.360318	1	10	34	31	17	22	24	0.85866092	1	17.125	10.5754095								
14	1E+08	14_132257076	104	68	121	99	86	110	0.834327	1	55	49	31	49	52	40	0.67381655	1	72	32.0668944								
14	1E+08	14_132317351	29	12	25	36	21	31	0.717186	1	24	26	15	18	25	17	0.61272849	1	23.125	7.82737869					1.3E+08	1.3E+08	0.79804	ID=promoter_17289;
14	1E+08	14_133146813	14	14	10	18	14	14	0.914609	1	18	1	10	20	10	15	0.49251655	1	13.125	6.12809688								
14	1E+08	14_133164551	173	97	130	129	135	130	0.274517	1	146	119	154	48	133	101	0.31794337	1	124.5	38.4967531								
14	1E+08	14_133213155	17	49	27	2	33	15	0.006796	0.29841	40	33	3	5	37	4	5.68E-06	0.0007768	22	18.0237938								
14	1E+08	14_133215008	17	21	26	6	19	16	0.617999	1	2	11	44	19	7	32	1.23E-04	0.0109354	18.25	13.0903454								
14	1E+08	14_133217099	14	27	0	3	21	2	7.54E-05	0.00697	13	19	7	0	16	4	0.04223275	1	10.375	9.65012953								
14	1E+08	14_133408948	1	13	16	7	7	12	0.724589	1	31	36	30	11	34	21	0.33784557	1	18.125	12.6766095	1E+08	1.3E+08	ID=ENSP000003346 42;PDZD8;NM_173791;	PDZD8				
14	1E+08	14_133414375	901	1177	1034	1339	1039	1187	0.824957	1	972	967	2402	715	970	1559	4.82E-41	7.56E-38	1188.4	523.999438					1.3E+08	1.3E+08	0.63799	ID=promoter_17318;
14	1E+08	14_133556281	1165	929	1617	2103	1047	1860	3.71E-26	#######	1064	796	1323	447	930	885	0.85629091	1	1180.5	510.56999					1.3E+08	1.3E+08	0.34944	ID=promoter_17323;
14	1E+08	14_133607537	14	34	9	26	24	18	0.351411	1	20	21	22	21	21	22	0.92273004	1	20.875	7.45342682								
14	1E+08	14_133687391	35	24	21	1	30	11	0.002281	0.12403	3	11	25	9	7	17	0.08351885	1	16.125	11.945561								
14	1E+08	14_133769744	27	17	60	47	22	54	0.011576	0.45508	1	25	17	3	13	10	0.90601506	1	24.625	20.3535711								
14	1E+08	14_133868463	152	121	164	176	137	170	0.886717	1	61	97	138	51	79	95	0.23034409	1	120	46.5863254								
14	1E+08	14_133900473	16	25	13	5	21	9	0.03105	0.99404	2	33	15	53	18	34	0.04464527	1	20.25	16.5680415								
14	1E+08	14_133986888	5	9	10	27	7	19	0.148295	1	0	0	6	53	0	30	9.40E-08	1.96E-05	13.75	17.9980158								
14	1E+08	14_134017297	18	60	18	43	39	31	0.266065	1	24	26	22	39	25	31	0.55457057	1	31.25	14.8396188								
14	1E+08	14_134168167	52	36	11	10	44	11	2.81E-06	0.00039	29	20	46	4	25	25	0.96475192	1	26	17.6716076								
14	1E+08	14_134201951	93	123	178	135	108	157	0.227896	1	98	84	80	63	91	72	0.60315677	1	106.75	36.9391391								
14	1E+08	14_134319598	14	14	4	3	14	4	0.02589	0.86655	2	17	18	0	10	9	0.99792604	1	9	7.42582367								
14	1E+08	14_134427842	41	8	21	32	25	27	0.956696	1	1	0	17	10	1	14	0.00214405	0.1161796	16.25	14.5773797								
14	1E+08	14_134483910	144	188	228	274	166	251	0.039203	1	202	184	61	2	193	32	3.38E-23	2.71E-20	160.38	89.2539515								
14	1E+08	14_134786968	6	32	13	16	19	15	0.513973	1	51	25	22	37	38	30	0.80512437	1	25.25	14.4593025								
14	1E+08	14_134924131	758	397	1042	935	578	989	3.72E-12	#######	273	237	261	77	255	169	0.00309503	0.1562651	497.5	361.911591								

14	1E+08	14_134996972	464	125	534	381	295	458	8.26E-04	0.05408	133	153	175	101	143	138	0.9449246	1	258.25	173.047269					1.3E+08	1.3E+08	0.57418	ID=promoter_17345;
14	1E+08	14_135184217	59	57	49	31	58	40	0.032784	1	47	59	32	14	53	23	0.00785432	0.3240285	43.5	16.3182632								
14	1E+08	14_135200637	5	12	2	1	9	2	0.058587	1	49	0	0	8	25	4	0.00107118	0.0664407	9.625	16.4658738								
14	1E+08	14_135309364	191	93	191	212	142	202	0.210886	1	140	39	119	5	90	62	0.19589823	1	123.75	74.9757104	1E+08	1.4E+08	ID=ENSP00000376609;GRK5;NM_005308;	GRK5				
14	1E+08	14_135743979	418	114	2	1	266	2	4.11E-67	#######	101	90	22	17	96	20	2.44E-10	7.76E-08	95.625	138.174773					1.4E+08	1.4E+08	0.24749	ID=promoter_17370;
14	1E+08	14_135788136	10	26	14	30	18	22	0.991654	1	10	26	16	23	18	20	0.85572507	1	19.375	7.83649885								
14	1E+08	14_135876090	40	70	113	103	55	108	0.007114	0.30807	146	115	141	96	131	119	0.98720941	1	103	35.1405342								
14	1E+08	14_136433264	2	31	0	8	17	4	0.005755	0.26328	0	0	0	0	0	0	1	1	5.125	10.8158283	1E+08	1.4E+08	ID=ENSP00000381302;PPAPDC1A;NM_001030059;	PPAPDC1A				
14	1E+08	14_136440910	15	29	21	28	22	25	0.99414	1	23	38	14	9	31	12	0.02977149	0.9232309	22.125	9.44816687					1.4E+08	1.4E+08	0.11862	ID=promoter_17377;
14	1E+08	14_136622675	61	46	30	60	54	45	0.234992	1	26	7	6	0	17	3	0.01239331	0.4669004	29.5	24.3075532								
14	1E+08	14_136786802	23	12	14	11	18	13	0.404491	1	16	11	16	7	14	12	0.98032967	1	13.75	4.77343841								
14	1E+08	14_137035694	3	0	5	2	2	4	0.823343	1	17	13	18	3	15	11	0.83933961	1	7.625	7.20986427								
14	1E+08	14_137041619	20	27	19	11	24	15	0.151024	1	31	1	37	8	16	23	0.39752759	1	19.25	12.197775								
14	1E+08	14_137093332	4	28	14	2	16	8	0.127562	1	30	22	2	1	26	2	8.18E-05	0.0077709	12.875	12.3223085								
14	1E+08	14_137468939	3024	1470	3164	3218	2247	3191	3.16E-11	#######	2379	1486	1882	660	1933	1271	1.44E-20	9.98E-18	2160.4	939.566456								
14	1E+08	14_137509753	65	24	59	91	45	75	0.173184	1	8	55	56	0	32	28	0.97537628	1	44.75	31.0931779								
14	1E+08	14_137615671	129	37	102	109	83	106	0.844886	1	53	91	59	22	72	41	0.0422523	1	75.25	37.889878								
14	1E+08	14_137830384	19	58	12	16	39	14	3.02E-04	0.02327	12	22	21	22	17	22	0.58360552	1	22.75	14.8203528	1E+08	1.4E+08	ID=ENSP00000358039;ATE1;NM_007041;	ATE1				
14	1E+08	14_137942548	22	9	7	10	16	9	0.196587	1	19	1	8	0	10	4	0.34296513	1	9.5	7.72750191	1E+08	1.4E+08	ID=ENSP00000384150;;					
14	1E+08	14_137969263	7	12	17	13	10	15	0.833288	1	76	26	16	22	51	19	0.00210847	0.1150505	23.625	21.967102								
14	1E+08	14_137975042	7	14	5	24	11	15	0.930409	1	15	34	12	2	25	7	0.01215165	0.4601029	14.125	10.548358								
14	1E+08	14_138004952	94	22	53	47	58	50	0.277127	1	22	20	34	23	21	29	0.37526954	1	39.375	25.3655419								
14	1E+08	14_138108501	19	6	14	7	13	11	0.725732	1	45	25	28	0	35	14	0.02362517	0.7761779	18	14.5602198					1.4E+08	1.4E+08	0.12517	ID=promoter_17457;
14	1E+08	14_138164932	1	0	4	0	1	2	0.907382	1	51	50	20	15	51	18	0.00122967	0.074208	17.625	21.5733798								
14	1E+08	14_138177716	9	35	6	19	22	13	0.135741	1	4	17	24	20	11	22	0.10262264	1	16.75	10.2782711								
14	1E+08	14_138179843	3	14	10	18	9	14	0.802715	1	34	13	44	26	24	35	0.22621095	1	20.25	13.5514891								
14	1E+08	14_138241495	10	13	6	10	12	8	0.45534	1	5	15	16	2	10	9	0.99792604	1	9.625	4.98390266	1E+08	1.4E+08	ID=ENSP00000260723;BTBD16;NM_144587;	BTBD16				
14	1E+08	14_138385169	549	481	561	519	515	540	0.190389	1	485	283	290	259	384	275	0.00466466	0.2152661	428.38	128.339881					1.4E+08	1.4E+08	0.63125	ID=promoter_17466;

14	1E+08	14_138405409	7	12	7	6	10	7	0.570701	1	0	0	32	1	0	17	1.04E-04	0.0094884	8.125	10.521237	1E+08	1.4E+08	ID=ENSP000003868 00;PLEKHA1;NM_001001974;	PLEKHA1				
14	1E+08	14_138439338	6	42	27	26	24	27	0.98899	1	19	66	23	18	43	21	0.04986116	1	28.375	18.2595376								
14	1E+08	14_138467021	183	168	166	95	176	131	3.70E-04	0.02765	121	42	43	30	82	37	0.00100787	0.0629514	106	62.7466561					1.4E+08	1.4E+08	0.7724	ID=promoter_17470;
14	1E+08	14_138468300	716	275	462	544	496	503	0.070752	1	304	274	244	159	289	202	0.0084574	0.34443	372.25	185.70695	1E+08	1.4E+08	ID=ENSP000003579 80;HTRA1;NM_002775;	HTRA1	1.4E+08	1.4E+08	0.7724	ID=promoter_17470;
14	1E+08	14_138477959	1	42	3	5	22	4	4.12E-04	0.03018	30	11	9	35	21	22	0.92273004	1	17	16.0979147	1E+08	1.4E+08	ID=ENSP000003579 80;HTRA1;NM_002775;	HTRA1				
14	1E+08	14_138556189	13	45	1	6	29	4	8.83E-06	0.00107	0	3	27	1	2	14	0.00692302	0.294895	12	16.1156534								
14	1E+08	14_138819036	21	18	10	4	20	7	0.013885	0.52965	29	0	9	1	15	5	0.11779525	1	11.5	10.3233439								
14	1E+08	14_138824477	66	35	68	19	51	44	0.325273	1	0	13	29	4	7	17	0.08351885	1	29.25	26.0425476					1.4E+08	1.4E+08	0.13029	ID=promoter_17496;
14	1E+08	14_139006501	71	106	91	373	89	232	4.06E-10	#######	40	89	104	41	65	73	0.53016587	1	114.38	107.605015					1.4E+08	1.4E+08	0.51695	ID=promoter_17511;
14	1E+08	14_139126930	832	546	786	664	689	725	0.124297	1	419	198	281	245	309	263	0.59523348	1	496.38	248.330964					1.4E+08	1.4E+08	0.90877	ID=promoter_17514;
14	1E+08	14_139143033	331	119	218	246	225	232	0.386716	1	54	109	44	29	82	37	0.00100787	0.0629514	143.75	109.559834	1E+08	1.4E+08	ID=ENSP000003411 08;HMX2;NM_005519;	HMX2	1.4E+08	1.4E+08	0.81252	ID=promoter_17518;
14	1E+08	14_139166228	43	28	48	24	36	36	0.794914	1	1	26	41	45	14	43	1.98E-04	0.0163228	32	15.6021976								
14	1E+08	14_139251077	17	15	5	28	16	17	0.960459	1	27	0	2	0	14	1	0.00574567	0.2548307	11.75	11.6588409								
14	1E+08	14_139292580	26	11	17	6	19	12	0.233425	1	58	9	5	20	34	13	0.01953062	0.6666599	19	17.336996								
14	1E+08	14_139310569	16	32	8	8	24	8	0.004476	0.21437	0	29	11	1	15	6	0.20085144	1	13.125	11.90963								
14	1E+08	14_139352029	7	21	3	17	14	10	0.480768	1	29	39	10	1	34	6	1.54E-04	0.0131747	15.875	13.2819695					1.4E+08	1.4E+08	0.1429	ID=promoter_17524;
14	1E+08	14_139365359	22	14	9	3	18	6	0.017303	0.62786	0	0	7	0	0	4	0.11562589	1	6.875	7.93612896								
14	1E+08	14_139469624	6	1	1	17	4	9	0.54411	1	2	18	0	11	10	6	0.69729498	1	7	7.4065608								
14	1E+08	14_139532712	20	21	6	2	21	4	7.04E-04	0.04721	23	34	10	0	29	5	5.02E-04	0.0355894	14.5	11.8562822								
14	1E+08	14_139795064	49	88	74	49	69	62	0.311447	1	21	22	57	51	22	54	3.31E-04	0.0251523	51.375	22.9343006	1E+08	1.4E+08	ID=ENSP000003699 79-D2;CPXM1;NM_019609;	CPXM1				
14	1E+08	14_139810458	17	0	8	5	9	7	0.713906	1	25	0	0	21	13	11	0.97545578	1	9.5	10.1559272	1E+08	1.4E+08	ID=ENSP000003699 79-D2;CPXM1;NM_019609;	CPXM1	1.4E+08	1.4E+08	0.14355	ID=promoter_17544;
14	1E+08	14_139851752	18	0	18	12	9	15	0.704209	1	58	34	1	0	46	1	2.28E-09	6.24E-07	17.625	20.099307	1E+08	1.4E+08	ID=ENSP000003699 79-D2;CPXM1;NM_019609;	CPXM1	1.4E+08	1.4E+08	0.15381	ID=promoter_17549;
14	1E+08	14_139903951	14	18	34	17	16	26	0.588052	1	25	56	21	1	41	11	5.18E-04	0.0365242	23.25	16.2282822								

14	1E+08	14_139990200	0	0	0	0	0	0	1	1	43	10	38	3	27	21	0.83317938	1	11.75	18.1166853	1E+08	1.4E+08	ID=ENSP0000333947-D4;CHST15;NM_015892;	CHST15	1.4E+08	1.4E+08	0.14	ID=promoter_17559;
14	1E+08	14_139997736	9	0	8	12	5	10	0.615109	1	18	8	6	0	13	3	0.06317581	1	7.625	5.95069024								
14	1E+08	14_140197483	12	3	10	7	8	9	0.996316	1	4	3	0	1	4	1	0.45332778	1	5	4.27617987								
14	1E+08	14_140396970	45	0	21	15	23	18	0.432563	1	0	0	7	1	0	4	0.11562589	1	11.125	15.8153407					1.4E+08	1.4E+08	0.14101	ID=promoter_17599;
14	1E+08	14_140397085	1	6	7	10	4	9	0.54411	1	23	0	6	8	12	7	0.61698677	1	7.625	7.06980501					1.4E+08	1.4E+08	0.14101	ID=promoter_17599;
14	1E+08	14_140603539	89	8	58	41	49	50	0.786177	1	0	11	8	21	6	15	0.10150651	1	29.5	30.9238697								
14	1E+08	14_140622199	83	32	70	13	58	42	0.055656	1	0	0	5	0	0	3	0.19828566	1	25.375	33.5002665								
14	1E+08	14_140646998	72	12	60	65	42	63	0.464879	1	94	43	36	25	69	31	0.0028472	0.1455015	50.875	26.8670669								
14	1E+08	14_140775340	2	9	6	13	6	10	0.79153	1	39	26	23	4	33	14	0.04245489	1	15.25	12.9366589	1E+08	1.4E+08	ID=ENSP0000357835;;NM_022126;					
14	1E+08	14_140785130	4	1	18	15	3	17	0.020784	0.72593	32	3	0	16	18	8	0.20901227	1	11.125	11.1154654	1E+08	1.4E+08	ID=ENSP0000357835;;NM_022126;					
14	1E+08	14_140810943	36	66	36	26	51	31	0.013326	0.51068	33	37	9	16	35	13	0.01427485	0.52128	32.375	17.045632	1E+08	1.4E+08	ID=ENSP0000357835;;NM_022126;					
14	1E+08	14_140818300	8	49	27	42	29	35	0.993567	1	7	33	31	17	20	24	0.69398062	1	26.75	15.2198179	1E+08	1.4E+08	ID=ENSP0000357835;;NM_022126;					
14	1E+08	14_140820929	0	18	10	10	9	10	0.993074	1	41	16	22	1	29	12	0.05423954	1	14.75	13.1230441	1E+08	1.4E+08	ID=ENSP0000357835;;NM_022126;		1.4E+08	1.4E+08	0.10676	ID=promoter_17647;
14	1E+08	14_140920490	210	93	123	162	152	143	0.165386	1	0	1	11	2	1	7	0.08320466	1	75.25	83.6211696	1E+08	1.4E+08	ID=ENSP0000338532;FAM53B;NM_014661;	FAM53B				
14	1E+08	14_141035691	3525	2018	3625	3387	2772	3506	0.012273	0.47814	1895	1544	2026	782	1720	1404	0.00189904	0.1051379	2350.3	1042.80552	1E+08	1.4E+08	ID=ENSP0000298492;FAM175B;NM_032182;	FAM175B				
14	1E+08	14_141070290	2	12	3	11	7	7	0.956352	1	0	16	7	4	8	6	0.92562801	1	6.875	5.61725657								
14	1E+08	14_141100006	33	53	46	19	43	33	0.181704	1	172	78	182	59	125	121	0.94415608	1	80.25	62.2523666					1.4E+08	1.4E+08	0.12992	ID=promoter_17685;
14	1E+08	14_141134738	0	17	17	8	9	13	0.89076	1	10	15	33	0	13	17	0.64388579	1	12.5	10.730464								
14	1E+08	14_141150616	433	204	398	233	319	316	0.103138	1	18	12	20	1	15	11	0.83933961	1	164.88	179.362711								
14	1E+08	14_141202579	20	12	17	11	16	14	0.724026	1	13	0	20	25	7	23	0.00743961	0.3120589	14.75	7.62982868								
14	1E+08	14_141223071	1832	817	1842	1527	1325	1685	0.088545	1	1004	548	1072	367	776	720	0.99986408	1	1126.1	560.017713	1E+08	1.4E+08	ID=ENSP0000311825;CTBP2;NM_022802;	CTBP2				
14	1E+08	14_141242042	11	63	10	6	37	8	6.99E-06	0.00087	33	22	67	19	28	43	0.11009196	1	28.875	23.8413656								
14	1E+08	14_141252423	0	9	14	6	5	10	0.615109	1	2	0	18	0	1	9	0.02974549	0.9232309	6.125	6.97828264								
14	1E+08	14_141331380	34	45	17	14	40	16	6.92E-04	0.04656	73	25	10	0	49	5	8.15E-08	1.73E-05	27.25	23.2363631								
14	1E+08	14_141411131	2	5	5	0	4	3	0.840045	1	18	6	24	0	12	12	0.98292303	1	7.5	8.78310066								

14	1E+08	14_141467912	9	6	1	6	8	4	0.357158	1	12	3	0	3	8	2	0.20550608	1	5	4.07080196								
14	1E+08	14_141510254	85	2	109	94	44	102	6.03E-04	0.04133	60	98	61	4	79	33	4.11E-04	0.0300788	64.125	41.3881884								
14	1E+08	14_141673680	56	27	37	48	42	43	0.821371	1	0	22	3	1	11	2	0.06154634	1	24.25	21.8026866								
14	1E+08	14_141724615	0	12	1	0	6	1	0.108618	1	22	1	0	19	12	10	0.96947392	1	6.875	9.35700654								
14	1E+08	14_141746157	9	27	8	4	18	6	0.017303	0.62786	2	0	32	49	1	41	1.11E-09	3.23E-07	16.375	17.6225302								
14	1E+08	14_141960510	13	47	29	14	30	22	0.240296	1	57	22	37	44	40	41	0.90282393	1	32.875	16.066269					1.4E+08	1.4E+08	0.17417	ID=promoter_17760;
[truncated: 612,549 more chars]
